# Access to enantioenriched compounds bearing challenging tetrasubstituted stereocenters via kinetic resolution of auxiliary adjacent alcohols

**DOI:** 10.1038/s41467-021-23990-4

**Published:** 2021-06-18

**Authors:** Shengtong Niu, Hao Zhang, Weici Xu, Prasanta Ray Bagdi, Guoxiang Zhang, Jinggong Liu, Shuang Yang, Xinqiang Fang

**Affiliations:** 1 State Key Laboratory of Structural Chemistry, and Key Laboratory of Coal to Ethylene Glycol and Its Related Technology, Center for Excellence in Molecular Synthesis, Fujian Institute of Research on the Structure of Matter, University of Chinese Academy of Sciences, Fuzhou, China; 2 Orthopedics Department, Guangdong Provincial Hospital of Traditional Chinese Medicine, The Second Affiliated Hospital of Guangzhou University of Chinese Medicine, Guangzhou, China

**Keywords:** Asymmetric catalysis, Organic chemistry

## Abstract

Contemporary asymmetric catalysis faces huge challenges when prochiral substrates bear electronically and sterically unbiased substituents and when substrates show low reactivities. One of the inherent limitations of chiral catalysts and ligands is their incapability in recognizing prochiral substrates bearing similar groups. This has rendered many enantiopure substances bearing several similar substituents inaccessible. Here we report the rationale, scope, and applications of the strategy of kinetic resolution of auxiliary adjacent alcohols (KRA*) that can be used to solve the above troubles. Using this method, a large variety of optically enriched tertiary alcohols, epoxides, esters, ketones, hydroxy ketones, epoxy ketones, β-ketoesters, and tetrasubstituted methane analogs with two, three, and four spatially and electronically similar groups can be readily obtained (totally 96 examples). At the current stage, the strategy serves as the optimal solution that can complement the inability caused by direct asymmetric catalysis in getting chiral molecules with challenging fully substituted stereocenters.

## Abstract (web summary)

A large number of enantiopure substances, such as those with tetrasubstituted carbon centres bearing several similar substituents, are inaccessible due to the incapability of chiral catalysts/ligands to recognize those substrates. Here, the authors develop kinetic resolution of auxiliary adjacent alcohols (KRA*) strategy to access various optically enriched compounds with two, three or four spatially and electronically similar groups.

## Introduction

The last several decades have witnessed great achievements in the field of asymmetric catalysis, which have laid a profound impact on various fields including chemical engineering, pharmaceutical industry, material science, and agrochemistry. The success relies mainly on the discovery of various elegantly designed chiral catalysts/ligands, which enable the excellent enantiodiscrimination of prochiral substrates in the reaction^1–4^. On the other side, fully substituted carbon stereocenters are ubiquitous in naturally occurred compounds, bioactive substances, and drug molecules, and the rapid and efficient construction of substances containing such key moieties represents one of the most important topics in asymmetric synthesis^5–10^.

**Fig. 1:**
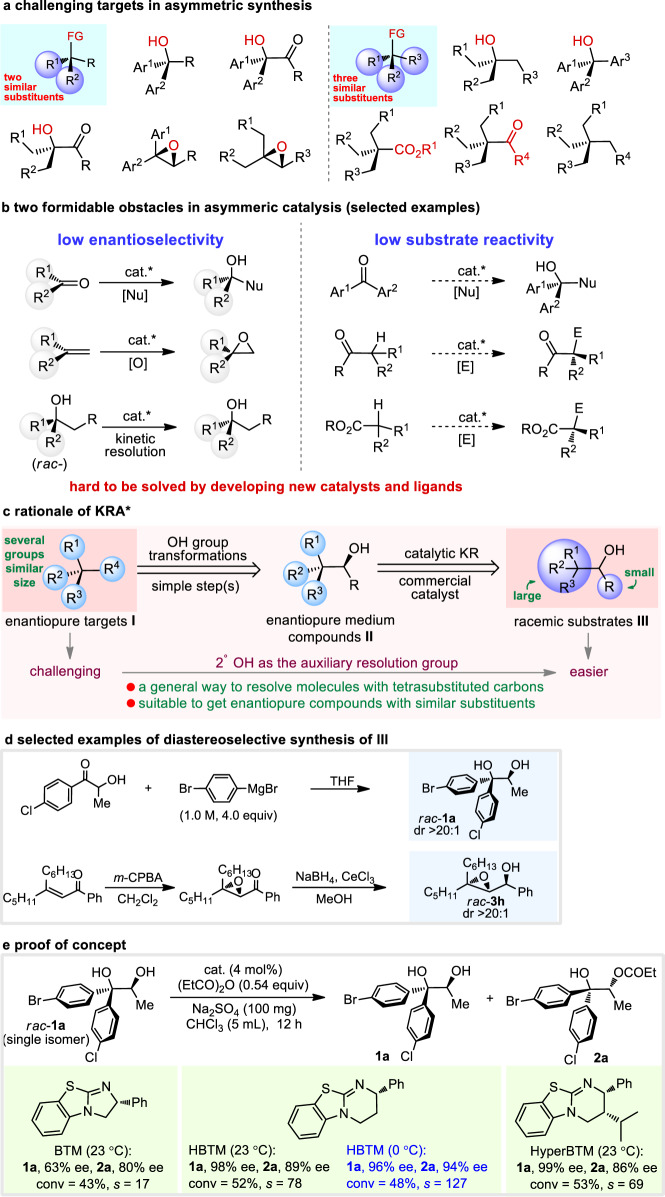
Inspiration for the development of KRA*. **a** Challenging targets in asymmetric synthesis. **b** Two formidable obstacles in asymmeric catalysis. Cat.* catalyst, O oxidants, Nu nucleophiles, E electrophiles. **c** Rationale of hydroxy addition-kinetic resolution. **d** Selected examples of diastereoselective synthesis of **III**. **e** Proof of concept. Ee values were determined via HPLC analysis on a chiral stationary phase; selectivity factors (*s*) were calculated according to the following equation: *s* = ln[(1 − conv)(1 − ee_1a_)]/ln[(1 − conv)(1 + ee_1a_)], conv = (ee_1a_)/(ee_1a_ + ee_2a_).

Although great progress has been made in addressing the above issue, a series of challenging targets still exist. For instance, Fig. 1a lists two general classes of functionalized compounds with tetrasubstituted carbon centers. Because all the stereogenic centers are connected by two, three, and even four electronically and sterically similar groups, most of them are hard to be rapidly produced via currently known direct catalytic methods. The origin arises from two innate limitations concerning asymmetric catalysis (Fig. 1b): (1) when prochiral substrates contain two similar substituents, low ee of the products will be expected owing to the poor facial enantiodiscrimination; (2) when substrates display low reactivities, the corresponding asymmetric catalysis will be more challenging and harsh conditions have to be employed, which is very probable to lead to low levels of enantioselectivity. For instance, enantiopure tertiary alcohols are important building blocks and key units in a large amount of naturally occurring and artificial bioactive molecules; asymmetric nucleophilic additions to ketones and kinetic resolution of racemic tertiary alcohols represent two mostly used strategies allowing access to tertiary alcohols with high enantiopurity^11–19^. However, for the former one, the frequently used prochiral ketones are aryl/alkyl ketones (e.g., acetophenone), α-ketoesters, trifluoromethyl ketones, and isatin derivatives^20–35^; ketones with electronically and sterically unbiased substituents such as Ar^1^COAr^2^ and R^1^CH_2_COCH_2_R^2^, and less reactive ketones such as ^*t*^BuCOPh and ^*t*^BuCO^*i*^Pr are not suitable reaction partners for nucleophilic additions. Kinetic resolution methods producing enantiopure tertiary alcohols are mostly constrained to those with aryl/alkyl substituents and sterically different alkyl-alkyl combinations^36–48^. A similar situation can also be found in the access to enantioenriched epoxides, ketones, and esters, etc. (Fig. 1a). Therefore, to promote the diversity of the current molecule types containing tetrasubstituted stereocenters and provide opportunities for bioactive molecule preparation and pharmaceutical discovery, a general solution to obtain all the above fully substituted molecules is highly desirable but still absent to date.

In this work, we develop an alternative strategy of kinetic resolution of auxiliary adjacent alcohols (KRA*) to address the big challenge that is hard to be solved by designing chiral catalysts and ligands. The work features the combination of diastereoselective secondary alcohol synthesis and the following kinetic resolution, and in the process, the hydroxy group serves as a kinetic resolution auxiliary group.

## Results

### Design of the project

As illustrated in Fig. 1c, enantiopure targets (**I**) represent all types of challenging compounds shown in Fig. 1a, and are hard to be obtained using direct synthetic methods. However, inspired by Corey’s retrosynthetic analysis, we imagine that **I** can be readily derived from enantiopure medium compounds **II** via OH group transformations; enantiopure **II** then can be obtained from racemic substrates **III** through kinetic resolution, because now the two groups connected to the second alcohol moiety are with different steric and (or) electronic properties. If successful, this method of combining diastereoselective synthesis of secondary alcohols bearing adjacent full-substituted carbon centers and the following kinetic resolution will be a general, robust, and practical strategy for the access to many different types of challenging compounds bearing tetrasubstituted stereogenic centers. Noteworthy is that substrates **III** are relatively easier to be obtained as racemic form, but hard to be produced in their enantioenriched form using known methods; therefore, the kinetic resolution of **III** is still the optimal way to get the enantiopure ones. Selected examples of highly diastereoselective synthesis of **III** are shown in Fig. 1d (see the Supplementary Material for more details). Moreover, the conventional kinetic resolution has mainly focused on the separation of racemic compounds with a single stereocenter; resolution on racemic substances bearing two or more stereocenters has been underdeveloped and its great potential in organic synthesis remains to be demonstrated. Nevertheless, we believe that resolution on easily available racemic substrates with multiple stereocenters will be an inevitable tendency for the further development of the whole field of kinetic resolution.

### Reaction optimization

To test the feasibility of our hypothesis, we commenced by selecting racemic diol **1a** as the model substrate to check the possibility of achieving an efficient resolution of tertiary alcohol with two aryl groups. Acyl transfer catalysts^49–52^ BTM, HBTM, and HyperBTM were selected for the survey. As shown in Fig. 1e, among the three catalysts, both HBTM and HyperBTM showed good results, with the former one giving slightly higher selectivity factor (*s*) of 78. To our pleasure, lowering the temperature proved beneficial to the resolution when HBTM was used as the catalyst, and up to 127 of the *s* value was observed, which also proved the feasibility of our hypothesis. Noteworthy is that direct asymmetric dihydroxylation of 1,1-diaryl alkenes using Sharpless’ conditions can only afford the corresponding alcohols with poor enantioselectivity^53^, and such type of getting enantioenriched tertiary alcohols using the assistance of secondary alcohol resolution has been not disclosed.

### Substrate scope

**Fig. 2:**
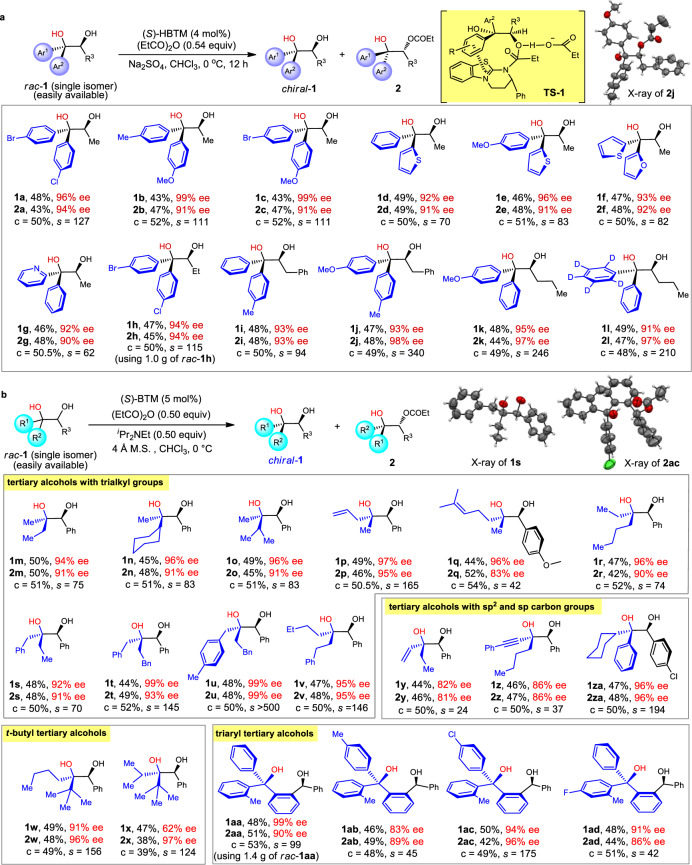
Scope of tertiary alcohols. **a** Scope of diaryl tertiary alcohols. **b** Scope of dialkyl tertiary alcohols and triaryl tertiary alcohols. Variations of the standard conditions: **1n**: (*S*)-BTM (10 mol%), rt; **1r**: anhydrous Na_2_SO_4_ (150 mg), toluene, 0 °C, 11 h; **1x**: rt, 20 h; **1y**: −40 °C; **1aa**–**1ad**: (*S*)-BTM (10 mol%), Na_2_SO_4_ (150 mg), toluene; **1ad**: −20 °C. c conversion.

Having proved the conceptual feasibility of KRA*, we investigated a series of diols bearing diary tertiary alcohol units, and in all cases, excellent enantiopurity (91–99% ee) of the recovered diols were observed, with up to 340 of the *s* value obtained (Fig. 2a, **1j**). It’s worthwhile to mention that although all racemic diols **1** can be readily made from the corresponding racemic α-hydroxy ketones, the state-of-art asymmetric synthesis of these α-hydroxy ketones has been not satisfied, and most of them cannot be obtained in a practically useful level (i.e., >60% yield and >95% ee, see the Supplementary Material for more details) for the further synthesis of chiral diols **1**. This method also tolerated aryl groups with a minimal difference, such as 4-Br-C_6_H_4_/4-Cl-C_6_H_4_ (Fig. 2a, **1a** and **1h**), 4-Me-C_6_H_4_/4-OMe-C_6_H_4_ (Fig. 2a, **1b** and **1j**), 2-thienyl/2-furyl (Fig. 2a, **1f**), 2-pyridinyl/Ph (Fig. 2a, **1g**), and C_6_D_5_/C_6_H_5_ (Fig. 2a, **1l**), and can be scaled up using gram scale of racemic **1h**. Actually, the method shows no limitation with respect to the steric and electronic difference of the two aryl substituents, and furthermore, through simply changing the synthesis route of the racemic substrates, both enantiopure diastereomeric diols can be obtained, as witnessed by **1b** and **1j** (the absolute configurations are opposite at the tertiary alcohol units). In literature reports, Rh-catalyzed asymmetric addition of aryl boric acids to ketones has been developed to make diaryl tertiary alcohols, but the system is usually compatible with activated ketones such as trifluoromethyl ketones and α-ketocarbonyls^21,54,55^. Titanium-catalyzed asymmetric Grignard addition to aryl ketones has also been developed, but in most cases, methyl aryl ketones were tested to give moderate ee, and ethyl aryl ketone proved inert to the reaction^20^. Also noteworthy is that, different from the established secondary alcohol kinetic resolution mode using HBTM^50^, the π-π stacking in this process occurs between the Ar^1^ group connected to the tertiary alcohol unit and the catalyst (Fig. 2a, **TS-1**). To the best of our knowledge, such a resolution model has been not established in known reports^49–52^. This is further confirmed by the X-ray single-crystal analysis of **2j** (Fig. 2a, and also see Supplementary Data 1).

Inspired by the success of accessing diaryl tertiary alcohols using KRA* method, we further surveyed tertiary alcohols with all aliphatic substituents (three sp^3^ carbons), which represents one of the most challenging topics in the field of asymmetric catalysis. Very limited methods have been developed to address the issue, and to date the most effective methods were reported by Hoveyda and co-workers, using asymmetric allylation and propargylation of methyl and mono, di, or trifluoromethyl ketones^31,32,56–58^ and the kinetic resolution of methyl tertiary alcohols^39^. Asymmetric desymmetrization has also been developed, but with specific types of substrates^59–62^. To our pleasure, under slightly modified conditions (using BTM instead of HBTM), a series of tertiary alcohols with Me/Et (Fig. 2b, **1m**), Me/cyclohexyl (Fig. 2b, **1n**), Me/^*i*^Pr (Fig. 2b, **1o**), Me/allyl (Fig. 2b, **1p**), Me/4-Me-3-pentenyl (Fig. 2b, **1q**), and Et/^*n*^Bu (Fig. 2b, **1r**) substituents were all successfully resolved, and the recovered diols were obtained with 94–97% ee. Synthetically more difficult tertiary alcohols with two methylene groups such as PhCH_2_/MeCH_2_ (Fig. 2b, **1s**), BnCH_2_/PhCH_2_ (Fig. 2b, **1t**), BnCH_2_/4-MeC_6_H_4_CH_2_ (Fig. 2b, **1u**), and EtCH_2_CH_2_/PhCH_2_CH_2_ (Fig. 2b, **1v**) can also be easily accessed via the KRA* method, and the *s* value can be up to >500 (Fig. 2b, **1u**). Moreover, sterically bulky ^*t*^Bu-substituted diols can also be efficiently resolved (Fig. 2b**, 1w and 1x**). To the best of our knowledge, **1r-1x** type tertiary alcohols are not accessible using currently known methods. Furthermore, tertiary alcohols bearing substituents with different electronic properties (Et/vinyl, ^*n*^Bu/phenylethynyl, cyclohexyl/Ph) were also tolerated under the standard conditions, leading to recovered **1y**−**1za** with good to excellent enantiopurities (Figs. 2b**, 1y−1za**). Additionally, the state-of-art of asymmetric catalysis cannot produce enantiopure triaryl tertiary alcohols as far as we know. To test the robustness of KRA*, we found that enantioenriched triaryl alcohols **1aa**−**1ad** can also be produced with up to 99% ee (Fig. 2b, **1aa**−**1ad**). Noteworthy is that the resolution of **1aa** was run on a 1.4 g scale. X-ray structure analysis of **1s** (Supplementary Data 2) and **2ac** (Supplementary Data 3) confirms the configuration of the enantioenriched alcohols.

**Fig. 3:**
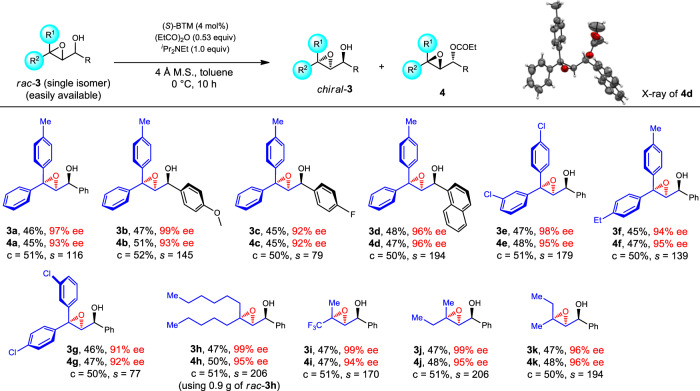
Scope of epoxides with similar geminal substituents. **3a**–**3g**, resolution of epoxides with two similar aromatic substituents. **3h**–**3k**, resolution of epoxides with two similar aliphatic substituents. c conversion.

Subsequently, we focused on the catalytic resolution of epoxides with geminal aryl or alkyl groups. The great importance of chiral epoxides has been well documented, and the asymmetric olefin epoxidation is the most direct and powerful approach to produce such molecules, as has been established by Sharpless, Jacobsen, Shi, and others^63–68^. However, alkenes having 1,1-diaryl or 1,1-dialkyl substituents with similar steric bulkiness are not suitable reaction partners for highly enantioselective epoxidation. Pleasingly, we found that the KRA* method can achieve the highly efficient resolution of epoxides containing diaryl substituents with minimal steric and electronic differences, producing **3a**–**3g** with 91–99% ee (Fig. 3**, 3a−3g**). Similarly, the system showed no negative influence on epoxides with geminal aliphatic groups, delivering **3h**–**3k** with 96–99% ee (Fig. 3**, 3h−3k**). The absolute configuration of **4d** was confirmed by single-crystal X-ray structure analysis (Supplementary Data 4). It’s worthwhile to mention that enantioenriched epoxy alcohols (EEAs) with three stereogenic centers also hold great importance in modern organic synthesis, but the predominant catalytic method to make EEAs is still the kinetic resolution of racemic allylic alcohols using Sharpless epoxidation^69,70^. Therefore, this method provides a complementary approach for getting EEAs with germinal similar substituents, which are hard to be formed with high enantiopurity using Sharpless’ asymmetric epoxidation protocol. Also noteworthy is that, in principle, all the chiral epoxy alcohols shown in Fig. 3 can be made from the diastereoselective reduction of chiral epoxy ketones or epoxidation of chiral allylic alcohols, but a global literature survey shows that all epoxy alcohols **3** cannot be obtained with a useful level of enantiopurity starting from chiral chemicals (see the Supplementary Material for more details).

**Fig. 4:**
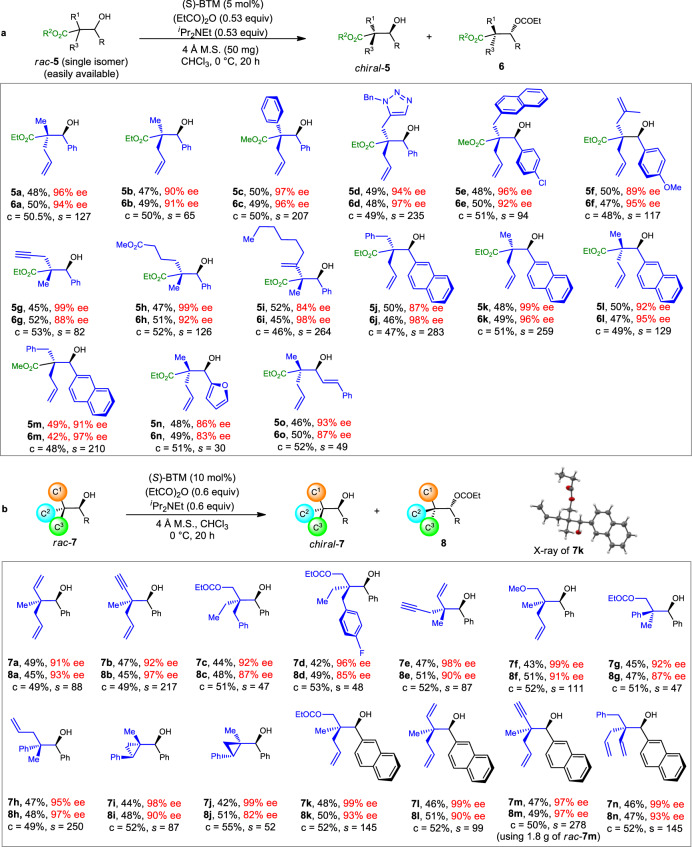
Resolution of ester and methane derivatives bearing quaternary stereocenters. **a** Scope of esters with α-quaternary stereocenters. Variation of the standard conditions: **5f**: 10 °C, 28 h; **5b**/**5j**/**5m**: 20 °C; **5c**/**5d**: (*S*)-BTM (10 mol%); **5g**/**5i**/**5l**/**5n**/**5o**: (*S*)-BTM (10 mol%), −10 °C. **b** Scope of tetrasubstituted methane derivatives. Variations of the standard conditions: **7c**/**7g**: −10 °C, 23 h; **7h**: (*S*)-BTM (15 mol%), −20 °C, 40 h; **7i**: (*S*)-BTM (4 mol%), *i*Pr_2_NEt (1.0 equiv), toluene; **7j**: ^*i*^Pr_2_NEt (1.0 equiv), toluene, 12 h; **7k**/**7m**: (*S*)-BTM (5 mol%). c conversion.

Asymmetric synthesis of acyclic esters with α-quaternary carbon centers has been a long-time challenge, owing to the limited activation methods towards esters, and the relatively low reactivity of α,α-disubstituted esters. Known methods have relied heavily on activated substrates such as α-branched β-ketoesters or α-cyano esters^71–76^. Catalytic approaches affording esters with α-quaternary carbons connected by three Csp^3^ groups are still rare. Therefore, our next goal is to test the capability of KRA* in accessing enantioenriched esters (especially unactivated esters) with α-quaternary stereocenters. As displayed in Fig. 4, racemic **5** can be readily made through the stereoselective reduction of the corresponding α,α-disubstituted β-ketoesters (see the Supplementary Material).

Using the KRA* method, a variety of α-allyl-β-hydroxy esters can be successfully resolved, providing enantioenriched **5a**–**5f** with 89–97% ee (Fig. 4a**, 5a−5f**). We were glad to find that esters with other functional groups such as propargyl group (**5g**), butyric ester group (**5h**), and vinyl group (**5i**) were also compatible under the resolution conditions (Fig. 4a**, 5g**−**5i**). Moreover, the substituents connected to the secondary alcohol moiety are not limited to the phenyl group, and other groups such as naphthyl, furyl, and styryl groups are also efficient in achieving the corresponding ester resolution (Fig. 4a, **5j**−**5o**). Because racemic **5** were obtained via diastereoselective reduction of the corresponding disubstituted ketoesters, one will imagine the possibility of getting chiral **5** from chiral α,α-disubtituted β-ketoesters. However, the known methods cannot afford all the corresponding α,α-disubtituted β-ketoesters used in this work in acceptable results (see the Supplementary Material for more details).

All-carbon quaternary stereogenic centers are ubiquitous in naturally occurred substances and artificial pharmaceuticals^10,77,78^. However, the construction of enantiopure compounds with all-carbon quaternary centers also represents one of the most challenging topics in asymmetric catalysis, especially for those existing in acyclic molecules. Asymmetric nucleophilic addition, cascade annulation, and allylic alkylation are powerful methods to achieve the purpose, but in most cases, they deliver products with electronically distinct substituents^5–10,79,80^. Therefore, a remaining hard topic is the rapid asymmetric formation of all-sp^3^-carbon-substituted quaternary centers, and very limited reports have achieved this purpose. In this context, we tested the possibility of getting enantioenriched compounds with various different all-carbon quaternary centers using KRA* approach. To our delight, all of them can be easily resolved, and in all cases, excellent 91–99% ee values were detected (Fig. 4b, **7a**–**7n**). The absolute configuration of **7k** was confirmed by single-crystal X-ray structure analysis (Supplementary Data 5). Noteworthy is that molecules with quaternary carbon centers bearing three methylene groups were also efficiently resolved (Fig. 4b, **7c** and **7d**). The reaction using 1.8 g of racemic **7m** also proved successful, again showing the potential of this method in big scale production.

### Synthetic transformations

**Fig. 5:**
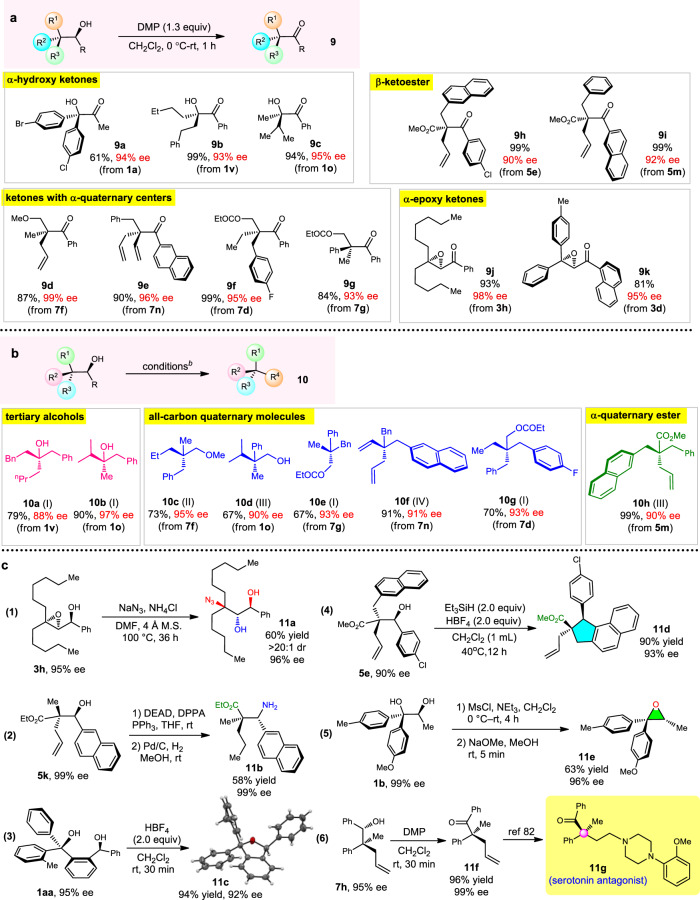
Various further transformations. **a** Dess-Marin periodinane (DMP) oxidation. **b** OH group removal. Conditions I: Raney Ni (3 equiv), EtOH, reflux, 12 h. Conditions II: CF_3_CO_2_H (8 equiv), CH_2_Cl_2_, rt, 24 h; then H_2_, Pd/C (10 %), EtOAc, rt, 12 h. Conditions III: HBF_4_ (2 equiv), Et_3_SiH (2 equiv), CH_2_Cl_2_, 40 °C, 3 h. Conditions IV: Et_3_SiH (2.5 equiv), CF_3_CO_2_H (6 equiv), CH_2_Cl_2_, 0 °C −rt, 12 h. **c** Various other transformations.

Having obtained a large number of enantioenriched molecules with quaternary and tetrasubstituted stereocenters and secondary alcohol units, we commenced completing the final purpose of getting the molecule types shown in Fig. 1a. To our pleasure, the readily transformable feature of the OH group makes further transformations simple. As shown in Fig. 5a, using easily operable Dess-Marin periodinane (DMP) oxidation conditions, α-hydroxy ketones with α,α-diaryl groups (**9a**), α,α-dimethylene substituents (**9b**), and bulky α-substituent (**9c**) were all concisely produced without any erosion of the enantiopurity. Then a variety of ketones with all-carbon α-quaternary centers were also released with 93–99% ee (Fig. 5b, **9d**–**9g**), and **9f** is a ketone with three different methylene groups. Furthermore, β-ketoesters with quaternary stereocenters are highly valuable building blocks in complex molecule synthesis, but in most cases, the α-functionalization of α-branched β-ketoesters cannot tolerate two bulky α-substituents (i.e., bulkier than a methyl group), which significantly limits the scope of this field^81^. In this study, ketoesters **9h** and **9i** bearing bulky substituents can also be easily obtained, thus complementing the drawbacks caused by the direct asymmetric catalysis. Additionally, the reaction could provide epoxy ketone bearing two aryl or two alkyl groups with minimal structural distinctions (Fig. 5b, **9j** and **9k**). Moreover, the secondary OH can be easily removed under reductive conditions, affording tertiary alcohols with three different alkyl groups (Fig. 5b, **10a** and **10b**). Similarly, enantioenriched molecules with all-carbon quaternary stereocenters such as **10c**–**10g** were also gotten with excellent 90–95% ee (Fig. 5b, **10c**–**10g**); noteworthy is that **10g** has four distinct methylene substituents, which is almost inaccessible via conventional methods. Finally, ester **10h** having three α-methylene substituents was also generated in quantitative yield with 90% ee, which is also a very challenging target using other protocols.

Besides the OH-oxidation and removal reactions, the enantiopure substances obtained in this study can also participate in a series of useful further transformations. For instance, the ring-opening of **3h** by NaN_3_ allowed access to 3-azido-1,2-diol **11a** with excellent diastereo- and enantioselectivity (Fig. 5c, eq 1), and β-amino acid derivative **11b** could be formed through a formal replacement of OH by NH_2_ group (Fig. 5c, eq 2). Moreover, **1aa** was readily transferred to 1,3-dihydroisobenzofuran **11c** in 94% yield with 92% ee (Fig. 5c, eq 3), and methyl ester **11d** with a cyclopenta[a]naphthalene unit was delivered by a formal Friedel-Crafts alkylation of **5e** (Fig. 5c, eq 4). The absolute configuration of **11c** was confirmed by single-crystal X-ray structure analysis (Supplementary Data 6). Diaryl epoxide **11e** was formed from **1b** via a two-step method (Fig. 5c, eq 5), and the formal synthesis of serotonin antagonist **11g** can be easily realized from **7h** (Fig. 5c, eq 6)^82^.

## Discussion

We have systematically demonstrated the rationale, scope, and applications of the strategy of kinetic resolution of auxiliary adjacent alcohol (KRA*). The combination of diastereoselective secondary alcohol synthesis and the following kinetic resolution has been exploited as a powerful strategy that can satisfactorily complement the limitations confronted by current direct asymmetric catalytic methods. The study shows a general scope in accessing enantioenriched molecules with various tetrasubstituted carbon stereocenters such as tertiary alcohols, epoxides, esters, quaternary methane derivatives, ketones, α-hydroxy ketones, epoxy ketones, β-ketoesters, etc., including those with two, three, and even four sterically and electronically unbiased substituents (96 examples). A detailed survey has shown that a vast majority of the products obtained via KRA* in this work cannot be gotten using either direct asymmetric catalysis or starting from enantiopure chiral chemicals. Moreover, besides the broad substrate scope and high resolution efficiency, the protocol is also featured by its very mild conditions, being able to scale up, and using readily available catalysts. These features guarantee the further application of KRA* in both academic and industrial communities. The study also enriches the toolbox during bioactive molecule synthesis and drug discovery, and represents a development tendency in the field of kinetic resolution. Studies on further applications of this strategy in accessing highly valuable enantioenriched molecules are ongoing in our group.

## Methods

### General

For ^1^H and ^13^C nuclear magnetic resonance (NMR) spectra of compounds in this manuscript and details of the synthetic procedures as well as more reaction conditions screening, see Supplementary Information.

### General procedures for the kinetic resolution of diols 1a–1l, 1za

To a flask containing **1a** (342 mg, 1.0 mmol), (*S*)-HBTM (10.65 mg, 0.04 mmol) and anhydrous Na_2_SO_4_ (200 mg) was added CHCl_3_ (5 mL) at 0 °C. After dissolvation of all compounds, (EtCO)_2_O (69.2 μL, 0.54 mmol) was added to the clear solution. After stirring for 12 h at 0 °C, solvent was removed under reduced pressure, the crude product was purified with a silica gel column chromatograghy using a mixture of hexane and ethyl acetate (10:1 v/v) as eluent gave **2a** (171 mg, 43% yield) as a white solid and **1a** (164 mg, 48% yield) as a white solid.

### General procedures for the kinetic resolution of diols 1m–1z, 1aa–1ad, 3a–3k, 5a–5n, 7a–7l

Catalyst (*S*)-BTM (2.5 mg, 0.01 mmol) and 4 Å molecular sieve (50 mg) were added to a Schlenk tube at room temperature under argon atmosphere. A solution of *rac*-**1a** (49.6 mg, 0.2 mmol) and ^*i*^Pr_2_NEt (16.5 μL, 0.1 mmol) in distilled CHCl_3_ (1 mL) was added via syringe. After stirring for 10 min at 0 °C, (EtCO)_2_O (12.8 μL, 0.1 mmol) was added to the reaction via microsyringe. After stirred at 0 °C for 11 h, the reaction mixture was diluted with petroleum ether, and then purified by flash chromatography sing a mixture of hexane and ethyl acetate (20:1 v:v) as eluent to afford the products **2m** (23.5 mg, 50% yield) and **1m** (18 mg, 50% yield).

## Supplementary information

Supplementary Information

Description of Additional Supplementary Files

Supplementary Data 1

Supplementary Data 2

Supplementary Data 3

Supplementary Data 4

Supplementary Data 5

Supplementary Data 6

## Data Availability

X-ray structural data for **1s** (CCDC 2026917), **2ac** (2026918), **2j** (CCDC 2026919), **4d** (CCDC 2026920), **7k** (CCDC 2026921), and **11c** (CCDC 2026922) can be obtained free of charge from the Cambridge Crystallographic Data Centre (CCDC) via http://www.ccdc.cam.ac.uk/data_request/cif. All other experimental data are available in the main text or the supplementary materials, and also are available from the corresponding author upon reasonable request.
