# Supplementary material for: Access to enantioenriched compounds bearing challenging tetrasubstituted stereocenters via kinetic resolution of auxiliary adjacent alcohols
Source: Nat Commun. 2021 Jun 18;12:3735. doi: 10.1038/s41467-021-23990-4 (PMC8213810; doi:10.1038/s41467-021-23990-4)
Supplement: Supplementary file 1 — Supplementary Information [file 41467_2021_23990_MOESM1_ESM.pdf]

Supplementary Information

Access to Enantioenriched Compounds Bearing Challenging Tetrasubstituted Stereocenters via Kinetic Resolution of Auxiliary Adjacent Alcohols

Shengtong Niu,† Hao Zhang,† Weici Xu,* Prasanta Ray Bagdi, Guoxiang Zhang, Jinggong Liu, Shuang Yang, and Xinqiang Fang*

State Key Laboratory of Structural Chemistry, and Key Laboratory of Coal to Ethylene Glycol and Its Related Technology, Center for Excellence in Molecular Synthesis, Fujian Institute of Research on the Structure of Matter, University of Chinese Academy of Sciences, Fuzhou 350100, China.

†These authors contributed equally to this work.
*Correspondence to: xqfang@fjirsm.ac.cn

Table of Contents

**I**	Supplementary Methods	S3
**II**	X-ray crystallographic analysis	S3
**III**	Typical procedures for the preparation of substrates	S10
**IV**	General procedure for the kinetic resolution reaction	S17
**V**	Procedures for the derivatizations of products	S18
**VI**	Characterizations of new compounds	S22
**VII**	^{1}H NMR and ^{13}C NMR spectra of substrates and products	S84
**VIII**	HPLC spectra for ee determination	S251
**IX**	Limitation of State-of-Art Asymmetric Synthesis of the Enantioenriched Products Obtained in This Study	S418

I. Supplementary Methods

Commercially available materials were used as received, unless otherwise noted, all reactions and manipulations involving air- or moisture-sensitive compounds were performed using standard Schlenk technique. All solvents were purified and dried using standard procedures. Proton nuclear magnetic resonance (^{1}H NMR) spectra were recorded on a Bruker AVANCE III HD400 (400 MHz) spectrometer and a JEOL ECZ600S (600 MHz). Chemical shifts were recorded in parts per million (ppm, δ) relative to tetramethylsilane (δ = 0.00 ppm) or chloroform (δ = 7.26 ppm). ^{1}H NMR splitting patterns are designated as singlet (s), doublet (d), triplet (t), quartet (q), dd (doublet of doublets), m (multiplet), and etc. All first-order splitting patterns were assigned on the basis of the appearance of the multiplet. Splitting patterns that could not be easily interpreted are designated as multiplet (m) or broad (br). Carbon nuclear magnetic resonance (^{13}C NMR) spectra were recorded on a Bruker AVANCE III HD400 (400 MHz) (100 MHz) spectrometer and a JEOL ECZ600S (600 MHz). High resolution mass spectral analysis (HRMS) was performed on Thermo Fisher Q Exactive Plus Hybrid Quadrupole-Orbitrap Mass Spectrometer. The determination of *e.e.* was performed via chiral HPLC analysis using Shimadzu LC-20AD HPLC workstation. X-ray crystallography analysis was performed on Agilent SuperNova X-ray diffractionmeter. Optical rotations were measured using a 1 mL cell with a 5 dm path length on an INESA SGW-1 polarimeter and are reported as follows: $[\alpha]_D^{rt}$ (c in g per 100 mL solvent). Analytical thin-layer chromatography (TLC) was carried out on WFH-203 F254 pre-coated silica gel plate (0.2 mm thickness). Visualization was performed using a UV lamp or 2,4-Dinitrophenylhydrazine or potassium permanganate stain.

II. X-ray crystallographic analysis

Method for single crystals cultivation: **2j** (20 mg) was dissolved in dichloromethane/petroleum ether (v:v = 1:10, 2 mL) in a vial at room temperature. The vial was properly sealed with parafilm and kept at room temperature to allow the slow evaporation of the solvents until a single crystal was obtained.

Supplementary Table 1. Crystal data and structure refinement for **2j**.

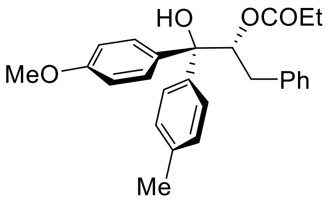 **2j**	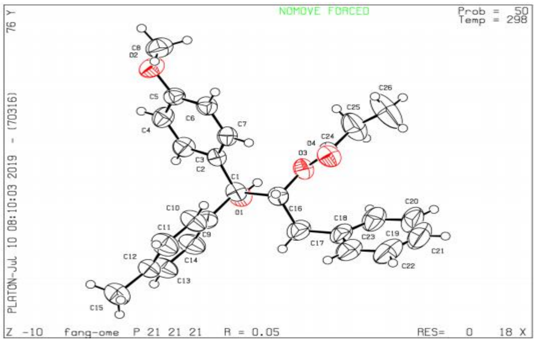
Identification code	2j

Empirical formula	$C_{26}H_{28}O_4$
Formula weight	404.48
Temperature/K	298.43(10)
Crystal system	orthorhombic
Space group	$P2_12_12_1$
a/Å	5.8152(2)
b/Å	18.8885(11)
c/Å	20.5975(11)
α/°	90
β/°	90
γ/°	90
Volume/Å^3	2262.44(19)
Z	4
ρ_{calc}g/cm^3	1.187
μ/mm^{-1}	0.405
F(000)	864.0
Crystal size/mm^3	0.12 × 0.11 × 0.1
Radiation	GaKα (λ = 1.3405)
2Θ range for data collection/°	5.52 to 112.652
Index ranges	$-4 \le h \le 6$, $-23 \le k \le 23$, $-25 \le l \le 23$
Reflections collected	10818
Independent reflections	4359 [R_{int} = 0.0353, R_{sigma} = 0.0499]
Data/restraints/parameters	4359/0/275
Goodness-of-fit on F^2	1.024
Final R indexes [I>=2σ (I)]	R_1 = 0.0498, wR_2 = 0.1055
Final R indexes [all data]	R_1 = 0.0825, wR_2 = 0.1182
Largest diff. peak/hole / e Å^{-3}	0.14/-0.16
Flack parameter	0.18(18)

Method for single crystals cultivation: **1s** (10 mg) was dissolved in dichloromethane/petroleum ether (v:v = 1:10, 2 mL) in a vial at room temperature. The vial was properly sealed with parafilm and kept at room temperature to allow the slow evaporation of the solvents until a single crystal was obtained.

Supplementary Table 2. Crystal data and structure refinement for **1s**.

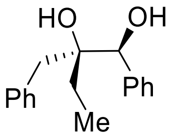**1s**	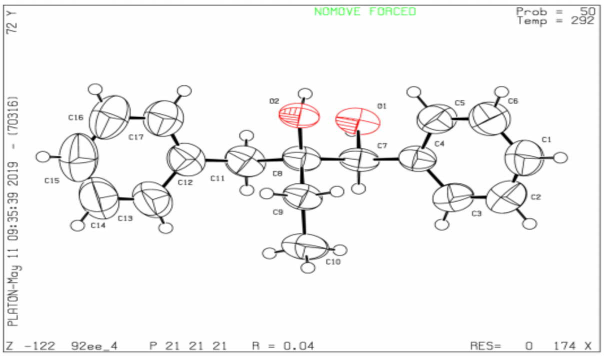

Identification code	1s
Empirical formula	$C_{17}H_{20}O_2$
Formula weight	256.35
Temperature/K	292.06(10)
Crystal system	orthorhombic
Space group	$P2_12_12_1$
a/Å	5.15638(14)
b/Å	16.8734(4)
c/Å	17.0315(5)
α/°	90
β/°	90
γ/°	90
Volume/Å^3	1481.84(7)
Z	4
ρ_{calc}g/cm^3	1.1490
μ/mm^{-1}	0.580
F(000)	553.7
Crystal size/mm^3	0.1 × 0.05 × 0.01
Radiation	Cu Kα (λ = 1.54184)
2Θ range for data collection/°	7.38 to 147.96
Index ranges	$-5 \le h \le 6$, $-19 \le k \le 20$, $-20 \le l \le 16$
Reflections collected	6817
Independent reflections	2914 [R_{int} = 0.0376, R_{sigma} = 0.0468]
Data/restraints/parameters	2914/0/175
Goodness-of-fit on F^2	0.961
Final R indexes [I>=2σ (I)]	R_1 = 0.0416, wR_2 = 0.1322
Final R indexes [all data]	R_1 = 0.0532, wR_2 = 0.1457
Largest diff. peak/hole / e Å^{-3}	0.11/-0.11
Flack parameter	0.2(4)

Method for single crystals cultivation: **2ac** (15 mg) was dissolved in ethyl acetate/petroleum ether (v:v = 1:10, 2 mL) in a vial at room temperature. The vial was

properly sealed with parafilm and kept at room temperature to allow the slow evaporation of the solvents until a single crystal was obtained.

Supplementary Table 3. Crystal data and structure refinement for **2ac**.

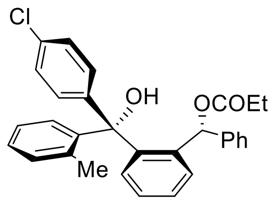

2ac

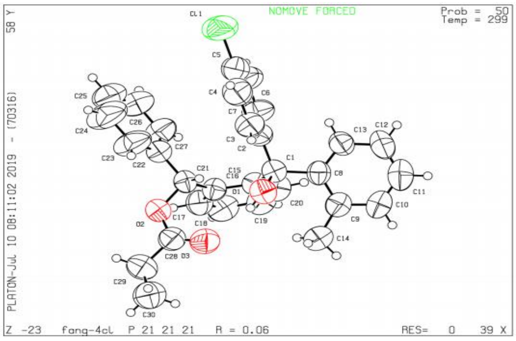

Identification code	2ac
Empirical formula	$C_{30}H_{27}ClO_3$
Formula weight	470.96
Temperature/K	298.50(10)
Crystal system	orthorhombic
Space group	$P2_12_12_1$
a/Å	8.5783(10)
b/Å	11.8604(16)
c/Å	25.331(4)
α/°	90
β/°	90
γ/°	90
Volume/Å^3	2577.2(6)
Z	4
ρ_{calc}g/cm^3	1.214
μ/mm^{-1}	0.994
F(000)	992.0
Crystal size/mm^3	0.12 × 0.11 × 0.1
Radiation	GaKα (λ = 1.3405)
2Θ range for data collection/°	6.066 to 121.906
Index ranges	$-11 \leq h \leq 5$, $-15 \leq k \leq 15$, $-29 \leq l \leq 32$
Reflections collected	16498
Independent reflections	5694 [R_{int} = 0.0329, R_{sigma} = 0.0321]
Data/restraints/parameters	5694/0/311
Goodness-of-fit on F^2	1.023
Final R indexes [I>=2σ (I)]	R_1 = 0.0611, wR_2 = 0.1728
Final R indexes [all data]	R_1 = 0.0979, wR_2 = 0.2146
Largest diff. peak/hole / e Å^{-3}	0.24/-0.20

Flack parameter	0.012(13)

Method for single crystals cultivation: **4d** (10 mg) was dissolved in dichloromethane/ petroleum ether (v:v = 1:10, 2 mL) in a vial at room temperature. The vial was properly sealed with parafilm and kept at room temperature to allow the slow evaporation of the solvents until a single crystal was obtained.

Supplementary Table 4. Crystal data and structure refinement for **4d**.

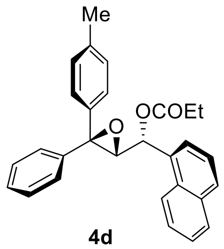

4d

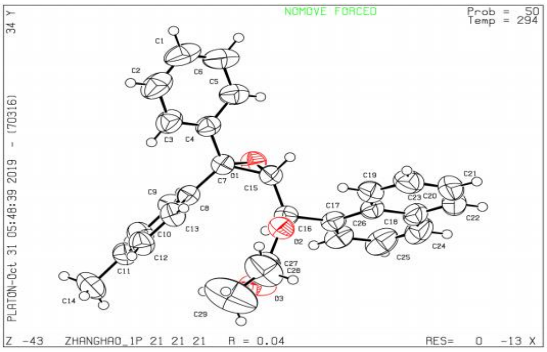

Identification code	4d
Empirical formula	$C_{29}H_{26}O_3$
Formula weight	422.53
Temperature/K	293.68(11)
Crystal system	orthorhombic
Space group	$P2_12_12_1$
a/Å	8.41923(11)
b/Å	13.67529(19)
c/Å	20.4353(3)
α/°	90
β/°	90
γ/°	90
Volume/Å^3	2352.83(5)
Z	4
ρ_{calc}g/cm^3	1.1927
μ/mm^{-1}	0.601
F(000)	898.8
Crystal size/mm^3	0.1 × 0.05 × 0.05
Radiation	Cu Kα (λ = 1.54184)
2Θ range for data collection/°	7.78 to 148.02
Index ranges	$-8 \le h \le 10$, $-16 \le k \le 13$, $-20 \le l \le 25$
Reflections collected	12152
Independent reflections	4627 [R_{int} = 0.0252, R_{sigma} = 0.0277]
Data/restraints/parameters	4627/0/291

Goodness-of-fit on F^2	1.052
Final R indexes [I>=2σ (I)]	R_1 = 0.0423, wR_2 = 0.1085
Final R indexes [all data]	R_1 = 0.0475, wR_2 = 0.1132
Largest diff. peak/hole / e $Å^{-3}$	0.18/-0.20
Flack parameter	0.1(2)

Method for single crystals cultivation: **7k** (15 mg) was dissolved in ethyl acetate/ petroleum ether (v:v = 1:10, 2 mL) in a vial at room temperature. The vial was properly sealed with parafilm and kept at room temperature to allow the slow evaporation of the solvents until a single crystal was obtained.

Supplementary Table 5. Crystal data and structure refinement for **7k**.

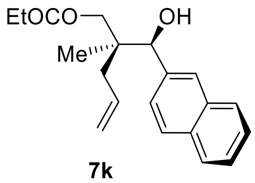

7k

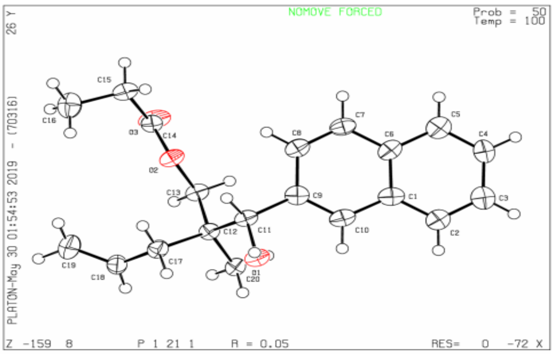

Identification code	7k
Empirical formula	$C_{20}H_{24}O_3$
Formula weight	312.39
Temperature/K	100.00(10)
Crystal system	monoclinic
Space group	$P2_1$
a/Å	8.1763(5)
b/Å	7.5690(3)
c/Å	14.4058(8)
α/°	90.00
β/°	104.121(6)
γ/°	90.00
Volume/$Å^3$	864.58(8)
Z	2
ρ_{calc}g/cm^3	1.200
μ/mm^{-1}	0.631
F(000)	336.0
Crystal size/mm^3	0.14 × 0.12 × 0.11
Radiation	CuKα (λ = 1.54178)
2Θ range for data collection/°	6.32 to 146.78

Index ranges	$-7 \le h \le 10$, $-9 \le k \le 9$, $-17 \le l \le 17$
Reflections collected	7101
Independent reflections	3075 [R_{int} = 0.0228, R_{sigma} = 0.0211]
Data/restraints/parameters	3075/1/219
Goodness-of-fit on F^2	1.057
Final R indexes [I>=2σ (I)]	R_1 = 0.0539, wR_2 = 0.1421
Final R indexes [all data]	R_1 = 0.0551, wR_2 = 0.1434
Largest diff. peak/hole / e $Å^{-3}$	0.51/-0.27
Flack parameter	-0.2(3)

Method for single crystals cultivation: **11c** (15 mg) was dissolved in ethyl acetate/ petroleum ether (v:v = 1:10, 2 mL) in a vial at room temperature. The vial was properly sealed with parafilm and kept at room temperature to allow the slow evaporation of the solvents until a single crystal was obtained.

Supplementary Table 6. Crystal data and structure refinement for **11c**.

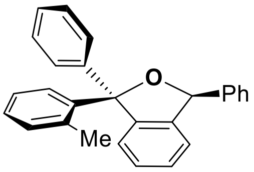

11c

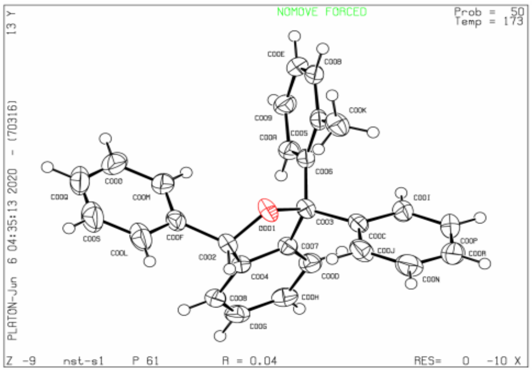

Identification code	11c
Empirical formula	$C_{27}H_{22}O$
Formula weight	362.44
Temperature/K	172.99(10)
Crystal system	hexagonal
Space group	$P6_1$
a/Å	23.5381(3)
b/Å	23.5381(3)
c/Å	6.60429(8)
α/°	90
β/°	90
γ/°	120
Volume/$Å^3$	3168.82(8)
Z	6
ρ_{calc} g/cm^3	1.140
μ/mm^{-1}	0.520

F(000)	1152.0
Crystal size/mm^3	0.1 × 0.01 × 0.01
Radiation	CuKα (λ = 1.54184)
2Θ range for data collection/°	7.512 to 148.348
Index ranges	-28 ≤ h ≤ 28, -29 ≤ k ≤ 28, -8 ≤ l ≤ 7
Reflections collected	26100
Independent reflections	4242 [R_{int} = 0.0588, R_{sigma} = 0.0313]
Data/restraints/parameters	4242/1/254
Goodness-of-fit on F^2	1.081
Final R indexes [I>=2σ (I)]	R_1 = 0.0359, wR_2 = 0.0941
Final R indexes [all data]	R_1 = 0.0369, wR_2 = 0.0952
Largest diff. peak/hole / e Å^{-3}	0.15/-0.18
Flack parameter	-0.06(15)

III. Typical procedures for the preparation of substrates

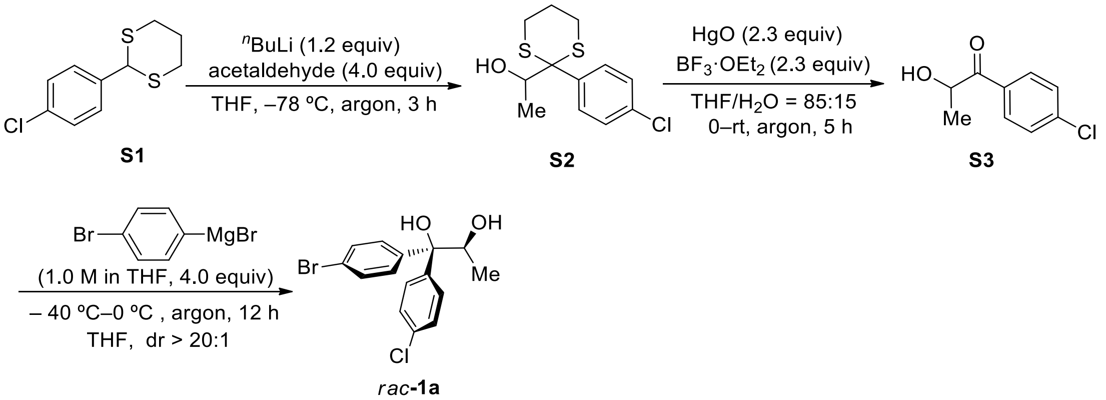

Supplementary Figure 1. General procedures for the preparation of substrates **1a**–**1l**, **1za**

To a solution of **S1** (1.15 g, 5 mmol) in THF (20 mL) was added ^{n}BuLi (2.5 M in hexanes, 2.4 mL, 6 mmol) dropwise at –78 ℃ under argon atmosphere. After stirring at –78 ℃ for 1 h, acetaldehyde (1.1 mL, 20 mmol) was added to the reaction mixture dropwise at –78 ℃ under argon atmosphere. The reaction mixture was stirred at –78 ℃ for another 2 h. After the starting material was consumed completely, water and brine were added and warmed to room temperature. The reaction mixture was stirred at room temperature for 10 min and extracted with ethyl acetate. The organic layer was then separated, dried over anhydrous Na_2SO_4 and concentrated using rotary evaporation. Purification of crude product through a silica gel column using a mixture of petroleum ether and ethyl acetate (10:1 v/v) as eluent gave **S2** (1.37 g, 88% yield) as a yellow oil.

To a solution of BF_3 Et$_2$O (1.24 mL, 10.12 mmol) in THF/H_2O = 85:15 (10 mL) was added HgO (2.19 g, 10.12 mmol) at 0 ℃ under argon atmosphere. After stirring at 0 ℃ for 5 min, the solution of **S2** (1.37 g, 4.4 mmol) in THF/H_2O = 85:15 (20 mL) was added to the reaction mixture. After completion stirring for 5 h at room

temperature, CH_2Cl_2 was added and the mixture was stirred for another 5 min. The solvent was removed after filtration of reaction mixture through a pad of celite and Na_2SO_4. Then residue of crude product was purified with a silica gel column chromatograghy using a mixture of petroleum ether and ethyl acetate (10:1 v/v) as eluent gave **S3** (634 mg, 78% yield) as a clear oil.

The freshly prepared para-bromo-phenylmagnesium bromide (1.0 M in THF, 13.73 mL, 13.73 mmol) was added dropwise to the solution of **S3** (634 mg, 3.43 mmol) in THF (20 mL) at –40 ℃ under argon atmosphere. The mixture was stirred for 5 h at –40 ℃ and then warmed to 0 ℃. After stirring for another 12 h at 0 ℃, the reaction was quenched with saturated aqueous NH_4Cl and the temperature was warmed to room temperature and stirred for 10 min at room temperature. Extraction of two layers mixture with ethyl acetate and the organic layers were then separated, dried over anhydrous Na_2SO_4 and concentrated using rotary evaporation. Purification of crude product through a silica gel column neutralized with Et_3N using a mixture of petroleum ether and ethyl acetate (10:1 v/v) as eluent gave *rac*-**1a** (820 mg, 70% yield) as a yellow oil.

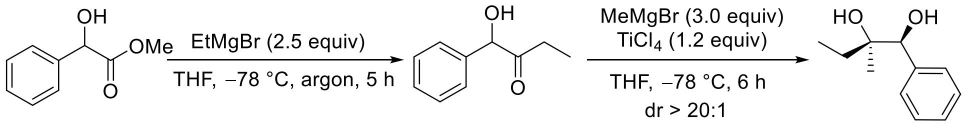

Supplementary Figure 2. General procedures for the preparation of substrates **1m**–**1z**

To a solution of **S4** (1.66 g, 10 mmol) in dry THF (30 mL) was added ethylmagnesium bromide (1.0 M in THF, 25 mL, 25 mmol) dropwise at –78 ℃ under argon atmosphere. After stirring at –78 ℃ for 5 h. The reaction was quenched with saturated aqueous NH_4Cl and extracted with ethyl acetate for three times. The combined organic layers were washed with brine, dried over Na_2SO_4, filtered, and concentrated under vacuum. Purification of crude product through a silica gel column using a mixture of petroleum ether and ethyl acetate (20:1 v/v) as eluent gave **S5** (820 mg, 50% yield) as colorless oil.

To a solution of **S5** (410 mg, 2.5 mmol) in dry THF (15 mL) was added titanium tetrachloride (0.33 mL, 3.0 mmol) dropwise at –78 ℃ under argon atmosphere. After stirring at –78 ℃ for 10 min, methylmagnesium bromide (3.0 M in THF, 2.5 mL, 7.5 mmol) was added to the reaction mixture dropwise at –78 ℃ under argon atmosphere. After stirring at –78 ℃ for 6 h. The reaction was quenched with saturated aqueous NH_4Cl and extracted with ethyl acetate for three times. The combined organic layers were washed with brine, dried over sodium sulfate, filtered, and concentrated under vacuum. Purification of crude product through a silica gel column using a mixture of petroleum ether and ethyl acetate (20:1 v/v) as eluent gave *rac*-**1m** (320 mg, 71% yield) as white solid.

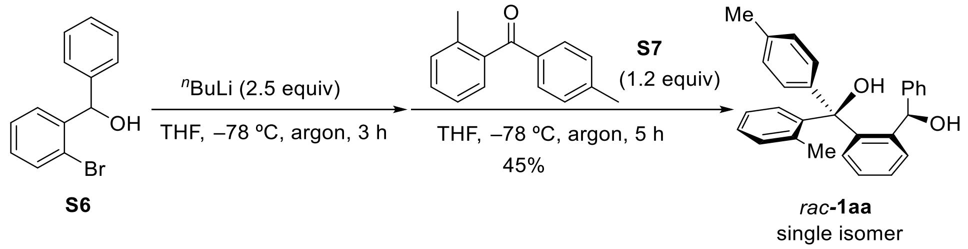

Supplementary Figure 3. General procedures for the preparation of substrates **1aa**–**1ad**

To a solution of **S6** (1.30 g, 5.0 mmol) in dry THF (20 mL) was added ^{n}BuLi (2.5 M in hexanes, 5.0 mL, 12.5 mmol) dropwise at –78 ℃ under argon atmosphere stirring at –78 ℃ for 3 h. After **S7** (1.26 g, 6.0 mmol) solution in dry THF (5.0 mL) was added to the reaction mixture dropwise at –78 ℃ under argon atmosphere. The reaction mixture was stirred at –78 ℃ for another 5 h. The reaction was quenched with saturated aqueous NH_4Cl and extracted with ethyl acetate for three times. The combined organic layers were washed with brine, dried over sodium sulfate, filtered, and concentrated under vacuum. Purification of crude product through a silica gel column using a mixture of petroleum ether and ethyl acetate (20:1 v/v) as eluent gave *rac*-**1aa** (880 mg, 45% yield) as white solid.

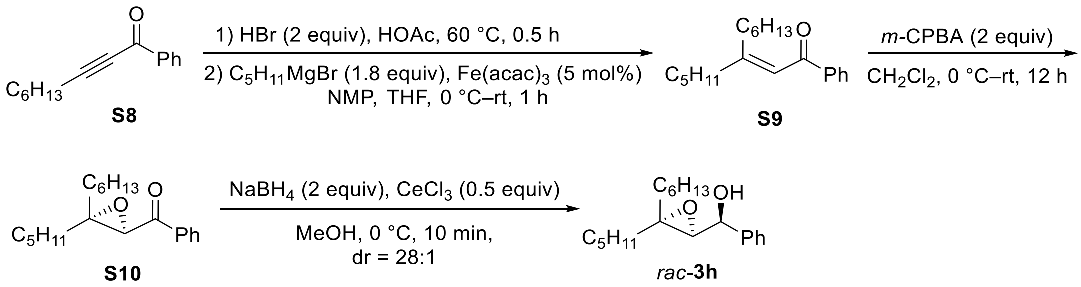

Supplementary Figure 4. General procedures for the preparation of substrates **3a**–**3k**, **7i**, **7j**

To a solution of **S8** (2.0 g, 9.16 mmol) in CH_3COOH (25 mL) was added HBr (40%, 2.8 mL, 19.32 mmol) at room temperature. After stirring for 1 h at 60 ℃, the reaction was quenched with aqueous $NaHCO_3$ and extracted with CH_2Cl_2 (3 times). The organic layer was then separated, dried over Na_2SO_4 and concentrated using rotary evaporation. Purification of crude product through a silica gel column using a mixture of petroleum ether and ethyl acetate (100:1 v/v) as eluent gave products (1.26 g, 43% yield) as a yellow oil.

$CH_3(CH_2)_4MgBr$ (0.45 M in THF, 12 mL, 5.4 mmol) was slowly added to a stirred solution of $Fe(acac)_3$ (53 mg, 0.15 mmol), NMP (2.6 mL, 27.0 mmol) and the first steps pruducts (886 mg, 3.0 mmol) in THF (15 mL) was added to the above mixture at 0 ℃. The mixture was stirred at room temperature for 1 h and then quenched with HCl (1 M) and the phases separated. The aqueous layer was extracted

with Et_2O and washed with aqueous $NaHCO_3$. The combined organic layers were dried over Na_2SO_4, and concentrated under reduced pressure. The residue was purified by flash chromatography using a mixture of petroleum ether and ethyl acetate (50:1–20:1 v:v) to give the product **S9** (695 mg, 81% yield).

A solution of **S9** (100 mg, 0.35 mmol) in CH_2Cl_2 (6 mL) was cooled to 0 ℃. *m*-CPBA (142 mg, 0.7 mmol) was added into the solution slowly. After stirring for 12 h at room temperature, the reaction was cooled to 0 ℃ and diluted with CH_2Cl_2 (10 mL), and then washed by saturated aqueous $NaHCO_3$ for two times. The organic layers were dried by Na_2SO_4 and concentrated under reduced pressure. The residue was purified by flash chromatography using a mixture of petroleum ether and ethyl acetate (50:1–20:1 v:v) to give the product **S10** (72 mg, 68% yield).

A solution of **S10** (200 mg, 0.66 mmol) in MeOH (11 mL) was cooled to 0 ℃. $CeCl_3$ (81 mg, 0.33 mmol) was added into the solution slowly. After stirring for 3 min at 0 ℃, $NaBH_4$ (50 mg, 1.32 mmol) was added into the solution slowly and continiue to stirr for 15 min at the same temperature. Then the reaction was diluted with ethyl acetate (20 mL), and then washed by saturated aqueous NaCl for two times. The organic layers were dried by Na_2SO_4 and concentrated under reduced pressure. The residue was purified by flash chromatography using a mixture of petroleum ether and ethyl acetate (50:1–20:1 v:v) to give the product *rac*-**3h** (161 mg, 80% yield).

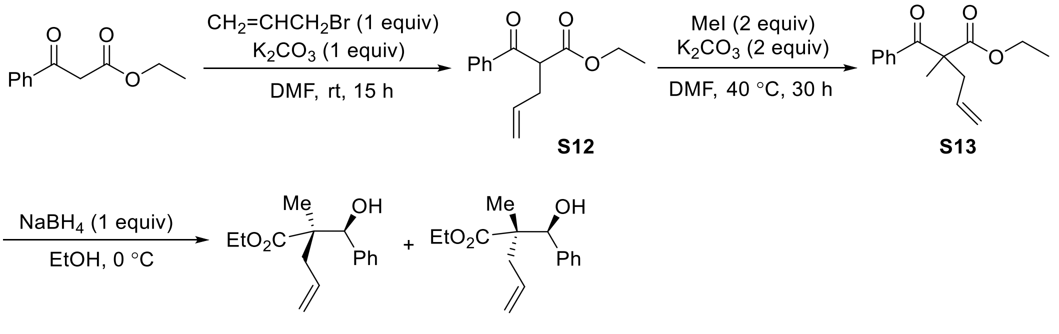

Supplementary Figure 5. General procedures for the preparation of substrates **5a**–**5o**

Potassium carbonate (2.76 g, 20.0 mmol) were dissolved in DMF (50 mL) at room temperature. Ethyl benzoylacetate (3.84 g, 20.0 mmol) and allyl bromide (2.42 g, 20.0 mmol) were added separately into the suspension at room temperature. After stirring for 15 h at room temperature, the reaction was diluted with ethyl acetate (250 mL), and then washed by saturated aqueous NaCl for three times. The organic layers were dried by Na_2SO_4 and concentrated under reduced pressure. The residue was purified by flash chromatography (petroleum ether/ethyl acetate, v:v = 100:1) to give the product **S12** (3.92 g, 95% yield).

Potassium carbonate (2.76 mg, 20.0 mmol) was dissolved in DMF (20 mL) at room temperature. **S12** (2.32 g, 10.0 mmol) and MeI (3.12 g, 20.0 mmol) were added separately into the suspension. After stirring for 30 h at 40 °C, the reaction was diluted with ethyl acetate (200 mL), and then washed by saturated aueous NaCl for three times. The organic layers were dried by Na_2SO_4 and concentrated under reduced

pressure. The residue was purified by flash chromatography using a mixture of petroleum ether and ethyl acetate (100:1 v:v) to give the product **S13** (1.60 g, 65% yield).

A solution of **S13** (1.23 g, 5.0 mmol) in EtOH (10 mL) was cooled to 0 °C. $NaBH_4$ (169 mg, 5.0 mmol) was added into the solution slowly. After stirring for 2 h at 0 °C, the reaction was diluted with ethyl acetate (100 mL), and then washed by saturated aqueous NaCl for two times. The organic layers were dried by Na_2SO_4 and concentrated under reduced pressure. The residue was purified by flash chromatography using a mixture of petroleum ether and ethyl acetate (50:1–20:1 v:v) to give the product *rac*-**5a** (645 mg, 52% yield) and *rac*-**5b** (533 mg, 43% yield).

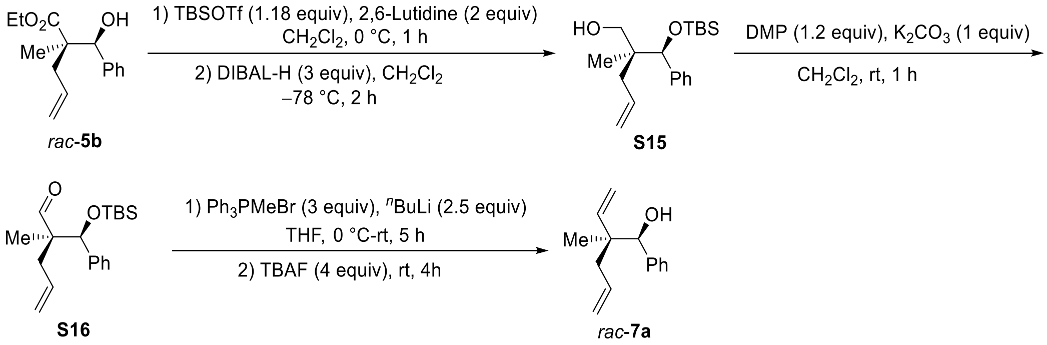

Supplementary Figure 6. General procedures for the preparation of substrates **7a**, **7e**, **7h**, **7l**, **7n**

A solution of *rac*-**5b** (0.42 g, 1.7 mmol) in CH_2Cl_2 (6 mL) was added 2,6-lutidene (0.4 mL, 3.4 mmoL) dropwised at 0 °C. TBSOTf (0.5 mL, 2.0 mmol) was slowly added into the reaction under same temperature. After stirring for 1 h at 0 °C, the reaction was diluted with CH_2Cl_2 (20 mL), and then washed by saturated aqueous NaCl for two times. The organic layers were dried by Na_2SO_4 and concentrated under reduced pressure. The residue was purified by flash chromatography using a mixture of petroleum ether and ethyl acetate (100:1 v:v) to give **S14** (0.56 g, 92% yield).

A solution of **S14** (0.56 g, 1.6 mmol) in CH_2Cl_2 (4.0 mL) was added DIBAL-H (1.0 M in hexanes, 4.8 mL, 4.8 mmoL) dropwised at −78 °C. After stirring for 1 h at −78 °C, the reaction was diluted with CH_2Cl_2 (20 mL) and then H_2O (0.19 mL), NaOH (0.19 mL, 15% in water) and H_2O (0.48 mL) was added in order. The mixture was stirred for 15 min at room temperature and then washed by saturated aqueous NaCl for two times. The organic layers were dried by Na_2SO_4 and concentrated under reduced pressure. The residue was purified by flash chromatography using a mixture of petroleum ether/ethyl acetate (20:1 v:v) to give **S15** (0.39 g, 76% yield).

To a 50 mL flame-dried flask was added **S15** (0.30 g, 0.94 mmol), CH_2Cl_2 (18 ml) and K_2CO_3 (0.13 g, 0.94 mmoL). The mixture was stirred for 3 min at room temperature and then was added Dess-Martin periodinane (0.48 g, 1.13 mmol). The reaction suspension was monitored by TLC until no **S15** remained. Then the reaction was quenched with aqueous $NaHCO_3$ solution, and then extracted with CH_2Cl_2. The combined organic layer was dried over Na_2SO_4, filtered and evaporated under reduced

pressure. The residue was purified by chromatography using a mixture of petroleum ether and ethyl acetate (20:1, v:v) to afford pure product **S16** (0.23 g, 77% yield).

A solution of Ph_3PMeBr (0.75 g, 2.13 mmoL) in THF (4 mL) was added ^{n}BuLi (2.5 M in hexanes, 0.7 mL, 1.78 mmoL) dropwised at 0 ℃ under argon atmosphere. The mixture was stirred for 1 h at 0 ℃ and then was added **S15** (0.23 g, 0.71 mmol) in THF (3 mL) dropwised in the same temperature. After stirring for 5 h at room temperature, the reaction was diluted with ethyl acetate (20 mL), and then washed by saturated aqueous NaCl for two times. The organic layers were dried by Na_2SO_4 and concentrated under reduced pressure. The residue was purified by flash chromatography using a mixture of petroleum ether and ethyl acetate (100:1 v:v) to give **S17** (0.20 g, 88% yield).

To a 25 mL flask was added **S17** (0.20 g, 0.62 mmol) and TBAF (1.0 M in THF, 2.5 mL, 2.5 mmol). After stirring for 4 h at room temperature, the reaction was diluted with ethyl acetate (20 mL), and then washed by saturated aqueous NaCl for two times. The organic layers were dried by Na_2SO_4 and concentrated under reduced pressure. The residue was purified by flash chromatography using a mixture of petroleum ether and ethyl acetate (20:1 v:v) to give *rac*-**7a** (0.11 g, 88% yield).

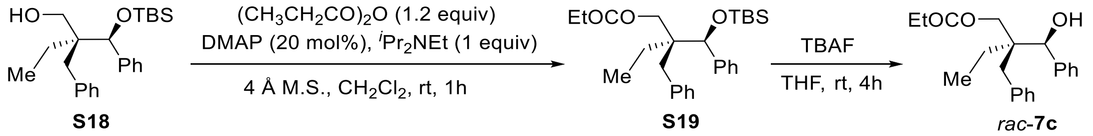

Supplementary Figure 7. General procedures for the preparation of substrates **7c**, **7d**, **7g**, **7k**

Catalyst DMAP (45.7 mg, 0.37 mmol) was added to a 25 mL flame-dried flask at room temperature. A solution of **S18** (0.72 g, 1.87 mmol), 4 Å molecular sieve (50 mg) and $^{i}Pr_2NEt$ (0.3 mL, 1.87 mmol) in distilled CH_2Cl_2 (9 mL) was added. $(EtCO)_2O$ (0.3 mL, 2.24 mmol) was added to the reaction. After stirred at room temperature for 1 h, the reaction mixture was concentrated under reduced pressure. The residue was purified by flash chromatography using a mixture of petroleum ether and ethyl acetate (20:1 v:v) to afford **S19** (0.65 g, 80% yield).

General route for synthesis of **S18** is same with synthesis of **S14**. General procedure to synthesize *rac*-**7c** from **S19** is same with the procedure from **S17** to *rac*-**7a**.

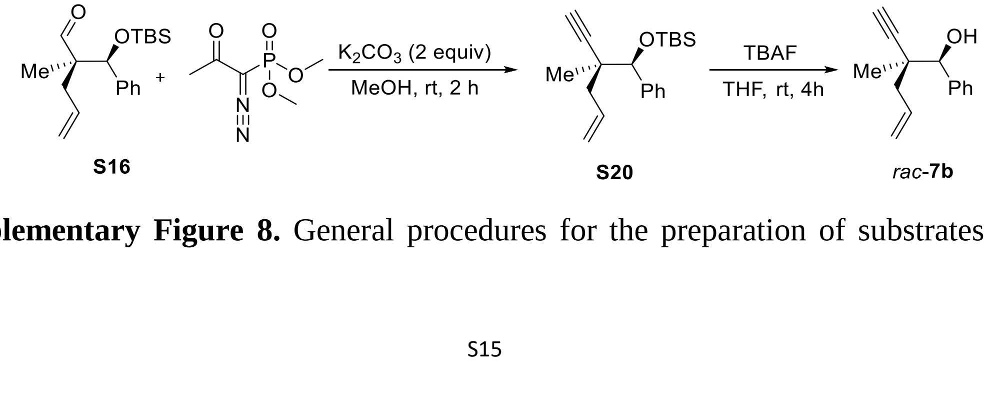

Supplementary Figure 8. General procedures for the preparation of substrates **7b**, **7m**

To a stirred solution of **S16** (0.25 g, 0.78 mmol) and dimethyl (1-diazo-2-oxopropyl) phosphonate (0.2 mL, 1.25 mmol) in dry MeOH (11 mL) was added K_2CO_3 (0.22 g, 1.56 mmol) at room temperature. The reaction suspension was monitored by TLC until no **S16** remained. Then the reaction mixture was diluted with water (20 mL), neutralized with 5% HCl (10 mL) and extracted with ethyl acetate (30 mL) for three times. The organic layers were dried by Na_2SO_4 and concentrated under reduced pressure. The crude residue was purified by flash chromatography using a mixture of petroleum ether and ethyl acetate (20:1 v:v) to give **S8a** (80 mg, 33% yield).

General procedure to synthesize *rac*-**7b** from **S20** is same with the procedure from **S17** to *rac*-**7a**.

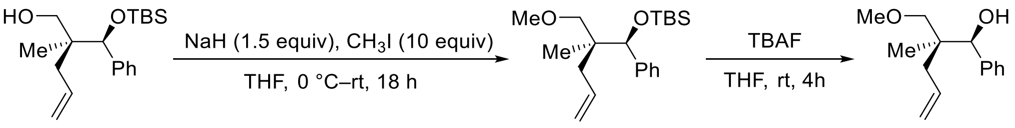

Supplementary Figure 9. General procedures for the preparation of substrates **7f**

A solution of **S15** (0.50 g, 1.56 mmoL) in THF (5.0 mL) was added NaH (60% dispersion in mineral oil, 94 mg, 2.34 mmol) at 0 ℃ under argon atmosphere. The mixture was stirred for 1 h at 0 ℃ and then was added CH_3I (1.0 mL, 15.6 mmol) dropwised in the same temperature. After stirring for 18 h at room temperature, the reaction was diluted with ethyl acetate (20 mL), and then washed by saturated aqueous NaCl for two times. The organic layers were dried by Na_2SO_4 and concentrated under reduced pressure. The residue was purified by flash chromatography using a mixture of petroleum ether and ethyl acetate (100:1 v:v) to give **S20** (0.93 g, 89% yield).

General procedure to synthesize *rac*-**7f** from **S21** is same with the procedure from **S17** to *rac*-**7a.**

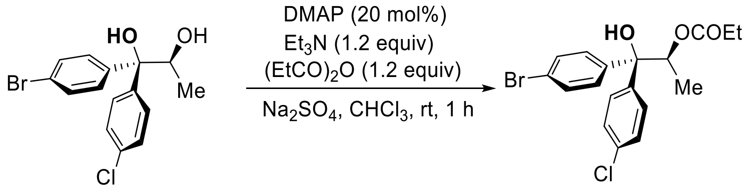

Supplementary Figure 10. General procedures for the preparation of racemic **2a**–**2ad**, **4a**–**4k**, **6a**–**6o**, **8a**–**8n**

To a flask containing **1a** (34.2 mg, 0.1 mmol), DMAP (2.4 mg, 0.2 mmol) and anhydrous Na_2SO_4 (20 mg) was added $CHCl_3$ (1.0 mL) at room temperature. After dissolvation of all compounds, Et_3N (16.7 µL, 1.2 mmol) was added to the clear solution and followed by addition of $(EtCO)_2O$ (15.4 µL, 1.2 mmol). After completion

of reaction, solvent was removed under reduced pressure, the crude product was purified with a silica gel column chromatograghy using a mixture of petroleum ether and ethyl acetate (10:1 v/v) as eluent to gave *rac*-**2a** (39.4 mg, 99% yield) as a white solid.

IV. General procedure for the kinetic resolution reaction

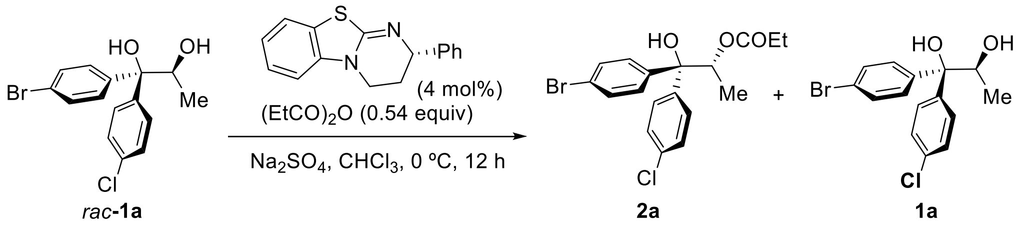

Supplementary Figure 11. General procedures for the kinetic resolution of diols **1a**–**1l**, **1za**

To a flask containing **1a** (342 mg, 1.0 mmol), (*S*)-HBTM (10.65 mg, 0.04 mmol) and anhydrous Na_2SO_4 (200 mg) was added $CHCl_3$ (5 mL) at 0 ℃. After dissolvation of all compounds, $(EtCO)_2O$ (69.2 μL, 0.54 mmol) was added to the clear solution. After stirring for 12 h at 0 ℃, solvent was removed under reduced pressure, the crude product was purified with a silica gel column chromatograghy using a mixture of hexane and ethyl acetate (10:1 v/v) as eluent gave **2a** (171 mg, 43% yield) as a white solid and **1a** (164 mg, 48% yield) as a white solid.

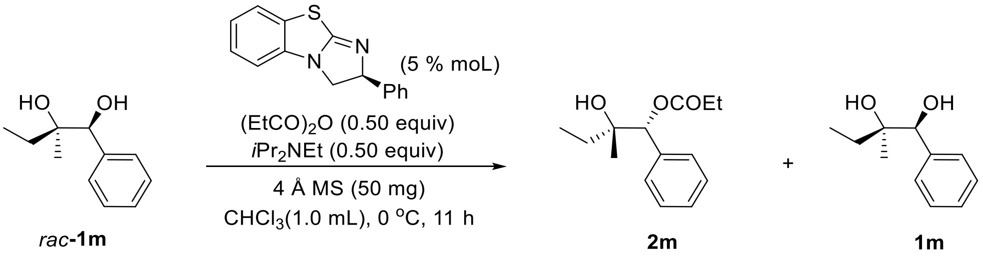

Supplementary Figure 12. General procedures for the kinetic resolution of diols **1m**–**1z**, **1aa**–**1ad**, **3a**–**3k**, **5a**–**5n**, **7a**–**7l**

Catalyst (*S*)-BTM (2.5 mg, 0.01 mmol) and 4 Å molecular sieve (50 mg) were added to a Schlenk tube at room temperature under argon atmosphere. A solution of *rac*-**1a** (49.6 mg, 0.2 mmol) and $^{i}Pr_2NEt$ (16.5 μL, 0.1 mmol) in distilled $CHCl_3$ (1 mL) was added via syringe. After stirring for 10 min at 0 ℃, $(EtCO)_2O$ (12.8 μL, 0.1 mmol) was added to the reaction via microsyringe. After stirred at 0 ℃ for 11 h, the reaction mixture was diluted with petroleum ether, and then purified by flash chromatography sing a mixture of hexane and ethyl acetate (20:1 v:v) as eluent to afford the products **2m** (23.5 mg, 50% yield) and **1m** (18 mg, 50% yield).

V. Procedures for the derivatizations of products

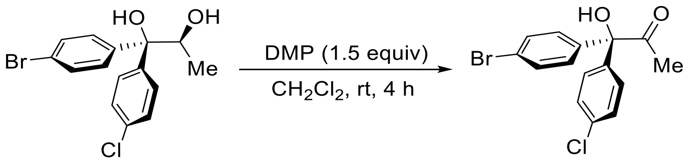

1a **9a**

Supplementary Figure 13. General procedures for the product derivatizations **9a**–**9k**

To a solution of **1a** (34.2 mg, 0.1 mmol, ee = 99%) in CH_2Cl_2 (1 mL) was added Dess-Martin periodinane (64 mg, 0.15 mmol) and stirred at room temperature for 4 h. The reaction was filtered through a celite column, washed with ethyl acetate, and then concentrated under reduced pressure. The residue was purified by flash chromatography using a mixture of petroleum and ethyl acetate (20:1 v:v) to give the product **9a** (20.7 mg, 61% yield, ee = 94%).

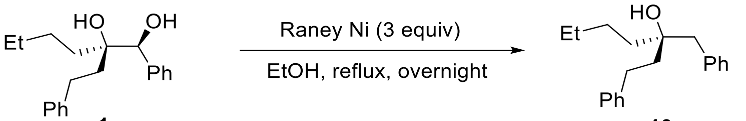

1v **10a**

Supplementary Figure 14. General procedures for the product derivatizations **10a**, **10b**, **10e**, **10g**

To a solution of **1v** (30 mg, 0.1 mmol, ee = 95%) in degassing EtOH in a Schlenk tube, Raney nickel (20 mg, 0.3 mmol) were added to at room temperature under argon atmosphere. After stirring for 10 h at 80 ℃. The reaction was filtered through a celite column, washed with ethyl acetate, and then concentrated under reduced pressure. The residue was purified by flash chromatography using a mixture of petroleum and ethyl acetate (20:1 v:v) to give the product **9a** (22.3 mg, 79% yield, ee = 88%).

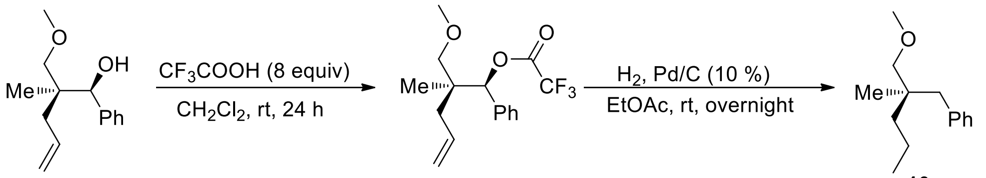

7e **7ea** **10c**

Supplementary Figure 15. General procedures for the product derivatizations **10c**

To a solution of **7e** (22 mg, 0.1 mmol, ee = 99%) in CH_2Cl_2 (1 mL) was added CF_3COOH (59 μL, 0.8 mmol) at room temperature. After stirring at room temperature for 24 h. The reaction was quenched with aqueous NH_4Cl and extracted with CH_2Cl_2 for three times.The organic layer was then separated, dried over Na_2SO_4 and concentrated using rotary evaporation. Purification of crude product through a silica

gel column using a mixture of petroleum ether and ethyl acetate (50:1 v/v) as eluent gave **7ea** (23.7 mg, 75% yield) as a colorless oil. Hydrogenation of **7ea** (15 mg, 0.05 mmol) was carried out overnight in ethyl acetate (0.8 mL) under H_2 (1 bars) in the presence of 10% Pd/C (8 mg). The mixture was filtered and the catalyst was washed with ethyl acetate. The filtrate and ethyl acetate washings were combined and evaporated to afford pure **10c** (15 mg, 97% yield, ee = 95%).

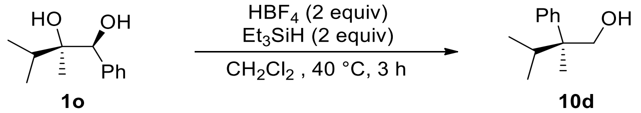

Supplementary Figure 16. General procedures for the product derivatizations **10d**, **10h**

1o (19.5 mg, 0.1 mmol, ee = 97%) was disolved in 1 mL CH_2Cl_2, employing a polypropylene vessel with a nitrogen atmosphere. Triethylsilane (23.3 mg, 0.2 mmol) was then introduced into reaction flask, followed by tetrafluoroboric acid (17.6 mg, 0.2 mmol). After stirring for 3 h at 40 ℃, the reaction was poured into a saturated aqueous Na_2CO_3 solution and was then extracted with Et_2O. The organic solution was concentrated and gave pure product **10d** (12 mg, 67% yield, ee = 90%).

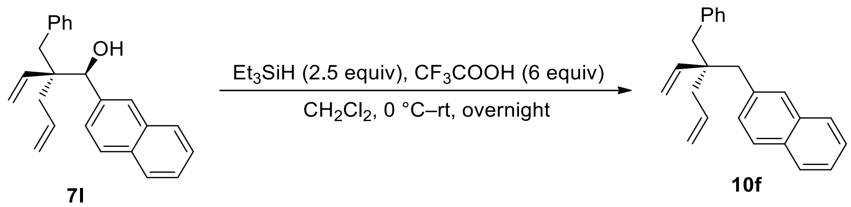

Supplementary Figure 17. General procedures for the derivatization of **10f**

To a solution of **7l** (33 mg, 0.1 mmol, ee = 96%) in CH_2Cl_2 (0.7 mL) was added CF_3COOH (44 μL, 0.6 mmol) and dimethylethylsilane (2.5 equiv) at 0 ℃. After stirring at rt for 10 h. The reaction was quenched with aqueous $NaHCO_3$ and extracted with CH_2Cl_2 for three times.The organic layer was then separated, dried over anhydrous sodium sulfate and concentrated using rotary evaporation. Purification of crude product through a silica gel column using a mixture of petroleum ether and ethyl acetate (50:1 v/v) as eluent gave **10f** (28.4 mg, 91% yield, ee = 91%) as a colorless oil.

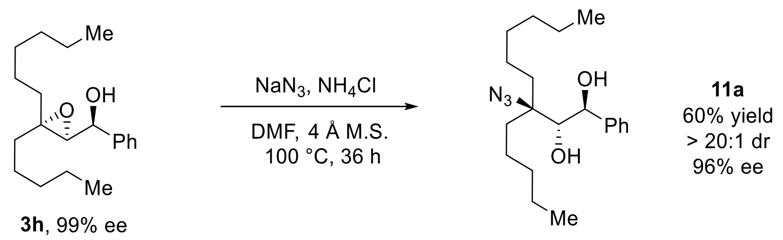

Supplementary Figure 18. Procedures for the product derivatizations **11a**

To a round-bottomed flask equiped with a dropping funnel, a condenser, and a magnetic stirrer was added **3h** (30.4 mg, 0.1 mmol, 98% ee), NaN_3 (13 mg, 0.2 mmol), NH_4Cl (10.6 mg, 0.2 mmol), 4 Å molecular sieve (50 mg) and DMF (0.4 mL). The reaction mixture was heated to 100 ℃ and stirred continuously for 36 h in an over-dried pressure-tight reaction tube. Then the mixture was extracted with CH_2Cl_2 (10 mL) for three times. The combined organic layer was dried over Na_2SO_4, filtered and evaporated under reduced pressure. The residue was purified by chromatography using a mixture of petroleum ether and ethyl acetate (10:1 v:v) to afford product **11a** (21 mg, 60% yield, 96% ee).

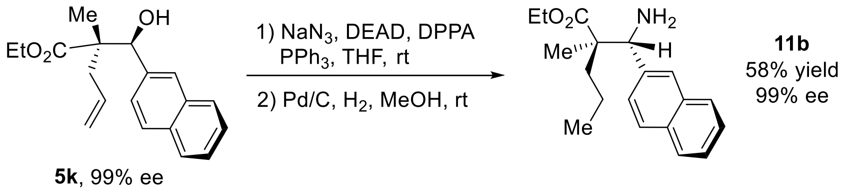

Supplementary Figure 19. Procedures for the product derivatizations **11b**

To a solution of **5k** (29.8 mg, 0.1 mmol, 99% ee) and triphenylphosphine (78.8 mg, 0.3 mmol) in THF (0.5 mL) was added diethyl azodicarboxylate (47.3 μL, 0.3 mmol) at 0 ℃. Diphenylphosphoryl azide (43.1 μL, 0.2 mmol) was added very slowly at 0 ℃. After stirring at room temperature for 13 h, the solvent was removed under reduced pressure. The residue was purified via flash chromatography using a mixture of petroleum ether and ethyl acetate (20:1 v:v) to obtain α-amino azide (21.3 mg, 66% yield). The α-amino azide (21.3 mg, 0.066 mmol) was taken in round bottom flask, evacuated and purge with hydrogen gas. Then Pd/C (10 mol%) and 0.5 mL of MeOH were added to the reaction mixture and allowed to stir for 5 h. After completion of the reaction, the reaction mixture was diluted with CH_2Cl_2 and filtered through celite. The solvent was evaporated under reduced pressure to give crude product. The crude product was then purified by chromatography using a mixture of CH_2Cl_2 and MeOH (9:1 v:v) to afford pure product **11b** (17.5 mg, 58% yield, 99% ee).

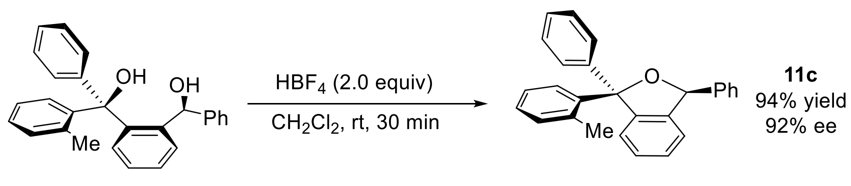

Supplementary Figure 20. Procedures for the product derivatizations **11c**

1aa (19.0 mg, 0.05 mmol, ee = 95%) was disolved in 1 mL CH_2Cl_2, employing a polypropylene vessel with a nitrogen atmosphere. Tetrafluoroboric acid (17.6 mg, 0.2 mmol) was added. After stirring for 30 min at room tempeture, the reaction was poured into a saturated aqueous Na_2CO_3 and was then extracted with Et_2O. The organic solution was concentrated and gave pure product **11c** (17 mg, 94% yield, ee = 92%).

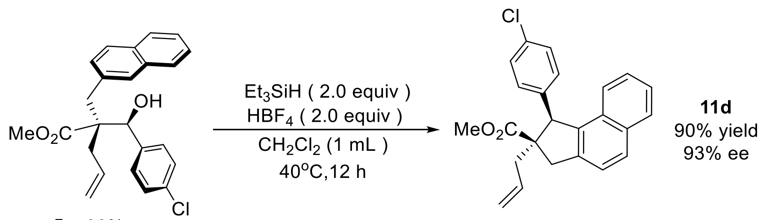

Supplementary Figure 21. Procedures for the product derivatizations **11d**

5e (39.4 mg, 0.1 mmol, ee = 90%) was disolved in 1 mL CH_2Cl_2, employing a polypropylene vessel with a nitrogen atmosphere. Triethylsilane (23.2 mg, 0.2 mmol) was then introduced into reaction flask, followed by tetrafluoroboric acid (17.6 mg, 0.2 mmol). After stirring for 3 h at 40 ℃, the reaction was poured into a saturated aqueous Na_2CO_3 and was then extracted with Et_2O. The organic solution was concentrated and gave pure product **11d** (34 mg, 90% yield, ee = 93%).

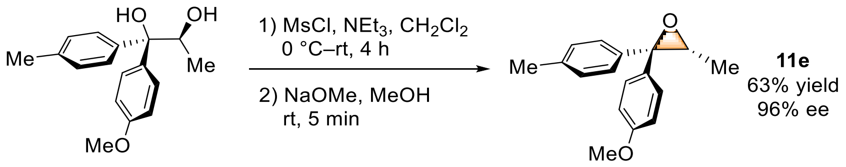

Supplementary Figure 22. Procedures for the product derivatizations **11e**

To a solution of **1b** (27.1 mg, 0.1 mmol, 99% ee) and Et_3N (27.6 mL, 0.2 mmol) in CH_2Cl_2 (0.5 mL) cooled in an ice-bath was added MsCl (8.9 μL, 0.115 mmol) dropwise. After stirring for 30 min at 0 ° C, the mixture was warmed to room temperature and stirred for another 4 h. Then CH_2Cl_2 (10 mL) was added and the organic layer was washed with water and brine, dried over Na_2SO_4 and evaporated

under reduced pressure. The obtaining residue was dissolved in MeOH (1 mL) and cooled in an ice-bath. A solution of NaOMe (0.4 g, 28% in MeOH, 217 mmol) was added dropwise and after the completion of the addition, the mixture was stirred for 15 min at 0 °C. Then the mixture was extracted with CH_2Cl_2 (10 mL) for three times. The combined organic layer was dried over Na_2SO_4, filtered and evaporated under reduced pressure. The residue was purified by chromatography using a mixture of petroleum ether and ethyl acetate(10:1 v:v) to afford product **11e** (16 mg, 63% yield, 96% ee).

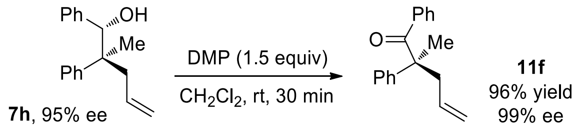

Supplementary Figure 23. Procedures for the product derivatizations **11f**

To a solution of **1a** (13 mg, 0.05 mmol, ee = 95%) in dichloromethane (1 mL) was added Dess-Martin periodinane (33 mg, 0.15 mmol) and stirred at room temperature for 30 min. The reaction was filtered through a celite column, washed with ethyl acetate, and then concentrated under reduced pressure. The residue was purified by flash chromatography using a mixture of petroleum and ethyl acetate (20:1 v:v) to give the product **11f** (12 mg, 96% yield, ee = 99%).

VI. Characterizations of new compounds

(1*S*,2*S*)-1-(4-Bromophenyl)-1-(4-chlorophenyl)propane-1,2-diol (**1a**)

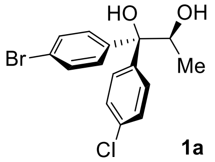

1a

Colorless oil, 164 mg, 48% yield. ^{1}H NMR (400 MHz, $CDCl_3$) δ 7.50–7.39 (m, 4H), 7.35–7.30 (m, 2H), 7.27–7.23 (m, 2H), 4.72 (q, *J* = 6.2 Hz, 1H), 3.05 (s, 1H), 1.84 (s, 1H), 1.07 (d, *J* = 6.2 Hz, 3H); ^{13}C NMR (100 MHz, $CDCl_3$) δ 144.3, 142.0, 132.9, 131.7, 128.4, 128.1, 127.0, 121.5, 79.3, 71.2, 16.9; IR (KBr thin film, cm^{-1}): ν 2962, 2927, 1489, 1398, 1261, 1093, 1011, 817, 534. $[\alpha]_D^{27}$: –49.3 (*c* 1.31, $CHCl_3$); HPLC analysis: 96% *ee* (Chiralcel OD-H, 5:95 ^{i}PrOH/hexane, 1.0 mL/min, 220 nm), R_t (major) = 14.6 min, R_t (minor) = 9.5 min.

(1*R*,2*R*)-1-(4-Bromophenyl)-1-(4-chlorophenyl)-1-hydroxypropan-2-yl propionate (**2a**)

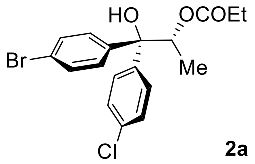

2a

White solid, 171 mg, 43% yield, mp 95–98 ℃. ^{1}H NMR (400 MHz, $CDCl_3$) δ 7.46–7.39 (m, 2H), 7.37–7.32 (m, 4H), 7.28–7.22 (m, 2H), 5.87 (q, *J* = 6.3 Hz, 1H), 2.82 (s,

1H), 2.24–2.10 (m, 2H), 1.12 (d, J = 6.3 Hz, 3H), 0.99 (t, J = 7.6 Hz, 3H); ^{13}C NMR (101 MHz, $CDCl_3$) δ 173.4, 144.0, 141.2, 133.2, 131.5, 128.5, 127.6, 127.0, 121.4, 79.1, 73.1, 27.7, 14.3, 9.0; IR (KBr thin film, cm^{-1}): ν 2986, 2941, 1735, 1491, 1192, 1093, 1011, 821. $[\alpha]_D^{27}$: +24.6 (*c* 2.98, $CHCl_3$); HPLC analysis: 94% *ee* (Chiralcel IA, 1:99 ^{i}PrOH/hexane, 1 mL/min, 220 nm), R_t (major) = 16.0 min, R_t (minor) = 13.6 min.

<u>(1*R*,2*S*)-1-(4-Methoxyphenyl)-1-(*p*-tolyl)propane-1,2-diol **(1b)**</u>

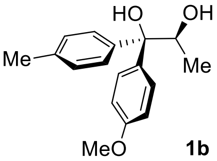

1b

Colorless oil, 117 mg, 43% yield. ^{1}H NMR (400 MHz, $CDCl_3$) δ 7.43 (d, J = 8.2 Hz, 2H), 7.31 (d, J = 8.8 Hz, 2H), 7.12 (d, J = 8.1 Hz, 2H), 6.78 (d, J = 8.8 Hz, 2H), 4.67 (q, J = 6.2 Hz, 1H), 3.72 (s, 3H), 2.29 (s, 3H), 1.06 (d, J = 6.3 Hz, 3H); ^{13}C NMR (101 MHz, $CDCl_3$) δ 158.2, 143.0, 136.7, 136.6, 129.2, 126.9, 126.2, 113.4, 79.6, 71.7, 55.2, 21.0, 16.8; HRMS (ESI, m/z): calcd. for $C_{17}H_{20}O_3Na^+$ 295.1305, found 295.1306; IR (KBr thin film, cm^{-1}): ν 2929, 1608, 1509, 1248, 1172, 1035, 828, 814. $[\alpha]_D^{27}$: –43.4 (*c* 2.55, $CHCl_3$); HPLC analysis: 99% *ee* (Chiralcel OD-H, 5:95 ^{i}PrOH/hexane, 1.0 mL/min, 220 nm), R_t (major) = 11.4 min, R_t (minor) = 9.4 min.

<u>(1*S*,2*R*)-1-Hydroxy-1-(4-methoxyphenyl)-1-(*p*-tolyl)propan-2-yl propionate **(2b)**</u>

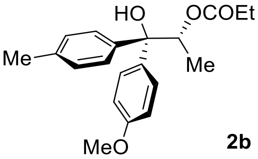

2b

White solid, 154 mg, 47% yield, mp 68–81 °C. ^{1}H NMR (400 MHz, $CDCl_3$) δ 7.35–7.31 (m, 4H), 7.09 (d, J = 8.1 Hz, 2H), 6.79 (d, J = 8.8 Hz, 2H), 5.88 (q, J = 6.2 Hz, 1H), 3.72 (s, 3H), 2.72 (s, 1H), 2.28 (s, 3H), 2.21–2.07 (m, 2H), 1.13 (d, J = 6.3 Hz, 3H), 0.97 (t, J = 7.6 Hz, 3H); ^{13}C NMR (101 MHz, $CDCl_3$) δ 173.6, 158.4, 142.6, 136.5, 135.8, 128.9, 126.8, 125.7, 113.5, 79.2, 73.7, 55.1, 27.8, 20.9, 14.4, 9.0; HRMS (ESI, m/z): calcd. for $C_{20}H_{24}O_4Na^+$ 354.1567, found 354.1570; IR (KBr thin film, cm^{-1}): ν 2986, 2939, 1733, 1510, 1250, 1181, 1061, 830, 814. $[\alpha]_D^{27}$: +61.4 (*c* 3.2, $CHCl_3$); HPLC analysis: 91% *ee* (Chiralcel IA, 1:99 ^{i}PrOH/hexane, 1.0 mL/min, 220 nm), R_t (major) = 17.0 min, R_t (minor) = 15.2 min.

<u>(1*S*,2*S*)-1-(4-Bromophenyl)-1-(4-methoxyphenyl)propane-1,2-diol **(1c)**</u>

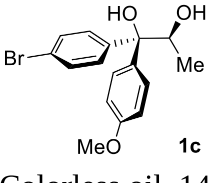

1c

Colorless oil, 145 mg, 43% yield. ^{1}H NMR (400 MHz, $CDCl_3$) δ 7.45 (s, 4H), 7.29 (d, J = 8.9 Hz, 2H), 6.82 (d, J = 8.9 Hz, 2H), 4.76–4.64 (m, 1H), 3.76 (s, 3H), 2.97 (s, 1H), 1.86 (d, J = 4.0 Hz, 1H), 1.10 (d, J = 6.3 Hz, 3H); ^{13}C NMR (101 MHz, $CDCl_3$)

δ 158.4, 144.9, 135.7, 131.5, 128.2, 126.8, 121.1, 113.6, 79.5, 71.6, 55.2, 17.0; HRMS (ESI, m/z): calcd. for $C_{16}H_{17}O_3BrNa^+$ 359.0253, found 359.0258; IR (KBr thin film, cm^{-1}): ν 2930, 1510, 1249, 1171, 1074, 1033, 1009, 825. $[\alpha]_D^{27}$: –27.3 (*c* 3.44, $CHCl_3$); HPLC analysis: 99% *ee* (Chiralcel OD-H, 5:95 ^{i}PrOH/hexane, 1.0 mL/min, 220 nm), R_t (major) = 15.1 min, R_t (minor) = 11.8 min.

(1*R*,2*R*)-1-(4-Bromophenyl)-1-hydroxy-1-(4-methoxyphenyl)propan-2-yl propionate (**2c**)

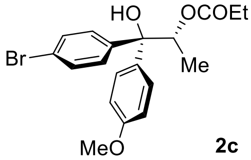

2c

White solid, 185 mg, 47% yield, mp 75–78 ℃. ^{1}H NMR (400 MHz, $CDCl_3$) δ 7.41 (d, *J* = 8.7 Hz, 2H), 7.35 (d, *J* = 8.7 Hz, 2H), 7.30 (d, *J* = 8.8 Hz, 2H), 6.81 (d, *J* = 8.8 Hz, 2H), 5.86 (q, *J* = 6.2 Hz, 1H), 3.75 (s, 3H), 2.72 (s, 1H), 2.29 – 2.04 (m, 2H), 1.14 (d, *J* = 6.2 Hz, 3H), 0.98 (t, *J* = 7.6 Hz, 3H); ^{13}C NMR (101 MHz, $CDCl_3$) δ 173.5, 158.6, 144.7, 135.0, 131.3, 127.8, 126.8, 121.1, 113.7, 79.2, 73.5, 55.2, 27.8, 14.4, 9.1; IR (KBr thin film, cm^{-1}): HRMS (ESI, m/z): calcd. for $C_{19}H_{21}O_4BrNa^+$ 415.0515, found 415.0523; IR (KBr thin film, cm^{-1}): ν 2987, 2939, 1733, 1511, 1250, 1181, 1061, 829. $[\alpha]_D^{25}$: +50.8 (*c* 3.91, $CHCl_3$); HPLC analysis: 92% *ee* (Chiralcel IA, 1:99 ^{i}PrOH/hexane, 1.0 mL/min, 220 nm), R_t (major) = 26.3 min, R_t (minor) = 18.8 min.

(1***S***,2***S***)-1-Phenyl-1-(thiophen-2-yl)propane-1,2-diol (**1d**)

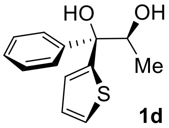

1d

White solid, 57.0 mg, 49% yield, mp 57–59 ˚C. ^{1}H NMR (400 MHz, $CDCl_3$) δ 7.55 (d, *J* = 7.5 Hz, 2H), 7.30–7.26 (m, 2H), 7.21–7.16 (m, 1H), 7.12–7.10 (m, 1H), 6.85–6.83 (m, 2H), 4.50 (q, *J* = 6.2 Hz, 1H), 3.07 (s, 1H), 1.89 (s, 1H), 1.12 (d, *J* = 6.3 Hz, 3H); ^{13}C NMR (100 MHz, $CDCl_3$) δ 148.7, 144.3, 128.7, 127.8, 126.9, 126.1, 124.8, 124.2, 79.8, 73.6, 16.9; IR (KBr thin film, cm^{-1}): ν 3447, 3061, 3026, 2962, 2927, 2855, 1601, 1515, 1495, 1448, 1395, 1199, 1058, 1008, 797, 700. $[\alpha]_D^{25}$: +5.7 (c 0.5, $CHCl_3$); HPLC analysis: 92% *ee* (Chiralcel OD-H, 10:90 ^{i}PrOH/Hexane, 1.0 mL/min, 220 nm), R_t (major) = 15.3 min, R_t (minor) = 10.8 min; R_t (minor) = 24.8 min.

(1*R*,2*R*)-1-Hydroxy-1-phenyl-1-(thiophen-2-yl)propan-2-yl propionate (**2d**)

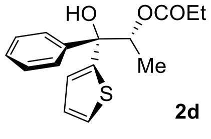

2d

White solid, 71.5 mg, 49% yield, mp 63–65 ˚C. ^{1}H NMR (400 MHz, $CDCl_3$) δ 7.57–7.54 (m, 2H), 7.34–7.29 (m, 2H), 7.26–7.22 (m, 1H), 7.19–7.18 (m, 1H), 6.93–6.90 (m, 2H), 5.81 (q, *J* = 6.3 Hz, 1H), 2.88 (s, 1H), 2.18–2.03 (m, 2H), 1.27 (d, *J* = 6.3 Hz,

3H), 0.90 (t, J = 7.6 Hz, 3H); ^{13}C NMR (100 MHz, $CDCl_3$) δ 173.6, 148.4, 144.3, 128.4, 127.6, 127.0, 125.7, 125.0, 124.3, 79.3, 74.8, 27.9, 14.9, 9.1; IR (KBr thin film, cm^{-1}): ν 3549, 3067, 3033, 2967, 2933, 2859, 1732, 1600, 1518, 1498, 1457, 1398, 1205, 1065, 1011, 793, 701. $[\alpha]_D^{25}$: +95.5 (c 0.56, $CHCl_3$); HPLC analysis: 91% *ee* (Chiralcel IA-H, 1:99 ^{i}PrOH/Hexane, 1.0 mL/min, 220 nm), R_t (major) = 17.2 min, R_t (minor) = 15.7 min.

(1*S*,2*S*)-1-(4-Methoxyphenyl)-1-(thiophen-2-yl)propane-1,2-diol (**1e**)

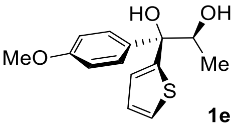

1e

Yellow oil, 61.0 mg, 46% yield. ^{1}H NMR (400 MHz, $CDCl_3$) δ 7.55–7.52 (m, 2H), 7.19–7.18 (m, 1H), 6.93–6.86 (m, 4H), 4.54 (q, J = 6.2 Hz, 1H), 3.78 (s, 3H), 3.00 (s, 1H), 1.89 (s, 1H), 1.19 (d, J = 6.3 Hz, 3H); ^{13}C NMR (100 MHz, $CDCl_3$) δ 159.2, 149.0, 136.3, 127.5, 126.9, 124.8, 124.3, 114.0, 79.6, 73.6, 55.5, 17.0; HRMS (ESI, m/z): calcd. for $C_{14}H_{16}O_3SNa^+$ 287.0712, found 287.0717; IR (KBr thin film, cm^{-1}): ν 3456, 3064, 3025, 2968, 2937, 2865, 1605, 1519, 1491, 1453, 1398, 1210, 1063, 1015, 795, 698. $[\alpha]_D^{25}$: +8.7 (c 0.59, $CHCl_3$); HPLC analysis: 96% *ee* (Chiralcel OD-H, 5:95 ^{i}PrOH/Hexane, 1.0 mL/min, 220 nm), R_t (major) = 29.7 min, R_t (minor) = 17.6 min.

(1*R*,2*R*)-1-Hydroxy-1-(4-methoxyphenyl)-1-(thiophen-2-yl)propan-2-yl propionate (**2e**)

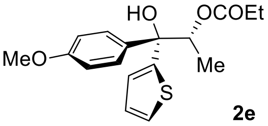

2e

Yellow oil, 77.0 mg, 48% yield. ^{1}H NMR (400 MHz, $CDCl_3$) δ 7.46–7.44 (m, 2H), 7.18–7.16 (m, 1H), 6.91–6.82 (m, 4H), 5.74 (q, J = 6.3 Hz, 1H), 3.76 (s, 3H), 2.75 (s, 1H), 2.20–2.06 (m, 2H), 1.23 (d, J = 6.3 Hz, 3H), 0.93 (t, J = 7.6 Hz, 3H); ^{13}C NMR (100 MHz, $CDCl_3$) δ 173.6, 159.0, 148.9, 136.5, 127.1, 127.0, 124.9, 124.3, 113.6, 79.0, 74.8, 55.4, 28.0, 15.0, 9.2; HRMS (ESI, m/z): calcd. for $C_{17}H_{20}O_4BrNa^+$ 343.0975, found 343.0979; IR (KBr thin film, cm-1): ν 3556, 3069, 3035, 2975, 2939, 2869, 1728, 1602, 1514, 1493, 1467, 1396, 1209, 1069, 1013, 796, 699. $[\alpha]_D^{25}$: +94.4 (c 0.60, $CHCl_3$); HPLC analysis: 91% *ee* (Chiralcel OD-H, 1:99 ^{i}PrOH/Hexane, 1.0 mL/min, 220 nm), R_t (major) = 16.5 min, R_t (minor) = 15.3 min.

(1*S*,2*S*)-1-(Furan-2-yl)-1-(thiophen-2-yl)propane-1,2-diol (**1f**)

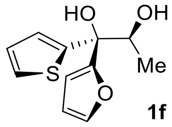

1f

Brown solid, 106.2 mg, 47% yield, mp 56–58 ˚C. ^{1}H NMR (600 MHz, $CDCl_3$) δ 7.40–7.39 (m, 1H), 7.28–7.27 (m, 1H), 7.11–7.10 (m, 1H), 7.01–6.99 (m, 1H), 6.39–

6.34 (m, 2H), 4.42–4.38 (m, 1H), 3.34 (s, 1H), 2.03 (d, *J* = 5.4 Hz, 1H), 1.16 (d, *J* = 6.4 Hz, 3H); ^{13}C NMR (150 MHz, $CDCl_3$) δ 155.5, 147.0, 142.3, 127.1, 125.5, 125.1, 110.6, 107.8, 77.5, 73.8, 17.2; HRMS (ESI, m/z): calcd. for $C_{11}H_{12}O_3SNa^+$ 247.0399, found 247.0401; IR (KBr thin film, cm^{-1}): ν 3449, 2969, 2925, 2862, 1598, 1511, 1498, 1449, 1398, 1260, 1180, 1065, 1018, 814, 700. $[\alpha]_D^{25}$: +24.6 (c 0.97, $CHCl_3$); HPLC analysis: 93% *ee* (Chiralcel IA, 2:98 ^{i}PrOH/Hexane, 1.0 mL/min, 220 nm), R_t (major) = 26.0 min, R_t (minor) = 28.9 min.

(1*R*,2*R*)-1-(Furan-2-yl)-1-hydroxy-1-(thiophen-2-yl)propan-2-yl propionate (**2f**)

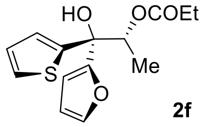

2f

Yellow oil, 135.0 mg, 48% yield. ^{1}H NMR (600 MHz, $CDCl_3$) δ 7.38–7.37 (m, 1H), 7.25–7.23 (m, 1H), 7.05–7.04 (m, 1H), 6.97–6.95 (m, 1H), 6.32–6.31 (m, 2H), 5.62 (q, *J* = 6.4 Hz, 1H), 3.11 (s, 1H), 2.26–2.16 (m, 2H), 1.20 (d, *J* = 6.4 Hz, 3H), 0.99 (t, *J* = 7.6 Hz, 3H); ^{13}C NMR (150 MHz, $CDCl_3$) δ 173.6, 155.1, 146.1, 142.5, 126.9, 125.2, 125.0, 110.5, 107.7, 76.6, 74.4, 28.0, 15.0, 9.2; IR (KBr thin film, cm^{-1}): ν 3511, 3062, 3029, 2982, 2940, 1727, 1601, 1512, 1495, 1449, 1363, 1203, 1180, 1080, 1013, 814, 699. $[\alpha]_D^{25}$: +24.4 (c 0.95, $CHCl_3$); HPLC analysis: 92% *ee* (Chiralcel IC, 1:99 ^{i}PrOH/Hexane, 1.0 mL/min, 220 nm), R_t (major) = 30.2 min, R_t (minor) = 37.5 min.

(1*S*,2*S*)-1-Phenyl-1-(pyridin-2-yl)propane-1,2-diol (**1g**)

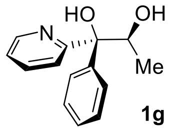

1g

White solid, 105.5 mg, 46% yield, mp 110–112 °C. ^{1}H NMR (400 MHz, $CDCl_3$) δ 8.49 (d, *J* = 4.9 Hz, 1H), 7.67–7.63 (m, 1H), 7.47–7.44 (m, 2H), 7.39 (d, *J* = 8.0 Hz, 1H), 7.31 (t, *J* = 7.5 Hz, 2H), 7.26–7.17 (m, 2H), 4.89 (q, *J* = 6.3 Hz 1H), 4.62 (s, 1H), 3.92 (s, 1H), 1.04 (d, *J* = 6.3 Hz, 3H); ^{13}C NMR (150 MHz, $CDCl_3$) δ 164.7, 147.2, 143.4, 137.6, 128.4, 127.2, 125.8, 123.1, 122.6, 80.2, 72.2, 16.6; HRMS (ESI, m/z): calcd. for $C_{14}H_{15}O_2NNa^+$ 252.0995, found 252.0998; IR (KBr thin film, cm^{-1}): ν 3481, 3061, 3027, 2962, 2930, 2836, 1607, 1510, 1454, 1252, 1178, 1083, 1033, 813, 700. $[\alpha]_D^{25}$: +49.3 (c 0.63, $CHCl_3$); HPLC analysis: 92% *ee* (Chiralcel IA, 1:99 ^{i}PrOH/Hexane, 1.0 mL/min), R_t (major) = 27.2 min, R_t (minor) = 23.7 min.

(1*R*,2*R*)-1-Hydroxy-1-phenyl-1-(pyridin-2-yl)propan-2-yl propionate (**2g**)

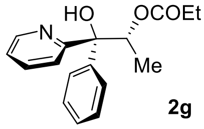

2g

White solid, 136.6 mg, 48% yield, mp 64–66 °C. ^{1}H NMR (400 MHz, $CDCl_3$) δ 8.41 (d, *J* = 4.8 Hz, 1H), 7.62–7.49 (m, 3H), 7.42 (d, *J* = 8.0 Hz, 1H), 7.24 (t, *J* = 7.6 Hz, 2H),

7.18–7.13 (m, 1H), 7.09–7.06 (m, 1H), 5.98 (q, *J* = 6.3 Hz, 1H), 5.91 (s, 1H), 2.07–1.85 (m, 2H), 1.18 (d, *J* = 6.3 Hz, 3H), 0.75 (t, *J* = 7.6 Hz, 3H); ^{13}C NMR (150 MHz, $CDCl_3$) δ 173.9, 161.3, 147.3, 143.2, 137.1, 128.6, 127.5, 126.1, 122.5, 120.8, 79.2, 74.2, 27.8, 14.6, 9.2; HRMS (ESI, m/z): calcd. for $C_{17}H_{19}O_3NNa^+$ 308.1257, found 308.1260; IR (KBr thin film, cm^{-1}): ν 3526, 3065, 3030, 2965, 2929, 2852, 1724, 1609, 1510, 1458, 1364, 1260, 1185, 1096, 1025, 812, 703. $[\alpha]_D^{25}$: +33.7 (c 0.84, $CHCl_3$); HPLC analysis: 90% *ee* (Chiralcel IA, 1:99 ^{i}PrOH/Hexane, 1.0 mL/min, 220 nm), R_t (major) = 9.9 min, R_t (minor) = 11.2 min.

(1*S*,2*S*)-1-(4-Bromophenyl)-1-(4-chlorophenyl)butane-1,2-diol **(1h)**

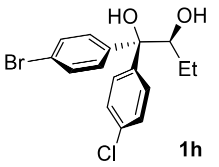

White solid, 167 mg, 47% yield, mp 87–90 ℃. ^{1}H NMR (400 MHz, $CDCl_3$) δ 7.50–7.42 (m, 4H), 7.36–7.30 (m, 2H), 7.28–7.23 (m, 2H), 4.38 (dd, *J* = 9.9, 1.8 Hz, 1H), 3.04 (s, 1H), 1.84 (s, 1H), 1.50–1.39 (m, 1H), 1.36–1.21 (m, 1H), 0.96 (t, *J* = 7.4 Hz, 3H); ^{13}C NMR (101 MHz, $CDCl_3$) δ 144.6, 142.0, 132.9, 131.7, 128.4, 128.1, 127.0, 121.5, 79.6, 76.9, 23.4, 10.8; HRMS (ESI, m/z): calcd. for $C_{16}H_{16}O_2BrClNa^+$ 376.9914, found 3376.9916; IR (KBr thin film, cm^{-1}): ν 2966, 2934, 1490, 1458, 1399, 1093, 1011, 822. $[\alpha]^{27.9}{}_D$: –54.5 (*c* 1.14, $CHCl_3$); HPLC analysis: 94% *ee* (Chiralcel OD-H, 5:95 ^{i}PrOH/hexane, 1.0 mL/min, 220 nm), R_t (major) = 13.2 min, R_t (minor) = 6.8 min.

(1*R*,2*R*)-1-(4-Bromophenyl)-1-(4-chlorophenyl)-1-hydroxybutan-2-yl propionate **(2h)**

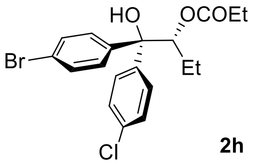

White solid, 185 mg, 45% yield, mp 85–89 ℃. ^{1}H NMR (600 MHz, $CDCl_3$) δ 7.41–7.35 (m, 6H), 7.25–7.24 (m, 2H), 5.82 (dd, *J* = 10.0, 2.8 Hz, 1H), 2.77 (s, 1H), 2.24–2.17 (m, 1H), 2.16–2.02 (m, 1H), 1.70–1.63 (m, 1H), 1.45–1.38 (m, 1H), 0.96 (t, *J* = 7.6 Hz, 3H), 0.80 (t, *J* = 7.5 Hz, 3H); ^{13}C NMR (151 MHz, $CDCl_3$) δ 173.9, 144.2, 141.4, 133.2, 131.5, 128.6, 127.6, 127.0, 121.4, 79.7, 77.8, 27.7, 22.4, 10.5, 9.2; IR (KBr thin film, cm^{-1}): ν 2976, 2939, 1734, 1490, 1460, 1180, 1093, 1011, 817. $[\alpha]^{27.3}{}_D$: +23.9 (*c* 3.54, $CHCl_3$); HPLC analysis: 94% *ee* (Chiralcel IA, 1:99 ^{i}PrOH/hexane, 1.0 mL/min, 220 nm), R_t (major) = 14.6 min, R_t (minor) = 12.1 min.

(1*R*,2*S*)-1,3-Diphenyl-1-(*p*-tolyl)propane-1,2-diol **(1i)**

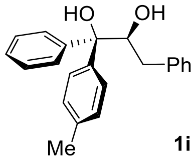

1i

White solid, 152.8 mg, 48% yield, mp 116–118 ˚C. ^{1}H NMR (400 MHz, $CDCl_3$) δ 7.56–7.54 (m, 2H), 7.38 (d, *J* = 8.2 Hz, 2H), 7.29–7.25 (m, 4H), 7.22–7.11 (m, 6H), 4.75–4.71 (m, 1H), 3.18 (s, 1H), 2.73–2.60 (m, 2H), 2.29 (s, 3H), 1.90 (d, *J* = 3.1 Hz, 1H); ^{13}C NMR (100 MHz, $CDCl_3$) δ 146.1, 141.1, 139.3, 136.6, 129.6, 129.2, 128.7, 128.6, 127.2, 126.6, 126.4, 125.6, 80.0, 76.7, 37.3, 21.2; HRMS (ESI, m/z): calcd. for $C_{22}H_{22}O_2Na^+$ 341.1512, found 342.1515; IR (KBr thin film, cm^{-1}): ν 3455, 3066, 3031, 2961, 2939, 2869, 1610, 1511, 1491, 1477, 1388, 1199, 1085, 1011, 811, 705. $[\alpha]_D^{25}$: +23.2 (c 0.55, $CHCl_3$); HPLC analysis: 93% *ee* (Chiralcel OD-H, 10:90 ^{i}PrOH/Hexane, 1.0 mL/min, 220 nm), R_t (major) = 13.8 min, R_t (minor) = 5.6 min.

(1*S*,2*R*)-1-Hydroxy-1,3-diphenyl-1-(*p*-tolyl)propan-2-yl propionate (**2i**)

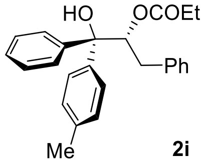

2i

White solid, 180.0 mg, 48% yield, mp 122−124 ˚C. ^{1}H NMR (400 MHz, $CDCl_3$) δ 7.54 (d, *J* = 7.5 Hz, 2H), 7.43 (d, *J* = 8.2 Hz, 2H), 7.25 (t, *J* = 7.7 Hz, 2H), 7.21–7.08 (m, 8H), 6.19 (dd, *J* = 9.9, 3.2 Hz, 1H), 2.95–2.89 (m, 1H), 2.87 (s, 1H), 2.81–2.77 (m, 1H), 2.28 (s, 3H), 1.95–1.88 (m, 2H), 0.73 (t, *J* = 7.6 Hz, 3H); ^{13}C NMR (100 MHz, $CDCl_3$) δ 172.6, 145.1, 140.1, 137.7, 136.6, 129.4, 129.0, 128.0, 127.9, 126.9, 126.1, 125.6, 125.3, 79.8, 76.6, 35.7, 27.3, 20.8, 8.8; IR (KBr thin film, cm^{-1}): ν 3565, 3059, 3027, 2969, 2929, 2878, 1731, 1608, 1513, 1497, 1487, 1378, 1215, 1077, 1021, 816, 700. $[\alpha]_D^{25}$: +76.0 (c 0.54, $CHCl_3$); HPLC analysis: 93% *ee* (Chiralcel IA, 1:99 ^{i}PrOH/Hexane, 1.0 mL/min, 220 nm), R_t (major) = 12.4 min, R_t (minor) = 14.4 min.

(1*S*,2*S*)-1-(4-Methoxyphenyl)-3-phenyl-1-(*p*-tolyl)propane-1,2-diol (**1j**)

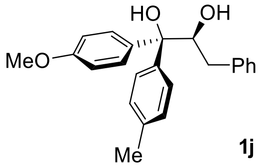

1j

White solid, 163.5 mg, 47% yield, mp 82–84 ˚C. ^{1}H NMR (400 MHz, $CDCl_3$) δ 7.49–7.47 (m, 2H), 7.36 (d, *J* = 8.2 Hz, 2H), 7.30–7.26 (m, 2H), 7.22–7.11 (m, 5H), 6.84–6.82 (m, 2H), 4.72–4.68 (m, 1H), 3.74 (s, 3H), 3.09 (s, 1H), 2.72–2.60 (m, 2H), 2.30 (s, 3H), 1.92 (d, *J* = 3.0 Hz, 1H); ^{13}C NMR (100 MHz, $CDCl_3$) δ 158.7, 141.4, 139.4, 138.2, 136.6, 129.5, 129.2, 128.8, 127.7, 126.6, 125.6, 113.9, 79.7, 76.8, 55.4, 37.4, 21.2; HRMS (ESI, m/z): calcd. for $C_{23}H_{24}O_3Na^+$ 376.1618, found 376.1620; IR (KBr thin film, cm^{-1}): ν 3734, 3124, 3024, 2952, 2919, 2895, 1615, 1518, 1485, 1412, 1305, 1241, 1132, 1023, 840, 722. $[\alpha]_D^{25}$: +21.0 (c 0.52, $CHCl_3$); HPLC analysis: 93% *ee* (Chiralcel

OD-H, 30:70 ^{i}PrOH/Hexane, 1.0 mL/min, 220 nm), R_t (major) = 15.5 min, R_t (minor) = 4.5 min.

(1*R*,2*R*)-1-Hydroxy-1-(4-methoxyphenyl)-3-phenyl-1-(*p*-tolyl)propan-2-yl propionate (**2j**)

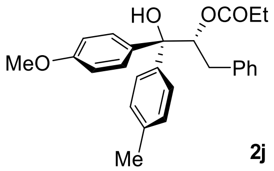

2j

White solid, 195.0 mg, 48% yield, mp 124–126 ˚C. ^{1}H NMR (400 MHz, $CDCl_3$) δ 7.46–7.39 (m, 4H), 7.22–7.07 (m, 7H), 6.79 (d, *J* = 8.9 Hz, 2H), 6.13 (dd, *J* = 10.0, 3.2 Hz, 1H), 3.72 (s, 3H), 2.93–2.87 (m, 1H), 2.80 (s, 1H), 2.78–2.74 (m, 1H), 2.29 (s, 3H), 1.98–1.92 (m, 2H), 0.76 (t, *J* = 7.6 Hz, 3H); ^{13}C NMR (100 MHz, $CDCl_3$) δ 173.0, 158.6, 140.8, 138.0, 137.7, 136.8, 129.7, 129.2, 128.2, 127.2, 126.5, 125.6, 113.7, 79.9, 77.8, 55.3, 36.1, 27.6, 21.1, 9.1; HRMS (ESI, m/z): calcd. for $C_{26}H_{28}O_4Na^+$ 427.1880, found 427.1883; IR (KBr thin film, cm^{-1}): ν 3578, 3115, 3034, 2959, 2939, 2885, 1729, 1609, 1516, 1489, 1409, 1295, 1221, 1089, 1013, 837, 718. $[\alpha]_D^{25}$: +79.7 (c 0.55, $CHCl_3$); HPLC analysis: 98% *ee* (Chiralcel IA-H, 1:99 ^{i}PrOH/Hexane, 1.0 mL/min, 220 nm), R_t (major) = 21.2 min, R_t (minor) = 26.9 min.

(1*S*,2*S*)-1-(4-Methoxyphenyl)-1-phenylpentane-1,2-diol (**1k**)

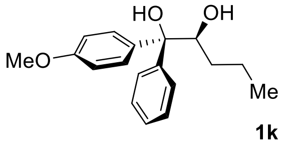

1k

White solid, 27.5 mg, 48% yield, mp 115–116 ℃. ^{1}H NMR (600 MHz, $CDCl_3$) δ 7.51 (d, *J* = 8.8 Hz, 2H), 7.41 (dd, *J* = 8.4, 1.2 Hz, 2H), 7.28 (t, *J* = 7.4 Hz, 2H), 7.20–7.17 (m, 1H), 6.87 (d, *J* = 8.8 Hz, 2H), 4.53 (ddd, *J* = 10.1, 4.0, 2.0 Hz, 1H), 3.78 (s, 3H), 2.91 (s, 1H), 1.82 (d, *J* = 4.0 Hz, 1H), 1.59–1.52 (m, 1H), 1.48–1.42 (m, 1H), 1.34–1.23 (m, 2H), 0.86 (t, *J* = 7.2 Hz, 3H). ^{13}C NMR (151 MHz, $CDCl_3$) δ 158.7, 144.2, 138.0, 128.2, 127.5, 126.8, 125.7, 114.0, 80.0, 75.6, 55.3, 32.5, 19.7, 14.1; HRMS (ESI, m/z): calcd. for $C_{18}H_{22}O_3Na^+$ 309.1461, found 309.1458; IR (KBr thin film, cm^{-1}): ν 3460, 2959, 2921, 2358, 1511, 1453, 1259, 1180, 1024, 748. $[\alpha]_D^{25}$: –43.7 (c 0.1, $CHCl_3$); HPLC analysis: 95% *ee* (Chiralcel AD-H, 10:90 ^{i}PrOH/hexane, 1.0 mL/min, 220 nm), R_t (major) = 8.0 min, R_t (minor) = 10.2 min.

(1*R*,2*R*)-1-Hydroxy-1-(4-methoxyphenyl)-1-phenylpentan-2-yl propionate (**2k**)

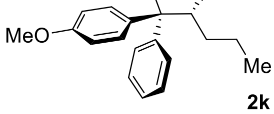

2k

White solid, 30 mg, 44% yield, mp 118–120 ℃. ^{1}H NMR (600 MHz, $CDCl_3$) δ 7.45–7.41 (m, 4H), 7.28 (t, *J* = 7.4 Hz, 2H), 7.20–7.17 (m, 1H), 6.81 (d, *J* = 8.9 Hz,

2H), 5.95 (dd, *J* = 10.0, 2.6 Hz, 1H), 3.75 (s, 3H), 2.64 (s, 1H), 2.23–2.17 (m, 1H), 2.13–2.07 (m, 1H), 1.68–1.62 (m, 1H), 1.38–1.33 (m, 1H), 1.28–1.16 (m, 2H), 0.94 (t, *J* = 7.6 Hz, 3H), 0.82 (t, *J* = 7.3 Hz, 3H). ^{13}C NMR (151 MHz, $CDCl_3$) δ 174.0, 158.5, 143.7, 137.7, 128.3, 127.1, 127.0, 125.6, 113.6, 80.0, 55.3, 31.7, 27.8, 19.3, 14.1, 9.3. HRMS (ESI, m/z): calcd. for $C_{21}H_{26}O_4Na^+$ 365.1723, found 365.1721; IR (KBr thin film, cm^{-1}): ν 3502, 2970, 2372, 1719, 1650, 1508, 1252, 1083, 758. $[\alpha]_D^{25}$: –33.1 (c 0.1, $CHCl_3$); HPLC analysis: 97% *ee* (Chiralcel AS-H, 5:95 ^{i}PrOH/hexane, 1.0 mL/min, 220 nm), R_t (major) = 3.3 min, R_t (minor) = 3.6 min.

(1*S*,2*S*)-1-Phenyl-1-(phenyl-*d*5)pentane-1,2-diol (**1l**)

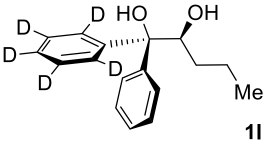

1l

White solid, 25.8 mg, 49% yield, mp 110–112 ℃. ^{1}H NMR (400 MHz, $CDCl_3$) δ 7.43 (d, *J* = 7.8 Hz, 2H), 7.29 (t, *J* = 7.4 Hz, 2H), 7.19 (t, *J* = 7.2 Hz, 1H), 4.59–4.57 (m, 1H), 2.99 (s, 1H), 1.81 (s, 1H), 1.52–1.43 (m, 2H), 1.36–1.24 (m, 2H), 0.87 (t, *J* = 7.0 Hz, 3H). ^{13}C NMR (100 MHz, $CDCl_3$) δ 145.7, 143.9, 128.3, 126.8, 125.7, 80.2, 75.6, 32.4, 19.7, 14.1. HRMS (ESI, m/z): calcd. for $C_{17}H_{15}D_5O_2Na^+$ 284.1669, found 284.1670; IR (KBr thin film, cm^{-1}): ν 3436, 2956, 2859, 2365, 2330, 1632, 1446, 1266, 1021,755, 696. $[\alpha]_D^{25}$: –7.4 (c 0.1, $CHCl_3$); HPLC analysis: 91% *ee* (Chiralcel AD-H, 5:95 ^{i}PrOH/hexane, 1.0 mL/min, 220 nm), R_t (major) = 9.3 min, R_t (minor) = 10.3 min.

(1*R*,2*R*)-1-Hydroxy-1-phenyl-1-(phenyl-*d*5)pentan-2-yl propionate (**2l**)

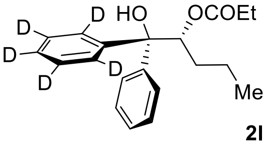

2l

White solid, 29.8 mg, 47% yield, mp 137–139 ℃. ^{1}H NMR (400 MHz, $CDCl_3$) δ 7.47 (d, *J* = 7.6 Hz, 2H), 7.29 (t, *J* = 7.5 Hz, 2H), 7.19 (t, *J* = 7.2 Hz, 1H), 6.00 (dd, *J* =10.0, 2.4 Hz, 1H), 2.70 (s, 1H), 2.22–2.01 (m, 2H), 1.73–1.63 (m, 1H), 1.43–1.35 (m, 1H), 1.30–1.18 (m, 2H), 0.91 (t, *J* = 7.6 Hz, 3H), 0.83 (t, *J* = 7.3 Hz, 3H). ^{13}C NMR (151 MHz, $CDCl_3$) δ 174.0, 145.3, 143.4, 128.4, 127.1, 125.6, 80.2, 31.6, 27.8, 19.3, 14.1, 9.2; HRMS (ESI, m/z): calcd. for $C_{20}H_{19}D_5O_3Na^+$ 340.1931, found 340.1931; IR (KBr thin film, cm^{-1}): ν 3453, 2959, 2355, 1743, 1650, 1442, 1266, 1166, 1079, 1079, 744. $[\alpha]_D^{25}$: –25.3 (c 0.1, $CHCl_3$); HPLC analysis: 97% *ee* (Chiralcel AD-H, 5:95 ^{i}PrOH/hexane, 1.0 mL/min, 220 nm), R_t (major) = 4.8 min, R_t (minor) = 4.4 min.

(1*S*,2*R*)-2-Methyl-1-phenylbutane-1,2-diol (**1m**)

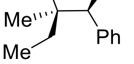

1m

White solid, 18 mg, 50% yield, mp 50–52 ℃. ^{1}H NMR (600 MHz, $CDCl_3$) δ 7.39–

7.37 (m, 2H), 7.35–7.32 (m, 2H), 7.30–7.28 (m, 1H), 4.57 (s, 1H), 2.49 (s, 1H), 1.88 (s, 1H), 1.57–1.51 (m, 1H), 1.29–1.23 (m, 1H), 1.20 (s, 3H), 0.90 (t, *J* = 7.6 Hz, 3H). ^{13}C NMR (150 MHz, CDCl3) δ 140.6, 128.1, 127.9, 127.7, 80.7, 75.2, 28.9, 22.8, 7.6. HRMS (ESI, m/z): calcd. for $C_{11}H_{16}O_2Na^+$ 203.1043, found 203.1041; IR (KBr thin film, cm^{-1}): ν 3567, 3391, 2967, 1723, 1697, 1541, 1456, 799. $[\alpha]_D^{25}$: –35.0 (c 0.1, $CHCl_3$); HPLC analysis: 94% *ee* (Chiralcel OD-H, 10:90 ^{i}PrOH/hexane, 1.0 mL/min, 220 nm), R_t (major) = 4.7 min, R_t (minor) = 4.0 min.

(1*R*,2*S*)-2-Hydroxy-2-methyl-1-phenylbutyl propionate (**2m**)

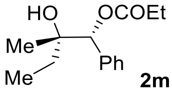

2m

White solid, 23.5 mg, 50% yield, mp 48–50 ℃. ^{1}H NMR (600 MHz, $CDCl_3$) δ 7.37–7.27 (m, 5H), 5.64 (s, 1H), 2.48–2.33 (m, 2H), 1.62 (s, 1H), 1.52–1.39 (m, 2H), 1.18 (s, 3H), 1.15 (t, *J* = 11.3 Hz, 3H), 0.93 (t, *J* = 11.2 Hz, 3H). ^{13}C NMR (151 MHz, $CDCl_3$) δ 173.4, 137.2, 128.2, 128.0, 80.4, 74.2, 30.7, 27.9, 22.7, 9.2, 7.7. HRMS (ESI, m/z): calcd. for $C_{14}H_{20}O_3Na^+$ 259.1305, found 259.1302; IR (KBr thin film, cm^{-1}): ν 2965, 2923, 2361, 1736, 1460, 1259, 1187, 748. $[\alpha]_D^{25}$: –37.8 (c 0.1, $CHCl_3$); HPLC analysis: 91% *ee* (Chiralcel IA, 2:98 ^{i}PrOH/hexane, 1.0 mL/min, 220 nm), R_t (major) = 9.1 min, R_t (minor) = 8.2 min.

(1*S*,2*R*)-2-Cyclohexyl-1-phenylpropane-1,2-diol (**1n**)

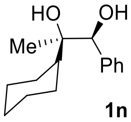

1n

White solid, 21 mg, 45% yield, mp 63–65 ℃. ^{1}H NMR (400 MHz, $CDCl_3$) δ 7.41–7.30 (m, 5H), 4.64 (d, *J* = 4.5 Hz, 1H), 2.38 (d, *J* = 4.6 Hz, 1H), 1.84–1.59 (m, 6H), 1.24 (s, 3H), 1.10–1.03 (m, 6H). ^{13}C NMR (100 MHz, CDCl3) δ 141.0, 128.3, 127.9, 127.8, 78.2, 76.9, 44.1, 28.3, 26.7, 26.5, 26.4, 20.2. HRMS (ESI, m/z): calcd. for $C_{18}H_{26}O_3Na^+$ 257.1512, found 257.1511; IR (KBr thin film, cm^{-1}): ν 3522, 3443, 3367, 2927, 2851, 2360, 2343, 1648, 1537, 1025, 749. $[\alpha]_D^{25}$: –24.2 (c 0.1, $CHCl_3$); HPLC analysis: 96% *ee* (Chiralcel AS-H, 7:93 ^{i}PrOH/hexane, 1.0 mL/min, 254nm), R_t (major) = 4.0 min, R_t (minor) = 4.6 min.

(1*R*,2*S*)-2-Cyclohexyl-2-hydroxy-1-phenylpropyl propionate (**2n**)

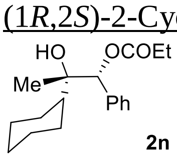

2n

White solid, 28 mg, 48% yield, mp 53–55 ℃. ^{1}H NMR (400 MHz, $CDCl_3$) δ 7.41–7.38 (m, 2H), 7.35–7.28 (m, 3H), 5.82 (s, 1H), 2.47–2.31 (m, 2H), 1.85 (t, *J* = 8.0 Hz, 2H), 1.74 (brs, 2H), 1.61 (brs, 1H), 1.46 (s, 1H), 1.16–1.06 (m, 12H). ^{13}C NMR (150 MHz, CDCl3) δ 173.4, 137.2, 128.3, 128.2, 128.1, 78.6, 76.0, 44.3, 28.2, 27.9, 26.6, 26.5, 26.4, 26.3, 20.2, 9.2. HRMS (ESI, m/z): calcd. for $C_{18}H_{26}O_3Na^+$ 313.1774,

found 313.1772; IR (KBr thin film, cm^{-1}): ν 3522, 2931, 2855, 1738, 1454, 1371, 1264, 1188, 1084, 1019, 801, 749. $[\alpha]_D^{25}$: –36.4 (c 0.1, $CHCl_3$); HPLC analysis: 91% *ee* (Chiralcel IA, 2:98 ^{i}PrOH/hexane, 1.0 mL/min, 254nm), R_t (major) = 9.2 min, R_t (minor) = 7.5 min.

(1*S*,2*R*)-2,3-Dimethyl-1-phenylbutane-1,2-diol (**1o**)

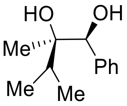

1o

White solid, 19 mg, 49% yield, mp 58–60 ℃. ^{1}H NMR (400 MHz, $CDCl_3$) δ 7.43 (d, *J* = 6.8 Hz, 2H), 7.36–7.28 (m, 3H), 4.62 (d, *J* = 5.0 Hz, 1H), 2.39 (d, *J* = 5.0 Hz, 1H), 1.83 (s, 1H), 7.36–7.28 (m, 1H), 1.22 (s, 3H), 0.93 (d, *J* = 6.8 Hz, 3H), 0.86 (d, *J* = 6.8 Hz, 3H). ^{13}C NMR (100 MHz, $CDCl_3$) δ 140.9, 128.3, 128.0, 128.0, 78.5, 77.2, 33.4, 19.1, 17.9, 16.9. HRMS (ESI, m/z): calcd. for $C_{12}H_{18}O_2Na^+$ 217.1199, found 217.1198; IR (KBr thin film, cm^{-1}): ν 3647, 2962, 2924, 2360, 1451, 1375, 1015, 700. $[\alpha]_D^{25}$: –14.2 (c 0.1, $CHCl_3$); HPLC analysis: 96% *ee* (Chiralcel OD-H, 10:90 ^{i}PrOH/hexane, 1.0 mL/min, 254nm), R_t (major) = 5.4 min, R_t (minor) = 4.6 min.

(1*R*,2*S*)-2-Hydroxy-2,3-dimethyl-1-phenylbutyl propionate (**2o**)

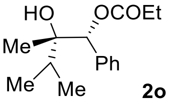

2o

White solid, 22.8 mg, 45% yield, mp 56–58 ℃. ^{1}H NMR (400 MHz, $CDCl_3$) δ 7.42 (dd, *J* = 8.0, 1.6 Hz, 2H), 7.35–7.27 (m, 3H), 5.81 (s, 1H), 2.47–2.31 (m, 2H), 1.63–1.53 (m, 1H), 1.48 (s, 1H), 1.16–1.12 (m, 6H), 0.95 (d, *J* = 6.8 Hz, 3H), 0.93 (d, *J* = 6.8 Hz, 3H). ^{13}C NMR (100 MHz, $CDCl_3$) δ 173.0, 136.8, 128.1, 127.9, 127.8, 78.5, 75.9, 33.3, 27.6, 19.0, 17.6, 16.3, 8.9. HRMS (ESI, m/z): calcd. for $C_{15}H_{22}O_3Na^+$ 273.1461, found 273.1459; IR (KBr thin film, cm^{-1}): ν 3450, 2983, 1734, 1644, 1382, 1261, 1019, 763, 697. $[\alpha]_D^{25}$: –45.6 (c 0.1, $CHCl_3$); HPLC analysis: 91% *ee* (Chiralcel IA, 2:98 ^{i}PrOH/hexane, 1.0 mL/min, 254 nm), R_t (major) = 6.4 min, R_t (minor) = 5.2 min.

(1*S*,2*S*)-2-Methyl-1-phenylpent-4-ene-1,2-diol (**1p**)

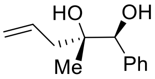

1p

Colorless oil, 19 mg, 49% yield. ^{1}H NMR (400 MHz, $CDCl_3$) δ 7.39–7.27 (m, 5H), 5.99–5.89 (m, 1H), 5.20–5.13 (m, 2H), 4.55 (s, 1H), 2.64 (s, 1H), 2.38–2.25 (m, 2H), 2.15 (s, 1H), 1.02 (s, 3H). ^{13}C NMR (100 MHz, $CDCl_3$) δ 140.3, 133.6, 128.1, 128.0, 127.7, 119.3, 79.3, 74.8, 43.6, 21.9. HRMS (ESI, m/z): calcd. for $C_{12}H_{18}O_2Na^+$ 217.1199, found 217.1198; HRMS (ESI, m/z): calcd. for $C_{12}H_{16}O_2Na^+$ 215.1041, found 215.1043; IR (KBr thin film, cm^{-1}): ν 3515, 3377, 2364, 2322, 1651, 1544, 1503, 1268, 1219, 770. $[\alpha]_D^{25}$: +36.5 (c 0.1, $CHCl_3$); HPLC analysis: 97% *ee* (Chiralcel OD-H, 5:95 ^{i}PrOH/hexane, 1.0 mL/min, 254 nm), R_t (major) = 7.5 min, R_t (minor) = 6.4 min.

(1*R*,2*R*)-2-Hydroxy-2-methyl-1-phenylpent-4-en-1-yl propionate (**2p**)

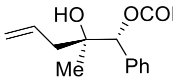

2p

Colorless oil, 23 mg, 46% yield. ^{1}H NMR (600 MHz, $CDCl_3$) δ 7.37–7.28 (m, 5H), 5.92–5.85 (m, 1H), 5.63 (s, 1H), 5.17–5.15 (m, 1H), 5.12–5.08 (m, 1H), 2.47–2.36 (m, 2H), 2.33–2.27 (m, 2H), 1.83 (s, 1H), 1.15 (t, *J* = 7.6 Hz, 3H), 1.08 (s, 3H). ^{13}C NMR (151 MHz, $CDCl_3$) δ 173.4, 137.1, 133.2, 128.3, 128.2, 128.1, 119.2, 80.1, 74.0, 43.6, 27.9, 23.2, 9.2. HRMS (ESI, m/z): calcd. for $C_{15}H_{20}O_3Na^+$ 271.1305, found 271.1304; IR (KBr thin film, cm^{-1}): ν 3474, 3073, 2983, 2941, 2367, 2326, 1738, 1641, 1454, 1364, 1274, 1181, 1015, 918, 759. $[\alpha]_D^{25}$: –31.7 (c 0.1, $CHCl_3$); HPLC analysis: 95% *ee* (Chiralcel IA, 5:95 iPrOH/hexane, 1.0 mL/min, 254 nm), R_t (major) = 5.2 min, R_t (minor) = 5.5 min.

(1*S*,2*S*)-1-(4-Methoxyphenyl)-2,6-dimethylhept-5-ene-1,2-diol (**1q**)

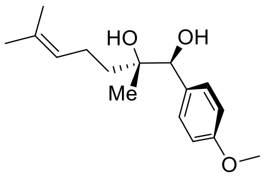

1q

Colorless oil, 23 mg, 44% yield. ^{1}H NMR (400 MHz, $CDCl_3$) δ 7.29 (d, *J* = 8.6 Hz, 2H), 6.86 (d, *J* = 8.6 Hz, 2H), 5.11 (t, *J* = 7.0 Hz, 1H), 4.50 (d, *J* = 2.5 Hz, 1H), 3.80 (s, 3H), 2.54 (d, *J* = 3.0 Hz, 1H), 2.16–2.07 (m, 3H), 1.68 (s, 3H), 1.62 (s, 3H), 1.57–1.45 (m, 2H), 1.03 (s, 3H). ^{13}C NMR (100 MHz, $CDCl_3$) δ 159.3, 132.7, 132.0, 128.8, 124.4, 113.5, 79.5, 75.5, 55.3, 38.7, 25.8, 22.3, 21.3, 17.8. HRMS (ESI, m/z): calcd. for $C_{16}H_{24}O_3Na^+$ 287.1618, found 287.1616; IR (KBr thin film, cm^{-1}): ν 3450, 2970, 2918, 2358, 1612, 1511, 1456, 1377, 1245, 1173, 1031, 834, 768. $[\alpha]_D^{25}$: +32.4 (c 0.1, $CHCl_3$); HPLC analysis: 96% *ee* (Chiralcel AD-H, 5:95 ^{i}PrOH/hexane, 1.0 mL/min, 254 nm), R_t (major) = 7.8 min, R_t (minor) = 8.7 min.

(1*R*,2*R*)-2-Hydroxy-1-(4-methoxyphenyl)-2,6-dimethylhept-5-en-1-yl propionate (**2q**)

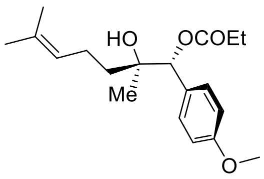

2q

Colorless oil, 33 mg, 52% yield. ^{1}H NMR (600 MHz, $CDCl_3$) δ 7.29 (d, *J* = 8.6 Hz, 2H), 6.85 (d, *J* = 8.8 Hz, 2H), 5.58 (s, 1H), 5.09–5.06 (m, 1H), 3.79 (s, 3H), 2.45–2.33 (m, 2H), 2.13–2.03 (m, 2H), 1.72 (s, 1H), 1.67 (s, 3H), 1.60 (s, 3H), 1.51 (t, *J* = 8.4 Hz, 2H), 1.13 (t, *J* = 7.6 Hz, 3H), 1.09 (s, 3H). ^{13}C NMR (150 MHz, $CDCl_3$) δ 173.5, 159.4, 132.0, 129.4, 129.3, 124.2, 113.6, 80.3, 74.4, 55.3, 38.7, 27.9, 25.8, 22.6, 22.3, 17.8, 9.2. HRMS (ESI, m/z): calcd. for $C_{19}H_{28}O_4Na^+$ 343.1880, found 343.1878; IR (KBr thin film, cm^{-1}): ν 3474, 2980, 2925, 2368, 1733, 1615, 1511, 1453, 1370, 1256, 1166, 1024, 801, 744. $[\alpha]_D^{25}$: –40.8 (c 0.1, $CHCl_3$); HPLC analysis: 83% *ee* (Chiralcel

IA, 5:95 iPrOH/hexane, 1.0 mL/min, 254 nm), R_t (major) = 7.6 min, R_t (minor) = 5.8 min.

(1*S*,2*R*)-2-Ethyl-1-phenylhexane-1,2-diol (**1r**)

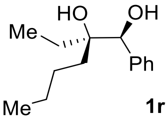

White solid, 21 mg, 47% yield, mp 65–67 °C. ^{1}H NMR (600 MHz, $CDCl_3$) δ 7.37 (d, *J* = 7.0 Hz, 2H), 7.33 (t, *J* = 7.2 Hz, 2H), 7.31–7.28 (m, 1H), 4.59 (d, *J* = 4.4 Hz, 1H), 2.47–2.43 (m, 1H), 1.83 (s, 1H), 1.77–1.71 (m, 1H), 1.64–1.59 (m, 1H), 1.29–1.18 (m, 6H), 0.95 (t, *J* = 7.4 Hz, 3H), 0.82 (t, *J* = 7.1 Hz, 3H). ^{13}C NMR (150 MHz, CDCl3) δ 140.8, 128.2, 127.9, 127.7, 77.6, 76.8, 34.1, 28.0, 25.3, 23.3 14.1, 8.2. HRMS (ESI, m/z): calcd. for $C_{14}H_{22}O_2Na^+$ 245.1512, found 245.1510; IR (KBr thin film, cm^{-1}): ν 3647, 2959, 2931, 2869, 2357, 1537, 1458, 1274, 1095, 977, 752. $[\alpha]_D^{25}$: –13.6 (c 0.1, $CHCl_3$); HPLC analysis: 96% *ee* (Chiralcel OD-H, 2:98 ^{i}PrOH/hexane, 1.0 mL/min, 220nm), R_t (major) = 15.6 min, R_t (minor) = 11.8 min.

(1*R*,2*S*)-2-Ethyl-2-hydroxy-1-phenylhexyl propionate (**2r**)

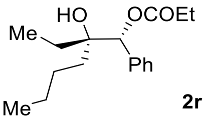

White solid, 23.5 mg, 42% yield, mp 85–87 °C. ^{1}H NMR (600 MHz, $CDCl_3$) δ 7.38–7.37 (m, 2H), 7.34–7.28 (m, 3H), 5.71 (s, 1H), 2.45–2.33 (m, 2H), 1.66–1.57 (m, 2H), 1.31–1.19 (m, 7H), 1.13 (t, *J* = 7.6 Hz, 3H), 0.92 (t, *J* = 7.5 Hz, 3H), 0.85 (t, *J* = 7.2 Hz, 3H). ^{13}C NMR (150 MHz, CDCl3) δ 173.4, 137.2, 128.3, 128.2, 78.7, 76.1, 34.3, 28.1, 27.9, 25.2, 23.3, 14.1, 9.2, 8.0. HRMS (ESI, m/z): calcd. for $C_{17}H_{26}O_3Na^+$ 301.1774, found 301.1772; IR (KBr thin film, cm^{-1}): ν 3472, 2965, 2360, 2333, 2926, 1721, 1644, 1278, 1219, 1012, 770. $[\alpha]_D^{25}$: –35.5 (c 0.1, $CHCl_3$); HPLC analysis: 90% *ee* (Chiralcel IA, 2:98 ^{i}PrOH/hexane, 1.0 mL/min, 220nm), R_t (major) = 6.7 min, R_t (minor) = 6.3 min.

(1*S*,2*S*)-2-Benzyl-1-phenylbutane-1,2-diol (**1s**)

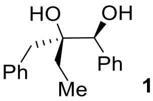

White solid, 25 mg, 48% yield, mp 75–77 °C. ^{1}H NMR (600 MHz, $CDCl_3$) δ 7.38–7.29 (m, 10H), 4.56 (d, *J* = 4.6 Hz, 1H), 3.01 (q, *J* = 13.6 Hz, 1H), 2.97 (d, *J* = 13.6 Hz, 1H), 2.47 (d, *J* = 4.6 Hz, 1H), 1.96 (s, 1H), 1.29–1.22 (m, 1H), 1.20–1.14 (m, 1H), 0.93 (t, *J* = 7.5 Hz, 3H). ^{13}C NMR (151 MHz, $CDCl_3$) δ 140.6, 137.5, 130.6, 128.4, 128.2, 128.1, 128.0, 126.6, 77.2, 77.0, 41.0, 27.4, 7.8. HRMS (ESI, m/z): calcd. for $C_{17}H_{20}O_2Na^+$ 279.1356, found 279.1355; IR (KBr thin film, cm^{-1}): ν 3502, 2959, 2927, 2360, 1651, 1492, 1454, 1261, 1088, 1022, 801, 752, 704. $[\alpha]_D^{25}$: –34.5 (c 0.1, $CHCl_3$); HPLC analysis: 92% *ee* (Chiralcel OD-H, 10:90 ^{i}PrOH/hexane, 1.0 mL/min, 220 nm), R_t (major) = 7.1 min, R_t (minor) = 6.3 min.

(1*R*,2*R*)-2-Benzyl-2-hydroxy-1-phenylbutyl propionate (**2s**)

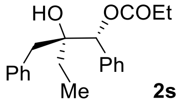

2s

White solid, 29.7 mg, 48% yield, mp 75–76 ℃. ^{1}H NMR (600 MHz, $CDCl_3$) δ 7.33 (d, *J* = 7.0 Hz, 2H), 7.27–7.20 (m, 5H), 7.18–7.13 (m, 3H), 5.65 (s, 1H), 2.84 (d, *J* = 13.8 Hz, 1H), 2.81 (d, *J* = 13.8 Hz, 1H), 2.27 (q, *J* = 7.4 Hz, 2H), 1.67 (s, 1H), 1.28–1.22 (m, 1H), 1.18–1.12 (m, 1H), 1.05 (t, *J* = 7.5 Hz, 3H), 0.87 (t, *J* = 7.4 Hz, 3H). ^{13}C NMR (151 MHz, $CDCl_3$) δ 173.2, 137.1, 137.0, 130.5, 128.4, 128.3, 128.2, 126.6, 78.4, 76.2, 41.8, 28.4, 27.8, 9.1, 7.8. HRMS (ESI, m/z): calcd. for $C_{20}H_{24}O_3Na^+$ 335.1618, found 335.1616; IR (KBr thin film, cm^{-1}): ν 3443, 2969, 2360, 2336, 1738, 1710, 1631, 1458, 1261, 1185, 1081, 1022, 745, 700. $[\alpha]_D^{25}$: –31.8 (c 0.1, $CHCl_3$); HPLC analysis: 91% *ee* (Chiralcel IA, 2:98 ^{i}PrOH/hexane, 1.0 mL/min, 220 nm), R_t (major) = 8.3 min, R_t (minor) = 7.2 min.

(1*S*,2*S*)-2-Benzyl-1,4-diphenylbutane-1,2-diol (**1t**)

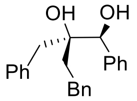

1t

White solid, 29 mg, 44% yield, mp 135–136 ℃. ^{1}H NMR (600 MHz, $CDCl_3$) δ 7.42 (d, *J* = 6.9 Hz, 2H), 7.39–7.27 (m, 8H), 7.23–7.19 (m, 2H), 7.13 (t, *J* = 7.2 Hz, 1H), 7.01 (d, *J* = 7.3 Hz, 2H), 4.65 (d, *J* = 4.0 Hz, 1H), 3.09 (d, *J* = 13.6 Hz, 1H), 3.04 (d, *J* = 13.6 Hz, 1H), 2.76–2.68 (m, 2H), 2.46 (t, *J* = 3.9 Hz, 1H), 2.13 (s, 1H), 1.60 (ddd, *J* = 17.6, 10.3, 7.3 Hz, 1H), 1.48 (ddd, *J* = 17.2, 11.5, 5.8 Hz, 1H). ^{13}C NMR (151 MHz, $CDCl_3$) δ 142.3, 140.3, 137.2, 130.6, 128.5, 128.4, 128.3, 128.2, 128.1, 126.8, 125.8, 77.6, 76.7, 42.0, 36.7, 29.7. HRMS (ESI, m/z): calcd. for $C_{23}H_{24}O_2Na^+$ 355.1669, found 355.1666; IR (KBr thin film, cm^{-1}): ν 3502, 2948, 2862, 2360, 1655, 1451, 1261, 1185, 1084, 1008, 794, 742. $[\alpha]_D^{25}$: –40.0 (c 0.1, $CHCl_3$); HPLC analysis: 99% *ee* (Chiralcel OD-H, 5:95 ^{i}PrOH/hexane, 1.0 mL/min, 220 nm), R_t (major) = 19.5 min, R_t (minor) = 18.7 min.

(1*R*,2*R*)-2-Benzyl-2-hydroxy-1,4-diphenylbutyl propionate (**2t**)

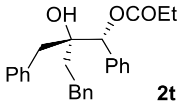

2t

White solid, 38 mg, 49% yield, mp 142–143 ℃. ^{1}H NMR (600 MHz, $CDCl_3$) δ 7.46–7.44 (m, 2H), 7.37–7.29 (m, 5H), 7.26–7.24 (m, 3H), 7.22–7.19 (m, 2H), 7.13 (t, *J* = 7.2 Hz, 1H), 6.99 (d, *J* = 7.0 Hz, 2H), 5.84 (s, 1H), 2.99 (d, *J* = 13.8 Hz, 1H), 2.95 (d, *J* = 13.8 Hz, 1H), 2.76–2.68 (m, 2H), 2.37 (q, *J* = 7.4 Hz, 2H), 1.86 (s, 1H), 1.63 (ddd, *J* = 17.4, 11.8, 5.6 Hz, 1H), 1.53 (ddd, *J* = 16.4, 11.8, 5.8 Hz, 1H), 1.15 (t, *J* = 7.4 Hz, 3H). ^{13}C NMR (151 MHz, $CDCl_3$) δ 173.2, 141.9, 136.9, 136.7, 128.6, 128.5, 128.4, 128.4, 128.3, 126.8, 125.9, 78.6, 75.9, 42.5, 37.7, 29.6, 27.9, 9.2. HRMS (ESI, m/z): calcd. for $C_{26}H_{28}O_3Na^+$ 411.1931, found 411.1930; IR (KBr thin film, cm^{-1}): ν 3458,

2965, 2848, 2353, 2326, 1724, 1451, 1278, 1188, 1084, 1012, 752, 700. $[\alpha]_D^{25}$: –38.6 (c 0.1, $CHCl_3$); HPLC analysis: 93% *ee* (Chiralcel OD-H, 5:95 ^{i}PrOH/hexane, 1.0 mL/min, 220 nm), R_t (major) = 5.4 min, R_t (minor) = 7.0 min.

(1*S*,2*S*)-2-(4-Methylbenzyl)-1,4-diphenylbutane-1,2-diol (**1u**)

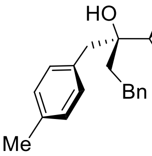

1u

White solid, 33 mg, 48% yield, mp 140–142 °C. ^{1}H NMR (600 MHz, $CDCl_3$) δ 7.42 (d, *J* = 7.0 Hz, 2H), 7.35–7.29 (m, 3H), 7.24 (d, *J* = 8.0 Hz, 2H), 7.20 (t, *J* = 7.4 Hz, 2H), 7.15–7.11 (m, 3H), 7.02 (d, *J* = 7.2 Hz, 2H), 4.64 (d, *J* = 4.0 Hz, 1H), 3.05 (d, *J* = 13.8 Hz, 1H), 2.99 (d, *J* = 13.6 Hz, 1H), 2.76–2.68 (m, 2H), 2.48 (d, *J* = 3.7 Hz, 1H), 2.34 (s, 3H), 2.11 (s, 1H), 1.62–1.56 (m, 1H), 1.48 (ddd, *J* = 20.0, 11.2, 6.0 Hz, 1H). ^{13}C NMR (151 MHz, $CDCl_3$) δ 142.3, 140.4, 136.3, 134.0, 130.5, 129.3, 128.4, 128.3, 128.3, 128.1, 128.1, 125.8, 77.6, 76.7, 41.6, 36.8, 29.7, 21.2. HRMS (ESI, m/z): calcd. for $C_{24}H_{26}O_2Na^+$ 369.1825, found 369.1823; IR (KBr thin film, cm^{-1}): ν 3522, 3439, 2965, 2924, 2855, 2357, 1648, 1506, 1261, 1098, 1015, 770, 693. $[\alpha]_D^{25}$: –20.9 (c 0.1, $CHCl_3$); HPLC analysis: 99% *ee* (Chiralcel AD-H, 5:95 ^{i}PrOH/hexane, 1.0 mL/min, 220 nm), R_t (major) = 14.1 min, R_t (minor) = 13.1 min.

(1*R*,2*R*)-2-Hydroxy-2-(4-methylbenzyl)-1,4-diphenylbutyl propionate (**2u**)

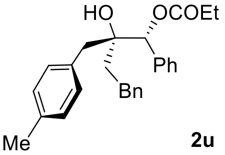

2u

White solid, 38 mg, 48% yield, mp 132–133 °C. ^{1}H NMR (600 MHz, $CDCl_3$) δ 7.43 (d, *J* = 6.8 Hz, 2H), 7.36–7.30 (m, 3H), 7.21 (t, *J* = 7.3 Hz, 2H), 7.14–7.10 (m, 5H), 7.00 (d, *J* = 7.2 Hz, 2H), 5.82 (s, 1H), 2.95 (d, *J* = 13.8 Hz, 1H), 2.91 (d, *J* = 13.8 Hz, 1H), 2.77–2.68 (m, 2H), 2.38 (q, *J* = 7.6 Hz, 2H), 2.32 (s, 3H), 1.84 (s, 1H), 1.62 (ddd, *J* = 17.6, 12.2, 5.4 Hz, 1H), 1.54 (ddd, *J* = 17.6, 11.5, 6.1 Hz, 1H), 1.15 (t, *J* = 7.6 Hz, 3H). ^{13}C NMR (151 MHz, $CDCl_3$) δ 173.2, 142.0, 137.0, 136.4, 133.4, 130.4, 129.2, 128.5, 128.4, 128.4, 128.3, 128.3, 125.9, 78.6, 75.9, 42.0, 37.6, 29.6, 27.9, 21.1, 9.2. HRMS (ESI, m/z): calcd. for $C_{27}H_{30}O_3Na^+$ 425.2087, found 425.2085; IR (KBr thin film, cm^{-1}): ν 3512, 3445, 2962, 2924, 2360, 2326, 1717, 1454, 1261, 1195, 1081, 1012, 749, 702. $[\alpha]_D^{25}$: –33.7 (c 0.1, $CHCl_3$); HPLC analysis: 99% *ee* (Chiralcel AD-H, 5:95 ^{i}PrOH/hexane, 1.0 mL/min, 220 nm), R_t (major) = 9.2 min, R_t (minor) = 8.4 min.

(1*S*,2*R*)-2-Phenethyl-1-phenylhexane-1,2-diol (**1v**)

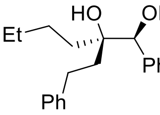

1v

White solid, 28 mg, 47% yield, mp 143–145 °C. ^{1}H NMR (400 MHz, $CDCl_3$) δ 7.38–7.27 (m, 5H), 7.24–7.19 (m, 2H), 7.14–7.05 (m, 3H), 4.64 (d, *J* = 3.8 Hz, 1H), 2.66–

2.51 (m, 2H), 2.45–2.41 (m, 1H), 2.00 (d, J = 6.6 Hz, 1H), 1.81–1.73 (m, 1H), 1.66–1.49 (m, 3H), 1.42–1.35 (m, 4H), 0.94 (t, J = 6.8 Hz, 3H). ^{13}C NMR (150 MHz, $CDCl_3$) δ 142.5, 140.6, 128.4, 128.4, 128.3, 128.0, 127.7, 125.8, 78.0, 76.5, 36.7, 35.5, 29.7, 26.0, 23.4, 14.2. HRMS (ESI, m/z): calcd. for $C_{20}H_{26}O_2Na^+$ 321.1825, found 321.1821; IR (KBr thin film, cm^{-1}): ν 2928, 2856, 2358, 1494, 1460, 1377, 1263, 1024, 803, 741, 696. $[\alpha]_D^{25}$: –12.8 (c 0.1, $CHCl_3$); HPLC analysis: 95% *ee* (Chiralcel AD-H, 5:95 ^{i}PrOH/hexane, 1.0 mL/min, 220 nm), R_t (major) = 10.6 min, R_t (minor) = 9.8 min.

(1*R*,2*S*)-2-Hydroxy-2-phenethyl-1-phenylhexyl propionate (**2v**)

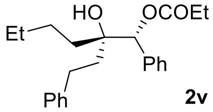

2v

White solid, 34 mg, 48% yield, mp 130–132 °C. ^{1}H NMR (600 MHz, $CDCl_3$) δ 7.41–7.39 (m, 2H), 7.35–7.29 (m, 3H), 7.26–7.23 (m, 2H), 7.18–7.15 (m, 1H), 7.11–7.09 (m, 2H), 5.78 (s, 1H), 2.69–2.61 (m, 2H), 2.47–2.36 (m, 2H), 1.72–1.55 (m, 5H), 1.42–1.32 (m, 4H), 1.16 (t, J = 7.6 Hz, 3H), 0.93 (t, J = 7.2 Hz, 3H). ^{13}C NMR (150 MHz, $CDCl_3$) δ 173.3, 142.2, 137.0, 128.5, 128.4, 128.3, 128.2, 125.9, 78.9, 75.8, 37.1, 35.5, 29.5, 27.9, 25.7, 23.3, 14.1, 9.2. HRMS (ESI, m/z): calcd. for $C_{23}H_{30}O_3Na^+$ 377.2087, found 377.2084; IR (KBr thin film, cm^{-1}): ν 3495, 2959, 2925, 2852, 2365, 1719, 1639, 1456, 1266, 1197, 1083, 1007, 744. $[\alpha]_D^{25}$: –31.6 (c 0.1, $CHCl_3$); HPLC analysis: 95% *ee* (Chiralcel AD-H, 5:95 ^{i}PrOH/hexane, 1.0 mL/min, 220 nm), R_t (major) = 7.5 min, R_t (minor) = 7.1 min.

(1*S*,2*R*)-2-(*tert*-Butyl)-1-phenylhexane-1,2-diol (**1w**)

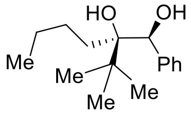

1w

White solid, 24 mg, 48% yield, mp 90–92 °C. ^{1}H NMR (600 MHz, $CDCl_3$) δ 7.46 (d, J = 7.4 Hz, 2H), 7.34–7.31 (m, 2H), 7.29–7.26 (m, 1H), 4.93 (d, J = 3.3 Hz, 1H), 2.01 (d, J = 3.4 Hz, 1H), 1.79–1.67 (m, 2H), 1.50 (s, 1H), 1.26–1.13 (m, 4H), 1.01 (s, 9H), 0.82 (t, J = 7.2 Hz, 3H). ^{13}C NMR (150 MHz, CDCl3) δ 142.4, 128.1, 127.8, 127.7, 78.3, 77.1, 39.1, 31.2, 27.1, 26.7, 23.7, 14.1. HRMS (ESI, m/z): calcd. for $C_{16}H_{26}O_2Na^+$ 273.1825, found 273.1824; IR (KBr thin film, cm^{-1}): ν 3526, 3446, 3384, 2962, 2921, 2360, 2333, 1651, 1537, 1278, 1019, 794, 749, 700. $[\alpha]_D^{25}$: –30.8 (c 0.1, $CHCl_3$); HPLC analysis: 91% *ee* (Chiralcel AD-H, 3:97 ^{i}PrOH/hexane, 1.0 mL/min, 254nm), R_t (major) = 5.1 min, R_t (minor) = 5.5 min.

(1*R*,2*S*)-2-(*tert*-Butyl)-2-hydroxy-1-phenylhexyl propionate (**2w**)

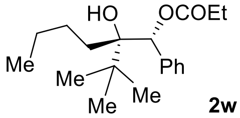

2w

Colorless oil, 30 mg, 49% yield. ^{1}H NMR (600 MHz, $CDCl_3$) δ 7.38–7.35 (m, 2H),

7.32–7.26 (m, 3H), 5.93 (s, 1H), 2.40 (q, J = 7.6 Hz, 2H), 1.79–1.70 (m, 2H), 1.27 (s, 1H), 1.25–1.09 (m, 4H), 1.15 (t, J = 7.6 Hz, 3H), 1.01 (s, 9H), 0.85–0.73 (m, 2H), 0.80 (t, J = 7.2 Hz, 3H). ^{13}C NMR (150 MHz, CDCl3) δ 172.9, 138.6, 128.1, 127.9, 127.8, 77.5, 77.2, 39.2, 31.9, 28.2, 26.9, 26.4, 23.6, 14.0, 9.1. HRMS (ESI, m/z): calcd. for $C_{19}H_{30}O_3Na^+$ 329.2088, found 329.2086; IR (KBr thin film, cm^{-1}): ν 2962, 2924, 2865, 2367, 1752, 1710, 1454, 1278, 1181, 1084, 1077, 796, 759, 742, 697. $[\alpha]_D^{25}$: –38.4 (c 0.1, $CHCl_3$); HPLC analysis: 96% *ee* (Chiralcel IA, 1:99 ^{i}PrOH/hexane, 1.0 mL/min, 254nm), R_t (major) = 5.0 min, R_t (minor) = 3.6 min.

(1*S*,2*S*)-2-*iso*-Propyl-3,3-dimethyl-1-phenylbutane-1,2-diol (**1x**)

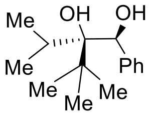

1x

White solid, 17 mg, 37% yield, mp 60–62 ℃. ^{1}H NMR (400 MHz, $CDCl_3$) δ 7.47 (d, J = 6.6 Hz, 2H), 7.34–7.28 (m, 3H), 5.00 (d, J = 3.8 Hz, 1H), 2.47 (s, 1H), 1.96 (d, J = 3.8 Hz, 1H), 1.94–1.84 (m, 1H), 1.19 (s, 9H), 0.99 (d, J = 7.2 Hz, 3H), 0.72 (d, J = 7.2 Hz, 3H). ^{13}C NMR (100 MHz, $CDCl_3$) δ 143.5, 128.7, 128.3, 128.0, 80.5, 77.3, 41.0, 34.8, 29.0, 19.4, 19.2. HRMS (ESI, m/z): calcd. for $C_{15}H_{24}O_2Na^+$ 259.1669, found 259.1666; IR (KBr thin film, cm^{-1}): ν 3443, 2965, 2879, 2353, 1648, 1454, 1368, 1278, 1057, 1005, 749, 704. $[\alpha]_D^{25}$: –24.5 (c 0.1, $CHCl_3$); HPLC analysis: 62% ee (Chiralcel IC, 2:98 ^{i}PrOH/hexane, 1.0 mL/min, 254 nm), R_t (major) = 4.0 min, R_t (minor) = 4.7 min.

(1*R*,2*R*)-2-Hydroxy-2-*iso*-propyl-3,3-dimethyl-1-phenylbutyl propionate (**2x**)

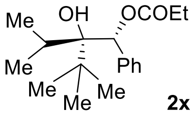

2x

White solid, 28 mg, 48% yield, mp 68–70 ℃. ^{1}H NMR (400 MHz, $CDCl_3$) δ 7.46 (d, J = 7.2 Hz, 2H), 7.31–7.26 (m, 3H), 6.03 (s, 1H), 2.38–2.27 (m, 2H), 2.00 (s, 1H), 1.99–1.93 (m, 1H), 1.10–1.03 (m, 12H), 1.04 (d, J = 7.1 Hz, 3H), 0.82 (d, J = 7.2 Hz, 3H). ^{13}C NMR (100 MHz, $CDCl_3$) δ 172.6, 139.2, 129.1, 128.0, 128.0, 80.2, 78.5, 40.8, 35.1, 28.7, 28.3, 19.3, 19.2, 8.9. HRMS (ESI, m/z): calcd. for $C_{18}H_{28}O_3Na^+$ 315.1931, found 315.1928; IR (KBr thin film, cm^{-1}): ν 3453, 2965, 2917, 2360, 1717, 1638, 1454, 1378, 1261, 1185, 1019, 749, 697. $[\alpha]_D^{25}$: –36.5 (c 0.1, $CHCl_3$); HPLC analysis: 97% ee (Chiralcel OD-H, 10:90 ^{i}PrOH/hexane, 1.0 mL/min, 254 nm), R_t (major) = 2.6 min, R_t (minor) = 2.8 min.

(1*S*,2*S*)-2-Ethyl-1-phenylbut-3-ene-1,2-diol (**1y**)

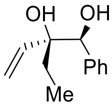

1y

White solid, 17 mg, 44% yield, mp 76–78 ℃. ^{1}H NMR (400 MHz, $CDCl_3$) δ 7.38–7.28 (m, 5H), 5.86–5.79 (m, 1H), 5.38 (d, J = 17.2 Hz, 1H), 5.31 (d, J = 10.8 Hz, 1H), 4.57 (s, 1H), 2.49 (s, 1H), 2.02 (s, 1H), 1.54–1.34 (m, 2H), 0.80 (t, J = 7.4 Hz, 3H).

^{13}C NMR (100 MHz, $CDCl_3$) δ 140.8, 139.4, 128.1, 128.0, 128.0, 115.6, 79.6, 78.4, 28.1, 7.4. HRMS (ESI, m/z): calcd. for $C_{12}H_{16}O_2Na^+$ 215.1043, found 215.1041; IR (KBr thin film, cm^{-1}): ν 3512, 2965, 2917, 2364, 2326, 1658, 1454, 1268, 1102, 1019, 752. $[\alpha]_D^{25}$: –29.5 (c 0.1, $CHCl_3$); HPLC analysis: 82% *ee* (Chiralcel OD-H, 10:90 ^{i}PrOH/hexane, 1.0 mL/min, 254 nm), R_t (major) = 5.0 min, R_t (minor) = 4.2 min.

(1*R*,2*R*)-2-Ethyl-2-hydroxy-1-phenylbut-3-en-1-yl propionate (**2y**)

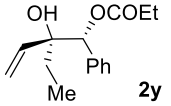

2y

White solid, 23 mg, 46% yield, mp 80–83 ℃. ^1H NMR (600 MHz, $CDCl_3$) δ 7.37–7.35 (m, 2H), 7.34–7.30 (m, 3H), 5.89–5.84 (m, 1H), 5.72 (s, 1H), 5.24 (dd, *J* = 5.7, 1.2 Hz, 1H), 5.22 (s, 1H), 2.42–2.32 (m, 2H), 1.72 (s, 1H), 1.49 (q, *J* = 7.5 Hz, 1H), 1.12 (t, *J* = 7.6 Hz, 3H), 0.83 (t, *J* = 7.6 Hz, 3H). ^{13}C NMR (150 MHz, $CDCl_3$) δ 173.2, 140.1, 136.5, 128.5, 128.4, 128.1, 115.2, 79.8, 77.6, 29.4, 27.9, 9.2, 7.3. HRMS (ESI, m/z): calcd. for $C_{15}H_{20}O_3Na^+$ 271.1305, found 271.1302; IR (KBr thin film, cm^{-1}): ν 3499, 2972, 2360, 2330, 2322, 1717, 1451, 1446, 1281, 1257, 1193, 752. $[\alpha]_D^{25}$: –39.0 (c 0.1, $CHCl_3$); HPLC analysis: 81% *ee* (Chiralcel IA, 2:98 ^{i}PrOH/hexane, 1.0 mL/min, 254 nm), R_t (major) = 7.7 min, R_t (minor) = 6.6 min.

(1*S*,2*S*)-1-Phenyl-2-(phenylethynyl)hexane-1,2-diol (**1z**)

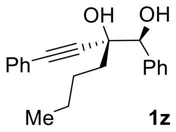

1z

White solid, 27 mg, 46% yield, mp 72–73 ℃. ^1H NMR (600 MHz, $CDCl_3$) δ 7.52–7.50 (m, 2H), 7.44–7.42 (m, 2H), 7.38–7.30 (m, 6H), 4.82 (d, *J* = 3.2 Hz, 1H), 2.78 (d, *J* = 3.6 Hz, 1H), 2.51 (s, 1H), 1.69–1.57 (m, 4H), 1.36–1.29 (m, 2H), 0.90 (t, *J* = 7.4 Hz, 3H). ^{13}C NMR (150 MHz, $CDCl_3$) δ 138.5, 131.8, 128.7, 128.4, 128.3, 128.0, 122.4, 89.9, 86.7, 79.7, 74.9, 36.5, 26.4, 23.0, 14.2. HRMS (ESI, m/z): calcd. for $C_{20}H_{22}O_2Na^+$ 317.1512, found 317.1513; IR (KBr thin film, cm^{-1}): ν 3512, 2962, 2921, 2862, 2364, 2329, 1651, 1541, 1264, 1098, 1015, 801, 752. $[\alpha]_D^{25}$: –30.7 (c 0.1, $CHCl_3$); HPLC analysis: 86% *ee* (Chiralcel AS-H, 2:98 ^{i}PrOH/hexane, 1.0 mL/min, 220 nm), R_t (major) = 14.6 min, R_t (minor) = 16.1 min.

(1*R*,2*R*)-2-Hydroxy-1-phenyl-2-(phenylethynyl)hexyl propionate (**2z**)

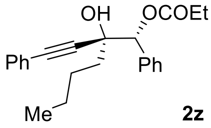

2z

White solid, 33 mg, 47% yield, mp 77–79 ℃. ^1H NMR (600 MHz, $CDCl_3$) δ 7.51 (dd, *J* = 7.8, 1.4 Hz, 2H), 7.41–7.30 (m, 8H), 5.87 (s, 1H), 2.50–2.39 (m, 2H), 2.22 (s, 1H), 1.72–1.60 (m, 4H), 1.37–1.30 (m, 2H), 1.16 (t, *J* = 7.6 Hz, 3H), 0.91 (t, *J* = 7.4 Hz, 3H). ^{13}C NMR (150 MHz, $CDCl_3$) δ 173.2, 138.7, 136.0, 131.7, 128.7, 128.6, 128.4,

128.1, 122.5, 89.4, 86.3, 79.6, 73.6, 37.8, 27.9, 26.3, 22.9, 14.2, 9.2. HRMS (ESI, m/z): calcd. for $C_{23}H_{26}O_3Na^+$ 373.1774, found 373.1776; IR (KBr thin film, cm^{-1}): ν 3446, 2962, 2917, 2862, 2367, 2333, 1741, 1638, 1385, 1264, 1174, 1019, 806, 749. $[\alpha]_D^{25}$: –32.2 (c 0.1, $CHCl_3$); HPLC analysis: 86% *ee* (Chiralcel AS-H, 2:98 ^{i}PrOH/hexane, 1.0 mL/min, 220 nm), Rt (major) = 5.5 min, Rt (minor) = 4.9 min.

(1*R*,2*S*)-2-(4-Chlorophenyl)-1-cyclohexyl-1-phenylethane-1,2-diol (**1za**)

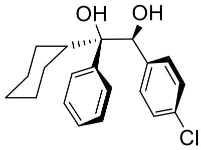

1za

White solid, 159.0 mg, 47% yield, mp 137–139 °C. ^{1}H NMR (600 MHz, $CDCl_3$) δ 7.19–7.14 (m, 3H), 7.10–7.08 (m, 2H), 7.07–7.04 (m, 2H), 6.96–6.93 (m, 2H), 5.23 (d, *J* = 4.2 Hz, 1H), 2.59 (d, *J* = 4.4 Hz, 1H), 2.53 (s, 1H), 2.10 (d, *J* = 13.0 Hz, 1H), 1.98–1.92 (m, 1H), 1.82–1.79 (m, 1H), 1.72–1.70 (m, 1H), 1.64 (d, *J* = 12.7 Hz, 2H), 1.33–1.18 (m, 2H), 1.10–1.03 (m, 2H), 1.00–0.93 (m, 1H); ^{13}C NMR (150 MHz, CDCl3) δ 140.5, 138.8, 133.5, 129.5, 127.9, 127.4, 126.9, 81.6, 75.7, 44.9, 28.1, 27.7, 27.0, 26.7, 26.6; IR (KBr thin film, cm^{-1}): ν 3550, 3462, 3119, 3035, 2969, 2930, 2853, 1611, 1519, 1491, 1411, 1307, 1239, 1093, 1039, 833, 721. $[\alpha]_D^{25}$: –7.3 (c 1.2, $CHCl_3$); HPLC analysis: 96% *ee* (Chiralcel IA, 5:95 ^{i}PrOH/Hexane, 1.0 mL/min, 220 nm), R_t (major) = 15.6 min, R_t (minor) = 14.3 min.

(1*R*,2*S*)-1-(4-Chlorophenyl)-2-cyclohexyl-2-hydroxy-2-phenylethyl propionate (**2za**)

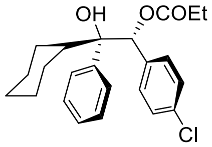

2za

White solid, 186.0 mg, 48% yield, mp 127–129 °C. ^{1}H NMR (600 MHz, $CDCl_3$) δ 7.26–7.18 (m, 5H), 7.08–7.05 (m, 2H), 7.01–7.00 (m, 2H), 6.37 (s, 1H), 2.49–2.38 (m, 2H), 2.05 (s, 1H), 2.00–1.90 (m, 1H), 1.75–1.61 (m, 4H), 1.29–1.24 (m, 2H), 1.17 (t, *J* = 7.6 Hz, 3H), 1.15–1.06 (m, 2H), 0.99–0.94 (m, 1H), 0.64–0.57 (m, 1H); ^{13}C NMR (150 MHz, $CDCl_3$) δ 173.6, 140.0, 135.6, 133.8, 130.0, 128.1, 127.6, 127.2, 126.9, 80.8, 77.0, 46.3, 28.1, 27.6, 27.1, 26.7, 26.5, 9.4; HRMS (ESI, m/z): calcd. for $C_{23}H_{27}O_3ClNa^+$ 409.1541, found 409.1544; IR (KBr thin film, cm^{-1}): ν 3543, 3152, 3043, 2979, 2929, 2853, 1716, 1618, 1540, 1489, 1417, 1304, 1234, 1091, 1017, 835, 719. $[\alpha]_D^{25}$: –10.7 (c 1.1, $CHCl_3$); HPLC analysis: 96% *ee* (Chiralcel IA-H, 5:95 ^{i}PrOH/Hexane, 1.0 mL/min, 220 nm), R_t (major) = 5.3 min, R_t (minor) = 9.2 min.

(*R*)-(2-((*R*)-Hydroxy(phenyl)methyl)phenyl)(phenyl)(o-tolyl)methanol (**1aa**)

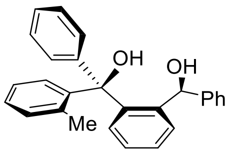

1aa

White solid, 28 mg, 48% yield, mp 135–137 ℃. ^{1}H NMR (600 MHz, $CDCl_3$) δ 7.74

(brs, 1H), 7.46 (brs, 1H), 7.35 (t, *J* = 6.8 Hz, 2H), 7.28–7.26 (m, 2H), 7.26–7.23 (m, 3H), 7.20–7.17 (m, 2H), 7.08 (td, *J* = 7.8, 1.3 Hz, 1H), 7.05–6.99 (m, 4H), 6.89 (brs, 1H), 6.78 (dd, *J* = 7.8, 1.3 Hz, 1H), 6.60 (dd, *J* = 7.8, 0.9 Hz, 1H), 3.93 (s, 1H), 3.54 (d, *J* = 3.4 Hz, 1H), 2.27 (s, 3H); ^{13}C NMR (150 MHz, $CDCl_3$) δ 146.9, 145.1, 143.9, 143.3, 142.8, 138.6, 133.1, 131.2, 129.7, 129.1, 128.3, 128.0, 127.6, 126.8, 126.8, 126.3, 125.3, 85.3, 71.7, 23.0. HRMS (ESI, m/z): calcd. for $C_{27}H_{24}O_2Na^+$ 403.1669, found 403.1666; IR (KBr thin film, cm^{-1}): ν 2365, 2330, 1273, 1259, 1093, 1014, 806, 751. $[\alpha]_D^{25}$: –26.4 (c 0.1, $CHCl_3$); HPLC analysis: 99% *ee* (Chiralcel IA, 10:90 ^{i}PrOH/Hexane, 1.0 mL/min, 220 nm), R_t (major) = 4.0 min, R_t (minor) = 10.8 min.

(*S*)-(2-((*S*)-Hydroxy(phenyl)(o-tolyl)methyl)phenyl)(phenyl)methyl propionate (**2aa**)

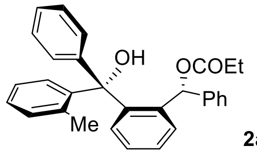

2aa

White solid, 34 mg, 51% yield, mp 126–128 ℃. ^{1}H NMR (600 MHz, $CDCl_3$) δ 7.72 (brs, 1H), 7.51 (dd, *J* = 7.8, 1.3 Hz, 1H), 7.47 (s, 1H), 7.36–7.29 (m, 2H), 7.25 (d, *J* = 7.0 Hz, 1H), 7.2 (dd, *J* = 7.3, 1.0 Hz, 1H), 7.18 (d, *J* = 7.4 Hz, 1H), 7.16–7.12 (m, 2H) 7.11–7.06 (m, 3H), 6.98 (t, *J* = 7.2 Hz, 1H), 6.83 (dd, *J* = 7.9, 1.0 Hz, 1H), 6.73 (d, *J* = 7.2 Hz, 2H), 6.56 (d, *J* = 7.9 Hz, 1H), 5.29 (s, 1H), 2.41–2.31 (m, 2H), 2.29 (s, 3H), 1.13 (t, *J* = 7.6 Hz, 3H); ^{13}C NMR (150 MHz, $CDCl_3$) δ 174.4, 146.6, 145.4, 143.9, 139.8, 139.2, 138.1, 132.7, 130.5, 129.3, 128.0, 127.9, 127.6, 127.3, 127.2, 127.0, 125.0, 84.0, 74.9, 28.0, 22.7, 9.2; HRMS (ESI, m/z): calcd. for $C_{30}H_{28}O_3Na^+$ 459.1931, found 459.1930; IR (KBr thin film, cm^{-1}): ν 3446, 2963, 1719, 1629, 1446, 1266, 1183, 1073, 1024, 765. $[\alpha]_D^{25}$: –21.5 (c 0.1, $CHCl_3$); HPLC analysis: 90% *ee* (Chiralcel IA, 2:98 ^{i}PrOH/Hexane, 1.0 mL/min, 220 nm), R_t (major) = 10.5 min, R_t (minor) = 5.0 min.

(*R*)-(2-((*R*)-Hydroxy(phenyl)methyl)phenyl)(*o*-tolyl)(*p*-tolyl)methanol (**1ab**)

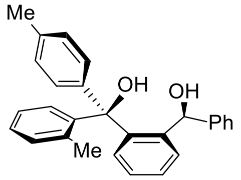

1ab

White solid, 29 mg, 49% yield, mp 130–132 ℃. ^{1}H NMR (400 MHz, $CDCl_3$) δ 7.55 (s, 1H), 7.26–7.16 (m, 9H), 7.09–7.01 (m, 5H), 6.75 (d, *J* = 7.6 Hz, 1H), 6.62 (d, *J* = 8.0 Hz, 1H), 6.15 (d, *J* = 3.4 Hz, 1H), 3.63 (s, 1H), 3.59 (s, 1H), 2.39 (s, 3H), 2.26 (s, 3H). ^{13}C NMR (100 MHz, $CDCl_3$) δ 145.3, 144.0, 143.9, 143.5, 143.0, 138.5, 137.3, 133.0, 131.2, 129.7, 129.1, 128.3, 128.3, 128.0, 126.8, 126.8, 126.4, 125.3, 85.2, 71.6, 23.0, 21.2. HRMS (ESI, m/z): calcd. for $C_{28}H_{26}O_2Na^+$ 417.1825, found 417.1822; IR (KBr thin film, cm^{-1}): ν 3450, 2361, 2323, 1639, 1384, 1259, 1017, 744. $[\alpha]_D^{25}$: –28.6 (c 0.1, $CHCl_3$); HPLC analysis: 83% *ee* (Chiralcel IA, 10:90 ^{i}PrOH/Hexane, 1.0 mL/min, 220 nm), R_t (major) = 4.3 min, R_t (minor) = 10.6 min.

(*S*)-(2-((*S*)-Hydroxy(*o*-tolyl)(*p*-tolyl)methyl)phenyl)(phenyl)methyl propionate (**2ab**)

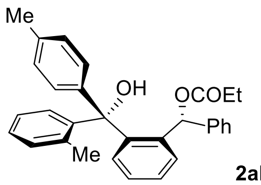
2ab

White solid, 31 mg, 46% yield, mp 125–127 ℃. ^{1}H NMR (600 MHz, $CDCl_3$) δ 7.52–7.50 (m, 2H), 7.45 (s, 1H), 7.32–7.29 (m, 1H), 7.24 (d, *J* = 7.0 Hz, 1H), 7.21–7.17 (m, 1H), 7.14–7.06 (m, 5H), 6.99–6.96 (m, 1H), 6.82 (dd, *J* = 8.0, 1.1 Hz, 1H), 6.75 (d, *J* = 7.4 Hz, 2 H), 6.61 (d, *J* = 7.8 Hz, 2H), 5.18 (s, 1H), 2.39–2.26 (m, 8H), 1.12 (t, *J* = 7.6 Hz, 3H). ^{13}C NMR (150 MHz, $CDCl_3$) δ 174.5, 145.4, 144.2, 143.8, 139.8, 139.3, 138.1, 136.6, 132.7, 130.5, 129.4, 129.2, 128.0, 127.8, 127.6, 127.3, 127.2, 127.1, 125.1, 100.0, 84.0, 75.0, 28.0, 22.8, 21.1, 9.2; HRMS (ESI, m/z): calcd. for $C_{31}H_{30}O_3Na^+$ 473.2083, found 473.2085; IR (KBr thin film, cm^{-1}): ν 3443, 2939, 1736, 1646, 1460, 1263, 1183, 1086, 1014, 748. $[\alpha]_D^{25}$: –26.1 (c 0.1, $CHCl_3$); HPLC analysis: 89% *ee* (Chiralcel IA, 2:98 ^{i}PrOH/Hexane, 1.0 mL/min, 220 nm), R_t (major) = 10.1 min, R_t (minor) = 5.0 min.

(*R*)-(4-Chlorophenyl)(2-((*R*)-hydroxy(phenyl)methyl)phenyl)(*o*-tolyl)methanol (**1ac**)

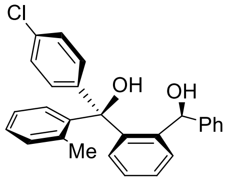
1ac

White solid, 31 mg, 50% yield, mp 149–151 ℃. ^{1}H NMR (600 MHz, CDCl3) δ 7.67 (s, 1H), 7.30–7.25 (m, 6H), 7.23–7.19 (m, 3H), 7.09 (td, *J* = 7.8, 1.5 Hz, 1H), 7.06–7.01 (m, 4H), 6.75 (dd, *J* = 7.8, 1.1 Hz, 1H), 6.55 (d, *J* = 8.4 Hz, 1H), 6.09 (d, *J* = 3.8 Hz, 1H), 4.11 (s, 1H), 3.43 (s, 1H), 2.25 (s, 3H). ^{13}C NMR (150 MHz, CDCl3) δ 145.6, 144.7, 143.6, 142.9 142.6, 138.3, 133.5, 133.2, 131.3, 129.4, 129.0, 128.5, 128.1, 127.0, 127.0, 126.3, 125.4, 84.8, 71.8, 22.8. HRMS (ESI, m/z): calcd. for $C_{27}H_{23}O_2ClNa^+$ 437.1279, found 437.1277; IR (KBr thin film, cm^{-1}): ν 3522, 2928, 2365, 2327, 1276, 1090, 1014, 803, 768, 699. $[\alpha]_D^{25}$: –34.8 (c 0.1, $CHCl_3$); HPLC analysis: 94% *ee* (Chiralcel IA, 10:90 ^{i}PrOH/Hexane, 1.0 mL/min, 220 nm), R_t (major) = 4.2 min, R_t (minor) = 9.4 min.

(*S*)-(2-((*S*)-(4-Chlorophenyl)(hydroxy)(*o*-tolyl)methyl)phenyl)(phenyl)methyl propionate (**2ac**)

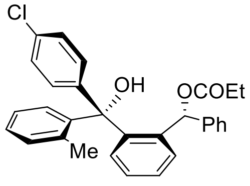
2ac

White solid, 29.5 mg, 42% yield, mp 138–140 ℃. ^{1}H NMR (600 MHz, $CDCl_3$) δ 7.70 (s, 1H), 7.56 (dd, *J* = 7.8 Hz, 1.4 Hz, 1H), 7.38 (s, 1H), 7.36–7.33 (m, 1H), 7.24–7.08 (m, 7H), 6.98 (t, *J* = 8.8 Hz, 1H), 6.82 (dd, *J* = 8.0, 1.3 Hz, 2H), 6.72 (d, *J* = 7.6 Hz,

2H), 6.51 (d, J = 7.9 Hz, 2H), 5.56 (s, 1H), 2.40–2.31 (m, 2H), 2.29 (s, 3H), 1.12 (t, J = 7.5 Hz, 3H). ^{13}C NMR (150 MHz, $CDCl_3$) δ 174.9, 145.3, 144.9, 143.2, 138.9, 138.7, 138.2, 132.9, 132.8, 130.2, 129.2, 129.1, 128.2, 128.0, 127.8, 127.5, 127.5, 125.1, 83.7, 75.2, 28.0, 22.8, 9.2; HRMS (ESI, m/z): calcd. for $C_{30}H_{27}O_3ClNa^+$ 493.1541, found 493.1541; IR (KBr thin film, cm^{-1}): ν 3450, 2355, 1719, 1643, 1280, 1187, 1024, 796, 751. $[\alpha]_D^{25}$: –21.7 (c 0.1, $CHCl_3$); HPLC analysis: 96% *ee* (Chiralcel IA, 2:98 ^{i}PrOH/Hexane, 1.0 mL/min, 220 nm), R_t (major) = 10.3 min, R_t (minor) = 5.2 min.

(*R*)-(4-Fluoro-2-methylphenyl)(2-((*R*)hydroxy(phenyl)methyl)phenyl)(phenyl)methanol (**1ad**)

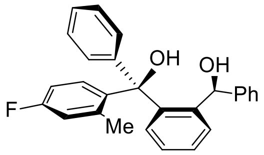

1ad

White solid, 28.6 mg, 48% yield, mp 94–96 ℃. ^{1}H NMR (600 MHz, CDCl3) δ 7.76 (s, 1H), 7.47 (s, 1H), 7.38–7.35 (m, 2H), 7.26–7.18 (m, 5H), 7.11 (td, J = 7.6, 1.3 Hz, 1H), 7.02–6.92 (m, 4H), 6.82–6.77 (m, 2H), 6.33 (dd, J = 11.0, 2.7 Hz, 1H), 6.07 (s, 1H), 4.30 (s, 1H), 3.42 (s, 1H), 2.22 (s, 3H); ^{13}C NMR (150 MHz, CDCl3) δ 160.5 (d, J_{C-F} = 242.2 Hz), 147.1 (d, J_{C-F} = 5.6 Hz), 146.5, 143.4, 142.8, 142.5, 134.1, 134.1, 134.0, 131.3, 129.0, 128.4, 128.1, 127.9, 127.1, 127.0, 126.3, 117.0 (d, J_{C-F} = 23.6 Hz), 114.6 (d, J_{C-F} = 20.5 Hz), 84.8, 71.9, 22.2; HRMS (ESI, m/z): calcd. for $C_{27}H_{23}O_2FNa^+$ 421.1574, found 421.1573; IR (KBr thin film, cm^{-1}): ν 3440, 2361, 2323, 1643, 1384, 1266, 1017, 748. $[\alpha]_D^{25}$: –39.3 (c 0.1, $CHCl_3$); HPLC analysis: 91% *ee* (Chiralcel IA, 10:90 ^{i}PrOH/Hexane, 1.0 mL/min, 220 nm), R_t (major) = 4.8 min, R_t (minor) = 12.8 min

(*S*)-(2-((*S*)-(4-Fluoro-2-methylphenyl)(hydroxy)(phenyl)methyl)phenyl)(phenyl)methyl propionate (**2ad**)

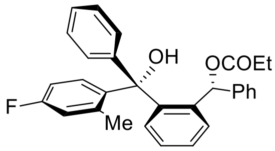

2ad

White solid, 29.8 mg, 44% yield, mp 74–76 ℃. ^{1}H NMR (400 MHz, CDCl3) δ 7.67 (s, 1H), 7.52 (d, J = 7.8 Hz, 1H), 7.41 (s, 1H), 7.32 (t, J = 7.7 Hz, 2H), 7.19–7.05 (m, 8H), 6.88 (td, J = 8.1, 2.7 Hz, 1H), 6.79 (d, J = 8.0 Hz, 1H), 6.73 (d, J = 7.1 Hz, 2H), 6.31 (dd, J = 11.2, 2.5 Hz, 1H), 5.50 (s, 1H), 2.42–2.28 (m, 2H), 2.24 (s, 3H), 1.12 (t, J = 7.6 Hz, 3H); ^{13}C NMR (150 MHz, CDCl3) δ 174.7, 160.5 (d, J_{C-F} = 240.8 Hz), 147.4 (d, J_F = 5.9 Hz), 146.0, 143.3, 139.6, 139.3, 133.8 (d, J_{C-F} = 7.7 Hz), 133.6 (d, J_{C-F} = 2.5 Hz) 130.6, 129.1, 128.3, 127.9, 127.5, 127.4, 127.3, 127.1, 116.7 (d, J_{C-F} = 23.4 Hz), 113.8 (d, J_{C-F} = 20.4 Hz), 83.7, 75.0, 28.0, 21.9, 9.2; IR (KBr thin film, cm^{-1}): ν 3443, 2935, 2351, 1733, 1636, 1446, 1373, 1263, 1173, 1083, 810, 758. $[\alpha]_D^{25}$: –19.1 (c 0.1, $CHCl_3$); HPLC analysis: 86% *ee* (Chiralcel IA, 5:95 ^{i}PrOH/Hexane, 1.0 mL/min, 220 nm), R_t (major) = 5.6 min, R_t (minor) = 3.4 min.

(*S*)-phenyl((2*S*,3*R*)-3-phenyl-3-(*p*-tolyl)oxiran-2-yl)methanol (**3a**)

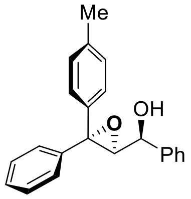

3a

White solid, 30 mg, 46% yield, mp 52–54 ºC. ^{1}H NMR (600 MHz, $CDCl_3$) δ 7.49–7.46 (m, 2H), 7.43–7.40 (m, 2H), 7.39–7.34 (m, 4H), 7.33–7.27 (m, 4H), 7.25–7.23 (m, 2H), 4.13 (dd, *J* = 8.3, 3.8 Hz, 1H), 3.55 (d, *J* = 8.3 Hz, 1H), 2.39 (s, 3H), 1.90 (d, *J* = 3.8 Hz, 1H); ^{13}C NMR (150 MHz, $CDCl_3$) δ 141.5, 140.5, 137.9, 134.1, 129.3, 128.7, 128.4, 128.2, 128.1, 128.0, 127.1, 126.3, 72.1, 68.7, 67.6, 21.4; HRMS (ESI, m/z): calcd. for $C_{22}H_{20}O_2Na^+$ 339.1356, found 339.1362; IR (KBr thin film, cm^{-1}): ν 3304, 3061, 3031, 2924, 2854, 1736, 1602, 1493, 1450, 1381, 814, 699. $[\alpha]_D^{25}$: –5.7 (c 5.5, $CHCl_3$); HPLC analysis: 97% ee (Chiralcel AD-H, 10:90 ^{i}PrOH/Hexane, 1.0 mL/min, 220 nm), R_t (major) = 7.5 min, R_t (minor) = 6.3 min.

(*R*)-phenyl((2*R*,3*S*)-3-phenyl-3-(*p*-tolyl)oxiran-2-yl)methyl propionate (**4a**)

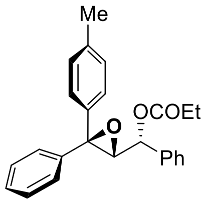

4a

Colorless oil, 33.4 mg, 45% yield. ^{1}H NMR (600 MHz, $CDCl_3$) δ 7.40–7.28 (m, 12H), 7.24–7.22 (m, 2H), 5.09 (d, *J* = 8.3 Hz, 1H), 3.73 (d, *J* = 8.3 Hz, 1H), 2.41–2.38 (m, 5H), 1.16 (t, *J* = 7.6 Hz, 3H); ^{13}C NMR (150 MHz, $CDCl_3$) δ 172.5, 140.2, 138.2, 138.0, 133.3, 129.2, 128.7, 128.4, 128.1, 127.8, 127.0, 126.8, 73.8, 67.7, 67.2, 27.7, 21.6, 9.3; HRMS (ESI, m/z): calcd. for $C_{25}H_{24}O_3Na^+$ 395.1618, found 395.1624; IR (KBr thin film, cm^{-1}): ν 3032, 1750, 1508, 1262, 1167, 1081, 812, 767, 698. $[\alpha]_D^{25}$: +34.7 (c 0.3, $CHCl_3$); HPLC analysis: 93% ee (Chiralcel AD-H, 1:99 ^{i}PrOH/Hexane, 1.0 mL/min, 220 nm), R_t (major) = 7.3 min, R_t (minor) = 5.0 min.

(*S*)-(4-methoxyphenyl)((2*S*,3*R*)-3-phenyl-3-(*p*-tolyl)oxiran-2-yl)methanol (**3b**)

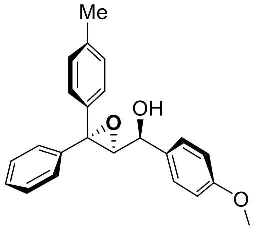

3b

White solid, 32.5 mg, 47% yield, mp 55–57 ºC. ^{1}H NMR (600 MHz, $CDCl_3$) δ 7.48–7.45 (m, 2H), 7.36–7.32 (m, 4H), 7.31–7.27 (m, 3H), 7.25–7.22 (m, 2H), 6.92–6.89 (m, 2H), 4.08 (d, *J* = 8.2 Hz, 1H), 3.81 (s, 3H), 3.54 (d, *J* = 8.2 Hz, 1H), 2.39 (s, 3H), 1.86 (s, 1H); ^{13}C NMR (100 MHz, $CDCl_3$) δ 159.5, 140.5, 137.8, 134.0, 133.5, 129.2, 128.3, 127.9, 127.8, 127.5, 126.9, 114.0, 71.7, 68.6, 67.5, 55.3, 21.3; HRMS (ESI,

m/z): calcd. for $C_{23}H_{22}O_3Na^+$ 369.1461, found 369.1462; IR (KBr thin film, cm^{-1}): ν 3445, 3032, 2956, 2930, 2859, 1716, 1493, 1456, 1379, 1025, 740, 699. $[\alpha]_D^{25}$: –30.0 (c 0.12, $CHCl_3$); HPLC analysis: 99% ee (Chiralcel AD-H, 10:90 ^{i}PrOH/Hexane, 1.0 mL/min, 220 nm), R_t (major) = 13.9 min, R_t (minor) = 10.6 min.

(*R*)-(4-methoxyphenyl)((2*R*,3*S*)-3-phenyl-3-(*p*-tolyl)oxiran-2-yl)methyl propionate (**4b**)

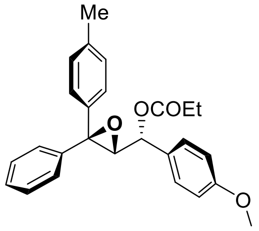

4b

White solid, 41 mg, 51% yield, mp 50–52 ºC. ^{1}H NMR (600 MHz, $CDCl_3$) δ 7.35–7.23 (m, 9H), 7.20–7.17 (m, 2H), 6.89–6.86 (m, 2H), 5.01 (d, *J* = 8.2 Hz, 1H), 3.78 (s, 3H), 3.69 (d, *J* = 8.2 Hz, 1H), 2.40–2.28 (m, 5H), 1.12 (t, *J* = 7.6 Hz, 3H); ^{13}C NMR (150 MHz, $CDCl_3$) δ 172.6, 159.7, 140.3, 138.0, 133.3, 130.2, 129.1, 128.4, 128.3, 128.1, 127.9, 127.0, 114.1, 73.3, 67.8, 67.2, 55.4, 27.7, 21.4, 9.3; HRMS (ESI, m/z): calcd. for $C_{26}H_{26}O_4Na^+$425.1723, found 425.1729; IR (KBr thin film, cm^{-1}): ν 3447,2835, 1771, 1716, 1652, 1515, 1385, 1159, 886, 772, 698. $[\alpha]_D^{25}$: +82.7 (c 0.4, $CHCl_3$); HPLC analysis: 93% ee (Chiralcel AD-H, 10:90 ^{i}PrOH/Hexane, 1.0 mL/min, 220 nm), R_t (major) = 5.8 min, R_t (minor) = 3.9 min.

(*S*)-(4-fluorophenyl)((2*S*,3*R*)-3-phenyl-3-(*p*-tolyl)oxiran-2-yl)methanol (**3c**)

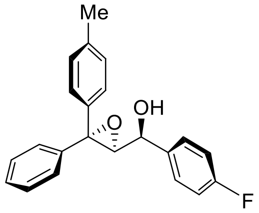

3c

Colorless oil, 30 mg, 45% yield. ^{1}H NMR (600 MHz, $CDCl_3$) δ 7.48–7.45 (m, 2H), 7.40–7.36 (m, 2H), 7.35–7.33 (m, 2H), 7.32–7.22 (m, 5H), 7.07–7.03 (m, 2H), 4.11 (dd, *J* = 8.3, 3.4 Hz, 1H), 3.51 (d, *J* = 8.3 Hz, 1H), 2.40 (s, 3H), 1.95 (d, *J* = 3.4 Hz, 1H); ^{13}C NMR (150 MHz, $CDCl_3$) δ 161.3 (d, J_{C-F} = 244.6 Hz), 139.0, 136.7, 135.9 (d, J_{C-F} = 1.7 Hz), 132.6, 128.0, 127.1, 126.8, 126.6, 126.6, 125.7, 114.2 (d, J_{C-F} = 21.4 Hz), 70.2, 67.3, 66.3, 20.1; HRMS (ESI, m/z): calcd. for $C_{22}H_{19}O_2FNa^+$ 357.1261, found 357.1267; IR (KBr thin film, cm^{-1}): ν3649, 3033, 2931, 2860, 1751, 1559, 1457, 1397, 1217, 767, 698. $[\alpha]_D^{25}$: –18.8 (c 0.7, $CHCl_3$); HPLC analysis: 92% ee (Chiralcel AD-H, 10:90 ^{i}PrOH/Hexane, 1.0 mL/min, 220 nm), R_t (major) = 8.5 min, R_t (minor) = 6.3 min.

(*R*)-(4-fluorophenyl)((2*R*,3*S*)-3-phenyl-3-(*p*-tolyl)oxiran-2-yl)methyl propionate (**4c**)

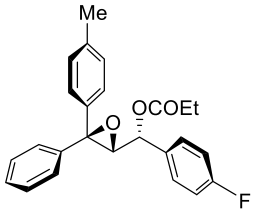

4c

Colorless oil, 35 mg, 45% yield. ^{1}H NMR (400 MHz, $CDCl_3$) δ 7.26–7.15 (m, 9H), 7.15–7.08 (m, 2H), 7.00–6.90 (m, 2H), 4.96 (d, *J* = 8.2 Hz, 1H), 3.60 (d, *J* = 8.2 Hz, 1H), 2.36–2.20 (m, 5H), 1.06 (t, *J* = 7.5 Hz, 3H); ^{13}C NMR (150 MHz, $CDCl_3$) δ 171.2, 161.5 (d, $J_{C\text{-}F}$ = 244.9 Hz), 138.8, 136.9, 132.7 (d, $J_{C\text{-}F}$ =2.6 Hz), 131.9, 128.0, 127.4 (d, $J_{C\text{-}F}$ = 8.4 Hz), 127.2, 126.9, 126.5, 125.7, 114.4 (d, $J_{C\text{-}F}$ = 21.5 Hz), 71.9, 66.6, 65.8, 26.4, 20.2, 8.0; HRMS (ESI, m/z):calcd. for $C_{25}H_{23}O_3FNa^+$ 413.1523, found 413.1522 IR (KBr thin film, cm^{-1}): ν 2931, 2860, 1751, 1558, 1508, 1457, 1262, 1217, 887, 766, 698. $[\alpha]_D^{25}$: +14.2 (c 0.6, $CHCl_3$); HPLC analysis: 92% ee (Chiralcel AD-H, 0.6:99.4 ^{i}PrOH/Hexane, 1.0 mL/min, 220 nm), R_t (major) = 11.7 min, R_t (minor) = 6.0 min.

<u>(*S*)-naphthalen-1-yl((2*S*,3*R*)-3-phenyl-3-(*p*-tolyl)oxiran-2-yl)methanol (**3d**)</u>

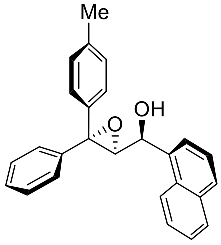

3d

White solid, 35 mg, 48% yield, mp 47–49 ºC. ^{1}H NMR (400 MHz, $CDCl_3$) δ 8.06–8.00 (m, 1H), 7.90–7.70 (m, 3H), 7.60–7.56 (m, 2H), 7.52–7.45 (m, 3H), 7.40–7.25 (m, 7H), 4.90 (dd, *J* = 8.2, 3.5 Hz, 1H), 3.79 (d, *J* = 8.2 Hz, 1H), 2.43 (s, 3H), 1.99 (d, *J* = 3.5 Hz, 1H); ^{13}C NMR (150 MHz, $CDCl_3$) δ 140.5, 138.0, 137.3, 134.0, 131.2, 129.4, 128.9, 128.8, 128.4, 128.1, 128.0, 127.0, 126.2, 125.8, 125.5, 124.1, 69.9, 68.7, 67.3, 21.4; HRMS (ESI, m/z): calcd. for $C_{26}H_{22}O_2Na^+$ 389.1512, found 389.1518; IR (KBr thin film, cm^{-1}): ν 3308, 3061, 2925, 2854, 1736, 1597, 1492, 1459, 1383, 1261, 801. $[\alpha]_D^{25}$: –16.0 (c 0.2, $CHCl_3$); HPLC analysis: 96% ee (Chiralcel AD-H, 10:90 ^{i}PrOH/Hexane, 1.0 mL/min, 220 nm), R_t (major) = 9.2 min, R_t (minor) = 7.0 min.

<u>(*R*)-naphthalen-1-yl((2*R*,3*S*)-3-phenyl-3-(*p*-tolyl)oxiran-2-yl)methyl propionate (**4d**)</u>

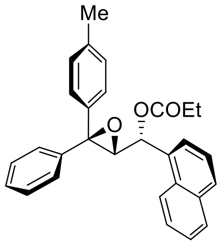

4d

White solid, 40 mg, 47% yield, mp 101–103 ºC. ^{1}H NMR (600 MHz, $CDCl_3$) δ 8.05–7.95 (m, 1H), 7.87–7.80 (m, 2H), 7.50–7.40 (m, 6H), 7.37–7.32 (m, 2H), 7.31–7.21 (m, 5H), 5.78 (d, *J* = 8.1 Hz, 1H), 3.91 (m, *J* = 8.1 Hz, 1H), 2.43–2.28 (m, 5H), 1.10 (t,

J = 7.6 Hz, 3H); ^{13}C NMR (150 MHz, $CDCl_3$) δ 172.5, 140.2, 138.1, 134.4, 134.0, 133.2, 131.0, 129.2, 128.8, 128.4, 128.1, 127.9, 127.0, 126.4, 125.9, 125.3, 125.1, 124.0, 72.0, 67.6, 67.5, 27.6, 21.5, 9.2; HRMS (ESI, m/z): calcd. for $C_{29}H_{26}O_3Na^+$ 445.1774, found 445.1; IR (KBr thin film, cm^{-1}): ν 2936, 2861, 1742, 1613, 1517, 1498, 1461, 1249, 1174, 833, 757. $[\alpha]_D^{25}$: +59.0 (c 0.2, $CHCl_3$); HPLC analysis: 96% ee (Chiralcel OD-H, 2:98 ^{i}PrOH/Hexane, 1.0 mL/min, 220 nm), R_t (major) = 5.6 min, R_t (minor) = 4.5 min.

(*S*)-((2*S*,3*S*)-3-(3-chlorophenyl)-3-(4-chlorophenyl)oxiran-2-yl)(phenyl)methanol (**3e**)

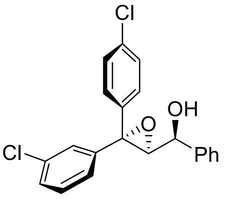

3e

Yellow solid, 35 mg, 47% yield, mp 61–63 ºC. ^{1}H NMR (600 MHz, $CDCl_3$) δ 7.53–7.50 (m, 2H), 7.43–7.40 (m, 2H), 7.38–7.35 (m, 4H), 7.34–7.29 (m, 2H), 7.26–7.20 (m, 2H), 7.19–7.16 (m, 1H), 4.04 (dd, J =8.3, 2.7 Hz, 1H), 3.49 (d, J = 8.3 Hz, 1H), 1.98 (d, J = 2.7 Hz, 1H); ^{13}C NMR (150 MHz, $CDCl_3$) δ 141.9, 141.0, 134.8, 134.6, 134.4, 129.8, 129.6, 128.9, 128.8, 128.6, 128.4, 127.0, 126.2, 125.1, 72.2, 68.8, 66.6; HRMS (ESI, m/z): calcd. for $C_{21}H_{16}O_2Cl_2Na^+$393.0420 found 393.0423; IR (KBr thin film, cm^{-1}): ν 3317,303463, 3032, 2925, 2858, 1737, 1597, 1493, 1453, 1382, 1264, 1092, 1016, 827.751, 700. $[\alpha]_D^{25}$: –9.7 (c 0.4, $CHCl_3$); HPLC analysis: 98% ee (Chiralcel OD-H, 1:99 ^{i}PrOH/Hexane, 1.0 mL/min, 220 nm), R_t (major) = 20.1 min, R_t (minor) = 22.8 min.

(*R*)-((2*R*,3*R*)-3-(3-chlorophenyl)-3-(4-chlorophenyl)oxiran-2-yl)(phenyl)methyl propionate (**4e**)

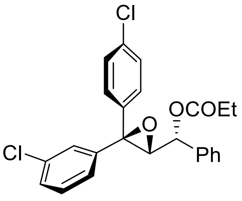

4e

Yellow oil, 41 mg, 48% yield. ^{1}H NMR (600 MHz, $CDCl_3$) δ 7.41–7.38 (m, 4H), 7.37–7.31 (m, 3H), 7.30–7.23 (m, 5H), 7.20–7.16 (m, 1H), 5.01 (d, J = 8.0 Hz, 1H), 3.67 (d, J = 8.0 Hz, 1H), 2.42–2.31 (m, 2H), 1.13 (t, J = 7.5 Hz, 3H); ^{13}C NMR (150 MHz, $CDCl_3$) δ 172.5, 141.5, 137.4, 134.6, 134.2, 129.9, 129.3, 128.9, 128.8, 128.7, 128.6, 127.1, 126.8, 125.2, 73.4, 67.2, 66.7, 27.6, 9.2; HRMS (ESI, m/z): calcd. for $C_{24}H_{20}O_3Cl_2Na^+$ 449.0682, found 449.0685; IR (KBr thin film, cm^{-1}): ν 3036, 2926, 2856, 1752, 1596, 1491, 1458, 1163, 1091, 883, 830, 755, 697. $[\alpha]_D^{25}$: +75.7 (c 0.8, $CHCl_3$); HPLC analysis: 95% ee (Chiralcel OD-H, 1:99 ^{i}PrOH/Hexane, 1.0 mL/min, 220 nm), R_t (major) = 4.0 min, R_t (minor) = 4.8 min.

(*S*)-((2*S*,3*S*)-3-(4-ethylphenyl)-3-(*p*-tolyl)oxiran-2-yl)(phenyl)methanol (**3f**)

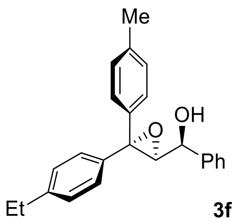

3f

White solid, 31 mg, 45% yield, mp 56–58 ºC. ^{1}H NMR (400 MHz, $CDCl_3$) δ 7.50–7.44 (m, 2H), 7.43–7.34 (m, 4H), 7.33–7.28 (m, 1H), 7.27–7.21 (m, 4H), 7.15–7.10 (m, 2H), 4.11 (dd, *J* =8.3, 3.6 Hz, 1H), 3.55 (d, *J* = 8.3 Hz, 1H), 2.61 (q, *J* = 7.6 Hz, 2H), 2.39 (s, 3H), 1.89 (d, *J* = 3.8 Hz, 1H), 1.19 (q, *J* = 7.6 Hz, 3H); ^{13}C NMR (150 MHz, $CDCl_3$) δ 144.1, 141.4, 137.7, 137.6, 134.2, 129.1, 128.6, 128.1, 127.9, 127.8, 127.0, 126.2, 72.1, 68.5, 67.3, 28.5, 21.3, 15.6; IR (KBr thin film, cm^{-1}): ν 3309, 3034, 2963, 2928, 1734, 1509, 1457, 1385, 1261, 1085, 1024, 799. $[\alpha]_D^{25}$: –5.8 (c 0.8, $CHCl_3$); HPLC analysis: 94% ee (Chiralcel AD-H, 10:90 ^{i}PrOH/Hexane, 1.0 mL/min, 220 nm), R_t (major) = 11.9 min, R_t (minor) = 8.6 min.

(*S*)-((2*S*,3*S*)-3-(4-ethylphenyl)-3-(*p*-tolyl)oxiran-2-yl)(phenyl)methyl propionate (**4f**)

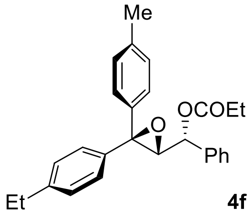

4f

Colorless oil, 38 mg, 47% yield. ^{1}H NMR (600 MHz, $CDCl_3$) δ 7.37–7.32 (m, 4H), 7.32–7.27 (m, 3H), 7.25–7.22 (m, 2H), 7.20–7.17 (m, 2H), 7.15–7.10 (m, 2H), 5.03 (d, *J* = 8.3 Hz, 1H), 3.69 (d, *J* = 8.3 Hz, 1H), 2.61 (q, *J* = 7.6 Hz, 2H), 2.44–2.28 (m, 5H), 1.20 (t, *J* = 7.6 Hz, 3H), 1.13 (t, *J* = 7.6 Hz, 3H); ^{13}C NMR (150 MHz, $CDCl_3$) δ 172.4, 144.3, 138.2, 137.9, 137.4, 133.4, 129.1, 128.7, 128.4, 127.9, 127.8, 127.0, 126.7, 73.8, 67.6, 67.3, 28.6, 27.7, 21.4, 15.7, 9.3; HRMS (ESI, m/z): calcd. for $C_{27}H_{28}O_3Na^+$ 423.1931, found 423.1936; IR (KBr thin film, cm^{-1}): ν 3031, 2974, 2880, 1751, 1605, 1509, 1456, 1166, 815, 764, 739, 700. $[\alpha]_D^{25}$: +24.5 (c 0.6, $CHCl_3$); HPLC analysis: 95% ee (Chiralcel AD-H, 3:97 ^{i}PrOH/Hexane, 1.0 mL/min, 220 nm), R_t (major) = 4.0 min, R_t (minor) = 4.8 min.

(*S*)-((2*S*,3*R*)-3-(3-chlorophenyl)-3-(4-chlorophenyl)oxiran-2-yl)(phenyl)methanol (**3g**)

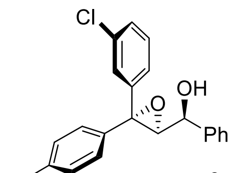

3g

Yellow solid, 34 mg, 46% yield, mp 63–65 ºC. ^{1}H NMR (600 MHz, $CDCl_3$) δ 7.59–7.57 (m, 1H), 7.48–7.46 (m, 1H), 7.40–7.36 (m, 6H), 7.35–7.32 (m, 1H), 7.30–7.24 (m, 4H), 4.09 (dd, *J* = 8.3, 3.6 Hz, 1H), 3.52 (d, *J* = 8.3 Hz, 1H), 1.90 (d, *J* = 3.6 Hz, 1H); ^{13}C NMR (150 MHz, $CDCl_3$) δ 141.0, 138.6, 138.1, 134.7, 134.3, 129.9, 128.8,

128.7, 128.6, 128.5, 128.4, 128.3, 126.3, 126.2, 72.2, 68.7, 66.6; HRMS (ESI, m/z): calcd. for $C_{21}H_{16}O_2Cl_2Na^+$ 393.0420, found 393.0424; IR (KBr thin film, cm^{-1}): ν 3030, 2953, 2725, 2854, 1737, 1492, 1460, 1376, 1261, 1092, 1016, 792, 755,699. $[\alpha]_D^{25}$: –15.7 (c 0.1, $CHCl_3$); HPLC analysis: 91% ee (Chiralcel OD-H, 1:99 ^{i}PrOH/Hexane, 1.0 mL/min, 220 nm), R_t (major) = 8.7 min, R_t (minor) = 7.8 min.

(*R*)-((2*R*,3*S*)-3-(3-chlorophenyl)-3-(4-chlorophenyl)oxiran-2-yl)(phenyl)methyl propionate (**4g**)

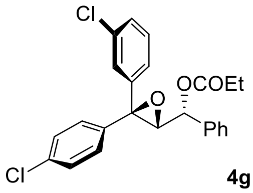

4g

Yellow oil, 40 mg, 47% yield. ^{1}H NMR (600 MHz, $CDCl_3$) δ 7.44–7.40 (m, 1H), 7.39–7.32 (m, 6H), 7.32–7.28 (m, 4H), 7.27–7.26 (m, 1H), 7.26–7.23 (m, 1H), 5.01 (d, *J* = 8.2 Hz, 1H), 3.68 (d, *J* = 8.2 Hz, 1H), 2.38 (q, *J* = 7.6 Hz, 2H), 1.14 (t, *J* = 7.6 Hz, 3H); ^{13}C NMR (150 MHz, $CDCl_3$) δ 172.4, 137.9, 137.7, 137.5, 134.5, 134.5, 130.0, 128.8, 128.7, 128.4, 128.1, 126.7, 126.0, 73.5, 67.1, 66.6, 27.6, 9.2; HRMS (ESI, m/z): calcd. for $C_{24}H_{20}O_3Cl_2Na^+$ 449.0682, found 449.0687; IR (KBr thin film, cm^{-1}): ν 2962, 2927, 2855, 1734, 1509, 1457, 1260, 1083, 1022, 799, 751, 701. $[\alpha]_D^{25}$: +20.1 (c 0.3, $CHCl_3$); HPLC analysis: 92% ee (Chiralcel IA, 1:99 ^{i}PrOH/Hexane, 1.0 mL/min, 220 nm), R_t (major) = 5.6 min, R_t (minor) = 5.1 min.

(*S*)-((2*S*,3*R*)-3-hexyl-3-pentyloxiran-2-yl)(phenyl)methanol (**3h**)

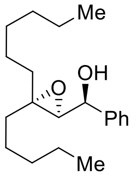

3h

Colorless oil, 28 mg, 47% yield. ^{1}H NMR (600 MHz, $CDCl_3$) δ 7.48–7.42 (m, 2H), 7.40–7.34 (m, 2H), 7.33–7.28 (m, 1H), 4.57 (d, *J* = 8.0 Hz, 1H), 2.91 (d, *J* = 8.0 Hz, 1H), 2.05–1.95 (m, 1H), 1.80–1.72 (m, 1H), 1.70–1.56 (m, 3H), 1.55–1.44 (m, 3H), 1.40–1.21 (m, 12H), 0.95–0.83 (m, 5H); ^{13}C NMR (150 MHz, $CDCl_3$) δ 141.8, 128.7, 128.1, 126.1, 71.8, 66.3, 65.4, 35.2, 32.3, 31.9, 30.2, 29.8, 29.4, 25.3, 24.9, 24.6, 22.7, 14.2; HRMS (ESI, m/z): calcd. for $C_{20}H_{32}O_2Na^+$ 327.2295, found 327.2299; IR (KBr thin film, cm^{-1}): ν 3309, 2963, 2928, 1734, 1654, 1509, 1458, 1386, 1261, 1094, 1025, 799. $[\alpha]_D^{25}$: –13 (c 0.5, $CHCl_3$); HPLC analysis: 99% ee (Chiralcel IC, 1:99 ^{i}PrOH/Hexane, 1.0 mL/min, 220 nm), R_t (major) = 5.8 min, R_t (minor) = 4.9 min.

(*S*)-((2*S*,3*R*)-3-hexyl-3-pentyloxiran-2-yl)(phenyl)methyl propionate (**4h**)

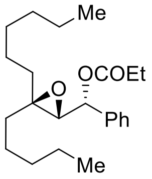

4h

Colorless oil, 36 mg, 50% yield. ^{1}H NMR (600 MHz, $CDCl_3$) δ 7.42–7.34 (m, 4H), 7.32–7.28 (m, 1H), 5.60 (d, *J* = 8.6 Hz, 1H), 3.03 (d, *J* = 8.6 Hz, 1H), 2.40 (q, *J* = 7.6 Hz, 2H), 1.75–1.60 (m, 3H), 1.50–1.25 (m, 15H), 1.16 (t, *J* = 7.6 Hz, 3H), 0.94–0.85 (m, 6H); ^{13}C NMR (150 MHz, $CDCl_3$) δ 172.9, 138.5, 128.7, 128.3, 126.7, 72.7, 65.6, 64.6, 34.9, 32.2, 31.8, 30.1, 29.7, 29.3, 27.8, 25.4, 24.9, 24.6, 22.7, 14.1, 9.2; HRMS (ESI, m/z): calcd. for $C_{23}H_{36}O_3Na^+$ 383.2557, found 383.2558; IR (KBr thin film, cm^{-1}): ν 2958, 2933, 2861, 1750, 1458, 1396, 1262, 1218, 769, 699. $[\alpha]_D^{25}$: +11.0 (c 0.9, $CHCl_3$); HPLC analysis: 95% ee (Chiralcel IC, 0.5:99.5 ^{i}PrOH/Hexane, 1.0 mL/min, 220 nm), R_t (major) = 7.7 min, R_t (minor) = 10.0 min.

(*S*)-((2*S*,3*R*)-3-methyl-3-(trifluoromethyl)oxiran-2-yl)(phenyl)methanol (**3i**)

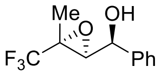

3i

Colorless oil, 22 mg, 47% yield. ^{1}H NMR (600 MHz, $CDCl_3$) δ 7.46–7.40 (m, 4H), 7.39–7.35 (m, 1H), 4.62 (dd, *J* = 7.6, 2.9 Hz, 1H), 3.44 (d, *J* = 7.6 Hz, 1H), 2.07 (d, *J* = 2.9 Hz, 1H), 1.71 (s, 3H); ^{13}C NMR (150 MHz, $CDCl_3$) δ 139.9, 128.8, 128.6, 125.9, 123.4 (q, $J_{C\text{-}F}$ =276.8 Hz), 70.9, 61.0, 58.5 (d, $J_{C\text{-}F}$ = 36.1 Hz), 11.0; HRMS (ESI, m/z): calcd. for $C_{11}H_8ON_3F_3$ 255.0614, found 255.0610; IR (KBr thin film, cm^{-1}): ν 3468, 3066, 3034, 2999, 2945, 1720, 1605, 1496, 1458, 1387, 1338, 1161, 893,746, 700. $[\alpha]_D^{25}$: +66.7 (c 5.4, $CHCl_3$); HPLC analysis: 99% ee (Chiralcel AD-H, 2:98 ^{i}PrOH/Hexane, 1.0 mL/min, 220 nm), R_t (major) = 9.5 min, R_t (minor) = 8.3 min.

(*R*)-((2*R*,3*S*)-3-methyl-3-(trifluoromethyl)oxiran-2-yl)(phenyl)methyl propionate (**4i**)

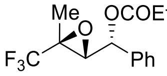

4i

Colorless oil, 27 mg, 47% yield. ^{1}H NMR (600 MHz, $CDCl_3$) δ 7.44–7.34 (m, 5H), 5.60 (d, *J* = 8.3 Hz, 1H), 3.52 (d, *J* = 8.3 Hz, 1H), 2.49–2.38 (m, 2H), 1.66 (s, 3H), 1.17 (t, *J* = 7.6 Hz, 3H); ^{13}C NMR (100 MHz, $CDCl_3$) δ 171.8, 135.5, 127.9, 127.8, 125.7, 122.4 (q, $J_{C\text{-}F}$ =277.4 Hz), 70.1, 58.7 (d, $J_{C\text{-}F}$ = 2.6 Hz), 57.9 (d, $J_{C\text{-}F}$ = 36.0 Hz), 26.5, 10.3, 8.0; HRMS (ESI, m/z): calcd. for $C_{14}H_{15}O_3F_3$ Na^+ 311.0866, found 311.0865; IR (KBr thin film, cm^{-1}): ν 2957, 2926, 2857, 1718, 1508, 1458, 1384, 1262, 1217, 768. $[\alpha]_D^{25}$: –85.2 (c 0.5, $CHCl_3$); HPLC analysis: 94% ee (Chiralcel AD-H, 0.8:99.2 ^{i}PrOH/Hexane, 0.5 mL/min, 40 ℃, 220 nm), R_t (major) = 6.1 min, R_t (minor) = 5.7 min.

(*S*)-((2*S*,3*S*)-3-ethyl-3-methyloxiran-2-yl)(phenyl)methanol (**3j**)

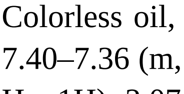

3j

Colorless oil, 18 mg, 47% yield. ^{1}H NMR (600 MHz, $CDCl_3$) δ 7.46–7.43 (m, 2H), 7.40–7.36 (m, 2H), 7.34–7.30 (m, 1H), 4.58 (dd, *J* = 7.9, 2.8 Hz, 1H), 2.93 (d, *J* = 7.9 Hz, 1H), 2.07 (s, 1H), 1.65–1.50 (m, 2H), 1.47 (s, 3H), 0.97 (t, *J* = 7.5 Hz, 3H); ^{13}C NMR (150 MHz, $CDCl_3$) δ 141.6, 128.7, 128.2, 126.1, 72.2, 65.7, 63.2, 31.4, 16.5, 9.3; HRMS (ESI, m/z): calcd. for $C_{12}H_{12}O_2Na^+$ 215.1043, found 215.1045; IR (KBr

thin film, cm^{-1}): ν 3445, 3031, 2954, 2925, 2855, 1559, 1541, 1457, 1378, 1261, 1094, 1024, 888, 749, 699. $[\alpha]_D^{25}$: –24.9 (c 0.6, $CHCl_3$); HPLC analysis: 99% ee (Chiralcel IA, 3:97 ^{i}PrOH/Hexane, 1.0 mL/min, 220 nm), R_t (major) = 8.6 min, R_t (minor) = 7.8 min.

(*R*)-((2*R*,3*R*)-3-ethyl-3-methyloxiran-2-yl)(phenyl)methyl propionate (**4j**)

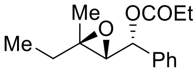

4j

Colorless oil, 24 mg, 48% yield. ^{1}H NMR (600 MHz, $CDCl_3$) δ 7.42–7.35 (m, 4H), 7.34–7.30 (m, 1H), 5.58 (d, *J* = 8.5 Hz, 1H), 3.02 (d, *J* = 8.5 Hz, 1H), 2.48–2.36 (m, 2H), 1.66–1.62 (m, 1H), 1.55–1.48 (m, 1H), 1.43 (s, 3H), 1.17 (t, *J* = 7.6 Hz, 3H), 0.96 (t, *J* = 7.5 Hz, 3H); ^{13}C NMR (150 MHz, $CDCl_3$) δ 171.9, 137.1, 127.6, 127.3, 125.6, 72.0, 63.0, 62.6, 30.0, 26.7, 15.5, 8.2, 8.1; HRMS (ESI, m/z): calcd. for $C_{15}H_{20}O_3Na^+$ 271.1305, found 271.1310; IR (KBr thin film, cm^{-1}): ν 2964, 2927, 2855, 1733, 1508, 1457, 1385, 1260, 1093, 1024, 800, 700. $[\alpha]_D^{25}$: +44.2 (c 0.5, $CHCl_3$); HPLC analysis: 95% ee (Chiralcel IC, 1:99 ^{i}PrOH/Hexane, 1.0 mL/min, 40 °C, 220 nm), R_t (major) = 4.8 min, R_t (minor) = 9.8 min.

(*S*)-((2*S*,3*R*)-3-ethyl-3-methyloxiran-2-yl)(phenyl)methanol (**3k**)

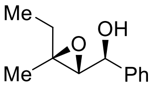

3k

Colorless oil, 18 mg, 47% yield. ^{1}H NMR (600 MHz, $CDCl_3$) δ 7.46–7.43 (m, 2H), 7.40–7.36 (m, 2H), 7.34–7.30 (m, 1H), 4.56 (dd, *J* = 8.1, 2.5 Hz, 1H), 2.96 (d, *J* = 8.1 Hz, 1H), 2.01 (d, *J* = 2.5 Hz, 1H), 1.85–1.78 (m, 1H), 1.76–1.69 (m, 1H), 1.34 (s, 3H), 1.11 (t, *J* = 7.6 Hz, 3H); ^{13}C NMR (150 MHz, $CDCl_3$) δ 141.7, 128.7, 128.2, 126.1, 72.1, 67.2, 63.5, 25.9, 21.6, 9.9; HRMS (ESI, m/z): calcd. for $C_{12}H_{12}O_2Na^+$ 215.1043, found 215.1044; IR (KBr thin film, cm^{-1}): ν 3445, 3031, 2965, 2927, 1600, 1493, 1452, 1380, 1262, 1094, 1024, 801, 748,699. $[\alpha]_D^{25}$: –11.7 (c 0.5, $CHCl_3$); HPLC analysis: 96% ee (Chiralcel IA, 3:97 ^{i}PrOH/Hexane, 1.0 mL/min, 220 nm), R_t (major) = 8.5 min, R_t (minor) = 7.9 min.

(*R*)-((2*R*,3*S*)-3-ethyl-3-methyloxiran-2-yl)(phenyl)methyl propionate (**4k**)

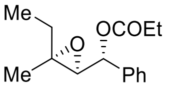

4k

Colorless oil, 24 mg, 48% yield. ^{1}H NMR (600 MHz, $CDCl_3$) δ 7.42–7.34 (m, 4H), 7.33–7.28 (m, 1H), 5.57 (d, *J* = 8.6 Hz, 1H), 3.07 (d, *J* = 8.6 Hz, 1H), 2.46–2.36 (m, 2H), 1.79–1.73 (m, 1H), 1.70–1.63 (m, 1H), 1.33 (s, 3H), 1.16 (t, *J* = 7.5 Hz, 3H), 1.03 (t, *J* = 7.6 Hz, 3H); ^{13}C NMR (150 MHz, $CDCl_3$) δ 173.0, 138.4, 128.7, 128.4, 126.7, 72.9, 65.6, 63.7, 27.8, 25.9, 21.3, 9.8, 9.2; HRMS (ESI, m/z): calcd. for $C_{15}H_{20}O_3Na^+$ 271.1305, found 271.1309; IR (KBr thin film, cm^{-1}): ν 2957, 2931, 2859, 1734, 1508, 1458, 1381, 1217, 1024, 766, 699. $[\alpha]_D^{25}$: +71.2 (c 0.3, $CHCl_3$); HPLC analysis: 96% ee (Chiralcel IC, 1:99 ^{i}PrOH/Hexane, 1.0 mL/min, 40 °C, 220 nm), R_t

(major) = 4.8 min, R_t (minor) = 9.9 min.

Ethyl (*R*)-2-((*S*)-hydroxy(phenyl)methyl)-2-methylpent-4-enoate (**5a**)

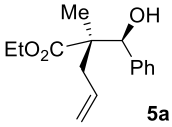

5a

Colorless semisolid, 23.7 mg, 48% yield. ^1H NMR (600 MHz, $CDCl_3$) δ 7.30–7.23 (m, 5H), 5.82–5.72 (m, 1H), 5.05 (ddt, *J* = 6.5, 2.0, 1.1 Hz, 1H), 5.02 (t, *J* = 1.1 Hz, 1H), 4.89 (d, *J* = 3.5 Hz, 1H), 4.14–4.05 (m, 2H), 3.17 (d, *J* = 3.6 Hz, 1H), 2.66 (dd, *J* = 13.8, 6.9 Hz, 1H), 2.18 (ddt, *J* = 13.8, 7.9, 1.0 Hz, 1H), 1.21 (t, *J* = 7.1 Hz, 3H), 1.06 (s, 3H). ^{13}C NMR (151 MHz, $CDCl_3$) δ 176.0, 140.2, 134.4, 127.9, 127.9, 127.6, 118.2, 78.5, 60.9, 51.6, 39.5, 17.7, 14.2. HRMS (ESI, m/z): calcd. for $C_{15}H_{20}O_3Na^+$ 271.1310, found 271.1313. IR (KBr thin film, cm^{-1}): ν 3437, 3238, 2378, 2313, 1637, 1369, 1277, 1259, 762, 745. $[\alpha]_D^{20}$: +17.0 (c 0.2, $CHCl_3$); HPLC analysis: 96% ee (Chiralcel AD-H, 3:97 ^{i}PrOH/hexane, 1.0 mL/min, 220 nm), R_t (major) = 7.1 min, R_t (minor) = 7.9 min.

Ethyl (*S*)-2-methyl-2-((*R*)-phenyl(propionyloxy)methyl)pent-4-enoate (**6a**)

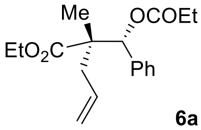

6a

Colorless semisolid, 30.2 mg, 50% yield. ^1H NMR (600 MHz, $CDCl_3$) δ 7.30–7.21 (m, 5H), 6.09 (s, 1H), 5.77–5.69 (m, 1H), 5.07–4.98 (m, 2H), 4.16–3.99 (m, 2H), 2.65 (dd, *J* = 13.7, 7.0 Hz, 1H), 2.45–2.31 (m, 2H), 2.24–2.14 (m, 1H), 1.21 (t, *J* = 7.2 Hz, 3H), 1.15 (t, *J* = 7.6 Hz, 3H), 1.12 (s, 3H). ^{13}C NMR (151 MHz, $CDCl_3$) δ 172.5, 171.9, 136.2, 132.6, 127.0, 126.9, 126.4, 117.4, 77.6, 59.7, 50.3, 39.6, 26.8, 15.5, 13.1, 8.1. HRMS (ESI, m/z): calcd. for $C_{18}H_{24}O_4Na^+$ 327.1572, found 327.1580. IR (KBr thin film, cm^{-1}): ν 3443, 3009, 2982, 2922, 2848, 2378, 2351, 2307, 1741, 1515, 1363, 1280, 1262, 762, 751. $[\alpha]_D^{20}$: –29.3 (c 0.1, $CHCl_3$); HPLC analysis: 94% ee (Chiralcel AD-H, 1:99 ^{i}PrOH/hexane, 1.0 mL/min, 220 nm), R_t (major) = 6.3 min, R_t (minor) = 5.7 min.

Ethyl (*S*)-2-((*S*)-hydroxy(phenyl)methyl)-2-methylpent-4-enoate (**5b**)

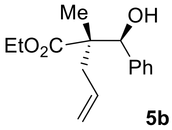

5b

Colorless semisolid, 23.5 mg, 47% yield. ^1H NMR (600 MHz, $CDCl_3$) δ 7.32–7.23 (m, 5H), 5.68 (ddt, *J* = 17.5, 10.3, 7.3 Hz, 1H), 5.10–4.98 (m, 2H), 4.85 (d, *J* = 5.2 Hz, 1H), 4.21–4.11 (m, 2H), 3.27 (d, *J* = 5.3 Hz, 1H), 2.49 (dd, *J* = 13.6, 7.1 Hz, 1H), 2.05–1.94 (m, 1H), 1.24 (t, *J* = 7.1 Hz, 3H), 1.05 (s, 3H). ^{13}C NMR (151 MHz, $CDCl_3$) δ 176.4, 140.2, 133.3, 128.0, 127.9, 127.7, 118.7, 78.4, 61.0, 51.7, 41.5, 16.4, 14.3.

HRMS (ESI, m/z): calcd. for $C_{15}H_{20}O_3Na^+$ 271.1310, found 271.1310. IR (KBr thin film, cm^{-1}): ν 3446, 3062, 3003, 2384, 2357, 2319, 1649, 1518, 1363, 1274, 1262, 765, 751. $[\alpha]_D^{20}$: +87.2 (c 0.1, $CHCl_3$); HPLC analysis: 90% ee (Chiralcel AD-H, 10:90 ^{i}PrOH/hexane, 1.0 mL/min, 220 nm), R_t (major) = 4.2 min, R_t (minor) = 5.0 min.

Ethyl (*R*)-2-methyl-2-((*R*)-phenyl(propionyloxy)methyl)pent-4-enoate (**6b**)

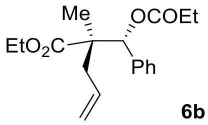

6b

Colorless semisolid, 29.8 mg, 49% yield. ^{1}H NMR (600 MHz, $CDCl_3$) δ 7.35–7.25 (m, 5H), 6.05 (s, 1H), 5.64–557 (m, 1H), 5.07–4.92 (m, 2H), 4.21–4.05 (m, 2H), 2.51 (dd, *J* = 13.5, 7.0 Hz, 1H), 2.40–2.27 (m, 2H), 1.83 (dd, *J* = 13.4, 7.6 Hz, 1H), 1.23 (t, *J* = 7.1 Hz, 3H), 1.14 (s, 3H), 1.10 (t, *J* = 7.6 Hz, 3H). ^{13}C NMR (151 MHz, $CDCl_3$) δ 174.2, 172.8, 136.8, 132.8, 128.2, 128.0, 127.9, 118.8, 79.2, 60.8, 51.0, 40.9, 27.8, 16.0, 14.3, 9.1. HRMS (ESI, m/z): calcd. for $C_{18}H_{24}O_4Na^+$ 327.1572, found 327.1575. IR (KBr thin film, cm^{-1}): ν 3449, 3006, 2985, 2920, 2381, 2307, 1747, 1637, 1509, 1366, 1277, 1259, 765, 748. $[\alpha]_D^{20}$: +32.0 (c 0.1, $CHCl_3$); HPLC analysis: 91% ee (Chiralcel OJ-H, 1:99 ^{i}PrOH/hexane, 1.0 mL/min, 220 nm), R_t (major) = 8.8 min, R_t (minor) = 4.1 min.

Methyl (*R*)-2-((*S*)-hydroxy(phenyl)methyl)-2-phenylpent-4-enoate (**5c**)

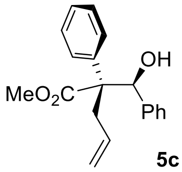

5c

Colorless semisolid, 29.6 mg, 50% yield. ^{1}H NMR (600 MHz, $CDCl_3$) δ 7.29–7.24 (m, 3H), 7.19–7.15 (m, 1H), 7.14–7.08 (m, 2H), 7.01–6.96 (m, 2H), 6.83–6.77 (m, 2H), 6.16–6.08 (m, 1H), 5.35 (d, *J* = 3.5 Hz, 1H), 5.12–5.04 (m, 2H), 4.26 (d, *J* = 3.5 Hz, 1H), 3.73 (s, 3H), 2.91 (ddt, *J* = 14.2, 6.4, 1.3 Hz, 1H), 2.68 (dd, *J* = 14.2, 8.1 Hz, 1H). ^{13}C NMR (151 MHz, $CDCl_3$) δ 177.1, 139.3, 138.5, 135.1, 128.3, 127.9, 127.7, 127.6, 127.5, 127.3, 117.6, 79.3, 60.9, 52.3, 36.0. HRMS (ESI, m/z): calcd. for $C_{19}H_{20}O_3Na^+$ 319.1310, found 319.1312. IR (KBr thin film, cm^{-1}): ν 3452, 3003, 2378, 2348, 2313, 1715, 1643, 1512, 1363, 1259, 765, 748. $[\alpha]_D^{20}$: –132.7 (c 0.2, $CHCl_3$); HPLC analysis: 97% *ee* (Chiralcel AD-H, 10:90 ^{i}PrOH/hexane, 1.0 mL/min, 220 nm), R_t (major) = 10.2 min, R_t (minor) = 4.2 min.

Methyl (*S*)-2-phenyl-2-((*R*)-phenyl(propionyloxy)methyl)pent-4-enoate (**6c**)

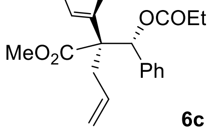

6c

Colorless semisolid, 34.5 mg, 49% yield. ^{1}H NMR (400 MHz, $CDCl_3$) δ 7.29–7.19 (m, 3H), 7.17–7.13 (m, 1H), 7.08 (t, *J* = 7.4 Hz, 2H), 6.99 (dd, *J* = 8.0, 1.5 Hz, 2H), 6.78 (d, *J* = 7.3 Hz, 2H), 6.45 (s, 1H), 5.95 (ddt, *J* = 17.2, 10.2, 7.0 Hz, 1H), 5.16–5.07 (m, 2H), 3.71 (s, 3H), 3.02 (dd, *J* = 14.4, 7.1 Hz, 1H), 2.89 (dd, *J* = 14.4, 7.0 Hz, 1H), 2.45–2.27 (m, 2H), 1.13 (t, *J* = 7.6 Hz, 3H). ^{13}C NMR (101 MHz, $CDCl_3$) δ 173.3, 172.8, 137.9, 136.9, 134.5, 128.3, 127.9, 127.8, 127.6, 127.3, 117.8, 77.9, 59.7, 52.1, 37.4, 28.0, 9.2. HRMS (ESI, m/z): calcd. for $C_{22}H_{24}O_4Na^+$ 375.1572, found 375.1575. IR (KBr thin film, cm^{-1}): ν 3455, 3009, 2381, 2316, 1735, 1640, 1500, 1363, 1277, 1173, 762, 748. $[\alpha]_D^{20}$: –118.4 (c 0.2, $CHCl_3$); HPLC analysis: 96% ee (Chiralcel AD-H, 1:99 ^{i}PrOH/hexane, 1.0 mL/min, 220 nm), R_t (major) = 8.4 min, R_t (minor) = 7.6 min.

Ethyl-(*R*)-2-((1-benzyl-1*H*-1,2,3-triazol-5-yl)methyl)-2-((*S*)-hydroxy(phenyl)methyl)pent-4-enoate (**5d**)

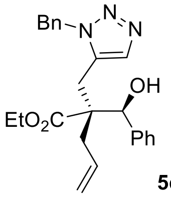

5d

Colorless semisolid, 39.7 mg, 49% yield. ^{1}H NMR (600 MHz, $CDCl_3$) δ 7.39–7.30 (m, 5H), 7.30–7.21 (m, 6H), 5.73 (dddd, *J* = 16.6, 10.3, 8.4, 6.2 Hz, 1H), 5.47 (dd, *J* = 33.4, 14.8 Hz, 2H), 5.11 (d, *J* = 6.7 Hz, 1H), 5.06–4.96 (m, 3H), 4.06–3.93 (m, 2H), 2.91 (s, 2H), 2.50–2.40 (m, 1H), 2.30 (dd, *J* = 14.1, 8.4 Hz, 1H), 1.05 (t, *J* = 7.2 Hz, 3H). ^{13}C NMR (151 MHz, $CDCl_3$) δ 174.5, 145.1, 140.3, 134.8, 134.0, 129.2, 128.8, 128.1, 127.9, 127.7, 123.4, 118.9, 77.1, 60.8, 55.1, 54.2, 37.4, 28.8, 14.0. HRMS (ESI, m/z): calcd. for $C_{24}H_{27}N_3O_3Na^+$ 428.1950, found 428.1950. IR (KBr thin film, cm^{-1}): ν 3437, 2917, 2848, 2381, 2354, 2307, 1720, 1634, 1360, 1277, 771, 751. $[\alpha]_D^{20}$: +14.0 (c 0.2, $CHCl_3$); HPLC analysis: 94% *ee* (Chiralcel OJ-H, 20:80 ^{i}PrOH/hexane, 1.0 mL/min, 220 nm), R_t (major) = 9.4 min, R_t (minor) = 7.1 min.

Ethyl-(*S*)-2-((1-benzyl-1*H*-1,2,3-triazol-5-yl)methyl)-2-((*R*)-phenyl(propionyloxy)methyl)pent-4-enoate (**6d**)

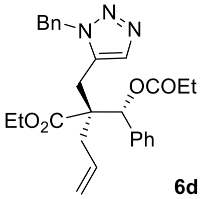

6d

Colorless semisolid, 44.7 mg, 48% yield. ^{1}H NMR (600 MHz, $CDCl_3$) δ 7.30–7.17 (m, 8H), 7.17–7.11 (m, 3H), 6.00 (s, 1H), 5.74–5.62 (m, 1H), 5.37 (dd, *J* = 22.0, 14.9 Hz, 2H), 4.98–4.90 (m, 2H), 4.07–3.88 (m, 2H), 3.02 (d, *J* = 14.9 Hz, 1H), 2.95 (d, *J* = 14.9 Hz, 1H), 2.36 (dd, *J* = 14.3, 6.8 Hz, 1H), 2.26 (dd, *J* = 14.3, 7.8 Hz, 1H), 2.12 (qd, *J* = 7.5, 1.1 Hz, 2H), 1.06 (t, *J* = 7.1 Hz, 3H), 0.97 (t, *J* = 7.6 Hz, 3H). ^{13}C NMR (151 MHz, $CDCl_3$) δ 171.9, 171.5, 143.3, 135.7, 134.0, 132.2, 128.0, 127.5, 127.2,

127.0, 126.9, 126.9, 122.1, 117.9, 77.1, 59.8, 53.6, 52.9, 36.0, 27.6, 26.6, 13.0, 7.9. HRMS (ESI, m/z): calcd. for $C_{27}H_{31}N_3O_4Na^+$ 484.2212, found 484.2206. IR (KBr thin film, cm^{-1}): ν 2979, 2925, 2845, 2381, 2313, 1729, 1596, 1506, 1363, 1274, 1262, 1167, 986, 771, 751. $[\alpha]_D^{20}$: +5.2 (c 0.4, $CHCl_3$); HPLC analysis: 97% *ee* (Chiralcel AD-H, 15:85 ^{i}PrOH/hexane, 1.0 mL/min, 220 nm), R_t (major) = 8.2 min, R_t (minor) = 7.3 min.

Methyl (*R*)-2-((*S*)-(4-chlorophenyl)(hydroxy)methyl)-2-(naphthalen-2-ylmethyl)pent-4-enoate (**5e**)

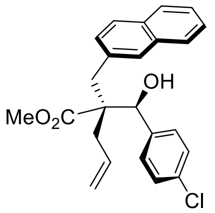

5e

Colorless semisolid, 38.1 mg, 48% yield. ^{1}H NMR (600 MHz, $CDCl_3$) δ 7.85–7.78 (m, 1H), 7.76–7.72 (m, 2H), 7.54 (s, 1H), 7.48–7.44 (m, 2H), 7.39 (d, *J* = 8.6 Hz, 2H), 7.37–7.32 (m, 2H), 7.20 (dd, *J* = 8.4, 1.6 Hz, 1H), 5.95 (ddt, *J* = 17.1, 10.3, 6.9 Hz, 1H), 5.24–5.08 (m, 3H), 3.94 (d, *J* = 3.3 Hz, 1H), 3.50 (s, 3H), 3.48 (d, *J* = 13.7 Hz, 1H), 2.85 (d, *J* = 13.7 Hz, 1H), 2.66 (dd, *J* = 15.2, 6.5 Hz, 1H), 2.23–2.14 (m, 1H). ^{13}C NMR (151 MHz, $CDCl_3$) δ 176.1, 138.3, 134.7, 134.5, 133.8, 133.4, 132.4, 129.5, 129.1, 128.6, 128.1, 127.7, 127.7, 127.6, 126.1, 125.7, 119.0, 76.0, 56.0, 51.7, 38.7, 36.0. HRMS (ESI, m/z): calcd. for $C_{24}H_{23}ClO_3Na^+$ 417.1233, found 417.1232. IR (KBr thin film, cm^{-1}): ν 3470, 3410, 2920, 2851, 1732, 1637, 1218, 1015, 629, 477. $[\alpha]_D^{20}$: –34.2 (c 0.3, $CHCl_3$); HPLC analysis: 96% *ee* (Chiralcel OD-H, 5:95 ^{i}PrOH/hexane, 1.0 mL/min, 254 nm), R_t (major) = 6.8 min, R_t (minor) = 8.1 min.

Methyl (*S*)-2-((*R*)-(4-chlorophenyl)(propionyloxy)methyl)-2-(naphthalen-2-ylmethyl) pent-4-enoate (**6e**)

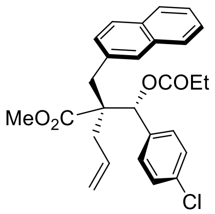

6e

Colorless semisolid, 45.2 mg, 50% yield. ^{1}H NMR (400 MHz, $CDCl_3$) δ 7.82–7.76 (m, 1H), 7.75–7.70 (m, 2H), 7.56 (s, 1H), 7.48–7.40 (m, 2H), 7.30 (d, *J* = 8.6 Hz, 2H), 7.27–7.18 (m, 3H), 6.19–6.05 (m, 2H), 5.26–5.09 (m, 2H), 3.61 (s, 3H), 3.21 (d, *J* = 13.7 Hz, 1H), 3.09 (d, *J* = 13.7 Hz, 1H), 2.57–2.29 (m, 4H), 1.14 (t, *J* = 7.6 Hz, 3H). ^{13}C NMR (101 MHz, $CDCl_3$) δ 173.1, 172.8, 136.1, 134.2, 134.1, 133.4, 132.4, 129.1, 129.1, 128.3, 128.3, 127.8, 127.7, 127.6, 126.1, 125.8, 118.9, 78.0, 55.7, 51.7, 39.4, 34.9, 27.9, 9.1. HRMS (ESI, m/z): calcd. for $C_{27}H_{27}ClO_4Na^+$ 473.1496, found 473.1493. IR (KBr thin film, cm^{-1}): ν 3473, 3413, 2979, 2920, 2851, 1741, 1640, 1462, 1176, 1081, 1018, 745, 611, 477. $[\alpha]_D^{20}$: +10.0 (c 0.4, $CHCl_3$); HPLC analysis:

92% *ee* (Chiralcel AD-H, 2:98 ^{i}PrOH/hexane, 1.0 mL/min, 254 nm), R_t (major) = 10.3 min, R_t (minor) = 8.9 min.

Ethyl (*R*)-2-allyl-2-((*S*)-hydroxy(4-methoxyphenyl)methyl)-4-methylpent-4-enoate **(5f)**

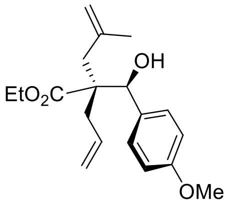

5f

Colorless semisolid, 31.6 mg, 50% yield. ^{1}H NMR (400 MHz, $CDCl_3$) δ 7.22 (d, *J* = 8.7 Hz, 2H), 6.84 (d, *J* = 8.7 Hz, 2H), 6.07–5.93 (m, 1H), 5.14–5.02 (m, 2H), 4.95 (d, *J* = 6.4 Hz, 1H), 4.89 (s, 1H), 4.76 (s, 1H), 4.16 (q, *J* = 7.2 Hz, 2H), 3.84 (d, *J* = 6.4 Hz, 1H), 3.80 (s, 3H), 2.52–2.40 (m, 3H), 2.19 (dd, *J* = 15.0, 7.9 Hz, 1H), 1.68 (s, 3H), 1.24 (t, *J* = 7.2 Hz, 3H). ^{13}C NMR (101 MHz, $CDCl_3$) δ 176.7, 159.2, 141.8, 134.6, 132.2, 128.7, 118.2, 114.3, 113.3, 70.8, 61.1, 55.3, 53.6, 41.9, 36.1, 24.1, 14.1. HRMS (ESI, m/z): calcd. for $C_{19}H_{26}O_4Na^+$ 341.1729, found 341.1730. IR (KBr thin film, cm^{-1}): ν 3735, 2976, 2928, 2378, 2348, 2316, 1726, 1512, 1363, 1277, 1176, 1033, 762, 748. $[\alpha]_D^{20}$: +25.8 (c 0.4, $CHCl_3$); HPLC analysis: 89% *ee* (Chiralcel AD-H, 10:90 ^{i}PrOH/hexane, 1.0 mL/min, 220 nm), R_t (major) = 4.7 min, R_t (minor) = 3.7 min.

Ethyl (*S*)-2-allyl-2-((*R*)-(4-methoxyphenyl)(propionyloxy)methyl)-4-methylpent-4-enoate **(6f)**

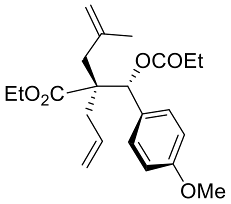

6f

Colorless semisolid, 35.4 mg, 47% yield. ^{1}H NMR (600 MHz, $CDCl_3$) δ 7.24–7.20 (m, 2H), 6.83–6.78 (m, 2H), 6.04–5.94 (m, 2H), 5.11–5.02 (m, 2H), 4.85–4.83 (m, 1H), 4.68 (d, *J* = 0.6 Hz, 1H), 4.14 (dq, *J* = 10.8, 7.2 Hz, 1H), 4.07 (dq, *J* = 10.8, 7.2 Hz, 1H), 3.78 (s, 3H), 2.60 (d, *J* = 14.1 Hz, 1H), 2.49 (dd, *J* = 15.4, 6.7 Hz, 1H), 2.36–2.29 (m, 1H), 2.29–2.22 (m, 1H), 2.17 (d, *J* = 14.1 Hz, 1H), 1.64 (s, 3H), 1.22 (t, *J* = 7.2 Hz, 3H), 1.11 (t, *J* = 7.6 Hz, 3H). ^{13}C NMR (151 MHz, $CDCl_3$) δ 173.6, 172.9, 159.5, 141.2, 135.3, 129.2, 129.1, 117.3, 115.6, 113.4, 78.6, 60.8, 55.3, 53.1, 42.0, 35.9, 28.0, 23.9, 14.1, 9.2. HRMS (ESI, m/z): calcd. for $C_{22}H_{30}O_5Na^+$ 397.1991, found 397.1992. IR (KBr thin film, cm^{-1}): ν 3455, 2982, 2922, 2381, 2348, 2307, 1741, 1637, 1512, 1363, 1274, 1262, 1179, 765, 748. $[\alpha]_D^{20}$: +57.9 (c 0.2, $CHCl_3$); HPLC analysis: 95% *ee* (Chiralcel AD-H, 2:98 ^{i}PrOH/hexane, 1.0 mL/min, 220 nm), R_t (major) = 6.0 min, R_t (minor) = 5.2 min.

Ethyl (*R*)-2-((*S*)-hydroxy(phenyl)methyl)-2-methylpent-4-ynoate **(5g)**

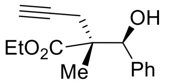

5g

Colorless semisolid, 22.3 mg, 45% yield. ^{1}H NMR (400 MHz, $CDCl_3$) δ 7.40–7.28 (m, 5H), 5.10 (d, J = 4.7 Hz, 1H), 4.32–4.14 (m, 2H), 3.47 (d, J = 4.7 Hz, 1H), 2.37 (qd, J = 16.7, 2.6 Hz, 2H), 2.09 (t, J = 2.6 Hz, 1H), 1.28 (t, J = 7.1 Hz, 3H), 1.20 (s, 3H). ^{13}C NMR (101 MHz, $CDCl_3$) δ 176.0, 139.2, 128.0, 128.0, 127.5, 80.3, 76.5, 71.5, 61.4, 50.9, 26.6, 17.1, 14.1. HRMS (ESI, m/z): calcd. for $C_{15}H_{18}O_3Na^+$ 269.1154, found 269.1152. IR (KBr thin film, cm^{-1}): ν 3449, 2991, 2381, 2351, 2316, 1637, 1369, 1274, 1262, 1099, 765, 748. $[\alpha]_D^{20}$: +92.6 (c 0.1, $CHCl_3$); HPLC analysis: 99% *ee* (Chiralcel AD-H, 5:95 ^{i}PrOH/hexane, 1.0 mL/min, 220 nm), R_t (major) = 7.1 min, R_t (minor) = 8.3 min.

Ethyl (*S*)-2-methyl-2-((*R*)-phenyl(propionyloxy)methyl)pent-4-ynoate (**6g**)

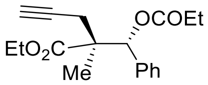

6g

Colorless semisolid, 31.2 mg, 52% yield. ^{1}H NMR (600 MHz, $CDCl_3$) δ 7.34–7.25 (m, 5H), 6.07 (s, 1H), 4.22–4.11 (m, 2H), 2.52 (dd, J = 16.5, 2.6 Hz, 1H), 2.40–2.28 (m, 2H), 2.17 (dd, J = 16.5, 2.7 Hz, 1H), 2.01 (t, J = 2.7 Hz, 1H), 1.28 (s, 3H), 1.23 (t, J = 7.1 Hz, 3H), 1.11 (t, J = 7.6 Hz, 3H). ^{13}C NMR (151 MHz, $CDCl_3$) δ 173.2, 172.7, 136.4, 128.5, 128.2, 127.6, 79.7, 78.0, 71.4, 61.2, 50.5, 27.8, 26.2, 17.0, 14.2, 9.2. HRMS (ESI, m/z): calcd. for $C_{18}H_{22}O_4Na^+$ 325.1416, found 325.1418. IR (KBr thin film, cm^{-1}): ν 3464, 3416, 2920, 2851, 2378, 2348, 2307, 1750, 1640, 1619, 1363, 1271, 1105, 1018, 768, 745. $[\alpha]_D^{20}$: +22.0 (c 0.2, $CHCl_3$); HPLC analysis: 88% *ee* (Chiralcel AD-H, 1:99 ^{i}PrOH/hexane, 1.0 mL/min, 220 nm), R_t (major) = 8.7 min, R_t (minor) = 10.5 min.

1-Ethyl 6-methyl (*R*)-2-((*S*)-hydroxy(phenyl)methyl)-2-methylhexanedioate (**5h**)

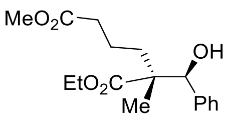

5h

Colorless semisolid, 29.2 mg, 47% yield. ^{1}H NMR (600 MHz, $CDCl_3$) δ 7.31–7.23 (m, 5H), 4.90 (d, J = 2.4 Hz, 1H), 4.12 (qd, J = 7.1, 1.8 Hz, 2H), 3.64 (s, 3H), 3.11 (d, J = 3.1 Hz, 1H), 2.34–2.20 (m, 2H), 1.92–1.81 (m, 1H), 1.65–1.53 (m, 2H), 1.50–1.42 (m, 1H), 1.22 (t, J = 7.1 Hz, 3H), 1.06 (s, 3H). ^{13}C NMR (151 MHz, $CDCl_3$) δ 176.3, 174.0, 140.2, 127.9, 127.9, 127.6, 78.5, 60.9, 51.7, 51.4, 34.5, 34.3, 20.5, 17.6, 14.2. HRMS (ESI, m/z): calcd. for $C_{17}H_{24}O_5Na^+$ 331.1521, found 331.1522. IR (KBr thin film, cm^{-1}): ν 3422, 2920, 2378, 2348, 2313, 1718, 1637, 1509, 1360, 1277, 1259, 1117, 831, 765, 748. $[\alpha]_D^{20}$: +36.6 (c 0.1, $CHCl_3$); HPLC analysis: 99% *ee* (Chiralcel AD-H, 5:95 ^{i}PrOH/hexane, 1.0 mL/min, 220 nm), R_t (major) = 10.2 min, R_t (minor) = 12.1 min.

1-Ethyl 6-methyl (*S*)-2-methyl-2-((*R*)-phenyl(propionyloxy)methyl)hexanedioate (**6h**)

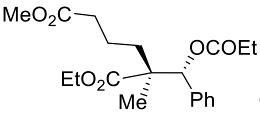

6h

Colorless semisolid, 37.1 mg, 51% yield. ^{1}H NMR (600 MHz, $CDCl_3$) δ 7.29–7.20 (m, 5H), 6.04 (s, 1H), 4.13–4.03 (m, 2H), 3.64 (s, 3H), 2.43–2.33 (m, 2H), 2.33–2.22 (m, 2H), 1.88–1.80 (m, 1H), 1.68–1.60 (m, 1H), 1.55–1.44 (m, 2H), 1.21 (t, J = 7.1 Hz, 3H), 1.15–1.11 (m, 6H). ^{13}C NMR (151 MHz, $CDCl_3$) δ 173.9, 173.7, 173.1, 137.3, 128.1, 128.0, 127.5, 79.0, 60.9, 51.6, 51.3, 35.5, 34.5, 27.9, 20.4, 16.7, 14.2, 9.2. HRMS (ESI, m/z): calcd. for $C_{20}H_{28}O_6Na^+$ 387.1784, found 387.1785. IR (KBr thin film, cm^{-1}): ν 3544, 3479, 3413, 3238, 2917, 2851, 2381, 1738, 1640, 1616, 1366, 1268, 1170, 751, 626. $[\alpha]_D^{20}$: +63.6 (c 0.2, $CHCl_3$); HPLC analysis: 92% *ee* (Chiralcel OD-H, 3:97 ^{i}PrOH/hexane, 1.0 mL/min, 220 nm), R_t (major) = 9.5 min, R_t (minor) = 6.0 min.

Ethyl (*R*)-2-((*S*)-hydroxy(phenyl)methyl)-2-methyl-3-methylenenonanoate (**5i**)

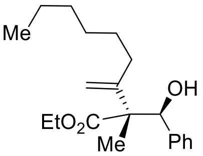

5i

Colorless semisolid, 33.1 mg, 52% yield. ^{1}H NMR (400 MHz, $CDCl_3$) δ 7.37–7.31 (m, 2H), 7.30–7.23 (m, 3H), 5.20 (s, 1H), 5.11 (s, 1H), 4.96 (s, 1H), 4.13 (q, J = 7.1 Hz, 2H), 3.22 (d, J = 1.8 Hz, 1H), 2.23–2.10 (m, 1H), 1.95–1.82 (m, 1H), 1.54–1.38 (m, 2H), 1.35–1.19 (m, 12H), 0.89 (t, J = 6.7 Hz, 3H). ^{13}C NMR (101 MHz, $CDCl_3$) δ 175.7, 147.7, 139.5, 128.3, 127.6, 127.4, 113.0, 77.0, 61.0, 57.2, 33.3, 31.9, 29.3, 28.4, 22.7, 18.2, 14.1, 14.0. HRMS (ESI, m/z): calcd. for $C_{20}H_{30}O_3Na^+$ 341.2093, found 341.2090. IR (KBr thin film, cm^{-1}): ν 3446, 2928, 2381, 2348, 2307, 1643, 1506, 1363, 1274, 1262, 765, 748. $[\alpha]_D^{20}$: +67.9 (c 0.1, $CHCl_3$); HPLC analysis: 84% *ee* (Chiralcel AD-H, 1:99 ^{i}PrOH/hexane, 1.0 mL/min, 220 nm), R_t (major) = 7.4 min, R_t (minor) = 9.5 min.

Ethyl (*S*)-2-methyl-3-methylene-2-((*R*)-phenyl(propionyloxy)methyl)nonanoate (**6i**)

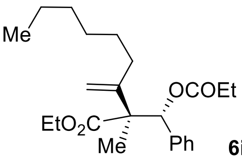

6i

Colorless semisolid, 33.7 mg, 45% yield. ^{1}H NMR (600 MHz, $CDCl_3$) δ 7.36–7.30 (m, 2H), 7.29–7.20 (m, 3H), 6.42 (s, 1H), 5.06 (t, J = 1.4 Hz, 1H), 5.03 (s, 1H), 4.15–3.98 (m, 2H), 2.36–2.22 (m, 2H), 2.14–2.05 (m, 2H), 1.49–1.42 (m, 2H), 1.33–1.26 (m, 9H), 1.20 (t, J = 7.2 Hz, 3H), 1.10 (t, J = 7.6 Hz, 3H), 0.88 (t, J = 6.9 Hz, 3H).^{13}C NMR (151 MHz, $CDCl_3$) δ 173.3, 172.8, 148.0, 137.7, 128.4, 128.0, 127.7, 112.5, 76.7, 61.0, 56.3, 32.9, 31.9, 29.4, 28.7, 27.9, 22.7, 17.7, 14.2, 14.1, 9.2. HRMS (ESI, m/z): calcd. for $C_{23}H_{34}O_4Na^+$ 397.2355, found 397.2353. IR (KBr thin film, cm^{-1}): ν 3425, 3003, 2378, 2310, 1634, 1512, 1363, 1274, 1259, 1108, 834, 765, 748. $[\alpha]_D^{20}$: –38.0 (c 0.1, $CHCl_3$); HPLC analysis: 98% *ee* (Chiralcel AD-H, 1:99 ^{i}PrOH/hexane,

1.0 mL/min, 220 nm), R_t (major) = 5.0 min, R_t (minor) = 3.7 min.

Ethyl (*R*)-2-benzyl-2-((*S*)-hydroxy(naphthalen-2-yl)methyl)pent-4-enoate (**5j**)

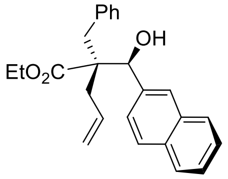

5j

Colorless semisolid, 37.4 mg, 50% yield. ^{1}H NMR (600 MHz, $CDCl_3$) δ 7.91 (s, 1H), 7.88–7.80 (m, 3H), 7.60 (dd, *J* = 8.5, 1.7 Hz, 1H), 7.55–7.41 (m, 2H), 7.27–7.17 (m, 3H), 7.12–7.10 (m, 2H), 6.01 (ddt, *J* = 17.1, 10.3, 6.9 Hz, 1H), 5.29 (d, *J* = 3.2 Hz, 1H), 5.23 (dd, *J* = 10.3, 1.7 Hz, 1H), 5.18 (ddd, *J* = 17.0, 3.3, 1.5 Hz, 1H), 4.14 (d, *J* = 3.4 Hz, 1H), 4.06 (dq, *J* = 10.8, 7.2 Hz, 1H), 3.94 (dq, *J* = 10.8, 7.2 Hz, 1H), 3.46 (d, *J* = 13.7 Hz, 1H), 2.77 (d, *J* = 13.7 Hz, 1H), 2.73–2.62 (m, 1H), 2.21 (dd, *J* = 15.1, 7.2 Hz, 1H), 1.03 (t, *J* = 7.2 Hz, 3H). ^{13}C NMR (151 MHz, $CDCl_3$) δ 175.9, 137.5, 137.4, 134.8, 133.2, 133.0, 130.5, 128.2, 128.1, 127.7, 127.4, 127.4, 126.6, 126.2, 126.1, 126.1, 118.8, 76.6, 61.0, 55.8, 38.6, 36.3, 18.8. HRMS (ESI, m/z): calcd. for $C_{25}H_{26}O_3Na^+$ 397.1774, found 397.1777. IR (KBr thin film, cm^{-1}): ν 3446, 2985, 2378, 2310, 1715, 1634, 1503, 1369, 1271, 1256, 765, 751. $[\alpha]_D^{20}$: –6.9 (c 0.5, $CHCl_3$); HPLC analysis: 87% *ee* (Chiralcel AD-H, 10:90 ^{i}PrOH/hexane, 1.0 mL/min, 220 nm), R_t (major) = 6.4 min, R_t (minor) = 7.4 min.

Ethyl (*S*)-2-benzyl-2-((*R*)-naphthalen-2-yl(propionyloxy)methyl)pent-4-enoate (**6j**)

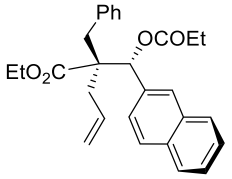

6j

Colorless semisolid, 39.6 mg, 46% yield. ^{1}H NMR (600 MHz, $CDCl_3$) δ 7.85–7.77 (m, 4H), 7.52–7.42 (m, 3H), 7.28–7.22 (m, 2H), 7.22–7.18 (m, 1H), 7.17–7.13 (m, 2H), 6.34 (s, 1H), 6.20–6.07 (m, 1H), 5.26–5.12 (m, 2H), 4.07 (qd, *J* = 7.2, 3.3 Hz, 2H), 3.15 (d, *J* = 13.8 Hz, 1H), 3.03 (d, *J* = 13.8 Hz, 1H), 2.58 (dd, *J* = 15.3, 7.3 Hz, 1H), 2.50–2.36 (m, 3H), 1.21–1.13 (m, 6H). ^{13}C NMR (151 MHz, $CDCl_3$) δ 172.9, 137.0, 135.0, 134.4, 133.2, 132.9, 130.4, 128.2, 128.2, 127.7, 127.7, 127.4, 126.8, 126.3, 126.3, 125.5, 118.6, 78.9, 61.0, 55.5, 39.3, 35.3, 28.0, 14.1, 9.2. HRMS (ESI, m/z): calcd. for $C_{28}H_{30}O_4Na^+$ 453.2042, found 453.2045. IR (KBr thin film, cm^{-1}): ν 3446, 2982, 2920, 2851, 2378, 2348, 2313, 1741, 1637, 1363, 1274, 1259, 762, 748. $[\alpha]_D^{20}$: –7.8 (c 0.4, $CHCl_3$); HPLC analysis: 98% *ee* (Chiralcel AD-H, 3:97 ^{i}PrOH/hexane, 1.0 mL/min, 220 nm), R_t (major) = 10.3 min, R_t (minor) = 6.8 min.

Ethyl (*R*)-2-((*S*)-hydroxy(naphthalen-2-yl)methyl)-2-methylpent-4-enoate (**5k**)

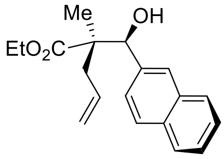

5k

Colorless semisolid, 28.8 mg, 48% yield. ^1H NMR (600 MHz, $CDCl_3$) δ 7.85–7.73 (m, 4H), 7.53–7.40 (m, 3H), 5.87–5.71 (m, 1H), 5.12–4.97 (m, 3H), 4.12 (q, J = 7.1 Hz, 2H), 3.31 (d, J = 3.5 Hz, 1H), 2.73 (dd, J = 13.8, 6.9 Hz, 1H), 2.22 (dd, J = 13.8, 7.9 Hz, 1H), 1.21 (t, J = 7.2 Hz, 3H), 1.11 (s, 3H). ^{13}C NMR (151 MHz, $CDCl_3$) δ 176.1, 137.8, 134.4, 133.1, 133.0, 128.2, 127.7, 127.5, 126.8, 126.1, 126.0, 125.7, 118.3, 78.6, 61.0, 51.8, 39.4, 17.9, 14.3. HRMS (ESI, m/z): calcd. for $C_{19}H_{22}O_3Na^+$ 312.1467, found 312.1465. IR (KBr thin film, cm^{-1}): ν 3446, 3003, 2381, 2313, 1709, 1637, 1512, 1360, 1271, 1262, 768, 745. $[\alpha]_D^{20}$: +23.6 (c 0.2, $CHCl_3$); HPLC analysis: 99% *ee* (Chiralcel OJ-H, 10:90 ^{i}PrOH/hexane, 1.0 mL/min, 254 nm), R_t (major) = 6.0 min, R_t (minor) = 13.1 min.

Ethyl (*S*)-2-methyl-2-((*R*)-naphthalen-2-yl(propionyloxy)methyl)pent-4-enoate (**6k**)

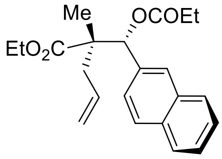

6k

Colorless semisolid, 34.9 mg, 49% yield. ^1H NMR (600 MHz, $CDCl_3$) δ 7.85–7.76 (m, 3H), 7.74 (s, 1H), 7.50–7.43 (m, 2H), 7.41 (dd, J = 8.5, 1.7 Hz, 1H), 6.29 (s, 1H), 5.78 (ddt, J = 16.1, 10.9, 7.4 Hz, 1H), 5.12–5.01 (m, 2H), 4.17–4.02 (m, 2H), 2.73 (dd, J = 13.7, 7.0 Hz, 1H), 2.50–2.38 (m, 2H), 2.28 (dd, J = 13.7, 7.7 Hz, 1H), 1.23 (t, J = 7.2 Hz, 3H), 1.21 (s, 3H), 1.18 (t, J = 7.6 Hz, 3H). ^{13}C NMR (151 MHz, $CDCl_3$) δ 173.7, 173.1, 134.9, 133.8, 133.2, 132.9, 128.2, 127.7, 127.7, 126.9, 126.3, 125.3, 118.6, 78.9, 60.9, 51.6, 40.8, 27.9, 16.9, 14.3, 9.3. HRMS (ESI, m/z): calcd. for $C_{22}H_{26}O_4Na^+$ 377.1729, found 377.1733. IR (KBr thin film, cm^{-1}): ν 3449, 2985, 2920, 2842, 2381, 2310, 1744, 1637, 1506, 1360, 1274, 1259, 768, 751. $[\alpha]_D^{20}$: +34.4 (c 0.3, $CHCl_3$); HPLC analysis: 92% *ee* (Chiralcel AD-H, 1:99 ^{i}PrOH/hexane, 1.0 mL/min, 254 nm), R_t (major) = 11.2 min, R_t (minor) = 8.4 min.

Ethyl (*S*)-2-((*S*)-hydroxy(naphthalen-2-yl)methyl)-2-methylpent-4-enoate (**5l**)

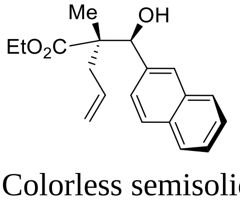

5l

Colorless semisolid, 29.8 mg, 50% yield. ^1H NMR (600 MHz, $CDCl_3$) δ 7.85–7.75 (m, 3H), 7.72 (d, J = 0.4 Hz, 1H), 7.51–7.44 (m, 2H), 7.42 (dd, J = 8.5, 1.7 Hz, 1H), 5.71 (ddt, J = 17.4, 10.2, 7.4 Hz, 1H), 5.12–4.98 (m, 3H), 4.25–4.15 (m, 2H), 3.40 (d, J = 5.3 Hz, 1H), 2.55 (dd, J = 13.7, 7.2 Hz, 1H), 2.06 (dd, J = 13.7, 7.5 Hz, 1H), 1.24 (t, J = 7.1 Hz, 3H), 1.10 (s, 3H). ^{13}C NMR (151 MHz, $CDCl_3$) δ 176.5, 137.8, 133.3, 133.1, 132.9, 128.1, 127.7, 127.5, 126.9, 126.2, 126.1, 125.7, 118.8, 78.5, 61.1, 51.9, 41.6, 16.5, 14.3. HRMS (ESI, m/z): calcd. for $C_{19}H_{22}O_3Na^+$ 312.1467, found 312.1466. IR

(KBr thin film, cm^{-1}): ν 3440, 3006, 2985, 2928, 2381, 2313, 1723, 1593, 1363, 1274, 1256, 765, 748. $[\alpha]_D^{20}$: –45.3 (c 0.1, $CHCl_3$); HPLC analysis: 92% *ee* (Chiralcel AD-H, 10:90 ^{i}PrOH/hexane, 1.0 mL/min, 254 nm), R_t (major) = 6.4 min, R_t (minor) = 7.4 min.

Ethyl (*R*)-2-methyl-2-((*R*)-naphthalen-2-yl(propionyloxy)methyl)pent-4-enoate (**6l**)

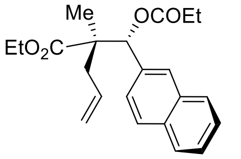

6l

Colorless semisolid, 33.3 mg, 47% yield. ^{1}H NMR (600 MHz, $CDCl_3$) δ 7.87–7.79 (m, 3H), 7.77 (d, *J* = 0.7 Hz, 1H), 7.51–7.45 (m, 2H), 7.45–7.41 (m, 1H), 6.24 (s, 1H), 5.67–5.58 (m, 1H), 5.06–4.97 (m, 2H), 4.24–4.10 (m, 2H), 2.61 (dd, *J* = 13.5, 7.0 Hz, 1H), 2.41–2.33 (m, 2H), 1.89 (dd, *J* = 13.4, 7.7 Hz, 1H), 1.27 (t, *J* = 7.1 Hz, 3H), 1.22 (s, 3H), 1.13 (t, *J* = 7.6 Hz, 3H). ^{13}C NMR (151 MHz, $CDCl_3$) δ 174.2, 172.9, 134.3, 133.2, 132.8, 132.8, 128.2, 127.8, 127.7, 127.3, 126.4, 125.5, 118.9, 79.4, 60.9, 51.2, 41.0, 27.9, 16.1, 14.4, 9.2. HRMS (ESI, m/z): calcd. for $C_{22}H_{26}O_4Na^+$ 377.1729, found 377.1740. IR (KBr thin film, cm^{-1}): ν 3440, 3006, 2988, 2920, 2854, 2381, 1744, 1655, 1506.78, 1360, 1277, 1262, 762, 748. $[\alpha]_D^{20}$: +18.0 (c 0.2, $CHCl_3$); HPLC analysis: 95% *ee* (Chiralcel AD-H, 1:99 ^{i}PrOH/hexane, 1.0 mL/min, 254 nm), R_t (major) = 10.7 min, R_t (minor) =12.0 min.

(*S*)-Methyl 2-benzyl-2-((*S*)-hydroxy(naphthalen-2-yl)methyl)pent-4-enoate (**5m**)

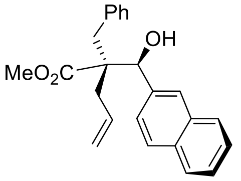

5m

Colorless oil, 35 mg, 49% yield. ^{1}H NMR (600 MHz, $CDCl_3$) δ 7.88–7.79 (m, 4H), 7.56–7.52 (m, 1H), 7.50–7.46 (m, 2H), 7.25–7.15 (m, 3H), 7.09–7.04 (m, 2H), 5.99 (ddt, J = 17.1, 10.2, 7.0 Hz, 1H), 5.26 (d, *J* = 3.5 Hz, 1H), 5.21 (dd, *J* = 10.2, 1.9 Hz, 1H), 5.16 (dd, *J* = 17.1, 1.9 Hz, 1H), 3.88 (d, *J* = 3.6 Hz, 1H), 3.52 (s, 3H), 3.40 (d, *J* = 13.7 Hz, 1H), 2.78 (d, *J* = 13.7 Hz, 1H), 2.65 (dd, *J* = 15.2, 6.6 Hz, 1H), 2.19 (dd, *J* = 15.2, 7.2 Hz, 1H); ^{13}C NMR (150 MHz, $CDCl_3$) δ 176.2, 137.4, 137.3, 134.8, 133.2, 132.9, 130.4, 128.2, 128.1, 127.7, 127.5, 127.3, 126.7, 126.2, 125.9, 118.9, 56.2, 51.6, 38.6, 36.0; HRMS (ESI, m/z): calcd. for $C_{24}H_{24}O_3Na^+$ 383.1618, found 383.1620; IR (KBr thin film, cm^{-1}): ν 3063, 3030, 1736, 1685, 1453, 1263, 1180, 915, 748, 698. $[\alpha]_D^{25}$: -8.7 (c 1.2, $CHCl_3$); HPLC analysis: 91% *ee* (Chiralcel AD-H, 10:90 ^{i}PrOH/Hexane, 1.0 mL/min, 220 nm), R_t (major) = 7.1 min, R_t (minor) = 8.6 min.

(*R*)-Methyl 2-benzyl-2-((*R*)-naphthalen-2-yl(propionyloxy)methyl)pent-4-enoate (**6m**)

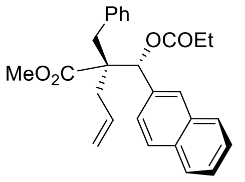

6m

Colorless oil, 35 mg, 42% yield. ^{1}H NMR (600 MHz, $CDCl_3$) δ 7.84–7.78 (m, 3H), 7.77–7.75 (m, 1H), 7.50–7.45 (m, 2H), 7.43–7.40 (m, 1H), 7.26–7.18 (m, 3H), 7.14–7.10 (m, 2H), 6.33 (s, 1H), 6.14 (ddt, *J* = 17.0, 10.3, 7.2 Hz, 1H), 5.22 (dd, *J* = 10.2, 2.0 Hz, 1H), 5.16 (dd, *J* = 17.0, 2.0 Hz, 1H), 3.62 (s, 3H), 3.14 (d, *J* = 13.8 Hz, 1H), 3.02 (d, *J* = 13.8 Hz, 1H), 2.56 (dd, *J* = 15.4, 7.2 Hz, 1H), 2.47–2 37 (m, 3H), 1.17 (t, *J* = 7.6 Hz, 3H); ^{13}C NMR (150 MHz, $CDCl_3$) δ 173.3, 172.9, 136.9, 135.0, 134.3, 133.2, 132.9, 130.3, 128.3, 128.2, 127.7, 127.2, 126.8, 126.3, 126.2, 125.3, 118.6, 78.8, 55.8, 51.6, 39.2, 34.9, 28.0, 9.2; HRMS (ESI, m/z): calcd. for $C_{27}H_{28}O_4Na^+$ 439.1880, found 439.1884; IR (KBr thin film, cm^{-1}): ν 3066, 3033, 2982, 1739, 1456, 1435, 1171, 1081, 1022, 811, 745, 698. $[\alpha]_D^{25}$: +63.0 (c 3.2, $CHCl_3$); HPLC analysis: 97% *ee* (Chiralcel AD-H, 2:98 ^{i}PrOH/Hexane, 1.0 mL/min, 220 nm), R_t (major) = 13.7 min, R_t (minor) = 9.0 min.

<u>Ethyl (*R*)-2-((*R*)-furan-2-yl(hydroxy)methyl)-2-methylpent-4-enoate (**5n**)</u>

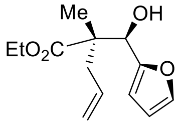

5n

Colorless semisolid, 22.8 mg, 48% yield. ^{1}H NMR (600 MHz, $CDCl_3$) δ 7.33 (dd, *J* = 1.8, 0.8 Hz, 1H), 6.31 (dd, *J* = 3.2, 1.8 Hz, 1H), 6.23 (dd, *J* = 2.6, 0.6 Hz, 1H), 5.84–5.70 (m, 1H), 5.10–5.01 (m, 2H), 4.82 (d, *J* = 6.8 Hz, 1H), 4.16 (q, *J* = 7.1 Hz, 2H), 3.22 (d, *J* = 6.9 Hz, 1H), 2.58 (dd, *J* = 13.9, 7.0 Hz, 1H), 2.25 (ddt, *J* = 13.9, 7.9, 1.0 Hz, 1H), 1.24 (t, *J* = 7.1 Hz, 3H), 1.18 (s, 3H). ^{13}C NMR (151 MHz, $CDCl_3$) δ 175.8, 153.8, 142.0, 133.9, 118.5, 110.3, 107.8, 72.9, 61.0, 50.7, 39.6, 18.4, 14.2. HRMS (ESI, m/z): calcd. for $C_{13}H_{18}O_4Na^+$ 261.1103, found 261.1103. IR (KBr thin film, cm^{-1}): ν 3449, 3009, 2920, 2381, 2351, 2310, 1741, 1509, 1360, 1274, 1259, 762, 748. $[\alpha]_D^{20}$: –81.2 (c 0.1, $CHCl_3$); HPLC analysis: 86% *ee* (Chiralcel AD-H, 5:95 ^{i}PrOH/hexane, 1.0 mL/min, 220 nm), R_t (major) = 5.5 min, R_t (minor) = 7.6 min.

<u>Ethyl (*S*)-2-((*S*)-furan-2-yl(propionyloxy)methyl)-2-methylpent-4-enoate (**6n**)</u>

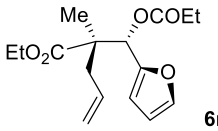

6n

Colorless semisolid, 28.9 mg, 49% yield. ^{1}H NMR (400 MHz, $CDCl_3$) δ 7.26 (d, *J* = 0.9 Hz, 1H), 6.23 (dd, *J* = 3.2, 1.8 Hz, 1H), 6.17 (d, *J* = 3.2 Hz, 1H), 6.10 (s, 1H), 5.73–5.58 (m, 1H), 5.03–4.92 (m, 2H), 4.11–3.96 (m, 2H), 2.53 (dd, *J* = 13.7, 7.0 Hz, 1H), 2.32 (q, *J* = 7.4 Hz, 2H), 2.19 (dd, *J* = 13.7, 7.7 Hz, 1H), 1.18–1.12 (m, 6H), 1.09 (t, *J* = 7.6 Hz, 3H). ^{13}C NMR (101 MHz, $CDCl_3$) δ 173.4, 173.1, 150.6, 142.3, 133.3,

118.5, 110.1, 108.9, 72.4, 60.9, 50.6, 40.6, 27.6, 17.0, 14.1, 9.1. HRMS (ESI, m/z): calcd. for $C_{16}H_{22}O_5Na^+$ 317.1365, found 317.1365; IR (KBr thin film, cm^{-1}): ν 3461, 3006, 2375, 2375, 2310, 1720, 1509, 1369, 1277, 1256, 920, 768, 748. $[\alpha]_D^{20}$: +69.7 (c 0.5, $CHCl_3$); HPLC analysis: 83% *ee* (Chiralcel AD-H, 1:99 ^{i}PrOH/hexane, 1.0 mL/min, 220 nm), R_t (major) = 5.6 min, R_t (minor) = 6.5 min.

Ethyl (2*R*,3*S*,*E*)-2-allyl-3-hydroxy-2-methyl-5-phenylpent-4-enoate (**5o**)

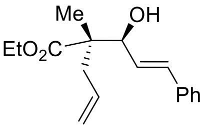

5o

Colorless semisolid, 25.4 mg, 46% yield. ^{1}H NMR (400 MHz, $CDCl_3$) δ 7.37 (d, *J* = 7.2 Hz, 2H), 7.31 (t, *J* = 7.4 Hz, 2H), 7.27–7.21 (m, 1H), 6.63 (d, *J* = 15.9 Hz, 1H), 6.24 (dd, *J* = 15.9, 6.8 Hz, 1H), 5.88–5.73 (m, 1H), 5.15–5.02 (m, 2H), 4.40 (t, *J* = 5.8 Hz, 1H), 4.24–4.12 (m, 2H), 2.79 (d, *J* = 5.3 Hz, 1H), 2.61 (dd, *J* = 13.8, 7.0 Hz, 1H), 2.29 (dd, *J* = 13.8, 7.8 Hz, 1H), 1.25 (t, *J* = 7.1 Hz, 3H), 1.22 (s, 3H). ^{13}C NMR (101 MHz, $CDCl_3$) δ 175.9, 136.7, 134.1, 132.7, 128.7, 127.9, 127.8, 126.7, 118.3, 77.2, 60.9, 51.0, 39.9, 18.1, 14.4. HRMS (ESI, m/z): calcd. for $C_{17}H_{22}O_3Na^+$ 297.1461, found 297.1464. IR (KBr thin film, cm^{-1}): ν 3449, 2982, 2925, 2381, 2348, 2313, 1718, 1640, 1491, 1366, 1274, 1259, 1215, 1021, 968, 762, 748. $[\alpha]_D^{20}$: –21.0 (c 0.4, $CHCl_3$); HPLC analysis: 93% *ee* (Chiralcel AD-H, 5:95 ^{i}PrOH/hexane, 1.0 mL/min, 220 nm), R_t (major) = 7.0 min, R_t (minor) = 7.8 min.

Ethyl (2*S*,3*R*,*E*)-2-allyl-2-methyl-5-phenyl-3-(propionyloxy)pent-4-enoate (**6o**)

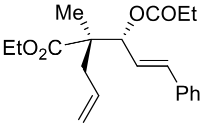

6o

Colorless semisolid, 33.1 mg, 50% yield. ^{1}H NMR (400 MHz, $CDCl_3$) δ 7.28 (d, *J* = 7.3 Hz, 2H), 7.23 (t, *J* = 7.4 Hz, 2H), 7.20–7.13 (m, 1H), 6.54 (d, *J* = 15.9 Hz, 1H), 6.08 (dd, *J* = 15.9, 7.7 Hz, 1H), 5.73–5.56 (m, 2H), 5.05–4.89 (m, 2H), 4.07 (q, *J* = 7.1 Hz, 2H), 2.49 (dd, *J* = 13.7, 7.0 Hz, 1H), 2.30 (q, *J* = 7.6 Hz, 2H), 2.13 (dd, *J* = 13.7, 7.7 Hz, 1H), 1.20–1.12 (m, 6H), 1.08 (t, *J* = 7.6 Hz, 3H). ^{13}C NMR (100 MHz, $CDCl_3$) δ 173.8, 173.3, 136.2, 134.7, 133.4, 128.6, 128.2, 126.8, 123.9, 118.6, 77.9, 60.9, 50.6, 40.8, 27.9, 17.4, 14.4, 9.3. HRMS (ESI, m/z): calcd. for $C_{20}H_{26}O_4Na^+$ 353.1729, found 353.1728; IR (KBr thin film, cm^{-1}): ν 3437, 2920, 2378, 2310, 1735, 1634, 1363, 1274, 1259, 1167, 762, 748. $[\alpha]_D^{20}$: +48.6 (c 0.2, $CHCl_3$); HPLC analysis: 87% ee (Chiralcel AS-H, 0.5:99.5 *i*PrOH/hexane, 1.0 mL/min, 220 nm), R_t (major) = 3.1 min, R_t (minor) = 3.8 min.

(1*R*,2*S*)-2-methyl-1-phenyl-2-vinylpent-4-en-1-ol (**7a**)

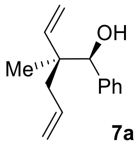

7a

Colorless oil, 20 mg, 49% yield. ^{1}H NMR (600 MHz, $CDCl_3$) δ 7.33–7.26 (m, 5H), 5.83 (dd, *J* = 17.6, 10.9 Hz, 1H), 5.78–5.70 (m, 1 H), 5.18 (dd, *J* = 10.9, 1.3Hz, 1H), 5.04–4.97 (m, 3H), 4.50 (d, *J* = 4.3 Hz, 1H), 2.25 (dd, *J* = 13.8, 6.4 Hz, 1H), 2.07 (dd, *J* = 13.8, 8.1 Hz, 1H), 2.05 (d, *J* = 4.3 Hz, 1H), 1.04 (s, 3H); ^{13}C NMR (150 MHz, $CDCl_3$) δ 142.5, 141.1, 135.1, 127.9, 127.6, 117.4, 115.3, 80.4, 45.2, 41.3, 19.4; HRMS (ESI, m/z): calcd. for $C_{14}H_{18}ONa^{+}$ 225.1250, found 225.1251; IR (KBr thin film, cm^{-1}): ν 3101, 3041, 3014, 2887, 2823, 1717, 1558, 1541, 1489, 1397, 1261, 1099, 1047, 885, 799, 766. $[\alpha]_D^{25}$: –18.2 (c 2.2, $CHCl_3$); HPLC analysis: 91% ee (Chiralcel OD-H, 10:90 ^{i}PrOH/Hexane, 1.0 mL/min, 220 nm), R_t (major) = 5.4 min, R_t (minor) = 3.7 min.

(1*S*,2*R*)-2-methyl-1-phenyl-2-vinylpent-4-en-1-yl propionate (**8a**)

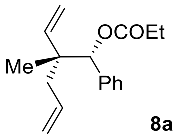

8a

Colorless oil, 23 mg, 45% yield. ^{1}H NMR (600 MHz, $CDCl_3$) δ 7.30–7.20 (m, 5H), 5.81 (dd, *J* = 17.5, 10.9 Hz, 1H), 5.77–5.69 (m, 1H), 5.61 (s, 1H), 5.09 (dd, *J* = 10.9, 1.1 Hz, 1H), 5.06–4.97 (m, 2H), 4.87 (dd, *J* = 17.5, 1.1 Hz, 1H), 2.46–2.32 (m, 2H), 2.25 (dd, *J* = 13.7, 6.4 Hz, 1H), 2.09 (dd, *J* = 13.8, 8.2 Hz, 1H), 1.15 (t, *J* = 7.6 Hz, 3H), 1.02 (s, 3H); ^{13}C NMR (150 MHz, $CDCl_3$) δ 173.4, 141.7, 137.9, 134.4, 128.1, 127.7, 127.6, 117.7, 114.9, 80.8, 44.1, 41.6, 28.0, 19.0, 9.2; HRMS (ESI, m/z): calcd. for $C_{17}H_{22}O_2Na^{+}$ 281.1512, found 281.1516; IR (KBr thin film, cm^{-1}): ν 3031, 2926, 2854, 1741, 1495, 1454, 1374, 1175, 1008, 768, 700. $[\alpha]_D^{25}$: –38.0 (c 1.8, $CHCl_3$); HPLC analysis: 93% ee (Chiralcel AD-H, 1:99 ^{i}PrOH/Hexane, 1.0 mL/min, 220 nm), R_t (major) = 3.5 min, R_t (minor) = 3.0 min.

(1*S*,2*S*)-2-ethynyl-2-methyl-1-phenylpent-4-en-1-ol (**7b**)

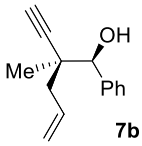

7b

Colorless oil, 19 mg, 47% yield. ^{1}H NMR (600 MHz, $CDCl_3$) δ 7.45–7.40 (m, 2H), 7.37–7.28 (m, 3H), 6.00–5.92 (m, 1H), 5.14–5.04 (m, 2H), 4.60 (d, *J* = 4.0 Hz, 1H), 2.42–2.36 (m, 2H), 2.28 (s, 1H), 1.96 (dd, *J* = 13.5, 8.0 Hz, 1H), 1.21 (s, 3H); ^{13}C NMR (150 MHz, $CDCl_3$) δ 139.8, 134.6, 128.1, 128.0, 127.8, 118.0, 87.7, 79.3, 72.5, 41.5, 41.3, 22.8; HRMS (ESI, m/z): calcd. for $C_{14}H_{16}ONa^{+}$ 223.1093, found 223.1096; IR (KBr thin film, cm^{-1}): ν 3309, 2964, 2927, 2885, 2310, 1743, 1684, 1653, 1509, 1457, 1385, 1260, 1084, 799, 765. $[\alpha]_D^{25}$: –18.2 (c 0.9, $CHCl_3$); HPLC analysis: 92% ee (Chiralcel AD-H, 1:99 ^{i}PrOH/Hexane, 1.0 mL/min, 220 nm), R_t

(major) = 4.2 min, R_t (minor) = 3.5 min.

(1*R*,2*R*)-2-ethynyl-2-methyl-1-phenylpent-4-en-1-yl propionate (**8b**)

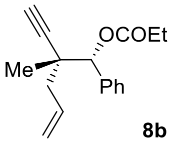

8b

Colorless oil, 23 mg, 45% yield. ^{1}H NMR (400 MHz, $CDCl_3$) δ 7.45–7.38 (m, 2H), 7.35–7.28 (m, 3H), 6.02–5.88 (m, 1H), 5.67 (s, 1H), 5.13 (d, *J* = 9.7 Hz, 1H), 5.06 (d, *J* = 17.0 Hz, 1H), 2.51–2.35 (m, 2H), 2.33 (dd, *J* = 13.5, 6.5 Hz, 1H), 2.21 (s, 1H), 2.10 (dd, *J* = 13.5, 7.8 Hz, 1H), 1.21 (s, 3H), 1.16 (t, *J* = 7.6 Hz, 3H); ^{13}C NMR (150 MHz, $CDCl_3$) δ 173.2, 137.2, 133.9, 128.2, 127.7, 118.4, 86.6, 79.1, 72.4, 42.5, 40.0, 27.9, 22.7, 9.2; HRMS (ESI, m/z): calcd. for $C_{17}H_{20}O_2Na^+$ 279.1356, found 279.1361; IR (KBr thin film, cm^{-1}): ν 2926, 2858, 2352, 1751, 1456, 1373, 1174, 698. $[\alpha]_D^{25}$: –11.4 (c 1.8, $CHCl_3$); HPLC analysis: 97% ee (Chiralcel OJ-H, 0.5:99.5 ^{i}PrOH/Hexane, 0.5 mL/min, 40 ℃, 220 nm), R_t (major) = 10.0 min, R_t (minor) = 6.8 min.

(*S*)-2-benzyl-2-((*S*)-hydroxy(phenyl)methyl)butyl propionate (**7c**)

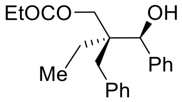

7c

Colorless oil, 29 mg, 44% yield, mp 62–65 ºC. ^{1}H NMR (600 MHz, $CDCl_3$) δ 7.34–7.30 (m, 4H), 7.30–7.27 (m, 1H), 7.26–7.24 (m, 2H), 7.22–7.19 (m, 1H), 7.18–7.15 (m, 2H), 4.74 (d, *J* = 3.7 Hz, 1H), 3.91 (d, *J* = 11.6 Hz, 1H), 3.88 (d, *J* = 11.6 Hz, 1H), 2.97 (d, *J* = 13.4 Hz, 1H), 2.72 (d, *J* = 13.4 Hz, 1H), 2.35 (d, *J* = 3.7 Hz, 1H), 2.16 (q, *J* = 7.6 Hz. 2H), 1.62 (dq, *J* = 15.1, 7.6 Hz, 1H), 1.36 (dq, *J* = 15.1, 7.6 Hz, 1H), 1.05 (t, *J* = 7.6 Hz, 3H), 1.02 (t, *J* = 7.6 Hz, 3H); ^{13}C NMR (150 MHz, $CDCl_3$) δ 174.1, 141.5, 137.9, 130.6, 128.2, 128.0, 127.7, 127.6, 126.3, 76.7, 67.2, 44.6, 37.6, 27.7, 23.7, 9.1, 8.6; HRMS (ESI, m/z): calcd. for $C_{21}H_{26}O_3Na^+$ 349.1774, found 349.1775; IR (KBr thin film, cm^{-1}): ν 3029, 2970, 2940, 2883, 1718, 1559, 1541, 1507, 1457, 1387, 1351, 1197, 1084, 1025, 751, 703. $[\alpha]_D^{25}$: –12.7 (c 1.3, $CHCl_3$); HPLC analysis: 92% ee (Chiralcel AD-H, 10:90 ^{i}PrOH/Hexane, 1.0 mL/min, 220 nm), R_t (major) = 5.1 min, R_t (minor) = 6.3 min.

(1*R*,2*R*)-2-benzyl-2-ethyl-1-phenylpropane-1,3-diyl dipropionate (**8c**)

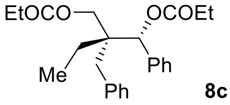

8c

Colorless oil, 37 mg, 48% yield. ^{1}H NMR (600 MHz, $CDCl_3$) δ 7.34–7.27 (m, 5H), 7.25–7.22 (m, 2H), 7.21–7.17 (m, 1H), 7.12–7.07 (m, 2H), 5.89 (s, 1H), 3.81 (s, 2H), 2.91 (d, *J* = 13.6 Hz, 1H), 2.76 (d, *J* = 13.6 Hz, 1H), 2.38 (qd, *J* = 7.6, 4.3 Hz, 2H), 2.17 (qd, *J* = 7.6 , 1.9 Hz, 2H), 1.55–1.45 (m, 1H), 1.36–1.27 (m, 1H), 1.16 (t, *J* = 7.6 Hz, 3H), 1.08–1.02 (m, 6H); ^{13}C NMR (150 MHz, $CDCl_3$) δ 174.0, 173.2, 137.9,

137.3, 130.4, 128.3, 128.1, 127.9, 127.6, 126.5, 77.9, 66.3, 44.2, 37.7, 28.0, 27.6, 23.7, 9.2, 9.1, 8.7; HRMS (ESI, m/z): calcd. for $C_{24}H_{30}O_4Na^+$ 405.2036, found 405.2043; IR (KBr thin film, cm^{-1}): ν 2972, 2941, 1739, 1496, 1457, 1385, 1177, 1082, 1018, 802, 750, 702. $[\alpha]_D^{25}$: –22.5 (c 1.1, $CHCl_3$); HPLC analysis: 87% ee (Chiralcel AD-H, 3.5:96.5 ^{i}PrOH/Hexane, 1.0 mL/min, 220 nm), R_t (major) = 4.0 min, R_t (minor) = 3.6 min.

(*S*)-2-(4-fluorobenzyl)-2-((*S*)-hydroxy(phenyl)methyl)butyl propionate (**7d**)

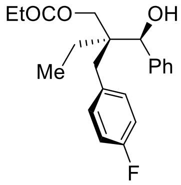

7d

White solid, 29 mg, 42% yield, mp 45–48 ºC. ^{1}H NMR (600 MHz, $CDCl_3$) δ 7.52–7.45 (m, 3H), 7.42–7.36 (m, 2H), 7.02–6.96 (m, 2H), 6.95–6.89 (m, 2H), 4.22 (d, *J* = 11.6 Hz, 1H), 4.16 (d, *J* = 11.6 Hz, 1H), 3.22 (d, *J* = 14.0 Hz, 1H), 3.06 (d, *J* = 14.0 Hz, 1H), 2.32 (q, *J* = 7.6 Hz, 2H), 1.92–1.75 (m, 2H), 1.12 (t, *J* = 7.6 Hz, 3H), 0.93 (t, *J* = 7.5 Hz, 3H); ^{13}C NMR (150 MHz, $CDCl_3$) δ 206.3, 173.9, 161.8 (d, *J* = 244.0 Hz), 139.5, 132.5 (d, *J* = 2.7 Hz), 131.8 (d, *J* = 8.1 Hz), 131.3, 128.5, 127.0, 115.3 (d, *J* = 20.7 Hz), 64.7, 56.1, 38.0, 27.6, 26.3, 9.2, 8.7; HRMS (ESI, m/z): calcd. for $C_{21}H_{25}FO_3Na^+$ 367.1680, found 367.1679; IR (KBr thin film, cm^{-1}): ν 3467, 3037, 2969, 2928, 2853, 1734, 1652, 1509, 1458, 1385, 1222, 841, 703. $[\alpha]_D^{25}$: –8.2 (c 0.5, $CHCl_3$); HPLC analysis: 96% ee (Chiralcel AD-H, 10:90 ^{i}PrOH/Hexane, 1.0 mL/min, 220 nm), R_t (major) = 4.4 min, R_t (minor) = 5.9 min.

(1*R*,2*R*)-2-ethyl-2-(4-fluorobenzyl)-1-phenylpropane-1,3-diyl dipropionate (**8d**)

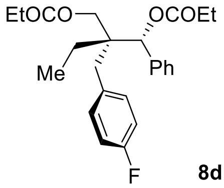

8d

Colorless oil, 39 mg, 49% yield. ^{1}H NMR (600 MHz, $CDCl_3$) δ 7.36–7.24 (m, 5H), 7.08–7.01 (m, 2H), 6.97–6.90 (m, 2H), 5.87 (s, 1H), 3.81 (d, *J* = 11.6 Hz, 1H), 3.78 (d, *J* = 11.6 Hz, 1H), 2.88 (d, *J* = 13.8 Hz, 1H), 2.73 (d, *J* = 13.8 Hz, 1H), 2.38 (qd, *J* = 7.7, 5.0 Hz, 2H), 2.18 (qd, *J* = 7.6, 1.8 Hz, 2H), 1.55–1.45 (m, 1H), 1.35–1.25 (m, 1H), 1.16 (t, *J* = 7.6 Hz, 3H), 1.10–1.00 (m, 6H). ^{13}C NMR (150 MHz, $CDCl_3$) δ 173.9, 173.1, 161.7 (d, *J* = 243.3 Hz), 137.7, 132.9 (d, *J* = 3.2 Hz), 131.8 (d, *J* = 7.4 Hz), 128.1, 128.0, 127.6, 115.1 (d, *J* = 20.7 Hz), 77.8, 66.1, 44.2, 36.8, 28.0, 27.6, 23.6, 9.2, 9.1, 8.6; HRMS (ESI, m/z): calcd. for $C_{24}H_{29}FO_4Na^+$ 423.1942, found 423.1941; IR (KBr thin film, cm^{-1}): ν 3062, 3031, 2928, 1741, 1509, 1461, 1184, 1081, 1015, 818, 758, 701. $[\alpha]_D^{25}$: –26.7 (c 0.6, $CHCl_3$); HPLC analysis: 85% ee (Chiralcel IC, 1:99 ^{i}PrOH/Hexane, 1.0 mL/min, 220 nm), R_t (major) = 7.9 min, R_t (minor) = 6.5 min.

(1*R*,2*R*)-2-methyl-1-phenyl-2-vinylpent-4-yn-1-ol (**7e**)

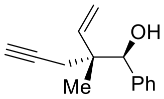

7e

Colorless oil, 19 mg, 47% yield. ^{1}H NMR (600 MHz, $CDCl_3$) δ 7.38–7.27 (m, 5H), 6.06 (dd, *J* = 17.6, 10.9 Hz, 1H), 5.27 (dd, *J* = 10.9, 0.9 Hz, 1H), 5.11 (dd, *J* = 17.6, 0.9 Hz, 1H), 4.75 (d, *J* = 3.2 Hz, 1H), 2.40 (dd, *J* = 16.6, 2.7 Hz, 1H), 2.22 (dd, *J* = 16.6, 2.7 Hz, 1H), 2.10 (t, *J* = 2.7 Hz, 1H), 2.07 (d, *J* = 3.2 Hz, 1H), 1.00 (s, 3H); ^{13}C NMR (150 MHz, $CDCl_3$) δ 141.7, 140.3, 127.9, 127.8, 116.0, 81.7, 78.1, 71.1, 44.9, 28.1, 18.7; HRMS (ESI, m/z): calcd. for $C_{14}H_{16}ONa^+$ 223.1093, found 223.1096; IR (KBr thin film, cm^{-1}): ν 3305, 3086, 3032, 2966, 2927, 2856, 2116, 1733, 1509, 1456, 1385, 1260, 1190, 1083, 1042, 1023, 798, 702, 631. $[\alpha]_D^{25}$: –29.8 (c 0.7, $CHCl_3$); HPLC analysis: 98% ee (Chiralcel OD-H, 2:98 ^{i}PrOH/Hexane, 1.0 mL/min, 220 nm), R_t (major) = 10.8 min, R_t (minor) = 7.4 min.

(1*S*,2*S*)-2-methyl-1-phenyl-2-vinylpent-4-yn-1-yl propionate (**8e**)

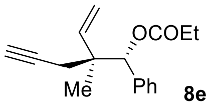

8e

Colorless oil, 26 mg, 51% yield. ^{1}H NMR (600 MHz, $CDCl_3$) δ 7.32–7.26 (m, 5H), 5.98 (dd, *J* = 17.5, 10.9 Hz, 1H), 5.82 (s, 1H), 5.18 (dd, *J* = 10.9, 1.0 Hz, 1H), 5.01 (dd, *J* = 17.5, 1.0 Hz, 1H), 2.42–2.30 (m, 3H), 2.21 (dd, *J* = 16.6, 2.6 Hz, 1H), 2.07 (t, *J* = 2.6 Hz, 1H), 1.14 (t, *J* = 7.6 Hz, 3H), 1.07 (s, 3H); ^{13}C NMR (150 MHz, $CDCl_3$) δ 173.1, 140.9, 137.4, 128.1, 128.0, 127.8, 115.4, 81.0, 79.4, 71.3, 44.0, 28.1, 28.0, 19.4, 9.2; HRMS (ESI, m/z): calcd. for $C_{17}H_{20}O_2Na^+$ 279.1356, found 279.1361; IR (KBr thin film, cm^{-1}): ν 2927, 1742, 1496, 1458, 1264, 1176, 1082, 1014, 817, 739, 702. $[\alpha]_D^{25}$: –40.0 (c 0.1, $CHCl_3$); HPLC analysis: 90% ee (Chiralcel IC, 1:99 ^{i}PrOH/Hexane, 1.0 mL/min, 220 nm), R_t (major) = 4.2 min, R_t (minor) = 3.4 min.

(1*S*,2*S*)-2-(methoxymethyl)-2-methyl-1-phenylpent-4-en-1-ol (**7f**)

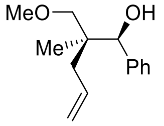

7f

Colorless oil, 19 mg, 43% yield. ^{1}H NMR (600 MHz, $CDCl_3$) δ 7.34–7.22 (m, 5H), 5.90–5.75 (m, 2H), 5.11–5.00 (m, 2H), 3.25 (s, 3H), 3.01 (d, *J* = 9.2 Hz, 1H), 2.85 (d, *J* = 9.2 Hz, 1H), 2.45–2.30 (m, 3H), 2.14 (dd, *J* = 13.6, 7.4 Hz, 1H), 1.15 (t, *J* = 7.6 Hz, 3H), 0.82 (s, 3H); ^{13}C NMR (150 MHz, $CDCl_3$) δ 173.2, 138.3, 134.8, 127.8, 127.7, 127.6, 117.8, 78.6, 76.0, 58.8, 42.0, 39.1, 28.0, 16.7, 9.3; HRMS (ESI, m/z): calcd. for $C_{17}H_{24}O_3Na^+$ 299.1618, found 299.1625; IR (KBr thin film, cm^{-1}): ν 2978, 2926, 2881, 1744, 1457, 1175, 1110, 1016, 913, 751, 704. $[\alpha]_D^{25}$: –30.3 (c 0.1, $CHCl_3$); HPLC analysis: 99% ee (Chiralcel AD-H, 3:97 ^{i}PrOH/Hexane, 1.0 mL/min, 220 nm), R_t (major) = 5.6 min, R_t (minor) = 5.2 min.

(1*R*,2*R*)-2-(methoxymethyl)-2-methyl-1-phenylpent-4-en-1-yl propionate (**8f**)

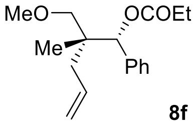

8f

Colorless oil, 28 mg, 51% yield. ^{1}H NMR (600 MHz, $CDCl_3$) δ 7.34–7.22 (m, 5H), 5.90–5.75 (m, 2H), 5.11–5.00 (m, 2H), 3.25 (s, 3H), 3.01 (d, *J* = 9.2 Hz, 1H), 2.85 (d, *J* = 9.2 Hz, 1H), 2.45–2.30 (m, 3H), 2.14 (dd, *J* = 13.6, 7.4 Hz, 1H), 1.15 (t, *J* = 7.6 Hz, 3H), 0.82 (s, 3H); ^{13}C NMR (150 MHz, $CDCl_3$) δ 173.2, 138.3, 134.8, 127.8, 127.7, 127.6, 117.8, 78.6, 76.0, 58.8, 42.0, 39.1, 28.0, 16.7, 9.3; HRMS (ESI, m/z): calcd. for $C_{17}H_{24}O_3Na^+$ 299.1618, found 299.1625; IR (KBr thin film, cm^{-1}): ν 2978, 2926, 2881, 1744, 1457, 1175, 1110, 1016, 913, 751, 704. $[\alpha]_D^{25}$: –23.7 (c 1.8, $CHCl_3$); HPLC analysis: 91% ee (Chiralcel AD-H, 0.5:99.5 ^{i}PrOH/Hexane, 0.5 mL/min, 220 nm), R_t (major) = 8.3 min, R_t (minor) = 7.4 min.

(2*S*,3*S*)-3-hydroxy-2-methyl-2,3-diphenylpropyl propionate (**7g**)

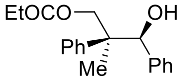

7g

Colorless oil, 27 mg, 45% yield. ^{1}H NMR (400 MHz, $CDCl_3$) δ 7.34–7.24 (m, 5H), 7.21–7.16 (m, 3H), 6.99–6.95 (m, 2H), 4.88 (s, 1H), 4.45 (d, *J* = 11.0 Hz, 1H), 4.33 (d, *J* = 11.0 Hz, 1H), 2.26 (q, *J* = 7.6 Hz, 2H), 2.15 (s, 1H), 1.32 (s, 3H), 1.05 (t, *J* = 7.6 Hz, 3H). ^{13}C NMR (150 MHz, $CDCl_3$) δ 174.7, 141.0, 140.1, 128.1, 127.8, 127.7, 127.0, 78.5, 69.2, 46.9, 27.7, 18.6, 9.2; HRMS (ESI, m/z): calcd. for $C_{19}H_{22}O_3Na^+$ 321.1461, found 321.1460; IR (KBr thin film, cm^{-1}): ν 3484, 2977, 2939, 2358, 1736, 1453, 1377, 1187, 1079, 1021, 703. $[\alpha]_D^{25}$: +19.2 (c 0.1, $CHCl_3$); HPLC analysis: 92% ee (Chiralcel IA, 5:95 ^{i}PrOH/hexane, 1.00 mL/min, 254 nm), Rt (major) = 8.7 min, Rt (minor) = 7.7 min.

(1*R*,2*R*)-2-methyl-1,2-diphenylpropane-1,3-diyl dipropionate (**8g**)

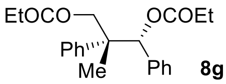

8g

Colorless oil, 33 mg, 47% yield. ^{1}H NMR (400 MHz, $CDCl_3$) δ 7.28–7.24 (m, 5H), 7.22–7.13 (m, 3H), 6.90 (d, *J* = 6.8 Hz, 2H), 6.04 (s, 1H), 4.52 (d, *J* = 11.0 Hz, 1H), 4.19 (d, *J* = 11.0 Hz, 1H), 2.37–2.22 (m, 4H), 1.42 (s, 3H), 1.09–1.03 (m, 6H). ^{13}C NMR (150 MHz, $CDCl_3$) δ 174.5, 173.1, 140.5, 137.0, 128.0, 127.9, 127.7, 127.6, 127.0, 78.7, 68.3, 45.8, 27.9, 27.9, 19.5, 9.2, 9.1; HRMS (ESI, m/z): calcd. for $C_{22}H_{26}O_4Na^+$ 377.1723, found 377.1725; IR (KBr thin film, cm^{-1}): ν 3474, 2983, 2885, 2845, 1743, 1643, 1460, 1263, 1180, 1079, 1014, 765, 696. $[\alpha]_D^{25}$: –32.0 (c 0.1, $CHCl_3$); HPLC analysis: 87% ee (Chiralcel IA, 5:95 ^{i}PrOH/hexane, 1.00 mL/min, 254 nm), Rt (major) = 3.9 min, Rt (minor) = 3.5 min.

(1*S*,2*S*)-2-methyl-1,2-diphenylpent-4-en-1-ol (**7h**)

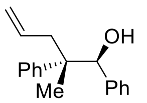

7h

Colorless oil, 24 mg, 48% yield. ^{1}H NMR (400 MHz, $CDCl_3$) δ 7.31–7.15 (m, 8H), 6.96–6.94 (m, 2H), 5.59–5.48 (m, 1H), 5.03 (d, *J* = 17.0 Hz, 1H), 4.93 (d, *J* = 10.2 Hz, 1H), 4.75 (s, 1H), 2.93 (dd, *J* = 14.3, 5.7 Hz, 1H), 2.42 (dd, *J* = 14.2, 8.4 Hz, 1H), 1.93 (s, 1H), 1.31 (s, 3H); ^{13}C NMR (100 MHz, $CDCl_3$) δ 143.5, 140.9, 135.2, 128.0, 127.8, 127.4, 126.4, 117.5, 82.3, 46.5, 41.2, 20.0; HRMS (ESI, m/z): calcd. for $C_{18}H_{20}ONa^+$ 275.1406, found 275.1408; IR (KBr thin film, cm^{-1}): ν 3448, 2957, 2937, 2849, 1743, 1643, 1460, 1263, 1180, 1079, 1014, 765, 754, 696. $[\alpha]_D^{25}$: +19.3 (c 0.1, $CHCl_3$); HPLC analysis: 95% ee (Chiralcel OJ-H, 5:95 ^{i}PrOH/Hexane, 1 mL/min, 220 nm), R_t (major) = 10.3 min, R_t (minor) = 5.3 min.

(1*R*,2*R*)-2-methyl-1,2-diphenylpent-4-en-1-yl propionate (**8h**)

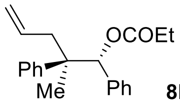

8h

Colorless oil, 29 mg, 48% yield. ^{1}H NMR (400 MHz, $CDCl_3$) δ 7.20–7.00 (m, 8H), 6.72 (d, *J* = 7.3 Hz, 2H), 5.86 (s, 1H), 5.44–5.34 (m, 1H), 4.94 (d, *J* = 16.9 Hz, 1H), 4.85 (d, *J* = 10.2 Hz, 1H), 2.79 (dd, *J* = 14.2, 5.8 Hz, 1H), 2.36–2.30 (m, 3H), 1.24 (s, 3H), 1.08 (t, *J* = 7.6 Hz, 3H); ^{13}C NMR (100 MHz, $CDCl_3$) δ 173.3, 142.6, 137.7, 134.6, 128.0, 127.9, 127.7, 127.4, 127.3, 126.5, 117.7, 82.2, 45.7, 42.5, 28.0, 19.2, 9.2; HRMS (ESI, m/z): calcd. for $C_{21}H_{24}O_2Na^+$ 331.1669, found 331.1671; IR (KBr thin film, cm^{-1}): ν 2987, 2926, 2881, 1769, 1490, 1175, 1130, 1016, 913, 743, 710. $[\alpha]_D^{25}$: +17.5 (c 0.1, $CHCl_3$); HPLC analysis: 95% ee (Chiralcel IA, 0.5:99.5 ^{i}PrOH/Hexane, 0.5 mL/min, 220 nm), R_t (major) = 9.3 min, R_t (minor) = 8.3 min.

(*R*)-((1*R*,2*S*)-1-methyl-2-phenylcyclopropyl)(phenyl)methanol (**7i**)

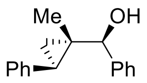

7i

White solid, 21 mg, 44% yield, mp 47–49 ºC. ^{1}H NMR (600 MHz, $CDCl_3$) δ 7.48–7.44 (m, 2H), 7.40–7.36 (m, 2H), 7.32–7.25 (m, 3H), 7.21–7.16 (m, 3H), 4.42 (s, 1H), 2.35 (dd, *J* = 8.9, 6.1 Hz, 1H), 1.97 (s, 1H), 1.26 (dd, *J* = 8.9, 5.1 Hz, 1H), 0.89 (dd, *J* = 6.1, 5.1 Hz, 1H), 0.68 (s, 3H); ^{13}C NMR (150 MHz, $CDCl_3$) δ 142.7, 139.2, 129.3, 128.3, 128.2, 127.6, 126.5, 126.0, 80.2, 28.7, 27.1, 15.3, 14.3; HRMS (ESI, m/z): calcd. for $C_{17}H_{18}ONa^+$ 261.1250, found 261.1249; IR (KBr thin film, cm^{-1}): ν 3061, 3028, 2927, 2855, 1733, 1601, 1496, 1451, 1384, 1265, 1190, 1082, 750, 699. $[\alpha]_D^{25}$: –12.5 (c 1.1, $CHCl_3$); HPLC analysis: 98% ee (Chiralcel OJ-H, 5:95 ^{i}PrOH/Hexane, 1.0 mL/min, 220 nm), R_t (major) = 9.0 min, R_t (minor) = 17.6 min.

(*S*)-((1*S*,2*R*)-1-methyl-2-phenylcyclopropyl)(phenyl)methyl propionate (**8i**)

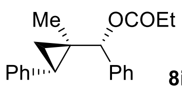

8i

Colorless oil, 28 mg, 48% yield. ^{1}H NMR (400 MHz, $CDCl_3$) δ 7.40–7.28 (m, 5H), 7.27–7.24 (m, 2H), 7.22–7.16 (m, 1H), 7.14–7.10 (m, 2H), 5.55 (s, 1H), 2.49 (qd, *J* = 7.7, 3.0 Hz, 2H), 2.39 (dd, *J* = 8.6, 6.5 Hz, 1H), 1.26–1.21 (m, 4H), 0.92–0.86 (m, 1H), 0.69 (s, 3H); ^{13}C NMR (150 MHz, $CDCl_3$) δ 173.7, 139.4, 138.8, 129.2, 128.3, 128.1, 127.7, 126.6, 126.1, 81.1, 28.1, 27.7, 27.0, 15.6, 14.4, 9.4; HRMS (ESI, m/z): calcd. for $C_{20}H_{22}O_2Na^+$ 317.1512, found 317.1511; IR (KBr thin film, cm^{-1}): ν 3029, 2929, 1737, 1500, 1451, 1176, 1081, 1008, 740, 699. $[\alpha]_D^{25}$: –19.4 (c 0.5, $CHCl_3$); HPLC analysis: 92% ee (Chiralcel AD-H, 3:97 ^{i}PrOH/Hexane, 1.0 mL/min, 220 nm), R_t (major) = 3.6 min, R_t (minor) = 3.3 min.

<u>(*R*)-((1*R*,2*S*)-1-methyl-2-phenylcyclopropyl)(phenyl)methanol</u> **<u>(7j)</u>**

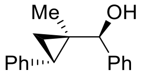

7j

White solid, 20 mg, 42% yield, mp 45–47 ºC. ^{1}H NMR (600 MHz, $CDCl_3$) δ 7.51–7.46 (m, 2H), 7.42–7.37 (m, 2H), 7.34–7.30 (m, 1H), 7.26–7.21 (m, 2H), 7.18–7.14 (m, 1H), 7.10–7.06 (m, 2H), 4.37 (s, 1H), 2.39 (dd, *J* = 8.9, 5.9 Hz, 1H), 2.04 (s, 1H), 1.18 (dd, *J* = 8.9, 4.9 Hz, 1H), 0.95 (dd, *J* = 5.9, 4.9 Hz, 1H), 0.67 (s, 3H); ^{13}C NMR (150 MHz, $CDCl_3$) δ 142.5, 138.6, 129.2, 128.2, 128.1, 127.5, 126.4, 126.0, 80.9, 28.8, 26.6, 16.0, 13.9; HRMS (ESI, m/z): calcd. for $C_{17}H_{18}ONa^+$ 261.1250, found 261.1249; IR (KBr thin film, cm^{-1}): ν 3061, 3028, 2927, 2855, 1735, 1602, 1496, 1450, 1384, 1265, 1189, 1081, 1024, 724, 699. $[\alpha]_D^{25}$: -12.5 (c 1.1, $CHCl_3$); HPLC analysis: 99% ee (Chiralcel OJ-H, 5:95 ^{i}PrOH/Hexane, 1.0 mL/min, 220 nm), R_t (major) = 10.2 min, R_t (minor) = 8.8 min.

<u>(*S*)-((1*S*,2*R*)-1-methyl-2-phenylcyclopropyl)(phenyl)methyl propionate</u> **<u>(8j)</u>**

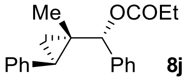

8j

Colorless oil, 30 mg, 51% yield. ^{1}H NMR (400 MHz, $CDCl_3$) δ 7.43–7.34 (m, 4H),7.34–7.27 (m, 1H), 7.25–7.18 (m, 2H), 7.18–7.12 (m, 1H), 7.06–7.00 (m, 2H), 5.56 (s, 1H), 2.47 (qd, *J* = 7.6, 3.4 Hz, 2H), 2.35 (dd, *J* = 8.8, 6.3 Hz, 1H), 1.25–1.15 (m, 4H), 0.95–0.88 (m, 1H), 0.70 (s, 3H); ^{13}C NMR (150 MHz, $CDCl_3$) δ 173.8, 139.4, 138.3, 129.2, 128.3, 128.1, 127.8, 126.6, 126.1, 81.2, 28.0, 26.9, 26.5, 16.3, 14.8, 9.4; HRMS (ESI, m/z): calcd. for $C_{20}H_2O_2Na^+$ 317.1512, found 317.1516; IR (KBr thin film, cm^{-1}): ν 3063, 3030, 2978, 2934, 1737, 1603, 1497, 1452, 1183, 1081, 1008, 774, 730, 699. $[\alpha]_D^{25}$: +19.4 (c 0.5, $CHCl_3$); HPLC analysis: 82% ee (Chiralcel OD-H, 0.5:99.5 ^{i}PrOH/Hexane, 1.0 mL/min, 40 ℃, 220 nm), R_t (major) = 5.5 min, R_t (minor) = 4.0 min.

<u>(*S*)-2-((*S*)-hydroxy(naphthalen-2-yl)methyl)-2-methylpent-4-en-1-yl propionate</u> **<u>(7k)</u>**

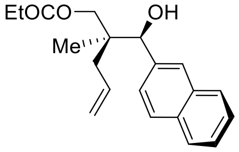

7k

Colorless semisolid, 30.1 mg, 48% yield. ^{1}H NMR (600 MHz, $CDCl_3$) δ 7.86–7.76 (m, 3H), 7.74 (s, 1H), 7.51–7.40 (m, 3H), 5.83 (ddt, *J* = 17.8, 10.3, 7.6 Hz, 1H), 5.15–5.01 (m, 2H), 4.82 (d, *J* = 3.2 Hz, 1H), 4.10 (d, *J* = 11.2 Hz, 1H), 3.79 (d, *J* = 11.2 Hz, 1H), 2.62 (d, *J* = 3.3 Hz, 1H), 2.45 (dd, *J* = 13.8, 7.8 Hz, 1H), 2.35 (q, *J* = 7.6 Hz, 2H), 2.16 (dd, *J* = 13.8, 7.3 Hz, 1H), 1.16 (t, *J* = 7.6 Hz, 3H), 0.91 (s, 3H). ^{13}C NMR (151 MHz, $CDCl_3$) δ 174.5, 138.5, 134.4, 133.0, 132.9, 128.1, 127.7, 127.4, 126.6, 126.2, 126.0, 125.9, 118.4, 77.8, 68.5, 42.1, 38.0, 27.8, 18.0, 9.3. HRMS (ESI, m/z): calcd. for $C_{20}H_{24}O_3Na^+$ 335.1623, found 335.1625. IR (KBr thin film, cm^{-1}): ν 3440, 2922, 2848, 2381, 2348, 2313, 1729, 1503, 1363, 1274, 762, 748. $[\alpha]_D^{20}$: –20.0 (c 0.2, $CHCl_3$); HPLC analysis: 99% ee (Chiralcel AD-H, 5:95 ^{i}PrOH/hexane, 1.0 mL/min, 254 nm), R_t (major) = 8.6 min, R_t (minor) = 9.4 min.

(1*R*,2*R*)-2-allyl-2-methyl-1-(naphthalen-2-yl)propane-1,3-diyl dipropionate **(8k)**

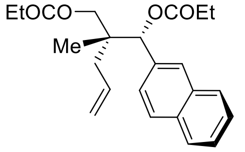

8k

Colorless semisolid, 36.9 mg, 50% yield. ^{1}H NMR (600 MHz, $CDCl_3$) δ 7.87–7.75 (m, 3H), 7.72 (s, 1H), 7.51–7.43 (m, 2H), 7.40 (dd, *J* = 8.5, 1.7 Hz, 1H), 5.96 (s, 1H), 5.81 (ddt, *J* = 17.5, 10.1, 7.5 Hz, 1H), 5.14–5.00 (m, 2H), 4.03 (d, *J* = 11.2 Hz, 1H), 3.71 (d, *J* = 11.2 Hz, 1H), 2.49–2.28 (m, 5H), 2.18 (dd, *J* = 13.8, 7.4 Hz, 1H), 1.17–1.14 (m, 6H), 0.99 (s, 3H). ^{13}C NMR (151 MHz, $CDCl_3$) δ 174.3, 173.3, 135.0, 133.6, 133.1, 132.9, 128.1, 127.7, 126.9, 126.3, 126.2, 125.5, 125.5, 118.7, 78.4, 67.4, 41.4, 38.9, 28.0, 27.7, 17.9, 9.3, 9.2. HRMS (ESI, m/z): calcd. for $C_{23}H_{28}O_4Na^+$ 391.1885, found 391.1886. IR (KBr thin film, cm^{-1}): ν 3470, 3413, 2982, 2917, 2848, 2848, 2045, 2045, 1732, 1634, 1462, 1277, 1179, 1081, 742, 605, 480. $[\alpha]_D^{20}$: –10.9 (c 0.5, $CHCl_3$). HPLC analysis: 93% ee (Chiralcel AD-H, 1:99 ^{i}PrOH/hexane, 1.0 mL/min, 254 nm), R_t (major) = 8.6 min, R_t (minor) = 7.4 min.

(1*R*,2*S*)-2-methyl-1-(naphthalen-2-yl)-2-vinylpent-4-en-1-ol **(7l)**

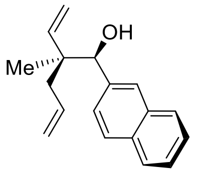

7l

Colorless semisolid, 23.2 mg, 46% yield. ^{1}H NMR (400 MHz, $CDCl_3$) δ 7.79 (dd, *J* = 5.2, 3.2 Hz, 2H), 7.74 (d, *J* = 8.5 Hz, 1H), 7.69 (s, 1H), 7.47–7.39 (m, 3H), 5.91 (dd, *J* = 17.6, 10.8 Hz, 1H), 5.78–5.64 (m, 1H), 5.22 (dd, *J* = 10.8, 0.9 Hz, 1H), 5.03–4.95 (m, 3H), 4.54 (s, 1H), 2.35–2.19 (m, 2H), 2.08 (dd, *J* = 13.6, 8.3 Hz, 1H), 0.89 (s, 3H). ^{13}C NMR (100 MHz, $CDCl_3$) δ 143.2, 138.2, 134.8, 133.0, 132.8, 128.1, 127.7, 127.1, 127.0, 126.3, 126.0, 125.9, 117.6, 115.9, 79.9, 45.9, 42.3, 17.6. HRMS (ESI, m/z): calcd. for $C_{18}H_{20}ONa^+$ 275.1412, found 275.1408; IR (KBr thin film, cm^{-1}): ν 3476, 3416, 2922, 2378, 2351, 2313, 1643, 1384, 1274, 917, 768, 751. $[\alpha]_D^{20}$: +101.9 (c 0.1, $CHCl_3$). HPLC analysis: 99% ee (Chiralcel OD-H, 5:95 ^{i}PrOH/hexane, 1.0 mL/min,

254 nm), R_t (major) = 9.1 min, R_t (minor) = 10.0 min.

(1*S*,2*R*)-2-methyl-1-(naphthalen-2-yl)-2-vinylpent-4-en-1-yl propionate (**8l**)

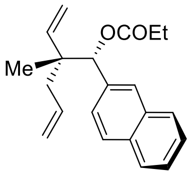

8l

Colorless semisolid, 31.4 mg, 51% yield. ^{1}H NMR (400 MHz, $CDCl_3$) δ 7.78–7.59 (m, 4H), 7.42–7.27 (m, 3H), 5.89 (dd, *J* = 17.6, 10.9 Hz, 1H), 5.73–5.58 (m, 2H), 5.08 (dd, *J* = 10.9, 0.8 Hz, 1H), 5.01–4.86 (m, 2H), 4.82 (dd, *J* = 17.6, 0.8 Hz, 1H), 2.41–2.24 (m, 2H), 2.20 (dd, *J* = 13.6, 6.3 Hz, 1H), 2.10 (dd, *J* = 13.6, 8.3 Hz, 1H), 1.06 (t, *J* = 7.6 Hz, 3H), 0.89 (s, 3H). ^{13}C NMR (101 MHz, $CDCl_3$) δ 172.2, 140.9, 134.3, 133.2, 131.9, 131.6, 127.0, 126.5, 126.3, 126.0, 125.1, 125.0, 116.8, 114.1, 79.9, 43.4, 41.1, 26.9, 17.7, 8.1. HRMS (ESI, m/z): calcd. for $C_{21}H_{24}O_2Na^+$ 331.1674, found 331.1675. IR (KBr thin film, cm^{-1}): ν 3446, 2988, 2378, 2307, 1738, 1637, 1506, 1363, 1274, 1262, 765, 751. $[\alpha]_D^{20}$: +7.5 (c 0.3, $CHCl_3$). HPLC analysis: 90% ee (Chiralcel OD-H, 1:99 ^{i}PrOH/hexane, 1.0 mL/min, 254 nm), R_t (major) = 3.6 min, R_t (minor) = 2.7 min.

(1*S*,2*S*)-2-ethynyl-2-methyl-1-(naphthalen-2-yl)pent-4-en-1-ol (**7m**)

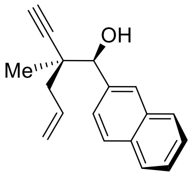

7m

Colorless semisolid, 23.6 mg, 47% yield. ^{1}H NMR (400 MHz, $CDCl_3$) δ 7.85–7.79 (m, 4H), 7.59 (dd, *J* = 8.5, 1.3 Hz, 1H), 7.53–7.43 (m, 2H), 6.13–5.87 (m, 1H), 5.18–5.10 (m, 2H), 4.71 (d, *J* = 4.0 Hz, 1H), 2.61 (d, *J* = 4.0 Hz, 1H), 2.51 (dd, *J* = 13.6, 6.5 Hz, 1H), 2.36 (s, 1H), 2.19 (dd, *J* = 13.6, 7.9 Hz, 1H), 1.07 (s, 3H). ^{13}C NMR (100 MHz, $CDCl_3$) δ 137.2, 134.4, 133.3, 132.9, 128.2, 127.7, 127.4, 127.1, 126.1, 125.9, 118.5, 87.7, 79.1, 72.8, 43.1, 42.0, 21.9. IR (KBr thin film, cm^{-1}): ν 3074, 3074, 2925, 2348, 1640, 1512, 1384, 1274, 1265, 914, 754, 474. $[\alpha]_D^{20}$: +15.6 (c 0.2, $CHCl_3$). HPLC analysis: 97% ee (Chiralcel AD-H, 5:95 ^{i}PrOH/hexane, 1.0 mL/min, 254 nm), R_t (major) = 10.1 min, R_t (minor) = 9.2 min.

(1*R*,2*R*)-2-ethynyl-2-methyl-1-(naphthalen-2-yl)pent-4-en-1-yl propionate (**8m**)

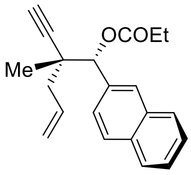

8m

Colorless semisolid, 29.8 mg, 49% yield. ^{1}H NMR (400 MHz, $CDCl_3$) δ 7.85–7.66 (m, 4H), 7.54 (dd, *J* = 8.5, 1.3 Hz, 1H), 7.44–7.35 (m, 2H), 6.01–5.80 (m, 1H), 5.73 (s, 1H), 5.10–5.01 (m, 2H), 2.46–2.28 (m, 3H), 2.22–2.11 (m, 2H), 1.09 (t, *J* = 7.6 Hz,

3H), 1.07 (s, 3H). ^{13}C NMR (101 MHz, $CDCl_3$) δ 172.3, 133.6, 132.7, 132.2, 131.6, 127.1, 126.6, 126.5, 126.3, 125.1, 125.0, 124.9, 117.5, 85.7, 78.3, 71.0, 41.7, 38.9, 26.8, 21.9, 8.1. HRMS (ESI, m/z): calcd. for $C_{21}H_{22}O_2Na^+$ 329.1517, found 329.1520. IR (KBr thin film, cm^{-1}): ν 3440, 3006, 2381, 2313, 1735, 1509, 1360, 1274, 1256, 765, 748. $[\alpha]_D^{20}$: –33.6 (c 0.2, $CHCl_3$). HPLC analysis: 97% ee (Chiralcel AD-H, 1:99 ^{i}PrOH/hexane, 1.0 mL/min, 254 nm), R_t (major) = 7.9 min, R_t (minor) = 5.6 min.

(1*R*,2*R*)-2-benzyl-1-(naphthalen-2-yl)-2-vinylpent-4-en-1-ol (**7n**)

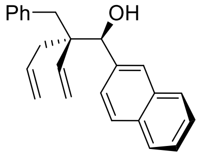

7n

Colorless oil, 30 mg, 46% yield. ^{1}H NMR (600 MHz, $CDCl_3$) δ 7.87–7.75 (m, 4H), 7.53–7.43 (m, 3H), 7.25–7.15 (m, 3H), 7.15–7.10 (m, 2H), 6.23–6.12 (m, 1H), 5.88 (dd, *J* = 17.8, 11.1 Hz, 1H), 5.30–5.23 (m, 3H), 4.96 (dd, *J* = 17.8, 0.8 Hz, 1H), 4.86 (d, *J* = 5.5 Hz, 1H), 2.78 (d, *J* = 13.5 Hz, 1H), 2.65 (d, *J* = 13.5 Hz, 1H), 2.61 (dd, *J* = 14.2, 8.2 Hz, 1H), 2.19 (d, *J* = 5.5 Hz, 1H), 2.09 (dd, *J* = 14.2, 6.1 Hz, 1H); ^{13}C NMR (150 MHz, $CDCl_3$) δ 140.7, 138.6, 137.9, 134.9, 133.0, 132.9, 130.9, 128.2, 127.8, 127.7, 127.3, 127.1, 126.5, 126.3, 126.2, 126.0, 118.6, 116.6, 77.2, 49.1, 40.9, 35.2; HRMS (ESI, m/z): calcd. for $C_{22}H_{24}ONa^+$ 351.1719, found 351.1719; IR (KBr thin film, cm^{-1}): ν 3061, 3029, 2925, 2855, 1741, 1601, 1495, 1455, 1358, 1177, 817, 754, 702.

(1*S*,2*S*)-2-benzyl-1-(naphthalen-2-yl)-2-vinylpent-4-en-1-yl propionate (**8n**)

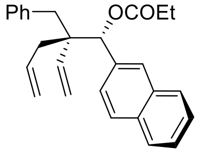

8n

Colorless oil, 36 mg, 47% yield. ^{1}H NMR (600 MHz, $CDCl_3$) δ 7.85–7.72 (m, 4H), 7.49–7.42 (m, 3H), 7.22–7.14 (m, 3H), 7.08–7.03 (m, 2H), 6.16–6.07 (m, 1H), 5.85 (dd, *J* = 17.6, 11.2 Hz, 1H), 5.83 (s, 1H), 5.24–5.17 (m, 2H), 5.12 (dd, *J* = 17.0, 1.8 Hz, 1H), 4.88 (dd, *J* = 17.6, 0.5 Hz, 1H), 2.83 (d, *J* = 13.6 Hz, 1H), 2.66 (d, *J* = 13.6 Hz, 1H), 2.51 (dd, *J* = 14.4, 7.9 Hz, 1H), 2.37 (qd, *J* = 7.6, 2.3 Hz, 2H), 2.13 (dd, *J* = 14.4, 6.3 Hz, 1H), 1.14 (t, *J* = 7.6 Hz, 3H); ^{13}C NMR (150 MHz, $CDCl_3$) δ 173.1, 140.2, 137.3, 135.3, 134.4, 133.1, 132.7, 130.9, 128.2, 127.8, 127.7, 127.3, 126.4, 126.3, 126.2, 118.6, 116.1, 79.1, 47.6, 40.4, 35.5, 28.0, 9.2; HRMS (ESI, m/z): calcd. for $C_{27}H_{28}O_2Na^+$ 407.1982, found 407.1989; IR (KBr thin film, cm^{-1}): ν 2964, 2929, 1741, 1509, 1457, 1385, 1176, 1082, 1017, 799, 752, 701. $[\alpha]_D^{25}$: –27.1 (c 0.8, $CHCl_3$); HPLC analysis: 93% ee (Chiralcel AD-H, 5:95 ^{i}PrOH/Hexane, 1.0 mL/min, 220 nm), R_t (major) = 4.3 min, R_t (minor) = 4.7 min.

(*R*)-1-(4-Bromophenyl)-1-(4-chlorophenyl)-1-hydroxypropan-2-one (**9a**)

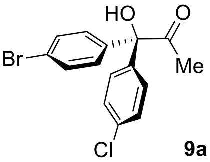

9a

Colorless oil, 20.7 mg, 61% yield. ^{1}H NMR (600 MHz, $CDCl_3$) 7.56–7.49 (m, 2H), 7.40–7.33 (m, 2H), 7.30–7.26 (m, 2H), 7.24–7.21 (m, 2H), 4.78 (s, 1H), 2.26 (s, 3H); ^{13}C NMR (100 MHz, $CDCl_3$) δ 207.5, 140.0, 139.4, 134.6, 131.8, 129.7, 129.5, 129.4, 128.8, 128.7, 127.9, 122.8, 84.9, 26.1; IR (KBr thin film, cm^{-1}): ν 3444, 2929, 1712, 1489, 1358, 1162, 1099, 1075, 1010, 891, 825, 703; $[\alpha]_D^{25}$: –7.2 (c 1.1, $CHCl_3$); HPLC analysis: 94% *ee* (Chiralcel AD-H, 0.5:99.5 ^{i}PrOH/Hexane, 1.0 mL/min, 220 nm), R_t (major) = 64.7 min, R_t (minor) = 58.5 min.

(*R*)-2-Hydroxy-2-phenethyl-1-phenylhexan-1-one (**9b**)

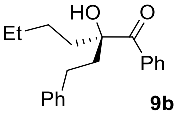

9b

Colorless oil, 30 mg, 99% yield. ^{1}H NMR (400 MHz, $CDCl_3$) δ 8.02 (d, *J* = 7.3 Hz, 2H), 7.61 (t, *J* = 7.4 Hz, 1H), 7.49 (t, *J* = 7.7 Hz, 2H), 7.20 (t, *J* = 7.3 Hz, 2H), 7.13 (t, *J* = 7.4 Hz, 1H), 7.02 (d, *J* = 7.0 Hz, 2H), 4.55 (s, 1H), 2.83–2.73 (m, 1H), 2.35–2.23 (m, 3H), 2.12–1.92 (m, 2H), 1.52–1.41 (m, 1H), 1.30–1.19 (m, 2H), 1.04–0.94 (m, 1H), 0.81 (t, *J* = 7.3 Hz, 3H). ^{13}C NMR (100 MHz, $CDCl_3$) δ 204.7, 141.8, 134.3, 133.5, 129.1, 128.8, 128.5, 128.4, 126.0, 81.7, 42.8, 40.6, 30.0, 25.6, 23.0, 13.9. HRMS (ESI, m/z): calcd. for $C_{20}H_{24}O_2Na^+$ 319.1669, found 319.1667. IR (KBr thin film, cm^{-1}): ν 3464, 2959, 2925, 2368, 1653, 1266, 1017, 751; $[\alpha]_D^{25}$: –37.1 (c 0.1, $CHCl_3$); HPLC analysis: 93% *ee* (Chiralcel OD-H, 5:95 ^{i}PrOH/Hexane, 1.0 mL/min, 220 nm), R_t (major) = 3.2 min, R_t (minor) = 3.5 min.

(*R*)-2-Hydroxy-2,3-dimethyl-1-phenylbutan-1-one (**9c**)

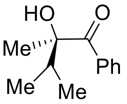

9c

Colorless oil, 18 mg, 94% yield. ^{1}H NMR (400 MHz, $CDCl_3$) δ 8.01–7.97 (m, 2H), 7.59–7.55 (m, 1H), 7.49–7.45 (m, 2H), 4.16 (s, 1H), 2.36–2.30 (m, 1H), 1.59 (s, 3H), 1.10 (d, *J* = 6.7 Hz, 3H), 0.71 (d, *J* = 6.7 Hz, 3H). ^{13}C NMR (150 MHz, $CDCl_3$) δ 205.6, 134.2, 133.2, 129.6, 128.7, 80.9, 36.0, 25.4, 17.2, 16.3; HRMS (ESI, m/z): calcd. for $C_{12}H_{16}O_2Na^+$ 215.1043, found 215.1041. IR (KBr thin film, cm^{-1}): ν 3467, 2970, 2928, 2849, 1670, 1456, 1263, 744; $[\alpha]_D^{25}$: –36.5 (c 0.1, $CHCl_3$); HPLC analysis: 95% *ee* (Chiralcel IA, 1:99 ^{i}PrOH/Hexane, 1.0 mL/min, 220 nm), R_t (major) = 6.6 min, R_t (minor) = 7.3 min.

(*S*)-2-(Methoxymethyl)-2-methyl-1-phenylpent-4-en-1-one (**9d**)

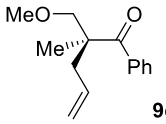

9d

Colorless oil, 19 mg, 87% yield. ^{1}H NMR (400 MHz, $CDCl_3$) δ 7.62–7.50 (m, 2H), 7.49–7.31 (m, 3H), 5.80–5.60 (m, 1H), 5.12–4.98 (m, 2H), 3.57 (d, *J* = 9.0 Hz, 1H), 3.45 (d, *J* = 9.0 Hz, 1H), 3.29 (s, 3H), 2.56 (dd, *J* = 13.8, 7.1 Hz, 1H), 2.39 (dd, *J* = 13.8, 7.6 Hz, 1H), 1.30 (s, 3H); ^{13}C NMR (150 MHz, $CDCl_3$) δ 208.7, 140.0, 133.5, 130.5, 128.1, 127.1, 118.6, 78.0, 59.2, 52.4, 40.2, 20.6; HRMS (ESI, m/z): calcd. for $C_{19}H_{20}O_3Na^+$ 319.1305, found 319.1310; IR (KBr thin film, cm^{-1}): ν 2905, 2929, 2887, 1685, 1233, 1111, 918, 942, 701; $[\alpha]_D^{25}$: –17.3 (c 0.4, $CHCl_3$); HPLC analysis: 99% *ee* (Chiralcel IA, 1:99 ^{i}PrOH/Hexane, 0.5 mL/min, 220 nm), R_t (major) = 6.9 min, R_t (minor) = 7.8 min.

(*R*)-2-Benzyl-1-(naphthalen-2-yl)-2-vinylpent-4-en-1-one (**9e**)

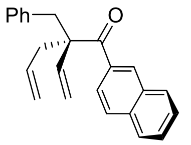

9e

Colorless oil, 29.5 mg, 90% yield. ^{1}H NMR (400 MHz, $CDCl_3$) δ 8.46 (s, 1H), 7.95–7.80 (m, 4H), 7.59–7.49 (m, 2H), 7.22–7.15 (m, 3H), 7.10–7.05 (m, 2H), 6.18 (dd, *J* = 17.8, 10.9 Hz, 1H), 5.84–5.72 (m, 1H), 5.41 (d, *J* = 10.9 Hz, 1H), 5.24 (d, *J* = 17.8 Hz, 1H), 5.07 (dd, *J* = 10.3, 1.9 Hz, 1H), 4.96 (dd, *J* = 17.1, 1.9 Hz, 1H), 3.30 (s, 2H), 2.72 (dd, *J* = 14.5, 7.4 Hz, 1H), 2.65 (dd, *J* = 14.5, 6.9 Hz, 1H); ^{13}C NMR (150 MHz, $CDCl_3$) δ 203.0, 141.6, 137.2, 135.0, 134.9, 133.6, 132.3, 131.0, 130.9, 129.7, 128.2, 127.9, 127.8, 127.7, 126.6, 126.5, 125.8, 119.0, 116.6, 58.1, 41.8, 39.4; HRMS (ESI, m/z): calcd. for $C_{24}H_{22}ONa^+$ 349.1563, found 349.1565; IR (KBr thin film, cm^{-1}): ν 2956, 1735, 1676, 1266, 1209, 1174, 745, 698; $[\alpha]_D^{25}$: –39.1 (c 1.3, $CHCl_3$); HPLC analysis: 96% *ee* (Chiralcel IC, 1:99 ^{i}PrOH/Hexane, 1.0 mL/min, 220 nm), R_t (major) = 5.2 min, R_t (minor) = 4.7 min.

(*S*)-2-Benzoyl-2-(4-fluorobenzyl)butyl propionate (**9f**)

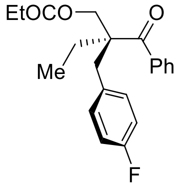

9f

Colorless oil, 34 mg, 99% yield. ^{1}H NMR (600 MHz, $CDCl_3$) δ 7.52–7.43 (m, 3H), 7.42–7.36 (m, 2H), 7.03–6.96 (m, 2H), 6.95–6.90 (m, 2H), 4.22 (d, *J* = 11.6 Hz, 1H), 4.16 (d, *J* = 11.6 Hz, 1H), 3.22 (d, *J* = 14.0 Hz, 1H), 3.06 (d, J = 14.0 Hz, 1H), 2.32 (q, *J* = 7.6 Hz, 2H), 1.90–1.75 (m, 2H), 1.12 (t, *J* = 7.6 Hz, 3H), 0.93 (t, *J* = 7.6 Hz, 3H); ^{13}C NMR (150 MHz, $CDCl_3$) δ 206.3, 173.9, 161.8 (d, *J* = 244.0 Hz), 139.5, 132.5 (d, *J* = 2.7 Hz), 131.8 (d, *J* = 8.1 Hz), 131.3, 128.5, 127.0, 115.3 (d, *J* = 20.7 Hz), 64.7, 56.1, 38.0, 27.6, 26.3, 9.2, 8.7; HRMS (ESI, m/z): calcd. for $C_{21}H_{23}O_3FNa^+$ 365.1523, found 365.1527; IR (KBr thin film, cm^{-1}): ν 2974, 2941, 1745, 1676, 1513,

1462, 1224, 1180, 1084, 1019, 706; $[\alpha]_D^{25}$: –7.2 (c 1.3, $CHCl_3$); HPLC analysis: 95% *ee* (Chiralcel IA, 1:99 ^{i}PrOH/Hexane, 1.0 mL/min, 220 nm), R_t (major) = 9.3 min, R_t (minor) = 7.5 min

(*S*)-2-Methyl-3-oxo-2,3-diphenylpropyl propionate (**9g**)

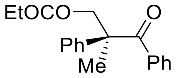

9g

Colorless oil, 27.5 mg, 93% yield. ^{1}H NMR (600 MHz, $CDCl_3$) δ 7.47–7.42 (m, 2H), 7.42–7.35 (m, 3H), 7.34–7.28 (m, 3H), 7.26–7.22 (m, 2H), 4.60 (d, *J* = 10.9 Hz, 1H), 4.47 (d, *J* = 10.9 Hz, 1H), 2.24 (q, *J* = 7.6 Hz, 2H), 1.72 (s, 3H), 1.04 (t, *J* = 7.6 Hz, 3H); ^{13}C NMR (150 MHz, $CDCl_3$) δ 201.5, 174.2, 140.4, 136.2, 132.1, 129.5, 129.2, 128.2, 127.7, 126.5, 70.0, 54.6, 27.6, 21.9, 9.1; HRMS (ESI, m/z): calcd. for $C_{19}H_{20}O_3Na^+$ 319.1305, found 319.1310; IR (KBr thin film, cm^{-1}): ν 2962, 2926, 1739, 1688, 1263, 1182, 1078, 1022, 974, 760, 715, 703; $[\alpha]_D^{25}$: –37.2 (c 1.1, $CHCl_3$); HPLC analysis: 93% *ee* (Chiralcel OJ-H, 1.5:98.5 ^{i}PrOH/Hexane, 1.0 mL/min, 220 nm), R_t (major) = 6.5 min, R_t (minor) = 7.5 min.

Methyl (*R*)-2-(4-chlorobenzoyl)-2-(naphthalen-2-ylmethyl)pent-4-enoate (**9h**)

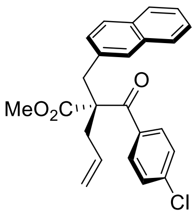

9h

Colorless oil, 39 mg, 99% yield, 90% ee. ^{1}H NMR (400 MHz, $CDCl_3$) δ 7.83–7.77 (m, 3H), 7.75–7.68 (m, 2H), 7.54–7.41 (m, 5H), 7.08 (dd, *J* = 8.5, 1.5 Hz, 1H), 5.81–5.71 (m, 1H), 5.18 (d, *J* = 9.7 Hz, 1H), 5.09 (dd, *J* = 16.9, 1.4 Hz, 2H) 3.64 (s, 3H), 3.59 (d, *J* = 14.2 Hz, 1H), 3.53 (d, *J* = 14.2 Hz, 1H), 2.82–2.74 (m, 2H); ^{13}C NMR (100 MHz, $CDCl_3$) δ 194.8, 173.0, 139.5, 134.4, 133.3, 132.5, 132.1, 130.0, 129.2, 129.1, 128.1, 127.9, 127.7, 126.1, 125.8, 119.9, 62.3, 52.5, 39.0, 37.3; HRMS (ESI, m/z): calcd. for $C_{24}H_{21}O_3ClNa^+$ 415.1071, found 415.1069; IR (KBr thin film, cm^{-1}): ν 3443, 2963, 2310, 1739, 1632, 1263, 1176, 1083, 1014, 800, 751; $[\alpha]_D^{25}$: –40.4 (c 0.1, $CHCl_3$); HPLC analysis: 90% *ee* (Chiralcel AD-H, 5:95 ^{i}PrOH/Hexane, 1.0 mL/min, 220 nm), R_t (major) = 6.7 min, R_t (minor) = 7.4 min.

(*R*)-Methyl 2-(2-naphthoyl)-2-benzylpent-4-enoate (**9i**)

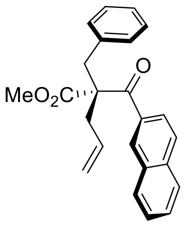

9i

Colorless oil, 35.4 mg, 99% yield. ^{1}H NMR (600 MHz, $CDCl_3$) δ 8.41–8.38 (m, 1H), 7.97–7.91 (m, 2H), 7.90–7.84 (m, 2H), 7.62–7.57 (m, 1H), 7.56–7.51 (m, 1H), 7.24–

7.17 (m, 3H), 7.03–6.99 (m, 2H), 5.80–5.71 (m, 1H), 5.16–5.11 (m, 1H), 5.10–5.04 (m, 1H), 3.61 (s, 3H), 3.51 (d, *J* = 14.2 Hz, 1H), 3.48 (d, *J* = 14.2 Hz, 1H), 2.87–2.77 (m, 2H); ^{13}C NMR (150 MHz, $CDCl_3$) δ 195.9, 173.4, 136.0, 135.4, 133.4, 132.6, 132.4, 130.3, 130.1, 130.0, 128.8, 128.6, 128.4, 127.8, 127.1, 127.0, 124.5, 119.7, 62.4, 52.5, 39.0, 37.4; IR (KBr thin film, cm^{-1}): ν 2962, 1733, 1632, 1385, 1260, 1102, 1022, 796; $[\alpha]_D^{25}$: −12.5 (c 1.1, $CHCl_3$); HPLC analysis: 92% *ee* (Chiralcel IA, 2:98 ^{i}PrOH/Hexane, 1.0 mL/min, 220 nm), R_t (major) = 6.1 min, R_t (minor) = 8.8 min.

((2*R*,3*R*)-3-Hexyl-3-pentyloxiran-2-yl)(phenyl)methanone (**9j**)

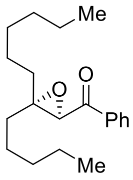

9j

Colorless oil, 28 mg, 93% yield. ^{1}H NMR (400 MHz, $CDCl_3$) δ 8.10–7.90 (m, 2H), 7.65–7.55 (m, 1H), 7.54–7.41 (m, 2H), 4.06 (s, 1H), 1.86–1.76 (m, 2H), 1.52–1.20 (m, 16H), 1.00–0.85 (m, 3H), 0.80–0.70 (m, 3H); ^{13}C NMR (100 MHz, $CDCl_3$) δ 194.5, 136.0, 133.8, 128.8, 128.3, 67.2, 63.7, 34.6, 32.0, 29.9, 29.5, 25.2, 24.8, 24.5, 22.7, 22.5, 14.1, 13.9; HRMS (ESI, m/z): calcd. for $C_{20}H_{30}O_2Na^+$ 325.2138, found 325.2141; IR (KBr thin film, cm^{-1}): ν 2956, 2932, 2861, 1694, 1450, 1227, 748, 692; $[\alpha]_D^{25}$: −8.2 (c 0.7, $CHCl_3$); HPLC analysis: 98% *ee* (Chiralcel IA, 2:98 ^{i}PrOH/Hexane, 1.0 mL/min, 220 nm), R_t (major) = 4.0 min, R_t (minor) = 4.5 min.

Naphthalen-1-yl((2*R*,3*R*)-3-phenyl-3-(*p*-tolyl)oxiran-2-yl)methanone (**9k**)

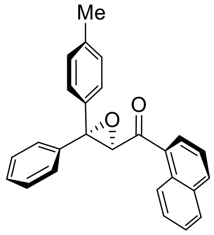

9k

White solid, 29.6 mg, 81% yield, mp 87–89 ºC. ^{1}H NMR (600 MHz, $CDCl_3$) δ 8.34–8.29 (m, 1H), 8.05–7.95 (m, 2H), 7.86–7.82 (m, 1H), 7.53–7.43 (m, 5H), 7.42–7.32 (m, 3H), 7.25–7.20 (m, 2H), 6.99–6.94 (m, 2H), 4.68 (s, 1H), 2.21 (s, 3H); ^{13}C NMR (150 MHz, $CDCl_3$) δ 195.2, 139.4, 138.0, 133.9, 133.7, 133.2, 132.0, 130.2, 129.1, 128.8, 128.7, 128.6, 128.3, 128.1, 128.0, 127.2, 126.7, 125.8, 124.2, 68.2, 67.7, 21.2; $[\alpha]_D^{25}$: −10.1 (c 1.0, $CHCl_3$); HRMS (ESI, m/z): calcd. for $C_{14}H_{18}O_2Na^+$ 387.1356, found 387.1363; IR (KBr thin film, cm^{-1}): ν 3060, 2962, 2926, 2869, 1733, 1685, 1510, 1275, 1245, 1180, 805, 781, 751; HPLC analysis: 95% *ee* (Chiralcel AD-H, 5:95 ^{i}PrOH/Hexane, 1.0 mL/min, 220 nm), R_t (major) = 8.0 min, R_t (minor) = 10.9 min.

(*R*)-3-Benzyl-1-phenylheptan-3-ol (**10a**)

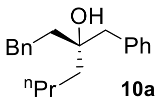

Colorless oil, 22 mg, 79% yield. ^{1}H NMR (600 MHz, $CDCl_3$) δ 7.32–7.22 (m, 8H), 7.19–7.16 (m, 2H), 2.81 (s, 2H), 2.70 (t, *J* = 8.8 Hz, 2H), 1.78–1.69 (m, 2H), 1.52–1.46 (m, 2H), 1.42–1.37 (m, 2H), 1.33 (q, *J* = 7.3 Hz, 2H), 1.29 (s, 1H), 0.93 (t, *J* = 7.3 Hz, 3H). ^{13}C NMR (150 MHz, CDCl3) δ 142.6, 137.2, 130.7, 128.5, 128.4, 126.6, 125.9, 74.2, 45.7, 40.7, 38.5, 30.2, 26.2, 23.3, 14.3; HRMS (ESI, m/z): calcd. for $C_{20}H_{26}ONa^+$ 305.1876, found 305.1875; IR (KBr thin film, cm^{-1}): ν 3453, 2963, 2925, 2852, 1460, 1266, 1086, 751; $[\alpha]_D^{25}$: –41.3 (c 0.1, $CHCl_3$); HPLC analysis: 88% *ee* (Chiralcel IA, 2:98 ^{i}PrOH/Hexane, 1.0 mL/min, 220 nm), R_t (major) = 8.9 min, R_t (minor) = 6.7 min.

(*S*)-2,3-Dimethyl-1-phenylbutan-2-ol (**10b**)

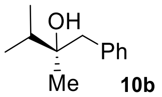

Colorless oil, 16 mg, 90% yield. ^{1}H NMR (400 MHz, $CDCl_3$) δ 7.32–7.29 (m, 2H), 7.26–7.22 (m, 3H), 2.81 (d, *J* = 13.3 Hz, 1H), 2.71 (d, *J* = 13.3 Hz, 1H), 1.77–1.70 (m, 1H), 1.23 (s, 1H), 1.04 (s, 3H), 1.00 (d, *J* = 6.8 Hz, 3H), 0.99 (d, *J* = 6.9 Hz, 3H). ^{13}C NMR (150 MHz, CDCl3) δ 137.8, 130.9, 128.3, 126.5, 74.7, 45.2, 37.2, 22.9, 18.0, 17.3; HRMS (ESI, m/z): calcd. for $C_{12}H_{18}ONa^+$ 201.1250, found 201.1248; IR (KBr thin film, cm^{-1}): ν 3457, 2963, 2928, 2856, 1453, 1263, 751; $[\alpha]_D^{25}$: –36.5 (c 0.1, $CHCl_3$); HPLC analysis: 97% *ee* (Chiralcel OD-H, 0.5:99.5 ^{i}PrOH/Hexane, 1.0 mL/min, 220 nm), R_t (major) = 7.3 min, R_t (minor) = 6.8 min.

(*R*)-(2-(Methoxymethyl)-2-methylpentyl)benzene (**10c**)

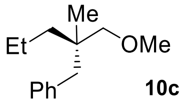

Colorless oil, 15 mg, 73% yield. ^{1}H NMR (600 MHz, $CDCl_3$) δ 7.29–7.24 (m, 2H), 7.22–7.17 (m, 1H), 7.15–7.11 (m, 2H), 3.33 (s, 3H), 2.95 (q, *J* = 8.9 Hz, 2H), 2.63 (d, *J* = 13.0 Hz, 1H), 2.54 (d, *J* = 13.0 Hz, 1H), 1.38–1.24 (m, 3H), 1.23–1.15 (m, 1H), 0.90 (t, *J* = 8.9 Hz, 3H), 0.81 (s, 3H); ^{13}C NMR (150 MHz, $CDCl_3$) δ 139.1, 130.8, 127.8, 125.8, 78.4, 58.9, 43.3, 40.0, 38.2, 21.8, 16.9, 15.0; IR (KBr thin film, cm^{-1}): ν 2956, 2923, 2872, 1450, 1388, 1182, 1114, 989, 730, 703; $[\alpha]_D^{25}$: +5.7 (c 0.8, $CHCl_3$); HPLC analysis: 95% *ee* (Chiralcel OD-H, 0.5:99.5 ^{i}PrOH/Hexane, 0.2 mL/min, 220 nm), R_t (major) = 12.1 min, R_t (minor) = 13.0 min.

(*S*)-2,3-Dimethyl-2-phenylbutan-1-ol (**10d**)

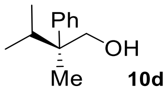

Colorless oil, 12 mg, 67% yield. ^{1}H NMR (400 MHz, CDCl3) δ 7.38–7.33 (m, 4H),

7.25–7.20 (m, 1H), 3.91 (d, J = 10.9 Hz, 1H), 3.62 (dd, J = 10.2 Hz, 7.4 Hz, 1H), 2.12–2.05 (m, 1H), 1.28 (s, 3H), 1.00 (br, 1H), 0.93 (d, J = 6.8 Hz, 3H), 0.63 (d, J = 6.8 Hz, 3H). ^{13}C NMR (101 MHz, $CDCl_3$) δ 145.2, 128.6, 127.2, 126.3, 71.0, 46.6, 34.5, 18.1, 17.5, 15.8; HRMS (ESI, m/z): calcd. for $C_{12}H_{18}ONa^+$ 201.1250, found 201.1248; IR (KBr thin film, cm^{-1}): ν 3446, 2963, 2928, 2856, 1453, 1083, 748; $[\alpha]_D^{25}$: –39.8 (c 0.1, $CHCl_3$); HPLC analysis: 90% *ee* (Chiralcel OD-H, 10:90 ^{i}PrOH/Hexane, 1.0 mL/min, 220 nm), R_t (major) = 3.6 min, R_t (minor) = 4.2 min.

(*S*)-2-Methyl-2,3-diphenylpropyl propionate (**10e**)

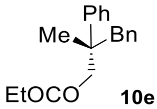

10e

Colorless oil, 19 mg, 67% yield. ^{1}H NMR (600 MHz, $CDCl_3$) δ 7.33–7.29 (m, 2H), 7.28–7.26 (m, 2H), 7.25–7.20 (m, 1H), 7.16–7.10 (m, 3H), 6.82–6.77 (m, 2H), 4.27 (d, J = 11.0 Hz. 1H), 4.21 (d, J = 11.0 Hz, 1H), 3.01 (d, J = 13.3 Hz, 1H), 2.97 (d, J = 13.3 Hz, 1H), 2.31 (q, J = 7.6 Hz, 2H), 1.33 (s, 3H), 1.09 (t, J = 7.6 Hz, 3H); ^{13}C NMR (150 MHz, $CDCl_3$) δ 174.3, 144.3, 137.2, 130.3, 128.0, 127.6, 126.5, 126.2, 126.1, 70.4, 45.5, 42.2, 27.6, 22.4, 9.1; HRMS (ESI, m/z): calcd. for $C_{19}H_{22}O_2Na^+$ 305.1512, found 305.1516; IR (KBr thin film, cm-1): ν 2962, 2965, 2858, 1739, 1260, 1075, 1013, 751; $[\alpha]_D^{25}$: +16.2 (c 1.2, $CHCl_3$); HPLC analysis: 93% *ee* (Chiralcel IC, 1:99 ^{i}PrOH/Hexane, 1.0 mL/min, 220 nm), R_t (major) = 4.3 min, R_t (minor) = 4.6 min.

(*S*)-2-(2-Benzyl-2-vinylpent-4-en-1-yl)naphthalene (**10f**)

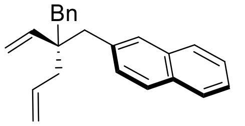

10f

Colorless oil, 28.4 mg, 91% yield. ^{1}H NMR (600 MHz, $CDCl_3$) δ 7.86–7.68 (m, 4H), 7.61 (s, 1H), 7.49–7.39 (m, 3H), 7.32–7.29 (m, 1H), 7.22–7.14 (m, 3H), 6.08–5.98 (m, 1H), 5.85 (dd, J = 17.7, 11.0 Hz, 1H), 5.20 (dd, J = 10.3, 2.2 Hz, 1H), 5.16–5.08 (m, 2H), 4.84 (dd, J = 17.7, 0.7 Hz, 1H), 2.91 (d, J = 13.4 Hz, 1H), 2.88 (d, J = 13.4 Hz, 1H), 2.80 (d, J = 13.4 Hz, 1H), 2.77 (d, J = 13.4 Hz, 1H), 2.15 (dd, J = 7.0, 1.1 Hz, 2H); ^{13}C NMR (150 MHz, $CDCl_3$) δ 144.5, 138.2, 135.9, 135.4, 133.2, 132.1, 131.1, 129.8, 129.5, 127.8, 127.7, 127.6, 126.9, 126.2, 125.8, 125.4, 118.0, 113.5, 44.6, 44.5, 44.3, 38.6; $[\alpha]_D^{25}$: –10.1 (c 1.0, $CHCl_3$); HPLC analysis: 91% *ee* (Chiralcel OJ-H, 20:80 ^{i}PrOH/Hexane, 0.2 mL/min, 220 nm), R_t (major) = 19.0 min, R_t (minor) = 23.9 min.

(*S*)-2-Benzyl-2-(4-fluorobenzyl)butyl propionate (**10g**)

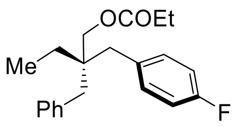

10g

Colorless oil, 23 mg, 70% yield. ^{1}H NMR (600 MHz, $CDCl_3$) δ 7.29–7.24 (m, 3H), 7.23–7.18 (m, 1H), 7.14–7.09 (m, 3H), 7.08–7.04 (m, 1H), 6.97–6.92 (m, 1H), 3.72–3.66 (m, 2H), 2.79–2.70 (m, 2H), 2.69–2.60 (m, 2H), 2.38 (q, J = 7.4 Hz, 2H), 1.27–1.22 (m, 2H), 1.19 (t, J = 7.6 Hz, 3H), 1.05 (t, J = 7.4 Hz. 3H) ; ^{13}C NMR (150 MHz, $CDCl_3$) δ 173.0, 161.4 (d, J = 242.9 Hz), 136.6 (d, J = 27.3 Hz), 132.3, 130.6 (d, J = 8.0 Hz), 129.3 (d, J = 4.5 Hz), 126.9 (d, J = 6.4 Hz), 125.1 (d, J = 11.0 Hz), 113.7 (d, J = 20.5 Hz), 66.3, 39.9 (d, J = 12.6 Hz), 39.3 (d, J = 12.6 Hz), 38.6, 26.6, 23.6 (d, J = 11.1 Hz), 8.1, 6.8; HRMS (ESI, m/z): calcd. for $C_{21}H_{25}O_2FNa^+$ 351.1731, found 351.1735; IR (KBr thin film, cm^{-1}): ν 2962, 2923, 2858, 1742, 1510, 1456, 1263, 1188, 1087, 1019, 802, 751, 698; $[\alpha]_D^{25}$: −38.8 (c 1.0, $CHCl_3$); HPLC analysis: 93% *ee* (Chiralcel OJ-H, 0.5:99.5 ^{i}PrOH/Hexane, 0.2 mL/min, 220 nm), R_t (major) = 29.4 min, R_t (minor) =34.6 min.

(*S*)-Methyl 2-benzyl-2-(naphthalen-2-ylmethyl)pent-4-enoate (**10h**)

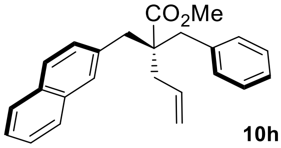

10h

Colorless oil, 34 mg, 99% yield. ^{1}H NMR (600 MHz, $CDCl_3$) δ 7.84–7.78 (m, 1H), 7.77–7.72 (m, 2H), 7.60 (s, 1H), 7.48–7.40 (m, 3H), 7.29–7.26 (m, 2H), 7.25–7.20 (m, 1H), 7.18–7.13 (m, 2H), 6.09–5.99 (m, 1H), 5.28 (dd, J = 10.3, 1.7 Hz, 1H), 5.19 (dd, J = 17.1, 1.7 Hz, 1H), 3.63 (s, 3H), 3.27 (d, J = 13.8 Hz, 1H), 3.16 (d, J = 13.8 Hz, 1H), 3.06 (d, J = 13.8 Hz, 1H), 2.95 (d, J = 13.8 Hz, 1H), 2.34 (d, J = 7.0 Hz, 2H); ^{13}C NMR (150 MHz, $CDCl_3$) δ 175.9, 137.4, 135.0, 134.2, 133.4, 132.3, 130.2, 128.9, 128.5, 128.2, 127.7, 127.6, 126.7, 126.0, 125.6, 119.2, 52.3, 51.5, 42.2, 35.9; HRMS (ESI, m/z): calcd. for $C_{24}H_{24}O_2Na^+$ 367.1669, found 367.1674; IR (KBr thin film, cm^{-1}): ν 2962, 2923, 2858, 1739, 1260, 1090, 1019, 805, 745, 701; $[\alpha]_D^{25}$: −29.7 (c 1.3, $CHCl_3$); HPLC analysis: 90% *ee* (Chiralcel IA, 1:99 ^{i}PrOH/Hexane, 0.5 mL/min, 220 nm), R_t (major) = 8.4 min, R_t (minor) = 9.8 min.

(1*S*,2*R*,3*S*)-3-Azido-3-pentyl-1-phenylnonane-1,2-diol (**11a**)

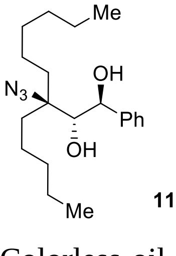

11a

Colorless oil, 21 mg, 60% yield. ^{1}H NMR (600 MHz, $CDCl_3$) δ 7.46–7.42 (m, 2H), 7.41–7.37 (m, 2H), 7.36–7.31 (m, 1H), 4.79 (dd, J = 7.2, 2.5 Hz, 1H), 3.73 (dd, J = 7.2, 4.0 Hz, 1H), 2.94–2.92 (m, 1H), 1.90–1.75 (m, 3H), 1.70–1.61 (m, 1H), 1.54–1.50 (m, 1H), 1.44–1.26 (m, 14H), 0.94–0.86 (m, 6H); ^{13}C NMR (150 MHz, $CDCl_3$) δ 141.2, 128.8, 128.6, 127.7, 75.1, 68.8, 34.1, 33.7, 32.3, 31.8, 29.9, 23.7, 23.4, 23.1, 22.7, 22.6, 14.1; HRMS (ESI, m/z): calcd. for $C_{20}H_{33}N_3O_2Na^+$ 370.2465, found 370.2469; IR (KBr thin film, cm^{-1}): ν 2969, 2926, 2863, 2105, 1459,1266, 1022,

748, 698; $[\alpha]_D^{25}$: –21.1 (c 1.1, $CHCl_3$); HPLC analysis: 96% *ee* (Chiralcel IC, 1:99 ^{i}PrOH/Hexane, 1.0 mL/min, 220 nm), R_t (major) = 9.0 min, R_t (minor) = 6.5 min.

(*R*)-Ethyl 2-((*R*)-amino(naphthalen-2-yl)methyl)-2-methylpentanoate (**11b**)

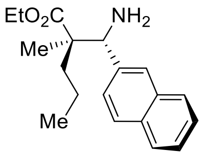

11b

Colorless oil 17.5 mg, 58% yield. ^{1}H NMR (600 MHz, $CDCl_3$) δ 7.85–7.81 (m, 2H), 7.80–7.77 (m, 1H), 7.74–7.72 (m, 1H), 7.51–7.44 (m, 2H), 7.44–7.40 (m, 1H), 4.39 (s, 1H), 4.21 (q, *J* = 7.1 Hz, 2H), 1.99 (s, 2H), 1.82–1.74 (m, 1H), 1.32–1.24 (m, 4H), 1.18–1.08 (m, 5H), 0.83 (t, *J* = 7.1 Hz, 3H); ^{13}C NMR (150 MHz, $CDCl_3$) δ 176.8, 139.6, 133.0, 132.9, 128.0, 127.6, 127.4, 127.3, 126.5, 126.1, 125.9, 62.3, 60.7, 52.0, 40.5, 18.1, 15.8, 14.7, 14.4; HRMS (ESI, m/z): calcd. for $C_{19}H_{26}O_2N$ 370.2465, found 370.2469; IR (KBr thin film, cm^{-1}): ν 2958, 2935, 2872, 1724, 1468, 1379, 1218, 1135, 1016, 855, 817, 742; $[\alpha]_D^{25}$: –13.3 (c 1.4, $CHCl_3$); HPLC analysis: 99% *ee* (Chiralcel AD-H, 20:80 ^{i}PrOH/Hexane, 1.0 mL/min, 220 nm), R_t (major) = 3.9 min, R_t (minor) = 5.6 min.

(1*R*,3*S*)-1,3-Diphenyl-1-(*o*-tolyl)-1,3-dihydroisobenzofuran (**11c**)

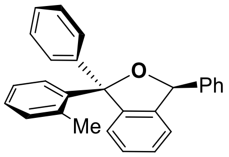

11c

Colorless oil, 17 mg, 94% yield. ^{1}H NMR (400 MHz, $CDCl_3$) δ 7.47–7.37 (m, 3H), 7.32–7.26 (m, 4H), 7.24–7.18 (m, 7H), 7.16–7.08 (m, 4H), 6.48 (s, 1H), 2.06 (s, 3H); ^{13}C NMR (150 MHz, $CDCl_3$) δ 147.2, 143.5, 142.4, 142.1, 141.9, 138.5, 132.6, 128.5, 128.4, 128.1, 127.9, 127.9, 127.7, 127.5, 126.9, 126.9, 125.7, 125.1, 124.8, 122.6, 94.6, 86.3, 22.0; HRMS (ESI, m/z): calcd. for $C_{27}H_{22}ONa^+$ 385.1563, found 385.1568; IR (KBr thin film, cm^{-1}): ν 3050, 2381, 1387, 1259, 1057, 792, 706; $[\alpha]_D^{25}$: –18.1 (c 0.1, $CHCl_3$); HPLC analysis: 92% *ee* (Chiralcel IA, 0.5:99.5 ^{i}PrOH/Hexane, 1.0 mL/min, 220 nm), R_t (major) = 3.4 min, R_t (minor) = 3.0 min.

Methyl(1*S*,2*R*)-2-allyl-1-(4-chlorophenyl)-2,3-dihydro-1*H*-cyclopenta[*a*]naphthalene-2-carboxylate (**11d**)

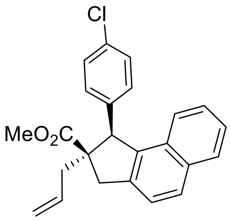

11d

Colorless oil, 34 mg, 90% yield. ^{1}H NMR (400 MHz, $CDCl_3$) δ 7.86–7.79 (m, 2H), 7.47–7.30 (m, 5H), 7.13 (d, *J* = 8.4 Hz, 2H), 6.93 (d, *J* = 8.1 Hz, 2H), 5.75–5.65 (m, 1H), 5.08–5.01 (m, 2H), 4.63 (s, 1H), 3.93 (d, *J* = 16.8 Hz, 1H), 3.32 (s, 3H), 3.12 (d,

J = 16.8 Hz, 1H), 2.90 (dd, J = 13.6, 6.0 Hz, 1H), 2.40 (dd, J = 13.6, 6.0 Hz, 1H); ^{13}C NMR (150 MHz, $CDCl_3$) δ 174.0, 139.0, 138.1, 133.8, 133.3, 132.7, 130.0, 129.9, 128.8, 128.7, 128.3, 126.5, 125.1, 123.9, 123.4, 119.0, 60.3, 51.6, 43.6, 38.1; IR (KBr thin film, cm^{-1}) ν 3457, 2368, 2323, 1733, 1636, 1273, 1211, 1017, 806, 748; $[\alpha]_D^{25}$: –7.4 (c 0.1, $CHCl_3$); HPLC analysis: 93% *ee* (Chiralcel AD-H, 0.5:99.5 ^{i}PrOH/Hexane, 1.0 mL/min, 220 nm), R_t (major) = 6.9 min, R_t (minor) = 8.8 min.

(2*S*,3*R*)-2-(4-Methoxyphenyl)-3-methyl-2-(*p*-tolyl)oxirane (**11e**)

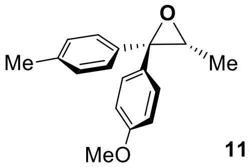

11e

Colorless oil, 16 mg, 63% yield. ^{1}H NMR (400 MHz, $CDCl_3$) δ 7.29–7.24 (m, 3H), 7.23–7.15 (m, 3H), 6.85–6.79 (m, 2H), 3.77 (s, 3H), 3.49 (q, J = 5.4 Hz, 1H), 2.36 (s, 3H), 1.17 (d, J = 5.4 Hz, 3H); ^{13}C NMR (100 MHz, $CDCl_3$) δ 159.1, 137.1, 134.7, 133.6, 128.8, 128.2, 128.0, 113.6, 65.8, 62.3, 55.3, 21.2, 15.5; IR (KBr thin film, cm^{-1}): ν 2959, 2926, 2858, 1507, 1269, 748; $[\alpha]_D^{25}$: −22.3 (c 1.2, $CHCl_3$); HPLC analysis: 96% *ee* (Chiralcel AD-H, 10:90 ^{i}PrOH/Hexane, 1.0 mL/min, 220 nm), R_t (major) = 3.4 min, R_t (minor) = 3.1 min.

(*S*)-2-Methyl-1,2-diphenylpent-4-en-1-one (**11f**)

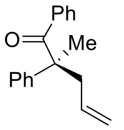

11f

Colorless oil, 12 mg, 96% yield. ^{1}H NMR (400 MHz, $CDCl_3$) δ 7.46–7.44 (m, 2H), 7.38–7.34 (m, 3H), 7.31–7.26 (m, 3H), 7.23–7.20 (m, 2H), 5.55–5.48 (m, 1H), 4.99–4.91 (m, 2H), 2.84 (dd, J = 9.2, 5.2 Hz, 1H), 2.76 (dd, J =9.2, 4.6 Hz, 1H), 1.58 (s, 3H); ^{13}C NMR (100 MHz, $CDCl_3$) δ 203.2, 143.6, 136.8, 134.1, 131.7, 129.6, 129.0, 128.0, 127.0, 126.3, 118.4, 54.4, 44.8, 23.7; HRMS (ESI, m/z): calcd. for $C_{18}H_{18}ONa^+$ 273.1250, found 273.1254; IR (KBr thin film, cm^{-1}): ν 3065, 2980 , 2920, 1675, 1597, 1521, 1376, 1161, 1138, 970,671, 696; $[\alpha]_D^{25}$: +140.0 (c 0.6, $CHCl_3$); HPLC analysis: 99% *ee* (Chiralcel AD-H, 2:98 ^{i}PrOH/Hexane, 1.0 mL/min, 220 nm), R_t (major) = 3.1 min, R_t (minor) = 4.0 min.

VII. ^{1}H NMR and ^{13}C NMR spectra of substrates and products

Supplementary Figure 24. ^{1}H NMR sepctra of (1*S*,2*S*)-1-(4-Bromophenyl)-1-(4-chlorophenyl)propane-1,2-diol (**1a**)

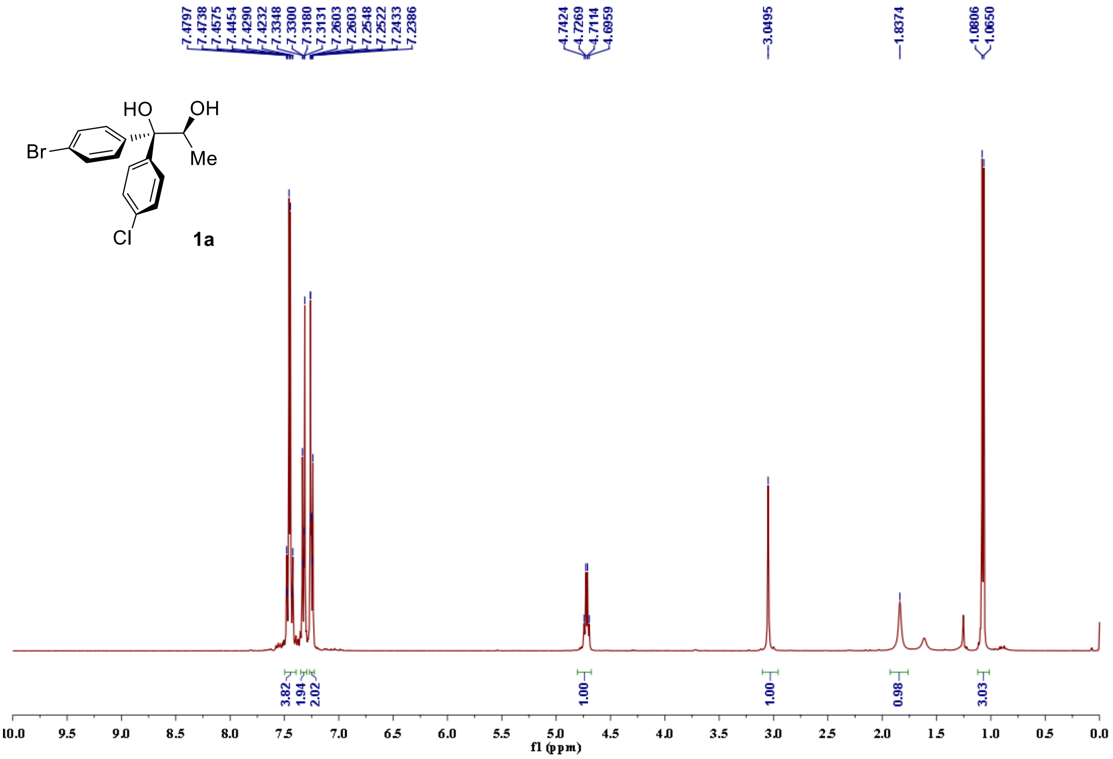

Supplementary Figure 25. ^{13}C NMR sepctra of (1*S*,2*S*)-1-(4-Bromophenyl)-1-(4-chlorophenyl)propane-1,2-diol (**1a**)

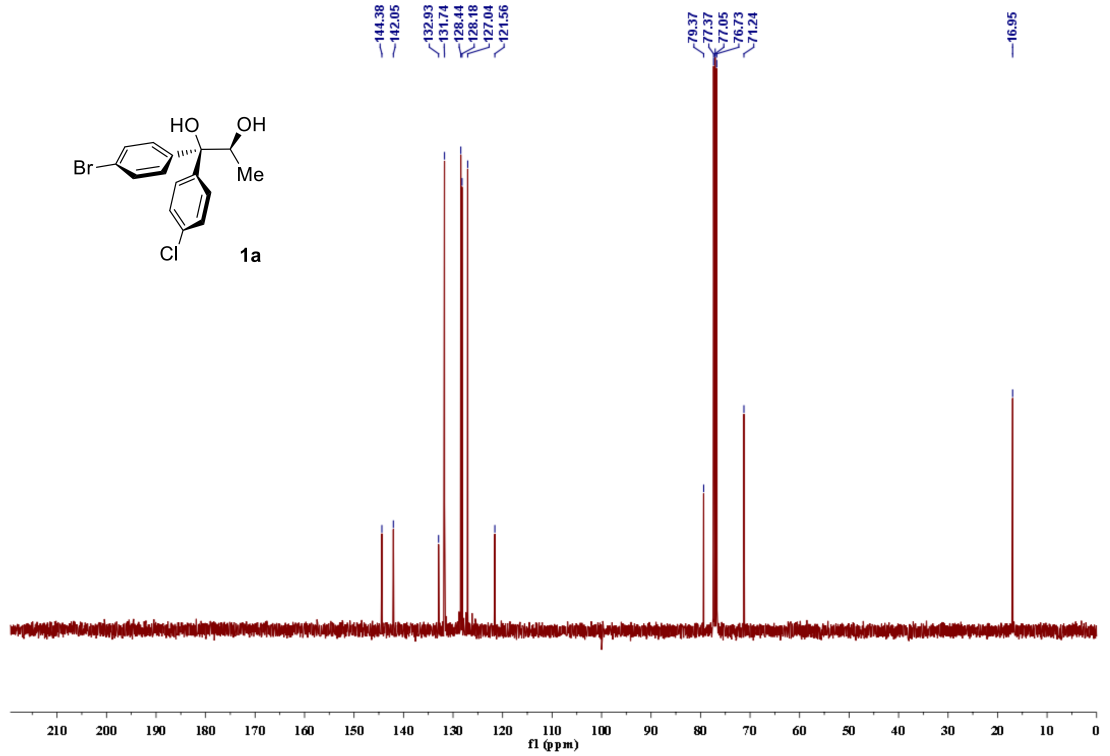

Supplementary Figure 26. ^{1}H NMR sepctra of (1*R*,2*R*)-1-(4-Bromophenyl)-1-(4-chlorophenyl)-1-hydroxypropan-2-yl propionate (**2a**)

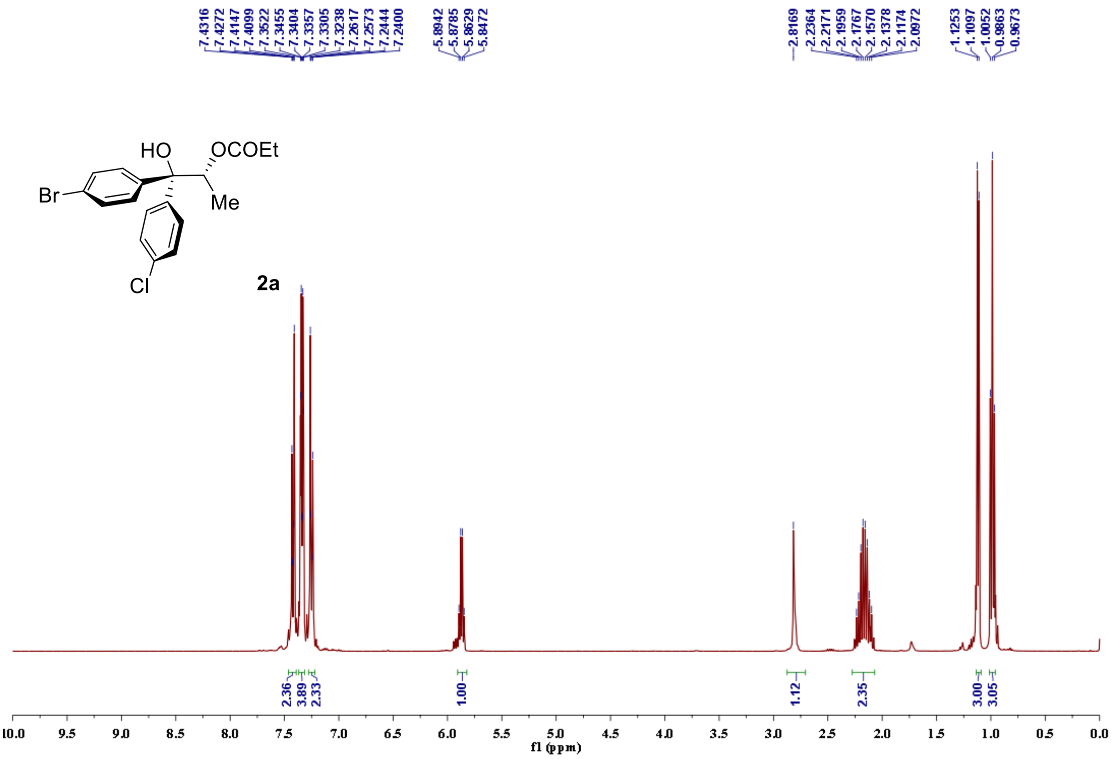

Supplementary Figure 27. ^{13}C NMR sepctra of (1*R*,2*R*)-1-(4-Bromophenyl)-1-(4-chlorophenyl)-1-hydroxypropan-2-yl propionate (**2a**)

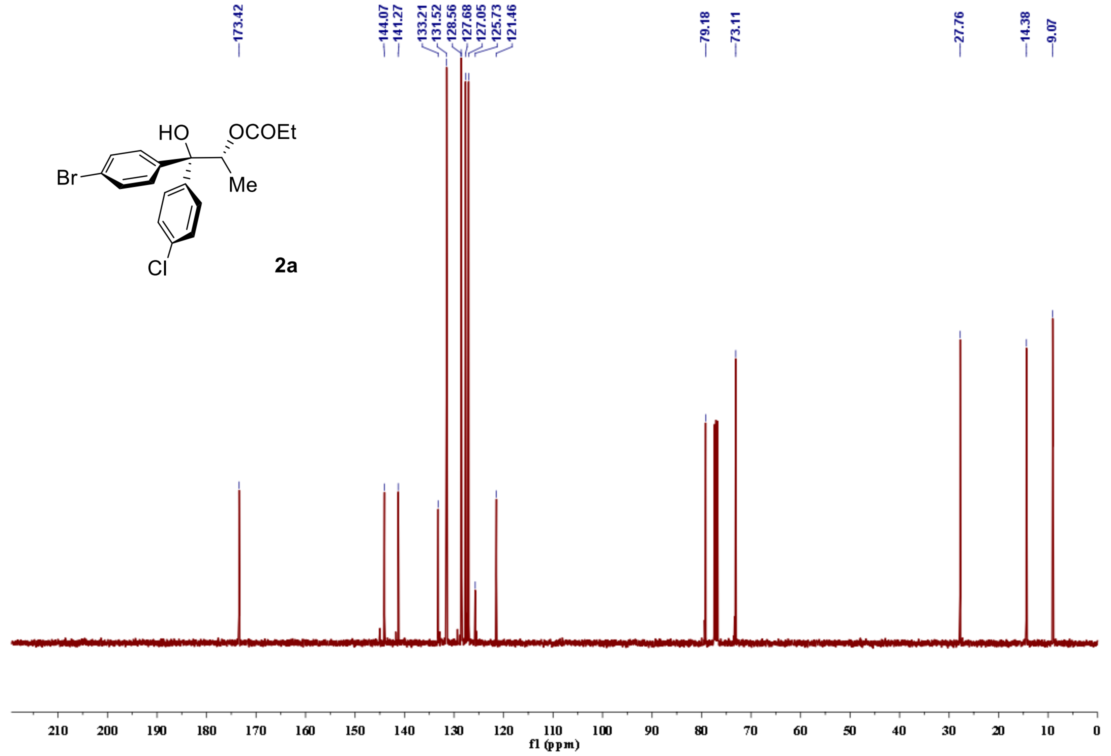

Supplementary Figure 28. ^{1}H NMR sepctra of (1*R*,2*S*)-1-(4-Methoxyphenyl)-1-(*p*-tolyl)propane-1,2-diol (**1b**)

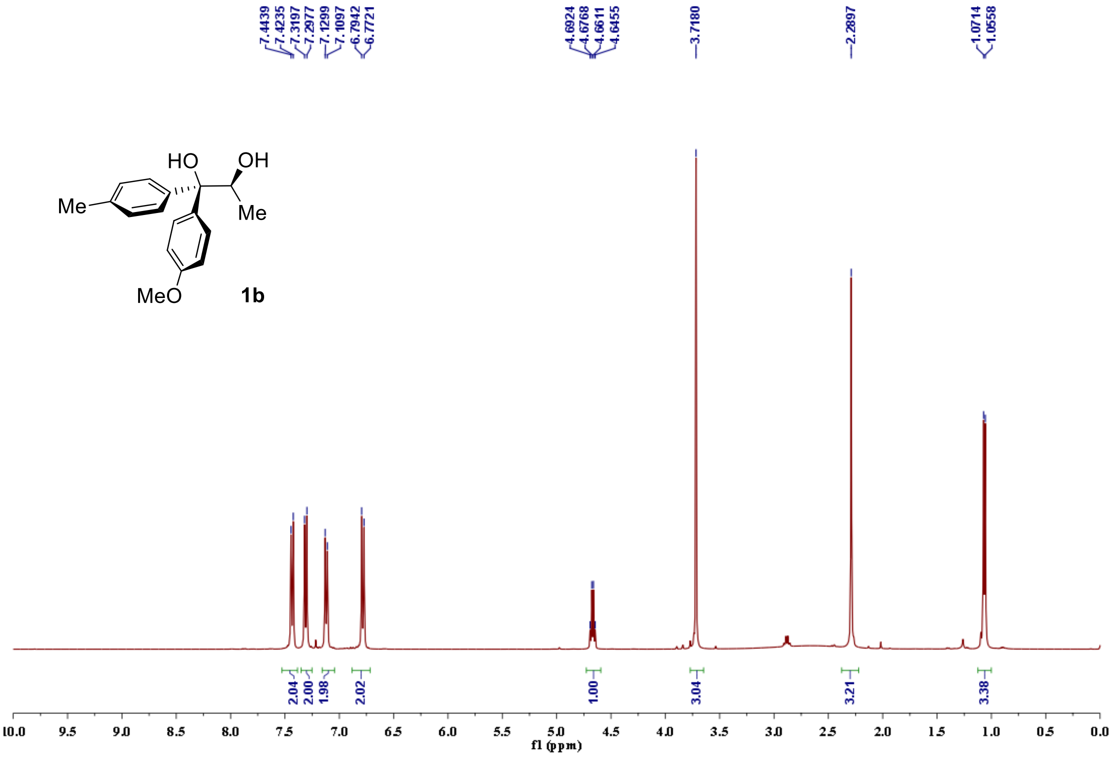

Supplementary Figure 29. ^{13}C NMR sepctra of (1*R*,2*S*)-1-(4-Methoxyphenyl)-1-(*p*-tolyl)propane-1,2-diol (**1b**)

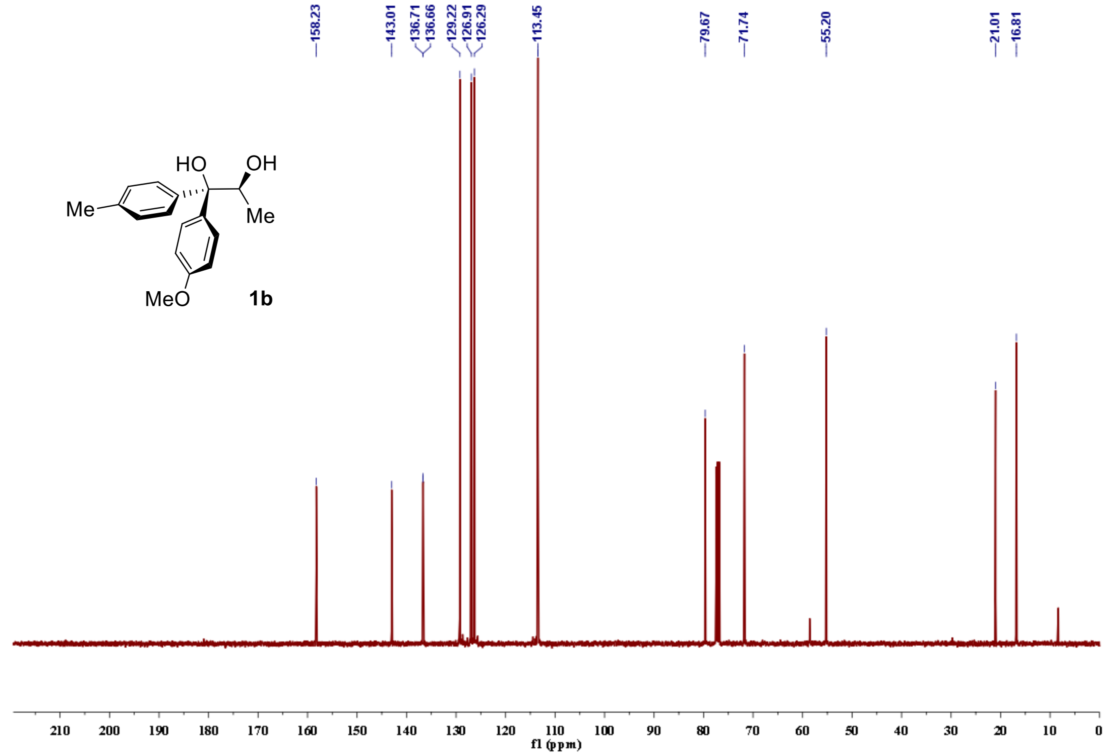

Supplementary Figure 30. ^{1}H NMR sepctra of (1*S*,2*R*)-1-Hydroxy-1-(4-methoxyphenyl)-1-(*p*-tolyl)propan-2-yl propionate (**2b**)

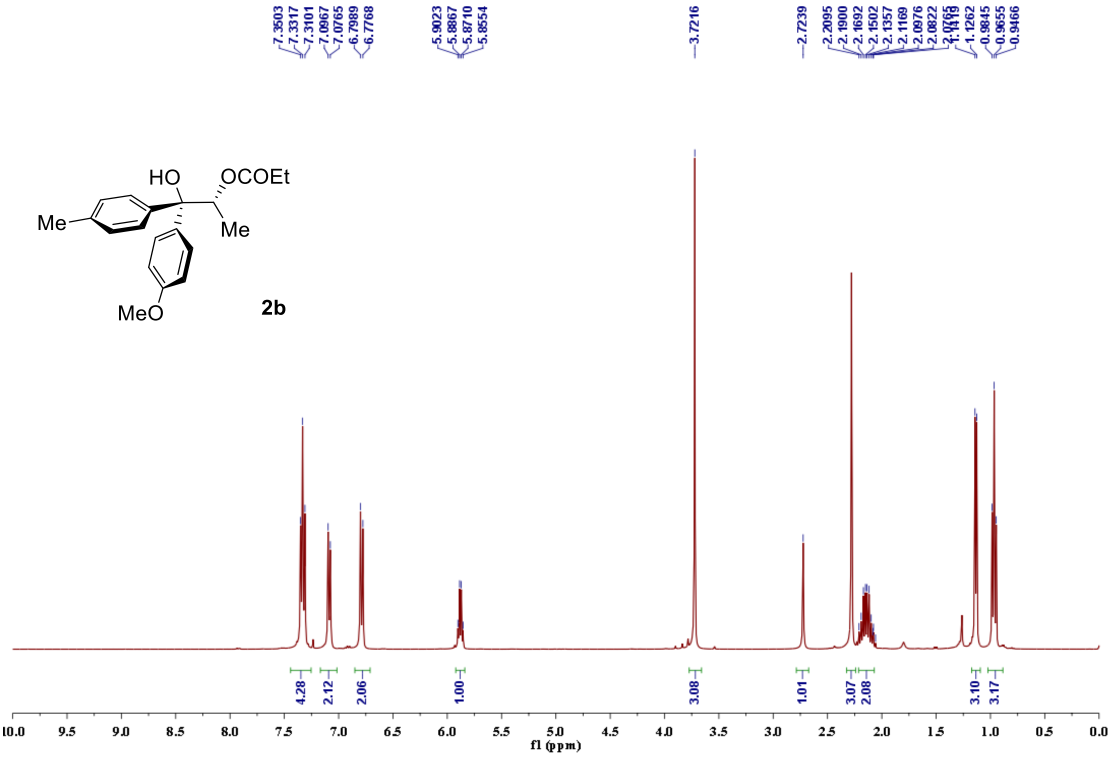

Supplementary Figure 31. ^{13}C NMR sepctra of (1*S*,2*R*)-1-Hydroxy-1-(4-methoxyphenyl)-1-(*p*-tolyl)propan-2-yl propionate (**2b**)

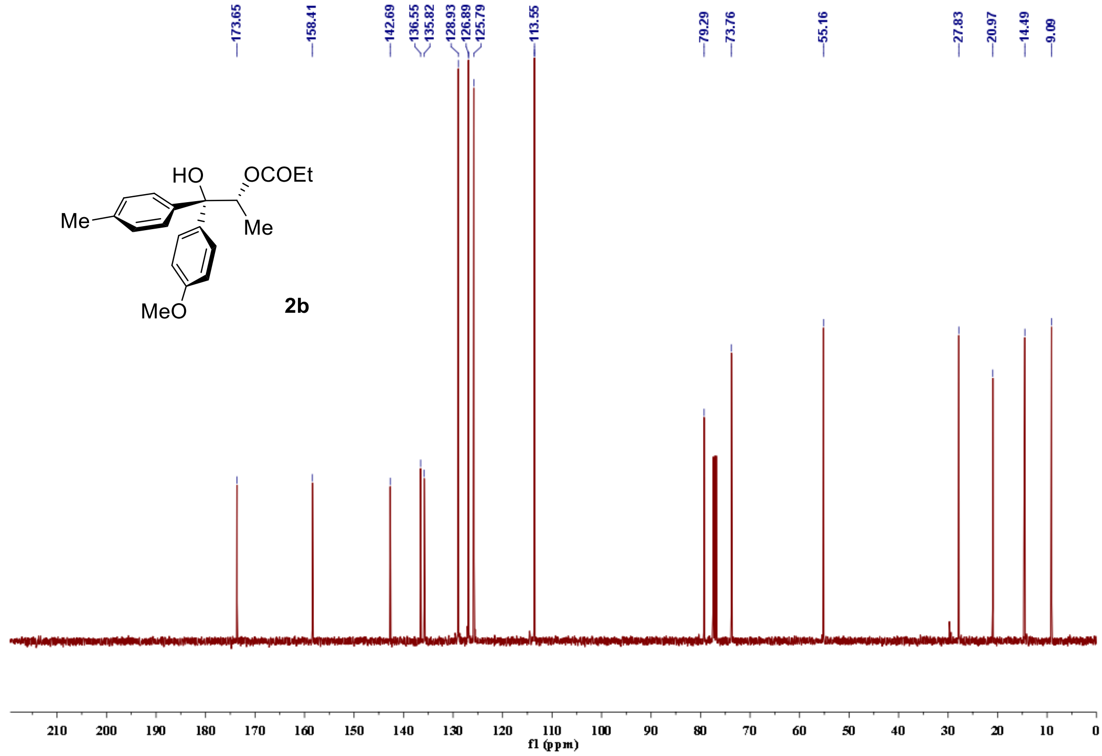

Supplementary Figure 32. ^{1}H NMR sepctra of (1*S*,2*S*)-1-(4-Bromophenyl)-1-(4-methoxyphenyl)propane-1,2-diol (**1c**)

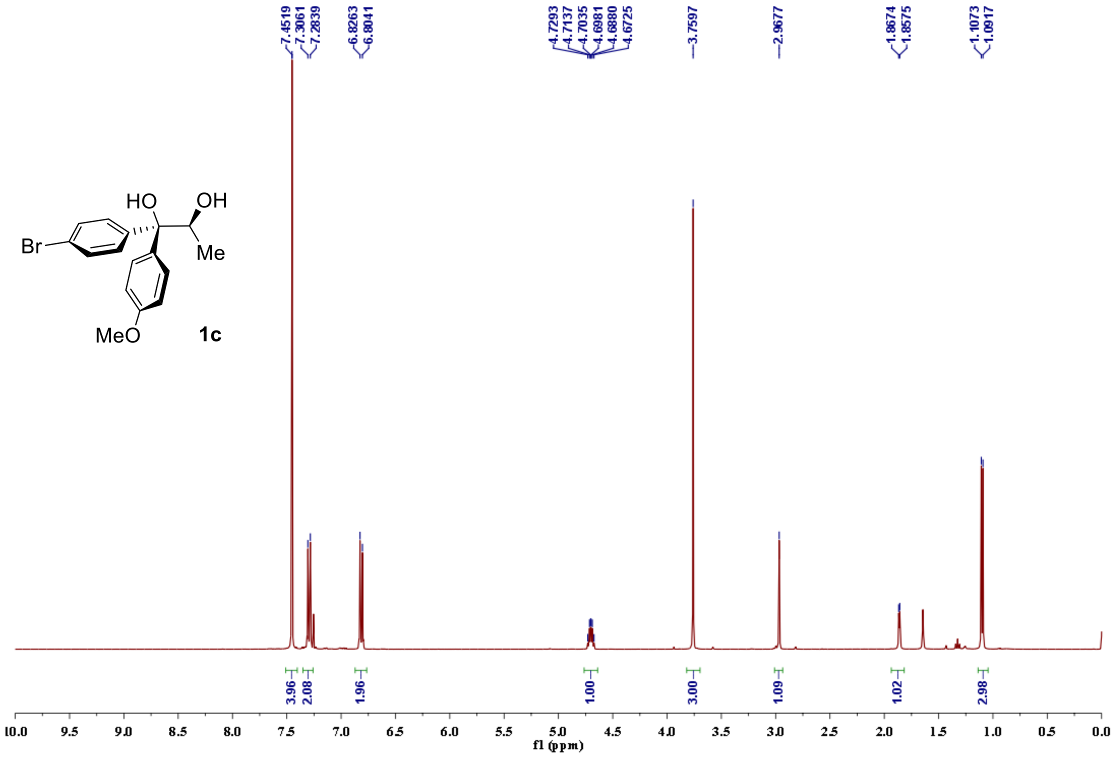

Supplementary Figure 33. ^{13}C NMR sepctra of (1*S*,2*S*)-1-(4-Bromophenyl)-1-(4-methoxyphenyl)propane-1,2-diol (**1c**)

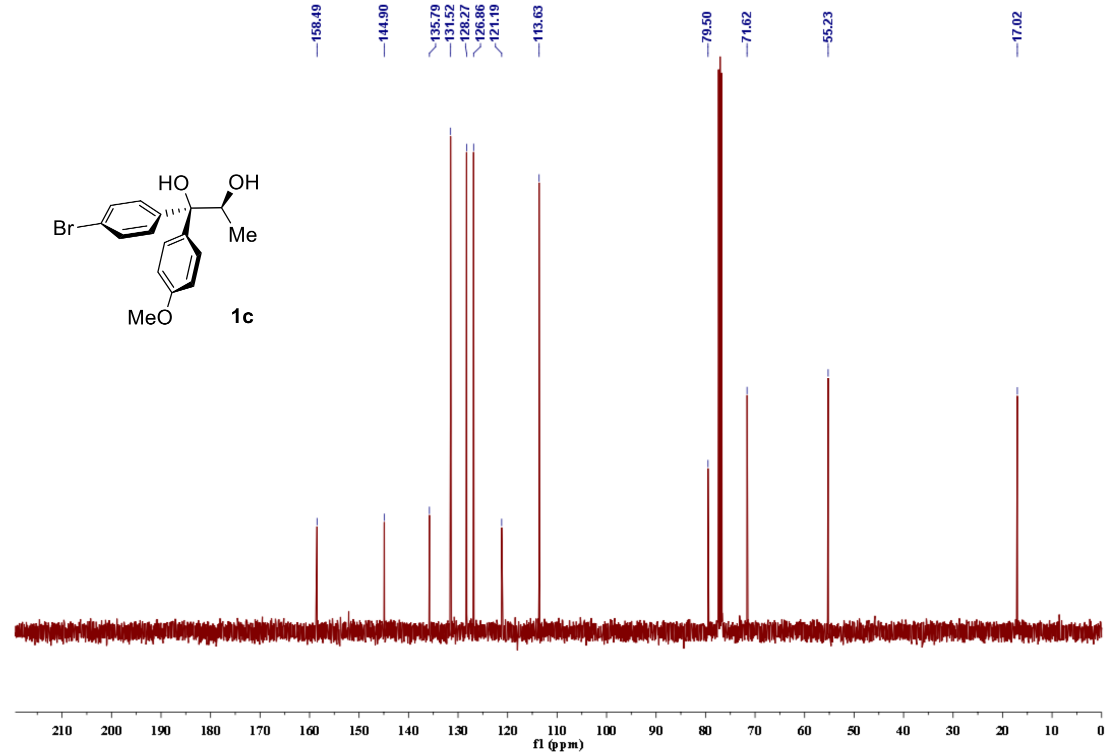

Supplementary Figure 34. ^{1}H NMR sepctra of (1*R*,2*R*)-1-(4-Bromophenyl)-1-hydroxy-1-(4-methoxyphenyl)propan-2-yl propionate (**2c**)

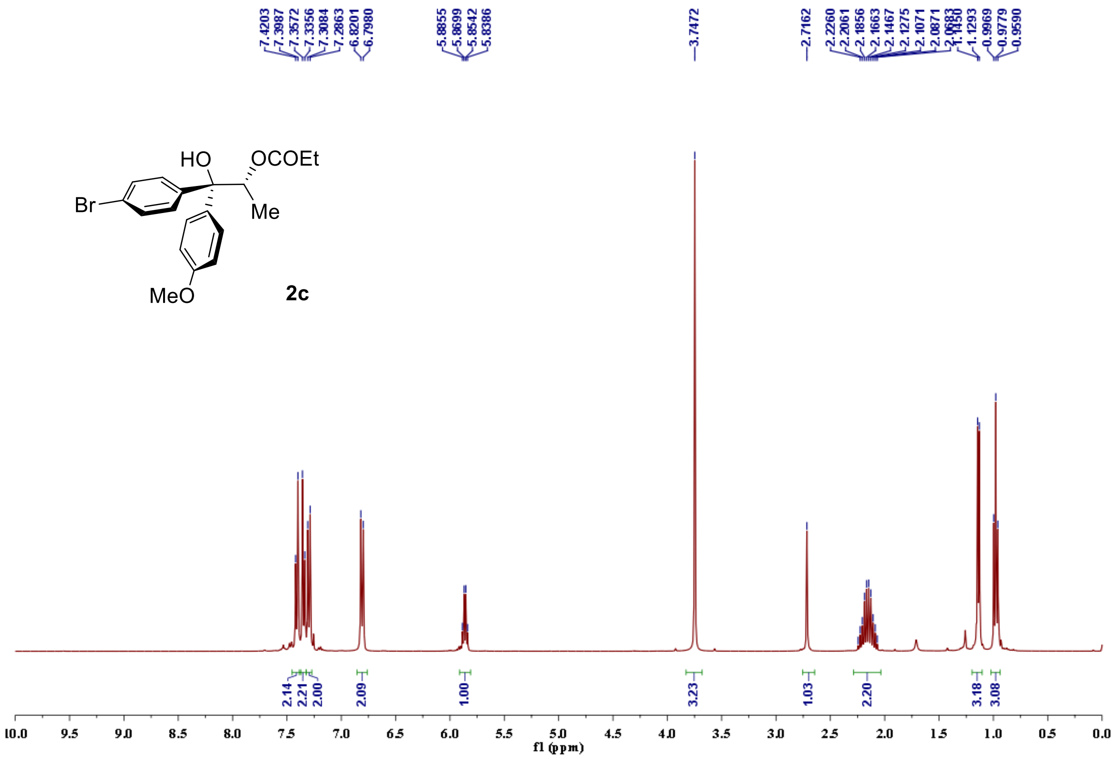

Supplementary Figure 35. ^{13}C NMR sepctra of (1*R*,2*R*)-1-(4-Bromophenyl)-1-hydroxy-1-(4-methoxyphenyl)propan-2-yl propionate (**2c**)

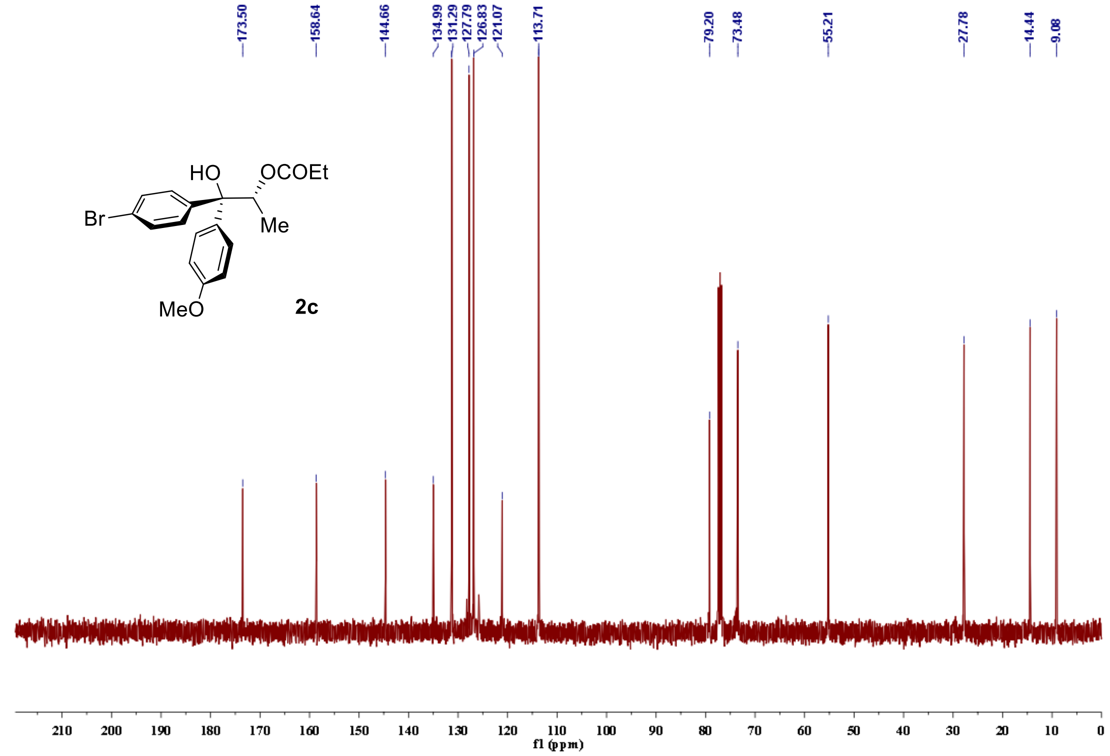

Supplementary Figure 36. ^{1}H NMR sepctra of (1***S***,2***S***)-1-Phenyl-1-(thiophen-2-yl)propane-1,2-diol (**1d**)

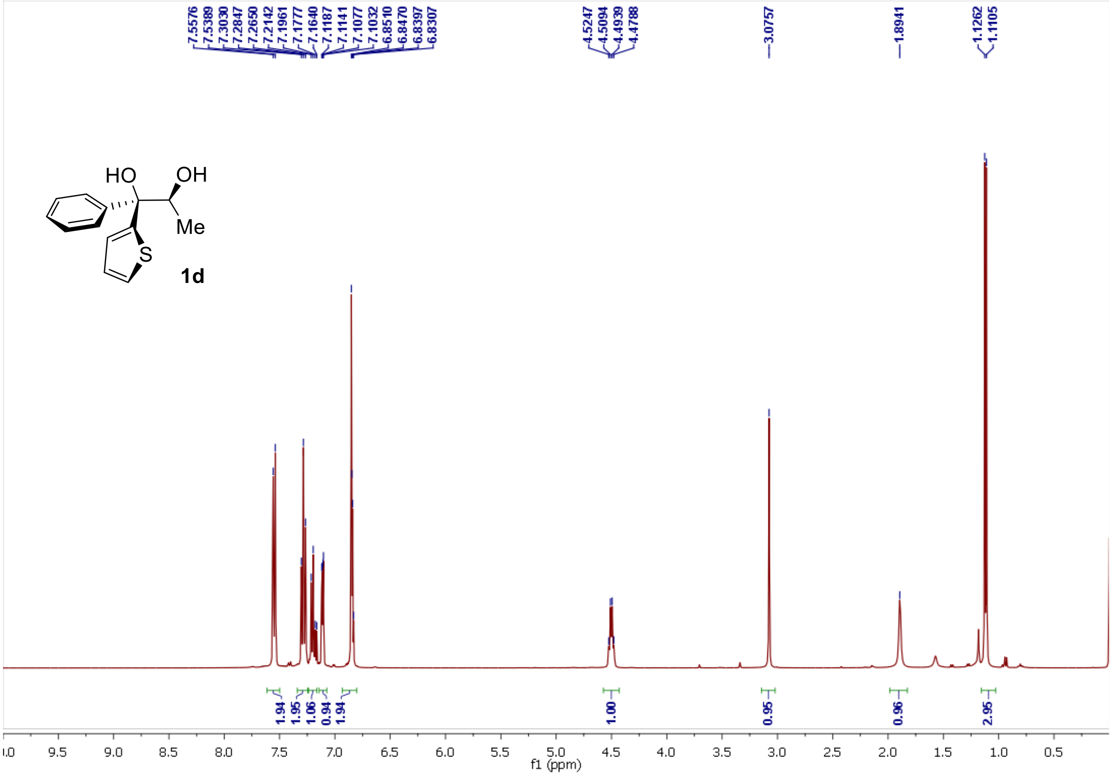

Supplementary Figure 37. ^{13}C NMR sepctra of (1***S***,2***S***)-1-Phenyl-1-(thiophen-2-yl)propane-1,2-diol (**1d**)

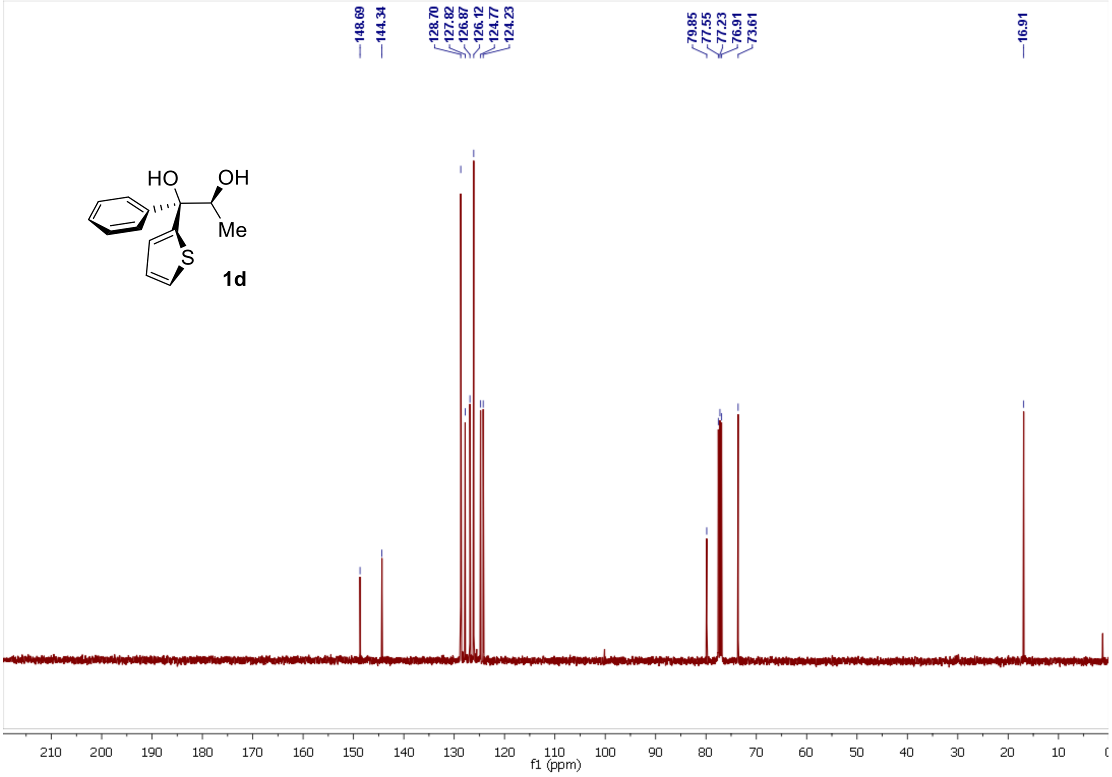

Supplementary Figure 38. ^{1}H NMR sepctra of (1*R*,2*R*)-1-Hydroxy-1-phenyl-1-(thiophen-2-yl)propan-2-yl propionate (**2d**)

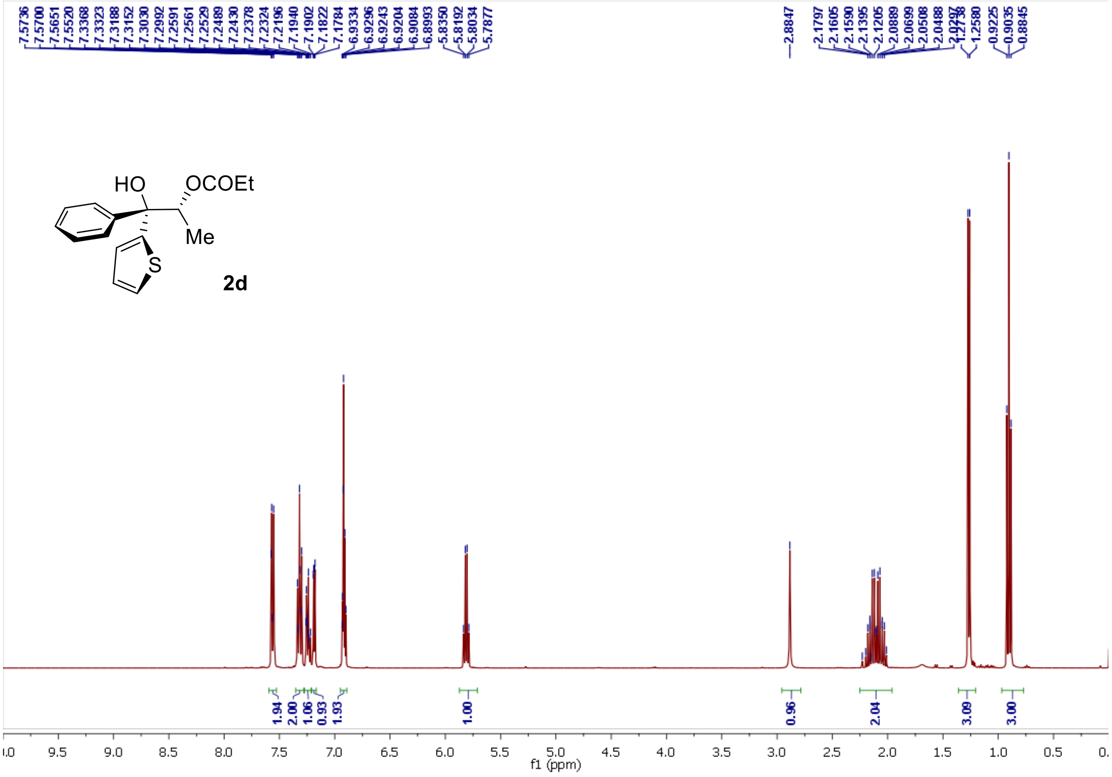

Supplementary Figure 39. ^{13}C NMR sepctra of (1*R*,2*R*)-1-Hydroxy-1-phenyl-1-(thiophen-2-yl)propan-2-yl propionate (**2d**)

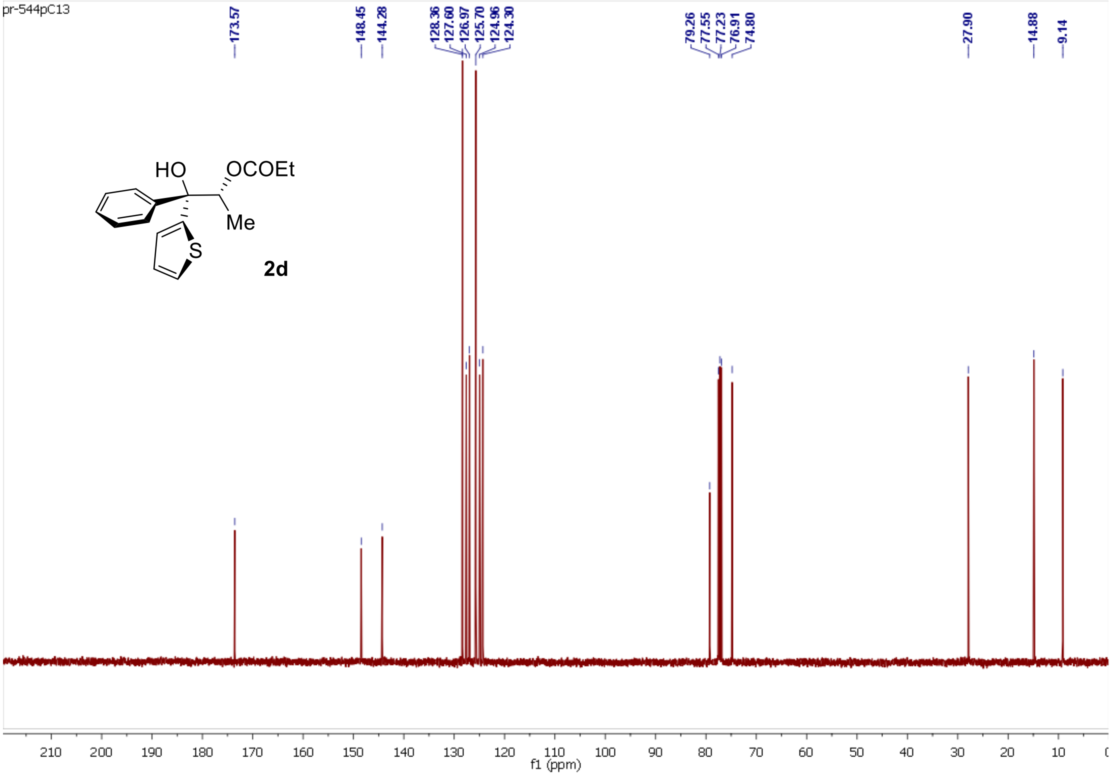

Supplementary Figure 40. ^1H NMR sepctra of (1*S*,2*S*)-1-(4-Methoxyphenyl)-1-(thiophen-2-yl)propane-1,2-diol (**1e**)

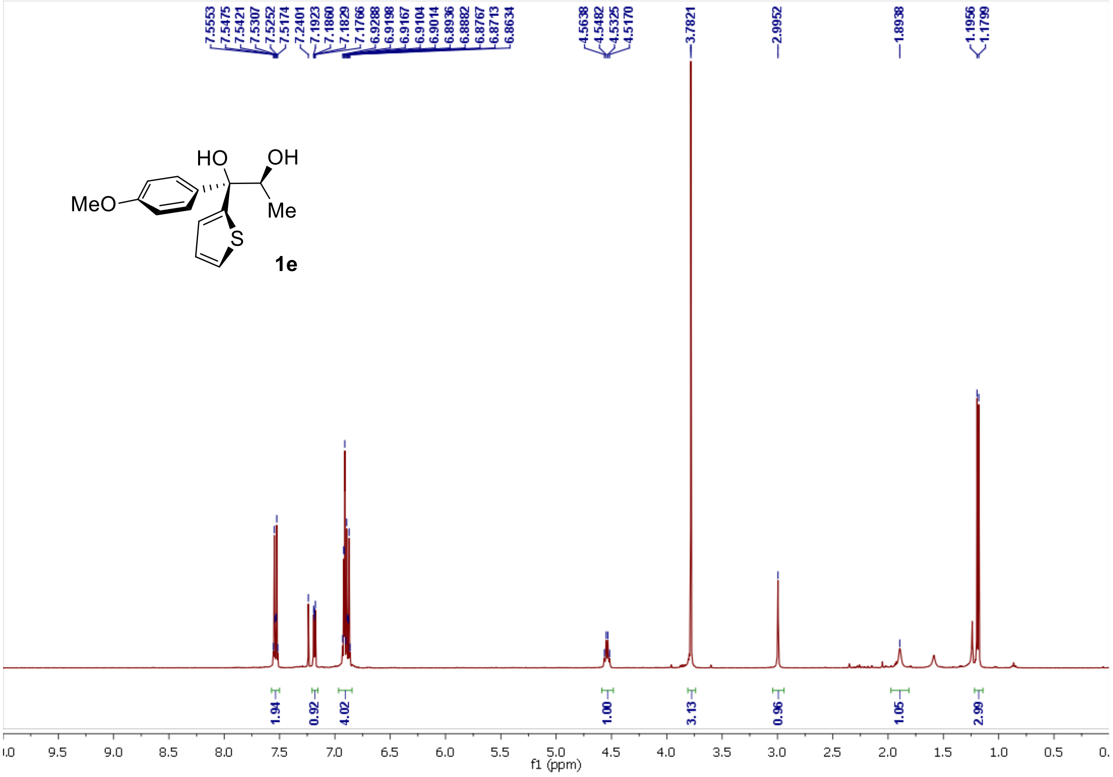

Supplementary Figure 41. ^{13}C NMR sepctra of (1*S*,2*S*)-1-(4-Methoxyphenyl)-1-(thiophen-2-yl)propane-1,2-diol (**1e**)

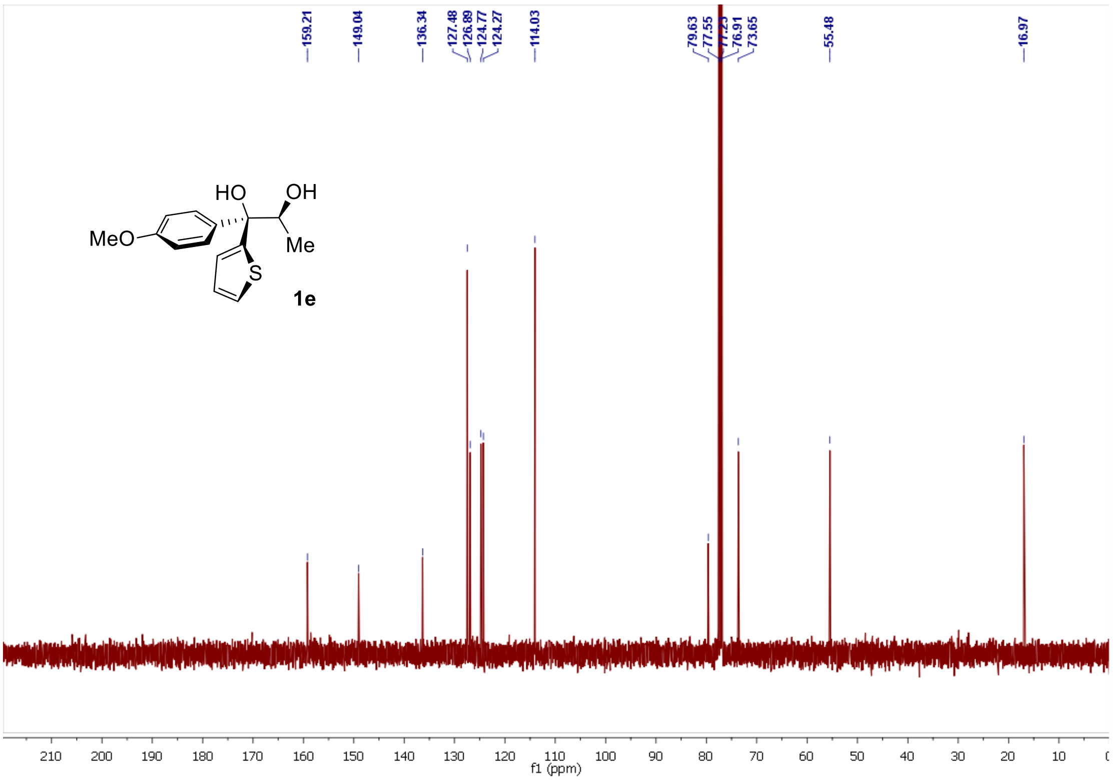

Supplementary Figure 42. ^{1}H NMR sepctra of (1*R*,2*R*)-1-Hydroxy-1-(4-methoxyphenyl)-1-(thiophen-2-yl)propan-2-yl propionate (**2e**)

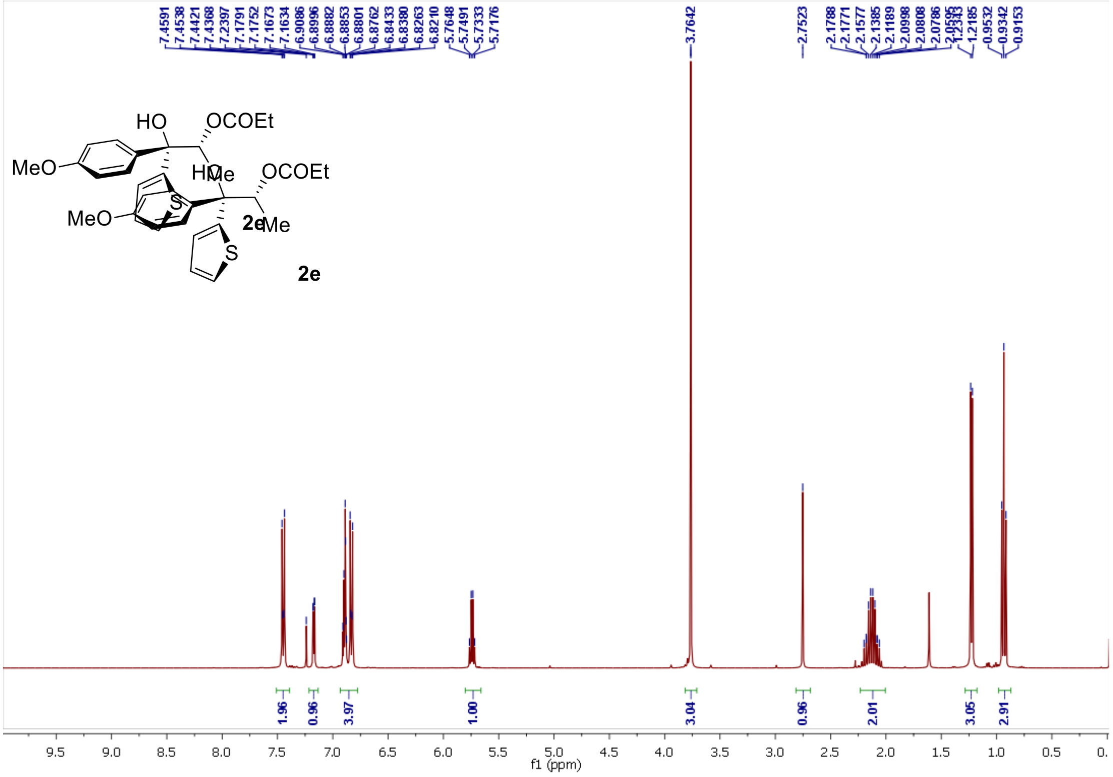

Supplementary Figure 43. ^{13}C NMR sepctra of (1*R*,2*R*)-1-Hydroxy-1-(4-methoxyphenyl)-1-(thiophen-2-yl)propan-2-yl propionate (**2e**)

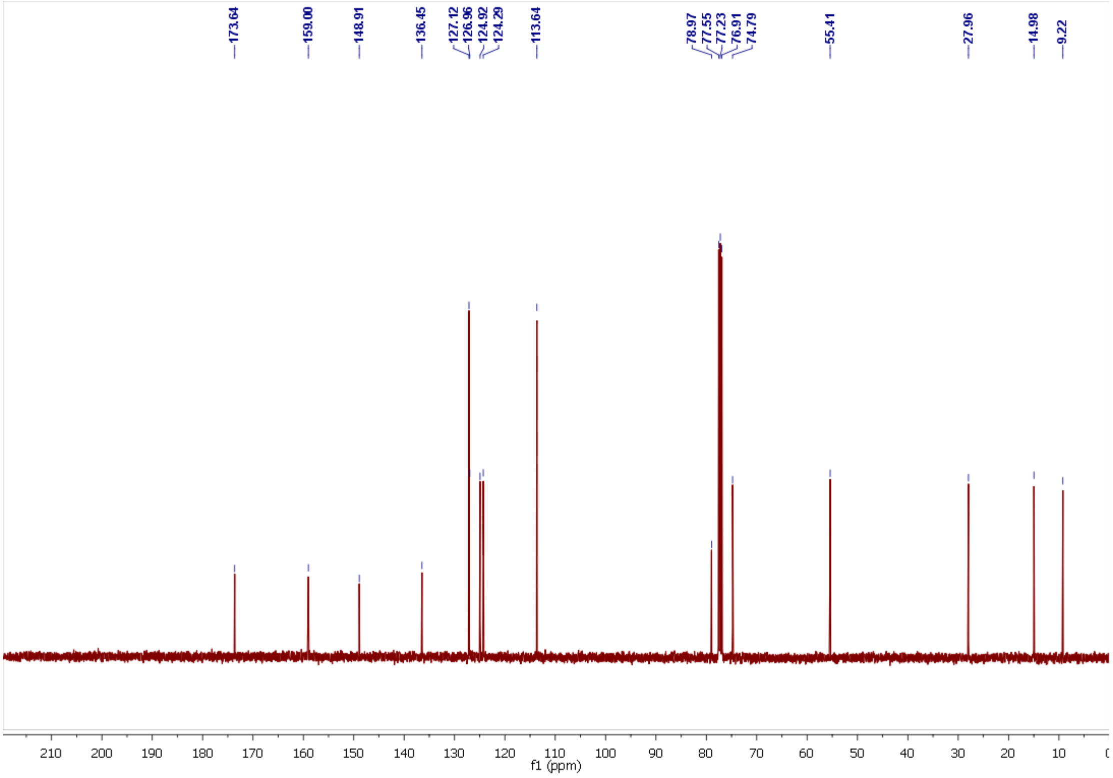

Supplementary Figure 44. ^{1}H NMR sepctra of (1*S*,2*S*)-1-(Furan-2-yl)-1-(thiophen-2-yl)propane-1,2-diol (**1f**)

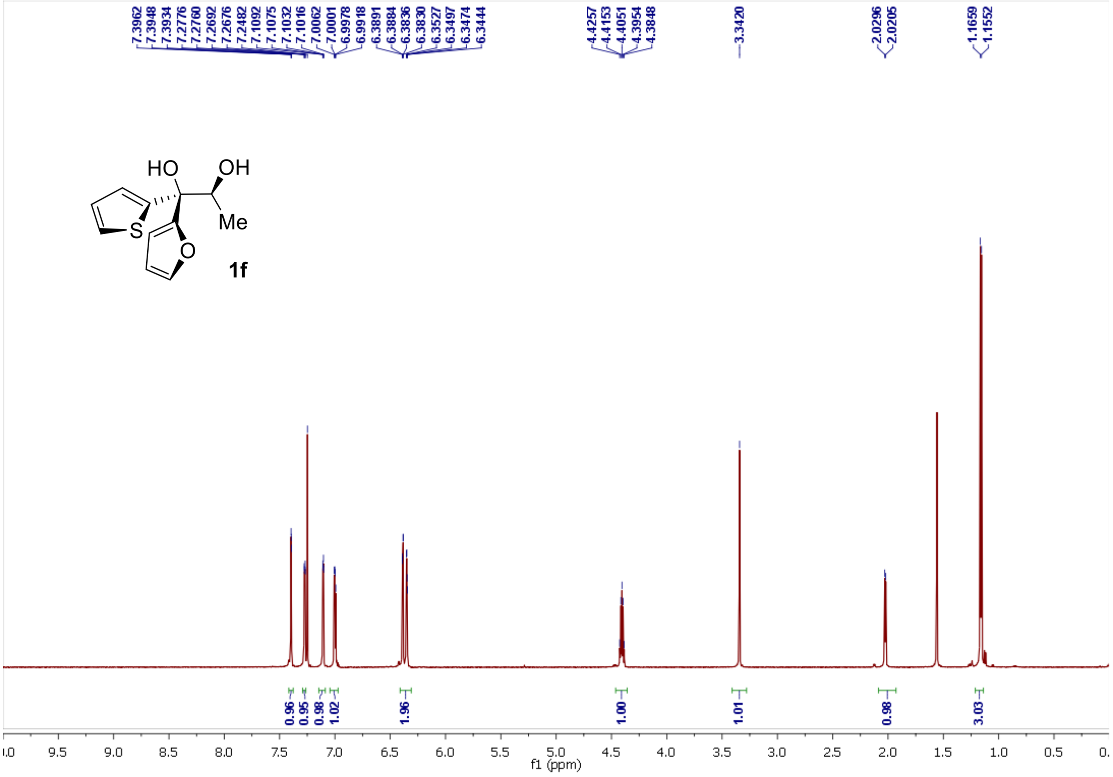

Supplementary Figure 45. ^{13}C NMR sepctra of (1*S*,2*S*)-1-(Furan-2-yl)-1-(thiophen-2-yl)propane-1,2-diol (**1f**)

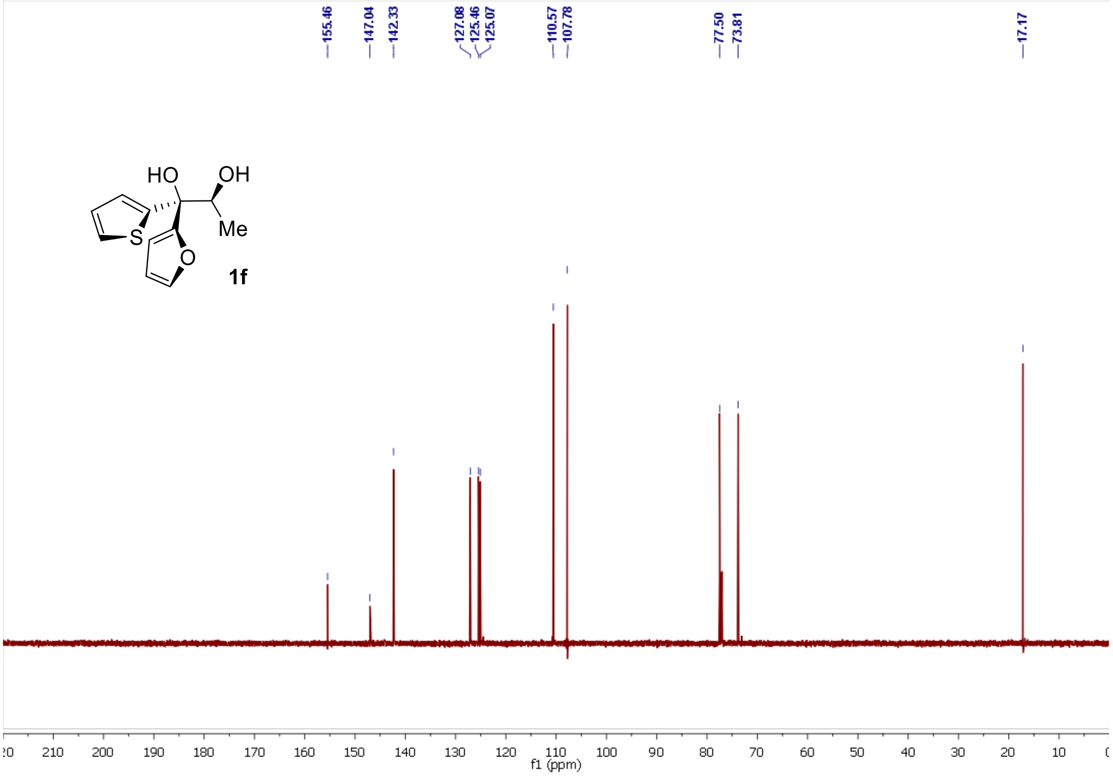

Supplementary Figure 46. ^{1}H NMR sepctra of (1*R*,2*R*)-1-(Furan-2-yl)-1-hydroxy-1-(thiophen-2-yl)propan-2-yl propionate (**2f**)

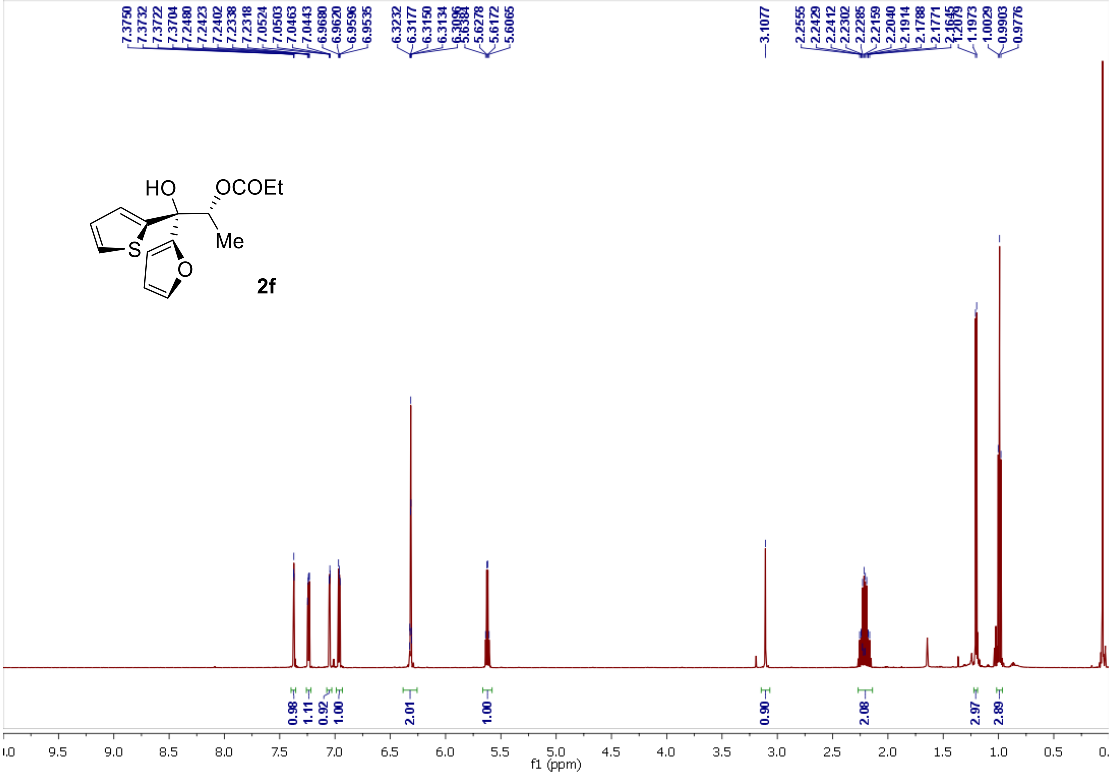

Supplementary Figure 47. ^{13}C NMR sepctra of (1*R*,2*R*)-1-(Furan-2-yl)-1-hydroxy-1-(thiophen-2-yl)propan-2-yl propionate (**2f**)

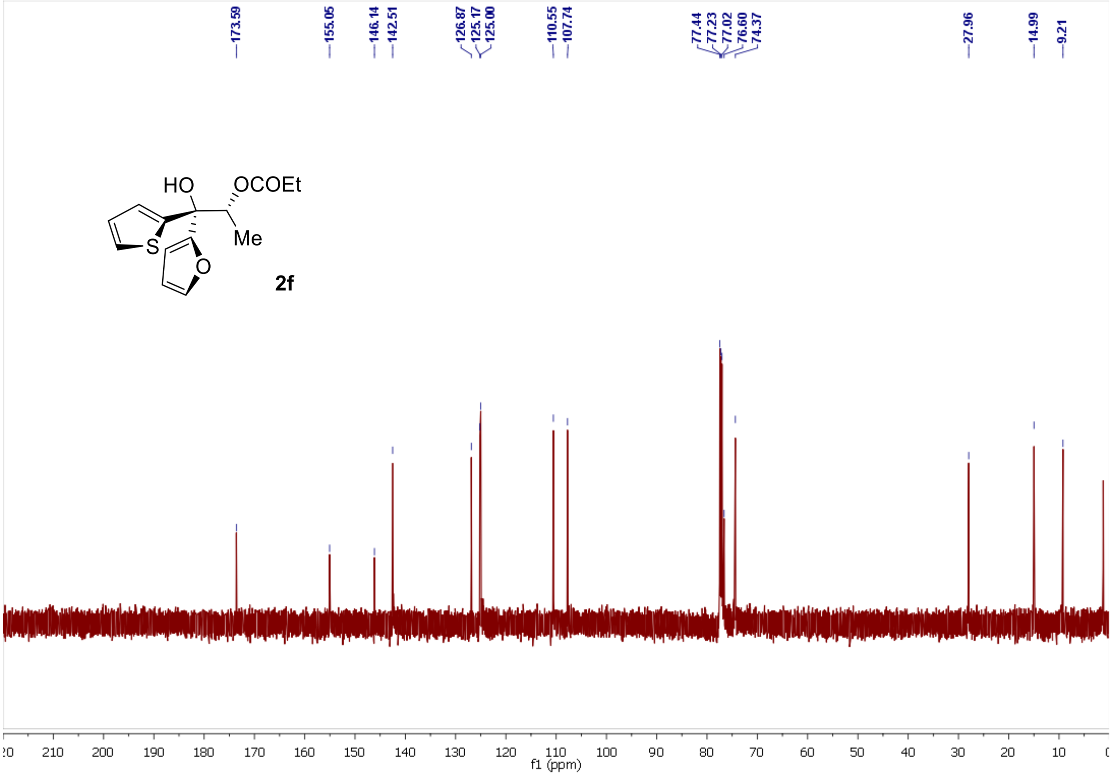

Supplementary Figure 48. ^{1}H NMR sepctra of (1*S*,2*S*)-1-Phenyl-1-(pyridin-2-yl)propane-1,2-diol (**1g**)

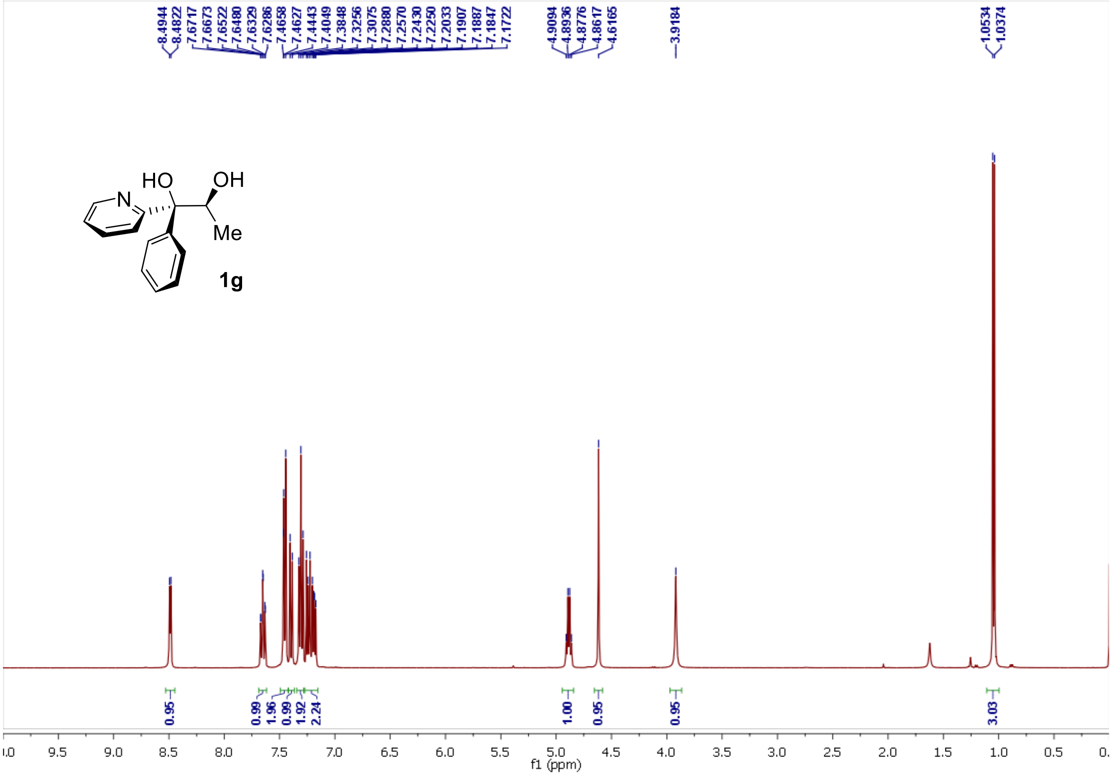

Supplementary Figure 49. ^{13}C NMR sepctra of (1*S*,2*S*)-1-Phenyl-1-(pyridin-2-yl)propane-1,2-diol (**1g**)

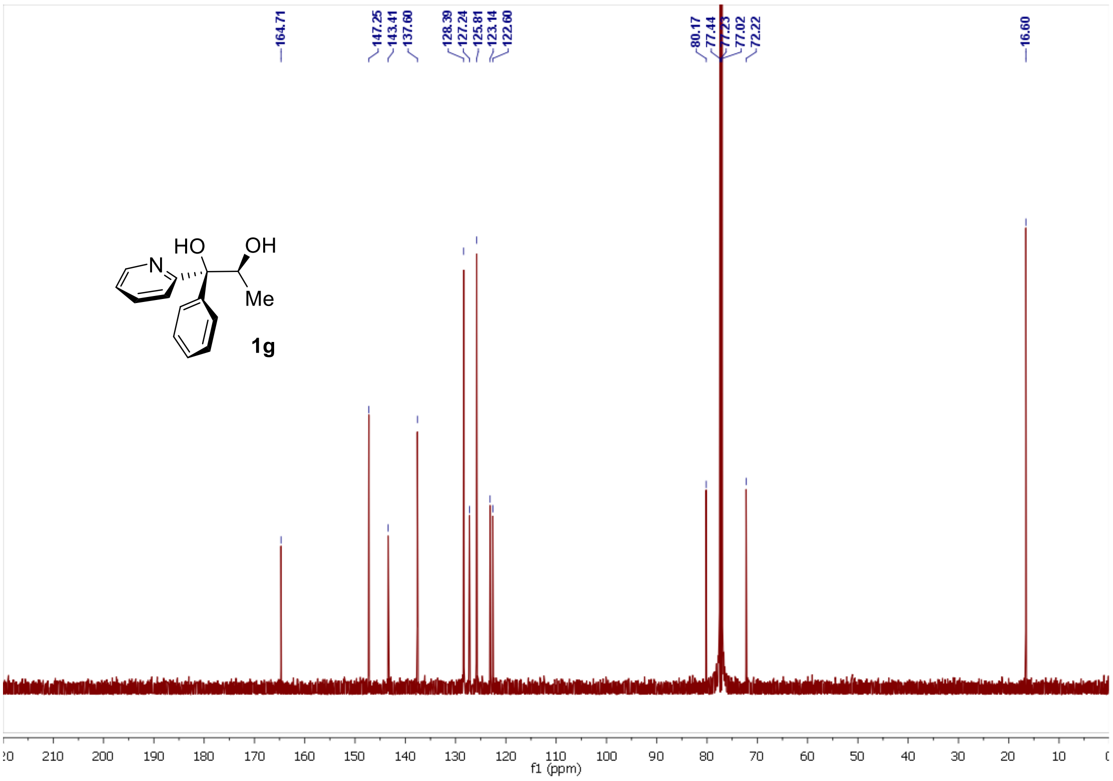

Supplementary Figure 50. ^{1}H NMR sepctra of (1*R*,2*R*)-1-Hydroxy-1-phenyl-1-(pyridin-2-yl)propan-2-yl propionate (**2g**)

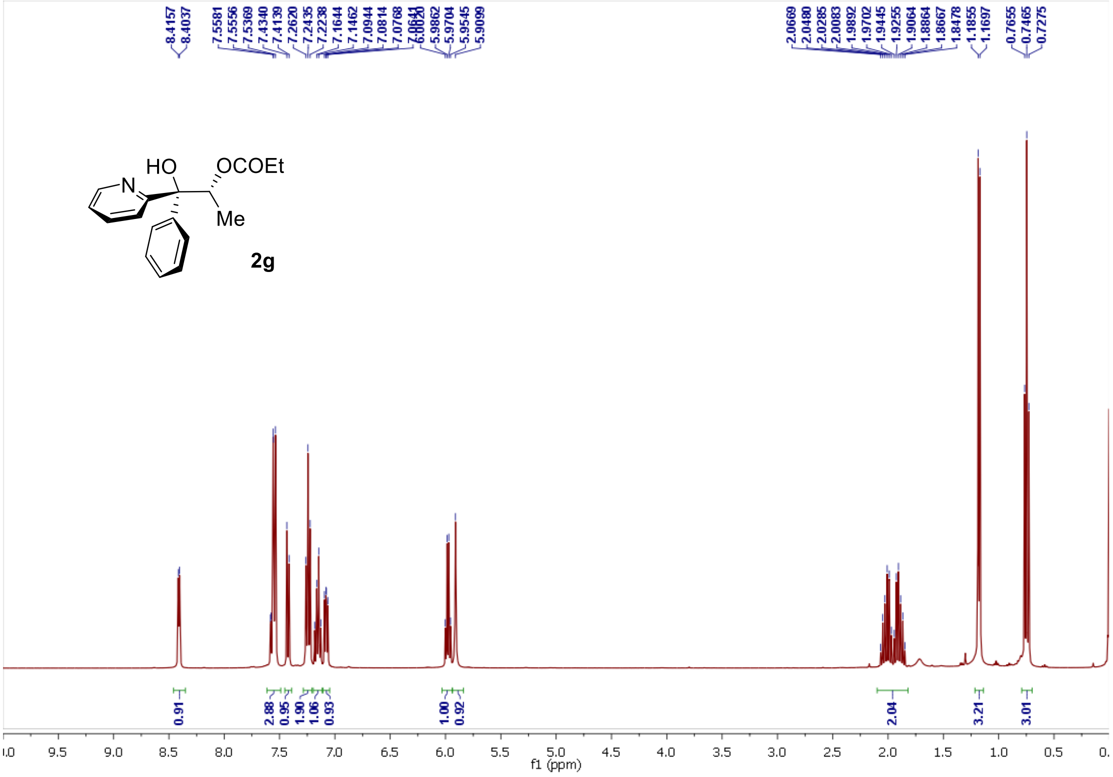

Supplementary Figure 51. ^{13}C NMR sepctra of (1*R*,2*R*)-1-Hydroxy-1-phenyl-1-(pyridin-2-yl)propan-2-yl propionate (**2g**)

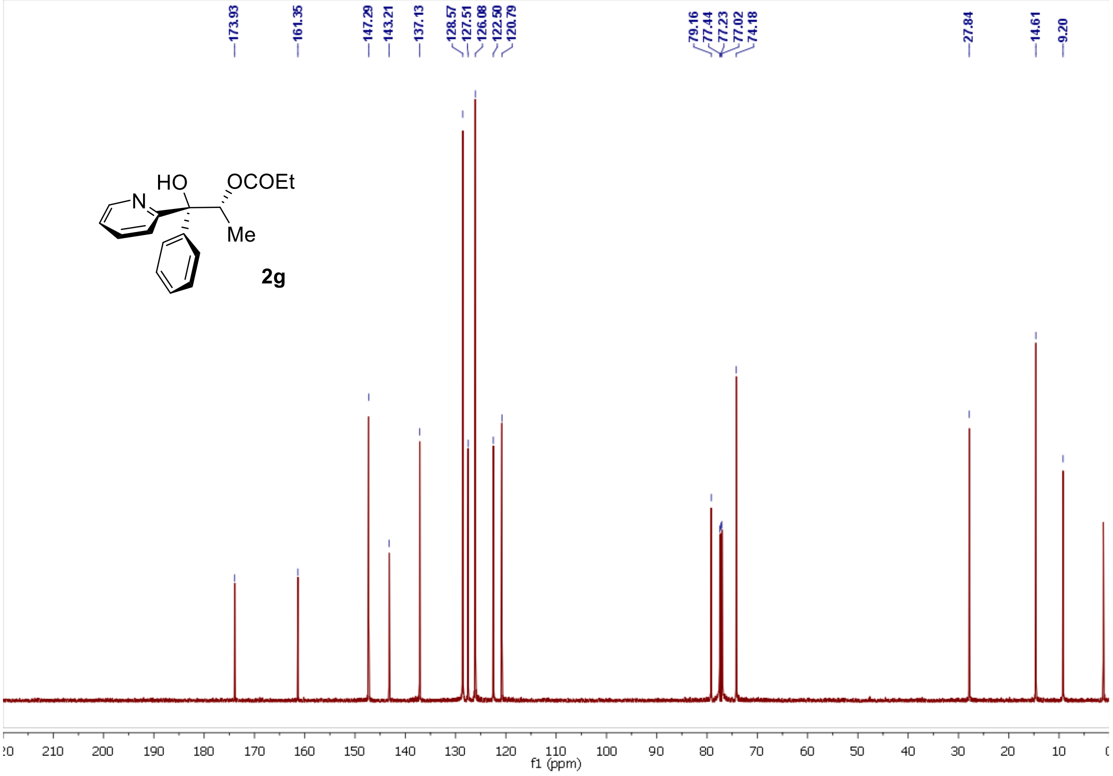

Supplementary Figure 52. ^{1}H NMR sepctra of (1*S*,2*S*)-1-(4-Bromophenyl)-1-(4-chlorophenyl)butane-1,2-diol (**1h**)

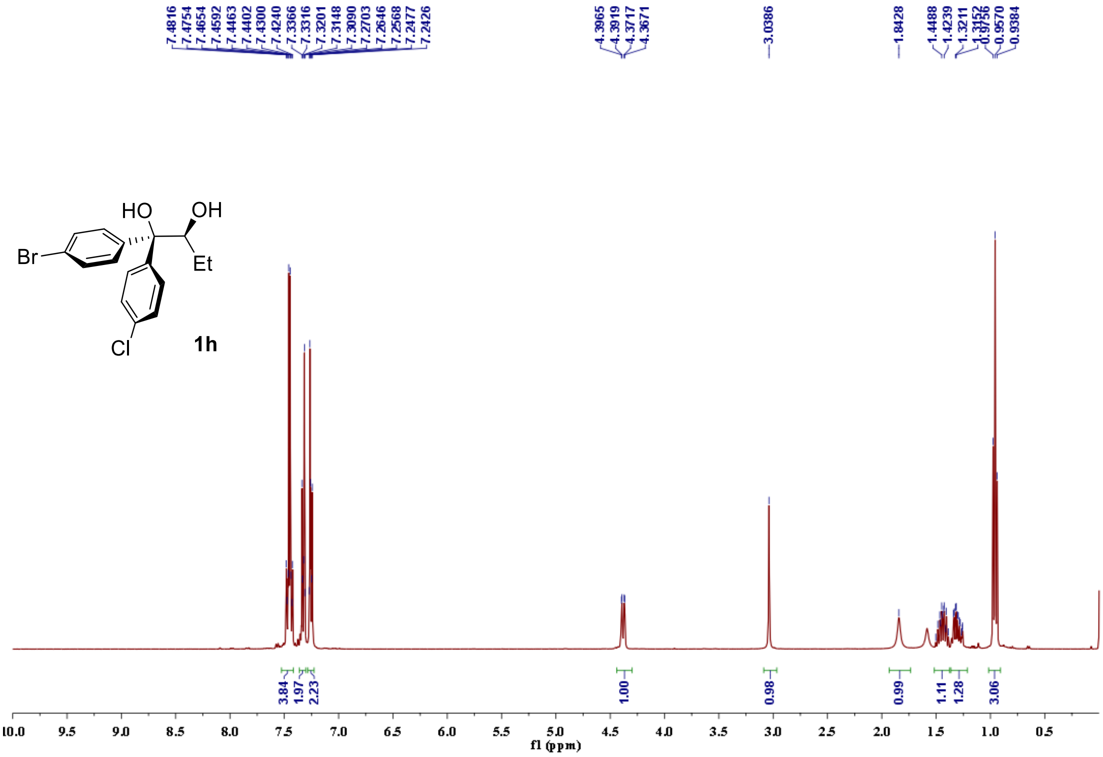

Supplementary Figure 53. ^{13}C NMR sepctra of (1*S*,2*S*)-1-(4-Bromophenyl)-1-(4-chlorophenyl)butane-1,2-diol (**1h**)

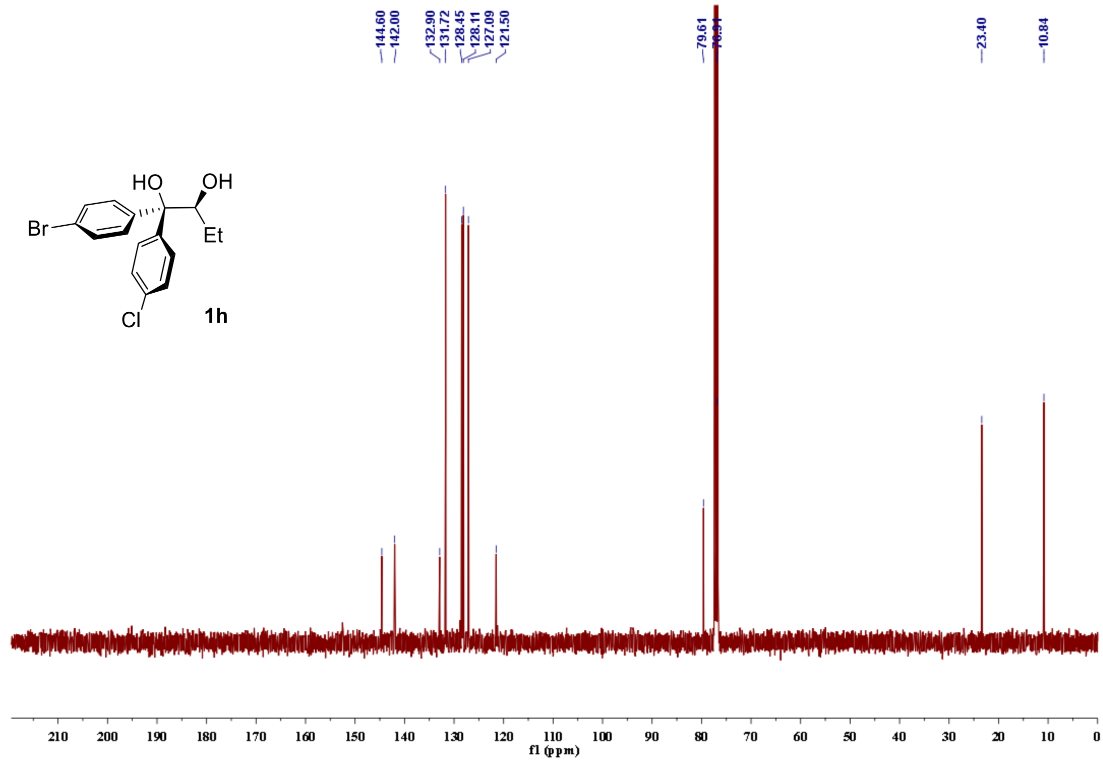

Supplementary Figure 54. ^{1}H NMR sepctra of (1*R*,2*R*)-1-(4-Bromophenyl)-1-(4-chlorophenyl)-1-hydroxybutan-2-yl propionate (**2h**)

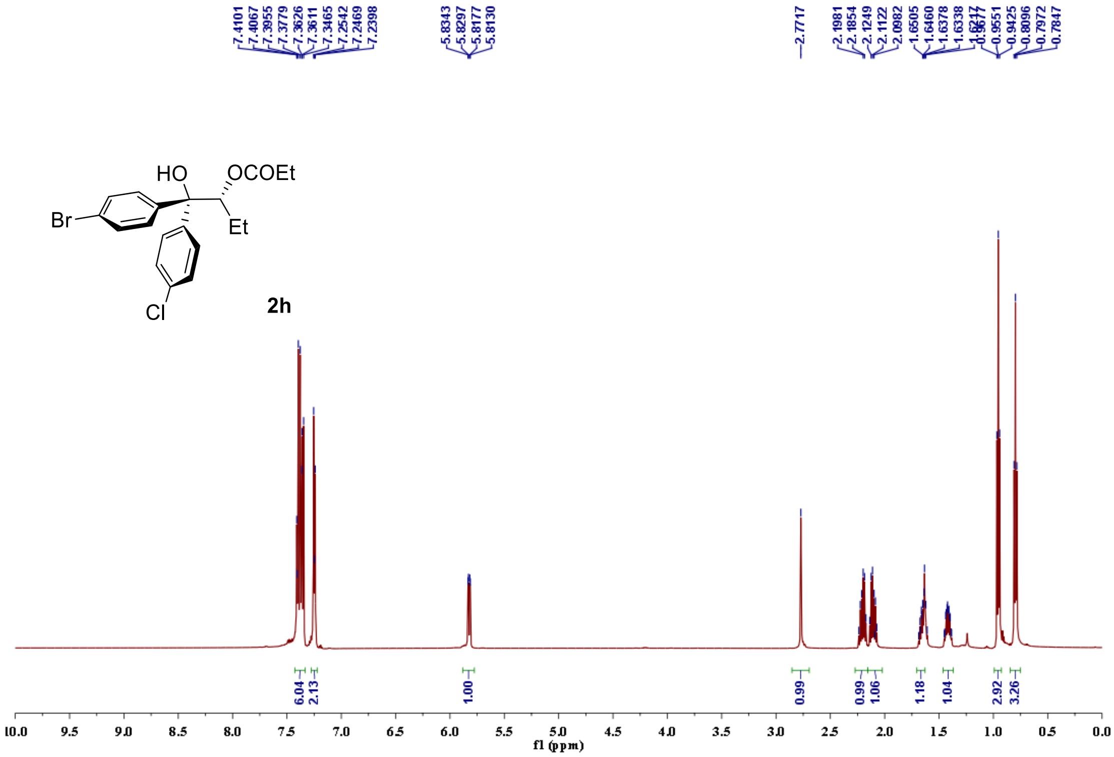

Supplementary Figure 55. ^{13}C NMR sepctra of (1*R*,2*R*)-1-(4-Bromophenyl)-1-(4-chlorophenyl)-1-hydroxybutan-2-yl propionate (**2h**)

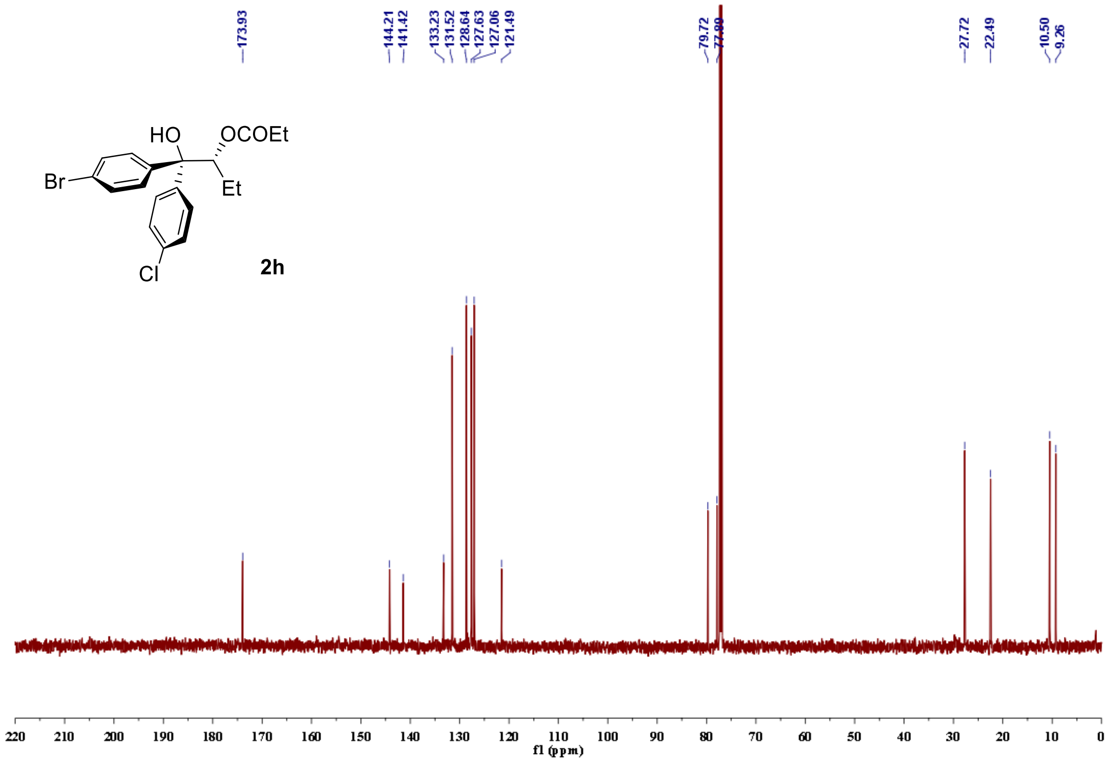

Supplementary Figure 56. ^{1}H NMR sepctra of (1*R*,2*S*)-1,3-Diphenyl-1-(*p*-tolyl)propane-1,2-diol (**1i**)

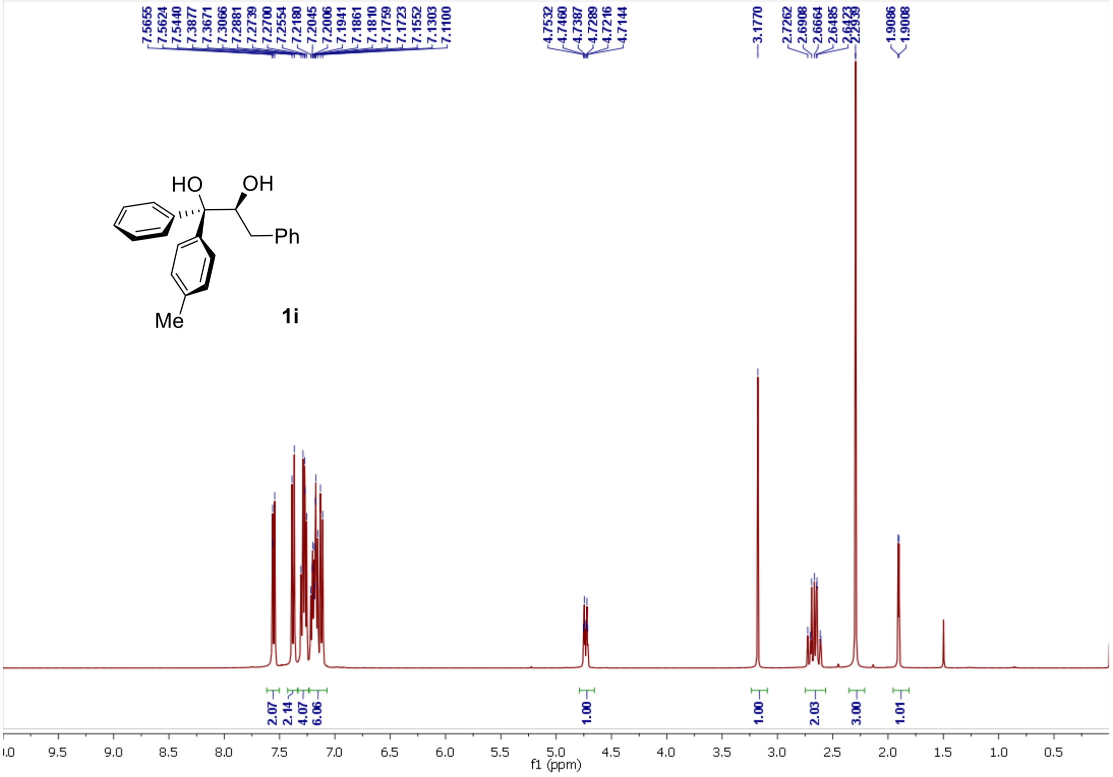

Supplementary Figure 57. ^{13}C NMR sepctra of (1*R*,2*S*)-1,3-Diphenyl-1-(*p*-tolyl)propane-1,2-diol (**1i**)

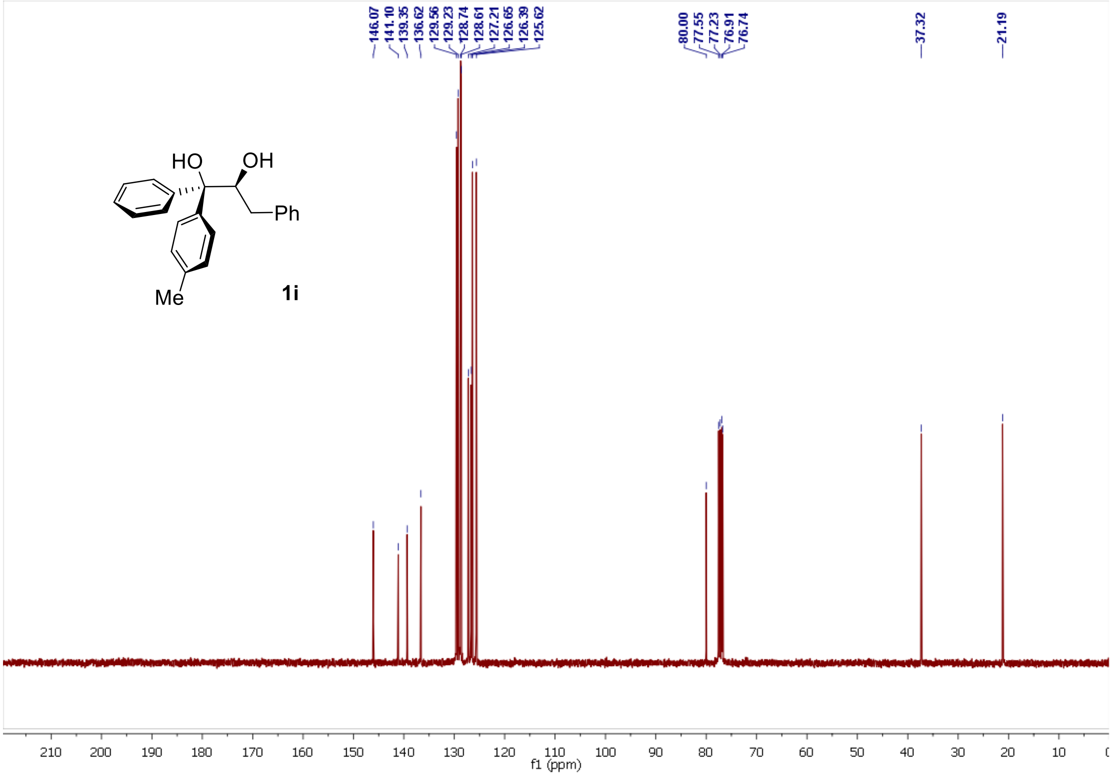

Supplementary Figure 58. ^{1}H NMR sepctra of (1*S*,2*R*)-1-Hydroxy-1,3-diphenyl-1-(*p*-tolyl)propan-2-yl propionate (**2i**)

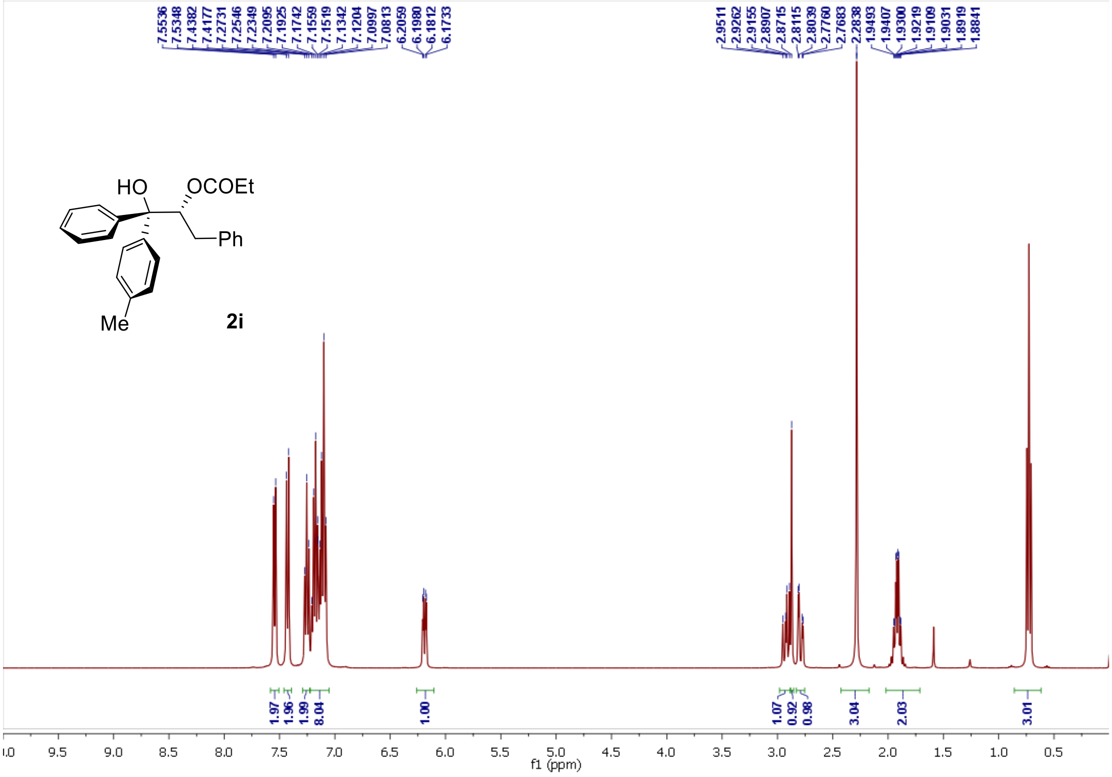

Supplementary Figure 59. ^{13}C NMR sepctra of (1*S*,2*R*)-1-Hydroxy-1,3-diphenyl-1-(*p*-tolyl)propan-2-yl propionate (**2i**)

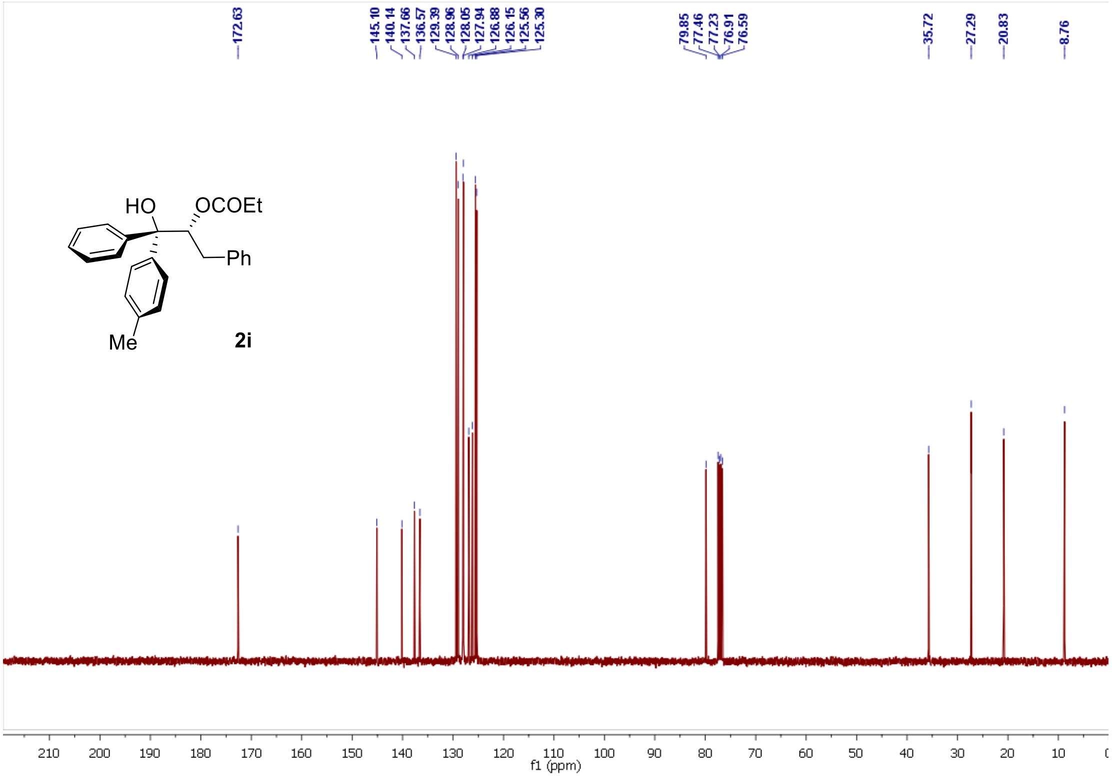

Supplementary Figure 60. ^{1}H NMR sepctra of (1*S*,2*S*)-1-(4-Methoxyphenyl)-3-phenyl-1-(*p*-tolyl)propane-1,2-diol (**1j**)

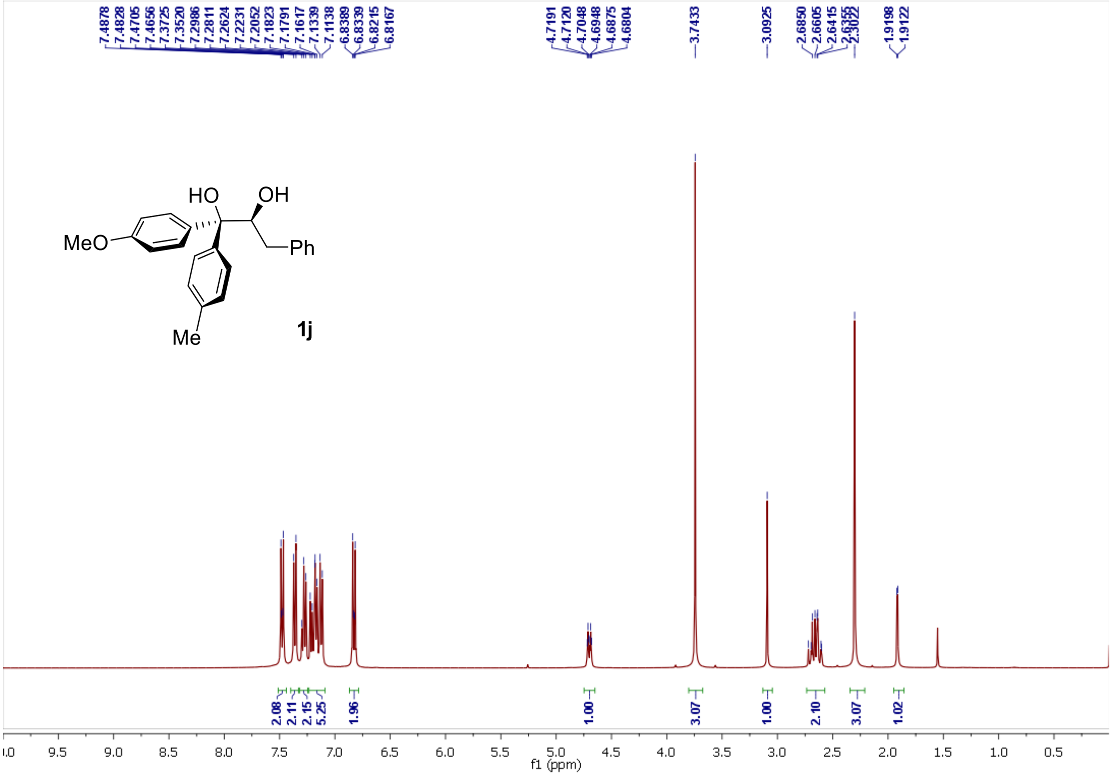

Supplementary Figure 61. ^{13}C NMR sepctra of (1*S*,2*S*)-1-(4-Methoxyphenyl)-3-phenyl-1-(*p*-tolyl)propane-1,2-diol (**1j**)

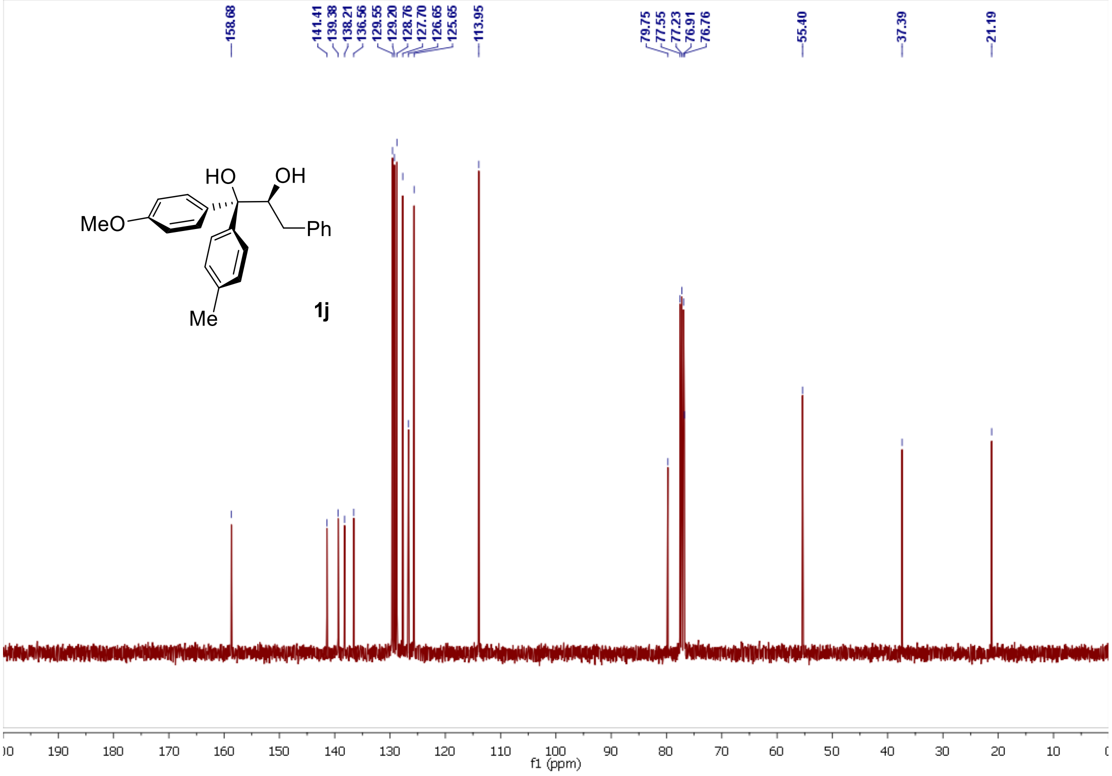

Supplementary Figure 62. ^{1}H NMR sepctra of (1*R*,2*R*)-1-Hydroxy-1-(4-methoxyphenyl)-3-phenyl-1-(*p*-tolyl)propan-2-yl propionate (**2j**)

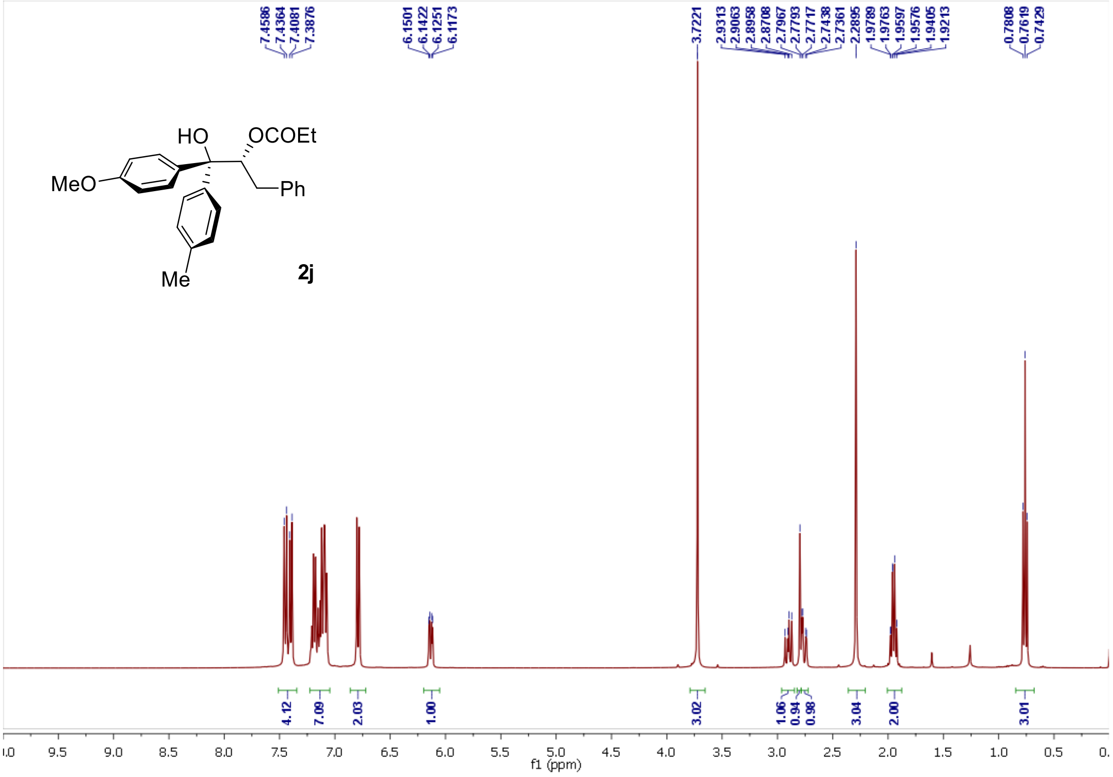

Supplementary Figure 63. ^{13}C NMR sepctra of (1*R*,2*R*)-1-Hydroxy-1-(4-methoxyphenyl)-3-phenyl-1-(*p*-tolyl)propan-2-yl propionate (**2j**)

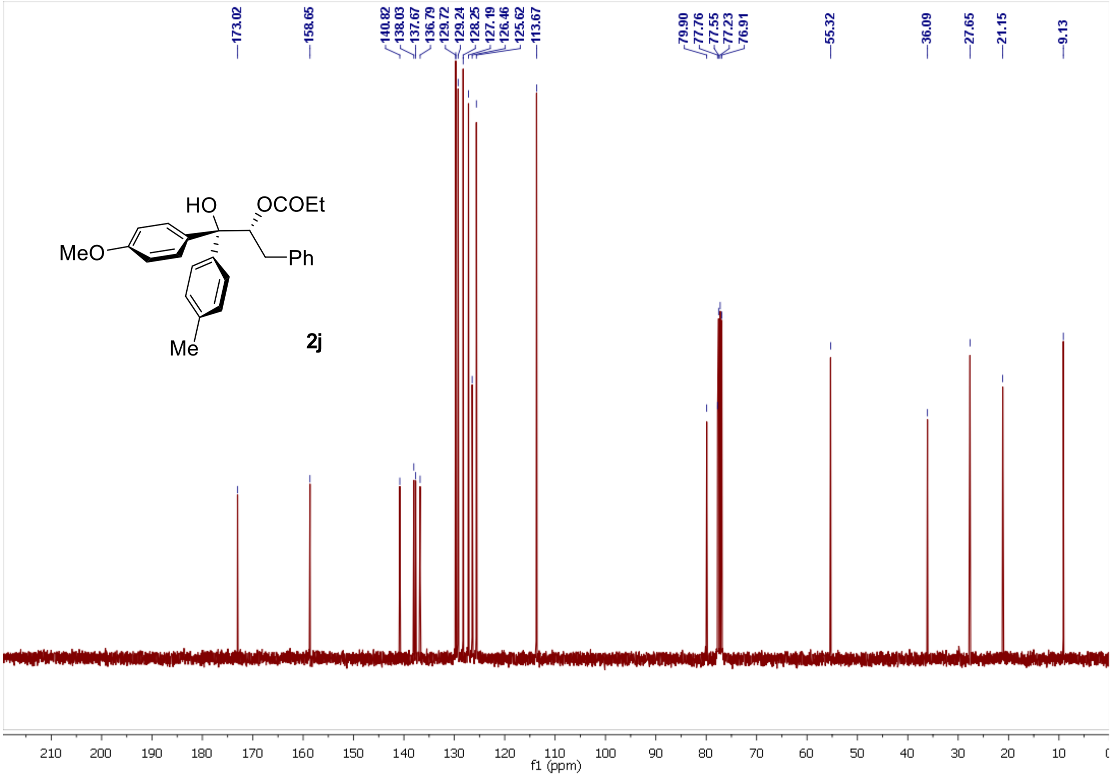

Supplementary Figure 64. ^{1}H NMR sepctra of (1*S*,2*S*)-1-(4-Methoxyphenyl)-1-phenylpentane-1,2-diol (**1k**)

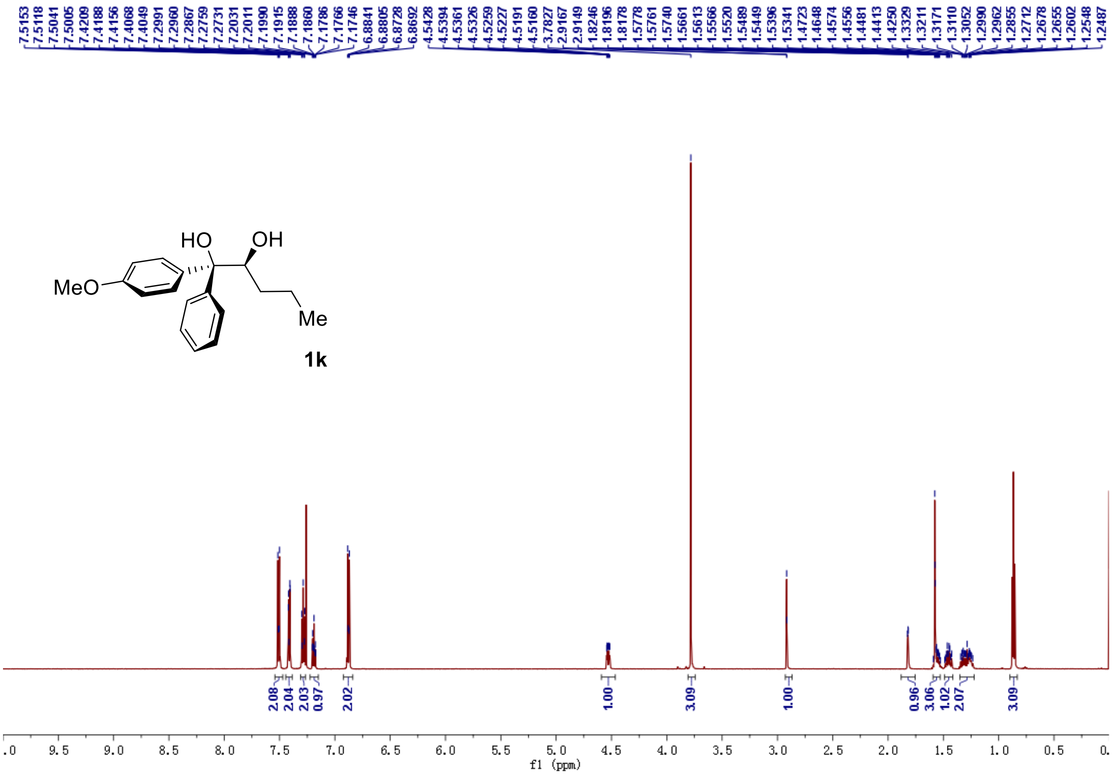

Supplementary Figure 65. ^{13}C NMR sepctra of (1*S*,2*S*)-1-(4-Methoxyphenyl)-1-phenylpentane-1,2-diol (**1k**)

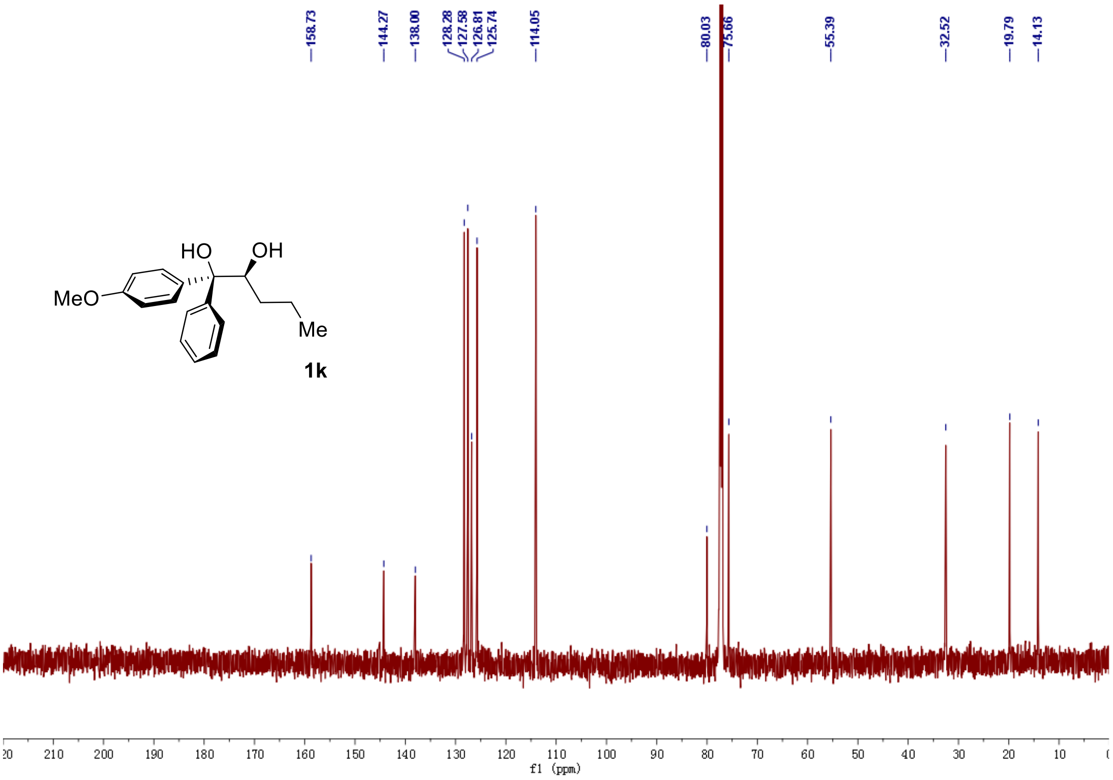

Supplementary Figure 66. ^{1}H NMR sepctra of (1*R*,2*R*)-1-Hydroxy-1-(4-methoxyphenyl)-1-phenylpentan-2-yl propionate (**2k**)

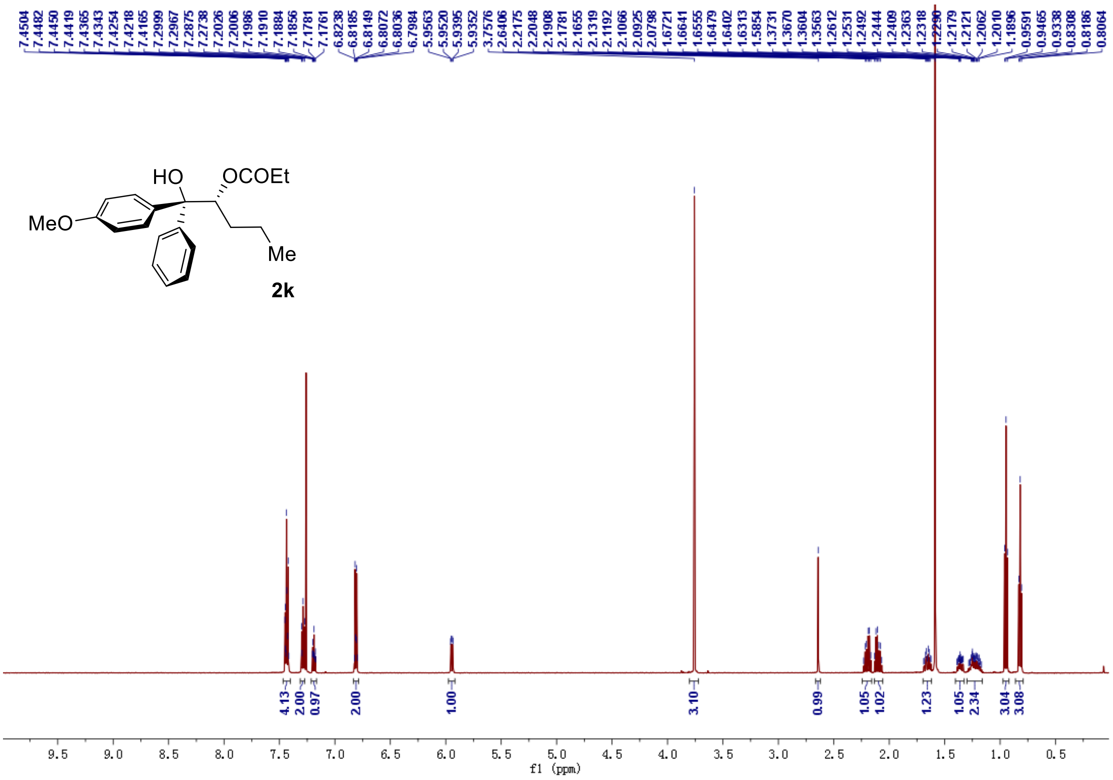

Supplementary Figure 67. ^{13}C NMR sepctra of (1*R*,2*R*)-1-Hydroxy-1-(4-methoxyphenyl)-1-phenylpentan-2-yl propionate (**2k**)

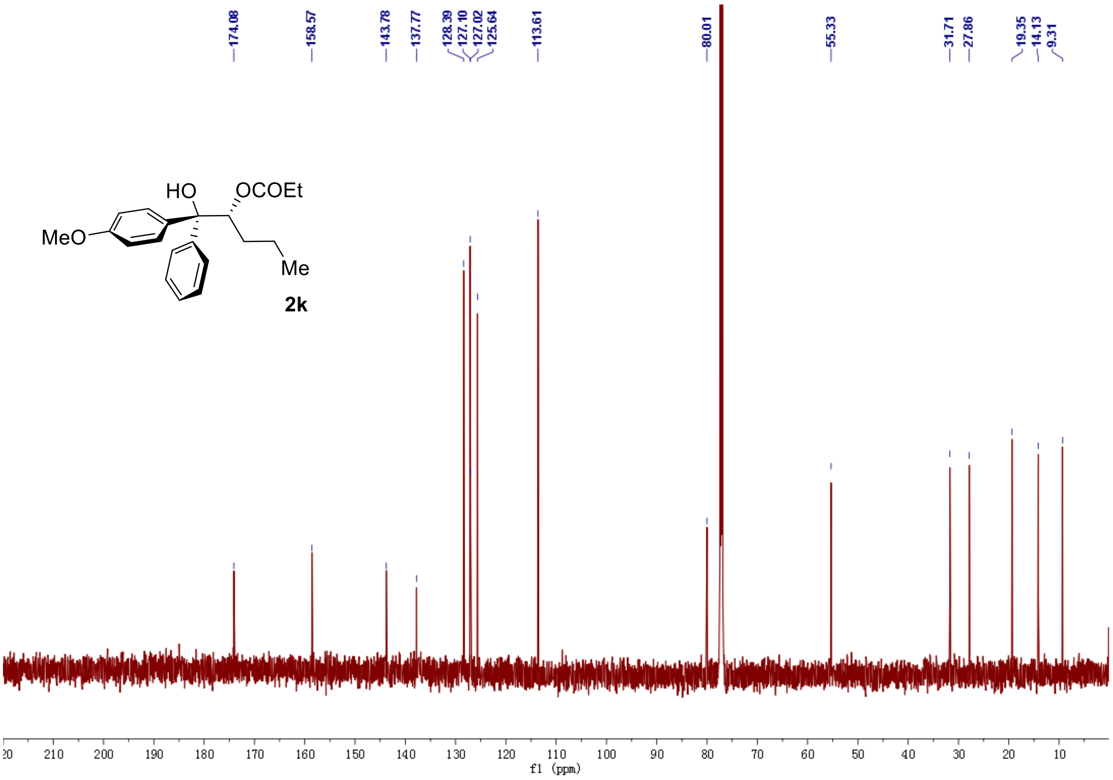

Supplementary Figure 68. ^{1}H NMR sepctra of (1*S*,2*S*)-1-Phenyl-1-(phenyl-*d*5)pentane-1,2-diol (**1l**)

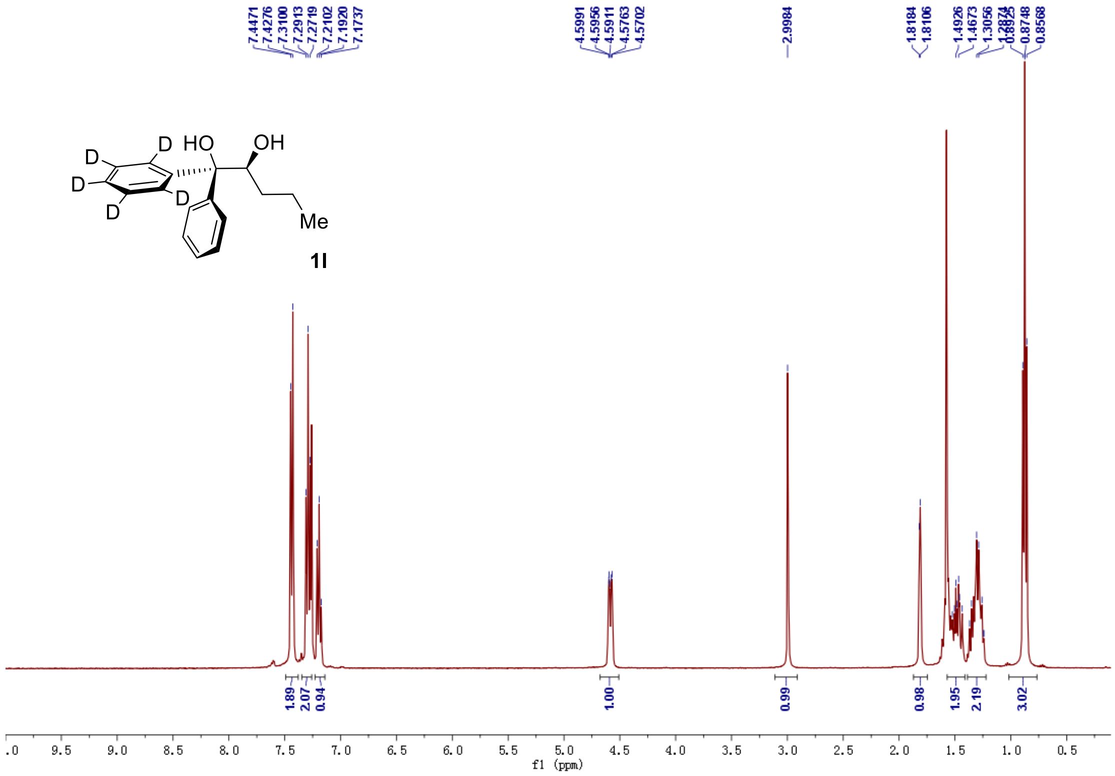

Supplementary Figure 69. ^{13}C NMR sepctra of (1*S*,2*S*)-1-Phenyl-1-(phenyl-*d*5)pentane-1,2-diol (**1l**)

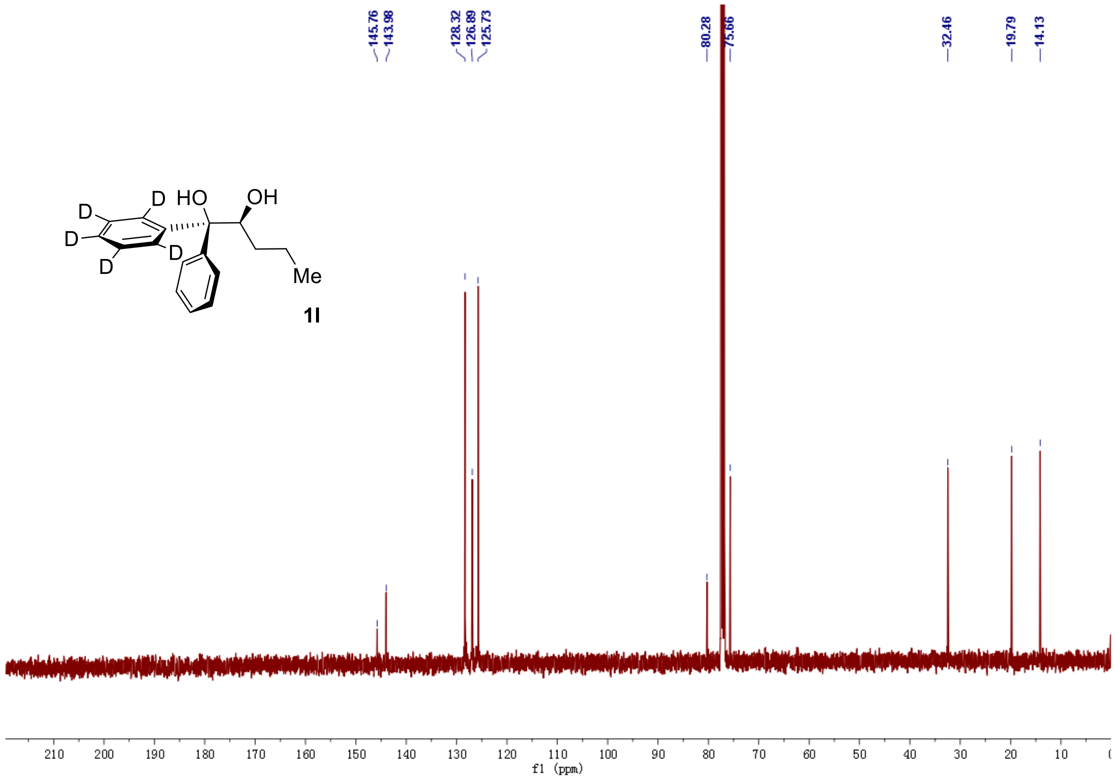

Supplementary Figure 70. ^{1}H NMR sepctra of (1*R*,2*R*)-1-Hydroxy-1-phenyl-1-(phenyl-*d*5)pentan-2-yl propionate (**2l**)

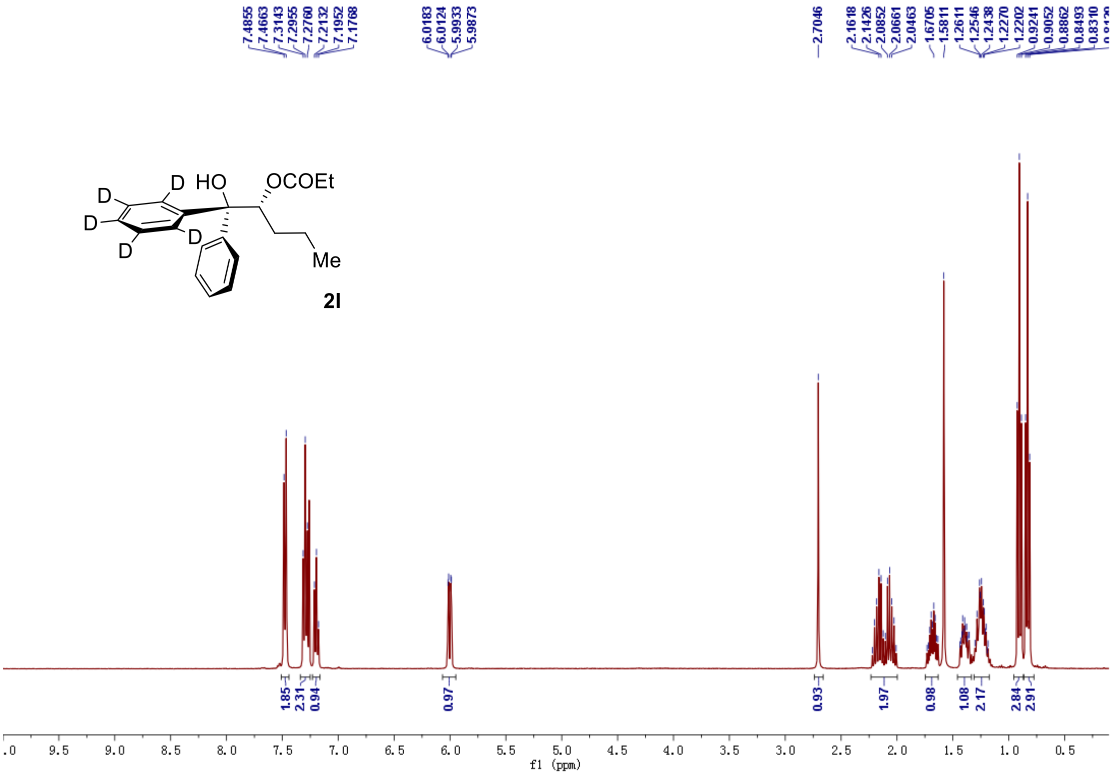

Supplementary Figure 71. ^{13}C NMR sepctra of (1*R*,2*R*)-1-Hydroxy-1-phenyl-1-(phenyl-*d*5)pentan-2-yl propionate (**2l**)

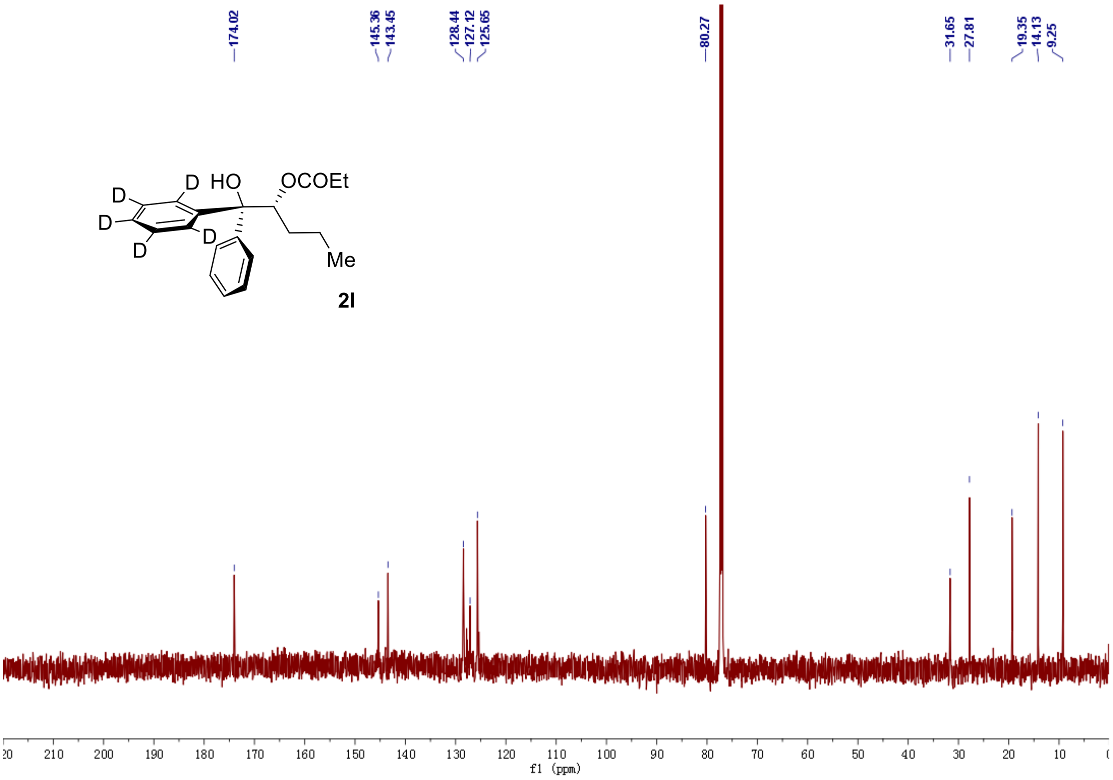

Supplementary Figure 72. ^{1}H NMR sepctra of (1*S*,2*R*)-2-Methyl-1-phenylbutane-1,2-diol (**1m**)

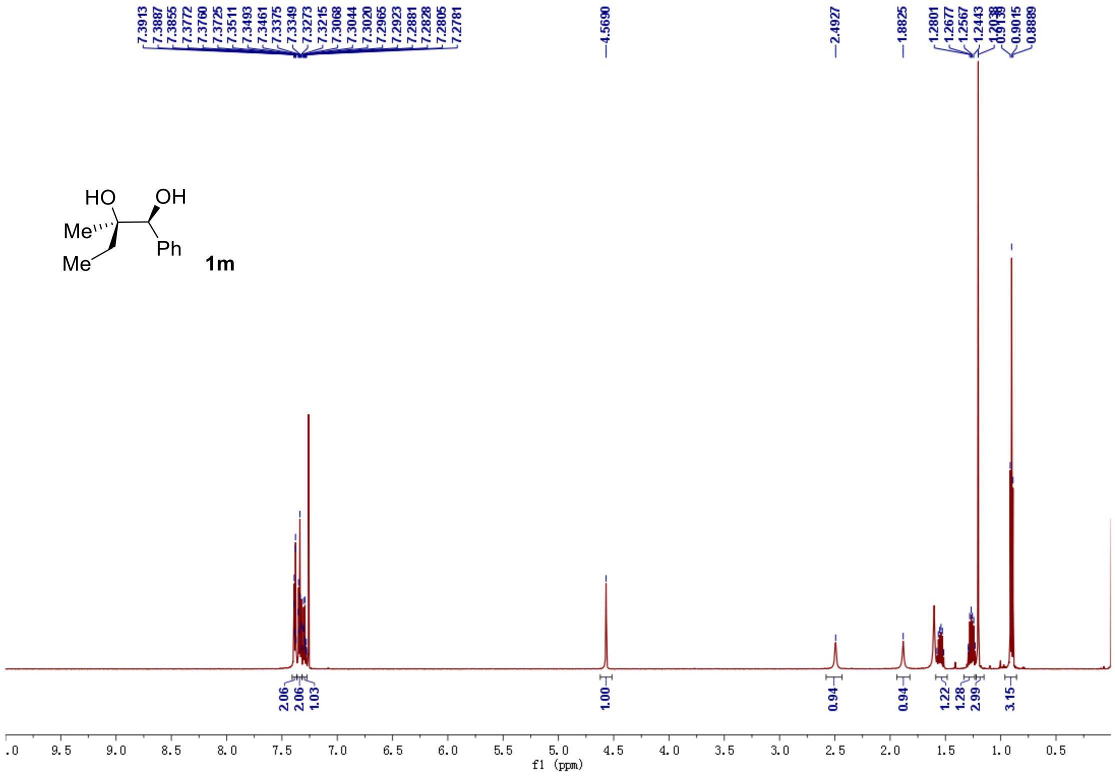

Supplementary Figure 73. ^{13}C NMR sepctra of (1*S*,2*R*)-2-Methyl-1-phenylbutane-1,2-diol (**1m**)

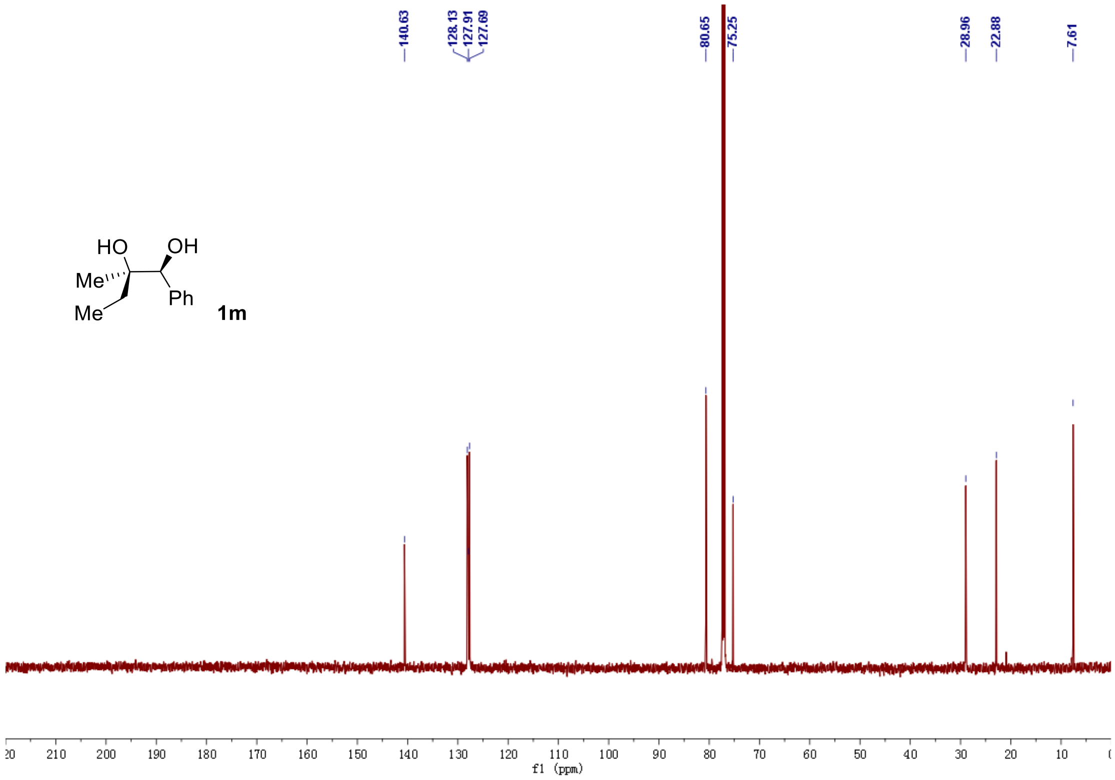

Supplementary Figure 74. ^{1}H NMR sepctra of (1*R*,2*S*)-2-Hydroxy-2-methyl-1-phenylbutyl propionate (**2m**)

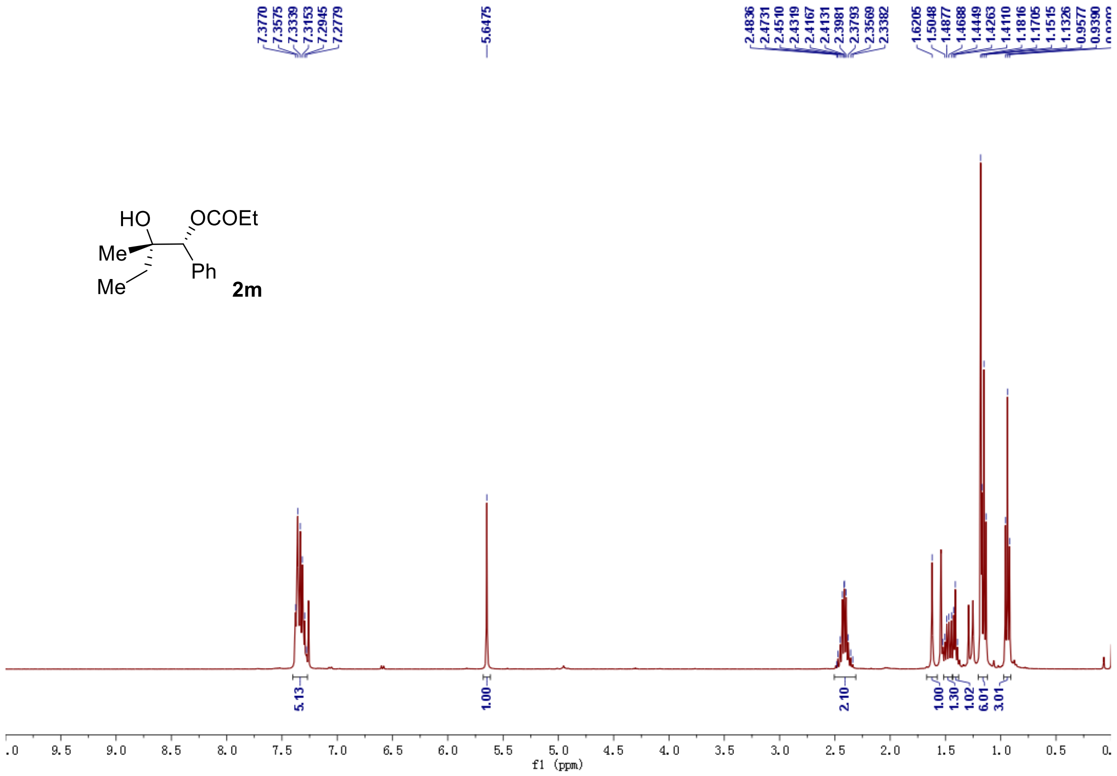

Supplementary Figure 75. ^{13}C NMR sepctra of (1*R*,2*S*)-2-Hydroxy-2-methyl-1-phenylbutyl propionate (**2m**)

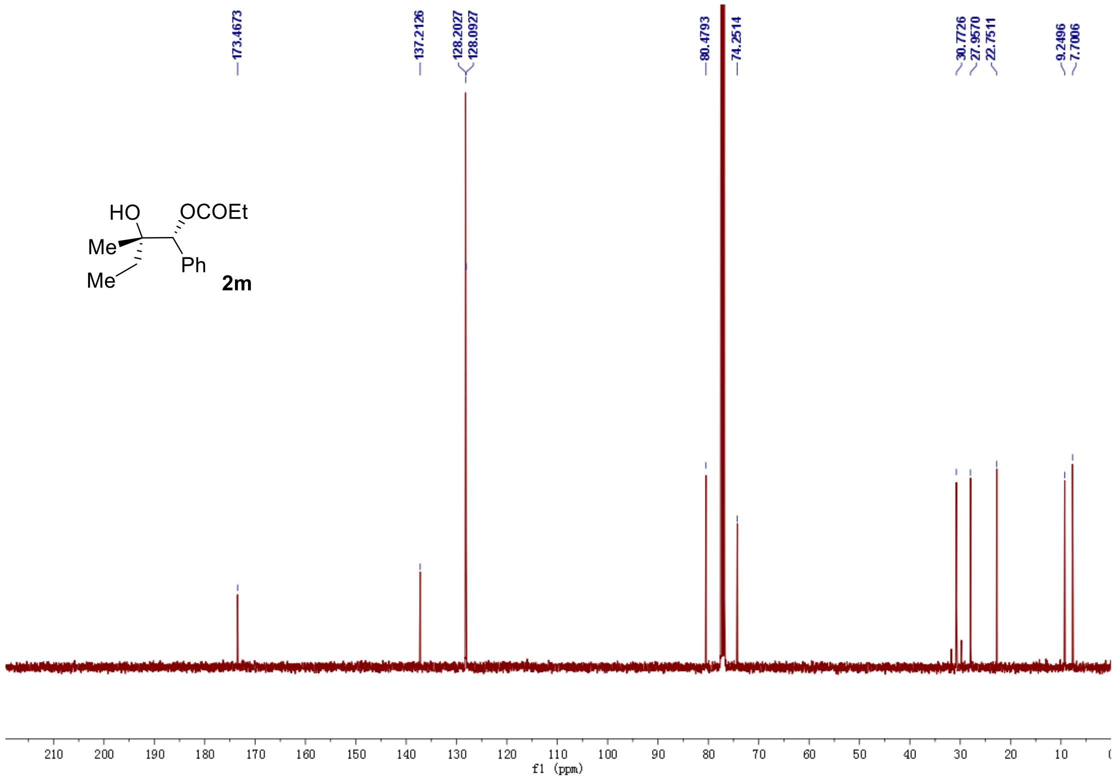

Supplementary Figure 76. ^{1}H NMR sepctra of (1*S*,2*R*)-2-Cyclohexyl-1-phenylpropane-1,2-diol (**1n**)

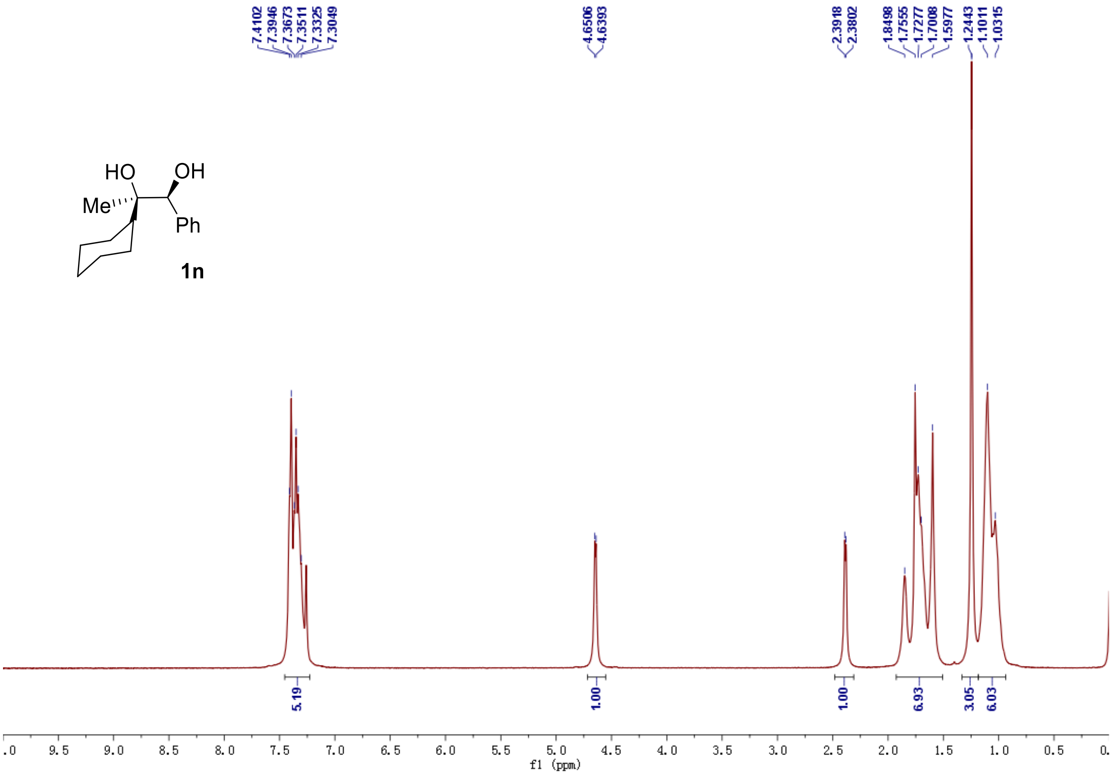

Supplementary Figure 77. ^{13}C NMR sepctra of (1*S*,2*R*)-2-Cyclohexyl-1-phenylpropane-1,2-diol (**1n**)

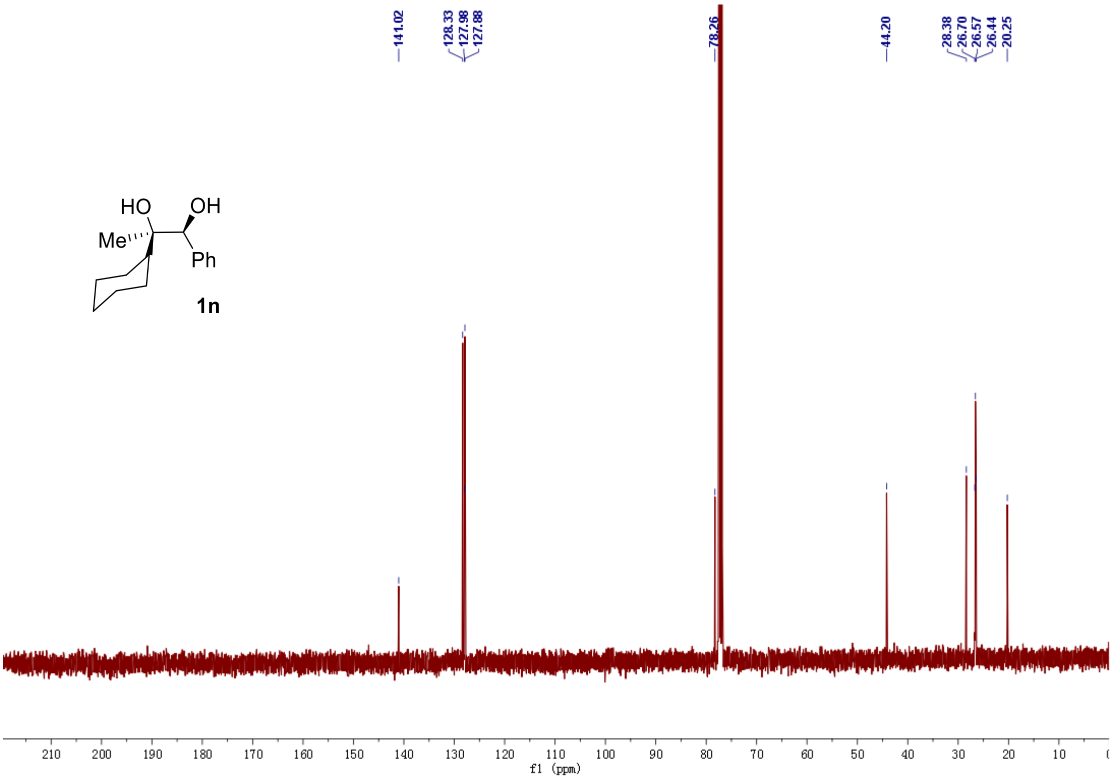

Supplementary Figure 78. ^{1}H NMR sepctra of (1*R*,2*S*)-2-Cyclohexyl-2-hydroxy-1-phenylpropyl propionate (**2n**)

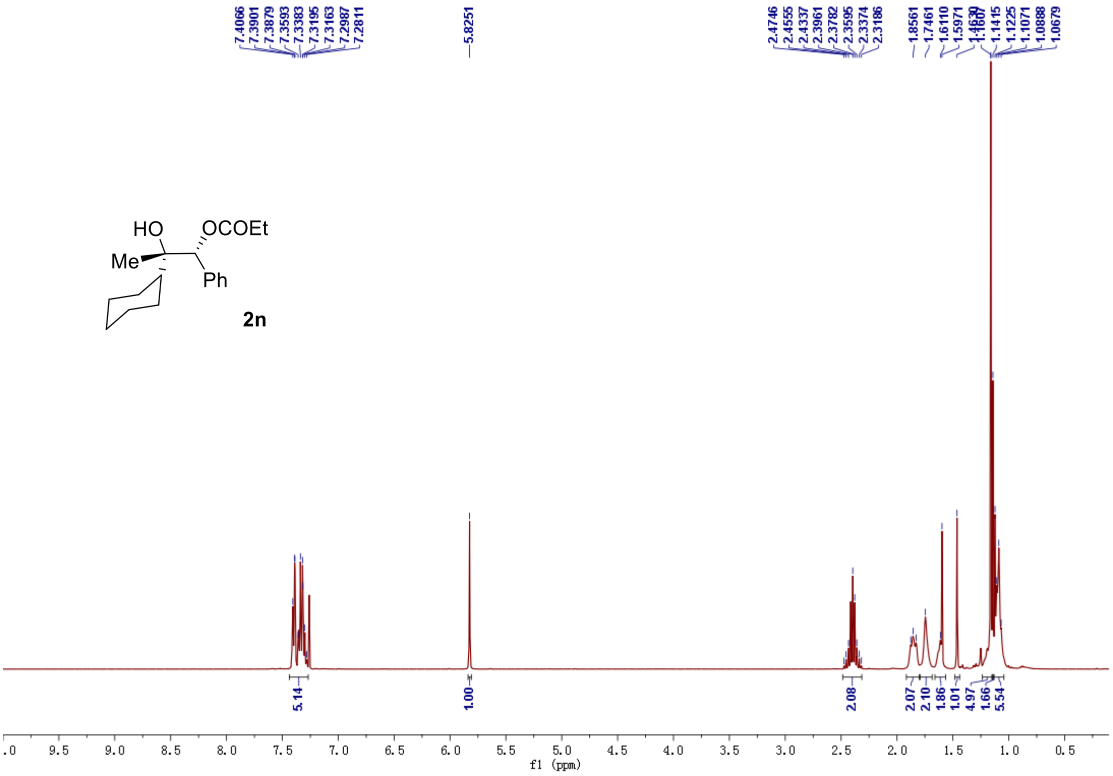

Supplementary Figure 79. ^{13}C NMR sepctra of (1*R*,2*S*)-2-Cyclohexyl-2-hydroxy-1-phenylpropyl propionate (**2n**)

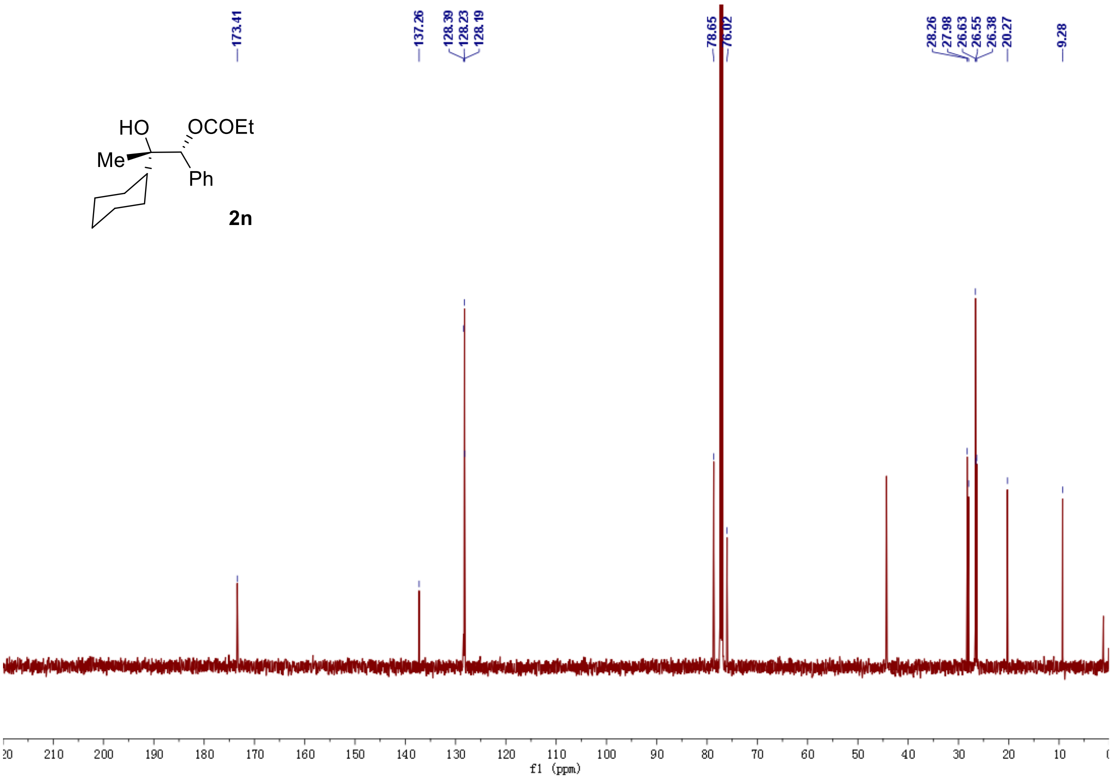

Supplementary Figure 80. ^{1}H NMR sepctra of (1*S*,2*R*)-2,3-Dimethyl-1-phenylbutane-1,2-diol (**1o**)

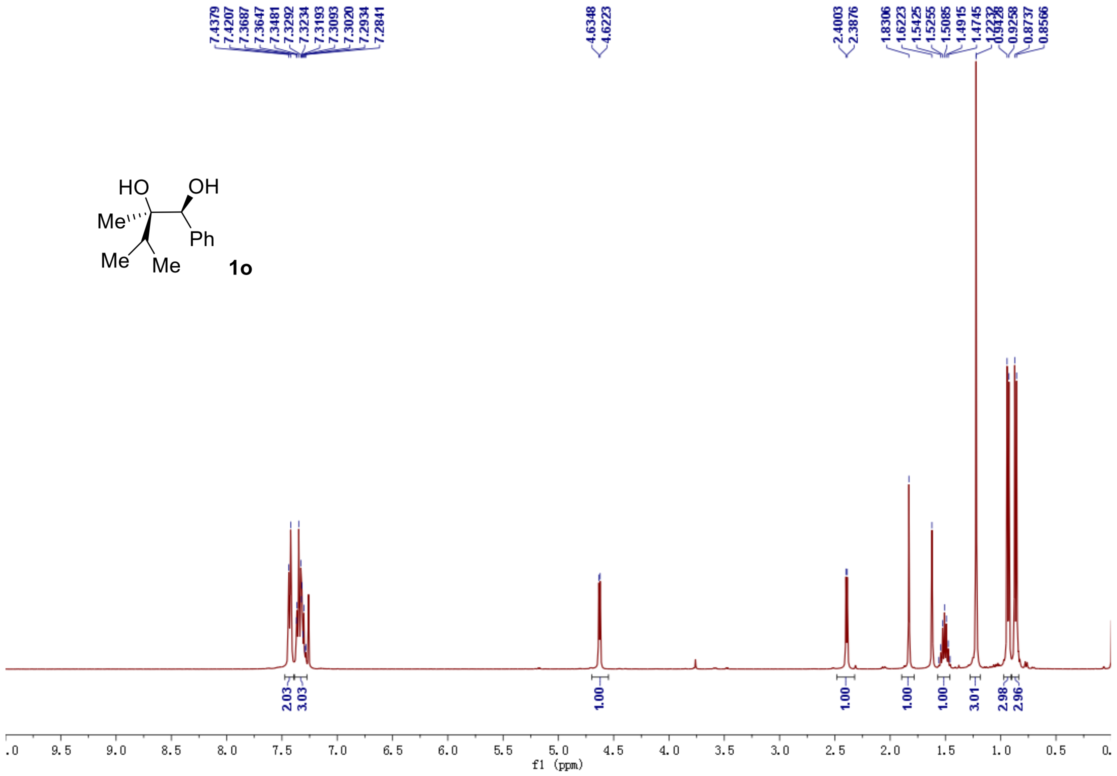

Supplementary Figure 81. ^{13}C NMR sepctra of (1*S*,2*R*)-2,3-Dimethyl-1-phenylbutane-1,2-diol (**1o**)

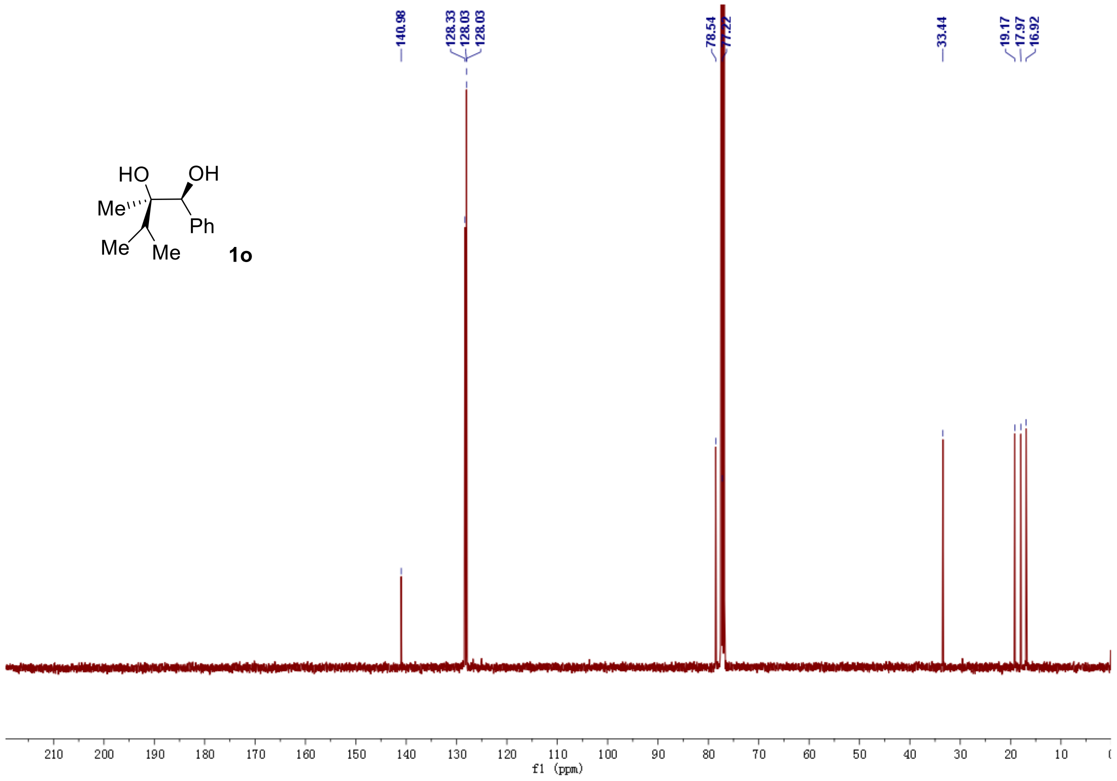

Supplementary Figure 82. ^{1}H NMR sepctra of (1*R*,2*S*)-2-Hydroxy-2,3-dimethyl-1-phenylbutyl propionate (**2o**)

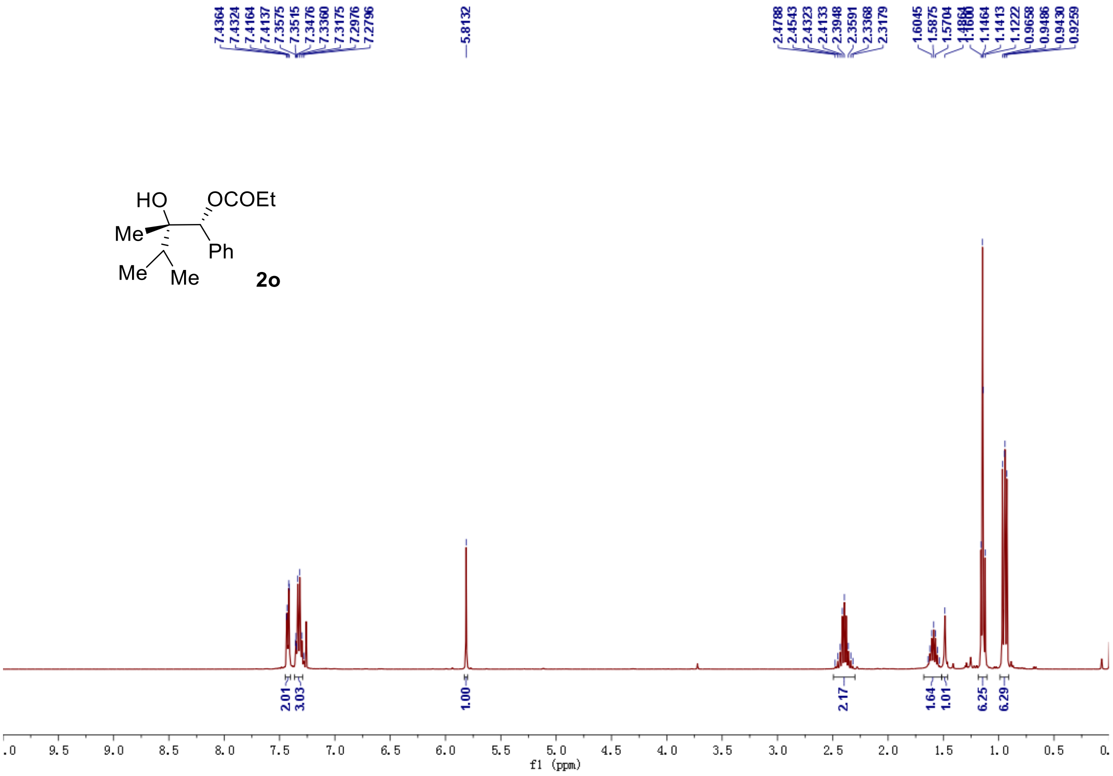

Supplementary Figure 83. ^{13}C NMR sepctra of (1*R*,2*S*)-2-Hydroxy-2,3-dimethyl-1-phenylbutyl propionate (**2o**)

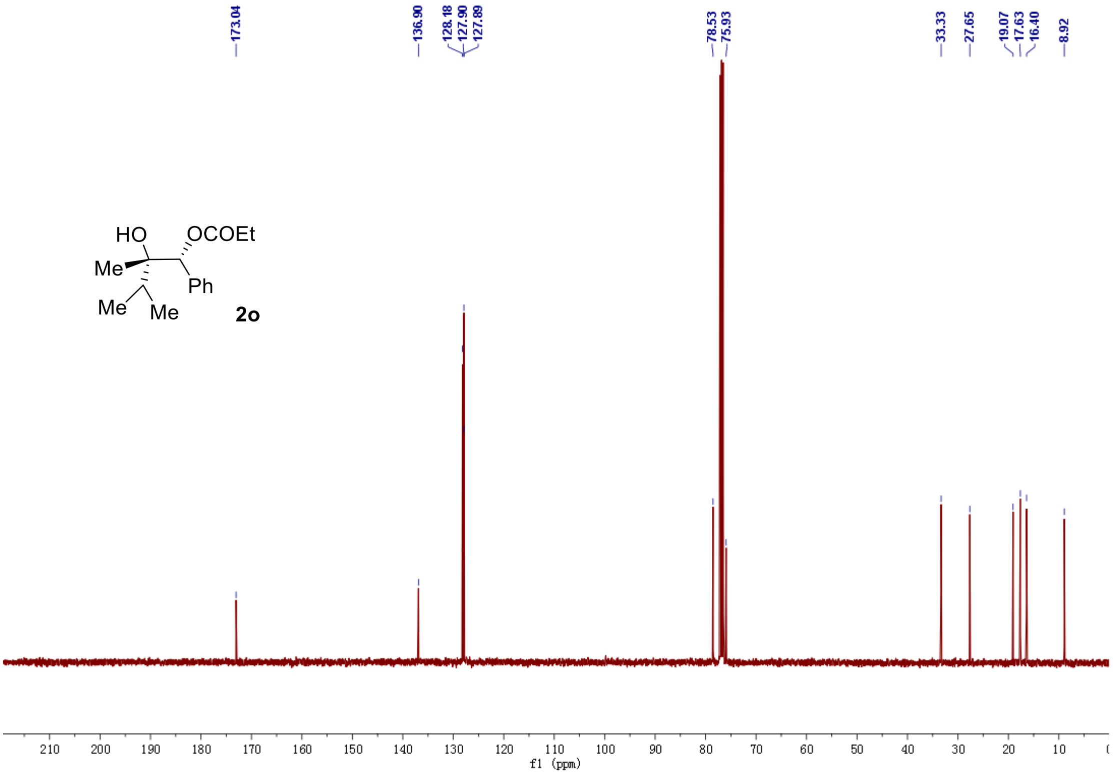

Supplementary Figure 84. ^{1}H NMR sepctra of (1*S*,2*S*)-2-Methyl-1-phenylpent-4-ene-1,2-diol (**1p**)

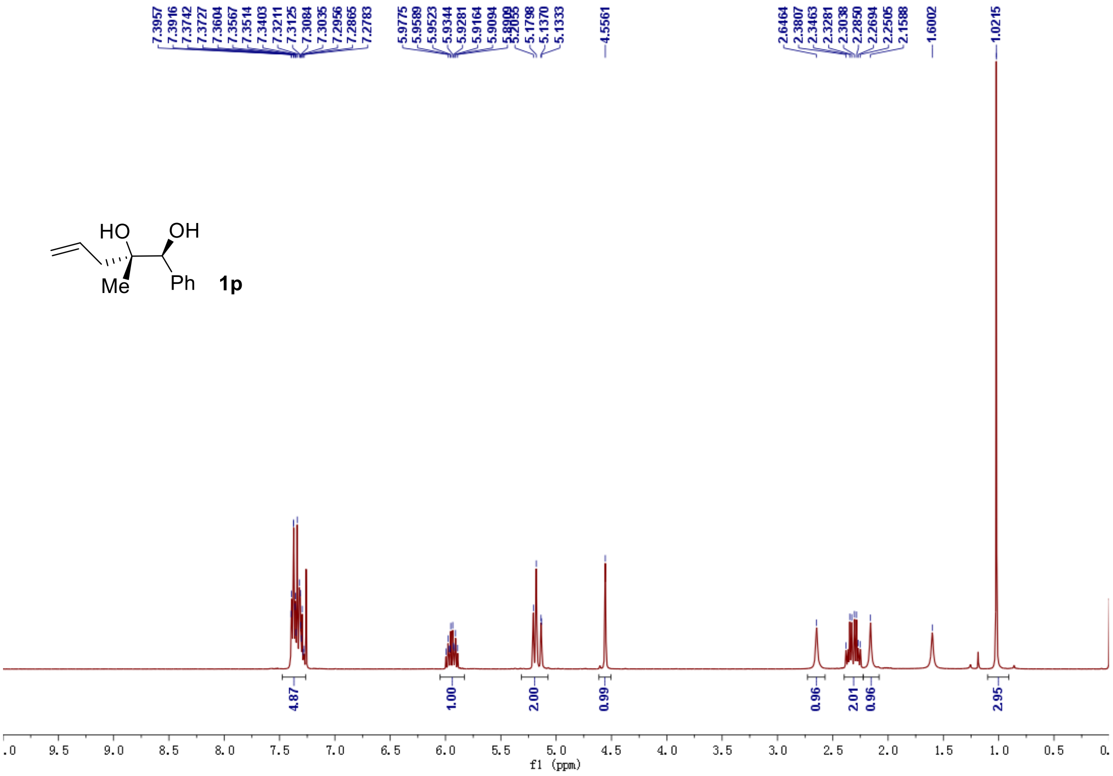

Supplementary Figure 85. ^{13}C NMR sepctra of (1*S*,2*S*)-2-Methyl-1-phenylpent-4-ene-1,2-diol (**1p**)

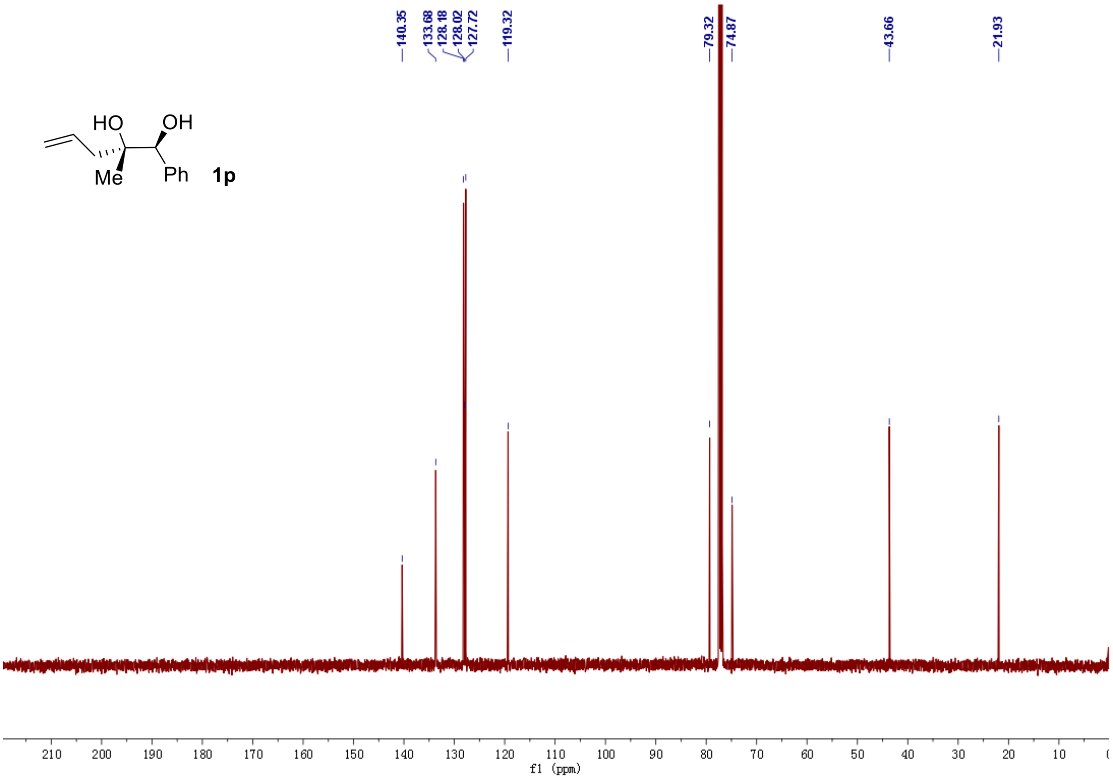

Supplementary Figure 86. ^{1}H NMR sepctra of (1*R*,2*R*)-2-Hydroxy-2-methyl-1-phenylpent-4-en-1-yl propionate (**2p**)

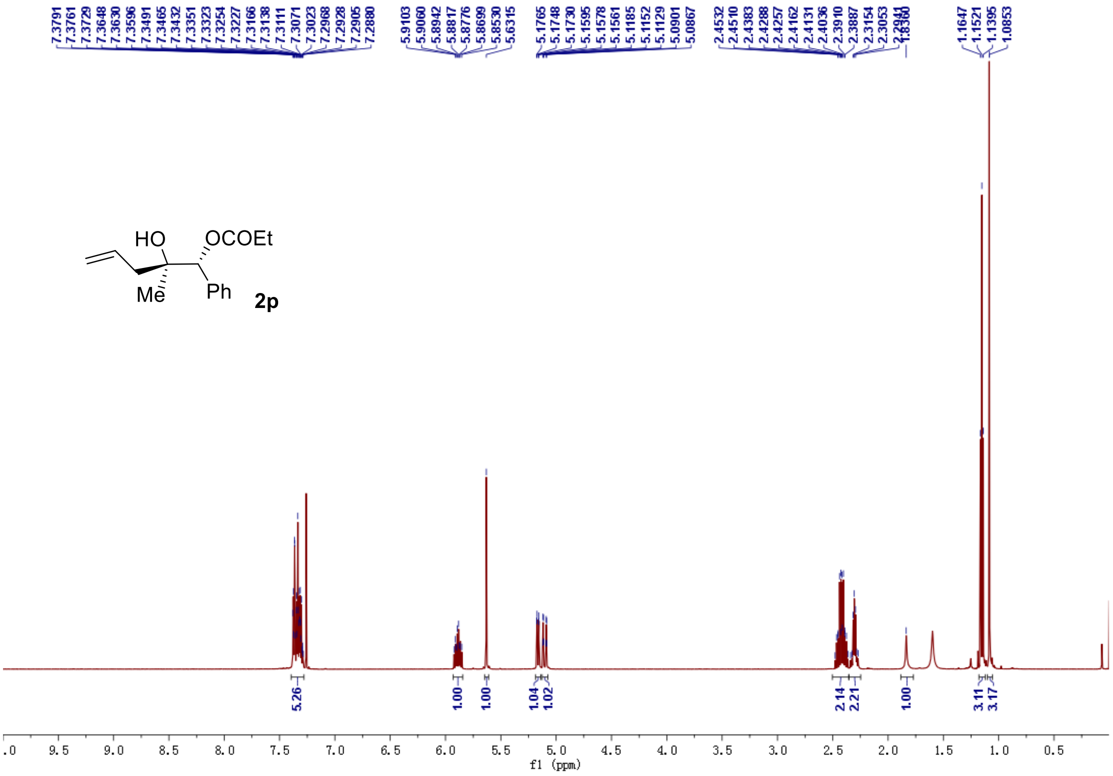

Supplementary Figure 87. ^{13}C NMR sepctra of (1*R*,2*R*)-2-Hydroxy-2-methyl-1-phenylpent-4-en-1-yl propionate (**2p**)

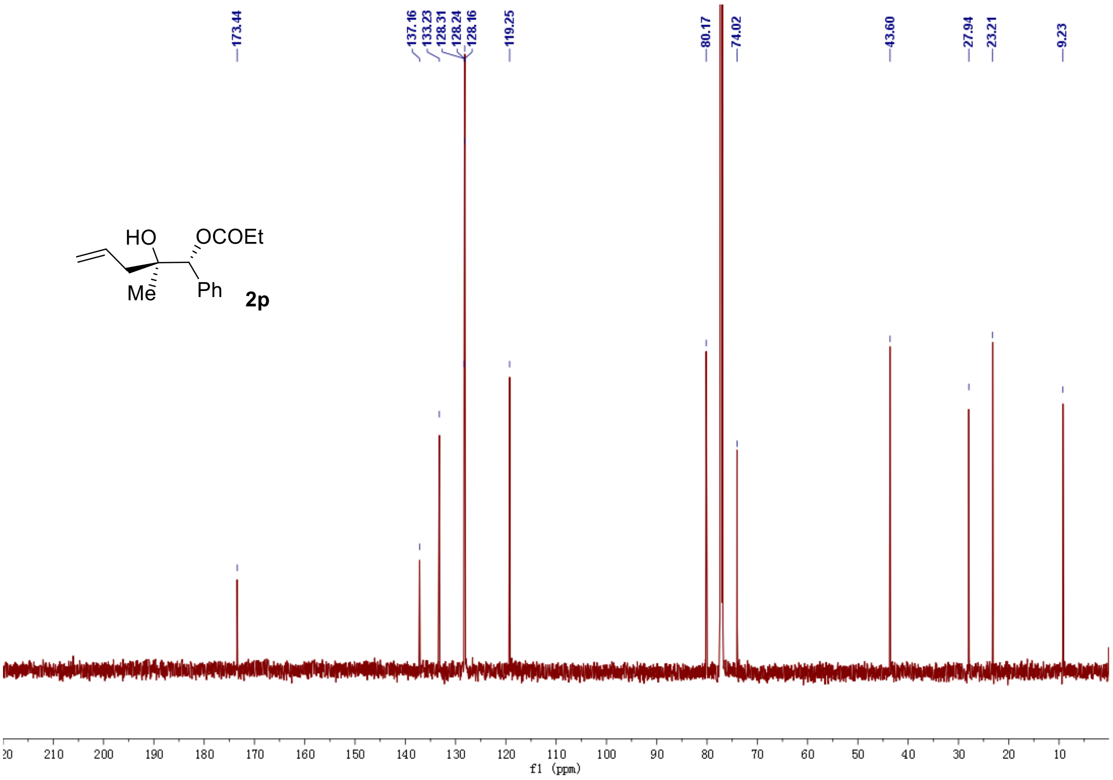

Supplementary Figure 88. ^{1}H NMR sepctra of (1*S*,2*S*)-1-(4-Methoxyphenyl)-2,6-dimethylhept-5-ene-1,2-diol (**1q**)

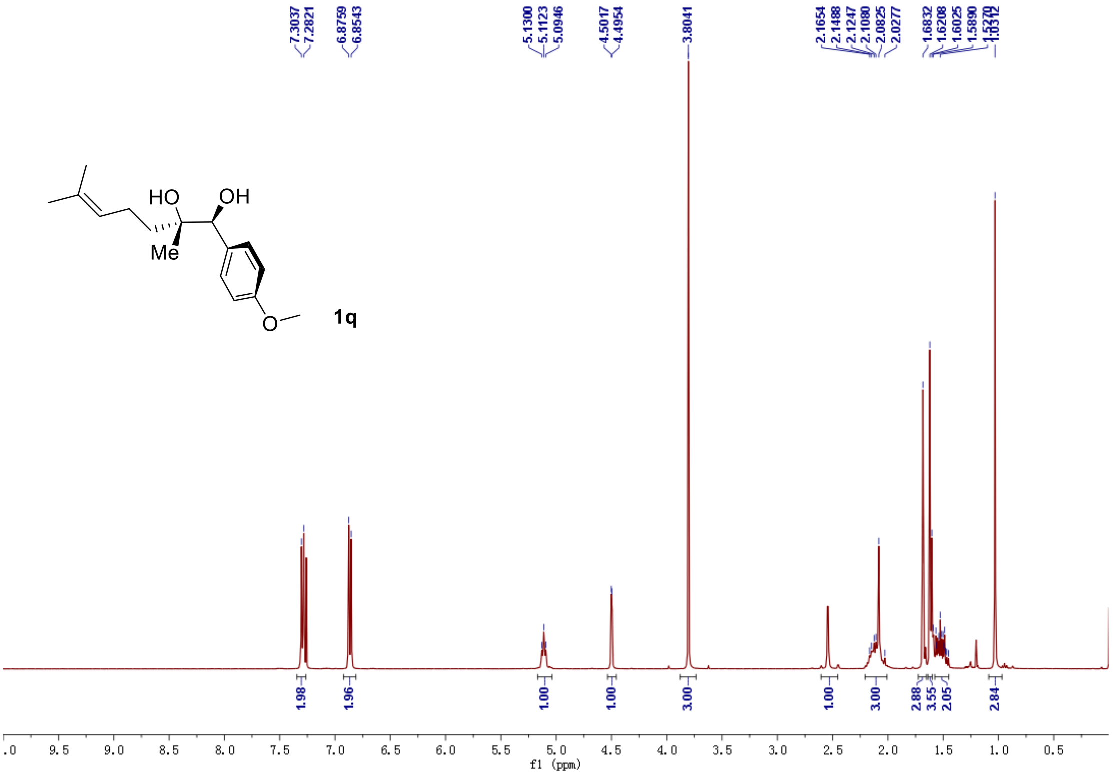

Supplementary Figure 89. ^{13}C NMR sepctra of (1*S*,2*S*)-1-(4-Methoxyphenyl)-2,6-dimethylhept-5-ene-1,2-diol (**1q**)

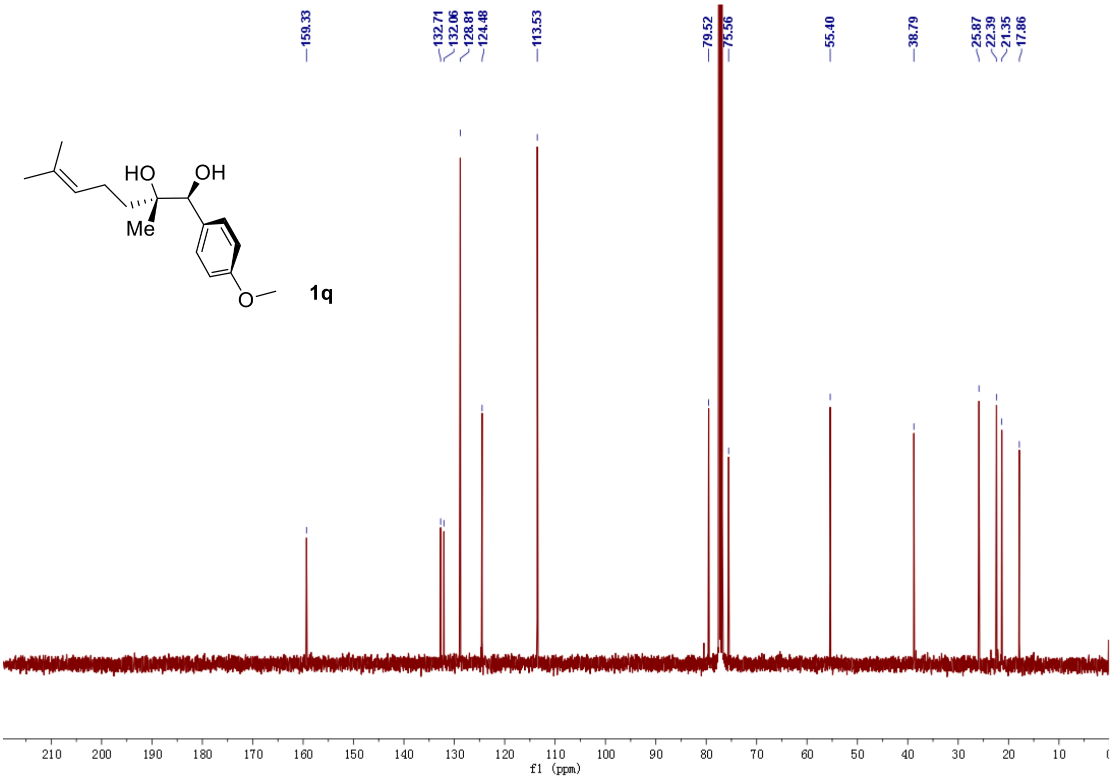

Supplementary Figure 90. ^{1}H NMR sepctra of (1*R*,2*R*)-2-Hydroxy-1-(4-methoxyphenyl)-2,6-dimethylhept-5-en-1-yl propionate (**2q**)

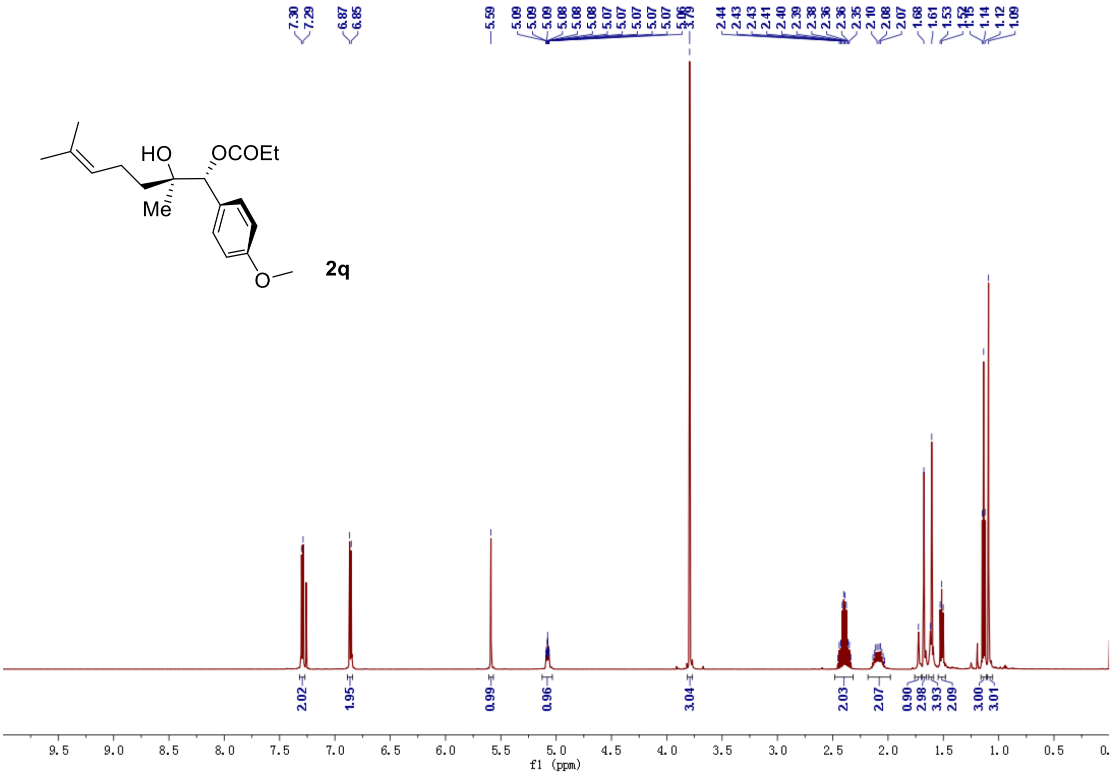

Supplementary Figure 91. ^{13}C NMR sepctra of (1*R*,2*R*)-2-Hydroxy-1-(4-methoxyphenyl)-2,6-dimethylhept-5-en-1-yl propionate (**2q**)

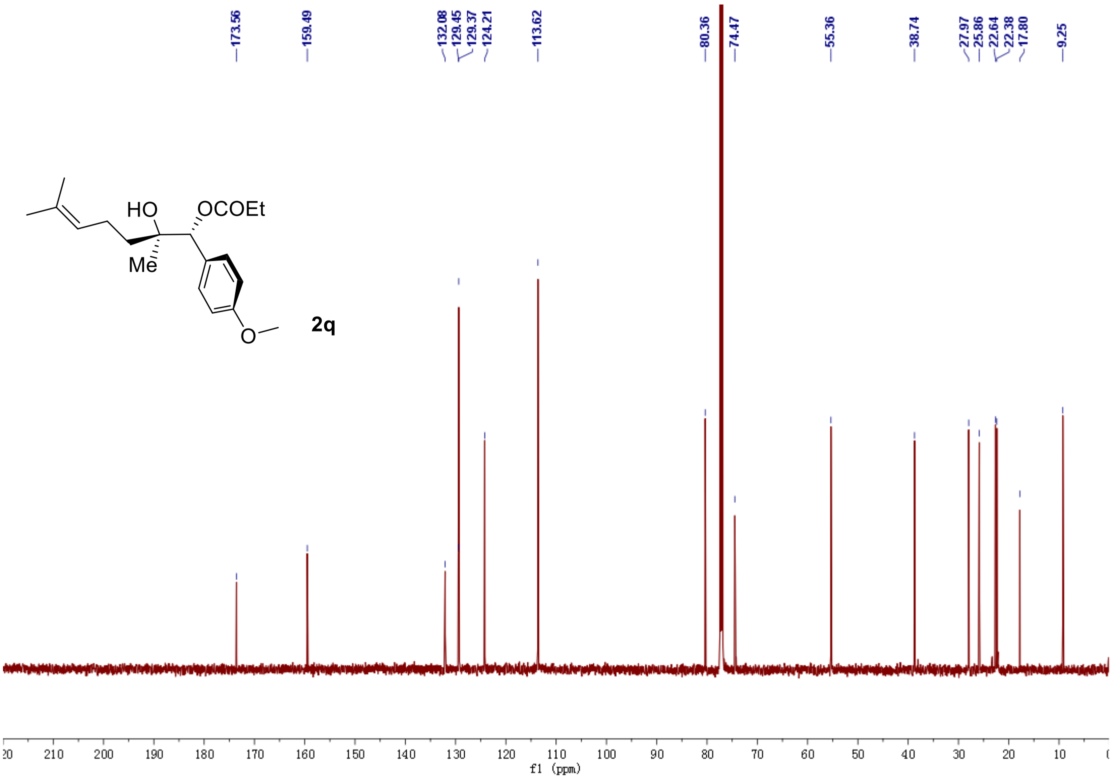

Supplementary Figure 92. ^{1}H NMR sepctra of (1*S*,2*R*)-2-Ethyl-1-phenylhexane-1,2-diol (**1r**)

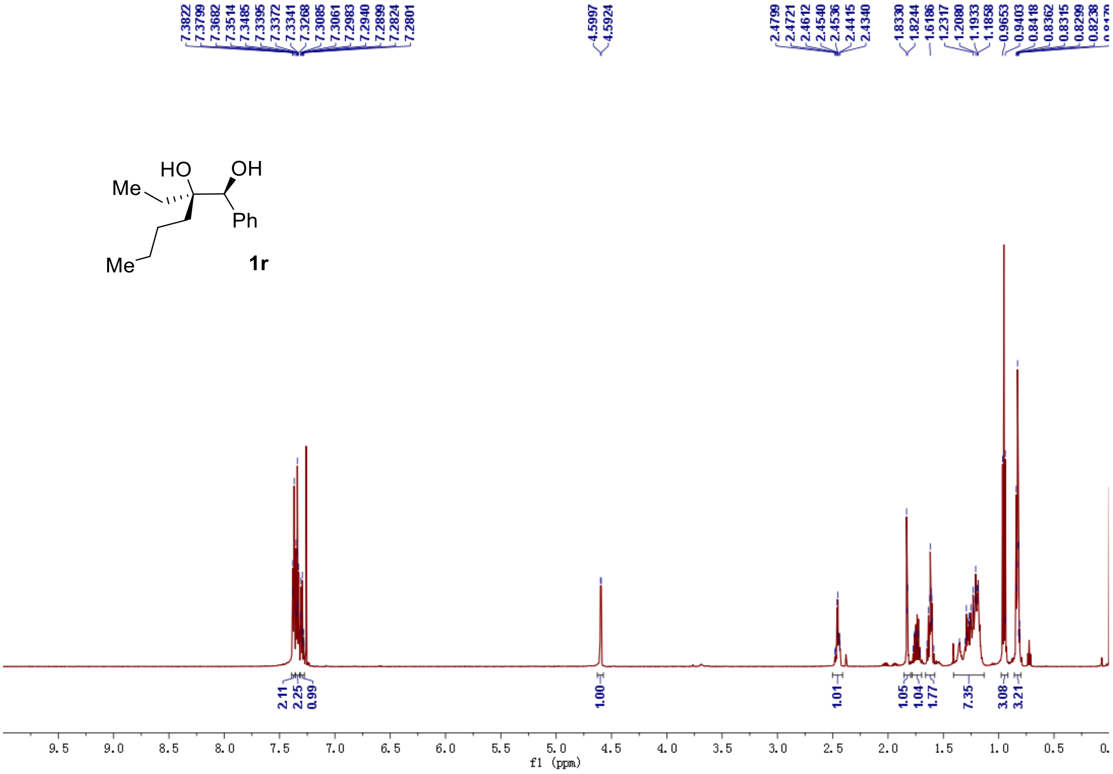

Supplementary Figure 93. ^{13}C NMR sepctra of (1*S*,2*R*)-2-Ethyl-1-phenylhexane-1,2-diol (**1r**)

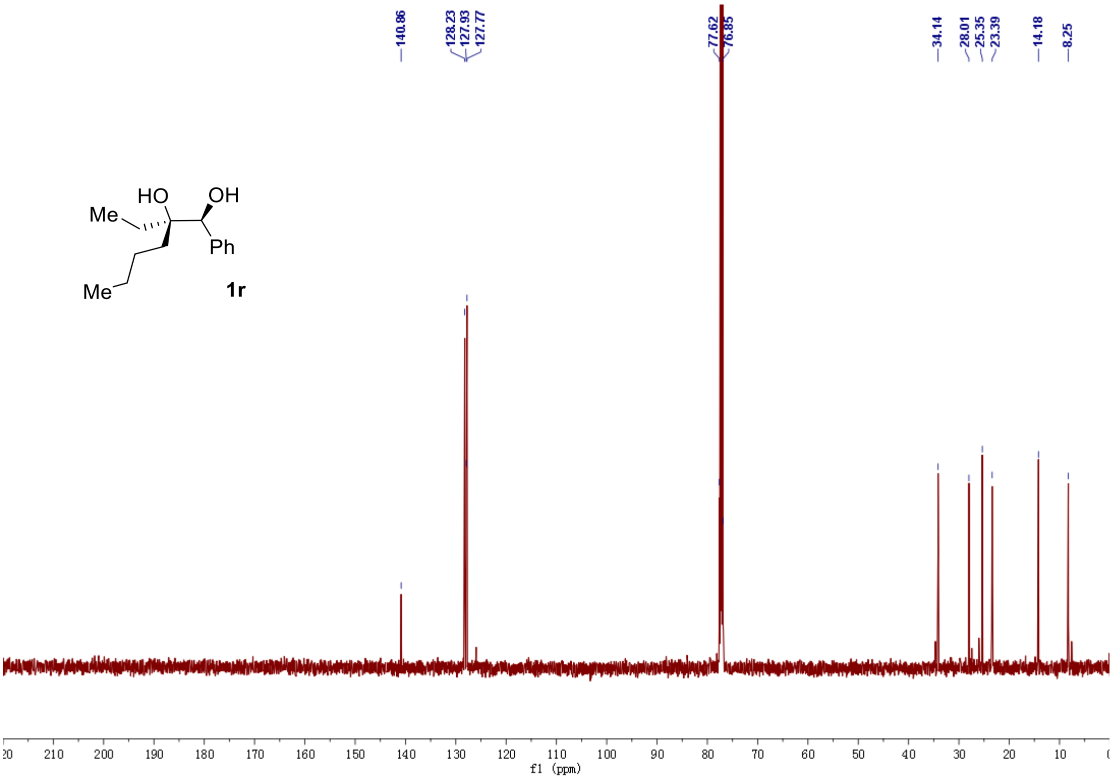

Supplementary Figure 94. ^{1}H NMR sepctra of (1*R*,2*S*)-2-Ethyl-2-hydroxy-1-phenylhexyl propionate (**2r**)

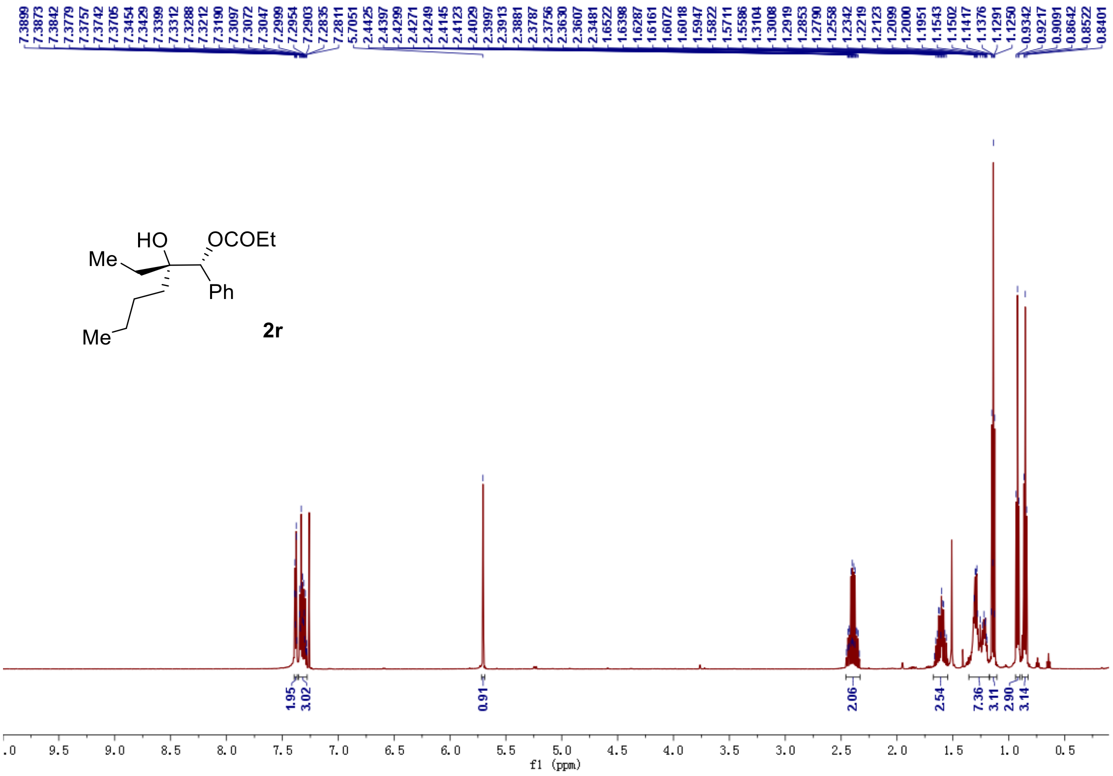

Supplementary Figure 95. ^{13}C NMR sepctra of (1*R*,2*S*)-2-Ethyl-2-hydroxy-1-phenylhexyl propionate (**2r**)

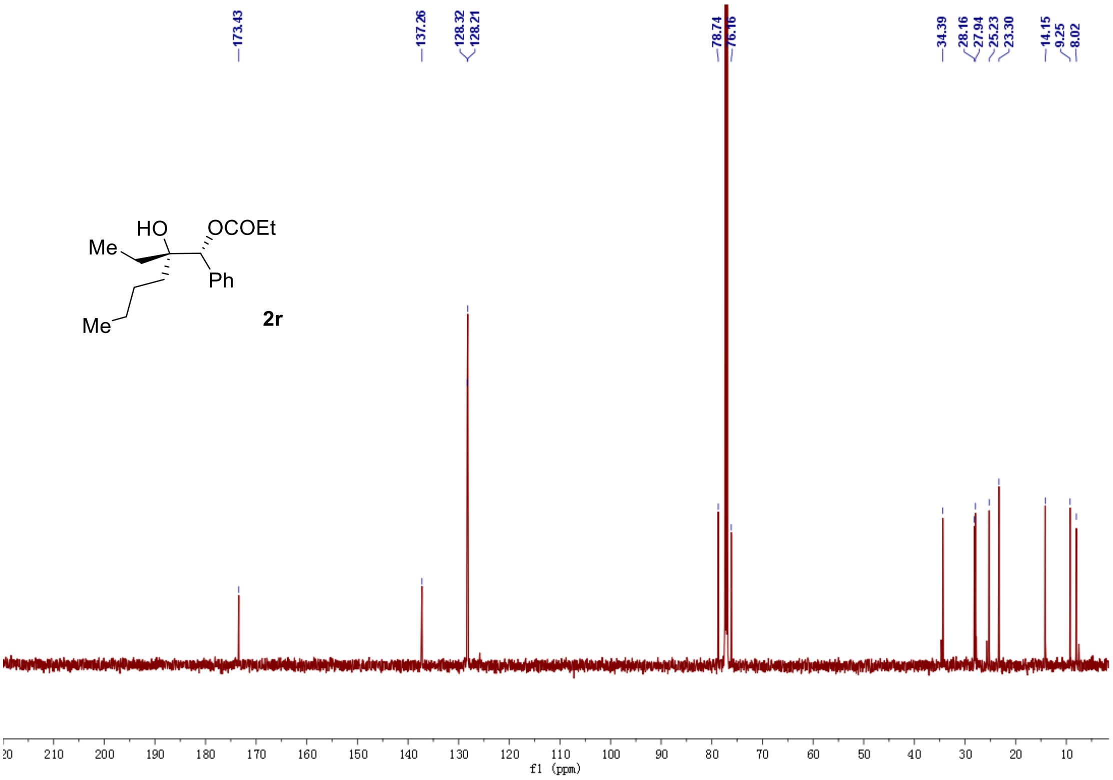

Supplementary Figure 96. ^{1}H NMR sepctra of (1*S*,2*S*)-2-Benzyl-1-phenylbutane-1,2-diol (**1s**)

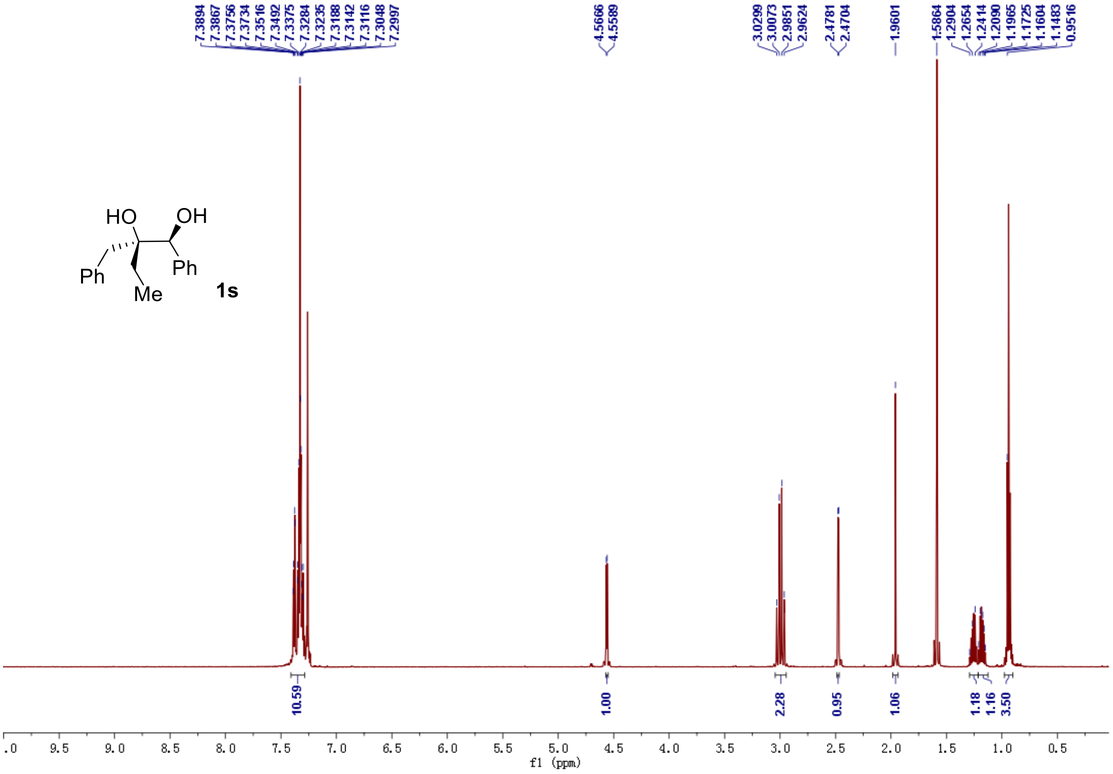

Supplementary Figure 97. ^{13}C NMR sepctra of (1*S*,2*S*)-2-Benzyl-1-phenylbutane-1,2-diol (**1s**)

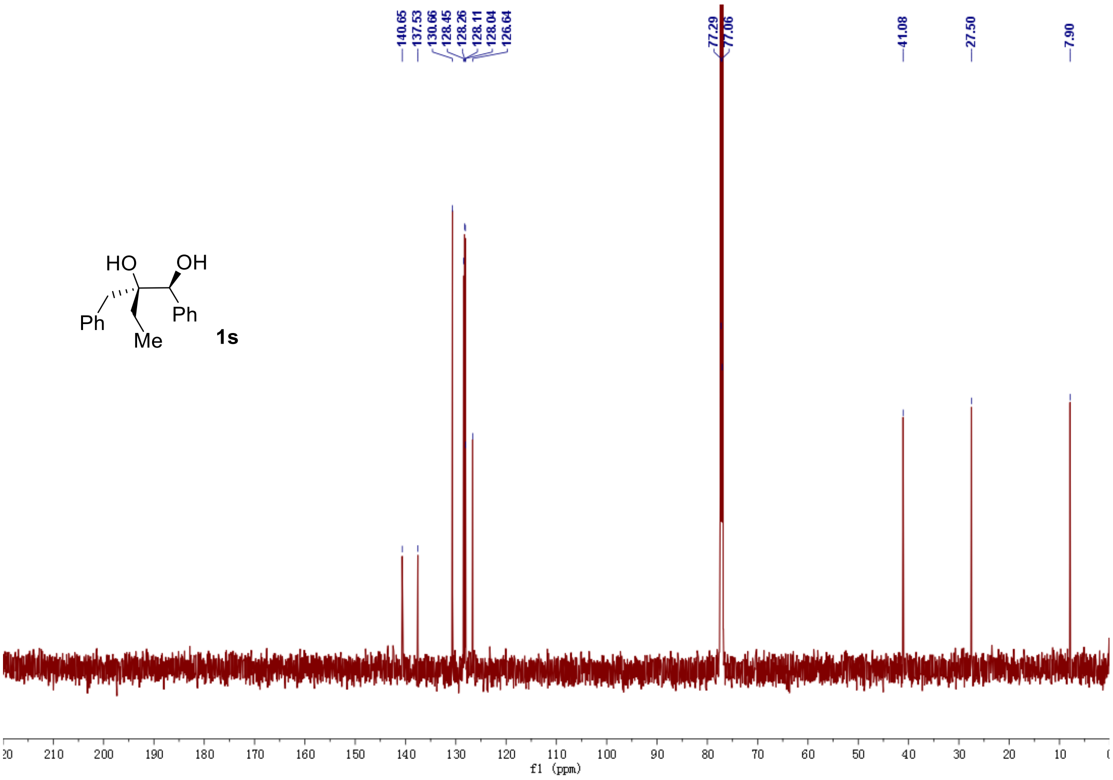

Supplementary Figure 98. ^{1}H NMR sepctra of (1*R*,2*R*)-2-Benzyl-2-hydroxy-1-phenylbutyl propionate (**2s**)

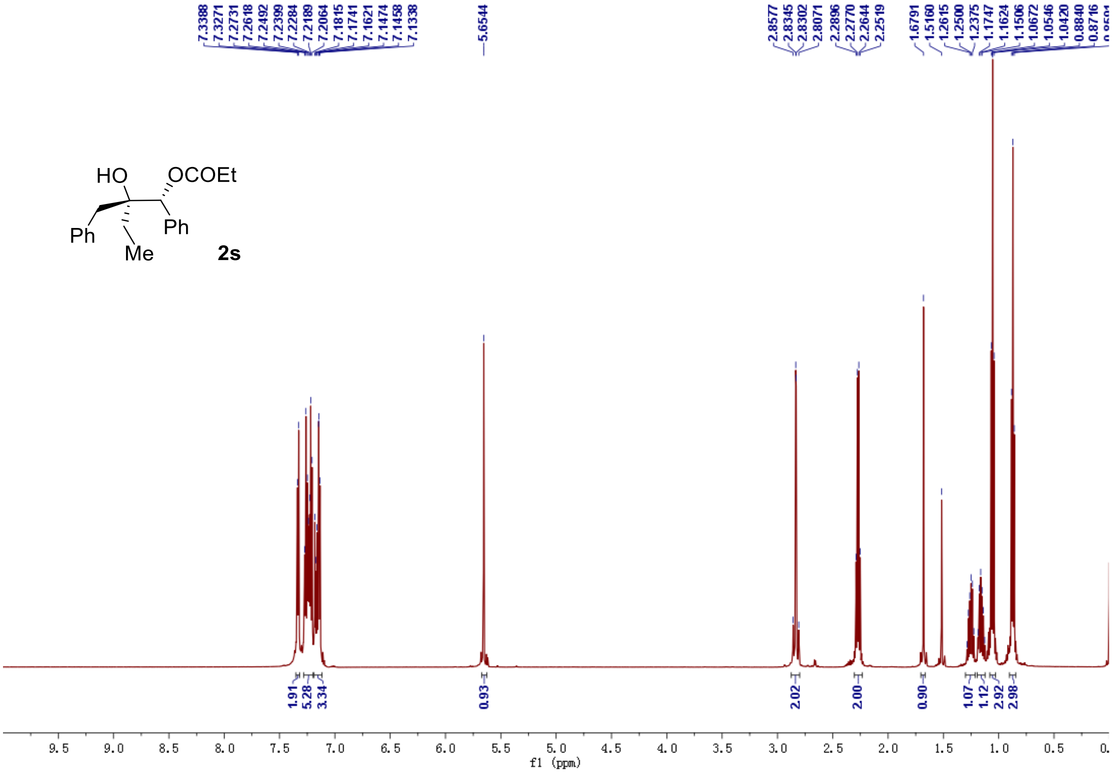

Supplementary Figure 99. ^{13}C NMR sepctra of (1*R*,2*R*)-2-Benzyl-2-hydroxy-1-phenylbutyl propionate (**2s**)

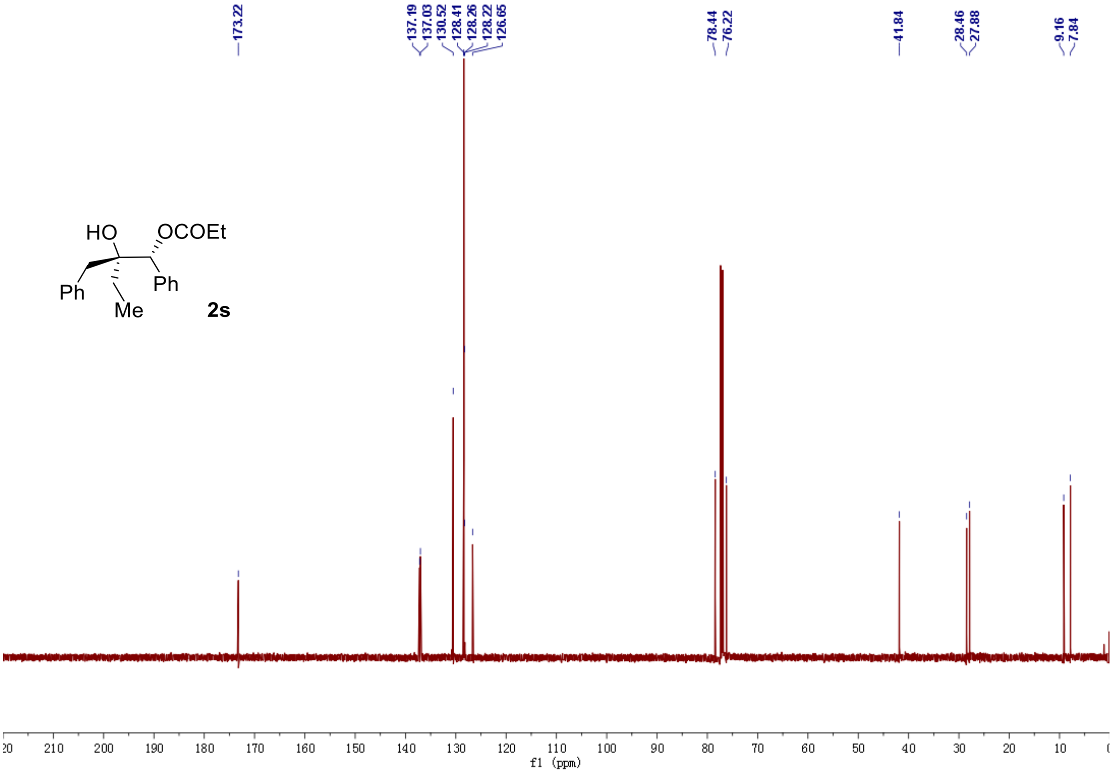

Supplementary Figure 100. ^{1}H NMR sepctra of (1*S*,2*S*)-2-Benzyl-1,4-diphenylbutane-1,2-diol (**1t**)

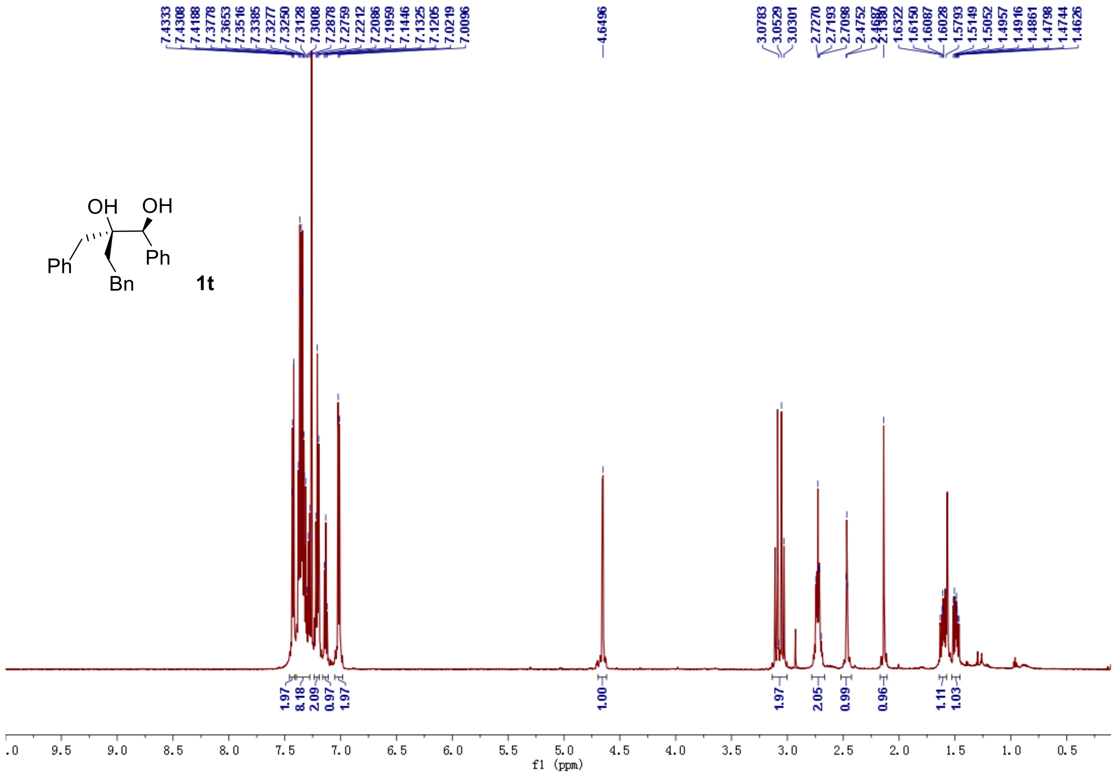

Supplementary Figure 101. ^{13}C NMR sepctra of (1*S*,2*S*)-2-Benzyl-1,4-diphenylbutane-1,2-diol (**1t**)

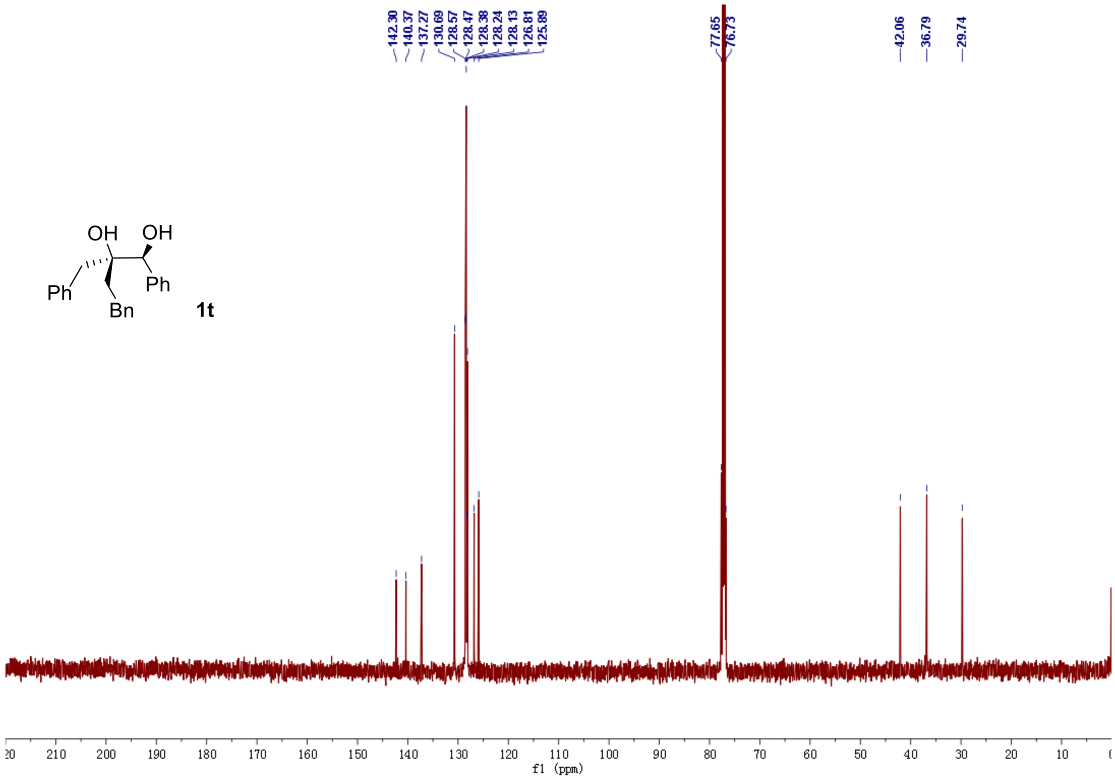

Supplementary Figure 102. ^{1}H NMR sepctra of (1*R*,2*R*)-2-Benzyl-2-hydroxy-1,4-diphenylbutyl propionate (**2t**)

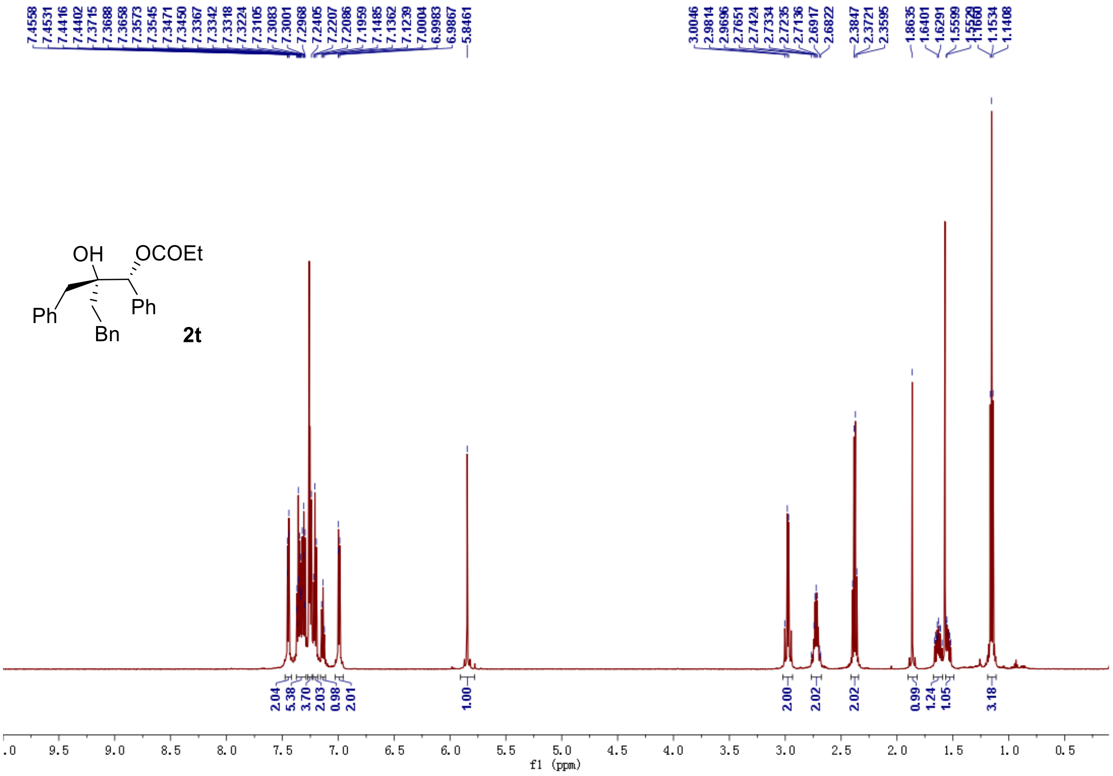

Supplementary Figure 103. ^{1}H NMR sepctra of (1*R*,2*R*)-2-Benzyl-2-hydroxy-1,4-diphenylbutyl propionate (**2t**)

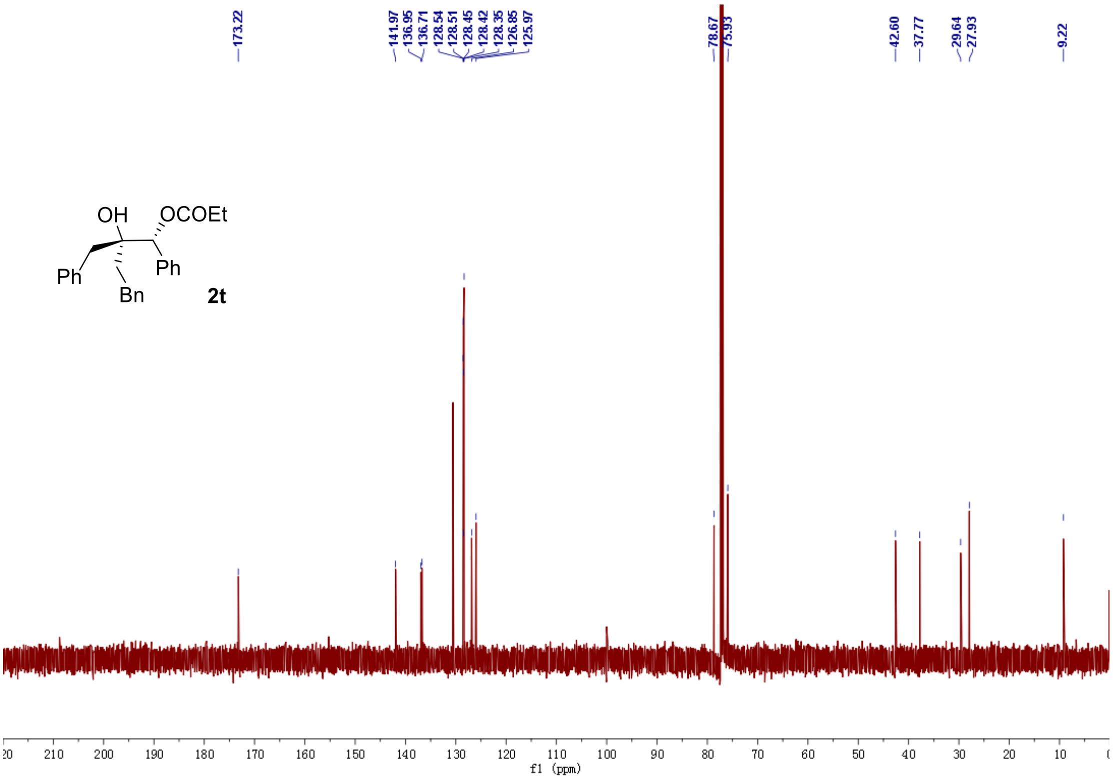

Supplementary Figure 104. ^{1}H NMR sepctra of (1*S*,2*S*)-2-(4-Methylbenzyl)-1,4-diphenylbutane-1,2-diol (**1u**)

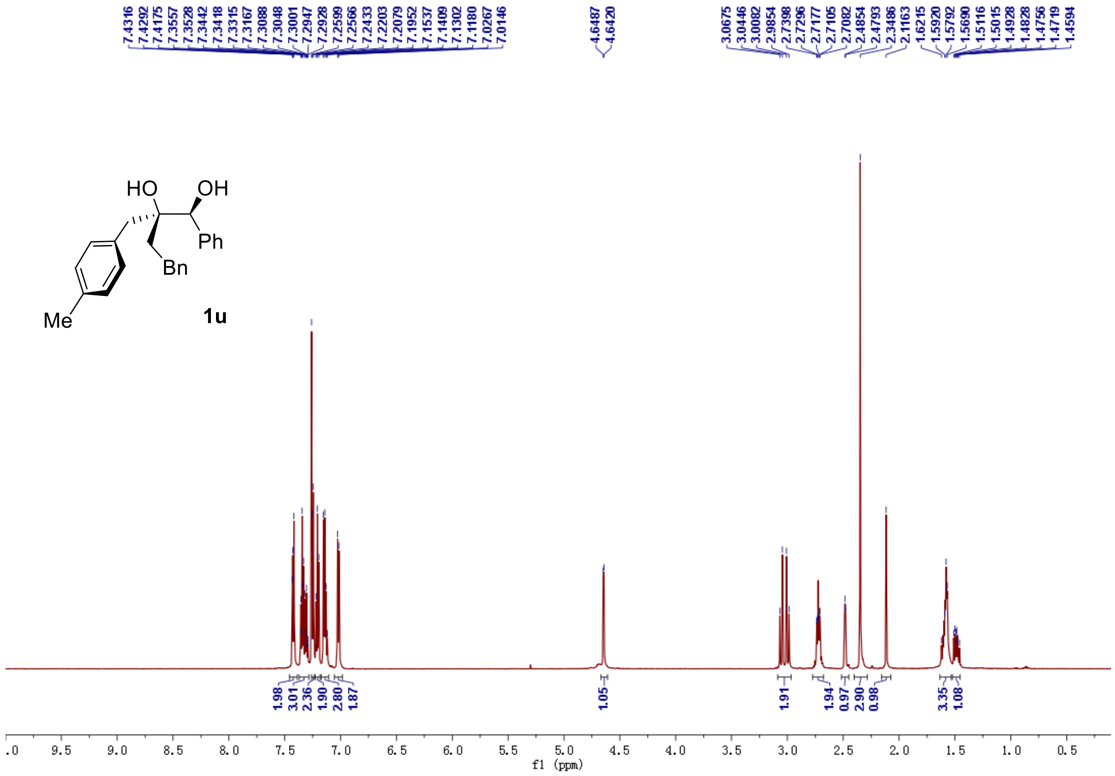

Supplementary Figure 105. ^{13}C NMR sepctra of (1*S*,2*S*)-2-(4-Methylbenzyl)-1,4-diphenylbutane-1,2-diol (**1u**)

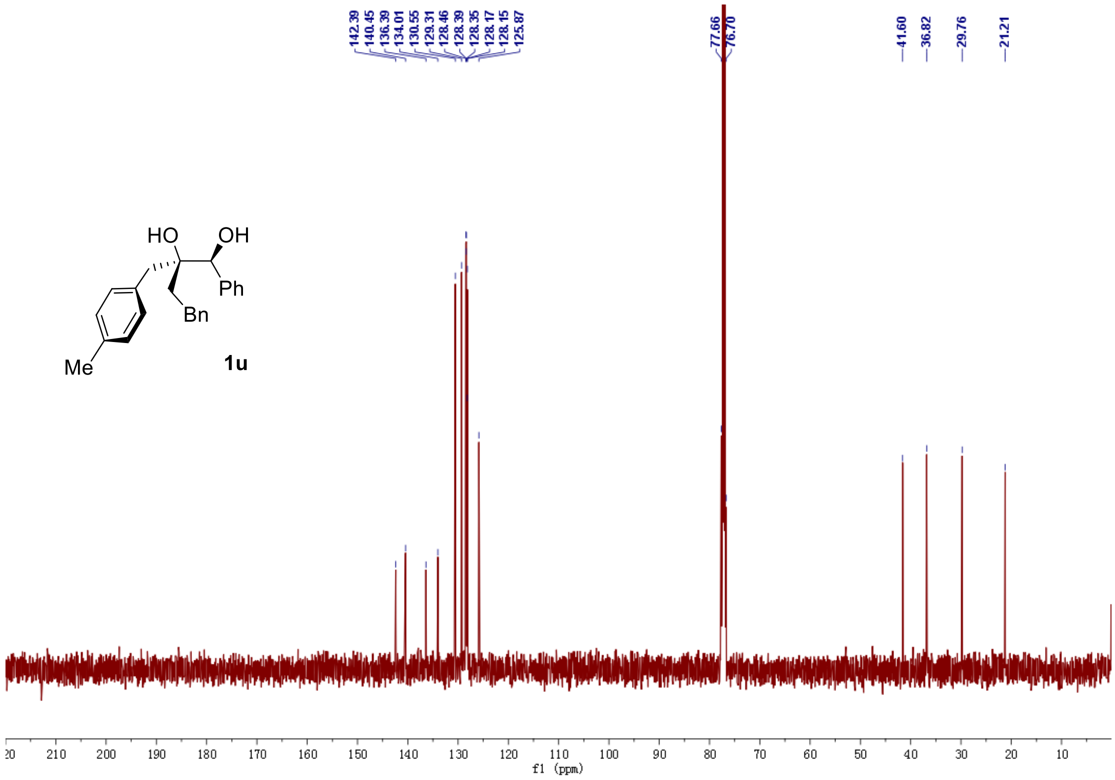

Supplementary Figure 106. ^{1}H NMR sepctra of (1*R*,2*R*)-2-Hydroxy-2-(4-methylbenzyl)-1,4-diphenylbutyl propionate (**2u**)

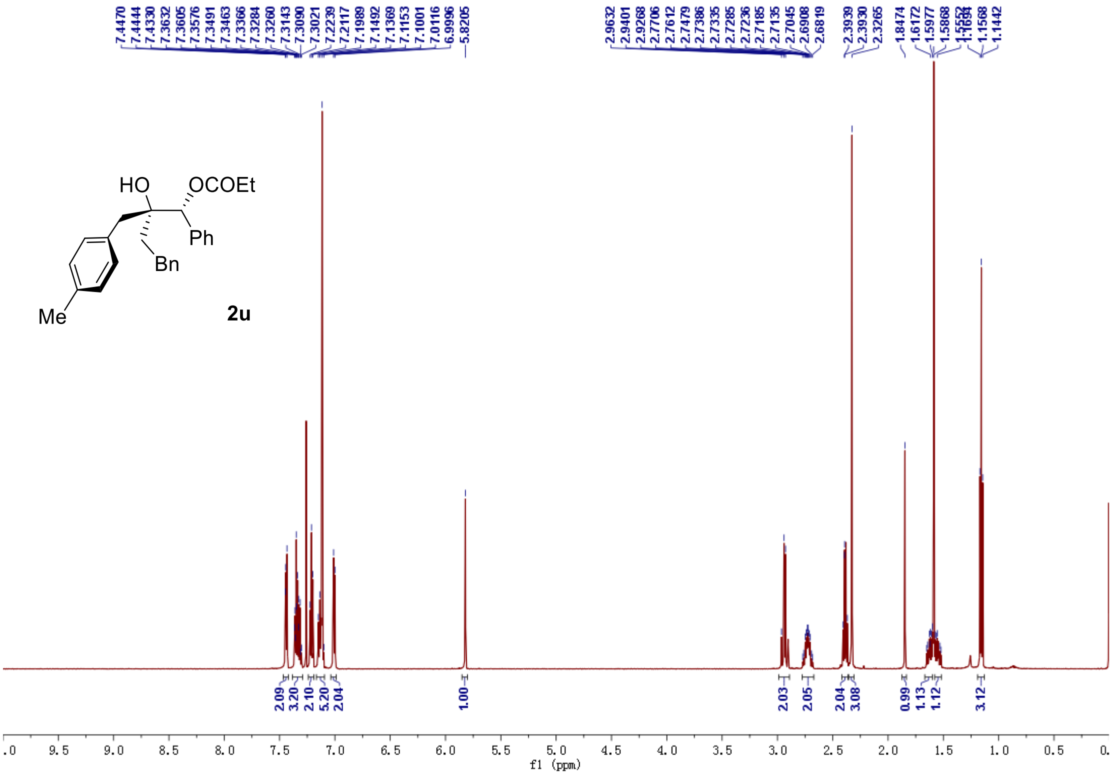

Supplementary Figure 107. ^{13}C NMR sepctra of (1*R*,2*R*)-2-Hydroxy-2-(4-methylbenzyl)-1,4-diphenylbutyl propionate (**2u**)

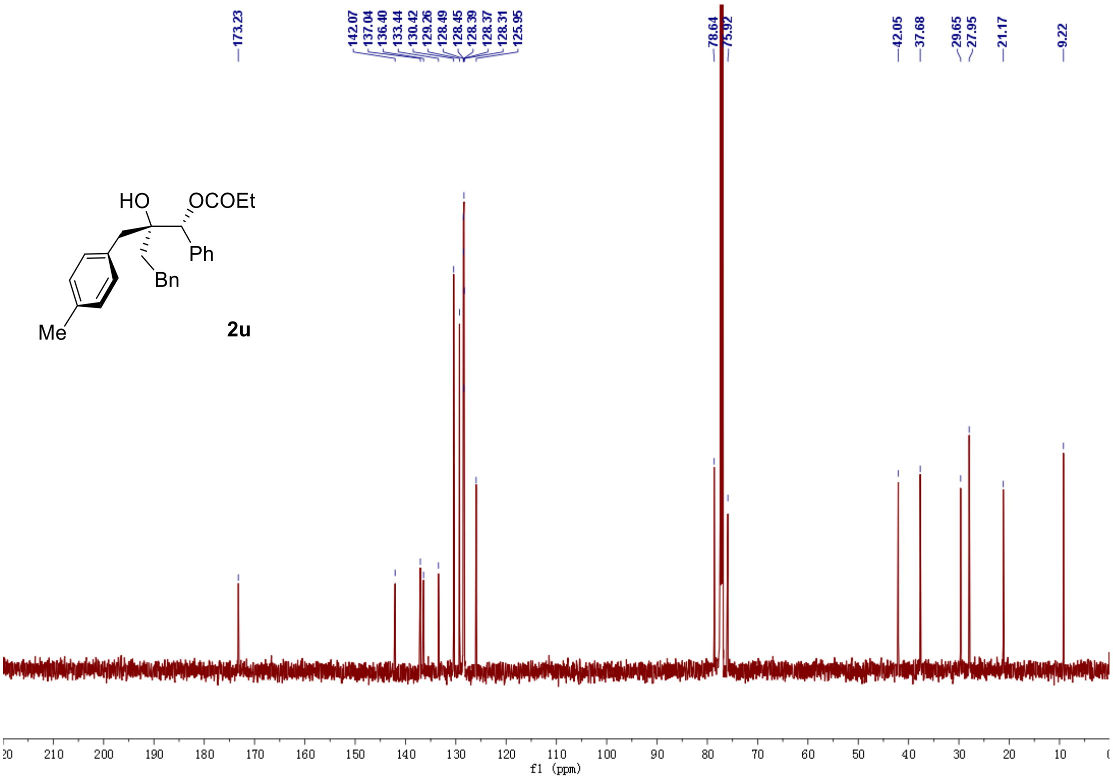

Supplementary Figure 108. ^{1}H NMR sepctra of (1*S*,2*R*)-2-Phenethyl-1-phenylhexane-1,2-diol (**1v**)

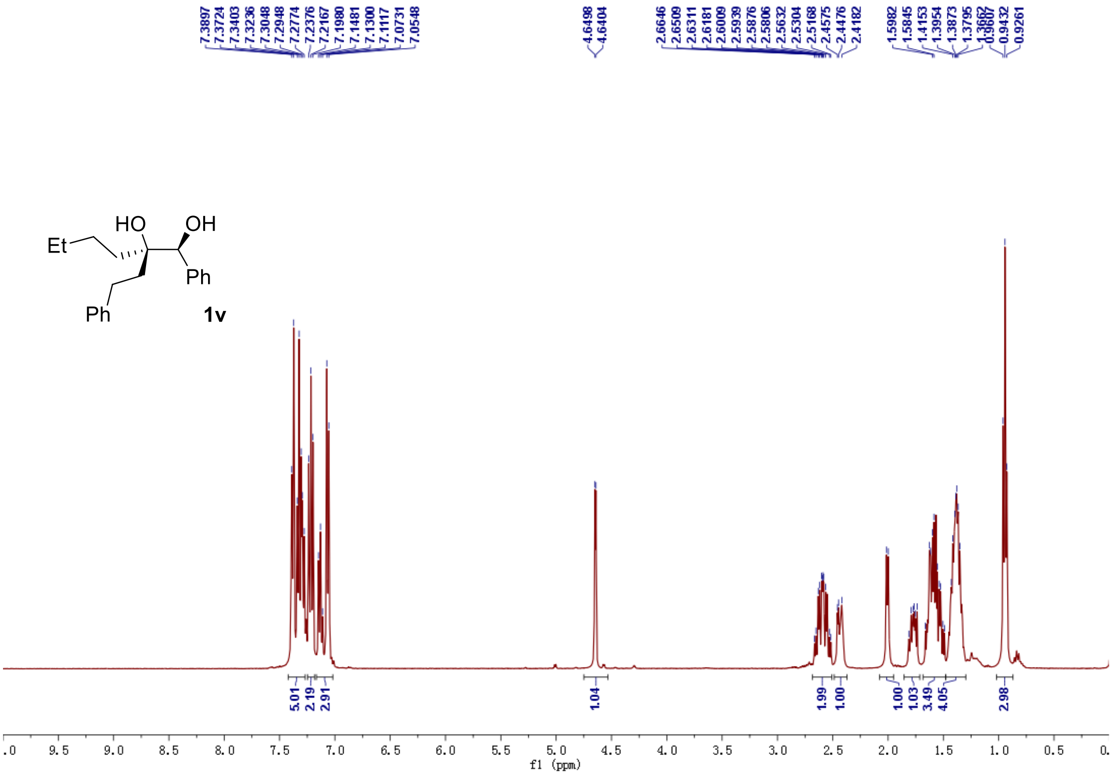

Supplementary Figure 109. ^{13}C NMR sepctra of (1*S*,2*R*)-2-Phenethyl-1-phenylhexane-1,2-diol (**1v**)

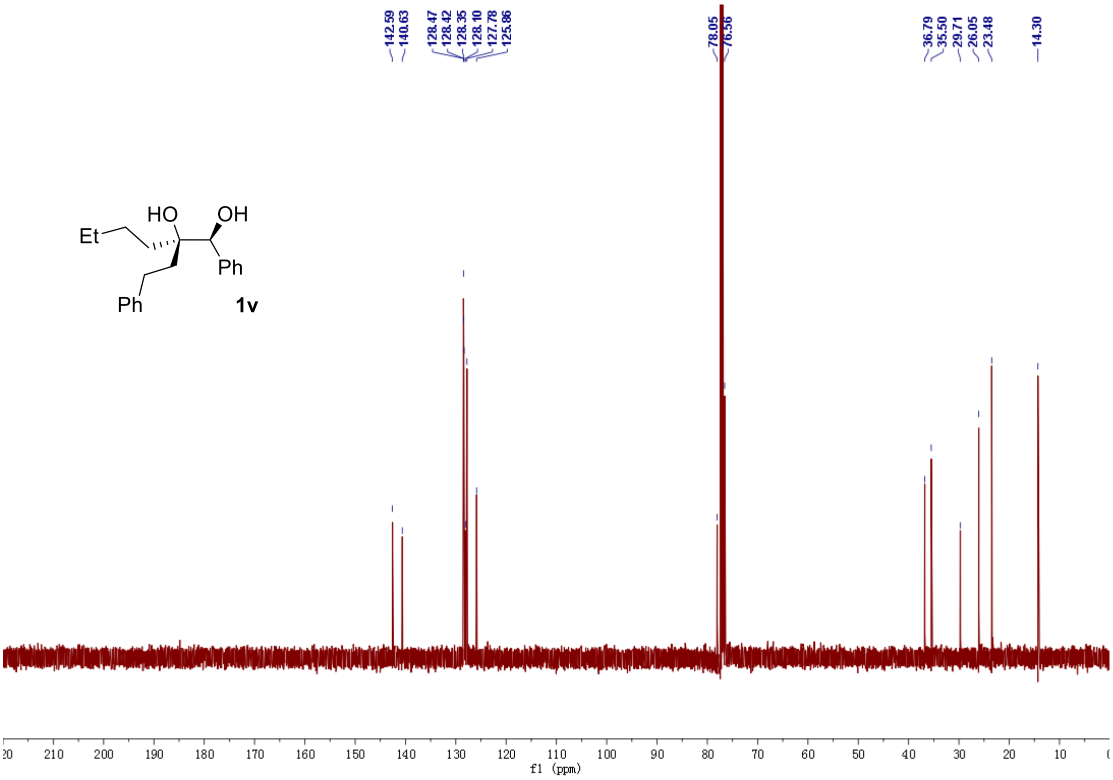

Supplementary Figure 110. ^{1}H NMR sepctra of (1*R*,2*S*)-2-Hydroxy-2-phenethyl-1-phenylhexyl propionate (**2v**)

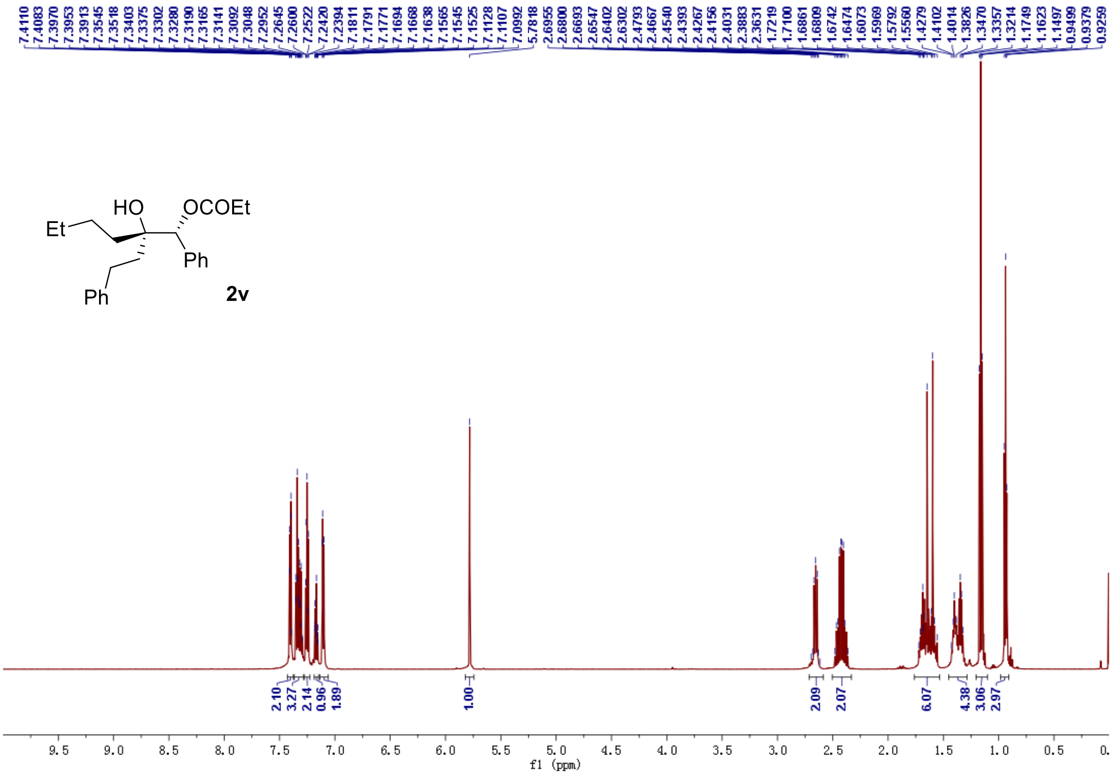

Supplementary Figure 111. ^{13}C NMR sepctra of (1*R*,2*S*)-2-Hydroxy-2-phenethyl-1-phenylhexyl propionate (**2v**)

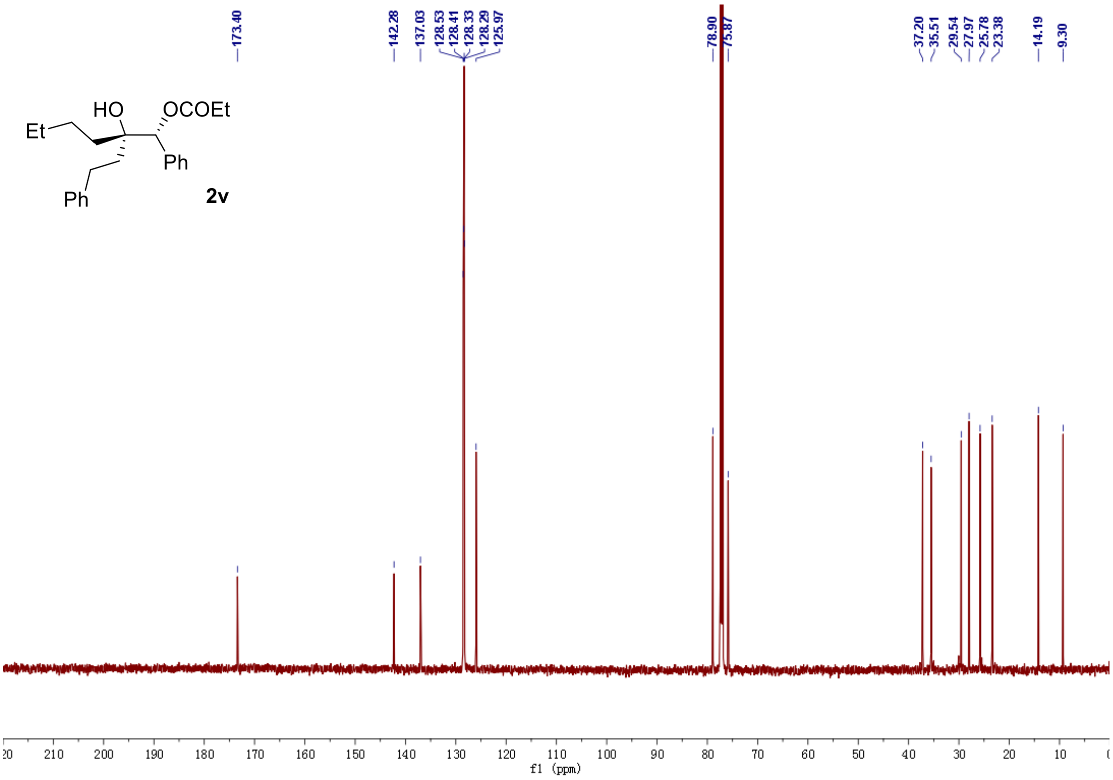

Supplementary Figure 112. ^{1}H NMR sepctra of (1*S*,2*R*)-2-(*tert*-Butyl)-1-phenylhexane-1,2-diol (**1w**)

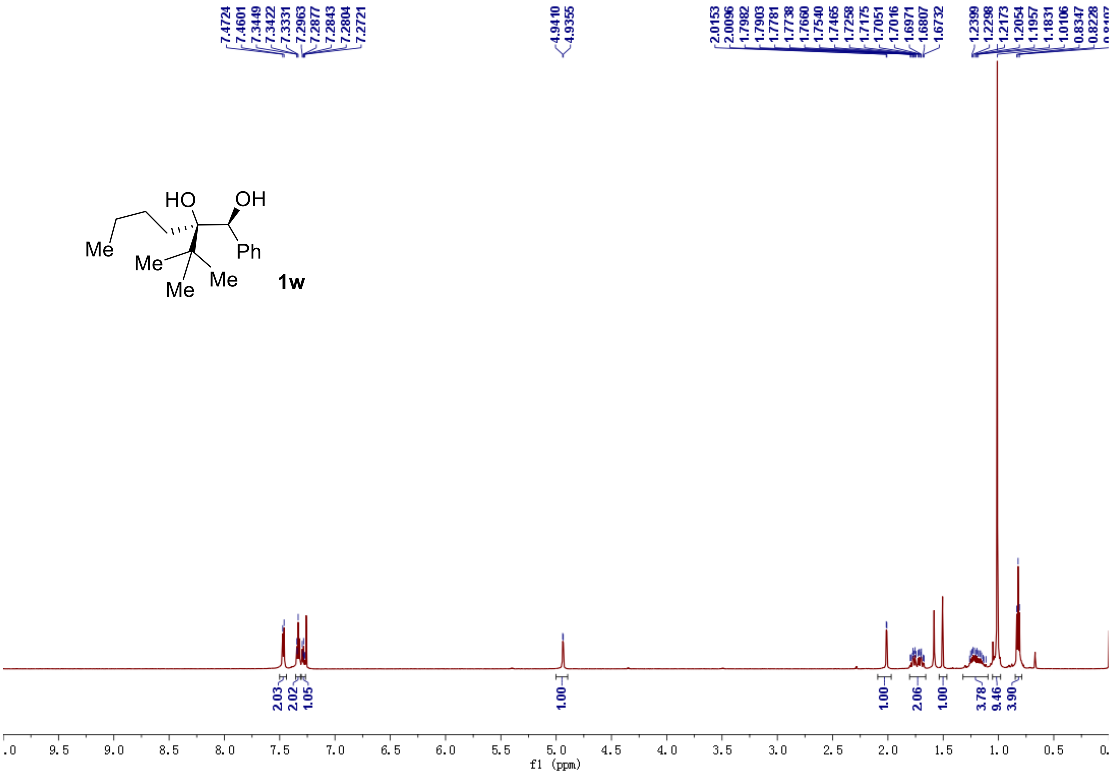

Supplementary Figure 113. ^{13}C NMR sepctra of (1*S*,2*R*)-2-(*tert*-Butyl)-1-phenylhexane-1,2-diol (**1w**)

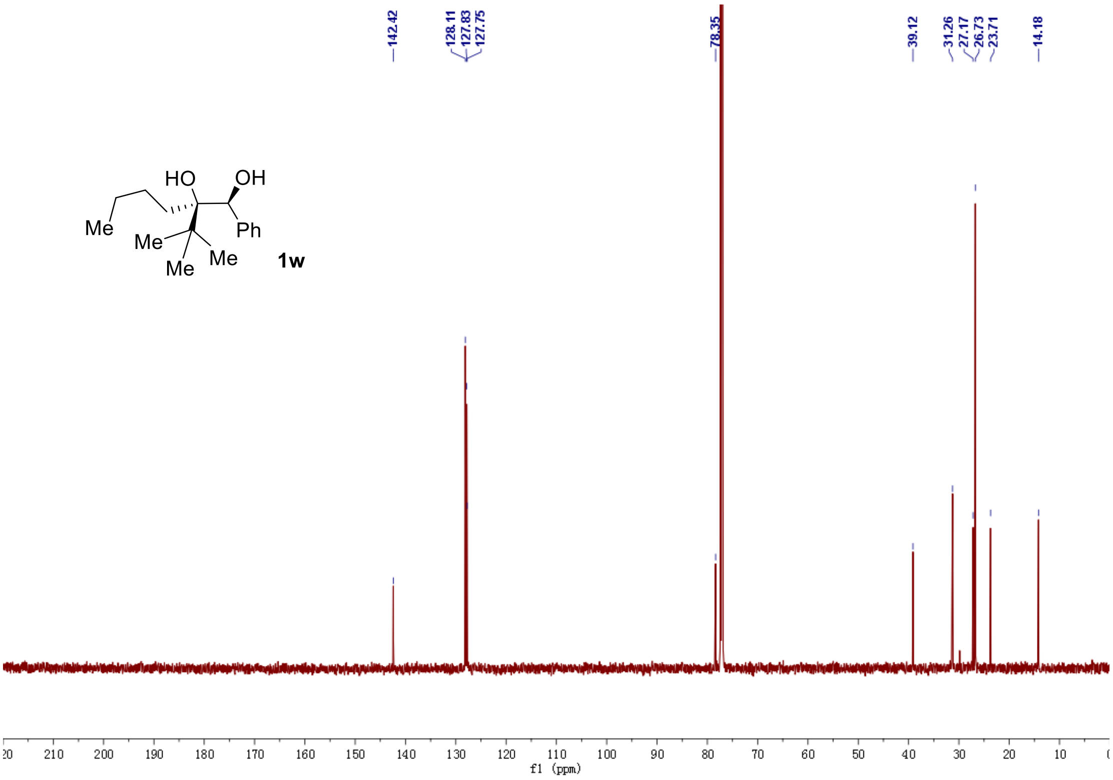

Supplementary Figure 114. ^{1}H NMR sepctra of (1*R*,2*S*)-2-(*tert*-Butyl)-2-hydroxy-1-phenylhexyl propionate (**2w**)

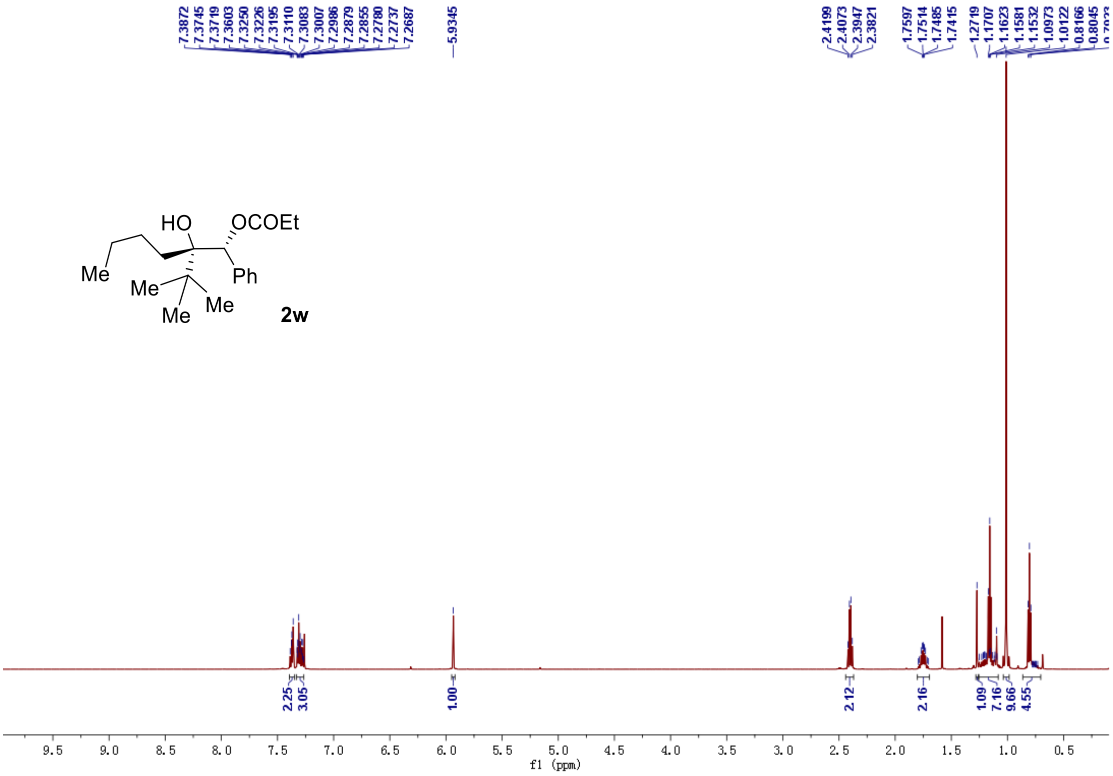

Supplementary Figure 115. ^{13}C NMR sepctra of (1*R*,2*S*)-2-(*tert*-Butyl)-2-hydroxy-1-phenylhexyl propionate (**2w**)

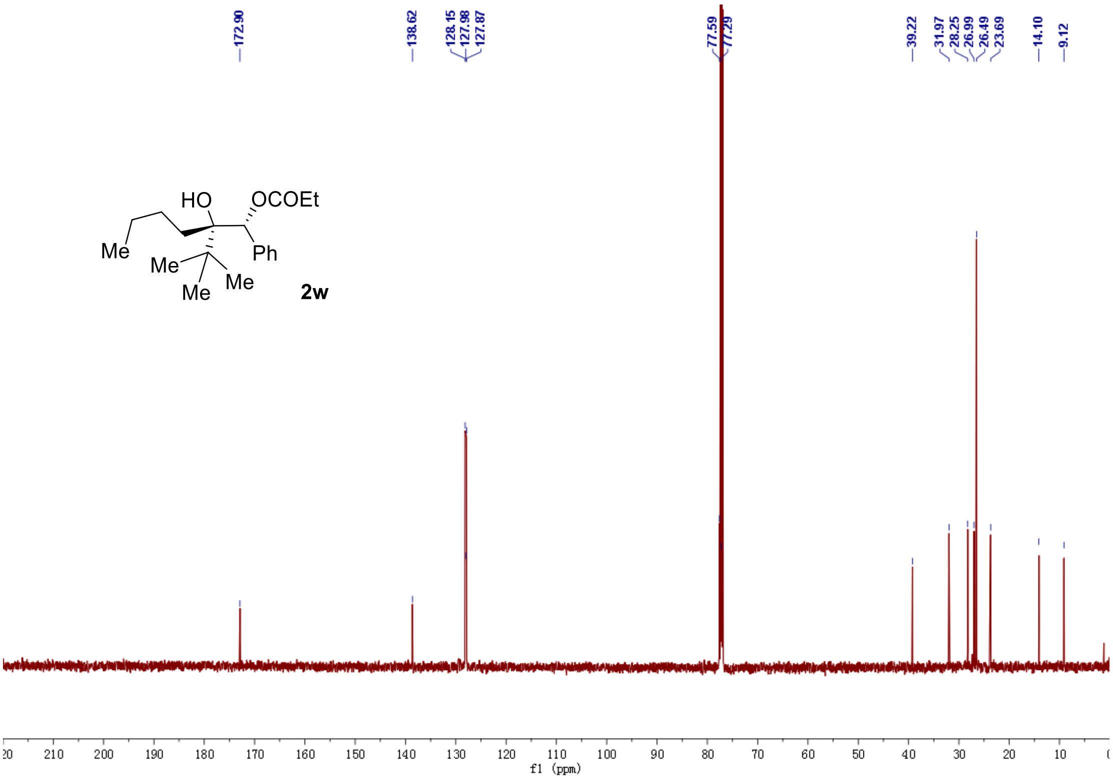

Supplementary Figure 116. ^{1}H NMR sepctra of (1*S*,2*S*)-2-*iso*-Propyl-3,3-dimethyl-1-phenylbutane-1,2-diol (**1x**)

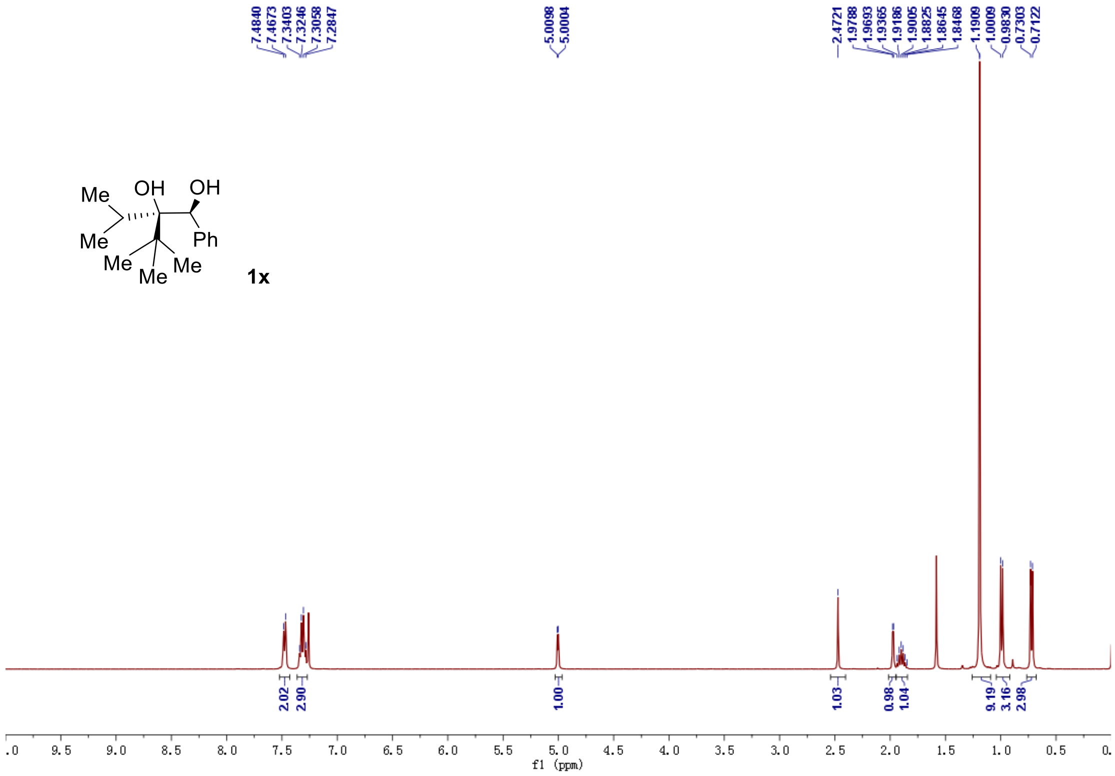

Supplementary Figure 117. ^{13}C NMR sepctra of (1*S*,2*S*)-2-*iso*-Propyl-3,3-dimethyl-1-phenylbutane-1,2-diol (**1x**)

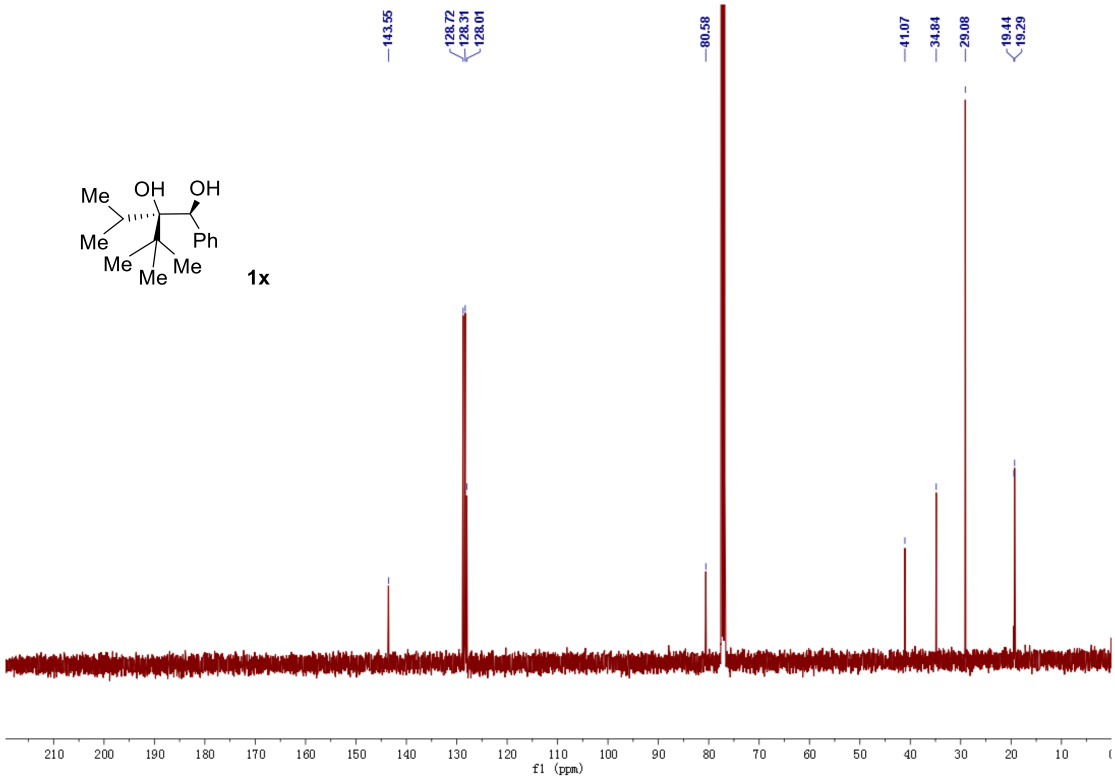

Supplementary Figure 118. ^{1}H NMR sepctra of (1*R*,2*R*)-2-Hydroxy-2-*iso*-propyl-3,3-dimethyl-1-phenylbutyl propionate (**2x**)

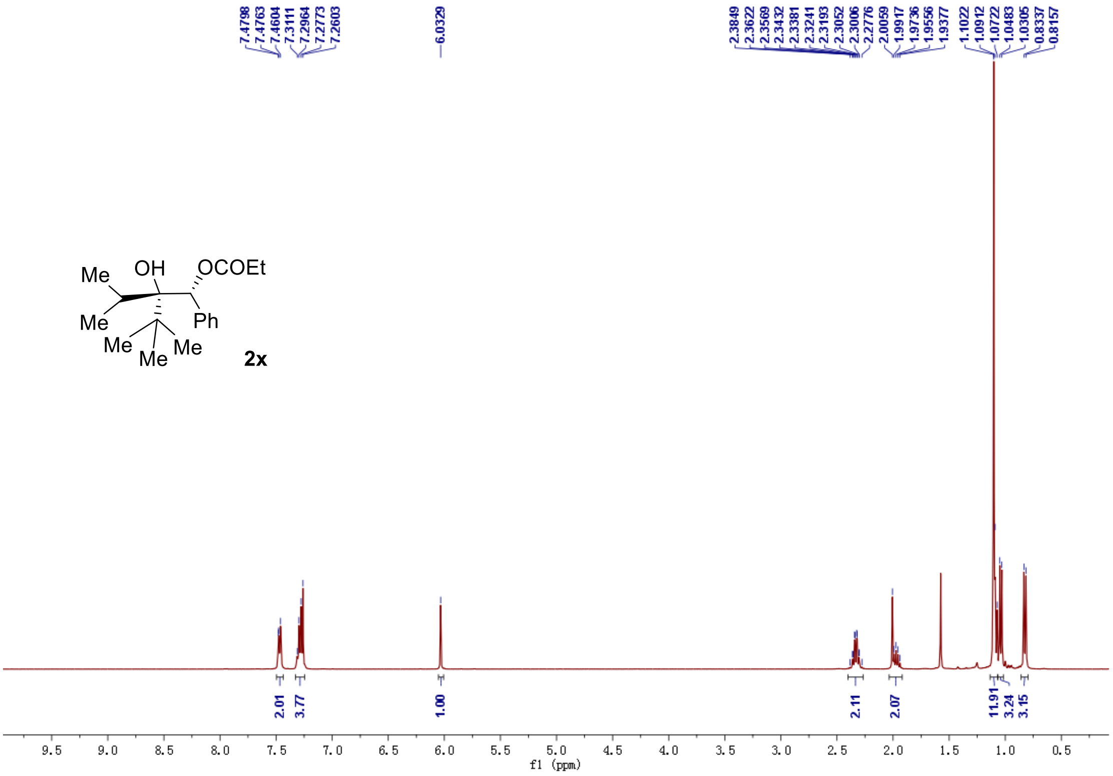

Supplementary Figure 119. ^{13}C NMR sepctra of (1*R*,2*R*)-2-Hydroxy-2-*iso*-propyl-3,3-dimethyl-1-phenylbutyl propionate (**2x**)

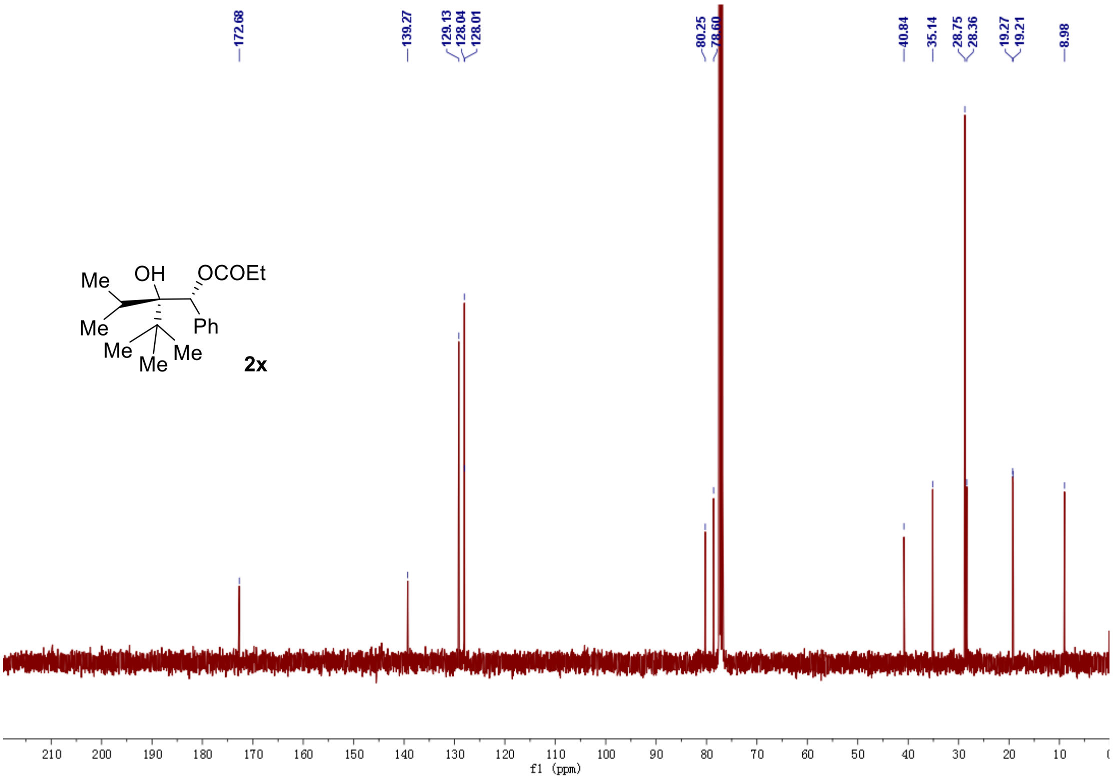

Supplementary Figure 120. ^{1}H NMR sepctra of (1*S*,2*S*)-2-Ethyl-1-phenylbut-3-ene-1,2-diol (**1y**)

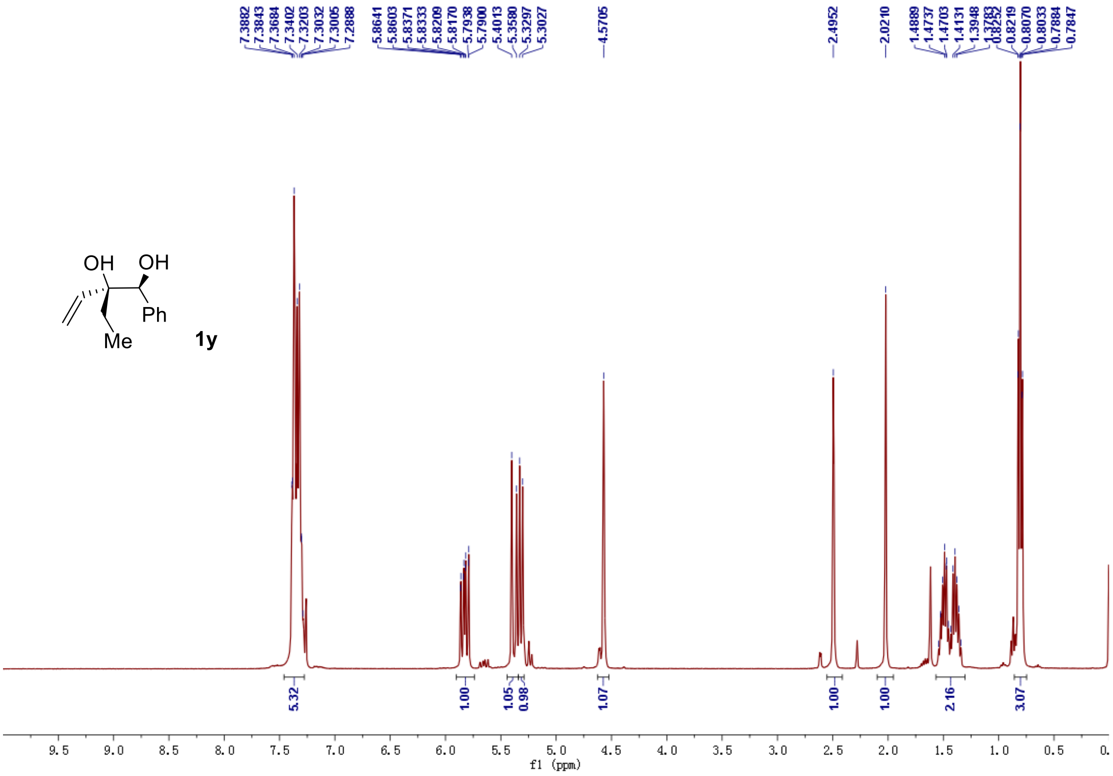

Supplementary Figure 121. ^{13}C NMR sepctra of (1*S*,2*S*)-2-Ethyl-1-phenylbut-3-ene-1,2-diol (**1y**)

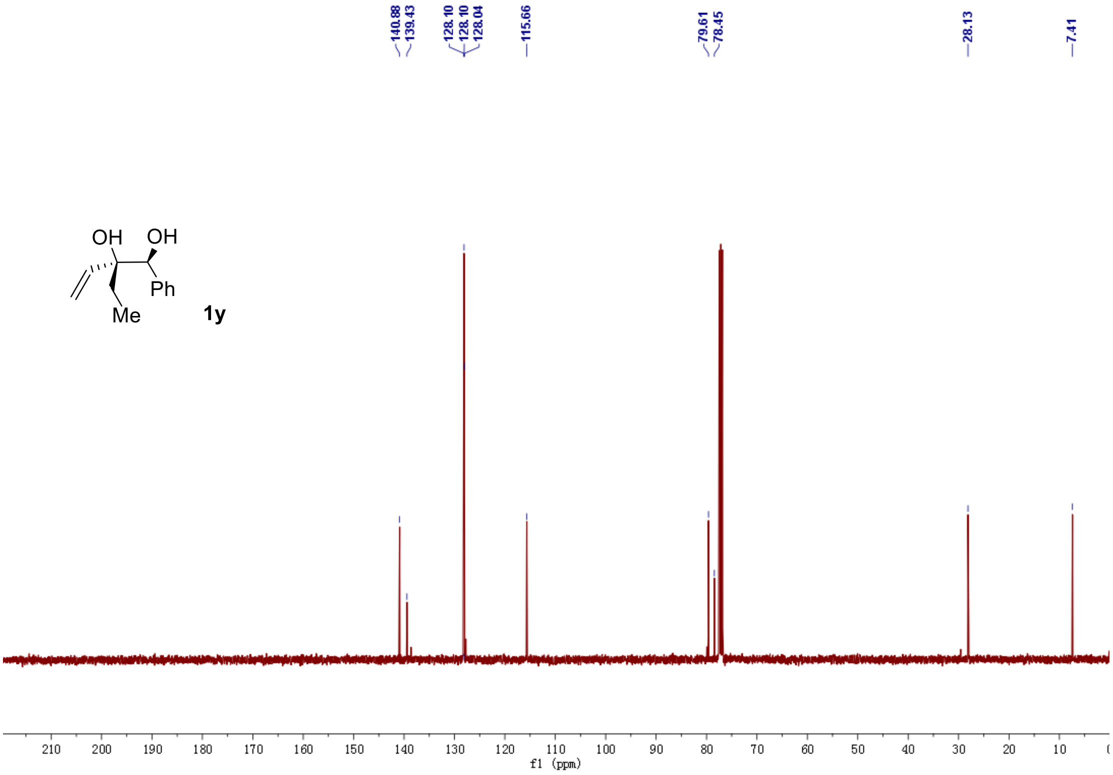

Supplementary Figure 122. ^{1}H NMR sepctra of (1*R*,2*R*)-2-Ethyl-2-hydroxy-1-phenylbut-3-en-1-yl propionate (**2y**)

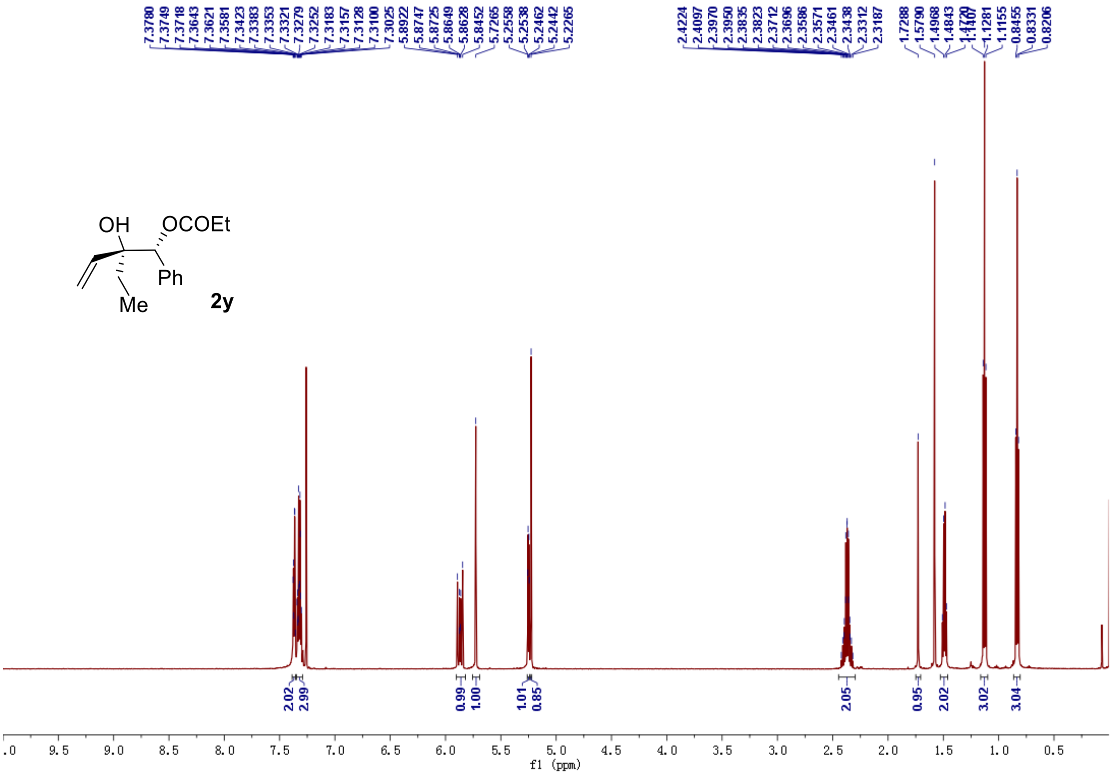

Supplementary Figure 123. ^{13}C NMR sepctra of (1*R*,2*R*)-2-Ethyl-2-hydroxy-1-phenylbut-3-en-1-yl propionate (**2y**)

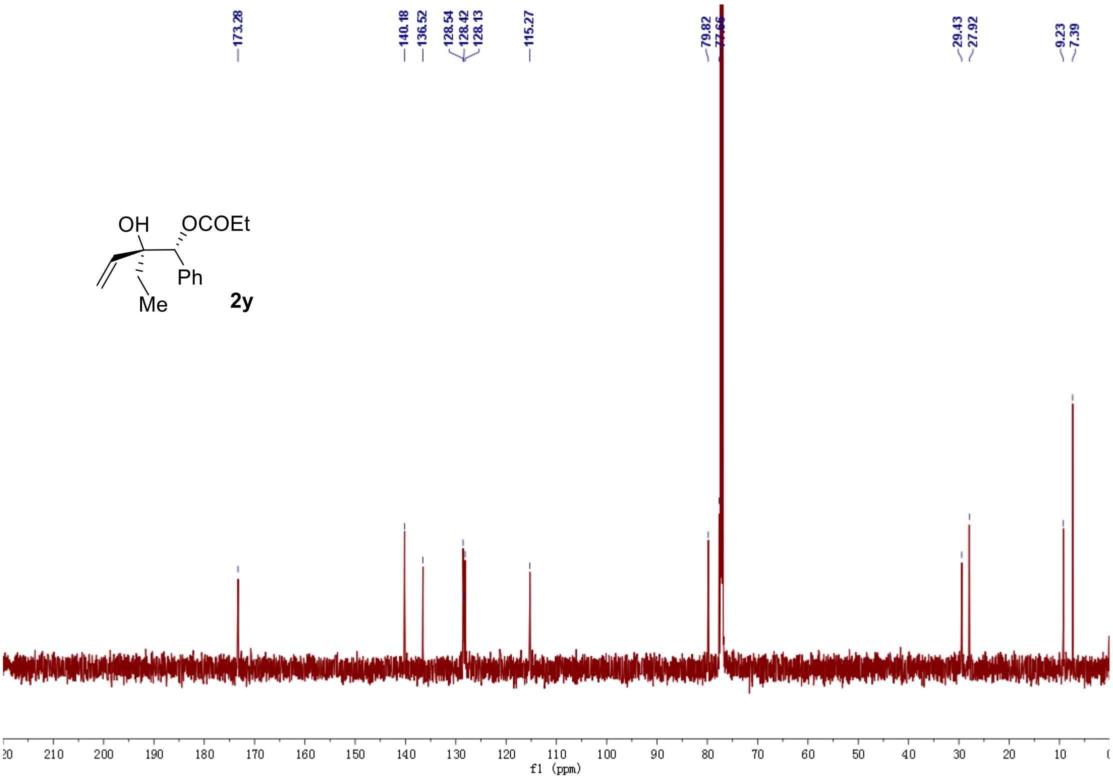

Supplementary Figure 124. ^{1}H NMR sepctra of (1*S*,2*S*)-1-Phenyl-2-(phenylethynyl)hexane-1,2-diol (**1z**)

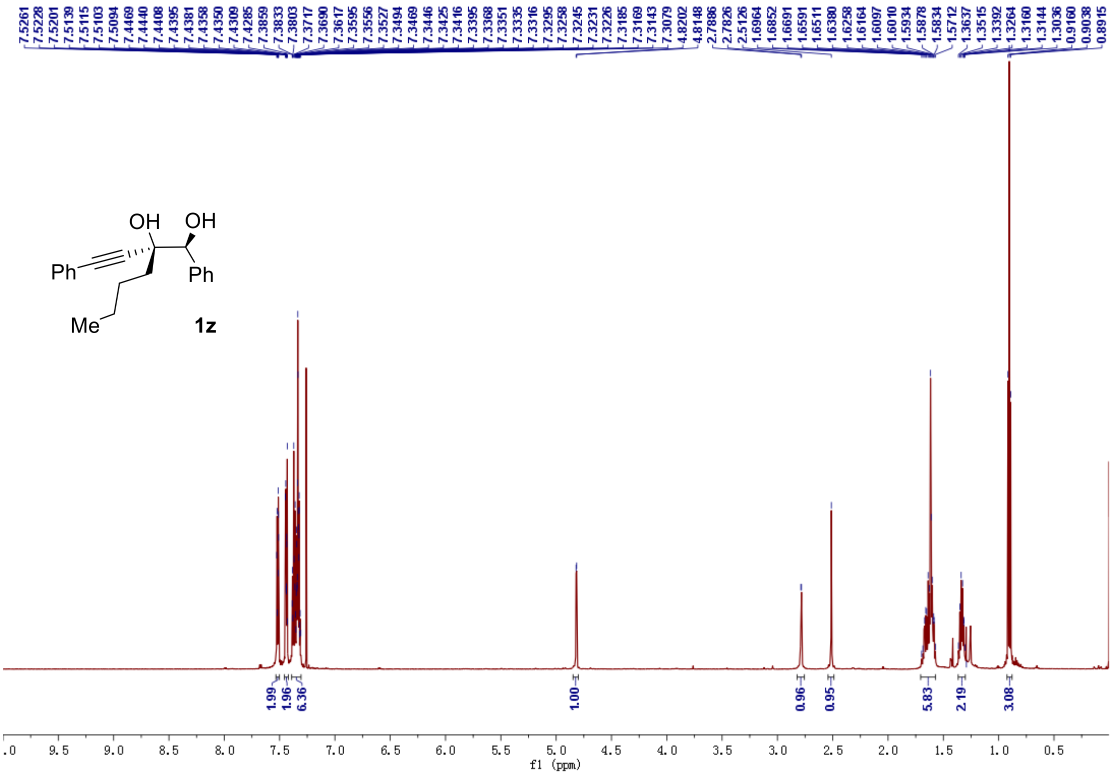

Supplementary Figure 125. ^{13}C NMR sepctra of (1*S*,2*S*)-1-Phenyl-2-(phenylethynyl)hexane-1,2-diol (**1z**)

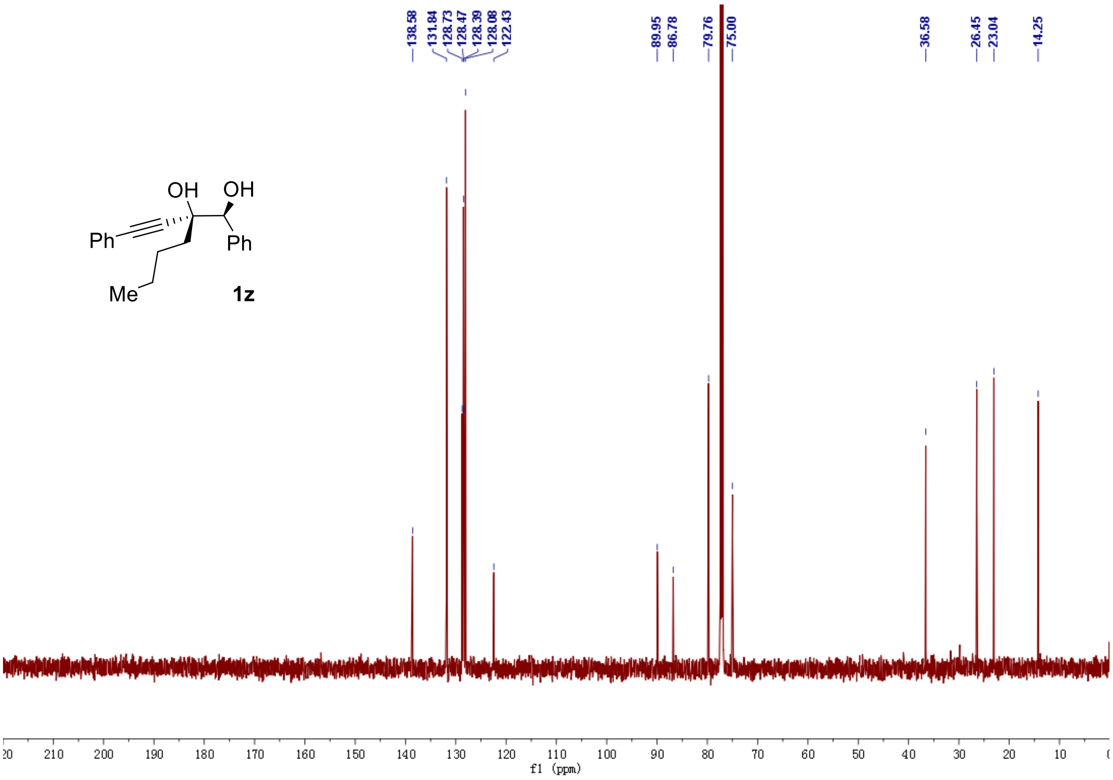

Supplementary Figure 126. ^{1}H NMR sepctra of (1*R*,2*R*)-2-Hydroxy-1-phenyl-2-(phenylethynyl)hexyl propionate (**2z**)

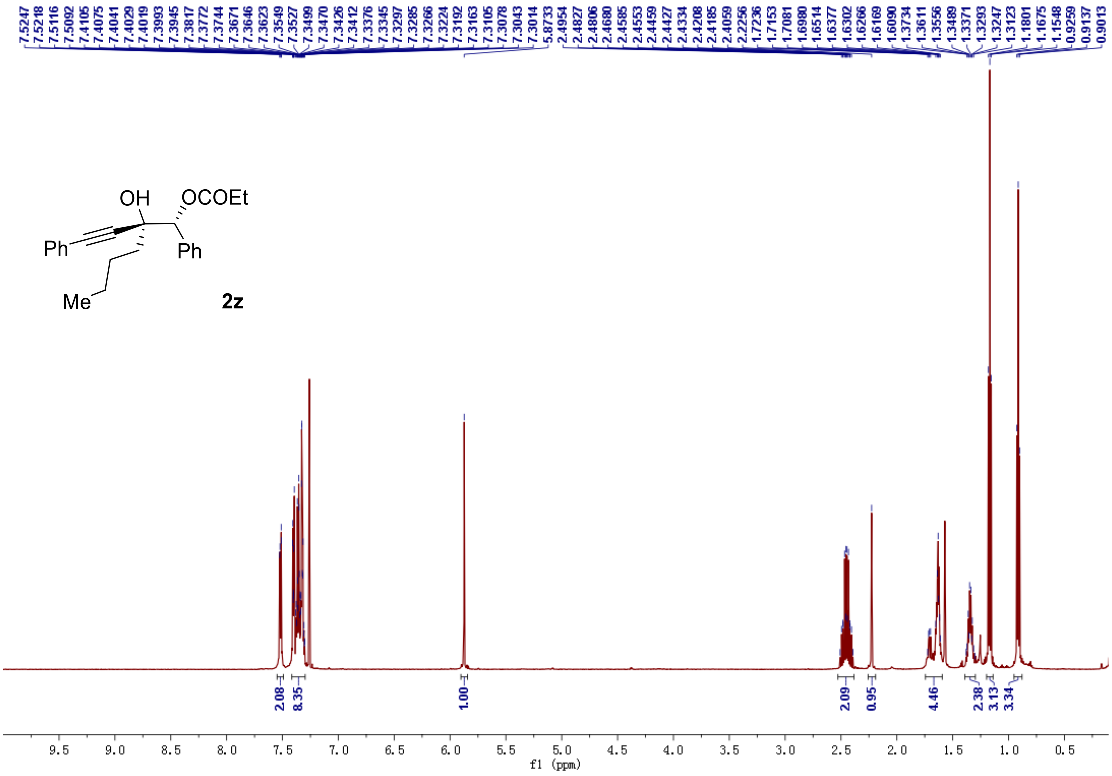

Supplementary Figure 127. ^{13}C NMR sepctra of (1*R*,2*R*)-2-Hydroxy-1-phenyl-2-(phenylethynyl)hexyl propionate (**2z**)

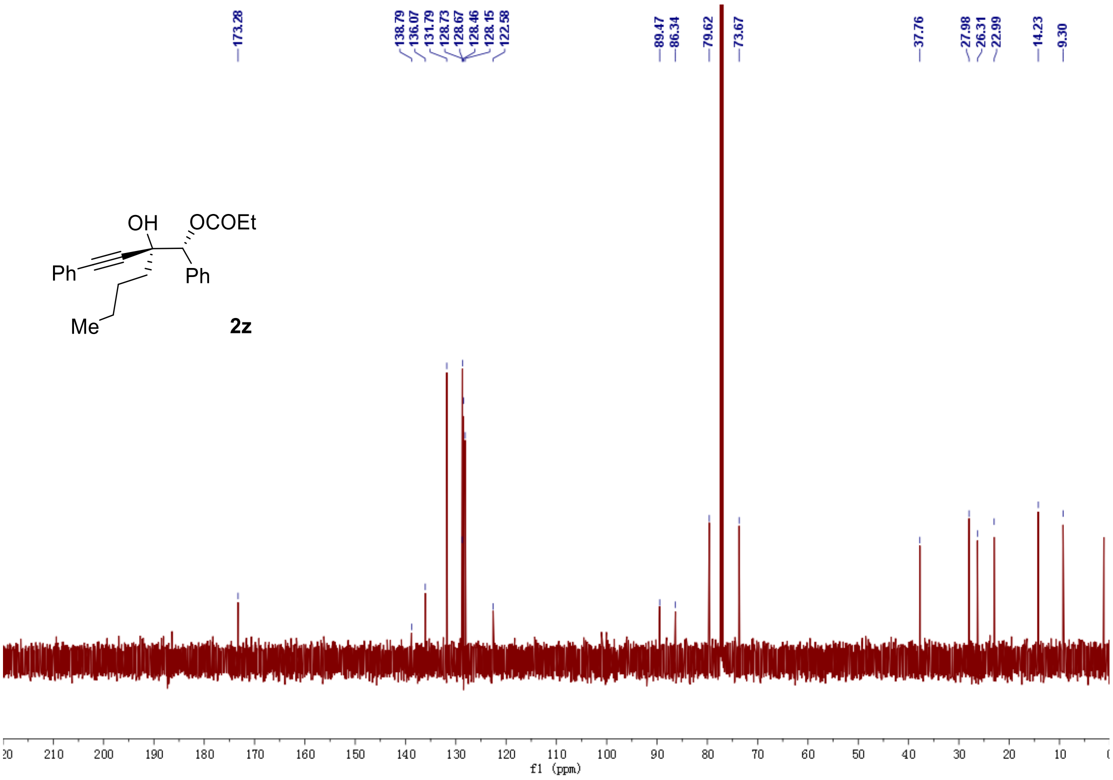

Supplementary Figure 128. ^{1}H NMR sepctra of (1*R*,2*S*)-2-(4-Chlorophenyl)-1-cyclohexyl-1-phenylethane-1,2-diol (**1za**)

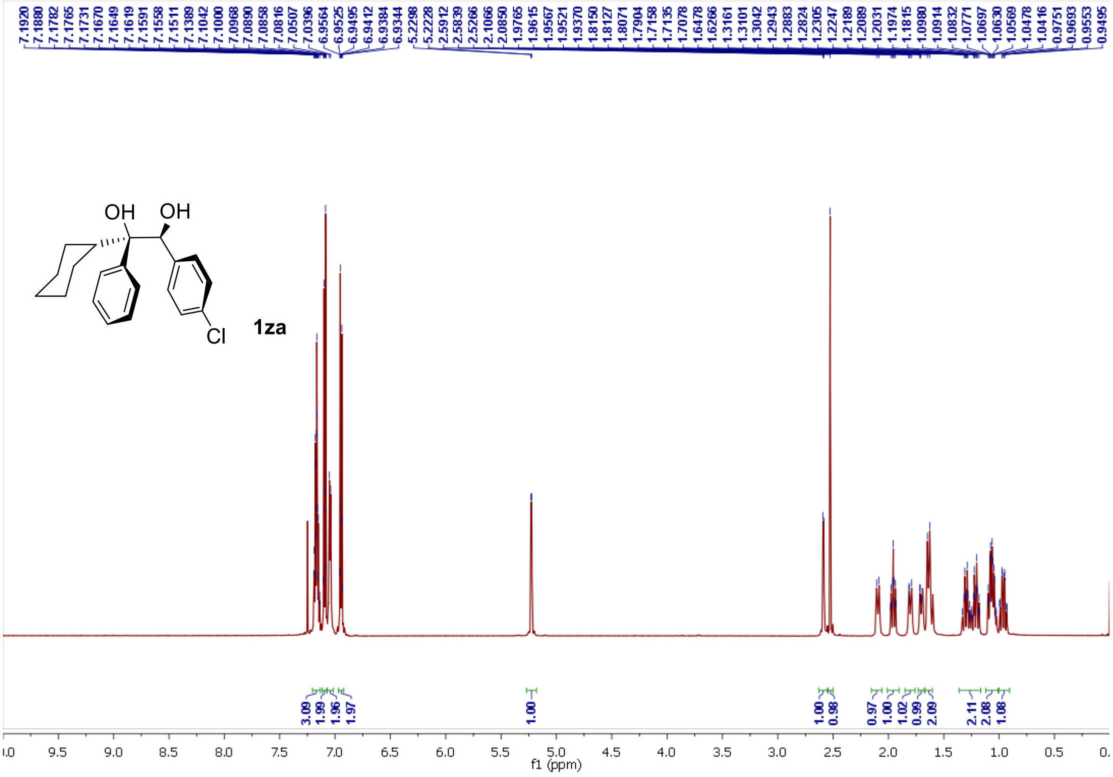

Supplementary Figure 129. ^{13}C NMR sepctra of (1*R*,2*S*)-2-(4-Chlorophenyl)-1-cyclohexyl-1-phenylethane-1,2-diol (**1za**)

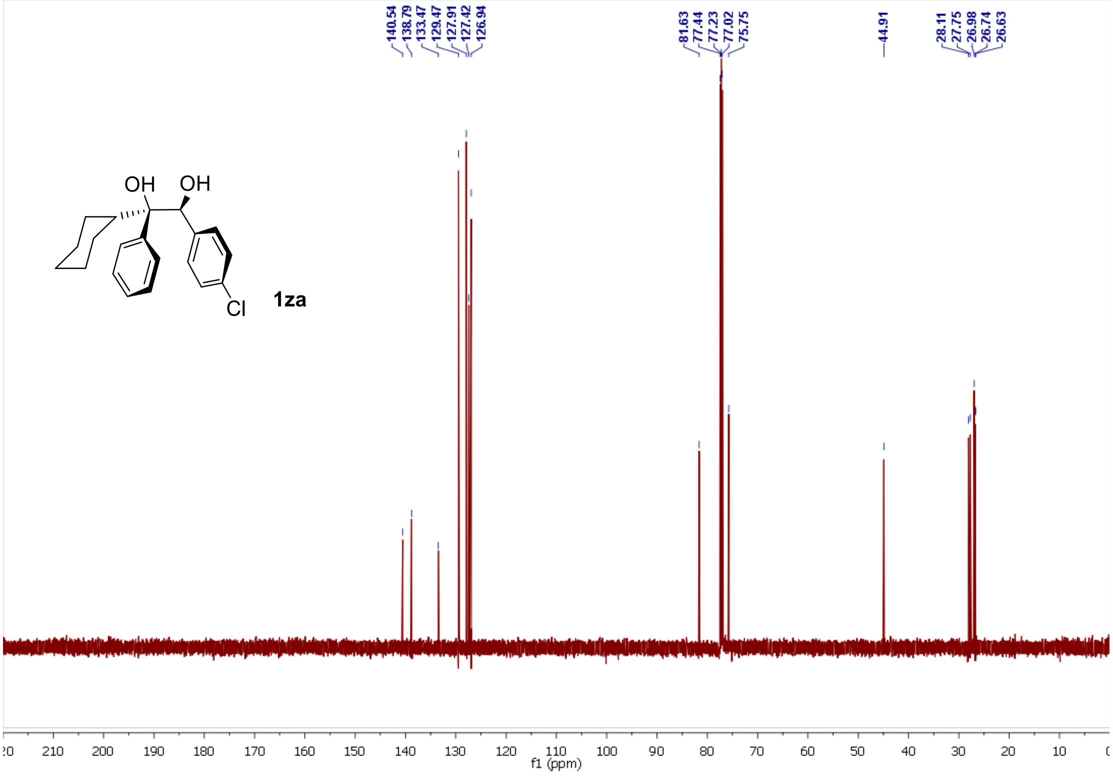

Supplementary Figure 130. ^{1}H NMR sepctra of (1*R*,2*S*)-1-(4-Chlorophenyl)-2-cyclohexyl-2-hydroxy-2-phenylethyl propionate (**2za**)

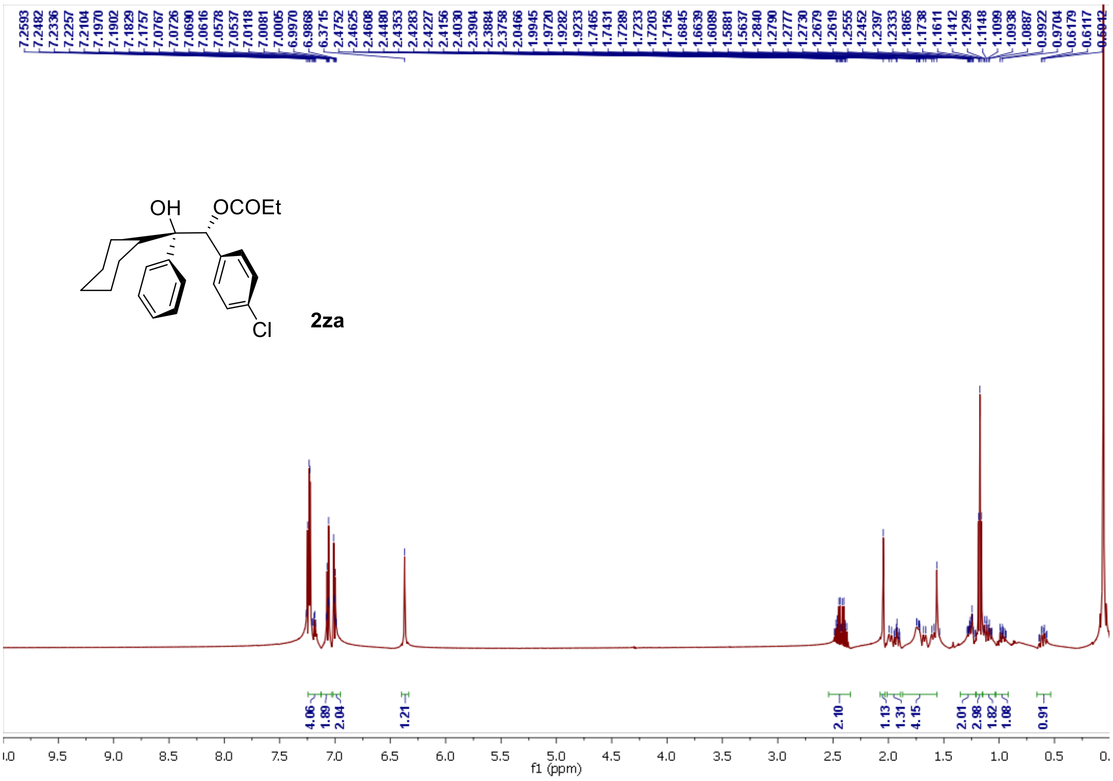

Supplementary Figure 131. ^{13}C NMR sepctra of (1*R*,2*S*)-1-(4-Chlorophenyl)-2-cyclohexyl-2-hydroxy-2-phenylethyl propionate (**2za**)

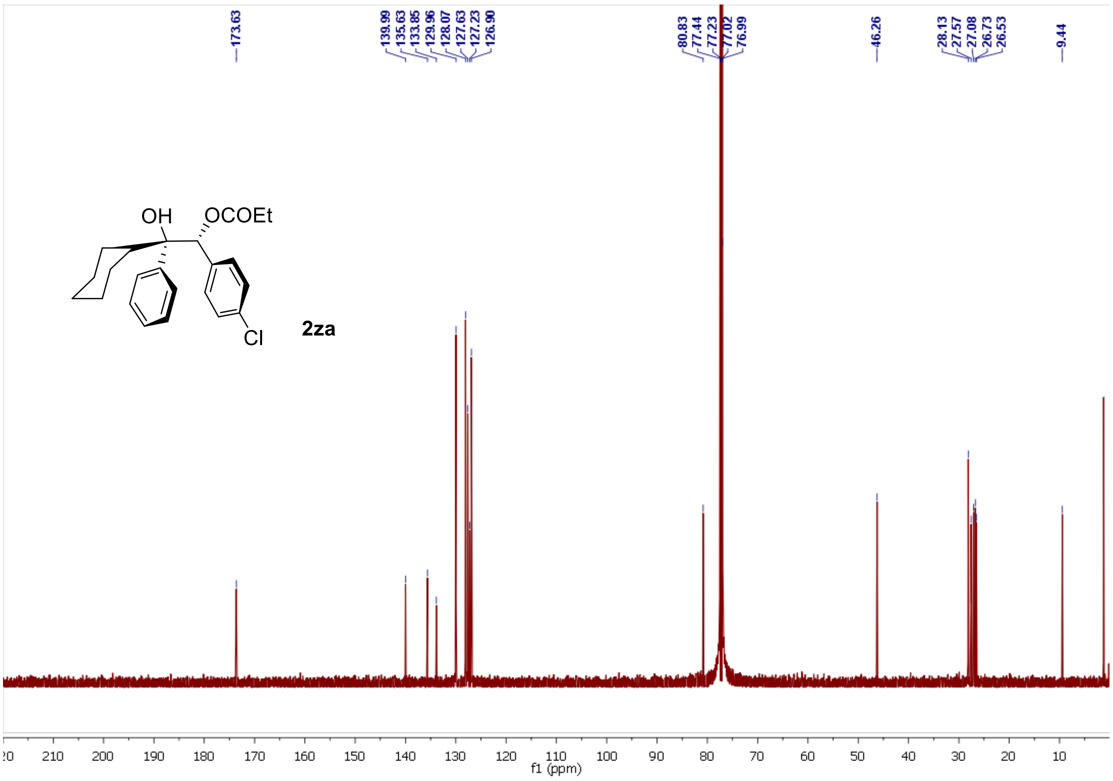

Supplementary Figure 132. ^{1}H NMR sepctra of (*R*)-(2-((*R*)-Hydroxy(phenyl)methyl)phenyl)(phenyl)(o-tolyl)methanol (**1aa**)

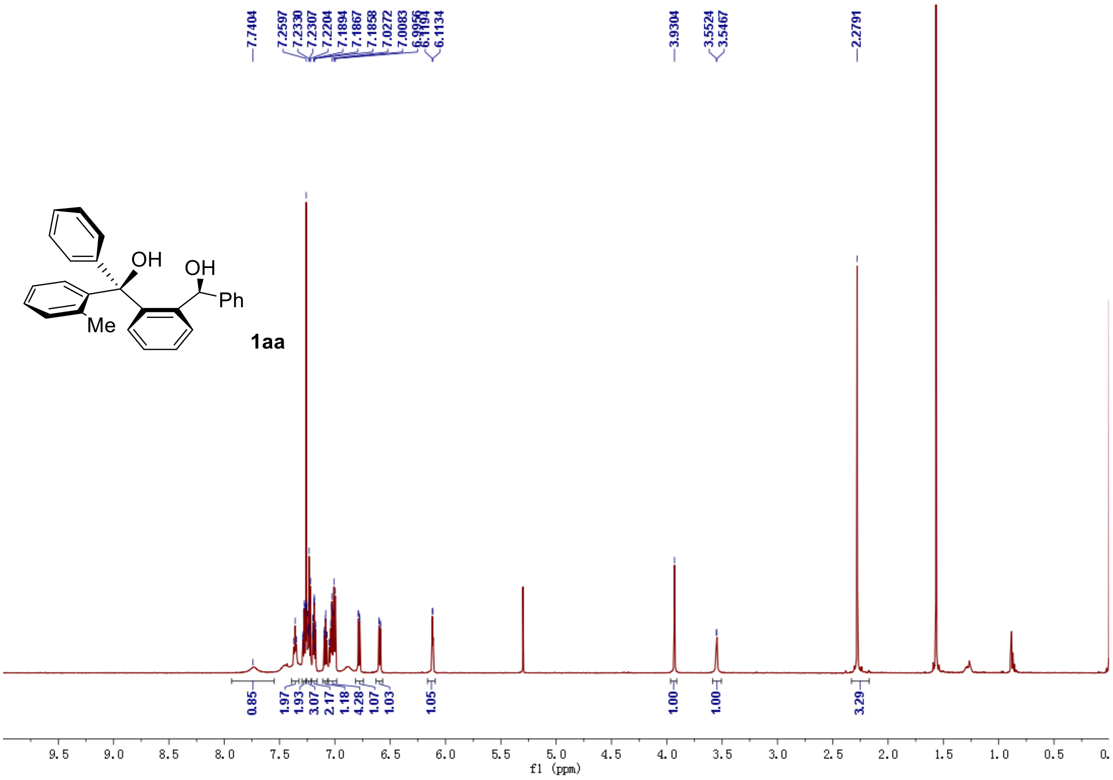

Supplementary Figure 133. ^{13}C NMR sepctra of (*R*)-(2-((*R*)-Hydroxy(phenyl)methyl)phenyl)(phenyl)(o-tolyl)methanol (**1aa**)

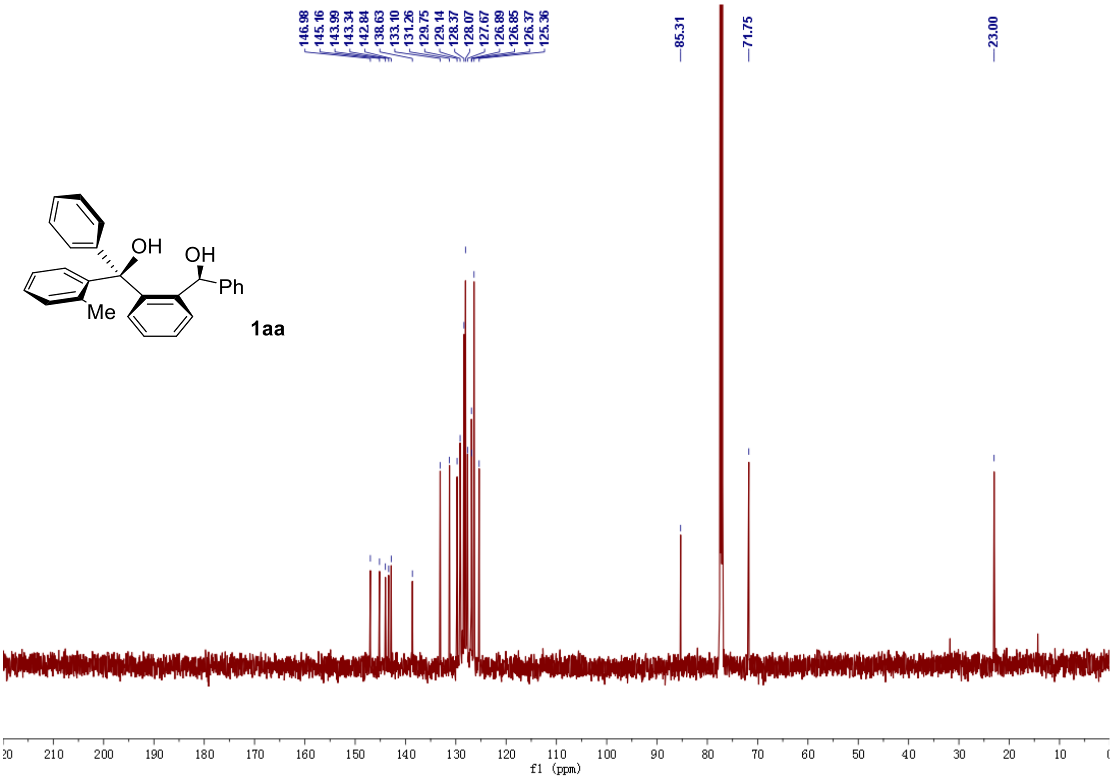

Supplementary Figure 134. ^{1}H NMR sepctra of (*S*)-(2-((*S*)-Hydroxy(phenyl)(o-tolyl)methyl)phenyl)(phenyl)methyl propionate (**2aa**)

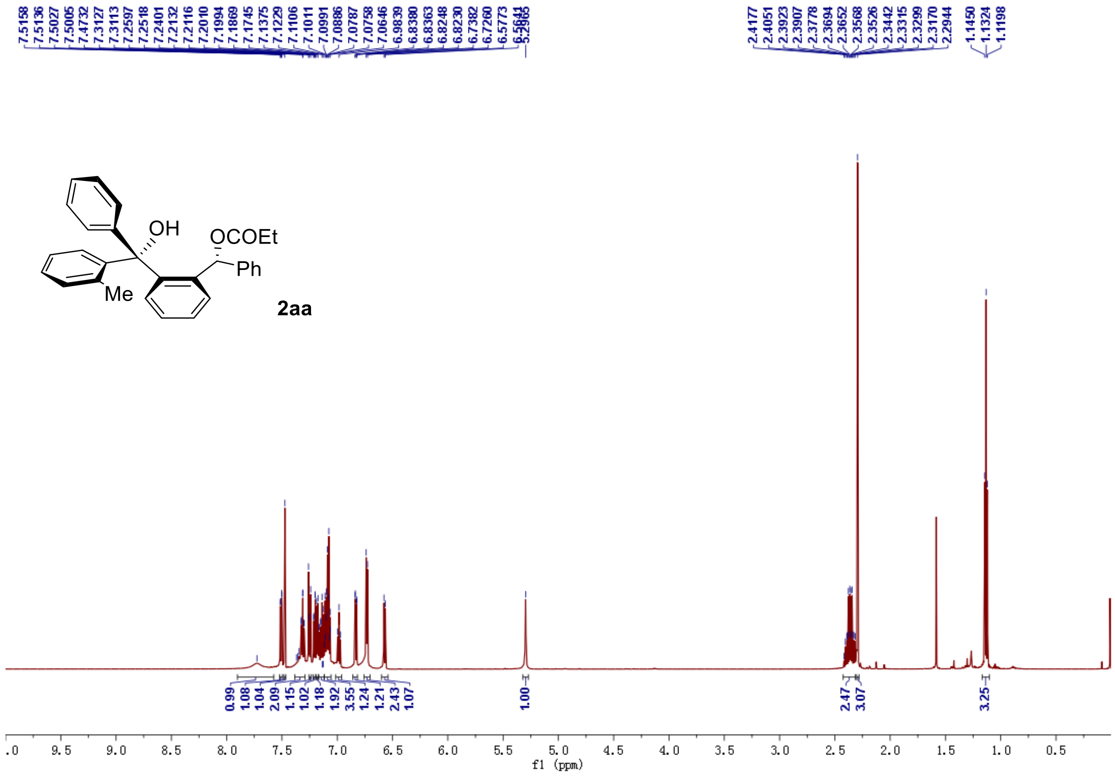

Supplementary Figure 135. ^{13}C NMR sepctra of (*S*)-(2-((*S*)-Hydroxy(phenyl)(o-tolyl)methyl)phenyl)(phenyl)methyl propionate (**2aa**)

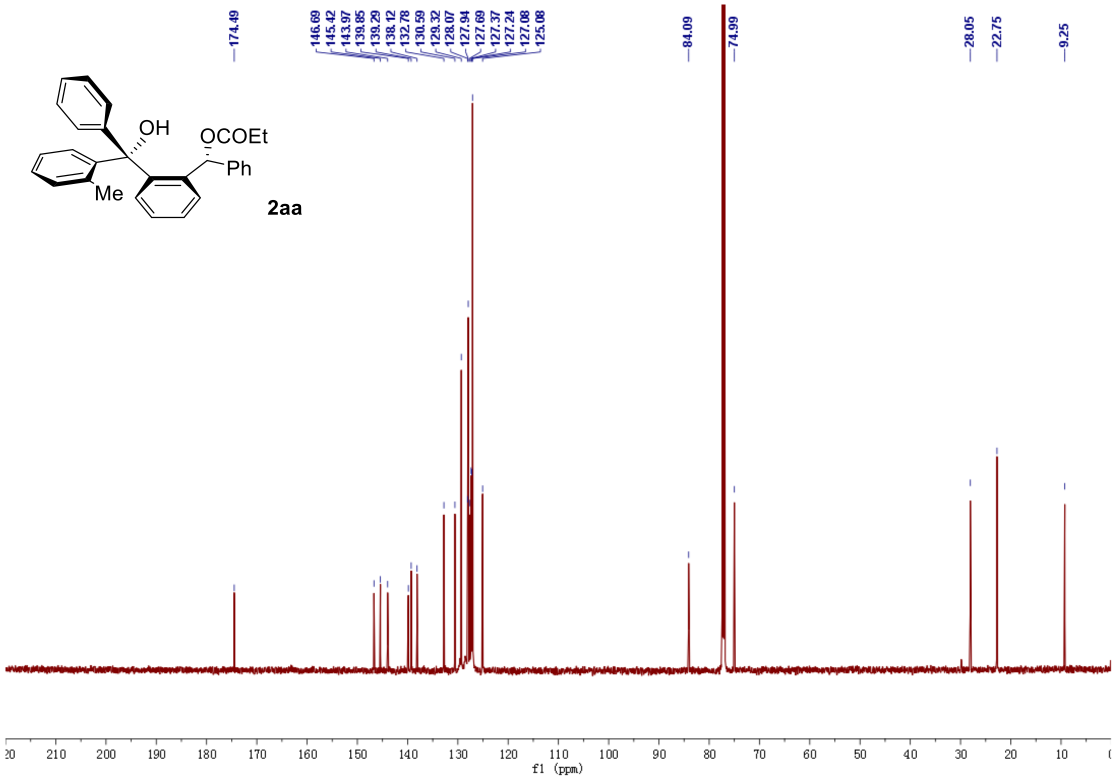

Supplementary Figure 136. ^{1}H NMR sepctra of (*R*)-(2-((*R*)-Hydroxy(phenyl)methyl)phenyl)(*o*-tolyl)(*p*-tolyl)methanol (**1ab**)

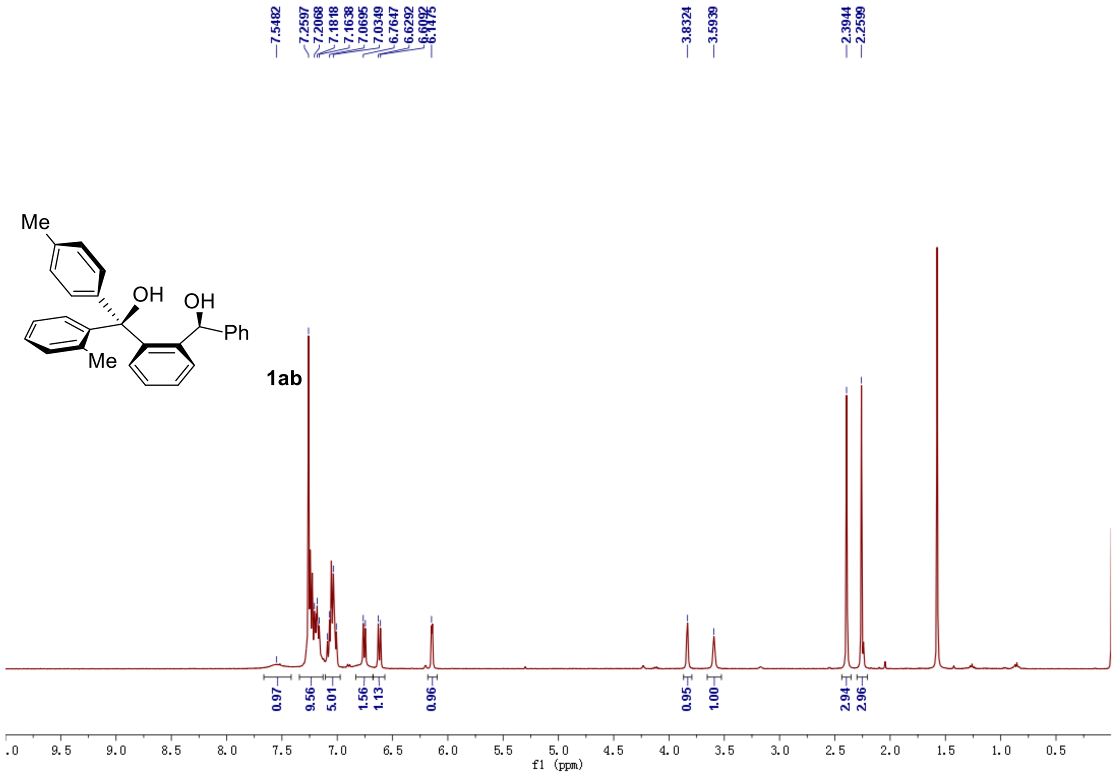

Supplementary Figure 137. ^{13}C NMR sepctra of (*R*)-(2-((*R*)-Hydroxy(phenyl)methyl)phenyl)(*o*-tolyl)(*p*-tolyl)methanol (**1ab**)

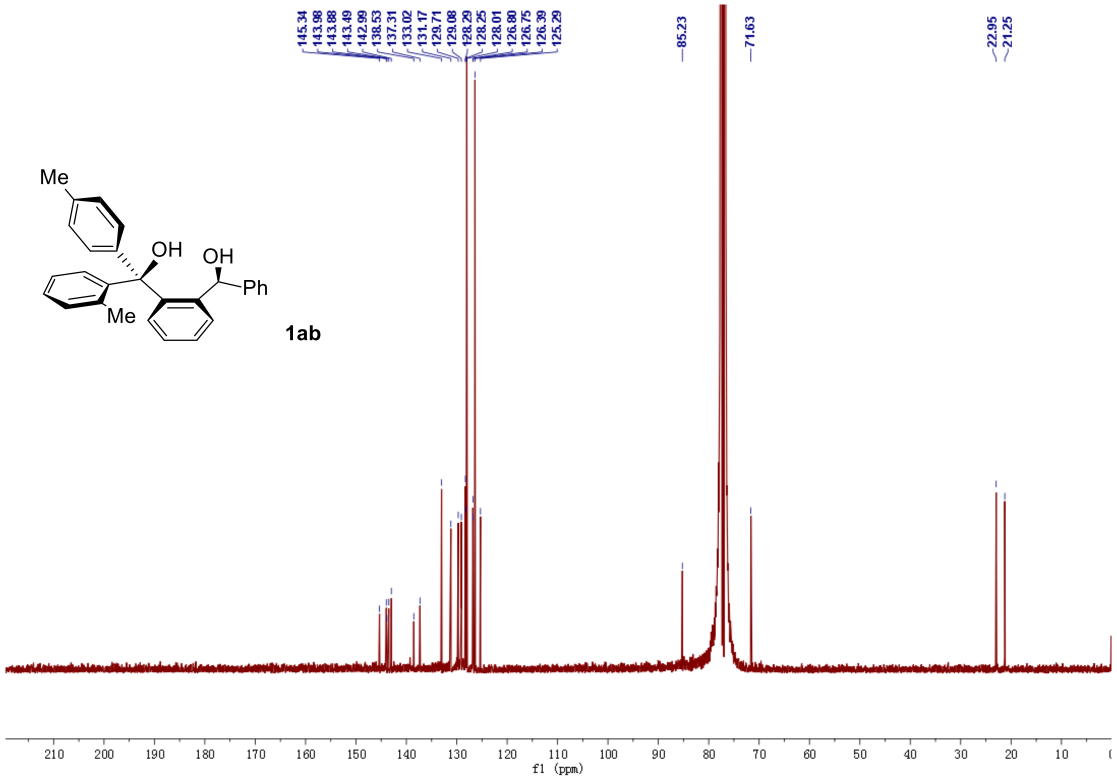

Supplementary Figure 138. ¹H NMR sepctra of (*S*)-(2-((*S*)-Hydroxy(*o*-tolyl)(*p*-tolyl)methyl)phenyl)(phenyl)methyl propionate (**2ab**)

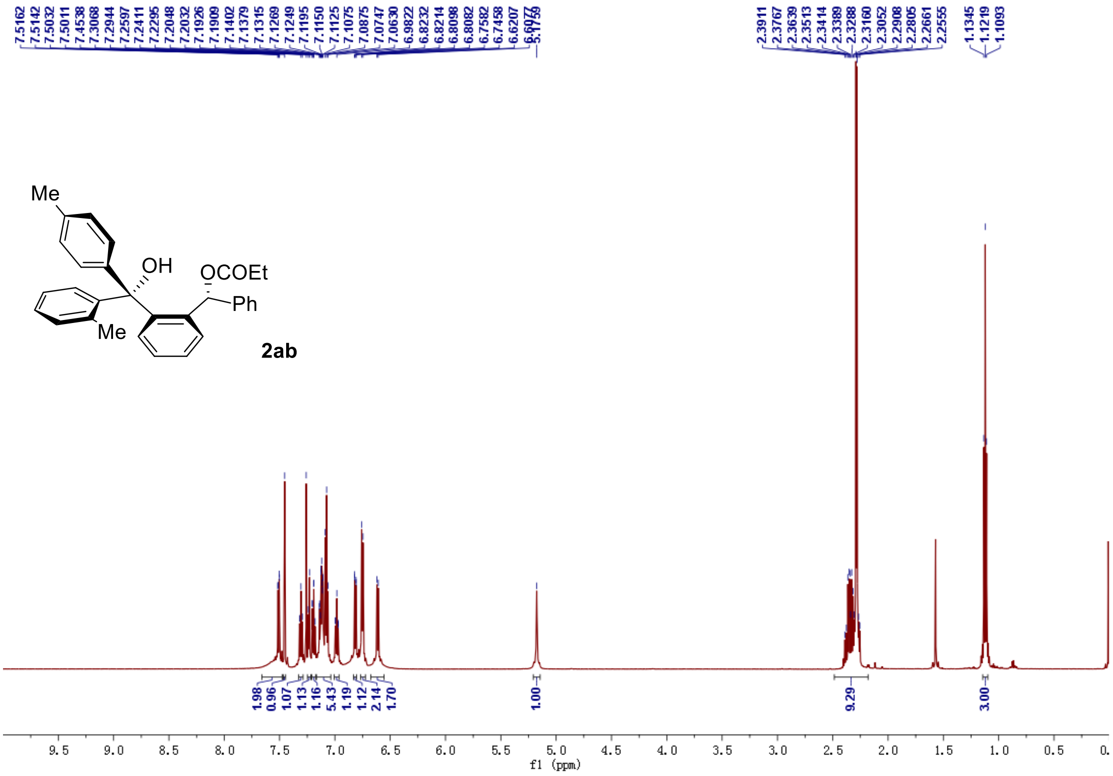

Supplementary Figure 139. ¹³C NMR sepctra of (*S*)-(2-((*S*)-Hydroxy(*o*-tolyl)(*p*-tolyl)methyl)phenyl)(phenyl)methyl propionate (**2ab**)

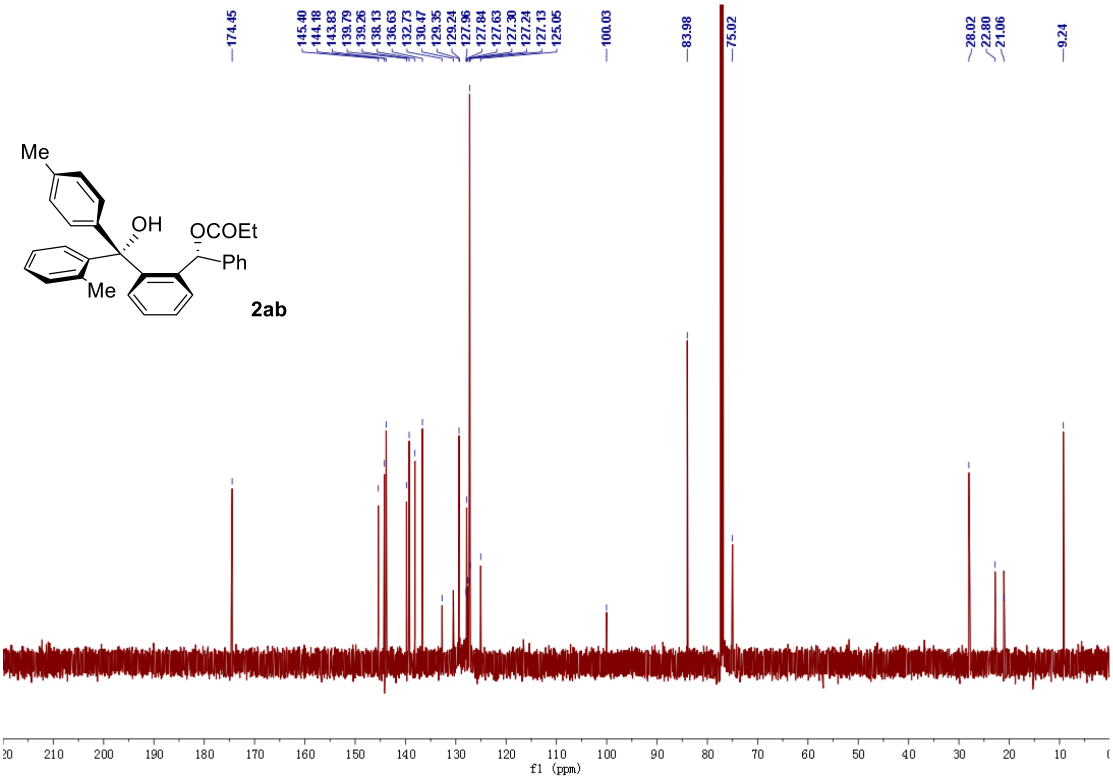

Supplementary Figure 140. ^{1}H NMR sepctra of (*R*)-(4-Chlorophenyl)(2-((*R*)-hydroxy(phenyl)methyl)phenyl)(*o*-tolyl)methanol (**1ac**)

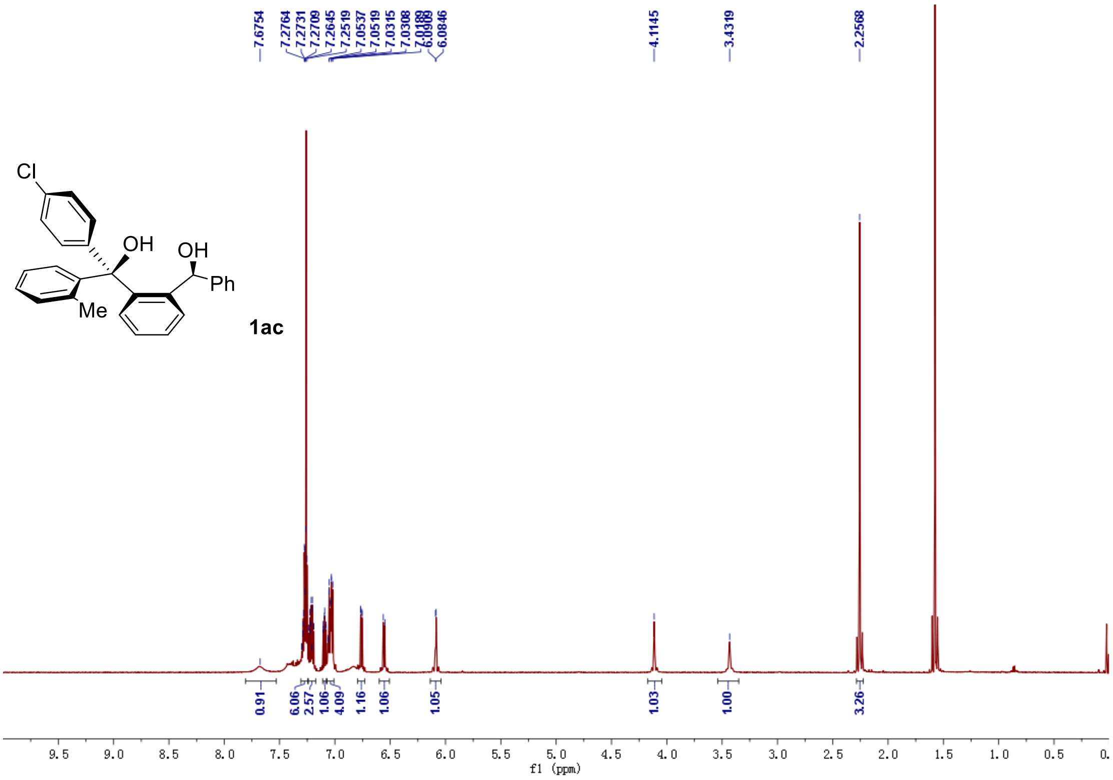

Supplementary Figure 141. ^{13}C NMR sepctra of (*R*)-(4-Chlorophenyl)(2-((*R*)-hydroxy(phenyl)methyl)phenyl)(*o*-tolyl)methanol (**1ac**)

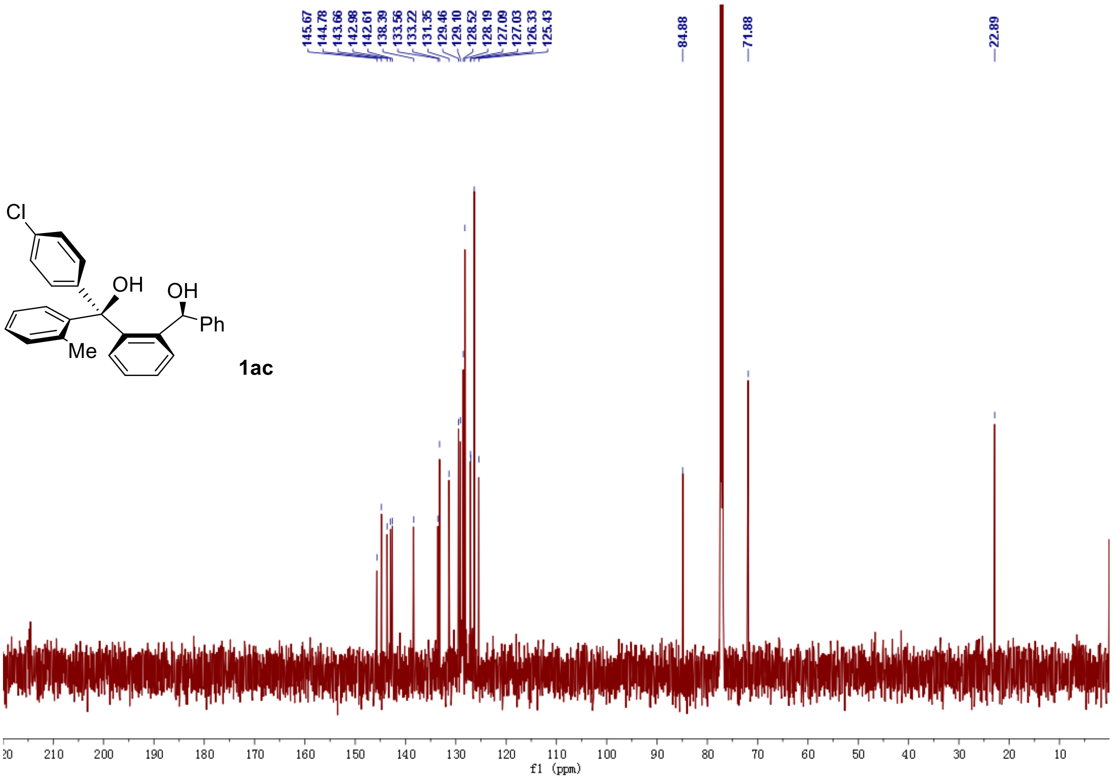

Supplementary Figure 142. ^{1}H NMR sepctra of (*S*)-(2-((*S*)-(4-Chlorophenyl)(hydroxy)(*o*-tolyl)methyl)phenyl)(phenyl)methyl propionate (**2ac**)

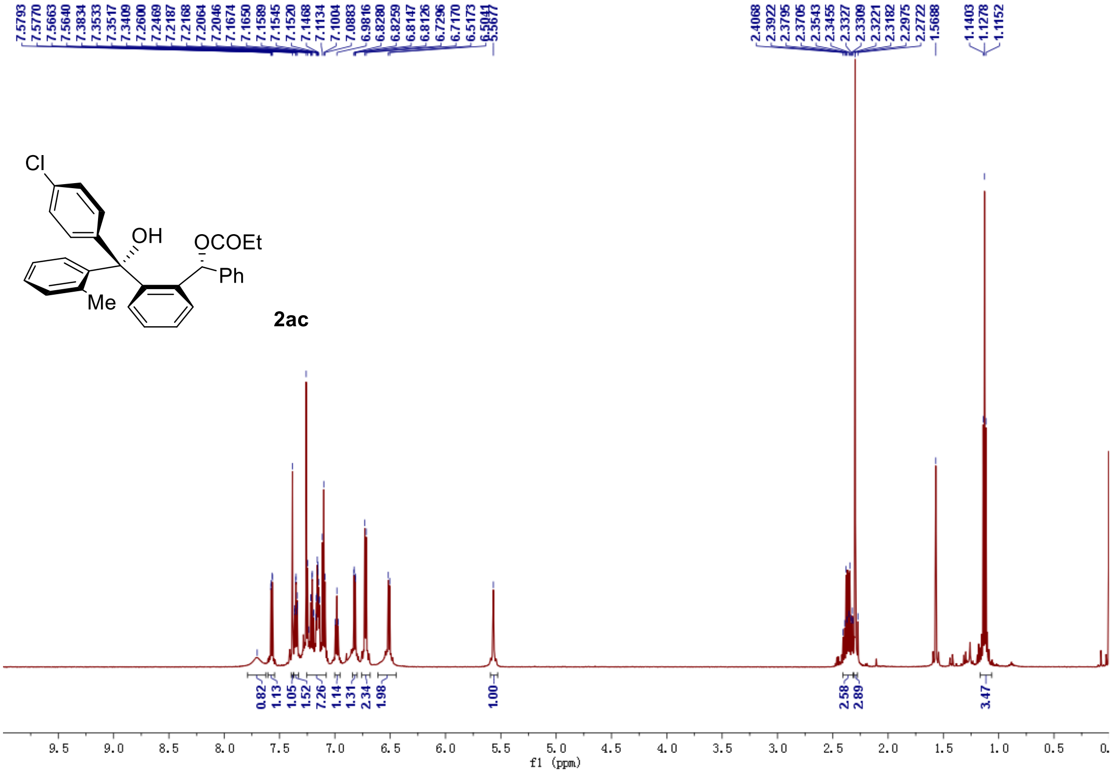

Supplementary Figure 143. ^{13}C NMR sepctra of (*S*)-(2-((*S*)-(4-Chlorophenyl)(hydroxy)(*o*-tolyl)methyl)phenyl)(phenyl)methyl propionate (**2ac**)

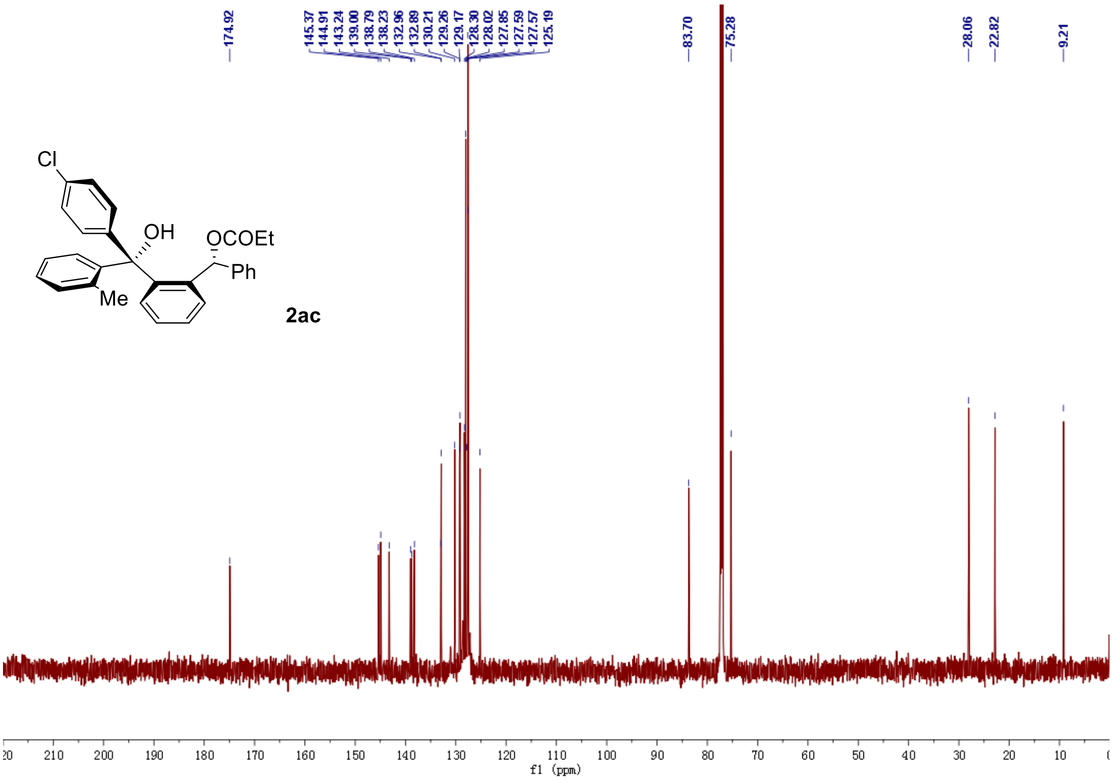

Supplementary Figure 144. ^{1}H NMR sepctra of (*R*)-(4-Fluoro-2-methylphenyl)(2-((*R*)hydroxy(phenyl)methyl)phenyl)(phenyl)methanol (**1ad**)

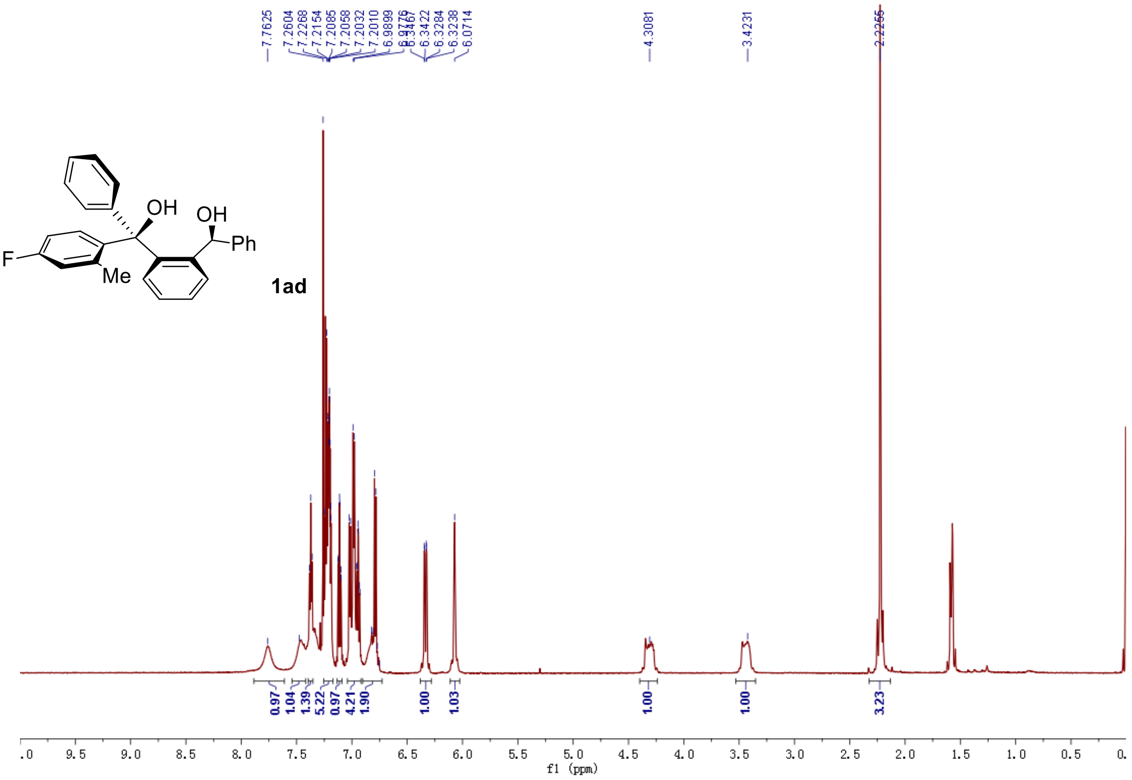

Supplementary Figure 145. ^{13}C NMR sepctra of (*R*)-(4-Fluoro-2-methylphenyl)(2-((*R*)hydroxy(phenyl)methyl)phenyl)(phenyl)methanol (**1ad**)

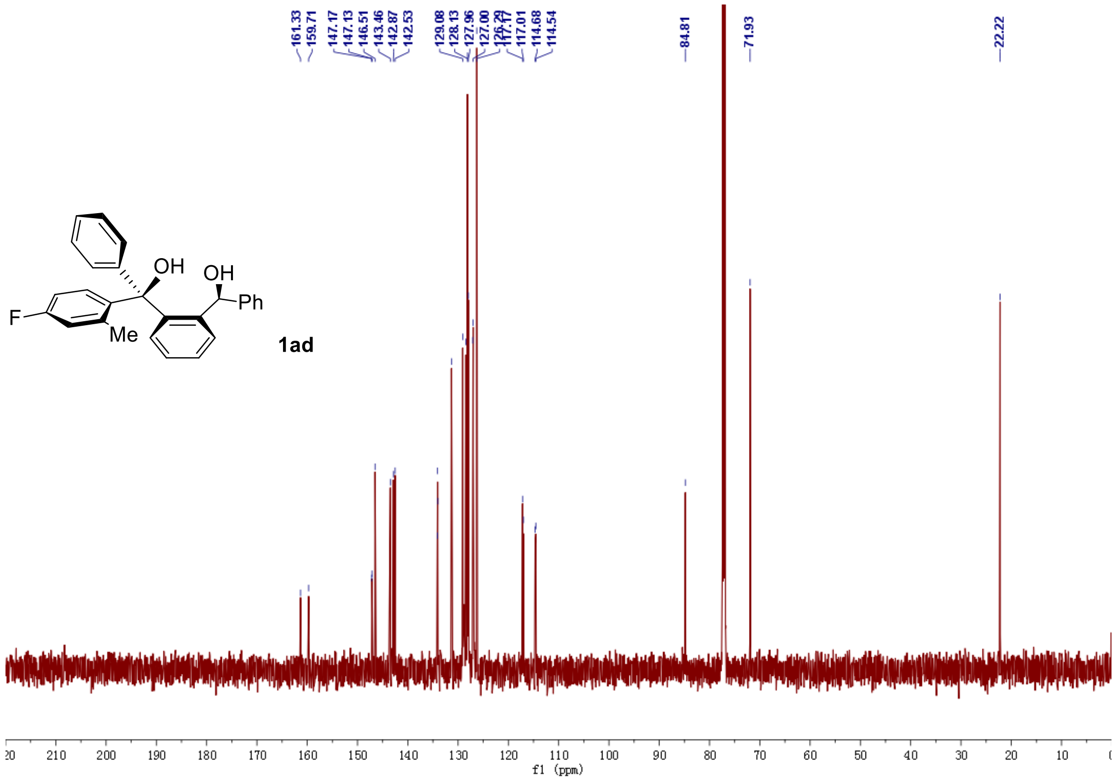

Supplementary Figure 146. ^{1}H NMR sepctra of (*S*)-(2-((*S*)-(4-Fluoro-2-methylphenyl)(hydroxy)(phenyl)methyl)phenyl)(phenyl)methyl propionate (**2ad**)

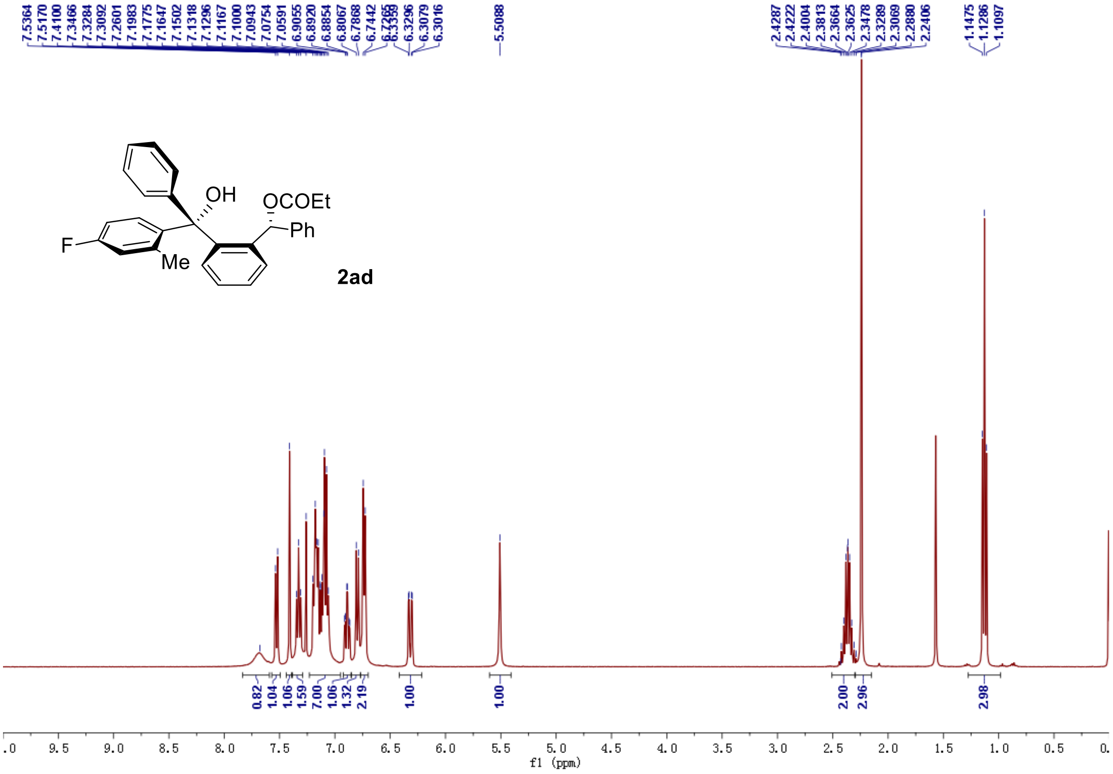

Supplementary Figure 147. ^{13}C NMR sepctra of (*S*)-(2-((*S*)-(4-Fluoro-2-methylphenyl)(hydroxy)(phenyl)methyl)phenyl)(phenyl)methyl propionate (**2ad**)

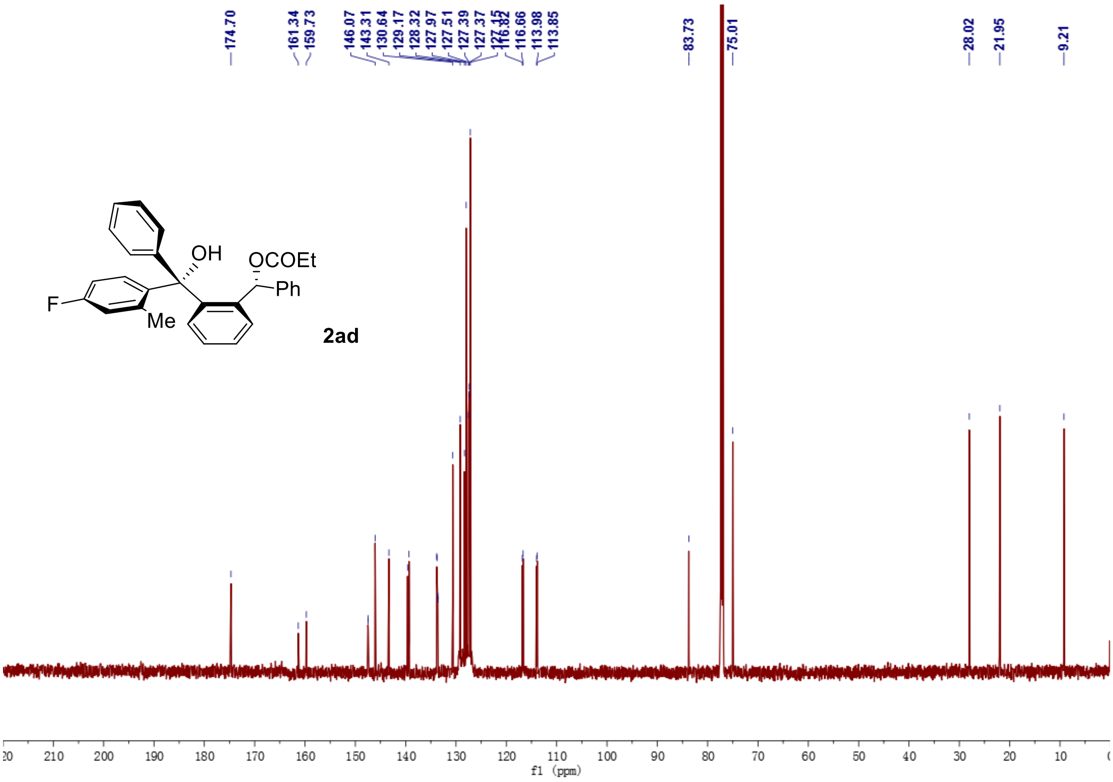

Supplementary Figure 148. ^{1}H NMR sepctra of (*S*)-phenyl((2*S*,3*R*)-3-phenyl-3-(*p*-tolyl)oxiran-2-yl)methanol (**3a**)

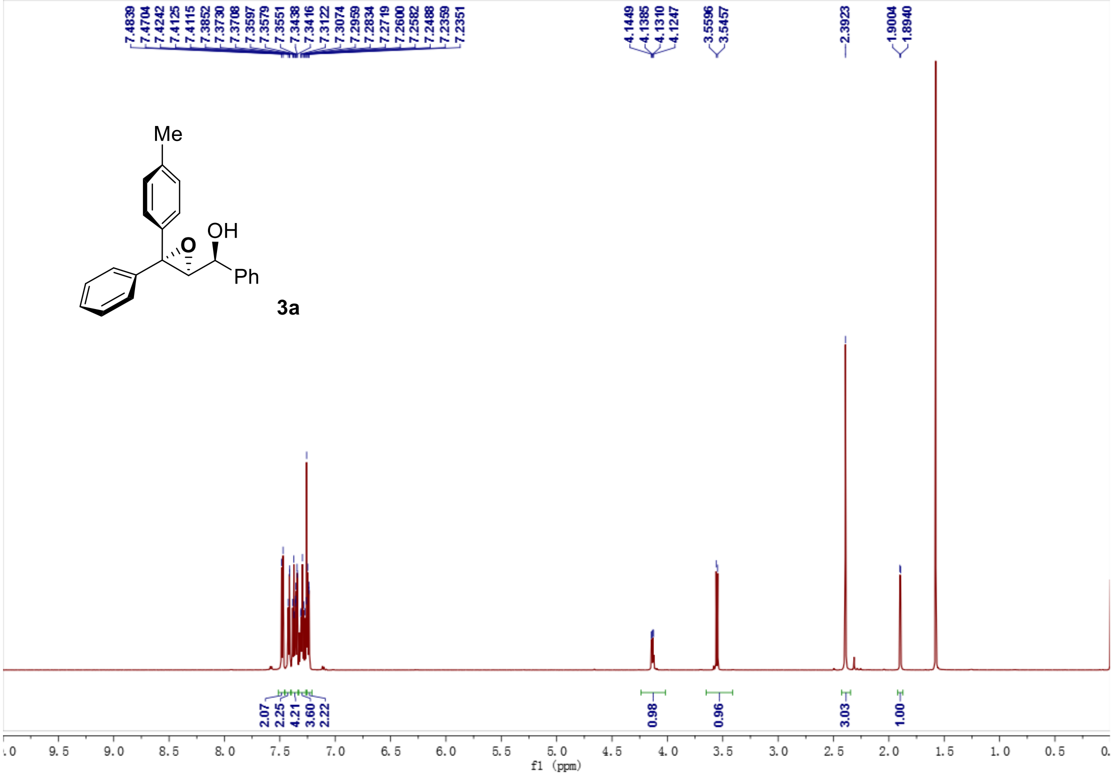

Supplementary Figure 149. ^{13}C NMR sepctra of (*S*)-phenyl((2*S*,3*R*)-3-phenyl-3-(*p*-tolyl)oxiran-2-yl)methanol (**3a**)

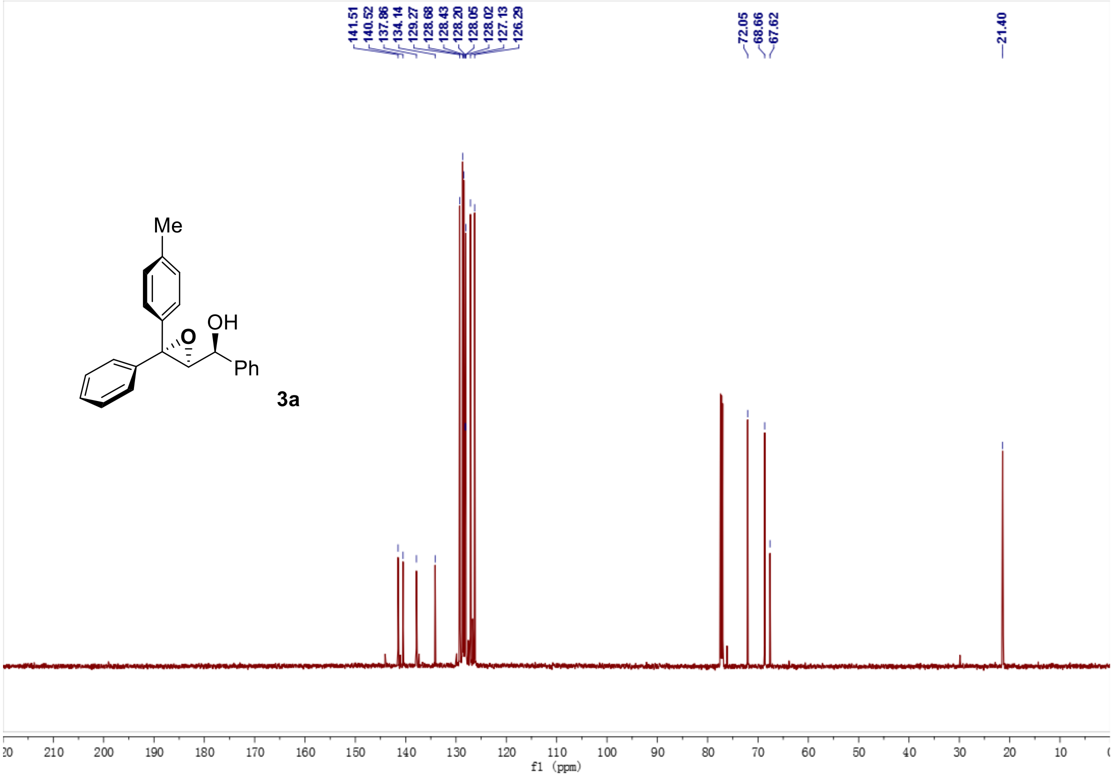

Supplementary Figure 150. ^{1}H NMR sepctra of (*R*)-phenyl((2*R*,3*S*)-3-phenyl-3-(*p*-tolyl)oxiran-2-yl)methyl propionate (**4a**)

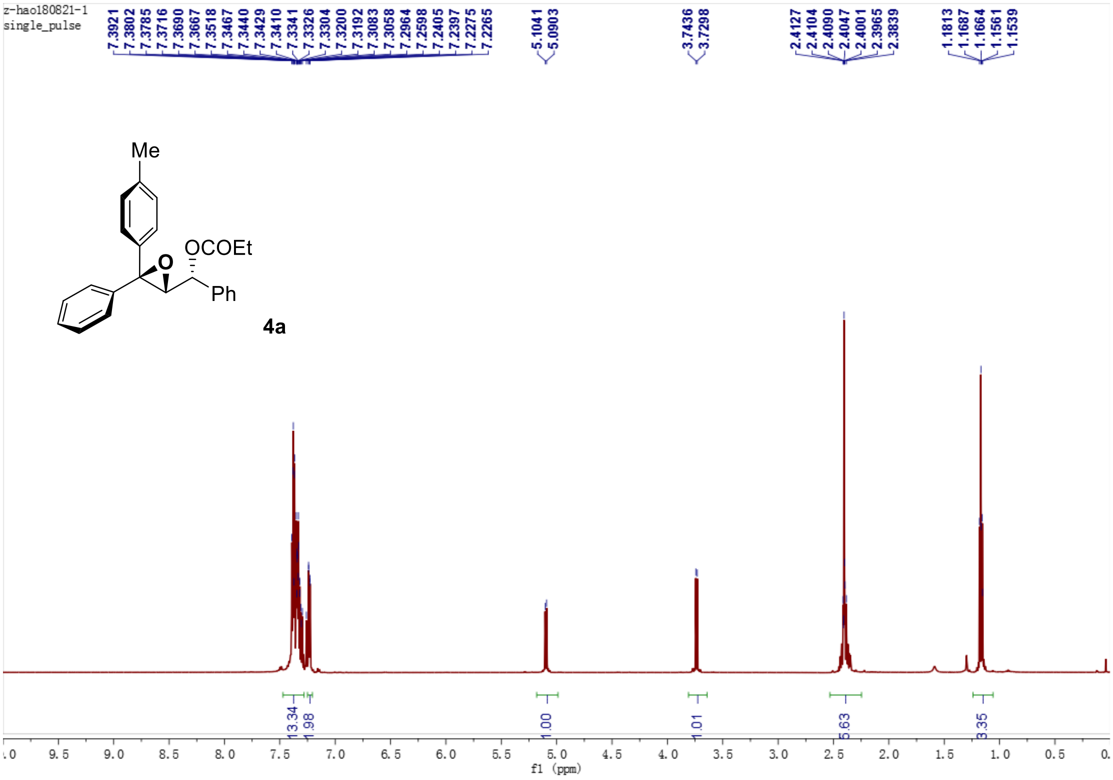

Supplementary Figure 151. ^{13}C NMR sepctra of (*R*)-phenyl((2*R*,3*S*)-3-phenyl-3-(*p*-tolyl)oxiran-2-yl)methyl propionate (**4a**)

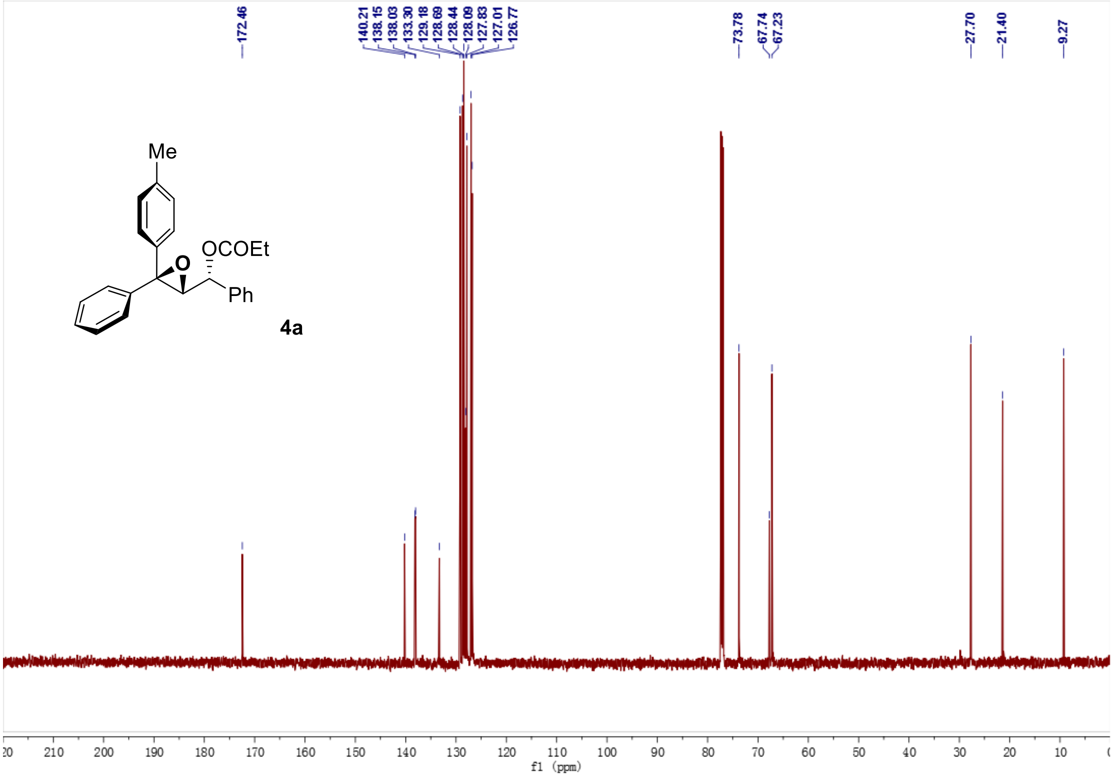

Supplementary Figure 152. ^{1}H NMR sepctra of (*S*)-(4-methoxyphenyl)((2*S*,3*R*)-3-phenyl-3-(*p*-tolyl)oxiran-2-yl)methanol (**3b**)

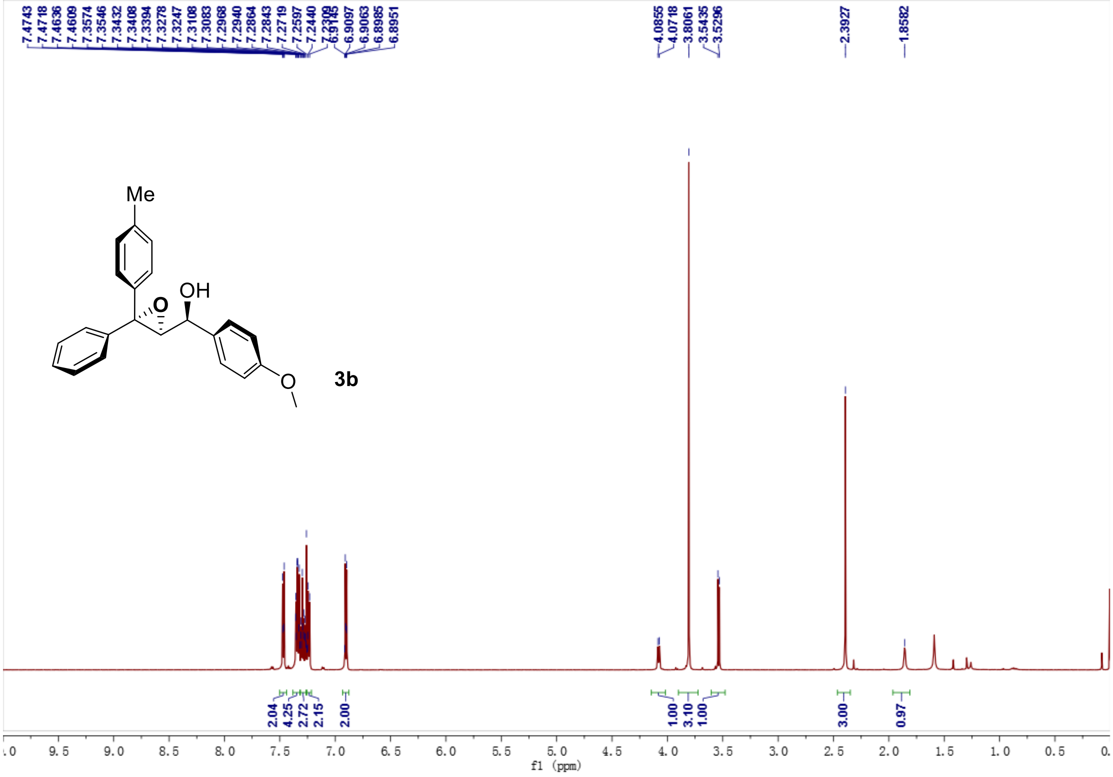

Supplementary Figure 153. ^{13}C NMR sepctra of (*S*)-(4-methoxyphenyl)((2*S*,3*R*)-3-phenyl-3-(*p*-tolyl)oxiran-2-yl)methanol (**3b**)

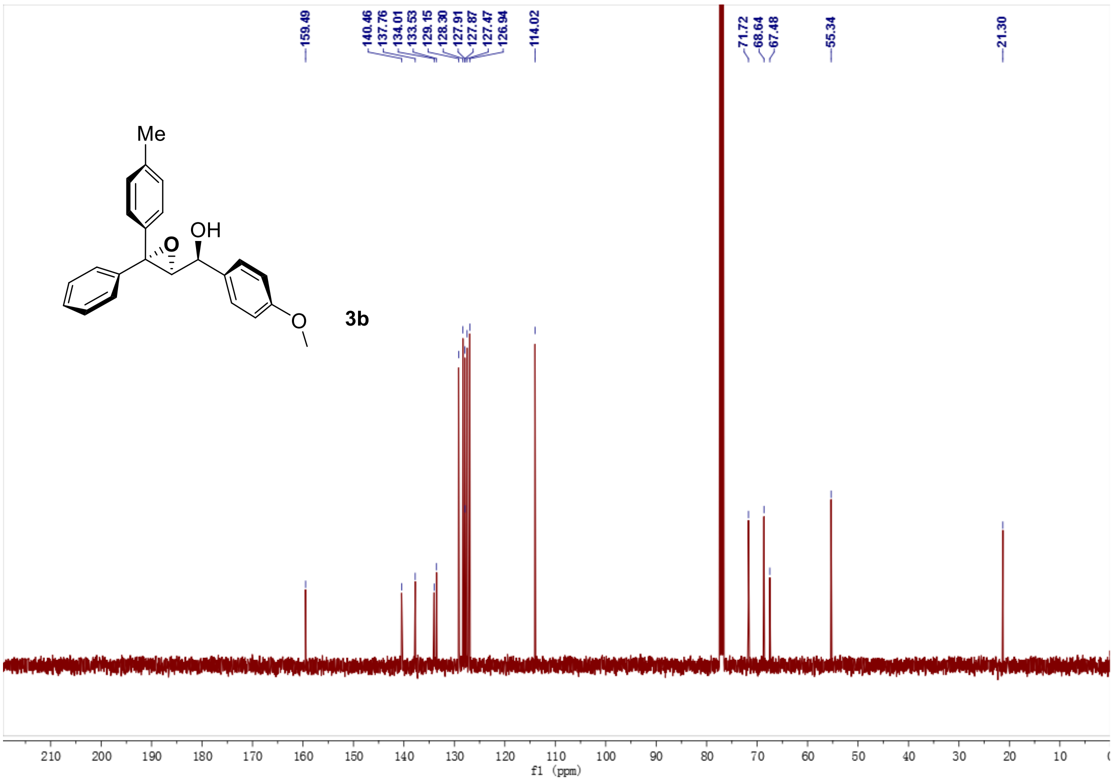

Supplementary Figure 154. ^{1}H NMR sepctra of (*R*)-(4-methoxyphenyl)((2*R*,3*S*)-3-phenyl-3-(*p*-tolyl)oxiran-2-yl)methyl propionate (**4b**)

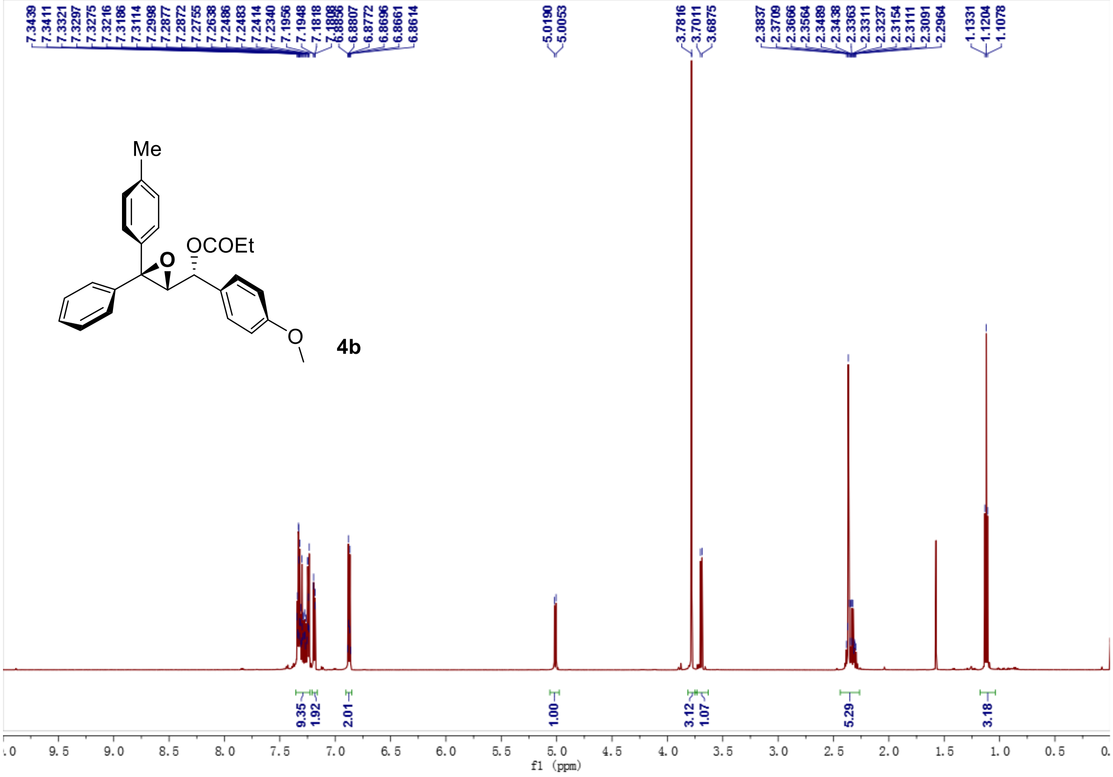

Supplementary Figure 155. ^{13}C NMR sepctra of (*R*)-(4-methoxyphenyl)((2*R*,3*S*)-3-phenyl-3-(*p*-tolyl)oxiran-2-yl)methyl propionate (**4b**)

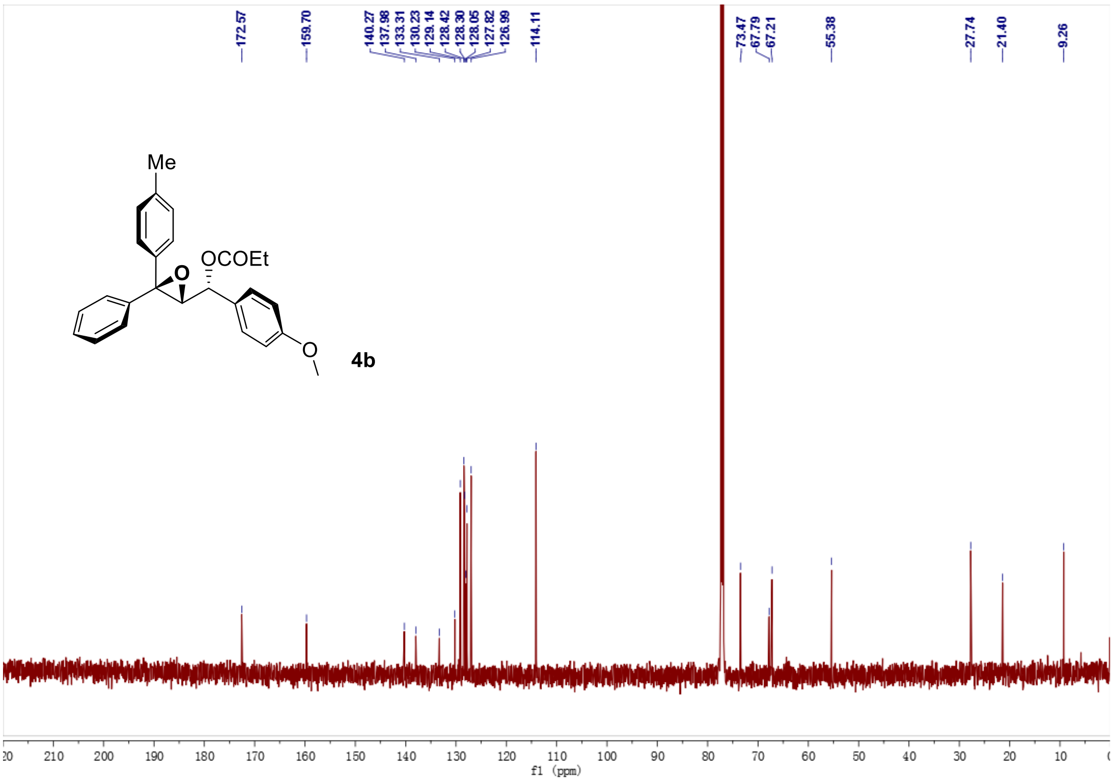

Supplementary Figure 156. ^{1}H NMR sepctra of (*S*)-(4-fluorophenyl)((2*S*,3*R*)-3-phenyl-3-(*p*-tolyl)oxiran-2-yl)methanol (**3c**)

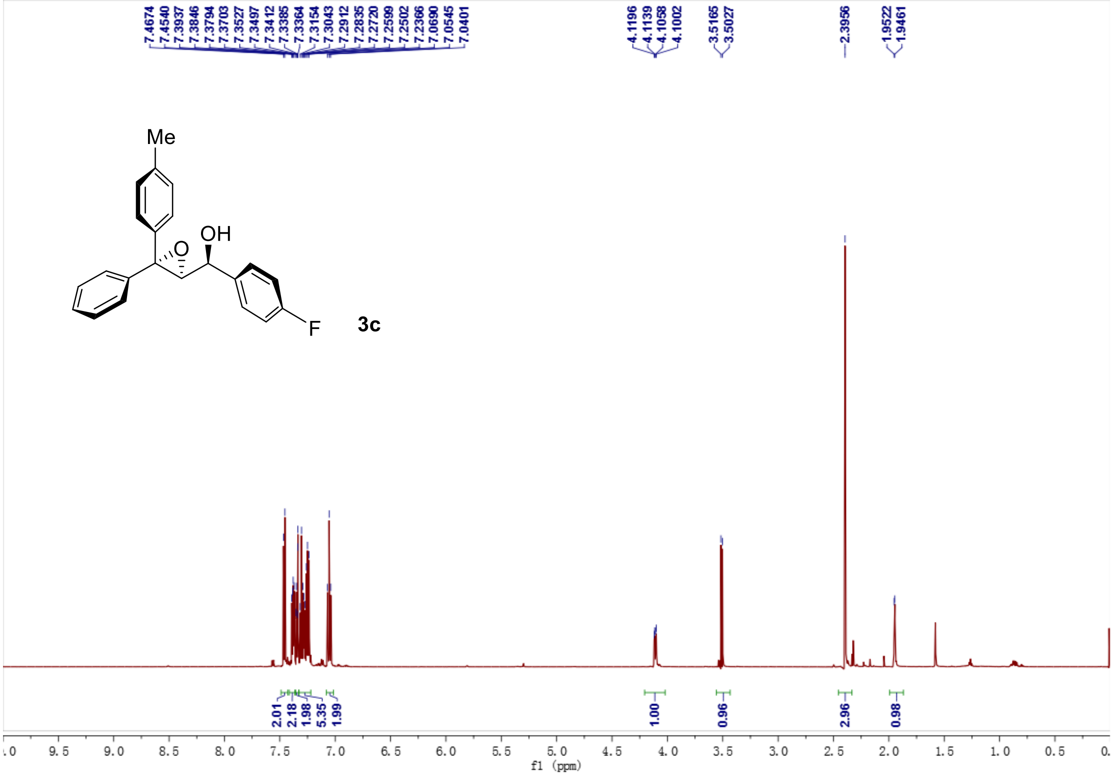

Supplementary Figure 157. ^{13}C NMR sepctra of (*S*)-(4-fluorophenyl)((2*S*,3*R*)-3-phenyl-3-(*p*-tolyl)oxiran-2-yl)methanol (**3c**)

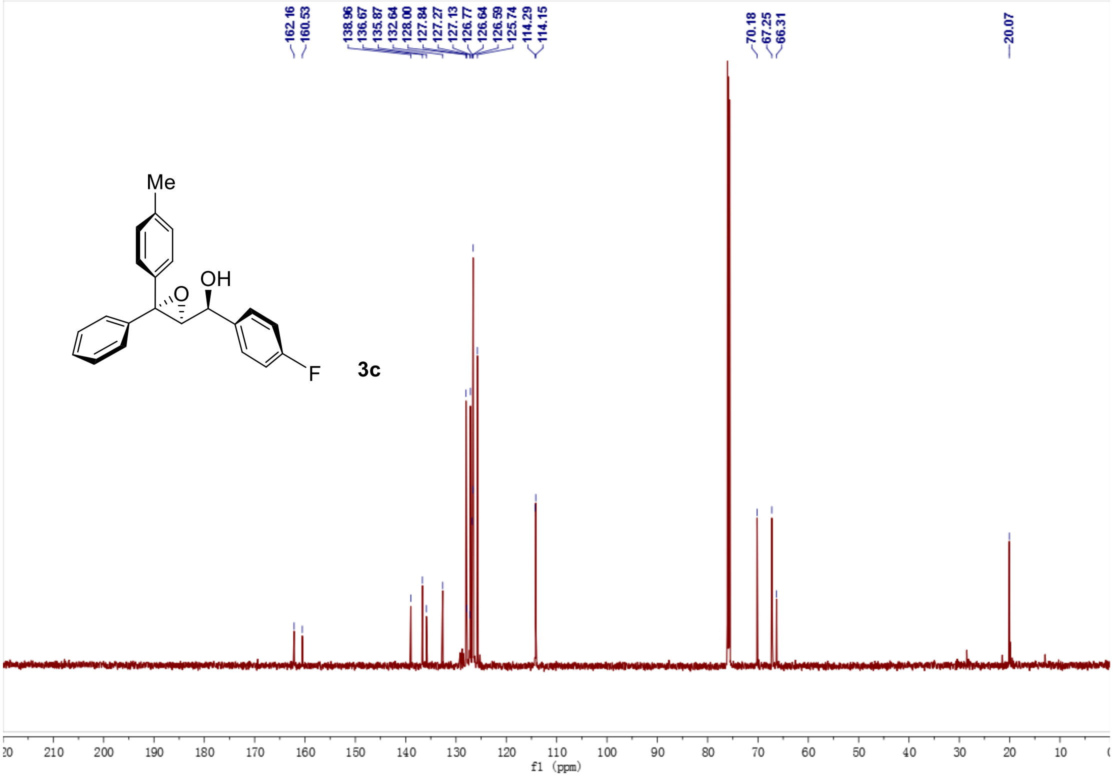

Supplementary Figure 158. ^{1}H NMR sepctra of (*R*)-(4-fluorophenyl)((2*R*,3*S*)-3-phenyl-3-(*p*-tolyl)oxiran-2-yl)methyl propionate (**4c**)

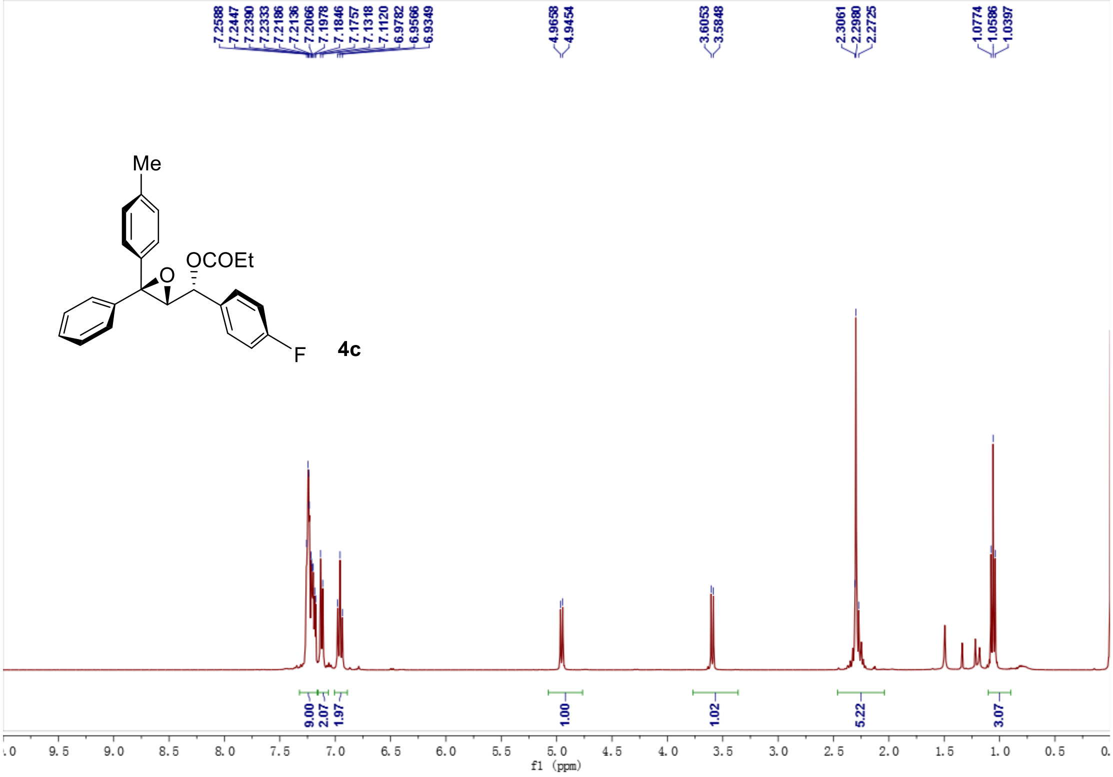

Supplementary Figure 159. ^{13}C NMR sepctra of (*R*)-(4-fluorophenyl)((2*R*,3*S*)-3-phenyl-3-(*p*-tolyl)oxiran-2-yl)methyl propionate (**4c**)

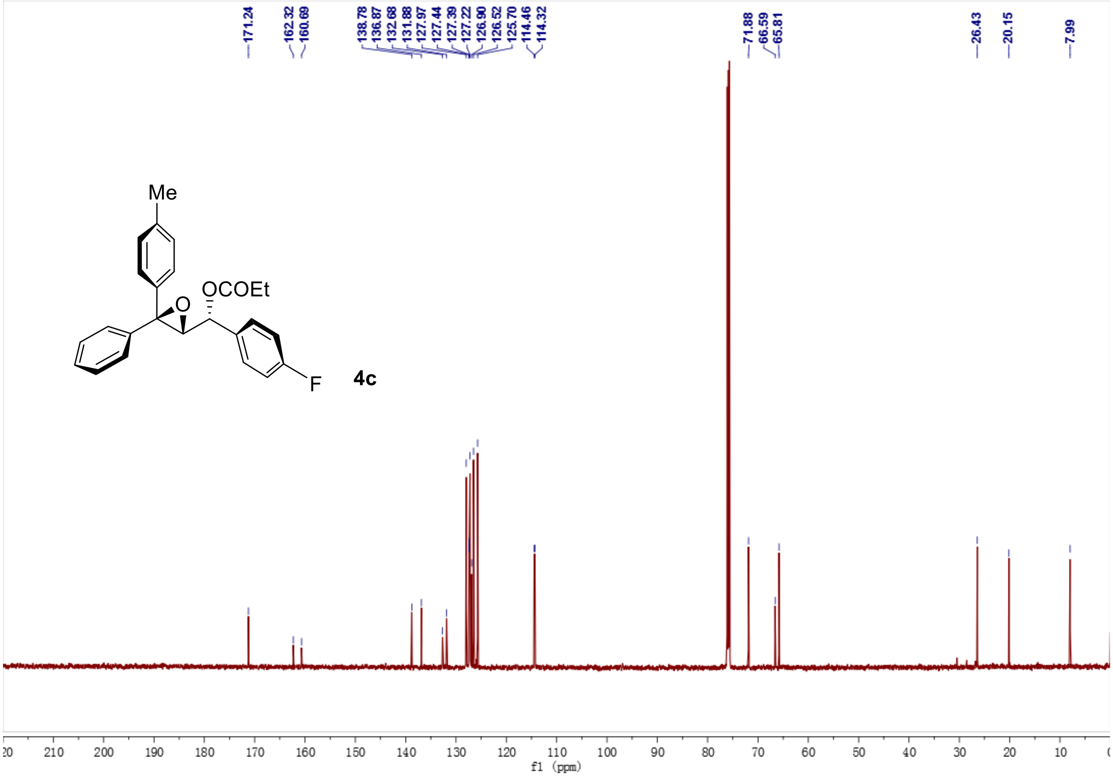

Supplementary Figure 160. ^{1}H NMR sepctra of (*S*)-naphthalen-1-yl((2*S*,3*R*)-3-phenyl-3-(*p*-tolyl)oxiran-2-yl)methanol (**3d**)

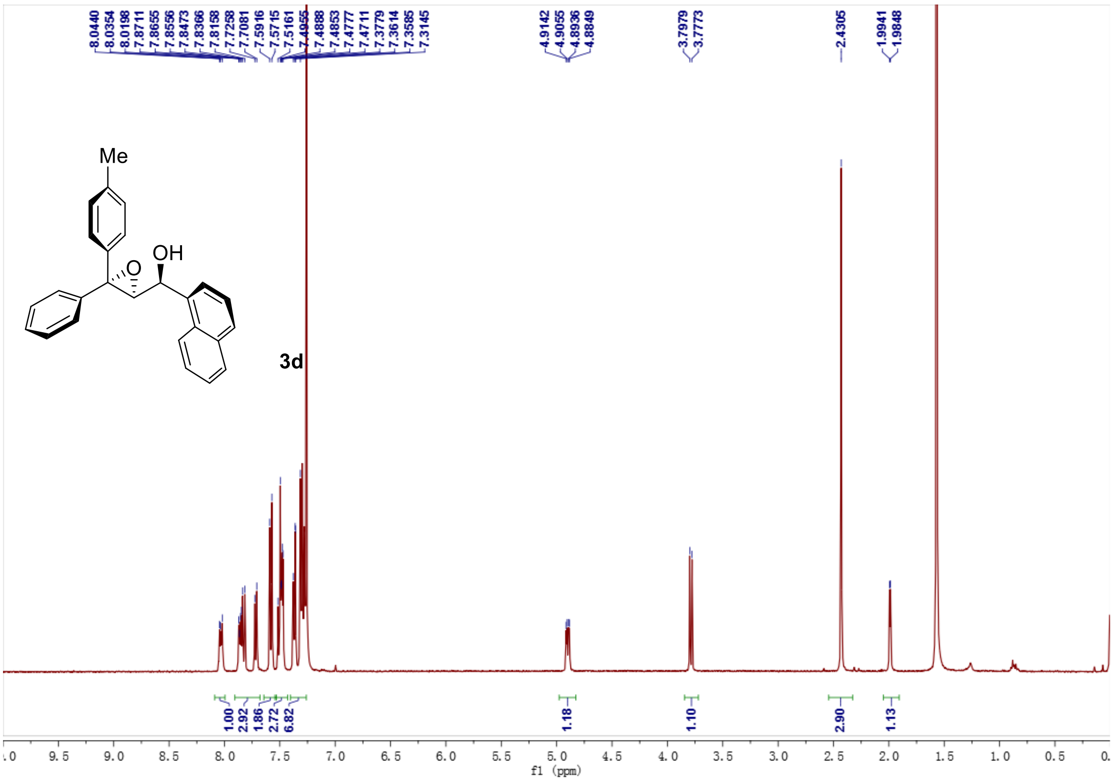

Supplementary Figure 161. ^{13}C NMR sepctra of (*S*)-naphthalen-1-yl((2*S*,3*R*)-3-phenyl-3-(*p*-tolyl)oxiran-2-yl)methanol (**3d**)

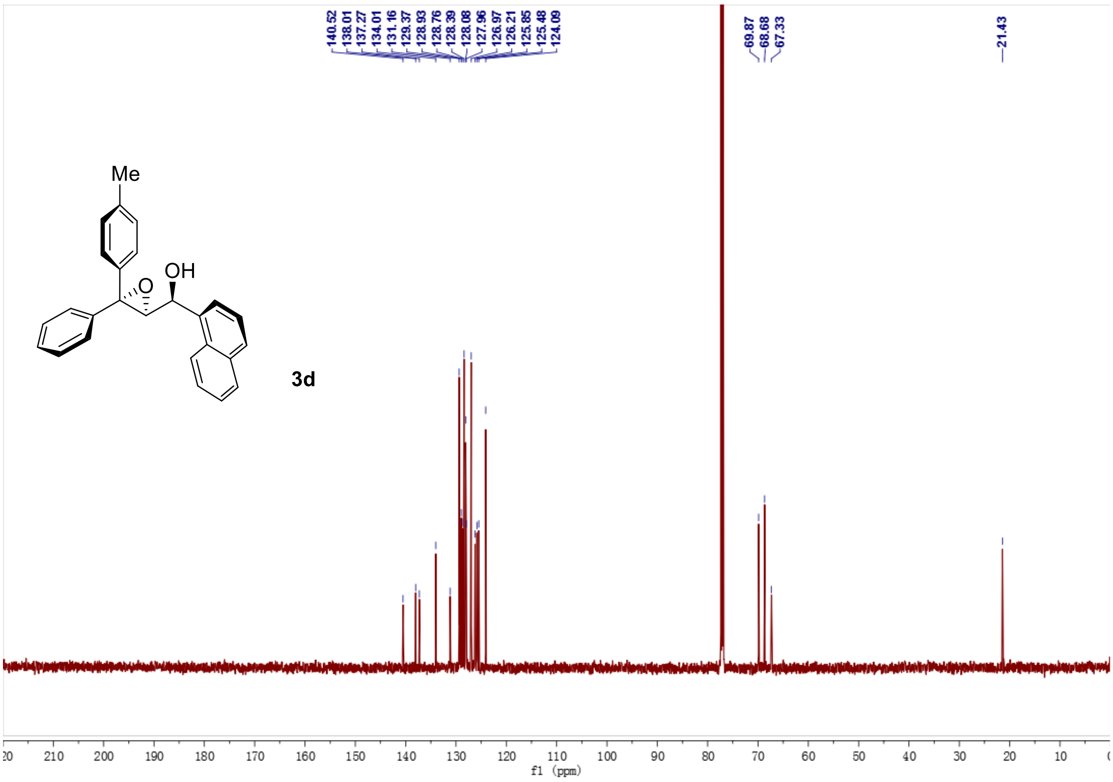

Supplementary Figure 162. ^{1}H NMR sepctra of (*R*)-naphthalen-1-yl((2*R*,3*S*)-3-phenyl-3-(*p*-tolyl)oxiran-2-yl)methyl propionate (**4d**)

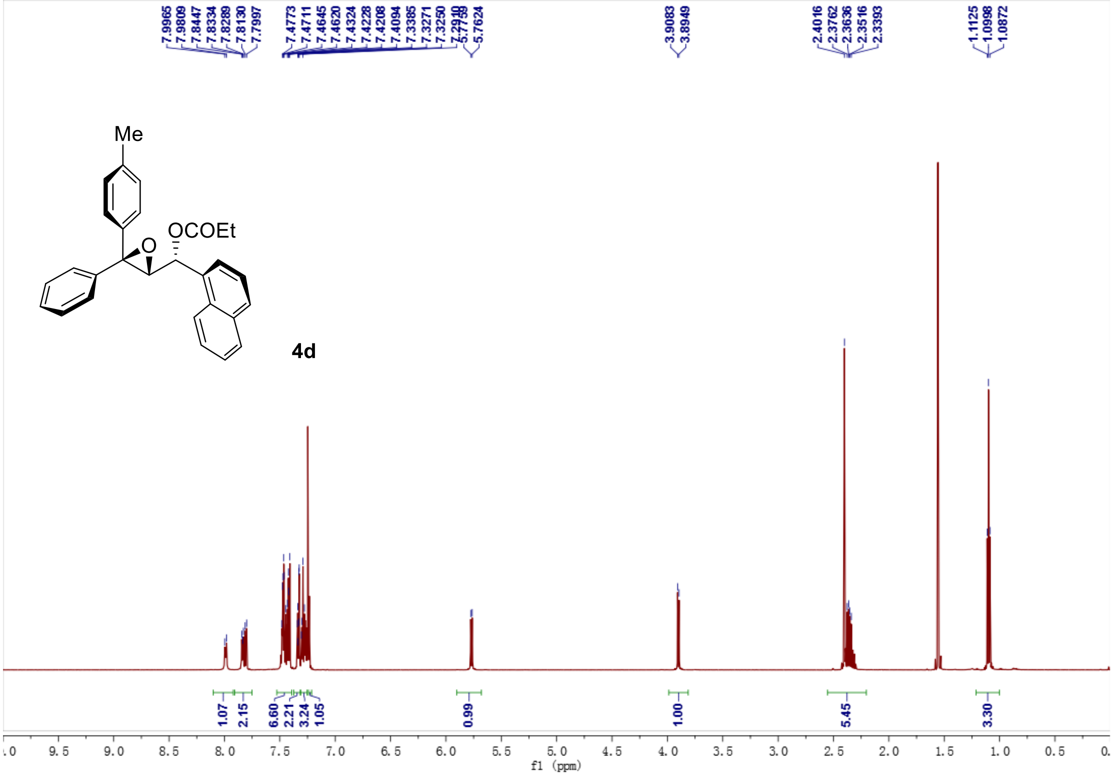

Supplementary Figure 163. ^{13}C NMR sepctra of (*R*)-naphthalen-1-yl((2*R*,3*S*)-3-phenyl-3-(*p*-tolyl)oxiran-2-yl)methyl propionate (**4d**)

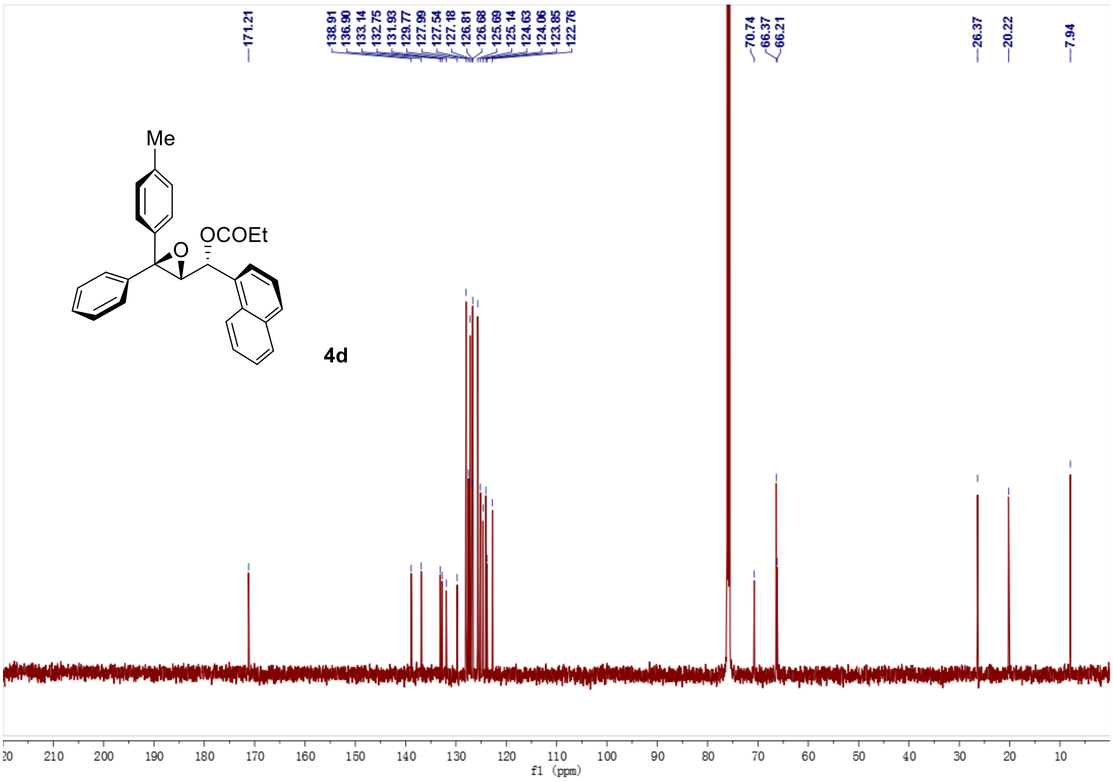

Supplementary Figure 164. ^{1}H NMR sepctra of (*S*)-((2*S*,3*S*)-3-(3-chlorophenyl)-3-(4-chlorophenyl)oxiran-2-yl)(phenyl)methanol (**3e**)

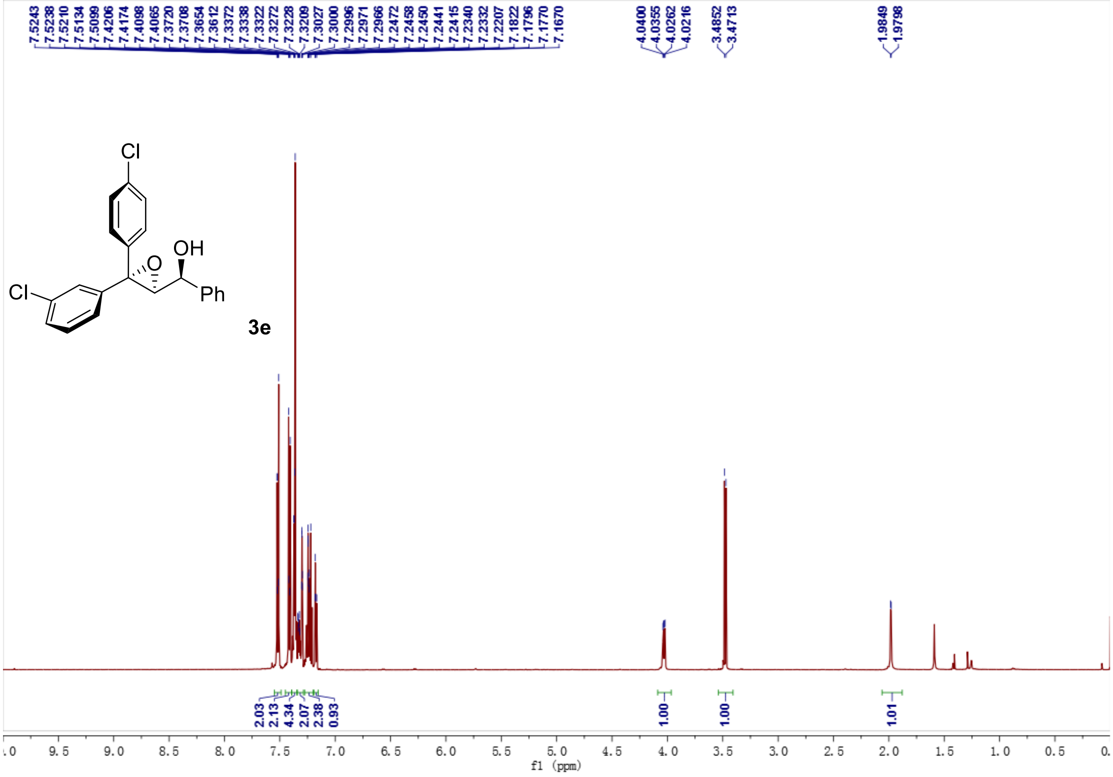

Supplementary Figure 165. ^{13}C NMR sepctra of (*S*)-((2*S*,3*S*)-3-(3-chlorophenyl)-3-(4-chlorophenyl)oxiran-2-yl)(phenyl)methanol (**3e**)

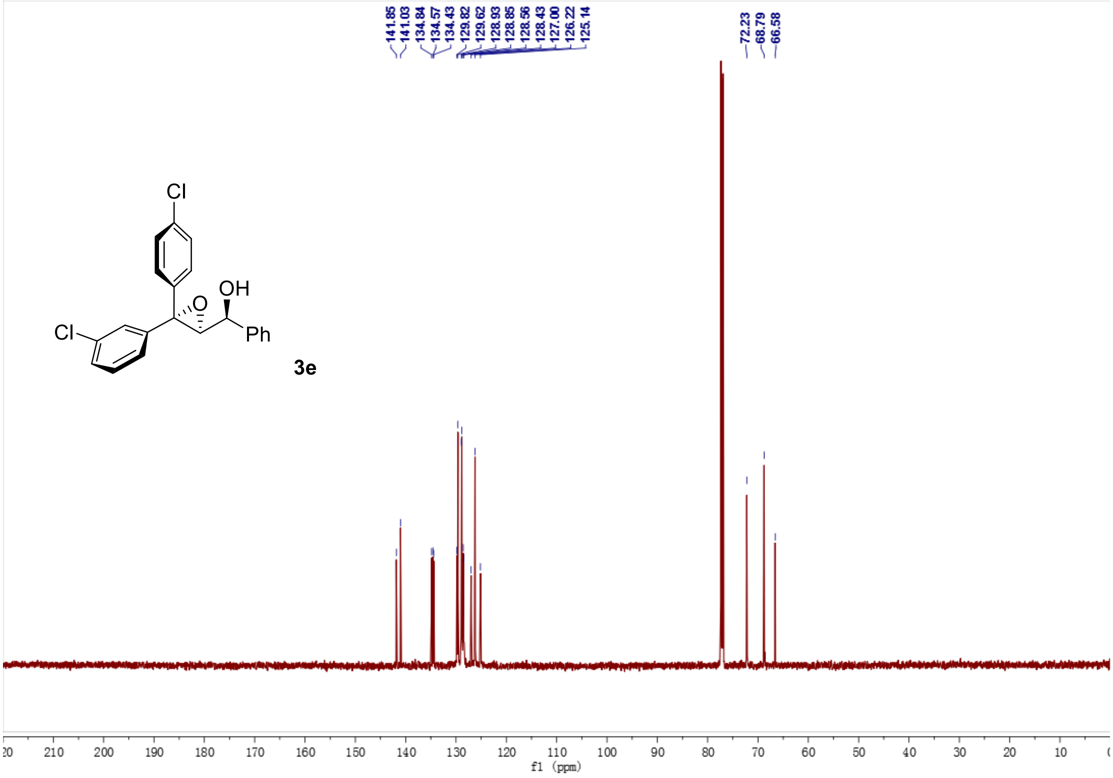

Supplementary Figure 166. ^{1}H NMR sepctra of (*R*)-((2*R*,3*R*)-3-(3-chlorophenyl)-3-(4-chlorophenyl)oxiran-2-yl)(phenyl)methyl propionate (**4e**)

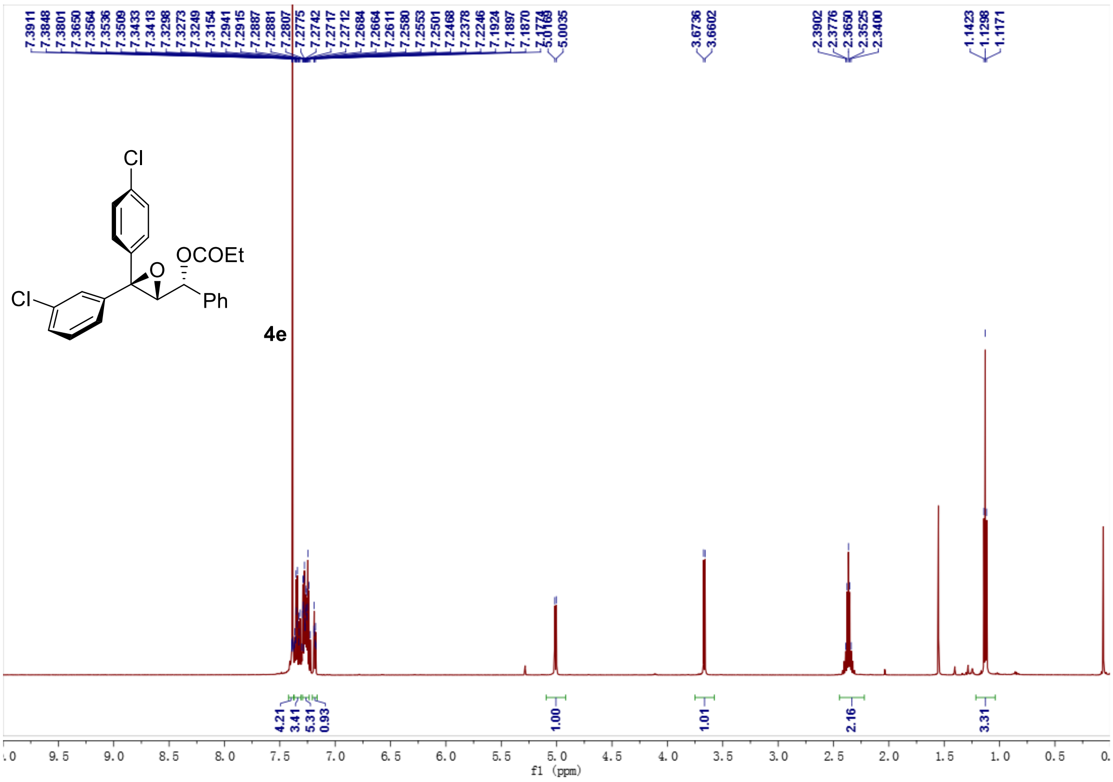

Supplementary Figure 167. ^{13}C NMR sepctra of (*R*)-((2*R*,3*R*)-3-(3-chlorophenyl)-3-(4-chlorophenyl)oxiran-2-yl)(phenyl)methyl propionate (**4e**)

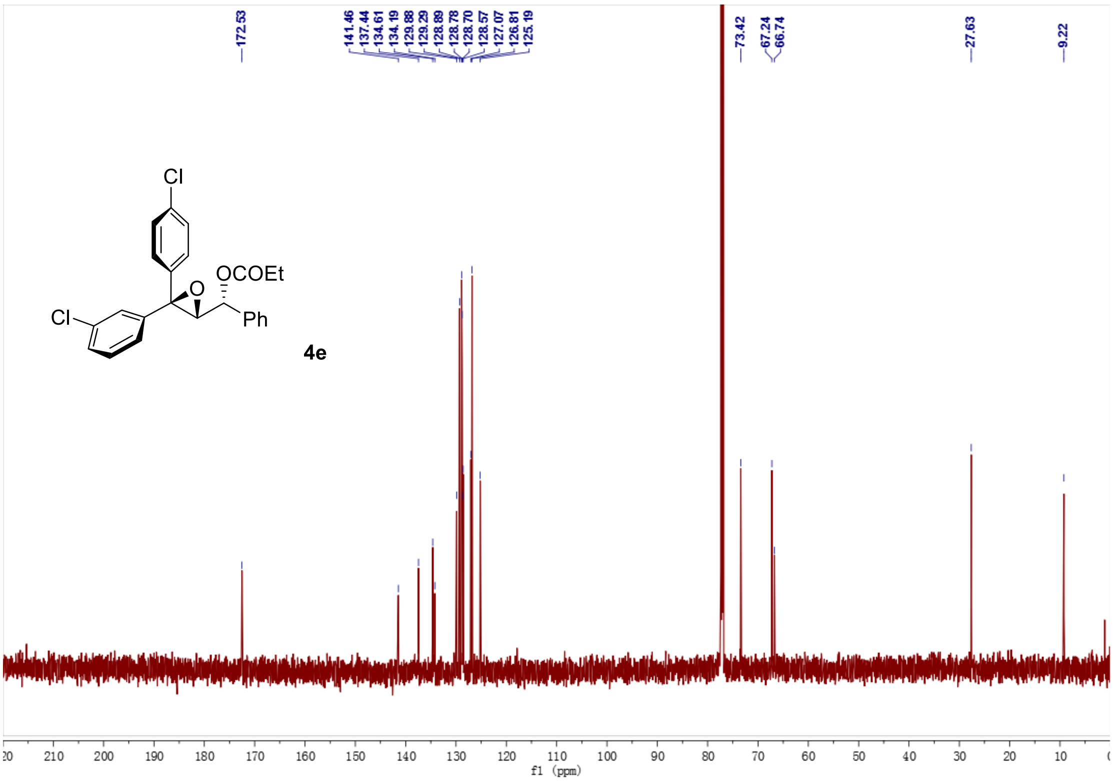

Supplementary Figure 168. ^{1}H NMR sepctra of (*S*)-((2*S*,3*S*)-3-(4-ethylphenyl)-3-(*p*-tolyl)oxiran-2-yl)(phenyl)methanol (**3f**)

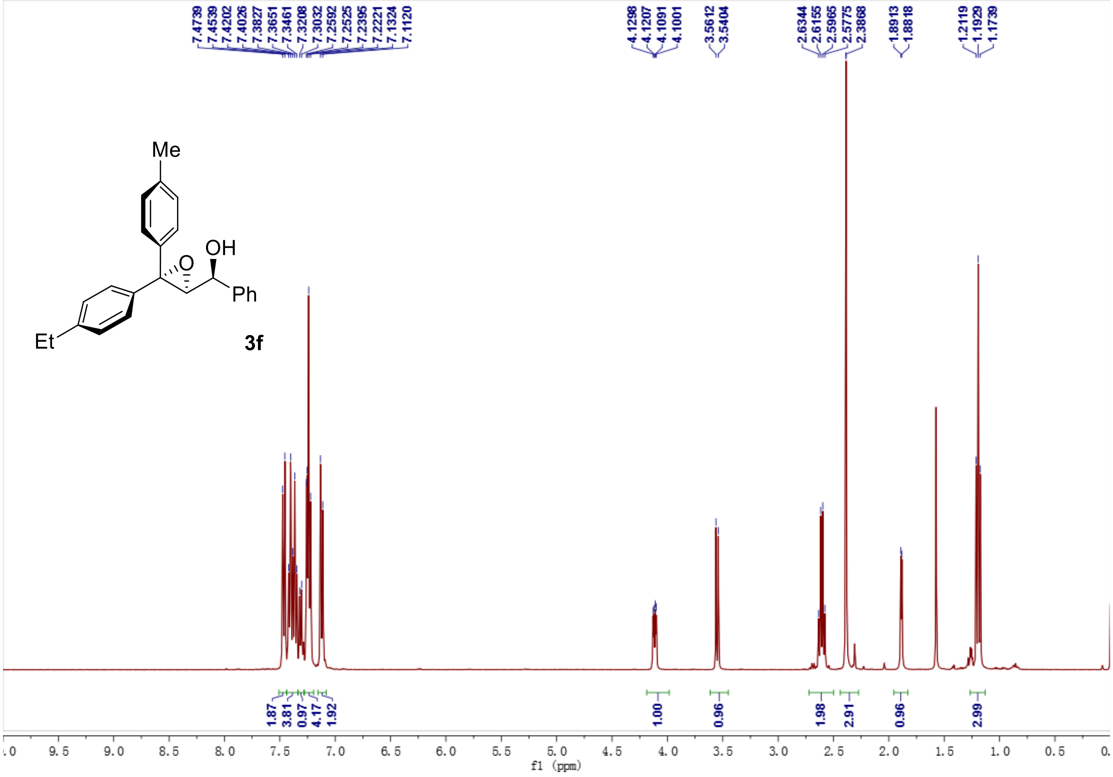

Supplementary Figure 169. ^{13}C NMR sepctra of (*S*)-((2*S*,3*S*)-3-(4-ethylphenyl)-3-(*p*-tolyl)oxiran-2-yl)(phenyl)methanol (**3f**)

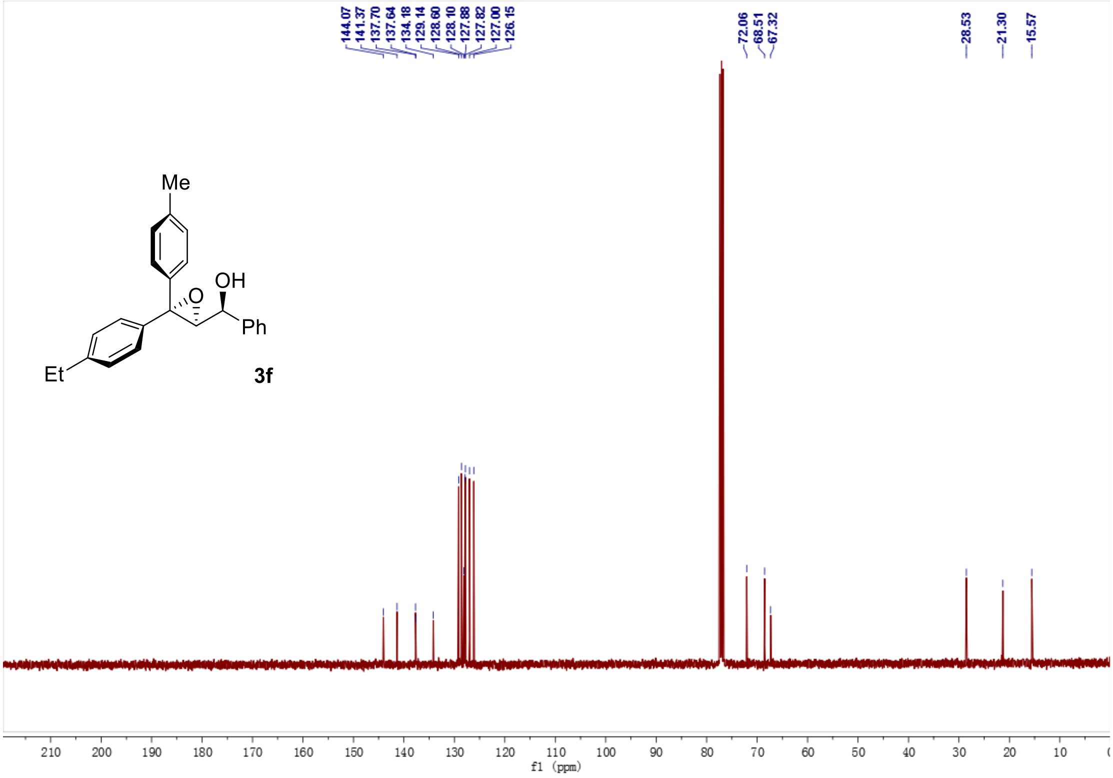

Supplementary Figure 170. ^{1}H NMR sepctra of (*S*)-((2*S*,3*S*)-3-(4-ethylphenyl)-3-(*p*-tolyl)oxiran-2-yl)(phenyl)methyl propionate (**4f**)

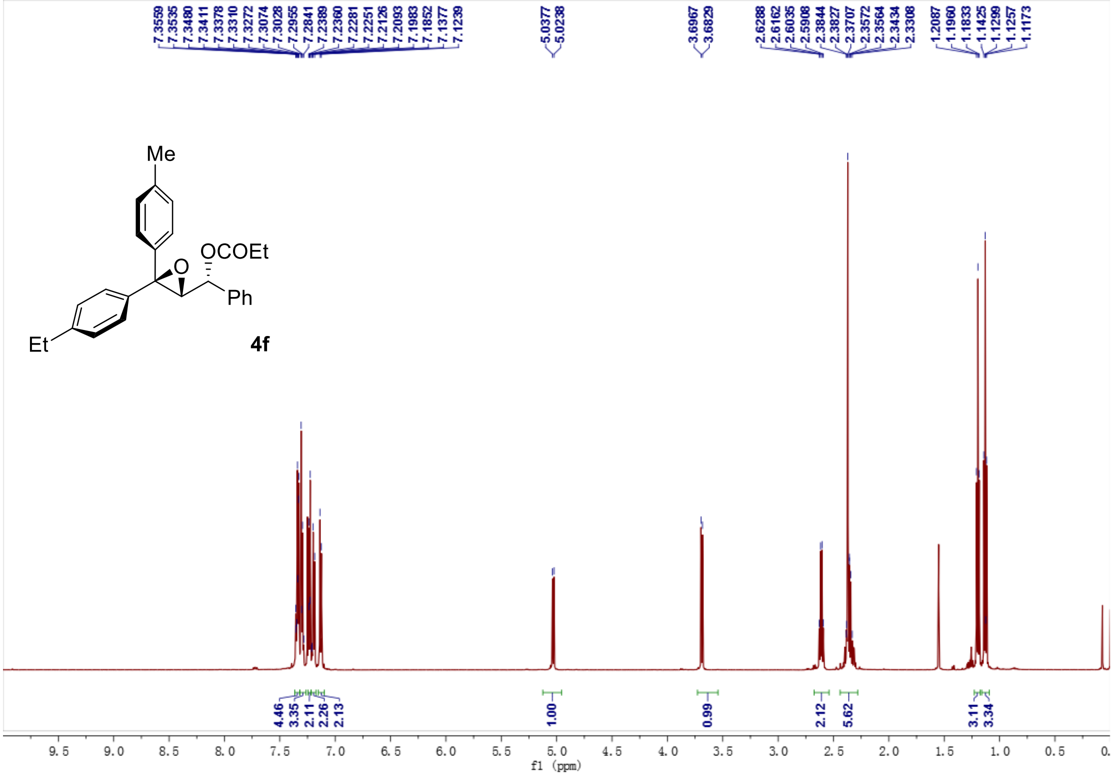

Supplementary Figure 171. ^{13}C NMR sepctra of (*S*)-((2*S*,3*S*)-3-(4-ethylphenyl)-3-(*p*-tolyl)oxiran-2-yl)(phenyl)methyl propionate (**4f**)

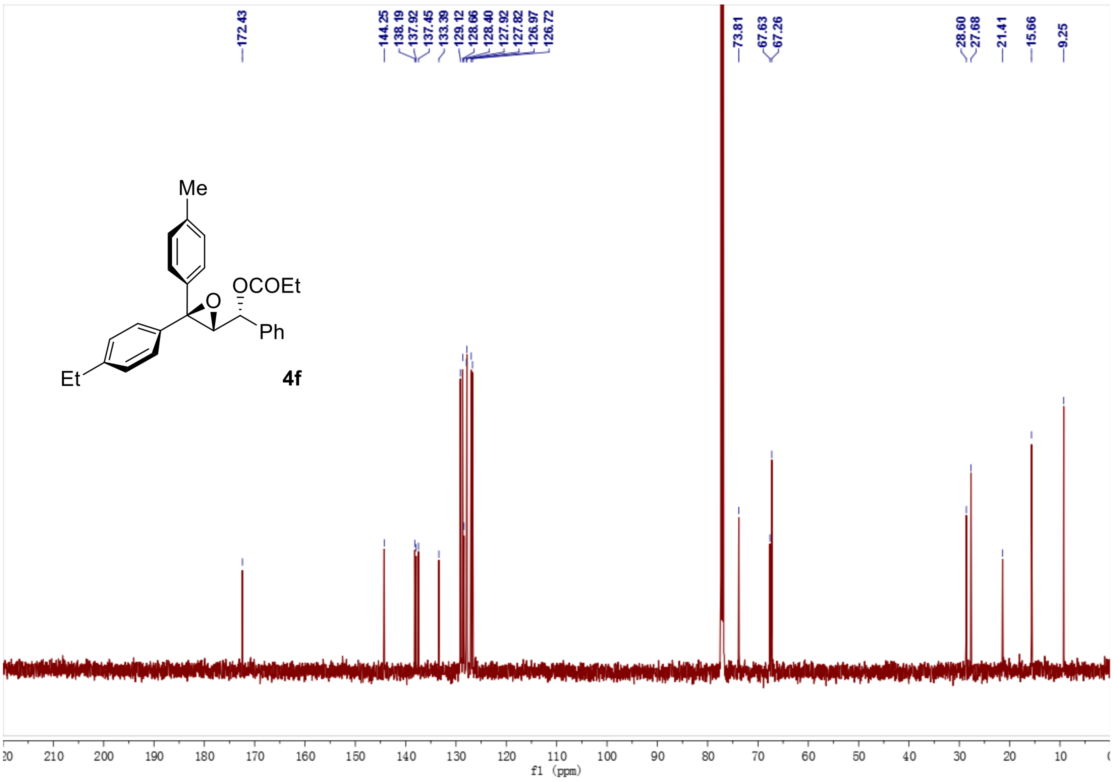

Supplementary Figure 172. ^{1}H NMR sepctra of (*S*)-((2*S*,3*R*)-3-(3-chlorophenyl)-3-(4-chlorophenyl)oxiran-2-yl)(phenyl)methanol (**3g**)

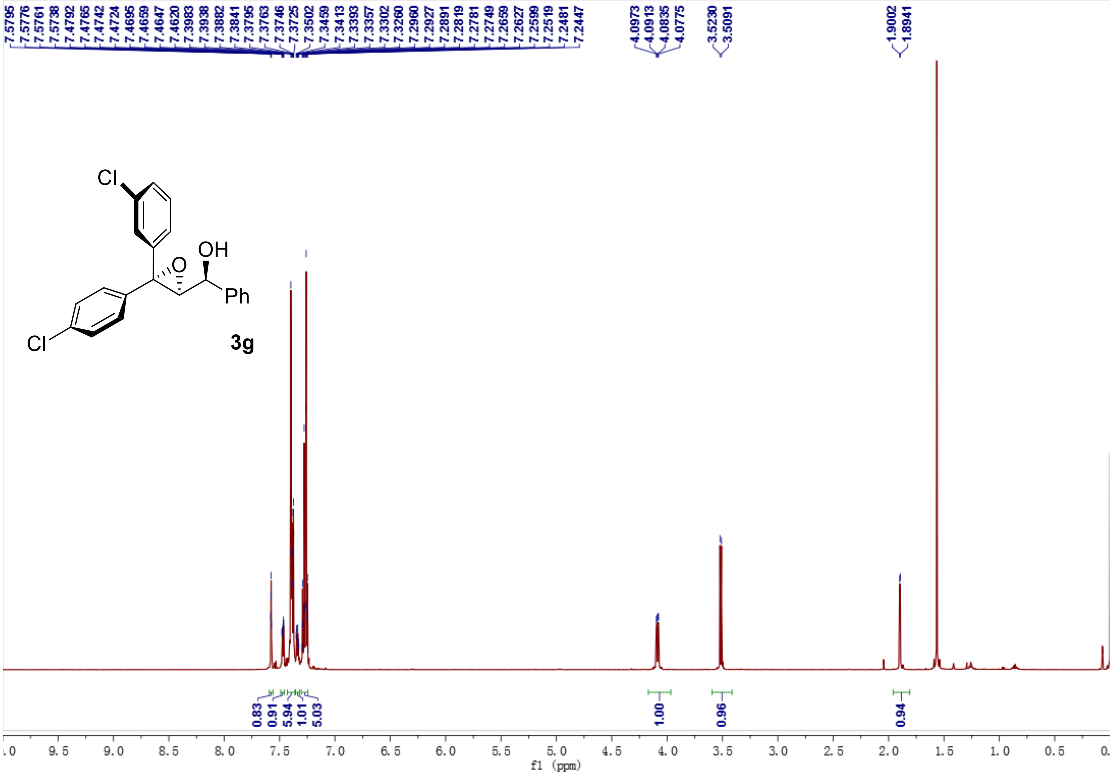

Supplementary Figure 173. ^{13}C NMR sepctra of (*S*)-((2*S*,3*R*)-3-(3-chlorophenyl)-3-(4-chlorophenyl)oxiran-2-yl)(phenyl)methanol (**3g**)

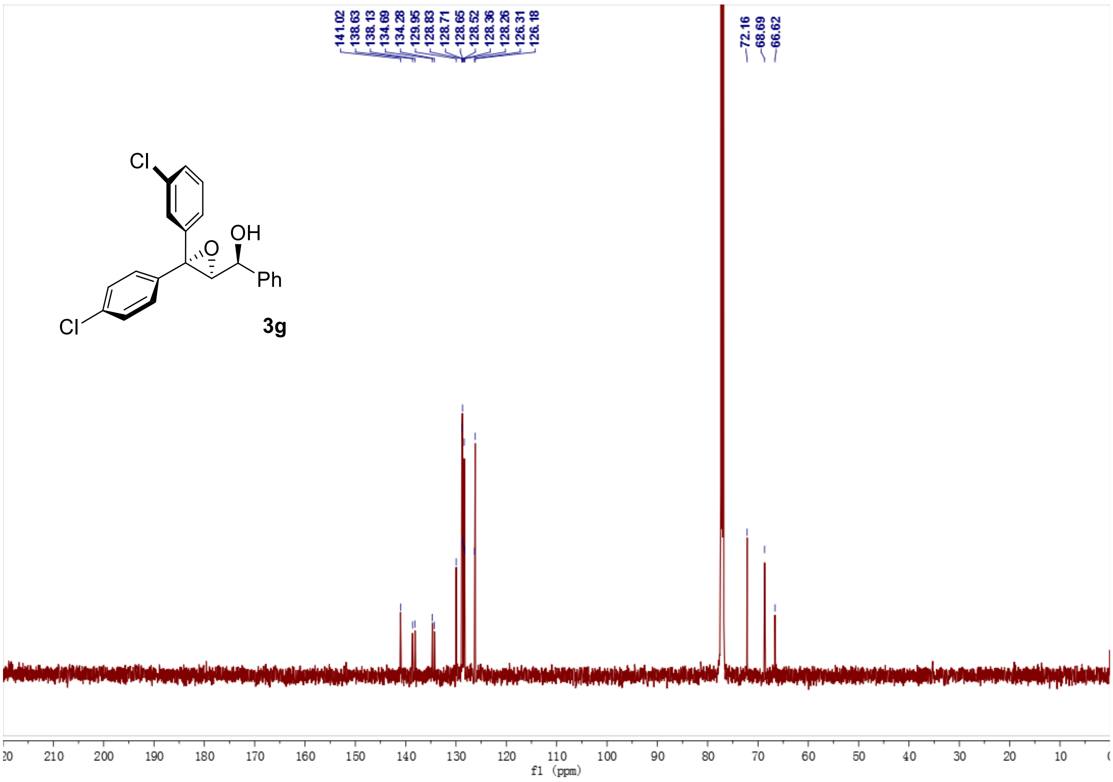

Supplementary Figure 174. ^{1}H NMR sepctra of (*R*)-((2*R*,3*S*)-3-(3-chlorophenyl)-3-(4-chlorophenyl)oxiran-2-yl)(phenyl)methyl propionate (**4g**)

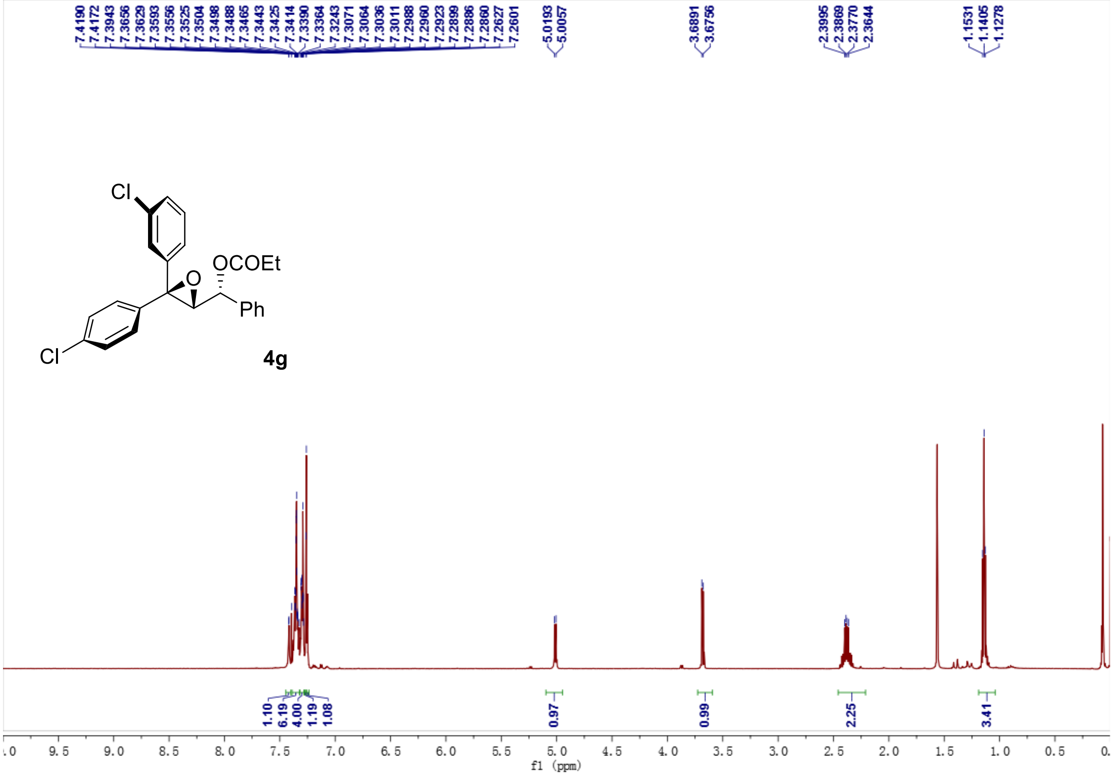

Supplementary Figure 175. ^{13}C NMR sepctra of (*R*)-((2*R*,3*S*)-3-(3-chlorophenyl)-3-(4-chlorophenyl)oxiran-2-yl)(phenyl)methyl propionate (**4g**)

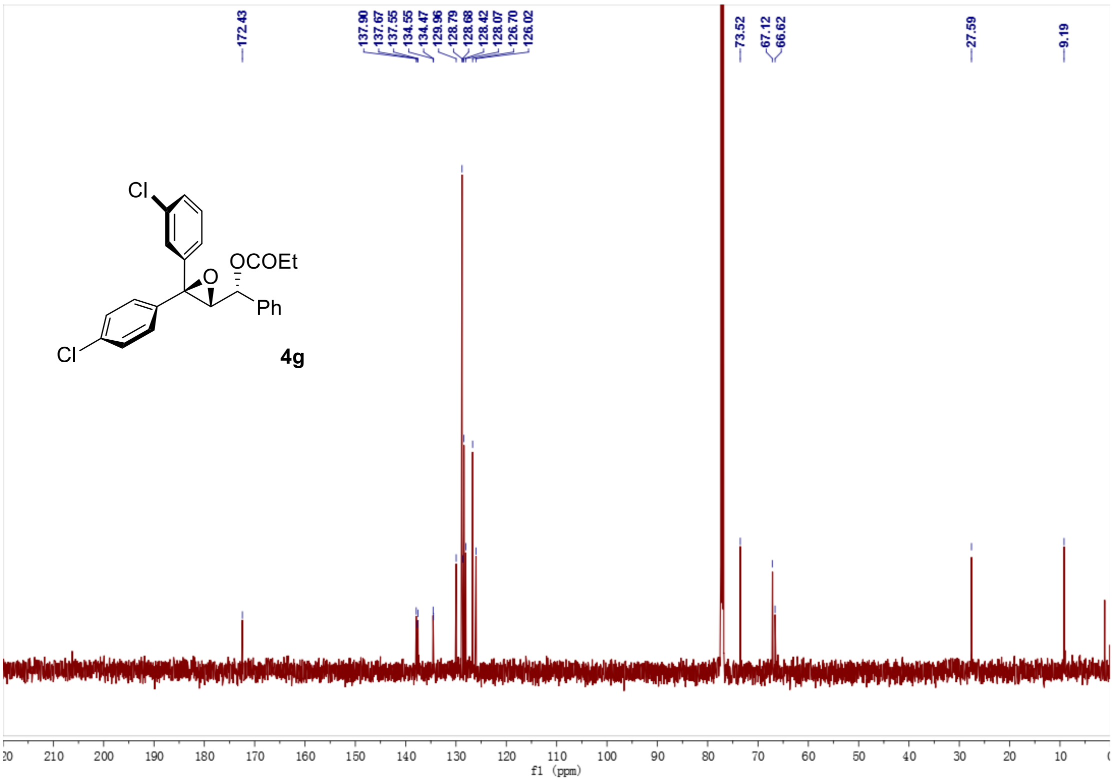

Supplementary Figure 176. ^{1}H NMR sepctra of (*S*)-((2*S*,3*R*)-3-hexyl-3-pentyloxiran-2-yl)(phenyl)methanol (**3h**)

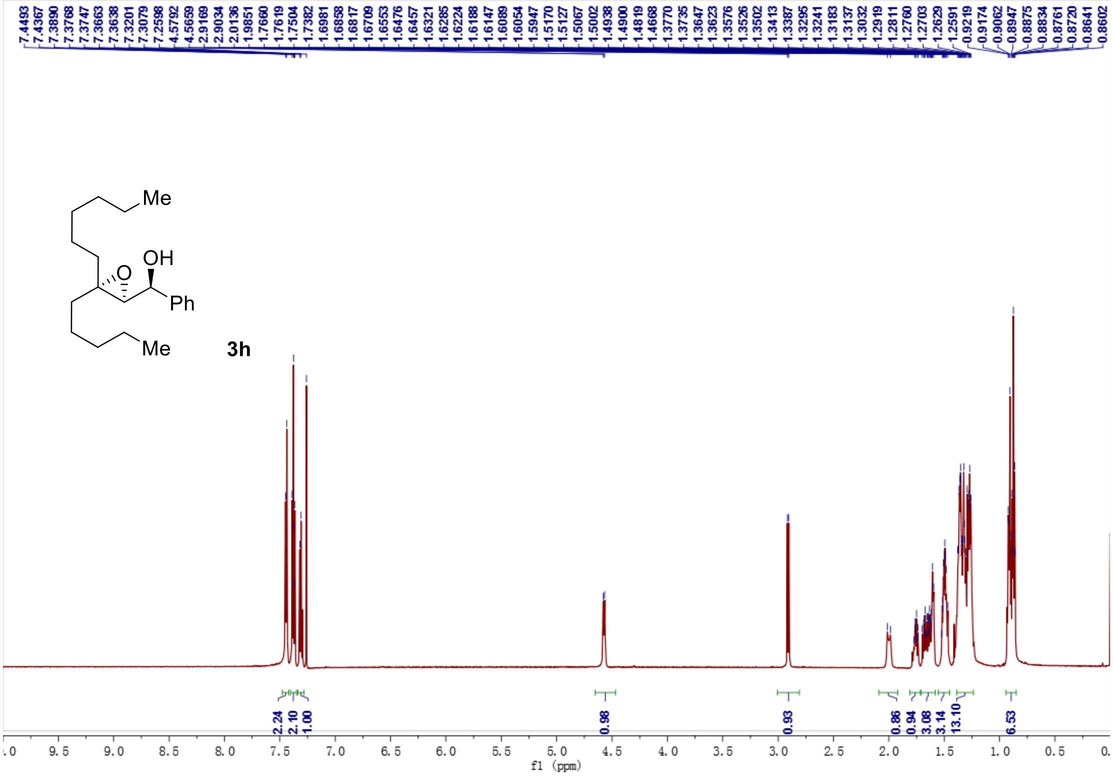

Supplementary Figure 177. ^{13}C NMR sepctra of (*S*)-((2*S*,3*R*)-3-hexyl-3-pentyloxiran-2-yl)(phenyl)methanol (**3h**)

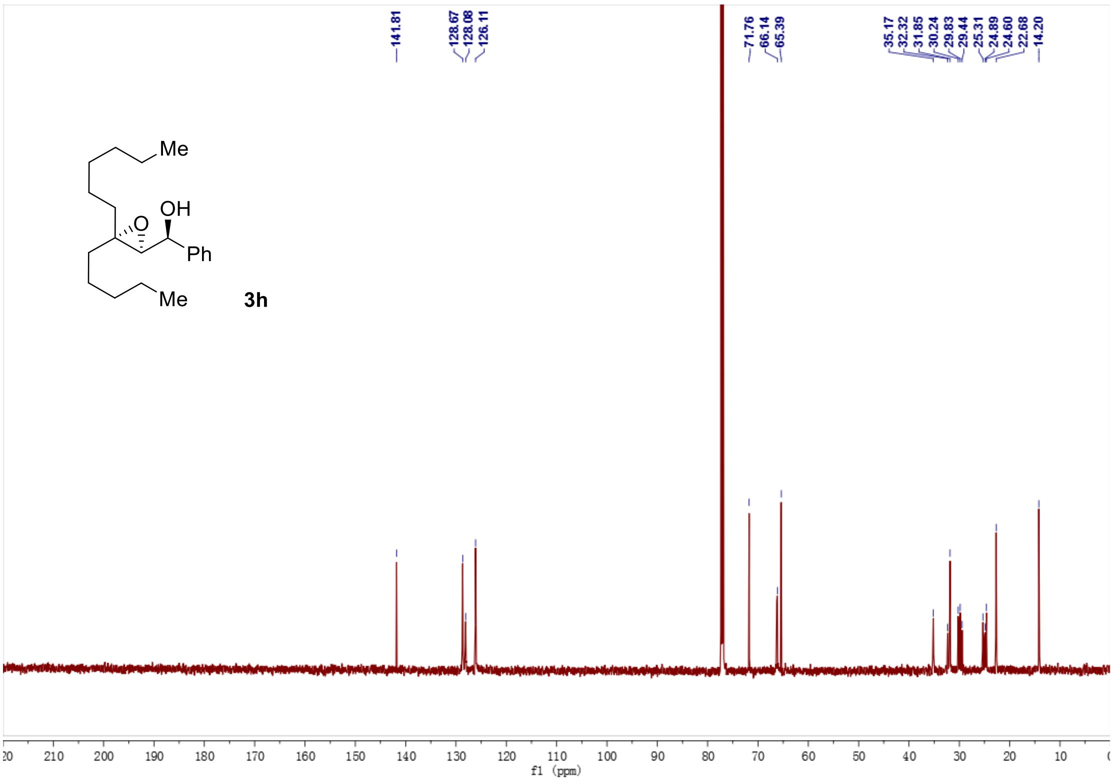

Supplementary Figure 178. ^{1}H NMR sepctra of (*S*)-((2*S*,3*R*)-3-hexyl-3-pentyloxiran-2-yl)(phenyl)methyl propionate (**4h**)

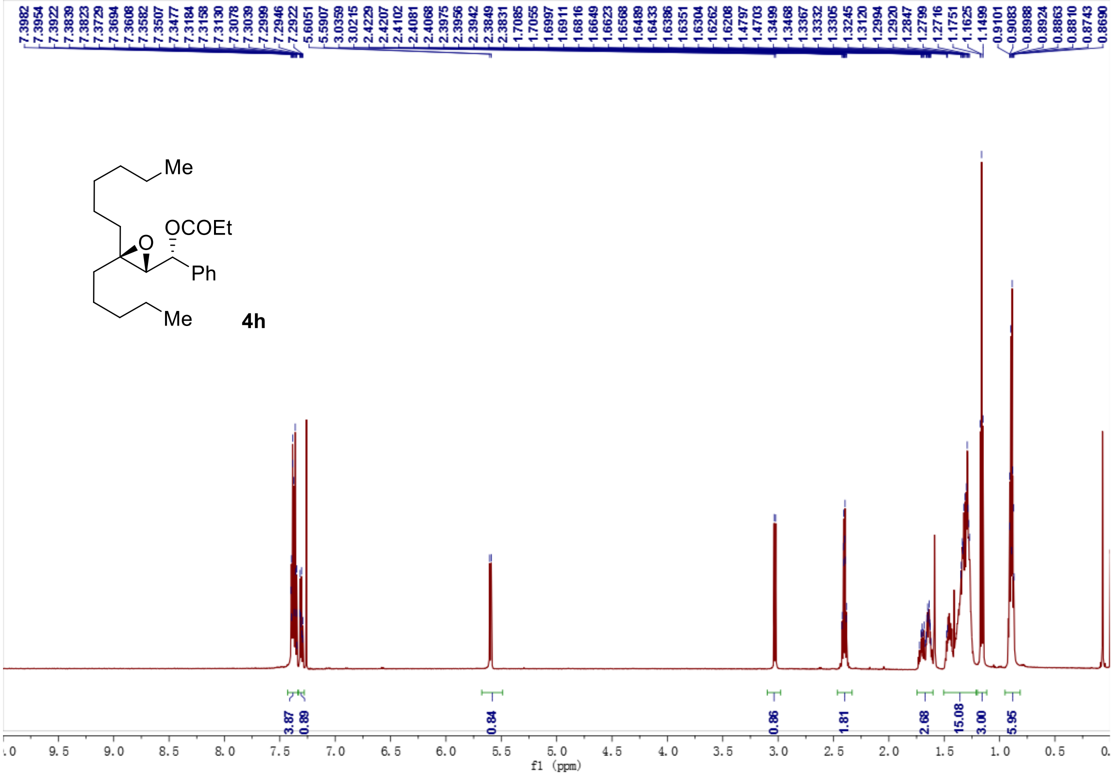

Supplementary Figure 179. ^{13}C NMR sepctra of (*S*)-((2*S*,3*R*)-3-hexyl-3-pentyloxiran-2-yl)(phenyl)methyl propionate (**4h**)

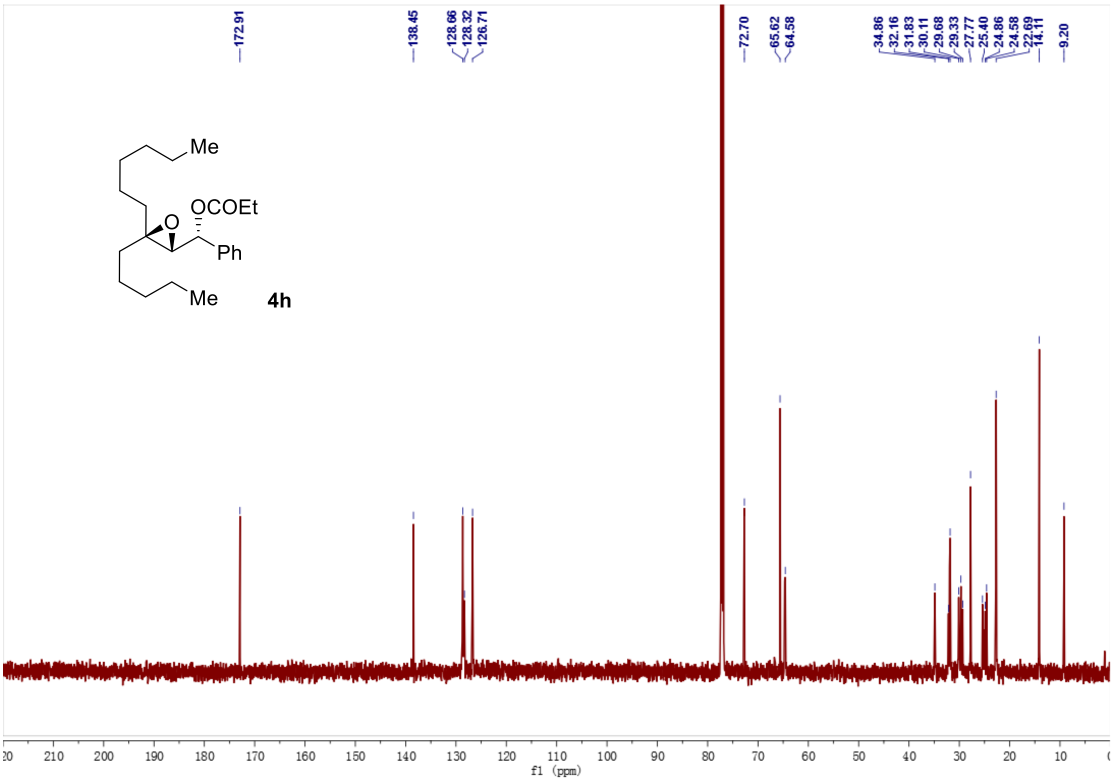

Supplementary Figure 180. ^{1}H NMR sepctra of (*S*)-((2*S*,3*R*)-3-methyl-3-(trifluoromethyl)oxiran-2-yl)(phenyl)methanol (**3i**)

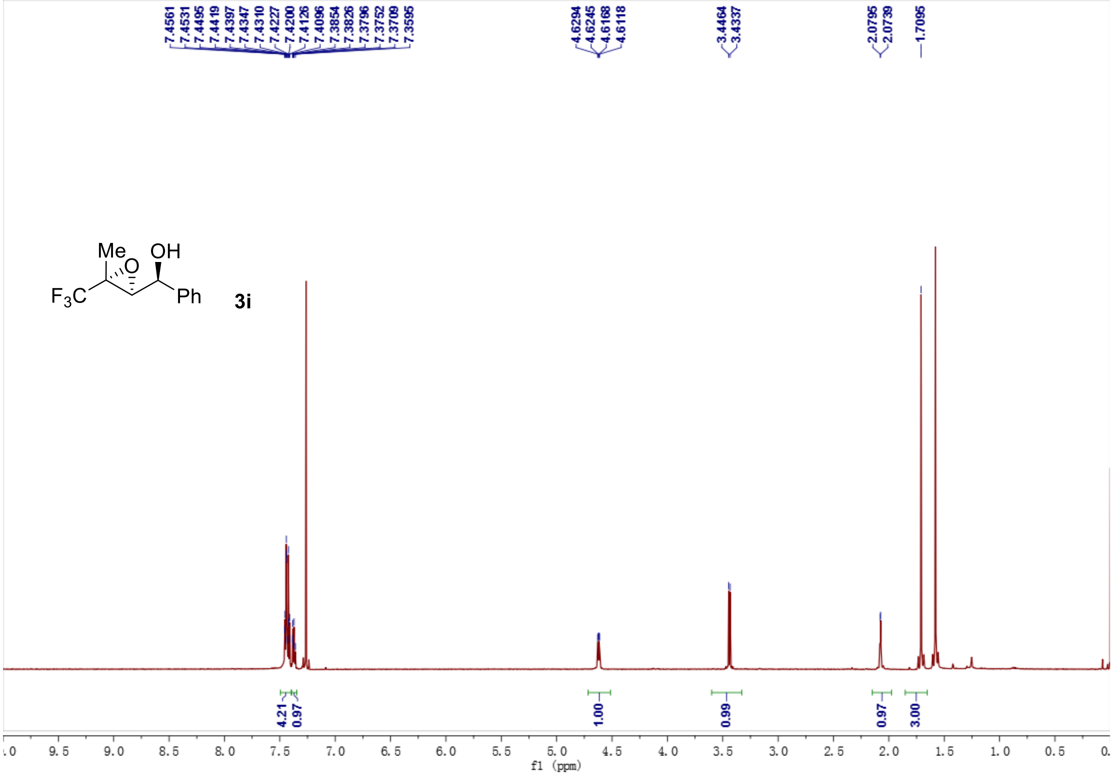

Supplementary Figure 181. ^{13}C NMR sepctra of (*S*)-((2*S*,3*R*)-3-methyl-3-(trifluoromethyl)oxiran-2-yl)(phenyl)methanol (**3i**)

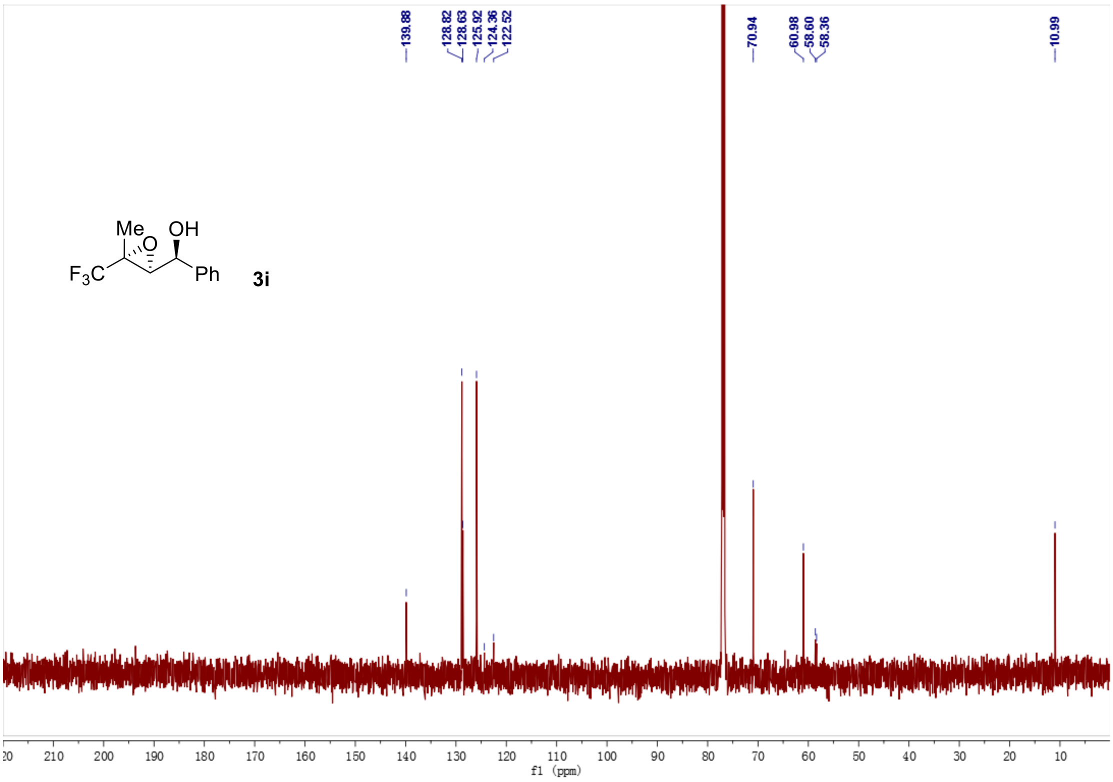

Supplementary Figure 182. ^{1}H NMR sepctra of (*R*)-((2*R*,3*S*)-3-methyl-3-(trifluoromethyl)oxiran-2-yl)(phenyl)methyl propionate (**4i**)

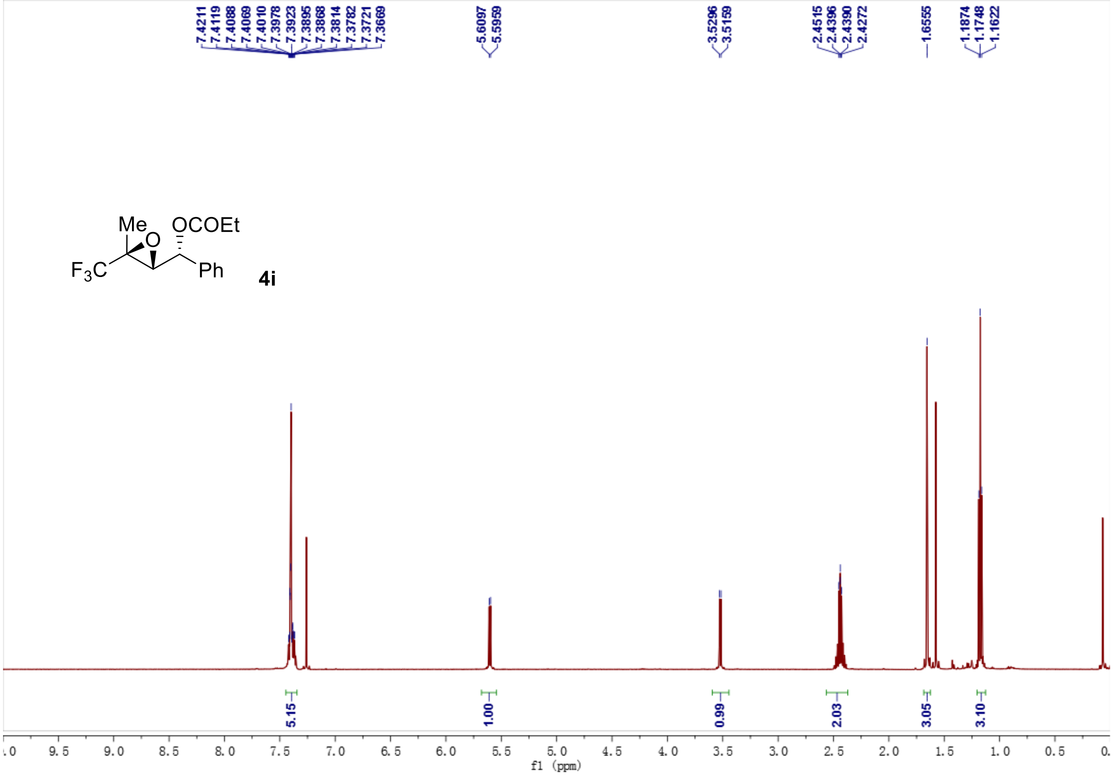

Supplementary Figure 183. ^{13}C NMR sepctra of (*R*)-((2*R*,3*S*)-3-methyl-3-(trifluoromethyl)oxiran-2-yl)(phenyl)methyl propionate (**4i**)

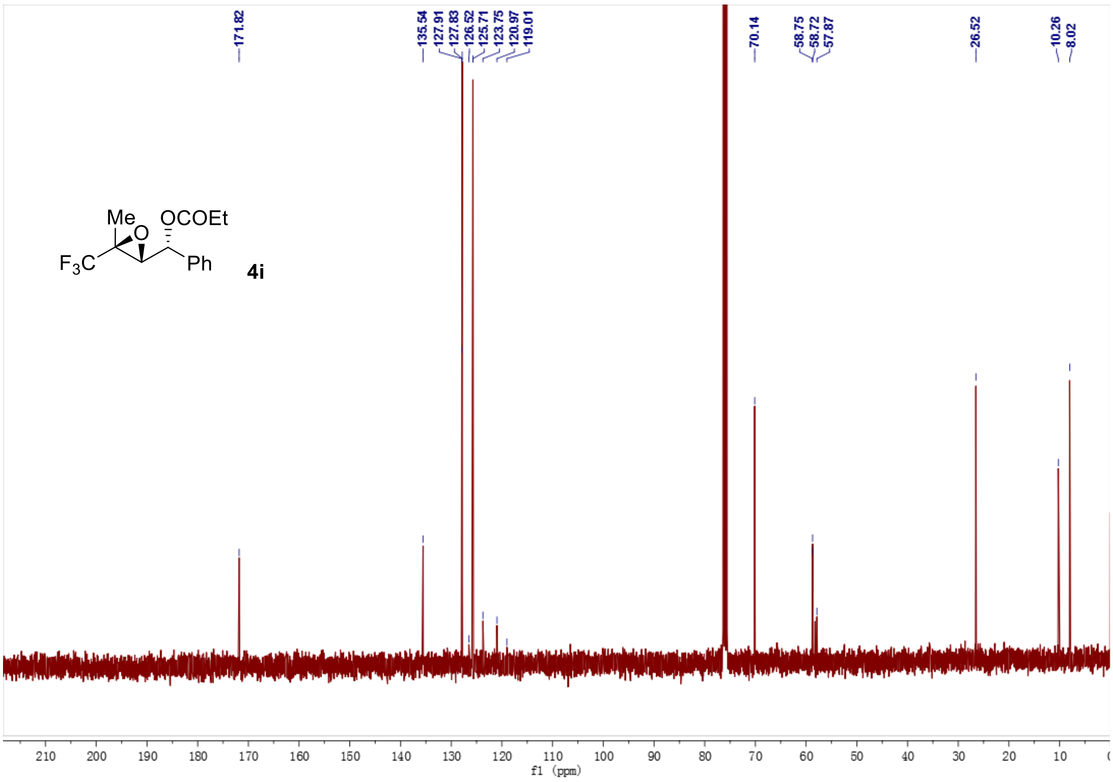

Supplementary Figure 184. ^{1}H NMR sepctra of (*S*)-((2*S*,3*S*)-3-ethyl-3-methyloxiran-2-yl)(phenyl)methanol (**3j**)

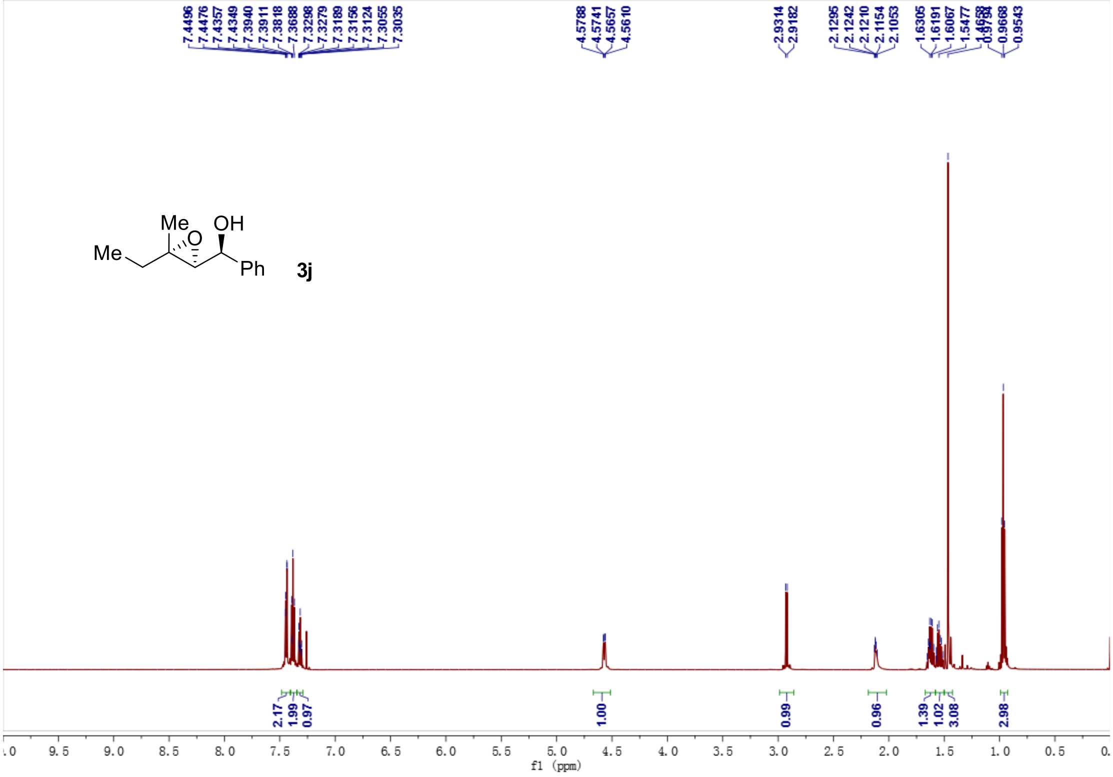

Supplementary Figure 185. ^{13}C NMR sepctra of (*S*)-((2*S*,3*S*)-3-ethyl-3-methyloxiran-2-yl)(phenyl)methanol (**3j**)

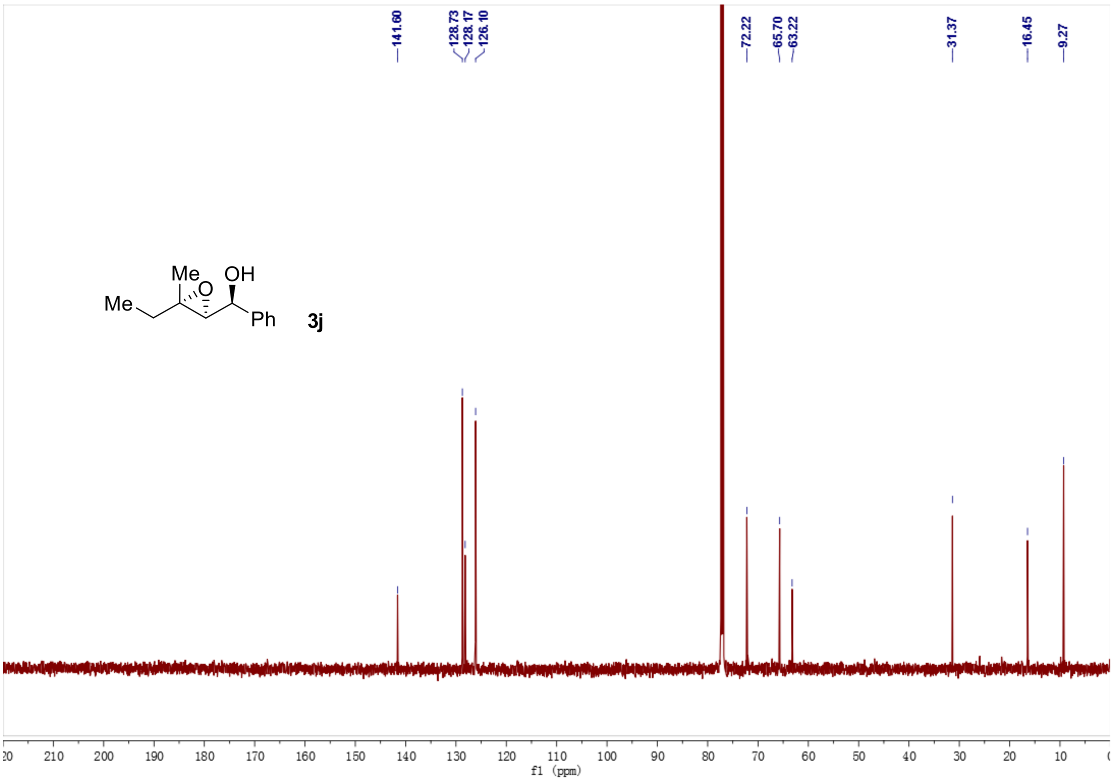

Supplementary Figure 186. ^{1}H NMR sepctra of (*R*)-((2*R*,3*R*)-3-ethyl-3-methyloxiran-2-yl)(phenyl)methyl propionate (**4j**)

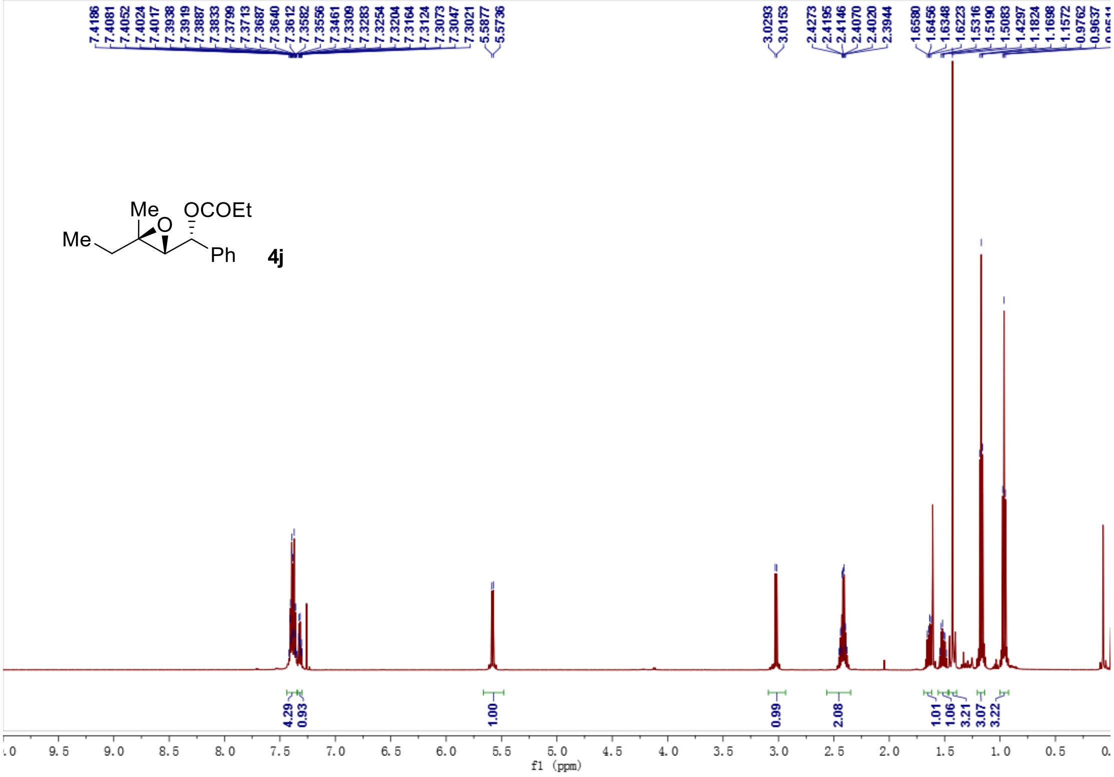

Supplementary Figure 187. ^{13}C NMR sepctra of (*R*)-((2*R*,3*R*)-3-ethyl-3-methyloxiran-2-yl)(phenyl)methyl propionate (**4j**)

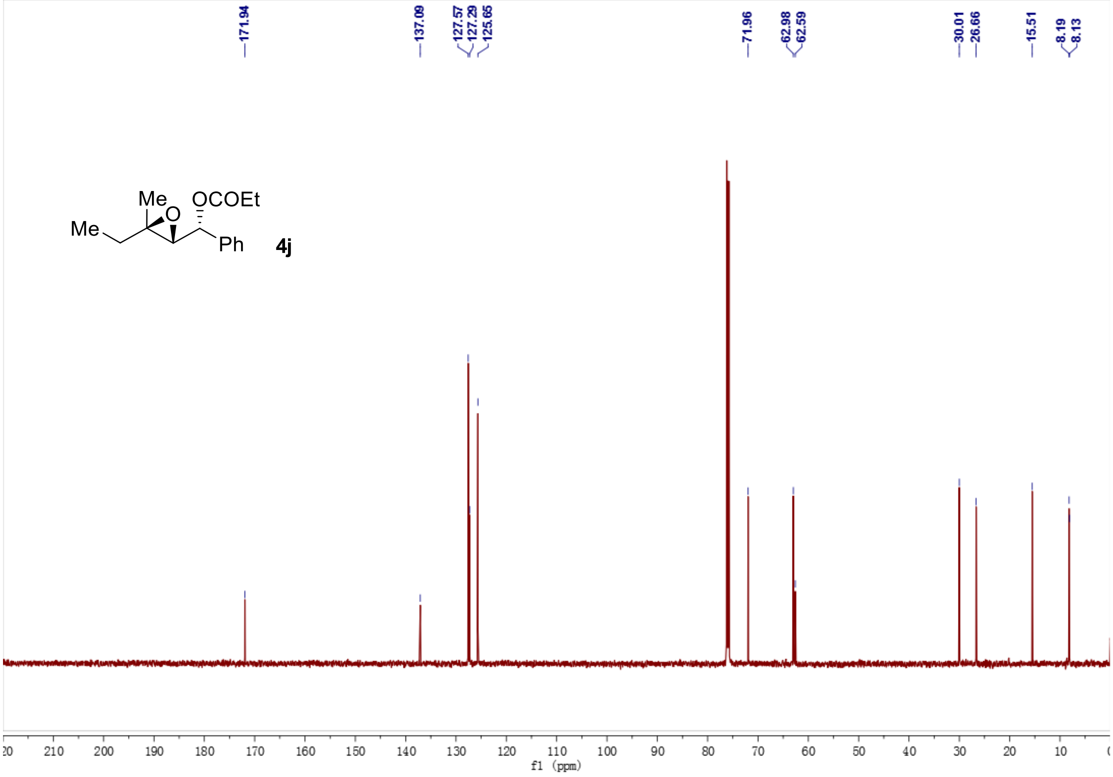

Supplementary Figure 188. ^{1}H NMR sepctra of (*S*)-((2*S*,3*R*)-3-ethyl-3-methyloxiran-2-yl)(phenyl)methanol (**3k**)

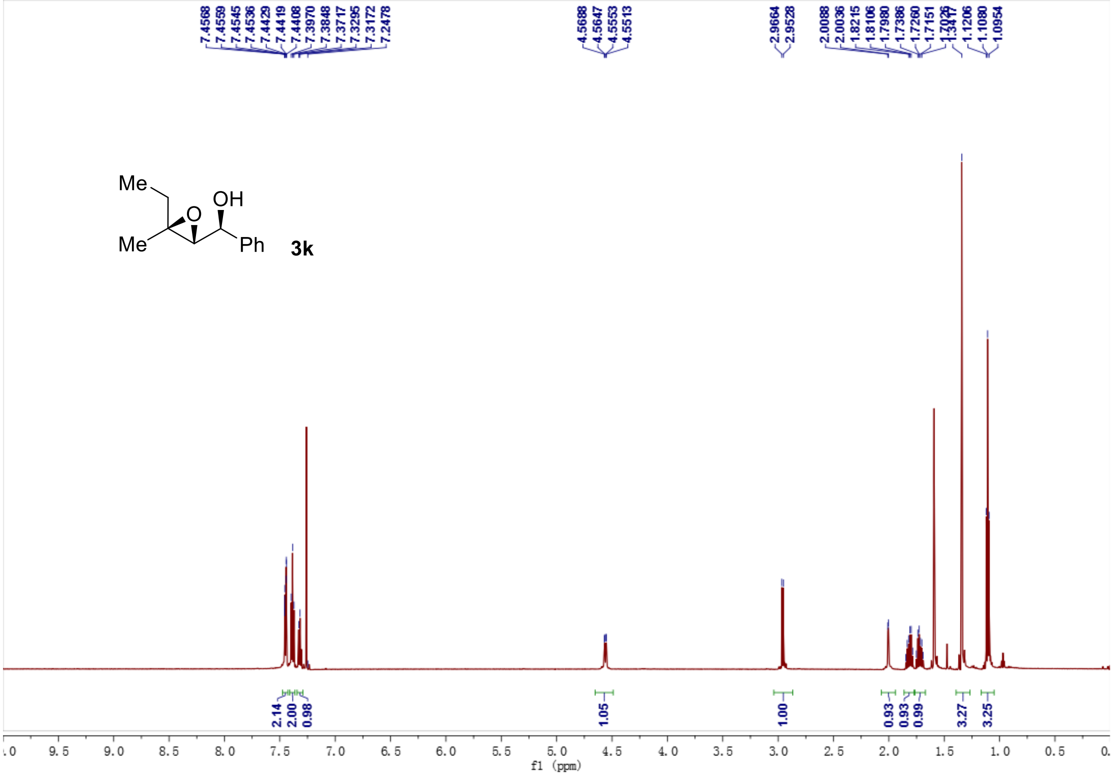

Supplementary Figure 189. ^{13}C NMR sepctra of (*S*)-((2*S*,3*R*)-3-ethyl-3-methyloxiran-2-yl)(phenyl)methanol (**3k**)

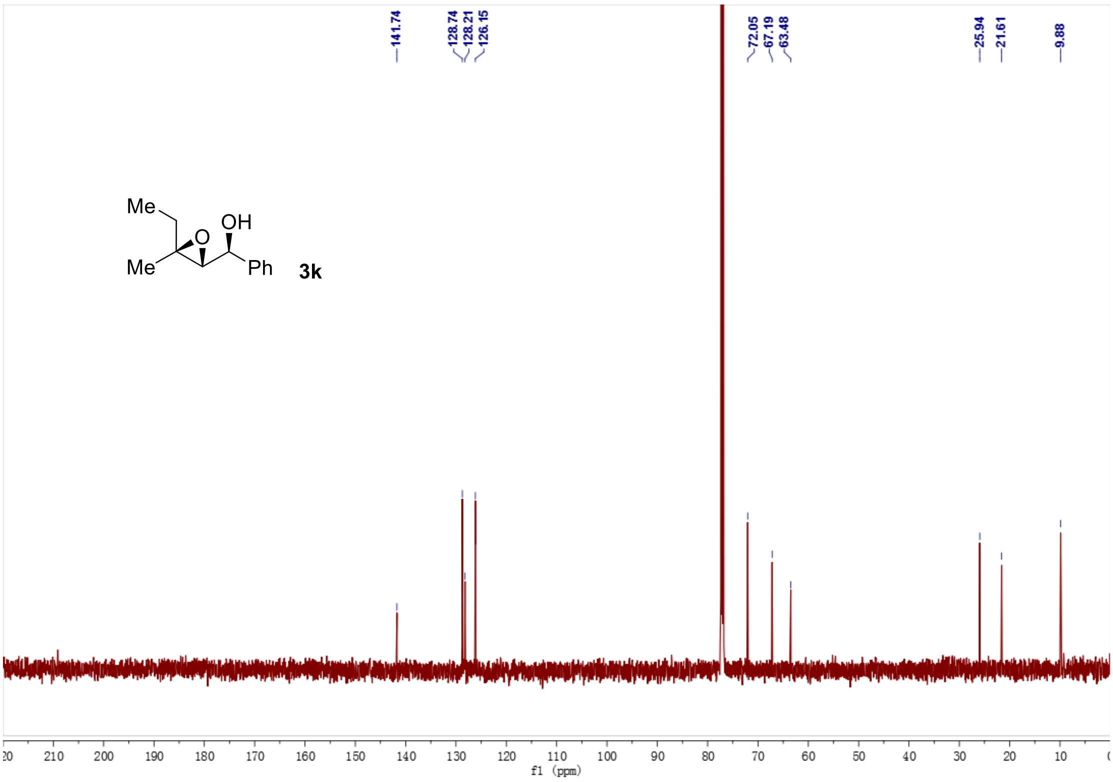

Supplementary Figure 190. ^{1}H NMR sepctra of (*R*)-((2*R*,3*S*)-3-ethyl-3-methyloxiran-2-yl)(phenyl)methyl propionate (**4k**)

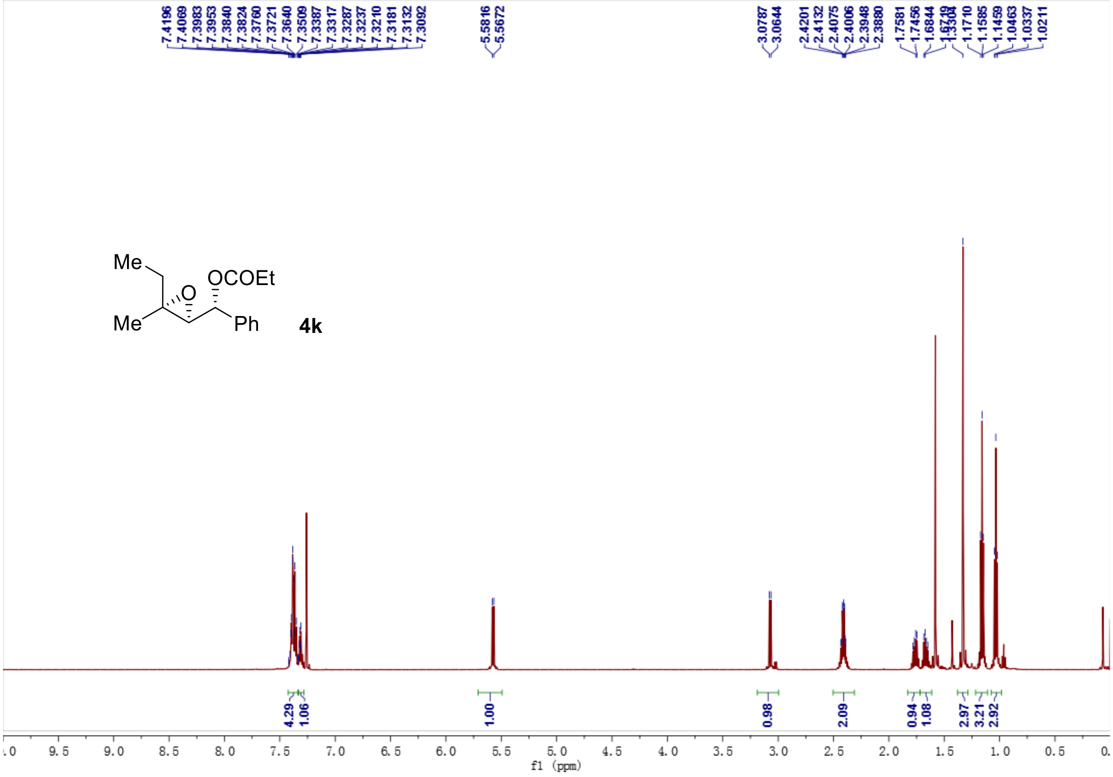

Supplementary Figure 191. ^{13}C NMR sepctra of (*R*)-((2*R*,3*S*)-3-ethyl-3-methyloxiran-2-yl)(phenyl)methyl propionate (**4k**)

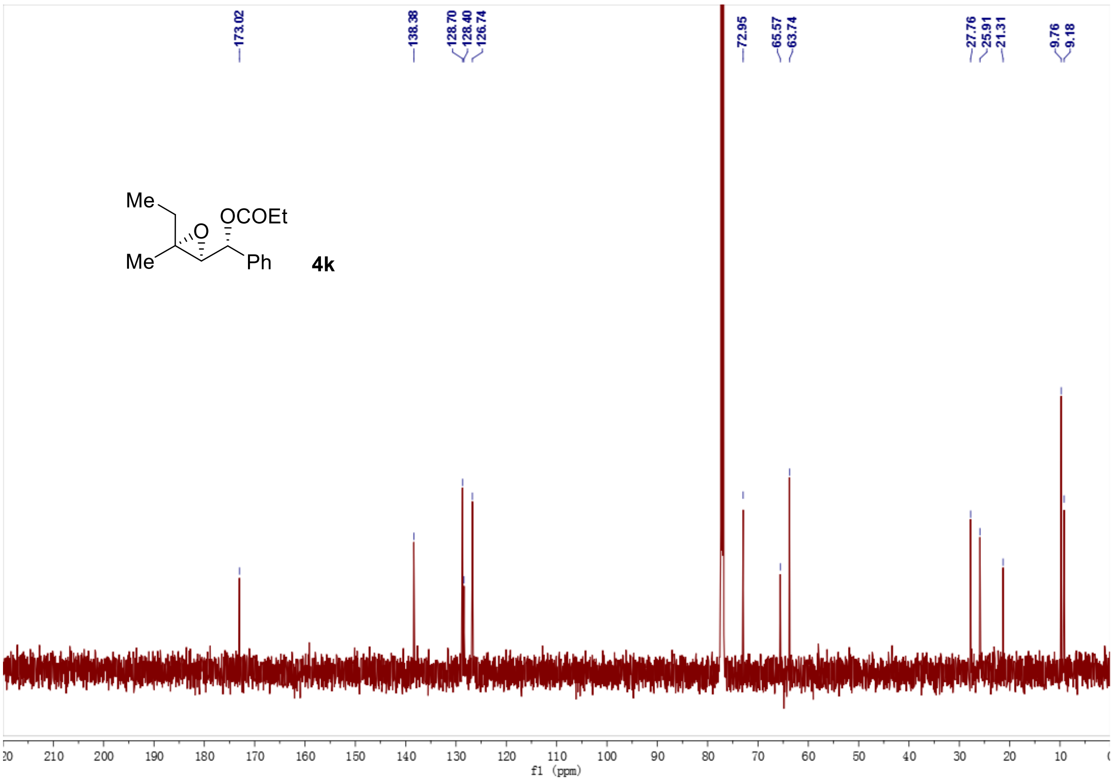

Supplementary Figure 192. ^{1}H NMR sepctra of Ethyl (*R*)-2-((*S*)-hydroxy(phenyl)methyl)-2-methylpent-4-enoate (**5a**)

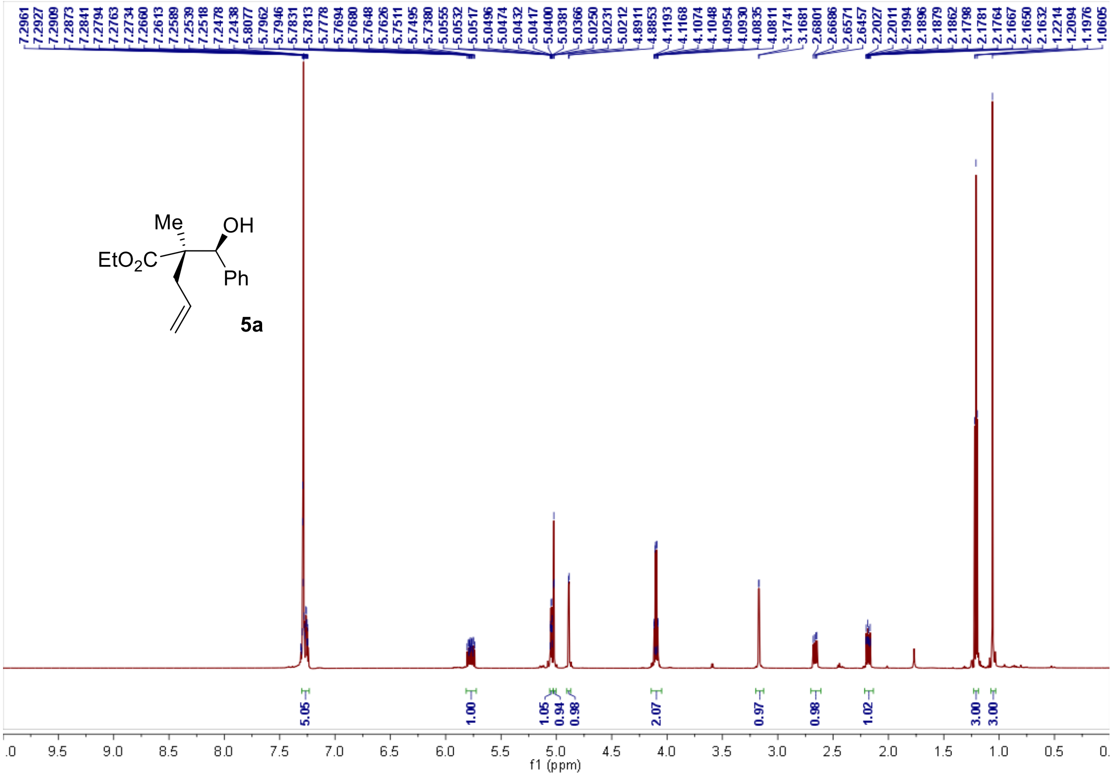

Supplementary Figure 193. ^{13}C NMR sepctra of Ethyl (*R*)-2-((*S*)-hydroxy(phenyl)methyl)-2-methylpent-4-enoate (**5a**)

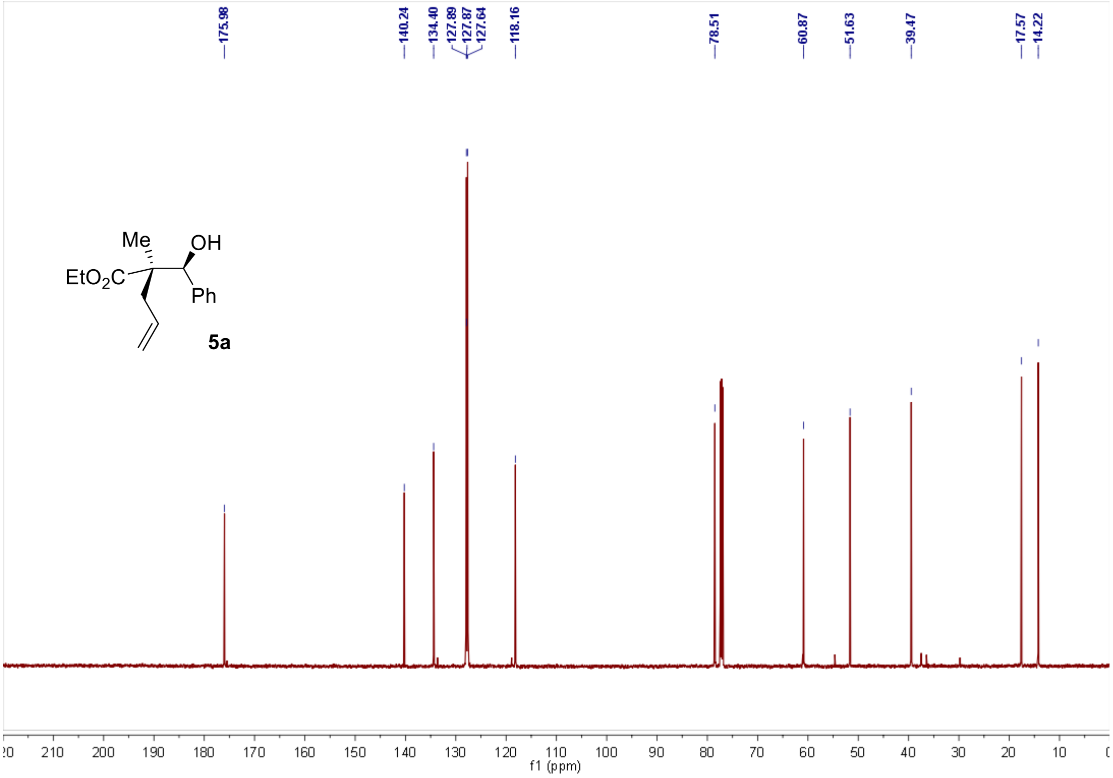

Supplementary Figure 194. ^{1}H NMR sepctra of Ethyl (*S*)-2-methyl-2-((*R*)-phenyl(propionyloxy)methyl)pent-4-enoate (**6a**)

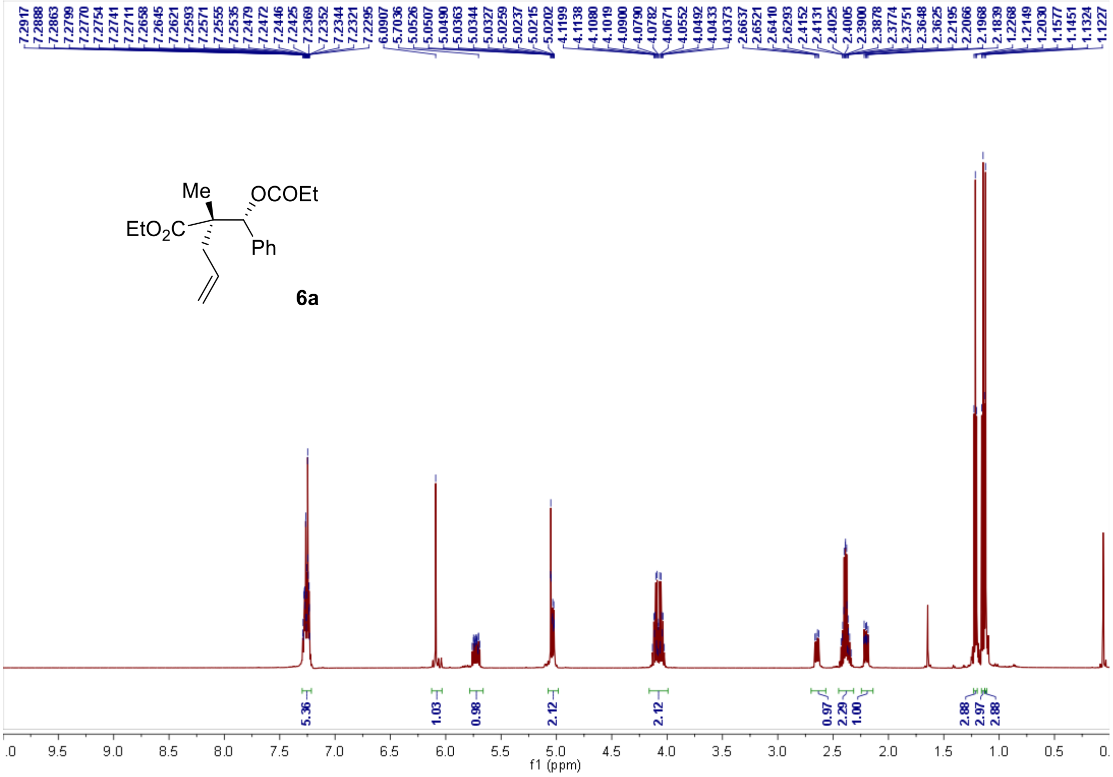

Supplementary Figure 195. ^{13}C NMR sepctra of Ethyl (*S*)-2-methyl-2-((*R*)-phenyl(propionyloxy)methyl)pent-4-enoate (**6a**)

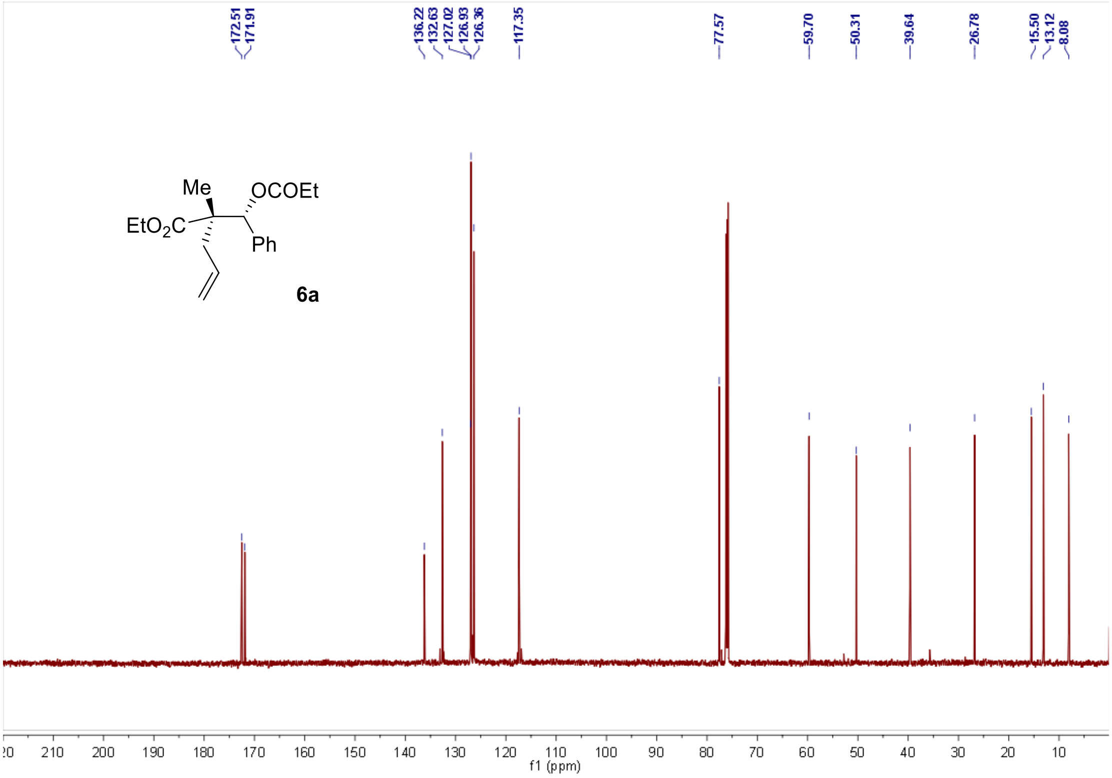

Supplementary Figure 196. ^{1}H NMR sepctra of Ethyl (*S*)-2-((*S*)-hydroxy(phenyl)methyl)-2-methylpent-4-enoate (**5b**)

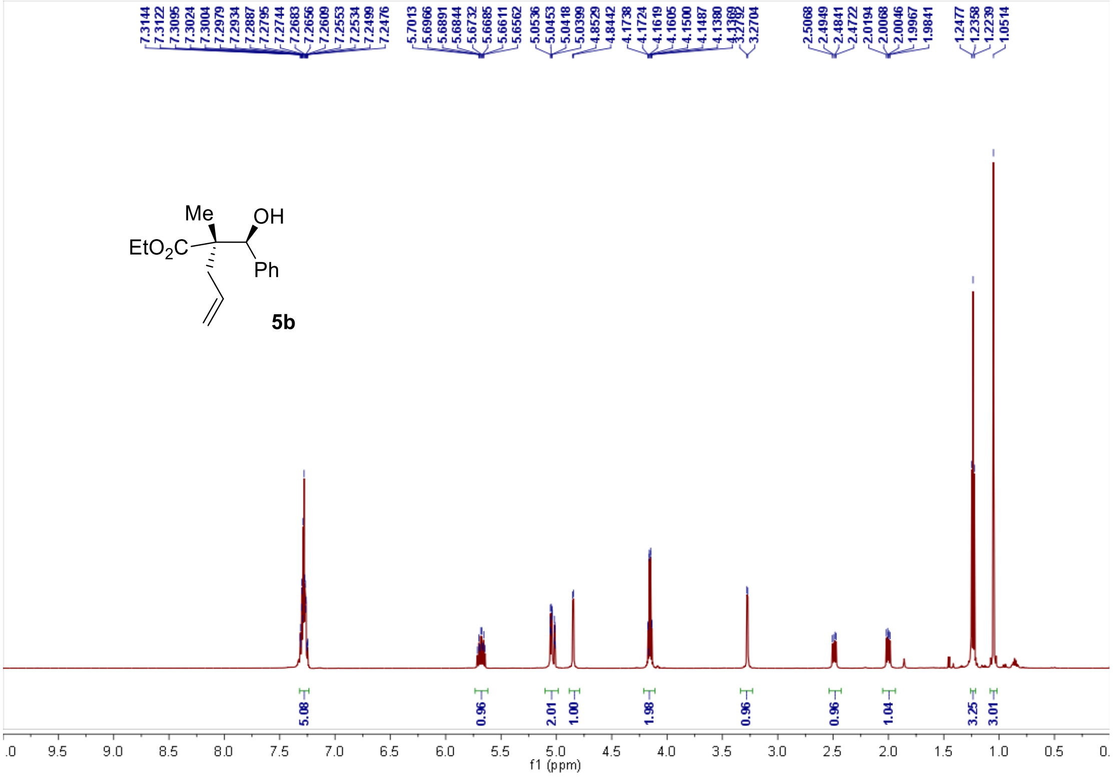

Supplementary Figure 197. ^{13}C NMR sepctra of Ethyl (*S*)-2-((*S*)-hydroxy(phenyl)methyl)-2-methylpent-4-enoate (**5b**)

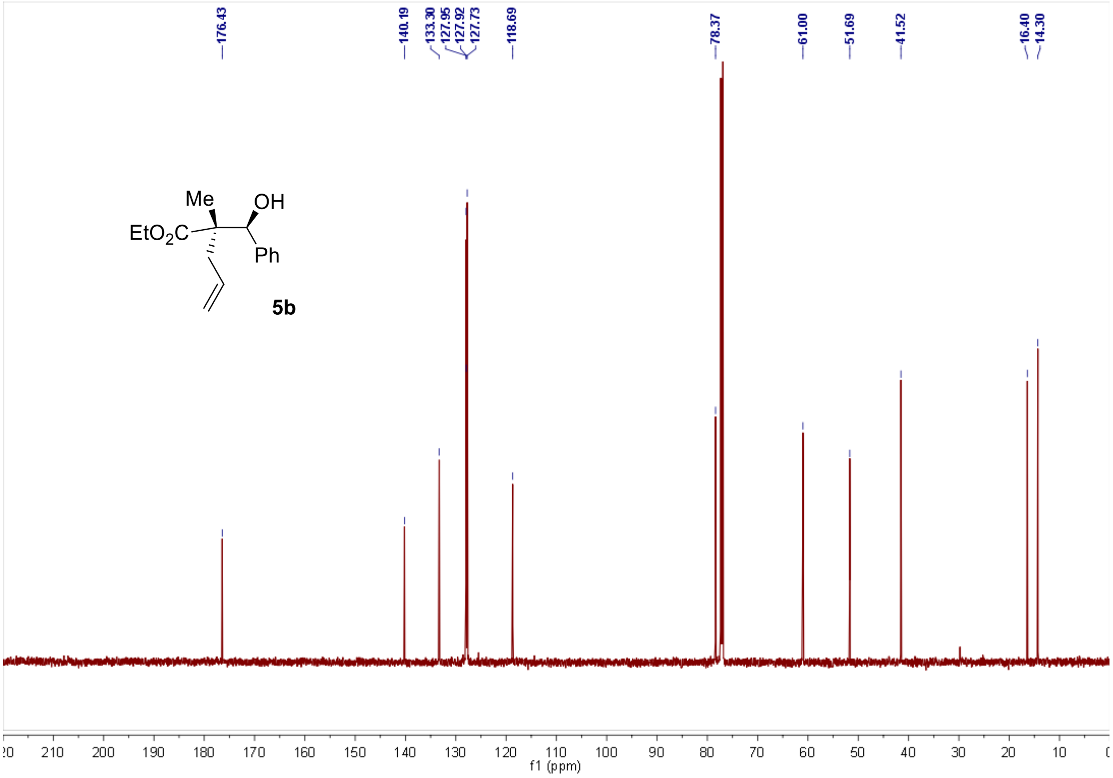

Supplementary Figure 198. ^{1}H NMR sepctra of Ethyl (*R*)-2-methyl-2-((*R*)-phenyl(propionyloxy)methyl)pent-4-enoate (**6b**)

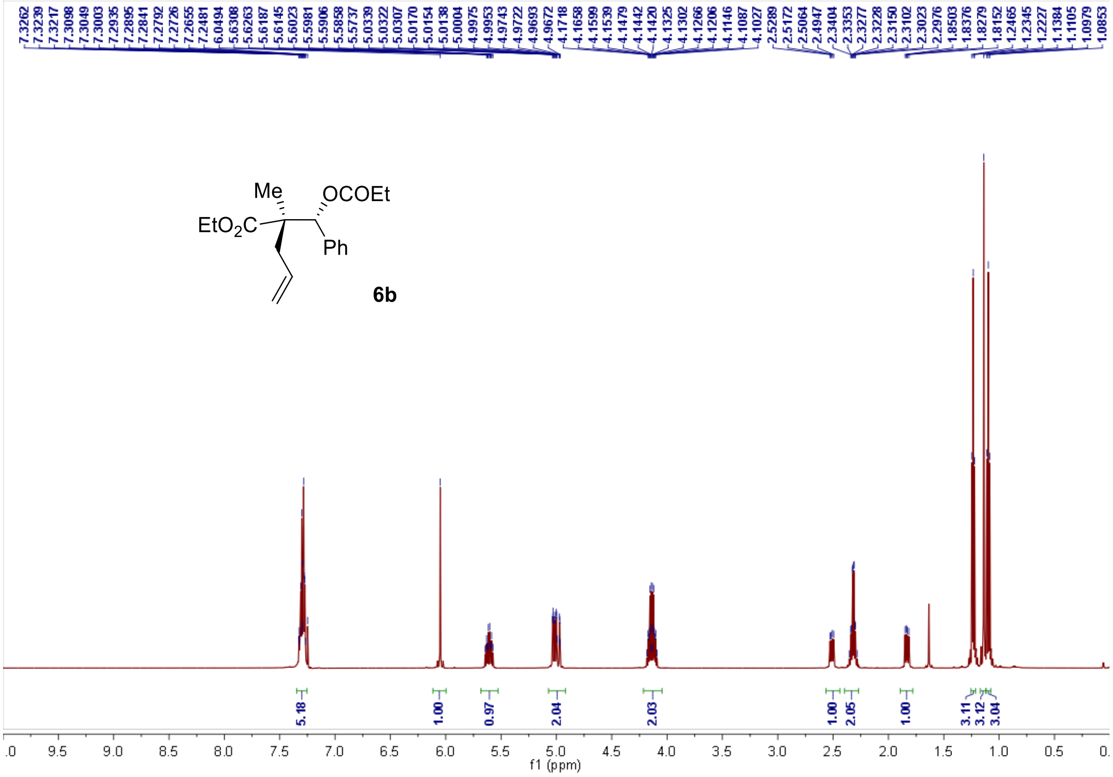

Supplementary Figure 199. ^{13}C NMR sepctra of Ethyl (*R*)-2-methyl-2-((*R*)-phenyl(propionyloxy)methyl)pent-4-enoate (**6b**)

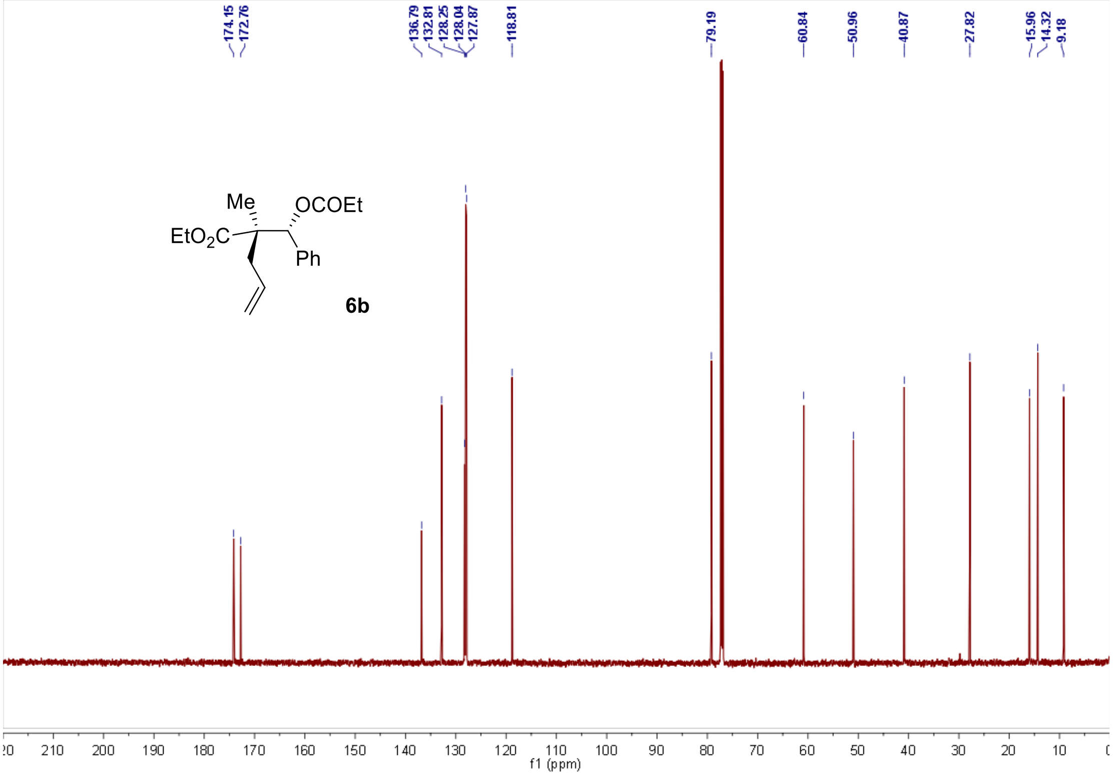

Supplementary Figure 200. ^{1}H NMR sepctra of Methyl (*R*)-2-((*S*)-hydroxy(phenyl)methyl)-2-phenylpent-4-enoate (**5c**)

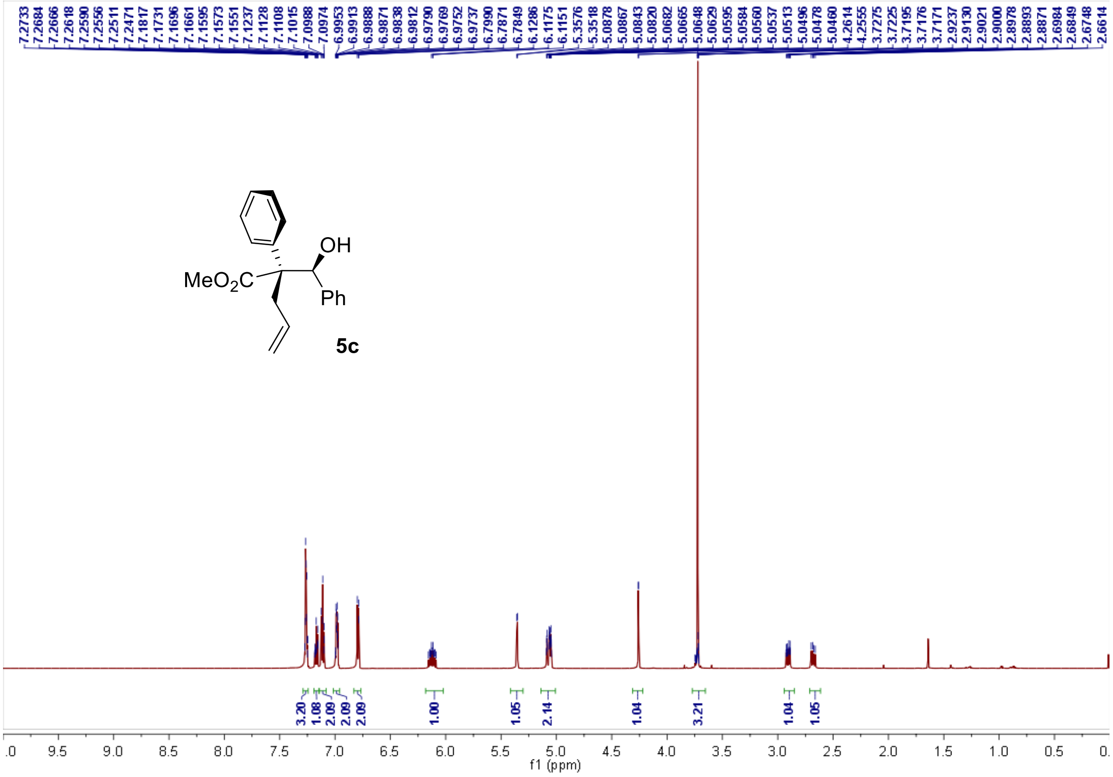

Supplementary Figure 201. ^{13}C NMR sepctra of Methyl (*R*)-2-((*S*)-hydroxy(phenyl)methyl)-2-phenylpent-4-enoate (**5c**)

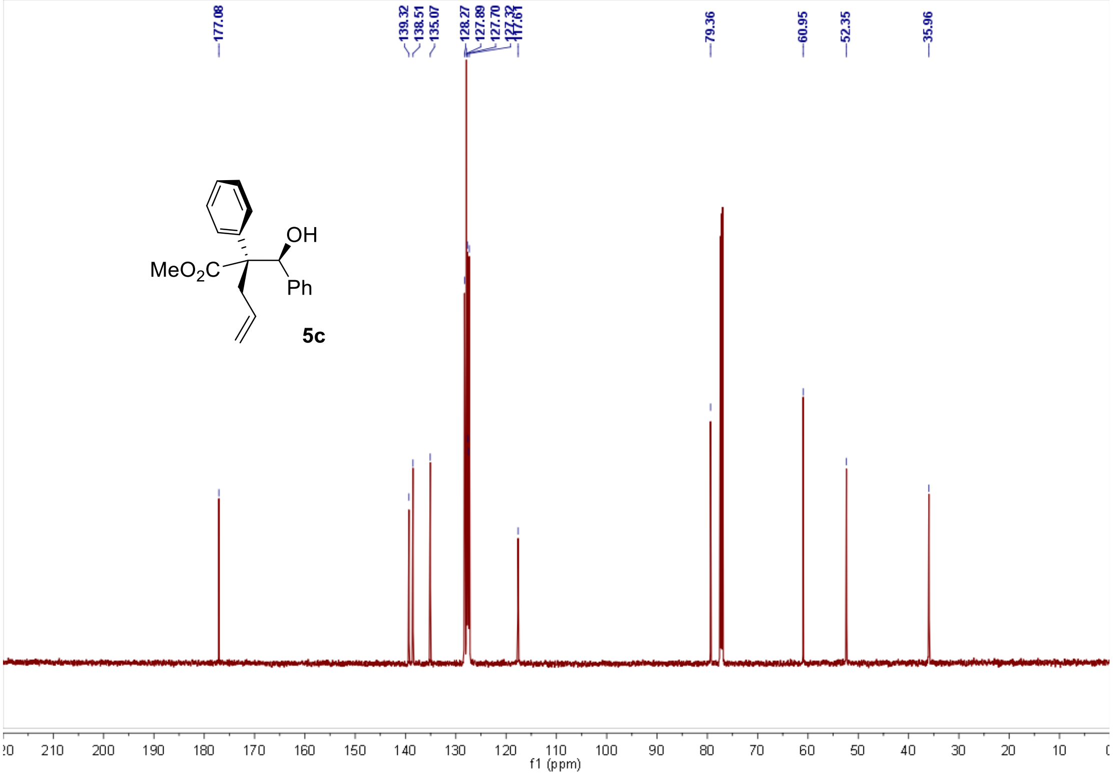

Supplementary Figure 202. ^{1}H NMR sepctra of Methyl (*S*)-2-phenyl-2-((*R*)-phenyl(propionyloxy)methyl)pent-4-enoate (**6c**)

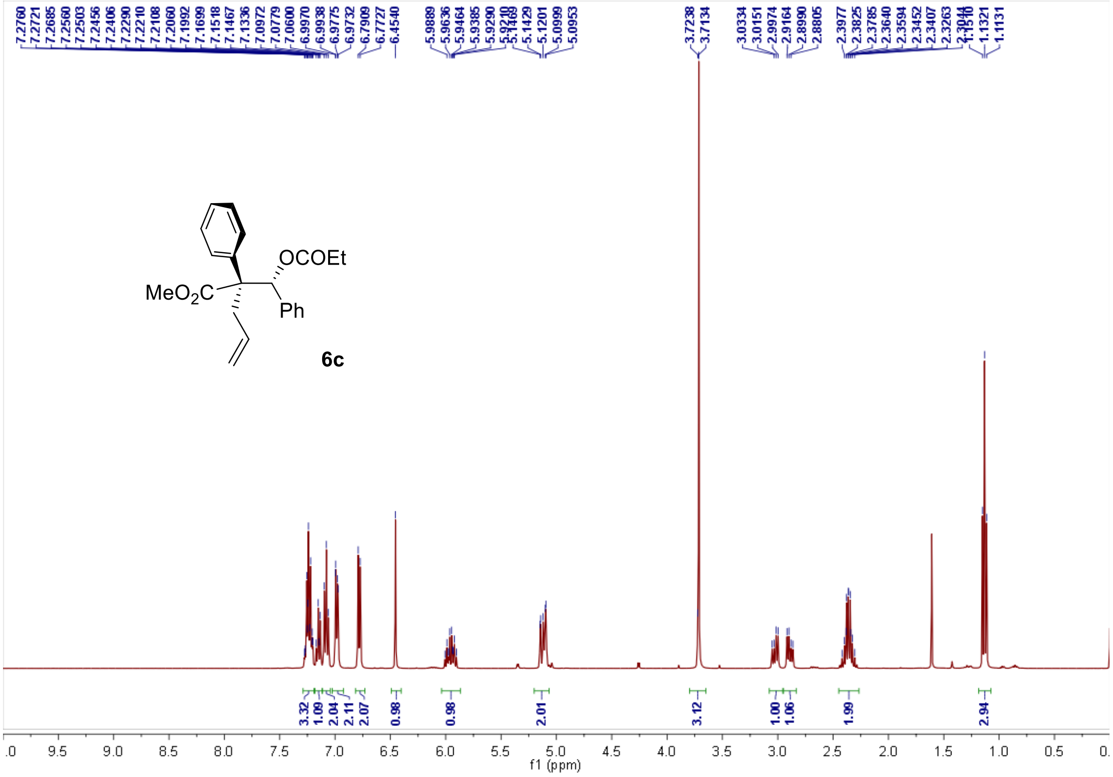

Supplementary Figure 203. ^{13}C NMR sepctra of Methyl (*S*)-2-phenyl-2-((*R*)-phenyl(propionyloxy)methyl)pent-4-enoate (**6c**)

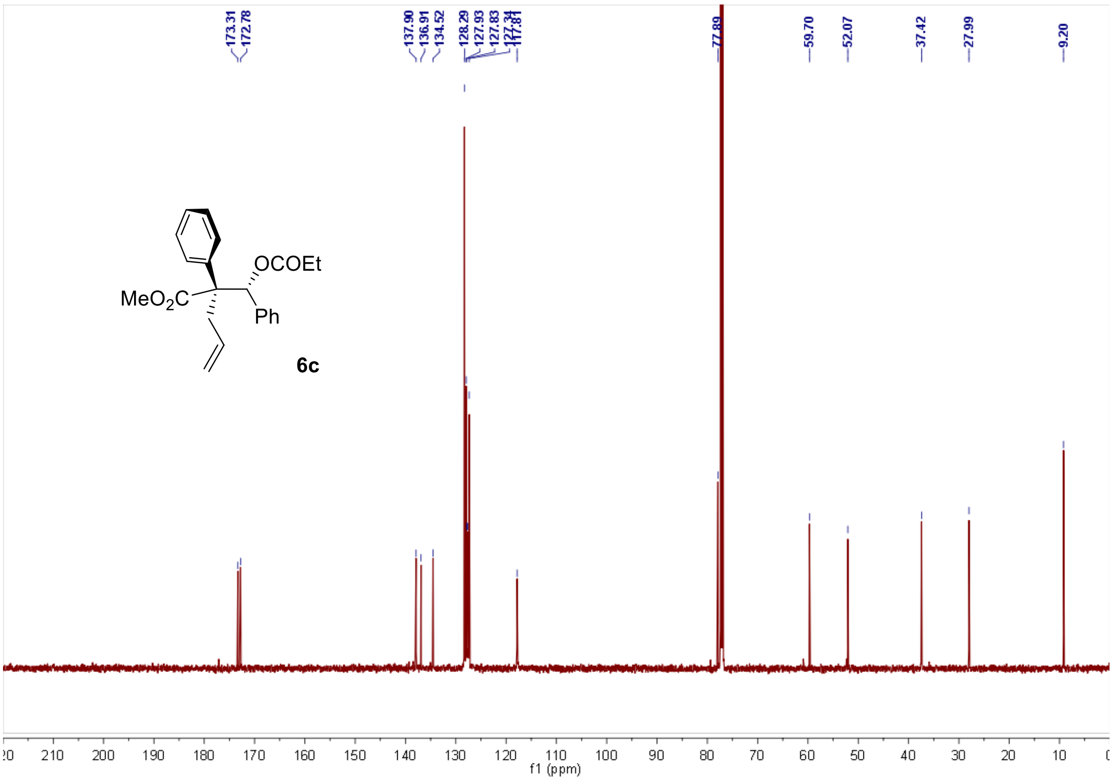

Supplementary Figure 204. ^{1}H NMR sepctra of Ethyl-(*R*)-2-((1-benzyl-1*H*-1,2,3-triazol-5-yl)methyl)-2-((*S*)-hydroxy(phenyl)methyl)pent-4-enoate (**5d**)

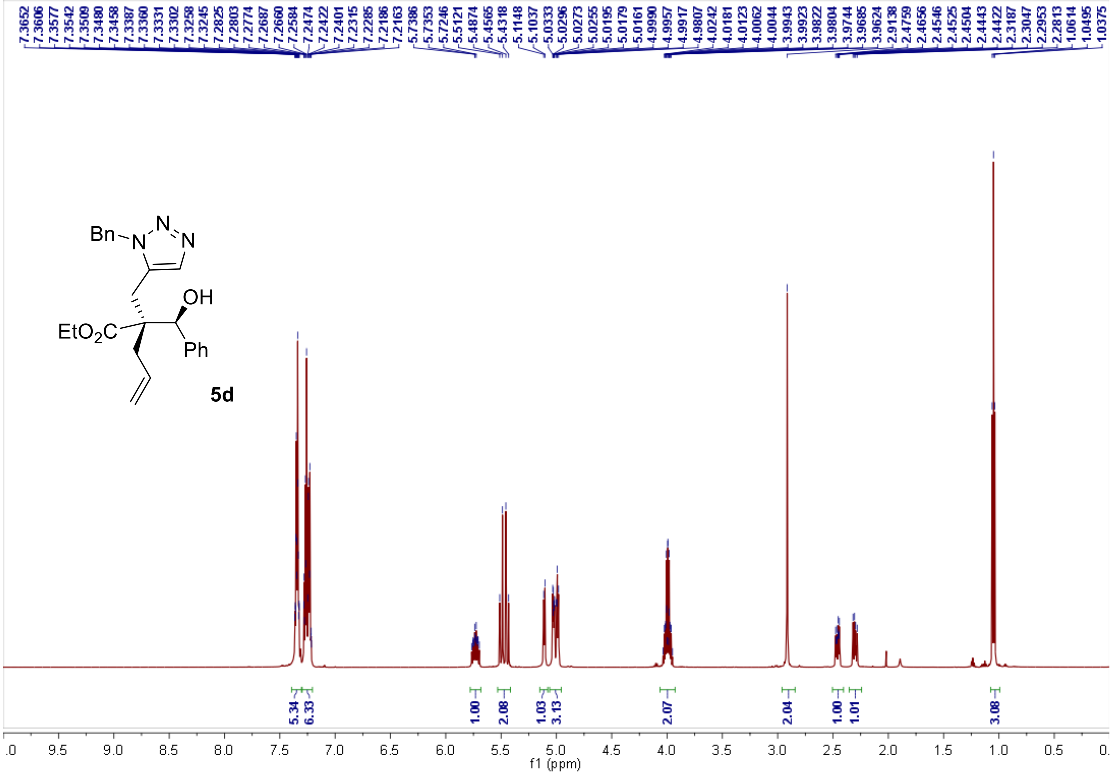

Supplementary Figure 205. ^{13}C NMR sepctra of Ethyl-(*R*)-2-((1-benzyl-1*H*-1,2,3-triazol-5-yl)methyl)-2-((*S*)-hydroxy(phenyl)methyl)pent-4-enoate (**5d**)

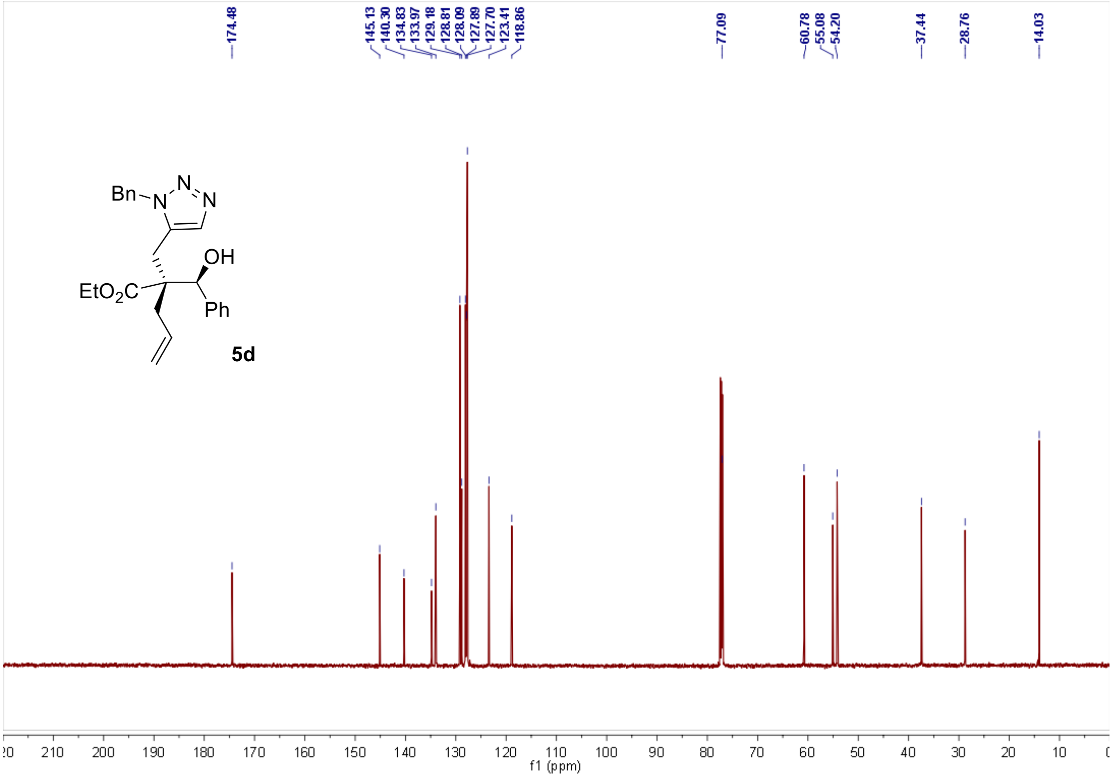

Supplementary Figure 206. ^{1}H NMR sepctra of Ethyl-(*S*)-2-((1-benzyl-1*H*-1,2,3-triazol-5-yl)methyl)-2-((*R*)-phenyl(propionyloxy)methyl)pent-4-enoate (**6d**)

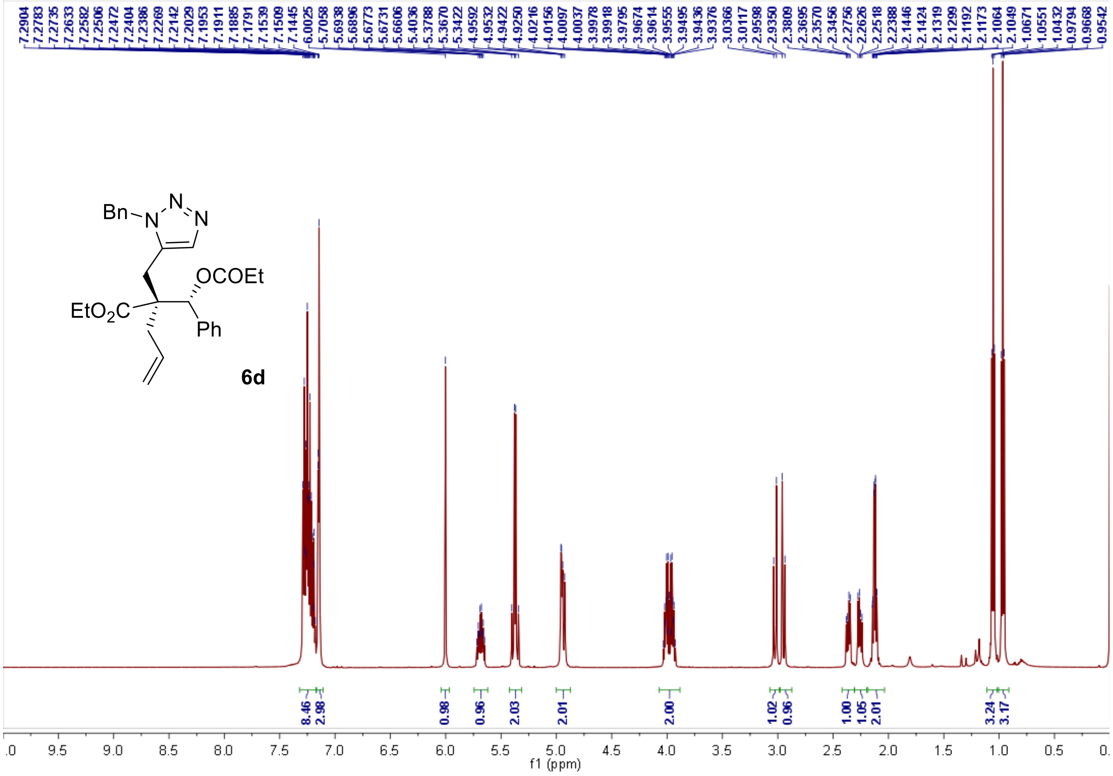

Supplementary Figure 207. ^{13}C NMR sepctra of Ethyl-(*S*)-2-((1-benzyl-1*H*-1,2,3-triazol-5-yl)methyl)-2-((*R*)-phenyl(propionyloxy)methyl)pent-4-enoate (**6d**)

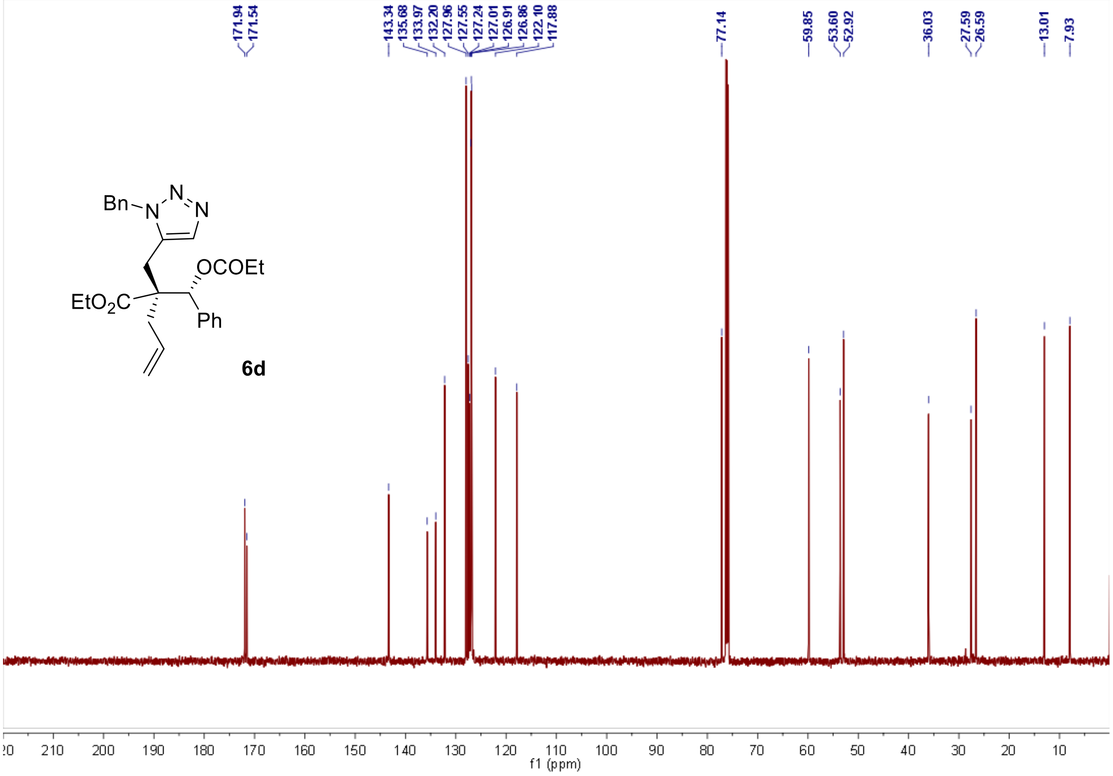

Supplementary Figure 208. ^{1}H NMR sepctra of Methyl (*R*)-2-((*S*)-(4-chlorophenyl)(hydroxy)methyl)-2-(naphthalen-2-ylmethyl)pent-4-enoate (**5e**)

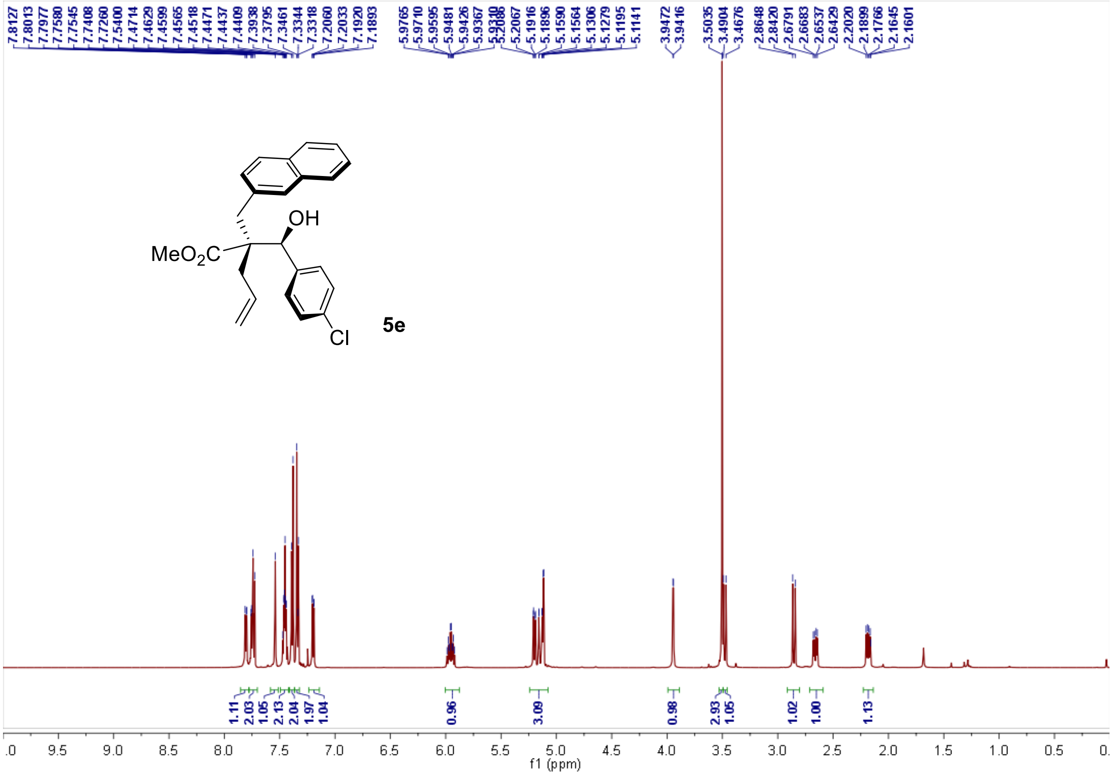

Supplementary Figure 209. ^{13}C NMR sepctra of Methyl (*R*)-2-((*S*)-(4-chlorophenyl)(hydroxy)methyl)-2-(naphthalen-2-ylmethyl)pent-4-enoate (**5e**)

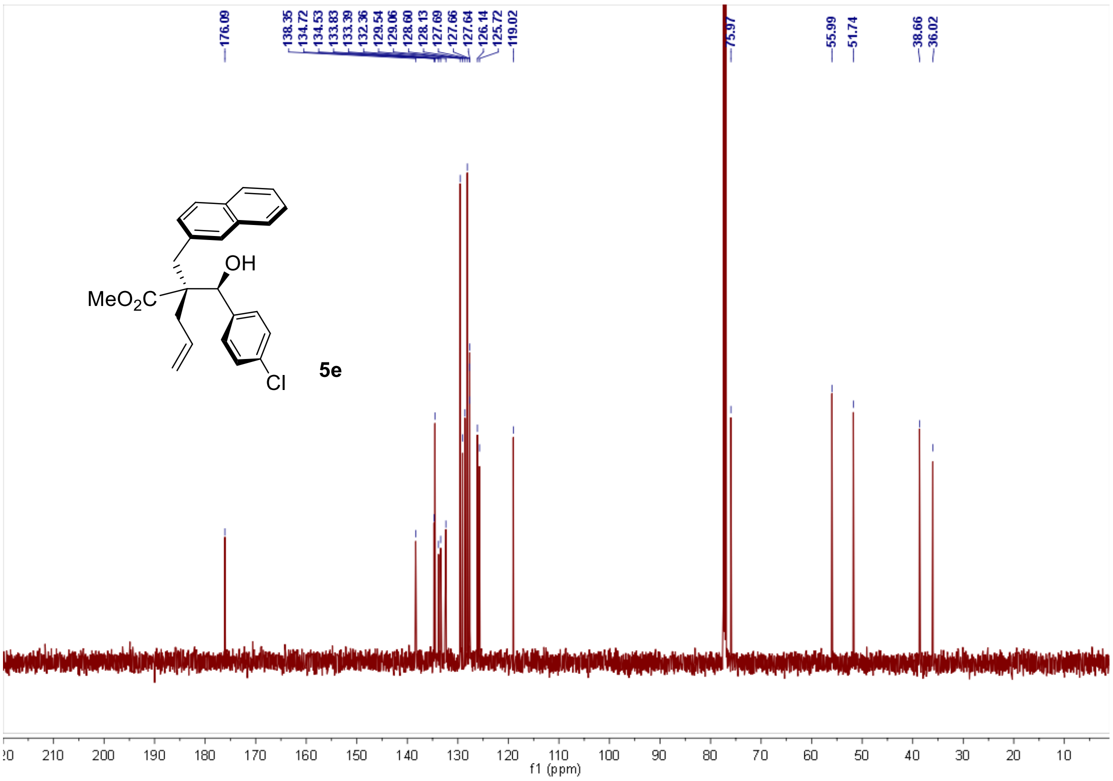

Supplementary Figure 210. ^{1}H NMR sepctra of Methyl (*S*)-2-((*R*)-(4-chlorophenyl)(propionyloxy)methyl)-2-(naphthalen-2-ylmethyl)pent-4-enoate (**6e**)

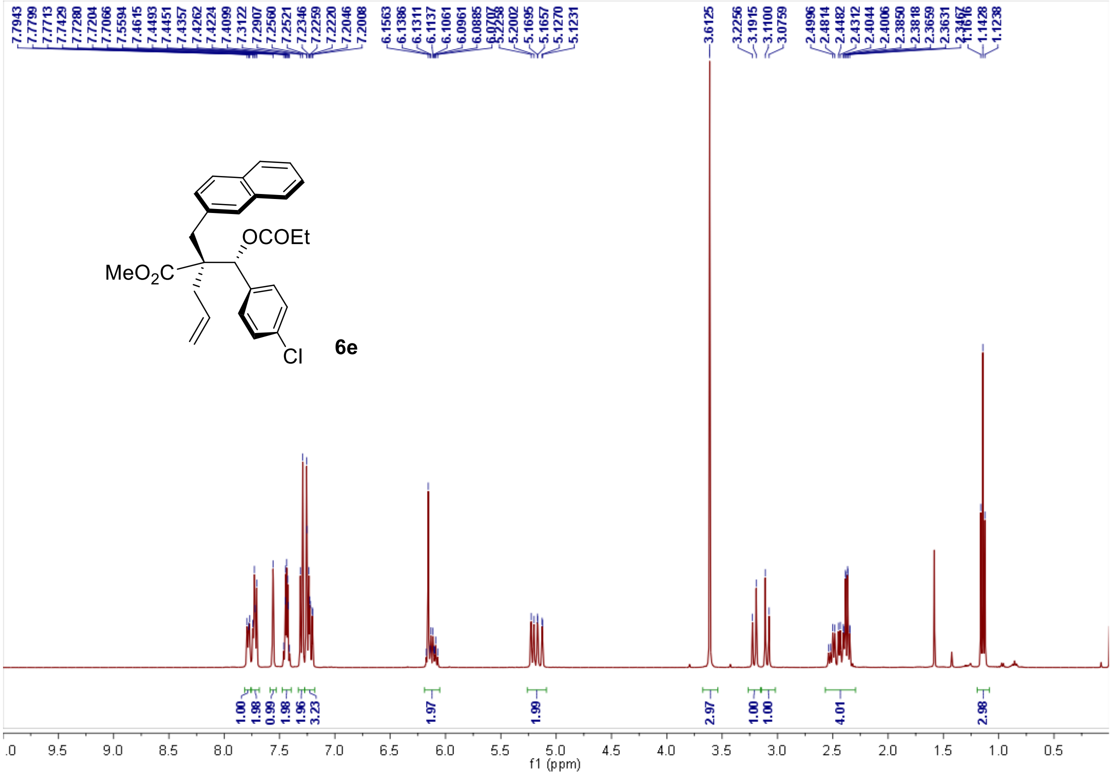

Supplementary Figure 211. ^{13}C NMR sepctra of Methyl (*S*)-2-((*R*)-(4-chlorophenyl)(propionyloxy)methyl)-2-(naphthalen-2-ylmethyl)pent-4-enoate (**6e**)

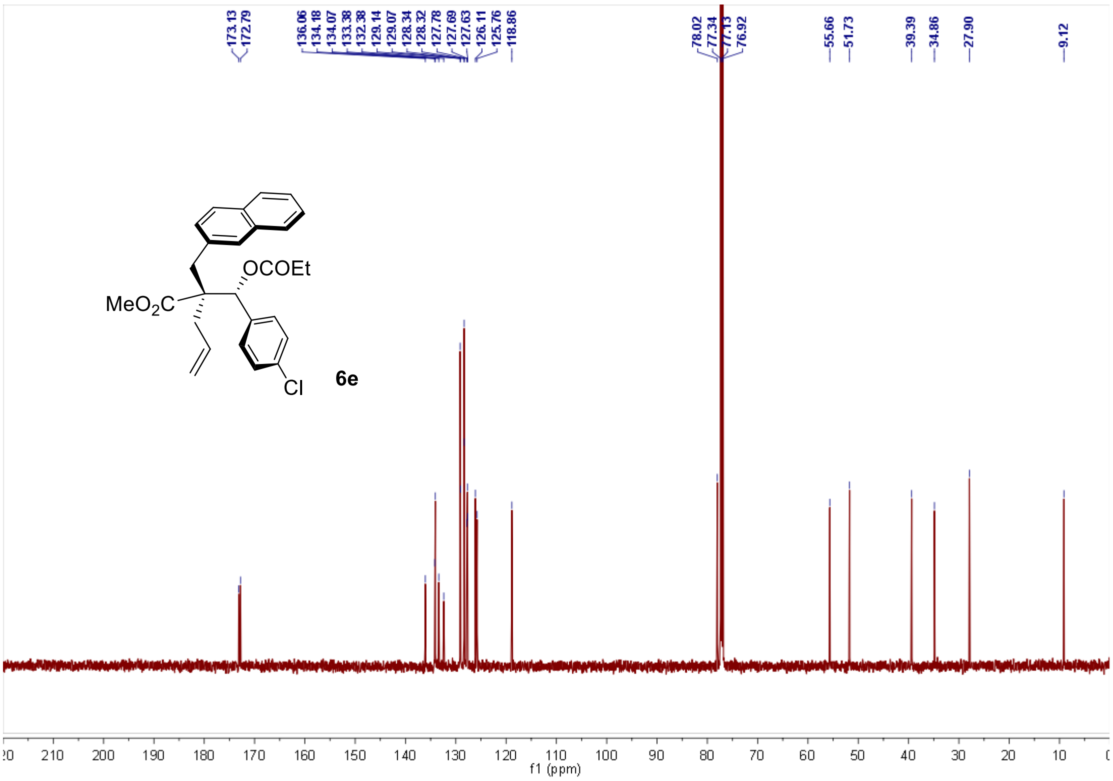

Supplementary Figure 212. ^{1}H NMR sepctra of Ethyl (*R*)-2-allyl-2-((*S*)-hydroxy(4-methoxyphenyl)methyl)-4-methylpent-4-enoate (**5f**)

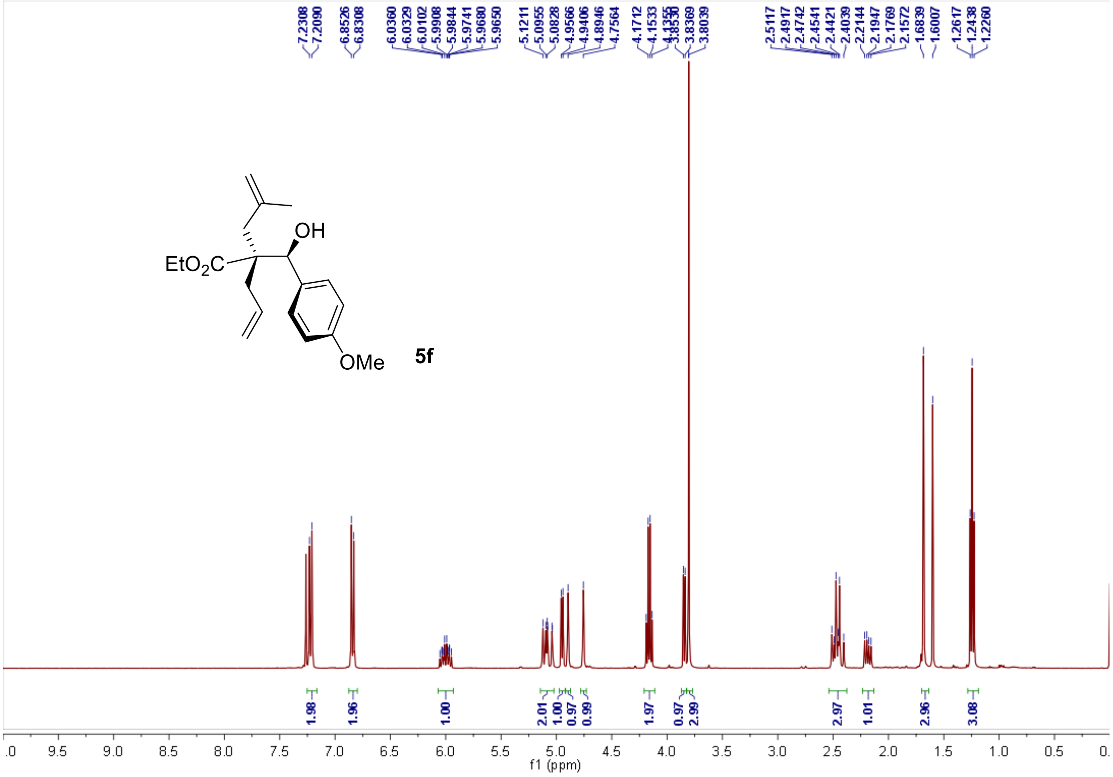

Supplementary Figure 213. ^{13}C NMR sepctra of Ethyl (*R*)-2-allyl-2-((*S*)-hydroxy(4-methoxyphenyl)methyl)-4-methylpent-4-enoate (**5f**)

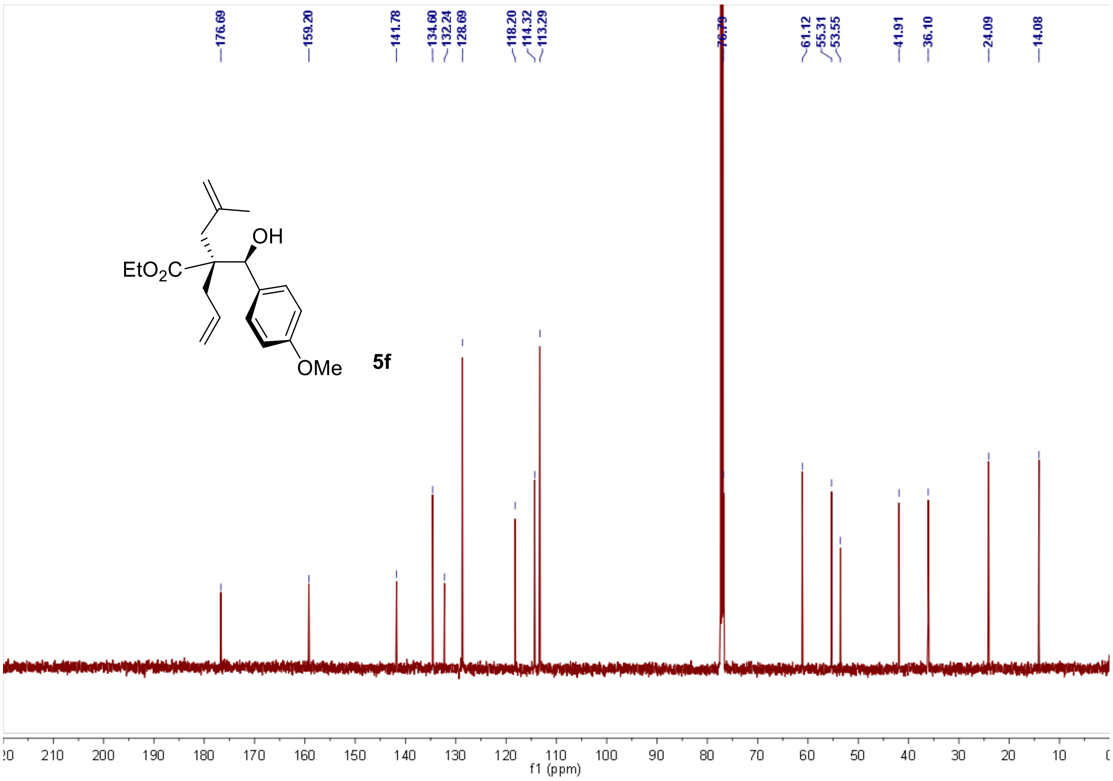

Supplementary Figure 214. ^{1}H NMR sepctra of Ethyl (*S*)-2-allyl-2-((*R*)-(4-methoxyphenyl)(propionyloxy)methyl)-4-methylpent-4-enoate **(6f)**

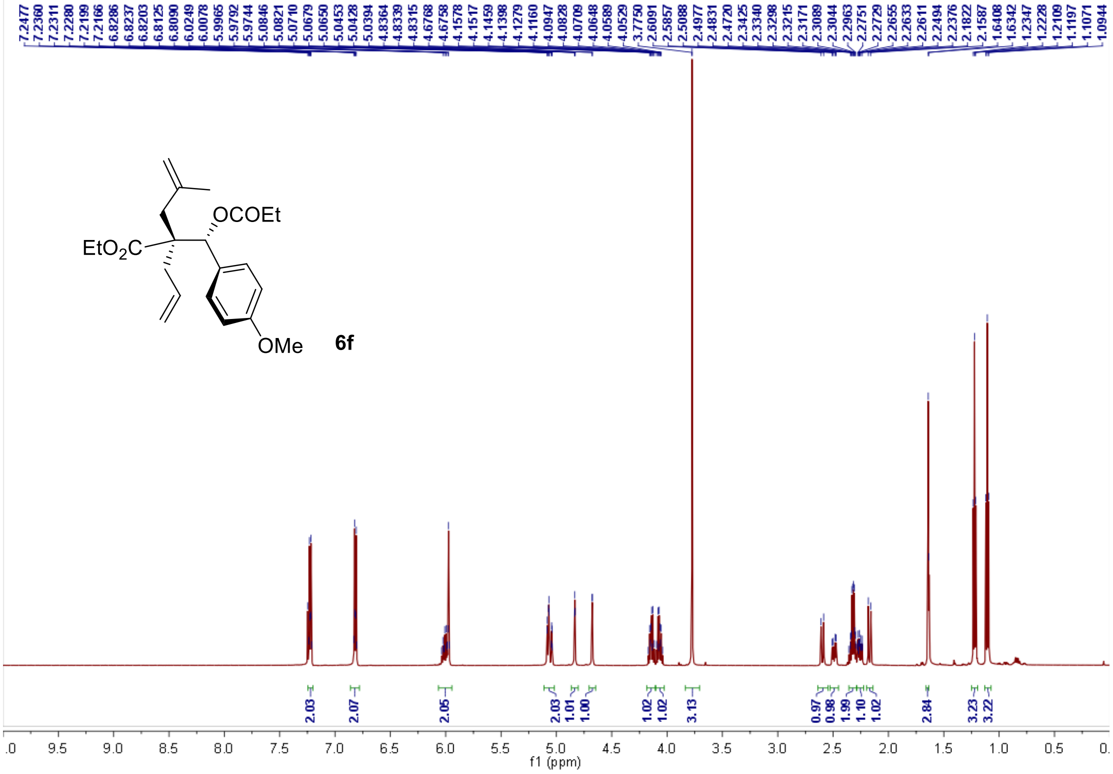

Supplementary Figure 215. ^{13}C NMR sepctra of Ethyl (*S*)-2-allyl-2-((*R*)-(4-methoxyphenyl)(propionyloxy)methyl)-4-methylpent-4-enoate **(6f)**

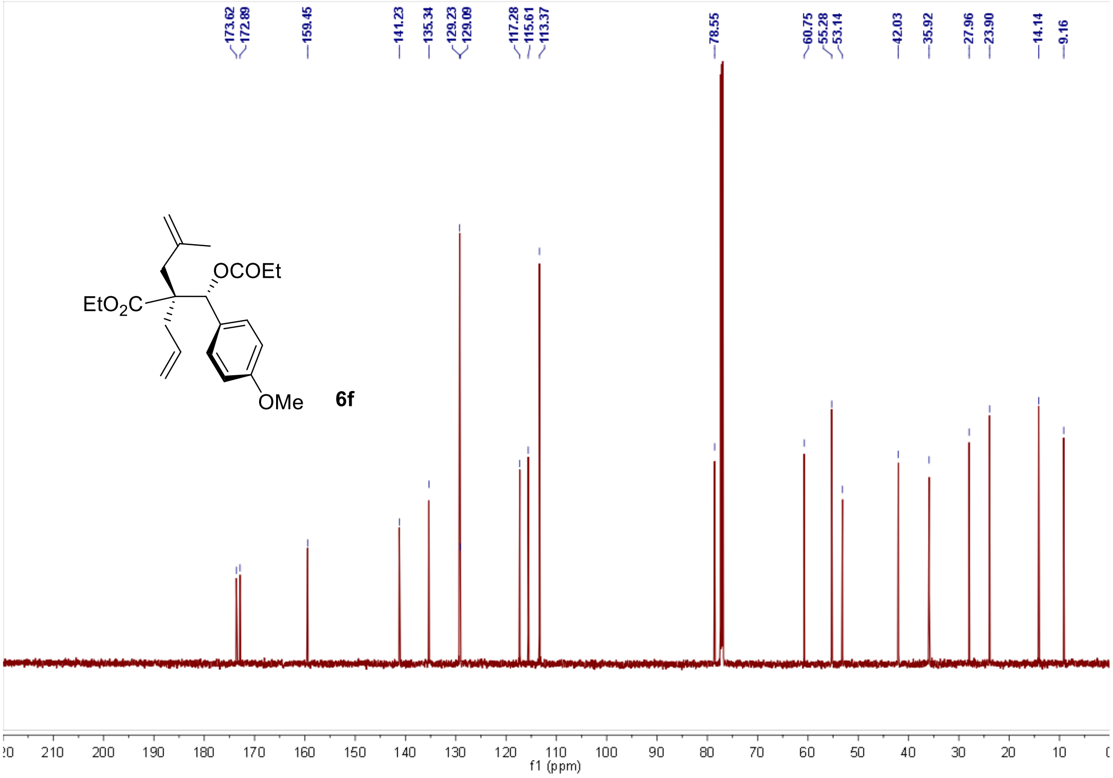

Supplementary Figure 216. ^{1}H NMR sepctra of Ethyl (*R*)-2-((*S*)-hydroxy(phenyl)methyl)-2-methylpent-4-ynoate (**5g**)

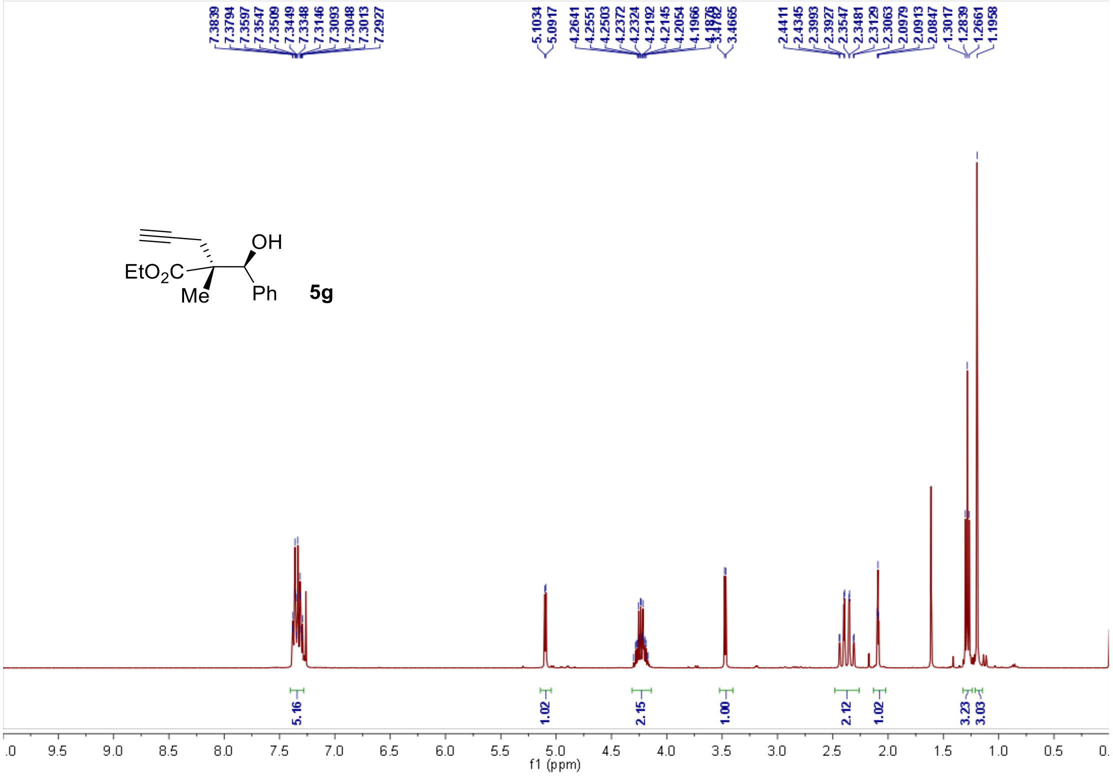

Supplementary Figure 217. ^{13}C NMR sepctra of Ethyl (*R*)-2-((*S*)-hydroxy(phenyl)methyl)-2-methylpent-4-ynoate (**5g**)

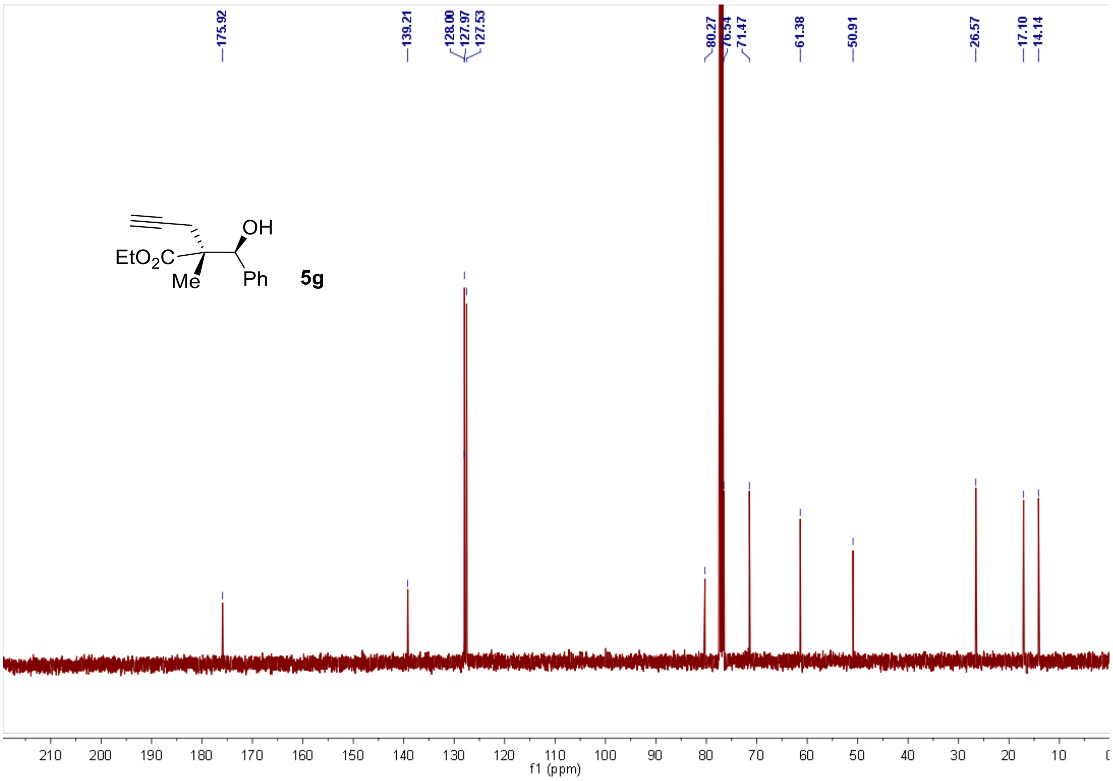

Supplementary Figure 218. ^{1}H NMR sepctra of Ethyl (*S*)-2-methyl-2-((*R*)-phenyl(propionyloxy)methyl)pent-4-ynoate (**6g**)

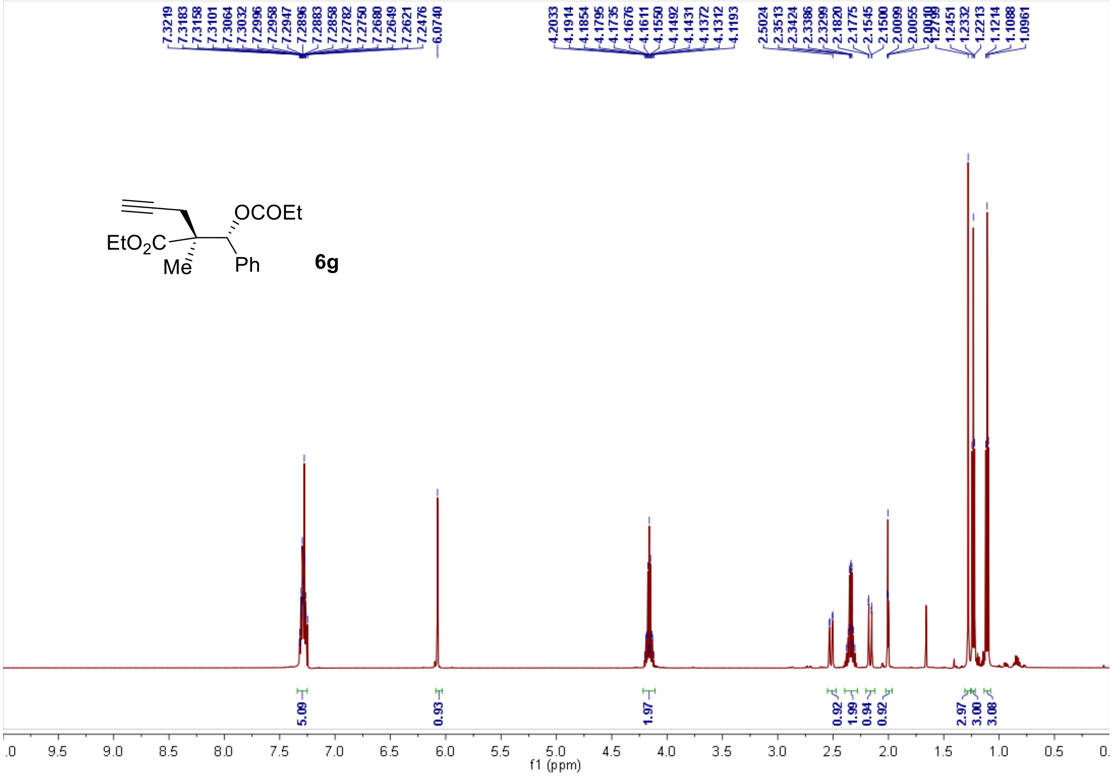

Supplementary Figure 219. ^{13}C NMR sepctra of Ethyl (*S*)-2-methyl-2-((*R*)-phenyl(propionyloxy)methyl)pent-4-ynoate (**6g**)

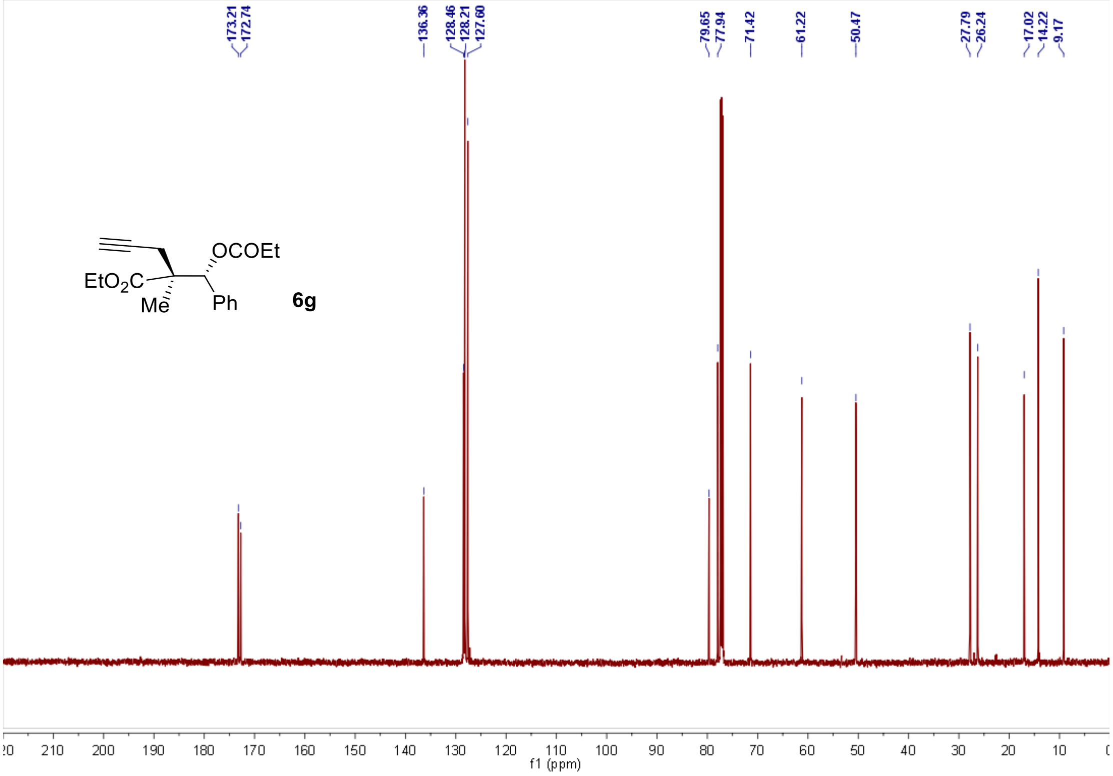

Supplementary Figure 220. ^{1}H NMR sepctra of 1-Ethyl 6-methyl (*R*)-2-((*S*)-hydroxy(phenyl)methyl)-2-methylhexanedioate (**5h**)

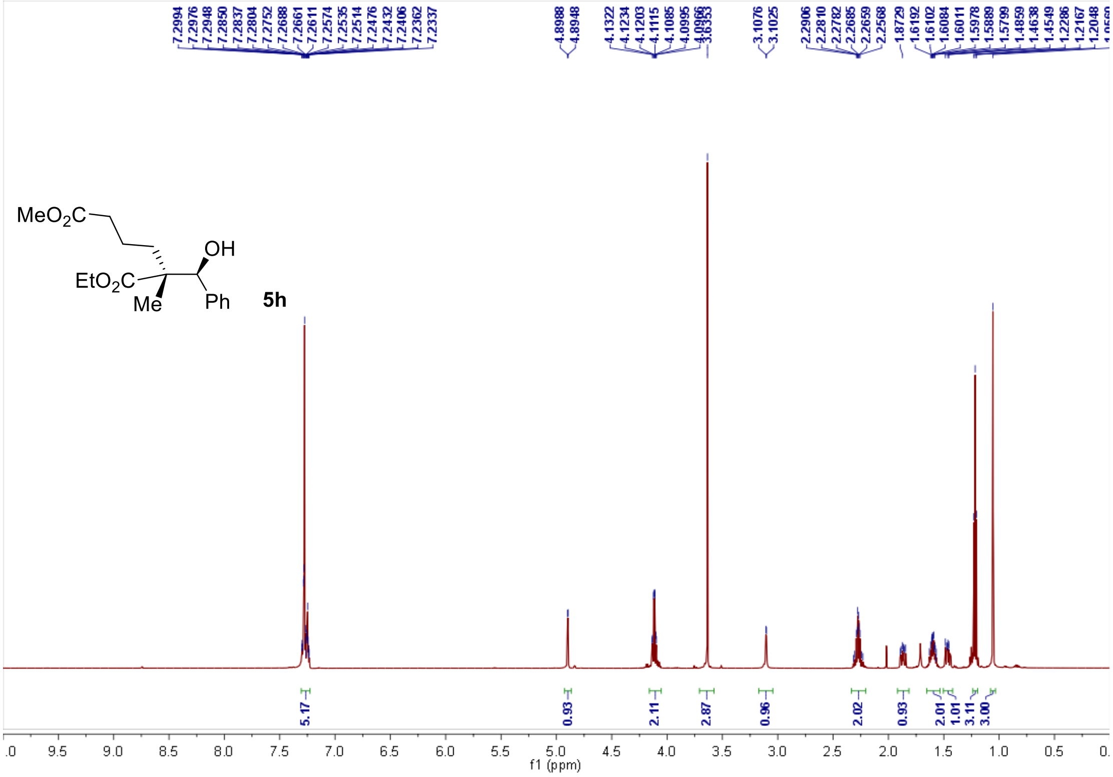

Supplementary Figure 221. ^{13}C NMR sepctra of 1-Ethyl 6-methyl (*R*)-2-((*S*)-hydroxy(phenyl)methyl)-2-methylhexanedioate (**5h**)

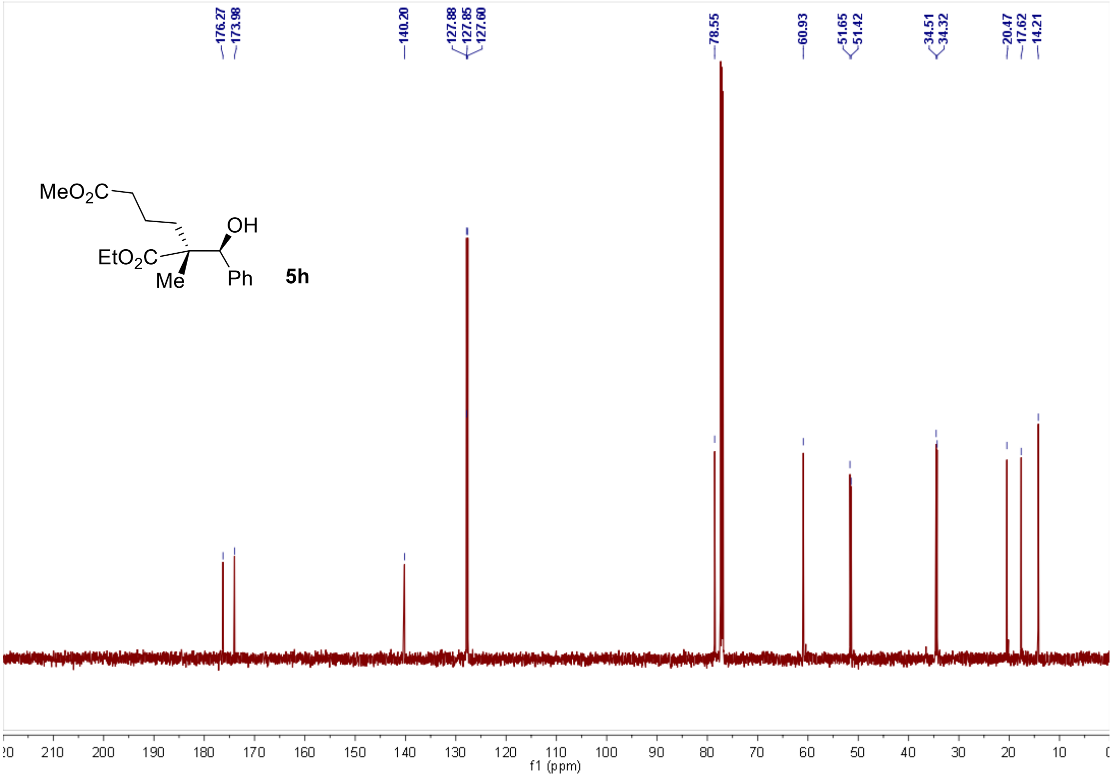

Supplementary Figure 222. ^{1}H NMR sepctra of 1-Ethyl 6-methyl (*S*)-2-methyl-2-((*R*)-phenyl(propionyloxy)methyl)hexanedioate (**6h**)

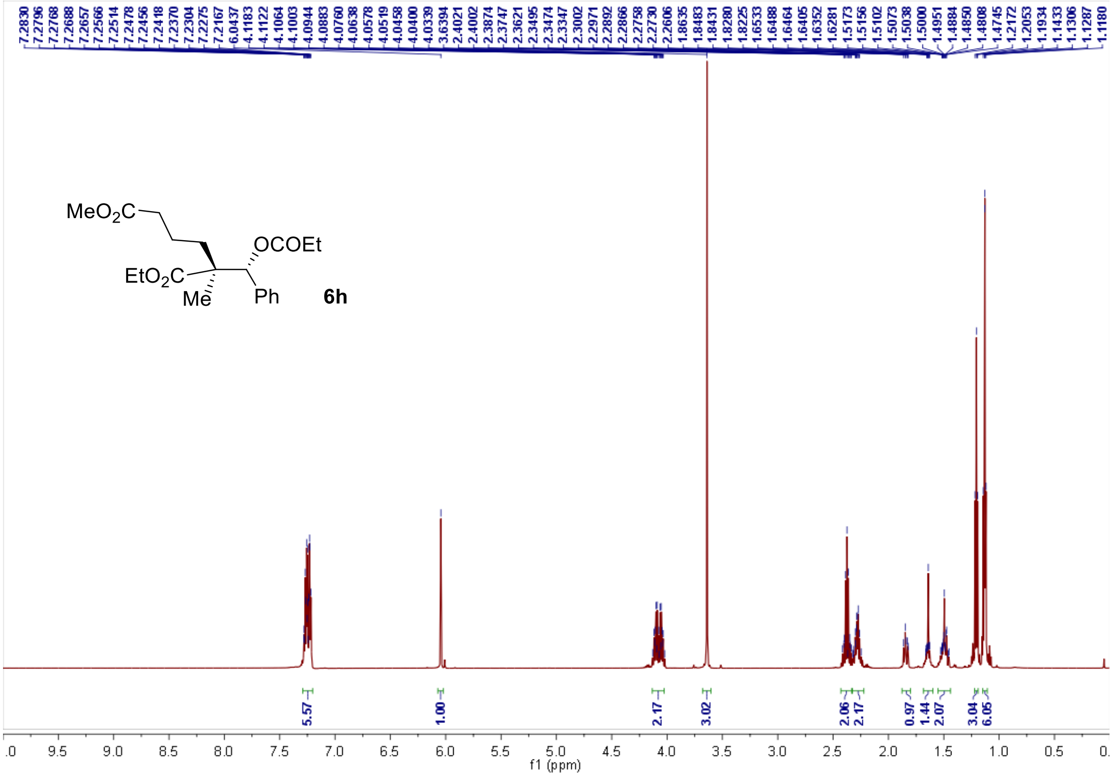

Supplementary Figure 223. ^{13}C NMR sepctra of 1-Ethyl 6-methyl (*S*)-2-methyl-2-((*R*)-phenyl(propionyloxy)methyl)hexanedioate (**6h**)

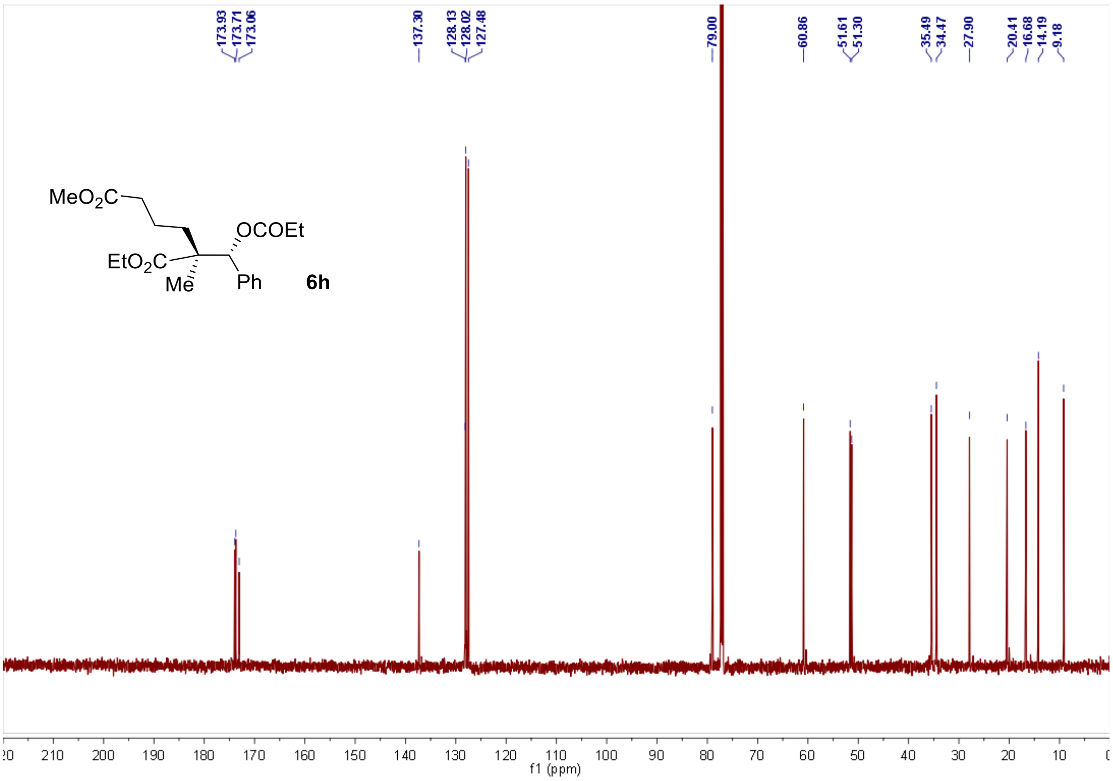

Supplementary Figure 224. ^{1}H NMR sepctra of Ethyl (*R*)-2-((*S*)-hydroxy(phenyl)methyl)-2-methyl-3-methylenenonanoate (**5i**)

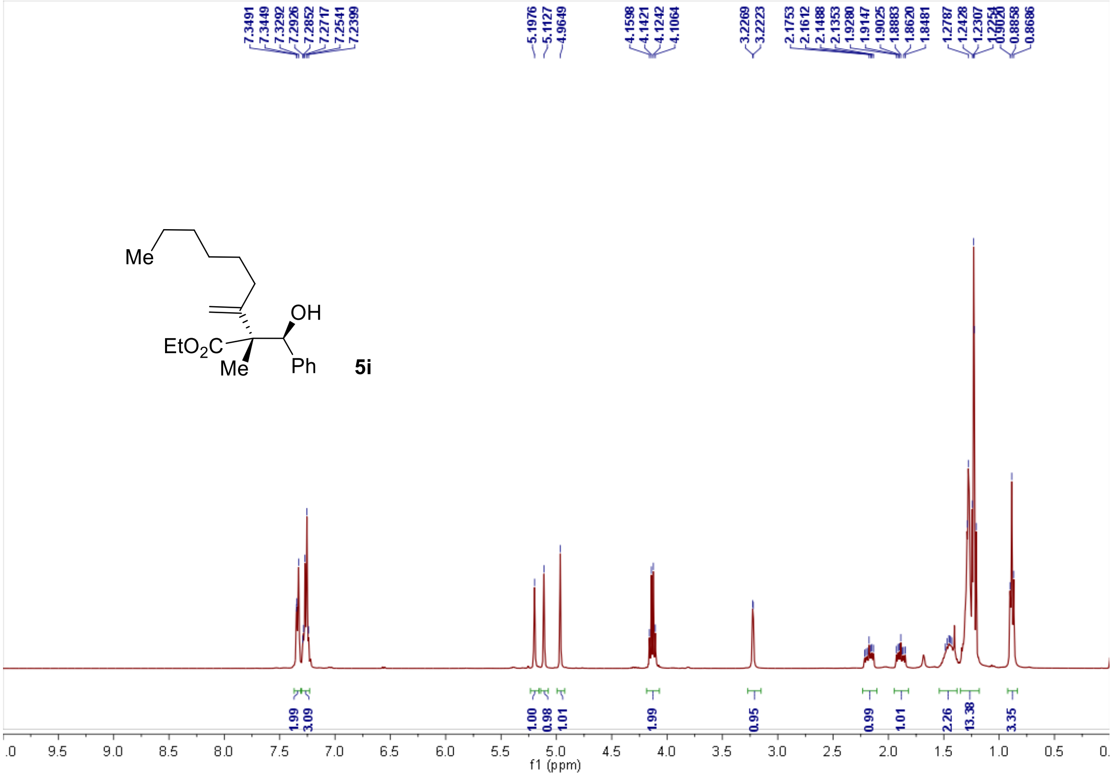

Supplementary Figure 225. ^{13}C NMR sepctra of Ethyl (*R*)-2-((*S*)-hydroxy(phenyl)methyl)-2-methyl-3-methylenenonanoate (**5i**)

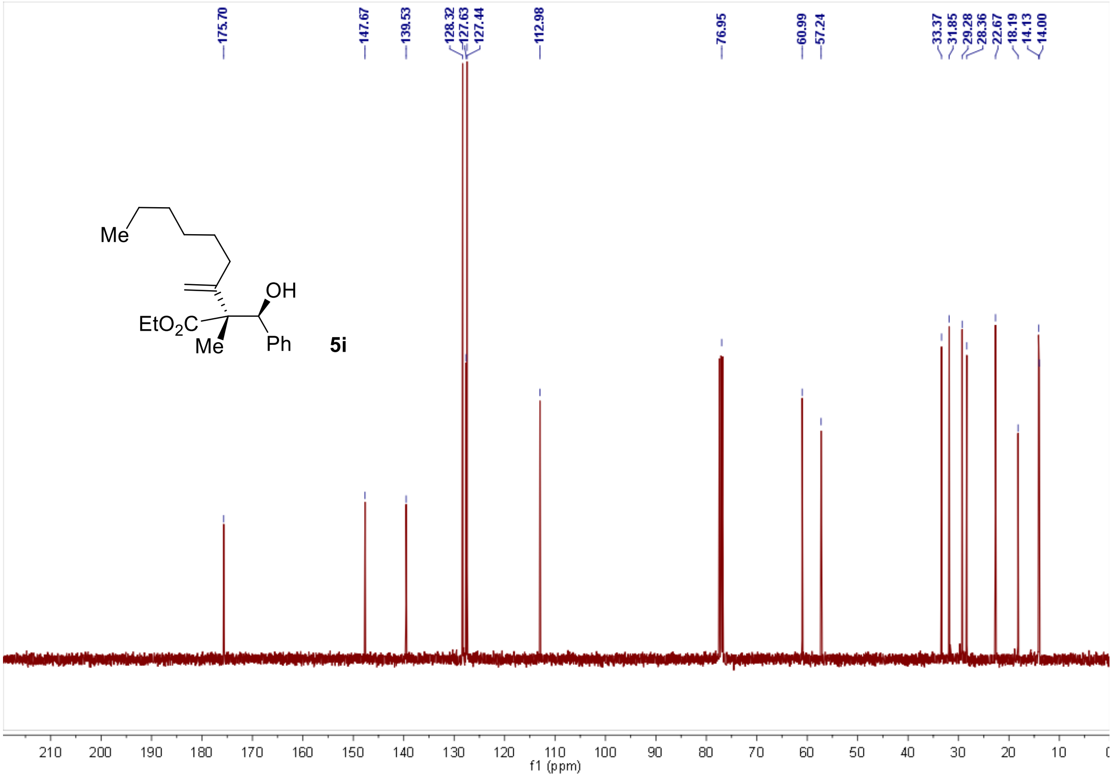

Supplementary Figure 226. ^{1}H NMR sepctra of Ethyl (*S*)-2-methyl-3-methylene-2-((*R*)-phenyl(propionyloxy)methyl)nonanoate (**6i**)

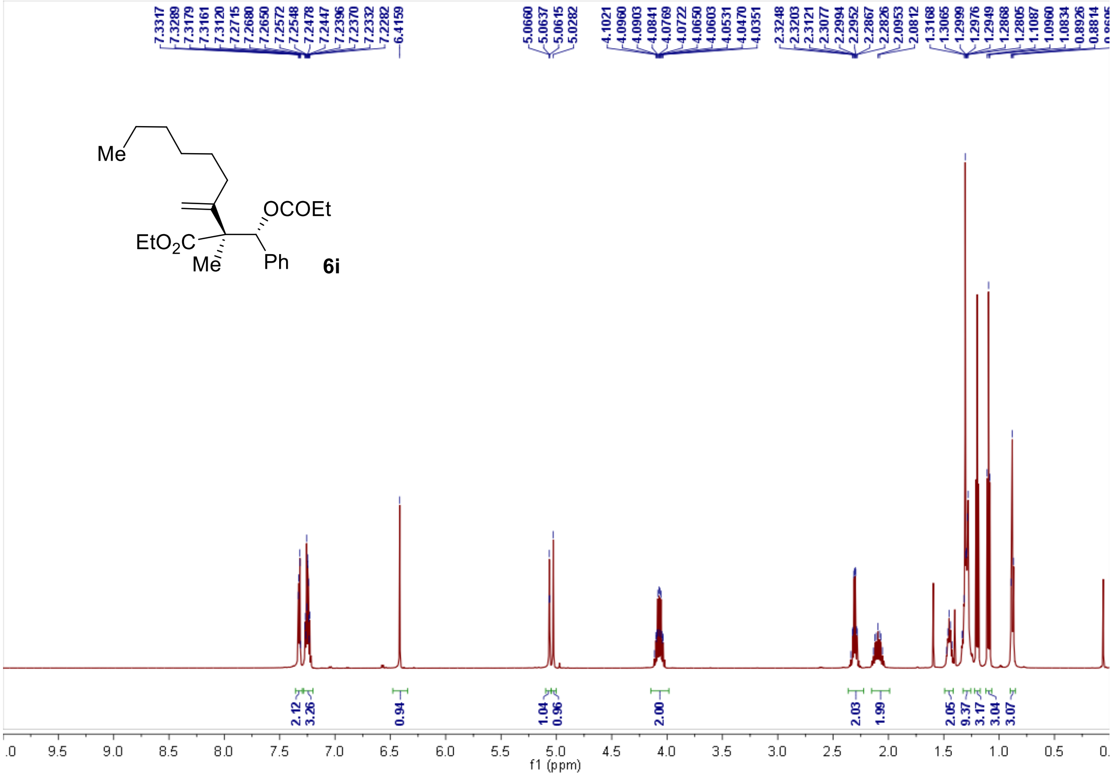

Supplementary Figure 227. ^{13}C NMR sepctra of Ethyl (*S*)-2-methyl-3-methylene-2-((*R*)-phenyl(propionyloxy)methyl)nonanoate (**6i**)

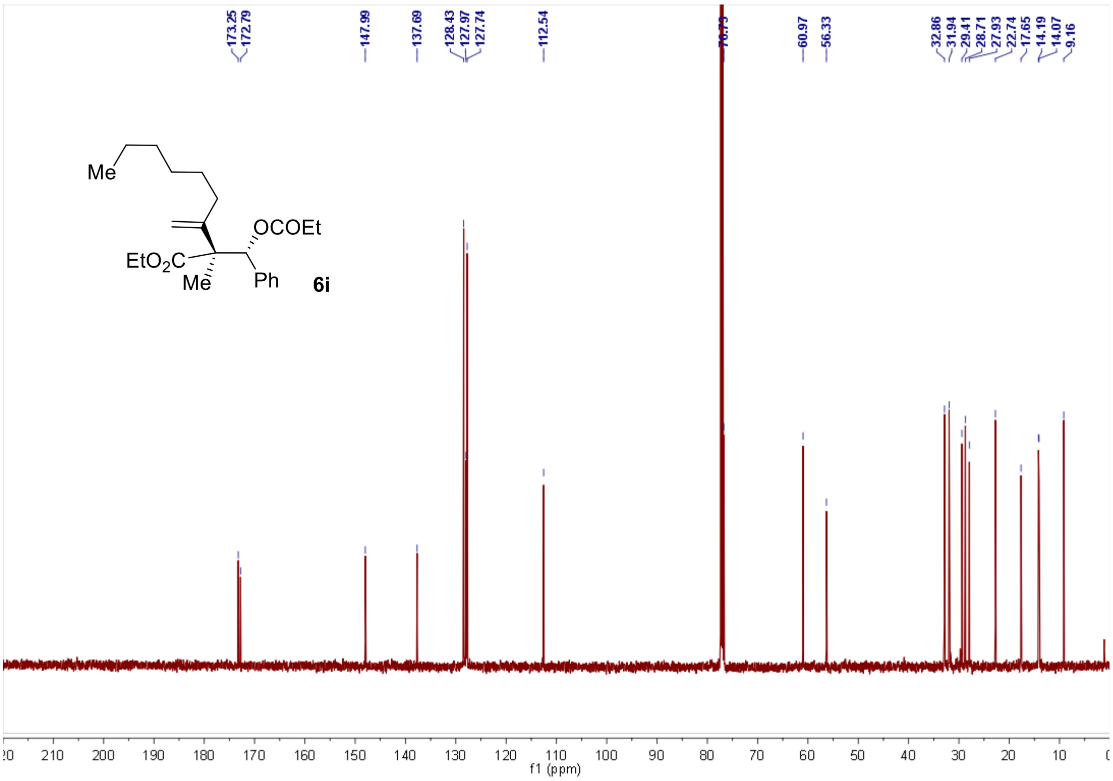

Supplementary Figure 228. ^1H NMR sepctra of Ethyl (*R*)-2-benzyl-2-((*S*)-hydroxy(naphthalen-2-yl)methyl)pent-4-enoate (**5j**)

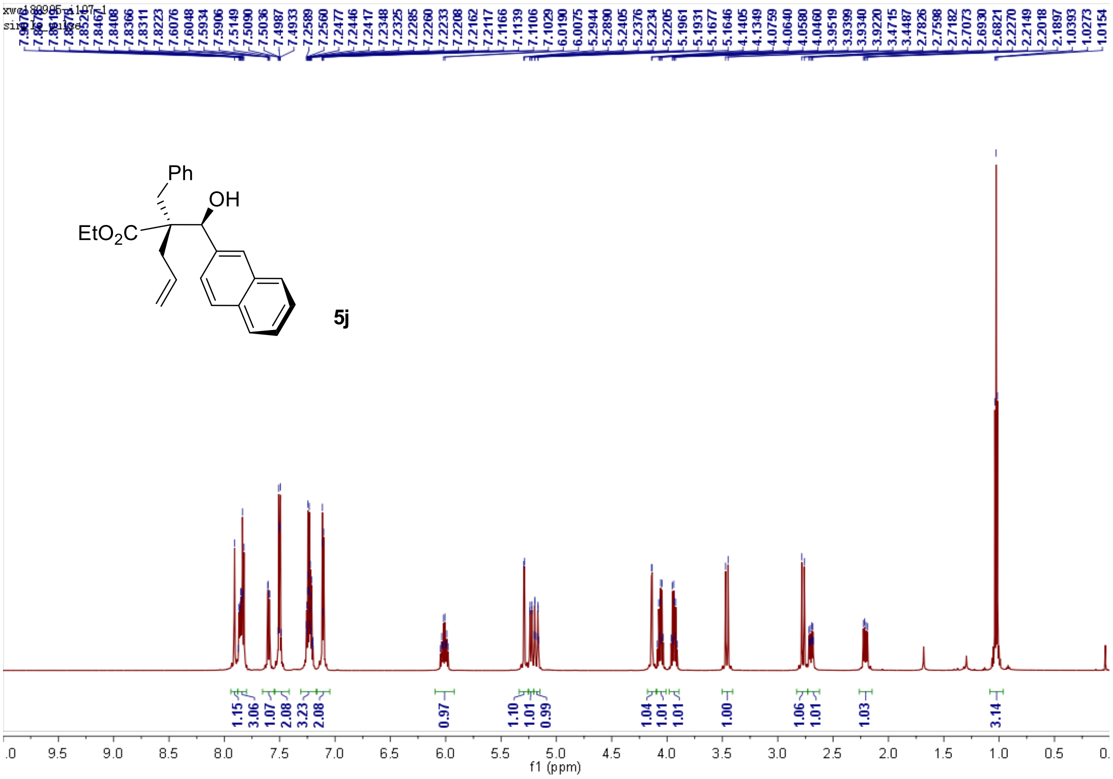

Supplementary Figure 229. ^{13}C NMR sepctra of Ethyl (*R*)-2-benzyl-2-((*S*)-hydroxy(naphthalen-2-yl)methyl)pent-4-enoate (**5j**)

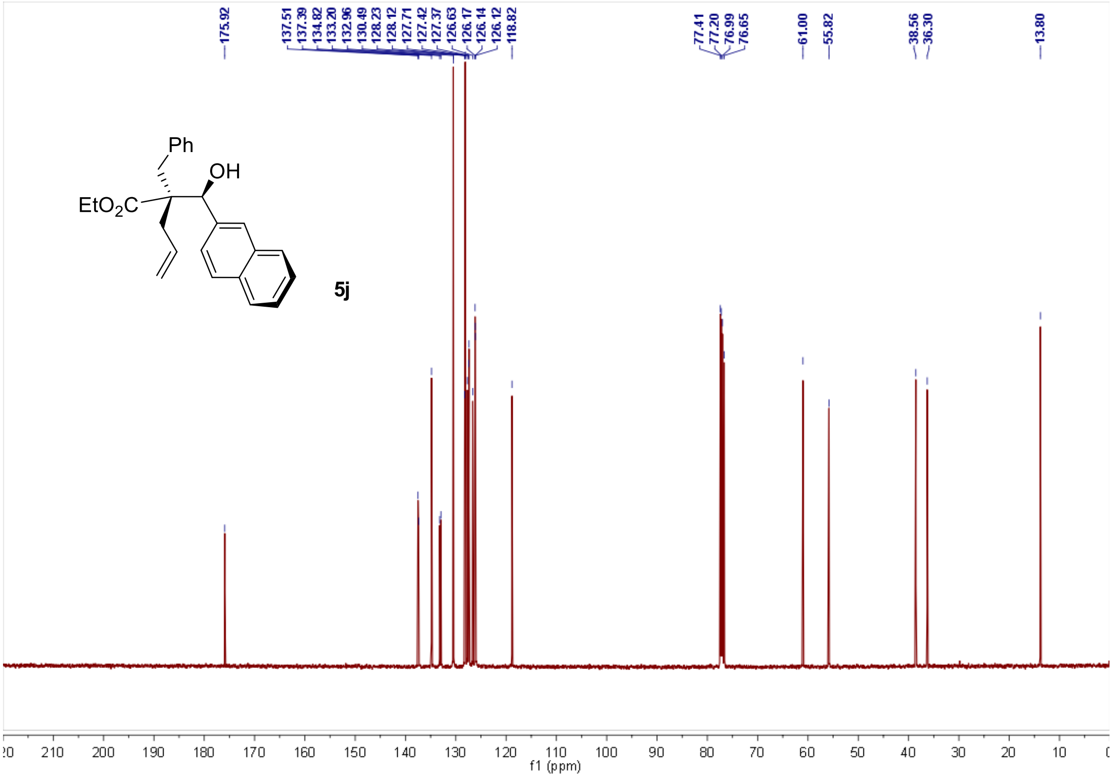

Supplementary Figure 230. ^{1}H NMR sepctra of Ethyl (*S*)-2-benzyl-2-((*R*)-naphthalen-2-yl(propionyloxy)methyl)pent-4-enoate (**6j**)

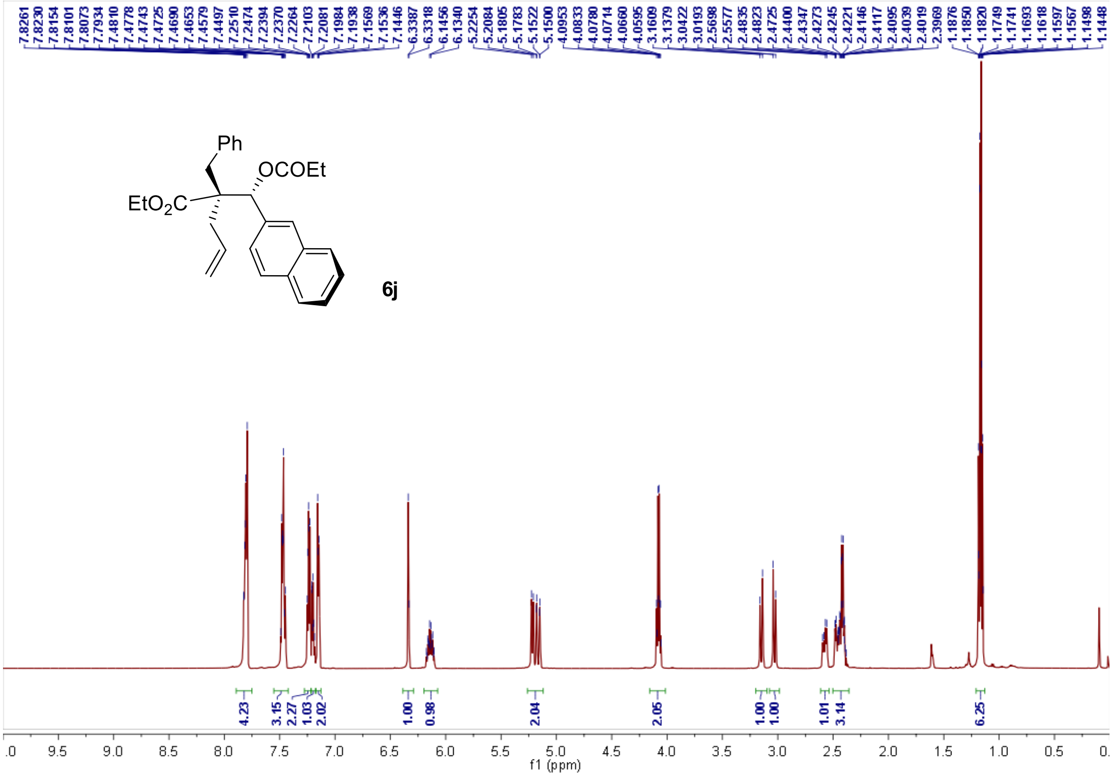

Supplementary Figure 231. ^{13}C NMR sepctra of Ethyl (*S*)-2-benzyl-2-((*R*)-naphthalen-2-yl(propionyloxy)methyl)pent-4-enoate (**6j**)

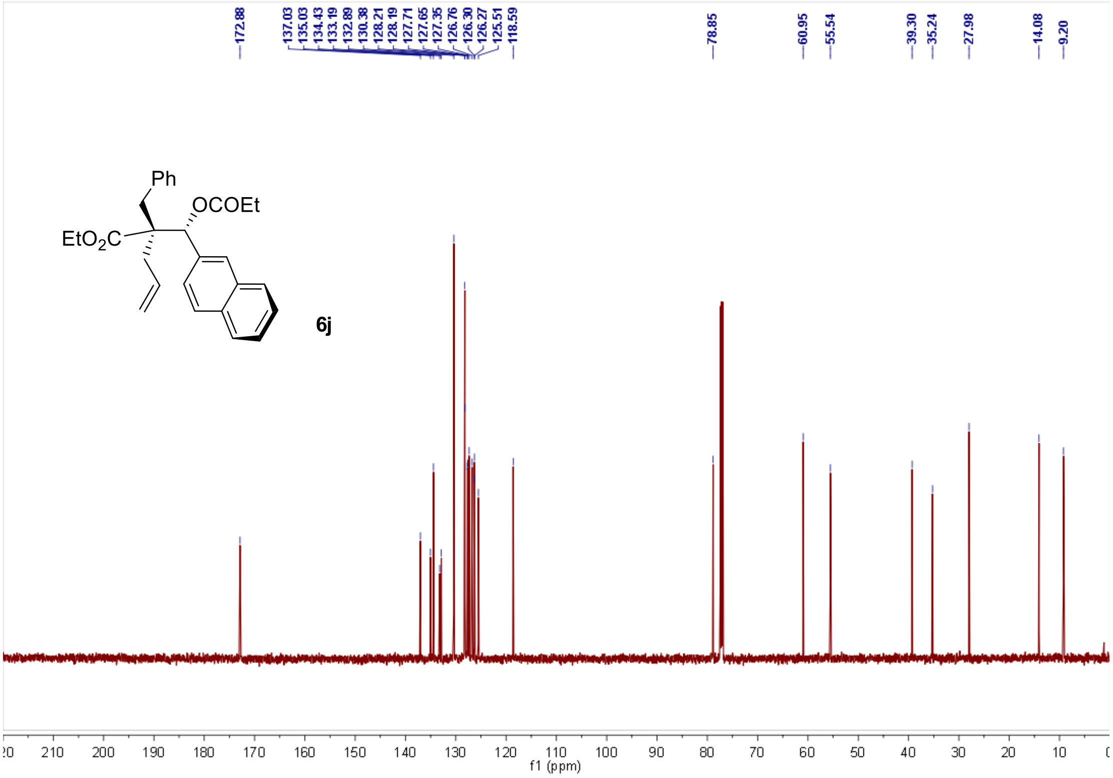

Supplementary Figure 232. ^{13}H NMR sepctra of Ethyl (*R*)-2-((*S*)-hydroxy(naphthalen-2-yl)methyl)-2-methylpent-4-enoate (**5k**)

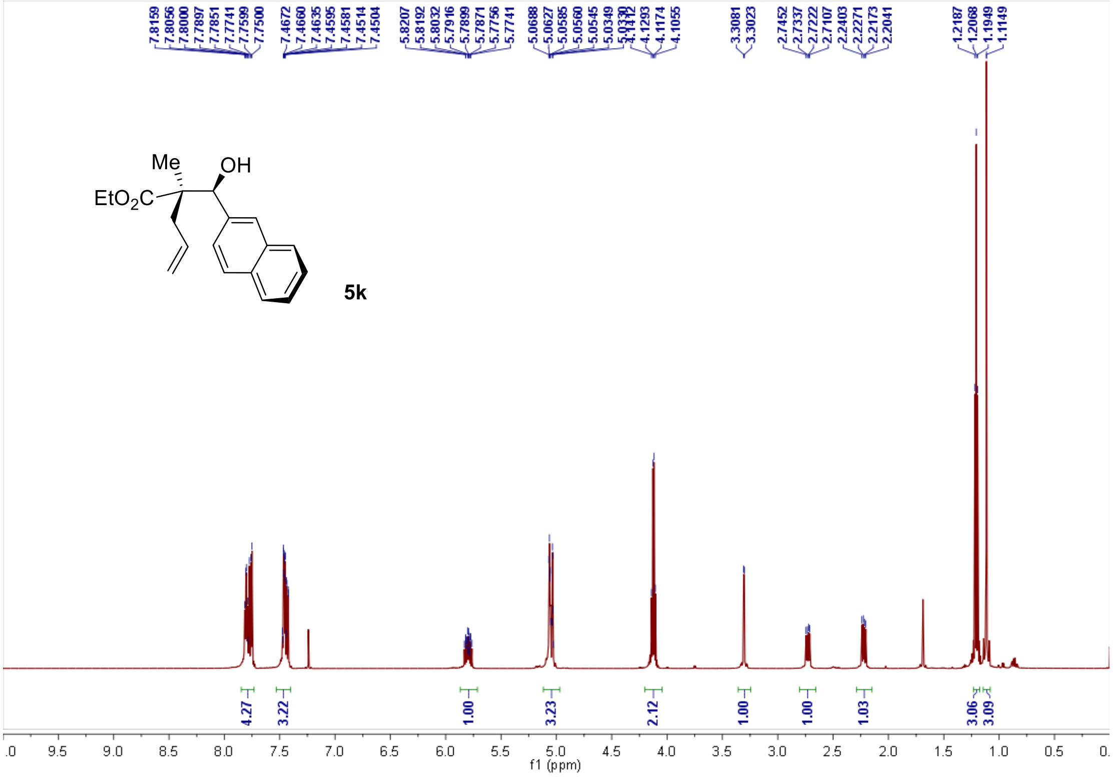

Supplementary Figure 233. ^{13}C NMR sepctra of Ethyl (*R*)-2-((*S*)-hydroxy(naphthalen-2-yl)methyl)-2-methylpent-4-enoate (**5k**)

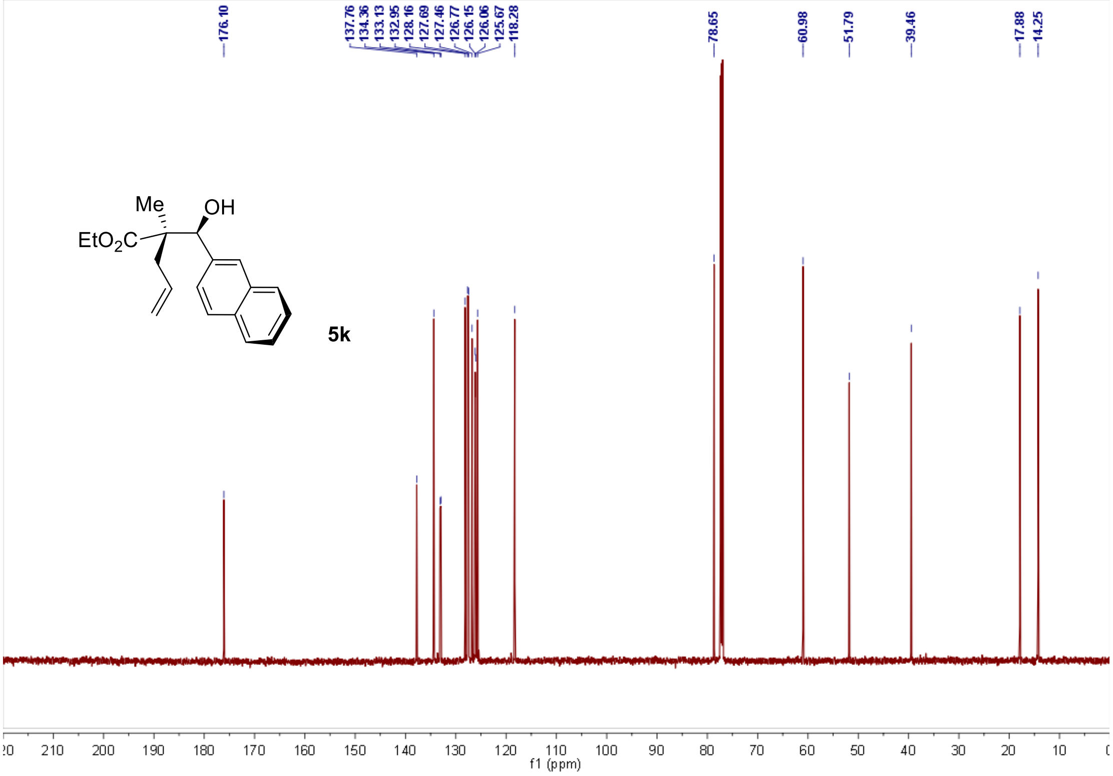

Supplementary Figure 234. ^{1}H NMR sepctra of Ethyl (*S*)-2-methyl-2-((*R*)-naphthalen-2-yl(propionyloxy)methyl)pent-4-enoate (**6k**)

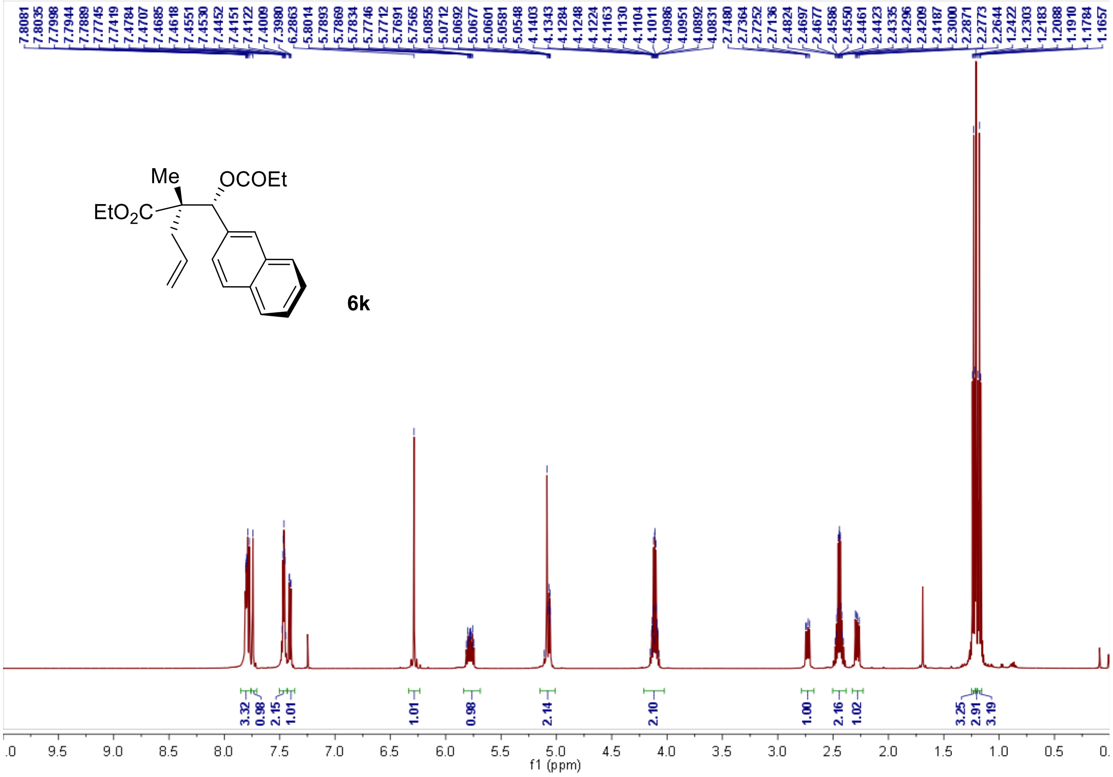

Supplementary Figure 235. ^{13}C NMR sepctra of Ethyl (*S*)-2-methyl-2-((*R*)-naphthalen-2-yl(propionyloxy)methyl)pent-4-enoate (**6k**)

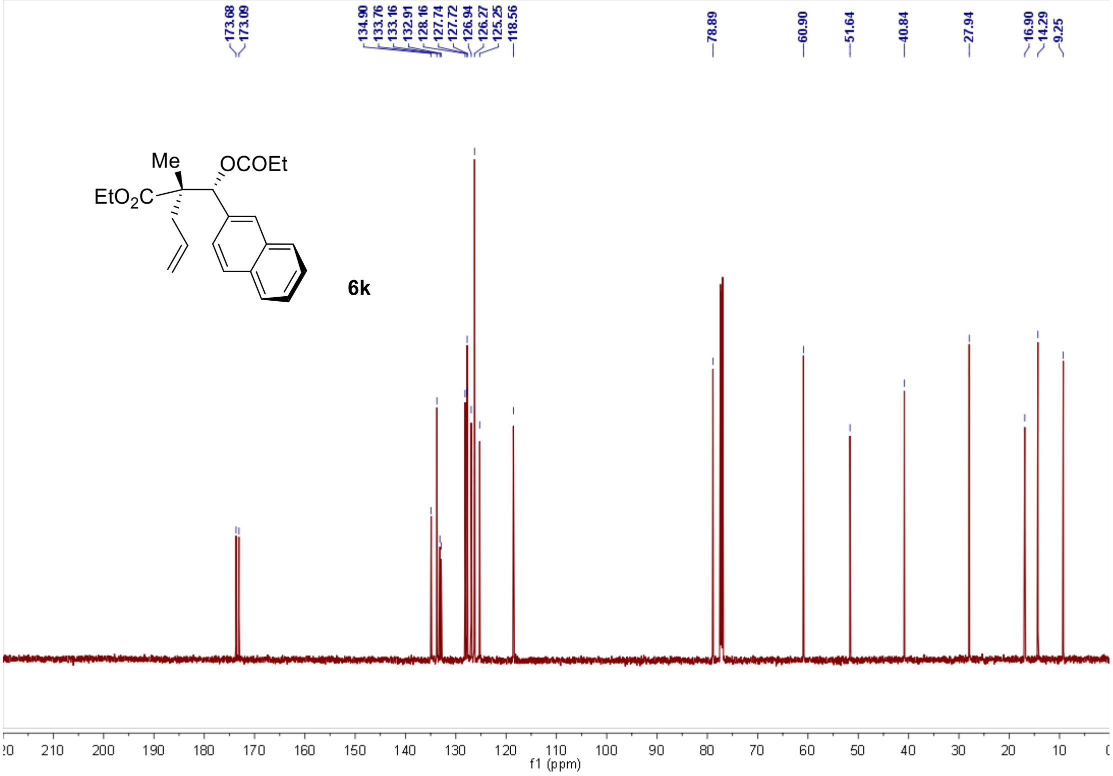

Supplementary Figure 236. ^{1}H NMR sepctra of Ethyl (*S*)-2-((*S*)-hydroxy(naphthalen-2-yl)methyl)-2-methylpent-4-enoate (**5l**)

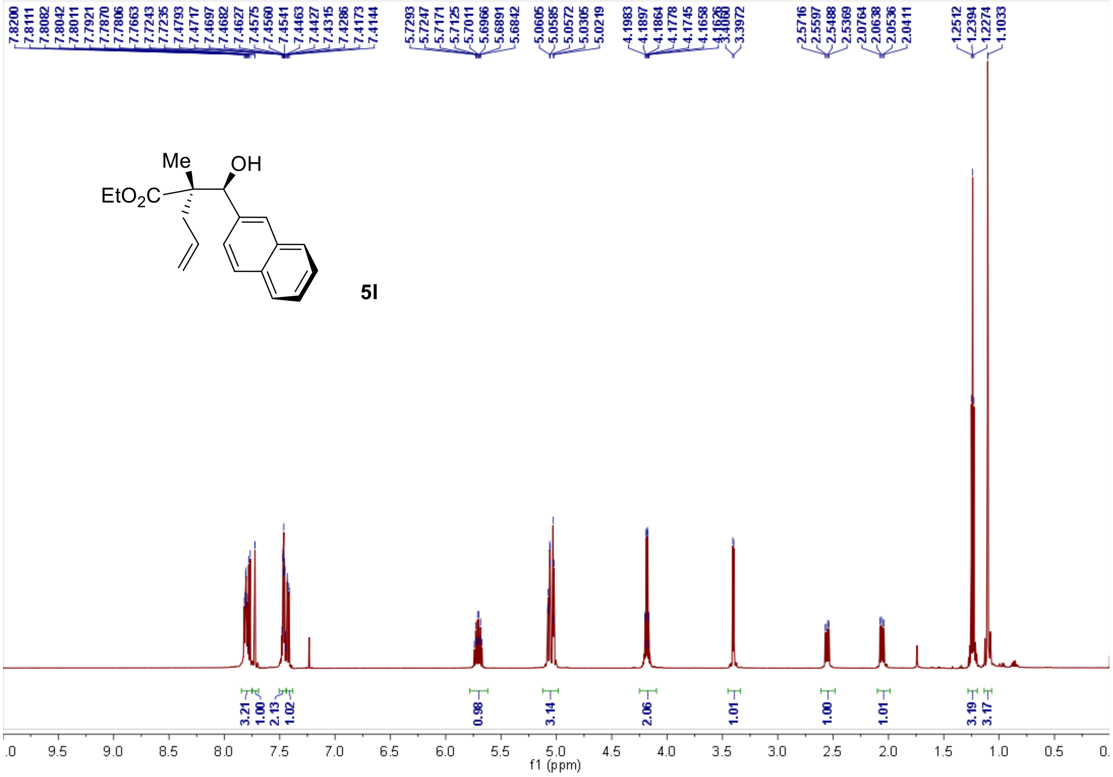

Supplementary Figure 237. ^{13}C NMR sepctra of Ethyl (*S*)-2-((*S*)-hydroxy(naphthalen-2-yl)methyl)-2-methylpent-4-enoate (**5l**)

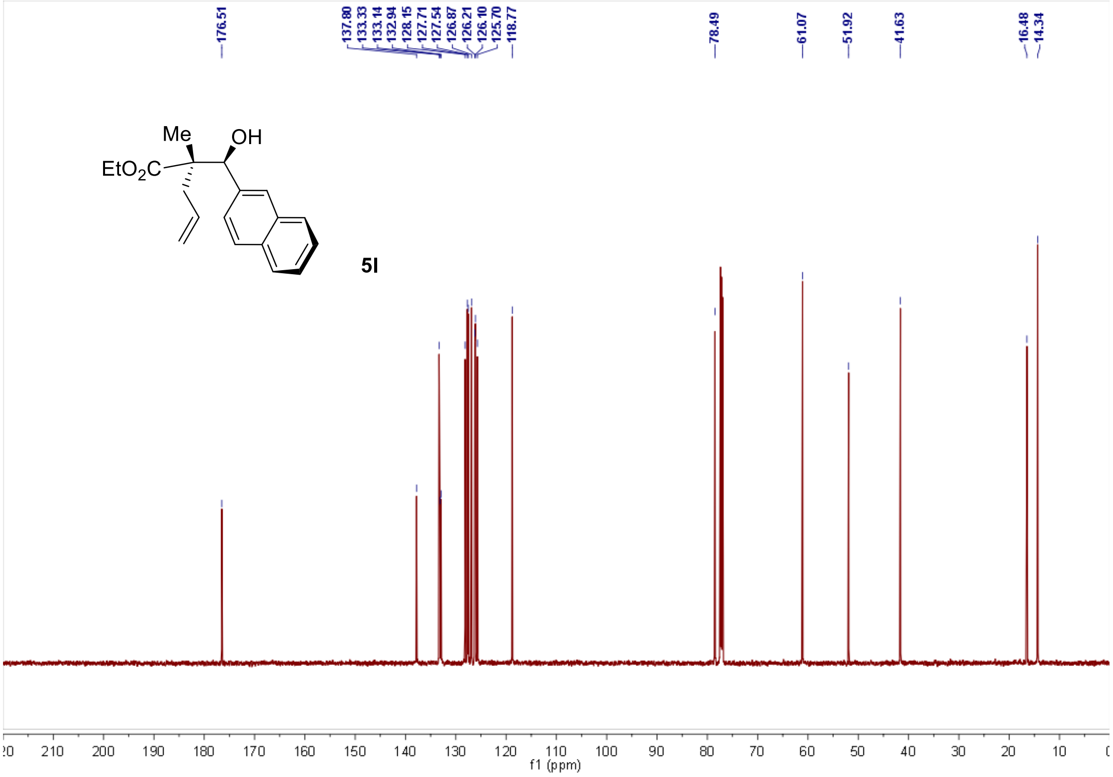

Supplementary Figure 238. ^{1}H NMR sepctra of Ethyl (*R*)-2-methyl-2-((*R*)-naphthalen-2-yl(propionyloxy)methyl)pent-4-enoate (**6l**)

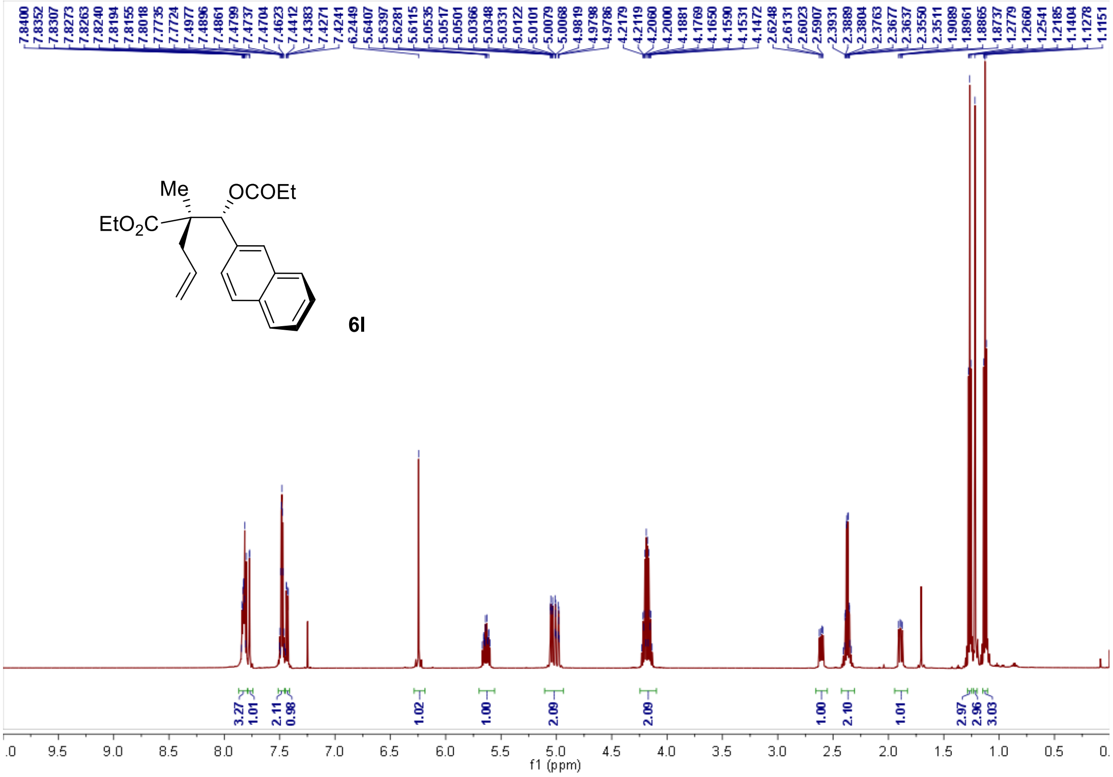

Supplementary Figure 239. ^{13}C NMR sepctra of Ethyl (*R*)-2-methyl-2-((*R*)-naphthalen-2-yl(propionyloxy)methyl)pent-4-enoate (**6l**)

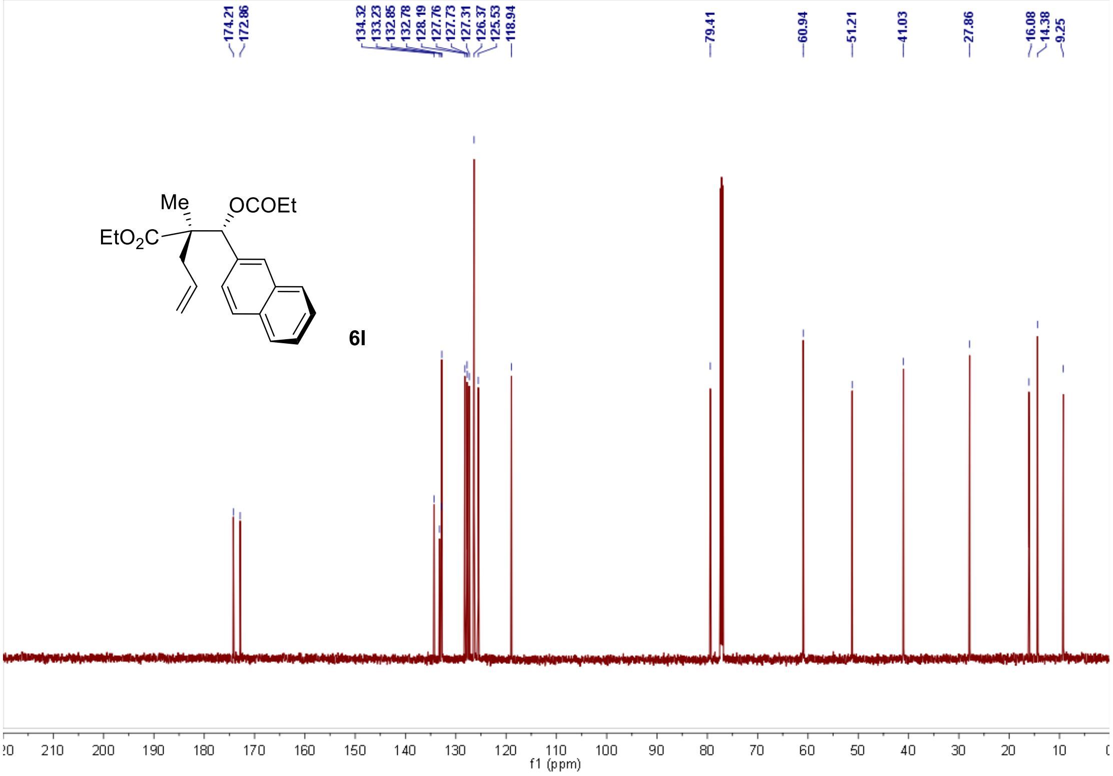

Supplementary Figure 240. ^{1}H NMR sepctra of (*S*)-Methyl 2-benzyl-2-((*S*)-hydroxy(naphthalen-2-yl)methyl)pent-4-enoate (**5m**)

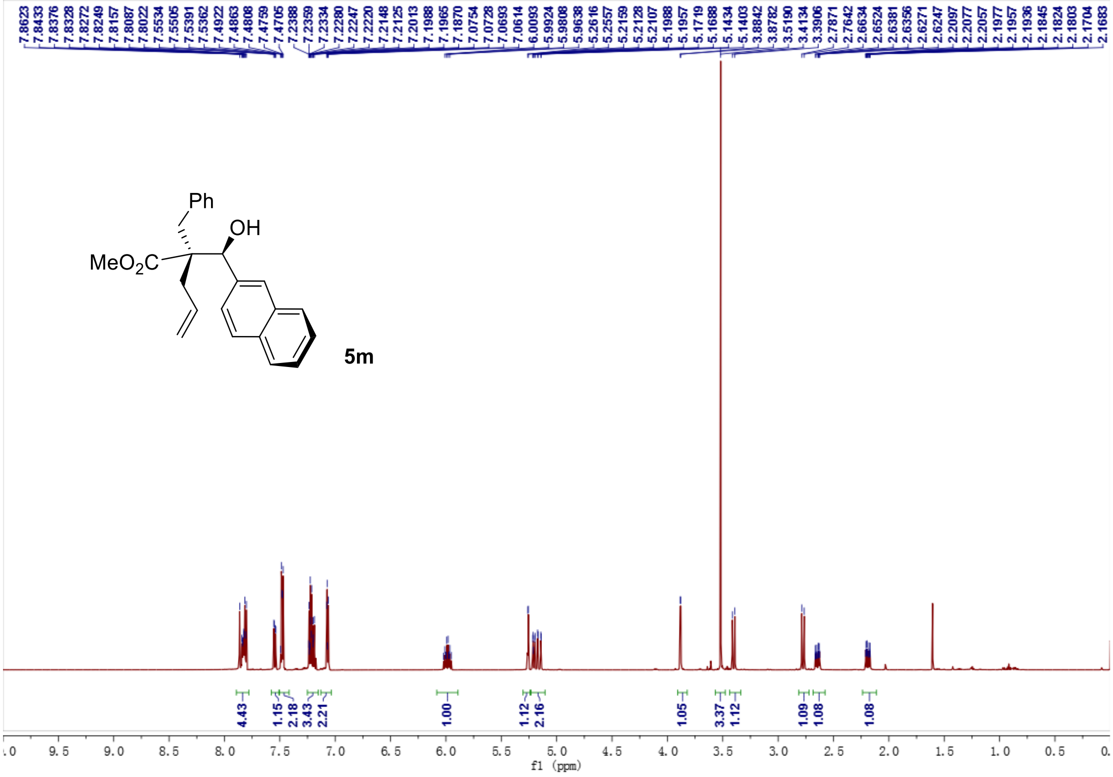

Supplementary Figure 241. ^{13}C NMR sepctra of (*S*)-Methyl 2-benzyl-2-((*S*)-hydroxy(naphthalen-2-yl)methyl)pent-4-enoate (**5m**)

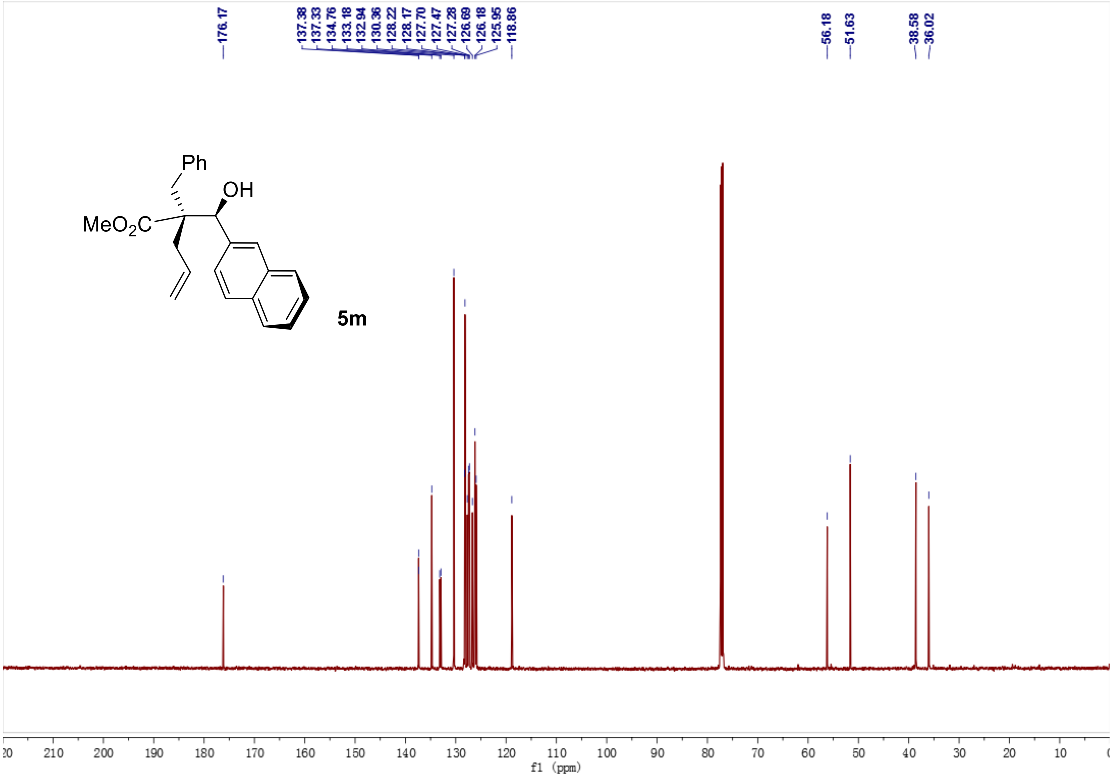

Supplementary Figure 242. ^{1}H NMR sepctra of (*R*)-Methyl 2-benzyl-2-((*R*)-naphthalen-2-yl(propionyloxy)methyl)pent-4-enoate (**6m**)

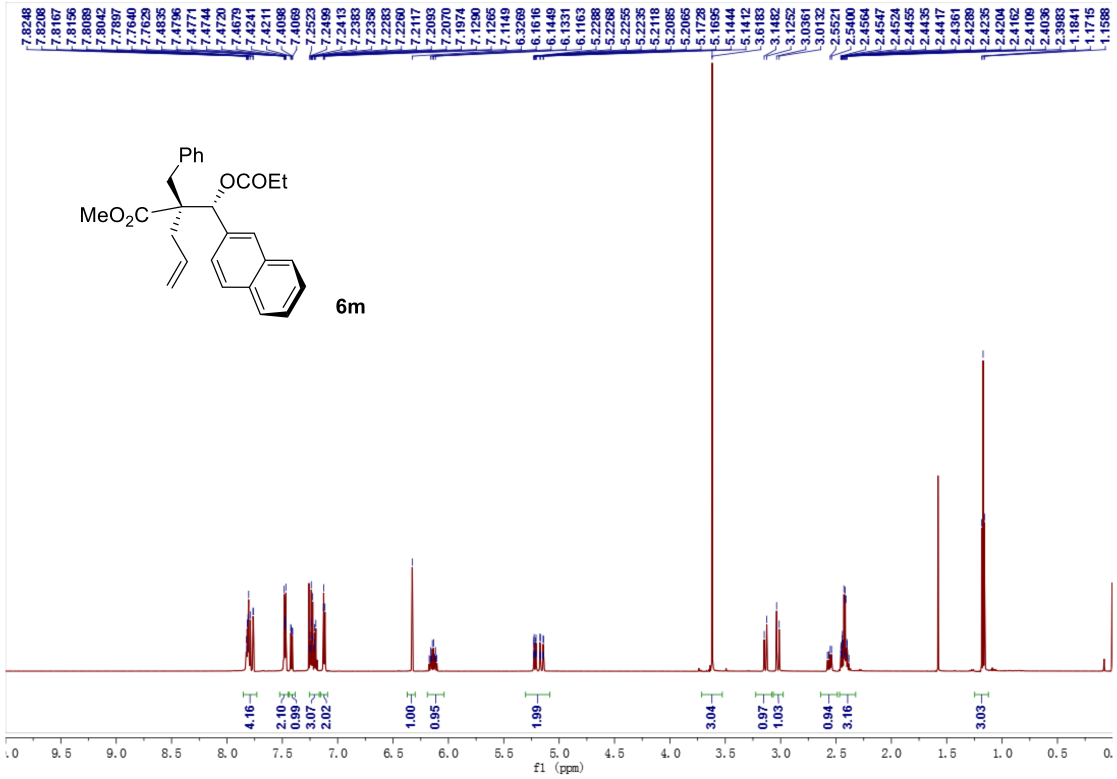

Supplementary Figure 243. ^{13}C NMR sepctra of (*R*)-Methyl 2-benzyl-2-((*R*)-naphthalen-2-yl(propionyloxy)methyl)pent-4-enoate (**6m**)

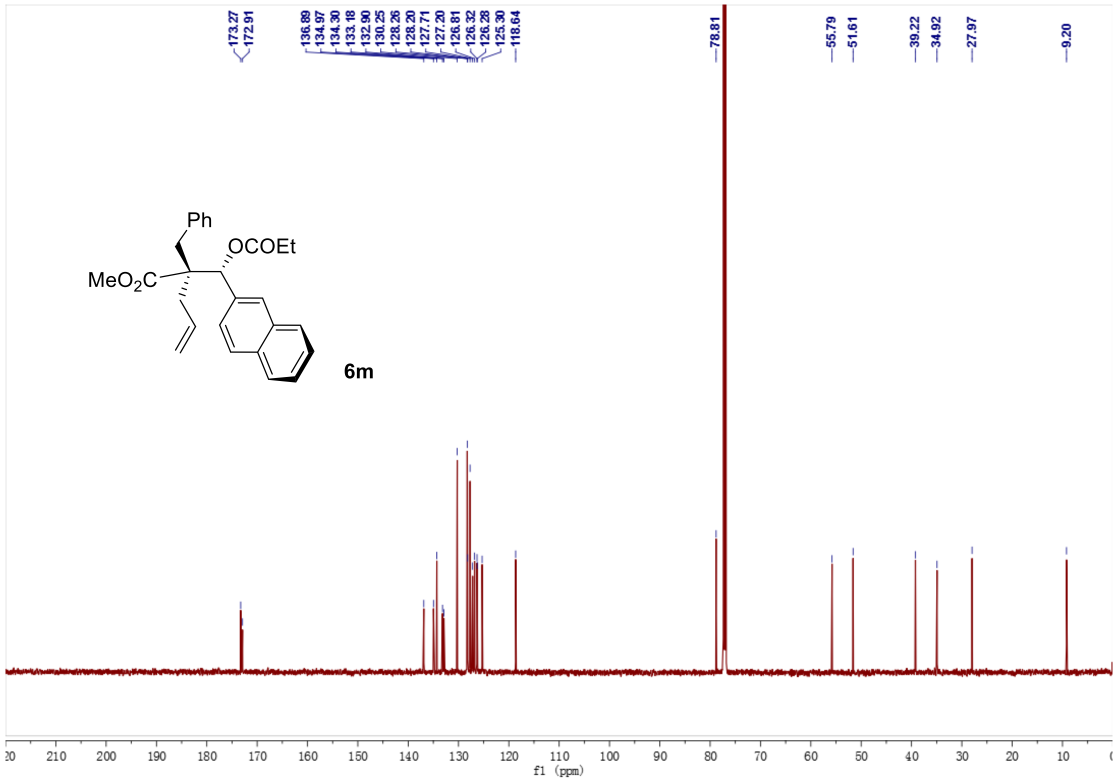

Supplementary Figure 244. ^{1}H NMR sepctra of Ethyl (*R*)-2-((*R*)-furan-2-yl(hydroxy)methyl)-2-methylpent-4-enoate (**5n**)

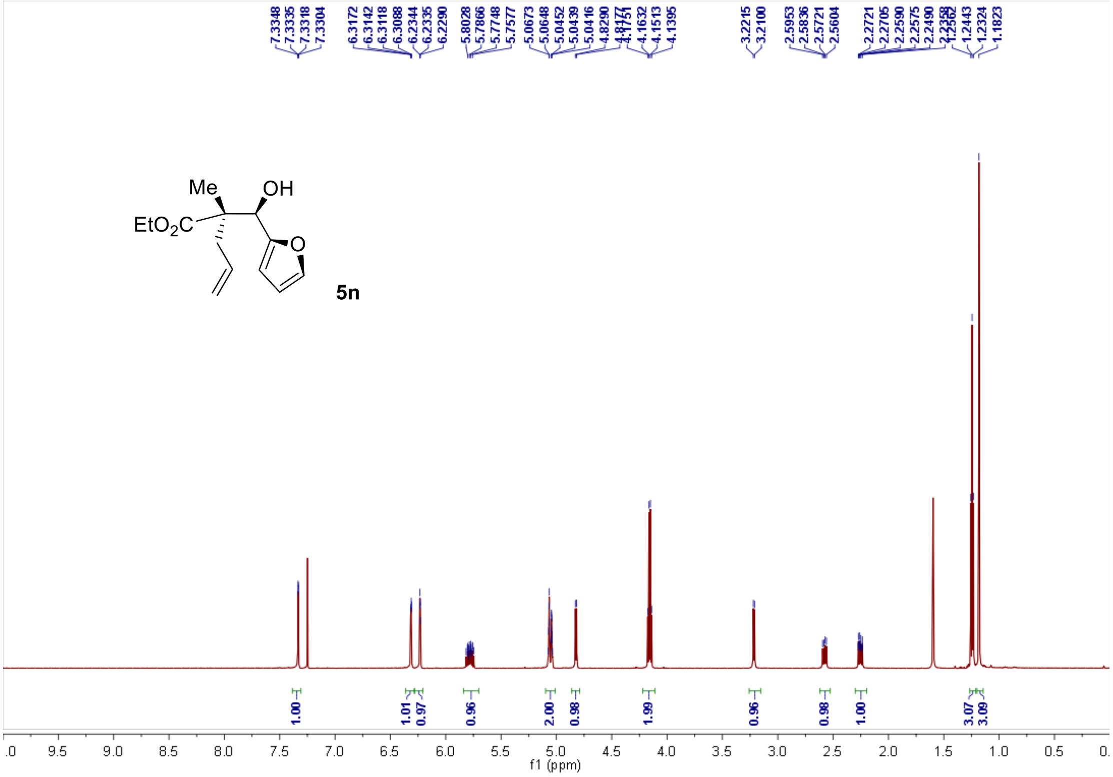

Supplementary Figure 245. ^{13}C NMR sepctra of Ethyl (*R*)-2-((*R*)-furan-2-yl(hydroxy)methyl)-2-methylpent-4-enoate (**5n**)

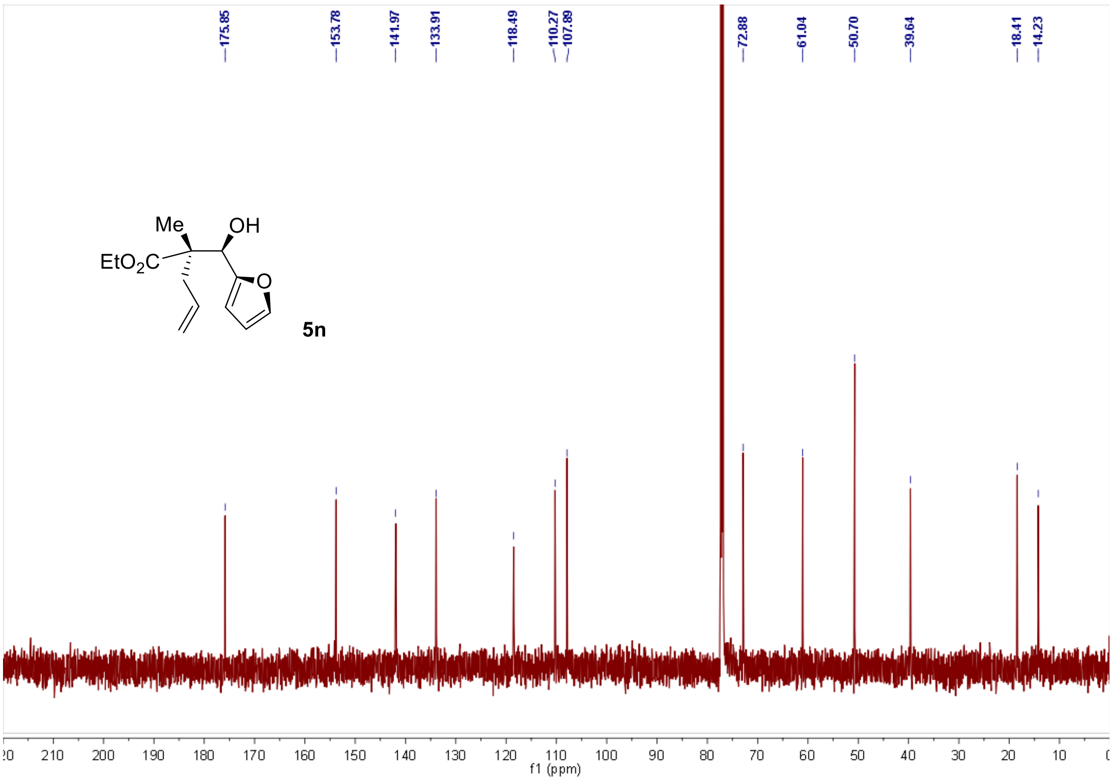

Supplementary Figure 246. ^{1}H NMR sepctra of Ethyl (*S*)-2-((*S*)-furan-2-yl(propionyloxy)methyl)-2-methylpent-4-enoate (**6n**)

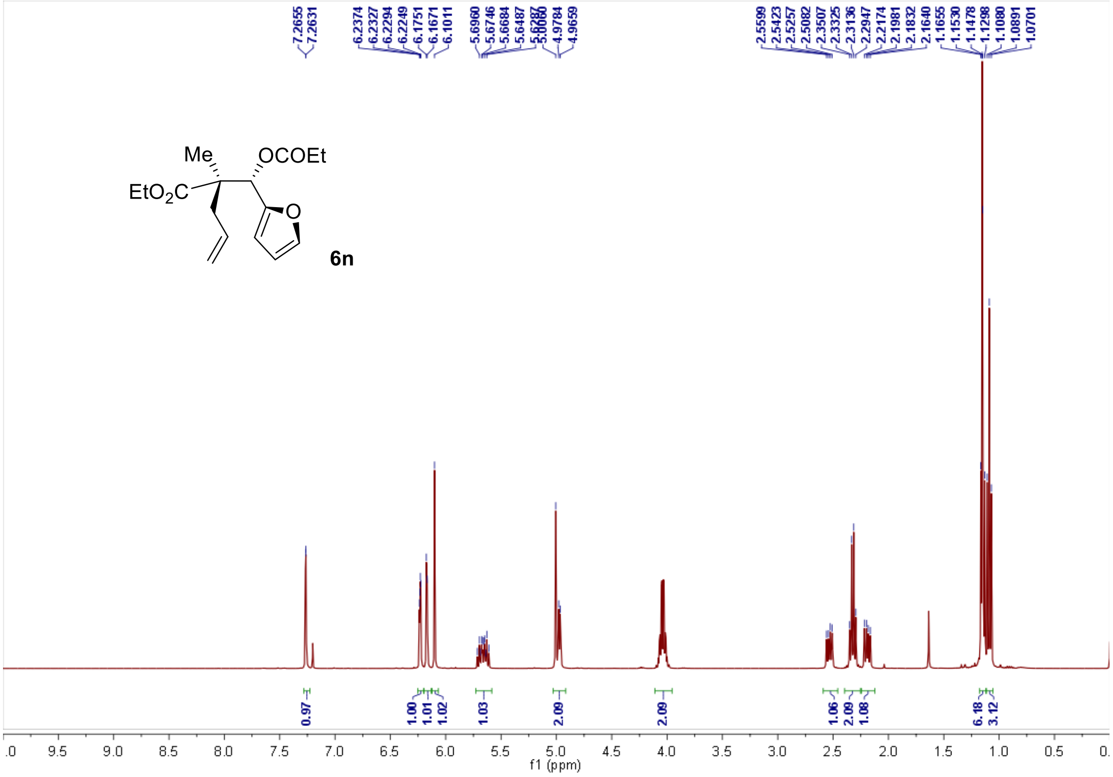

Supplementary Figure 247. ^{13}C NMR sepctra of Ethyl (*S*)-2-((*S*)-furan-2-yl(propionyloxy)methyl)-2-methylpent-4-enoate (**6n**)

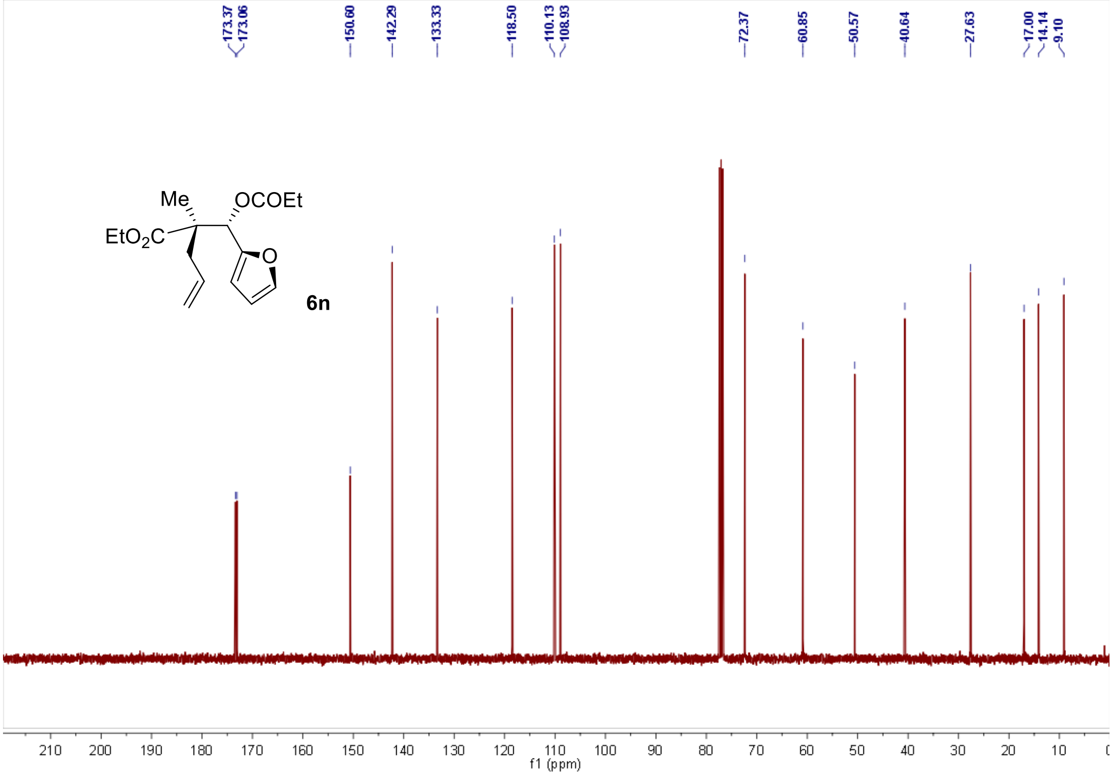

Supplementary Figure 248. ^{1}H NMR sepctra of Ethyl (2*R*,3*S*,*E*)-2-allyl-3-hydroxy-2-methyl-5-phenylpent-4-enoate (**5o**)

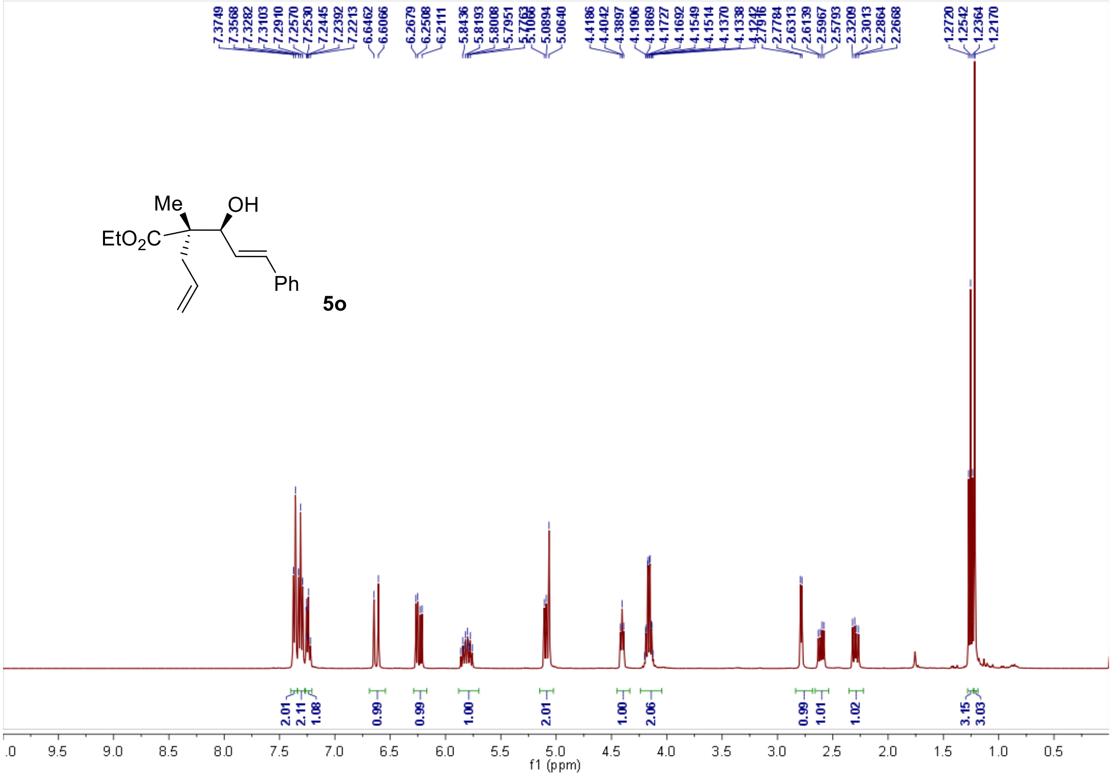

Supplementary Figure 249. ^{13}C NMR sepctra of Ethyl (2*R*,3*S*,*E*)-2-allyl-3-hydroxy-2-methyl-5-phenylpent-4-enoate (**5o**)

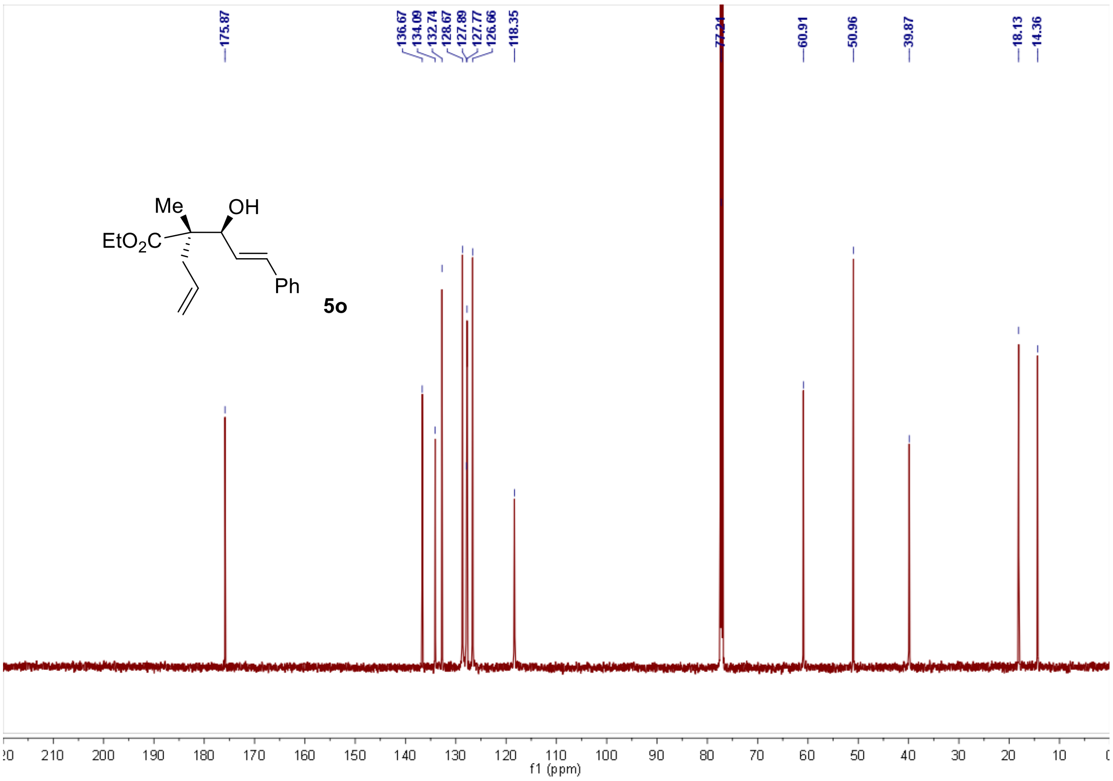

Supplementary Figure 250. ^{1}H NMR sepctra of Ethyl (2*S*,3*R*,*E*)-2-allyl-2-methyl-5-phenyl-3-(propionyloxy)pent-4-enoate (**6o**)

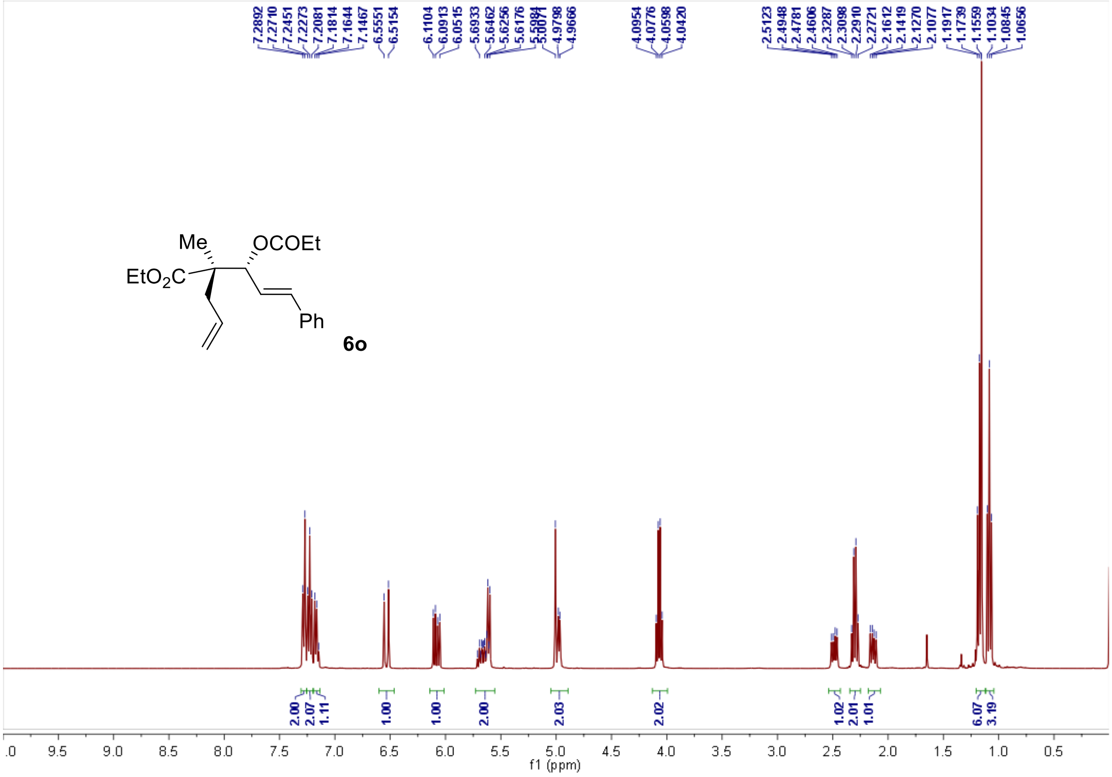

Supplementary Figure 251. ^{13}C NMR sepctra of Ethyl (2*S*,3*R*,*E*)-2-allyl-2-methyl-5-phenyl-3-(propionyloxy)pent-4-enoate (**6o**)

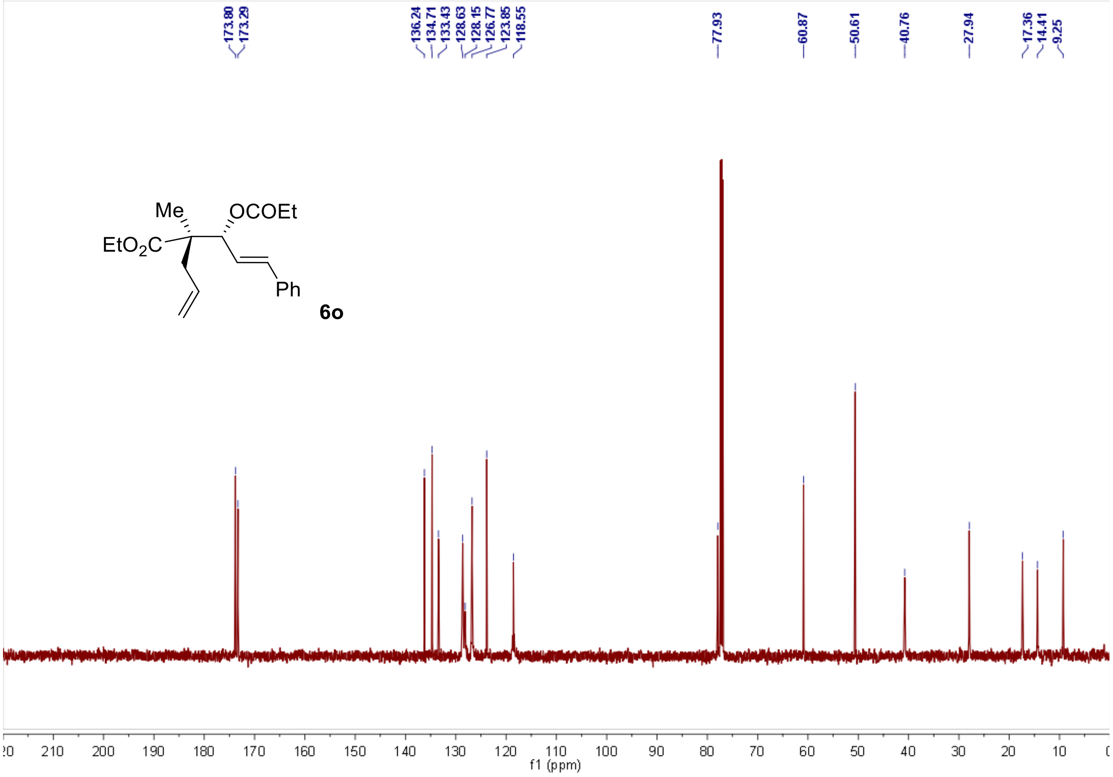

Supplementary Figure 252. ^{1}H NMR sepctra of (1*R*,2*S*)-2-methyl-1-phenyl-2-vinylpent-4-en-1-ol (**7a**)

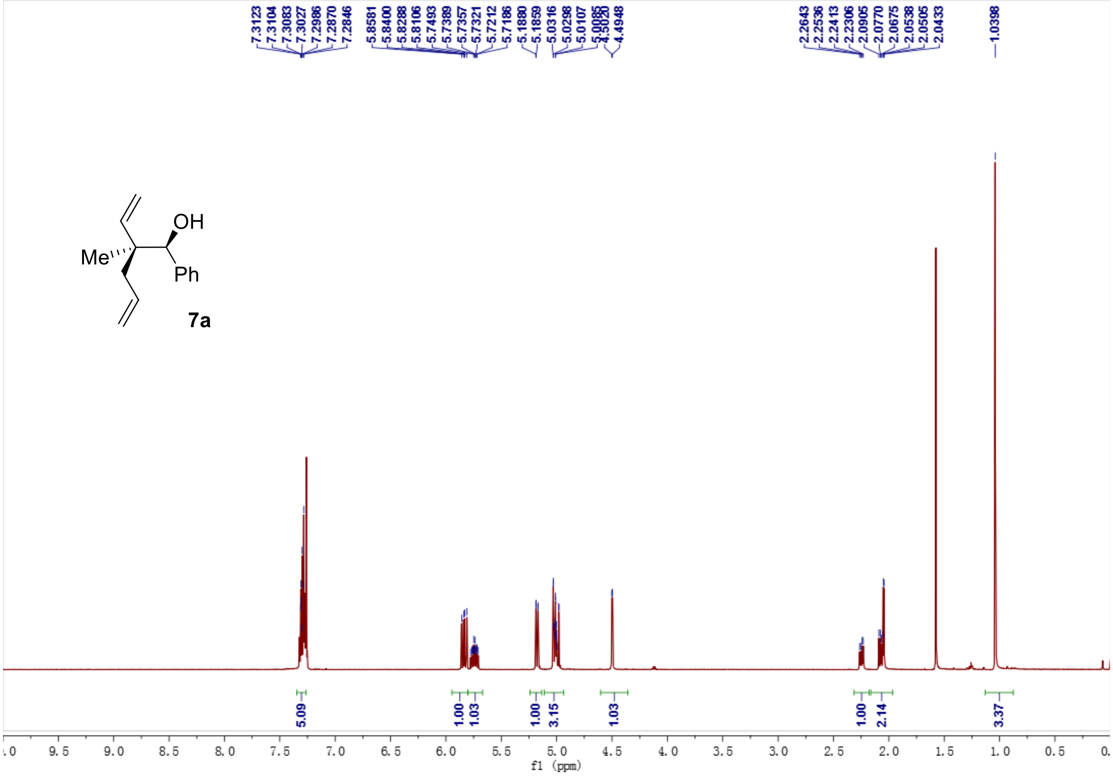

Supplementary Figure 253. ^{13}C NMR sepctra of (1*R*,2*S*)-2-methyl-1-phenyl-2-vinylpent-4-en-1-ol (**7a**)

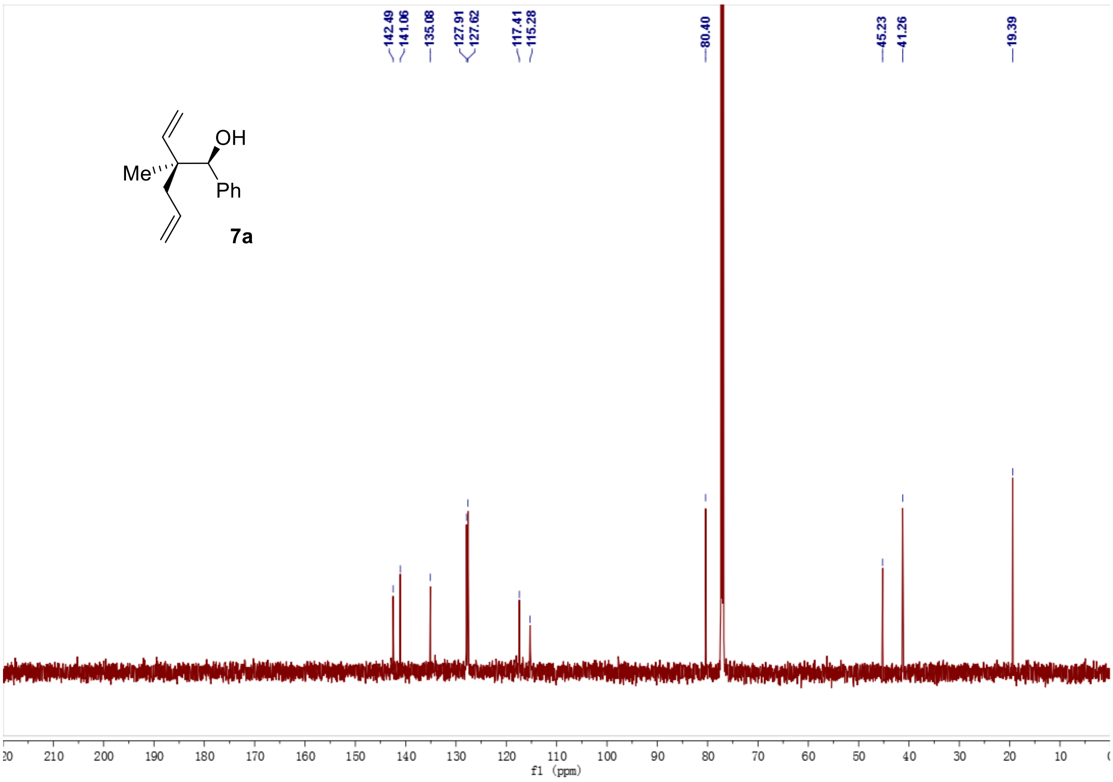

Supplementary Figure 254. ^{1}H NMR sepctra of (1*S*,2*R*)-2-methyl-1-phenyl-2-vinylpent-4-en-1-yl propionate (**8a**)

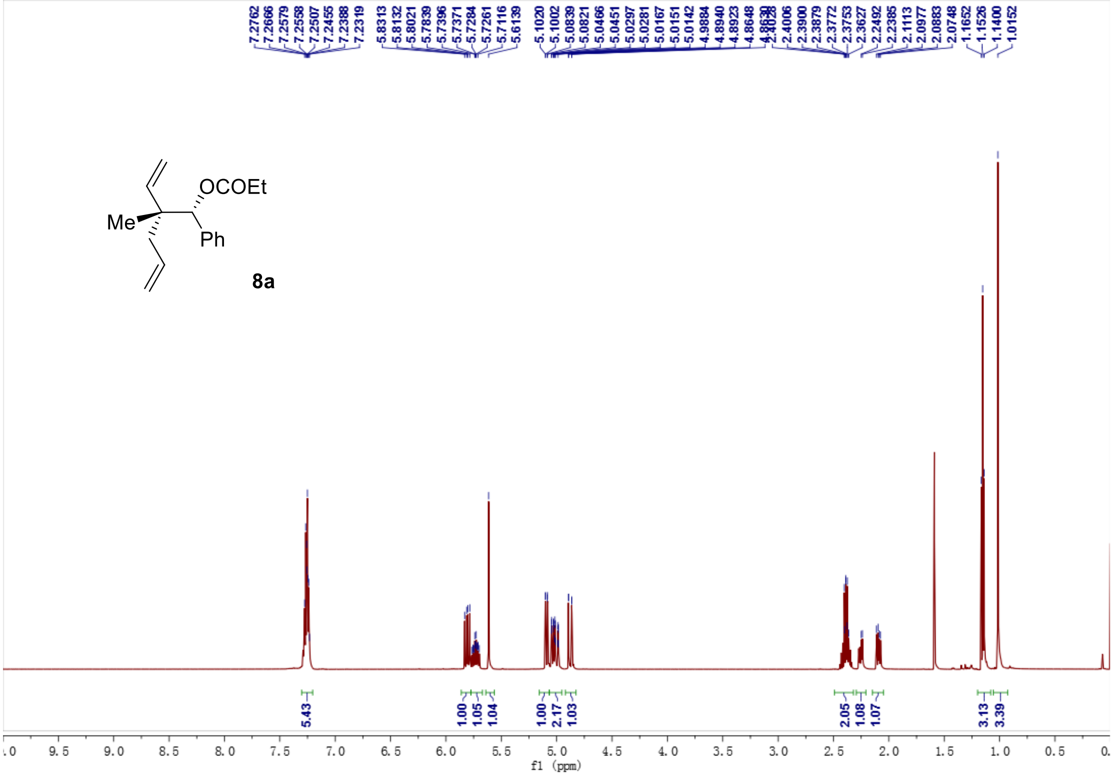

Supplementary Figure 255. ^{13}C NMR sepctra of (1*S*,2*R*)-2-methyl-1-phenyl-2-vinylpent-4-en-1-yl propionate (**8a**)

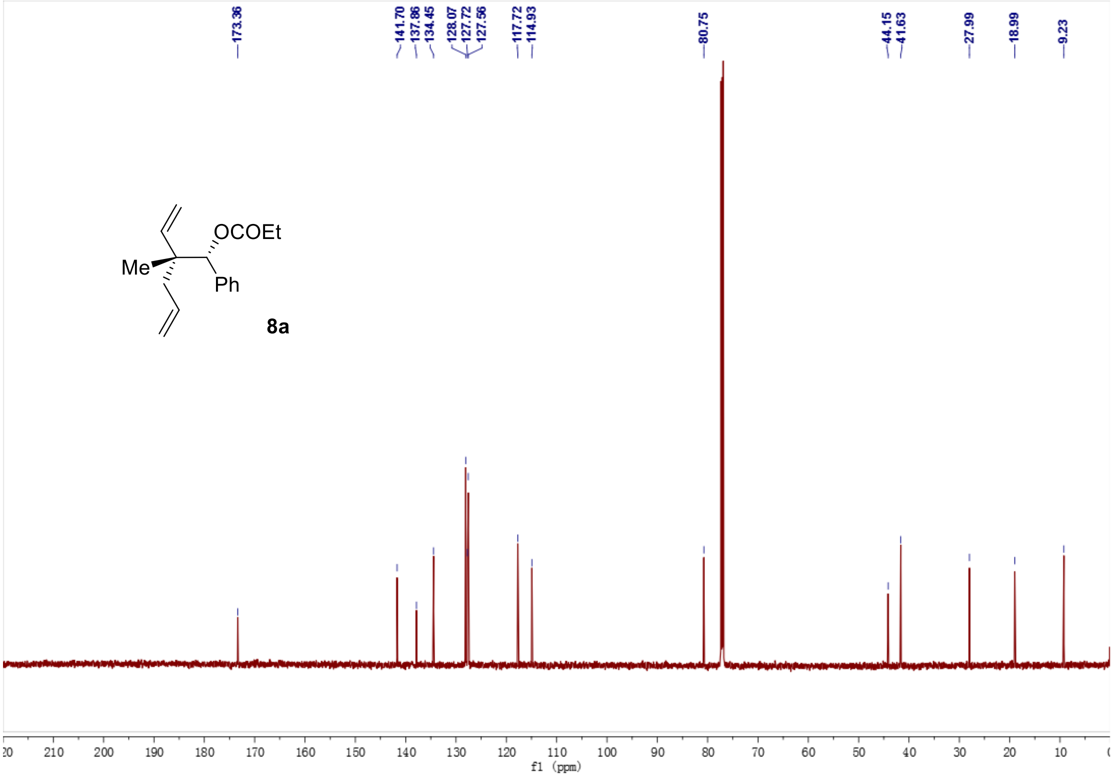

Supplementary Figure 256. ^{1}H NMR sepctra of (1*S*,2*S*)-2-ethynyl-2-methyl-1-phenylpent-4-en-1-ol (**7b**)

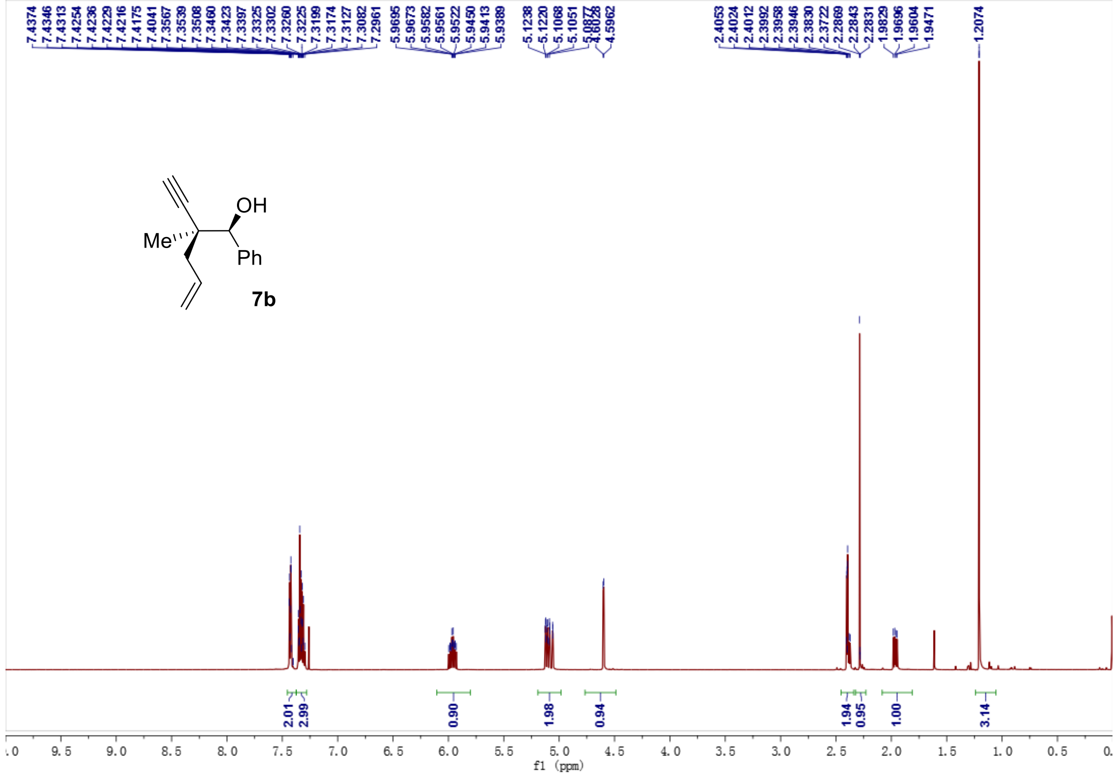

Supplementary Figure 257. ^{13}C NMR sepctra of (1*S*,2*S*)-2-ethynyl-2-methyl-1-phenylpent-4-en-1-ol (**7b**)

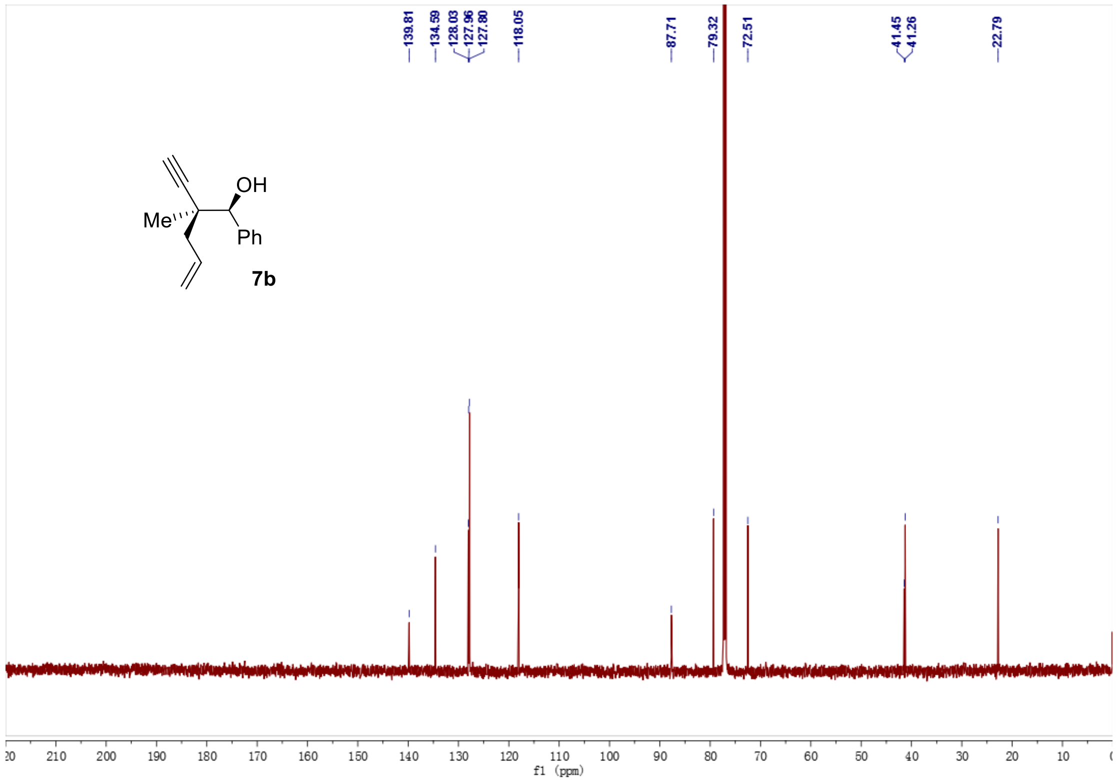

Supplementary Figure 258. ^{1}H NMR sepctra of (1*R*,2*R*)-2-ethynyl-2-methyl-1-phenylpent-4-en-1-yl propionate (**8b**)

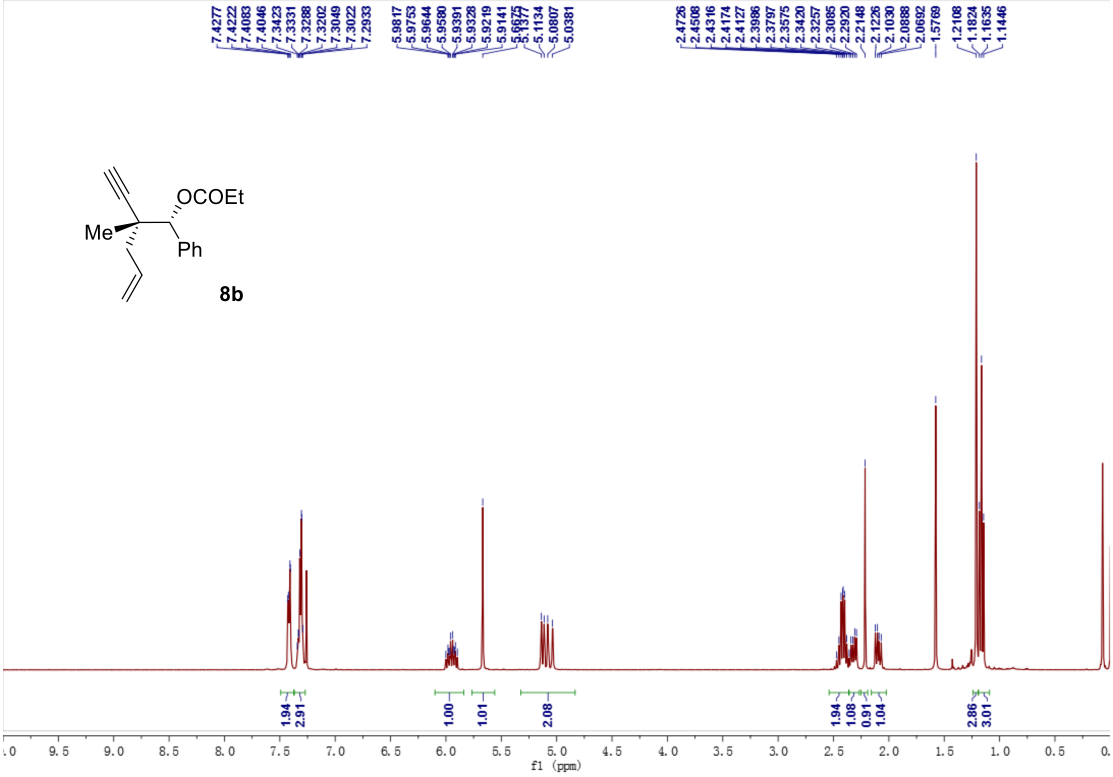

Supplementary Figure 259. ^{13}C NMR sepctra of (1*R*,2*R*)-2-ethynyl-2-methyl-1-phenylpent-4-en-1-yl propionate (**8b**)

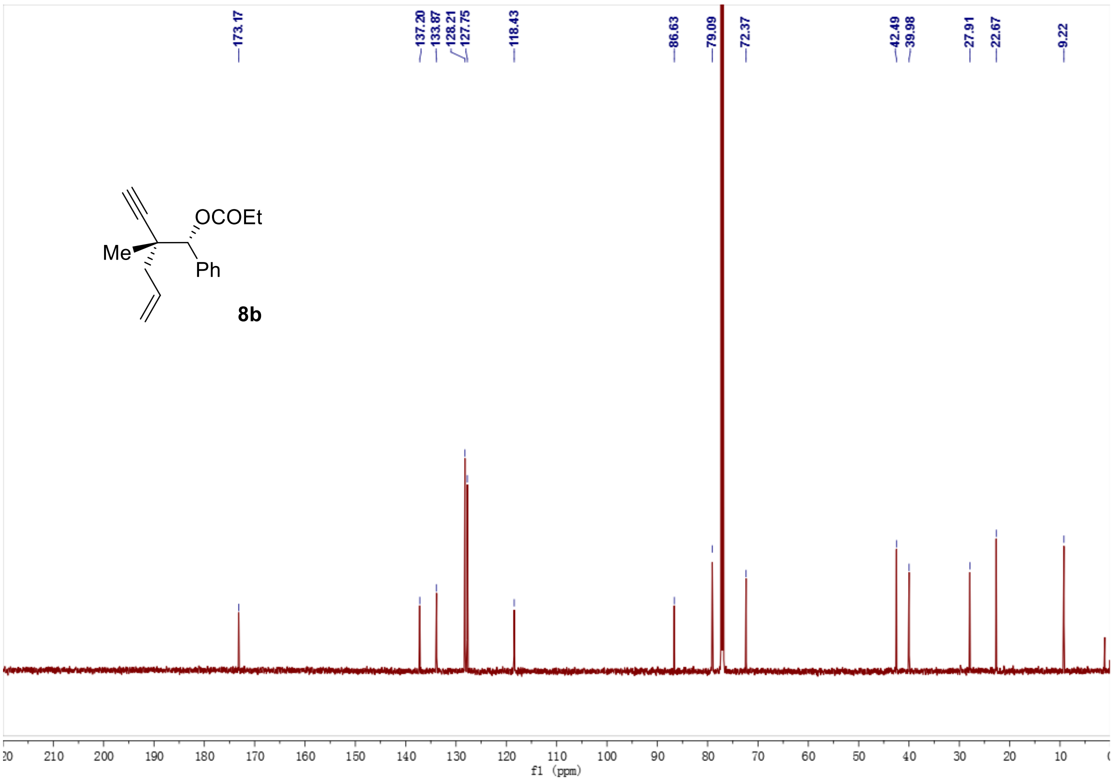

Supplementary Figure 260. ^{1}H NMR sepctra of (*S*)-2-benzyl-2-((*S*)-hydroxy(phenyl)methyl)butyl propionate (**7c**)

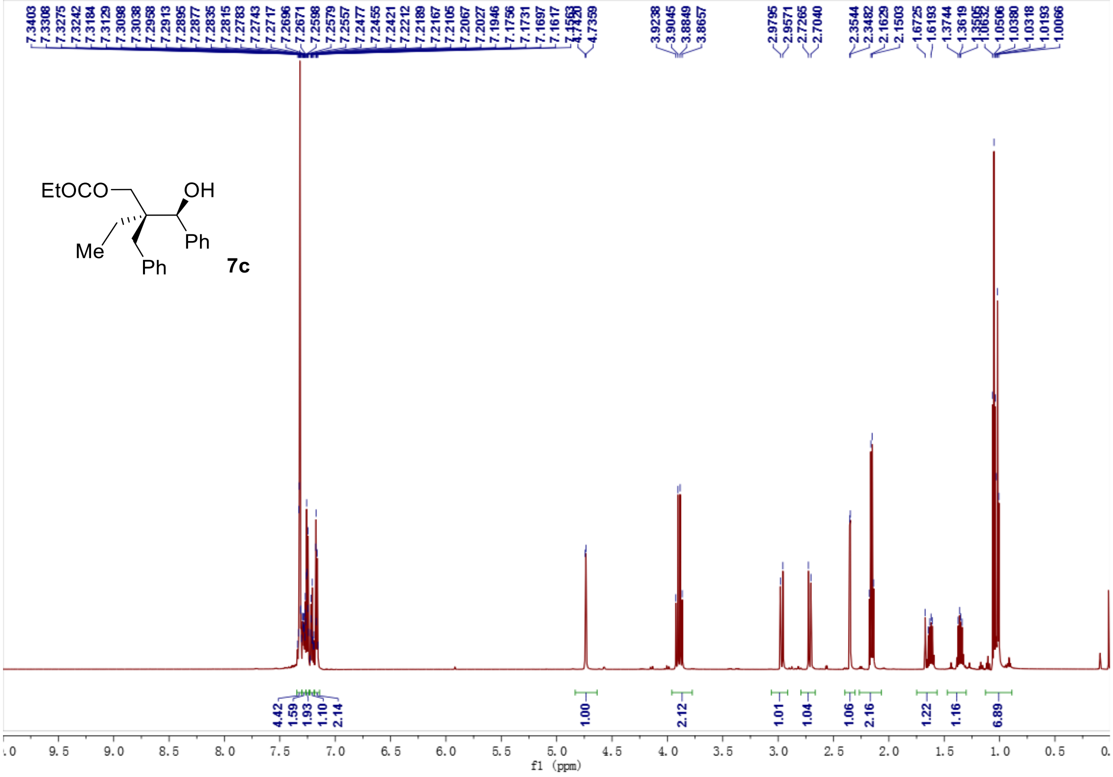

Supplementary Figure 261. ^{13}C NMR sepctra of (*S*)-2-benzyl-2-((*S*)-hydroxy(phenyl)methyl)butyl propionate (**7c**)

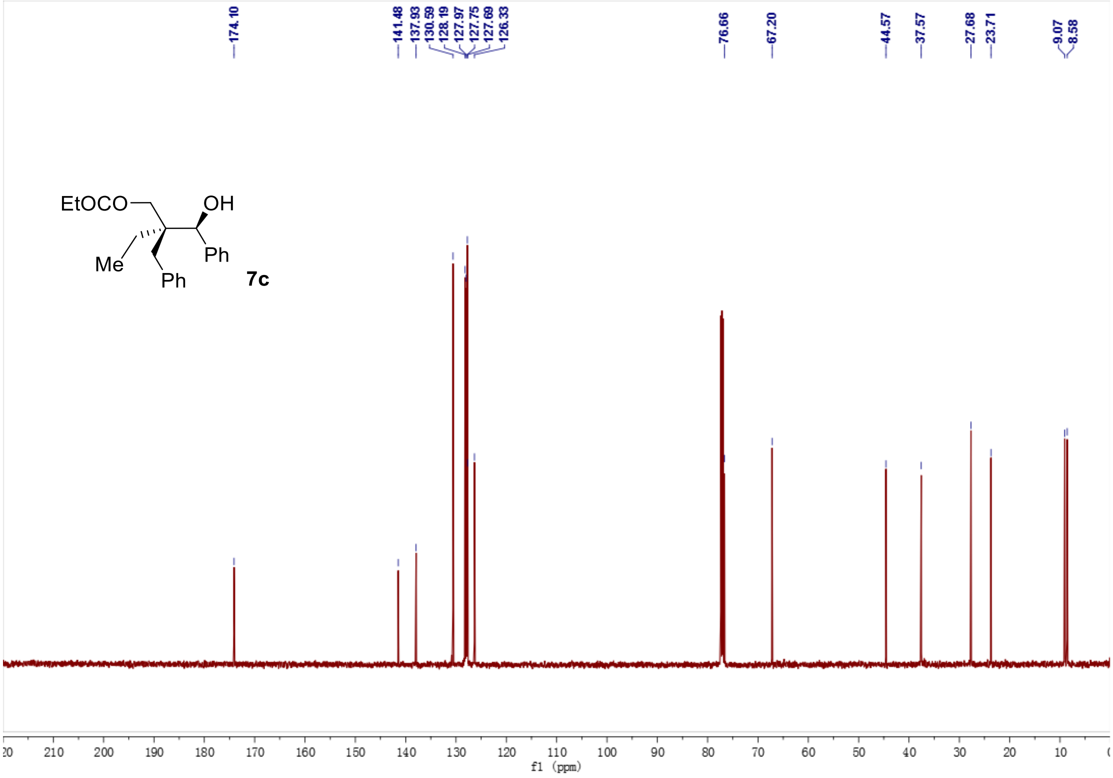

Supplementary Figure 262. ^{1}H NMR sepctra of (1*R*,2*R*)-2-benzyl-2-ethyl-1-phenylpropane-1,3-diyl dipropionate (**8c**)

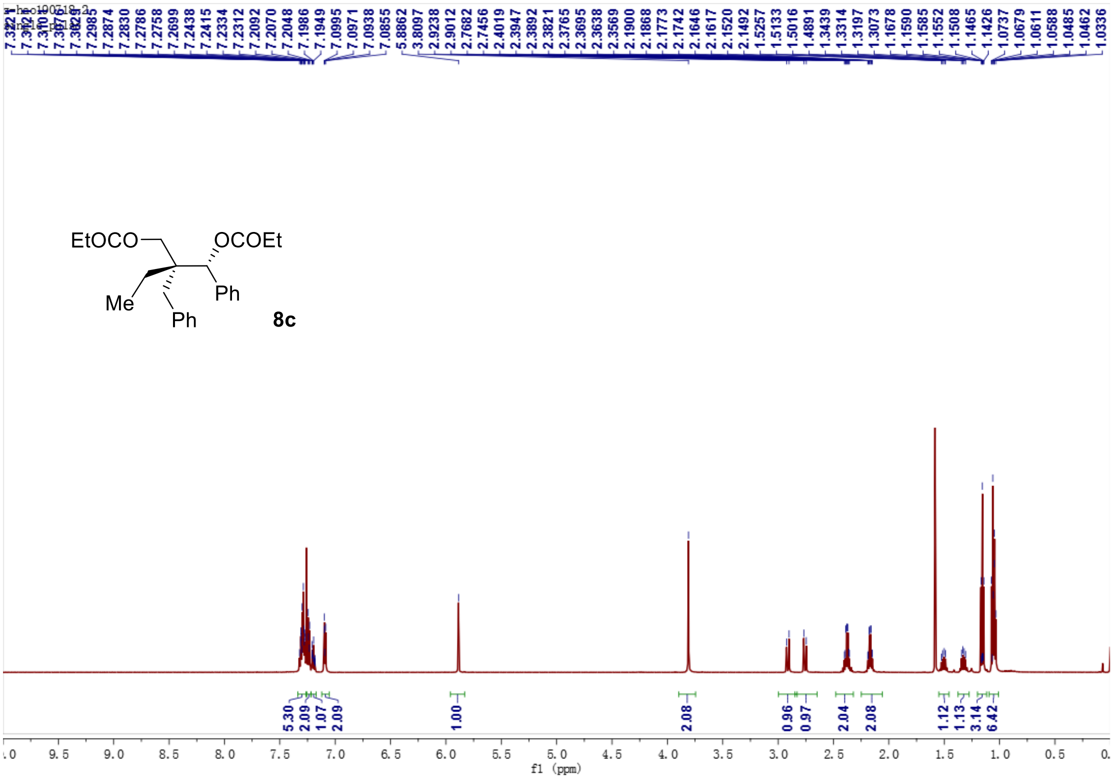

Supplementary Figure 263. ^{13}C NMR sepctra of (1*R*,2*R*)-2-benzyl-2-ethyl-1-phenylpropane-1,3-diyl dipropionate (**8c**)

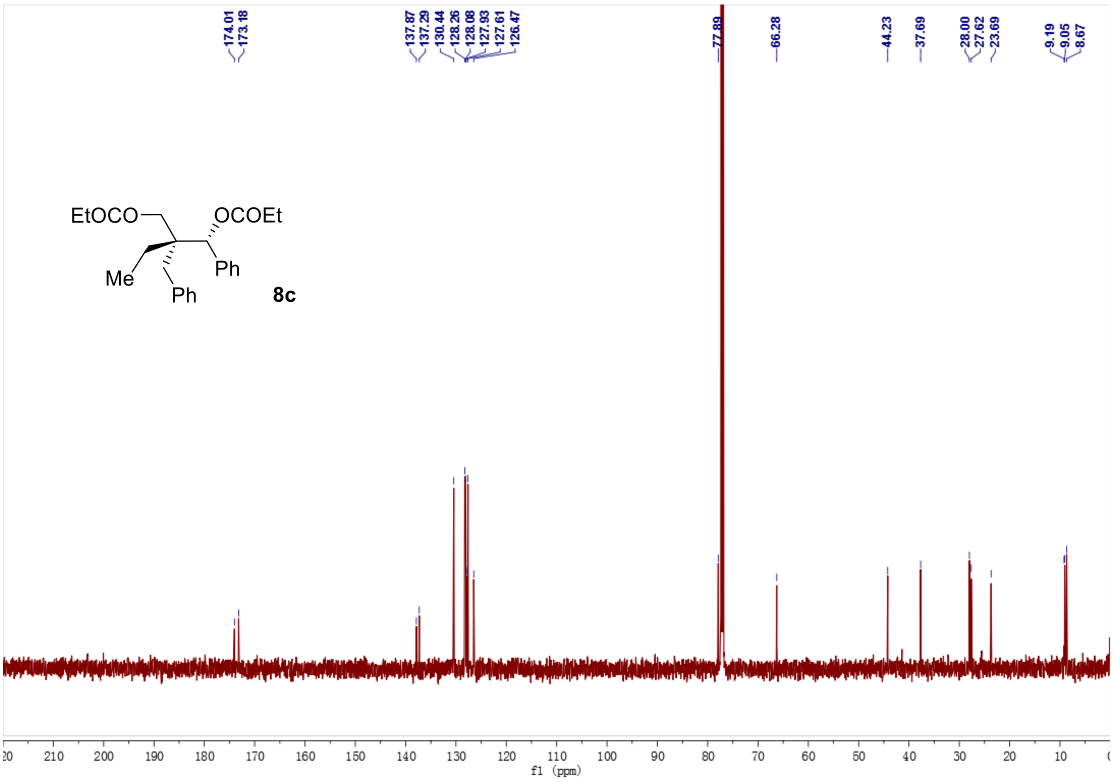

Supplementary Figure 264. ^{1}H NMR sepctra of (*S*)-2-(4-fluorobenzyl)-2-((*S*)-hydroxy(phenyl)methyl)butyl propionate (**7d**)

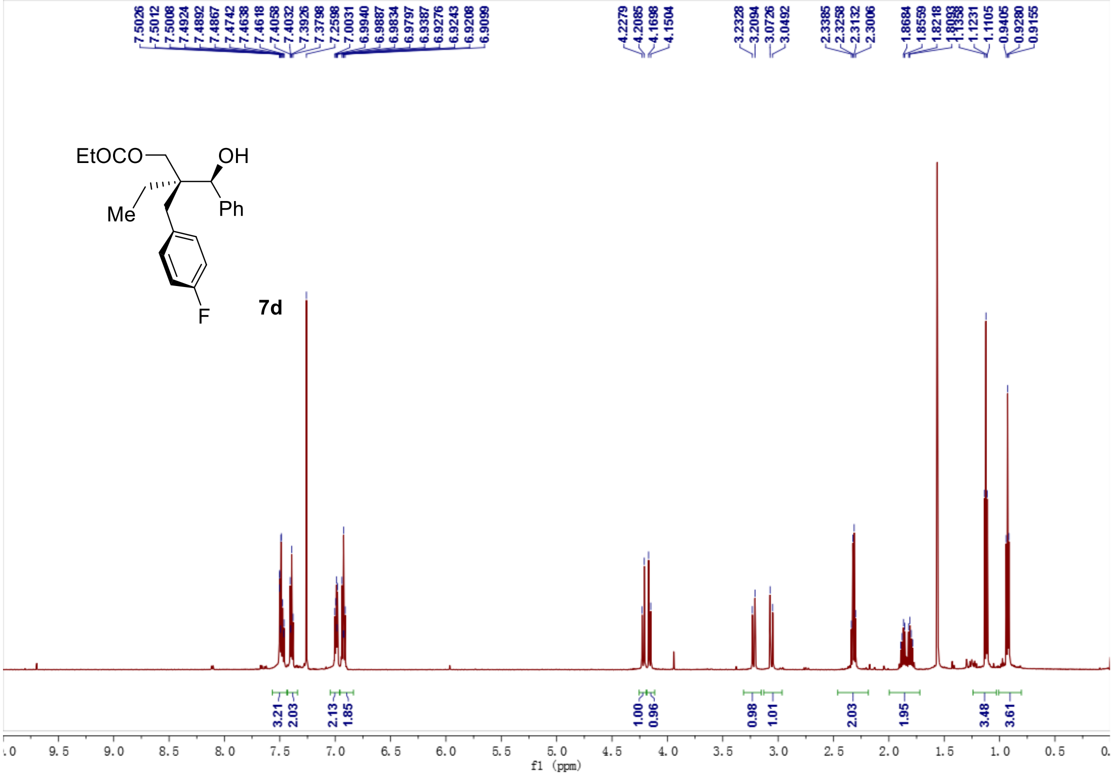

Supplementary Figure 265. ^{13}C NMR sepctra of (*S*)-2-(4-fluorobenzyl)-2-((*S*)-hydroxy(phenyl)methyl)butyl propionate (**7d**)

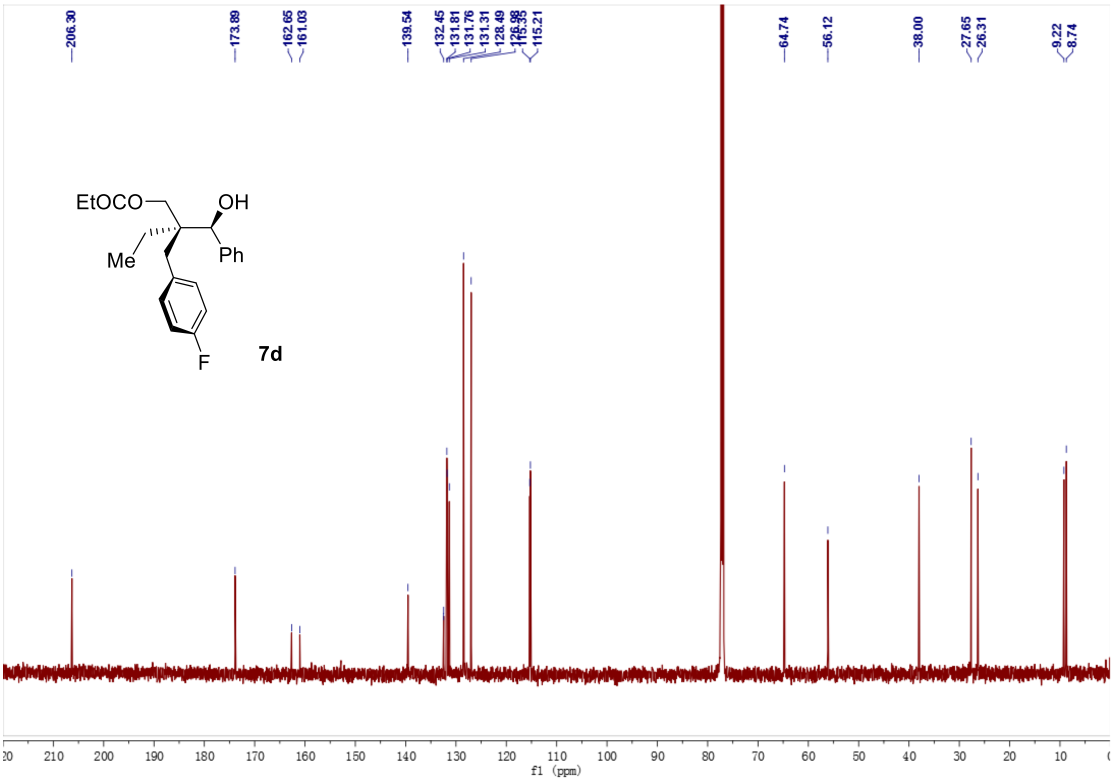

Supplementary Figure 266. ^{1}H NMR sepctra of (1*R*,2*R*)-2-ethyl-2-(4-fluorobenzyl)-1-phenylpropane-1,3-diyl dipropionate (**8d**)

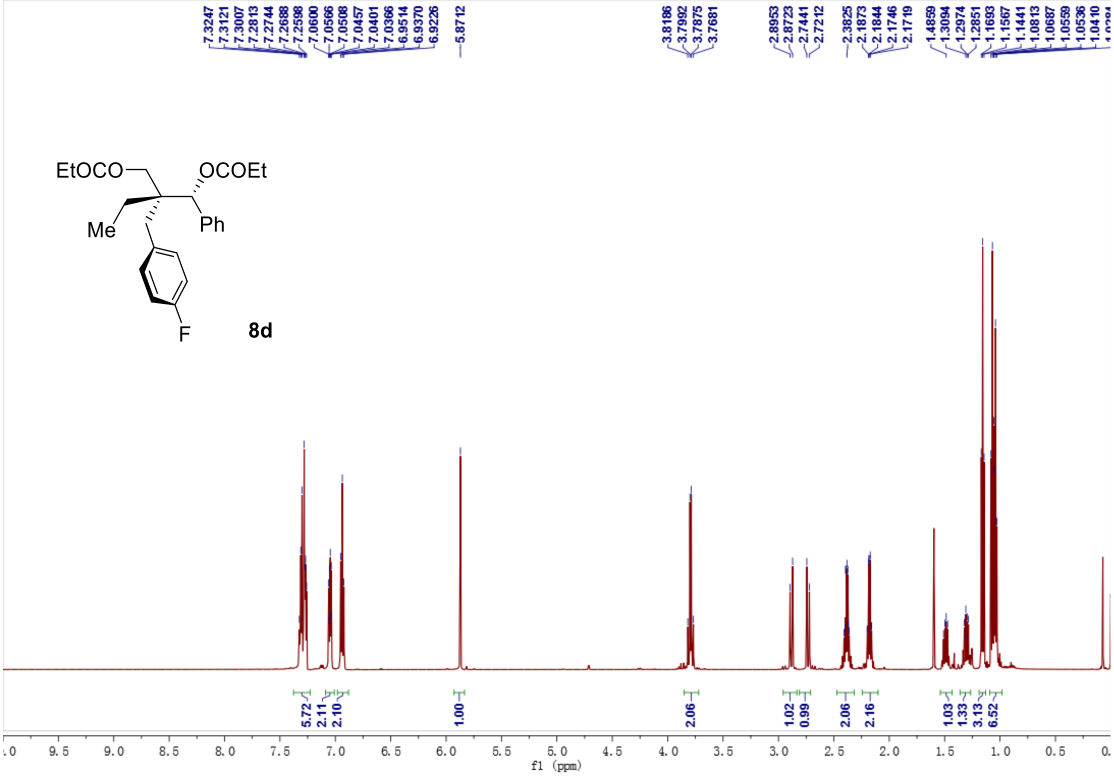

Supplementary Figure 267. ^{13}C NMR sepctra of (1*R*,2*R*)-2-ethyl-2-(4-fluorobenzyl)-1-phenylpropane-1,3-diyl dipropionate (**8d**)

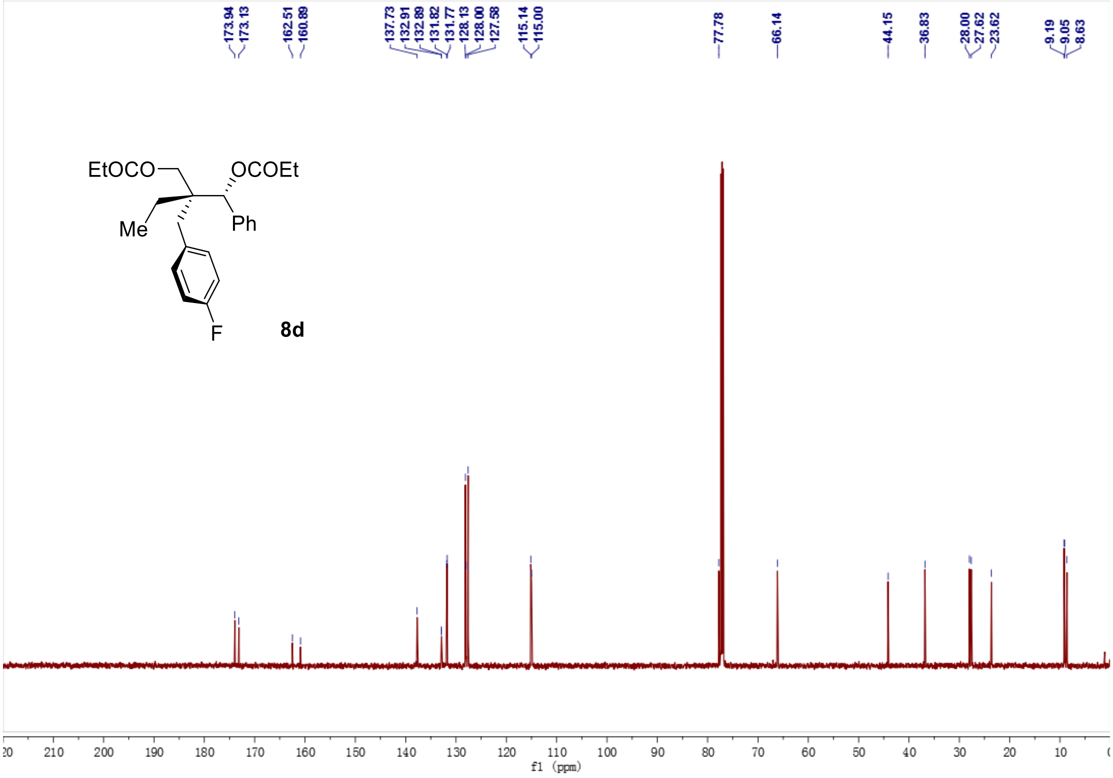

Supplementary Figure 268. ^{1}H NMR sepctra of (1*R*,2*R*)-2-methyl-1-phenyl-2-vinylpent-4-yn-1-ol (**7e**)

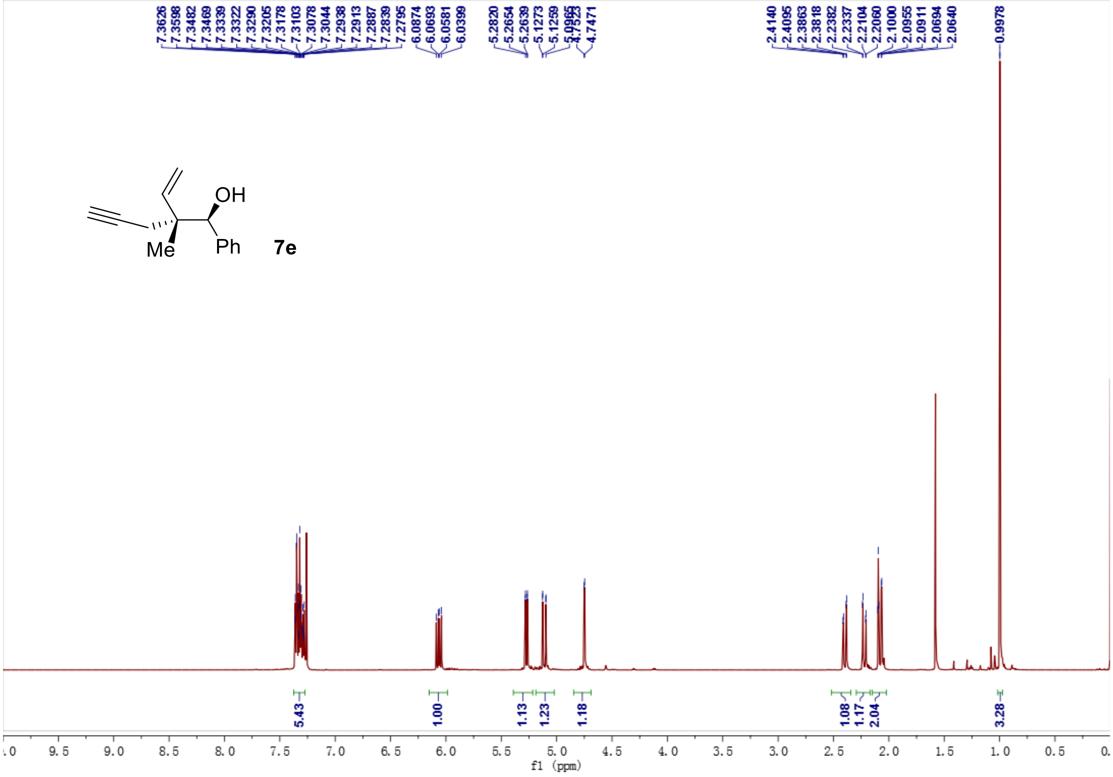

Supplementary Figure 269. ^{13}C NMR sepctra of (1*R*,2*R*)-2-methyl-1-phenyl-2-vinylpent-4-yn-1-ol (**7e**)

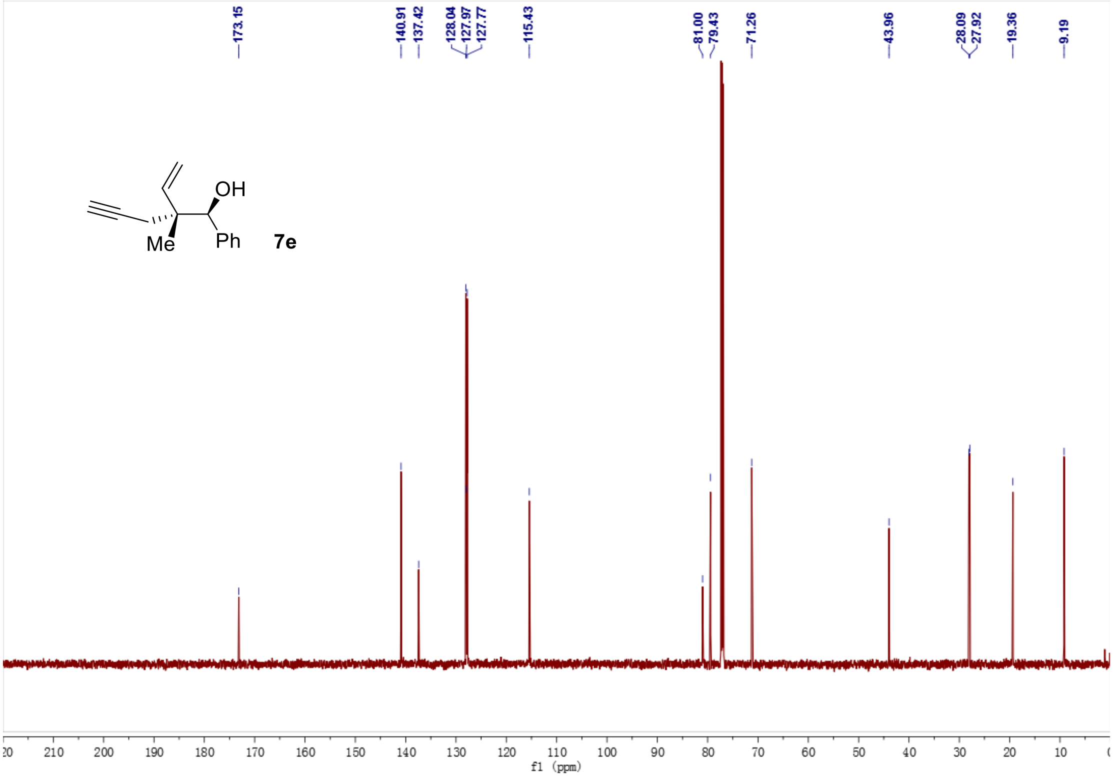

Supplementary Figure 270. ^{1}H NMR sepctra of (1*S*,2*S*)-2-methyl-1-phenyl-2-vinylpent-4-yn-1-yl propionate (**8e**)

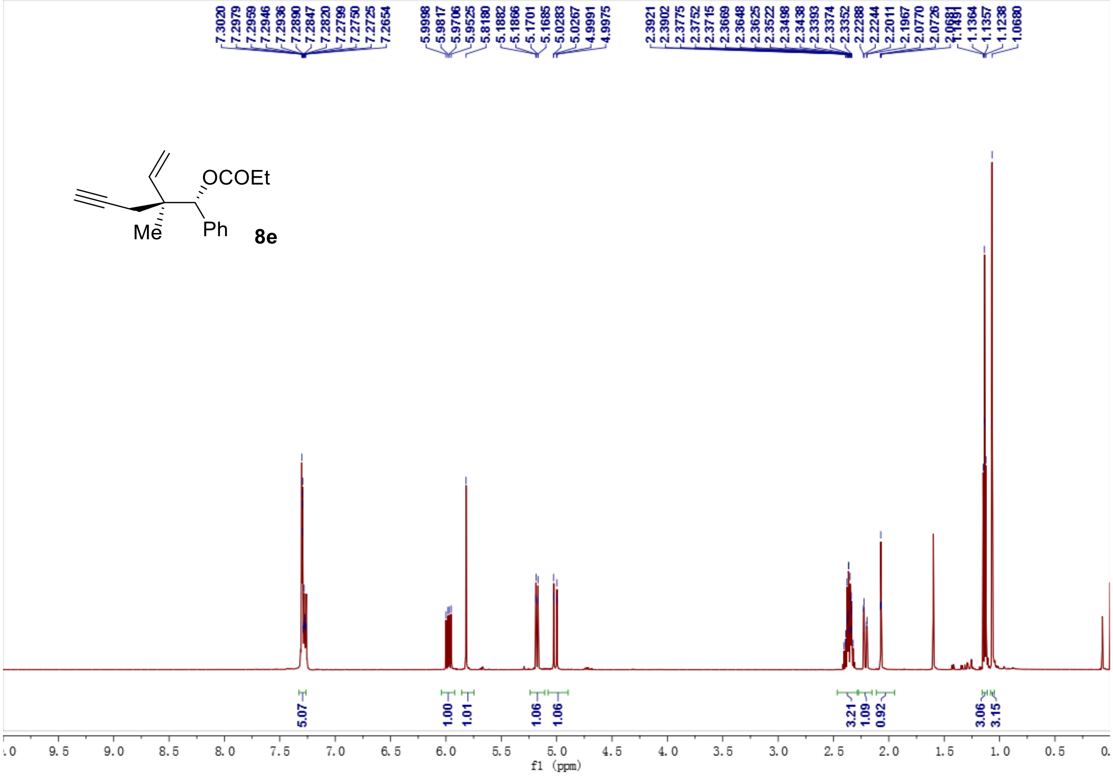

Supplementary Figure 271. ^{13}C NMR sepctra of (1*S*,2*S*)-2-methyl-1-phenyl-2-vinylpent-4-yn-1-yl propionate (**8e**)

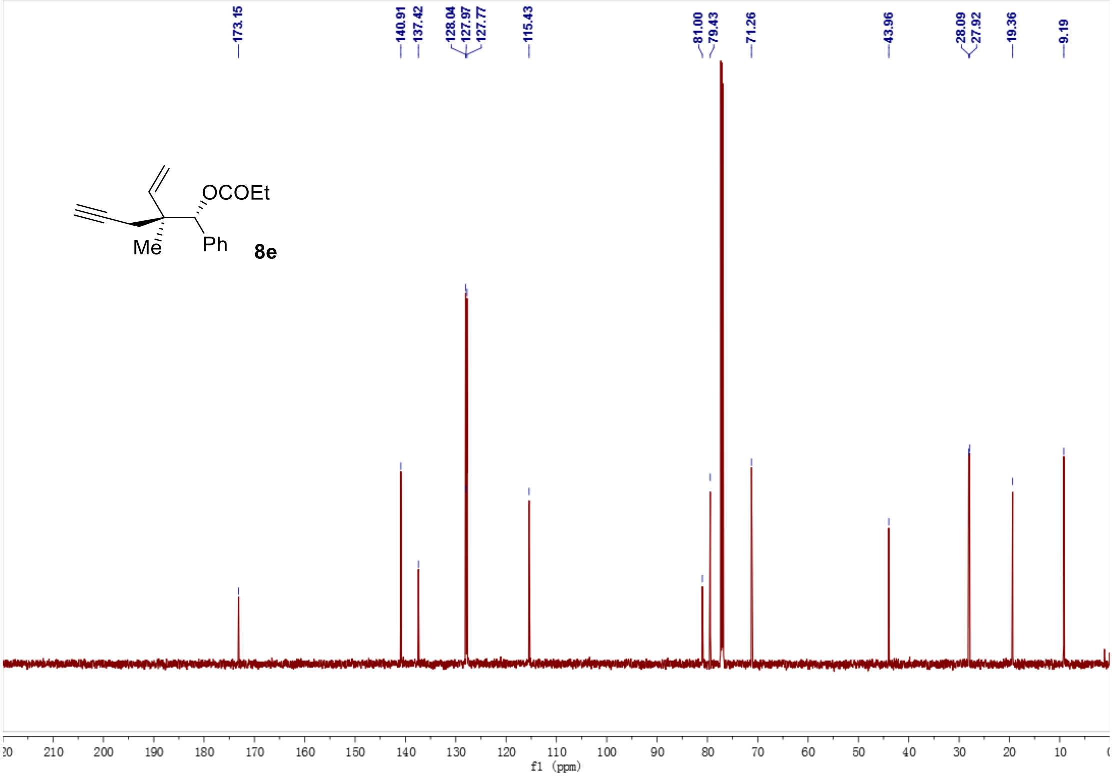

Supplementary Figure 272. ^{1}H NMR sepctra of (1*S*,2*S*)-2-(methoxymethyl)-2-methyl-1-phenylpent-4-en-1-ol (**7f**)

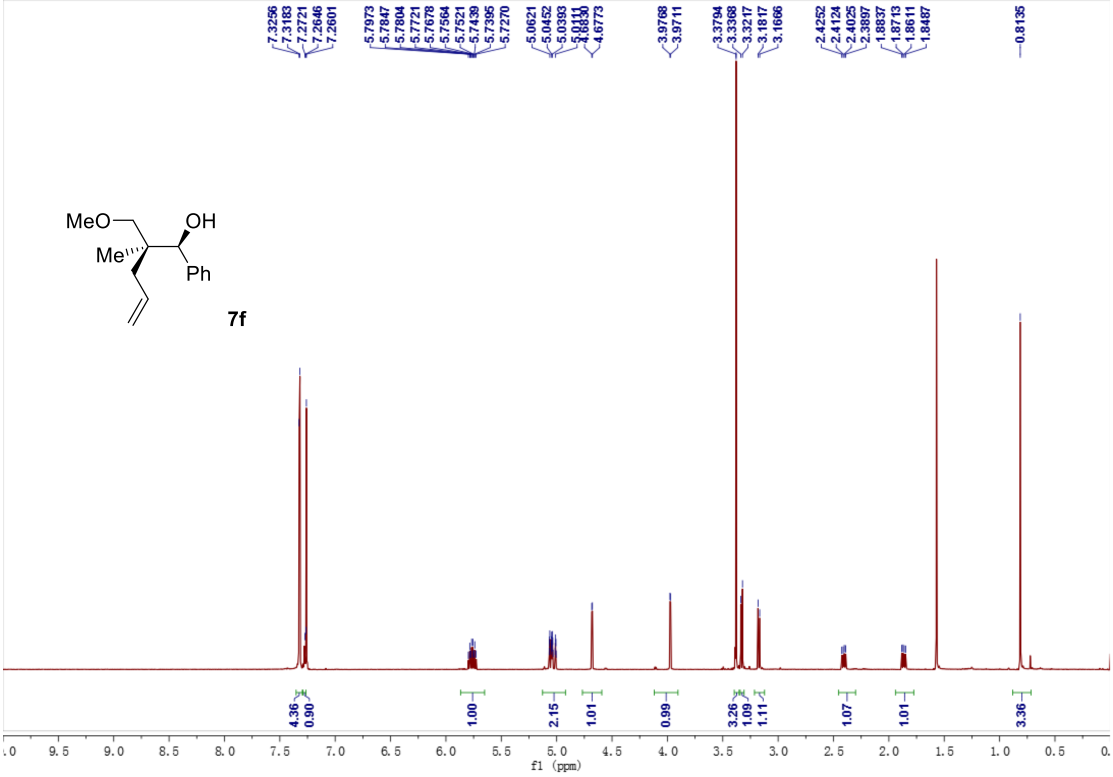

Supplementary Figure 273. ^{13}C NMR sepctra of (1*S*,2*S*)-2-(methoxymethyl)-2-methyl-1-phenylpent-4-en-1-ol (**7f**)

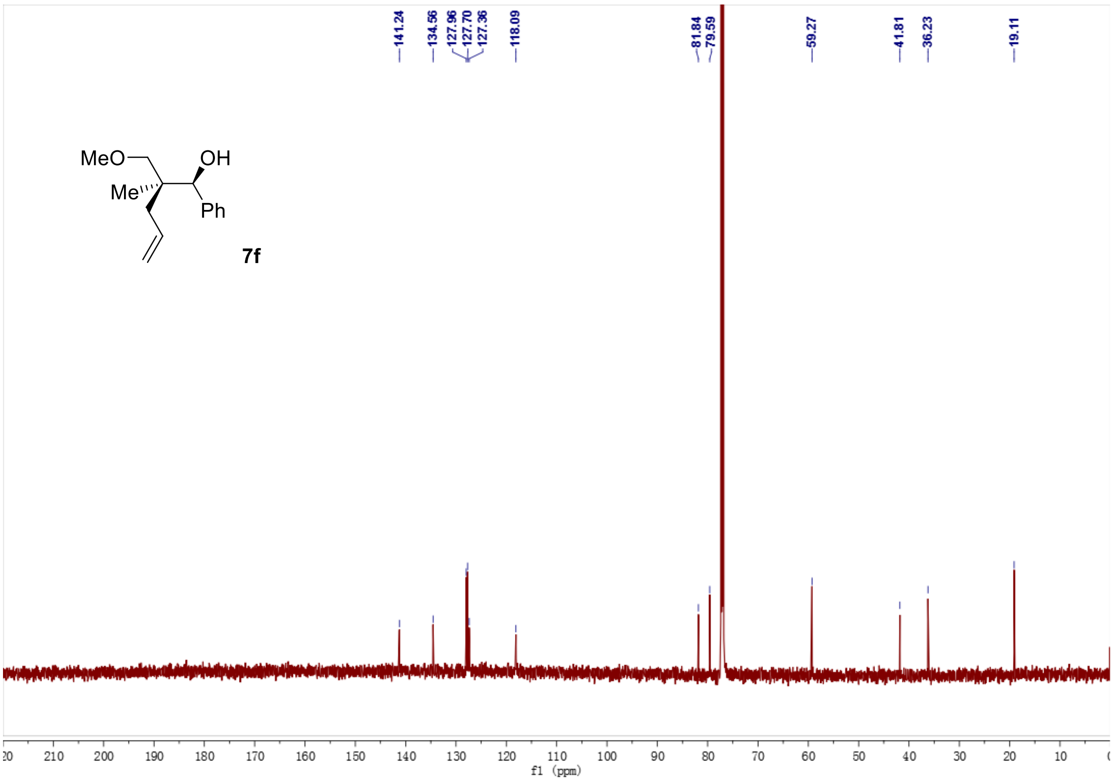

Supplementary Figure 274. ^{1}H NMR sepctra of (1*R*,2*R*)-2-(methoxymethyl)-2-methyl-1-phenylpent-4-en-1-yl propionate (**8f**)

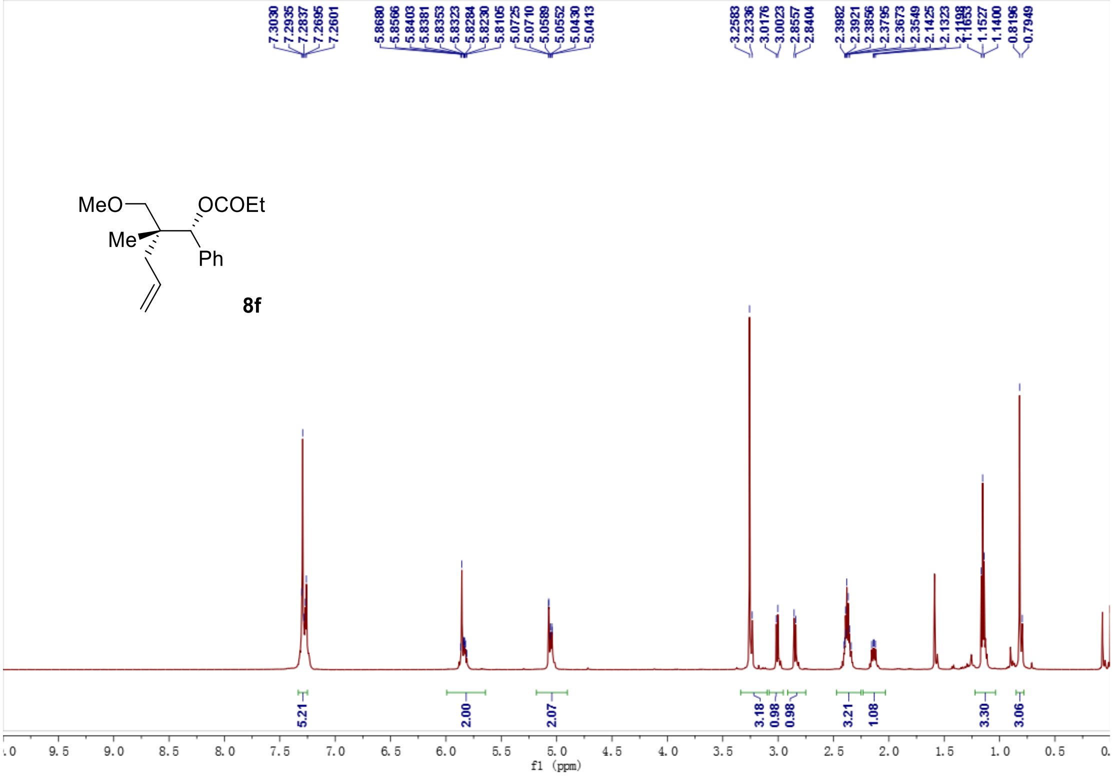

Supplementary Figure 275. ^{13}C NMR sepctra of (1*R*,2*R*)-2-(methoxymethyl)-2-methyl-1-phenylpent-4-en-1-yl propionate (**8f**)

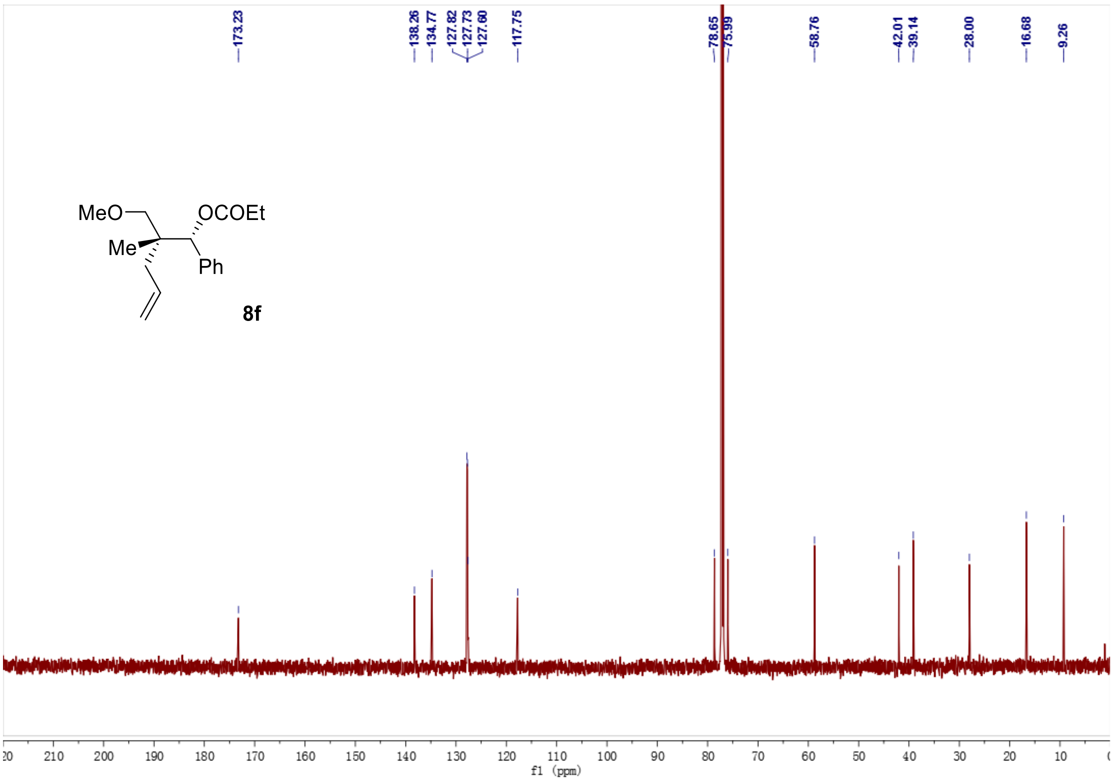

Supplementary Figure 276. ^{1}H NMR sepctra of (2*S*,3*S*)-3-hydroxy-2-methyl-2,3-diphenylpropyl propionate (**7g**)

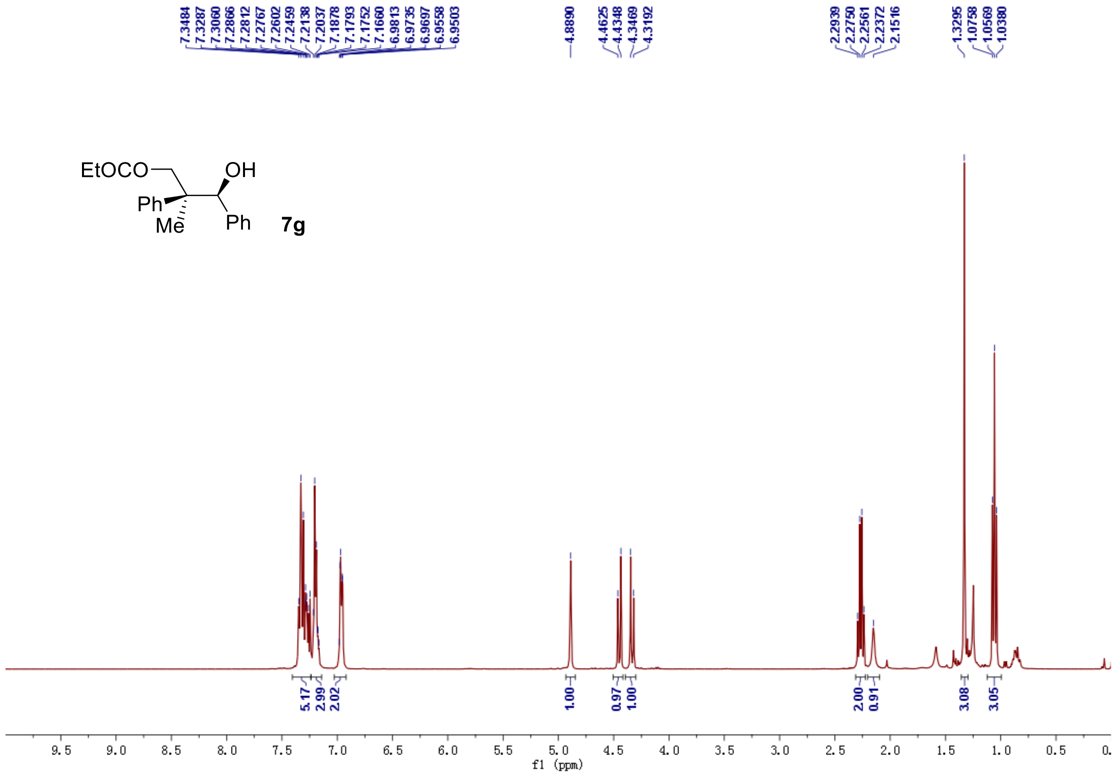

Supplementary Figure 277. ^{13}C NMR sepctra of (2*S*,3*S*)-3-hydroxy-2-methyl-2,3-diphenylpropyl propionate (**7g**)

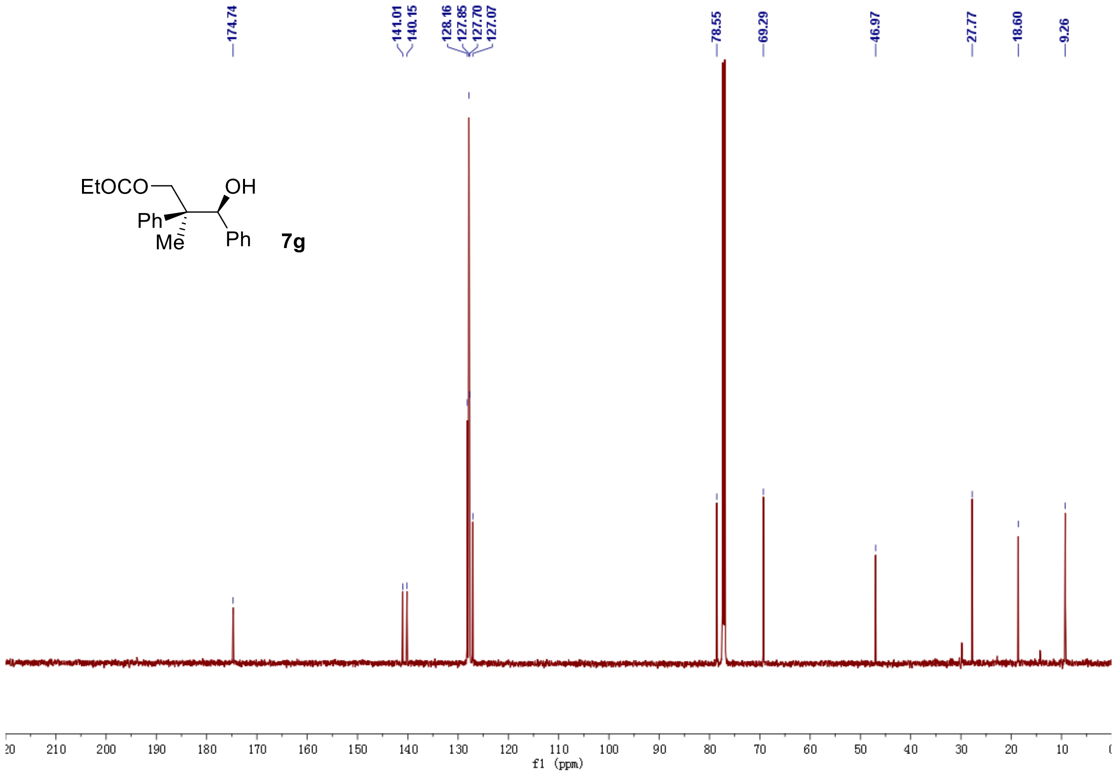

Supplementary Figure 278. ^{1}H NMR sepctra of (1*R*,2*R*)-2-methyl-1,2-diphenylpropane-1,3-diyl dipropionate (**8g**)

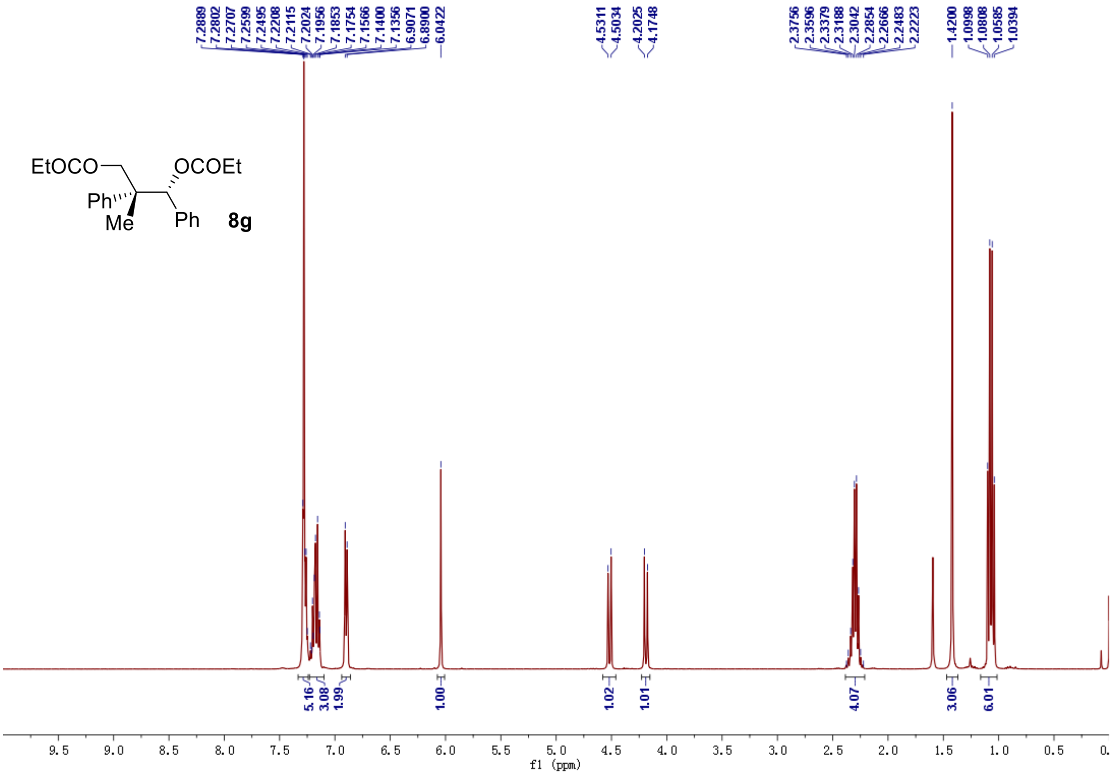

Supplementary Figure 279. ^{13}C NMR sepctra of (1*R*,2*R*)-2-methyl-1,2-diphenylpropane-1,3-diyl dipropionate (**8g**)

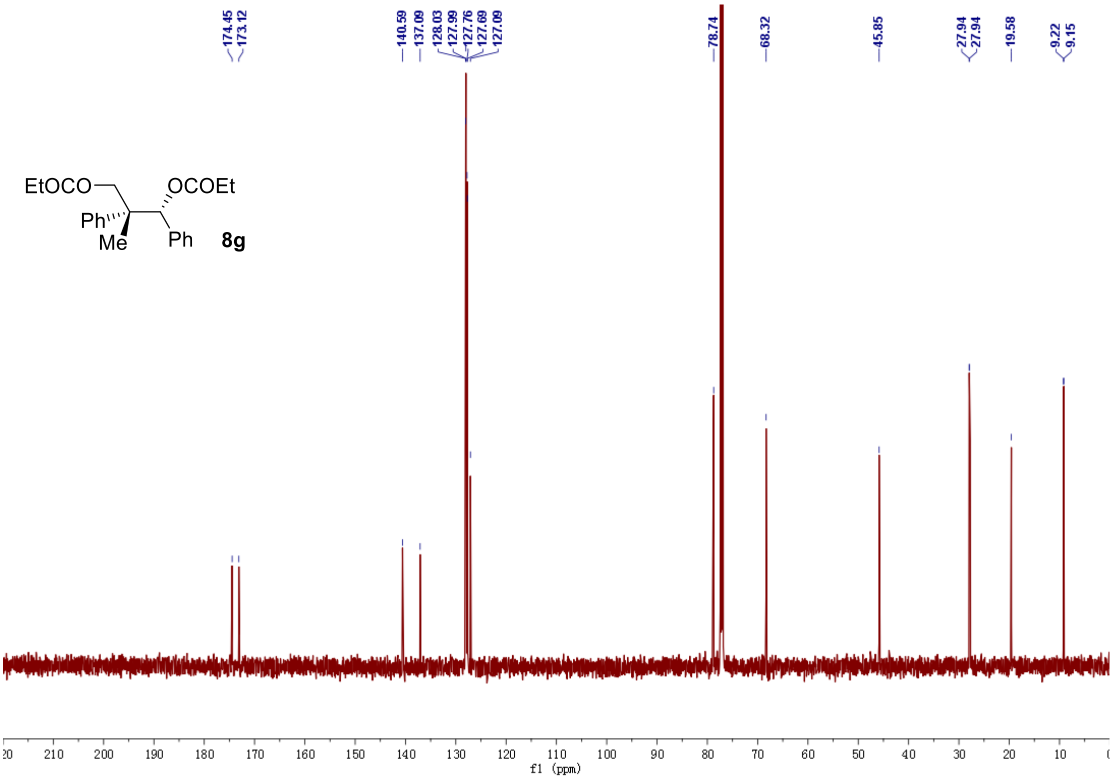

Supplementary Figure 280. ^{1}H NMR sepctra of (1*S*,2*S*)-2-methyl-1,2-diphenylpent-4-en-1-ol (**7h**)

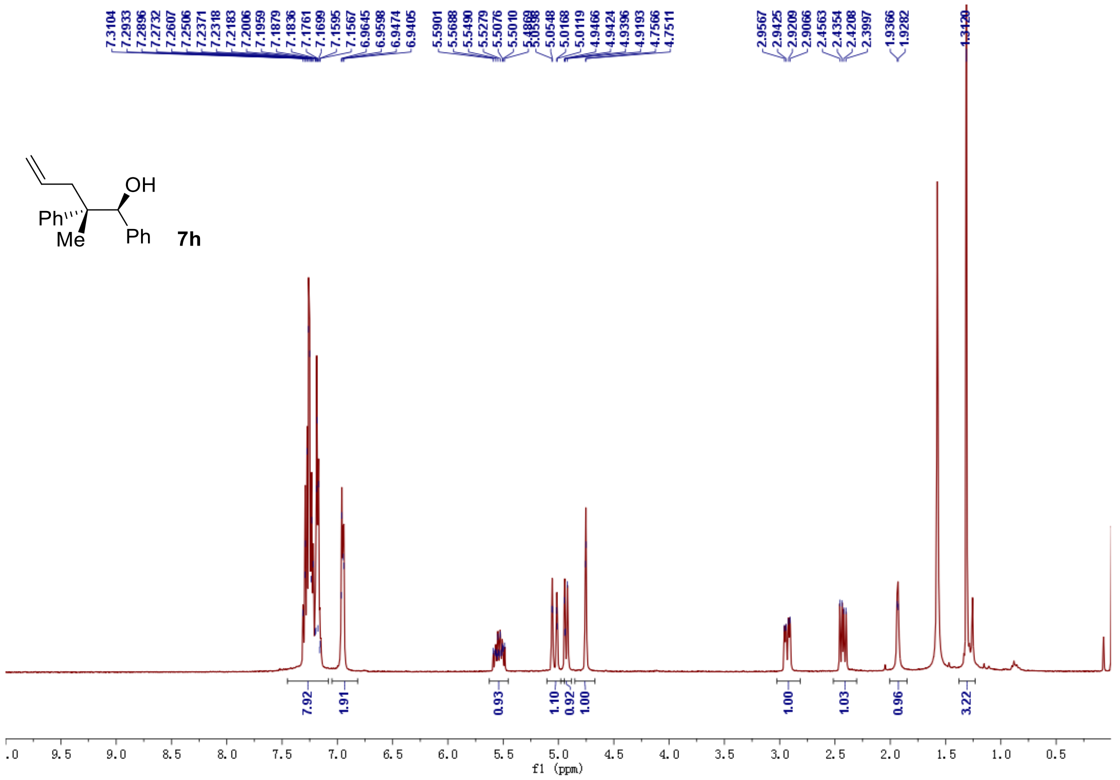

Supplementary Figure 281. ^{13}C NMR sepctra of (1*S*,2*S*)-2-methyl-1,2-diphenylpent-4-en-1-ol (**7h**)

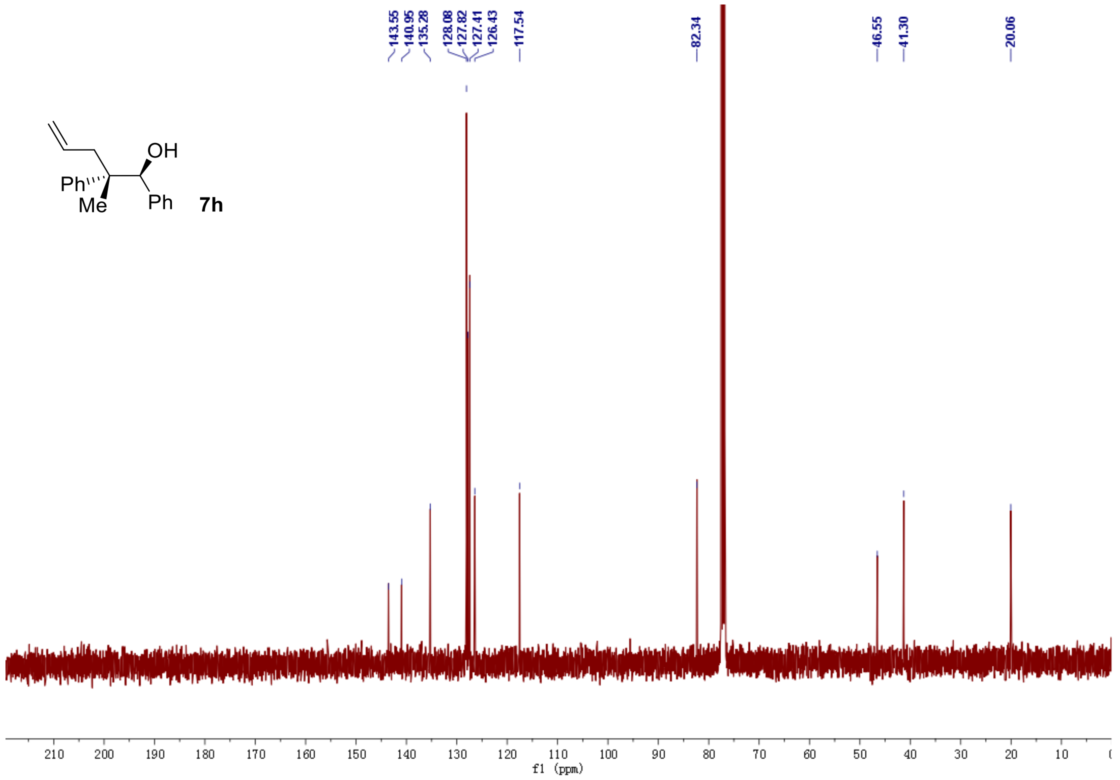

Supplementary Figure 282. ^1H NMR sepctra of (1*R*,2*R*)-2-methyl-1,2-diphenylpent-4-en-1-yl propionate (**8h**)

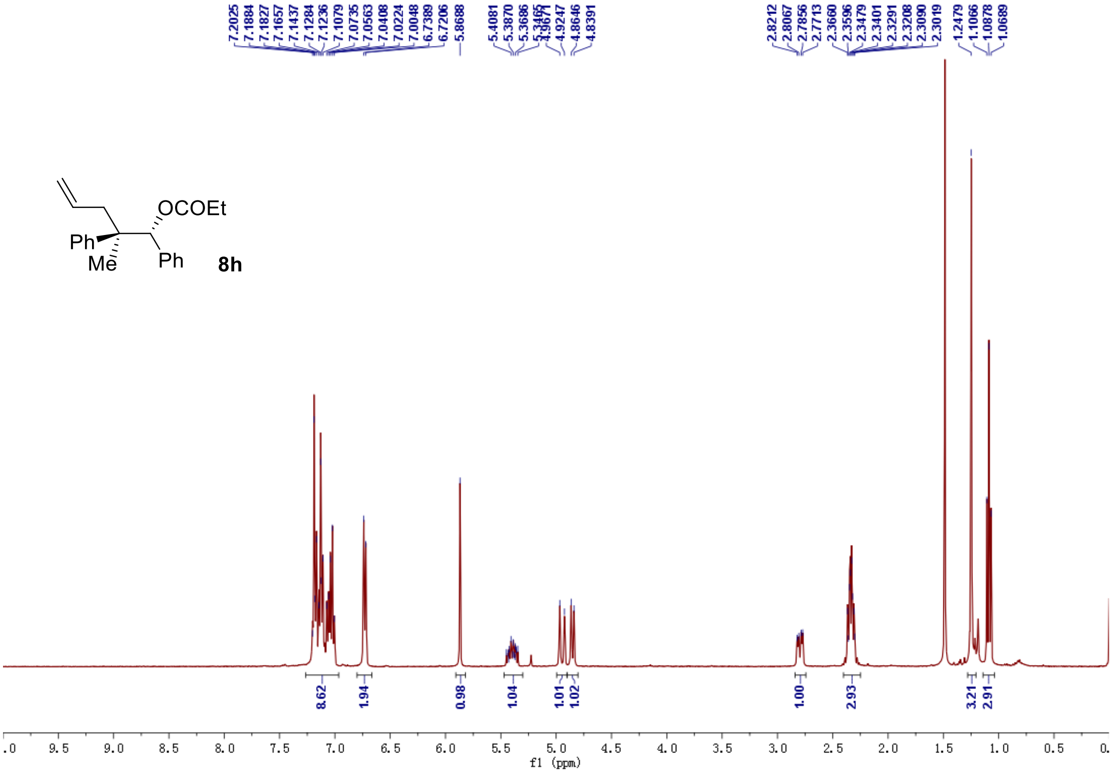

Supplementary Figure 283. ^{13}C NMR sepctra of (1*R*,2*R*)-2-methyl-1,2-diphenylpent-4-en-1-yl propionate (**8h**)

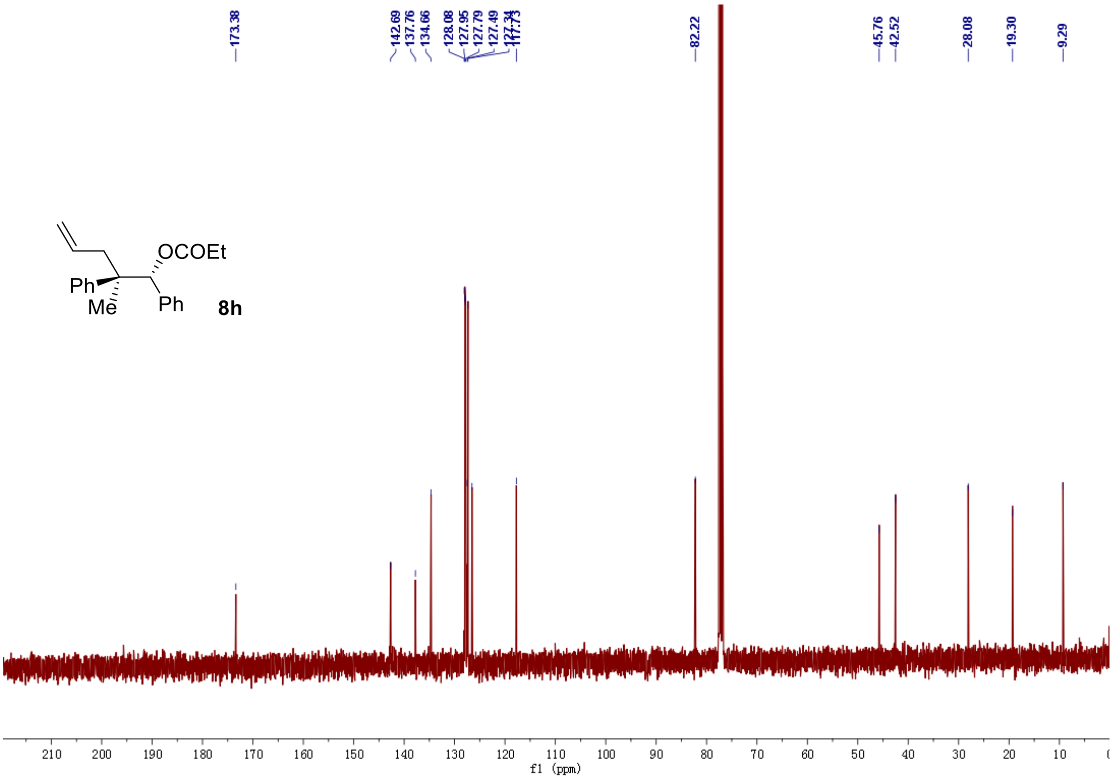

Supplementary Figure 284. ^{1}H NMR sepctra of (*R*)-((1*R*,2*S*)-1-methyl-2-phenylcyclopropyl)(phenyl)methanol (**7i**)

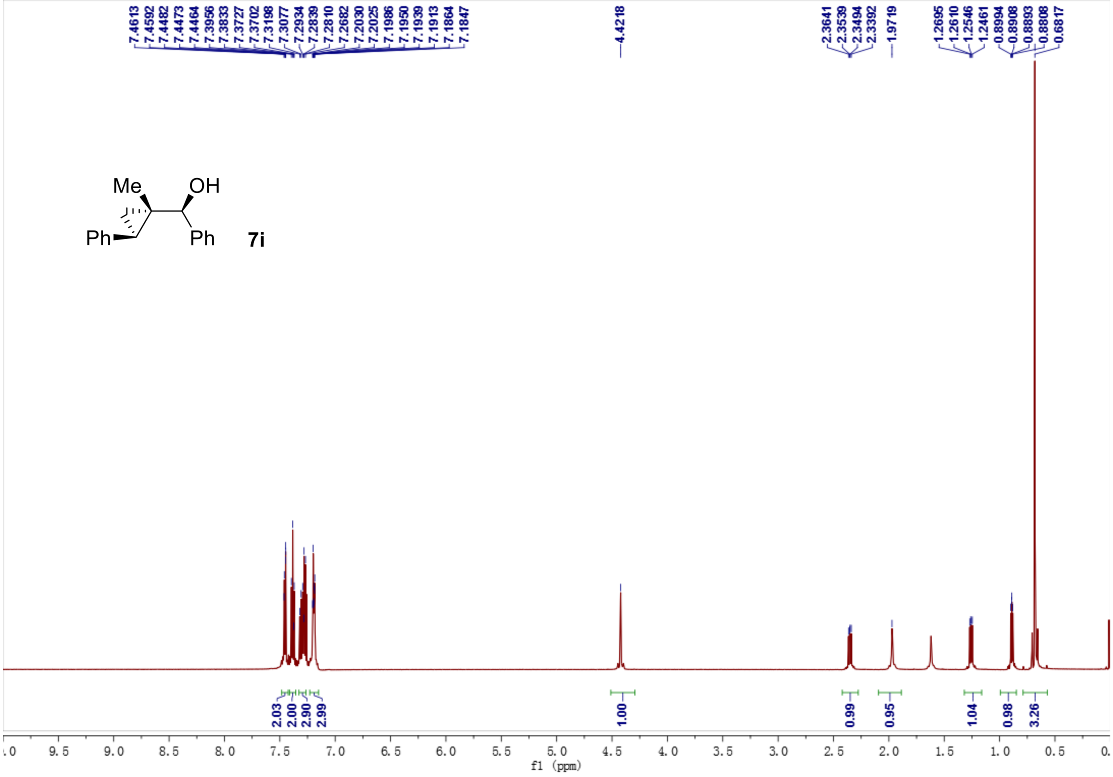

Supplementary Figure 285. ^{13}C NMR sepctra of (*R*)-((1*R*,2*S*)-1-methyl-2-phenylcyclopropyl)(phenyl)methanol (**7i**)

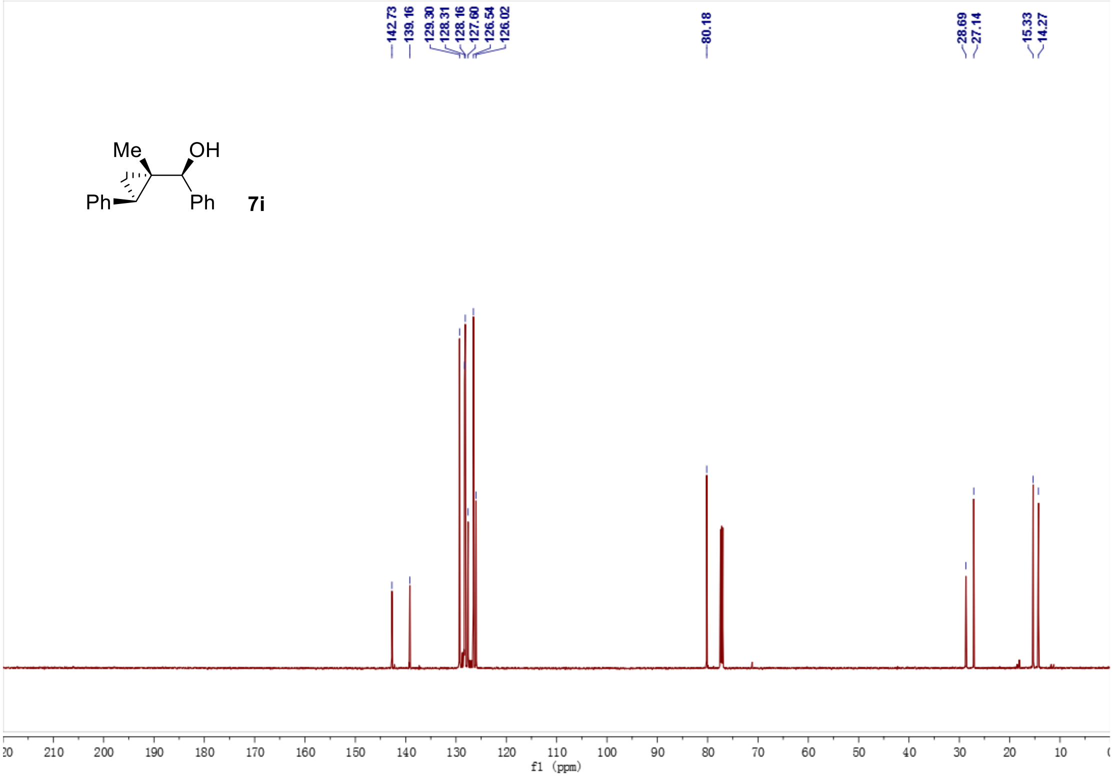

Supplementary Figure 286. ^{1}H NMR sepctra of (*S*)-((1*S*,2*R*)-1-methyl-2-phenylcyclopropyl)(phenyl)methyl propionate (**8i**)

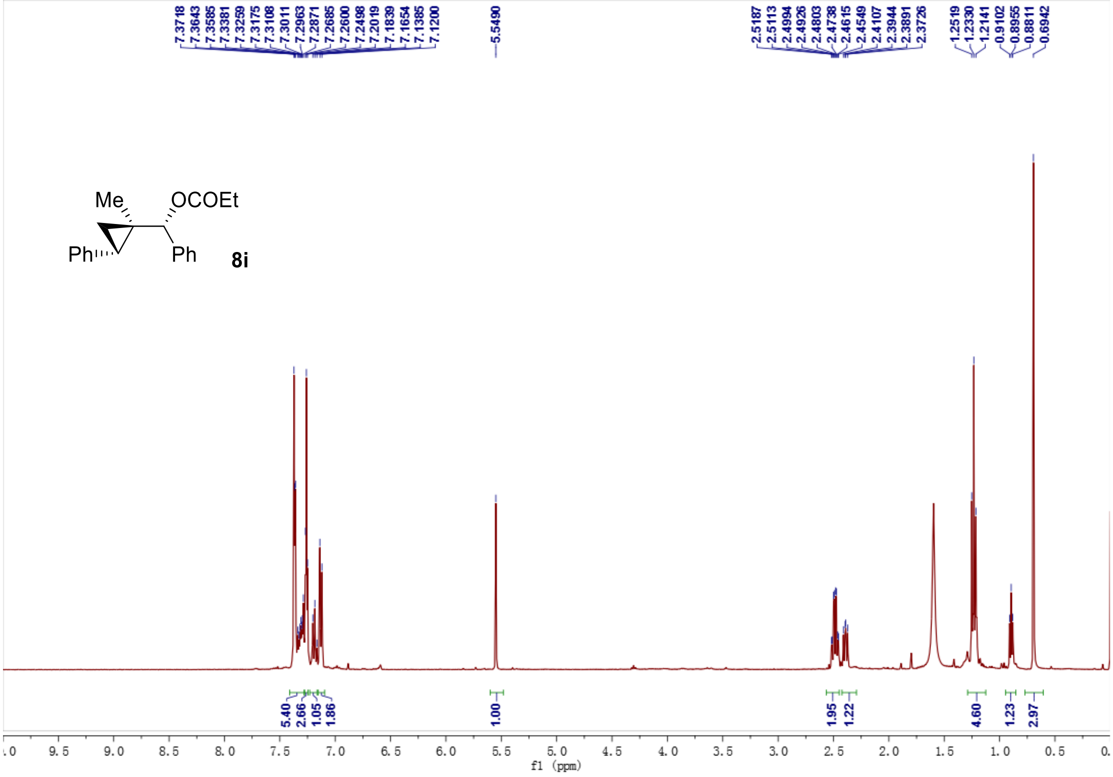

Supplementary Figure 287. ^{13}C NMR sepctra of (*S*)-((1*S*,2*R*)-1-methyl-2-phenylcyclopropyl)(phenyl)methyl propionate (**8i**)

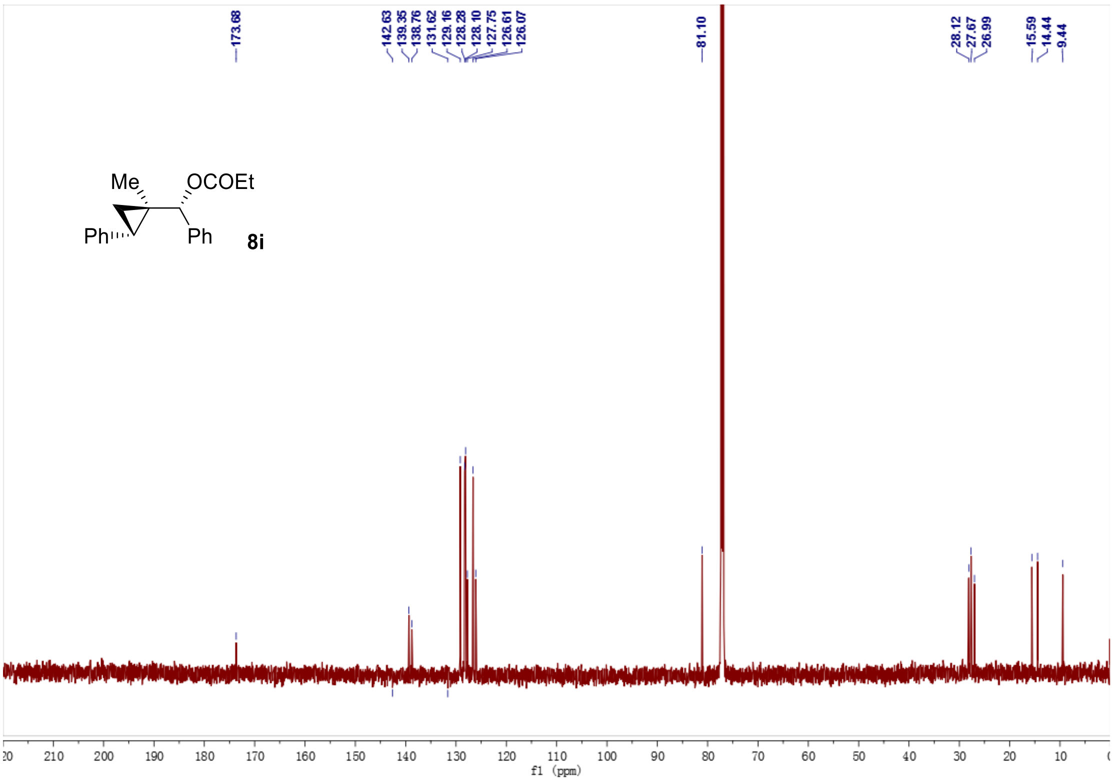

Supplementary Figure 288. ^{1}H NMR sepctra of (*R*)-((1*R*,2*S*)-1-methyl-2-phenylcyclopropyl)(phenyl)methanol (**7j**)

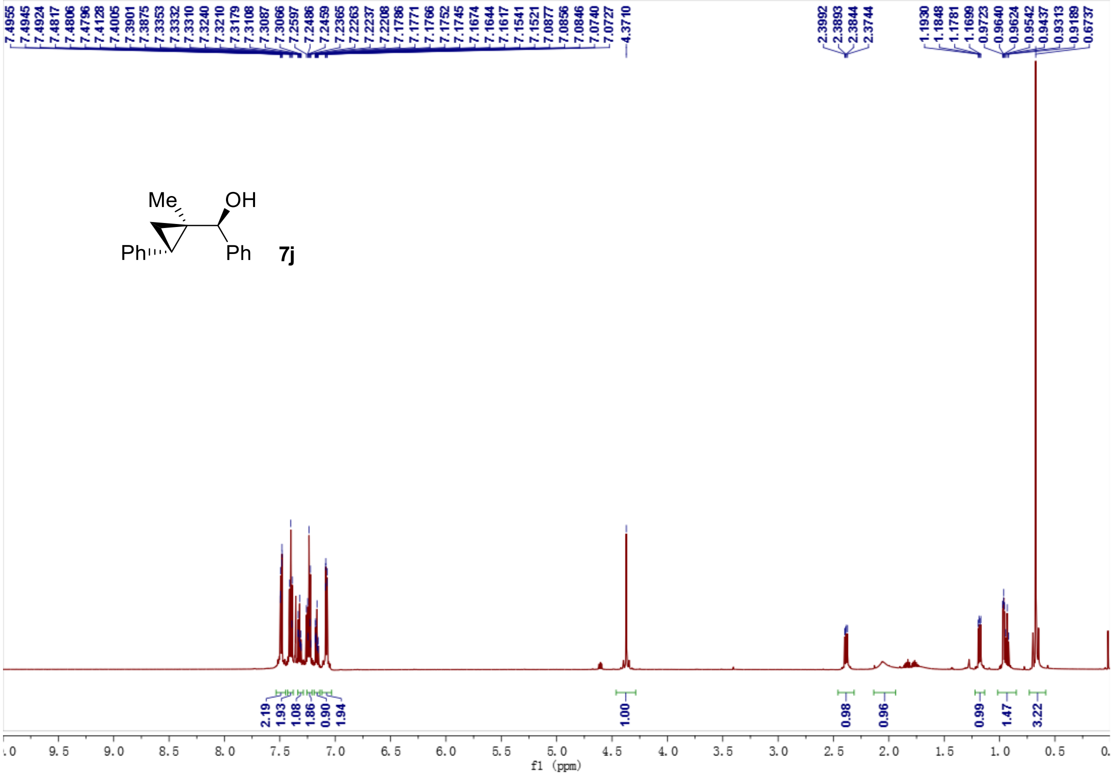

Supplementary Figure 289. ^{13}C NMR sepctra of (*R*)-((1*R*,2*S*)-1-methyl-2-phenylcyclopropyl)(phenyl)methanol (**7j**)

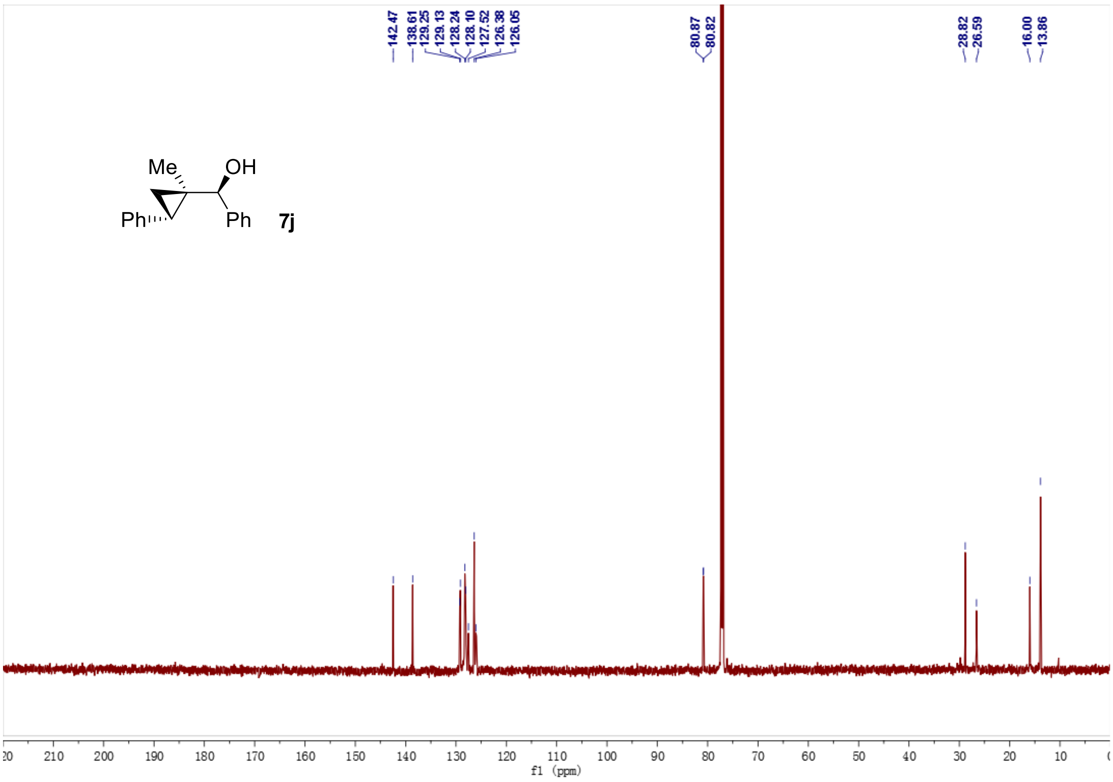

Supplementary Figure 290. ^{1}H NMR sepctra of (*S*)-((1*S*,2*R*)-1-methyl-2-phenylcyclopropyl)(phenyl)methyl propionate (**8j**)

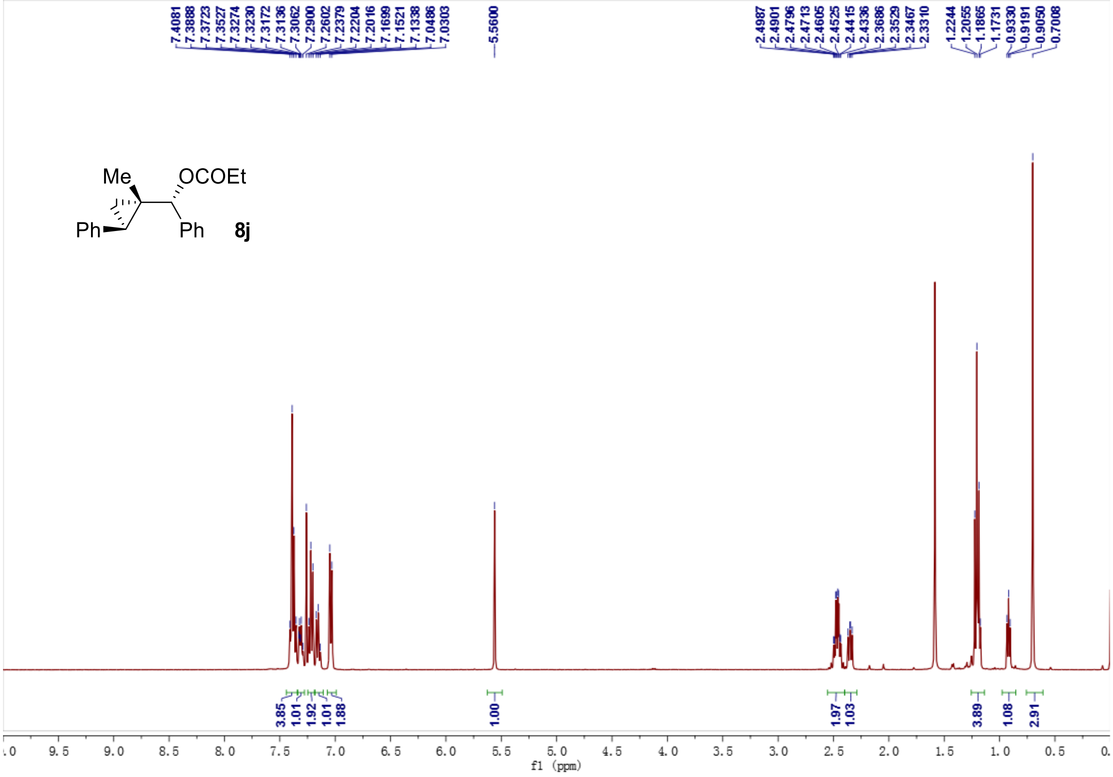

Supplementary Figure 291. ^{13}C NMR sepctra of (*S*)-((1*S*,2*R*)-1-methyl-2-phenylcyclopropyl)(phenyl)methyl propionate (**8j**)

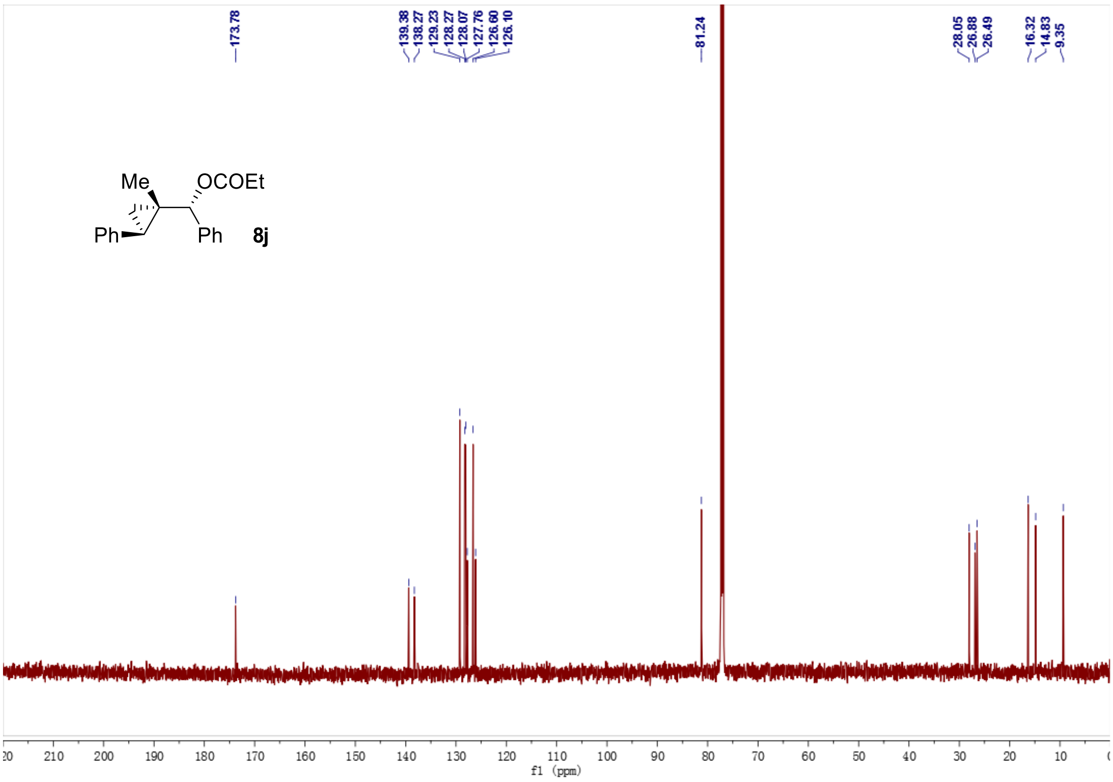

Supplementary Figure 292. ^{1}H NMR sepctra of (*S*)-2-((*S*)-hydroxy(naphthalen-2-yl)methyl)-2-methylpent-4-en-1-yl propionate (**7k**)

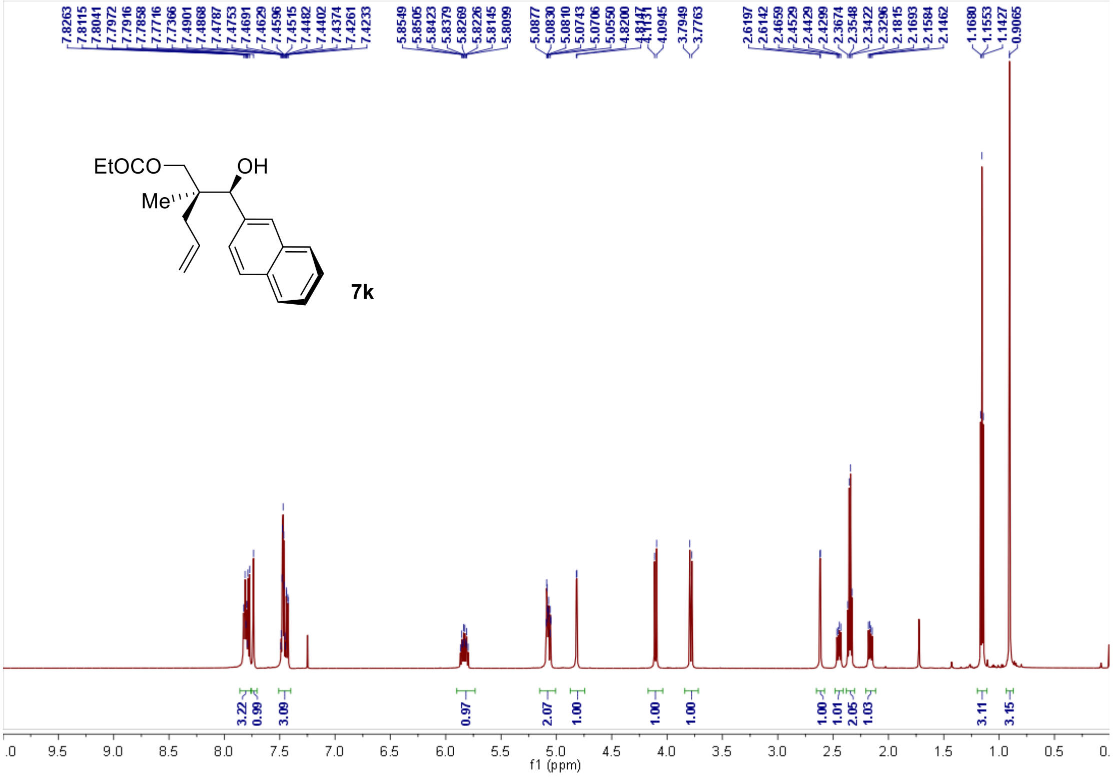

Supplementary Figure 293. ^{13}C NMR sepctra of (*S*)-2-((*S*)-hydroxy(naphthalen-2-yl)methyl)-2-methylpent-4-en-1-yl propionate (**7k**)

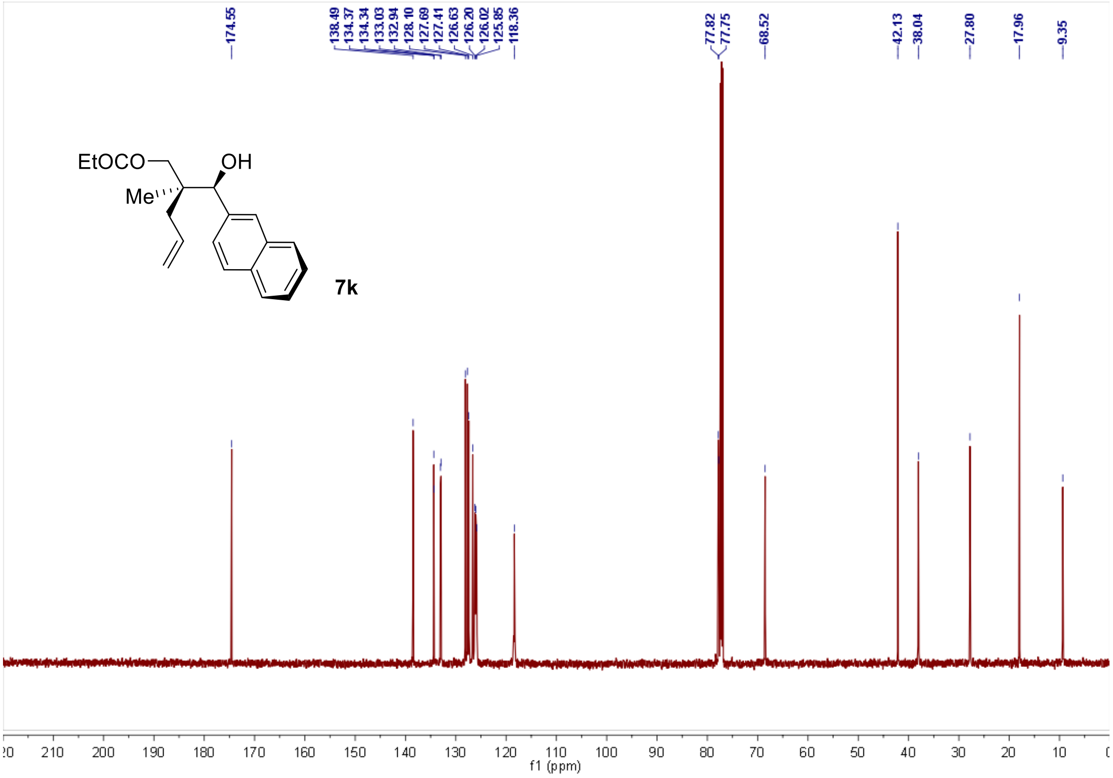

Supplementary Figure 294. ^{1}H NMR sepctra of (1*R*,2*R*)-2-allyl-2-methyl-1-(naphthalen-2-yl)propane-1,3-diyl dipropionate (**8k**)

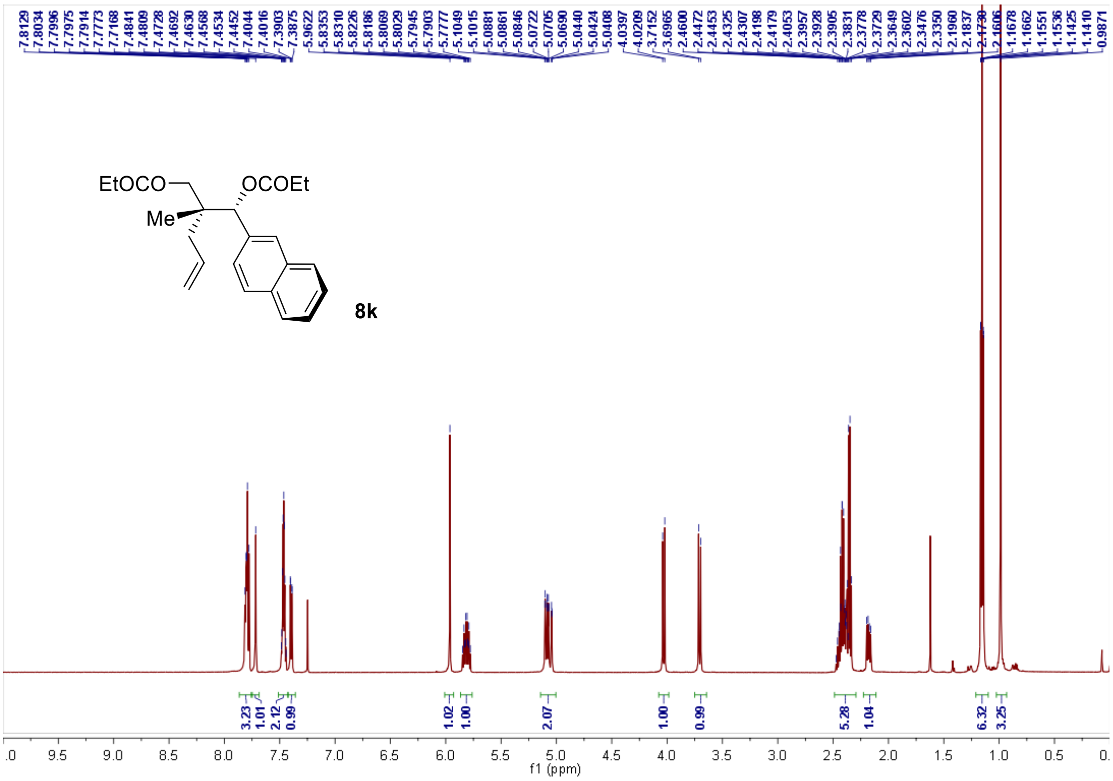

Supplementary Figure 295. ^{13}C NMR sepctra of (1*R*,2*R*)-2-allyl-2-methyl-1-(naphthalen-2-yl)propane-1,3-diyl dipropionate (**8k**)

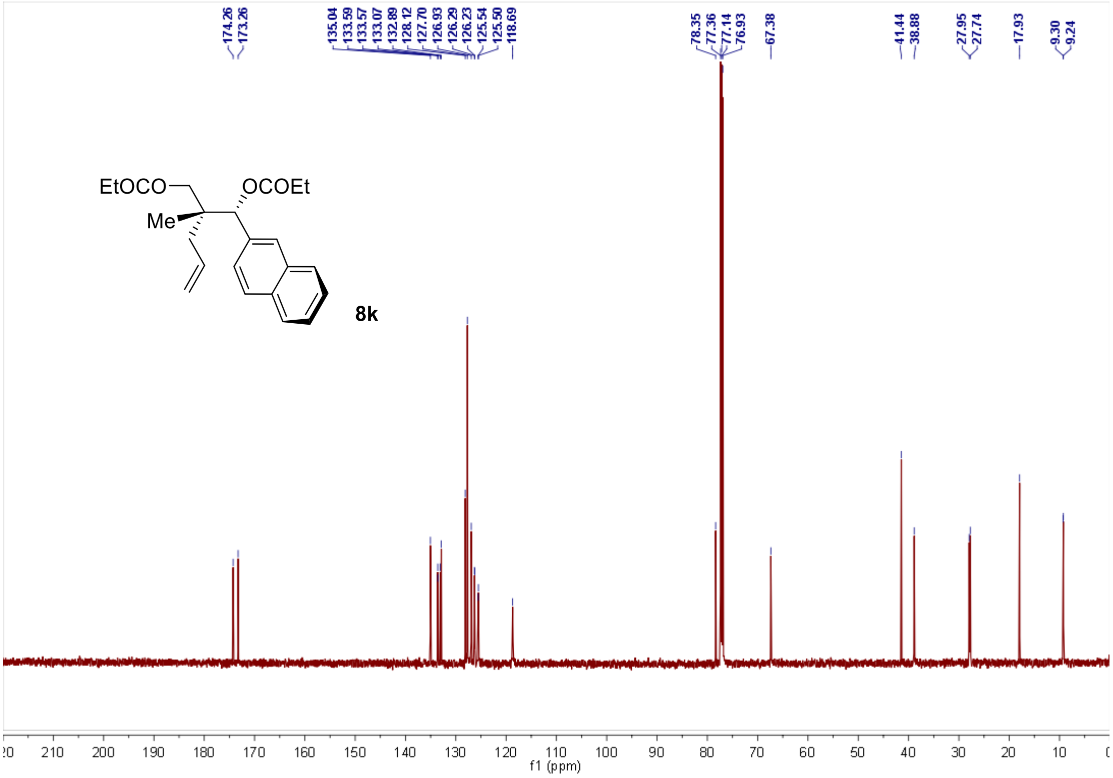

Supplementary Figure 296. ^{1}H NMR sepctra of (1*R*,2*S*)-2-methyl-1-(naphthalen-2-yl)-2-vinylpent-4-en-1-ol (**7l**)

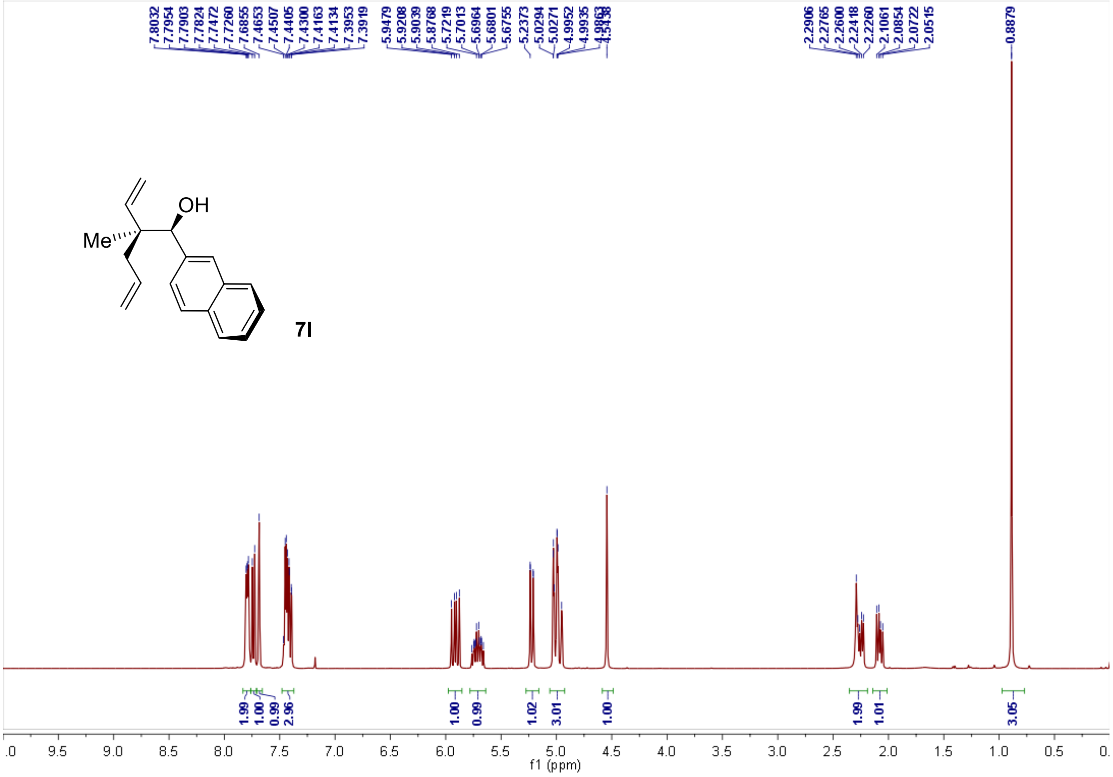

Supplementary Figure 297. ^{13}C NMR sepctra of (1*R*,2*S*)-2-methyl-1-(naphthalen-2-yl)-2-vinylpent-4-en-1-ol (**7l**)

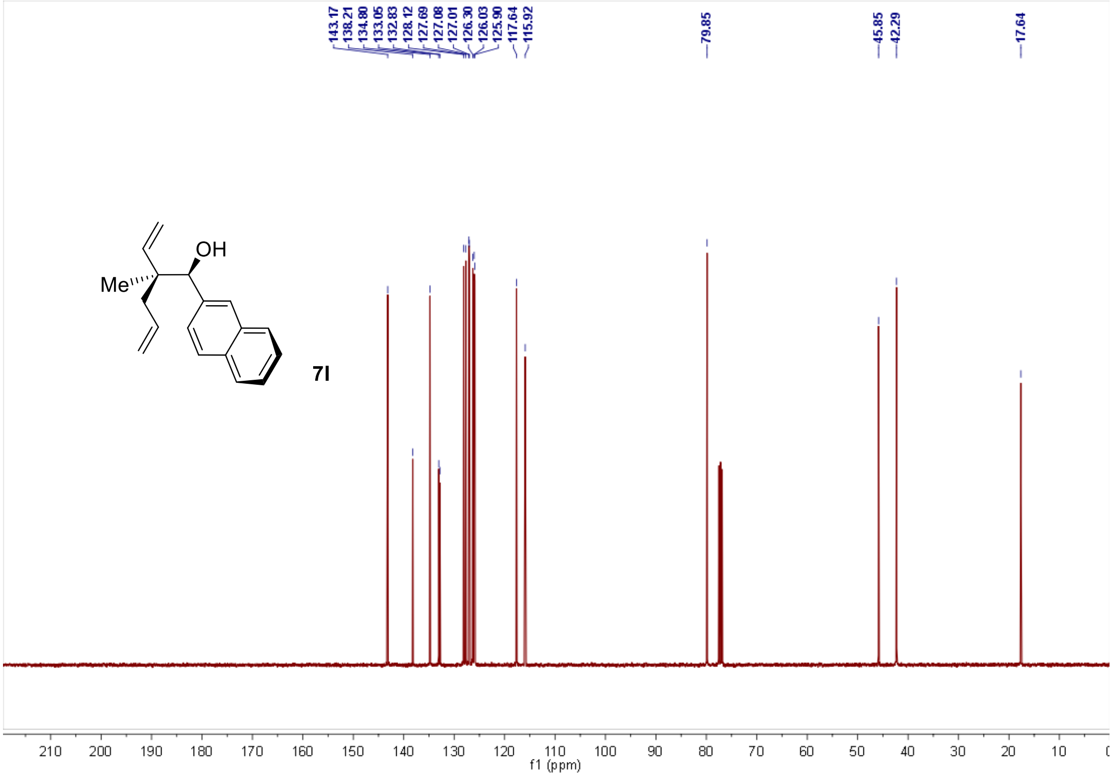

Supplementary Figure 298. ^{1}H NMR sepctra of (1*S*,2*R*)-2-methyl-1-(naphthalen-2-yl)-2-vinylpent-4-en-1-yl propionate (**8l**)

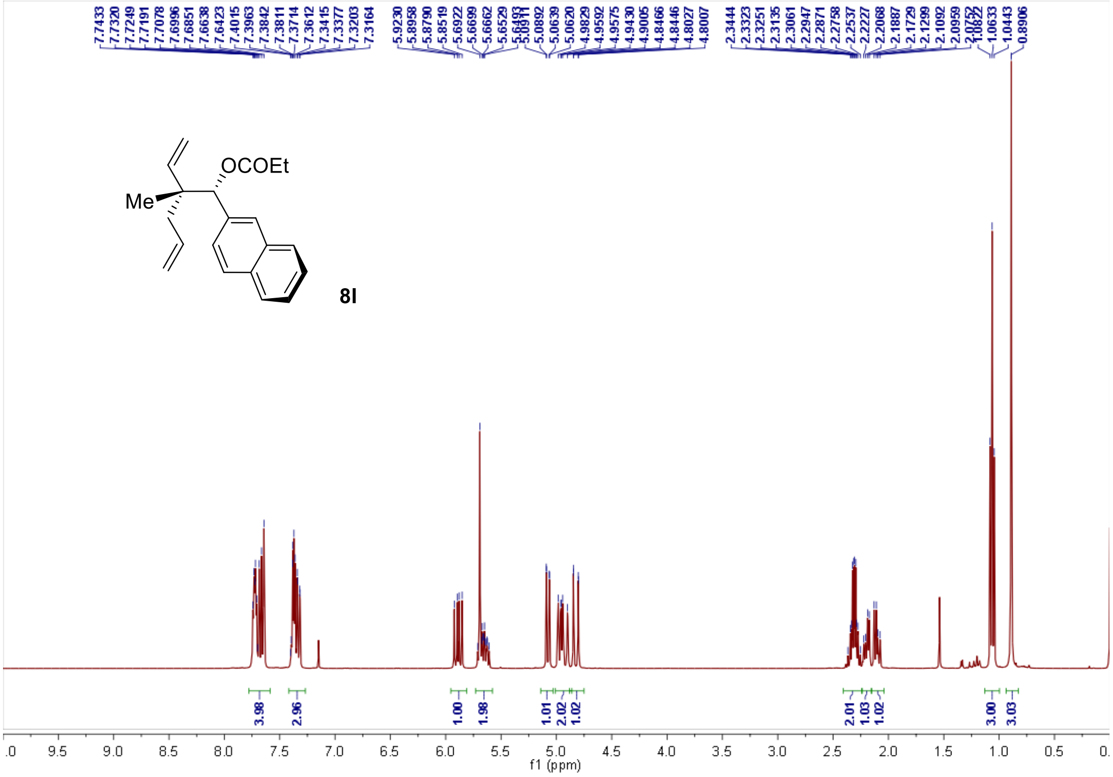

Supplementary Figure 299. ^{13}C NMR sepctra of (1*S*,2*R*)-2-methyl-1-(naphthalen-2-yl)-2-vinylpent-4-en-1-yl propionate (**8l**)

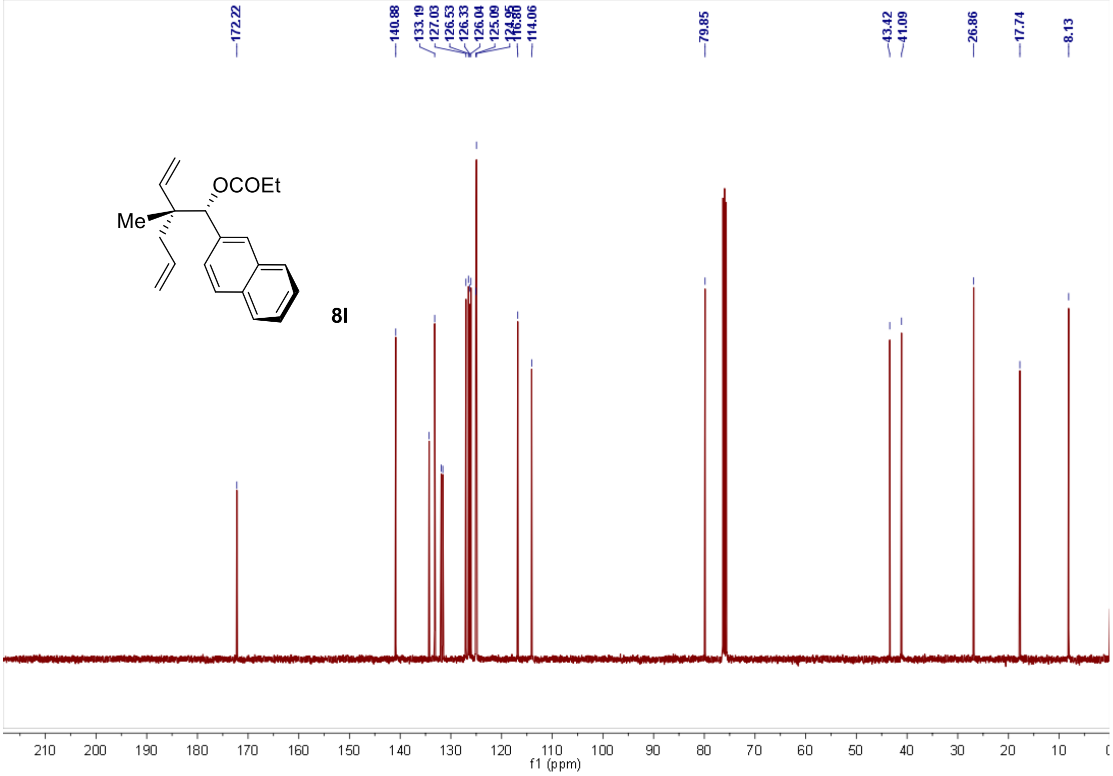

Supplementary Figure 300. ^{1}H NMR sepctra of (1*S*,2*S*)-2-ethynyl-2-methyl-1-(naphthalen-2-yl)pent-4-en-1-ol (**7m**)

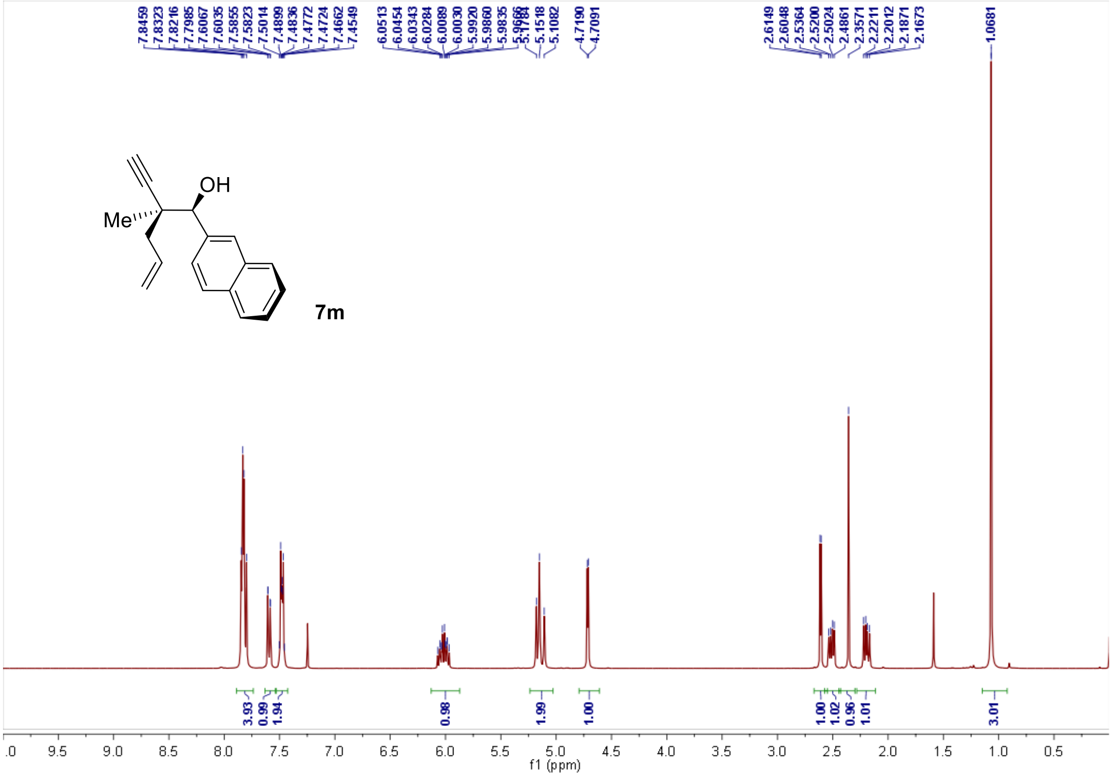

Supplementary Figure 301. ^{13}C NMR sepctra of (1*S*,2*S*)-2-ethynyl-2-methyl-1-(naphthalen-2-yl)pent-4-en-1-ol (**7m**)

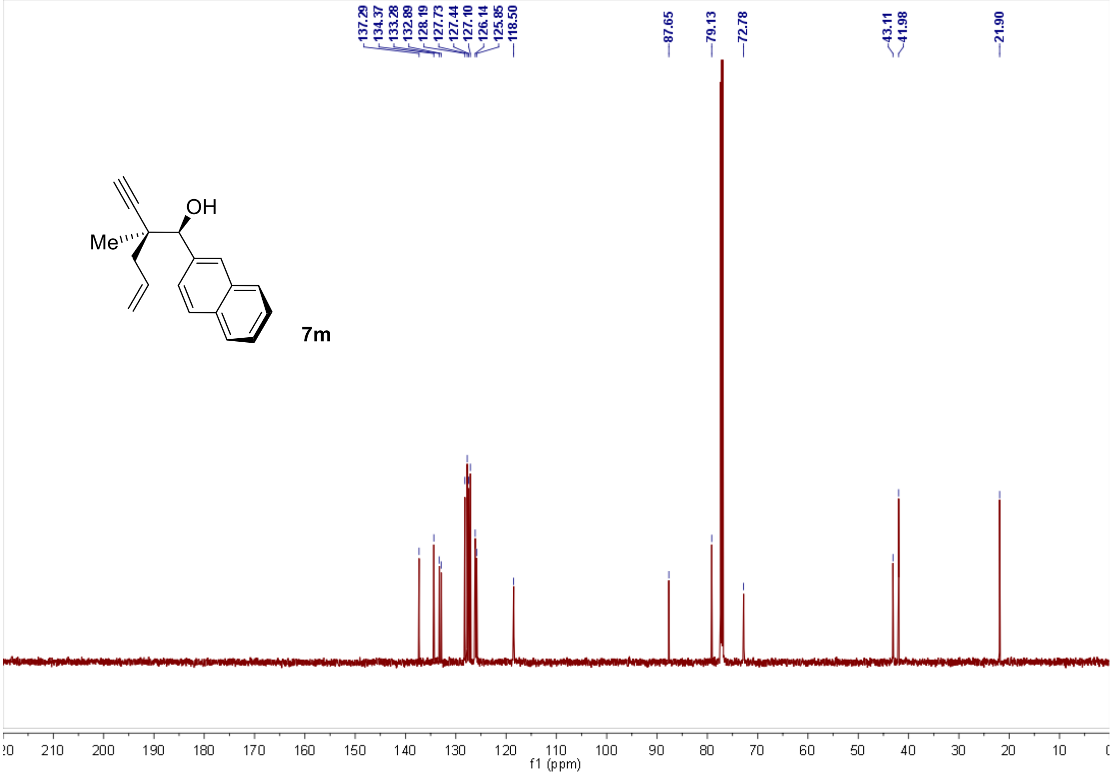

Supplementary Figure 302. ^{1}H NMR sepctra of (1*R*,2*R*)-2-ethynyl-2-methyl-1-(naphthalen-2-yl)pent-4-en-1-yl propionate (**8m**)

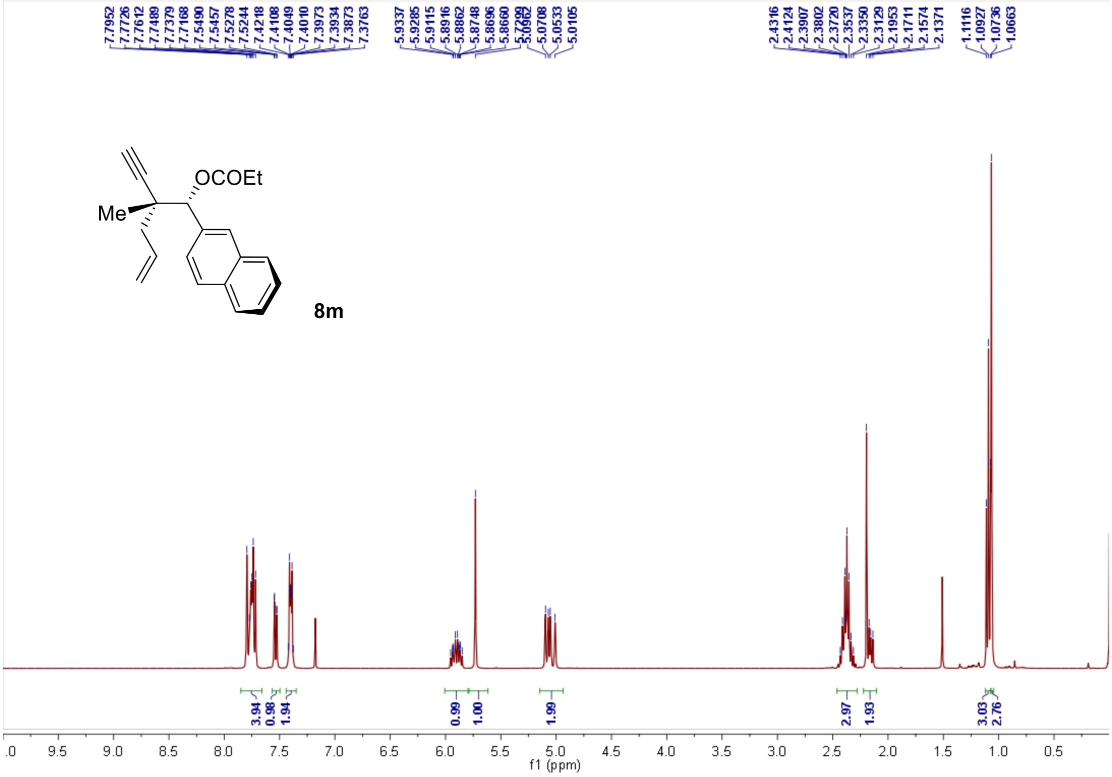

Supplementary Figure 303. ^{13}C NMR sepctra of (1*R*,2*R*)-2-ethynyl-2-methyl-1-(naphthalen-2-yl)pent-4-en-1-yl propionate (**8m**)

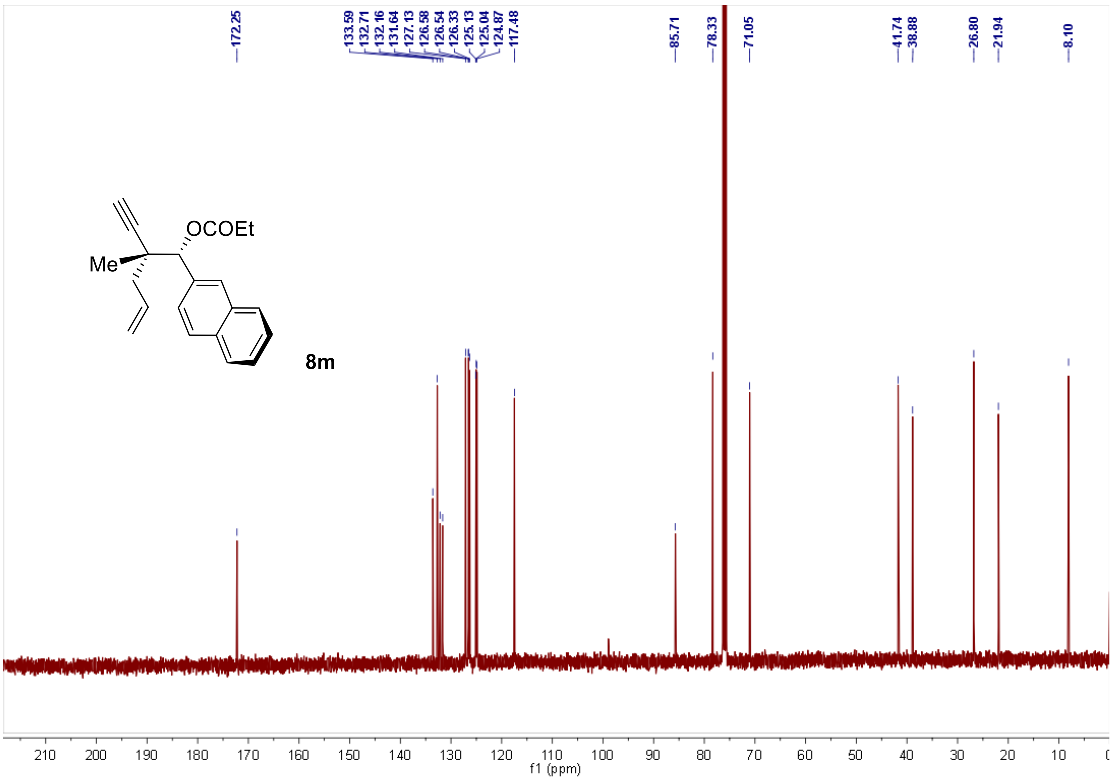

Supplementary Figure 304. ^{1}H NMR sepctra of (1*R*,2*R*)-2-benzyl-1-(naphthalen-2-yl)-2-vinylpent-4-en-1-ol (**7n**)

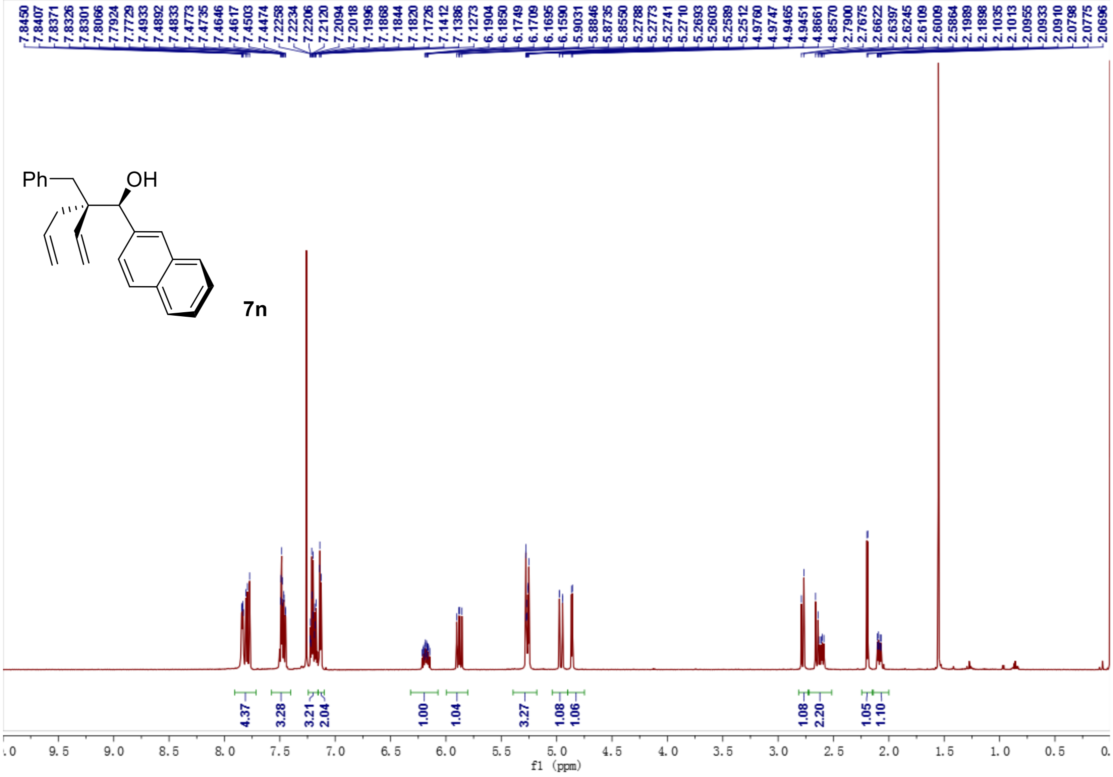

Supplementary Figure 305. ^{13}C NMR sepctra of (1*R*,2*R*)-2-benzyl-1-(naphthalen-2-yl)-2-vinylpent-4-en-1-ol (**7n**)

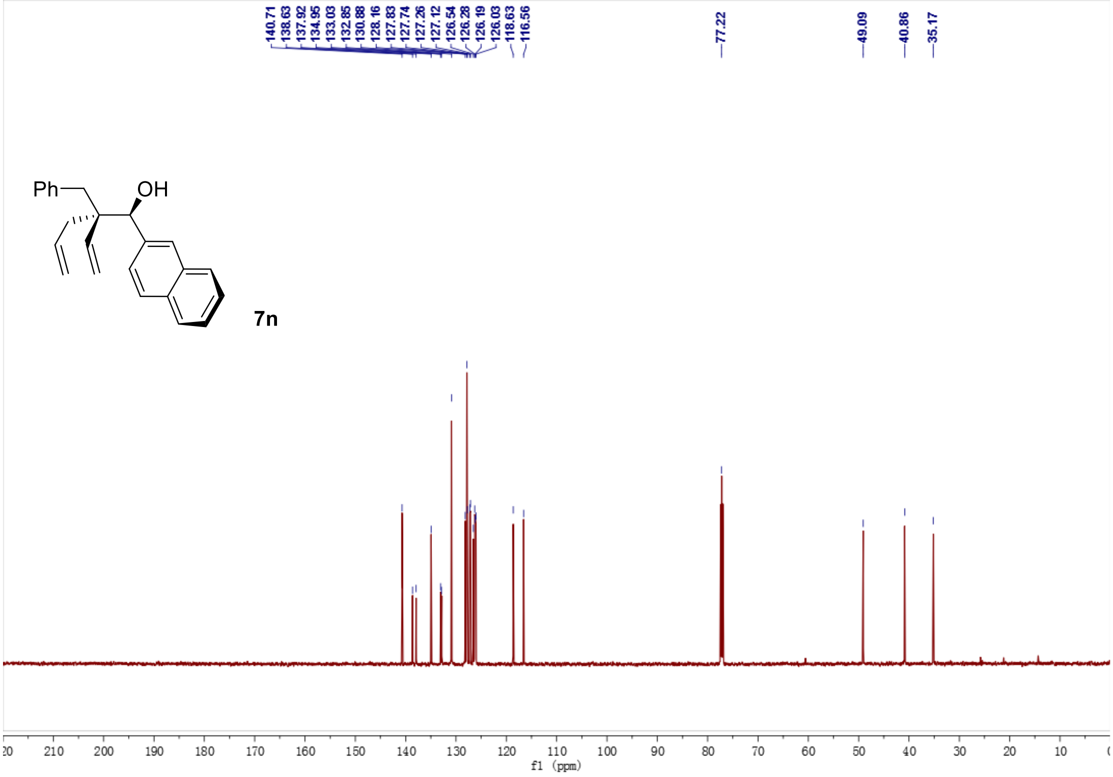

Supplementary Figure 306. ^{1}H NMR sepctra of (1*S*,2*S*)-2-benzyl-1-(naphthalen-2-yl)-2-vinylpent-4-en-1-yl propionate (**8n**)

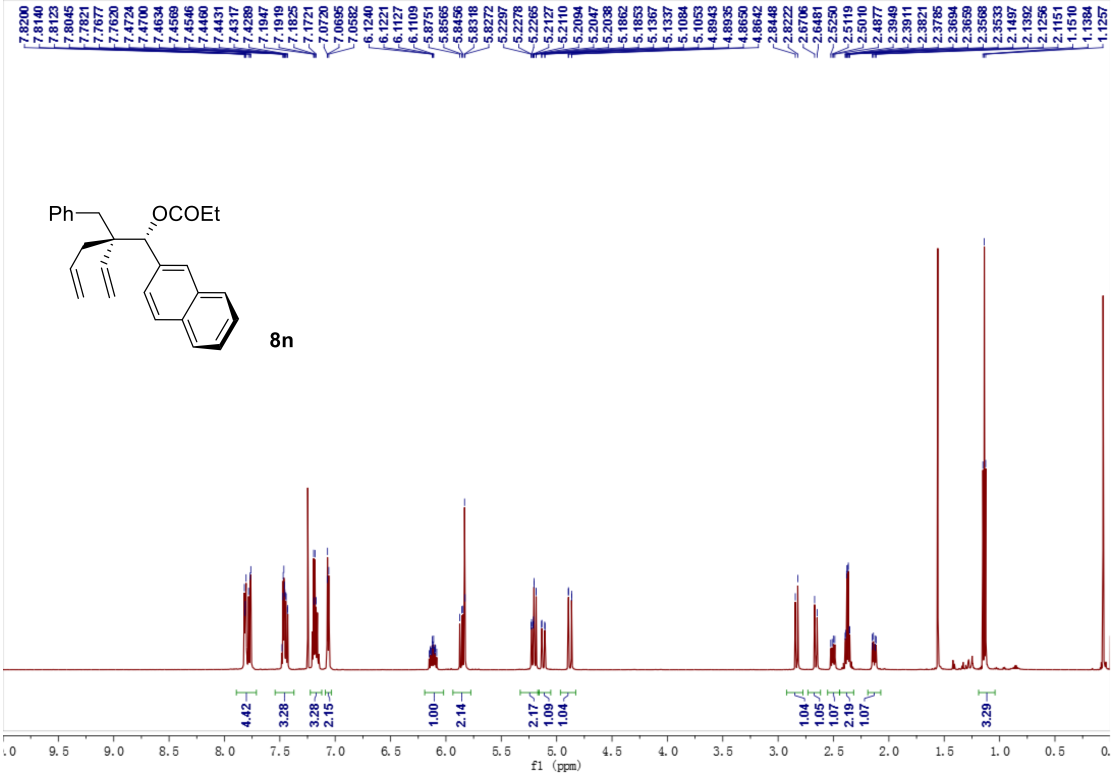

Supplementary Figure 307. ^{13}C NMR sepctra of (1*S*,2*S*)-2-benzyl-1-(naphthalen-2-yl)-2-vinylpent-4-en-1-yl propionate (**8n**)

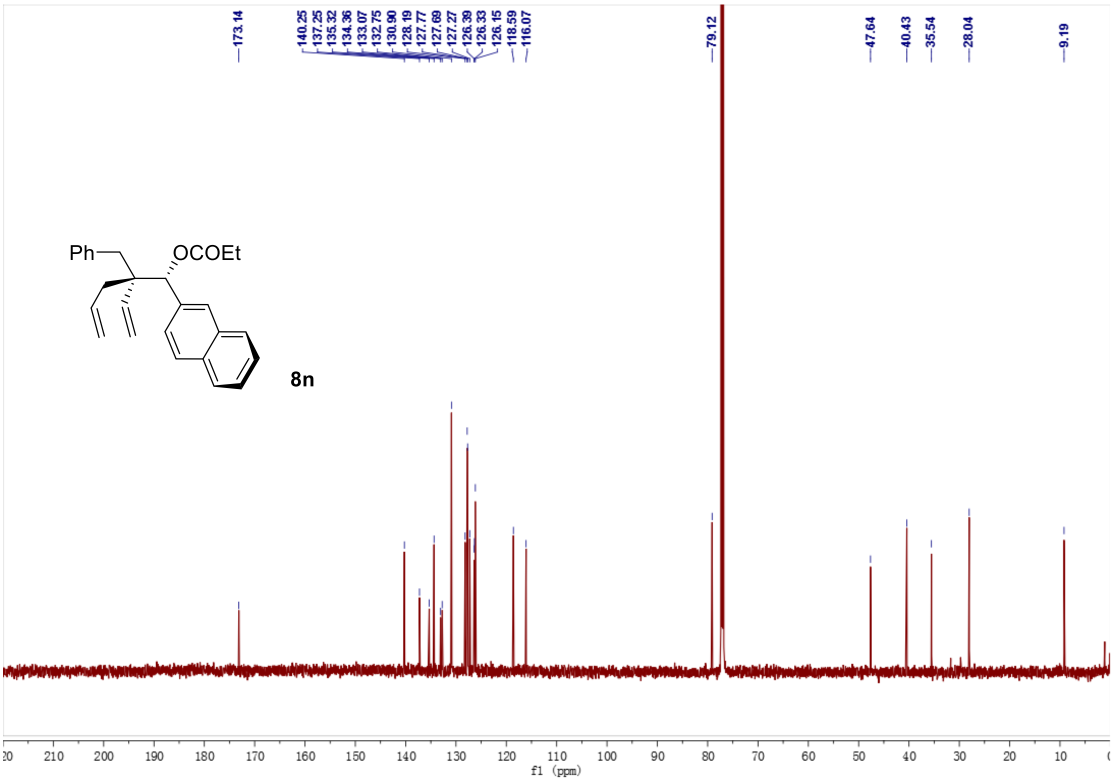

Supplementary Figure 308. ^{1}H NMR sepctra of (*R*)-1-(4-Bromophenyl)-1-(4-chlorophenyl)-1-hydroxypropan-2-one (**9a**)

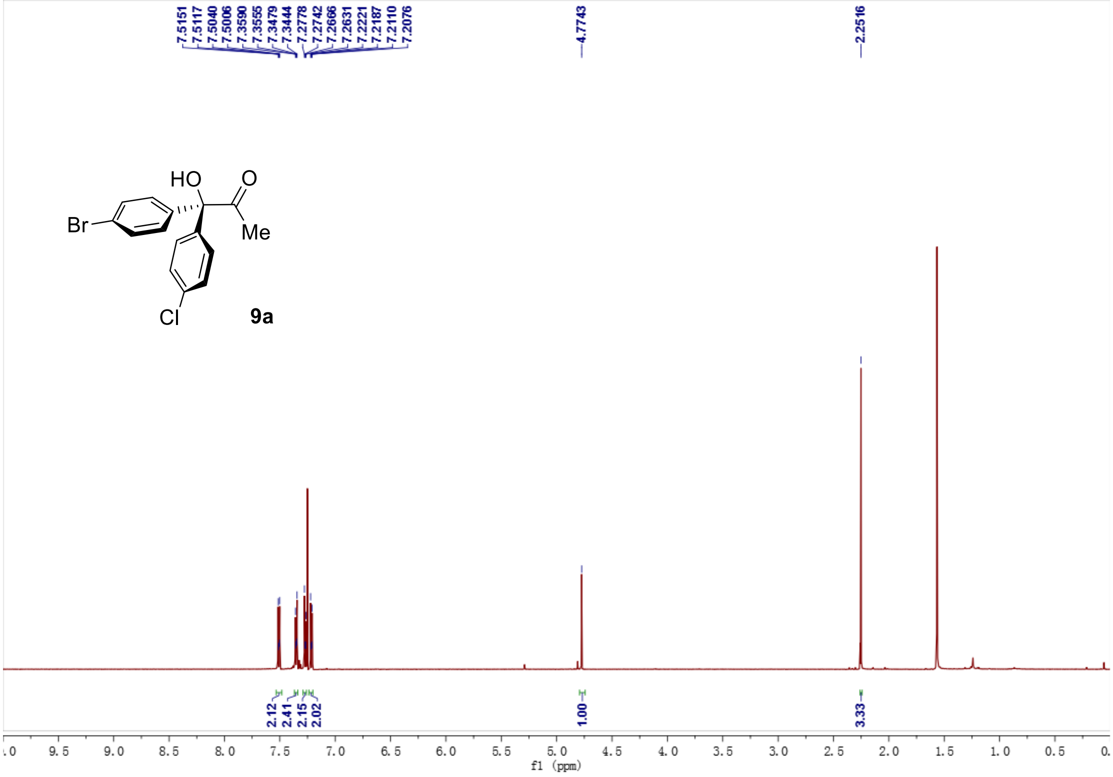

Supplementary Figure 309. ^{13}C NMR sepctra of (*R*)-1-(4-Bromophenyl)-1-(4-chlorophenyl)-1-hydroxypropan-2-one (**9a**)

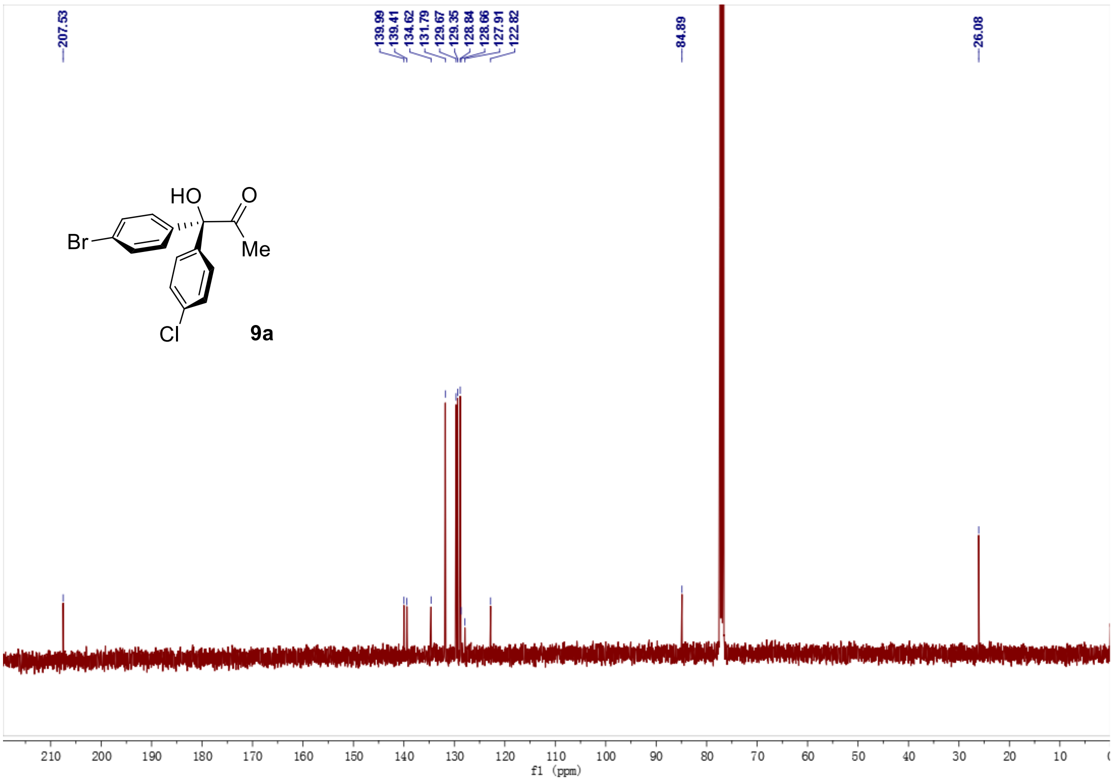

Supplementary Figure 310. ^{1}H NMR sepctra of (*R*)-2-Hydroxy-2-phenethyl-1-phenylhexan-1-one (**9b**)

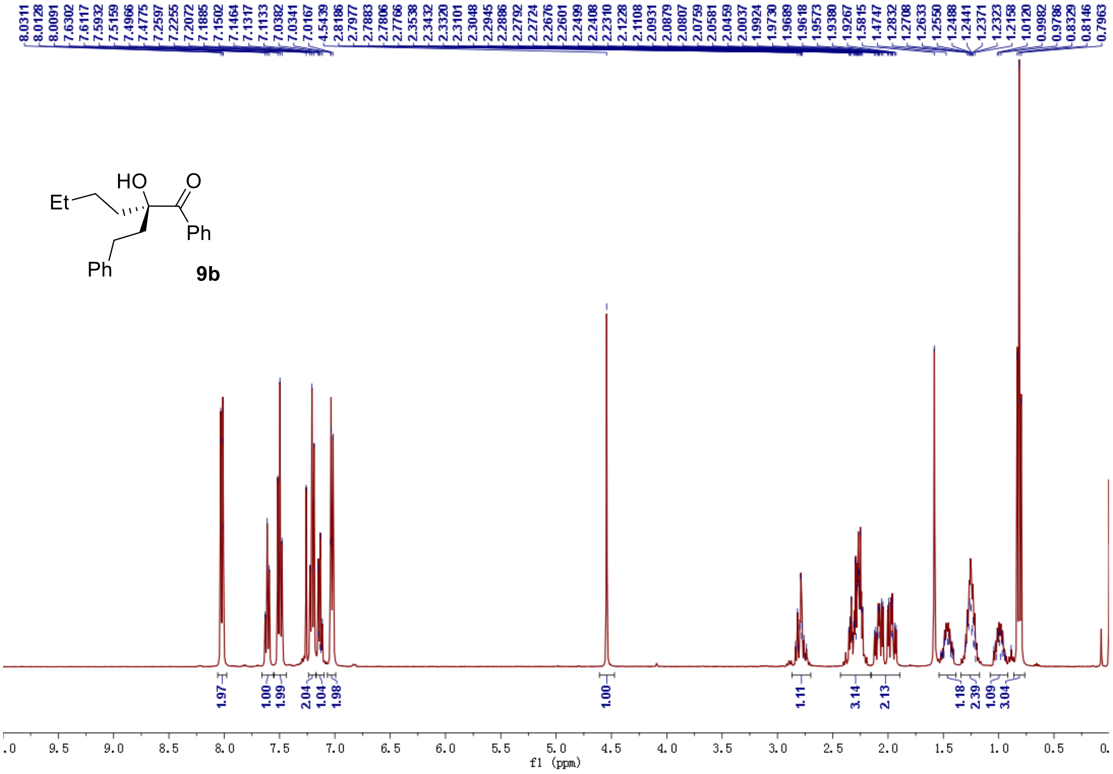

Supplementary Figure 311. ^{13}C NMR sepctra of (*R*)-2-Hydroxy-2-phenethyl-1-phenylhexan-1-one (**9b**)

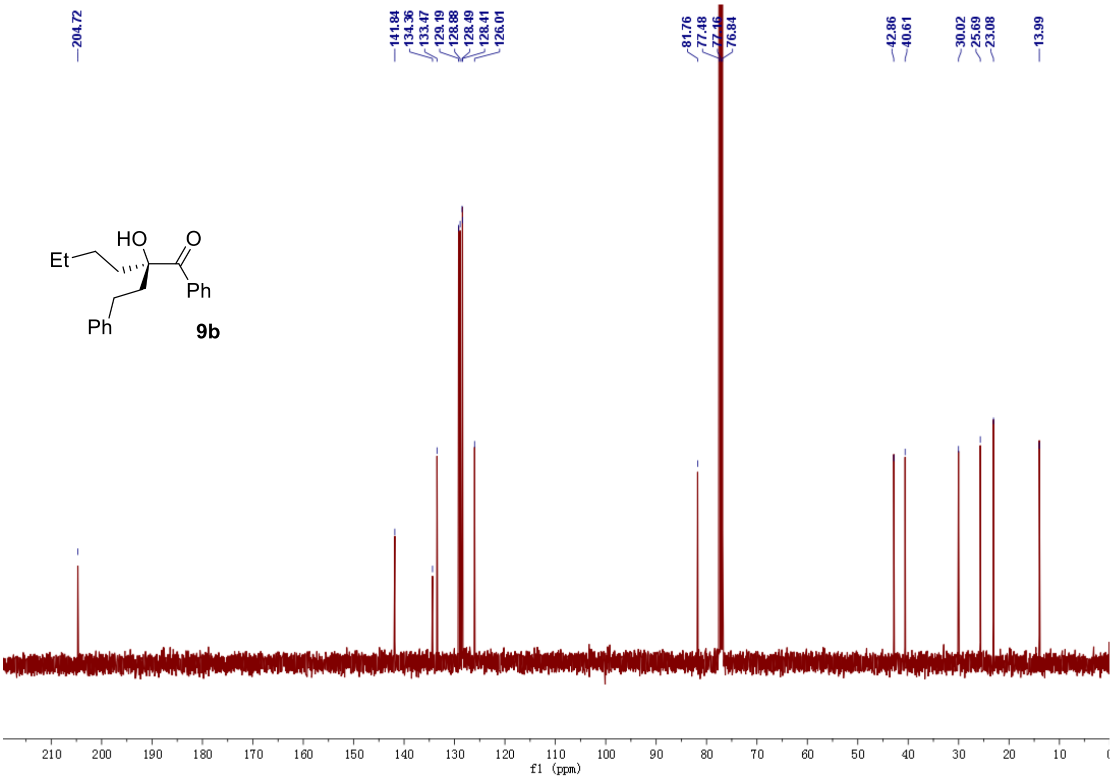

Supplementary Figure 312. ^{1}H NMR sepctra of (*R*)-2-Hydroxy-2,3-dimethyl-1-phenylbutan-1-one (**9c**)

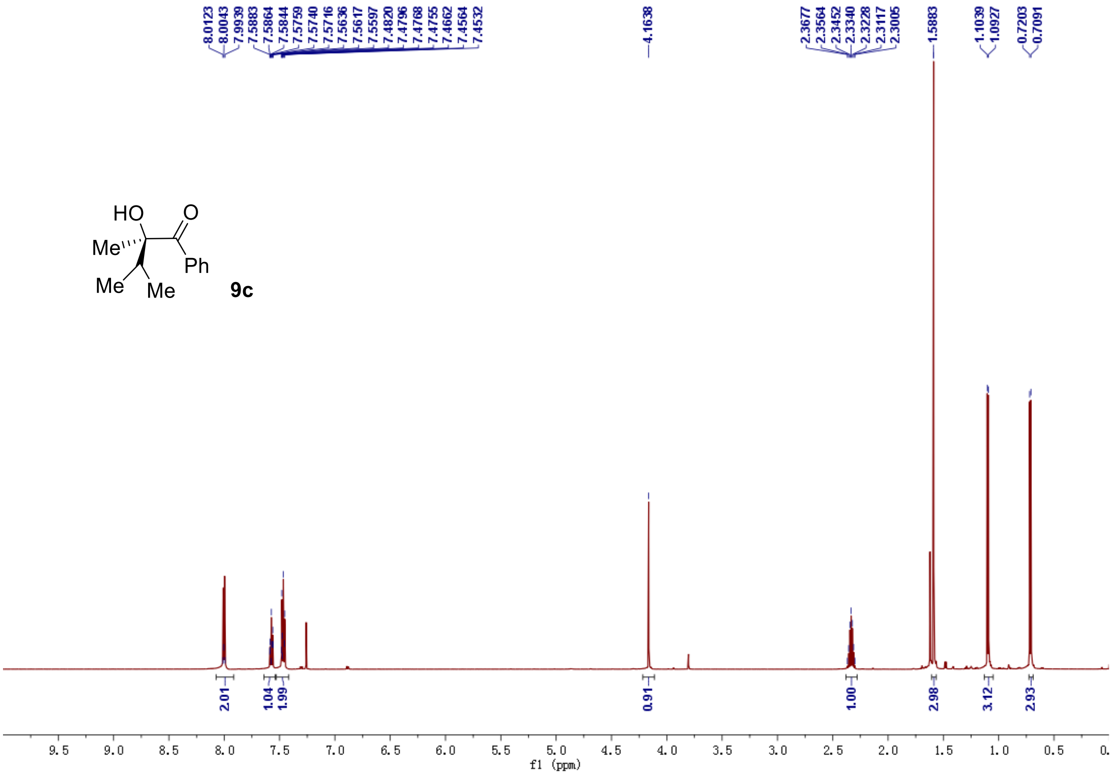

Supplementary Figure 313. ^{13}C NMR sepctra of (*R*)-2-Hydroxy-2,3-dimethyl-1-phenylbutan-1-one (**9c**)

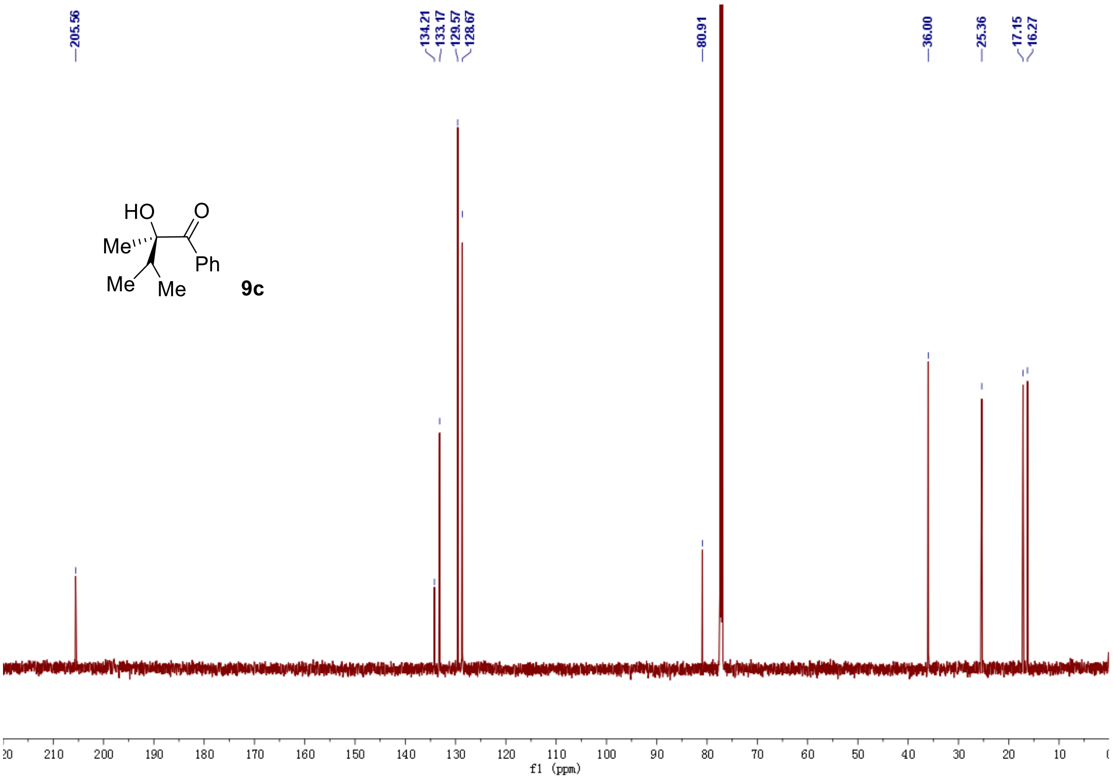

Supplementary Figure 314. ^{1}H NMR sepctra of (*S*)-2-(Methoxymethyl)-2-methyl-1-phenylpent-4-en-1-one (**9d**)

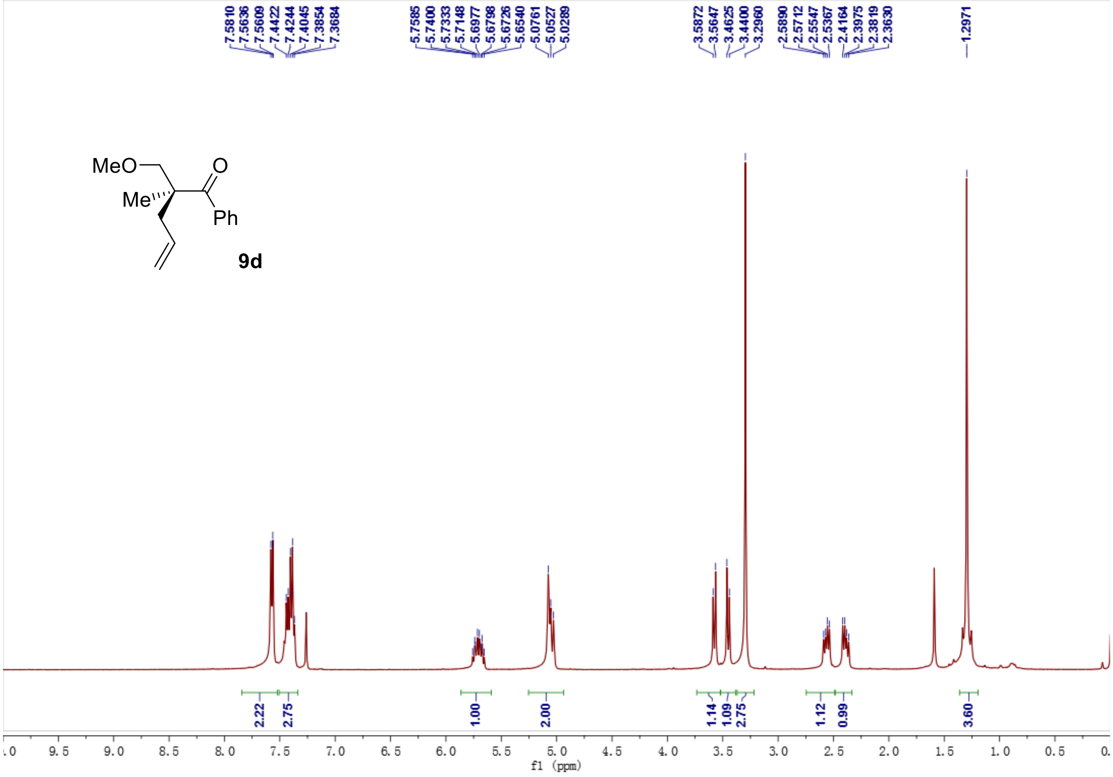

Supplementary Figure 315. ^{13}C NMR sepctra of (*S*)-2-(Methoxymethyl)-2-methyl-1-phenylpent-4-en-1-one (**9d**)

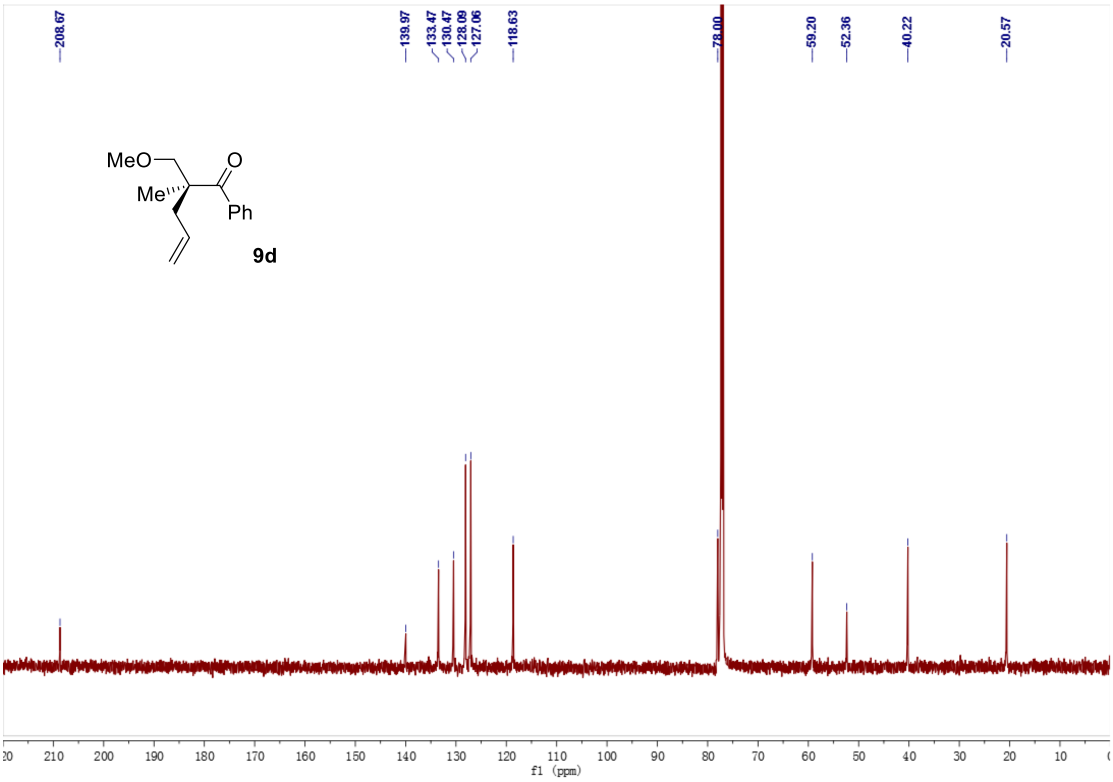

Supplementary Figure 316. ^{1}H NMR sepctra of (*R*)-2-Benzyl-1-(naphthalen-2-yl)-2-vinylpent-4-en-1-one (**9e**)

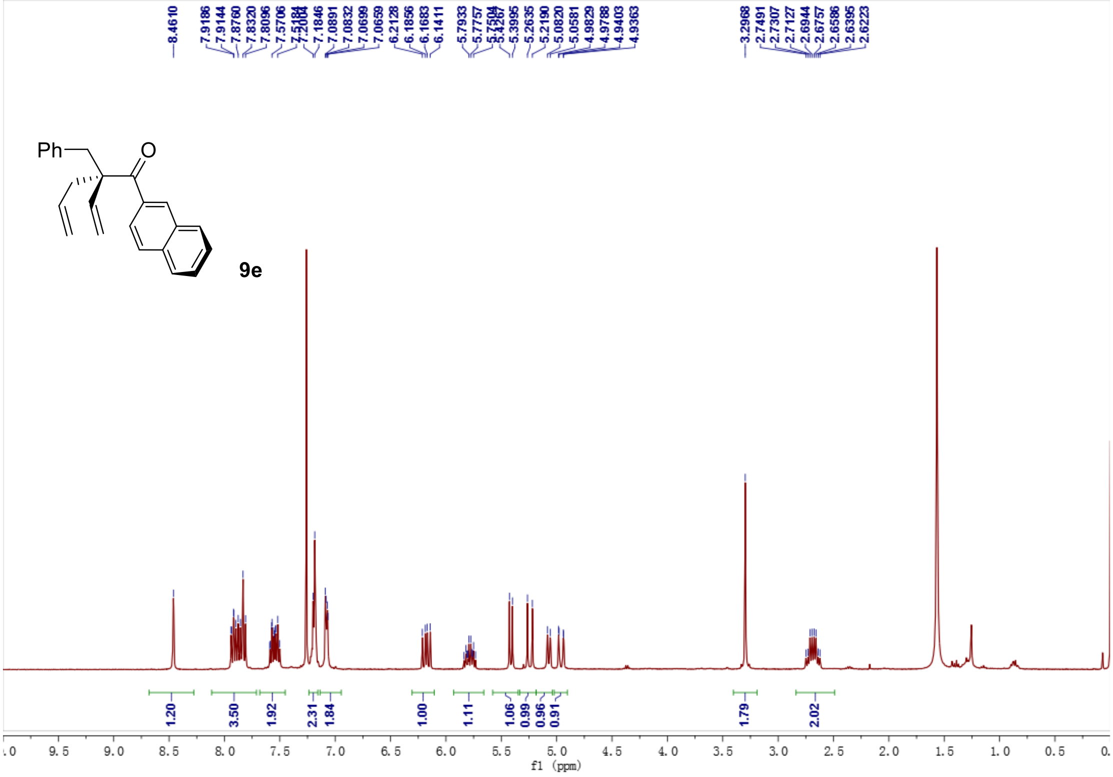

Supplementary Figure 317. ^{13}C NMR sepctra of (*R*)-2-Benzyl-1-(naphthalen-2-yl)-2-vinylpent-4-en-1-one (**9e**)

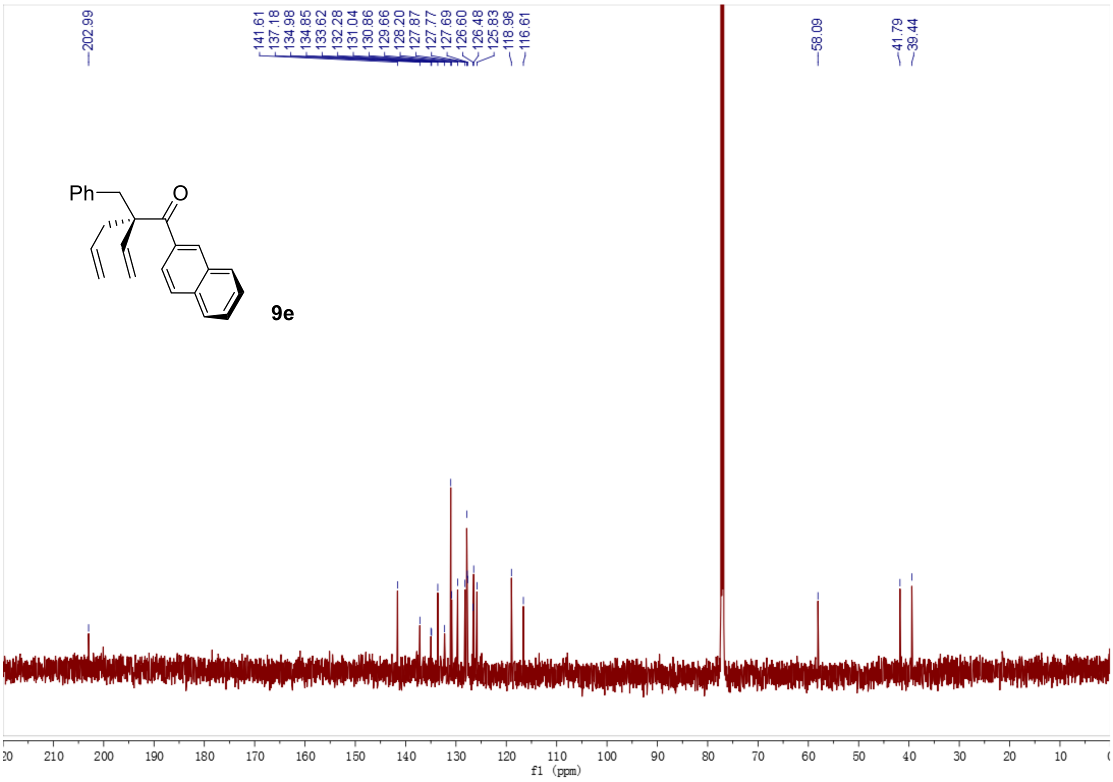

Supplementary Figure 318. ^{1}H NMR sepctra of (*S*)-2-Benzoyl-2-(4-fluorobenzyl)butyl propionate (**9f**)

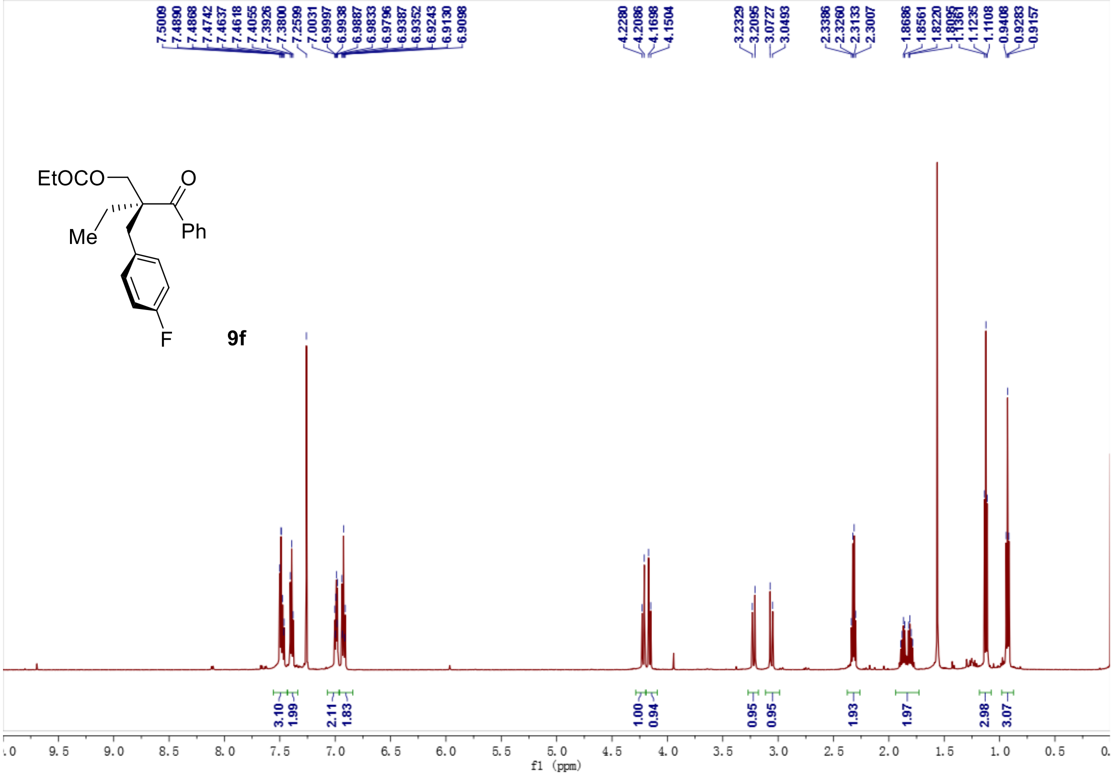

Supplementary Figure 319. ^{13}C NMR sepctra of (*S*)-2-Benzoyl-2-(4-fluorobenzyl)butyl propionate (**9f**)

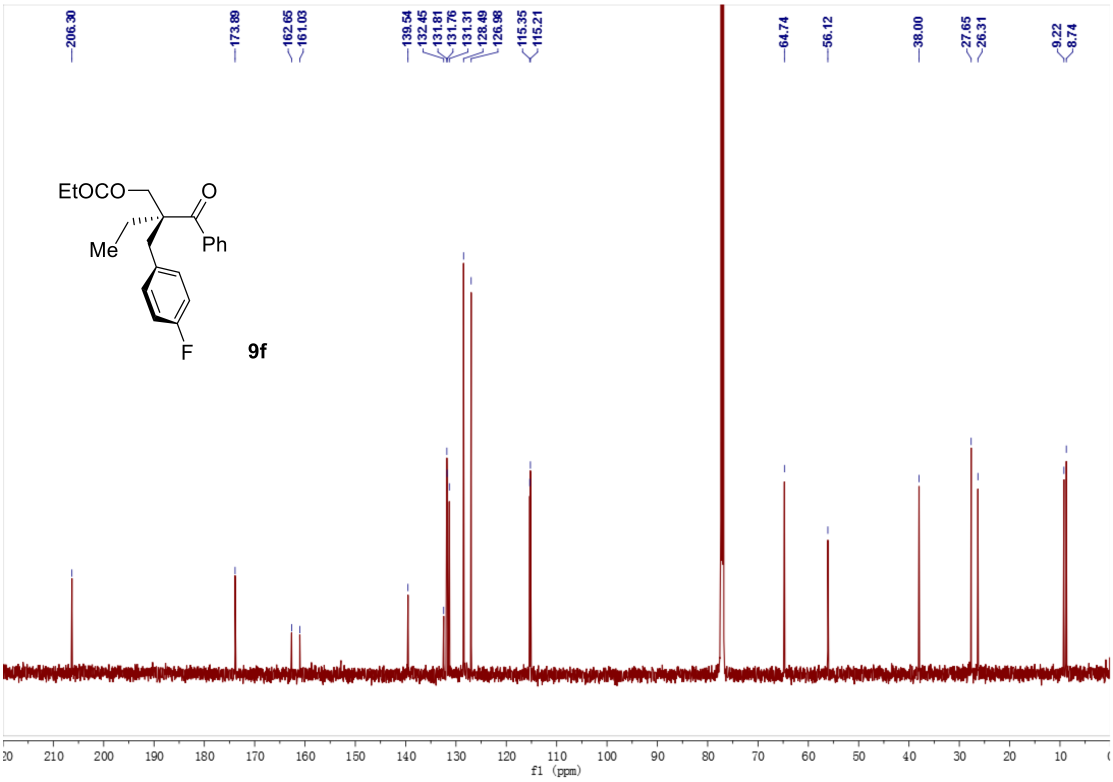

Supplementary Figure 320. ^{1}H NMR sepctra of (*S*)-2-Methyl-3-oxo-2,3-diphenylpropyl propionate (**9g**)

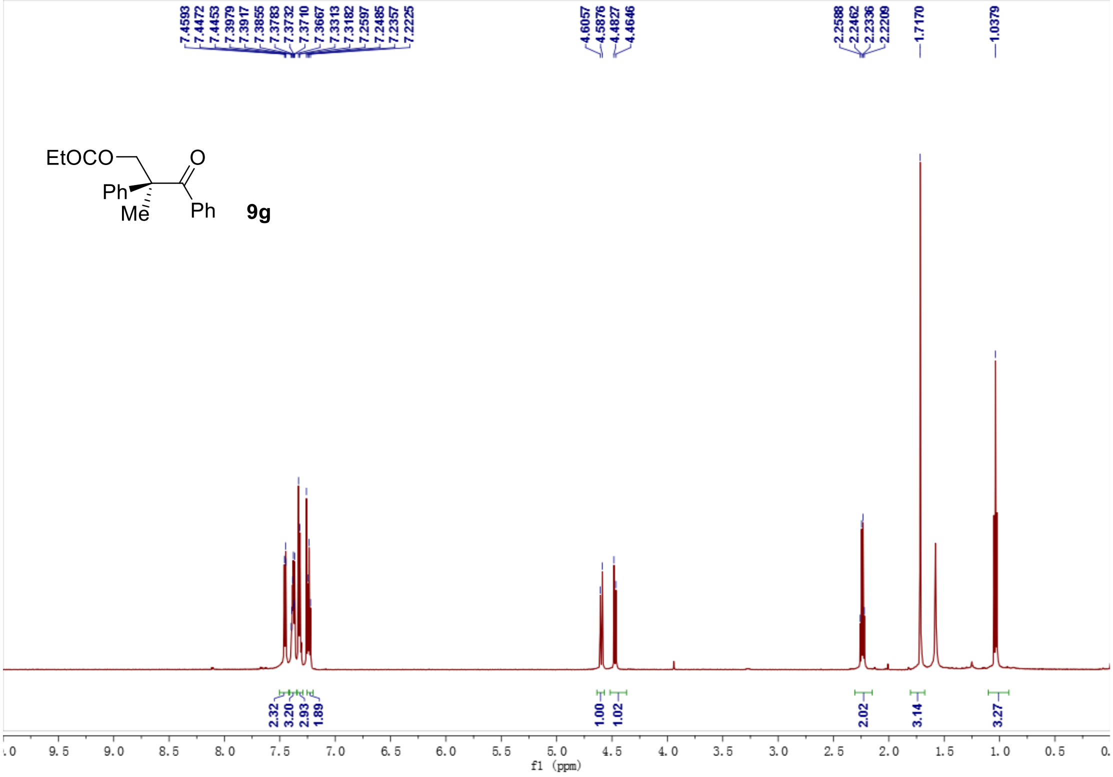

Supplementary Figure 321. ^{13}C NMR sepctra of (*S*)-2-Methyl-3-oxo-2,3-diphenylpropyl propionate (**9g**)

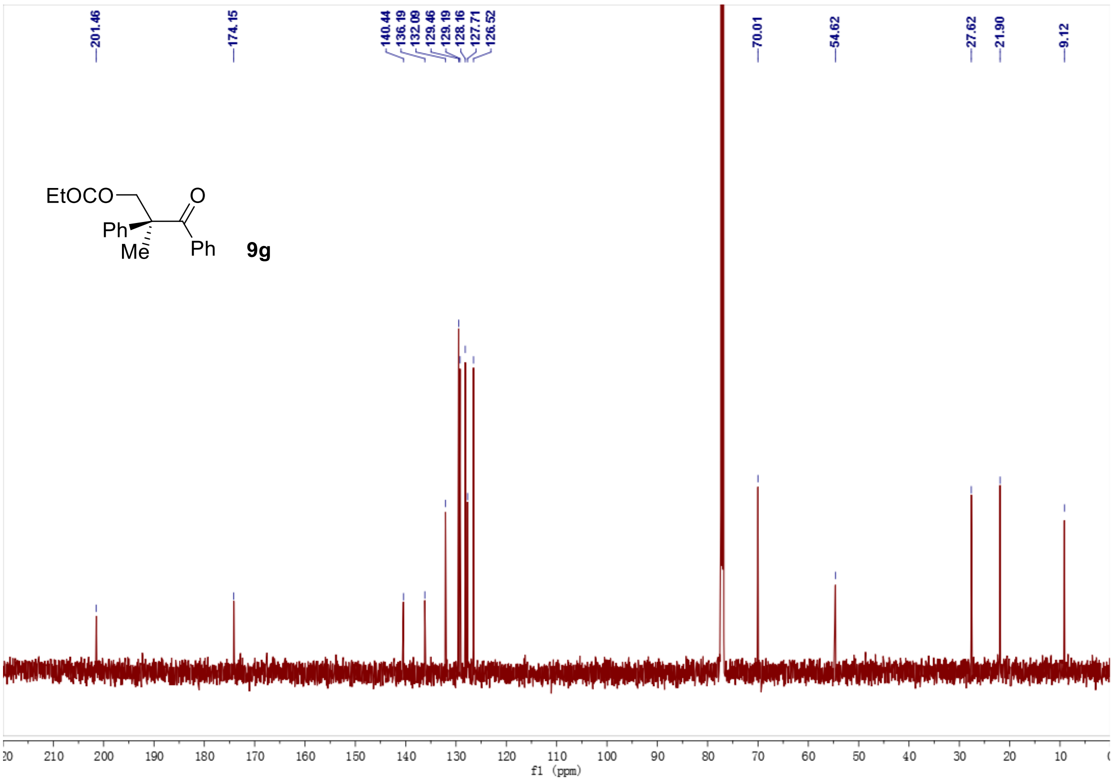

Supplementary Figure 322. ^{1}H NMR sepctra of Methyl (*R*)-2-(4-chlorobenzoyl)-2-(naphthalen-2-ylmethyl)pent-4-enoate (**9h**)

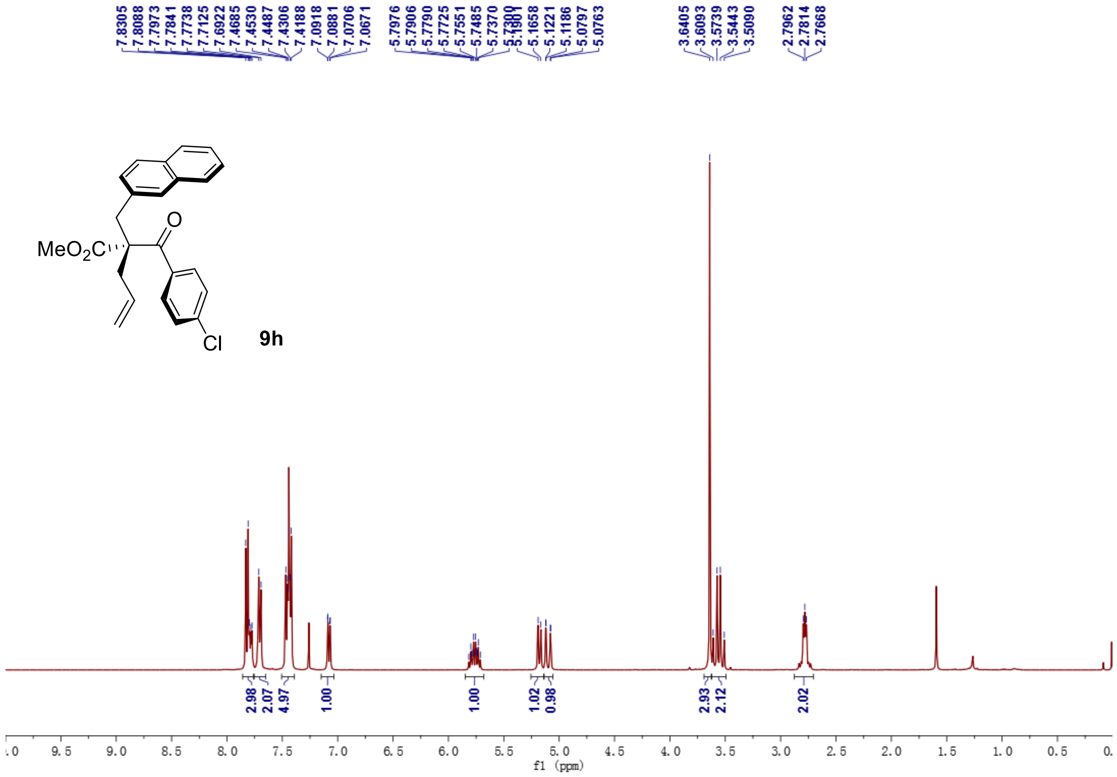

Supplementary Figure 323. ^{13}C NMR sepctra of Methyl (*R*)-2-(4-chlorobenzoyl)-2-(naphthalen-2-ylmethyl)pent-4-enoate (**9h**)

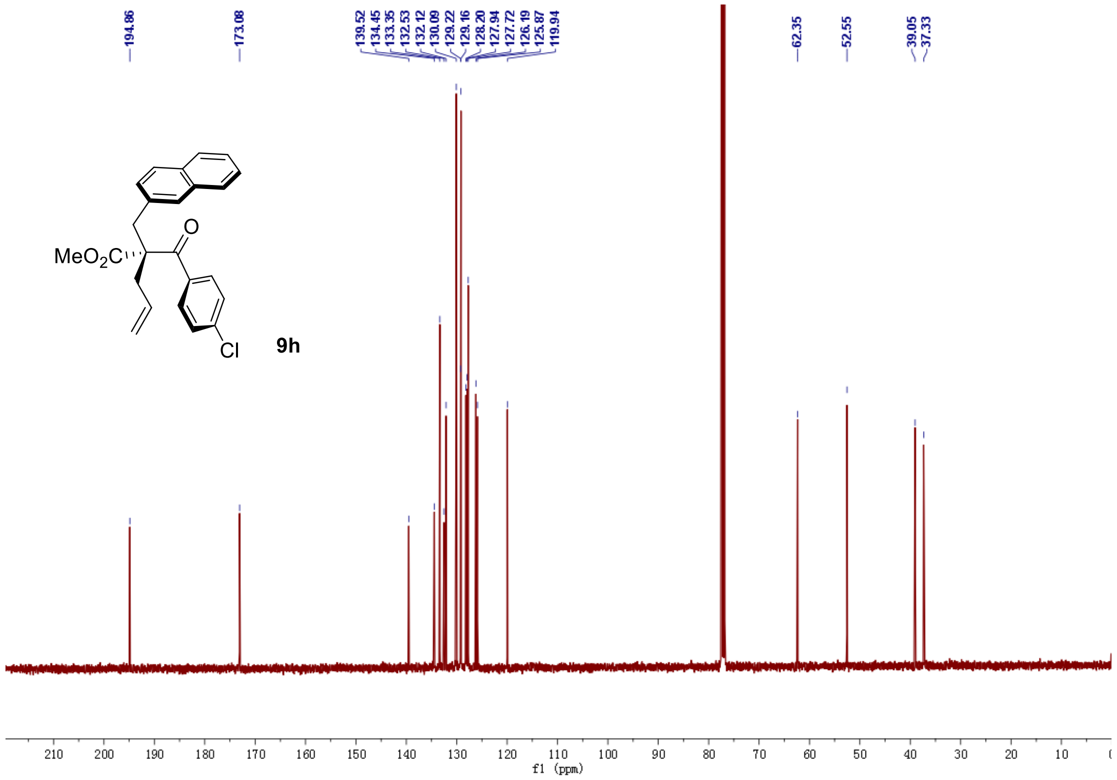

Supplementary Figure 324. ^{1}H NMR sepctra of (*R*)-Methyl 2-(2-naphthoyl)-2-benzylpent-4-enoate (**9i**)

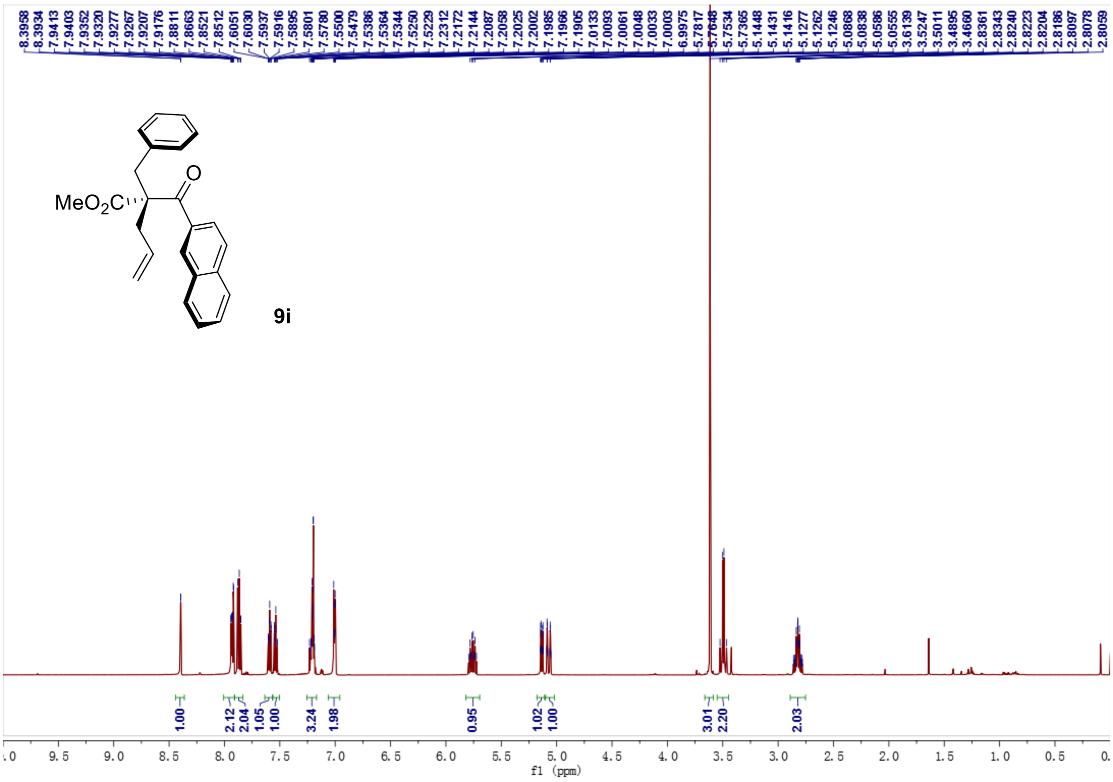

Supplementary Figure 325. ^{13}C NMR sepctra of (*R*)-Methyl 2-(2-naphthoyl)-2-benzylpent-4-enoate (**9i**)

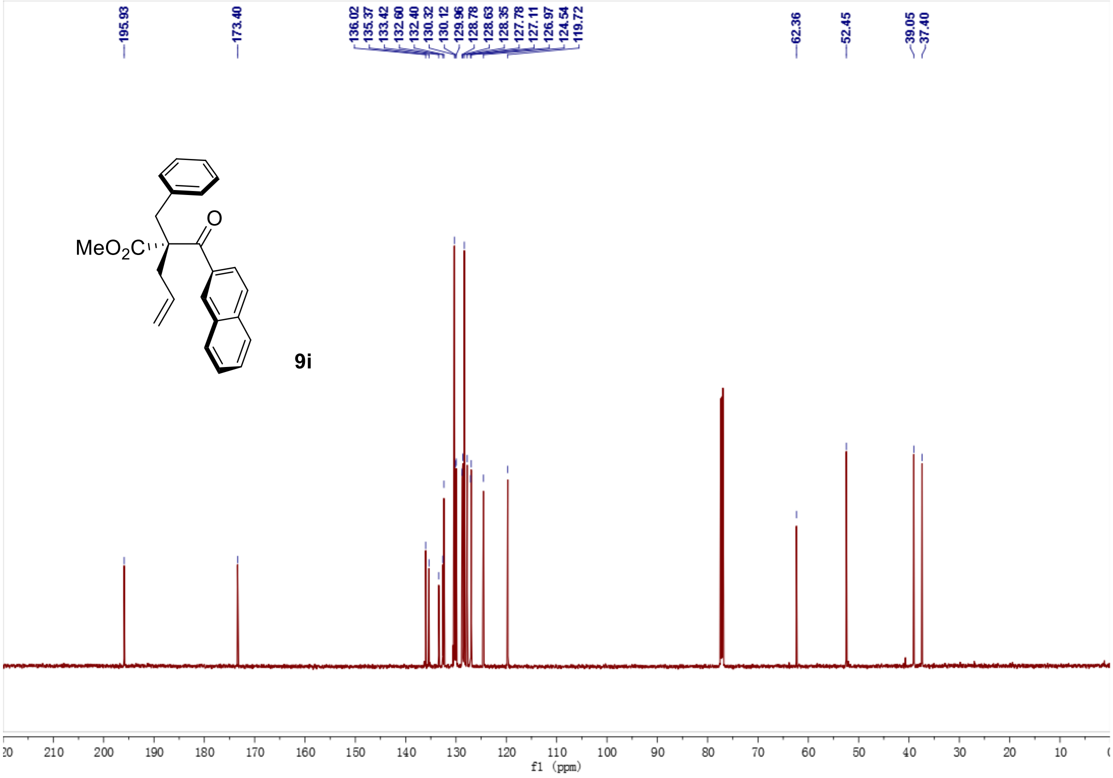

Supplementary Figure 326. ^{1}H NMR sepctra of ((2*R*,3*R*)-3-Hexyl-3-pentyloxiran-2-yl)(phenyl)methanone (**9j**)

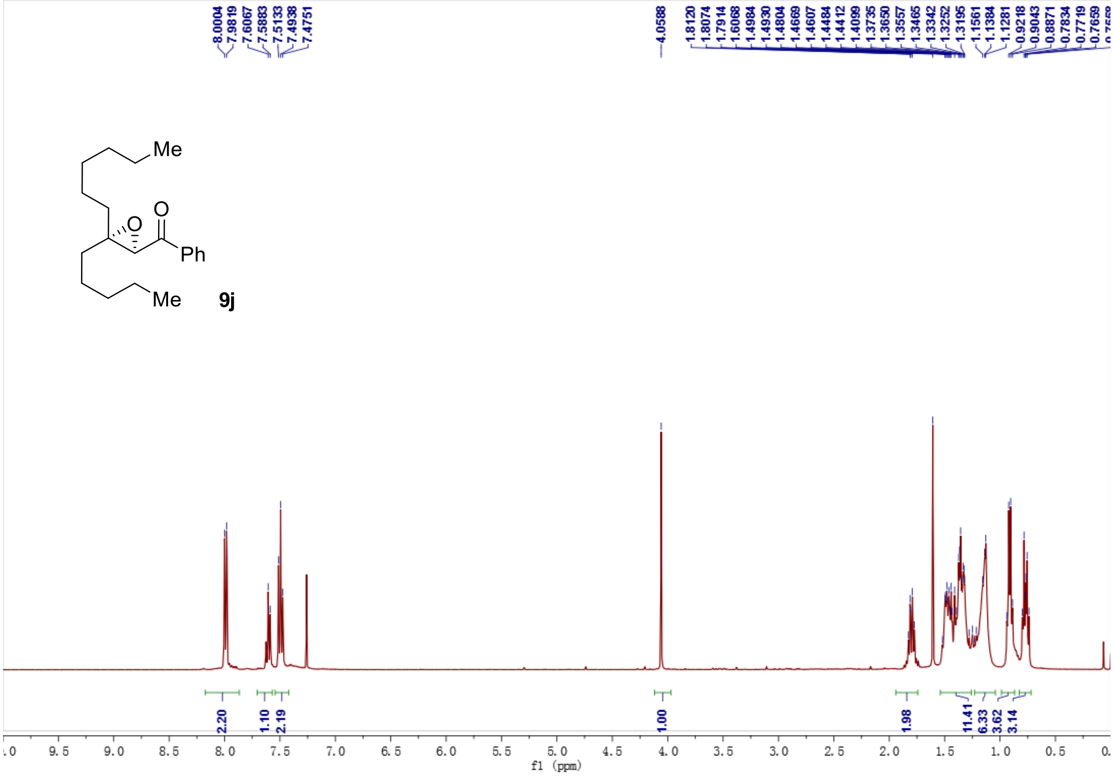

Supplementary Figure 327. ^{13}C NMR sepctra of ((2*R*,3*R*)-3-Hexyl-3-pentyloxiran-2-yl)(phenyl)methanone (**9j**)

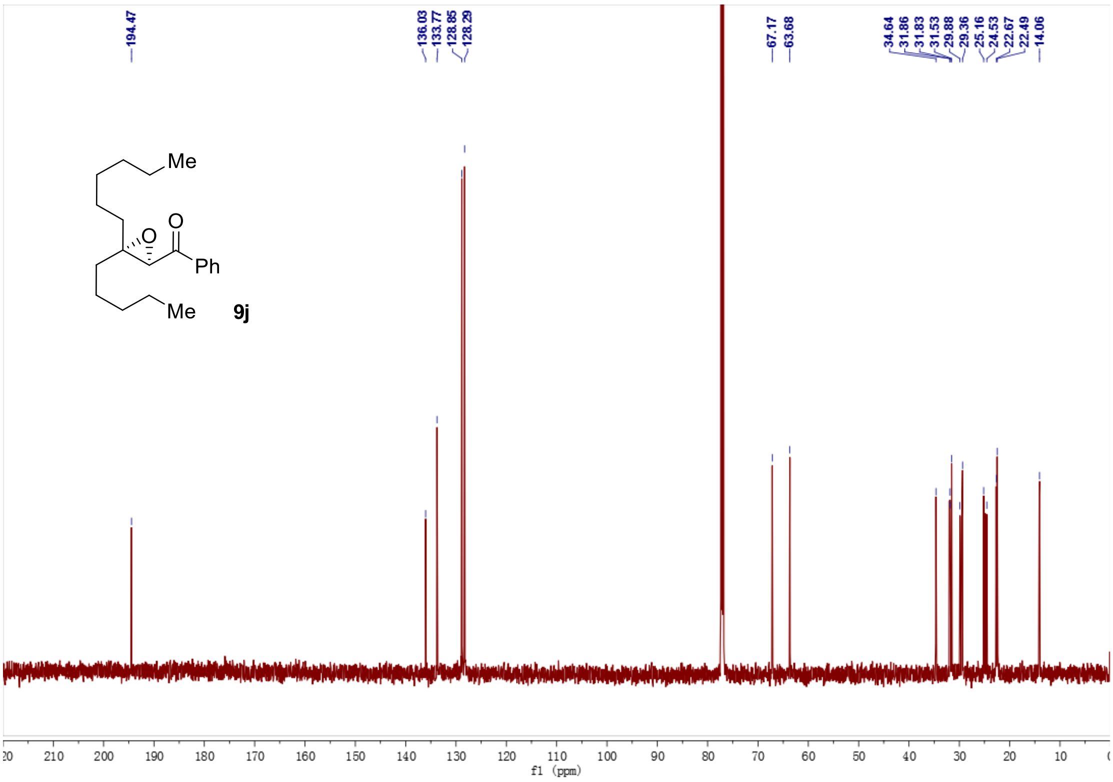

Supplementary Figure 328. ^{1}H NMR sepctra of Naphthalen-1-yl((2*R*,3*R*)-3-phenyl-3-(*p*-tolyl)oxiran-2-yl)methanone (**9k**)

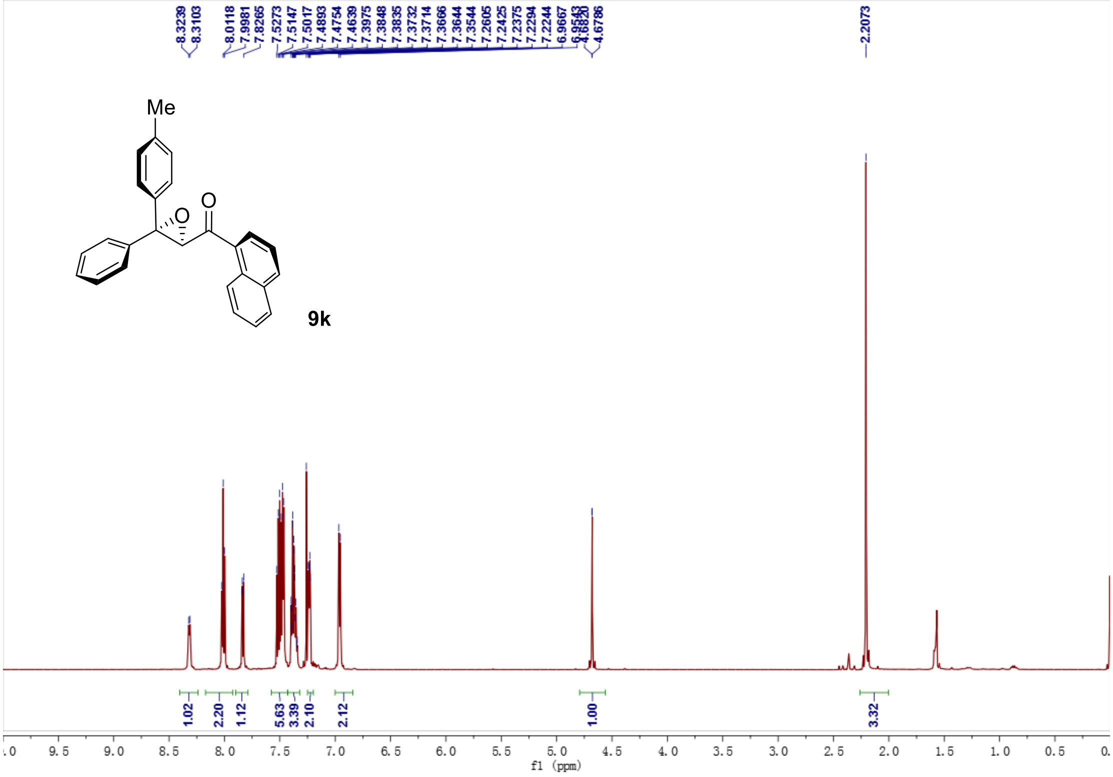

Supplementary Figure 329. ^{13}C NMR sepctra of Naphthalen-1-yl((2*R*,3*R*)-3-phenyl-3-(*p*-tolyl)oxiran-2-yl)methanone (**9k**)

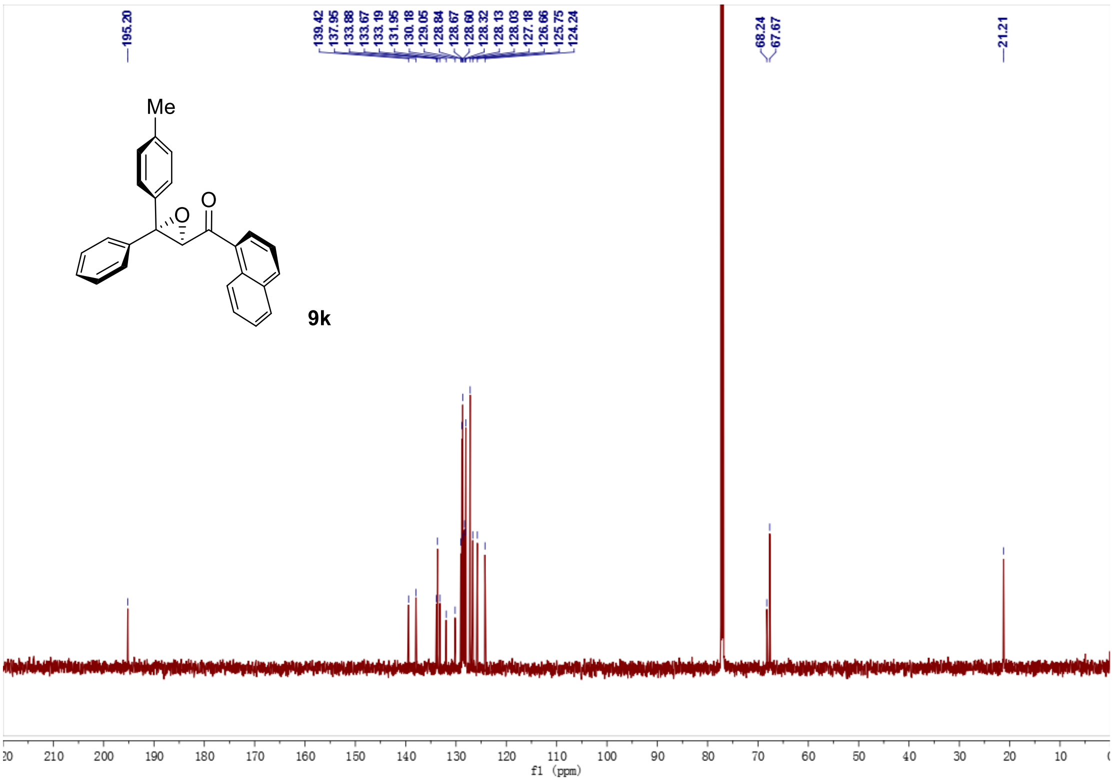

Supplementary Figure 330. ^{1}H NMR sepctra of (*R*)-3-Benzyl-1-phenylheptan-3-ol (**10a**)

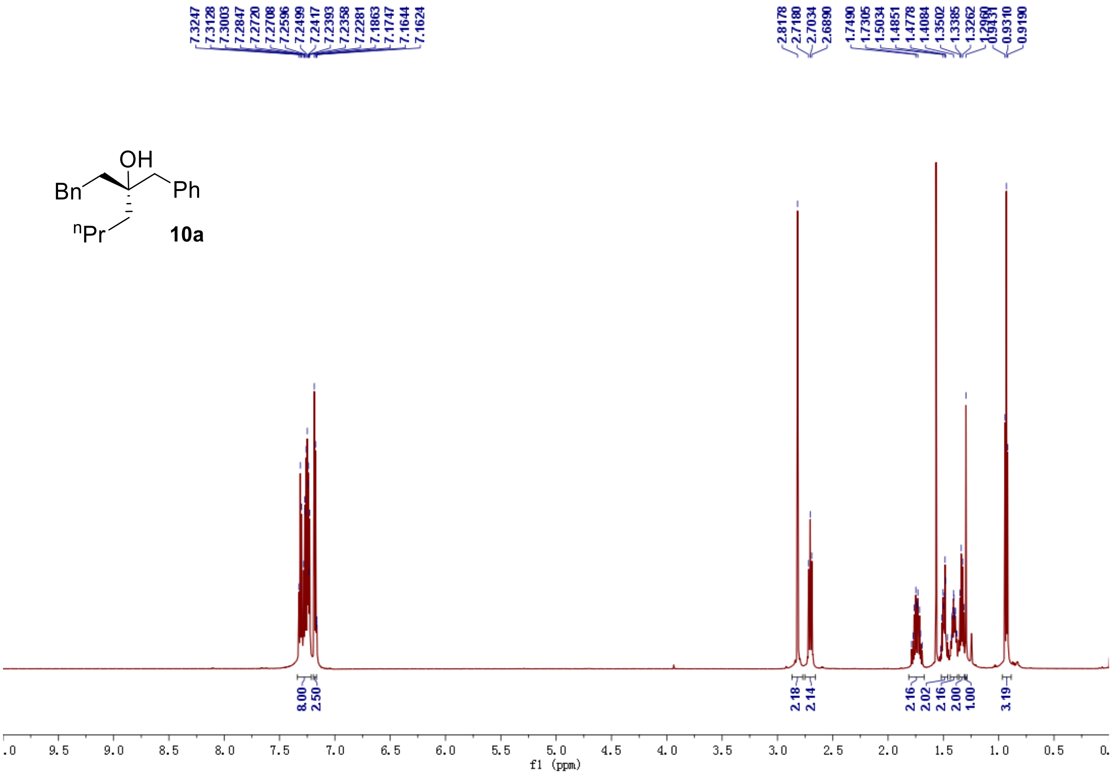

Supplementary Figure 331. ^{13}C NMR sepctra of (*R*)-3-Benzyl-1-phenylheptan-3-ol (10a)

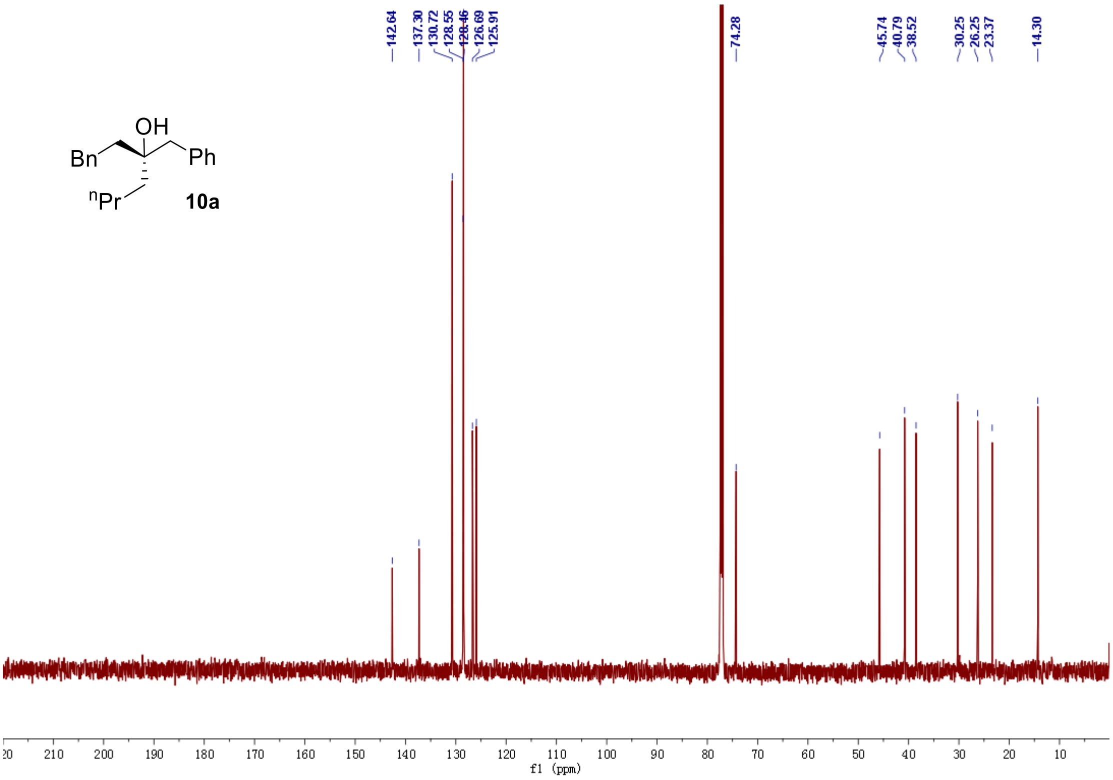

Supplementary Figure 332. ^{1}H NMR sepctra of (*S*)-2,3-Dimethyl-1-phenylbutan-2-ol (**10b**)

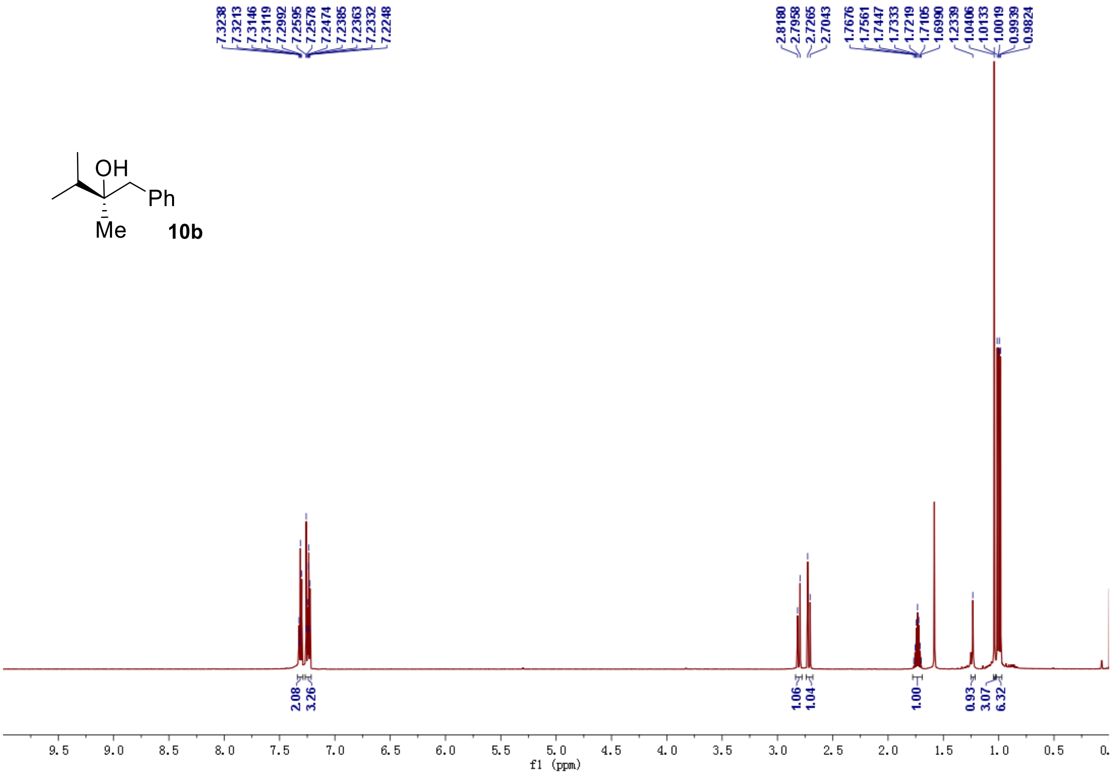

Supplementary Figure 333. ^{13}C NMR sepctra of (*S*)-2,3-Dimethyl-1-phenylbutan-2-ol (**10b**)

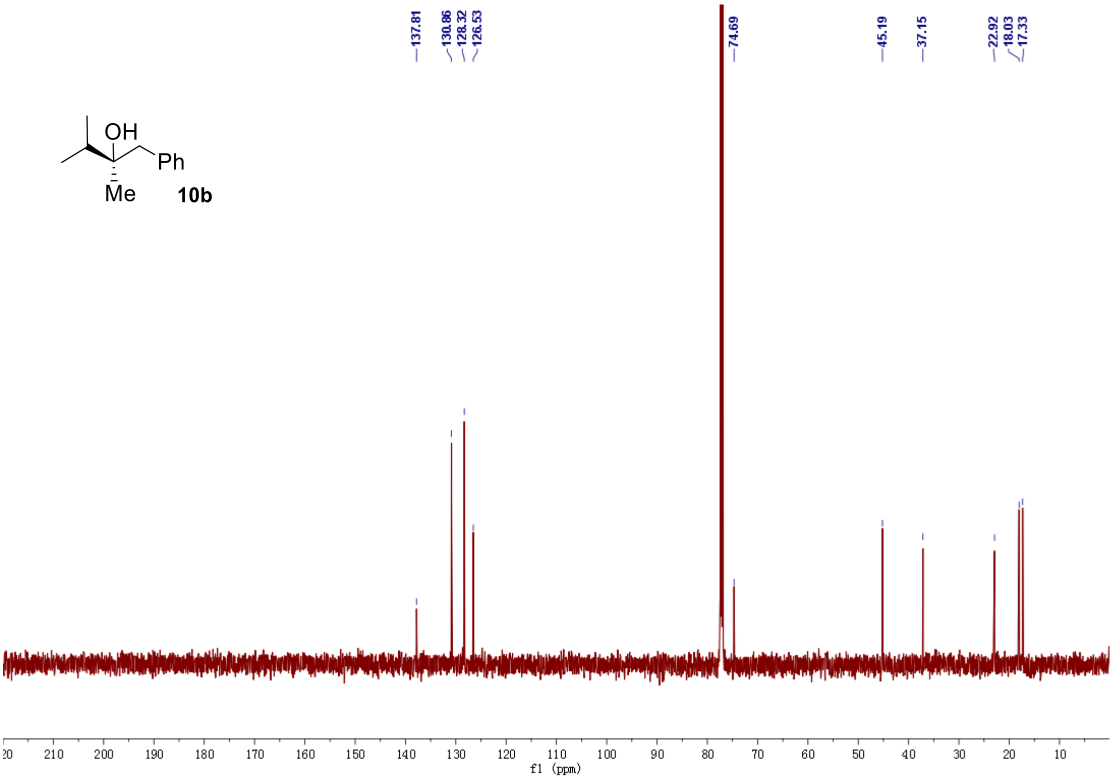

Supplementary Figure 334. ^{1}H NMR sepctra of (*R*)-(2-(Methoxymethyl)-2-methylpentyl)benzene (**10c**)

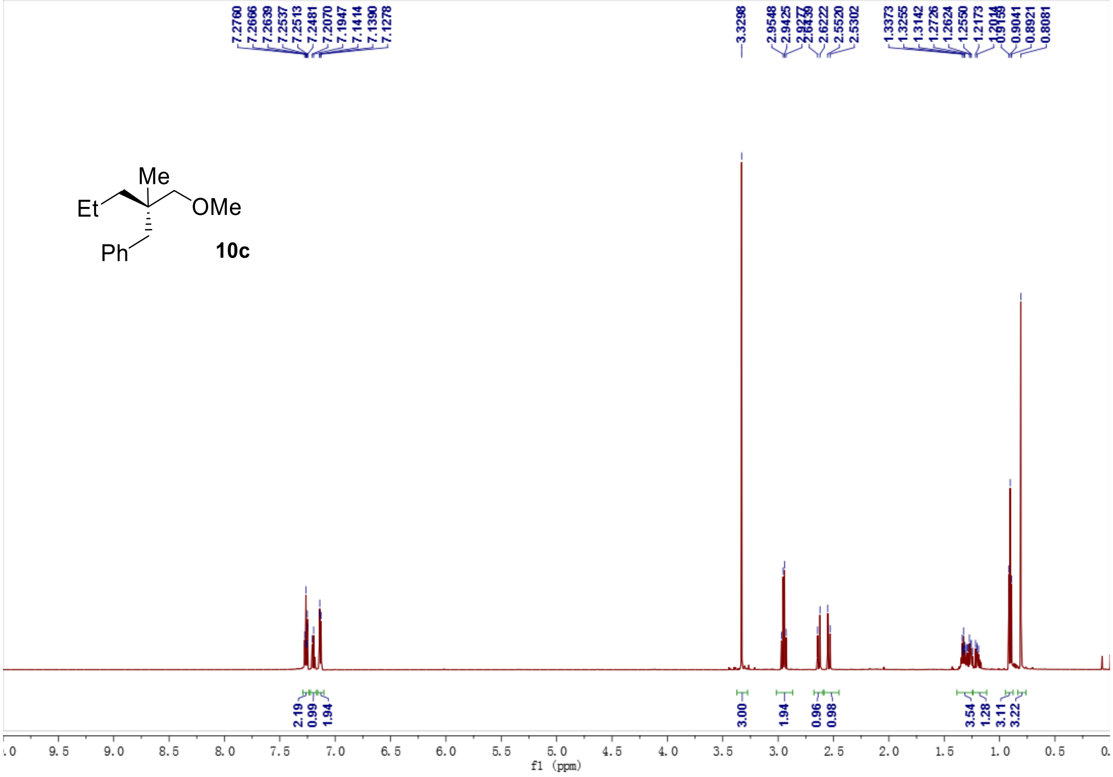

Supplementary Figure 335. ^{13}C NMR sepctra of (*R*)-(2-(Methoxymethyl)-2-methylpentyl)benzene (**10c**)

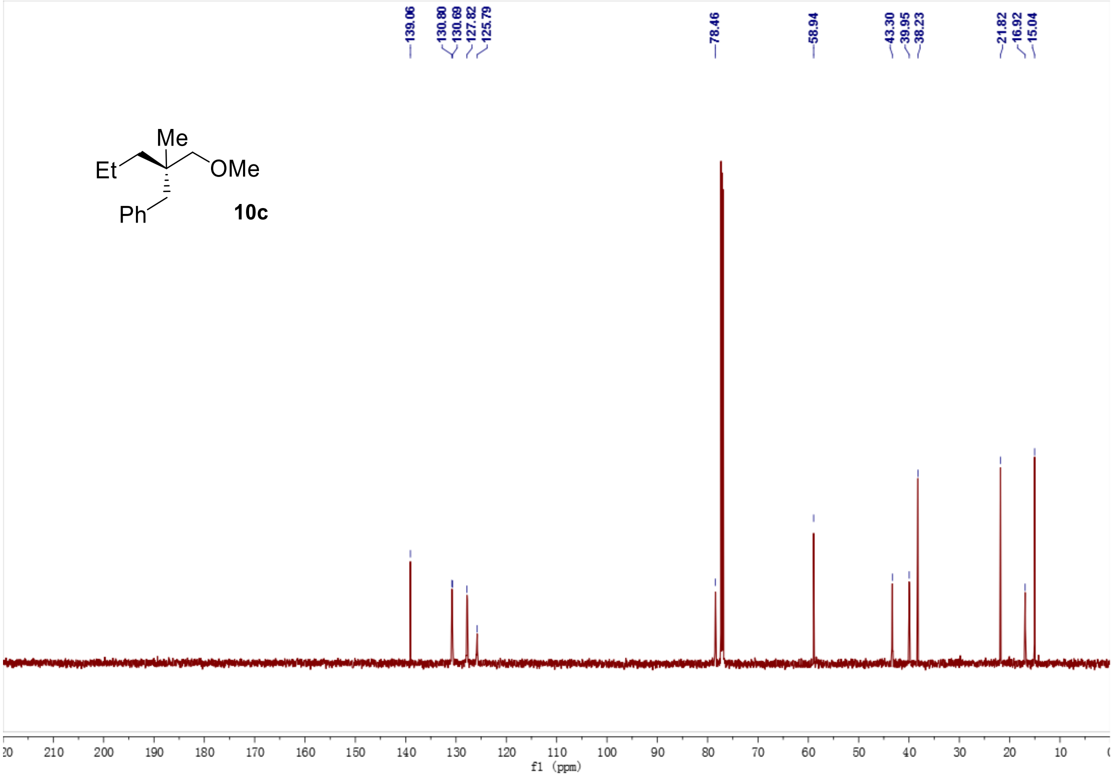

Supplementary Figure 336. ^{1}H NMR sepctra of (*S*)-2,3-Dimethyl-2-phenylbutan-1-ol (**10d**)

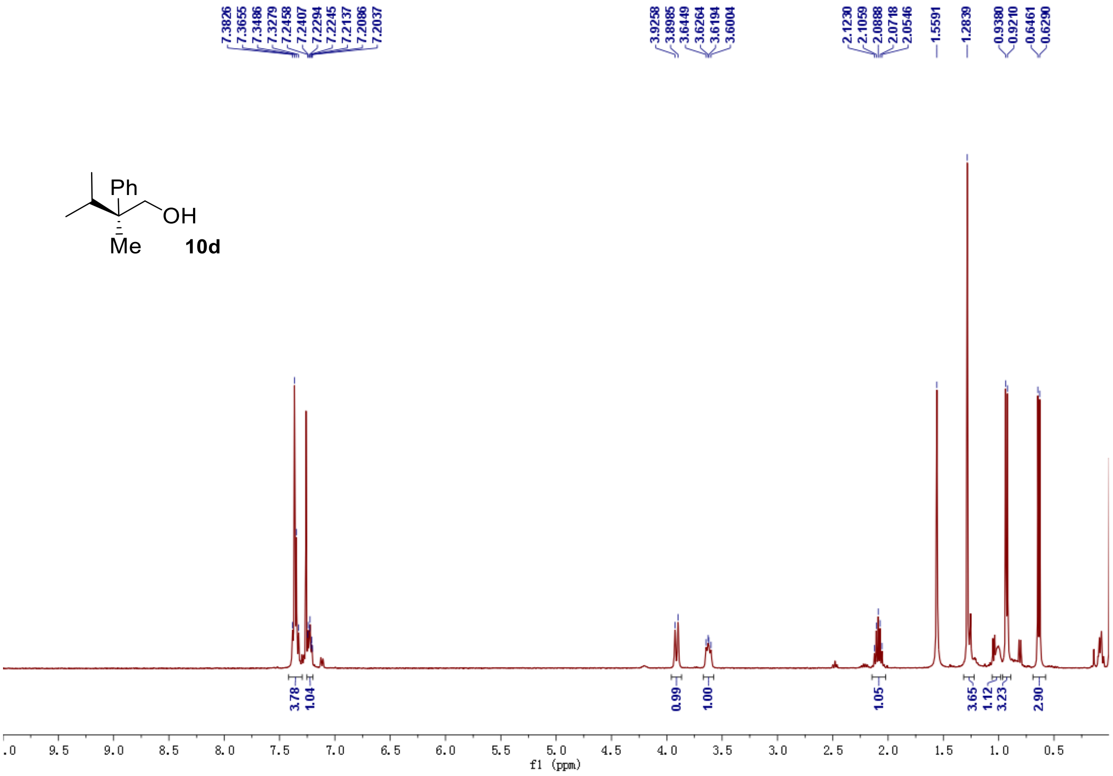

Supplementary Figure 337. ^{13}C NMR sepctra of (*S*)-2,3-Dimethyl-2-phenylbutan-1-ol (**10d**)

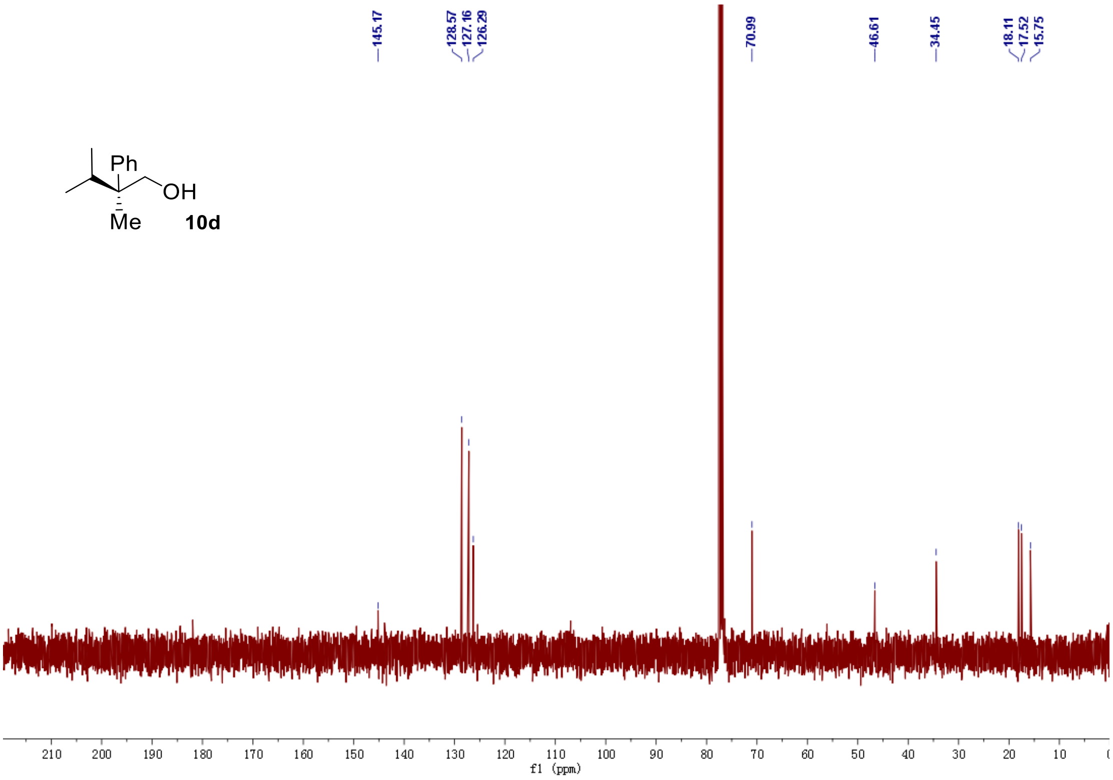

Supplementary Figure 338. ^{1}H NMR sepctra of (*S*)-2-Methyl-2,3-diphenylpropyl propionate (**10e**)

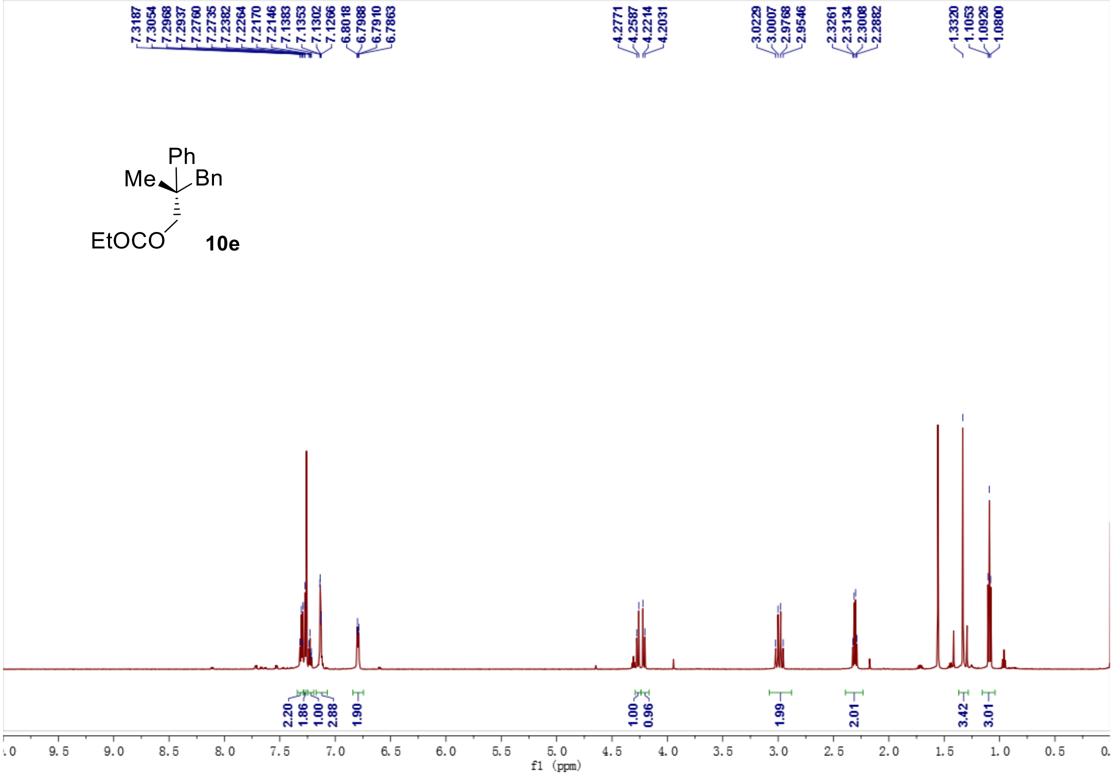

Supplementary Figure 339. ^{13}C NMR sepctra of (*S*)-2-Methyl-2,3-diphenylpropyl propionate (**10e**)

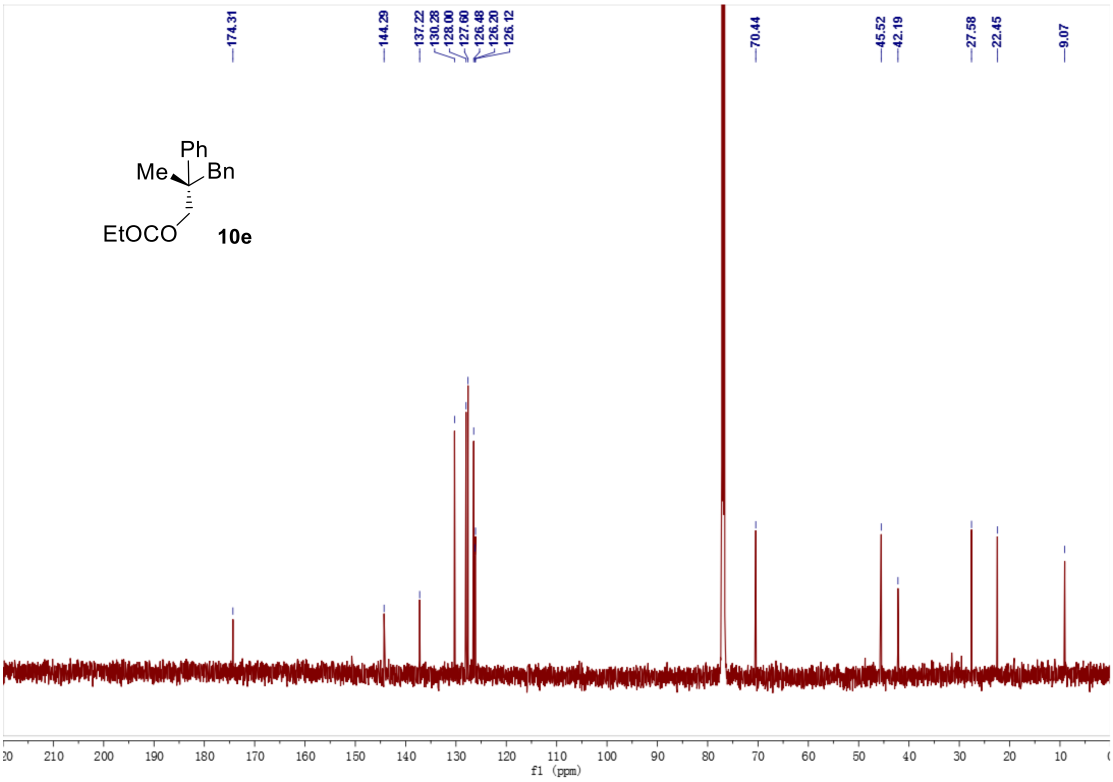

Supplementary Figure 340. ^{1}H NMR sepctra of (*S*)-2-(2-Benzyl-2-vinylpent-4-en-1-yl)naphthalene (**10f**)

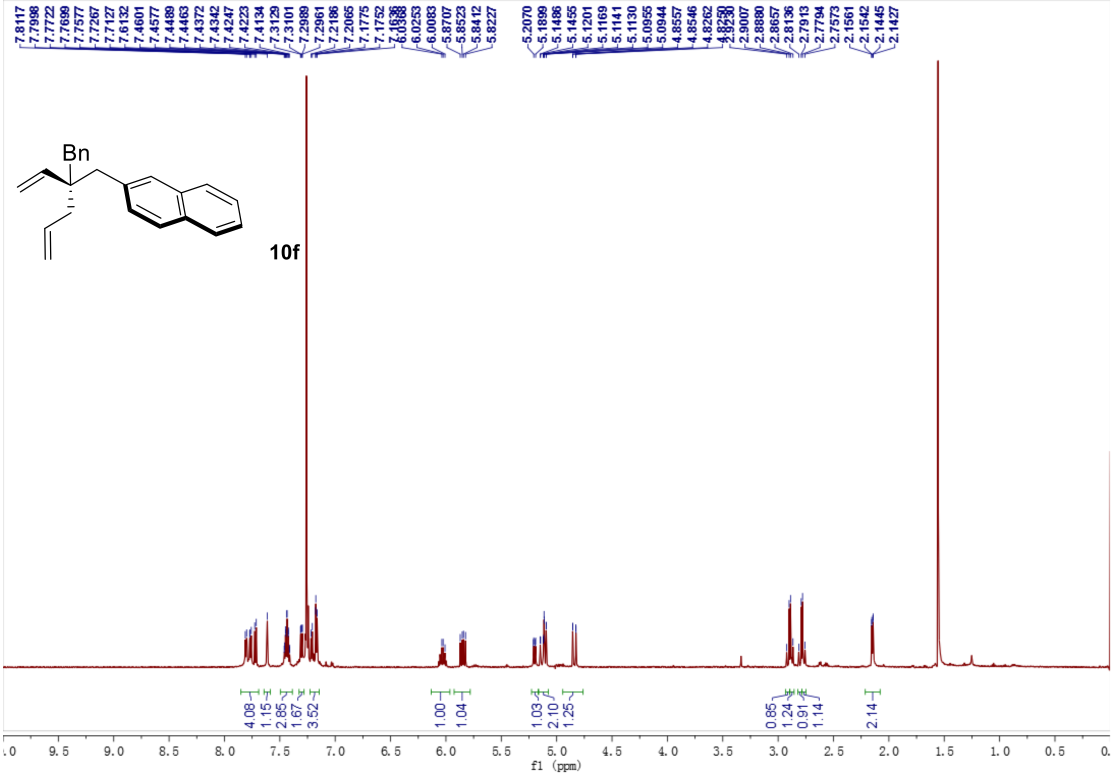

Supplementary Figure 341. ^{13}C NMR sepctra of (*S*)-2-(2-Benzyl-2-vinylpent-4-en-1-yl)naphthalene (**10f**)

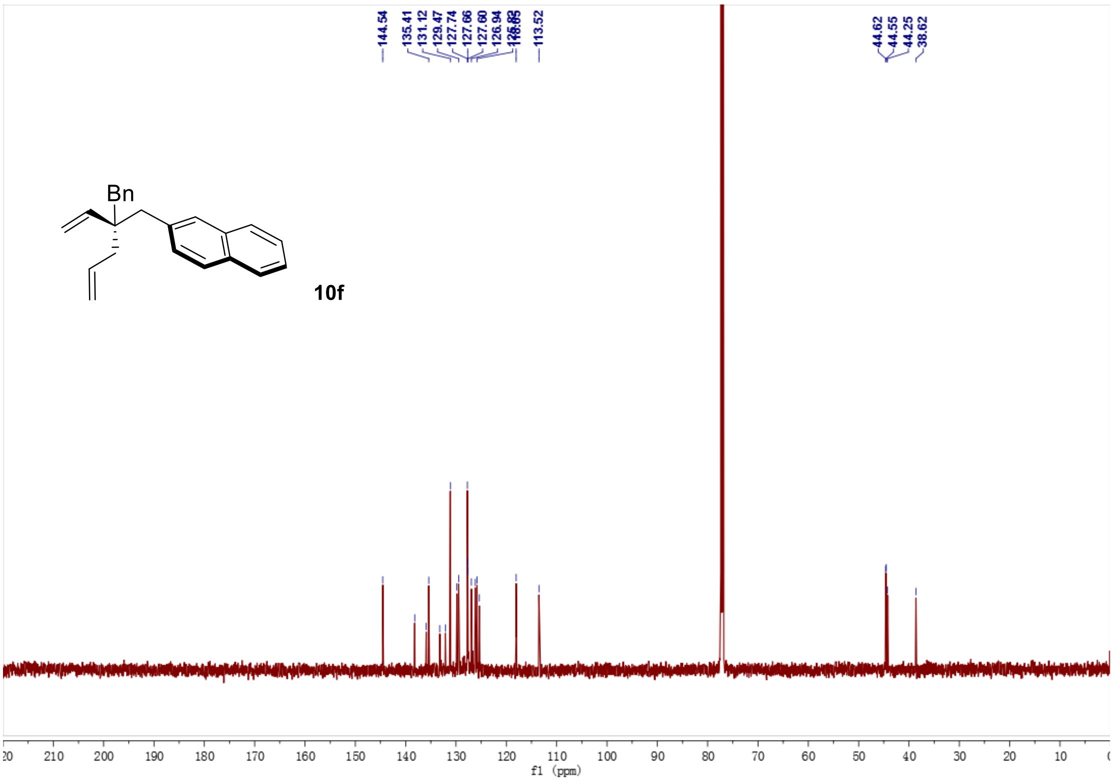

Supplementary Figure 342. ^{1}H NMR sepctra of (*S*)-2-Benzyl-2-(4-fluorobenzyl)butyl propionate (**10g**)

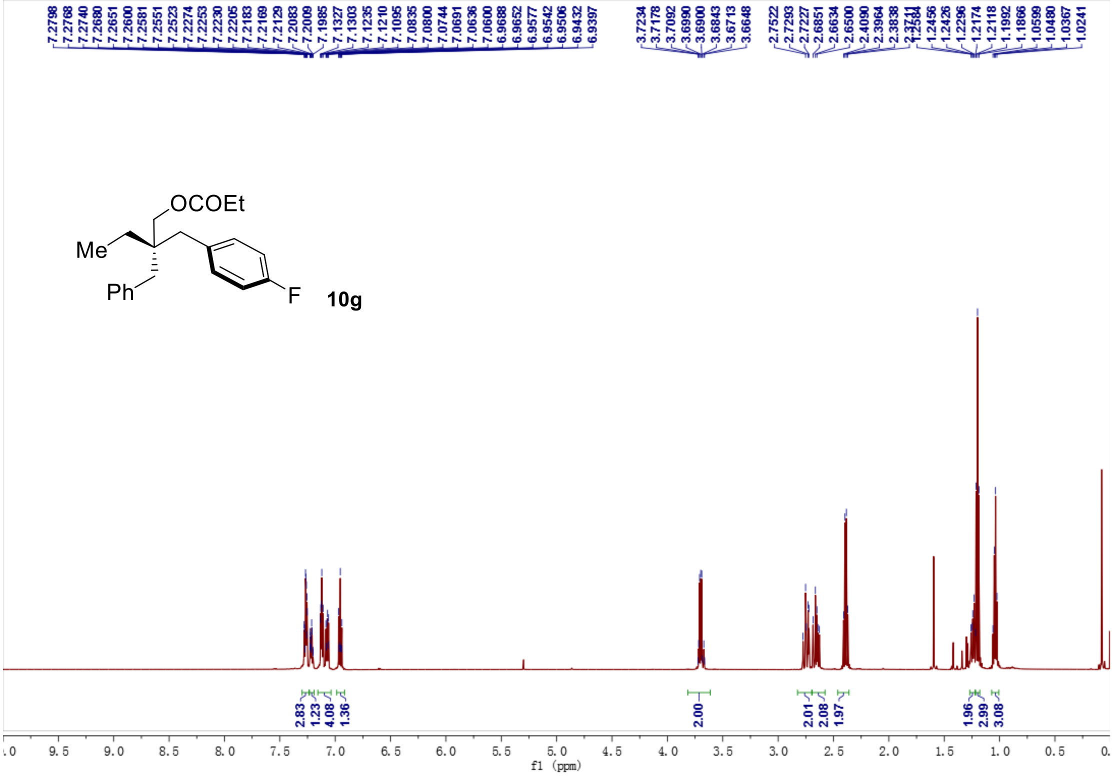

Supplementary Figure 343. ^{13}C NMR sepctra of (*S*)-2-Benzyl-2-(4-fluorobenzyl)butyl propionate (**10g**)

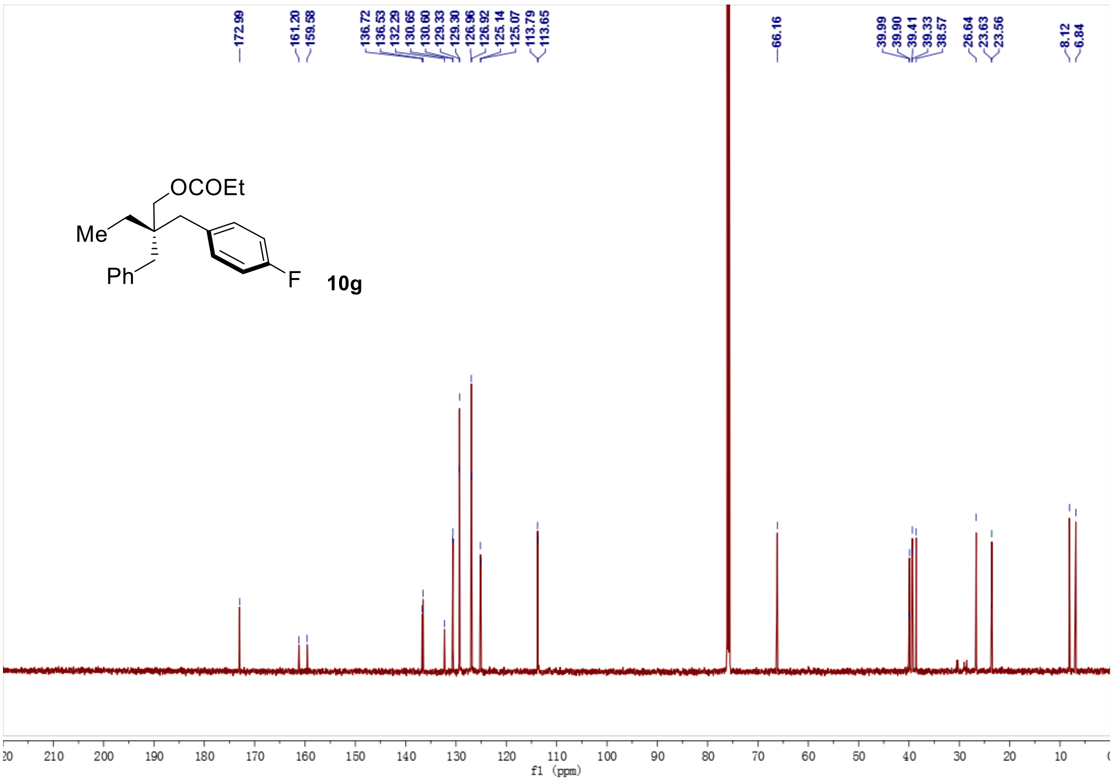

Supplementary Figure 344. ^{1}H NMR sepctra of (*S*)-Methyl 2-benzyl-2-(naphthalen-2-ylmethyl)pent-4-enoate (**10h**)

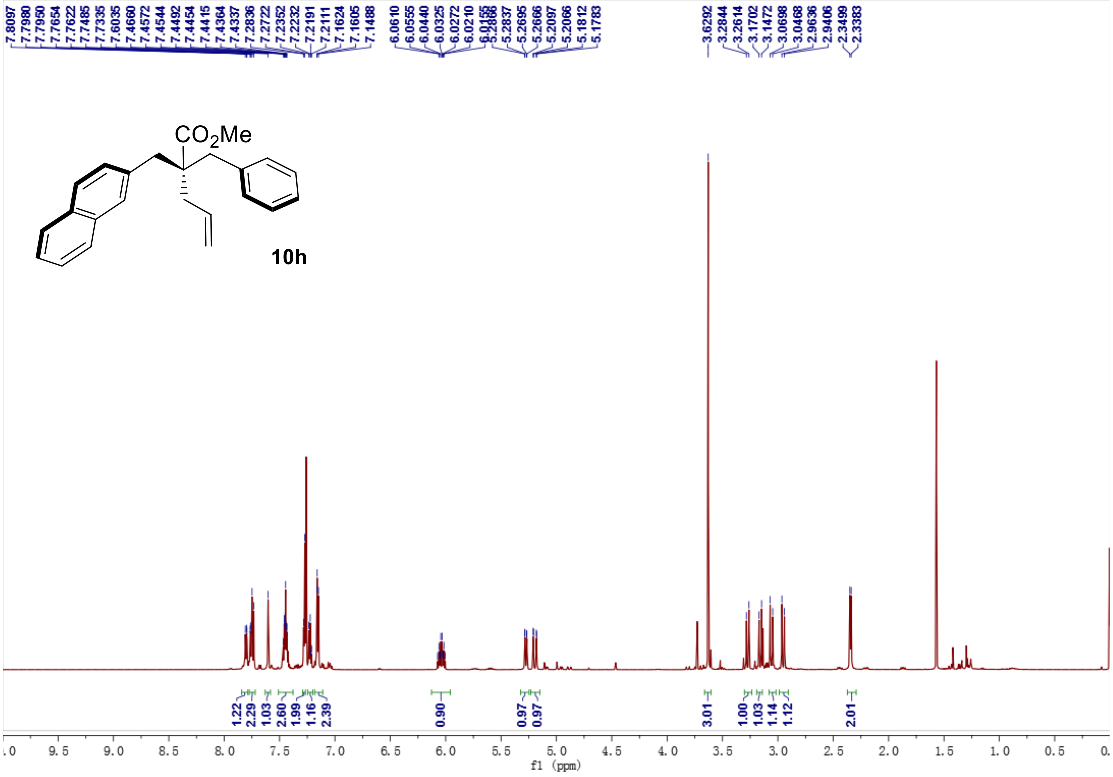

Supplementary Figure 345. ^{13}C NMR sepctra of (*S*)-Methyl 2-benzyl-2-(naphthalen-2-ylmethyl)pent-4-enoate (**10h**)

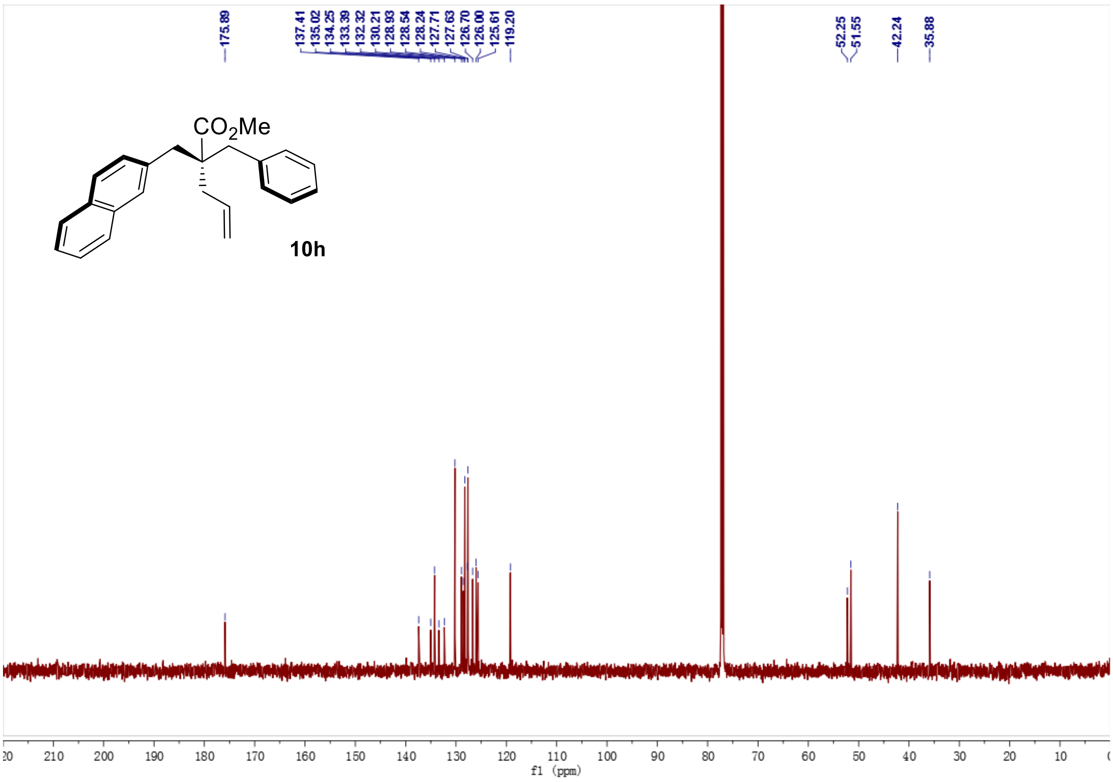

Supplementary Figure 346. ^{1}H NMR sepctra of (1*S*,2*R*,3*S*)-3-Azido-3-pentyl-1-phenylnonane-1,2-diol (**11a**)

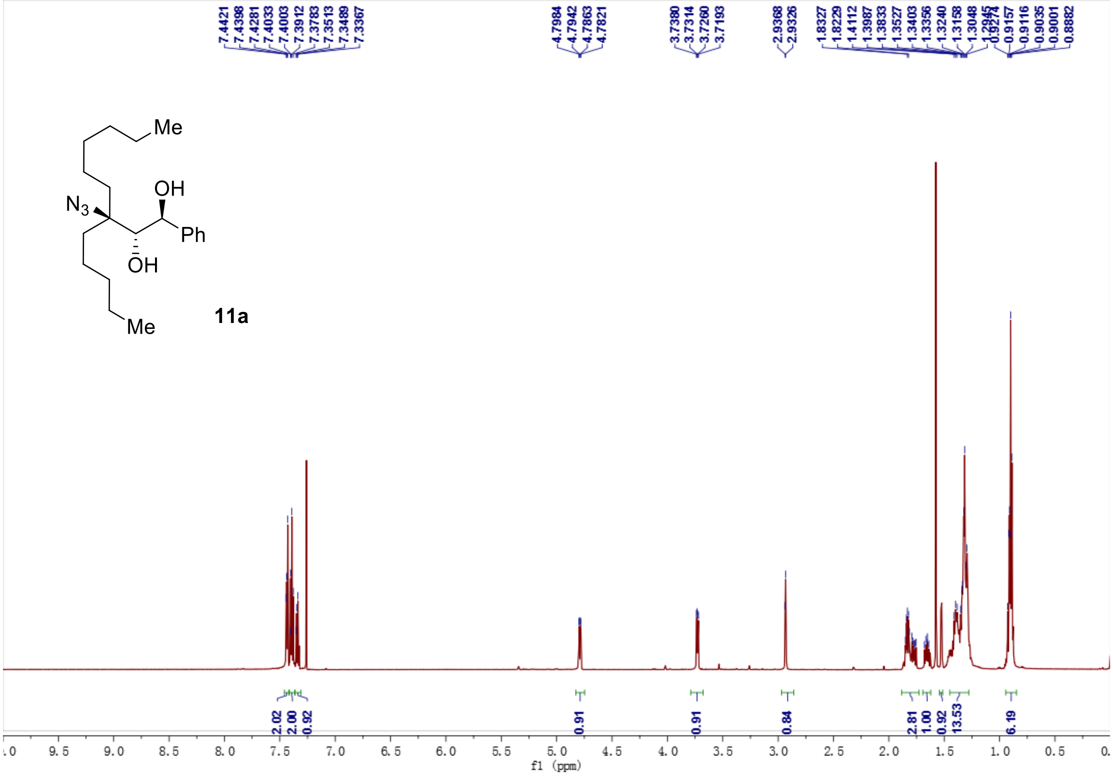

Supplementary Figure 347. ^{13}C NMR sepctra of (1*S*,2*R*,3*S*)-3-Azido-3-pentyl-1-phenylnonane-1,2-diol (**11a**)

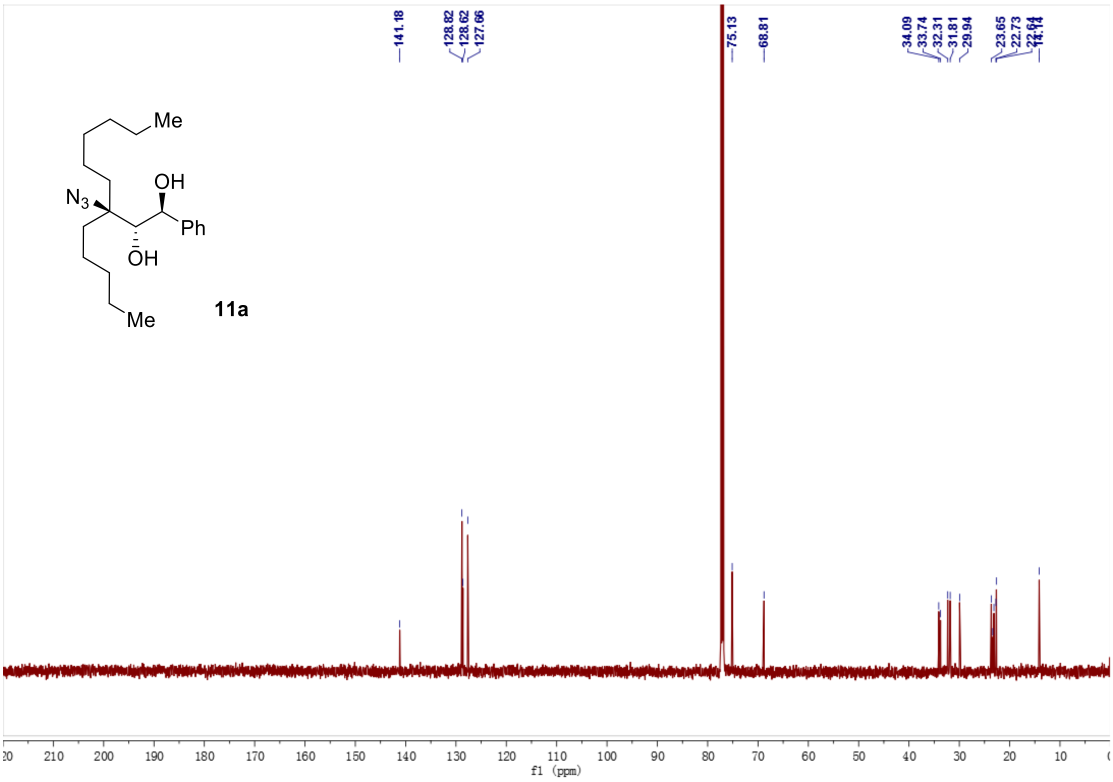

Supplementary Figure 348. ^{1}H NMR sepctra of (*R*)-Ethyl 2-((*R*)-amino(naphthalen-2-yl)methyl)-2-methylpentanoate (**11b**)

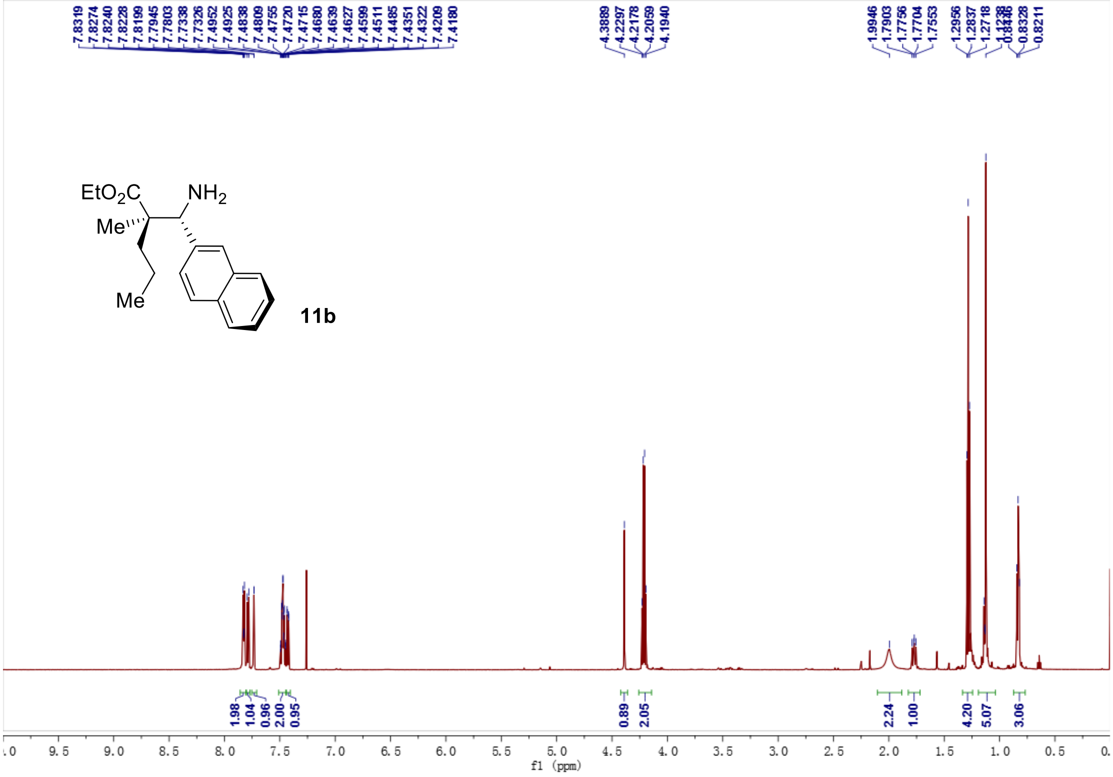

Supplementary Figure 349. ^{13}C NMR sepctra of (*R*)-Ethyl 2-((*R*)-amino(naphthalen-2-yl)methyl)-2-methylpentanoate (**11b**)

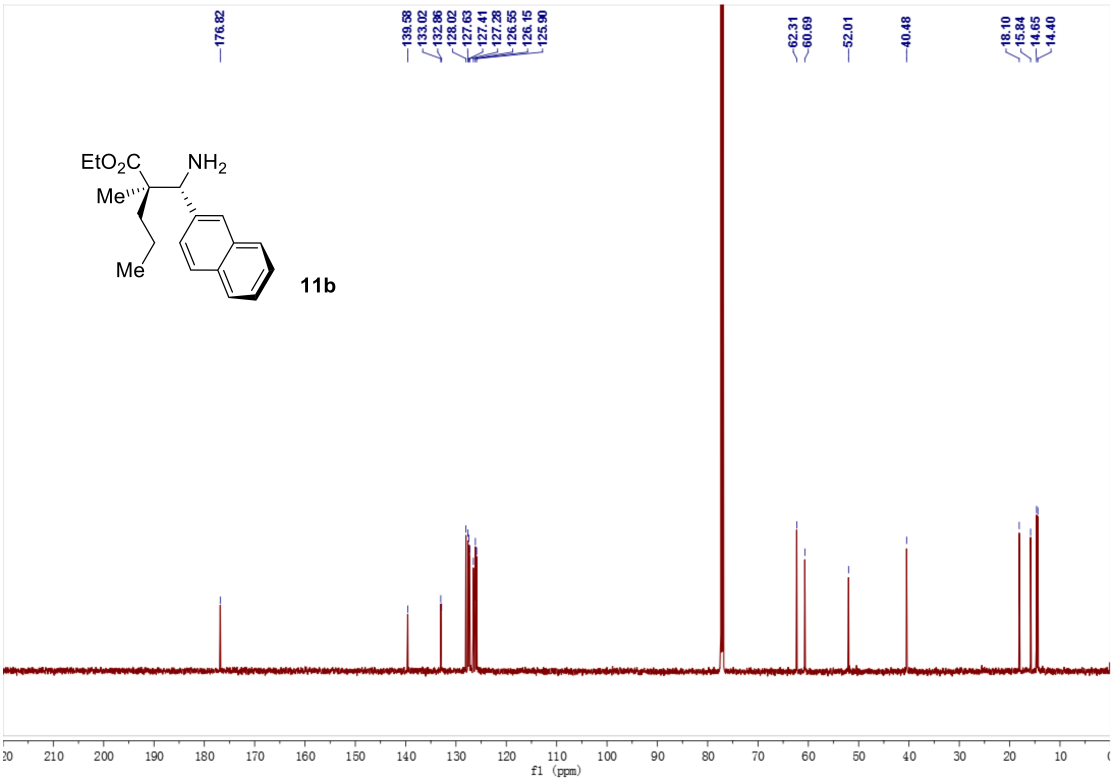

Supplementary Figure 350. ^{1}H NMR sepctra of (1*R*,3*S*)-1,3-Diphenyl-1-(*o*-tolyl)-1,3-dihydroisobenzofuran (**11c**)

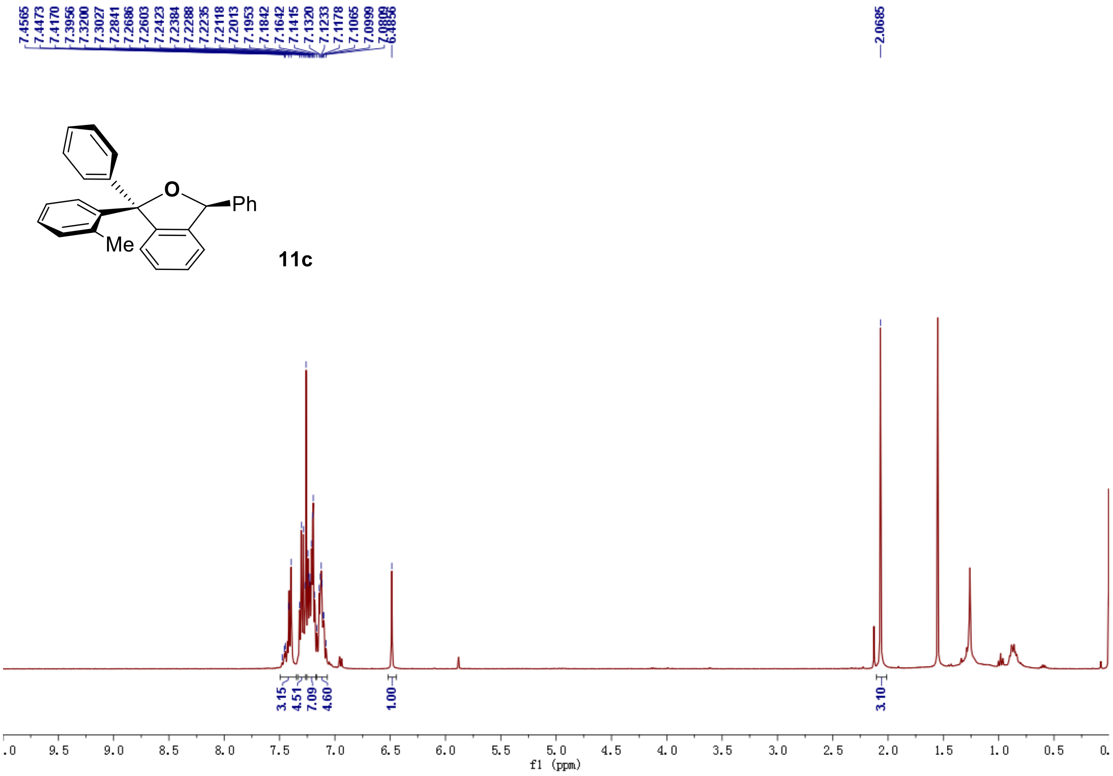

Supplementary Figure 351. ^{13}C NMR sepctra of (1*R*,3*S*)-1,3-Diphenyl-1-(*o*-tolyl)-1,3-dihydroisobenzofuran (**11c**)

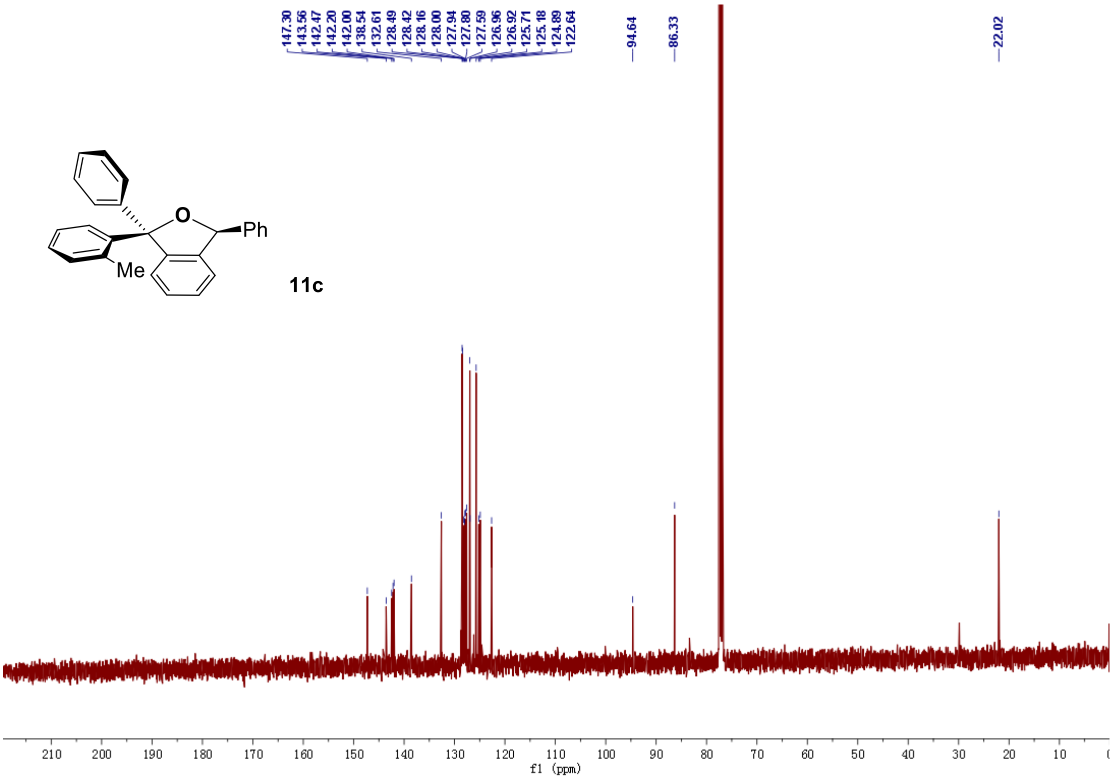

Supplementary Figure 352. ^{1}H NMR sepctra of Methyl(1*S*,2*R*)-2-allyl-1-(4-chlorophenyl)-2,3-dihydro-1*H*-cyclopenta[*a*]naphthalene-2-carboxylate (**11d**)

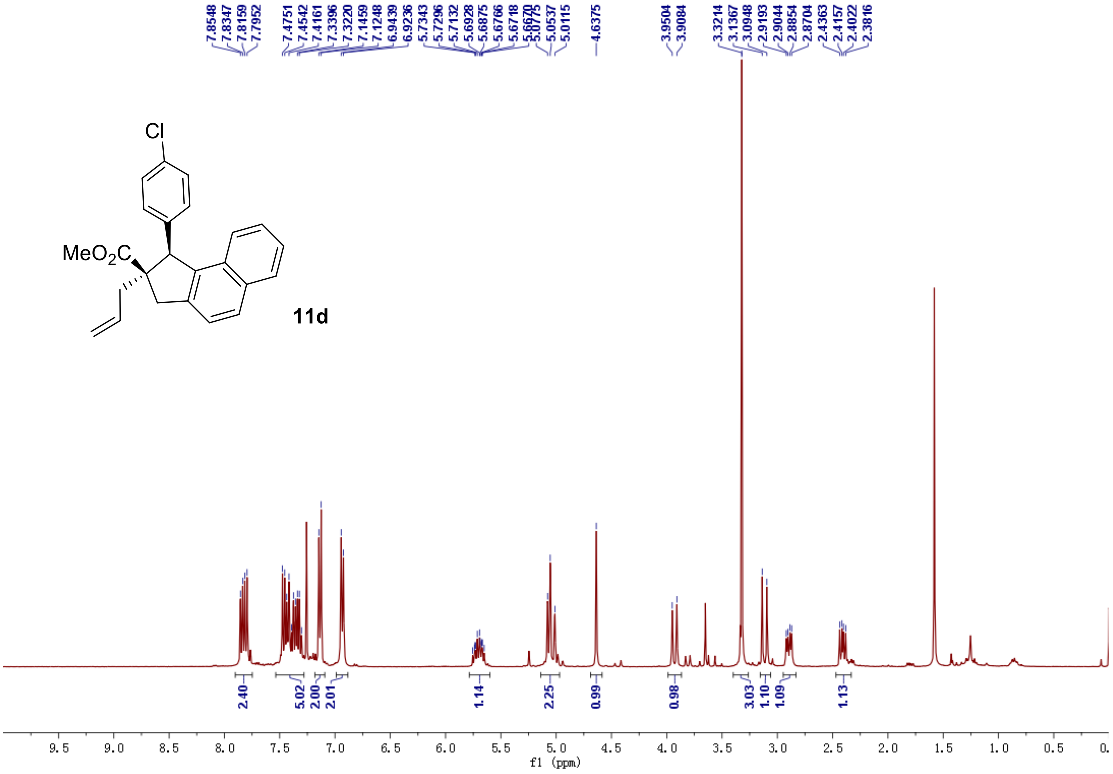

Supplementary Figure 353. ^{13}C NMR sepctra of Methyl(1*S*,2*R*)-2-allyl-1-(4-chlorophenyl)-2,3-dihydro-1*H*-cyclopenta[*a*]naphthalene-2-carboxylate (**11d**)

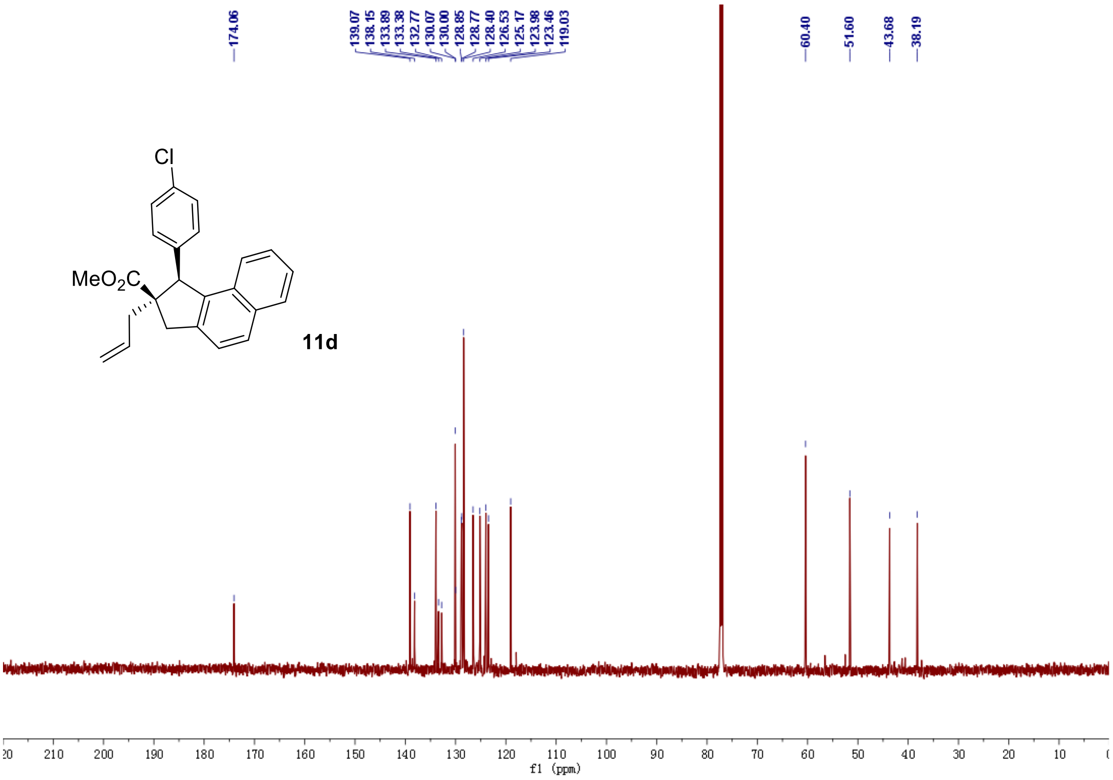

Supplementary Figure 354. NOE Spectrum of **11d**

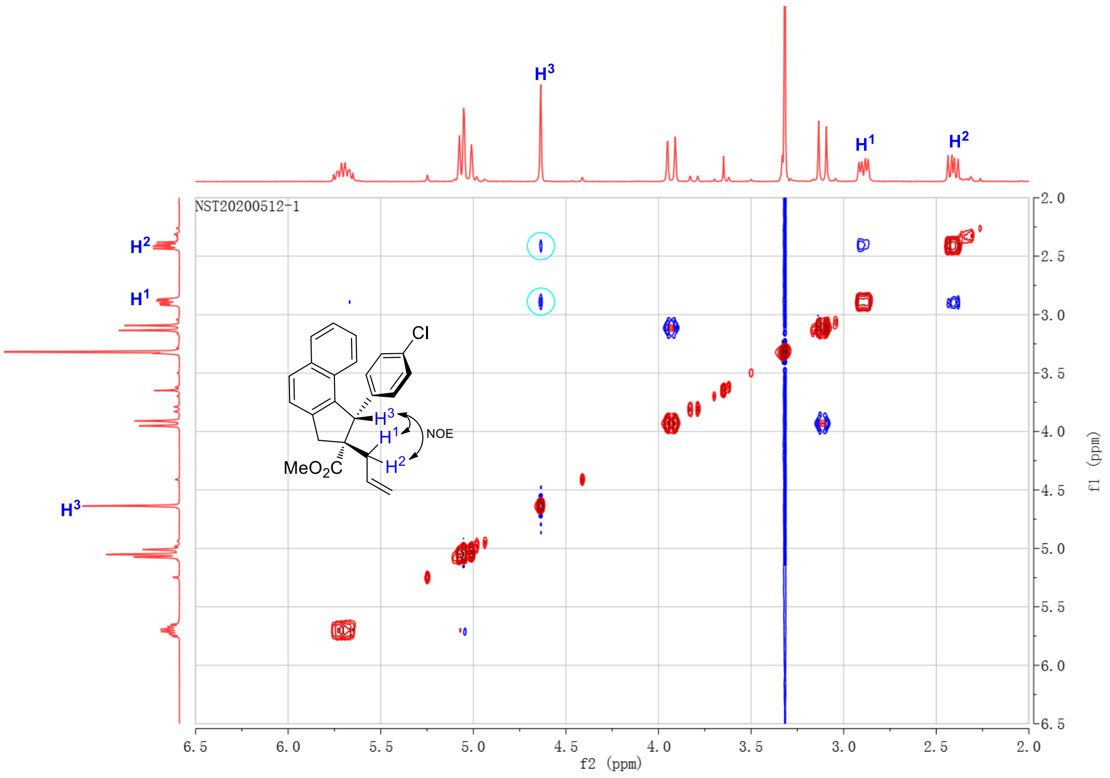

Supplementary Figure 355. ^{1}H NMR sepctra of (2*S*,3*R*)-2-(4-Methoxyphenyl)-3-methyl-2-(*p*-tolyl)oxirane (**11e**)

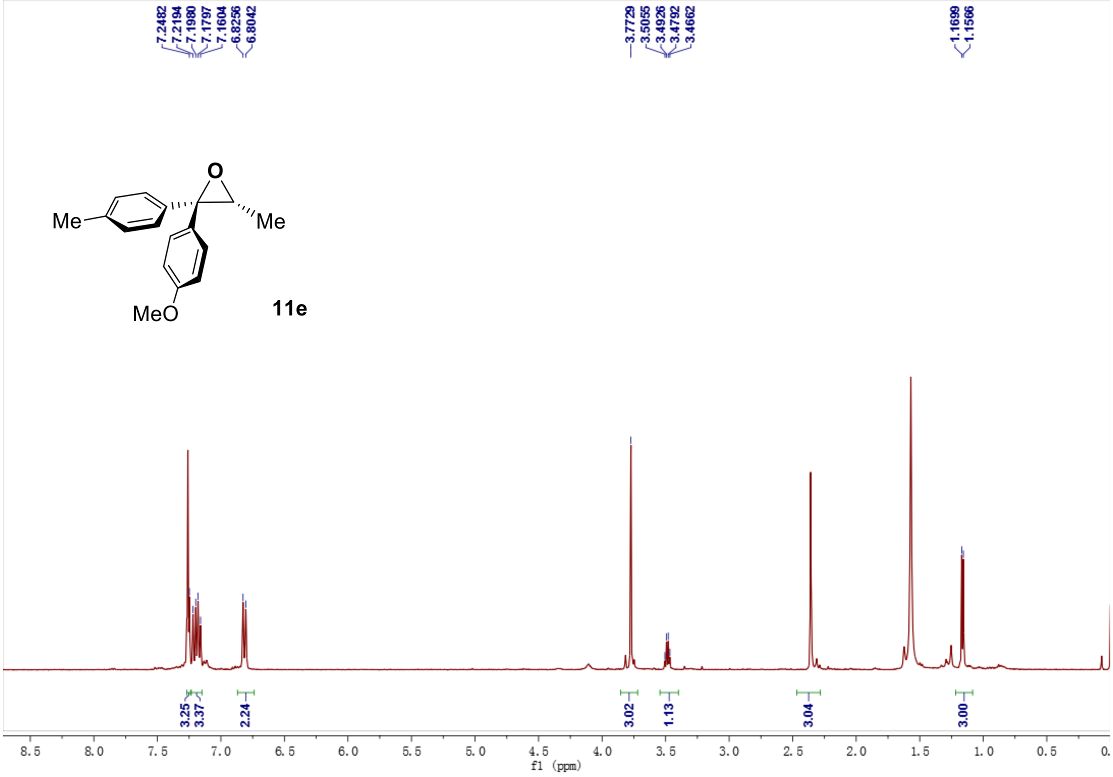

Supplementary Figure 356. ^{13}C NMR sepctra of (2*S*,3*R*)-2-(4-Methoxyphenyl)-3-methyl-2-(*p*-tolyl)oxirane (**11e**)

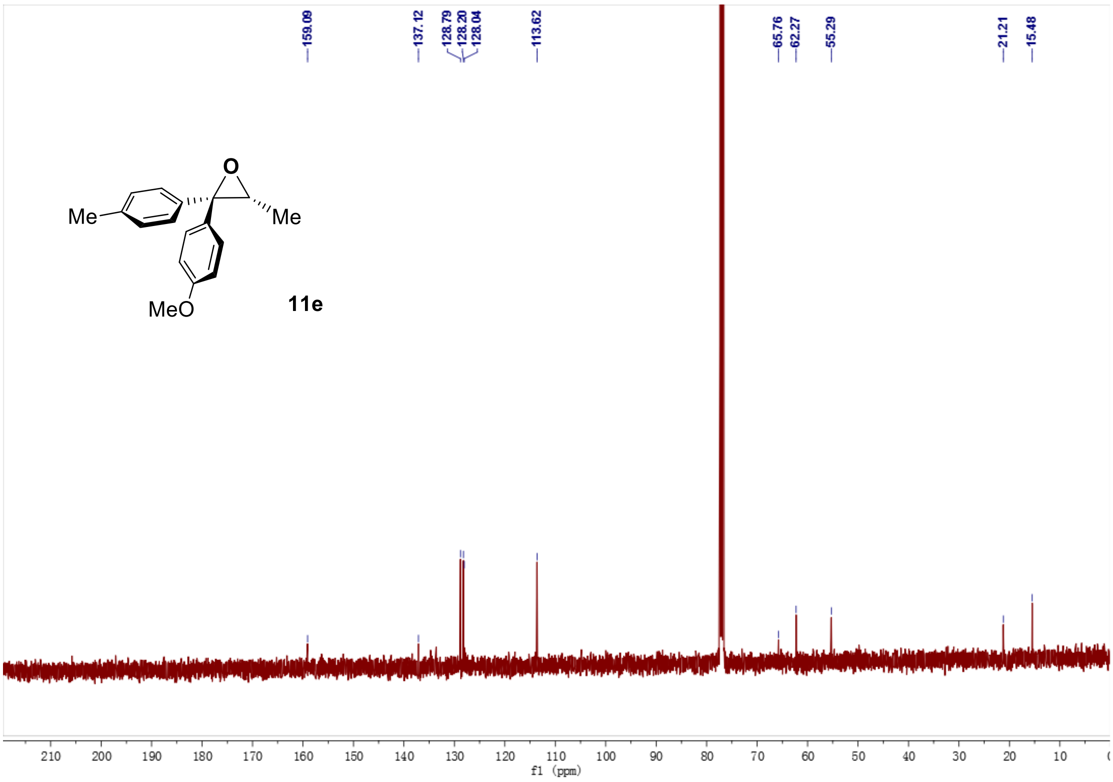

Supplementary Figure 357. ^{1}H NMR sepctra of (*S*)-2-Methyl-1,2-diphenylpent-4-en-1-one (**11f**)

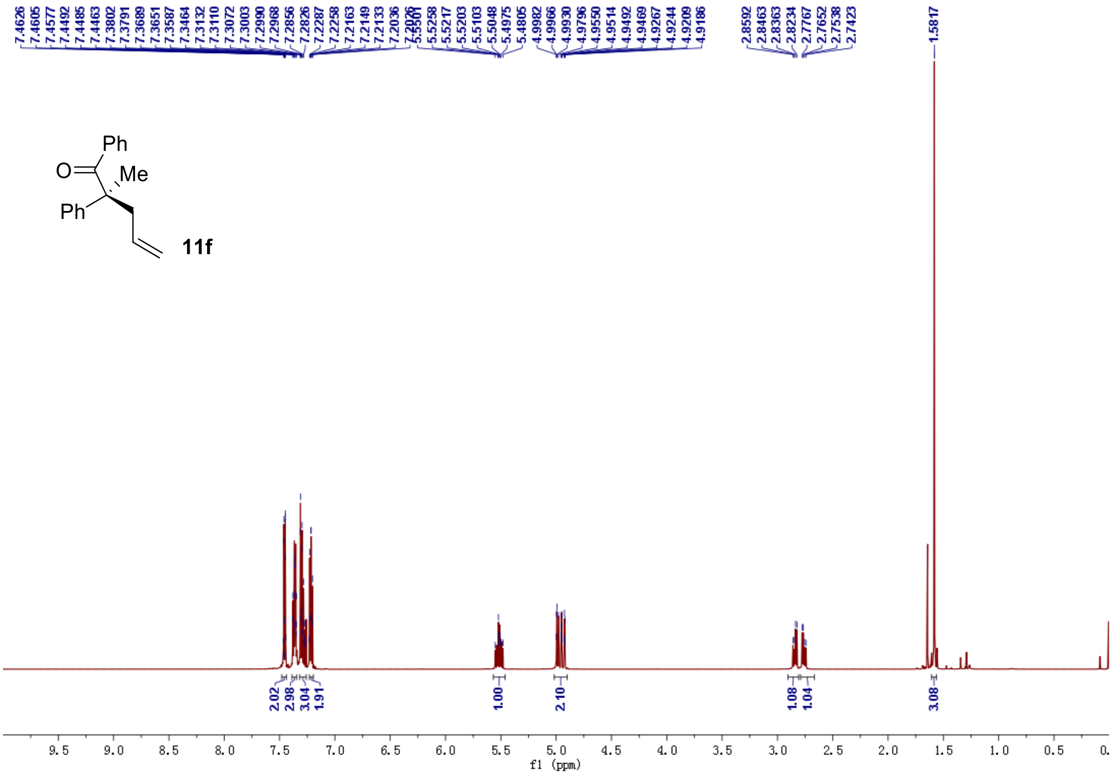

Supplementary Figure 358. ^{13}C NMR sepctra of (*S*)-2-Methyl-1,2-diphenylpent-4-en-1-one (**11f**)

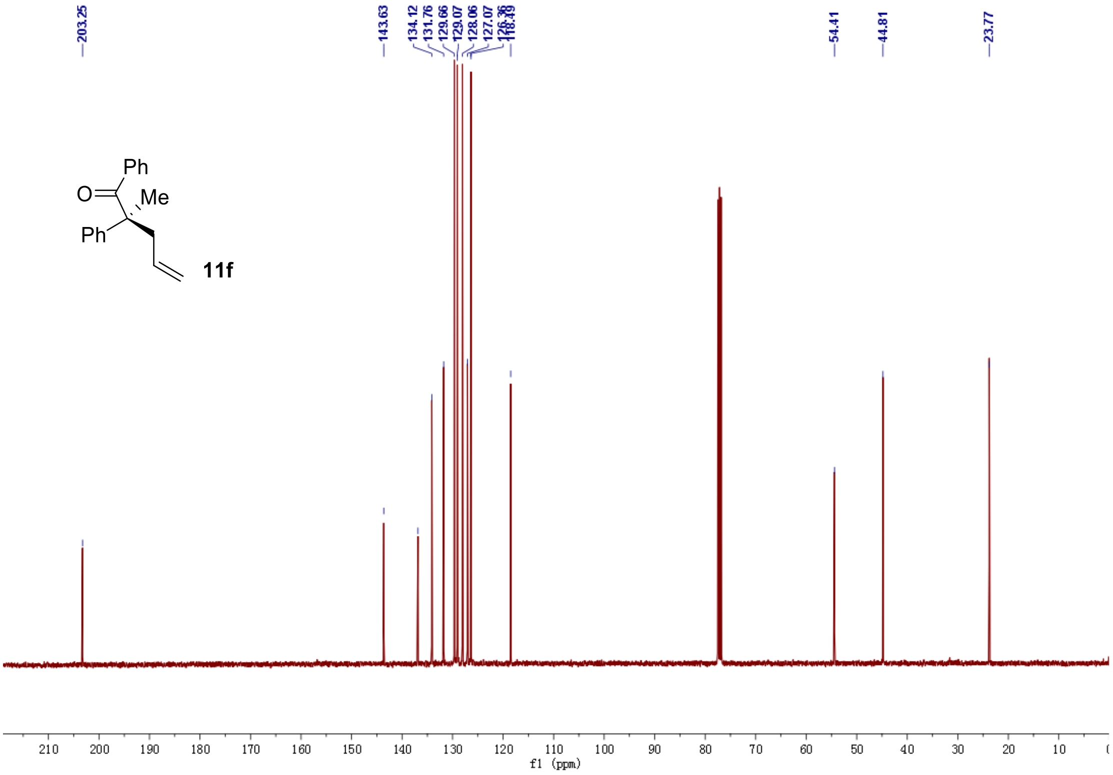

VIII. HPLC spectra for ee determination

(1*S*,2*S*)-1-(4-Bromophenyl)-1-(4-chlorophenyl)propane-1,2-diol (**1a**)

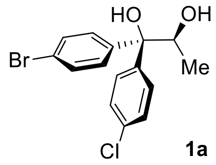

1a

<Chromatogram>

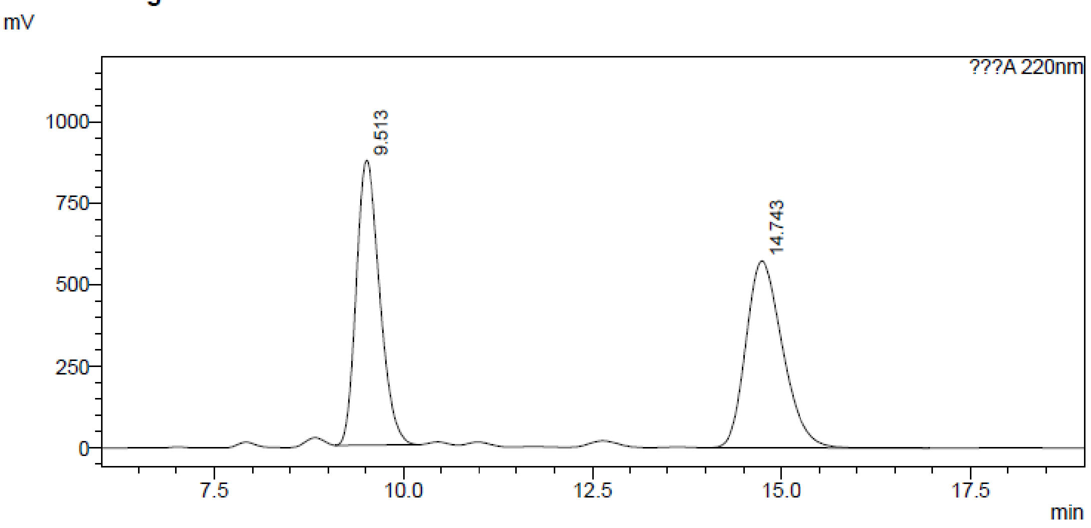

<Peak Table>

???A 220nm

Peak#	Ret. Time	Area	Height	Conc.	Unit	Mark	Name
1	9.513	18803542	872450	49.320			
2	14.743	19322029	572086	50.680			
Total		38125571	1444536				

<Chromatogram>

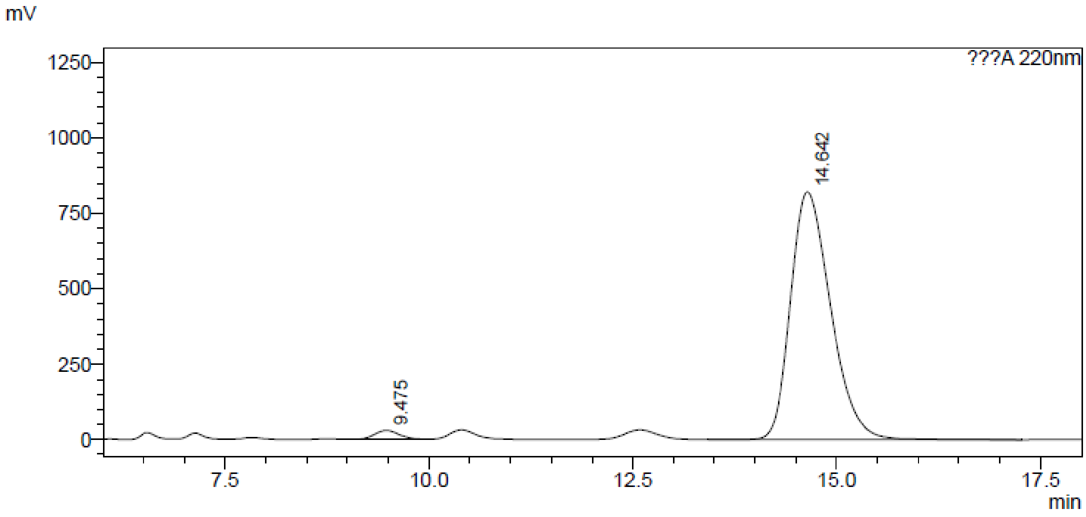

<Peak Table>

???A 220nm

Peak#	Ret. Time	Area	Height	Conc.	Unit	Mark	Name
1	9.475	599414	28721	2.089			
2	14.642	28101144	821963	97.911			
Total		28700558	850683				

(1*R*,2*R*)-1-(4-Bromophenyl)-1-(4-chlorophenyl)-1-hydroxypropan-2-yl propionate (2a)

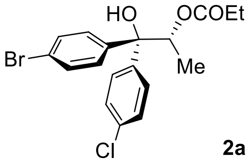

2a

<Chromatogram>

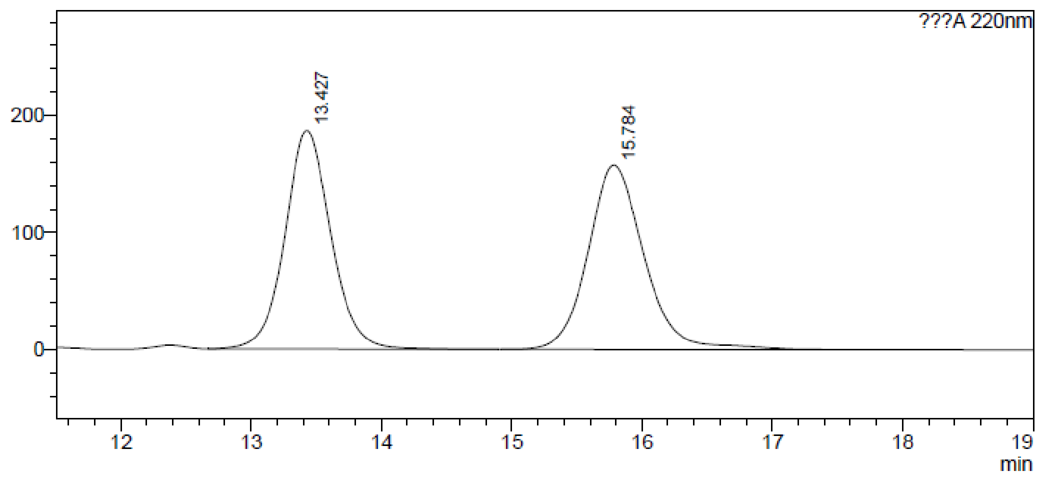

<Peak Table>

???A 220nm

Peak#	Ret. Time	Area	Height	Conc.	Unit	Mark	Name
1	13.427	4592088	187059	49.281			
2	15.784	4726110	157600	50.719			
Total		9318198	344659				

<Chromatogram>

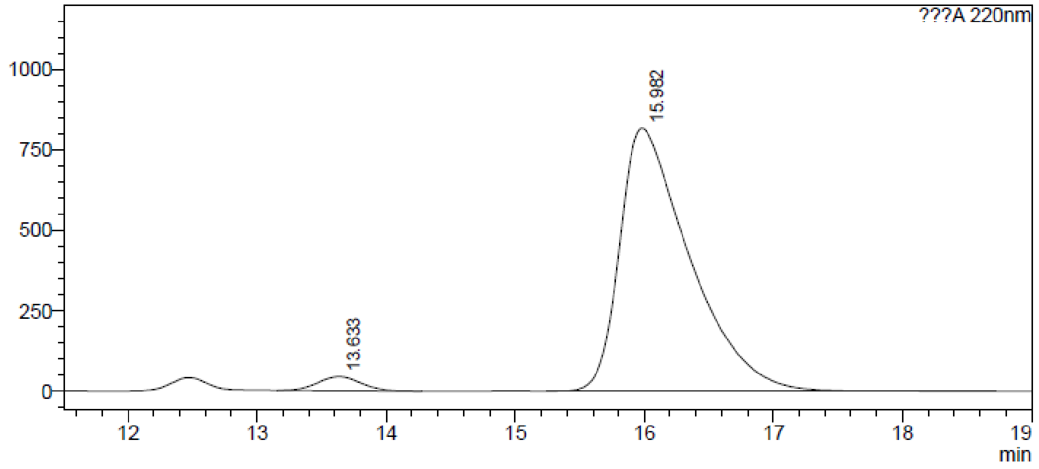

<Peak Table>

???A 220nm

Peak#	Ret. Time	Area	Height	Conc.	Unit	Mark	Name
1	13.633	1031986	44003	3.240			
2	15.982	30822945	816390	96.760			
Total		31854931	860393				

(1*R*,2*S*)-1-(4-Methoxyphenyl)-1-(*p*-tolyl)propane-1,2-diol (**1b**)

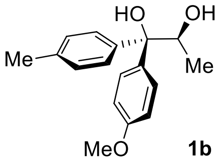

<Chromatogram>

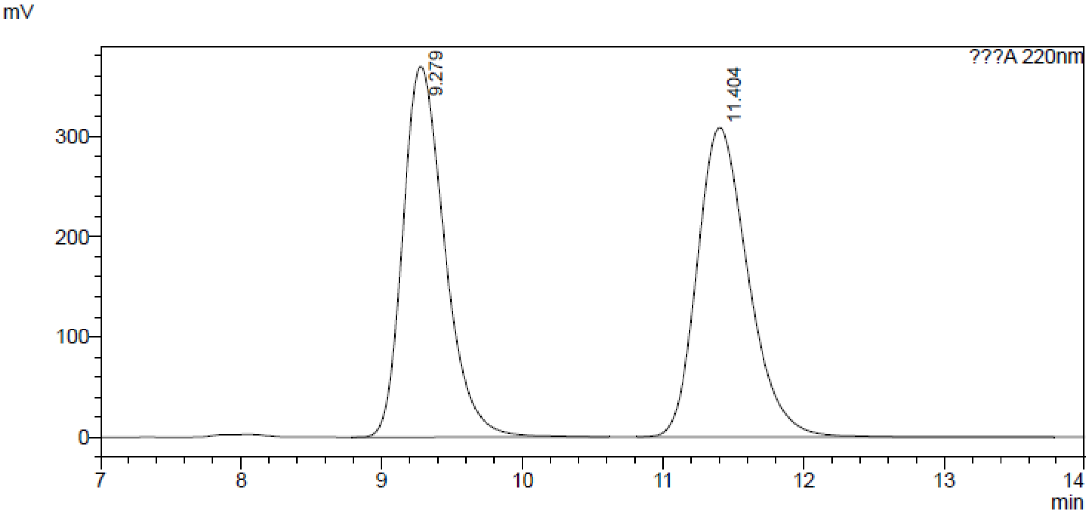

<Peak Table>

???A 220nm

Peak#	Ret. Time	Area	Height	Conc.	Unit	Mark	Name
1	9.279	7624709	369108	49.574			
2	11.404	7755735	308111	50.426			
Total		15380444	677220				

<Chromatogram>

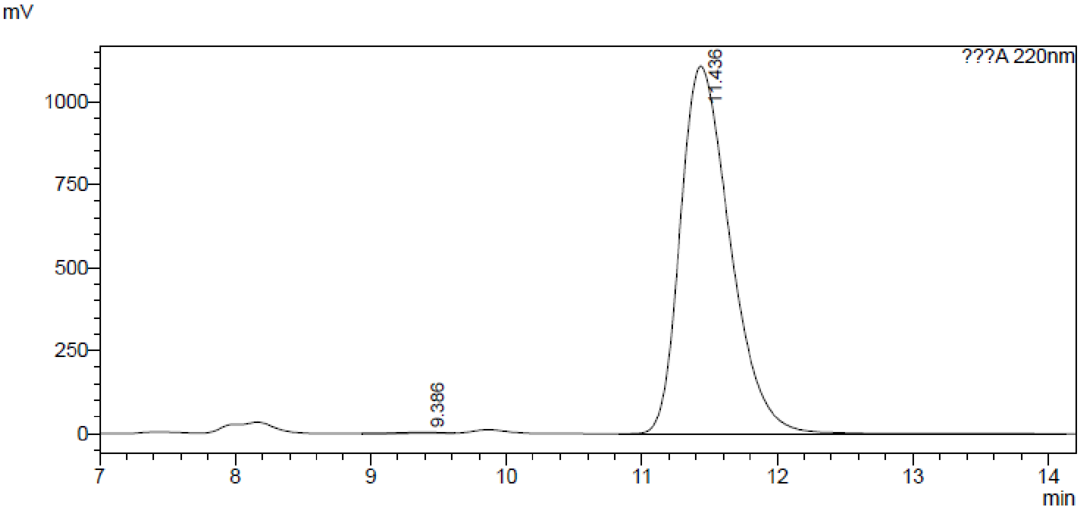

<Peak Table>

???A 220nm

Peak#	Ret. Time	Area	Height	Conc.	Unit	Mark	Name
1	9.386	52074	2460	0.184			
2	11.436	28255315	1105628	99.816			
Total		28307388	1108088				

(1*S*,2*R*)-1-Hydroxy-1-(4-methoxyphenyl)-1-(*p*-tolyl)propan-2-yl propionate (**2b**)

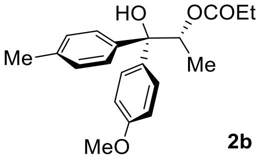

2b

<Chromatogram>

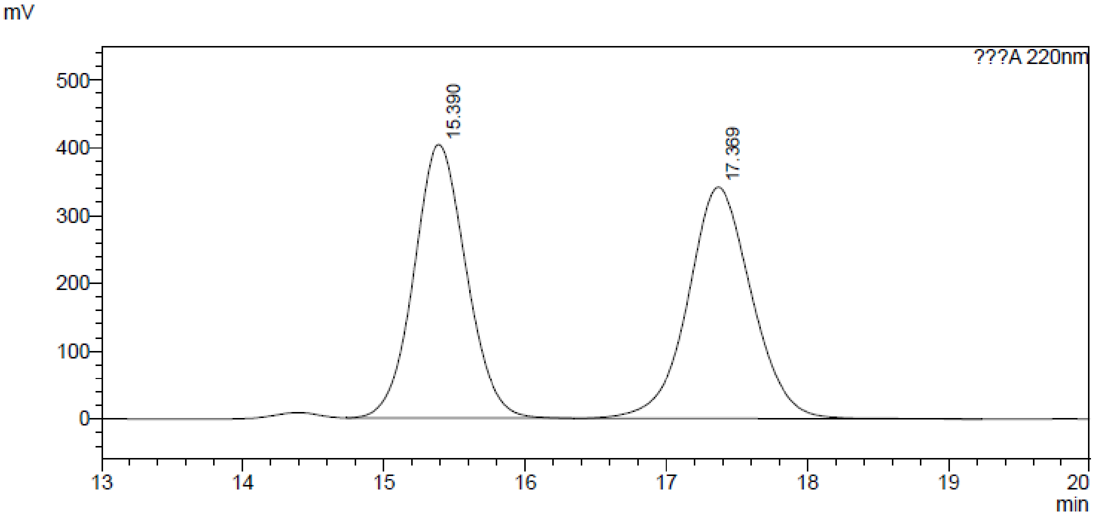

<Peak Table>

???A 220nm

Peak#	Ret. Time	Area	Height	Conc.	Unit	Mark	Name
1	15.390	10394816	403805	49.301			
2	17.369	10689459	341251	50.699		V	
Total		21084276	745056				

<Chromatogram>

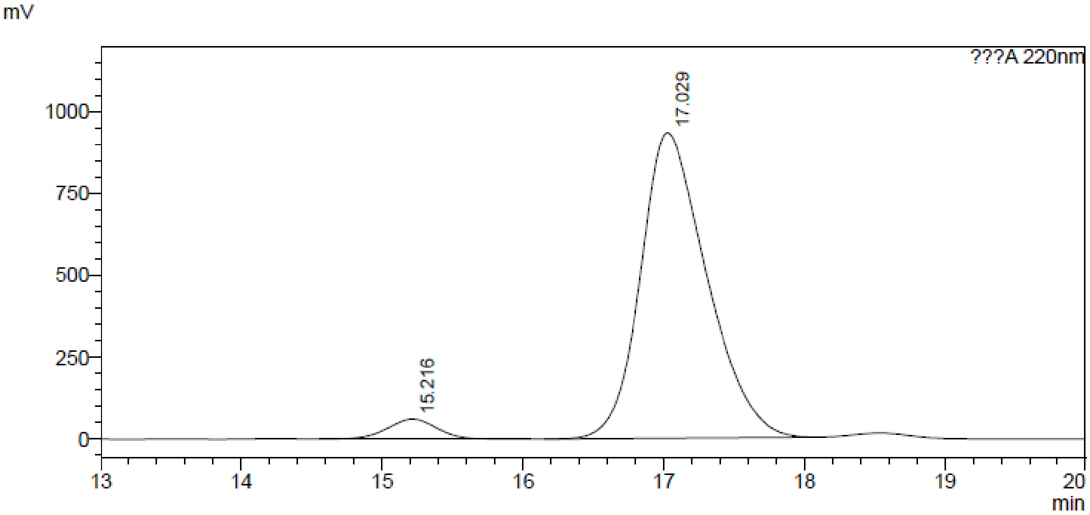

<Peak Table>

???A 220nm

Peak#	Ret. Time	Area	Height	Conc.	Unit	Mark	Name
1	15.216	1486947	60428	4.640			
2	17.029	30561922	932410	95.360			
Total		32048869	992838				

(1*S*,2*S*)-1-(4-Bromophenyl)-1-(4-methoxyphenyl)propane-1,2-diol (**1c**)

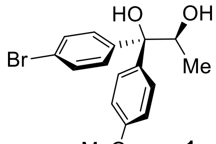

1c

<Chromatogram>

mV

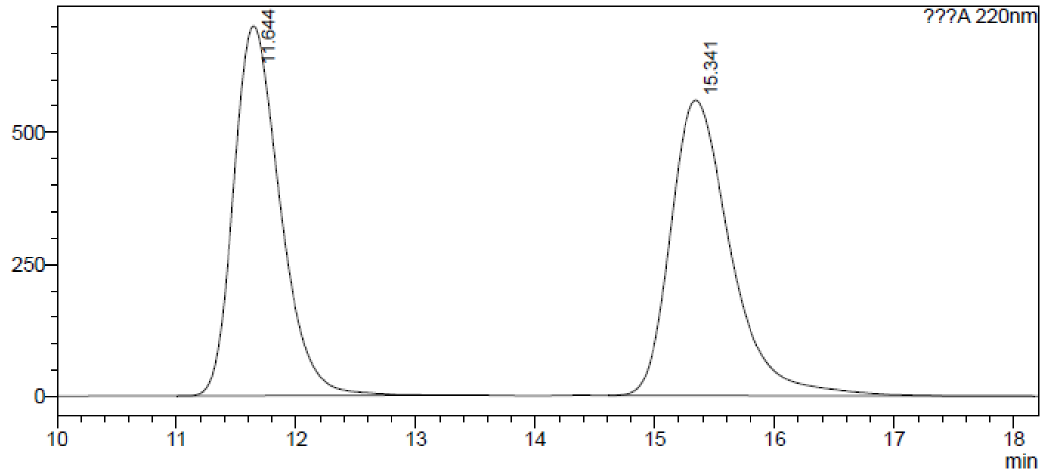

<Peak Table>

???A 220nm

Peak#	Ret. Time	Area	Height	Conc.	Unit	Mark	Name
1	11.644	18861730	698924	49.169			
2	15.341	19499625	558573	50.831			
Total		38361355	1257497				

<Chromatogram>

mV

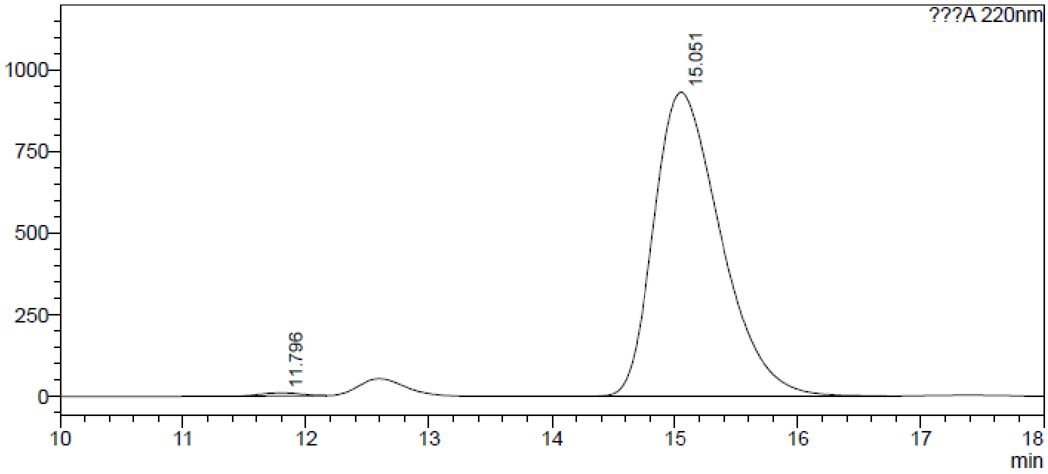

<Peak Table>

???A 220nm

Peak#	Ret. Time	Area	Height	Conc.	Unit	Mark	Name
1	11.796	217741	9732	0.617			
2	15.051	35084196	929065	99.383			
Total		35301937	938797				

(1*R*,2*R*)-1-(4-Bromophenyl)-1-hydroxy-1-(4-methoxyphenyl)propan-2-yl propionate (**2c**)

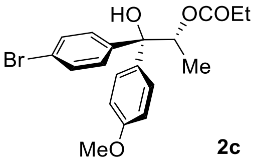

2c

<Chromatogram>

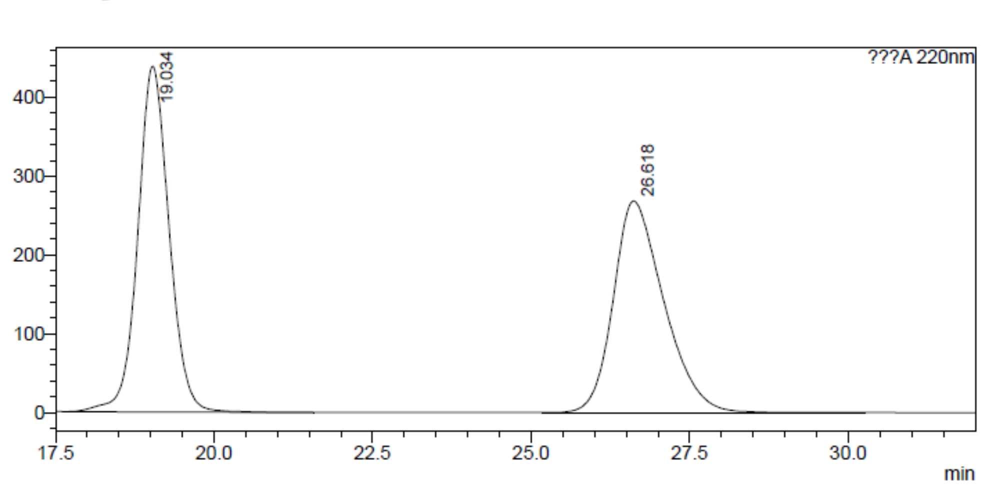

<Peak Table>

???A 220nm

Peak#	Ret. Time	Area	Height	Conc.	Unit	Mark	Name
1	19.034	15092766	438711	50.274			
2	26.618	14927983	268572	49.726			
Total		30020749	707283				

<Chromatogram>

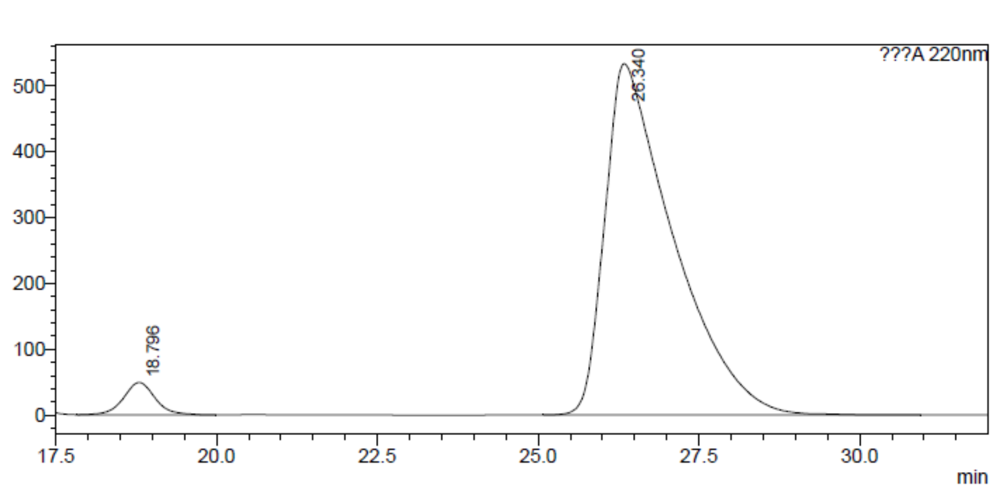

<Peak Table>

???A 220nm

Peak#	Ret. Time	Area	Height	Conc.	Unit	Mark	Name
1	18.796	1673204	49024	4.053			
2	26.340	39608955	533536	95.947			
Total		41282159	582561				

(1S,2S)-1-Phenyl-1-(thiophen-2-yl)propane-1,2-diol (**1d**)

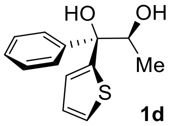

1d

<Chromatogram>

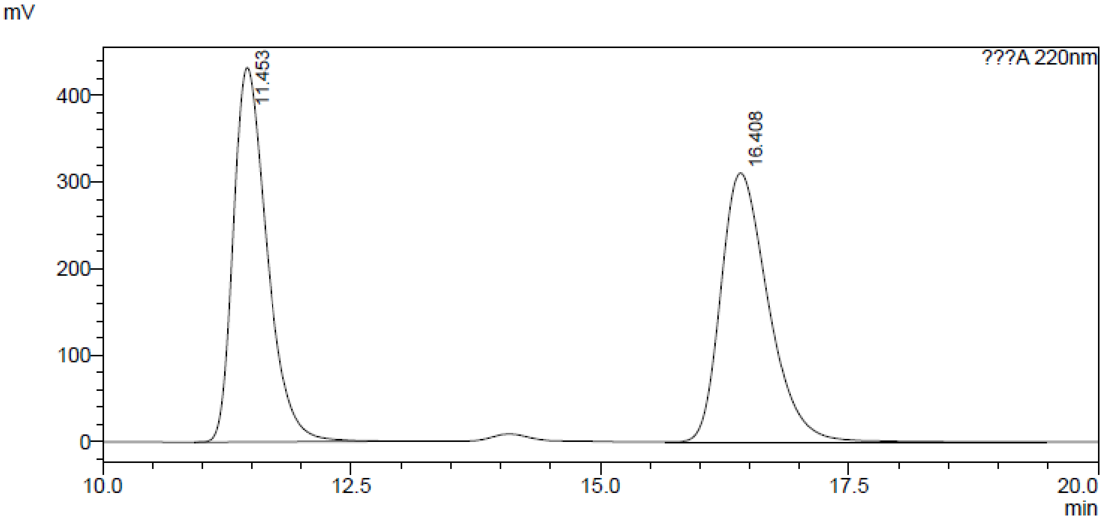

<Peak Table>

???A 220nm

Peak#	Ret. Time	Area	Height	Conc.	Unit	Mark	Name
1	11.453	10224484	432836	49.730			
2	16.408	10335674	310817	50.270			
Total		20560158	743653				

<Chromatogram>

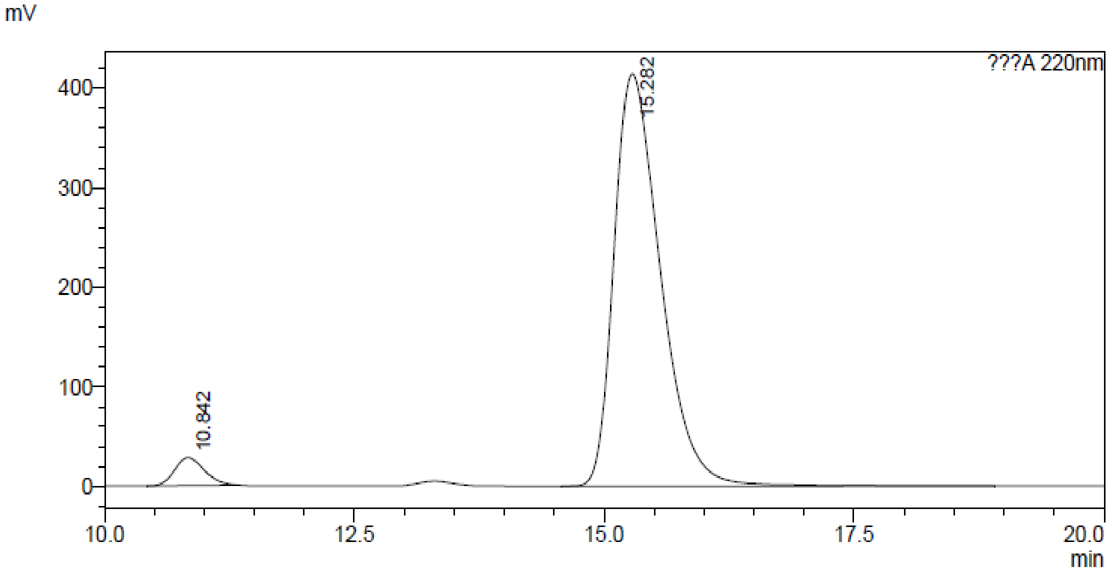

<Peak Table>

???A 220nm

Peak#	Ret. Time	Area	Height	Conc.	Unit	Mark	Name
1	10.842	584928	28226	4.196		M	
2	15.282	13355552	414095	95.804			
Total		13940480	442321				

(1*R*,2*R*)-1-Hydroxy-1-phenyl-1-(thiophen-2-yl)propan-2-yl propionate (**2d**)

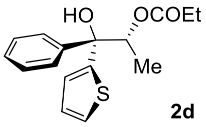

2d

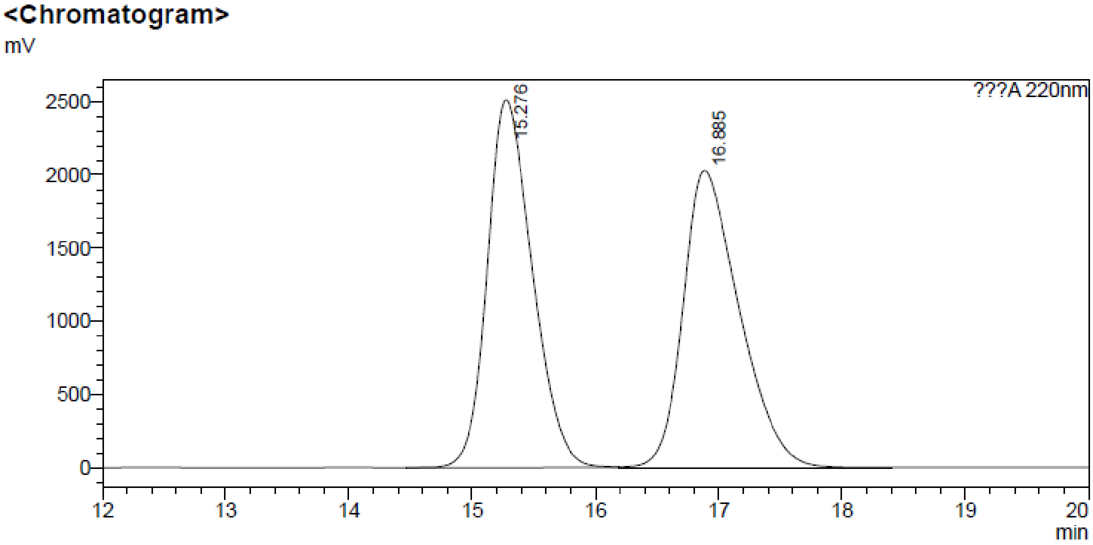

<Peak Table>

???A 220nm

Peak#	Ret. Time	Area	Height	Conc.	Unit	Mark	Name
1	15.276	63887402	2511507	49.839			
2	16.885	64299498	2028209	50.161		V	
Total		128186900	4539716				

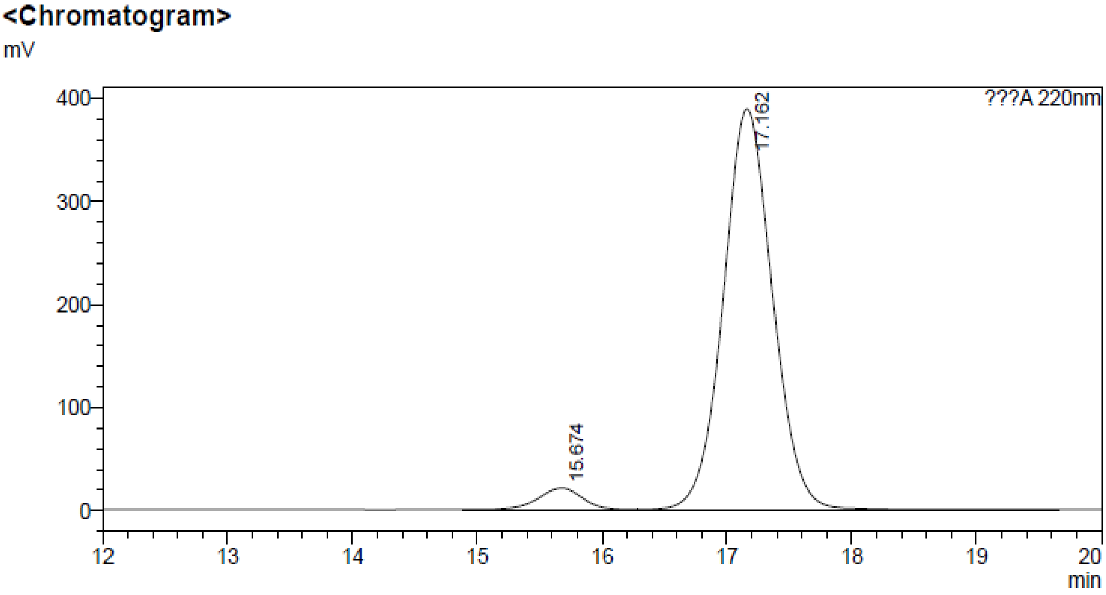

<Peak Table>

???A 220nm

Peak#	Ret. Time	Area	Height	Conc.	Unit	Mark	Name
1	15.674	506402	20838	4.540			
2	17.162	10647544	388971	95.460		V	
Total		11153946	409810				

(1*S*,2*S*)-1-(4-Methoxyphenyl)-1-(thiophen-2-yl)propane-1,2-diol (**1e**)

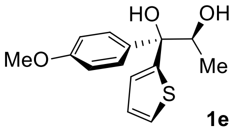

1e

<Chromatogram>

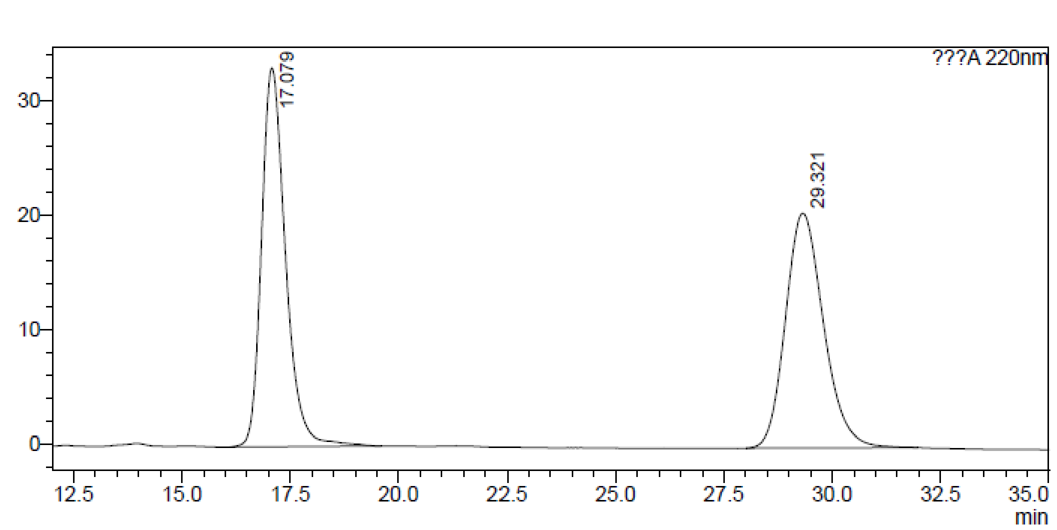

<Peak Table>

???A 220nm

Peak#	Ret. Time	Area	Height	Conc.	Unit	Mark	Name
1	17.079	1278565	33122	50.452			
2	29.321	1255647	20528	49.548			
Total		2534212	53650				

<Chromatogram>

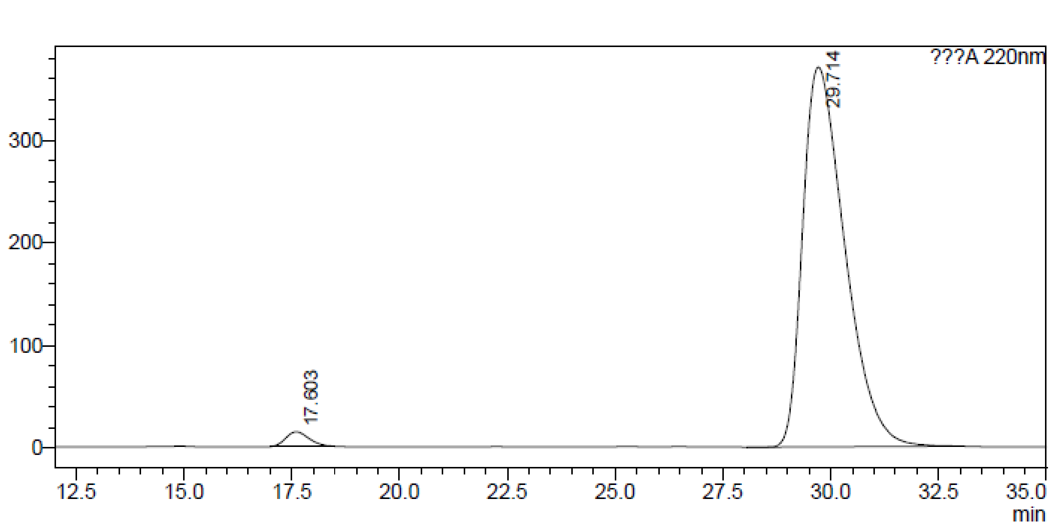

<Peak Table>

???A 220nm

Peak#	Ret. Time	Area	Height	Conc.	Unit	Mark	Name
1	17.603	537671	14496	2.099		M	
2	29.714	25081872	370516	97.901		M	
Total		25619543	385012				

(1*R*,2*R*)-1-Hydroxy-1-(4-methoxyphenyl)-1-(thiophen-2-yl)propan-2-yl propionate (2e)

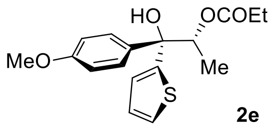

2e

<Chromatogram>

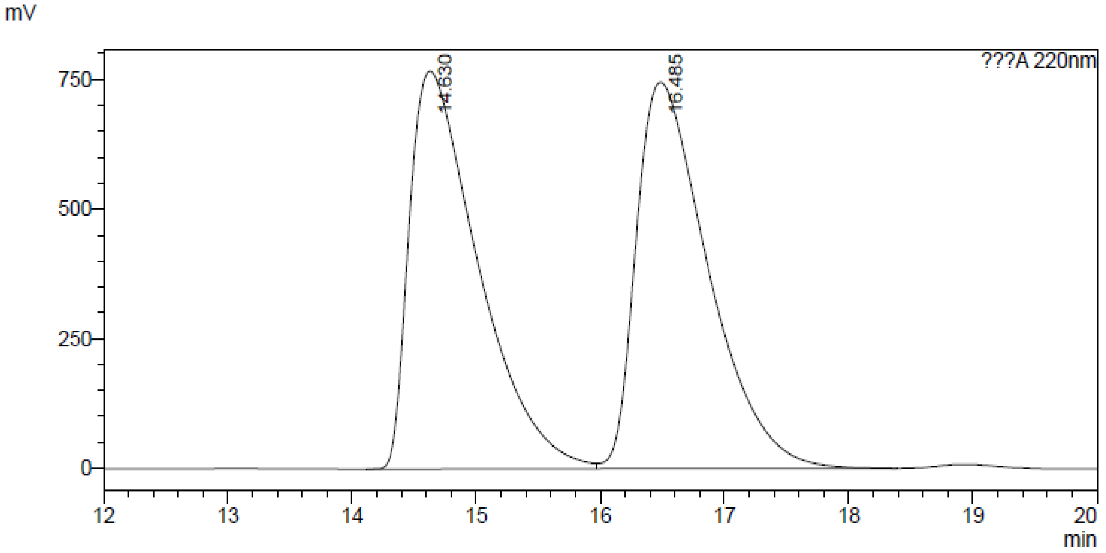

<Peak Table>

???A 220nm

Peak#	Ret. Time	Area	Height	Conc.	Unit	Mark	Name
1	14.630	29645085	768223	49.592			
2	16.485	30132358	746407	50.408		V	
Total		59777442	1514630				

<Chromatogram>

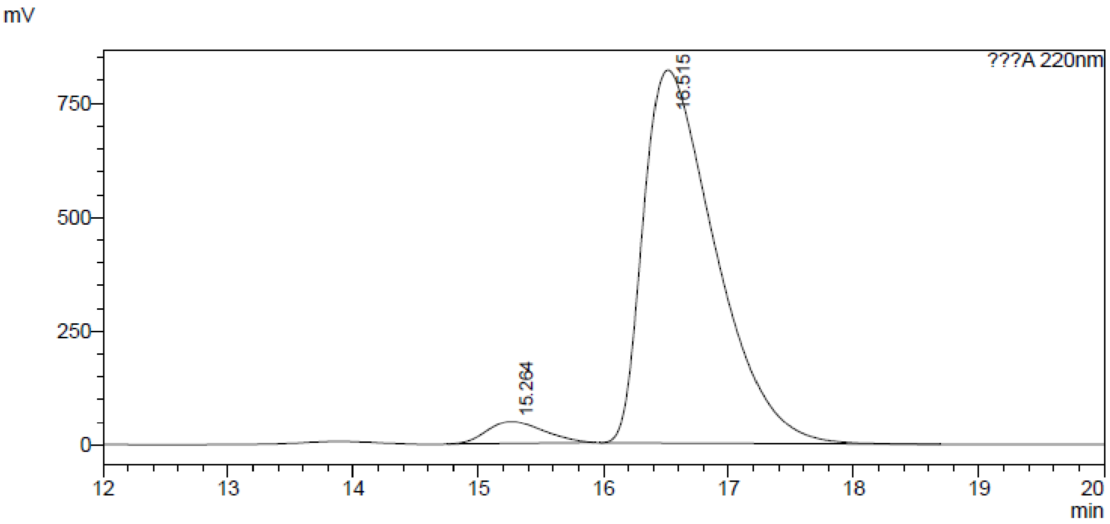

<Peak Table>

???A 220nm

Peak#	Ret. Time	Area	Height	Conc.	Unit	Mark	Name
1	15.264	1530473	48271	4.464		M	
2	16.515	32751517	818539	95.536			
Total		34281989	866810				

(1*S*,2*S*)-1-(Furan-2-yl)-1-(thiophen-2-yl)propane-1,2-diol (**1f**)

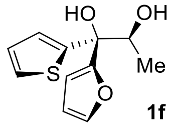

1f

<Chromatogram>

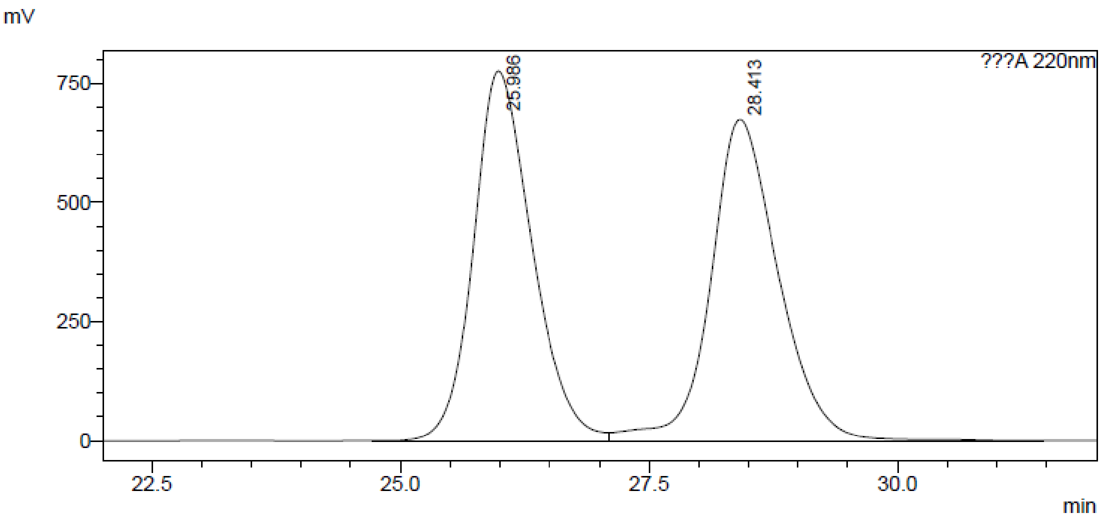

<Peak Table>

???A 220nm

Peak#	Ret. Time	Area	Height	Conc.	Unit	Mark	Name
1	25.986	31836240	776183	49.795			
2	28.413	32098639	673991	50.205		V	
Total		63934879	1450174				

<Chromatogram>

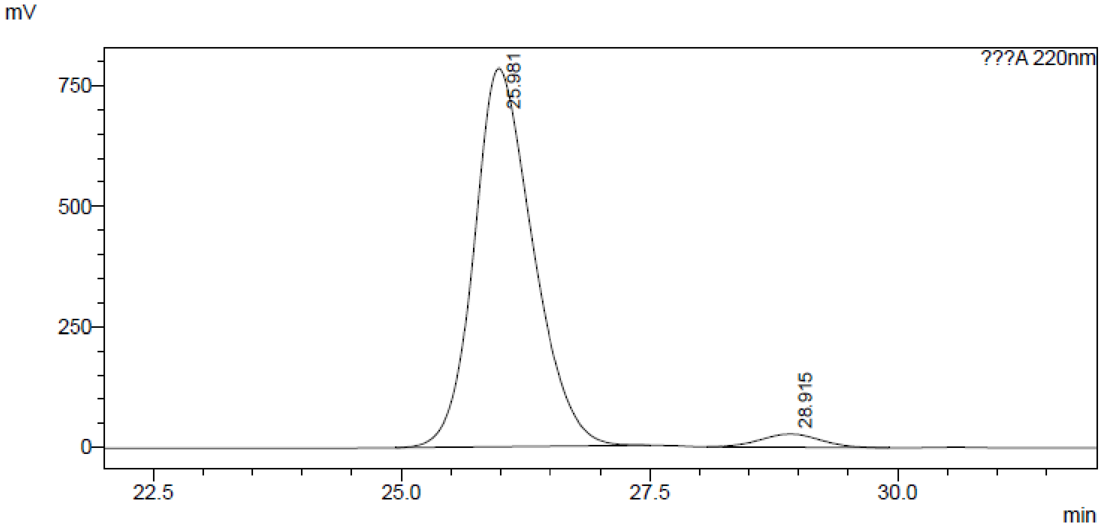

<Peak Table>

???A 220nm

Peak#	Ret. Time	Area	Height	Conc.	Unit	Mark	Name
1	25.981	32288384	783624	96.560		M	
2	28.915	1150340	27607	3.440			
Total		33438723	811231				

(1*R*,2*R*)-1-(Furan-2-yl)-1-hydroxy-1-(thiophen-2-yl)propan-2-yl propionate (**2f**)

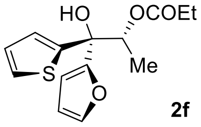

2f

<Chromatogram>

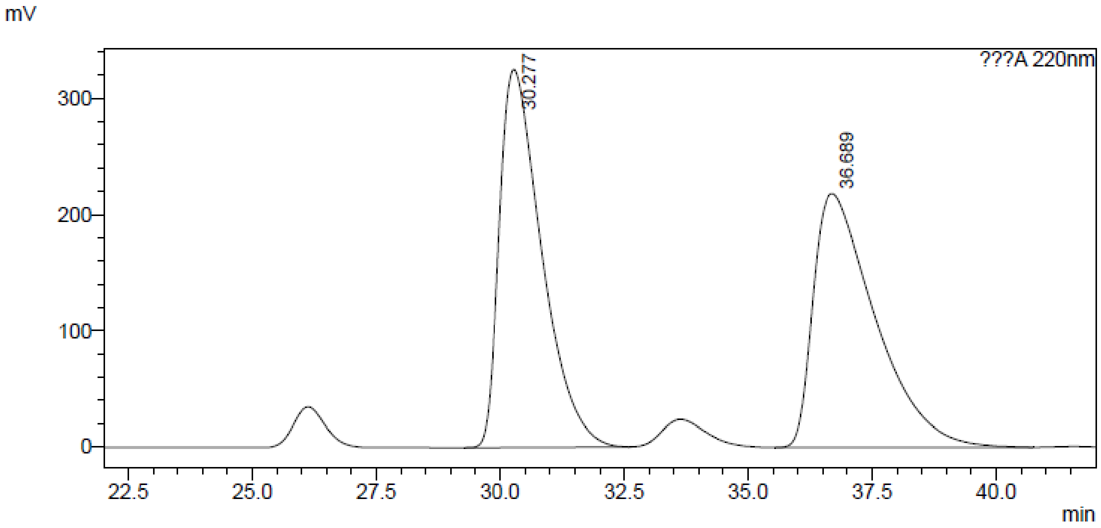

<Peak Table>

???A 220nm

Peak#	Ret. Time	Area	Height	Conc.	Unit	Mark	Name
1	30.277	19277800	325377	50.397			
2	36.689	18974085	218598	49.603			
Total		38251885	543975				

<Chromatogram>

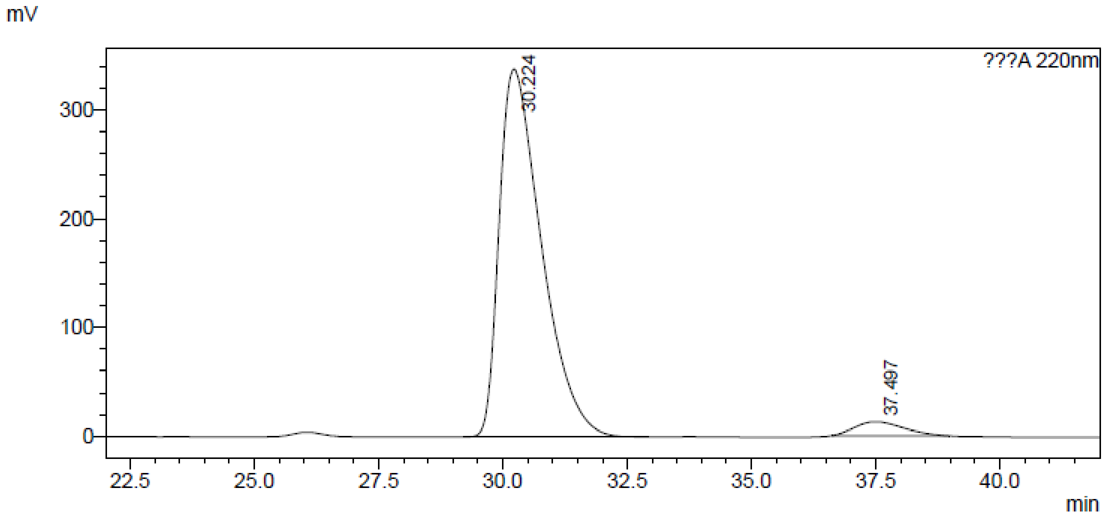

<Peak Table>

???A 220nm

Peak#	Ret. Time	Area	Height	Conc.	Unit	Mark	Name
1	30.224	20027231	338512	95.826			
2	37.497	872283	12857	4.174		M	
Total		20899514	351369				

(1*S*,2*S*)-1-Phenyl-1-(pyridin-2-yl)propane-1,2-diol (**1g**)

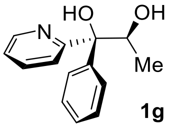

1g

<Chromatogram>

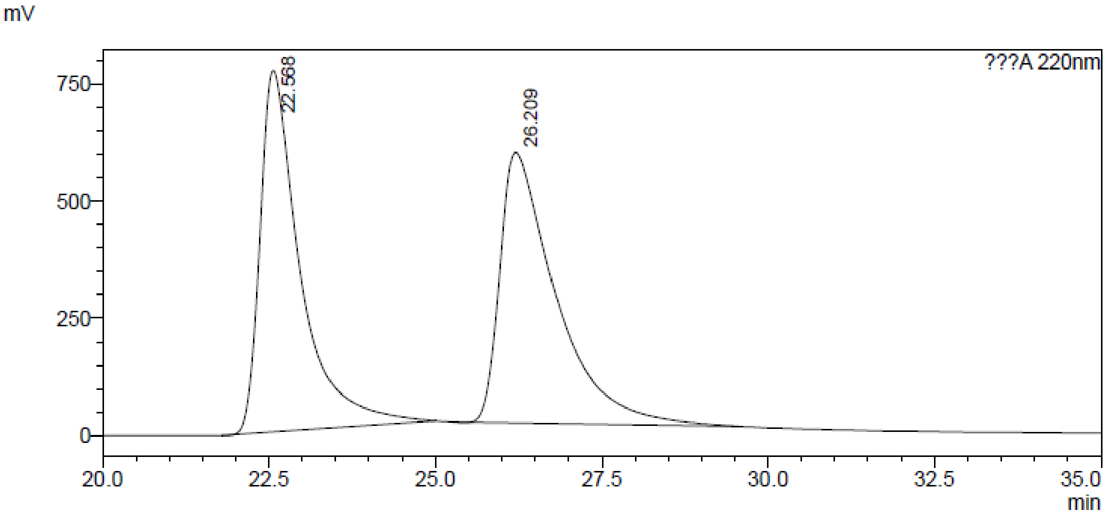

<Peak Table>

???A 220nm

Peak#	Ret. Time	Area	Height	Conc.	Unit	Mark	Name
1	22.568	32544162	772913	49.057		M	
2	26.209	33795318	578299	50.943		M	
Total		66339480	1351212				

<Chromatogram>

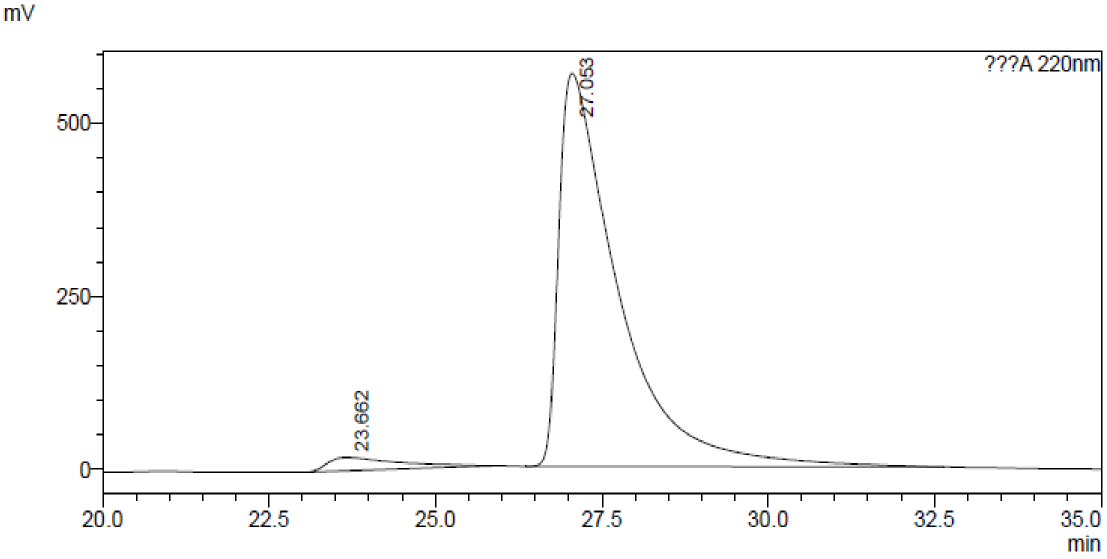

<Peak Table>

???A 220nm

Peak#	Ret. Time	Area	Height	Conc.	Unit	Mark	Name
1	23.662	1412778	19390	3.844		M	
2	27.053	35342170	567377	96.156		M	
Total		36754948	586767				

(1*R*,2*R*)-1-Hydroxy-1-phenyl-1-(pyridin-2-yl)propan-2-yl propionate (**2g**)

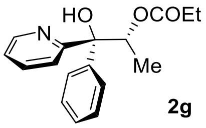

2g

<Chromatogram>

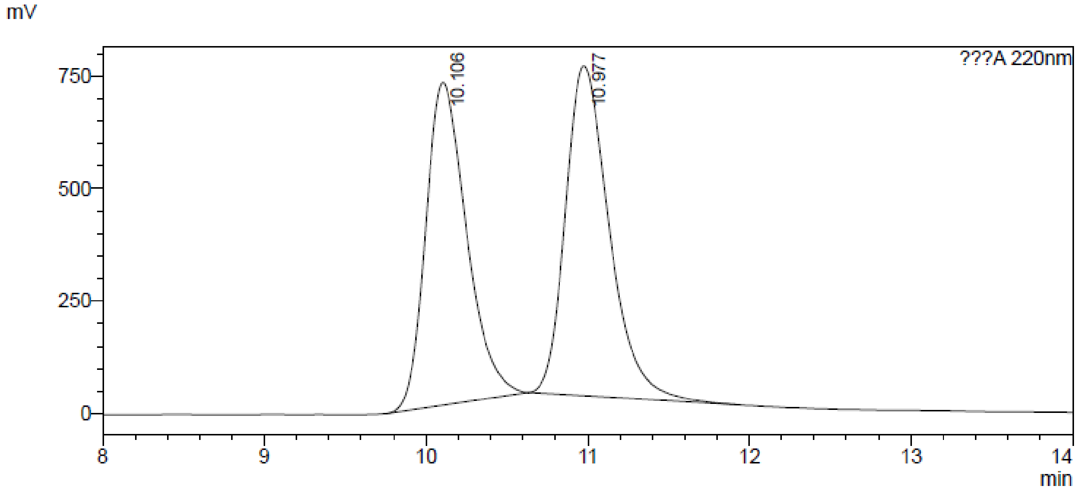

<Peak Table>

???A 220nm

Peak#	Ret. Time	Area	Height	Conc.	Unit	Mark	Name
1	10.106	12595694	715920	47.722			
2	10.977	13798465	731254	52.278		M	
Total		26394159	1447174				

<Chromatogram>

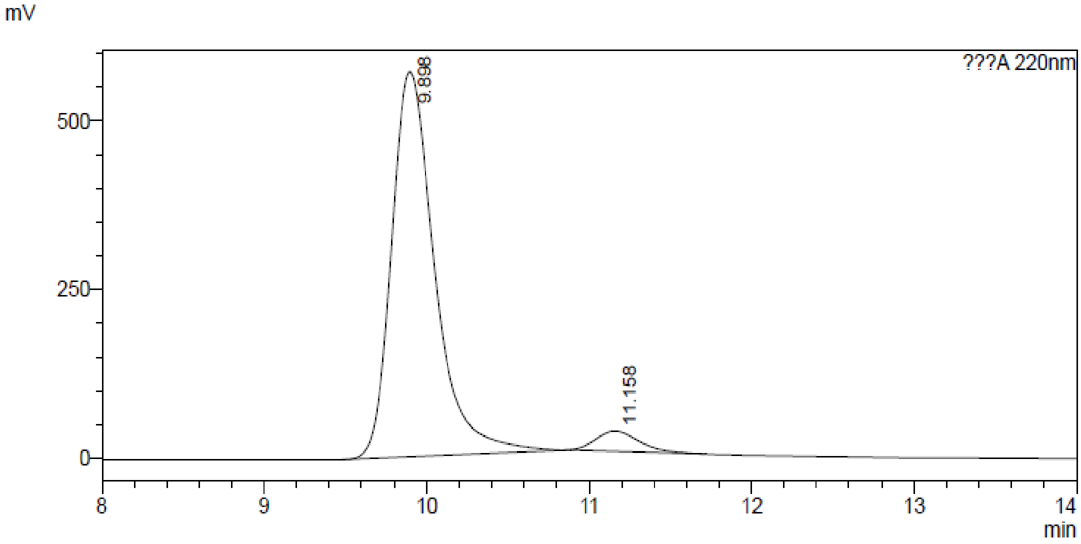

<Peak Table>

???A 220nm

Peak#	Ret. Time	Area	Height	Conc.	Unit	Mark	Name
1	9.898	10522147	569739	95.042		M	
2	11.158	548919	29743	4.958		M	
Total		11071066	599482				

(1*S*,2*S*)-1-(4-Bromophenyl)-1-(4-chlorophenyl)butane-1,2-diol (**1h**)

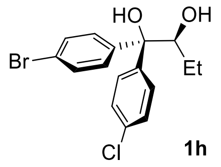

1h

<Chromatogram>

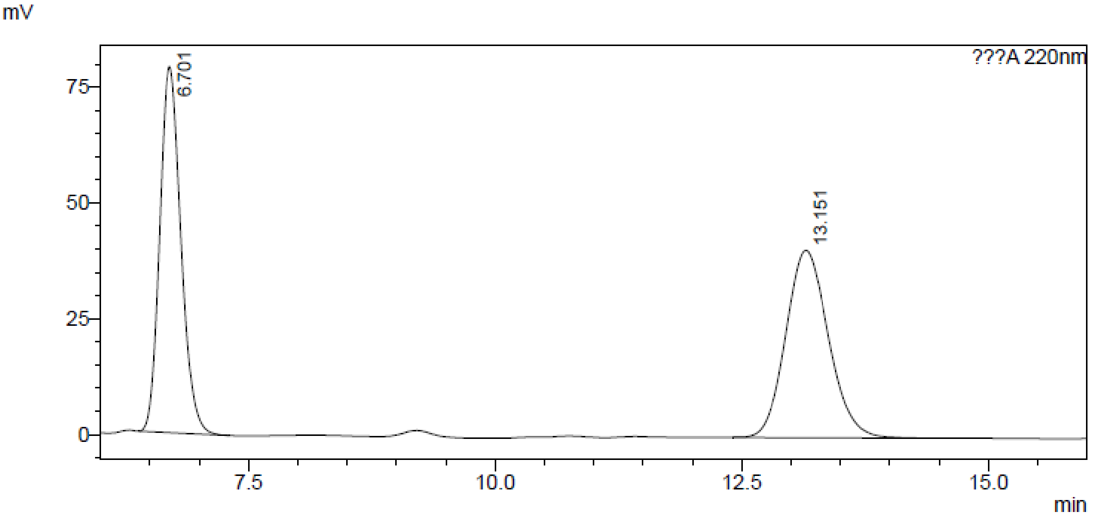

<Peak Table>

???A 220nm

Peak#	Ret. Time	Area	Height	Conc.	Unit	Mark	Name
1	6.701	1190305	79068	49.485			
2	13.151	1215059	40399	50.515			
Total		2405365	119467				

<Chromatogram>

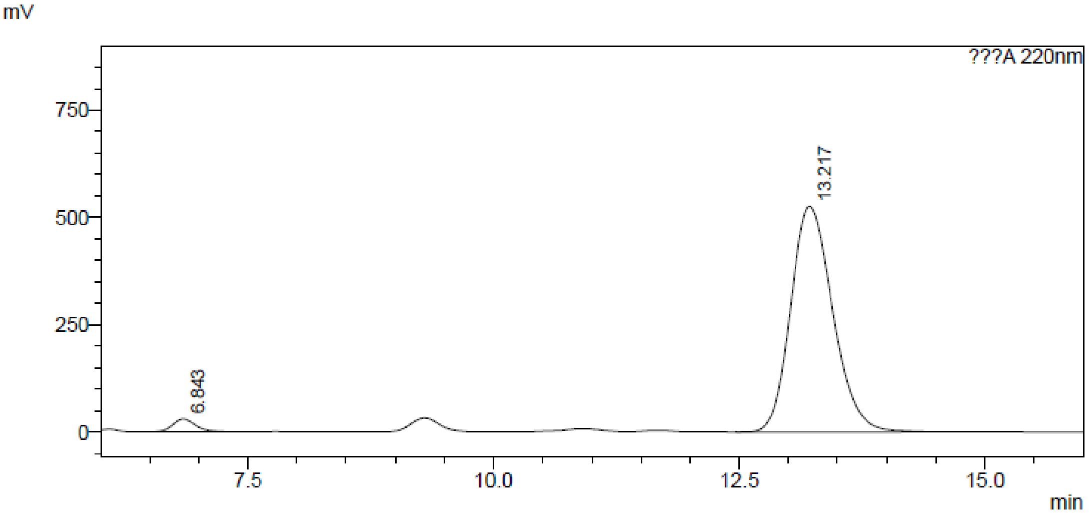

<Peak Table>

???A 220nm

Peak#	Ret. Time	Area	Height	Conc.	Unit	Mark	Name
1	6.843	464026	29256	2.872			
2	13.217	15690426	525695	97.128			
Total		16154452	554951				

(1*R*,2*R*)-1-(4-Bromophenyl)-1-(4-chlorophenyl)-1-hydroxybutan-2-yl propionate (**2h**)

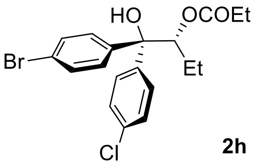

2h

<Chromatogram>

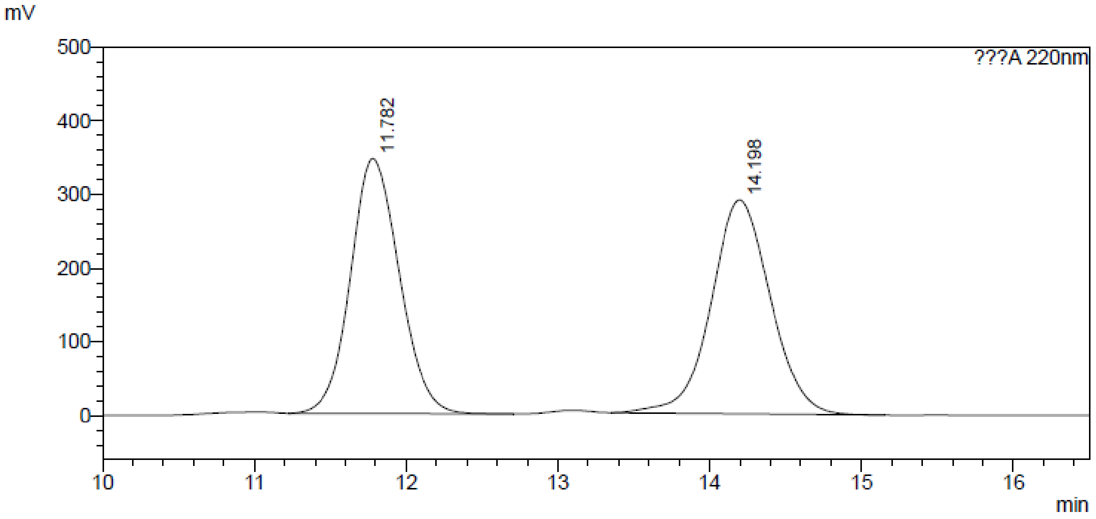

<Peak Table>

???A 220nm

Peak#	Ret. Time	Area	Height	Conc.	Unit	Mark	Name
1	11.782	7759252	345831	49.650			
2	14.198	7868572	289755	50.350			
Total		15627824	635586				

<Chromatogram>

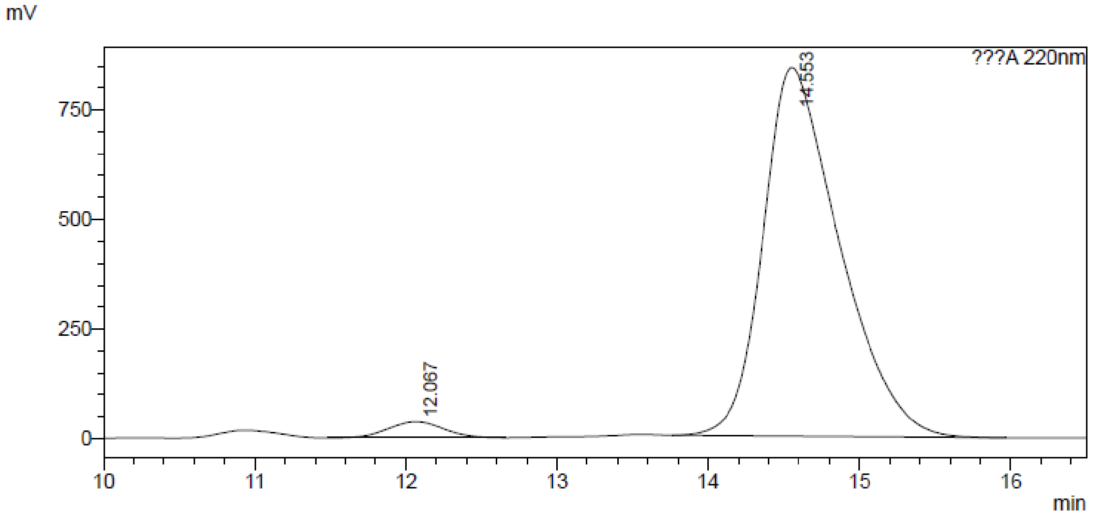

<Peak Table>

???A 220nm

Peak#	Ret. Time	Area	Height	Conc.	Unit	Mark	Name
1	12.067	918890	36109	3.006			
2	14.553	29648360	841121	96.994			
Total		30567250	877230				

(1*R*,2*S*)-1,3-Diphenyl-1-(*p*-tolyl)propane-1,2-diol (**1i**)

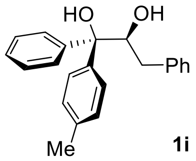

1i

<Chromatogram>

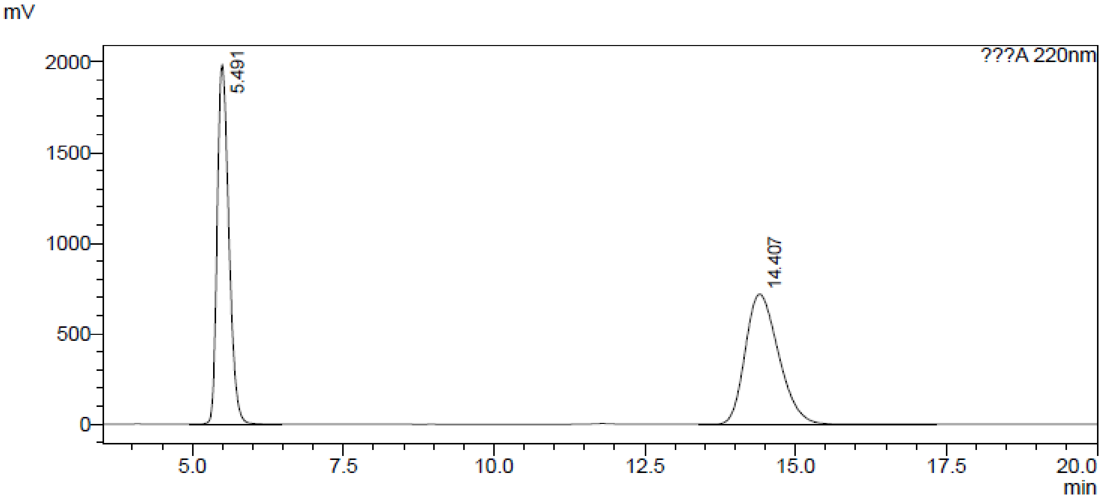

<Peak Table>

???A 220nm

Peak#	Ret. Time	Area	Height	Conc.	Unit	Mark	Name
1	5.491	26666920	1982633	49.083			
2	14.407	27663667	718211	50.917			
Total		54330587	2700844				

<Chromatogram>

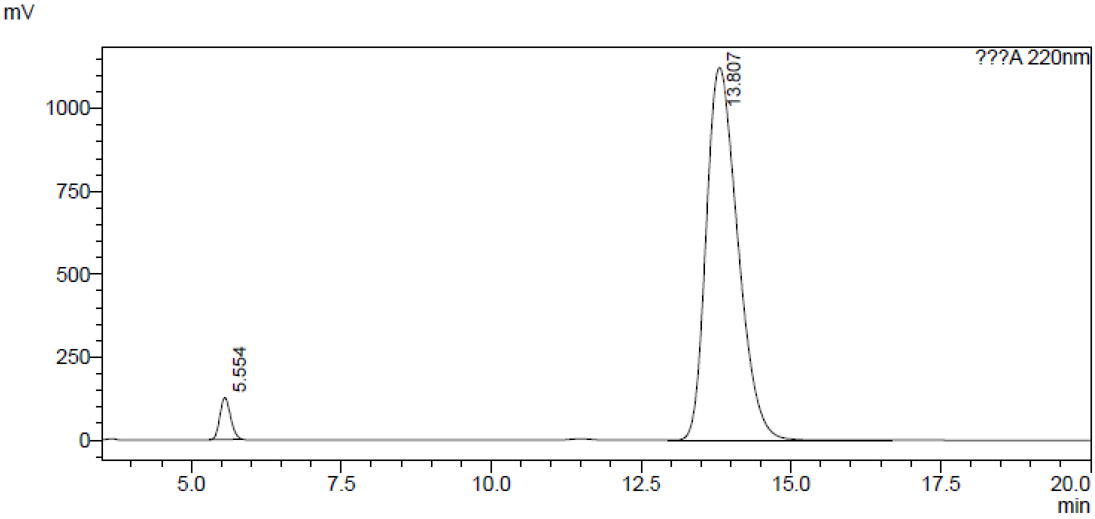

<Peak Table>

???A 220nm

Peak#	Ret. Time	Area	Height	Conc.	Unit	Mark	Name
1	5.554	1558931	127426	3.627		M	
2	13.807	41423931	1124461	96.373			
Total		42982862	1251887				

(1*S*,2*R*)-1-Hydroxy-1,3-diphenyl-1-(*p*-tolyl)propan-2-yl propionate (**2i**)

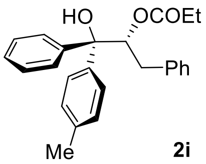

2i

<Chromatogram>

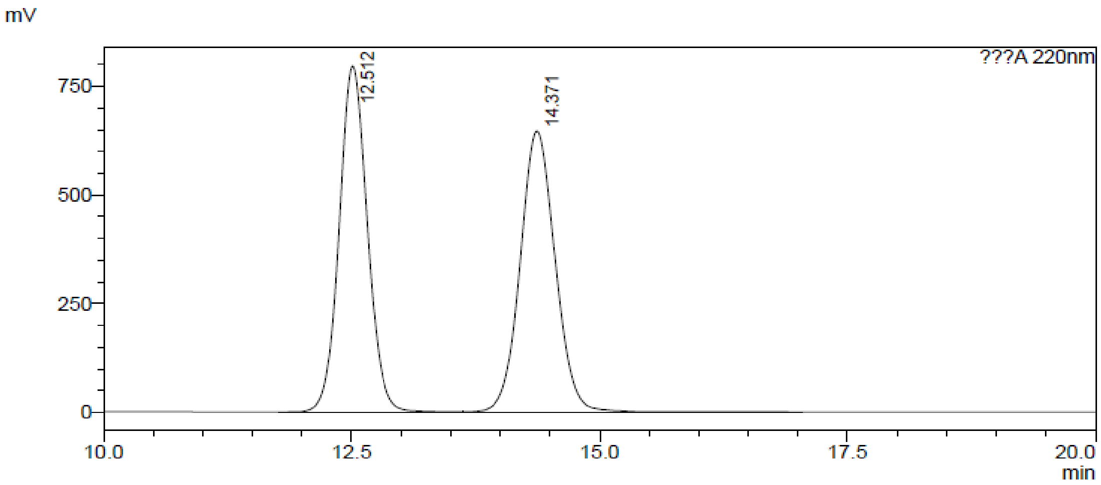

<Peak Table>

???A 220nm

Peak#	Ret. Time	Area	Height	Conc.	Unit	Mark	Name
1	12.512	15755751	795588	49.902			
2	14.371	15817873	645974	50.098		V	
Total		31573624	1441562				

<Chromatogram>

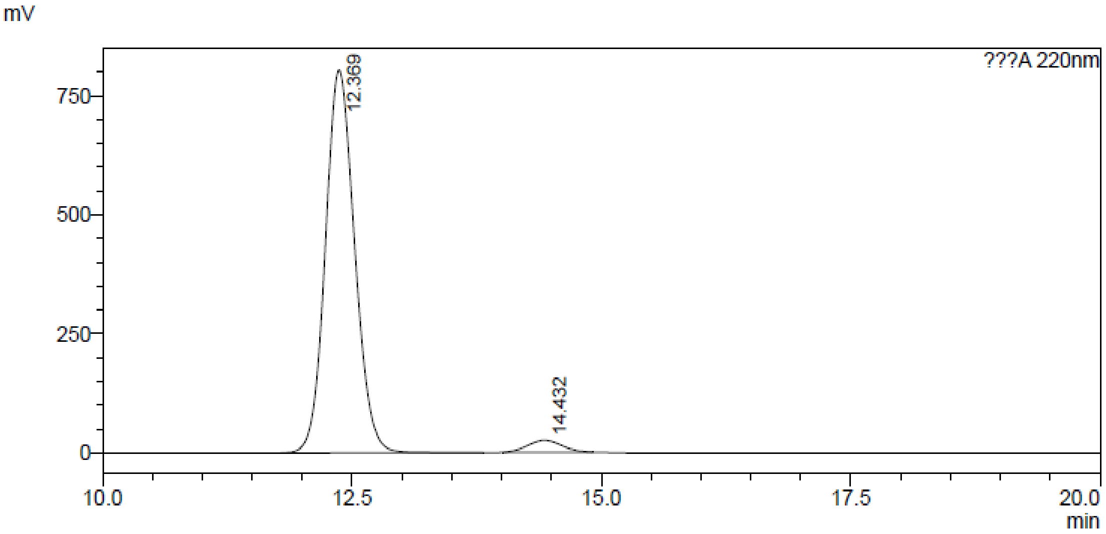

<Peak Table>

???A 220nm

Peak#	Ret. Time	Area	Height	Conc.	Unit	Mark	Name
1	12.369	16433343	804214	96.532			
2	14.432	590442	24941	3.468		M	
Total		17023785	829155				

(1*S*,2*S*)-1-(4-Methoxyphenyl)-3-phenyl-1-(*p*-tolyl)propane-1,2-diol (**1j**)

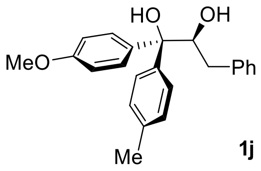

<Chromatogram>

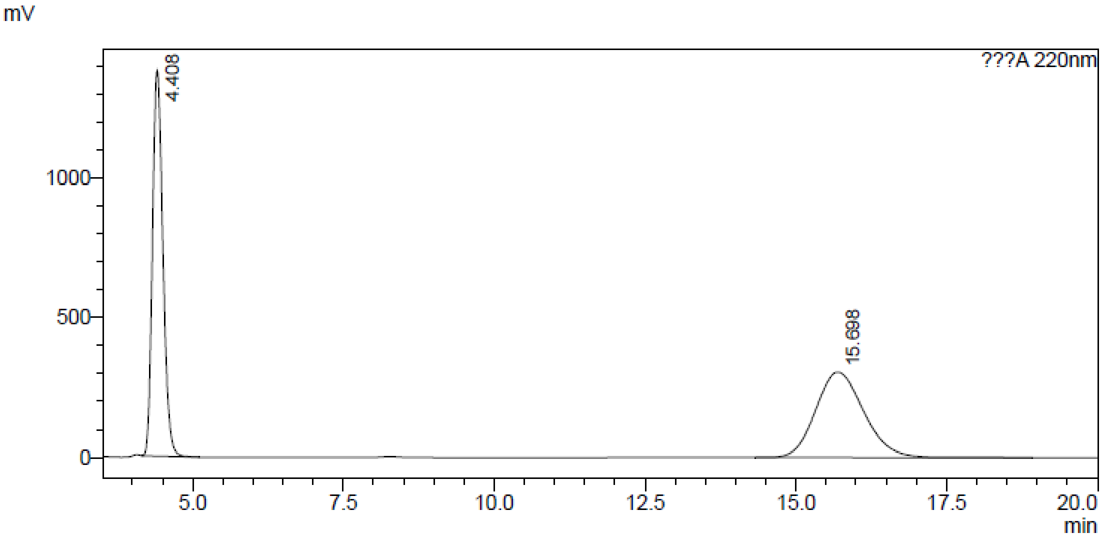

<Peak Table>

???A 220nm

Peak#	Ret. Time	Area	Height	Conc.	Unit	Mark	Name
1	4.408	16582747	1378152	49.299			
2	15.698	17054229	305958	50.701			
Total		33636976	1684110				

<Chromatogram>

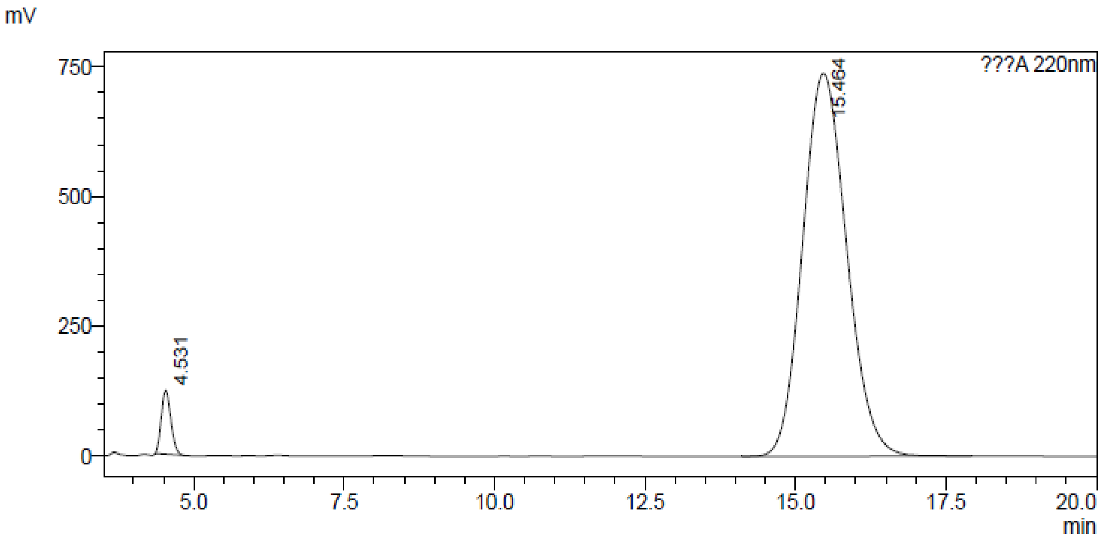

<Peak Table>

???A 220nm

Peak#	Ret. Time	Area	Height	Conc.	Unit	Mark	Name
1	4.531	1357165	122346	3.499		M	
2	15.464	37429180	737302	96.501		M	
Total		38786345	859648				

(1*R*,2*R*)-1-Hydroxy-1-(4-methoxyphenyl)-3-phenyl-1-(*p*-tolyl)propan-2-yl propionate (2j)

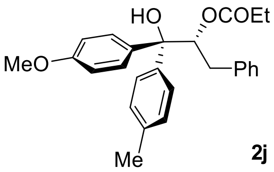

2j

<Chromatogram>

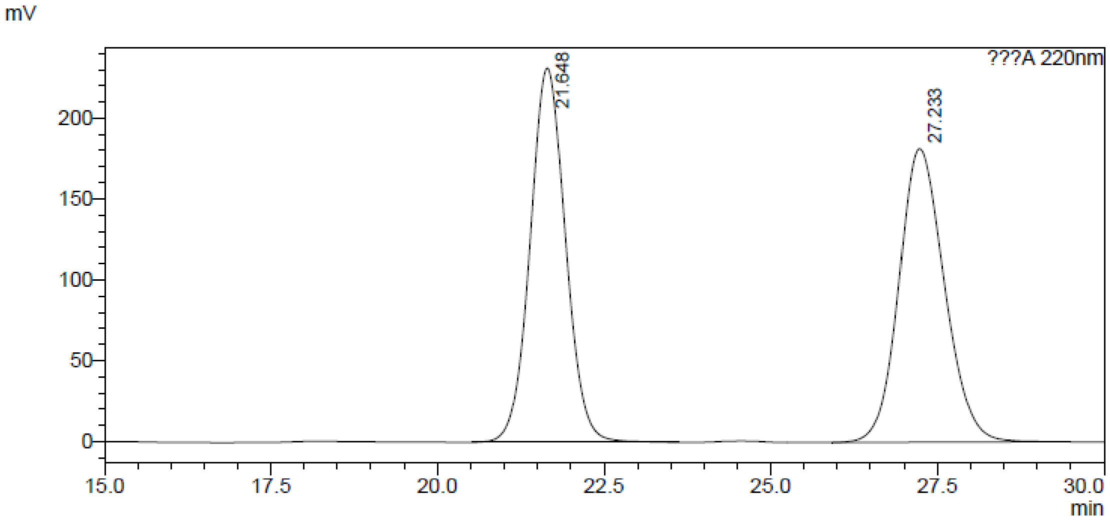

<Peak Table>

???A 220nm

Peak#	Ret. Time	Area	Height	Conc.	Unit	Mark	Name
1	21.648	8588685	231248	49.959			
2	27.233	8602949	181459	50.041			
Total		17191634	412708				

<Chromatogram>

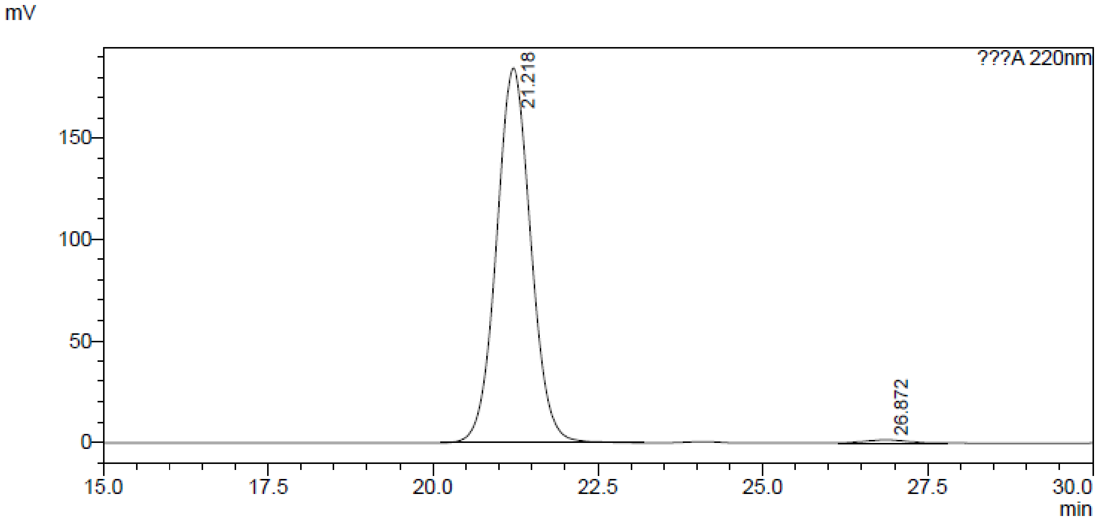

<Peak Table>

???A 220nm

Peak#	Ret. Time	Area	Height	Conc.	Unit	Mark	Name
1	21.218	6714357	184601	99.064			
2	26.872	63411	1484	0.936			
Total		6777768	186085				

(1*S*,2*S*)-1-(4-Methoxyphenyl)-1-phenylpentane-1,2-diol (**1k**)

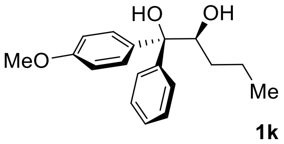

1k

<Chromatogram>

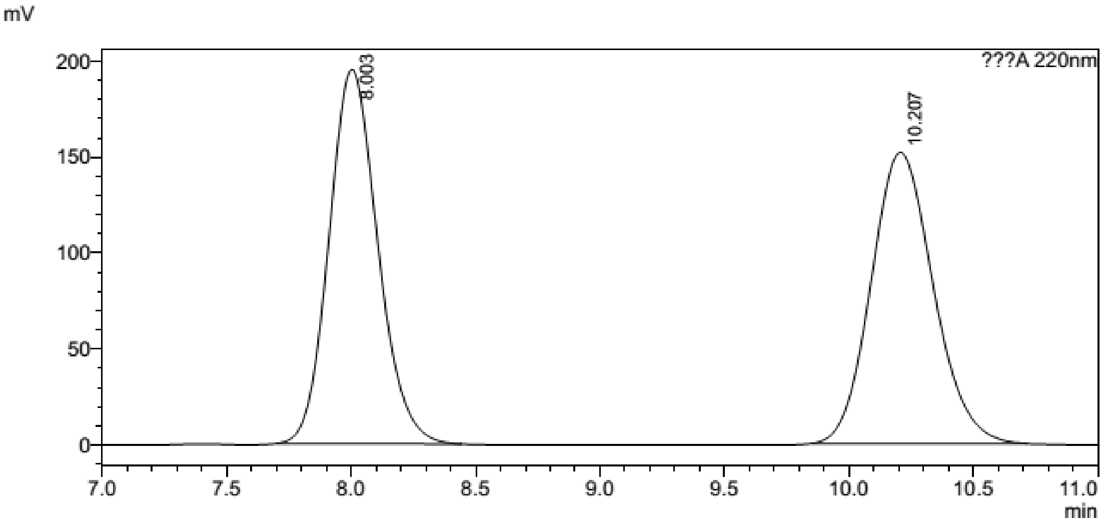

<Peak Table>

???A 220nm

Peak#	Ret. Time	Area	Height	Conc.	Unit	Mark	Name
1	8.003	2663262	195112	50.186			
2	10.207	2643469	151904	49.814			
Total		5306731	347017				

<Chromatogram>

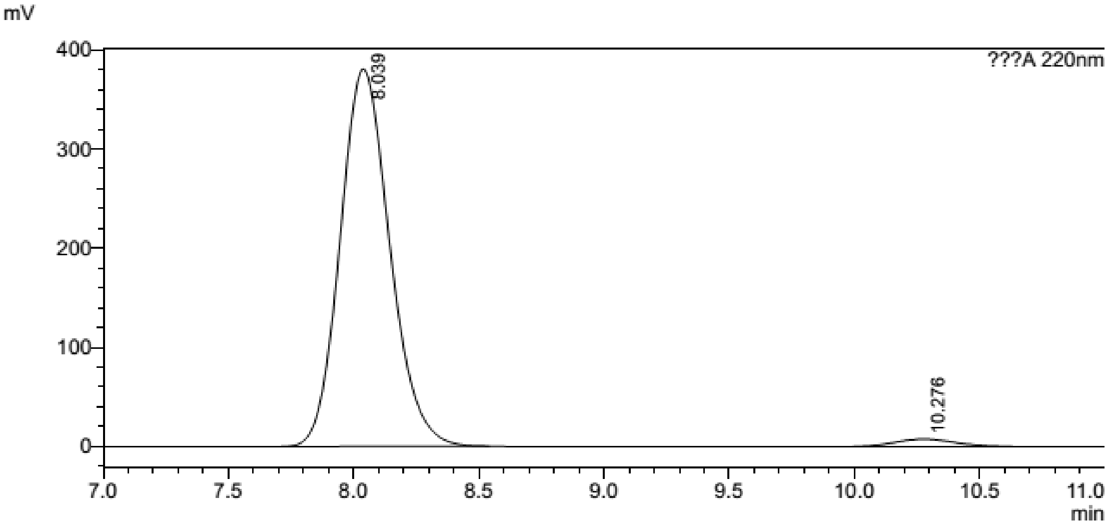

<Peak Table>

???A 220nm

Peak#	Ret. Time	Area	Height	Conc.	Unit	Mark	Name
1	8.039	5250559	380427	97.657		M	
2	10.276	125972	7518	2.343		M	
Total		5376531	387945				

(1*R*,2*R*)-1-Hydroxy-1-(4-methoxyphenyl)-1-phenylpentan-2-yl propionate (**2k**)

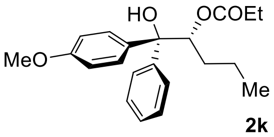

2k

<Chromatogram>

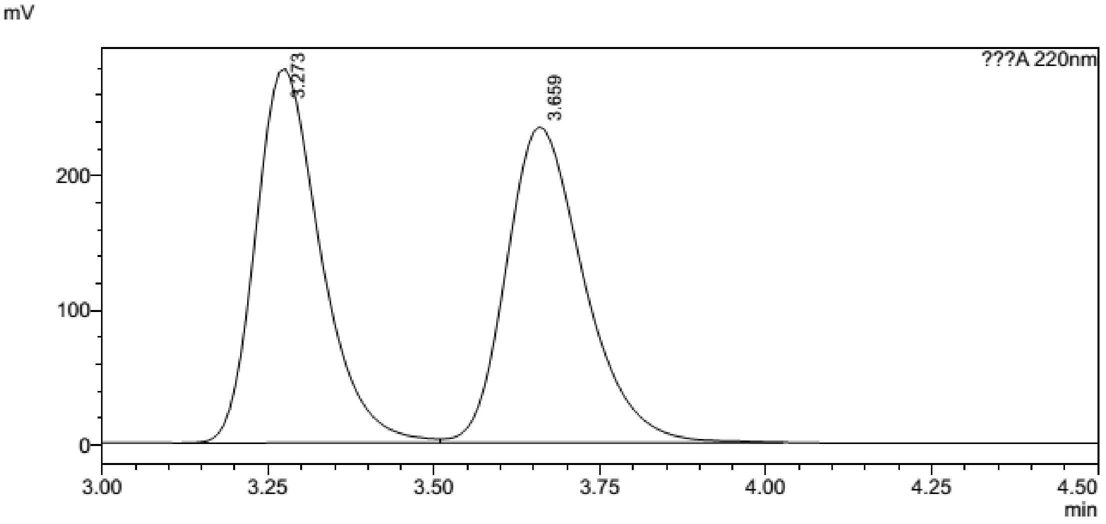

<Peak Table>

???A 220nm

Peak#	Ret. Time	Area	Height	Conc.	Unit	Mark	Name
1	3.273	1899808	278198	49.845			
2	3.659	1911638	234840	50.155		V	
Total		3811446	513038				

<Chromatogram>

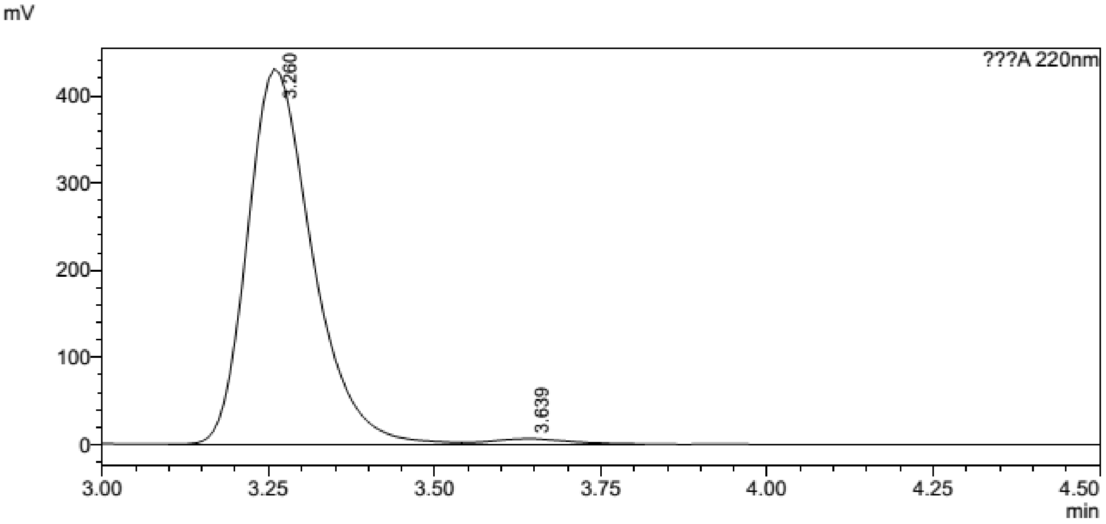

<Peak Table>

???A 220nm

Peak#	Ret. Time	Area	Height	Conc.	Unit	Mark	Name
1	3.260	2920099	430488	98.295			
2	3.639	50661	6346	1.705		V	
Total		2970760	436834				

(1*S*,2*S*)-1-Phenyl-1-(phenyl-*d*5)pentane-1,2-diol (**1l**)

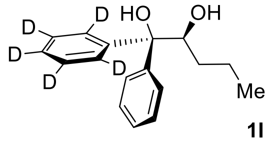

1l

<Chromatogram>

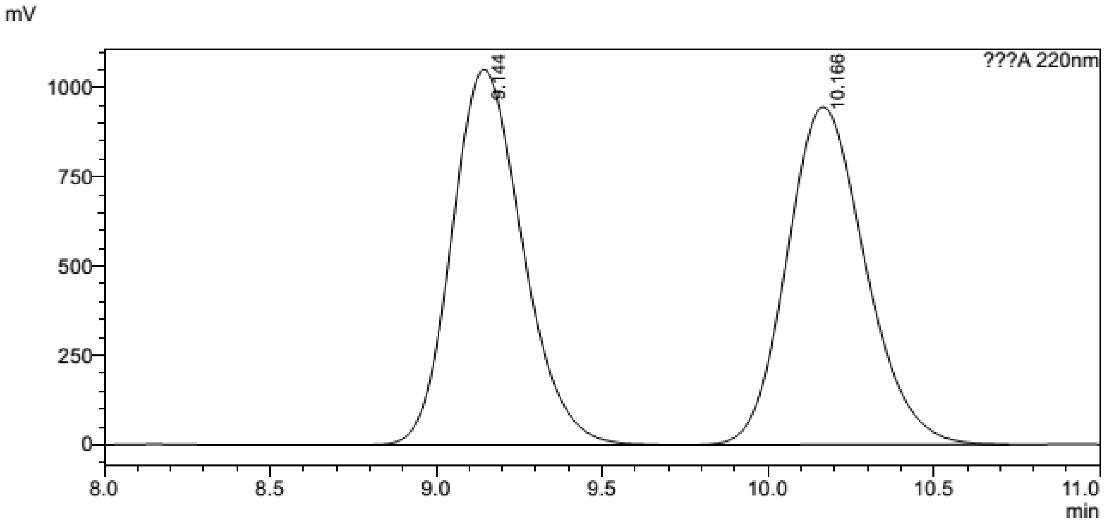

<Peak Table>

???A 220nm

Peak#	Ret. Time	Area	Height	Conc.	Unit	Mark	Name
1	9.144	15529582	1050663	49.981			
2	10.166	15541536	946008	50.019			
Total		31071117	1996671				

<Chromatogram>

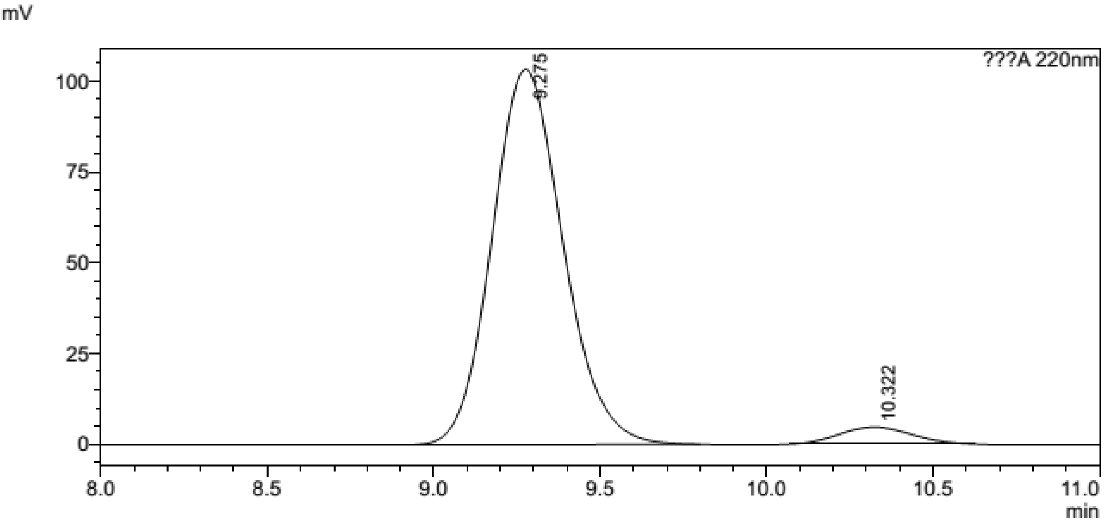

<Peak Table>

???A 220nm

Peak#	Ret. Time	Area	Height	Conc.	Unit	Mark	Name
1	9.275	1538637	103464	95.539		M	
2	10.322	71842	4688	4.461		M	
Total		1610479	108152				

(1*R*,2*R*)-1-Hydroxy-1-phenyl-1-(phenyl-*d*5)pentan-2-yl propionate (**2l**)

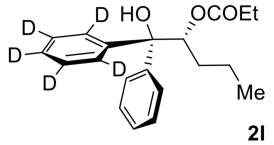

2l

<Chromatogram>

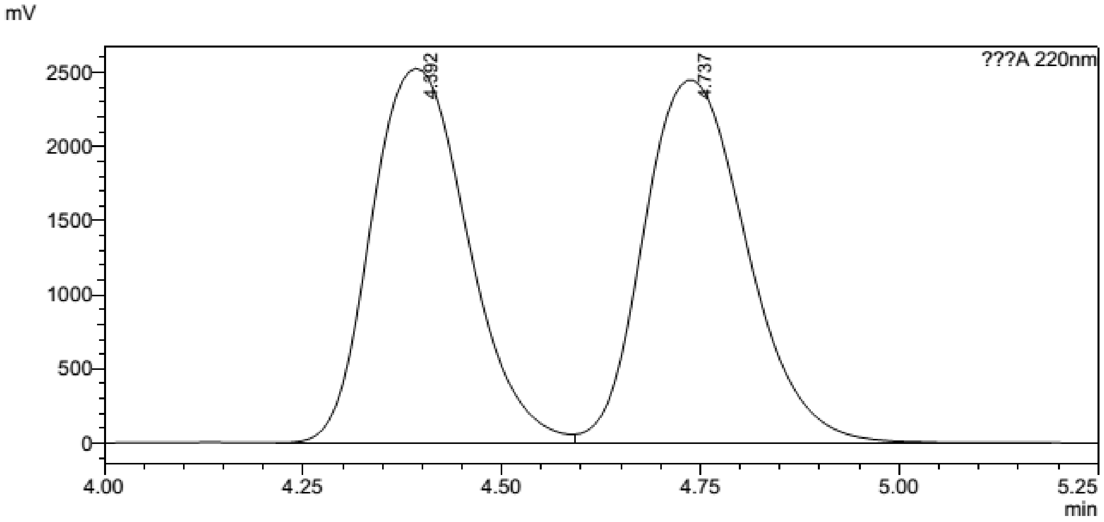

<Peak Table>

???A 220nm

Peak#	Ret. Time	Area	Height	Conc.	Unit	Mark	Name
1	4.392	21314271	2524606	49.133			
2	4.737	22066890	2443890	50.867		V	
Total		43381161	4968497				

<Chromatogram>

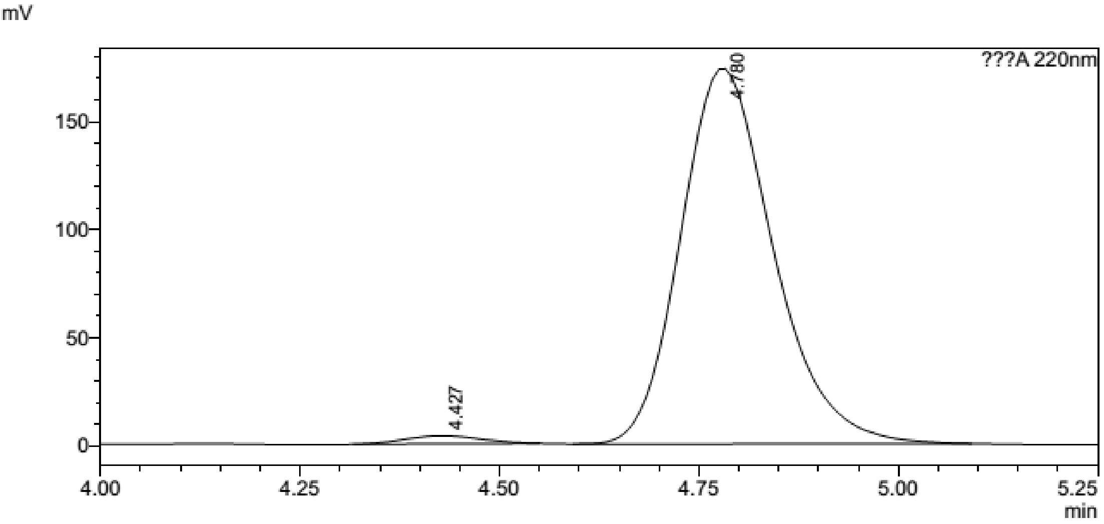

<Peak Table>

???A 220nm

Peak#	Ret. Time	Area	Height	Conc.	Unit	Mark	Name
1	4.427	25227	3735	1.763		M	
2	4.780	1405356	173487	98.237		M	
Total		1430583	177222				

(1*S*,2*R*)-2-Methyl-1-phenylbutane-1,2-diol (**1m**)

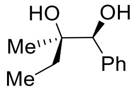

1m

<Chromatogram>

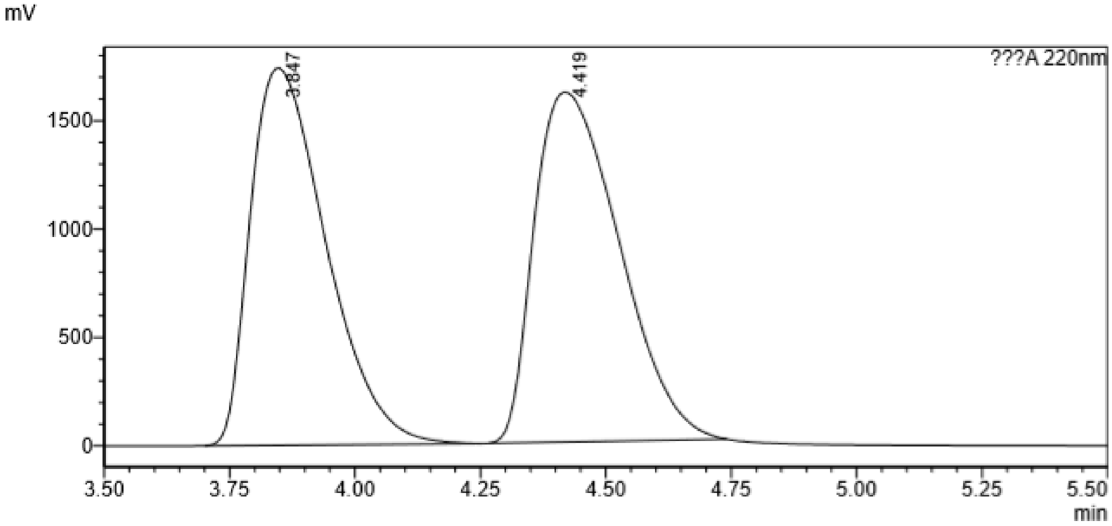

<Peak Table>

???A 220nm

Peak#	Ret. Time	Area	Height	Conc.	Unit	Mark	Name
1	3.847	18321574	1740427	49.010		M	
2	4.419	19061411	1614872	50.990		M	
Total		37382985	3355300				

<Chromatogram>

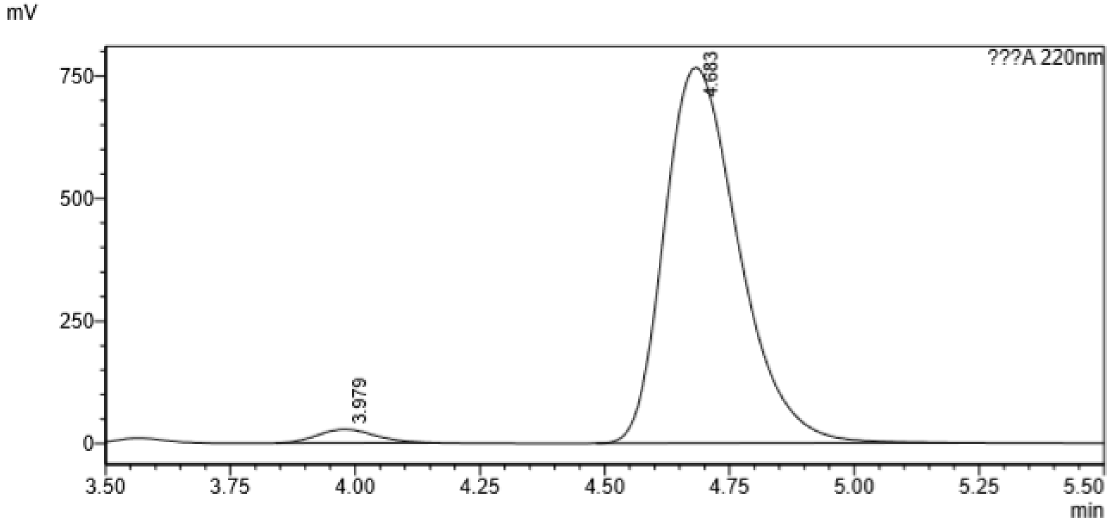

<Peak Table>

???A 220nm

Peak#	Ret. Time	Area	Height	Conc.	Unit	Mark	Name
1	3.979	227011	27931	2.773		M	
2	4.683	7960251	766763	97.227		M	
Total		8187261	794694				

(1*R*,2*S*)-2-Hydroxy-2-methyl-1-phenylbutyl propionate (**2m**)

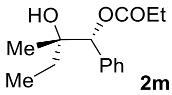

2m

<Chromatogram>

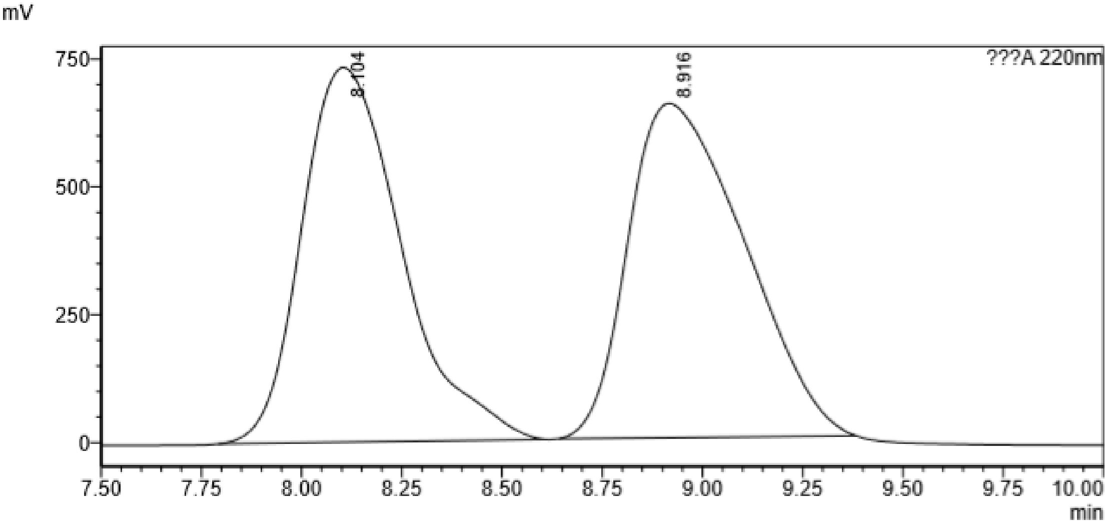

<Peak Table>

???A 220nm

Peak#	Ret. Time	Area	Height	Conc.	Unit	Mark	Name
1	8.104	12701796	732389	49.108		M	
2	8.916	13163171	653382	50.892		M	
Total		25864967	1385770				

<Chromatogram>

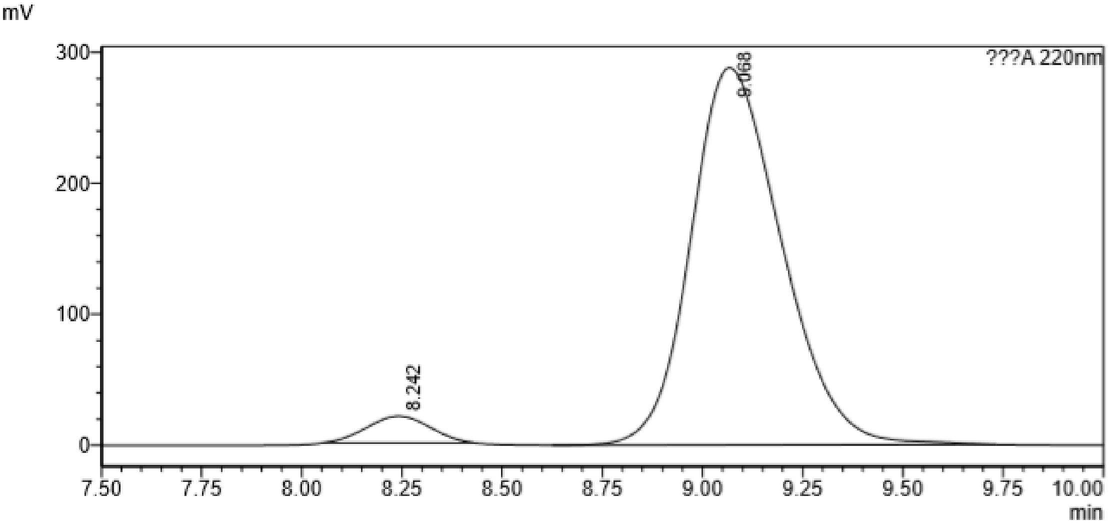

<Peak Table>

???A 220nm

Peak#	Ret. Time	Area	Height	Conc.	Unit	Mark	Name
1	8.242	226298	20426	4.746		M	
2	9.068	4541538	288015	95.254		M	
Total		4767835	308441				

(1*S*,2*R*)-2-Cyclohexyl-1-phenylpropane-1,2-diol (**1n**)

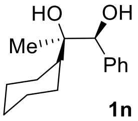

1n

<Chromatogram>

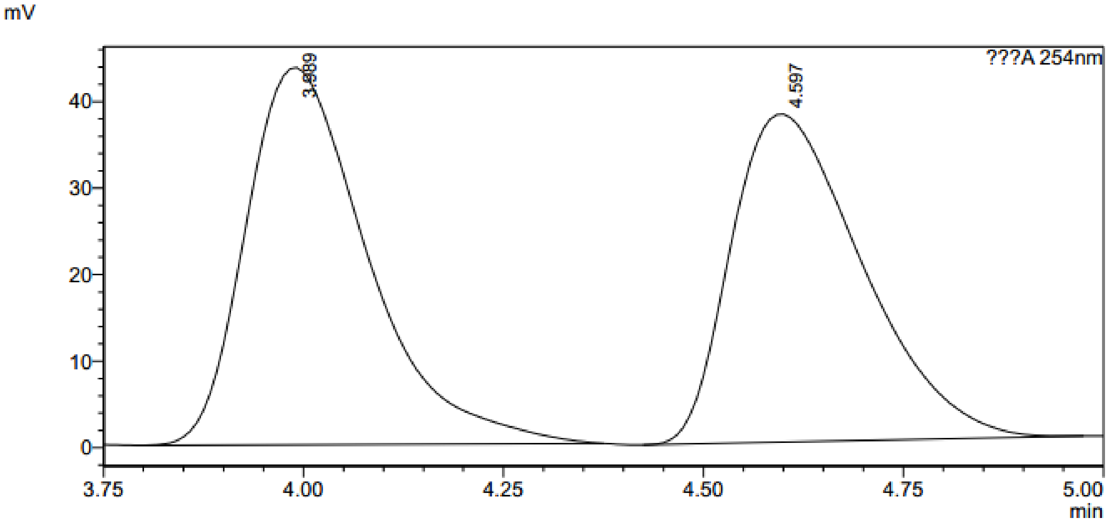

<Peak Table>

???A 254nm

Peak#	Ret. Time	Area	Height	Conc.	Unit	Mark	Name
1	3.989	461745	43570	51.602		M	
2	4.597	433073	37907	48.398		M	
Total		894818	81477				

<Chromatogram>

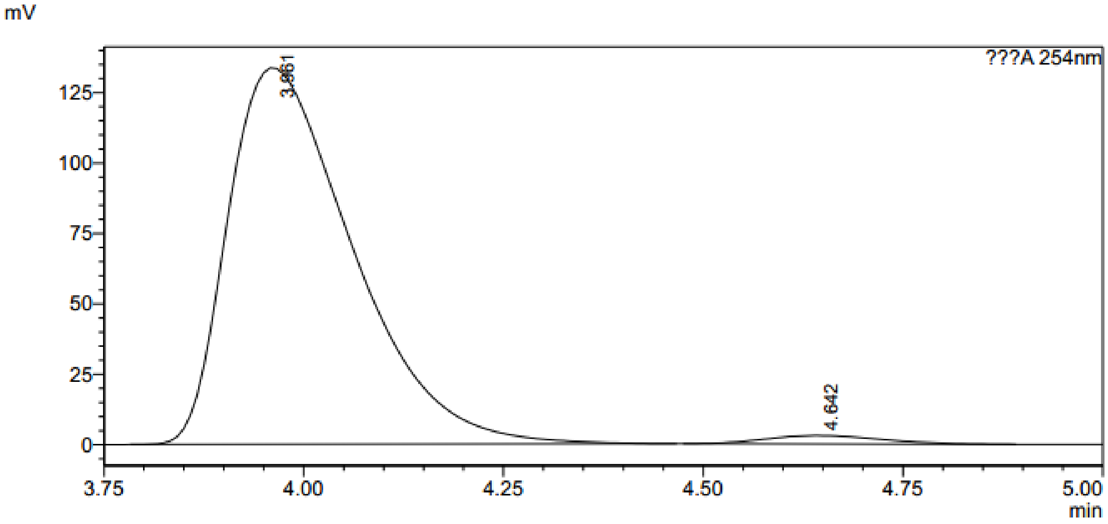

<Peak Table>

???A 254nm

Peak#	Ret. Time	Area	Height	Conc.	Unit	Mark	Name
1	3.961	1451912	133686	97.959		M	
2	4.642	30244	3010	2.041		M	
Total		1482156	136696				

(1*R*,2*S*)-2-Cyclohexyl-2-hydroxy-1-phenylpropyl propionate (**2n**)

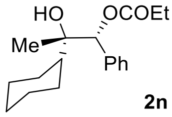

2n

<Chromatogram>

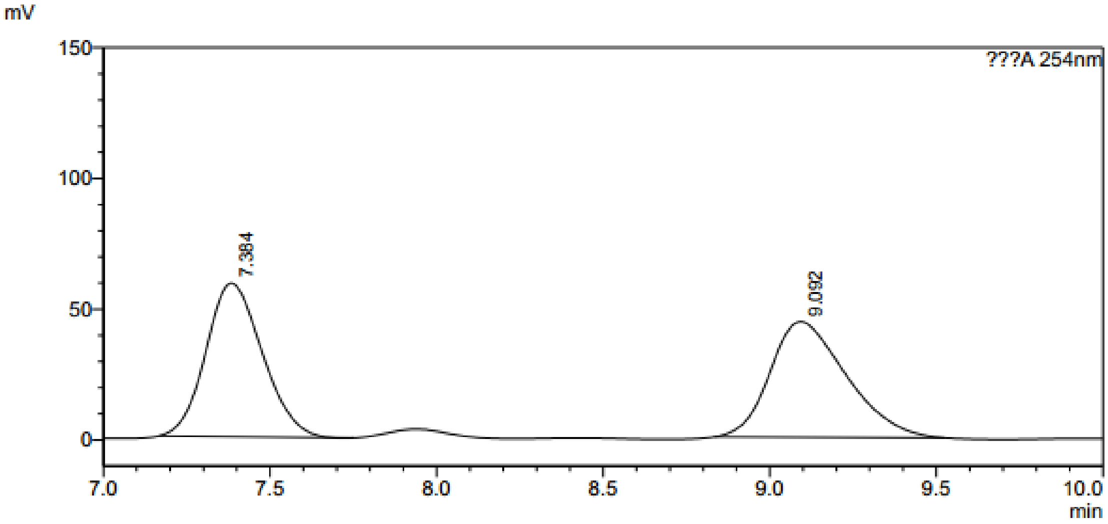

<Peak Table>

???A 254nm

Peak#	Ret. Time	Area	Height	Conc.	Unit	Mark	Name
1	7.384	691762	58710	49.588		M	
2	9.092	703248	44190	50.412		M	
Total		1395010	102899				

<Chromatogram>

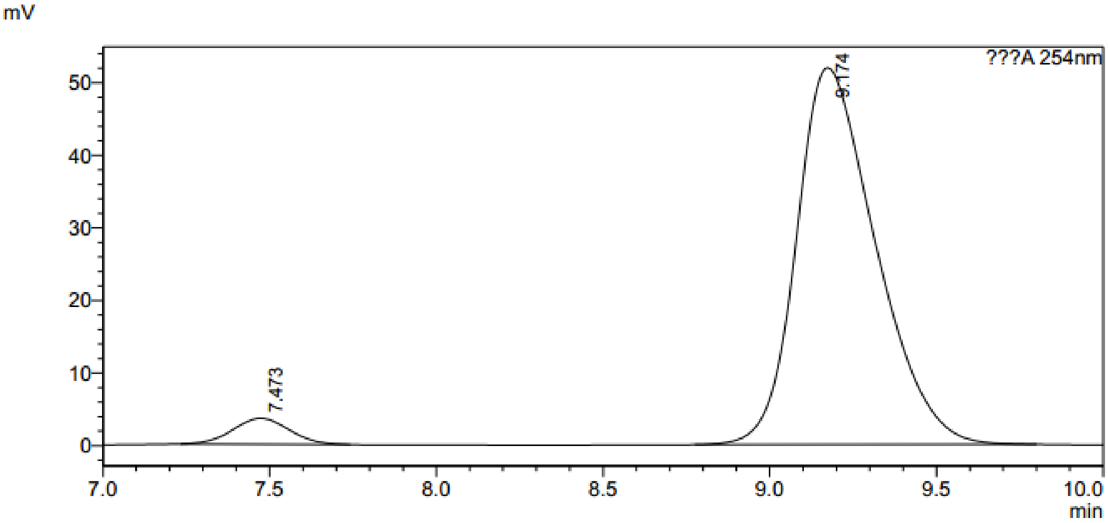

<Peak Table>

???A 254nm

Peak#	Ret. Time	Area	Height	Conc.	Unit	Mark	Name
1	7.473	41929	3561	4.658		M	
2	9.174	858304	51910	95.342		M	
Total		900233	55471				

(1*S*,2*R*)-2,3-Dimethyl-1-phenylbutane-1,2-diol (**1o**)

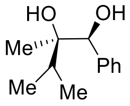

1o

<Chromatogram>

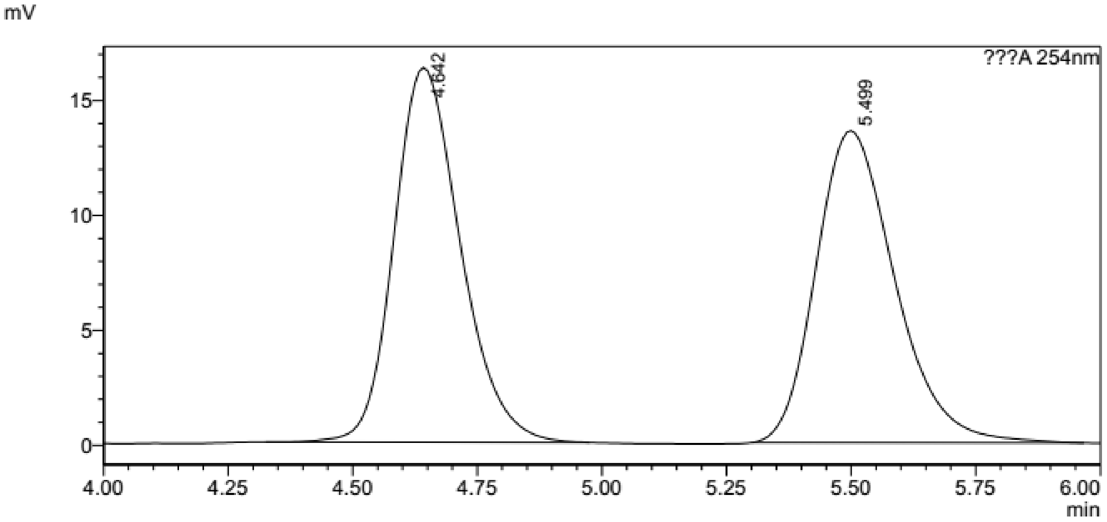

<Peak Table>

???A 254nm

Peak#	Ret. Time	Area	Height	Conc.	Unit	Mark	Name
1	4.642	151322	16296	50.218		M	
2	5.499	150011	13574	49.782		M	
Total		301333	29869				

<Chromatogram>

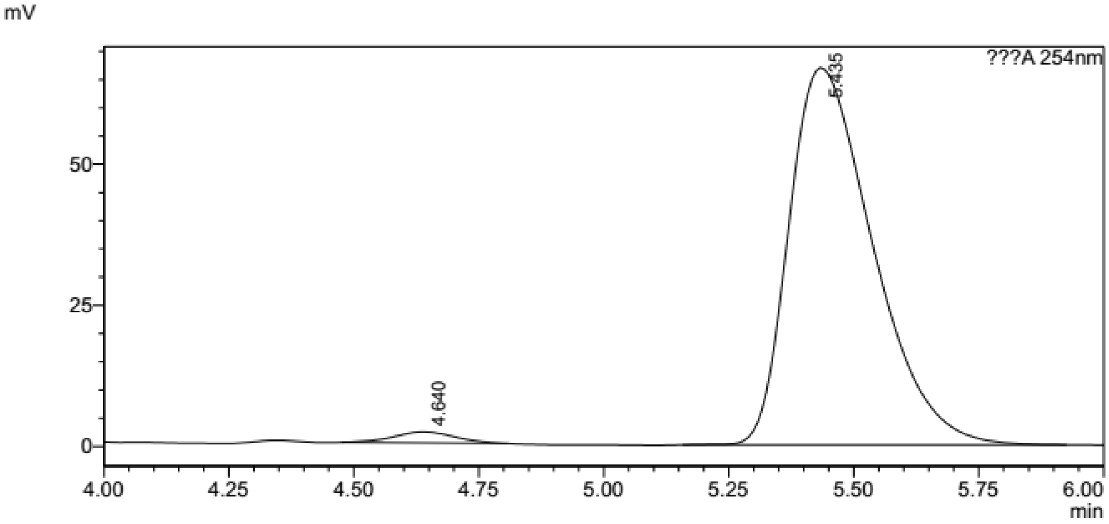

<Peak Table>

???A 254nm

Peak#	Ret. Time	Area	Height	Conc.	Unit	Mark	Name
1	4.640	16964	1967	2.127		M	
2	5.435	780435	66804	97.873		M	
Total		797399	68771				

(1*R*,2*S*)-2-Hydroxy-2,3-dimethyl-1-phenylbutyl propionate (**2o**)

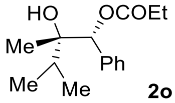

2o

<Chromatogram>

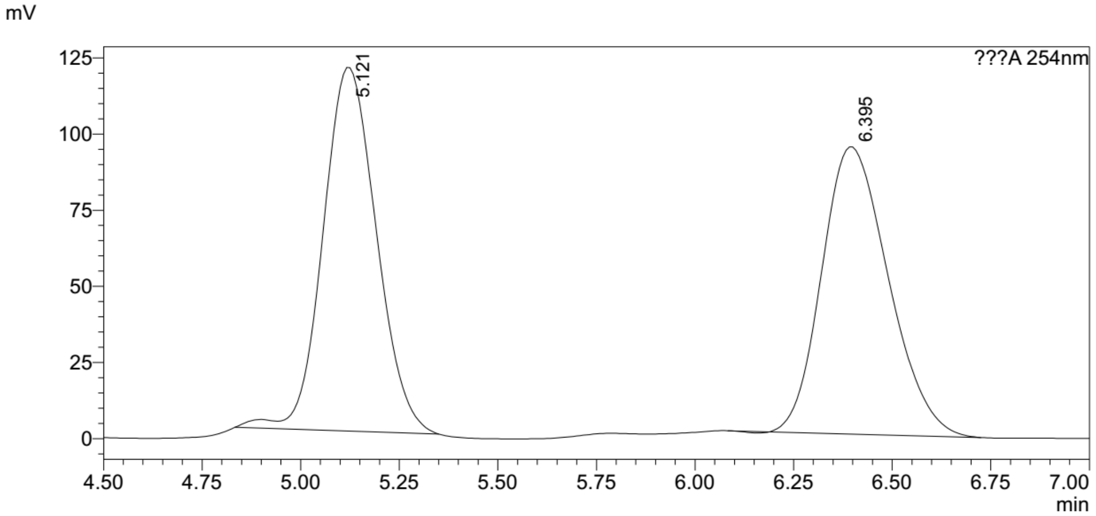

<Peak Table>

???A 254nm

Peak#	Ret. Time	Area	Height	Conc.	Unit	Mark	Name
1	5.121	1126174	119378	50.437		M	
2	6.395	1106661	94287	49.563		M	
Total		2232835	213665				

<Chromatogram>

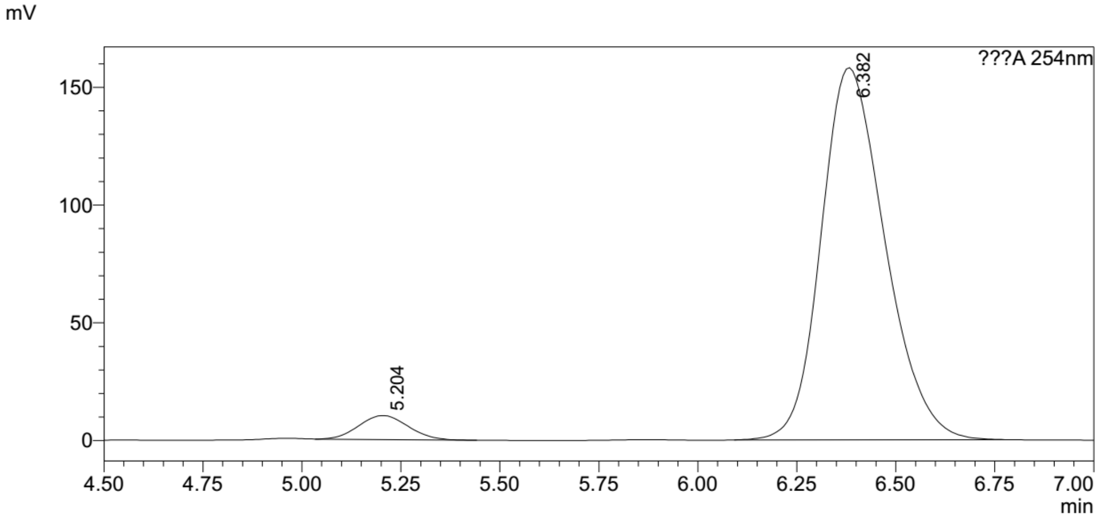

<Peak Table>

???A 254nm

Peak#	Ret. Time	Area	Height	Conc.	Unit	Mark	Name
1	5.204	88532	10202	4.760			
2	6.382	1771334	157949	95.240		M	
Total		1859866	168152				

(1*S*,2*S*)-2-Methyl-1-phenylpent-4-ene-1,2-diol (**1p**)

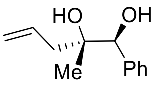

1p

<Chromatogram>

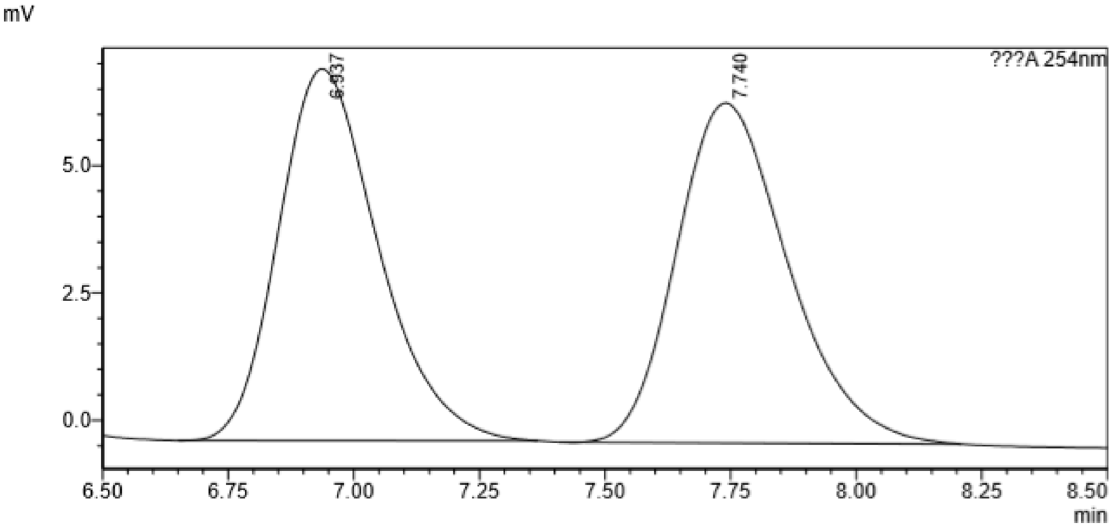

<Peak Table>

???A 254nm

Peak#	Ret. Time	Area	Height	Conc.	Unit	Mark	Name
1	6.937	100938	7295	49.484		M	
2	7.740	103041	6674	50.516		M	
Total		203979	13969				

<Chromatogram>

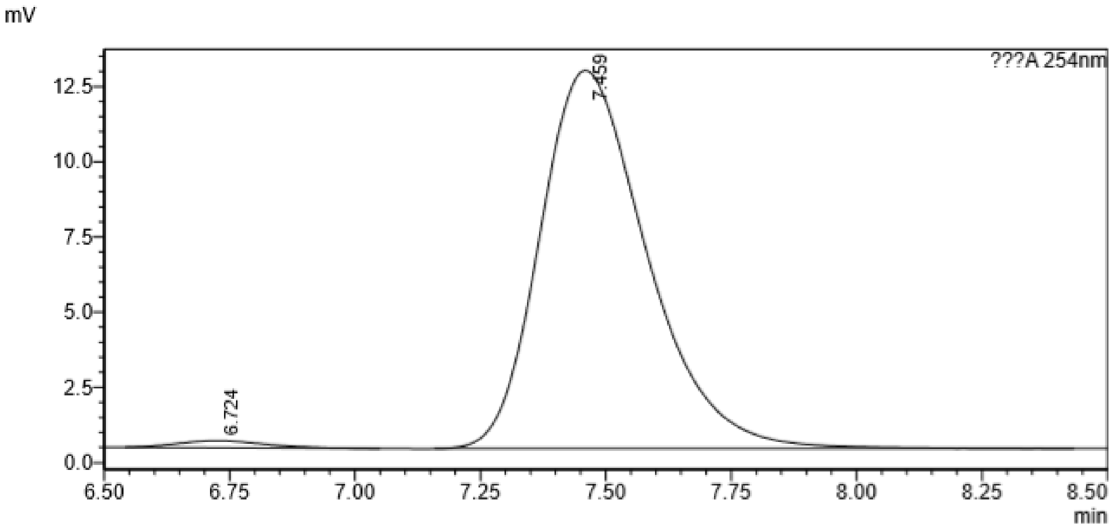

<Peak Table>

???A 254nm

Peak#	Ret. Time	Area	Height	Conc.	Unit	Mark	Name
1	6.724	2845	235	1.500		M	
2	7.459	186866	12574	98.500		M	
Total		189711	12810				

(1*R*,2*R*)-2-Hydroxy-2-methyl-1-phenylpent-4-en-1-yl propionate (**2p**)

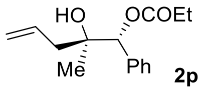

2p

<Chromatogram>

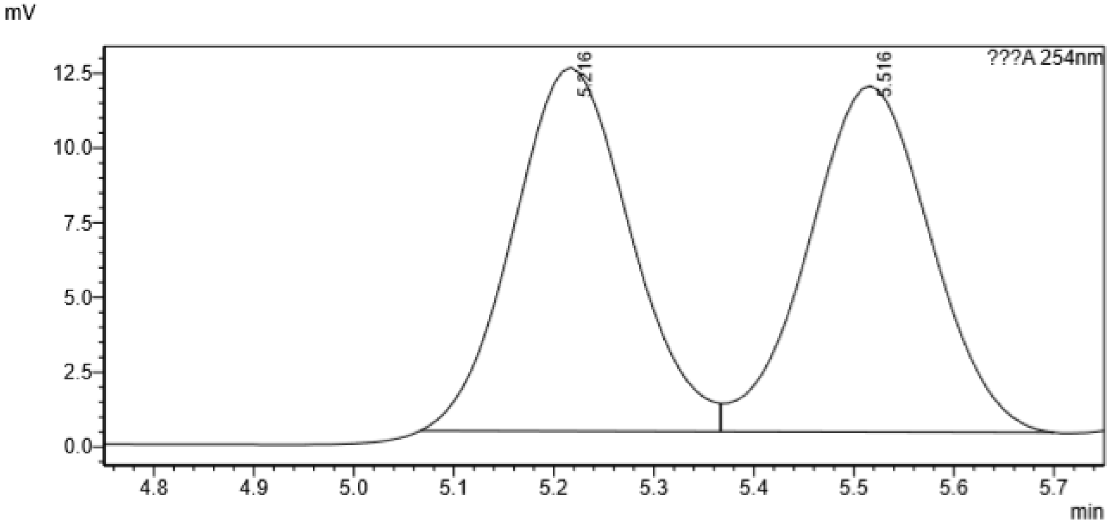

<Peak Table>

???A 254nm

Peak#	Ret. Time	Area	Height	Conc.	Unit	Mark	Name
1	5.216	99340	12164	50.277			
2	5.516	98246	11575	49.723		V	
Total		197586	23740				

<Chromatogram>

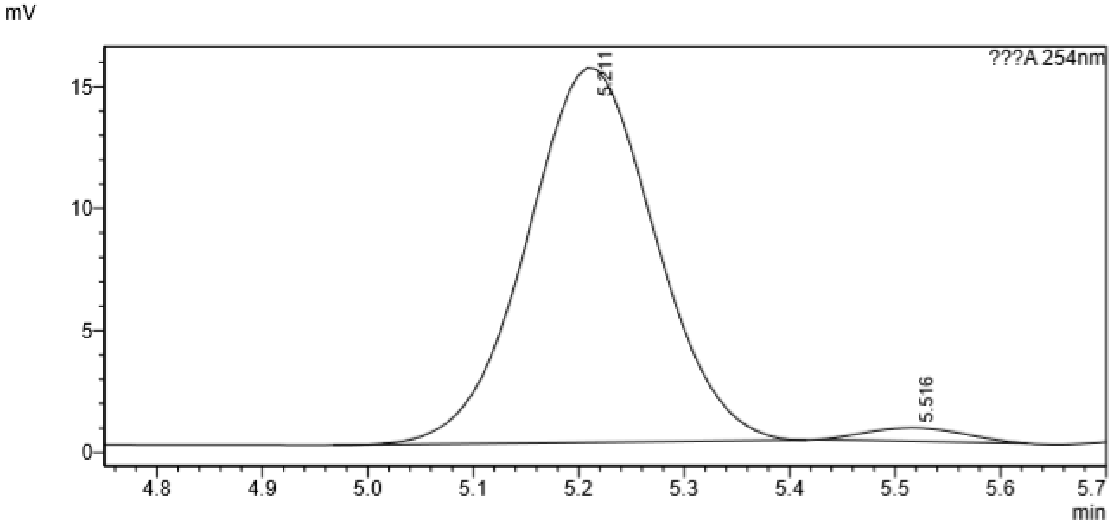

<Peak Table>

???A 254nm

Peak#	Ret. Time	Area	Height	Conc.	Unit	Mark	Name
1	5.211	129852	15356	97.348		M	
2	5.516	3538	549	2.652		M	
Total		133390	15905				

(1*S*,2*S*)-1-(4-Methoxyphenyl)-2,6-dimethylhept-5-ene-1,2-diol (**1q**)

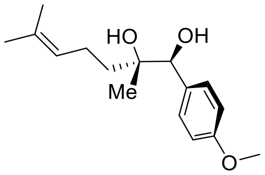

1q

<Chromatogram>

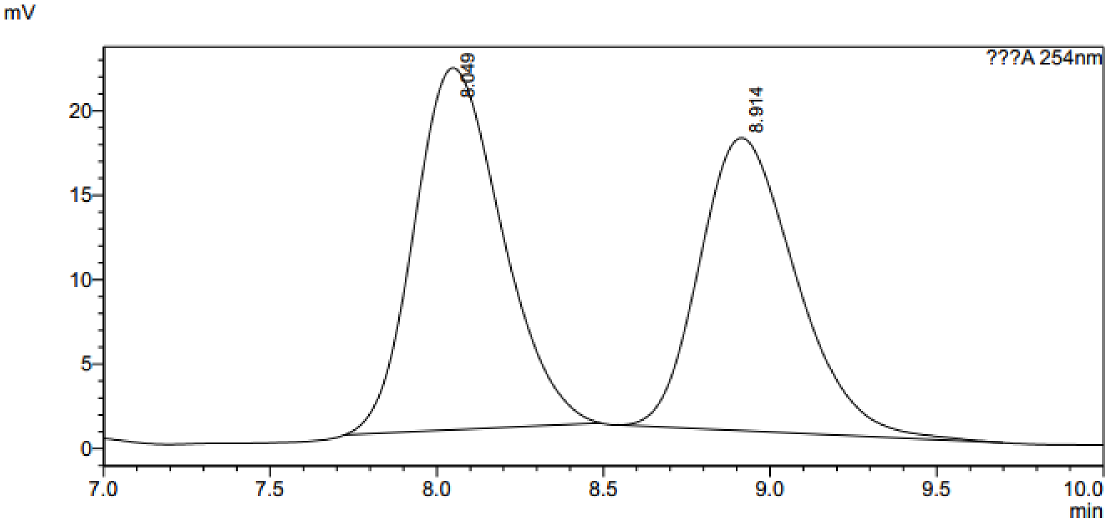

<Peak Table>

???A 254nm

Peak#	Ret. Time	Area	Height	Conc.	Unit	Mark	Name
1	8.049	392533	21425	52.810		M	
2	8.914	350762	17329	47.190		M	
Total		743295	38754				

<Chromatogram>

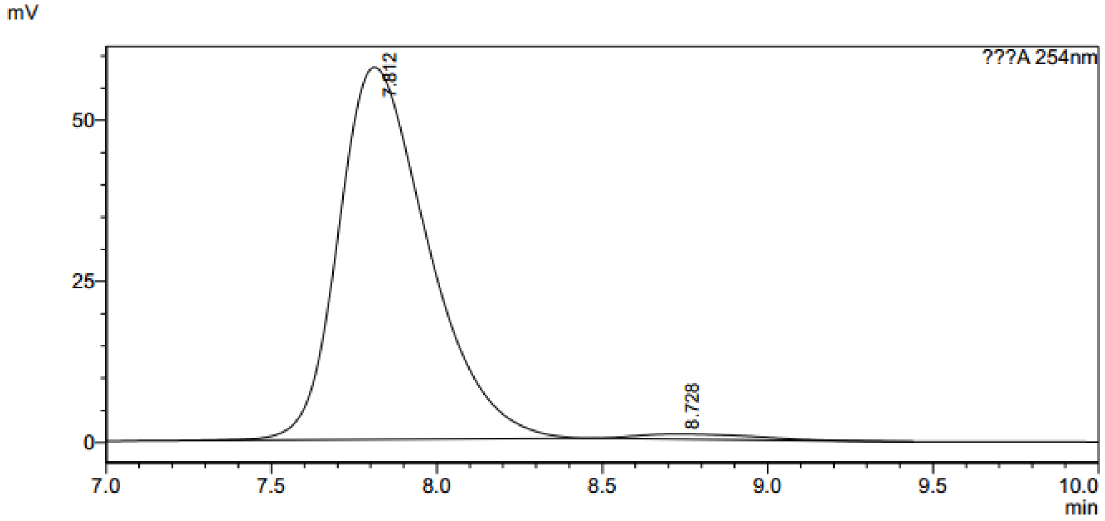

<Peak Table>

???A 254nm

Peak#	Ret. Time	Area	Height	Conc.	Unit	Mark	Name
1	7.812	1089280	57735	98.236		M	
2	8.728	19556	791	1.764		M	
Total		1108836	58526				

(1*R*,2*R*)-2-Hydroxy-1-(4-methoxyphenyl)-2,6-dimethylhept-5-en-1-yl propionate (**2q**)

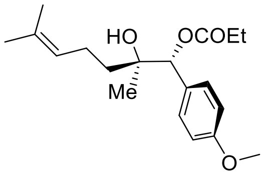

2q

<Chromatogram>

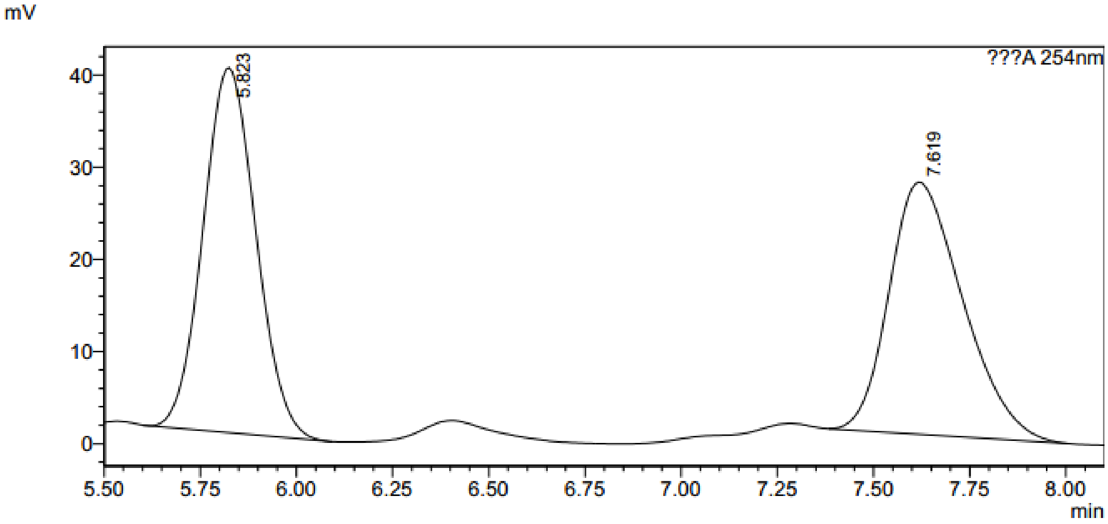

<Peak Table>

???A 254nm

Peak#	Ret. Time	Area	Height	Conc.	Unit	Mark	Name
1	5.823	378228	39591	50.887		M	
2	7.619	365036	27374	49.113		M	
Total		743264	66965				

<Chromatogram>

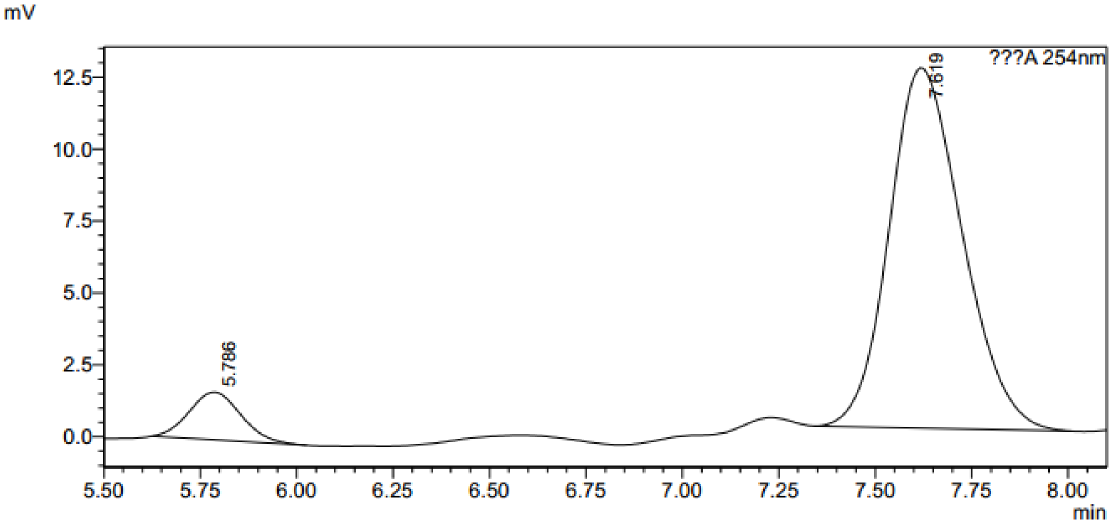

<Peak Table>

???A 254nm

Peak#	Ret. Time	Area	Height	Conc.	Unit	Mark	Name
1	5.786	15428	1654	8.523		M	
2	7.619	165584	12524	91.477		M	
Total		181012	14178				

(1*S*,2*R*)-2-Ethyl-1-phenylhexane-1,2-diol (**1r**)

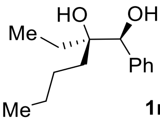

1r

<Chromatogram>

mV

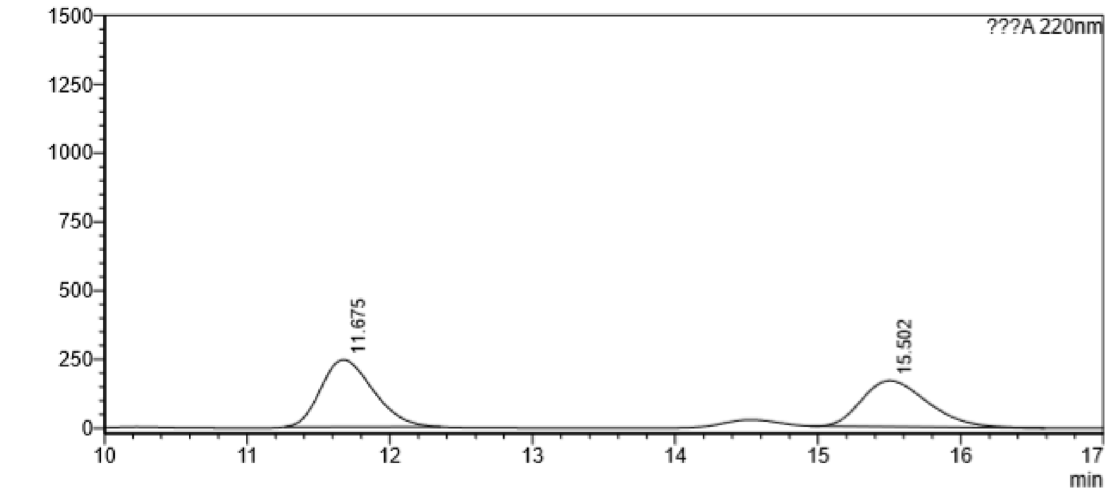

<Peak Table>

???A 220nm

Peak#	Ret. Time	Area	Height	Conc.	Unit	Mark	Name
1	11.675	6262046	244239	53.832		M	
2	15.502	5370593	167052	46.168		M	
Total		11632639	411291				

<Chromatogram>

mV

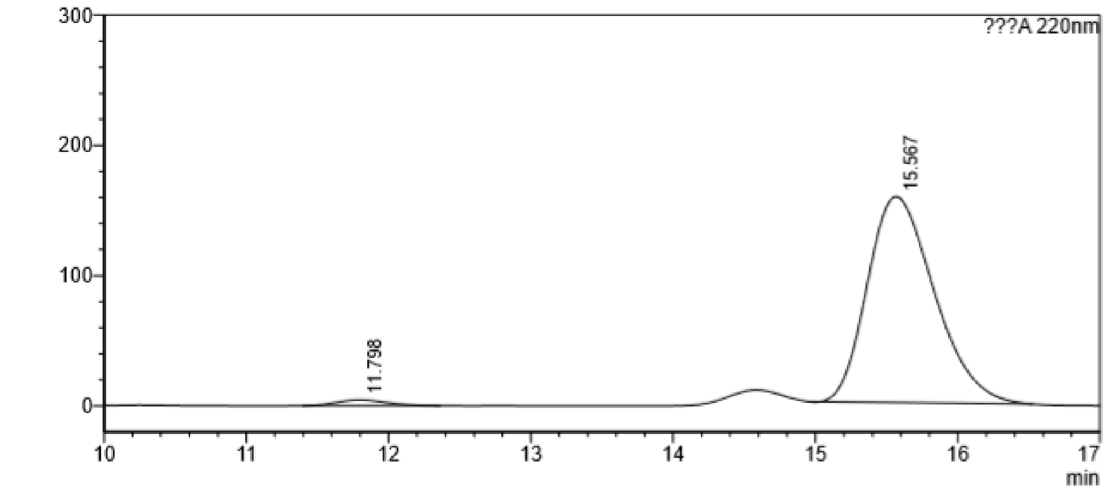

<Peak Table>

???A 220nm

Peak#	Ret. Time	Area	Height	Conc.	Unit	Mark	Name
1	11.798	106824	4404	2.023		M	
2	15.567	5174685	158139	97.977		M	
Total		5281509	162543				

(1*R*,2*S*)-2-Ethyl-2-hydroxy-1-phenylhexyl propionate (**2r**)

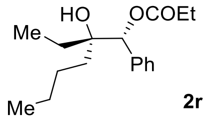

2r

<Chromatogram>

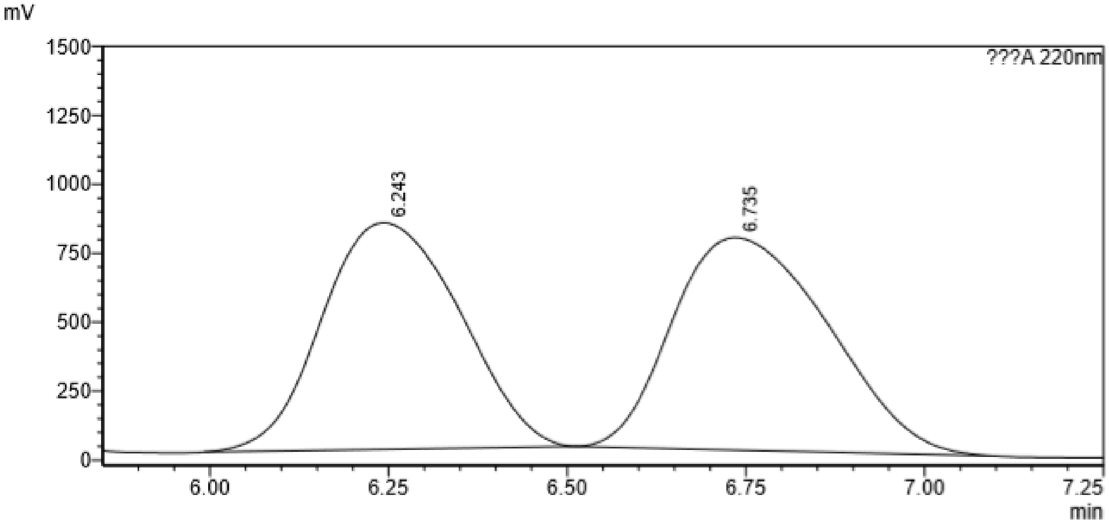

<Peak Table>

???A 220nm

Peak#	Ret. Time	Area	Height	Conc.	Unit	Mark	Name
1	6.243	11042268	822382	48.519		M	
2	6.735	11716218	770792	51.481		M	
Total		22758486	1593174				

<Chromatogram>

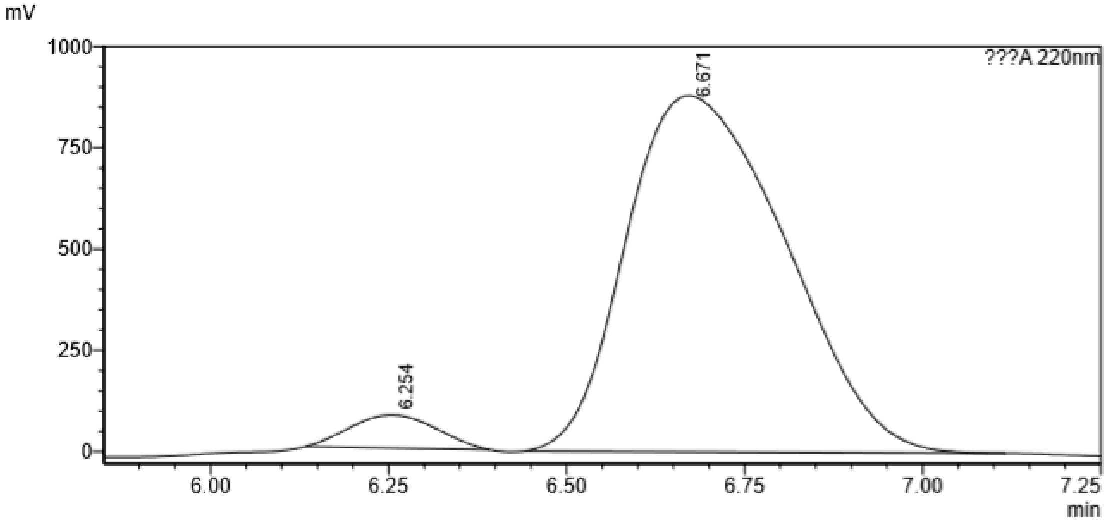

<Peak Table>

???A 220nm

Peak#	Ret. Time	Area	Height	Conc.	Unit	Mark	Name
1	6.254	687637	81874	4.783		M	
2	6.671	13688144	878568	95.217		M	
Total		14375781	960442				

(1*S*,2*S*)-2-Benzyl-1-phenylbutane-1,2-diol (**1s**)

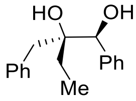

1s

<Chromatogram>

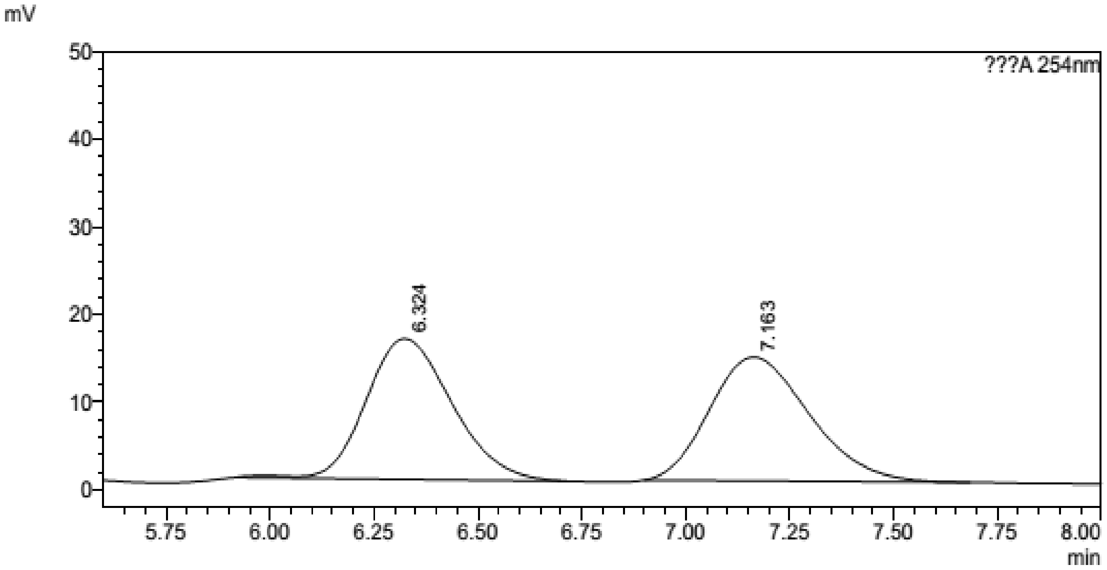

<Peak Table>

???A 254nm

Peak#	Ret. Time	Area	Height	Conc.	Unit	Mark	Name
1	6.324	230528	16033	49.398		M	
2	7.163	236151	14123	50.602		M	
Total		466679	30156				

<Chromatogram>

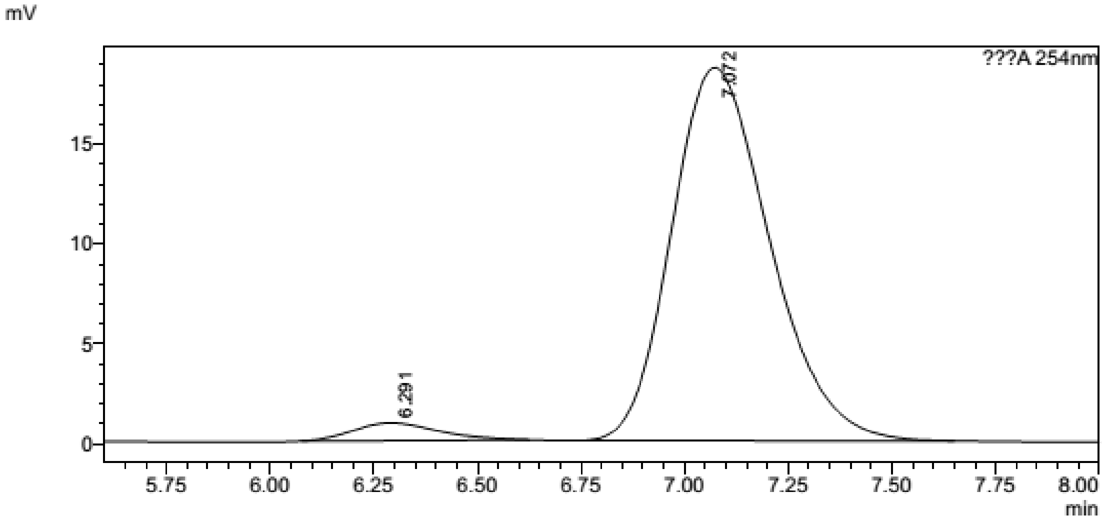

<Peak Table>

???A 254nm

Peak#	Ret. Time	Area	Height	Conc.	Unit	Mark	Name
1	6.291	12833	893	3.986		M	
2	7.072	309109	18713	96.014		M	
Total		321942	19607				

(1*R*,2*R*)-2-Benzyl-2-hydroxy-1-phenylbutyl propionate (**2s**)

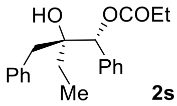

2s

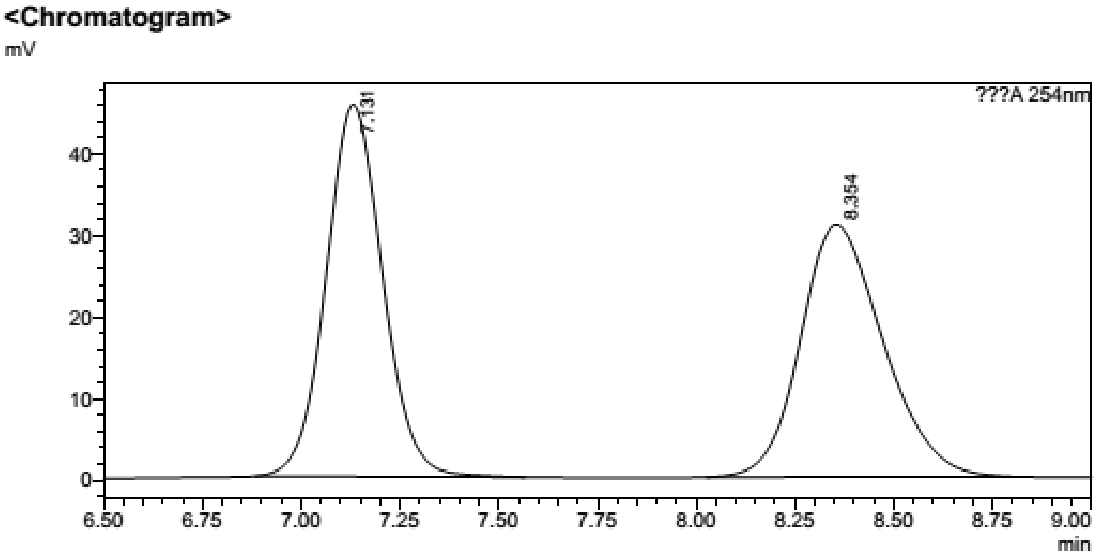

<Peak Table>

???A 254nm

Peak#	Ret. Time	Area	Height	Conc.	Unit	Mark	Name
1	7.131	454878	45623	50.206		M	
2	8.354	451154	30898	49.794		M	
Total		906032	76520				

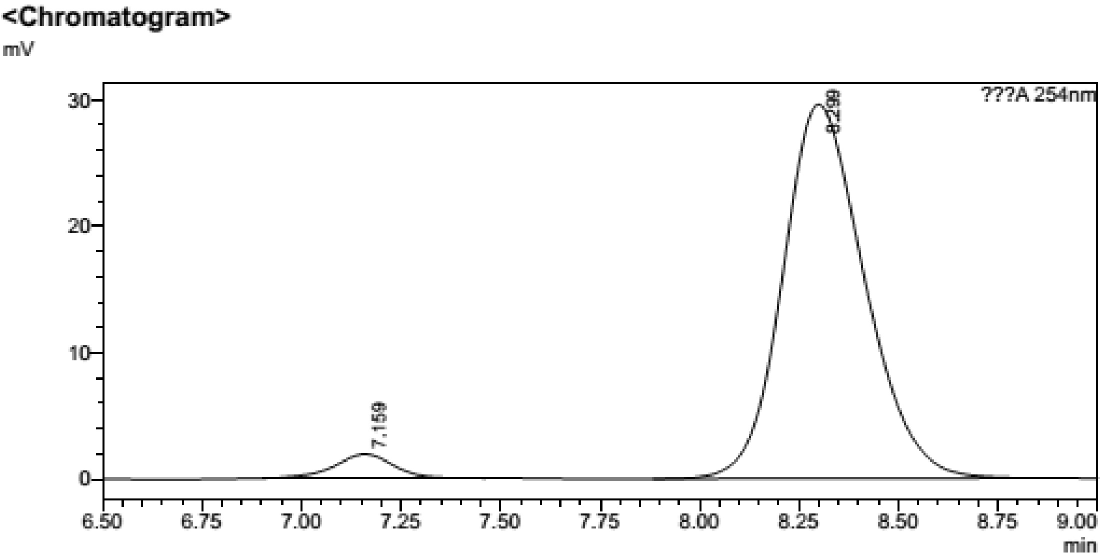

<Peak Table>

???A 254nm

Peak#	Ret. Time	Area	Height	Conc.	Unit	Mark	Name
1	7.159	19515	1900	4.404		M	
2	8.299	423549	29588	95.596		M	
Total		443064	31488				

(1*S*,2*S*)-2-Benzyl-1,4-diphenylbutane-1,2-diol (**1t**)

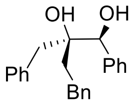

1t

<Chromatogram>

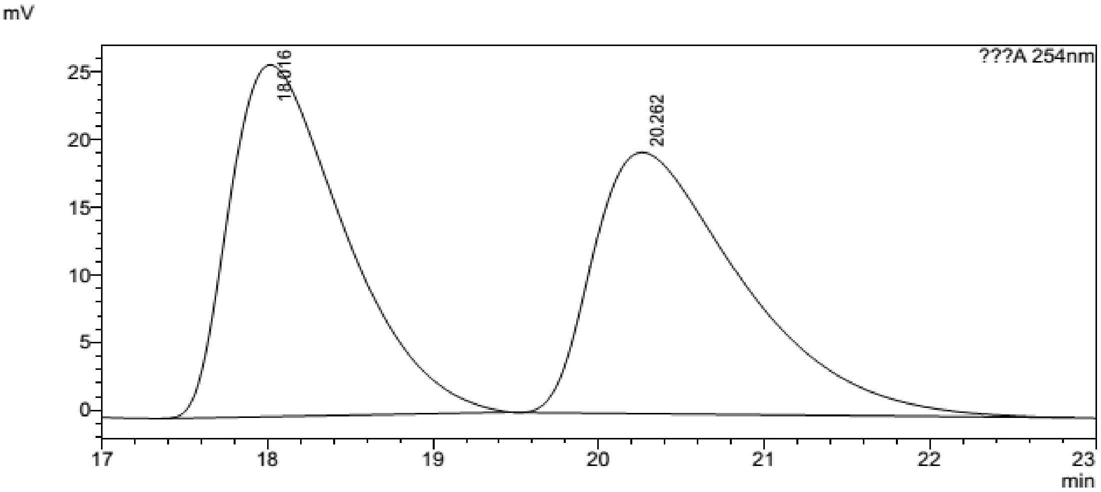

<Peak Table>

???A 254nm

Peak#	Ret. Time	Area	Height	Conc.	Unit	Mark	Name
1	18.016	1253814	26002	50.814		M	
2	20.262	1213620	19307	49.186		M	
Total		2467434	45309				

<Chromatogram>

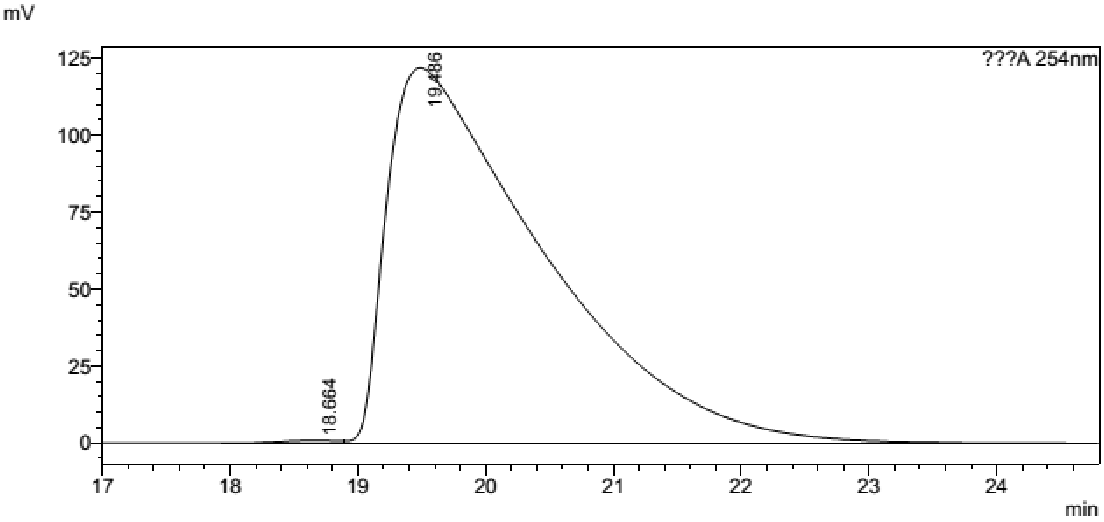

<Peak Table>

???A 254nm

Peak#	Ret. Time	Area	Height	Conc.	Unit	Mark	Name
1	18.664	30477	962	0.292			
2	19.486	10411901	121934	99.708		V	
Total		10442378	122896				

(1*R*,2*R*)-2-Benzyl-2-hydroxy-1,4-diphenylbutyl propionate (**2t**)

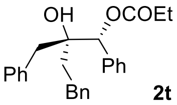

2t

<Chromatogram>

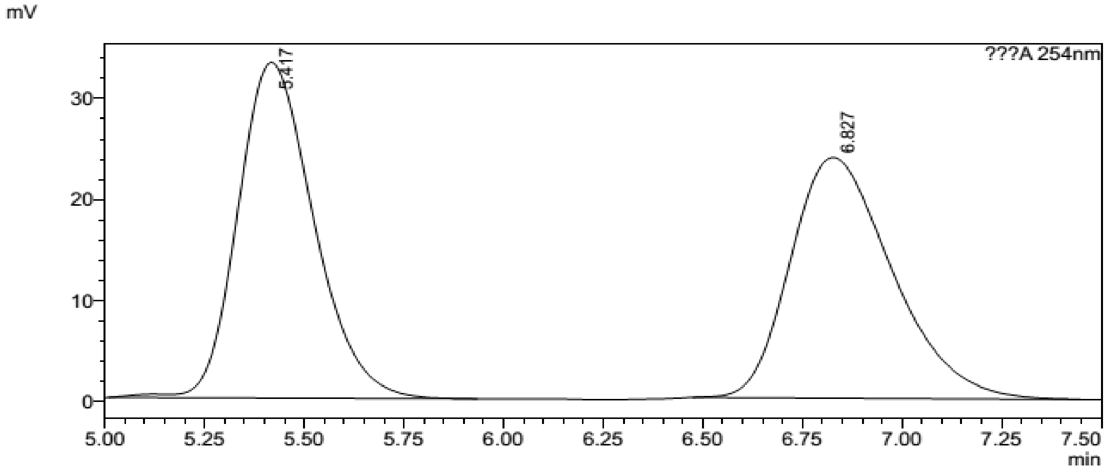

<Peak Table>

???A 254nm

Peak#	Ret. Time	Area	Height	Conc.	Unit	Mark	Name
1	5.417	440844	33219	50.976		M	
2	6.827	423965	23817	49.024		M	
Total		864809	57036				

<Chromatogram>

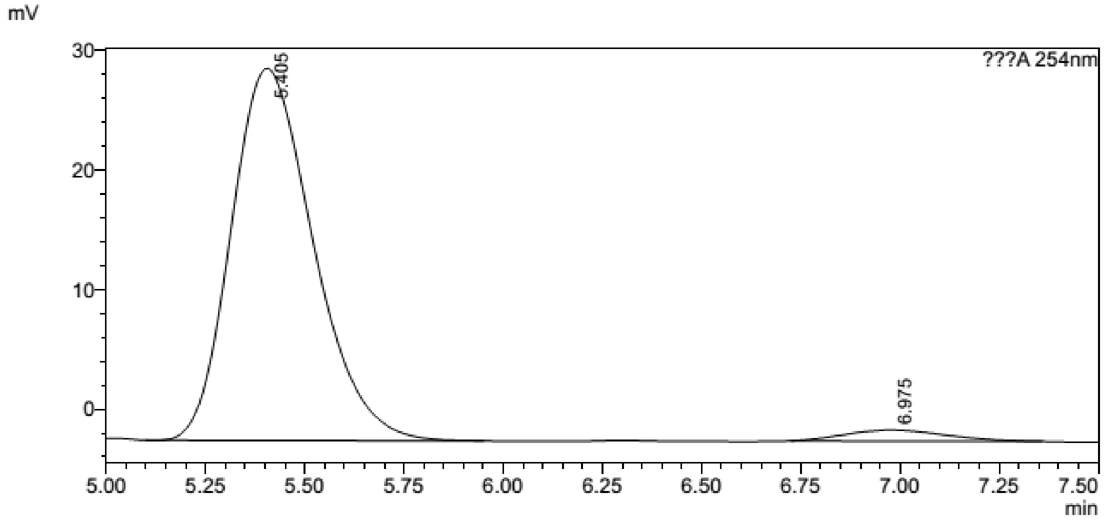

<Peak Table>

???A 254nm

Peak#	Ret. Time	Area	Height	Conc.	Unit	Mark	Name
1	5.405	448761	31144	96.428		M	
2	6.975	16623	930	3.572		M	
Total		465384	32074				

(1*S*,2*S*)-2-(4-Methylbenzyl)-1,4-diphenylbutane-1,2-diol (**1u**)

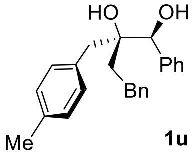

1u

<Chromatogram>

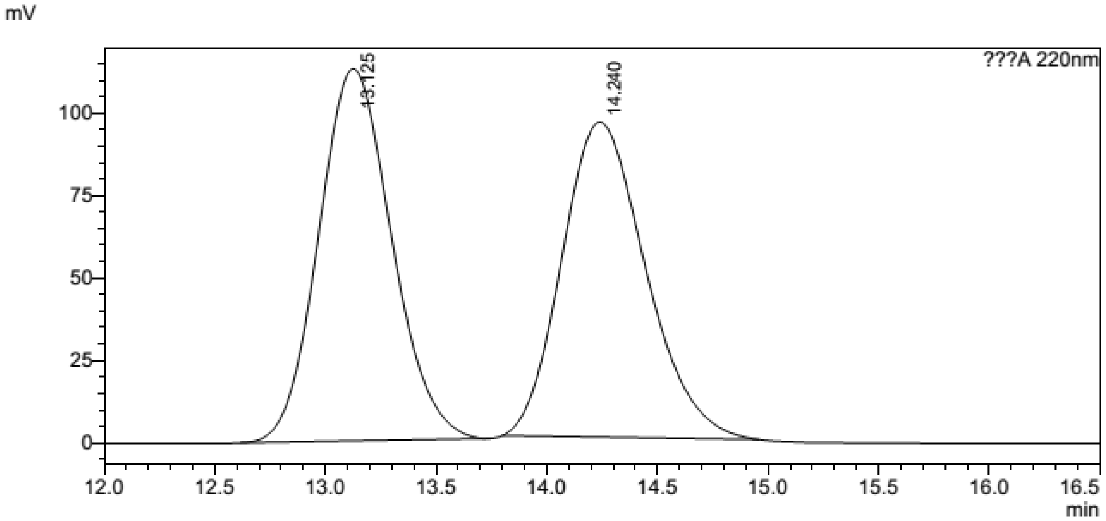

<Peak Table>

???A 220nm

Peak#	Ret. Time	Area	Height	Conc.	Unit	Mark	Name
1	13.125	2585484	112819	50.726		M	
2	14.240	2511441	95467	49.274		M	
Total		5096925	208286				

<Chromatogram>

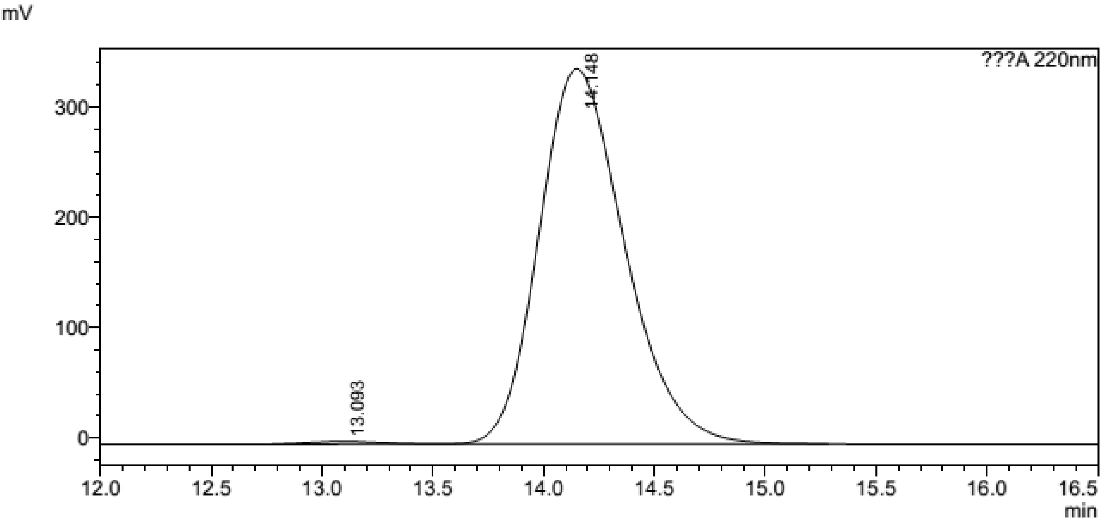

<Peak Table>

???A 220nm

Peak#	Ret. Time	Area	Height	Conc.	Unit	Mark	Name
1	13.093	52374	2500	0.569		M	
2	14.148	9154585	340249	99.431		M	
Total		9206960	342750				

(1*R*,2*R*)-2-Hydroxy-2-(4-methylbenzyl)-1,4-diphenylbutyl propionate (**2u**)

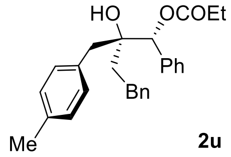

2u

<Chromatogram>

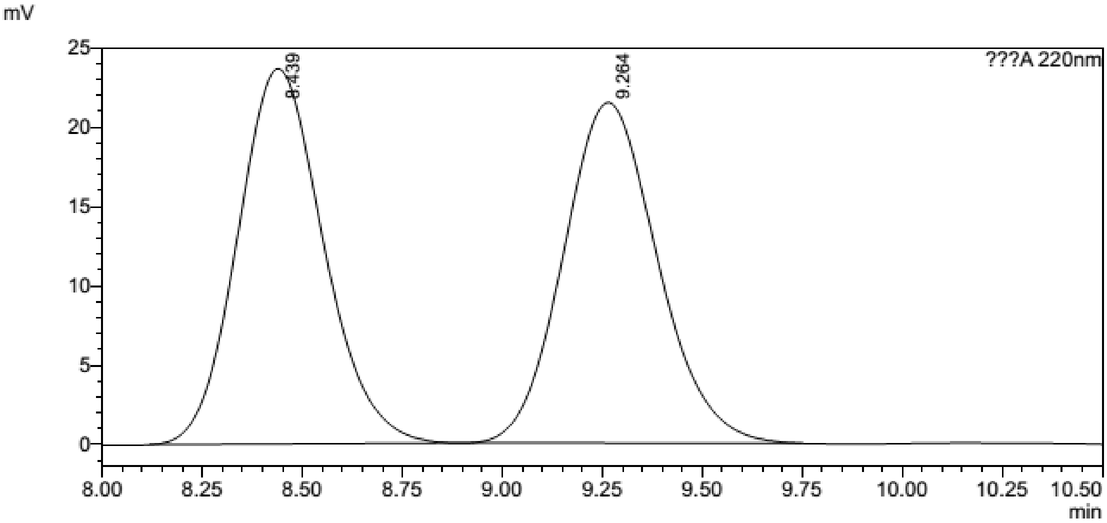

<Peak Table>

???A 220nm

Peak#	Ret. Time	Area	Height	Conc.	Unit	Mark	Name
1	8.439	352757	23639	50.078		M	
2	9.264	351652	21457	49.922		M	
Total		704409	45096				

<Chromatogram>

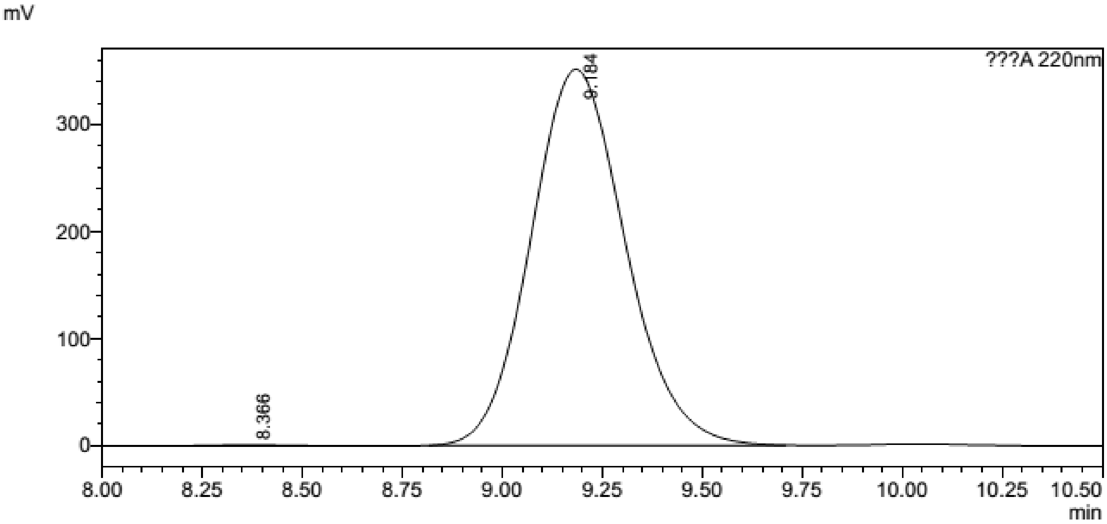

<Peak Table>

???A 220nm

Peak#	Ret. Time	Area	Height	Conc.	Unit	Mark	Name
1	8.366	9537	653	0.165		M	
2	9.184	5770114	351333	99.835			
Total		5779651	351985				

(1*S*,2*R*)-2-Phenethyl-1-phenylhexane-1,2-diol (**1v**)

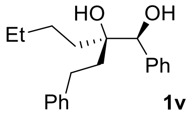

1v

<Chromatogram>

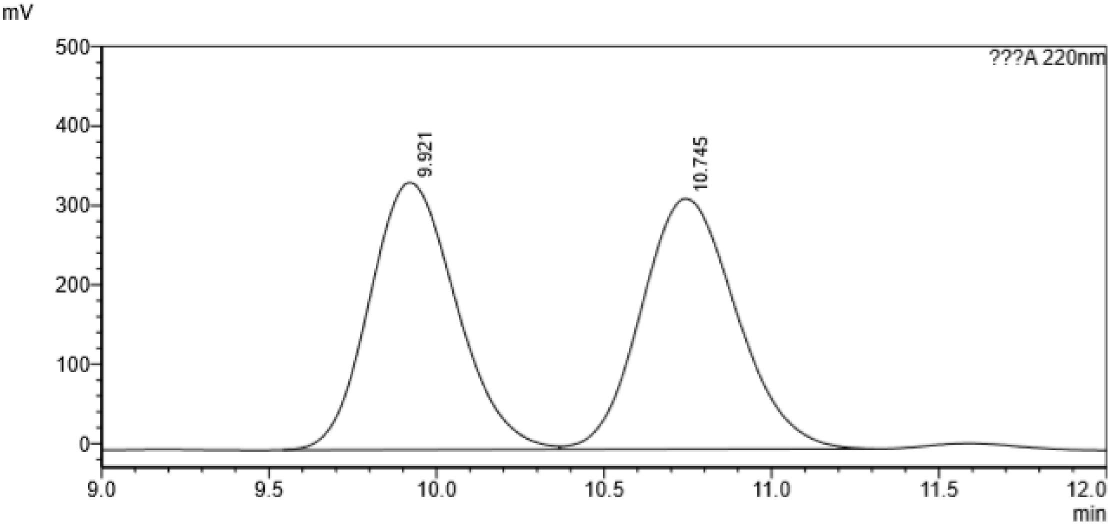

<Peak Table>

???A 220nm

Peak#	Ret. Time	Area	Height	Conc.	Unit	Mark	Name
1	9.921	6071068	336335	49.285			
2	10.745	6247200	315746	50.715		V	
Total		12318269	652081				

<Chromatogram>

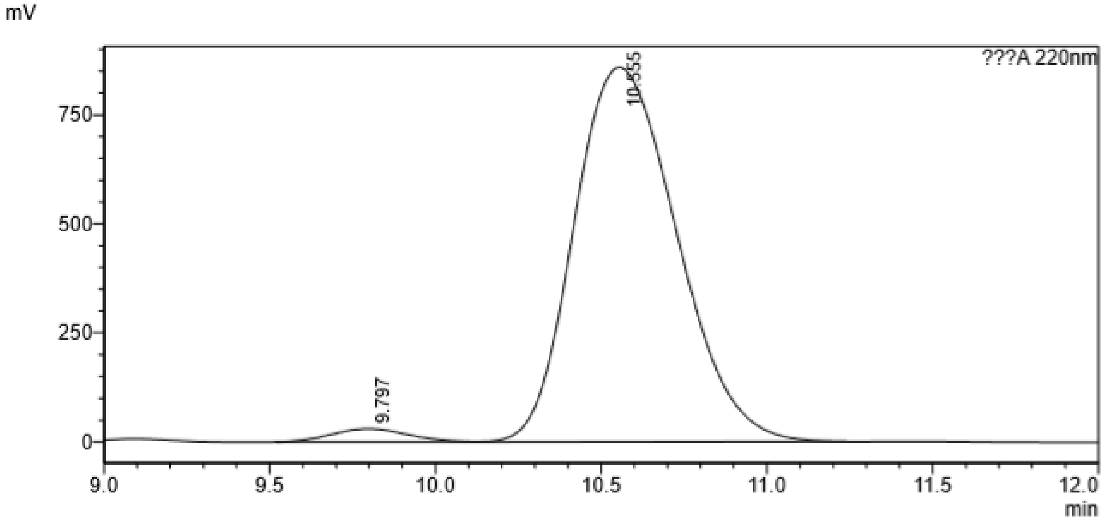

<Peak Table>

???A 220nm

Peak#	Ret. Time	Area	Height	Conc.	Unit	Mark	Name
1	9.797	488239	30321	2.570			
2	10.555	18506806	858052	97.430		V	
Total		18995045	888373				

<u>(1*S*,2*R*)-2-Phenethyl-1-phenylhexane-1,2-diol (**1v**)</u>

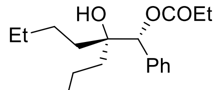

2v

<Chromatogram>

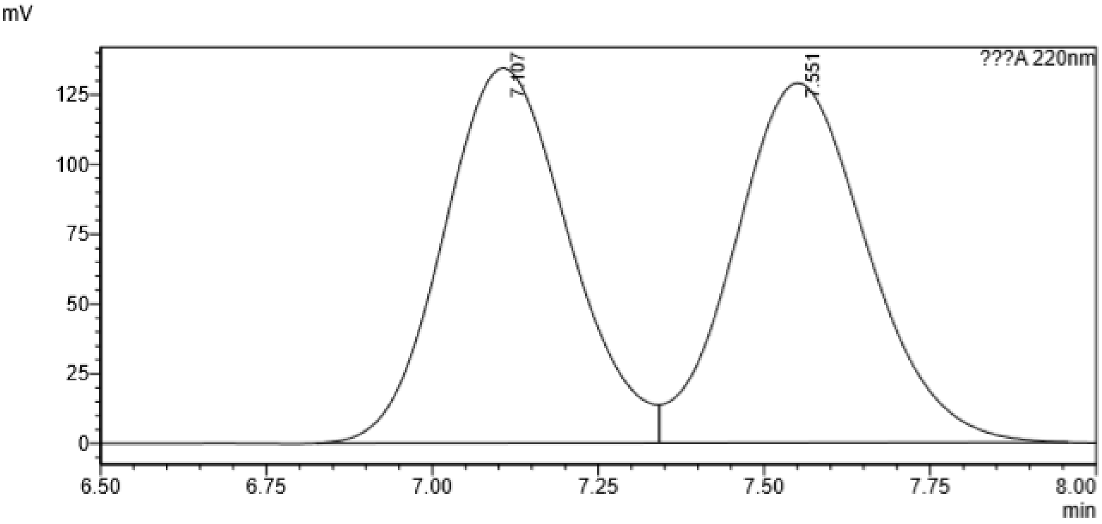

<Peak Table>

???A 220nm

Peak#	Ret. Time	Area	Height	Conc.	Unit	Mark	Name
1	7.107	1764612	134358	49.629			
2	7.551	1790973	128983	50.371		V	
Total		3555585	263341				

<Chromatogram>

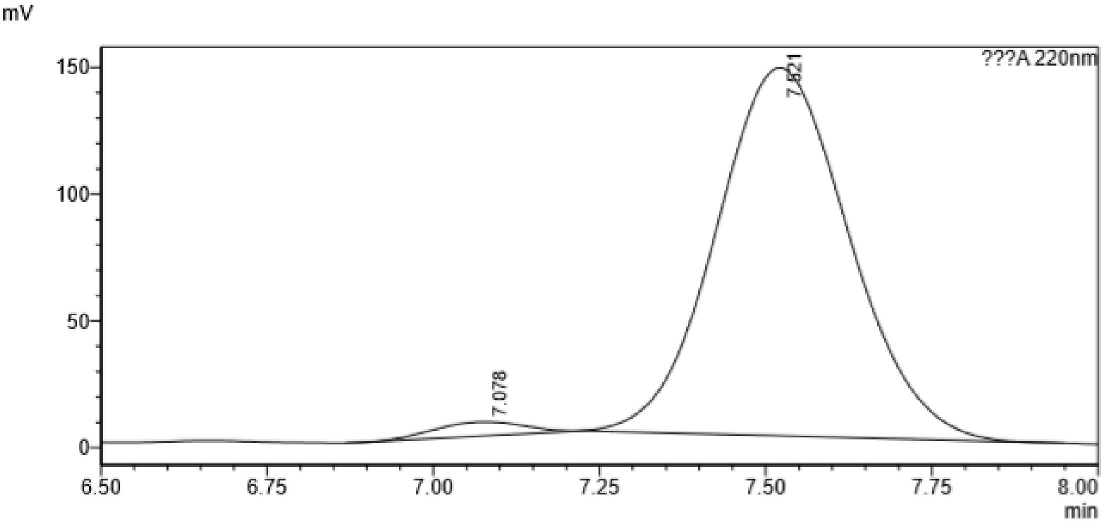

<Peak Table>

???A 220nm

Peak#	Ret. Time	Area	Height	Conc.	Unit	Mark	Name
1	7.078	52269	5475	2.526		M	
2	7.521	2016697	144972	97.474		M	
Total		2068966	150448				

(1*S*,2*R*)-2-(*tert*-Butyl)-1-phenylhexane-1,2-diol (**1w**)

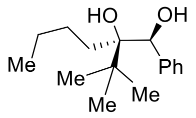

<Chromatogram>

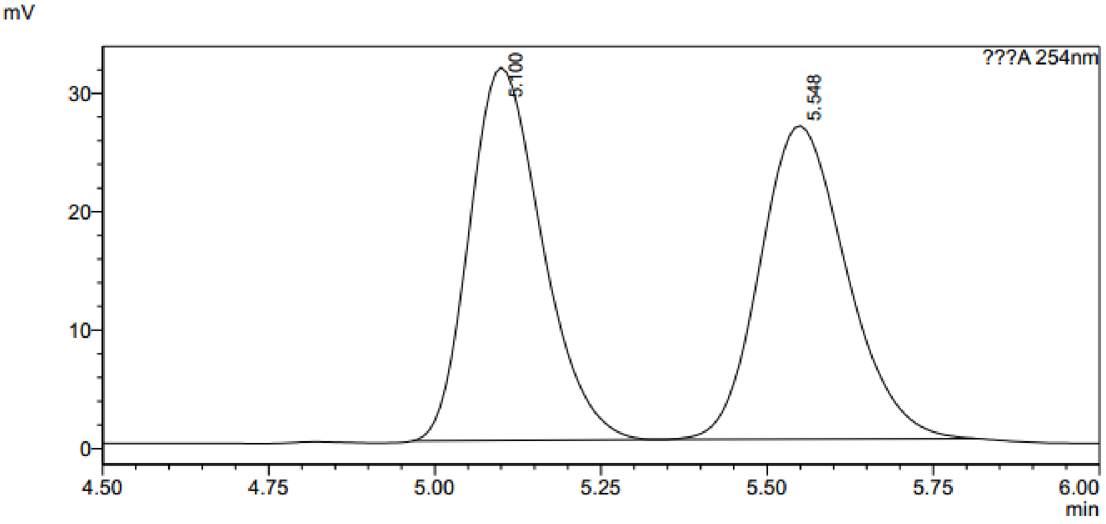

<Peak Table>

???A 254nm

Peak#	Ret. Time	Area	Height	Conc.	Unit	Mark	Name
1	5.100	239755	31511	49.946		M	
2	5.548	240269	26475	50.054		M	
Total		480024	57986				

<Chromatogram>

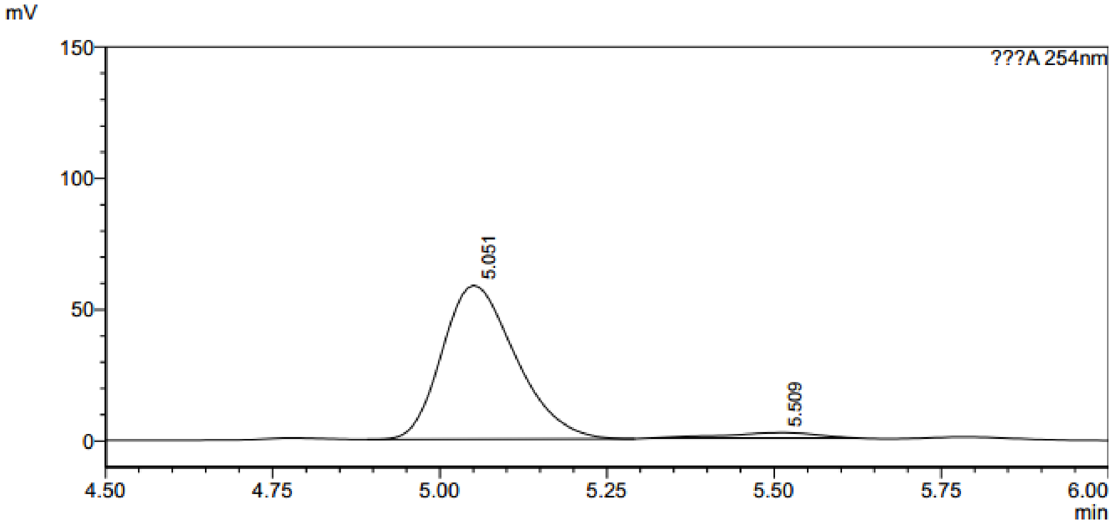

<Peak Table>

???A 254nm

Peak#	Ret. Time	Area	Height	Conc.	Unit	Mark	Name
1	5.051	449737	58606	95.711		M	
2	5.509	20156	2107	4.289		M	
Total		469893	60713				

(1*R*,2*S*)-2-(*tert*-Butyl)-2-hydroxy-1-phenylhexyl propionate (**2w**)

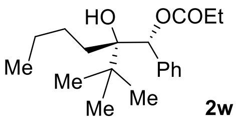

2w

<Chromatogram>

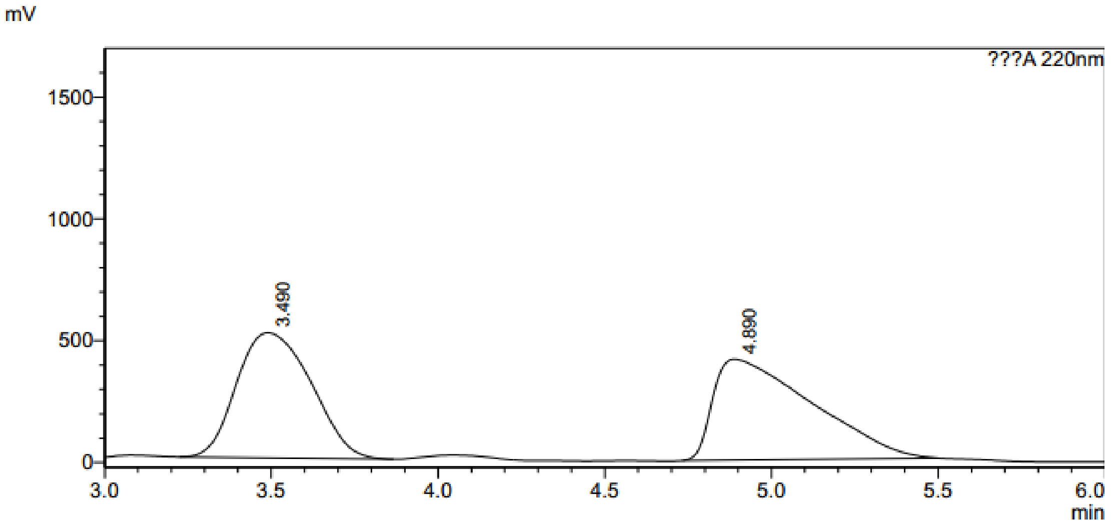

<Peak Table>

???A 220nm

Peak#	Ret. Time	Area	Height	Conc.	Unit	Mark	Name
1	3.490	7989789	513996	48.390		M	
2	4.890	8521494	414535	51.610		M	
Total		16511284	928530				

<Chromatogram>

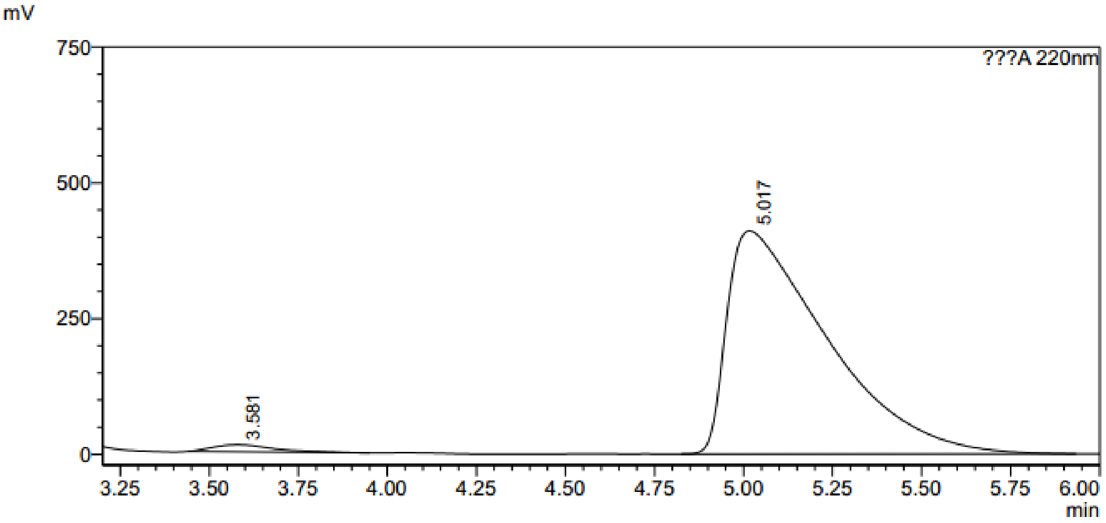

<Peak Table>

???A 220nm

Peak#	Ret. Time	Area	Height	Conc.	Unit	Mark	Name
1	3.581	157104	12923	1.916		M	
2	5.017	8042188	411146	98.084		M	
Total		8199292	424069				

(1*S*,2*S*)-2-*iso*-Propyl-3,3-dimethyl-1-phenylbutane-1,2-diol (**1x**)

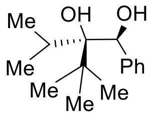

1x

<Chromatogram>

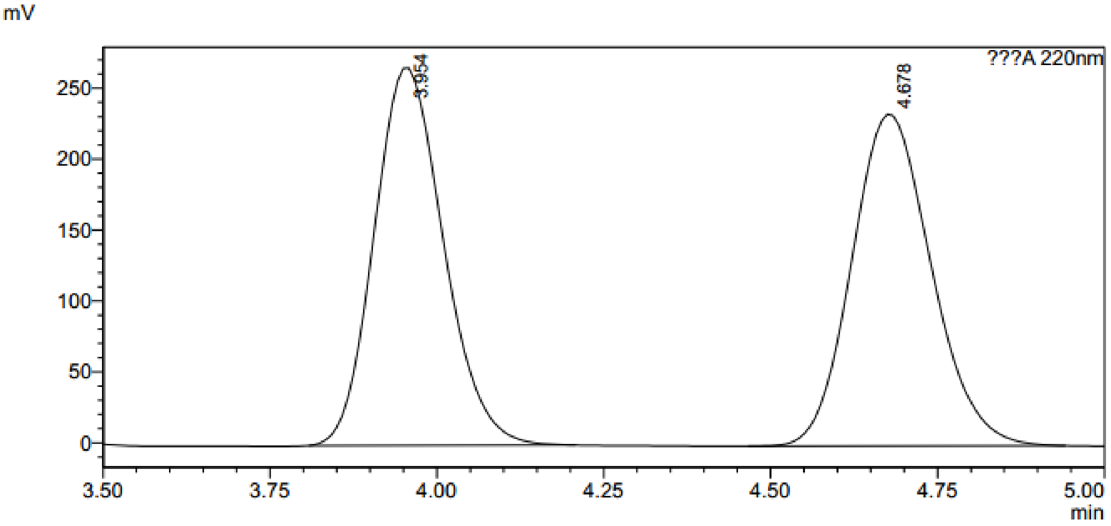

<Peak Table>

???A 220nm

Peak#	Ret. Time	Area	Height	Conc.	Unit	Mark	Name
1	3.954	1927640	265781	49.831		M	
2	4.678	1940747	234045	50.169		M	
Total		3868387	499827				

<Chromatogram>

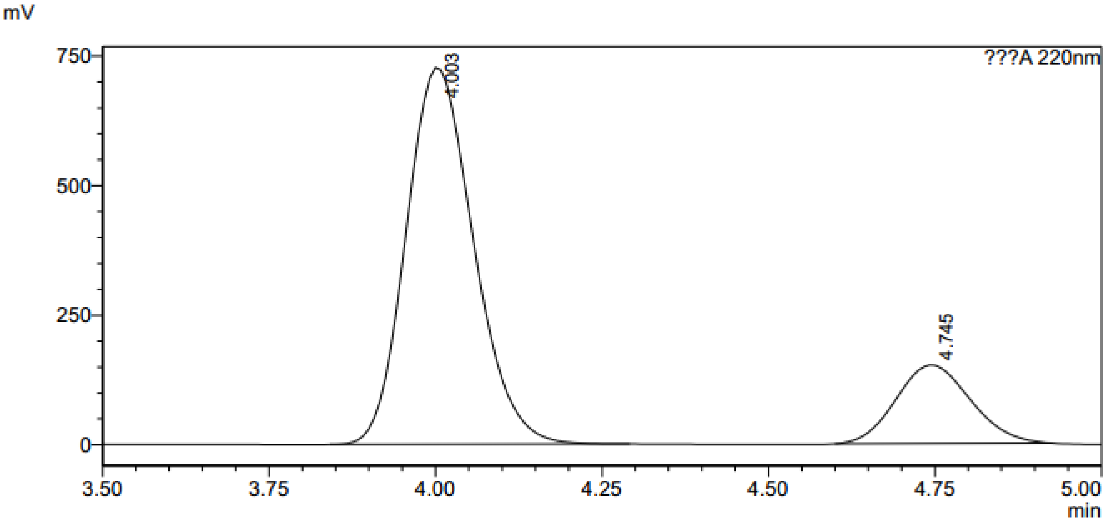

<Peak Table>

???A 220nm

Peak#	Ret. Time	Area	Height	Conc.	Unit	Mark	Name
1	4.003	5122107	726521	81.232		M	
2	4.745	1183449	151491	18.768		M	
Total		6305556	878012				

(1*R*,2*R*)-2-Hydroxy-2-*iso*-propyl-3,3-dimethyl-1-phenylbutyl propionate (**2x**)

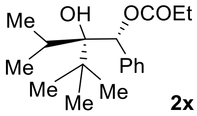

2x

<Chromatogram>

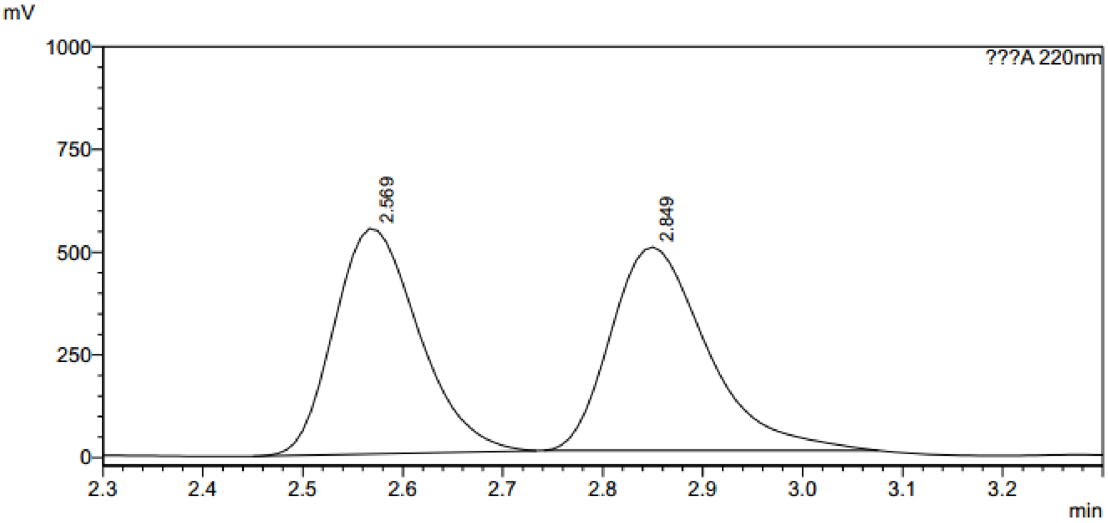

<Peak Table>

???A 220nm

Peak#	Ret. Time	Area	Height	Conc.	Unit	Mark	Name
1	2.569	3245044	548590	49.343		M	
2	2.849	3331398	495605	50.657		M	
Total		6576442	1044195				

<Chromatogram>

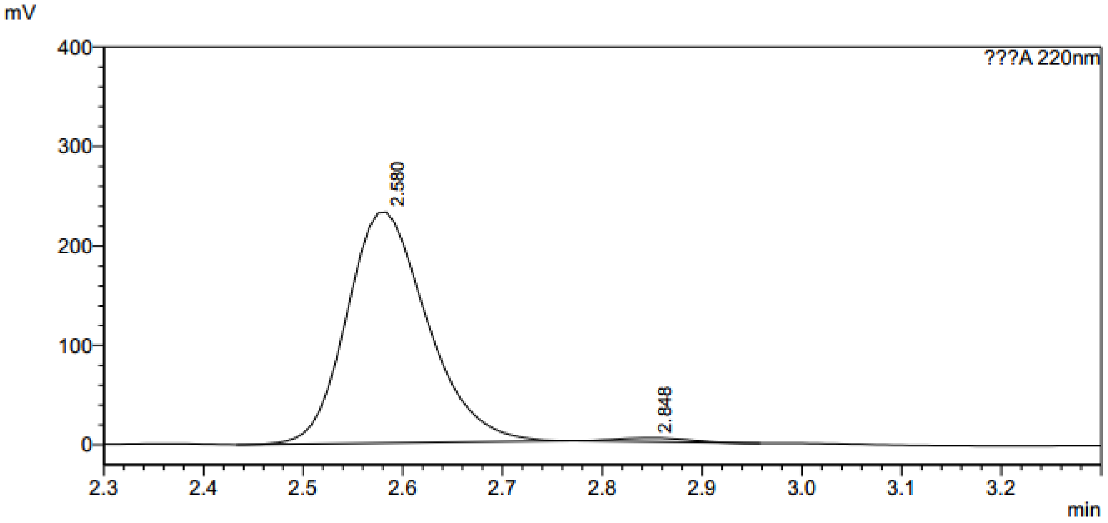

<Peak Table>

???A 220nm

Peak#	Ret. Time	Area	Height	Conc.	Unit	Mark	Name
1	2.580	1309489	232391	98.605		M	
2	2.848	18528	3922	1.395		M	
Total		1328016	236313				

(1*R*,2*R*)-2-Hydroxy-2-*iso*-propyl-3,3-dimethyl-1-phenylbutyl propionate (**2x**)

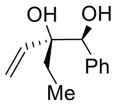

1y

<Chromatogram>

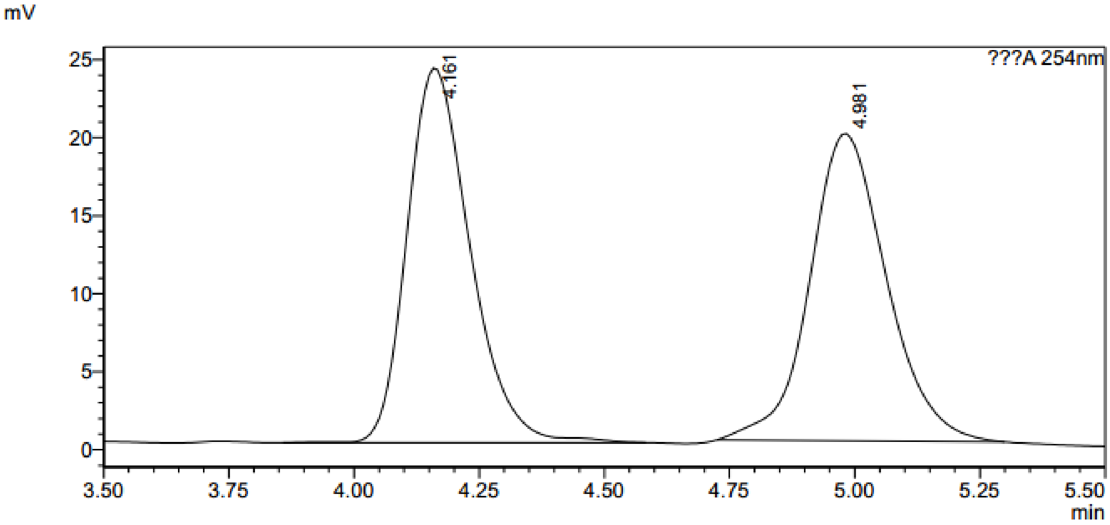

<Peak Table>

???A 254nm

Peak#	Ret. Time	Area	Height	Conc.	Unit	Mark	Name
1	4.161	210577	24001	49.980		M	
2	4.981	210744	19671	50.020		M	
Total		421321	43672				

<Chromatogram>

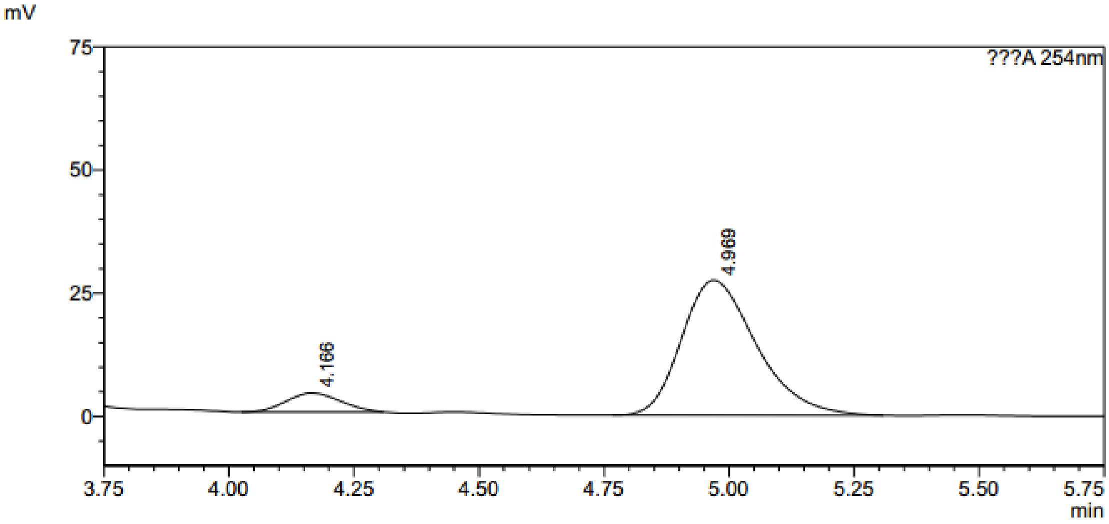

<Peak Table>

???A 254nm

Peak#	Ret. Time	Area	Height	Conc.	Unit	Mark	Name
1	4.166	29057	3823	9.218		M	
2	4.969	286170	27398	90.782		M	
Total		315227	31221				

(1*R*,2*R*)-2-Ethyl-2-hydroxy-1-phenylbut-3-en-1-yl propionate (**2y**)

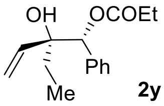

2y

<Chromatogram>

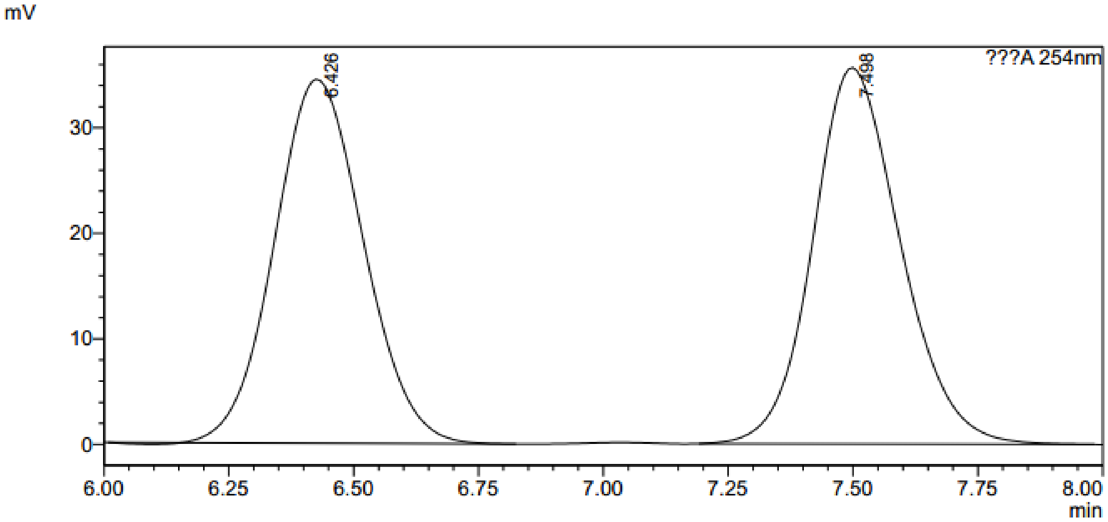

<Peak Table>

???A 254nm

Peak#	Ret. Time	Area	Height	Conc.	Unit	Mark	Name
1	6.426	429277	34489	49.568		M	
2	7.498	436768	35658	50.432		M	
Total		866045	70147				

<Chromatogram>

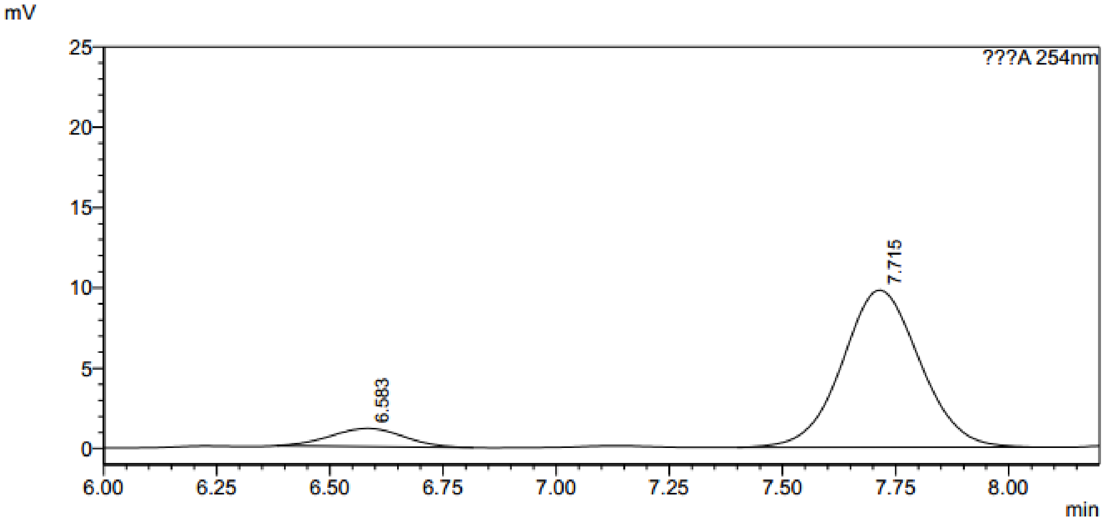

<Peak Table>

???A 254nm

Peak#	Ret. Time	Area	Height	Conc.	Unit	Mark	Name
1	6.583	12639	1131	9.740		M	
2	7.715	117127	9784	90.260		M	
Total		129765	10915				

(1*S*,2*S*)-1-Phenyl-2-(phenylethynyl)hexane-1,2-diol (**1z**)

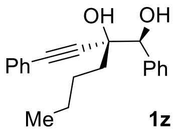

1z

<Chromatogram>

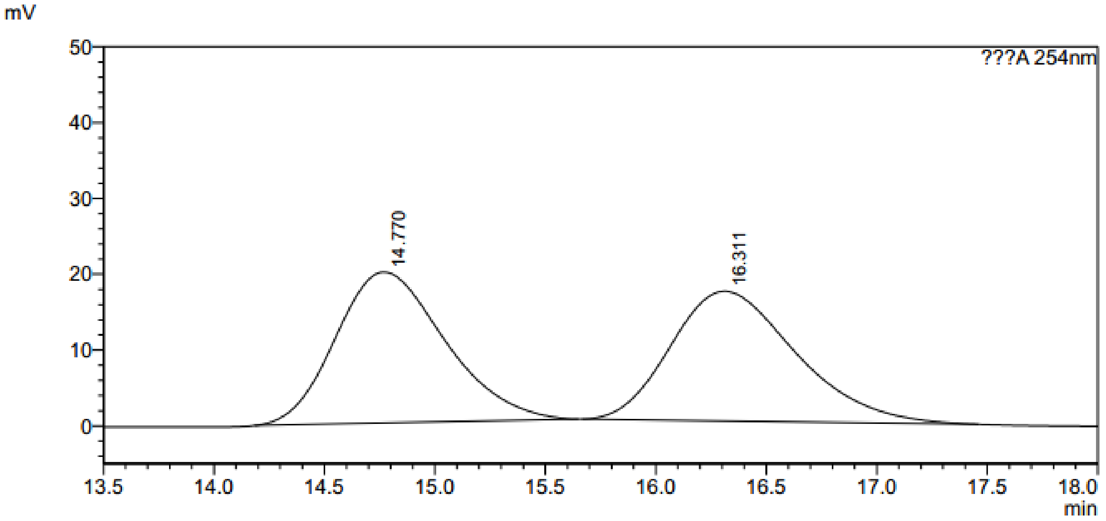

<Peak Table>

???A 254nm

Peak#	Ret. Time	Area	Height	Conc.	Unit	Mark	Name
1	14.770	686517	19898	50.413		M	
2	16.311	675259	17112	49.587		M	
Total		1361777	37010				

<Chromatogram>

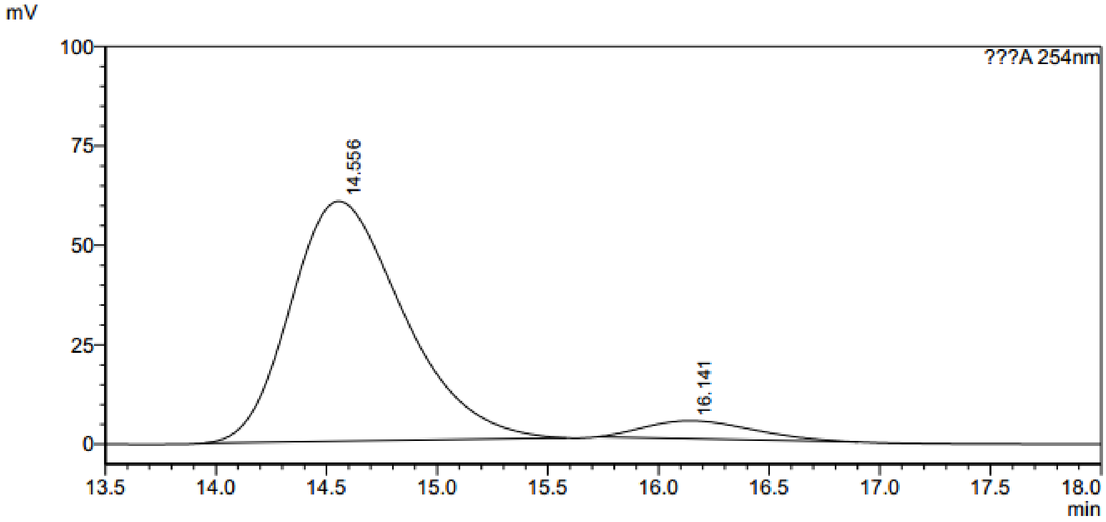

<Peak Table>

???A 254nm

Peak#	Ret. Time	Area	Height	Conc.	Unit	Mark	Name
1	14.556	2143616	60342	93.360		M	
2	16.141	152465	4502	6.640		M	
Total		2296081	64844				

(1*R*,2*R*)-2-Hydroxy-1-phenyl-2-(phenylethynyl)hexyl propionate (**2z**)

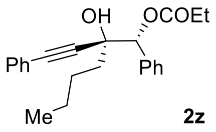

2z

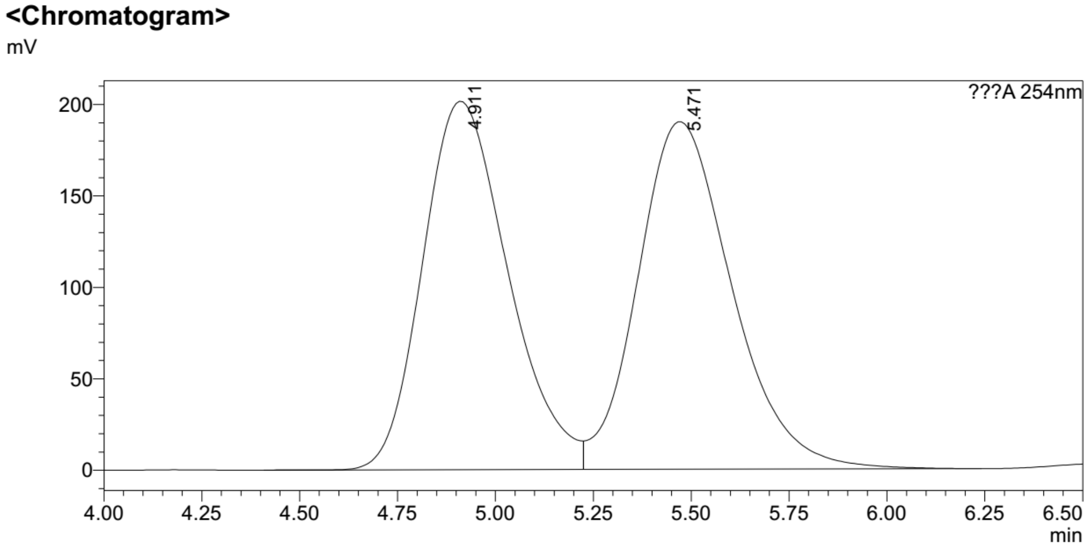

<Peak Table>

???A 254nm

Peak#	Ret. Time	Area	Height	Conc.	Unit	Mark	Name
1	4.911	3064274	201368	49.116			
2	5.471	3174593	189975	50.884		V	
Total		6238867	391342				

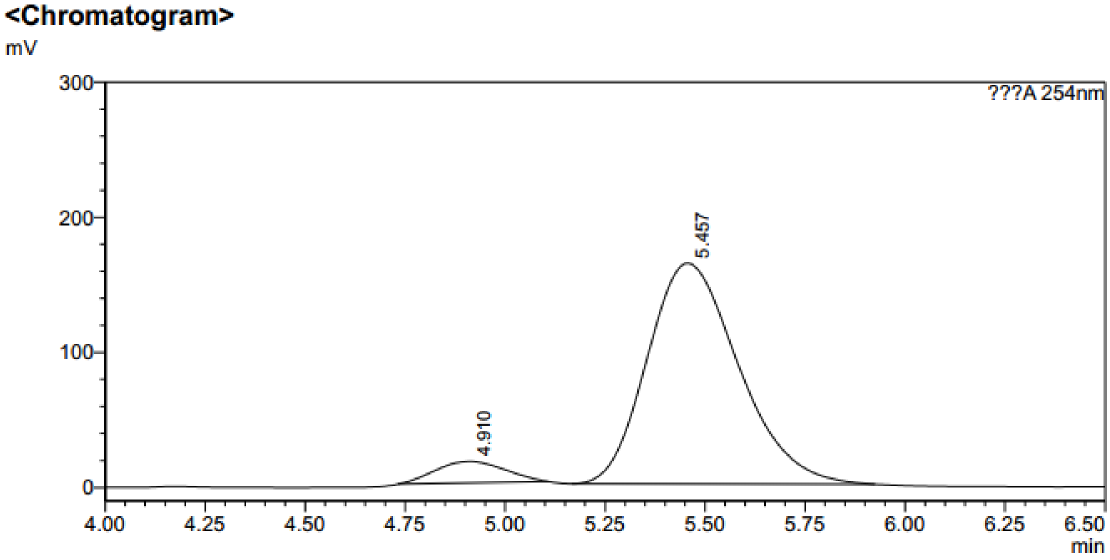

<Peak Table>

???A 254nm

Peak#	Ret. Time	Area	Height	Conc.	Unit	Mark	Name
1	4.910	193563	15946	6.931		M	
2	5.457	2599234	163378	93.069		M	
Total		2792797	179324				

(1*R*,2*S*)-2-(4-Chlorophenyl)-1-cyclohexyl-1-phenylethane-1,2-diol (**1za**)

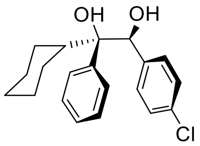

1za

<Chromatogram>

mV

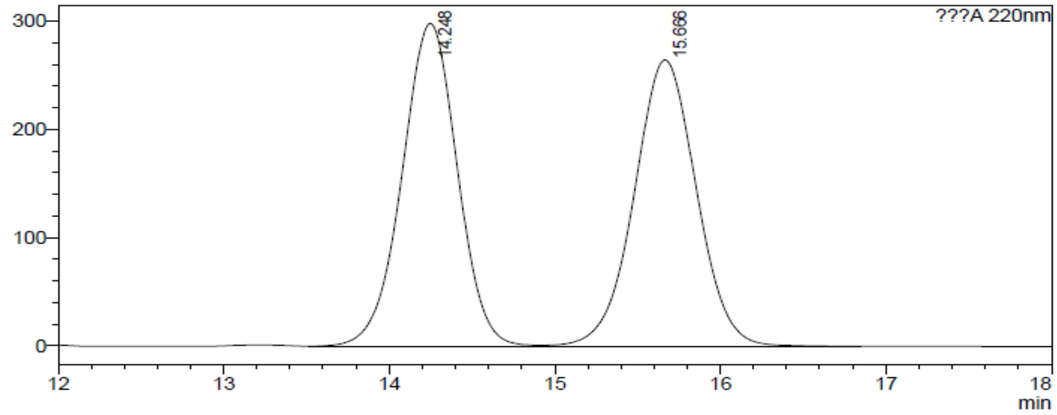

<Peak Table>

???A 220nm

Peak#	Ret. Time	Area	Height	Conc.	Unit	Mark	Name
1	14.248	6930721	298418	50.061			
2	15.666	6913958	264710	49.939		V	
Total		13844679	563128				

<Chromatogram>

mV

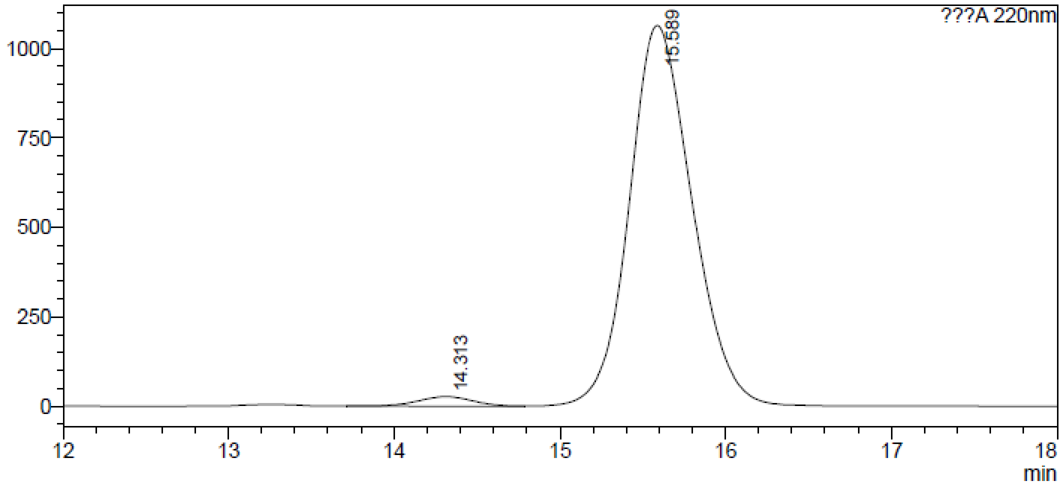

<Peak Table>

???A 220nm

Peak#	Ret. Time	Area	Height	Conc.	Unit	Mark	Name
1	14.313	627580	27127	2.170			
2	15.589	28286539	1063828	97.830		V	
Total		28914119	1090954				

(1*R*,2*S*)-1-(4-Chlorophenyl)-2-cyclohexyl-2-hydroxy-2-phenylethyl propionate (**2za**)

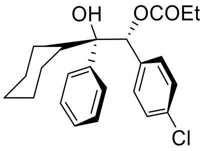

2za

<Chromatogram>

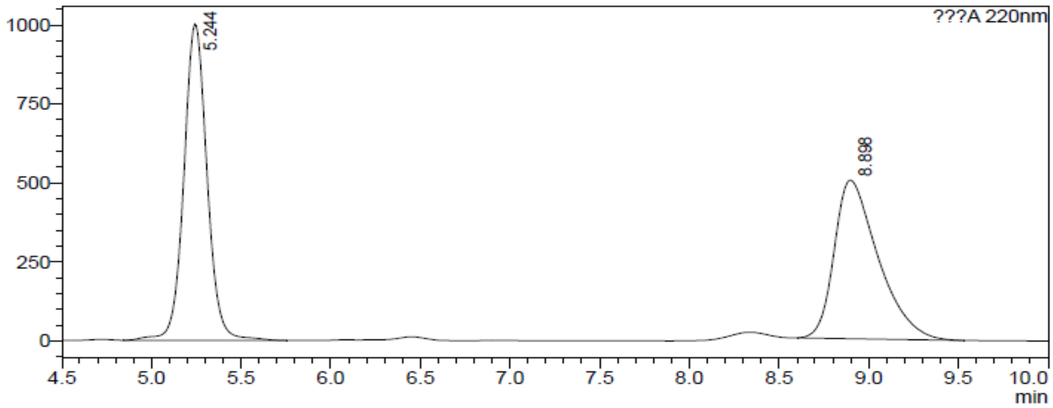

<Peak Table>

???A 220nm

Peak#	Ret. Time	Area	Height	Conc.	Unit	Mark	Name
1	5.244	9043879	1000679	51.375			
2	8.898	8559857	501731	48.625		M	
Total		17603736	1502410				

<Chromatogram>

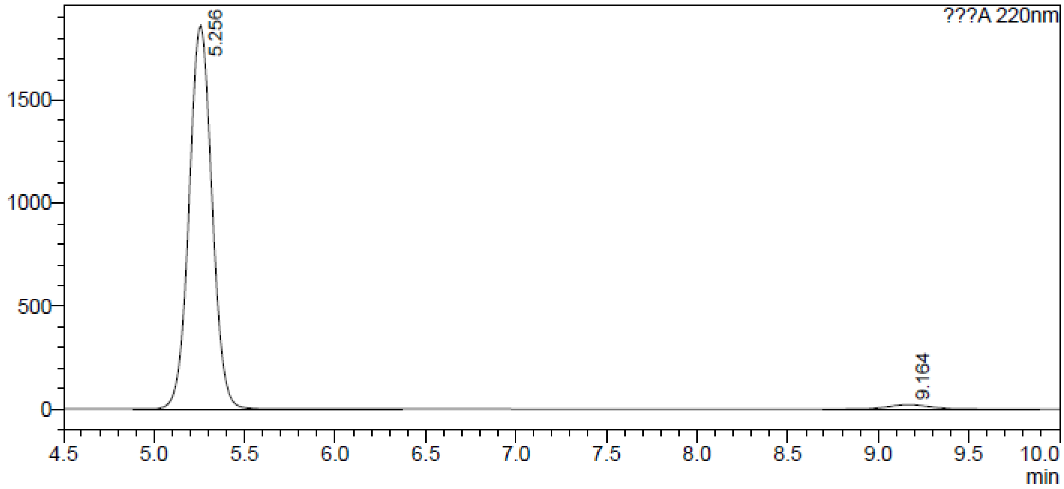

<Peak Table>

???A 220nm

Peak#	Ret. Time	Area	Height	Conc.	Unit	Mark	Name
1	5.256	16436914	1859601	97.868		S	
2	9.164	358037	21918	2.132			
Total		16794951	1881519				

(*R*)-(2-((*R*)-Hydroxy(phenyl)methyl)phenyl)(phenyl)(o-tolyl)methanol (**1aa**)

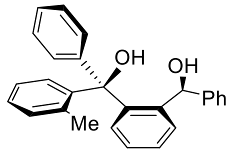

1aa

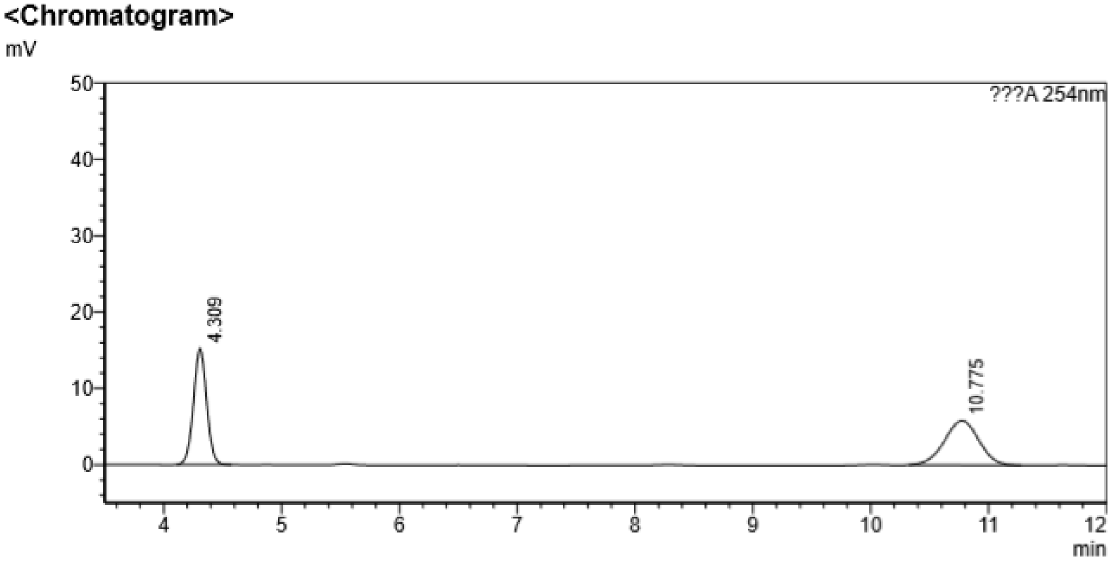

<Peak Table>

???A 254nm

Peak#	Ret. Time	Area	Height	Conc.	Unit	Mark	Name
1	4.309	118581	15185	50.259		M	
2	10.775	117359	5801	49.741		M	
Total		235940	20986				

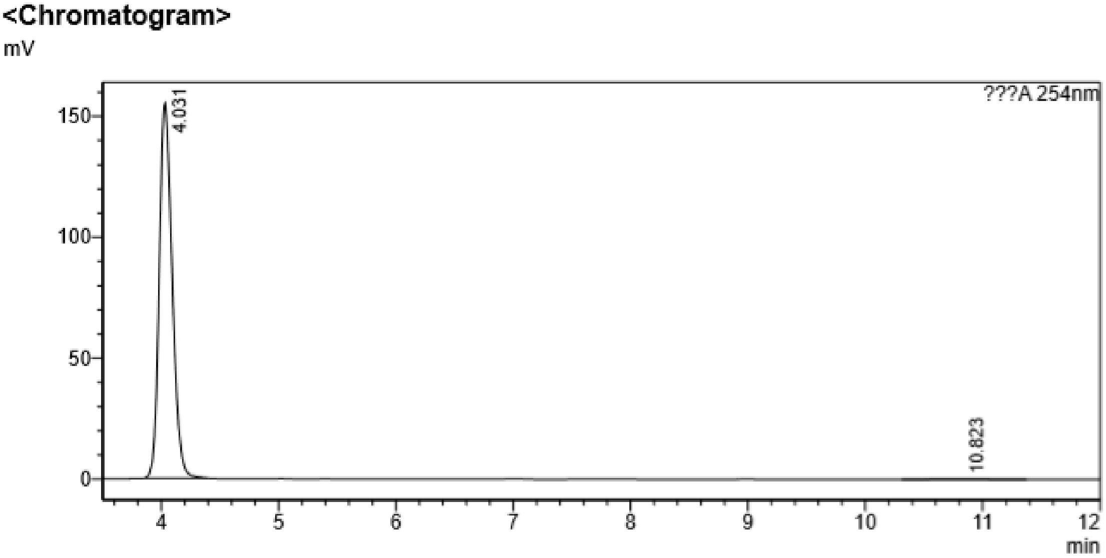

<Peak Table>

???A 254nm

Peak#	Ret. Time	Area	Height	Conc.	Unit	Mark	Name
1	4.031	1184706	154950	99.912		M	
2	10.823	1041	47	0.088		M	
Total		1185747	154997				

(*S*)-(2-((*S*)-Hydroxy(phenyl)(o-tolyl)methyl)phenyl)(phenyl)methyl propionate (**2aa**)

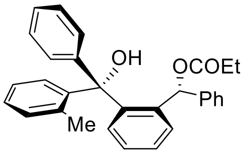

2aa

<Chromatogram>

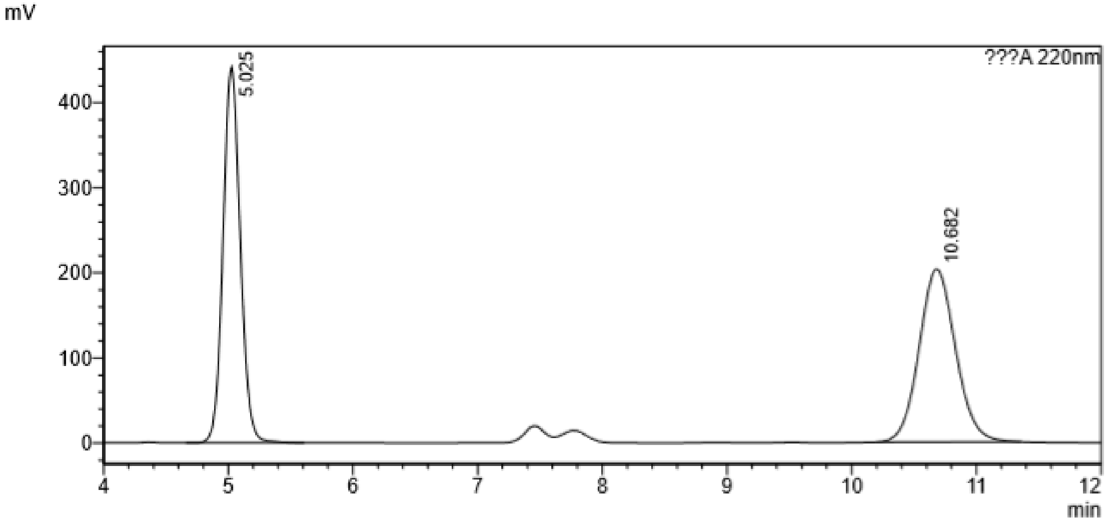

<Peak Table>

???A 220nm

Peak#	Ret. Time	Area	Height	Conc.	Unit	Mark	Name
1	5.025	4241666	441635	50.627			
2	10.682	4136629	203286	49.373		M	
Total		8378295	644921				

<Chromatogram>

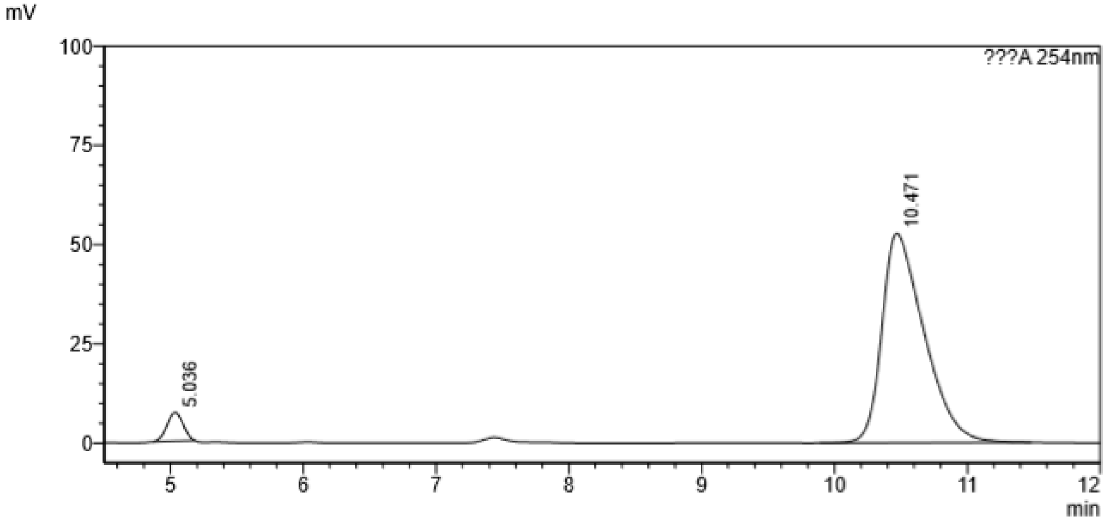

<Peak Table>

???A 254nm

Peak#	Ret. Time	Area	Height	Conc.	Unit	Mark	Name
1	5.036	61319	7197	4.997		M	
2	10.471	1165796	52700	95.003		M	
Total		1227115	59897				

(*R*)-(2-((*R*)-Hydroxy(phenyl)methyl)phenyl)(*o*-tolyl)(*p*-tolyl)methanol (**1ab**)

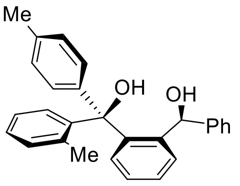

1ab

<Chromatogram>

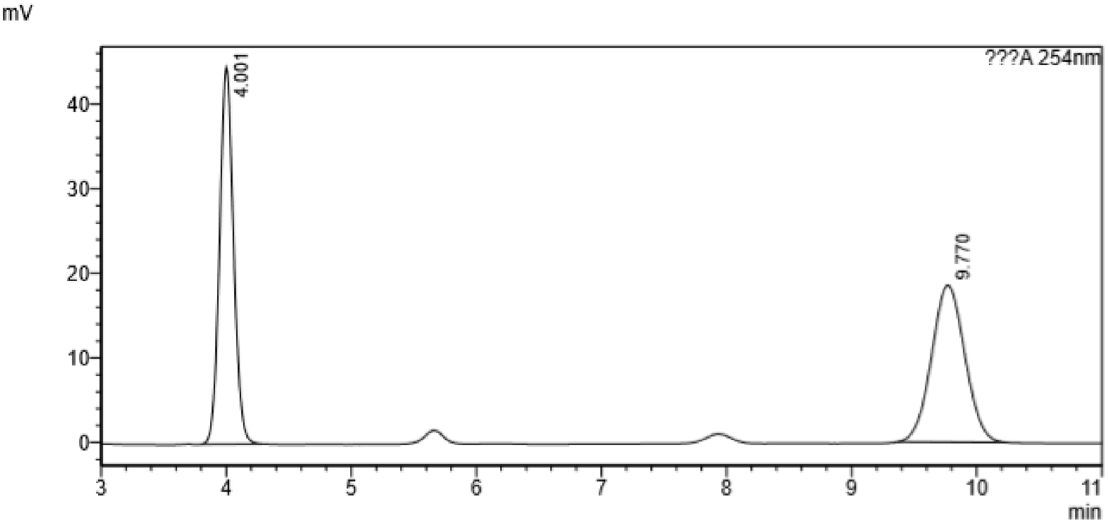

<Peak Table>

???A 254nm

Peak#	Ret. Time	Area	Height	Conc.	Unit	Mark	Name
1	4.001	346334	44507	50.142		M	
2	9.770	344377	18559	49.858		M	
Total		690711	63066				

<Chromatogram>

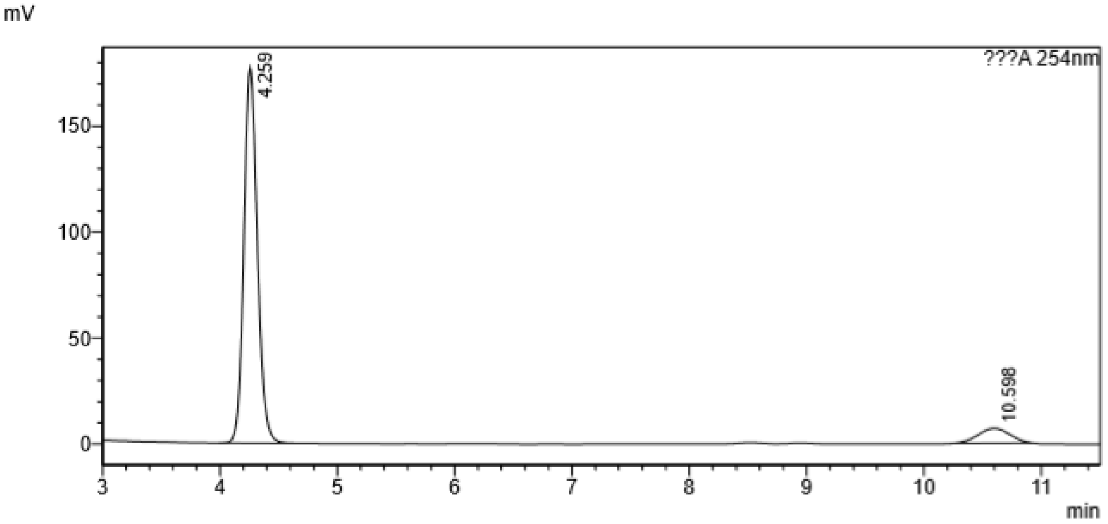

<Peak Table>

???A 254nm

Peak#	Ret. Time	Area	Height	Conc.	Unit	Mark	Name
1	4.259	1414967	176982	91.509		M	
2	10.598	131286	7030	8.491		M	
Total		1546253	184012				

(*S*)-(2-((*S*)-Hydroxy(*o*-tolyl)(*p*-tolyl)methyl)phenyl)(phenyl)methyl propionate (**2ab**)

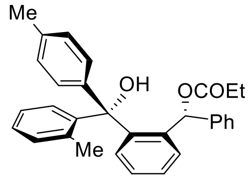

2ab

<Chromatogram>

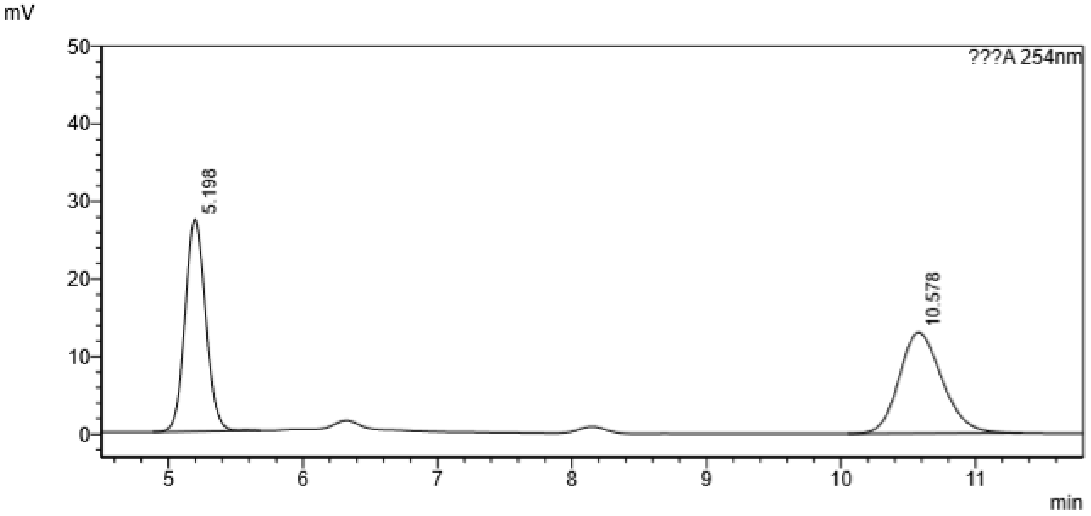

<Peak Table>

???A 254nm

Peak#	Ret. Time	Area	Height	Conc.	Unit	Mark	Name
1	5.198	291855	27340	50.160		M	
2	10.578	289988	13022	49.840		M	
Total		581843	40363				

<Chromatogram>

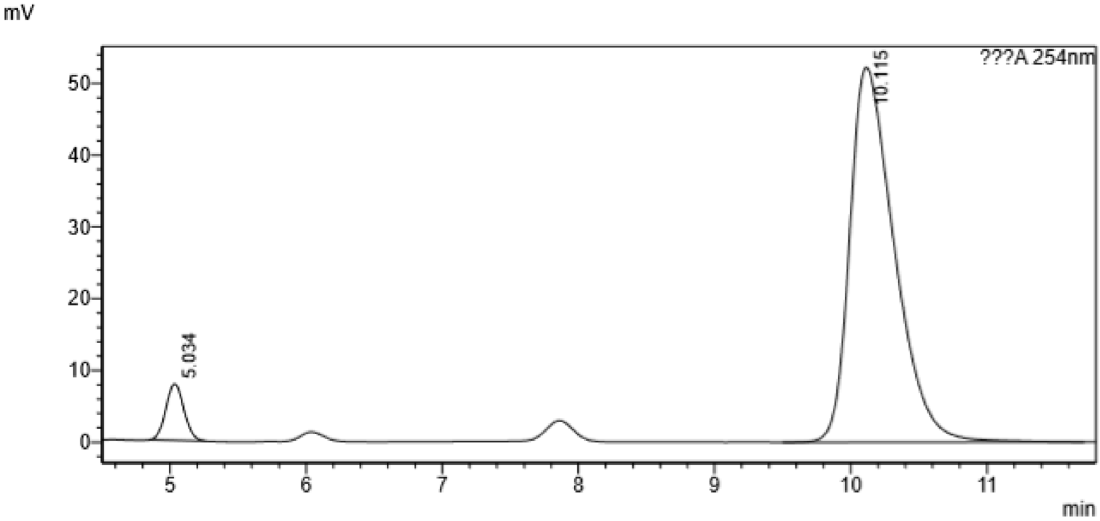

<Peak Table>

???A 254nm

Peak#	Ret. Time	Area	Height	Conc.	Unit	Mark	Name
1	5.034	72826	7878	5.778		M	
2	10.115	1187473	52241	94.222		M	
Total		1260299	60119				

(*R*)-(4-Chlorophenyl)(2-((*R*)-hydroxy(phenyl)methyl)phenyl)(*o*-tolyl)methanol (**1ac**)

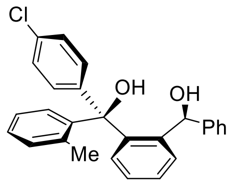

1ac

<Chromatogram>

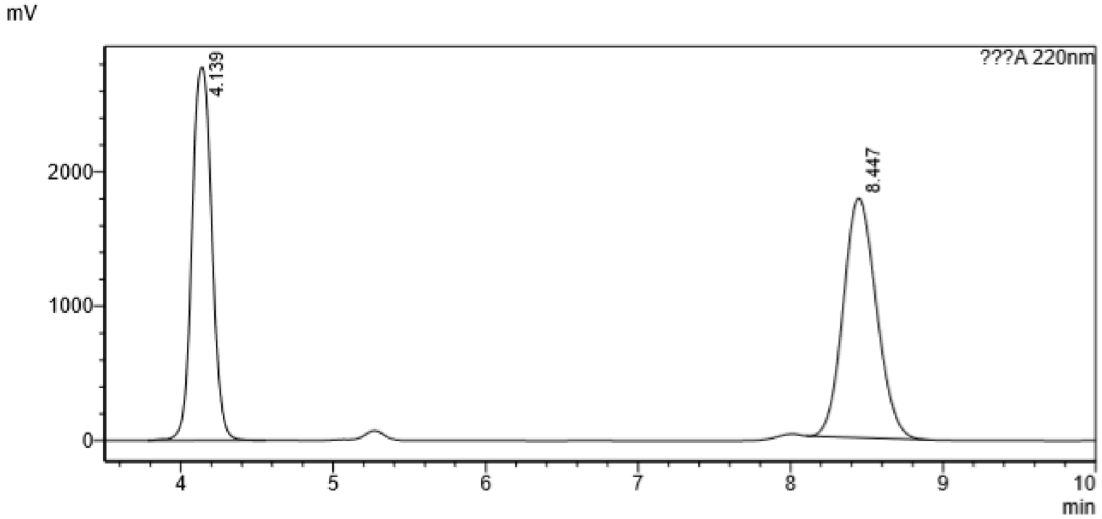

<Peak Table>

???A 220nm

Peak#	Ret. Time	Area	Height	Conc.	Unit	Mark	Name
1	4.139	25108370	2776216	48.167		M	
2	8.447	27019463	1781533	51.833		M	
Total		52127833	4557749				

<Chromatogram>

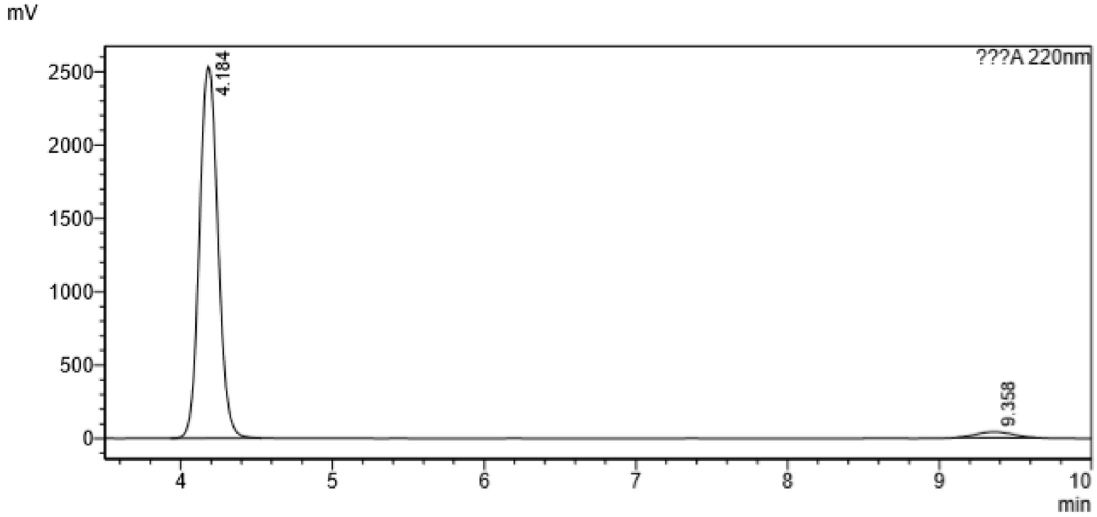

<Peak Table>

???A 220nm

Peak#	Ret. Time	Area	Height	Conc.	Unit	Mark	Name
1	4.184	21423955	2530883	97.077		M	
2	9.358	645047	39710	2.923		M	
Total		22069002	2570593				

<u>(*S*)-(2-((*S*)-(4-Chlorophenyl)(hydroxy)(*o*-tolyl)methyl)phenyl)(phenyl)methyl propionate (**2ac**)</u>

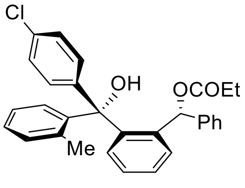

2ac

<Chromatogram>

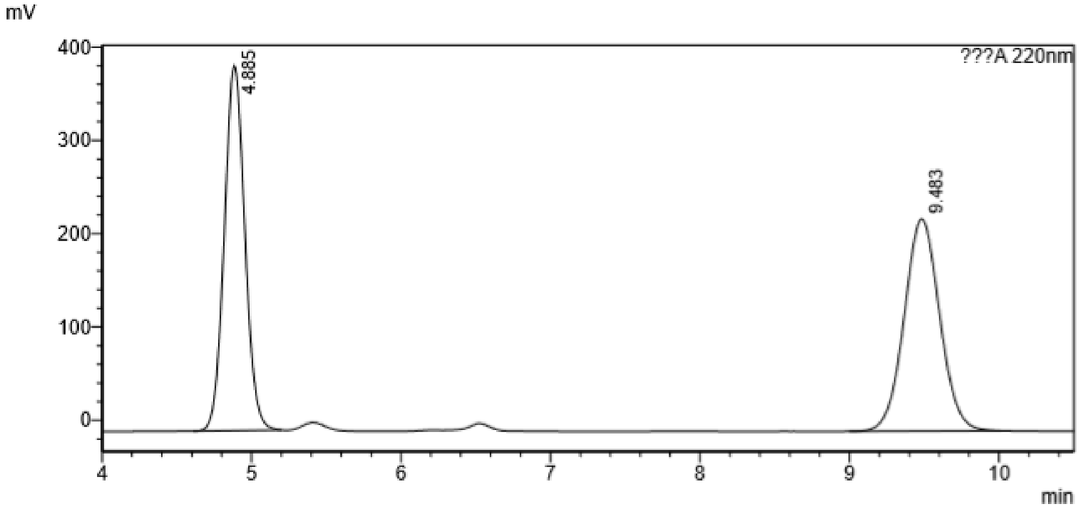

<Peak Table>

???A 220nm

Peak#	Ret. Time	Area	Height	Conc.	Unit	Mark	Name
1	4.885	3740580	391085	49.887		M	
2	9.483	3757498	227332	50.113		M	
Total		7498078	618417				

<Chromatogram>

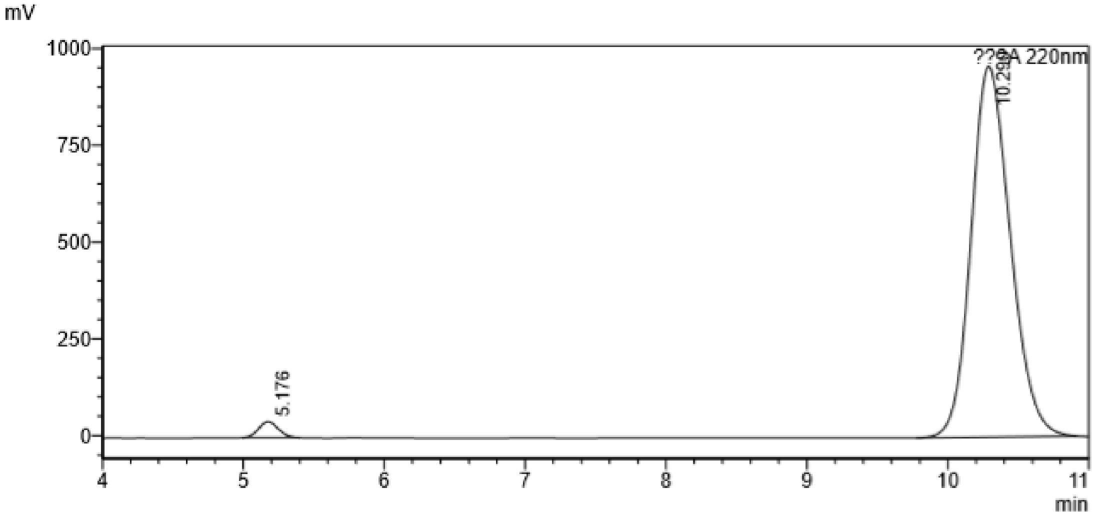

<Peak Table>

???A 220nm

Peak#	Ret. Time	Area	Height	Conc.	Unit	Mark	Name
1	5.176	406069	41345	2.144		M	
2	10.290	18537268	956626	97.856		M	
Total		18943338	997971				

(*R*)-(4-Fluoro-2-methylphenyl)(2-((*R*)hydroxy(phenyl)methyl)phenyl)(phenyl)methanol (**1ad**)

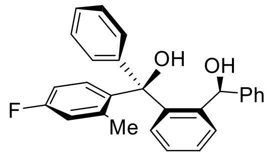

1ad

<Chromatogram>

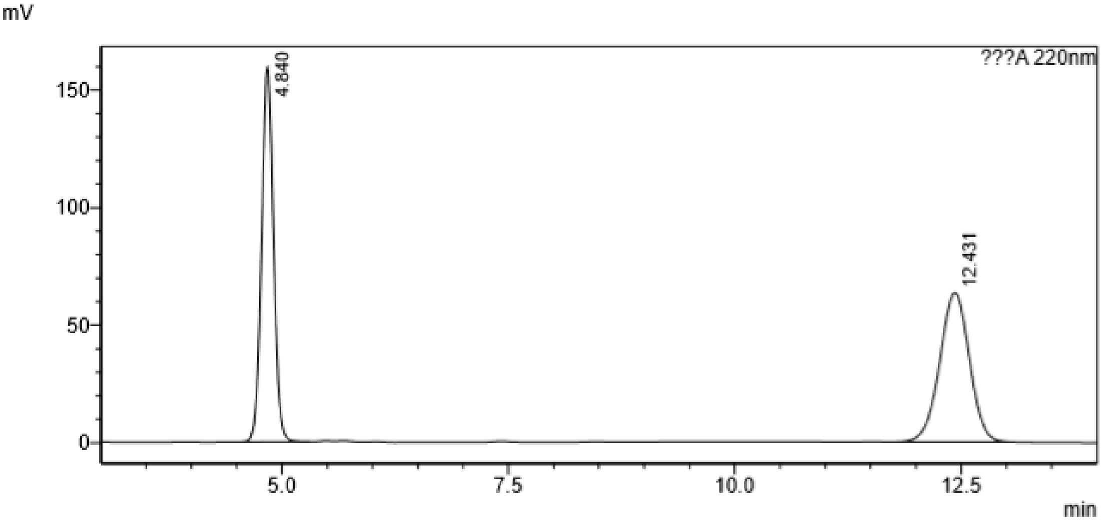

<Peak Table>

???A 220nm

Peak#	Ret. Time	Area	Height	Conc.	Unit	Mark	Name
1	4.840	1460064	159237	50.244			
2	12.431	1445858	63357	49.756		M	
Total		2905922	222594				

<Chromatogram>

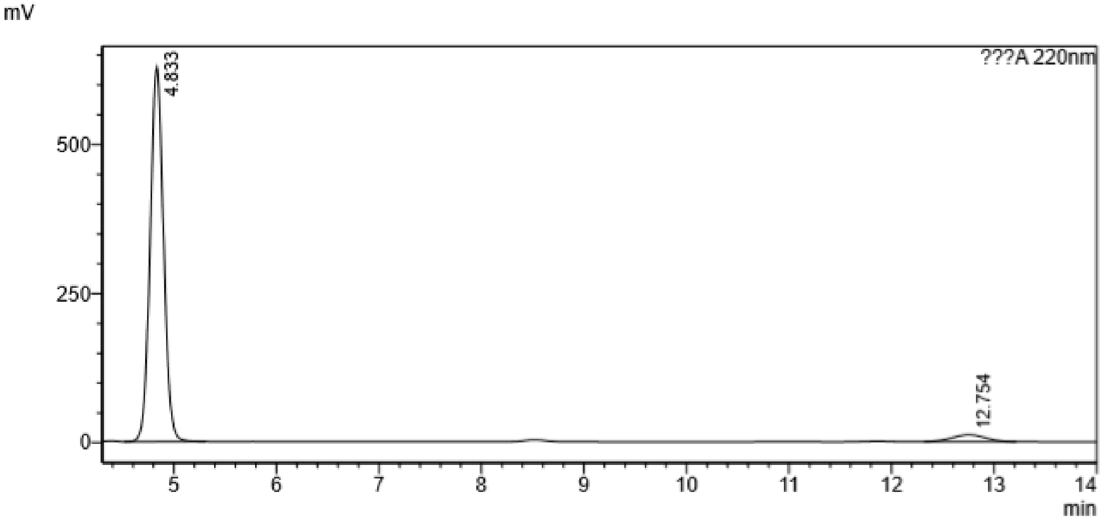

<Peak Table>

???A 220nm

Peak#	Ret. Time	Area	Height	Conc.	Unit	Mark	Name
1	4.833	5707411	628136	95.472		M	
2	12.754	270658	11796	4.528		M	
Total		5978069	639933				

(*S*)-(2-((*S*)-(4-Fluoro-2-methylphenyl)(hydroxy)(phenyl)methyl)phenyl)(phenyl)methyl propionate (**2ad**)

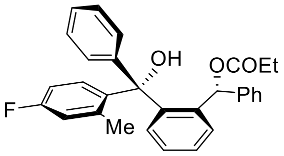

2ad

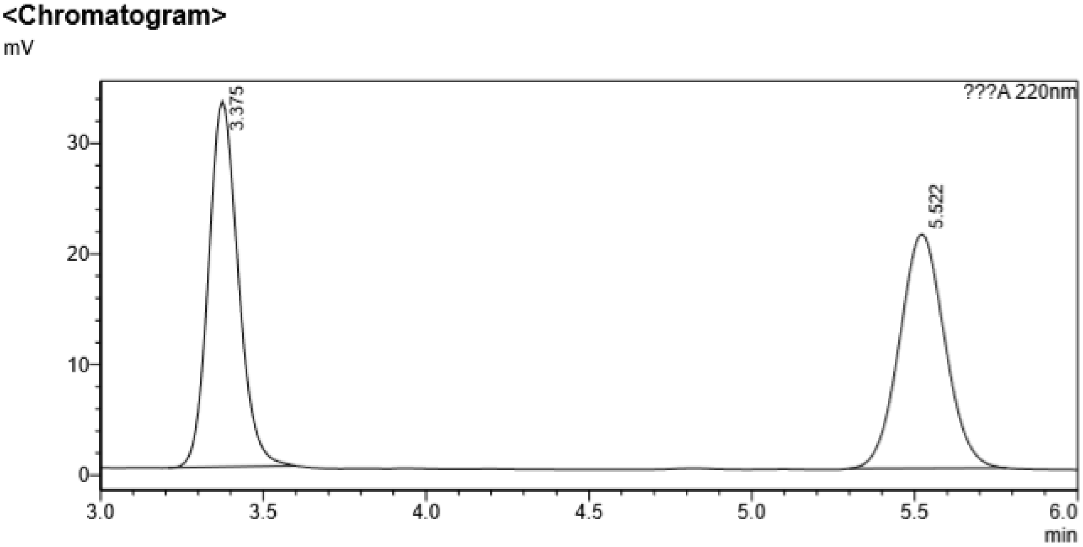

<Peak Table>

???A 220nm

Peak#	Ret. Time	Area	Height	Conc.	Unit	Mark	Name
1	3.375	210070	33036	50.587		M	
2	5.522	205198	21157	49.413		M	
Total		415268	54193				

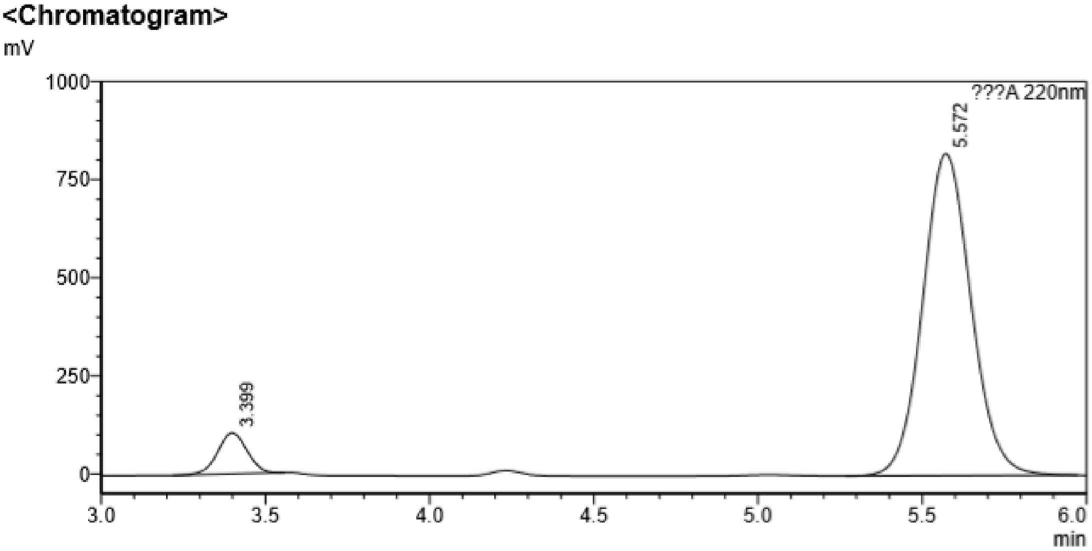

<Peak Table>

???A 220nm

Peak#	Ret. Time	Area	Height	Conc.	Unit	Mark	Name
1	3.399	652162	104617	7.193		M	
2	5.572	8414796	820975	92.807		M	
Total		9066958	925592				

(*S*)-phenyl((2*S*,3*R*)-3-phenyl-3-(*p*-tolyl)oxiran-2-yl)methanol (**3a**)

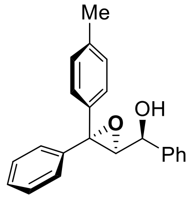

3a

<Chromatogram>

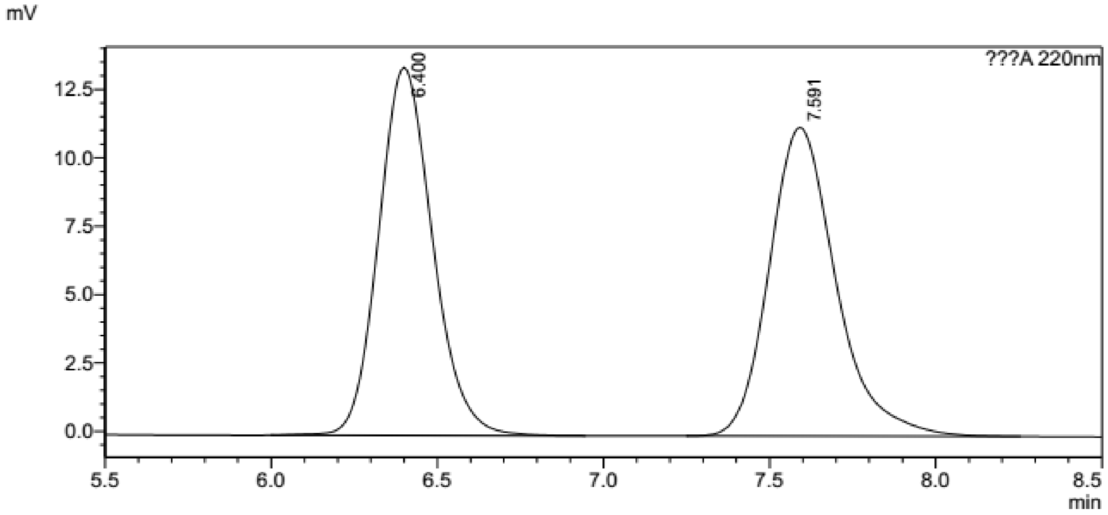

<Peak Table>

???A 220nm

Peak#	Ret. Time	Area	Height	Conc.	Unit	Mark	Name
1	6.400	152537	13446	49.158			
2	7.591	157759	11289	50.842			
Total		310296	24736				

<Chromatogram>

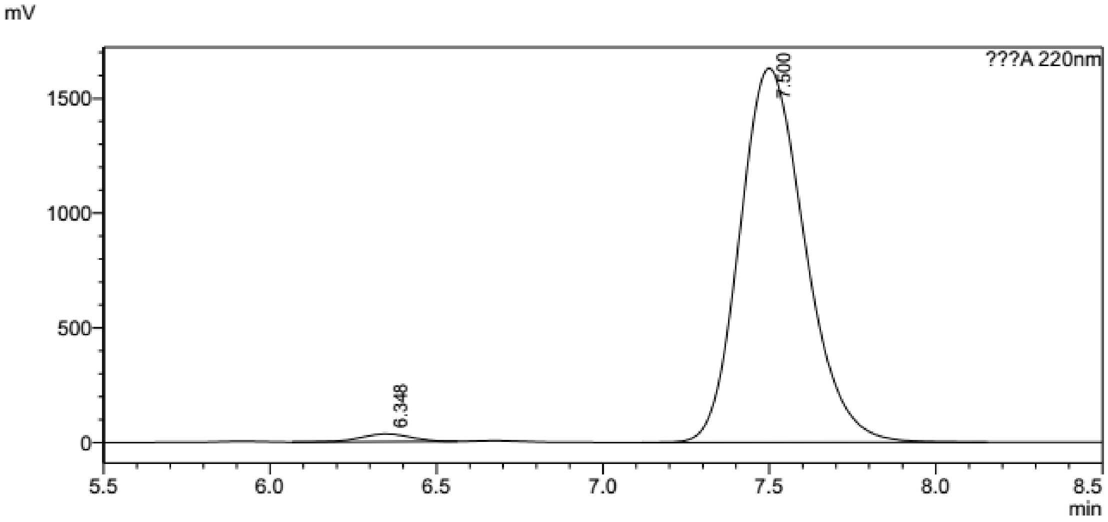

<Peak Table>

???A 220nm

Peak#	Ret. Time	Area	Height	Conc.	Unit	Mark	Name
1	6.348	364048	34641	1.610			
2	7.500	22246953	1631306	98.390			
Total		22611002	1665947				

(*R*)-phenyl((2*R*,3*S*)-3-phenyl-3-(*p*-tolyl)oxiran-2-yl)methyl propionate (**4a**)

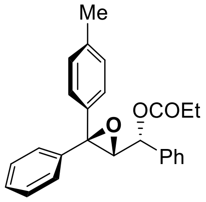

4a

<Chromatogram>

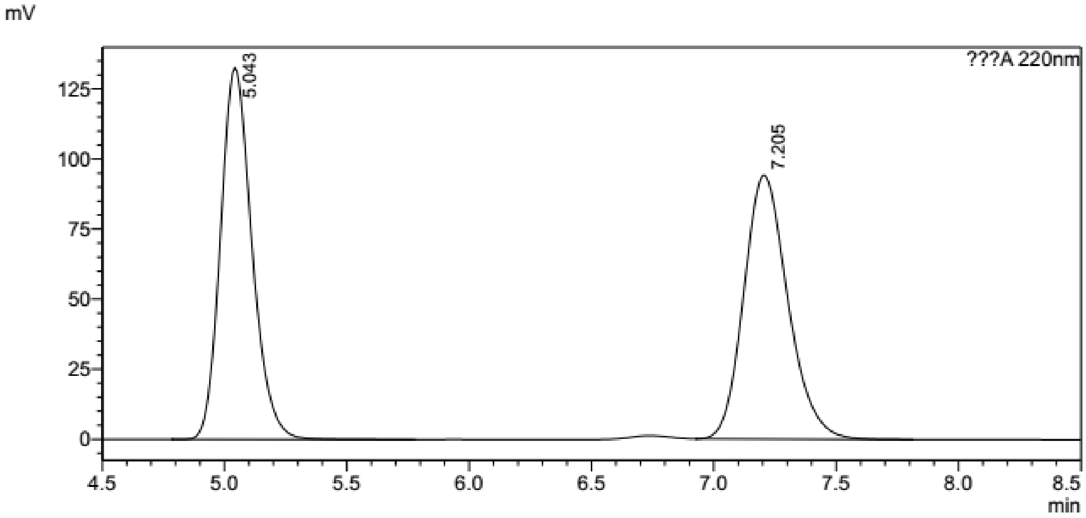

<Peak Table>

???A 220nm

Peak#	Ret. Time	Area	Height	Conc.	Unit	Mark	Name
1	5.043	1187632	132559	50.112			
2	7.205	1182339	94157	49.888			
Total		2369971	226716				

<Chromatogram>

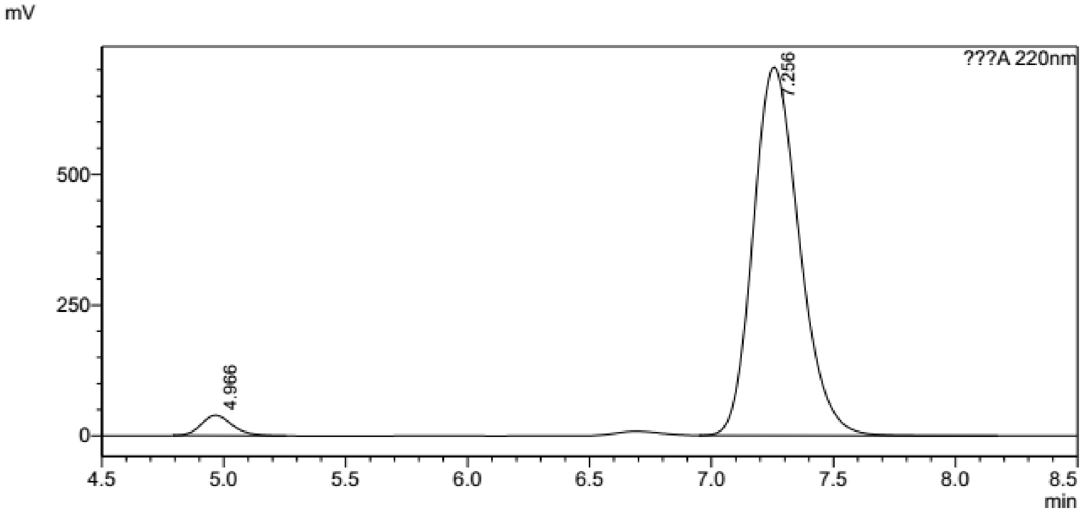

<Peak Table>

???A 220nm

Peak#	Ret. Time	Area	Height	Conc.	Unit	Mark	Name
1	4.966	346227	39015	3.601		M	
2	7.256	9267858	704444	96.399			
Total		9614085	743459				

(*S*)-(4-methoxyphenyl)((2*S*,3*R*)-3-phenyl-3-(*p*-tolyl)oxiran-2-yl)methanol (**3b**)

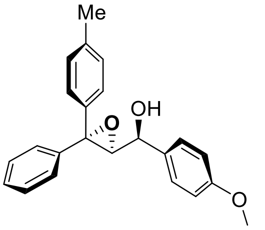

3b

<Chromatogram>

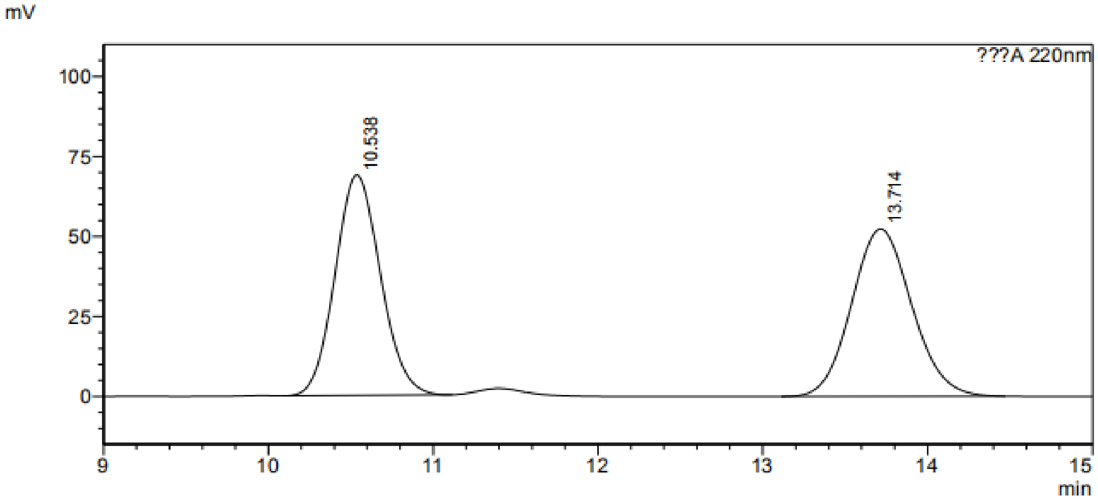

<Peak Table>

???A 220nm

Peak#	Ret. Time	Area	Height	Conc.	Unit	Mark	Name
1	10.538	1321789	68881	49.846		M	
2	13.714	1329932	52313	50.154		M	
Total		2651721	121194				

<Chromatogram>

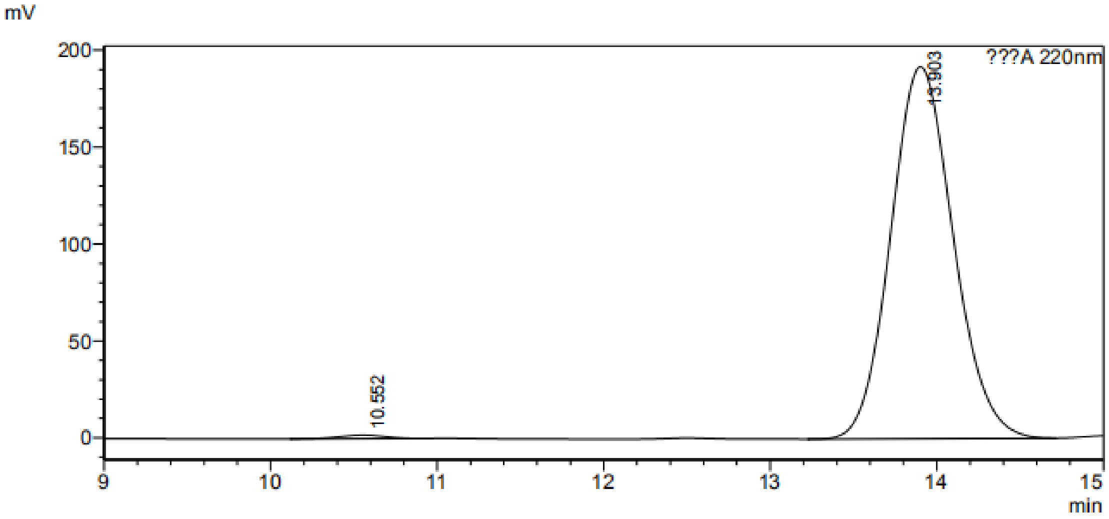

<Peak Table>

???A 220nm

Peak#	Ret. Time	Area	Height	Conc.	Unit	Mark	Name
1	10.552	35356	1894	0.700			
2	13.903	5014380	191941	99.300			
Total		5049736	193835				

(*R*)-(4-methoxyphenyl)((2*R*,3*S*)-3-phenyl-3-(*p*-tolyl)oxiran-2-yl)methyl propionate (**4b**)

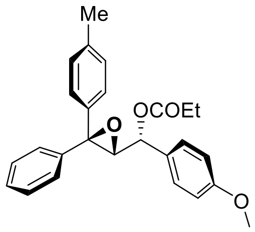

4b

<Chromatogram>

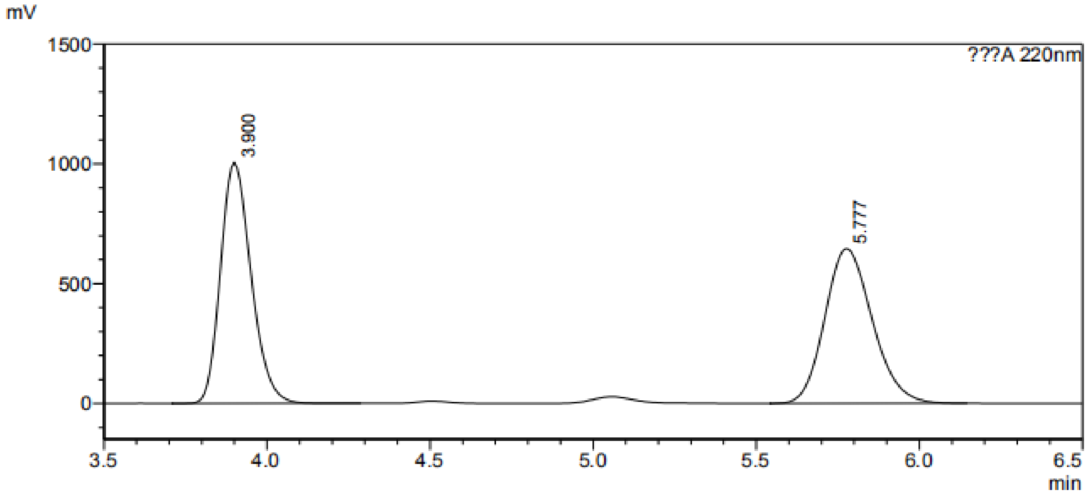

<Peak Table>

???A 220nm

Peak#	Ret. Time	Area	Height	Conc.	Unit	Mark	Name
1	3.900	6555694	1005603	49.998			
2	5.777	6556211	646538	50.002		M	
Total		13111906	1652141				

<Chromatogram>

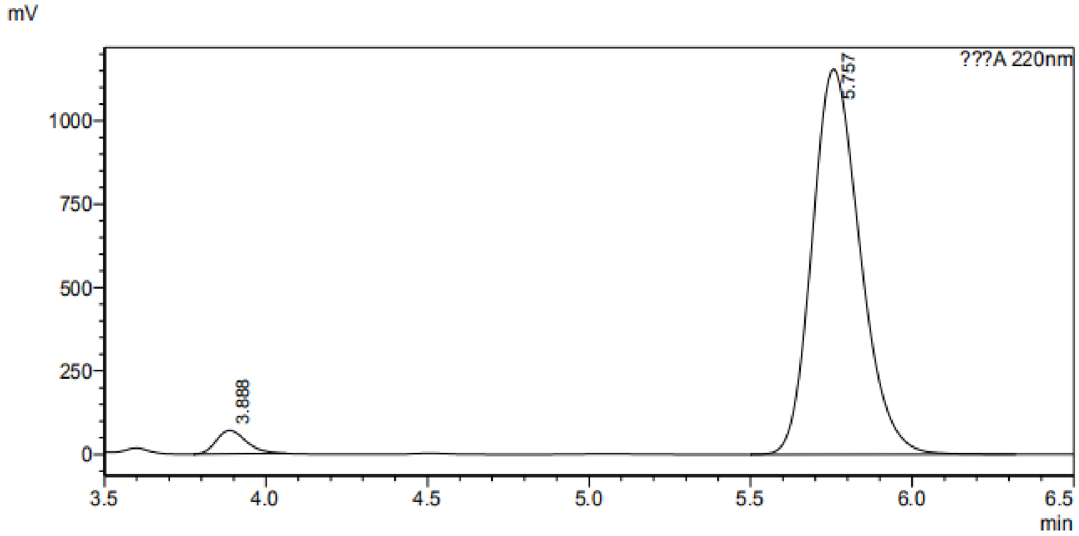

<Peak Table>

???A 220nm

Peak#	Ret. Time	Area	Height	Conc.	Unit	Mark	Name
1	3.888	449877	70014	3.637		M	
2	5.757	11920550	1155143	96.363		M	
Total		12370426	1225156				

(*S*)-(4-fluorophenyl)((2*S*,3*R*)-3-phenyl-3-(*p*-tolyl)oxiran-2-yl)methanol (**3c**)

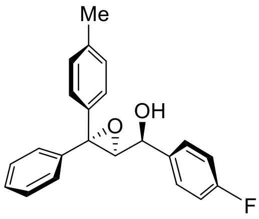

3c

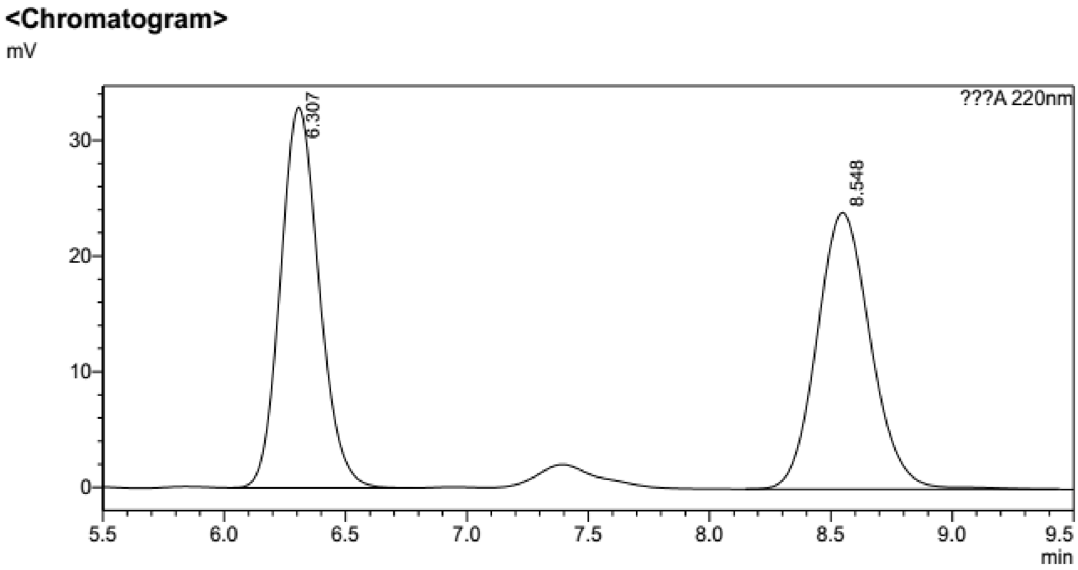

<Peak Table>

???A 220nm

Peak#	Ret. Time	Area	Height	Conc.	Unit	Mark	Name
1	6.307	365437	32889	49.788			
2	8.548	368548	23914	50.212		S	
Total		733986	56803				

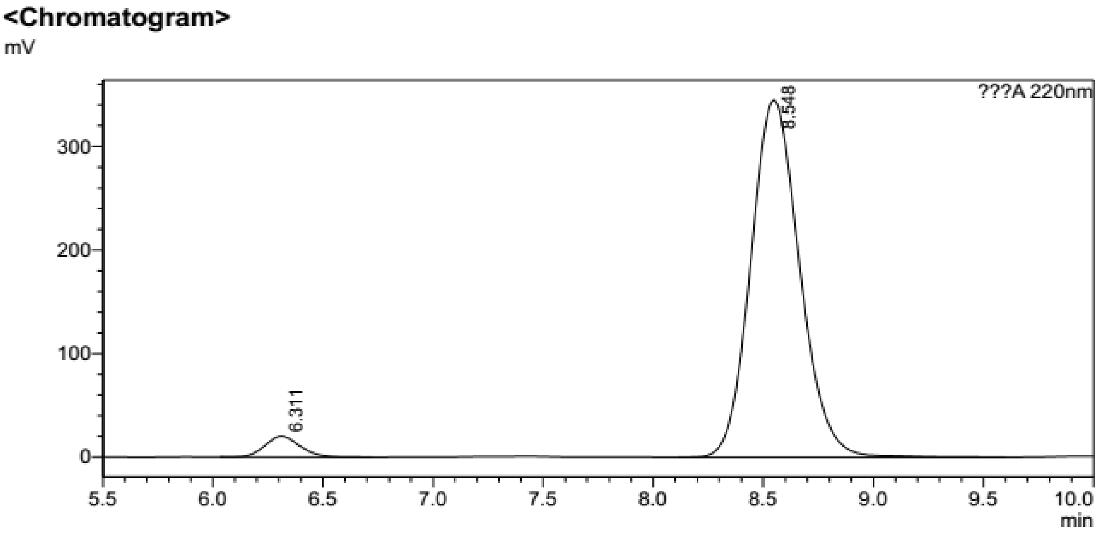

<Peak Table>

???A 220nm

Peak#	Ret. Time	Area	Height	Conc.	Unit	Mark	Name
1	6.311	222284	20006	4.008			
2	8.548	5324381	344821	95.992			
Total		5546665	364827				

(*R*)-(4-fluorophenyl)((2*R*,3*S*)-3-phenyl-3-(*p*-tolyl)oxiran-2-yl)methyl propionate (**4c**)

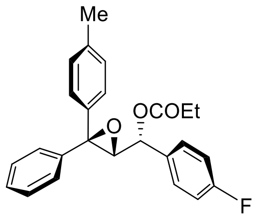

4c

<Chromatogram>

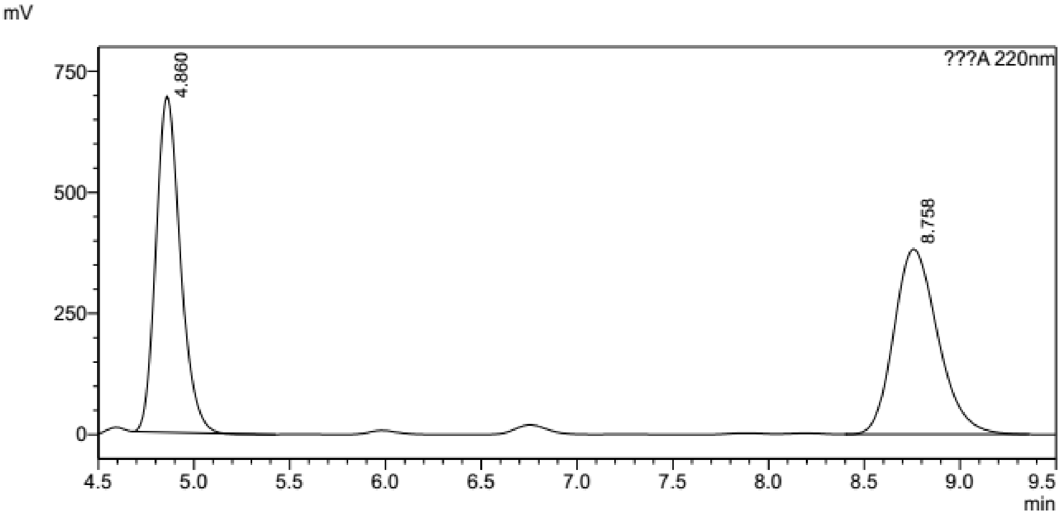

<Peak Table>

???A 220nm

Peak#	Ret. Time	Area	Height	Conc.	Unit	Mark	Name
1	4.860	6159489	692597	50.074		M	
2	8.758	6141364	382428	49.926		M	
Total		12300852	1075026				

<Chromatogram>

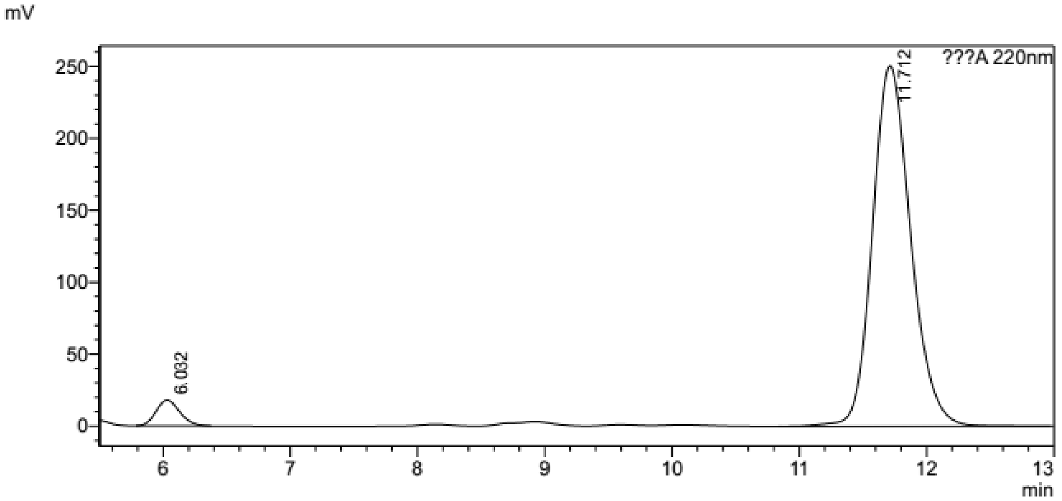

<Peak Table>

???A 220nm

Peak#	Ret. Time	Area	Height	Conc.	Unit	Mark	Name
1	6.032	233745	17917	4.272		M	
2	11.712	5238340	250448	95.728			
Total		5472085	268364				

(*S*)-naphthalen-1-yl((2*S*,3*R*)-3-phenyl-3-(*p*-tolyl)oxiran-2-yl)methanol (**3d**)

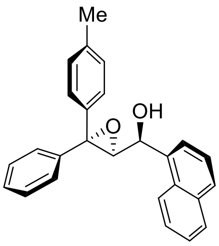

3d

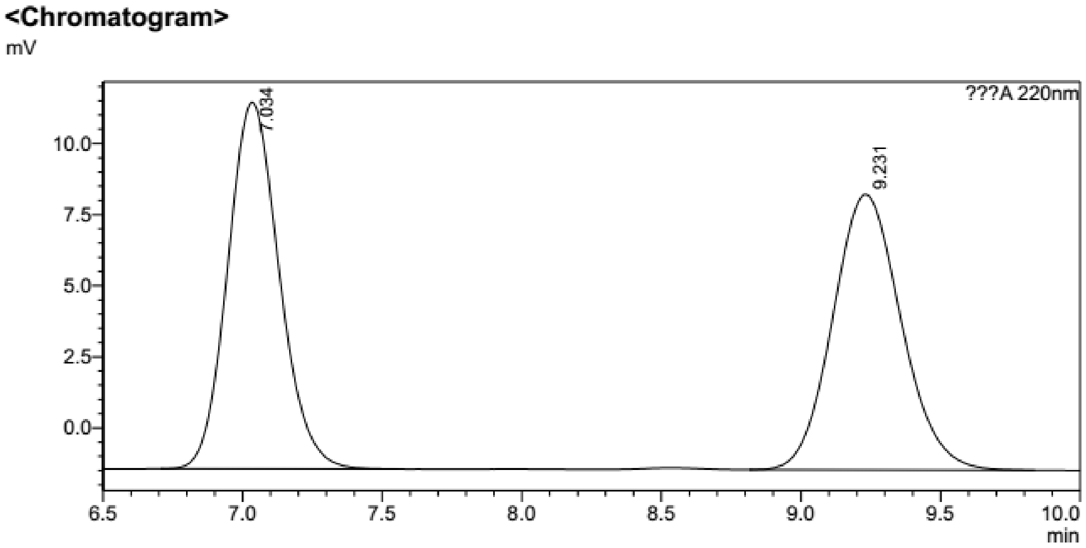

<Peak Table>

???A 220nm

Peak#	Ret. Time	Area	Height	Conc.	Unit	Mark	Name
1	7.034	165744	12890	50.049			
2	9.231	165417	9688	49.951			
Total		331161	22578				

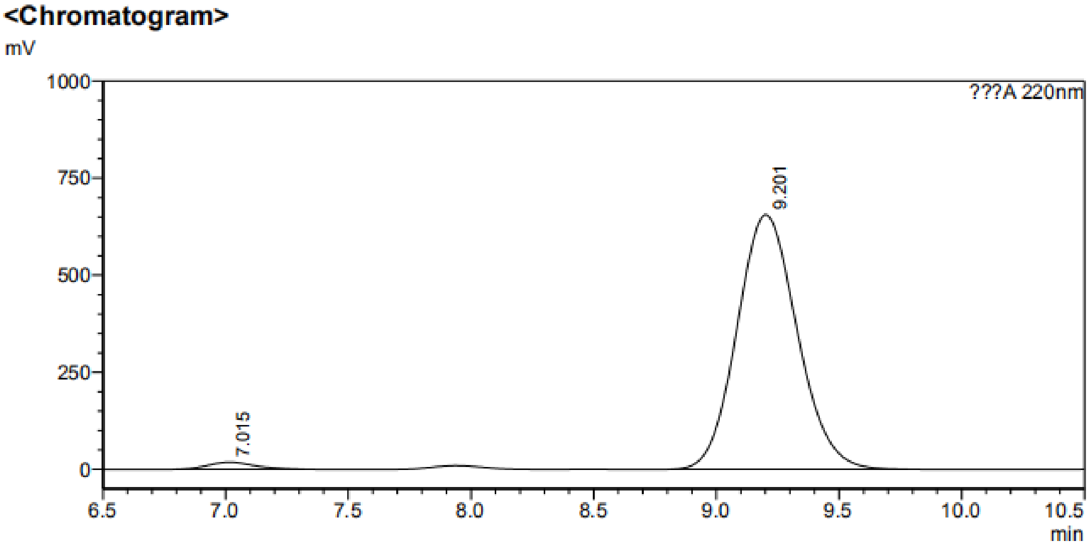

<Peak Table>

???A 220nm

Peak#	Ret. Time	Area	Height	Conc.	Unit	Mark	Name
1	7.015	237122	18221	2.072		M	
2	9.201	11204549	655669	97.928		M	
Total		11441671	673890				

(*R*)-naphthalen-1-yl((2*R*,3*S*)-3-phenyl-3-(*p*-tolyl)oxiran-2-yl)methyl propionate (**4d**)

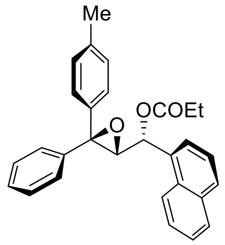

4d

<Chromatogram>

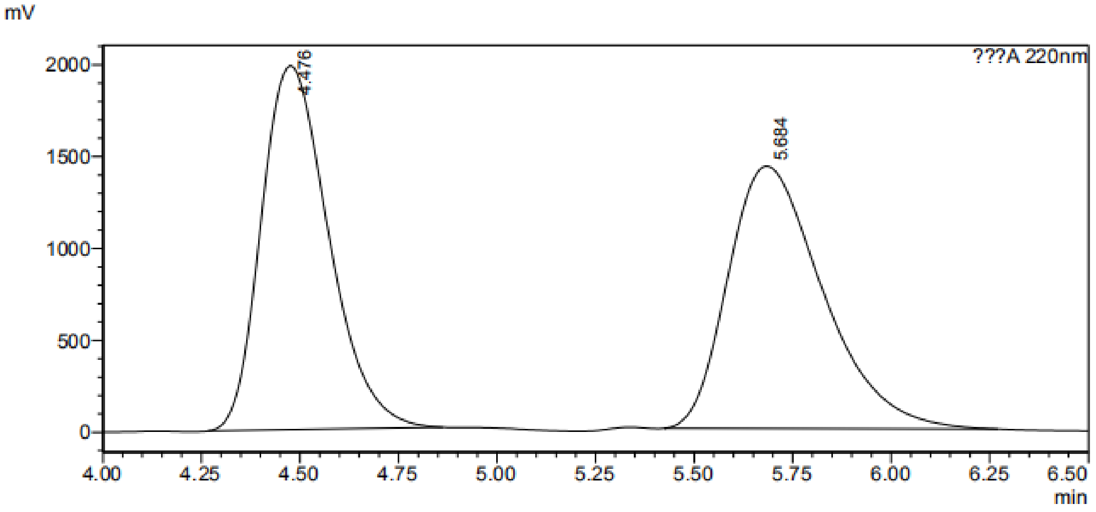

<Peak Table>

???A 220nm

Peak#	Ret. Time	Area	Height	Conc.	Unit	Mark	Name
1	4.476	23598348	1980815	49.617		M	
2	5.684	23962907	1427884	50.383		M	
Total		47561256	3408699				

<Chromatogram>

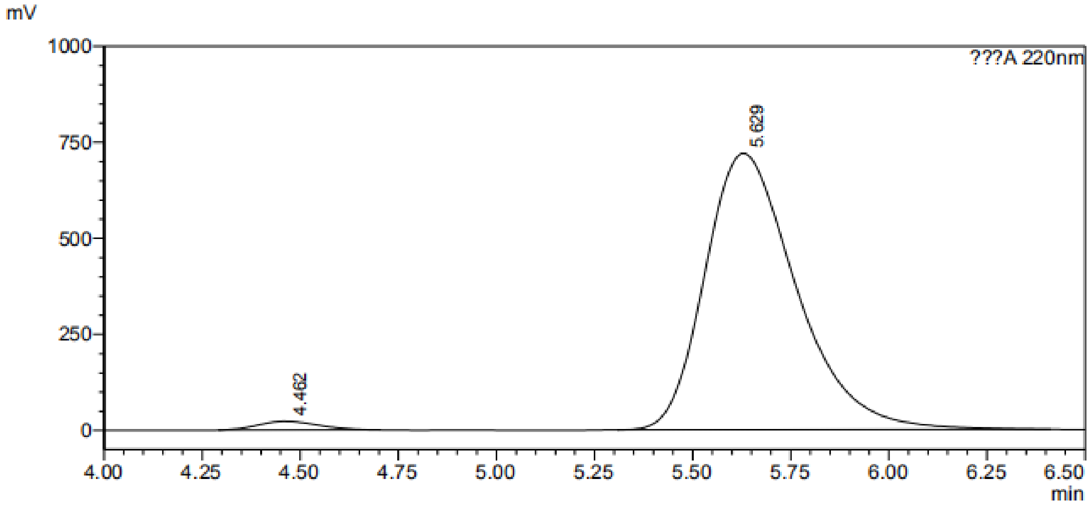

<Peak Table>

???A 220nm

Peak#	Ret. Time	Area	Height	Conc.	Unit	Mark	Name
1	4.462	247970	23160	2.075		M	
2	5.629	11700413	718916	97.925		M	
Total		11948383	742075				

(*S*)-((2*S*,3*S*)-3-(3-chlorophenyl)-3-(4-chlorophenyl)oxiran-2-yl)(phenyl)methanol (**3e**)

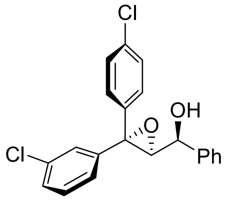

3e

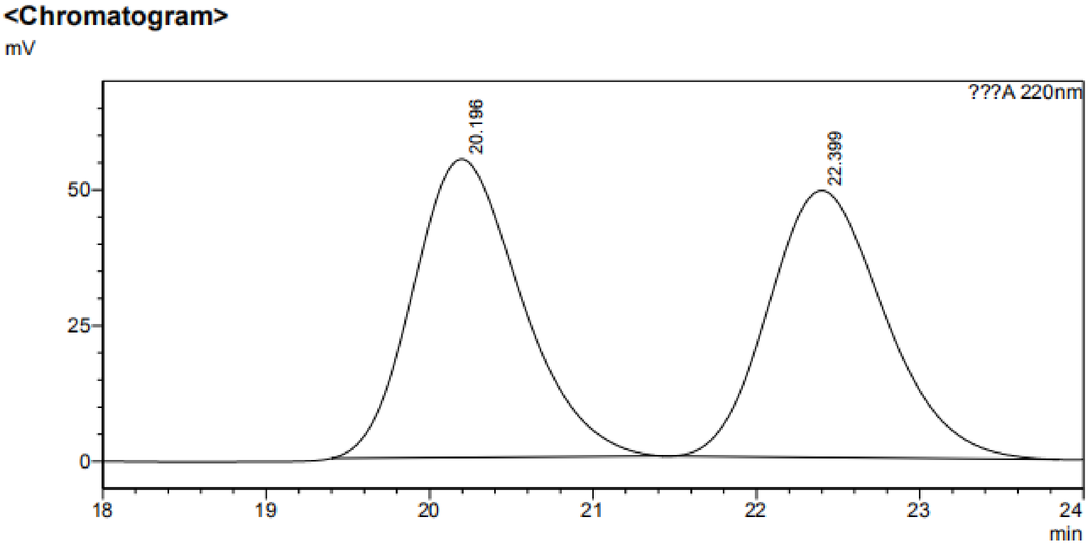

<Peak Table>

???A 220nm

Peak#	Ret. Time	Area	Height	Conc.	Unit	Mark	Name
1	20.196	2522846	54875	50.953		M	
2	22.399	2428445	49117	49.047		M	
Total		4951291	103992				

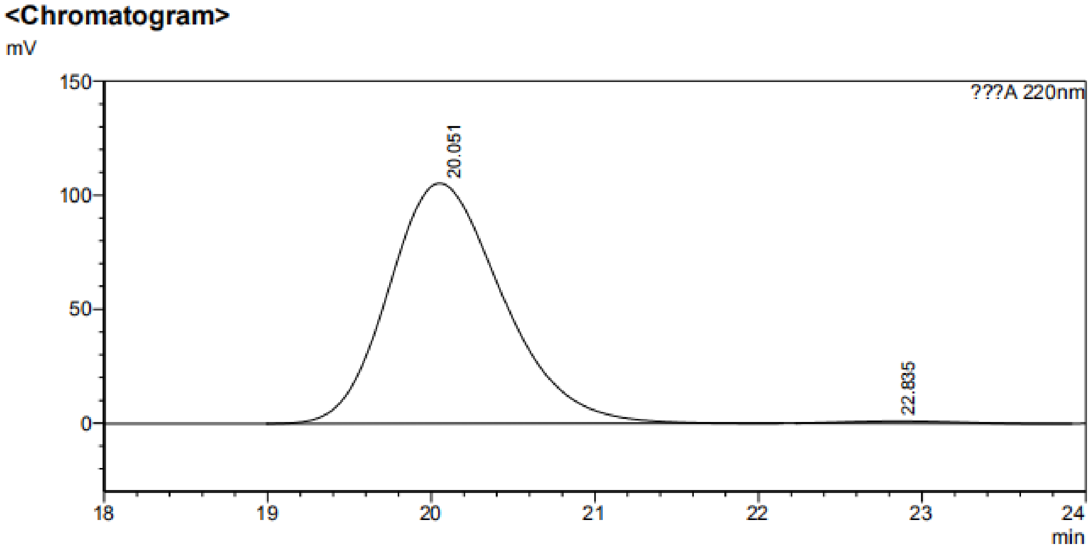

<Peak Table>

???A 220nm

Peak#	Ret. Time	Area	Height	Conc.	Unit	Mark	Name
1	20.051	5104932	105398	99.121		M	
2	22.835	45284	1012	0.879		M	
Total		5150216	106410				

(*R*)-((2*R*,3*R*)-3-(3-chlorophenyl)-3-(4-chlorophenyl)oxiran-2-yl)(phenyl)methyl propionate (**4e**)

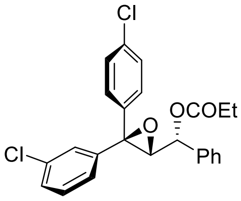

4e

<Chromatogram>

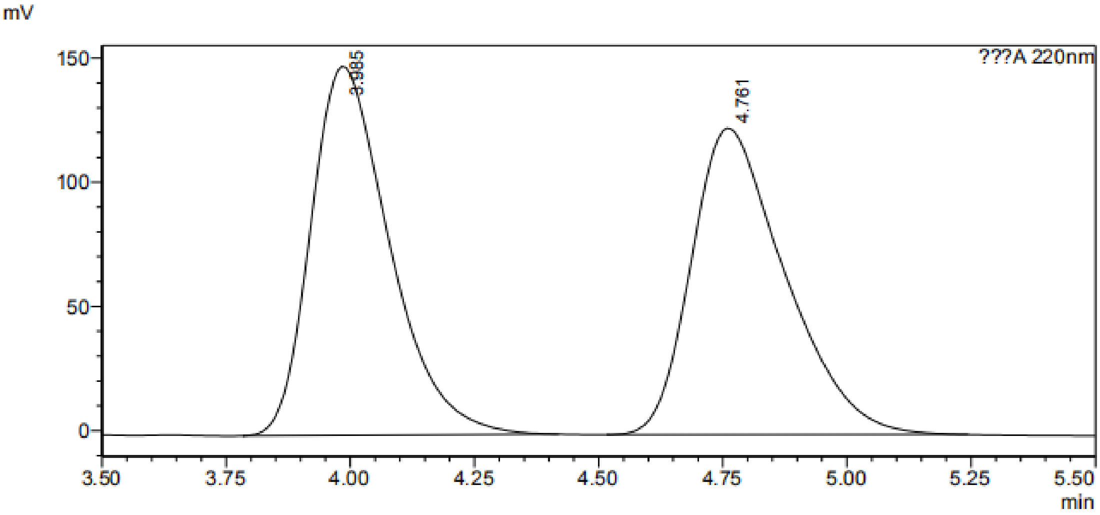

<Peak Table>

???A 220nm

Peak#	Ret. Time	Area	Height	Conc.	Unit	Mark	Name
1	3.985	1640458	148591	50.254		M	
2	4.761	1623901	123381	49.746		M	
Total		3264359	271972				

<Chromatogram>

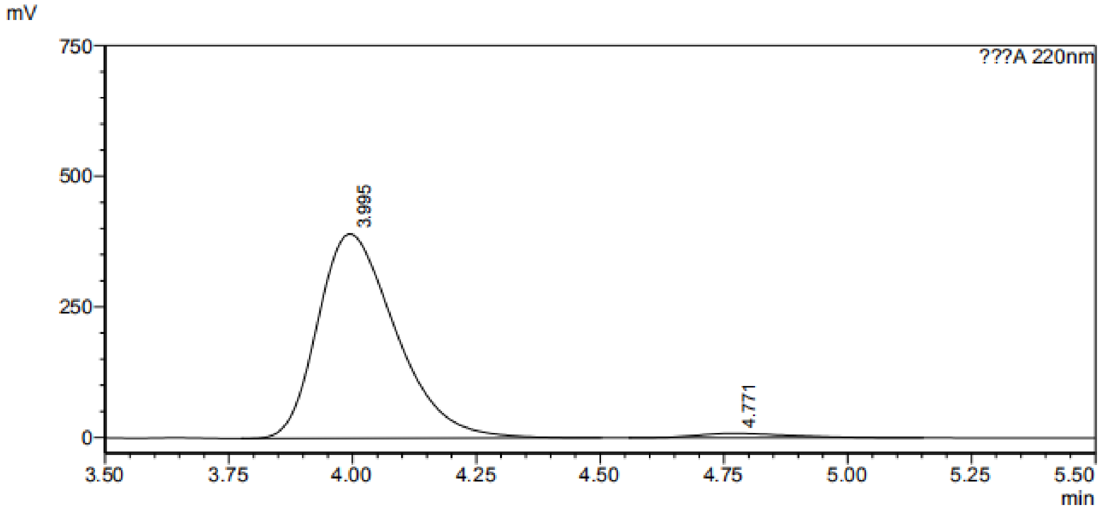

<Peak Table>

???A 220nm

Peak#	Ret. Time	Area	Height	Conc.	Unit	Mark	Name
1	3.995	4298172	391353	97.507		M	
2	4.771	109872	8545	2.493		M	
Total		4408044	399899				

(*S*)-((2*S*,3*S*)-3-(4-ethylphenyl)-3-(*p*-tolyl)oxiran-2-yl)(phenyl)methanol (**3f**)

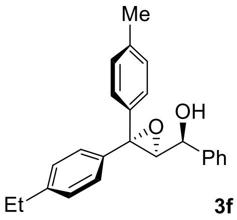

3f

<Chromatogram>

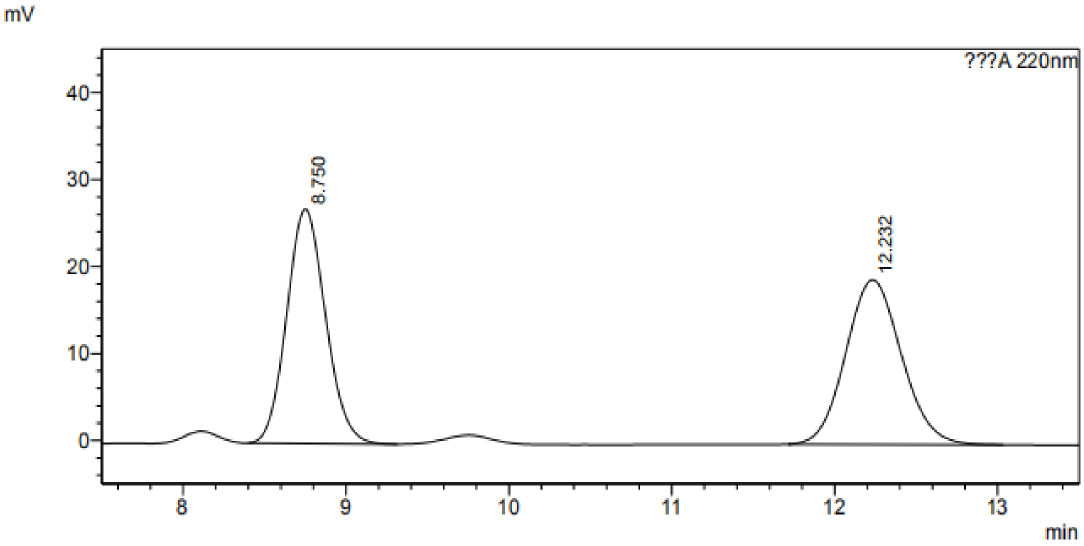

<Peak Table>

???A 220nm

Peak#	Ret. Time	Area	Height	Conc.	Unit	Mark	Name
1	8.750	447178	26959	49.809		M	
2	12.232	450600	18918	50.191		M	
Total		897778	45878				

<Chromatogram>

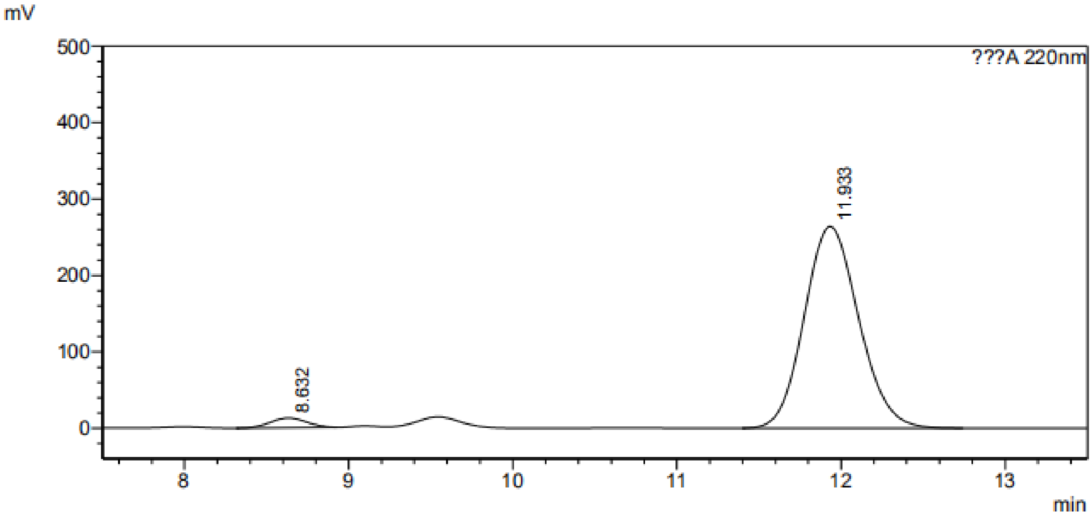

<Peak Table>

???A 220nm

Peak#	Ret. Time	Area	Height	Conc.	Unit	Mark	Name
1	8.632	197101	12944	3.128		M	
2	11.933	6103658	264122	96.872		M	
Total		6300759	277066				

(*S*)-((2*S*,3*S*)-3-(4-ethylphenyl)-3-(*p*-tolyl)oxiran-2-yl)(phenyl)methyl propionate (**4f**)

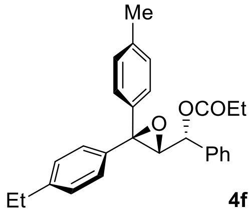

4f

<Chromatogram>

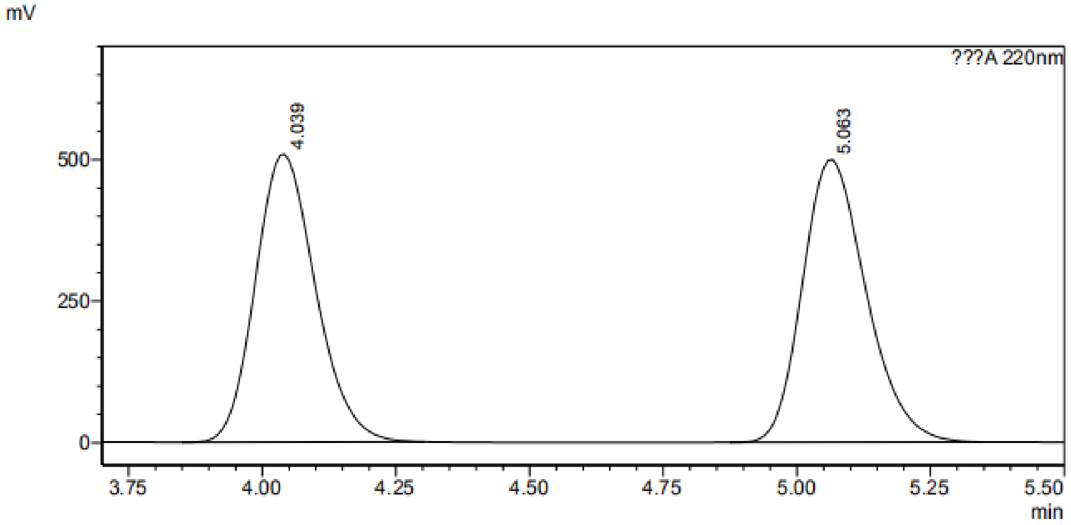

<Peak Table>

???A 220nm

Peak#	Ret. Time	Area	Height	Conc.	Unit	Mark	Name
1	4.039	3989011	508413	49.063		M	
2	5.063	4141397	498941	50.937		M	
Total		8130408	1007354				

<Chromatogram>

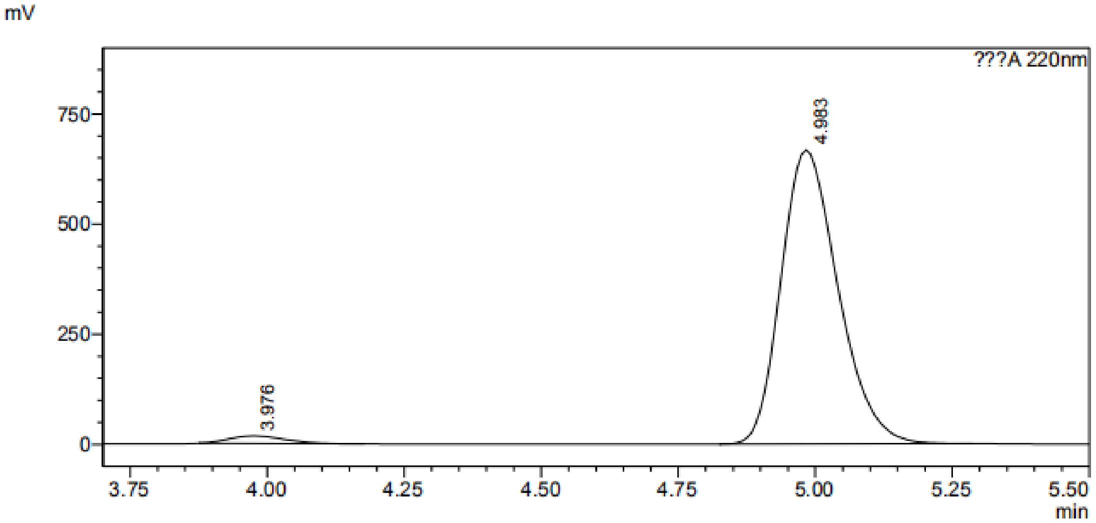

<Peak Table>

???A 220nm

Peak#	Ret. Time	Area	Height	Conc.	Unit	Mark	Name
1	3.976	121441	17326	2.454		M	
2	4.983	4827667	667235	97.546		M	
Total		4949108	684561				

(*S*)-((2*S*,3*R*)-3-(3-chlorophenyl)-3-(4-chlorophenyl)oxiran-2-yl)(phenyl)methanol (**3g**)

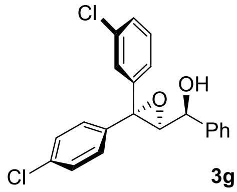

3g

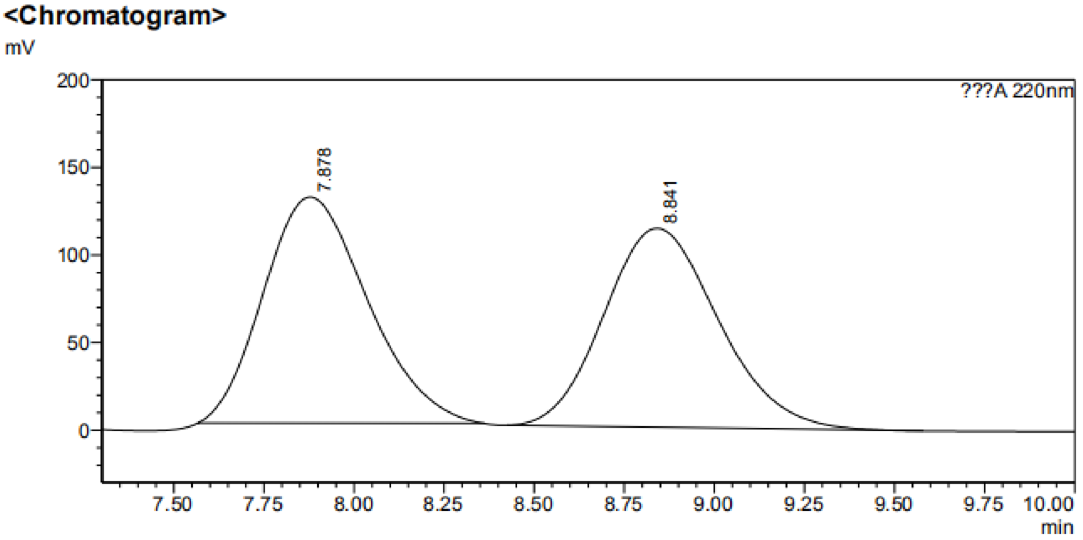

<Peak Table>

???A 220nm

Peak#	Ret. Time	Area	Height	Conc.	Unit	Mark	Name
1	7.878	2599508	129097	50.849		M	
2	8.841	2512728	113684	49.151		M	
Total		5112235	242781				

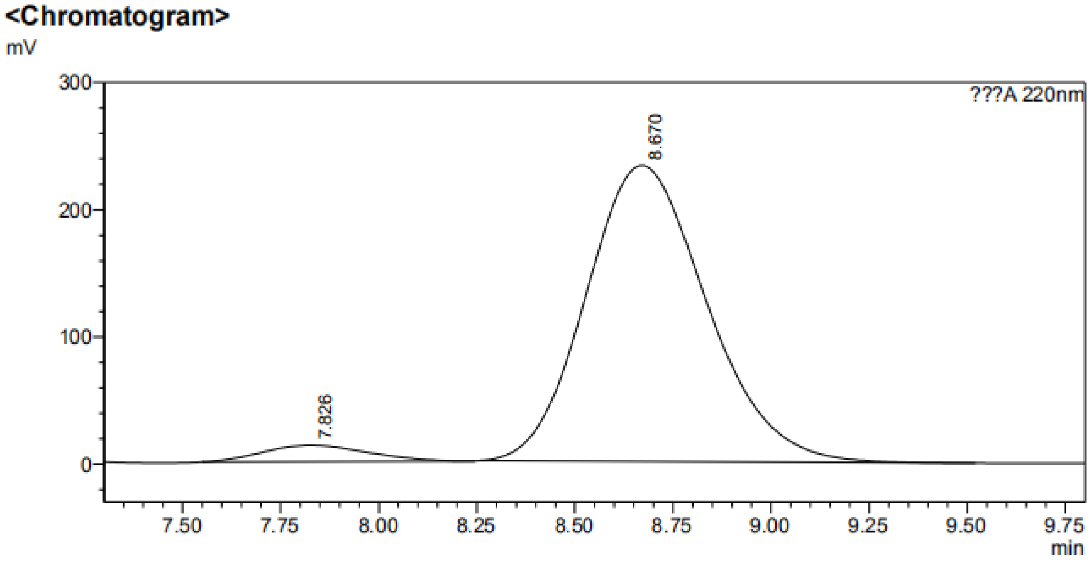

<Peak Table>

???A 220nm

Peak#	Ret. Time	Area	Height	Conc.	Unit	Mark	Name
1	7.826	240624	12983	4.602		M	
2	8.670	4988258	232698	95.398		M	
Total		5228881	245681				

(*R*)-((2*R*,3*S*)-3-(3-chlorophenyl)-3-(4-chlorophenyl)oxiran-2-yl)(phenyl)methyl propionate (**4g**)

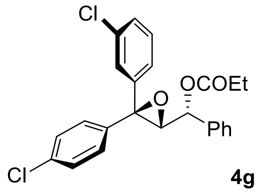

4g

<Chromatogram>

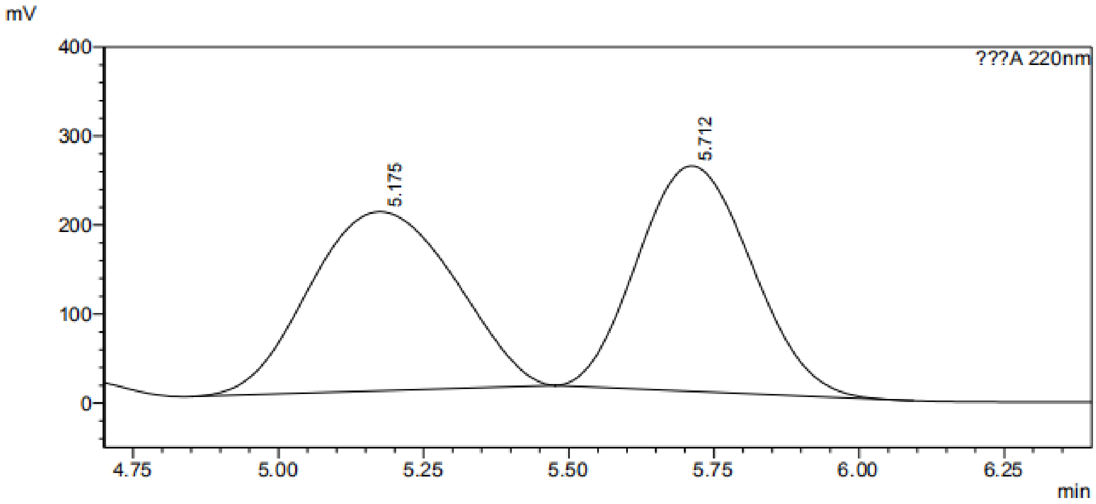

<Peak Table>

???A 220nm

Peak#	Ret. Time	Area	Height	Conc.	Unit	Mark	Name
1	5.175	3500219	201257	50.251		M	
2	5.712	3465222	253242	49.749		M	
Total		6965442	454499				

<Chromatogram>

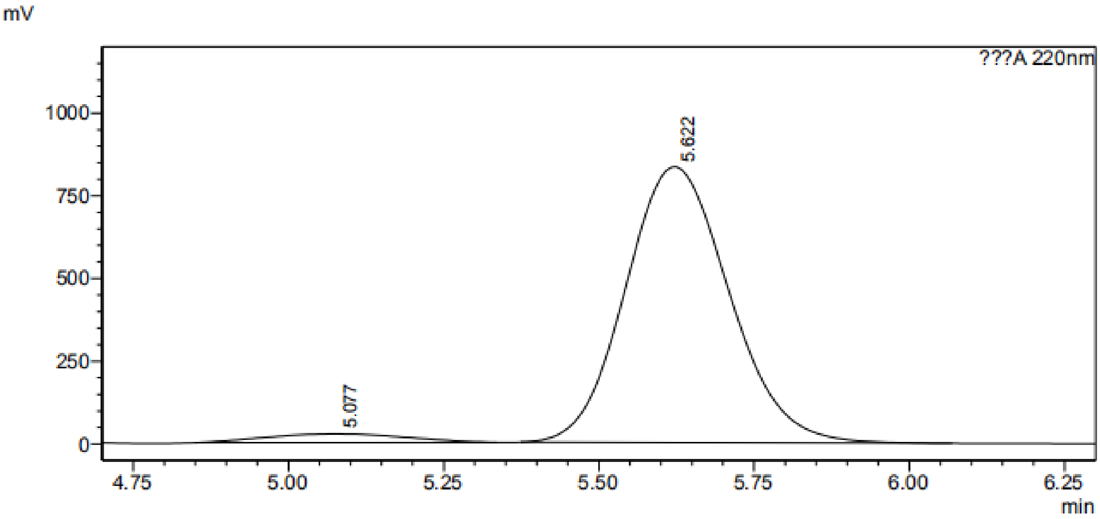

<Peak Table>

???A 220nm

Peak#	Ret. Time	Area	Height	Conc.	Unit	Mark	Name
1	5.077	402570	25673	3.989		M	
2	5.622	9690375	833622	96.011		M	
Total		10092945	859295				

(*S*)-((2*S*,3*R*)-3-hexyl-3-pentyloxiran-2-yl)(phenyl)methanol (**3h**)

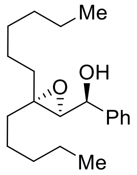

3h

<Chromatogram>

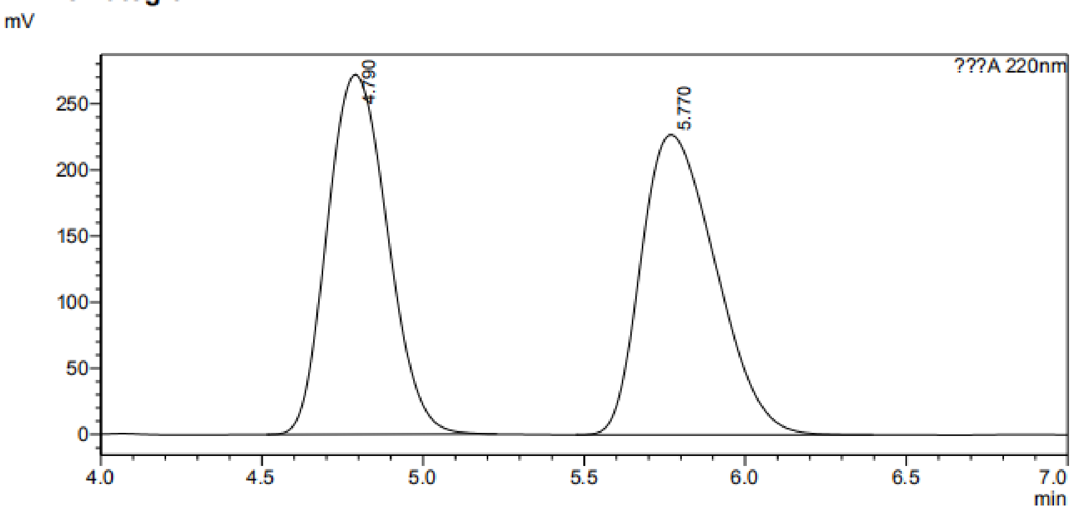

<Peak Table>

???A 220nm

Peak#	Ret. Time	Area	Height	Conc.	Unit	Mark	Name
1	4.790	3538926	271983	49.171		M	
2	5.770	3658182	226893	50.829			
Total		7197109	498876				

<Chromatogram>

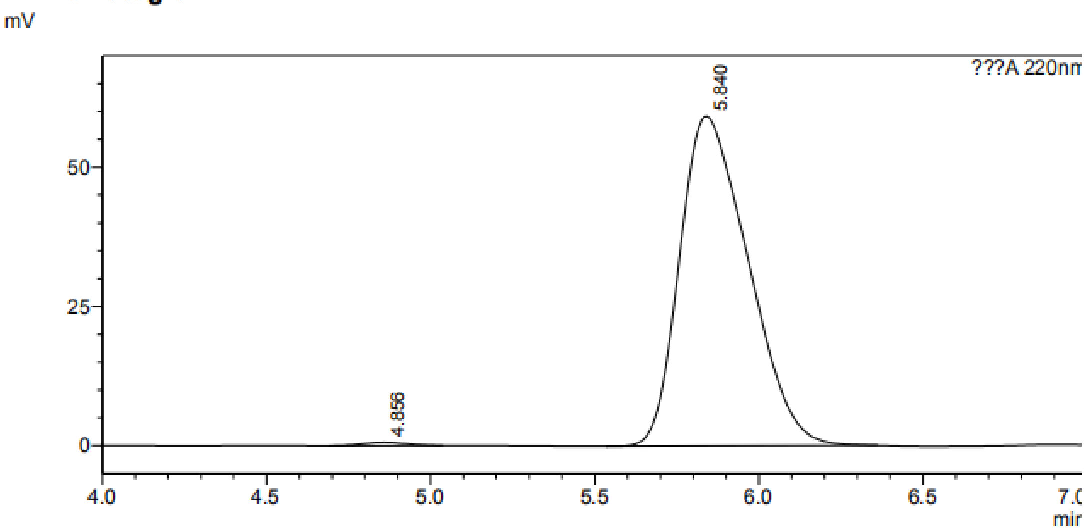

<Peak Table>

???A 220nm

Peak#	Ret. Time	Area	Height	Conc.	Unit	Mark	Name
1	4.856	5429	588	0.627		M	
2	5.840	859907	59190	99.373		M	
Total		865336	59778				

(*S*)-((2*S*,3*R*)-3-hexyl-3-pentyloxiran-2-yl)(phenyl)methyl propionate **(4h)**

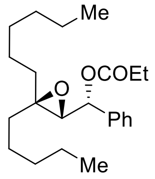

4h

<Chromatogram>

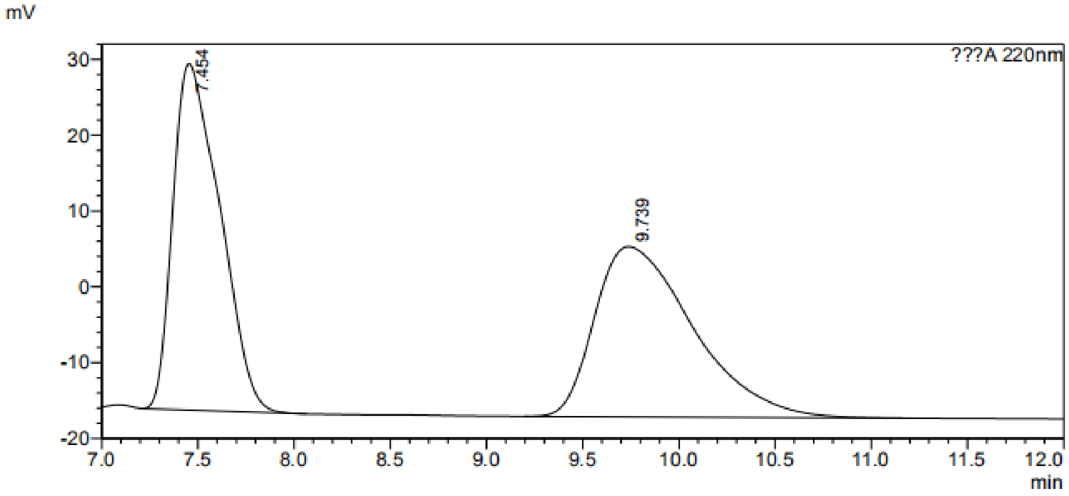

<Peak Table>

???A 220nm

Peak#	Ret. Time	Area	Height	Conc.	Unit	Mark	Name
1	7.454	775355	45651	49.256			
2	9.739	798788	22440	50.744			
Total		1574143	68092				

<Chromatogram>

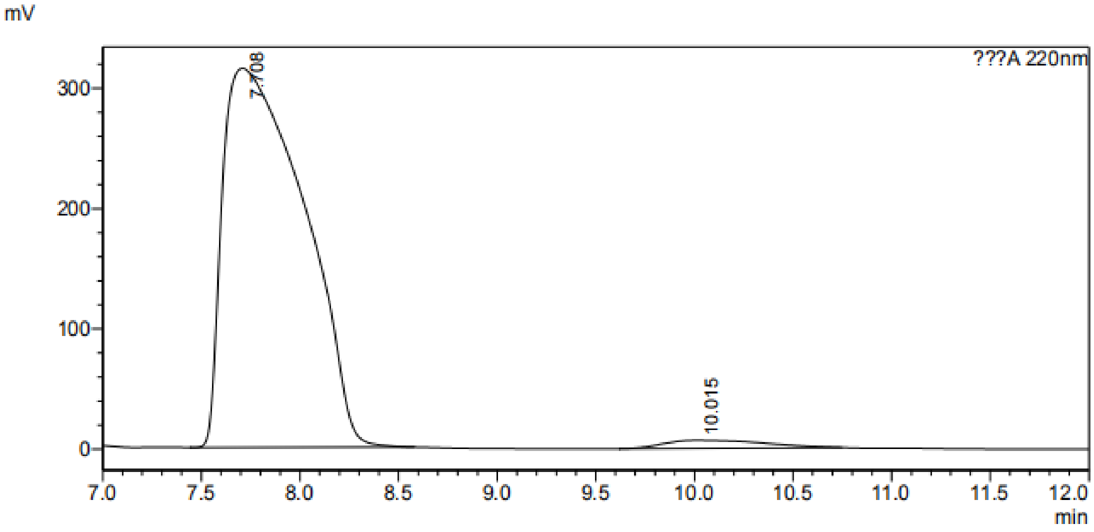

<Peak Table>

???A 220nm

Peak#	Ret. Time	Area	Height	Conc.	Unit	Mark	Name
1	7.708	9042159	315339	97.482		M	
2	10.015	233555	6692	2.518		M	
Total		9275714	322031				

(*S*)-((2*S*,3*R*)-3-methyl-3-(trifluoromethyl)oxiran-2-yl)(phenyl)methanol (**3i**)

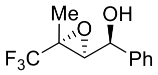

3i

<Chromatogram>

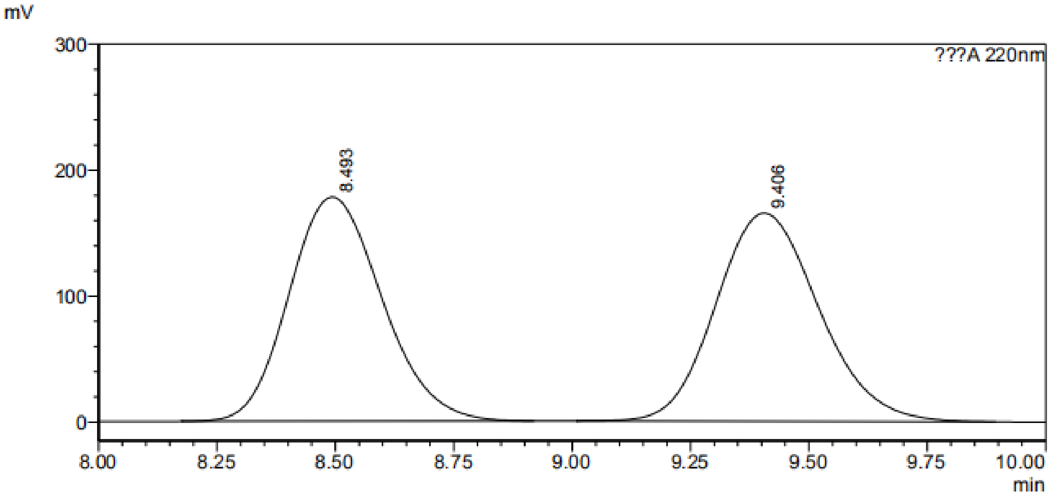

<Peak Table>

???A 220nm

Peak#	Ret. Time	Area	Height	Conc.	Unit	Mark	Name
1	8.493	2439056	178003	49.306		M	
2	9.406	2507680	165163	50.694		M	
Total		4946737	343165				

<Chromatogram>

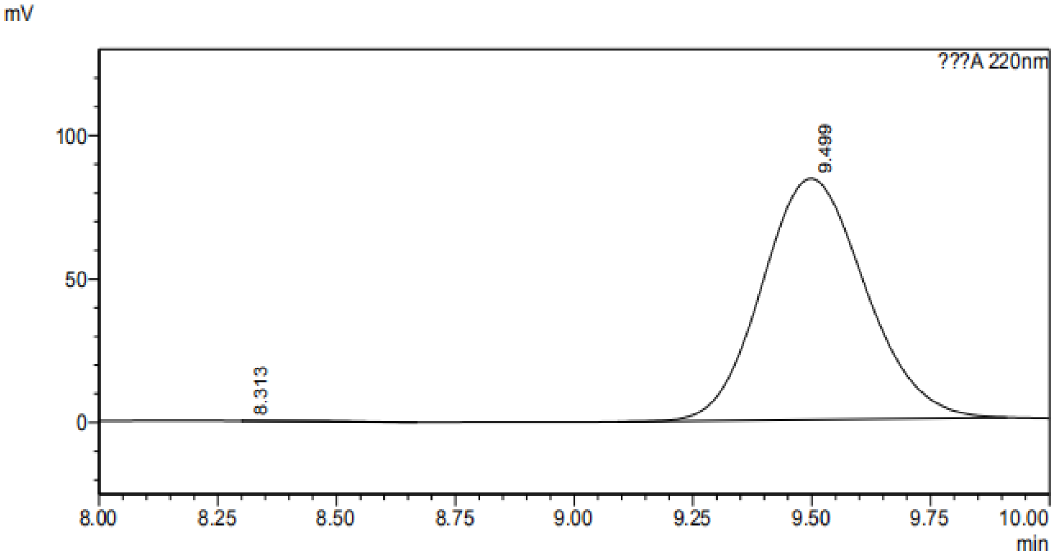

<Peak Table>

???A 220nm

Peak#	Ret. Time	Area	Height	Conc.	Unit	Mark	Name
1	8.313	2020	13	0.158		M	
2	9.499	1273552	84036	99.842		M	
Total		1275572	84049				

(*R*)-((2*R*,3*S*)-3-methyl-3-(trifluoromethyl)oxiran-2-yl)(phenyl)methyl propionate (**4i**)

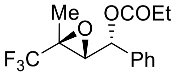

4i

<Chromatogram>

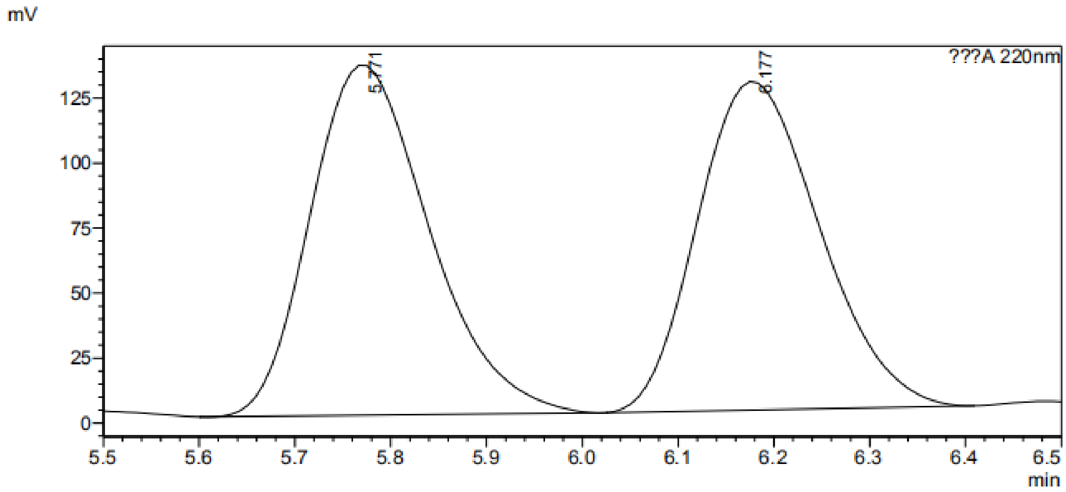

<Peak Table>

???A 220nm

Peak#	Ret. Time	Area	Height	Conc.	Unit	Mark	Name
1	5.771	1161786	134467	50.892		M	
2	6.177	1121078	126323	49.108		M	
Total		2282864	260789				

<Chromatogram>

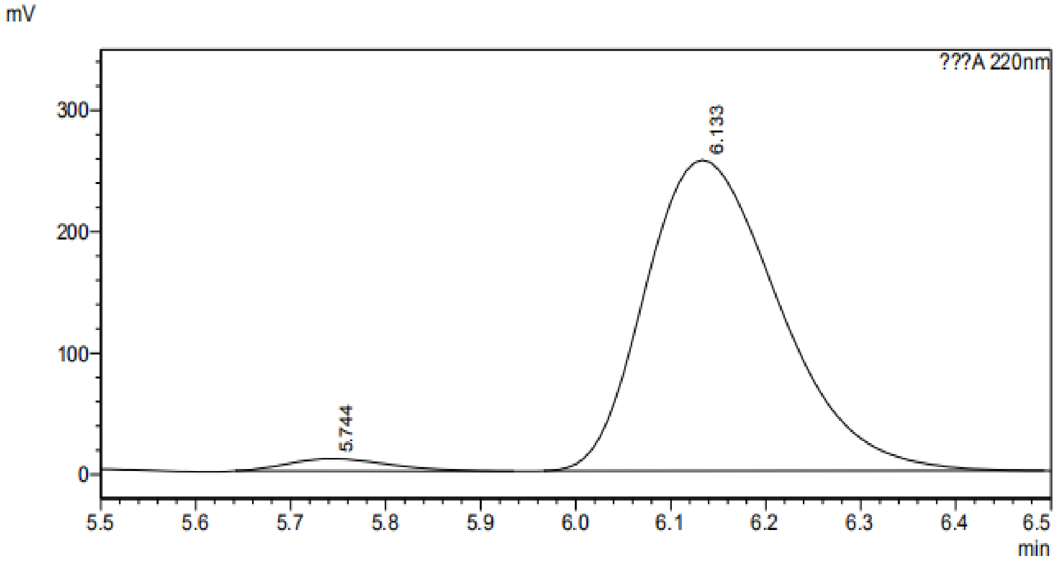

<Peak Table>

???A 220nm

Peak#	Ret. Time	Area	Height	Conc.	Unit	Mark	Name
1	5.744	76729	10277	2.967		M	
2	6.133	2509588	256146	97.033		M	
Total		2586317	266424				

(*S*)-((2*S*,3*S*)-3-ethyl-3-methyloxiran-2-yl)(phenyl)methanol (**3j**)

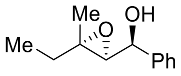

3j

<Chromatogram>

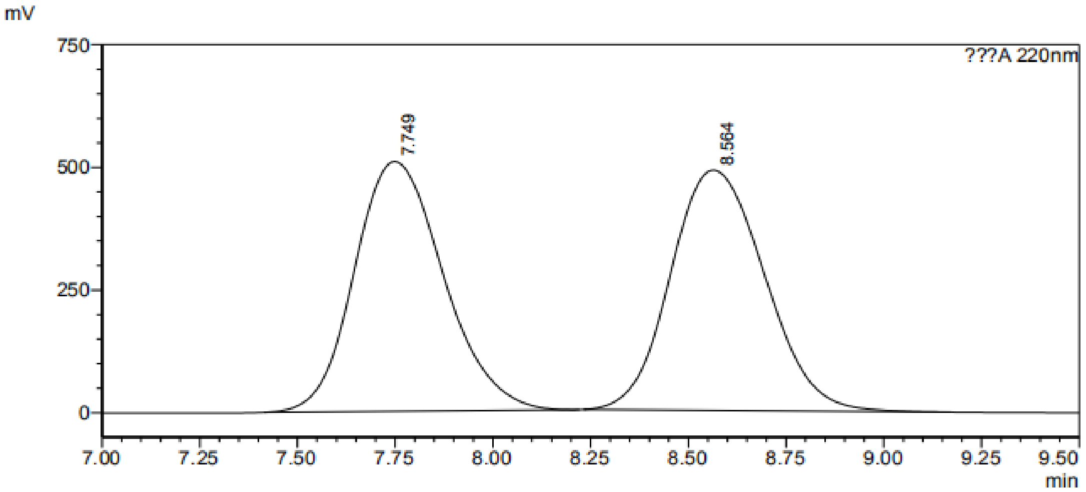

<Peak Table>

???A 220nm

Peak#	Ret. Time	Area	Height	Conc.	Unit	Mark	Name
1	7.749	8076297	509219	49.588		M	
2	8.564	8210478	489796	50.412		M	
Total		16286775	999015				

<Chromatogram>

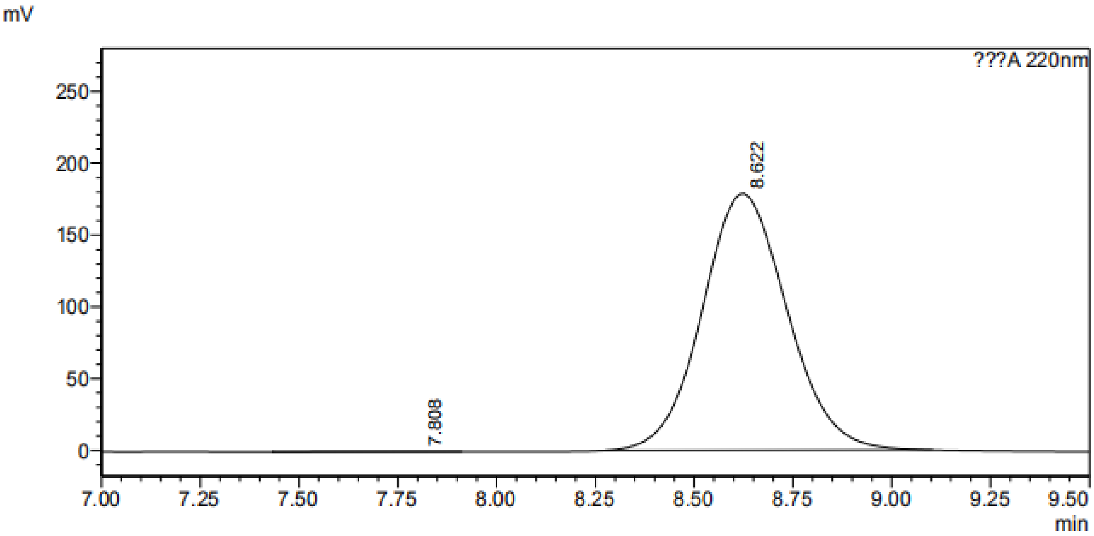

<Peak Table>

???A 220nm

Peak#	Ret. Time	Area	Height	Conc.	Unit	Mark	Name
1	7.808	354	31	0.013		M	
2	8.622	2686173	178684	99.987		M	
Total		2686528	178716				

(*R*)-((2*R*,3*R*)-3-ethyl-3-methyloxiran-2-yl)(phenyl)methyl propionate (**4j**)

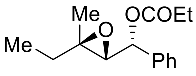

4j

<Chromatogram>

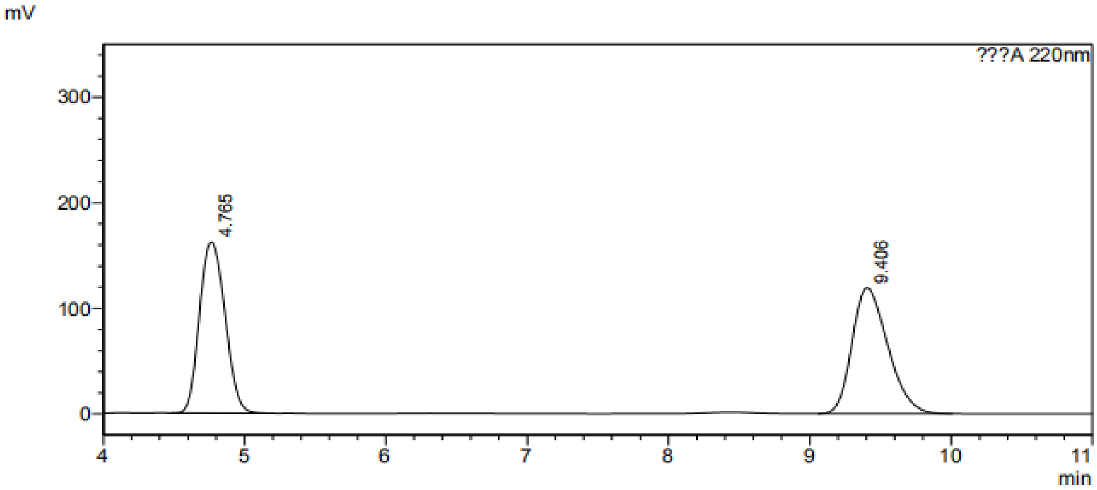

<Peak Table>

???A 220nm

Peak#	Ret. Time	Area	Height	Conc.	Unit	Mark	Name
1	4.765	2039021	161988	49.237		M	
2	9.406	2102190	119221	50.763		M	
Total		4141211	281208				

<Chromatogram>

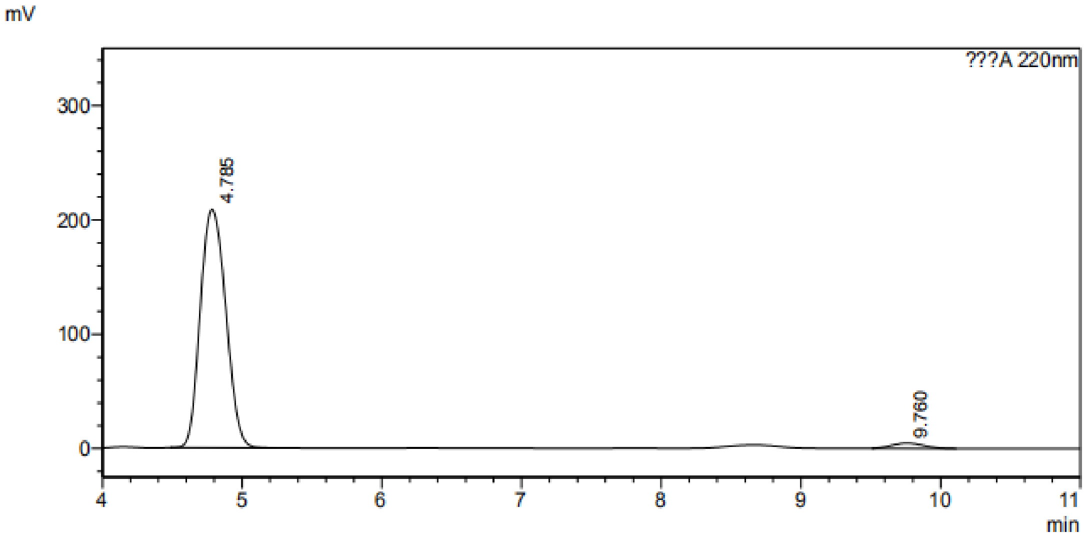

<Peak Table>

???A 220nm

Peak#	Ret. Time	Area	Height	Conc.	Unit	Mark	Name
1	4.785	2649035	208569	97.289		M	
2	9.760	73828	4724	2.711		M	
Total		2722863	213293				

(*S*)-((2*S*,3*R*)-3-ethyl-3-methyloxiran-2-yl)(phenyl)methanol (**3k**)

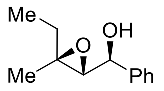

3k

<Chromatogram>

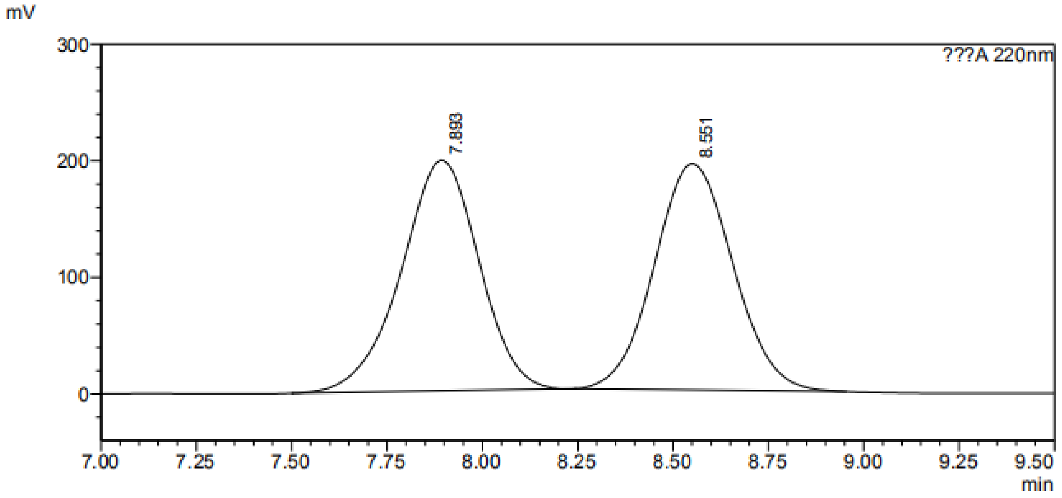

<Peak Table>

???A 220nm

Peak#	Ret. Time	Area	Height	Conc.	Unit	Mark	Name
1	7.893	2813704	197769	50.183		M	
2	8.551	2793172	194261	49.817		M	
Total		5606876	392030				

<Chromatogram>

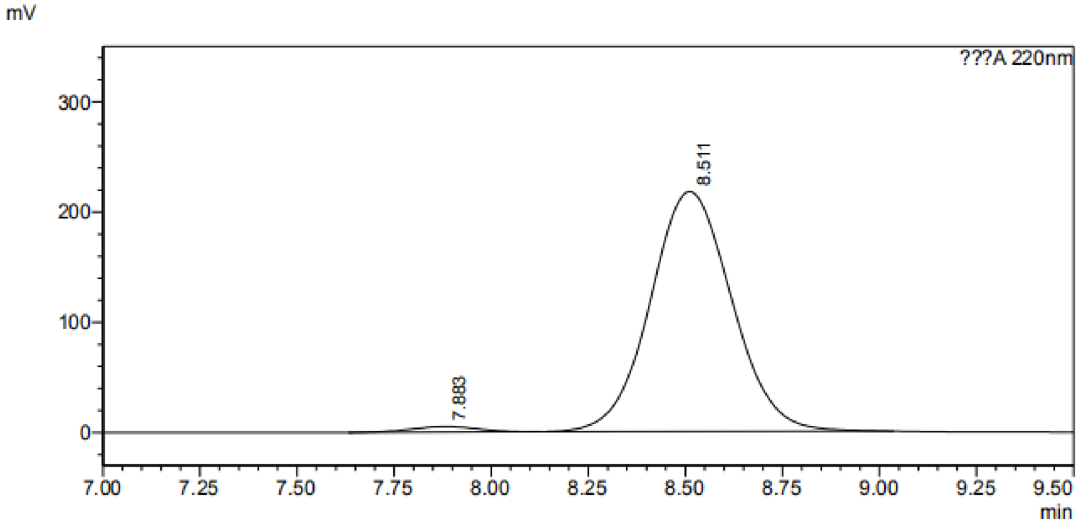

<Peak Table>

???A 220nm

Peak#	Ret. Time	Area	Height	Conc.	Unit	Mark	Name
1	7.883	62022	5188	1.910		M	
2	8.511	3184440	217689	98.090		M	
Total		3246462	222877				

<u>(*R*)-((2*R*,3*S*)-3-ethyl-3-methyloxiran-2-yl)(phenyl)methyl propionate</u> <u>(**4k**)</u>

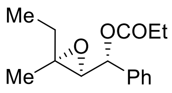

4k

<Chromatogram>

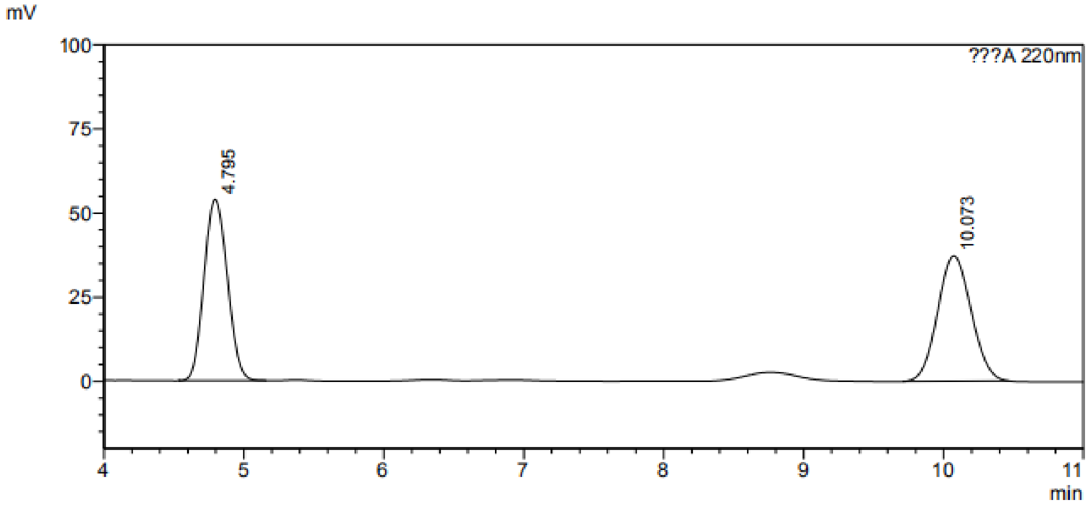

<Peak Table>

???A 220nm

Peak#	Ret. Time	Area	Height	Conc.	Unit	Mark	Name
1	4.795	626055	53843	50.180		M	
2	10.073	621563	37197	49.820		M	
Total		1247619	91040				

<Chromatogram>

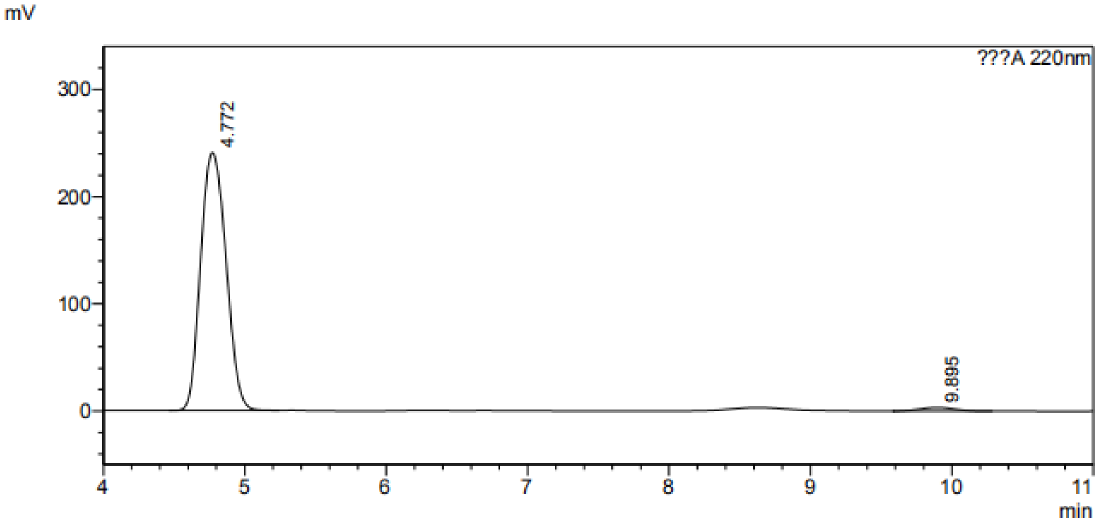

<Peak Table>

???A 220nm

Peak#	Ret. Time	Area	Height	Conc.	Unit	Mark	Name
1	4.772	3007617	240679	98.241		M	
2	9.895	53848	3250	1.759		M	
Total		3061466	243929				

Ethyl (*R*)-2-((*S*)-hydroxy(phenyl)methyl)-2-methylpent-4-enoate (**5a**)

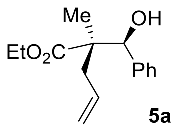

5a

<Chromatogram>

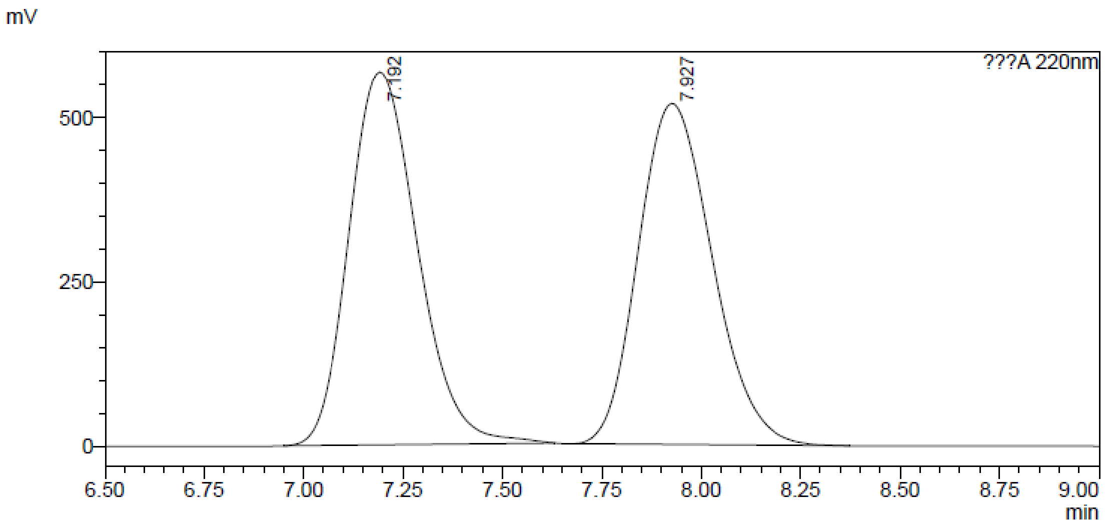

<Peak Table>

???A 220nm

Peak#	Ret. Time	Area	Height	Conc.	Unit	Mark	Name
1	7.192	6796459	567917	50.309		M	
2	7.927	6712865	518873	49.691		M	
Total		13509325	1086790				

<Chromatogram>

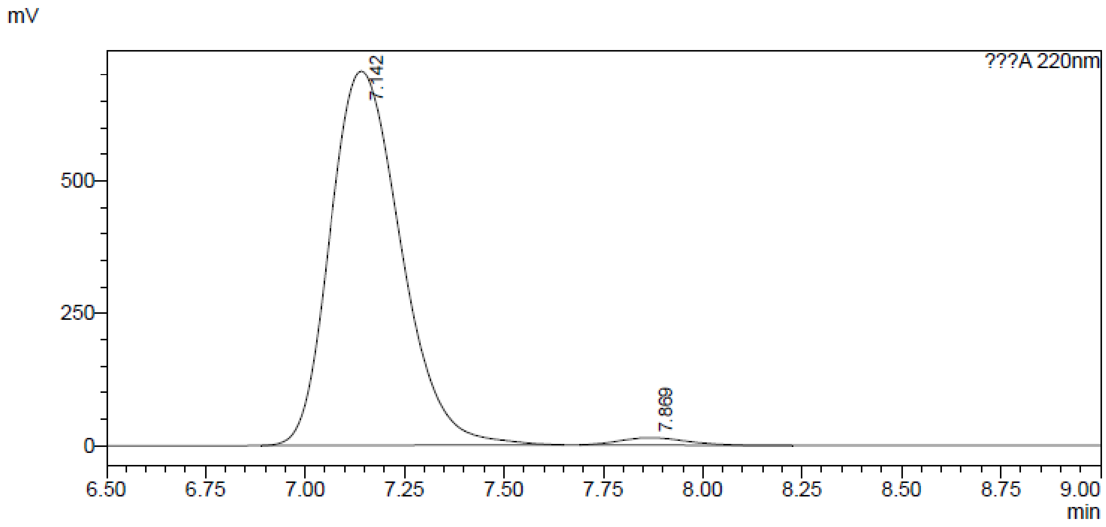

<Peak Table>

???A 220nm

Peak#	Ret. Time	Area	Height	Conc.	Unit	Mark	Name
1	7.142	8751907	707821	98.220		M	
2	7.869	158605	13910	1.780		M	
Total		8910512	721731				

Ethyl (*S*)-2-methyl-2-((*R*)-phenyl(propionyloxy)methyl)pent-4-enoate (**6a**)

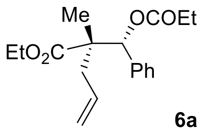

6a

<Chromatogram>

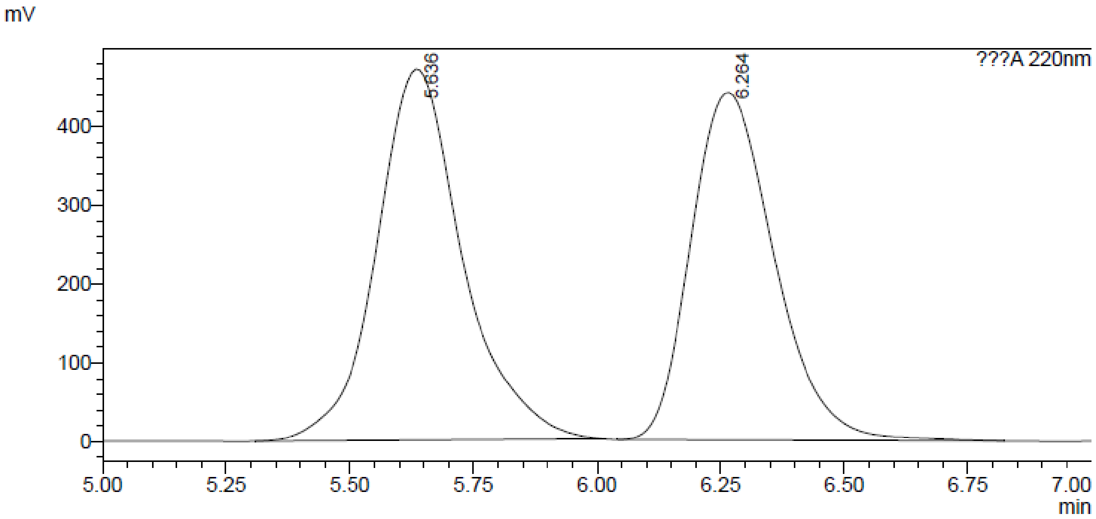

<Peak Table>

???A 220nm

Peak#	Ret. Time	Area	Height	Conc.	Unit	Mark	Name
1	5.636	5698399	471098	52.409		M	
2	6.264	5174506	441193	47.591		M	
Total		10872905	912291				

<Chromatogram>

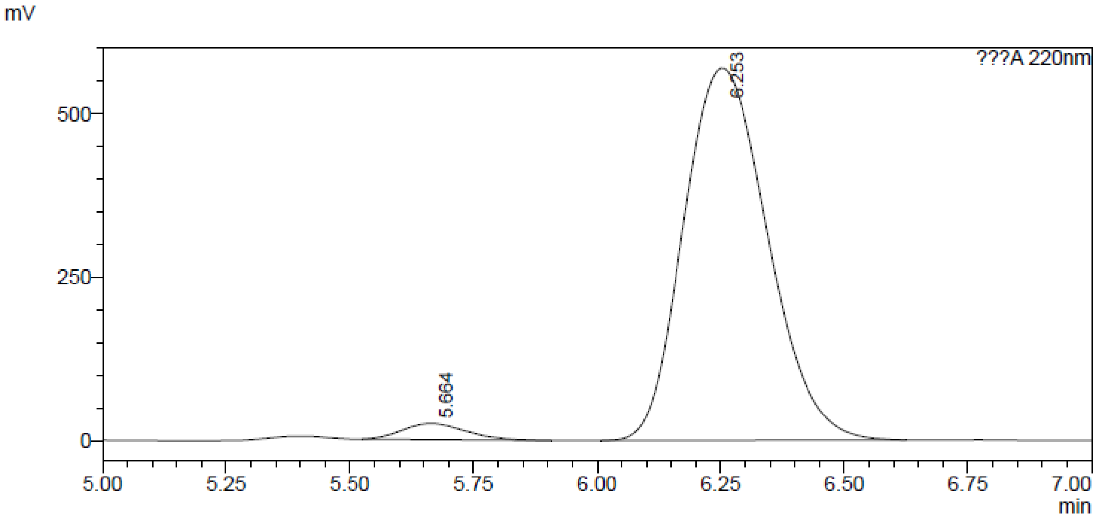

<Peak Table>

???A 220nm

Peak#	Ret. Time	Area	Height	Conc.	Unit	Mark	Name
1	5.664	216868	24399	3.124		M	
2	6.253	6725286	569115	96.876		M	
Total		6942154	593514				

Ethyl (*S*)-2-((*S*)-hydroxy(phenyl)methyl)-2-methylpent-4-enoate (**5b**)

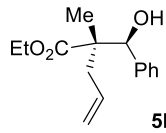

5b

<Chromatogram>

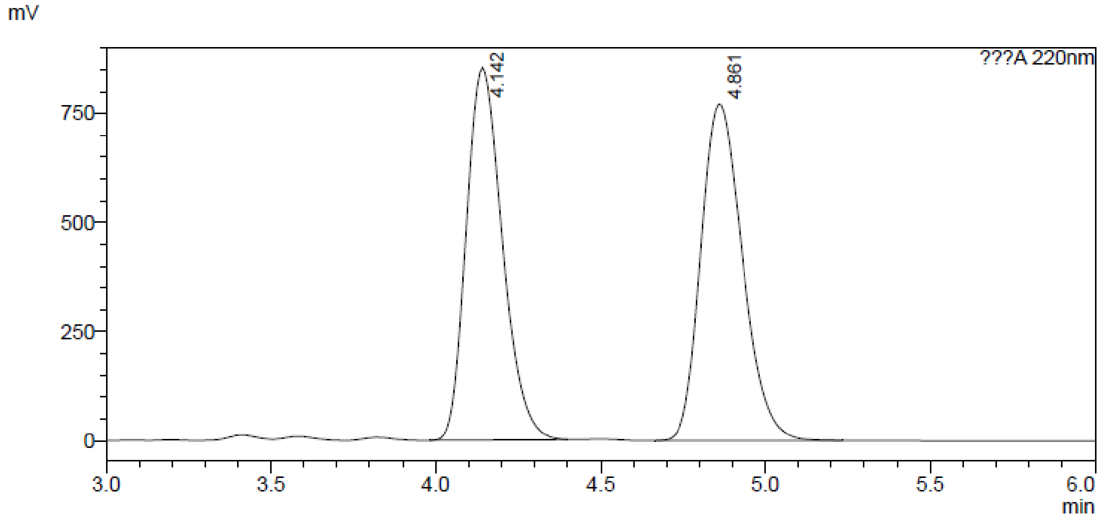

<Peak Table>

???A 220nm

Peak#	Ret. Time	Area	Height	Conc.	Unit	Mark	Name
1	4.142	6542971	853058	49.105		M	
2	4.861	6781522	771012	50.895		M	
Total		13324493	1624070				

<Chromatogram>

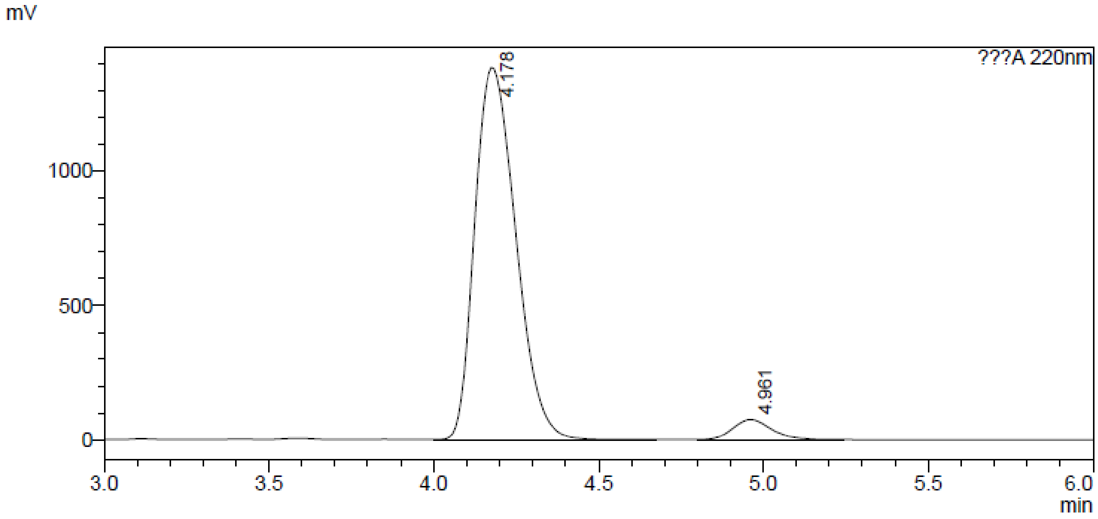

<Peak Table>

???A 220nm

Peak#	Ret. Time	Area	Height	Conc.	Unit	Mark	Name
1	4.178	12042896	1384903	95.074		M	
2	4.961	623952	75651	4.926		M	
Total		12666848	1460554				

Ethyl (*R*)-2-methyl-2-((*R*)-phenyl(propionyloxy)methyl)pent-4-enoate (**6b**)

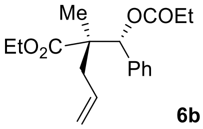

6b

<Chromatogram>

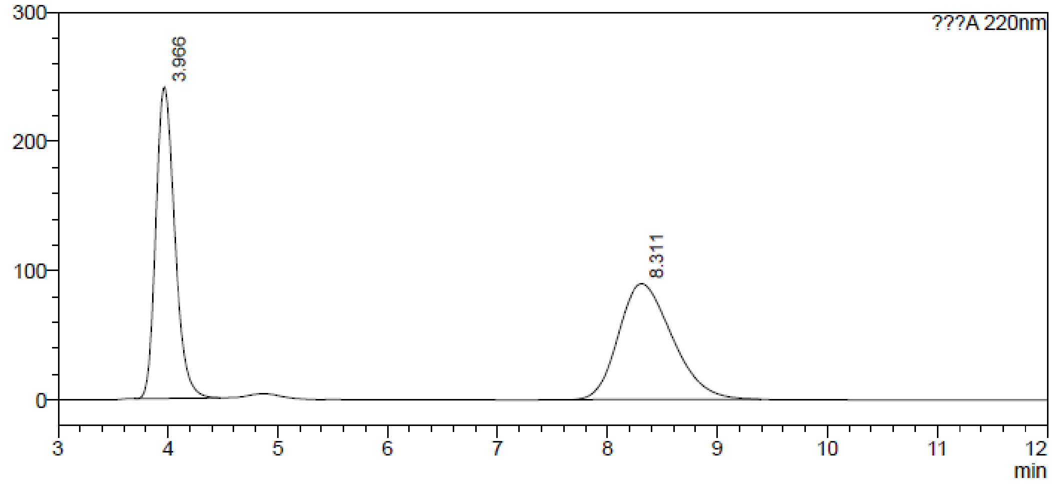

<Peak Table>

???A 220nm

Peak#	Ret. Time	Area	Height	Conc.	Unit	Mark	Name
1	3.966	2897428	240901	48.725		M	
2	8.311	3049025	89539	51.275		M	
Total		5946453	330441				

<Chromatogram>

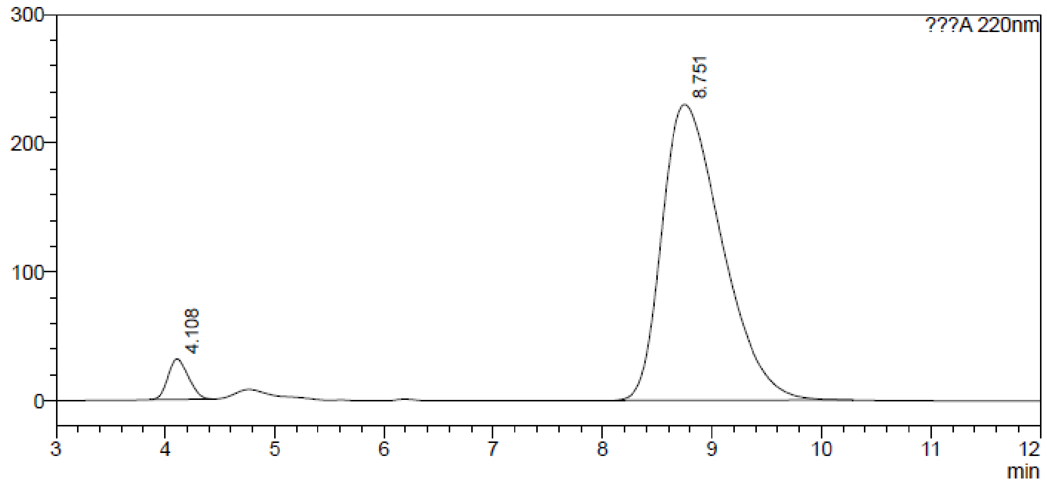

<Peak Table>

???A 220nm

Peak#	Ret. Time	Area	Height	Conc.	Unit	Mark	Name
1	4.108	402516	31414	4.389		M	
2	8.751	8768710	229529	95.611		M	
Total		9171226	260942				

Methyl (*R*)-2-((*S*)-hydroxy(phenyl)methyl)-2-phenylpent-4-enoate (**5c**)

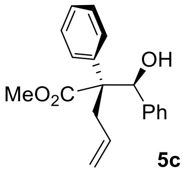

5c

<Chromatogram>

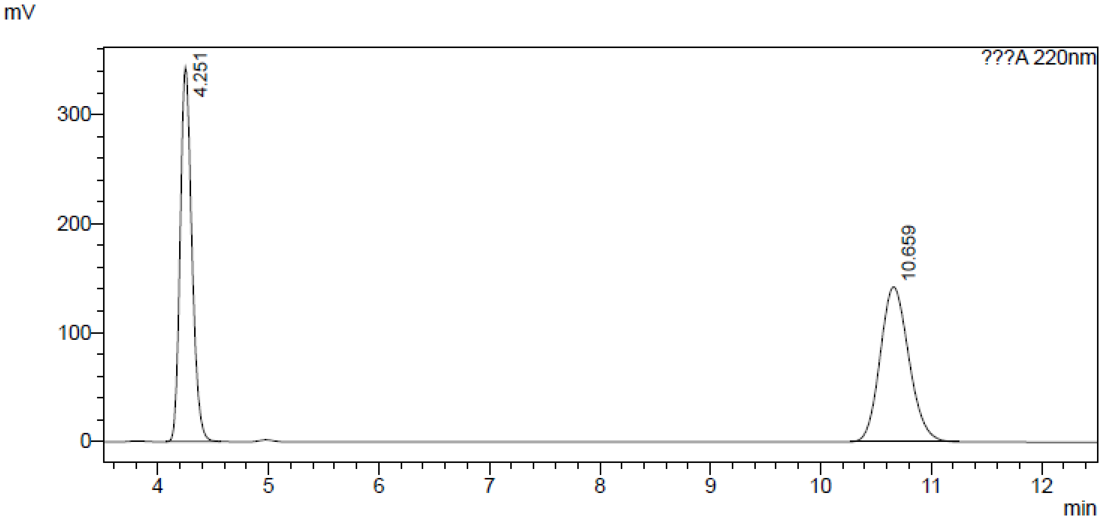

<Peak Table>

???A 220nm

Peak#	Ret. Time	Area	Height	Conc.	Unit	Mark	Name
1	4.251	2521231	343432	49.548		M	
2	10.659	2567187	142348	50.452		M	
Total		5088418	485780				

<Chromatogram>

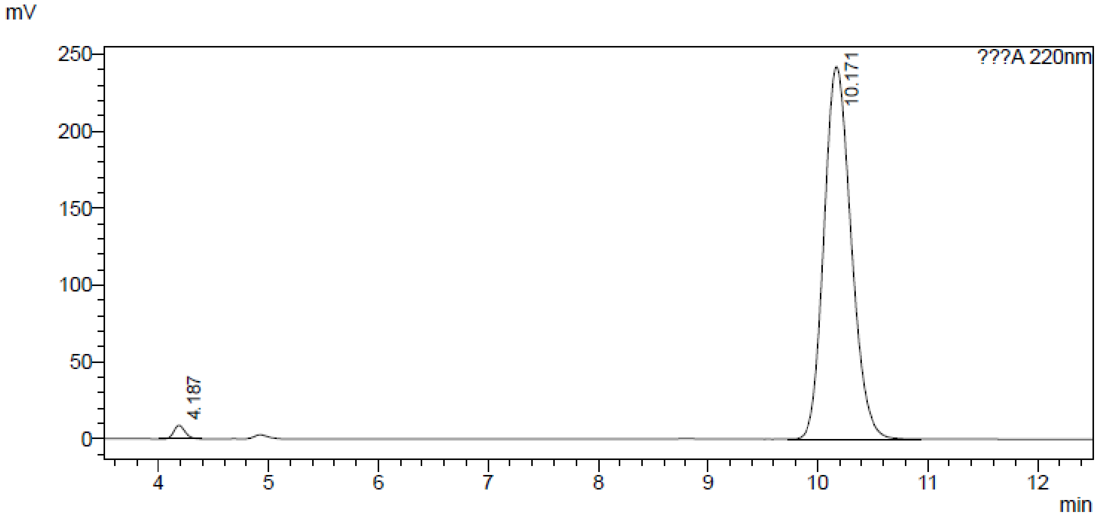

<Peak Table>

???A 220nm

Peak#	Ret. Time	Area	Height	Conc.	Unit	Mark	Name
1	4.187	59869	8635	1.424		M	
2	10.171	4144428	241757	98.576		M	
Total		4204297	250392				

Methyl (*S*)-2-phenyl-2-((*R*)-phenyl(propionyloxy)methyl)pent-4-enoate (**6c**)

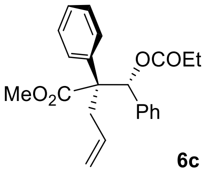

6c

<Chromatogram>

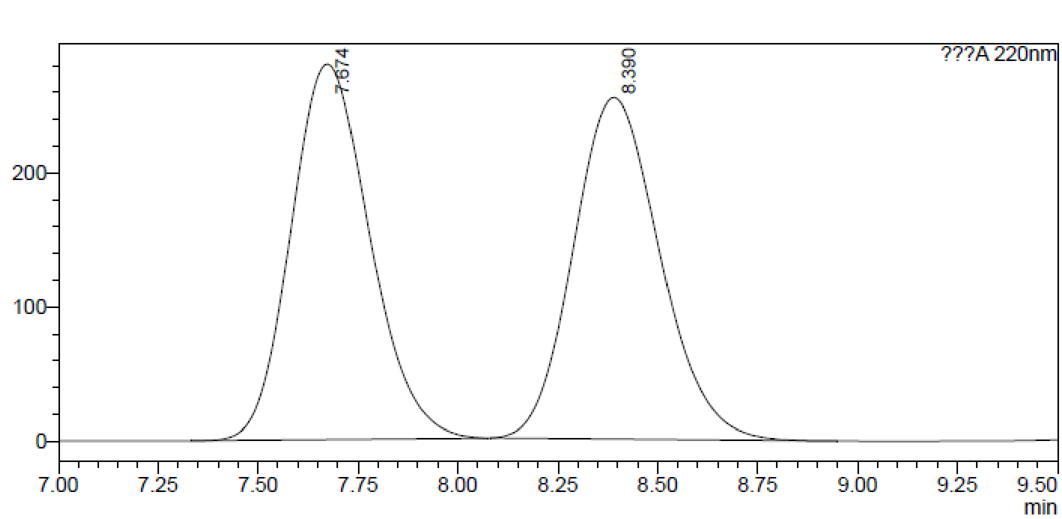

<Peak Table>

???A 220nm

Peak#	Ret. Time	Area	Height	Conc.	Unit	Mark	Name
1	7.674	3833186	279961	49.920		M	
2	8.390	3845457	254785	50.080		M	
Total		7678642	534747				

<Chromatogram>

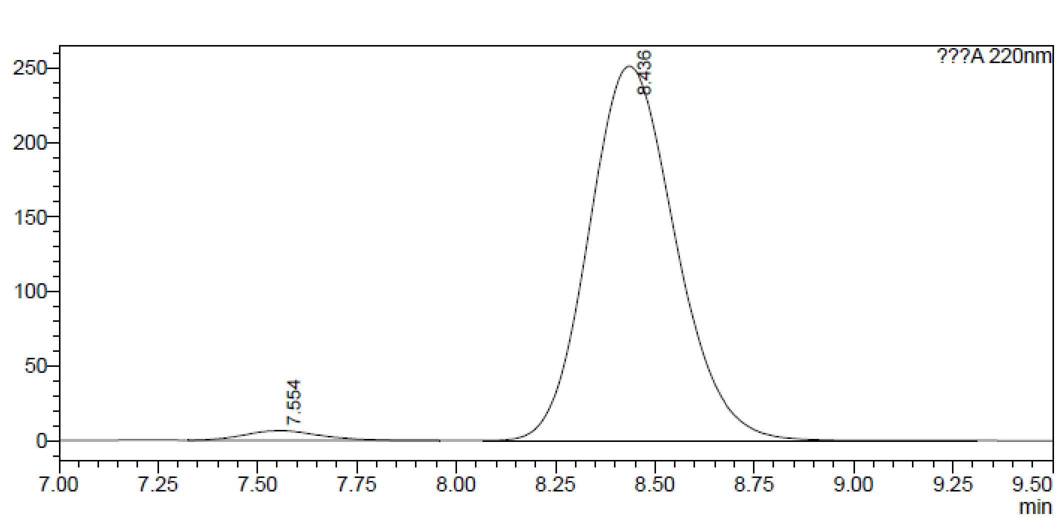

<Peak Table>

???A 220nm

Peak#	Ret. Time	Area	Height	Conc.	Unit	Mark	Name
1	7.554	82434	6428	2.126		M	
2	8.436	3795658	251058	97.874		M	
Total		3878093	257486				

Ethyl-(*R*)-2-((1-benzyl-1*H*-1,2,3-triazol-5-yl)methyl)-2-((*S*)-hydroxy(phenyl)methyl)pent-4-enoate (**5d**)

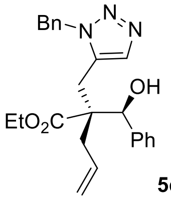

5d

<Chromatogram>

mV

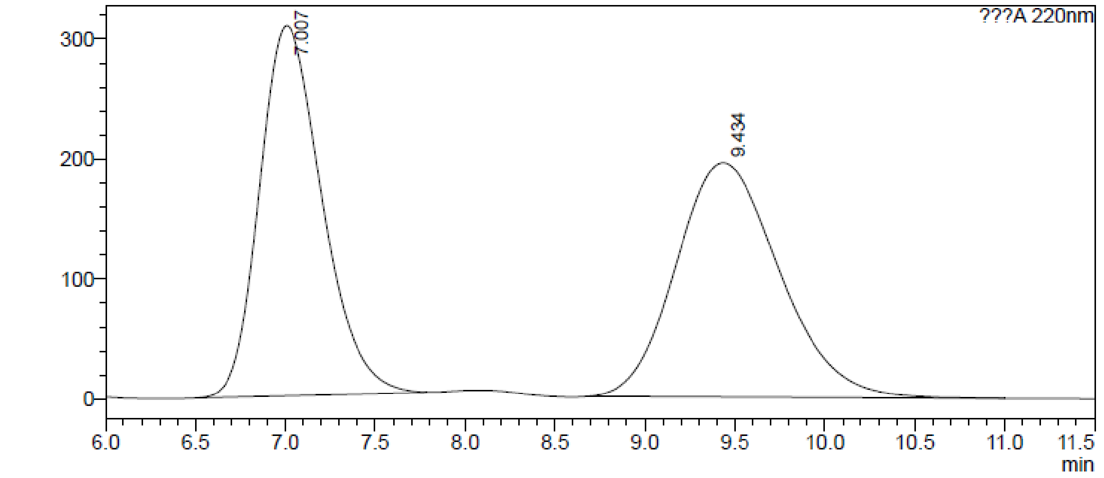

<Peak Table>

???A 220nm

Peak#	Ret. Time	Area	Height	Conc.	Unit	Mark	Name
1	7.007	7450308	308247	48.809		M	
2	9.434	7813881	195297	51.191		M	
Total		15264189	503543				

<Chromatogram>

mV

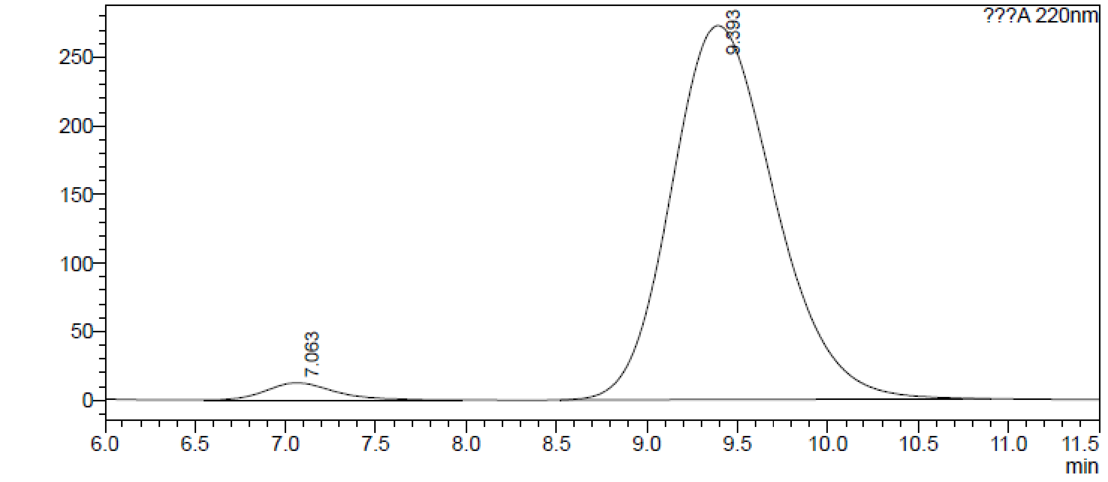

<Peak Table>

???A 220nm

Peak#	Ret. Time	Area	Height	Conc.	Unit	Mark	Name
1	7.063	326228	12419	2.910		M	
2	9.393	10884626	272923	97.090		M	
Total		11210854	285342				

Ethyl-(*S*)-2-((1-benzyl-1*H*-1,2,3-triazol-5-yl)methyl)-2-((*R*)-phenyl(propionyloxy)methyl)pent-4-enoate (**6d**)

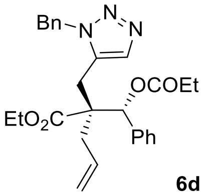

6d

<Chromatogram>

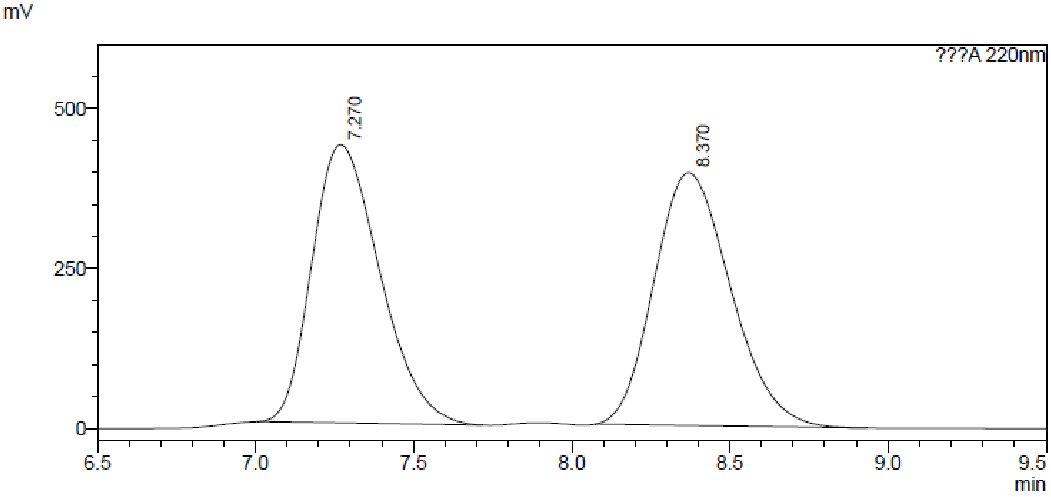

<Peak Table>

???A 220nm

Peak#	Ret. Time	Area	Height	Conc.	Unit	Mark	Name
1	7.270	6515560	435089	49.403		M	
2	8.370	6673032	395192	50.597		M	
Total		13188592	830281				

<Chromatogram>

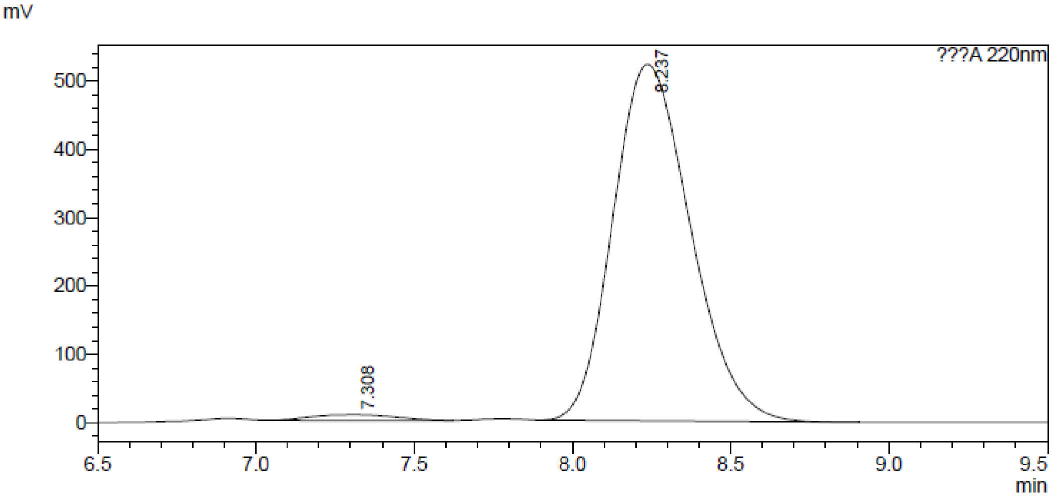

<Peak Table>

???A 220nm

Peak#	Ret. Time	Area	Height	Conc.	Unit	Mark	Name
1	7.308	148886	8098	1.647		M	
2	8.237	8889092	521168	98.353		M	
Total		9037978	529266				

(*R*)-2-((*S*)-(4-chlorophenyl)(hydroxy)methyl)-2-(naphthalen-2-ylmethyl)pent-4-enoate (**5e**)

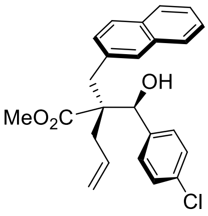

5e

<Chromatogram>

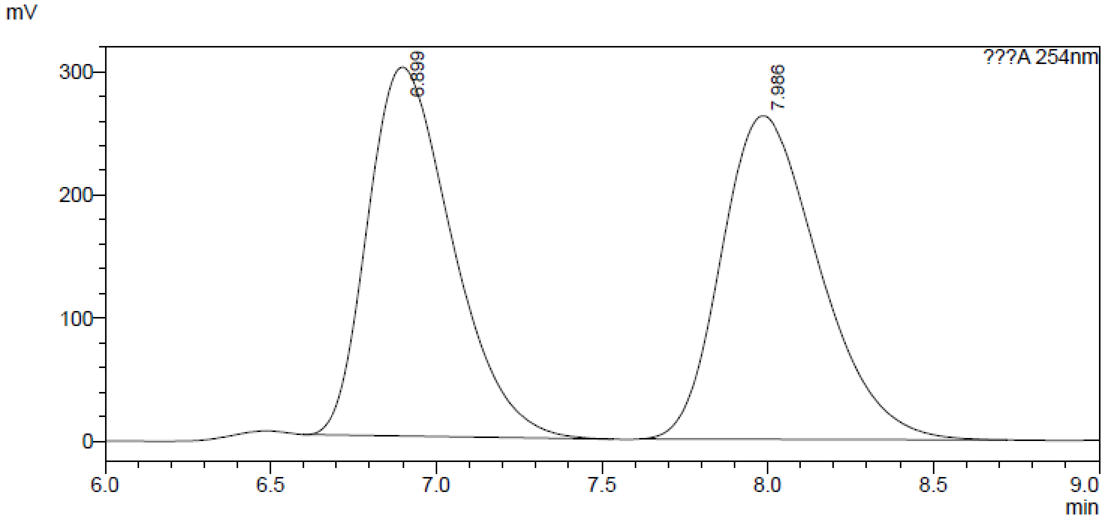

<Peak Table>

???A 254nm

Peak#	Ret. Time	Area	Height	Conc.	Unit	Mark	Name
1	6.899	5306518	299469	49.483		M	
2	7.986	5417366	262698	50.517		M	
Total		10723883	562167				

<Chromatogram>

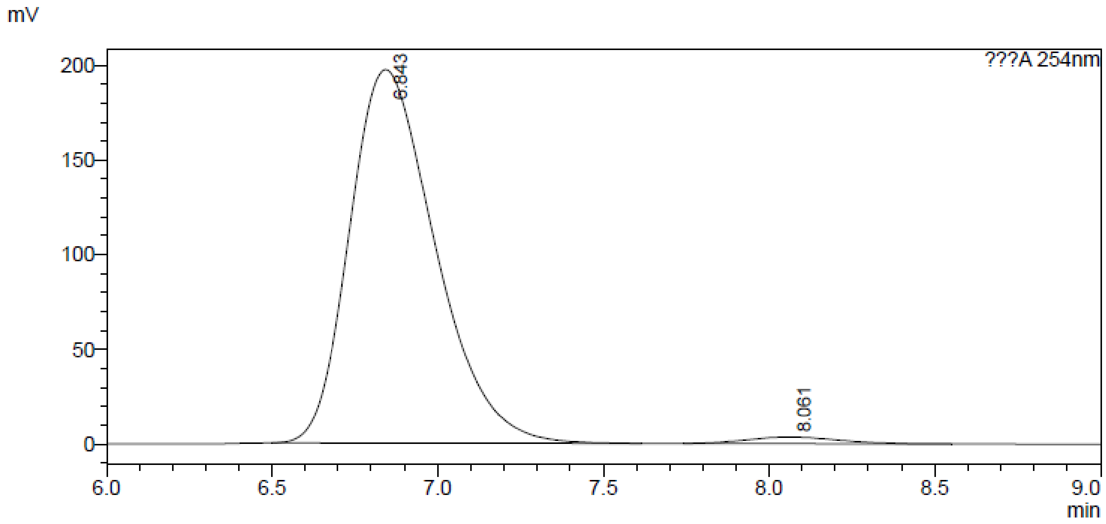

<Peak Table>

???A 254nm

Peak#	Ret. Time	Area	Height	Conc.	Unit	Mark	Name
1	6.843	3547341	197068	98.141		M	
2	8.061	67183	3480	1.859		M	
Total		3614524	200548				

(*S*)-2-((*R*)-(4-chlorophenyl)(propionyloxy)methyl)-2-(naphthalen-2-ylmethyl)pent-4-enoate (**6e**)

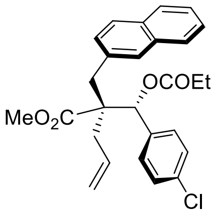

6e

<Chromatogram>

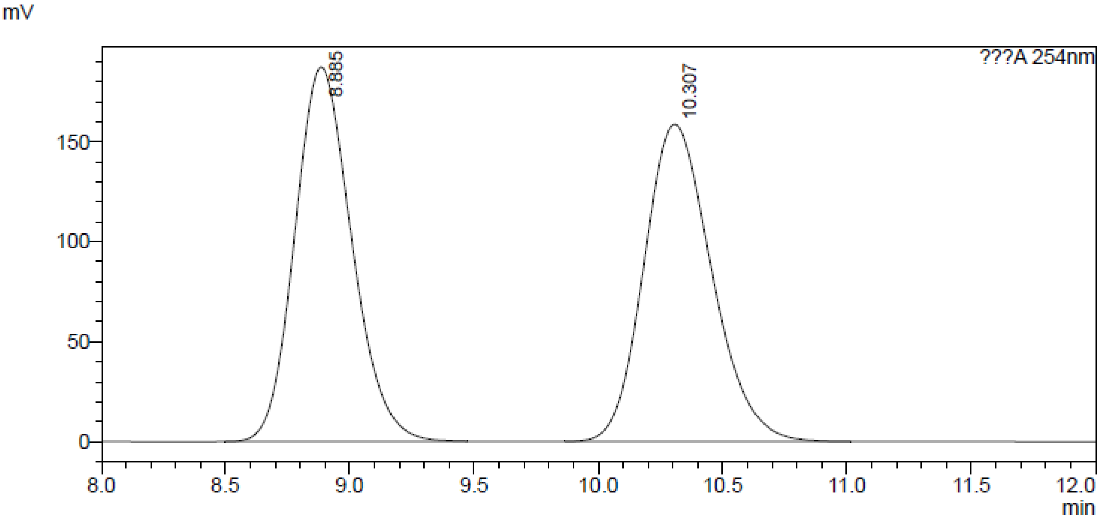

<Peak Table>

???A 254nm

Peak#	Ret. Time	Area	Height	Conc.	Unit	Mark	Name
1	8.885	3005525	187198	49.985		M	
2	10.307	3007369	158705	50.015		M	
Total		6012894	345903				

<Chromatogram>

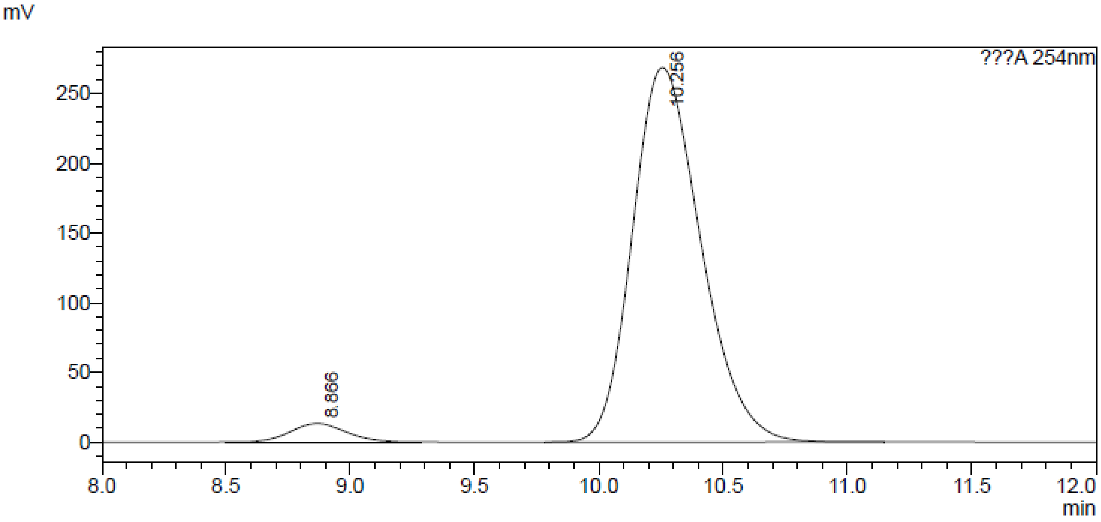

<Peak Table>

???A 254nm

Peak#	Ret. Time	Area	Height	Conc.	Unit	Mark	Name
1	8.866	209636	13279	3.923		M	
2	10.256	5134798	268800	96.077		M	
Total		5344434	282080				

Ethyl (*R*)-2-allyl-2-((*S*)-hydroxy(4-methoxyphenyl)methyl)-4-methylpent-4-enoate (5f)

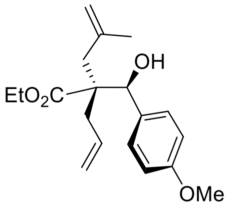

5f

<Chromatogram>

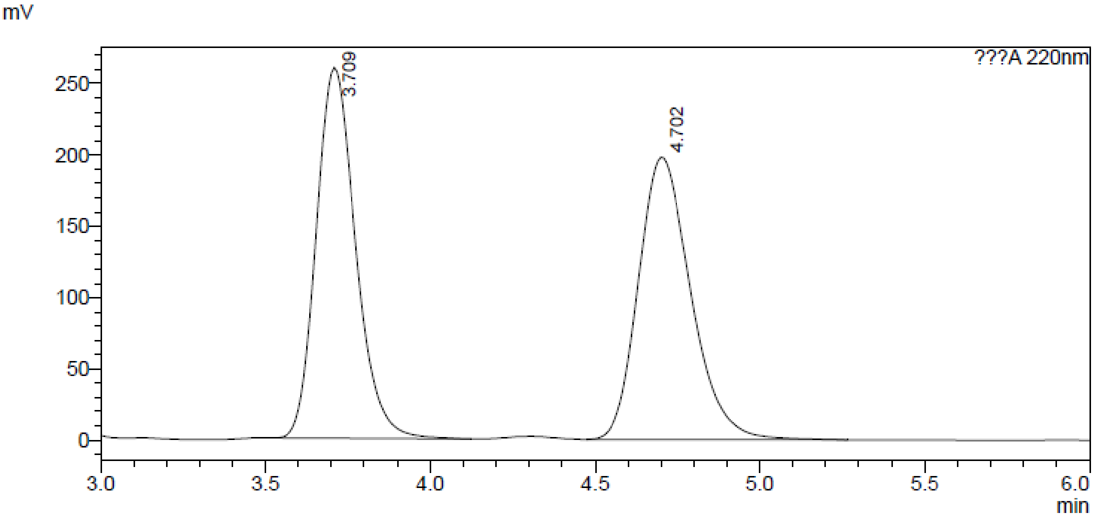

<Peak Table>

???A 220nm

Peak#	Ret. Time	Area	Height	Conc.	Unit	Mark	Name
1	3.709	2157753	259841	49.680		M	
2	4.702	2185574	197724	50.320		M	
Total		4343328	457566				

<Chromatogram>

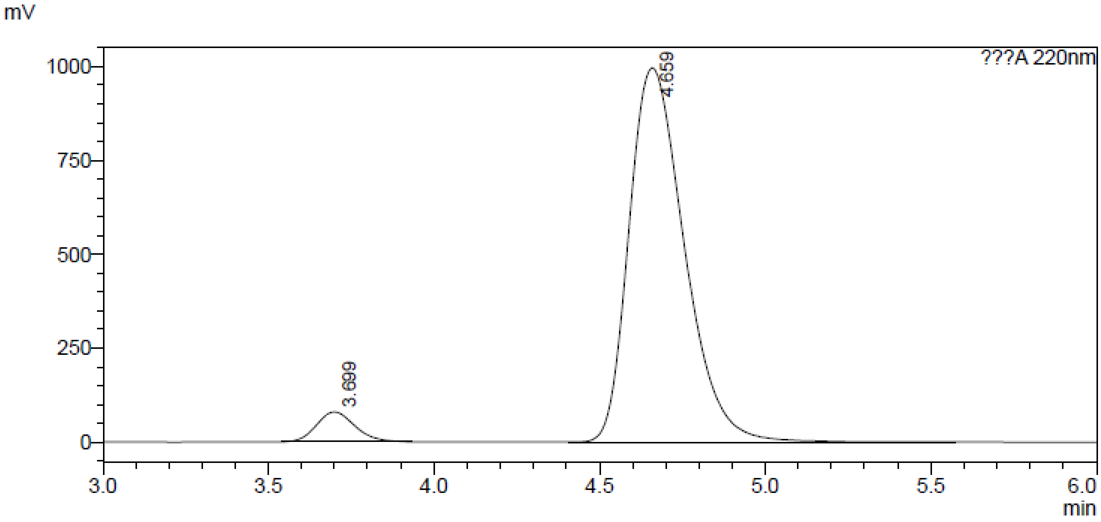

<Peak Table>

???A 220nm

Peak#	Ret. Time	Area	Height	Conc.	Unit	Mark	Name
1	3.699	639909	79215	5.255		M	
2	4.659	11536858	997052	94.745		M	
Total		12176767	1076266				

Ethyl (*S*)-2-allyl-2-((*R*)-(4-methoxyphenyl)(propionyloxy)methyl)-4-methylpent-4-enoate (**6f**)

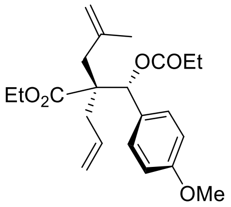

6f

<Chromatogram>

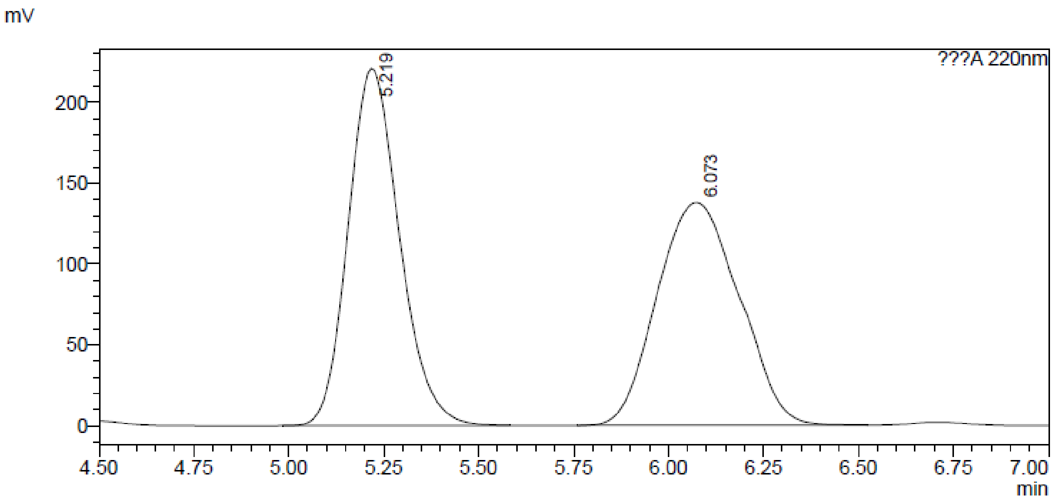

<Peak Table>

???A 220nm

Peak#	Ret. Time	Area	Height	Conc.	Unit	Mark	Name
1	5.219	2077952	220772	50.045		M	
2	6.073	2074212	137711	49.955		M	
Total		4152164	358483				

<Chromatogram>

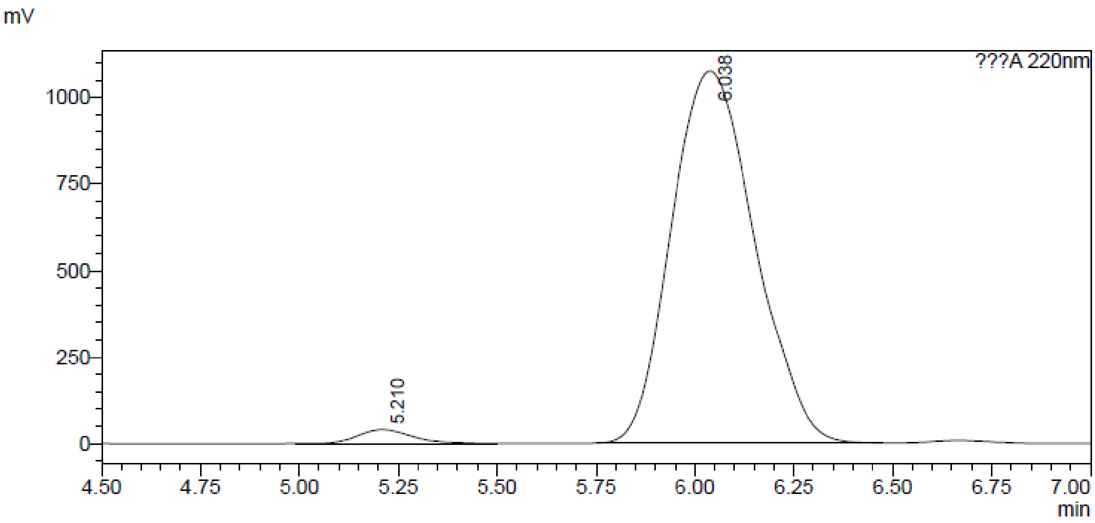

<Peak Table>

???A 220nm

Peak#	Ret. Time	Area	Height	Conc.	Unit	Mark	Name
1	5.210	377515	40038	2.340		M	
2	6.038	15757819	1073856	97.660		M	
Total		16135334	1113893				

Ethyl (*R*)-2-((*S*)-hydroxy(phenyl)methyl)-2-methylpent-4-ynoate (**5g**)

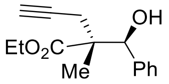

5g

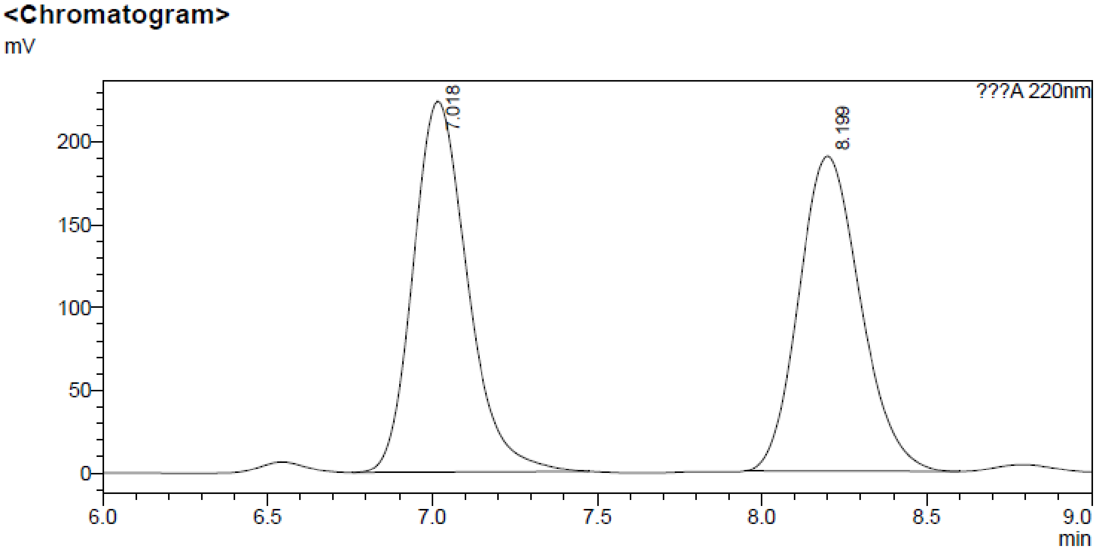

<Peak Table>

???A 220nm

Peak#	Ret. Time	Area	Height	Conc.	Unit	Mark	Name
1	7.018	2578458	224302	50.923		M	
2	8.199	2485034	190608	49.077		M	
Total		5063492	414910				

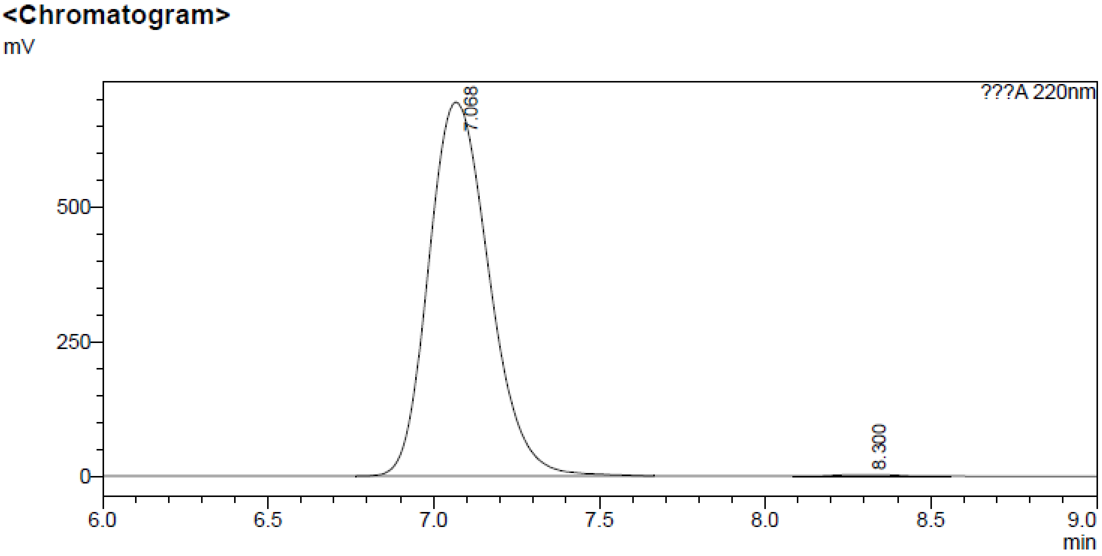

<Peak Table>

???A 220nm

Peak#	Ret. Time	Area	Height	Conc.	Unit	Mark	Name
1	7.068	8797718	695693	99.608		M	
2	8.300	34665	2868	0.392		M	
Total		8832383	698561				

Ethyl (*S*)-2-methyl-2-((*R*)-phenyl(propionyloxy)methyl)pent-4-ynoate (**6g**)

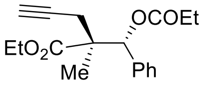

6g

<Chromatogram>

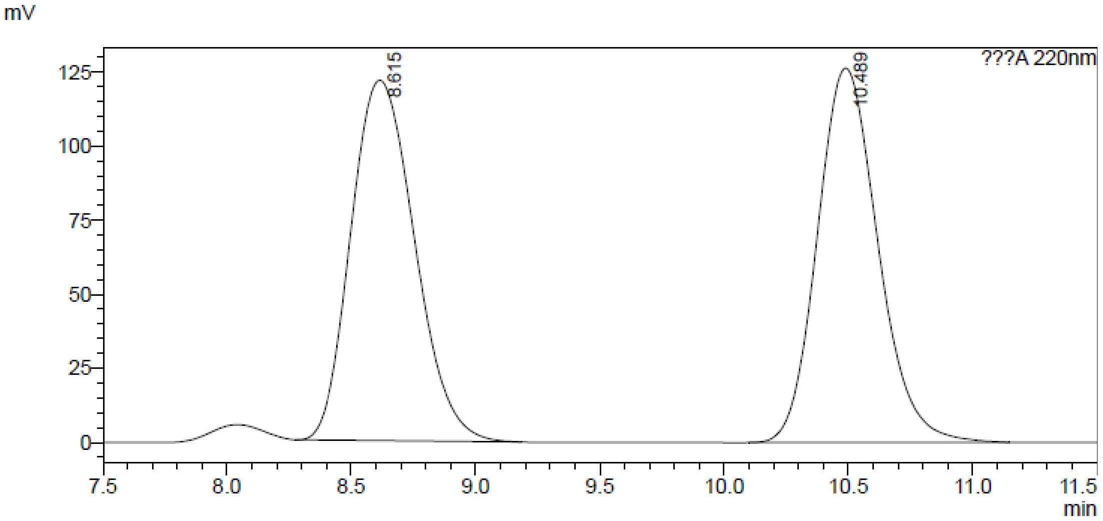

<Peak Table>

???A 220nm

Peak#	Ret. Time	Area	Height	Conc.	Unit	Mark	Name
1	8.615	2168875	121493	50.389		M	
2	10.489	2135357	126312	49.611		M	
Total		4304233	247805				

<Chromatogram>

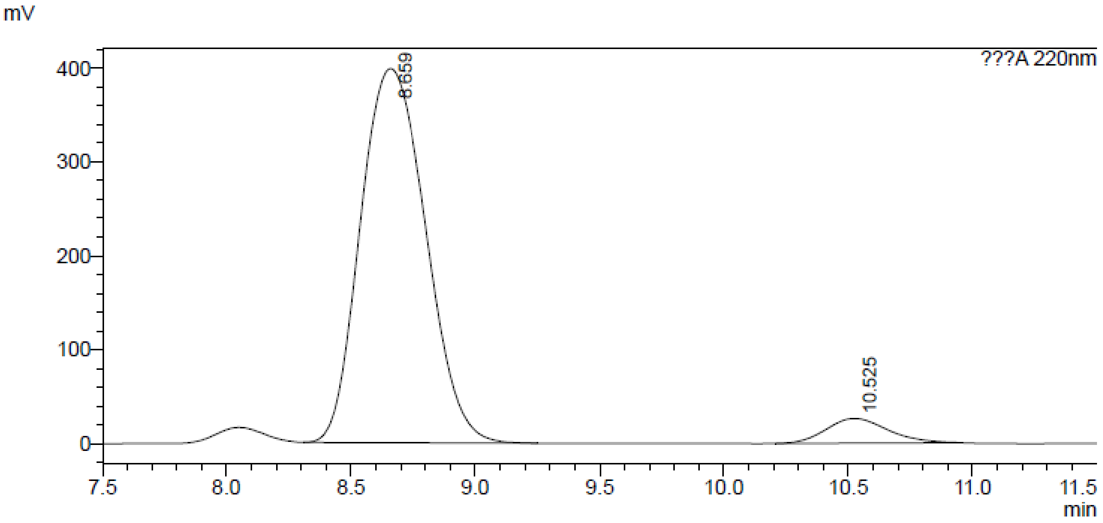

<Peak Table>

???A 220nm

Peak#	Ret. Time	Area	Height	Conc.	Unit	Mark	Name
1	8.659	7291124	398491	94.131		M	
2	10.525	454625	26362	5.869		M	
Total		7745749	424853				

1-Ethyl 6-methyl (*R*)-2-((*S*)-hydroxy(phenyl)methyl)-2-methylhexanedioate (**5h**)

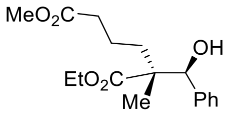

5h

<Chromatogram>

mV

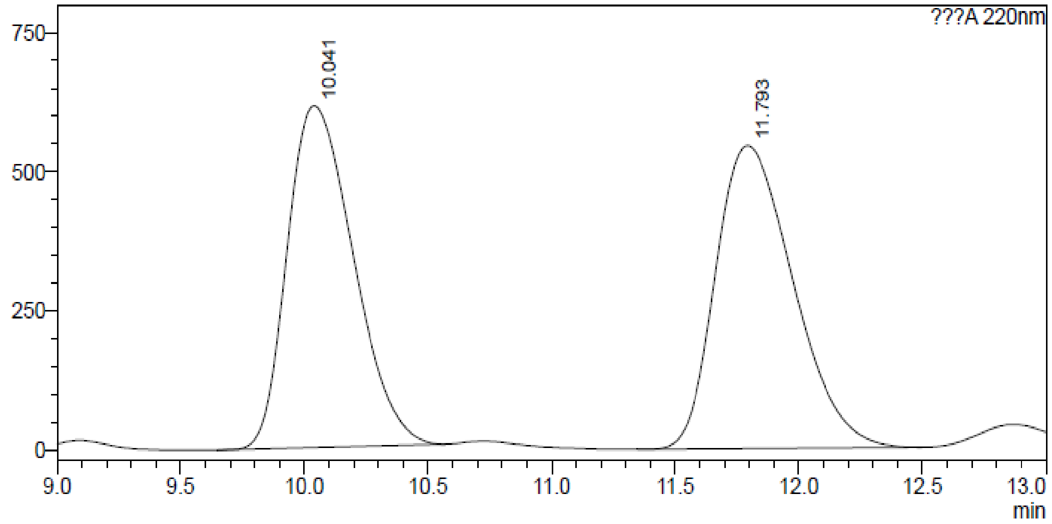

<Peak Table>

???A 220nm

Peak#	Ret. Time	Area	Height	Conc.	Unit	Mark	Name
1	10.041	11425641	613988	48.945		M	
2	11.793	11917992	544771	51.055		M	
Total		23343633	1158759				

<Chromatogram>

mV

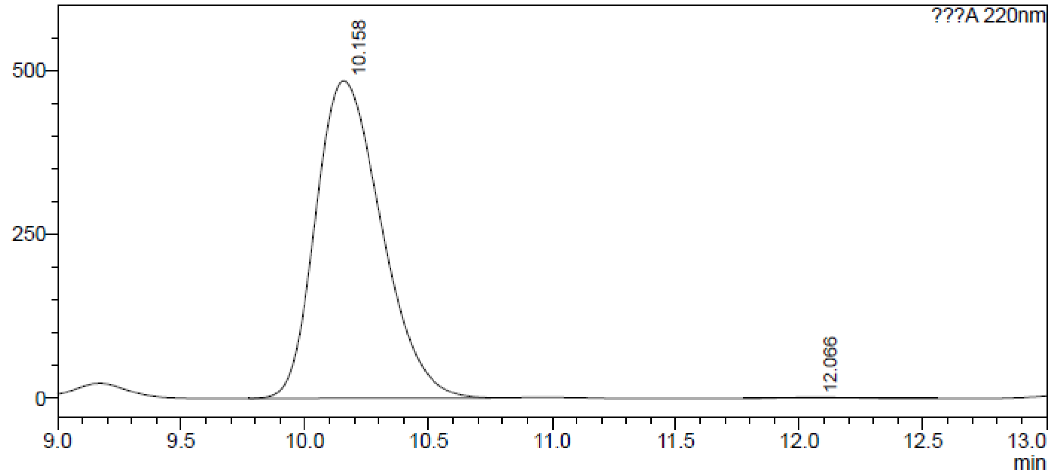

<Peak Table>

???A 220nm

Peak#	Ret. Time	Area	Height	Conc.	Unit	Mark	Name
1	10.158	8968017	484054	99.599		M	
2	12.066	36096	1888	0.401		M	
Total		9004114	485941				

1-Ethyl 6-methyl (*S*)-2-methyl-2-((*R*)-phenyl(propionyloxy)methyl)hexanedioate (**6h**)

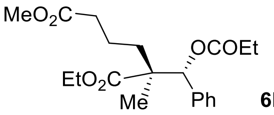

6h

<Chromatogram>

mV

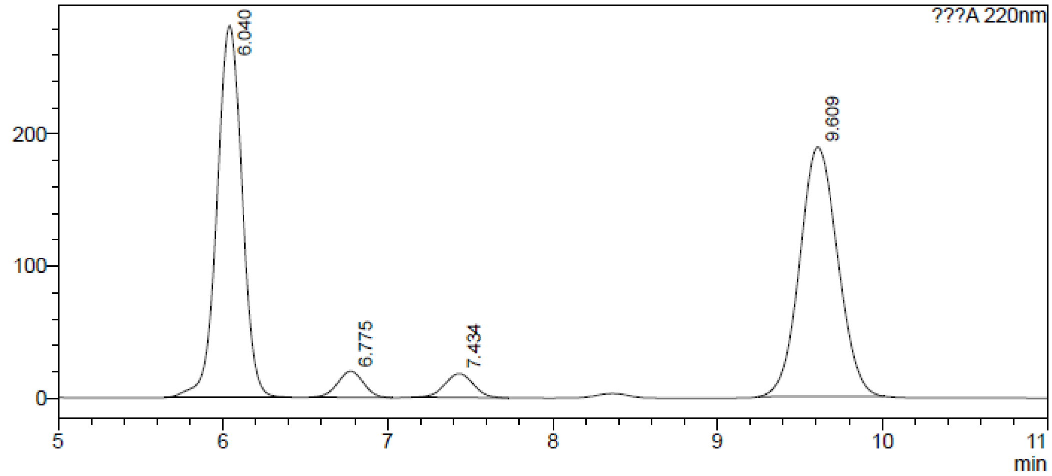

<Peak Table>

???A 220nm

Peak#	Ret. Time	Area	Height	Conc.	Unit	Mark	Name
1	6.040	2982829	281641	46.263		M	
2	6.775	213025	19657	3.304		M	
3	7.434	212788	17887	3.300		M	
4	9.609	3038967	188931	47.133		M	
Total		6447610	508116				

<Chromatogram>

mV

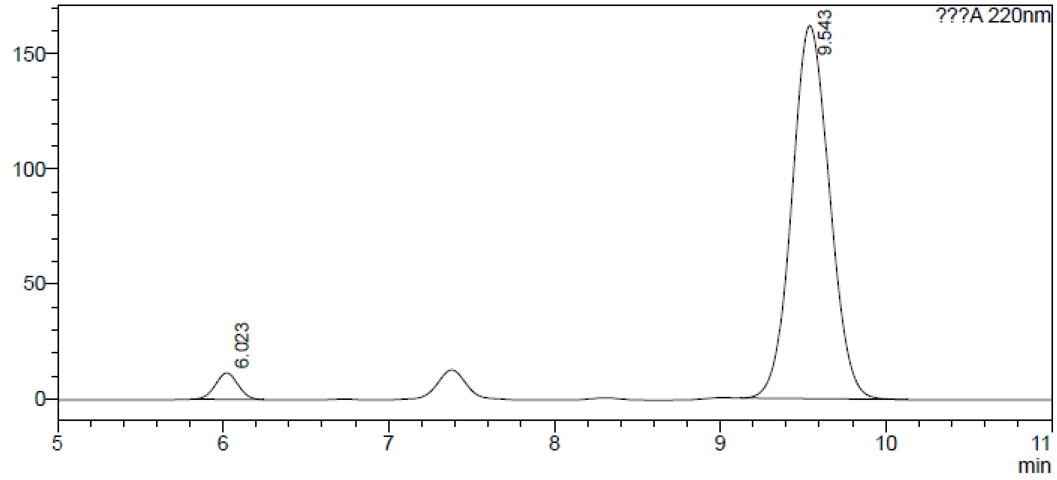

<Peak Table>

???A 220nm

Peak#	Ret. Time	Area	Height	Conc.	Unit	Mark	Name
1	6.023	108575	11458	4.077		M	
2	9.543	2554714	161982	95.923		M	
Total		2663289	173440				

Ethyl (*R*)-2-((*S*)-hydroxy(phenyl)methyl)-2-methyl-3-methylenenonanoate (**5i**)

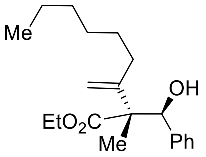

5i

<Chromatogram>

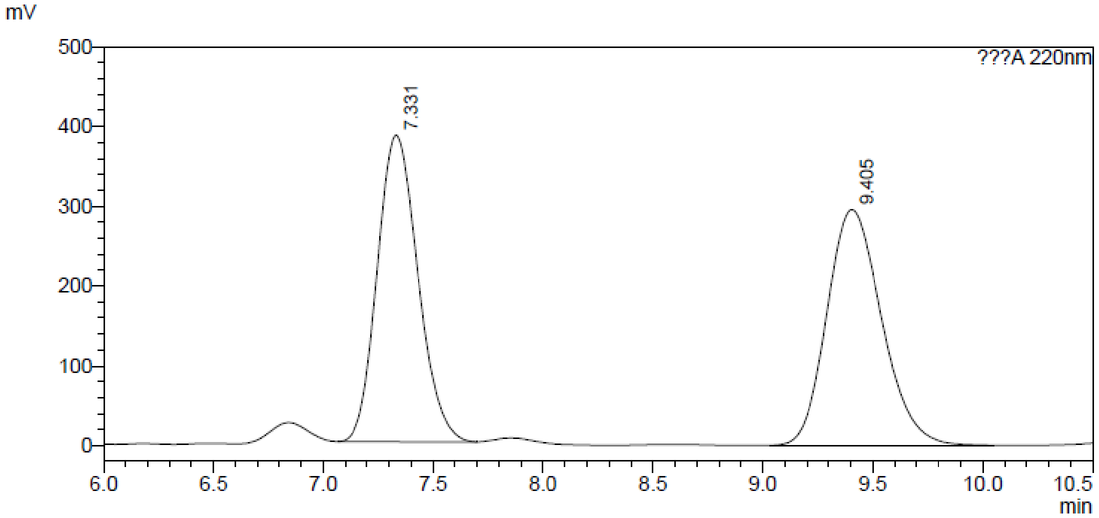

<Peak Table>

???A 220nm

Peak#	Ret. Time	Area	Height	Conc.	Unit	Mark	Name
1	7.331	4950097	384176	49.237		M	
2	9.405	5103508	295102	50.763		M	
Total		10053606	679278				

<Chromatogram>

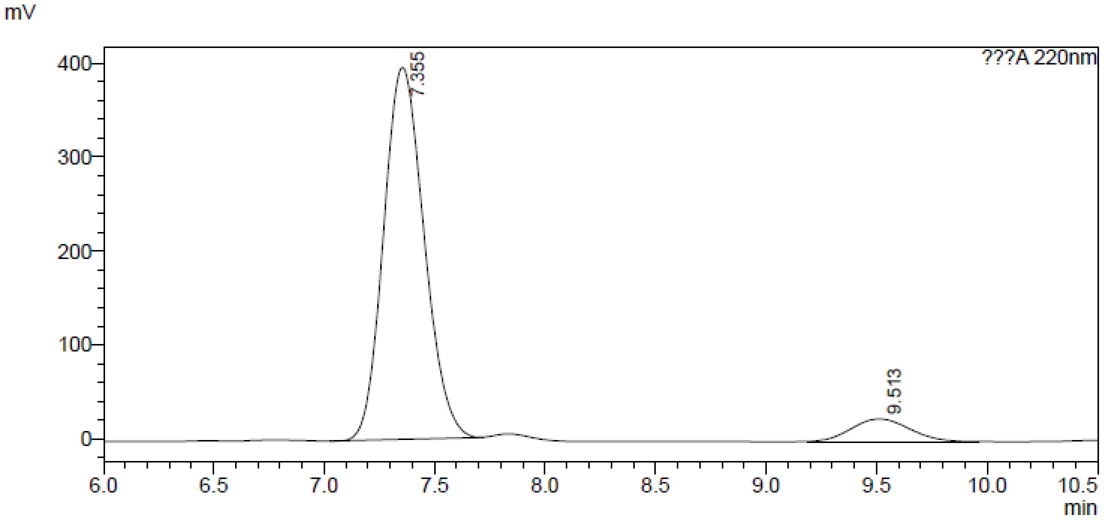

<Peak Table>

???A 220nm

Peak#	Ret. Time	Area	Height	Conc.	Unit	Mark	Name
1	7.355	5033237	395737	92.009		M	
2	9.513	437111	23965	7.991		M	
Total		5470348	419702				

Ethyl (*S*)-2-methyl-3-methylene-2-((*R*)-phenyl(propionyloxy)methyl)nonanoate (**6i**)

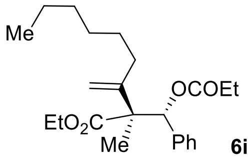

6i

<Chromatogram>

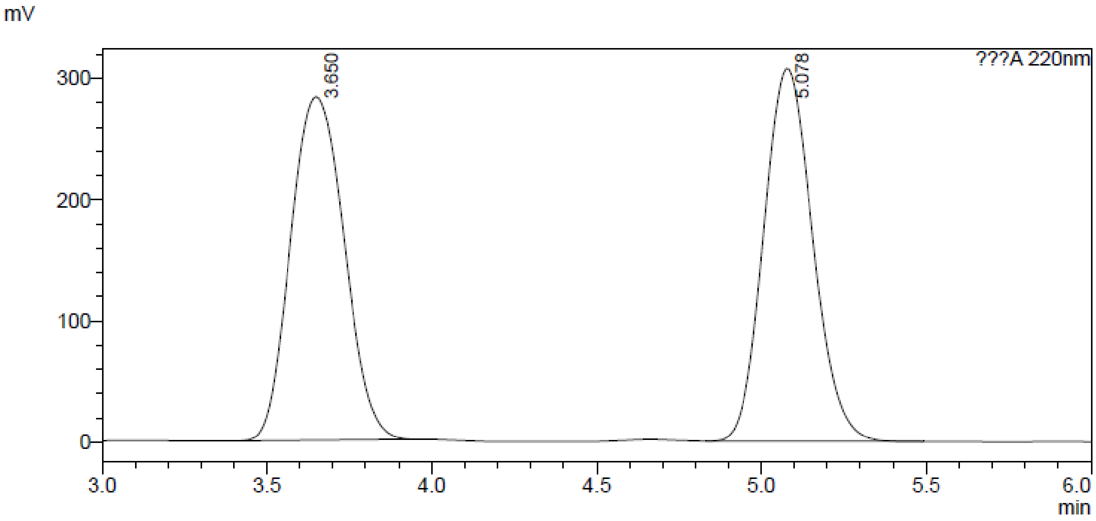

<Peak Table>

???A 220nm

Peak#	Ret. Time	Area	Height	Conc.	Unit	Mark	Name
1	3.650	3199356	283103	50.127		M	
2	5.078	3183187	307042	49.873		M	
Total		6382543	590145				

<Chromatogram>

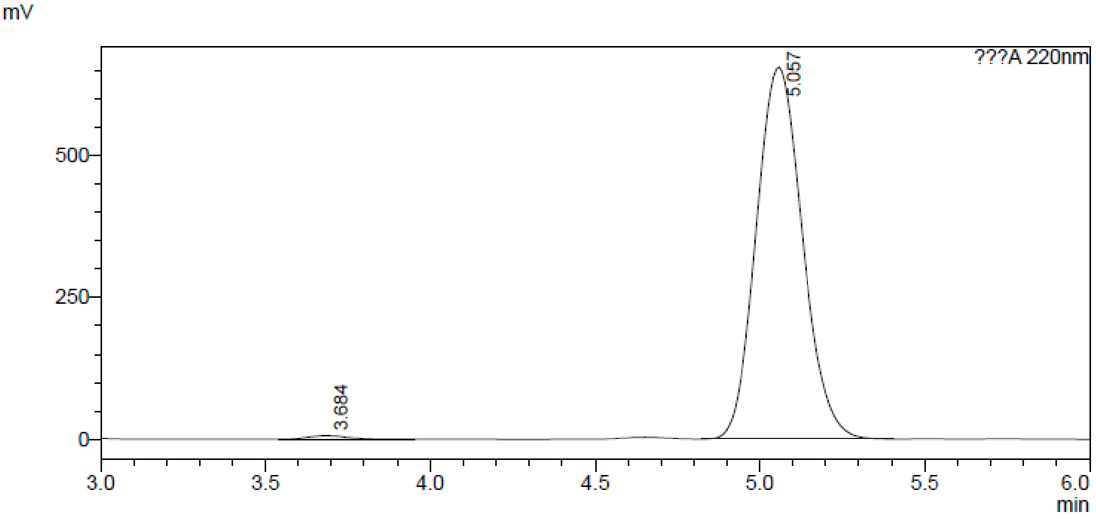

<Peak Table>

???A 220nm

Peak#	Ret. Time	Area	Height	Conc.	Unit	Mark	Name
1	3.684	53890	6603	0.839		M	
2	5.057	6367207	654033	99.161		M	
Total		6421098	660636				

Ethyl (*R*)-2-benzyl-2-((*S*)-hydroxy(naphthalen-2-yl)methyl)pent-4-enoate (**5j**)

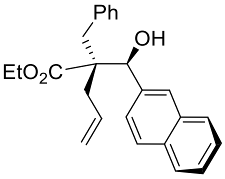

5j

<Chromatogram>

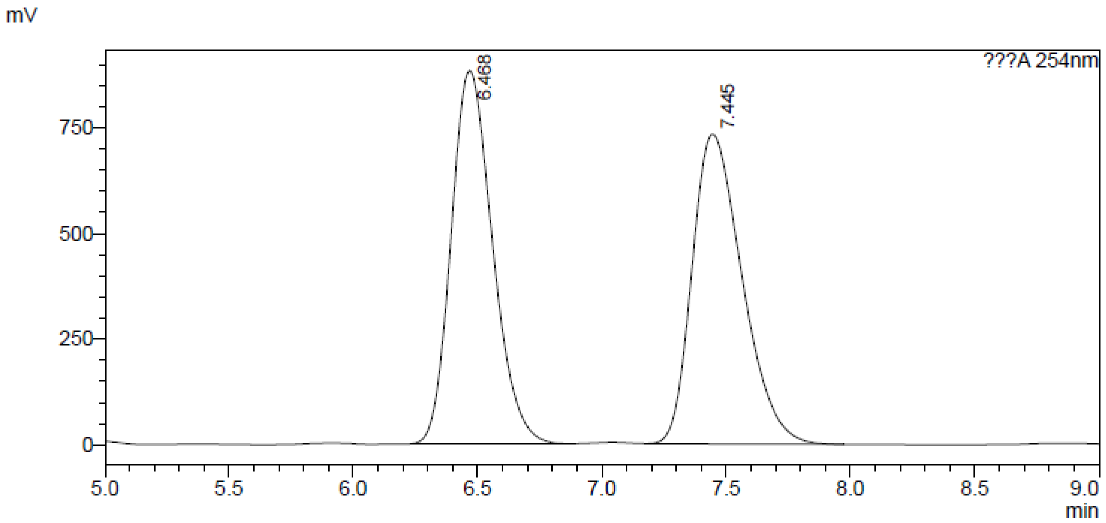

<Peak Table>

???A 254nm

Peak#	Ret. Time	Area	Height	Conc.	Unit	Mark	Name
1	6.468	10217691	883608	49.892		M	
2	7.445	10261816	732155	50.108		M	
Total		20479507	1615763				

<Chromatogram>

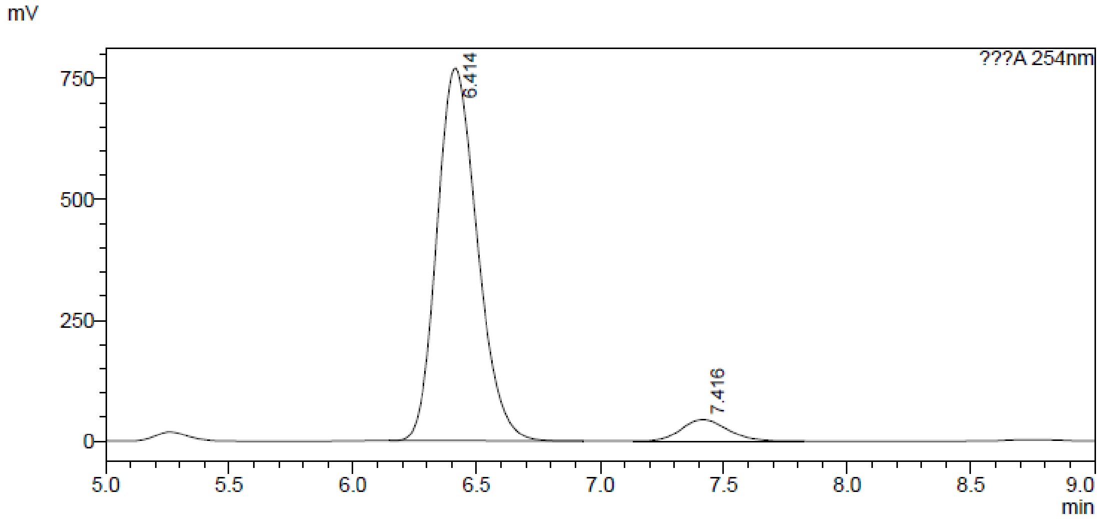

<Peak Table>

???A 254nm

Peak#	Ret. Time	Area	Height	Conc.	Unit	Mark	Name
1	6.414	8731783	770210	93.656		M	
2	7.416	591465	45289	6.344		M	
Total		9323248	815499				

Ethyl (*S*)-2-benzyl-2-((*R*)-naphthalen-2-yl(propionyloxy)methyl)pent-4-enoate (**6j**)

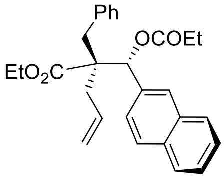

6j

<Chromatogram>

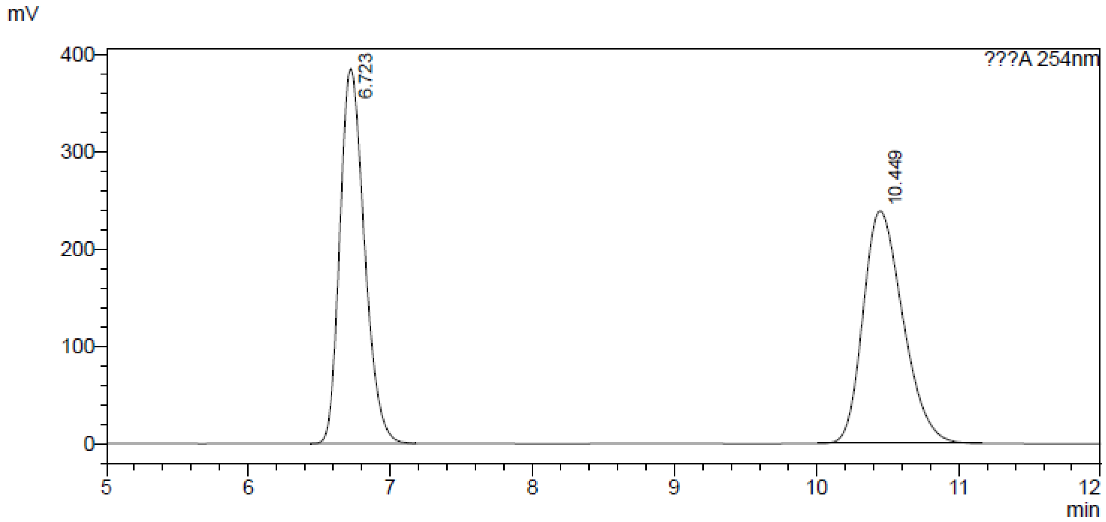

<Peak Table>

???A 254nm

Peak#	Ret. Time	Area	Height	Conc.	Unit	Mark	Name
1	6.723	4633343	384511	49.909		M	
2	10.449	4650203	238663	50.091		M	
Total		9283546	623175				

<Chromatogram>

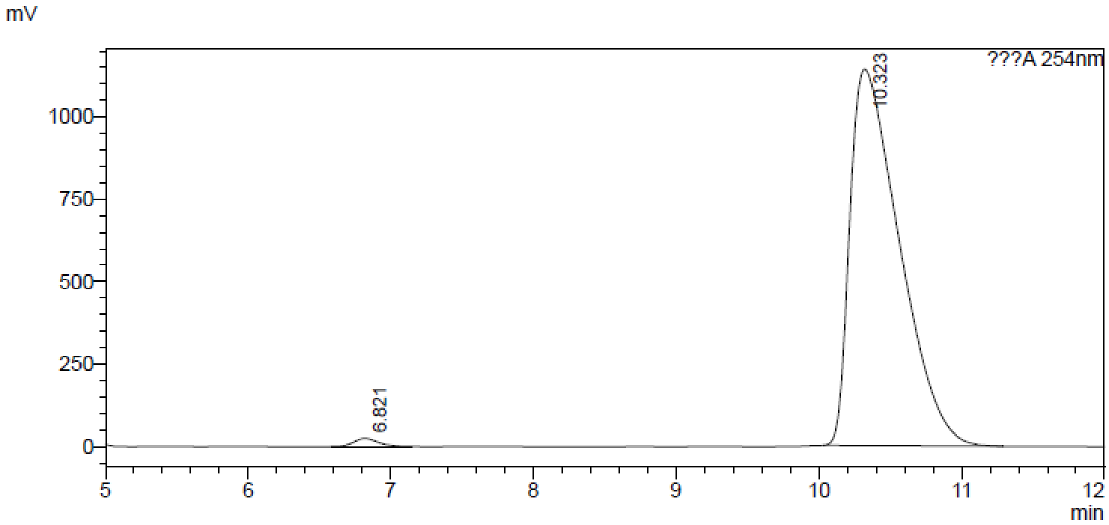

<Peak Table>

???A 254nm

Peak#	Ret. Time	Area	Height	Conc.	Unit	Mark	Name
1	6.821	298946	24737	1.081		M	
2	10.323	27344909	1141370	98.919		M	
Total		27643855	1166107				

Ethyl (*R*)-2-((*S*)-hydroxy(naphthalen-2-yl)methyl)-2-methylpent-4-enoate (**5k**)

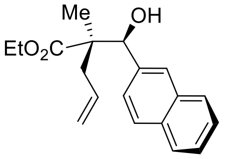

5k

<Chromatogram>

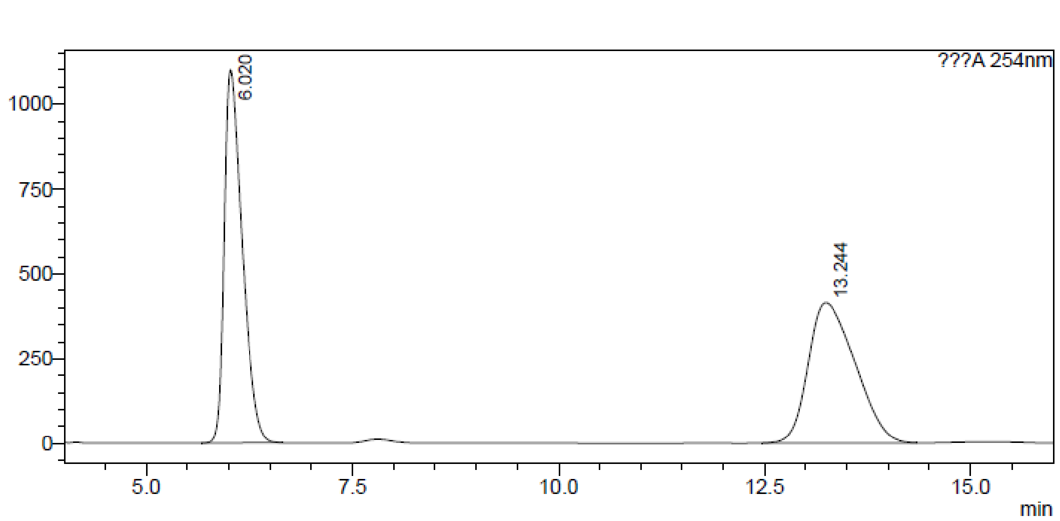

<Peak Table>

???A 254nm

Peak#	Ret. Time	Area	Height	Conc.	Unit	Mark	Name
1	6.020	16705579	1098693	50.540		M	
2	13.244	16348500	413195	49.460		M	
Total		33054079	1511888				

<Chromatogram>

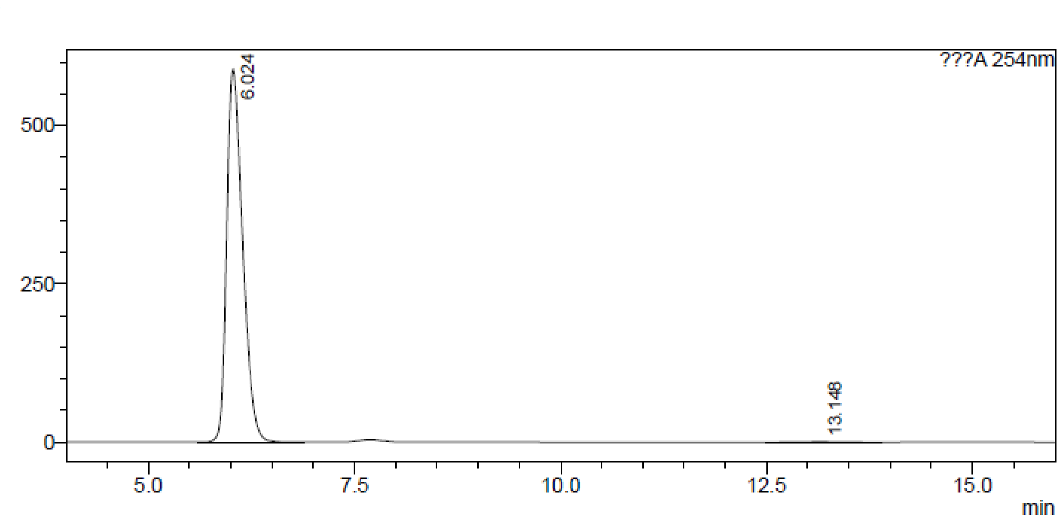

<Peak Table>

???A 254nm

Peak#	Ret. Time	Area	Height	Conc.	Unit	Mark	Name
1	6.024	7917917	588121	99.601		M	
2	13.148	31742	1019	0.399		M	
Total		7949659	589141				

Ethyl (*S*)-2-methyl-2-((*R*)-naphthalen-2-yl(propionyloxy)methyl)pent-4-enoate (**6k**)

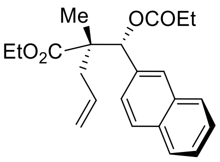

6k

<Chromatogram>

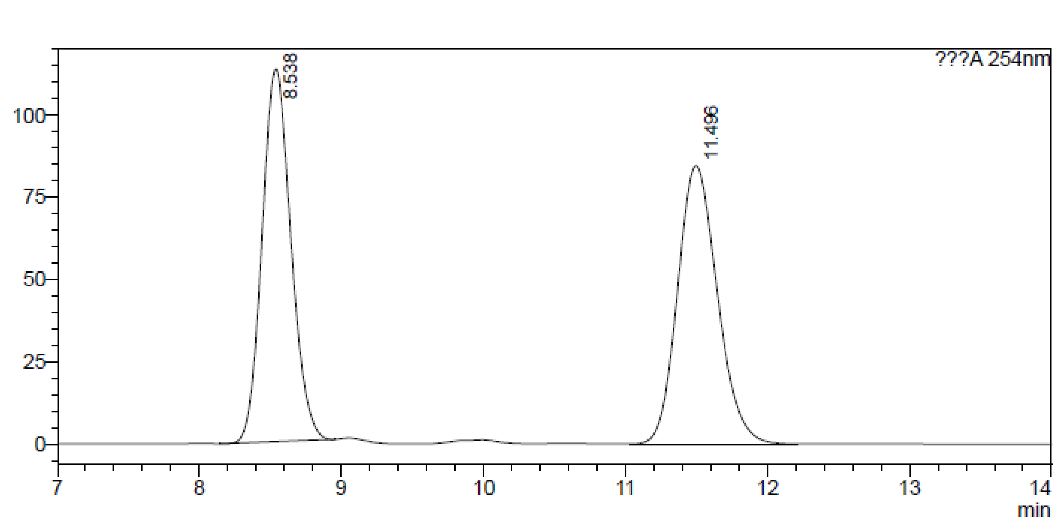

<Peak Table>

???A 254nm

Peak#	Ret. Time	Area	Height	Conc.	Unit	Mark	Name
1	8.538	1616171	113273	49.472		M	
2	11.496	1650642	84441	50.528		M	
Total		3266813	197715				

<Chromatogram>

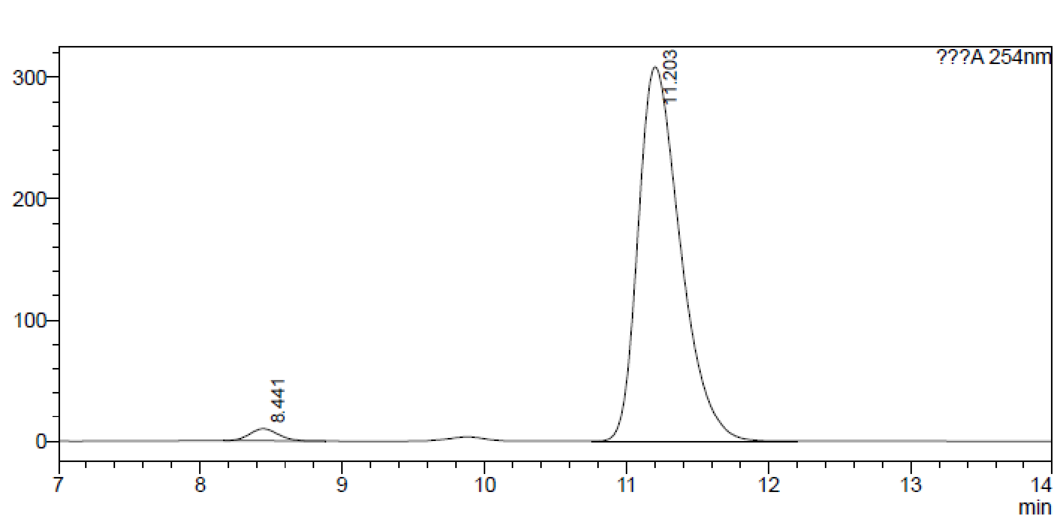

<Peak Table>

???A 254nm

Peak#	Ret. Time	Area	Height	Conc.	Unit	Mark	Name
1	8.441	138857	9915	2.133		M	
2	11.203	6370054	308336	97.867		M	
Total		6508911	318251				

Ethyl (*S*)-2-((*S*)-hydroxy(naphthalen-2-yl)methyl)-2-methylpent-4-enoate (**5l**)

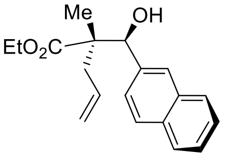

5l

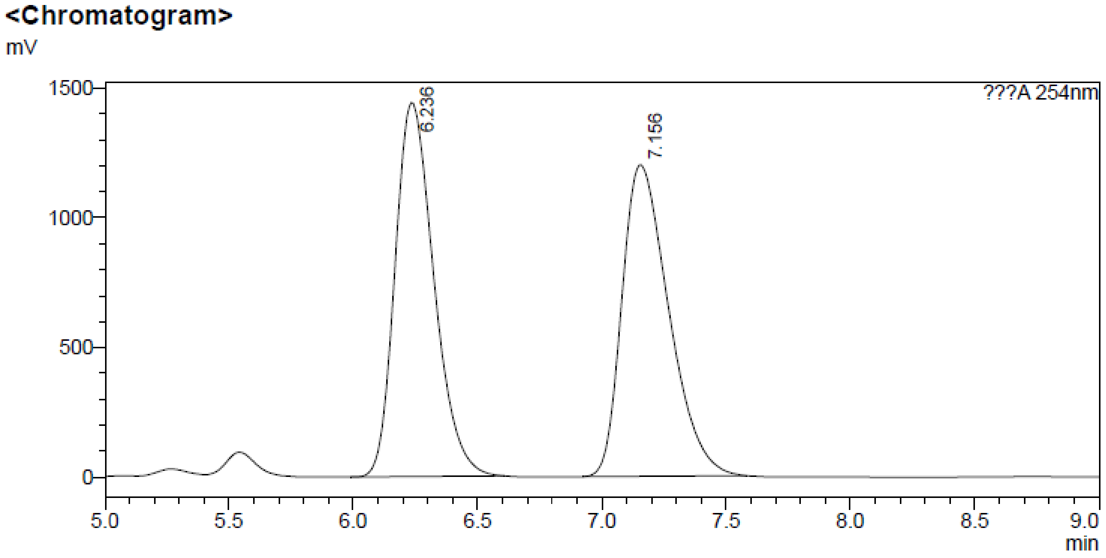

<Peak Table>

???A 254nm

Peak#	Ret. Time	Area	Height	Conc.	Unit	Mark	Name
1	6.236	15651980	1442442	49.941		M	
2	7.156	15688918	1200048	50.059		M	
Total		31340898	2642490				

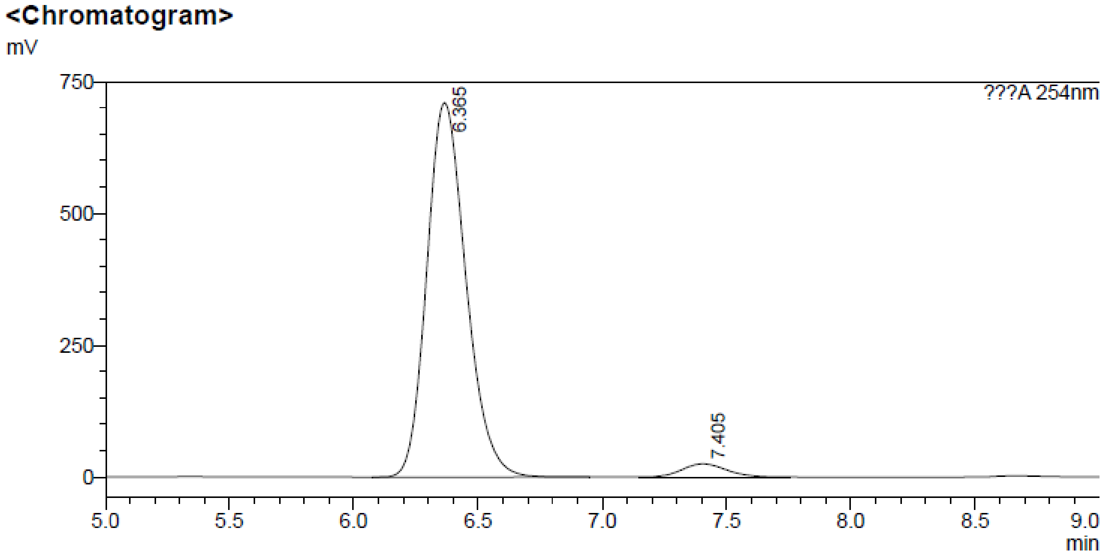

<Peak Table>

???A 254nm

Peak#	Ret. Time	Area	Height	Conc.	Unit	Mark	Name
1	6.365	7770687	710166	96.132		M	
2	7.405	312623	25046	3.868		M	
Total		8083310	735211				

Ethyl (*R*)-2-methyl-2-((*R*)-naphthalen-2-yl(propionyloxy)methyl)pent-4-enoate (**6l**)

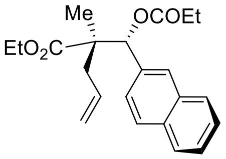

6l

<Chromatogram>

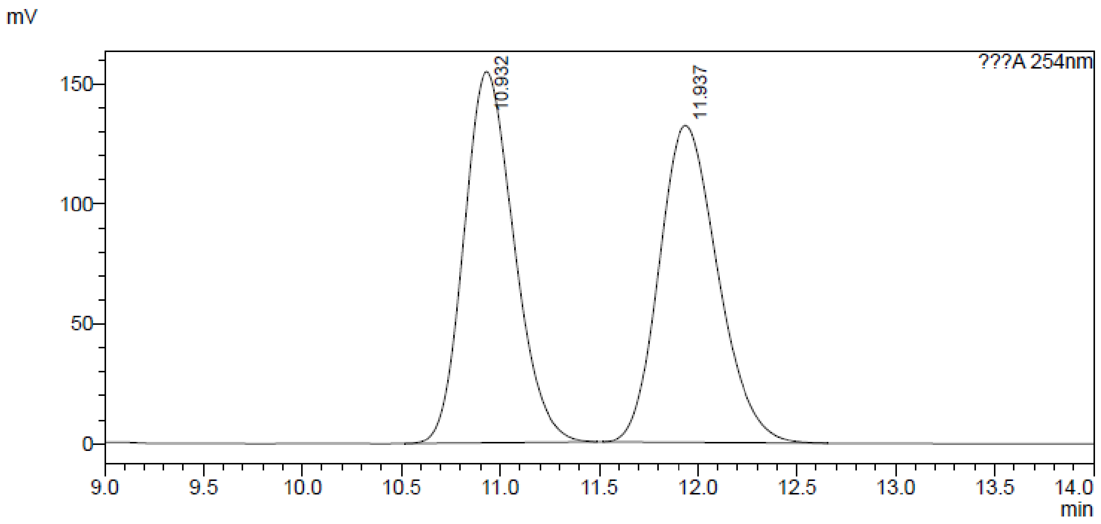

<Peak Table>

???A 254nm

Peak#	Ret. Time	Area	Height	Conc.	Unit	Mark	Name
1	10.932	2735058	154724	50.029		M	
2	11.937	2731888	132025	49.971		M	
Total		5466945	286749				

<Chromatogram>

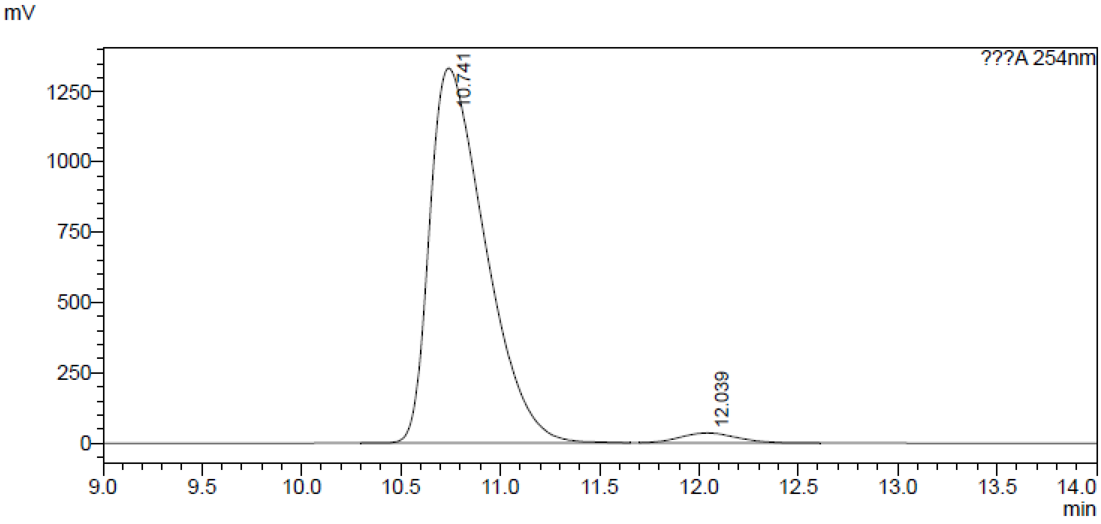

<Peak Table>

???A 254nm

Peak#	Ret. Time	Area	Height	Conc.	Unit	Mark	Name
1	10.741	25877531	1333721	97.439		M	
2	12.039	680210	35012	2.561		M	
Total		26557741	1368734				

(*S*)-Methyl 2-benzyl-2-((*S*)-hydroxy(naphthalen-2-yl)methyl)pent-4-enoate (**5m**)

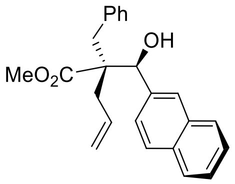

5m

<Chromatogram>

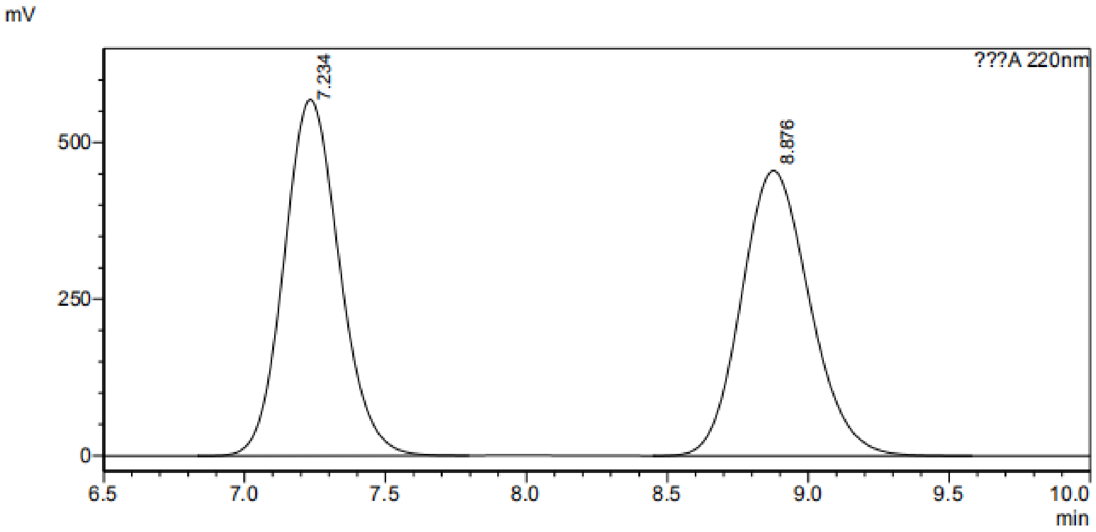

<Peak Table>

???A 220nm

Peak#	Ret. Time	Area	Height	Conc.	Unit	Mark	Name
1	7.234	7795647	569152	50.335			
2	8.876	7691938	455186	49.665			
Total		15487586	1024338				

<Chromatogram>

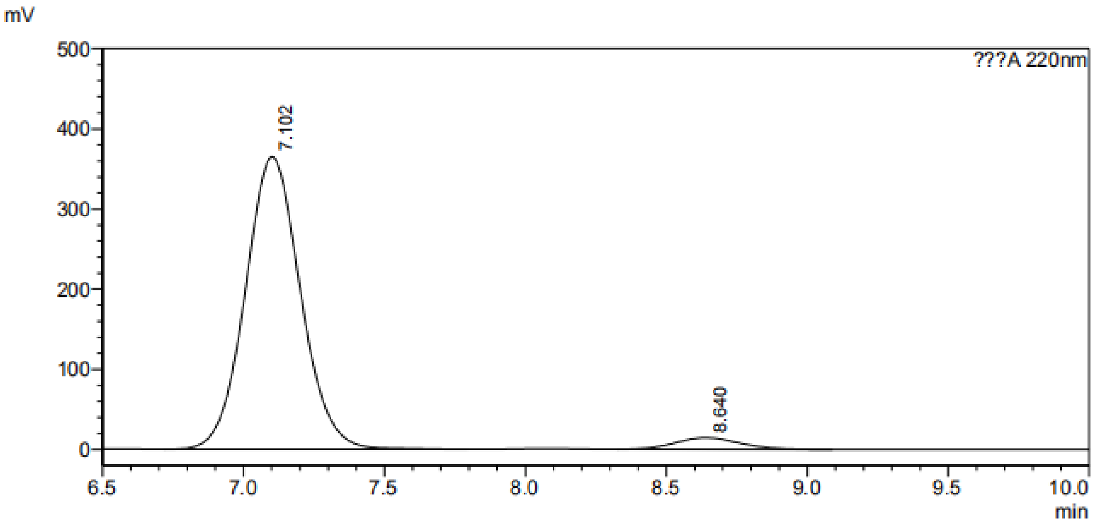

<Peak Table>

???A 220nm

Peak#	Ret. Time	Area	Height	Conc.	Unit	Mark	Name
1	7.102	4970789	364995	95.583			
2	8.640	229717	14630	4.417			
Total		5200506	379625				

(*S*)-Methyl 2-benzyl-2-((*S*)-hydroxy(naphthalen-2-yl)methyl)pent-4-enoate (**5m**)

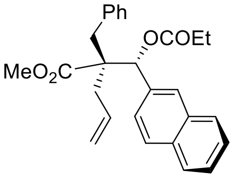

6m

<Chromatogram>

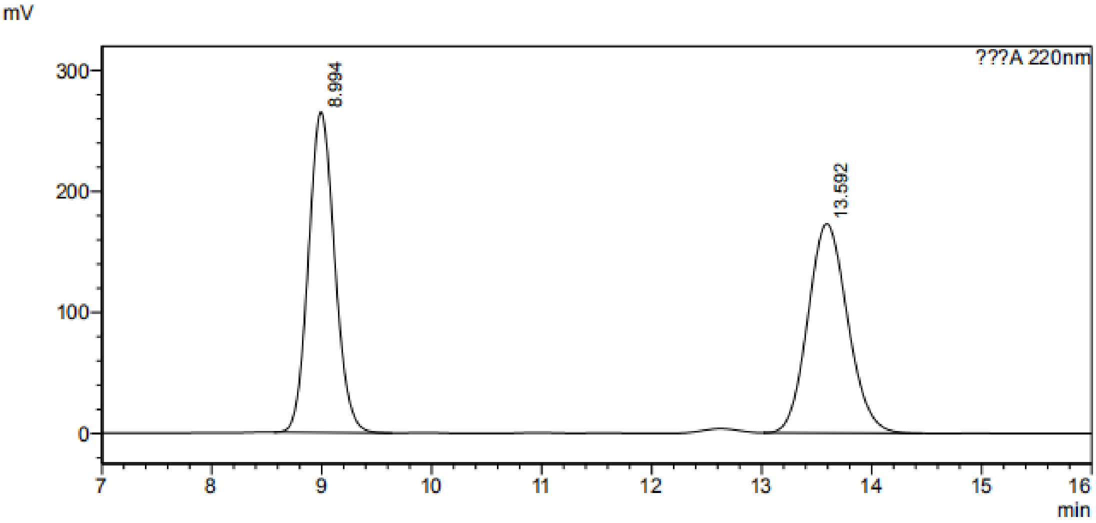

<Peak Table>

???A 220nm

Peak#	Ret. Time	Area	Height	Conc.	Unit	Mark	Name
1	8.994	4394199	264988	50.213			
2	13.592	4356881	172974	49.787			
Total		8751080	437962				

<Chromatogram>

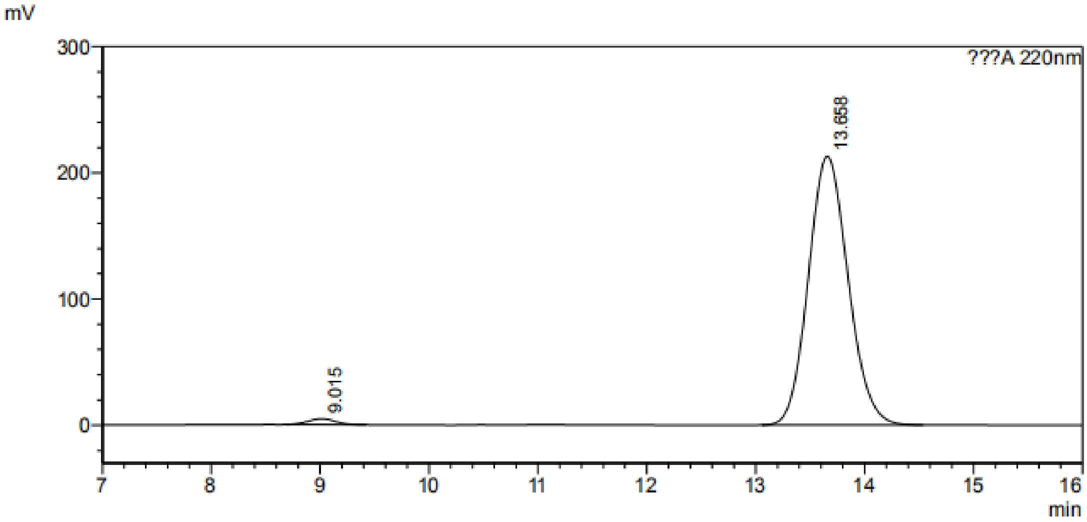

<Peak Table>

???A 220nm

Peak#	Ret. Time	Area	Height	Conc.	Unit	Mark	Name
1	9.015	84542	4803	1.539			
2	13.658	5410106	213014	98.461			
Total		5494648	217818				

Ethyl (*R*)-2-((*R*)-furan-2-yl(hydroxy)methyl)-2-methylpent-4-enoate (**5n**)

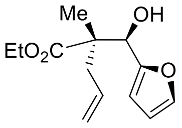

5n

<Chromatogram>

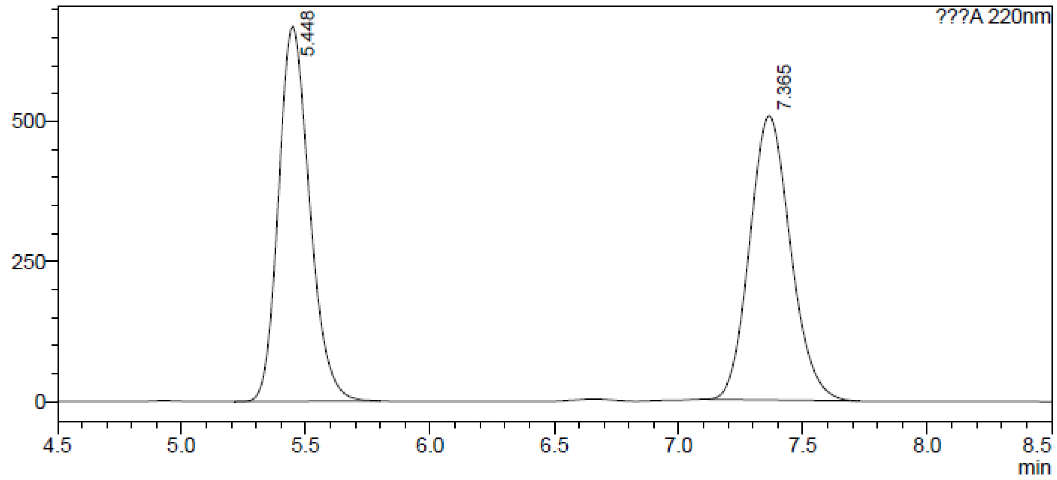

<Peak Table>

???A 220nm

Peak#	Ret. Time	Area	Height	Conc.	Unit	Mark	Name
1	5.448	5873929	669134	50.286		M	
2	7.365	5807014	506851	49.714		M	
Total		11680943	1175985				

<Chromatogram>

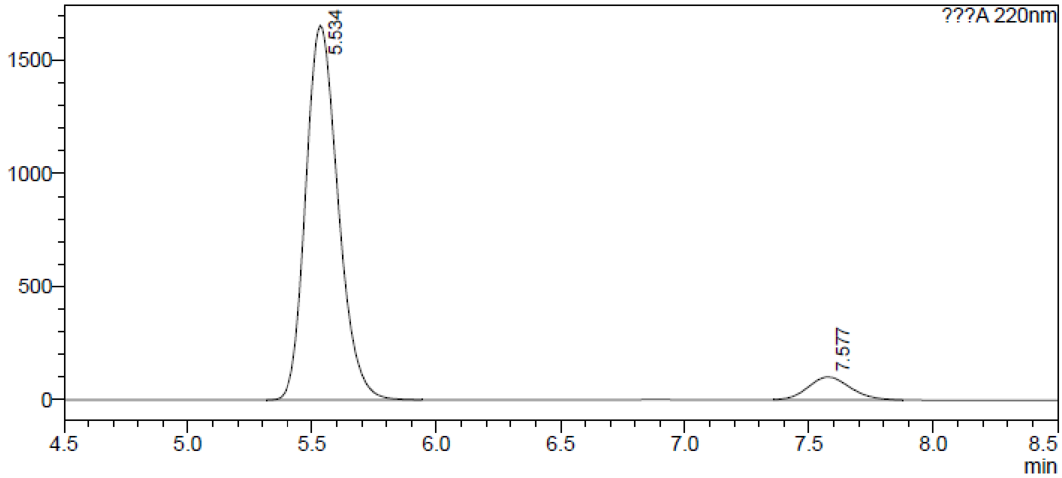

<Peak Table>

???A 220nm

Peak#	Ret. Time	Area	Height	Conc.	Unit	Mark	Name
1	5.534	15005408	1656990	92.762		M	
2	7.577	1170867	100310	7.238		M	
Total		16176275	1757300				

Ethyl (*S*)-2-((*S*)-furan-2-yl(propionyloxy)methyl)-2-methylpent-4-enoate (**6n**)

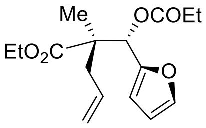

6n

<Chromatogram>

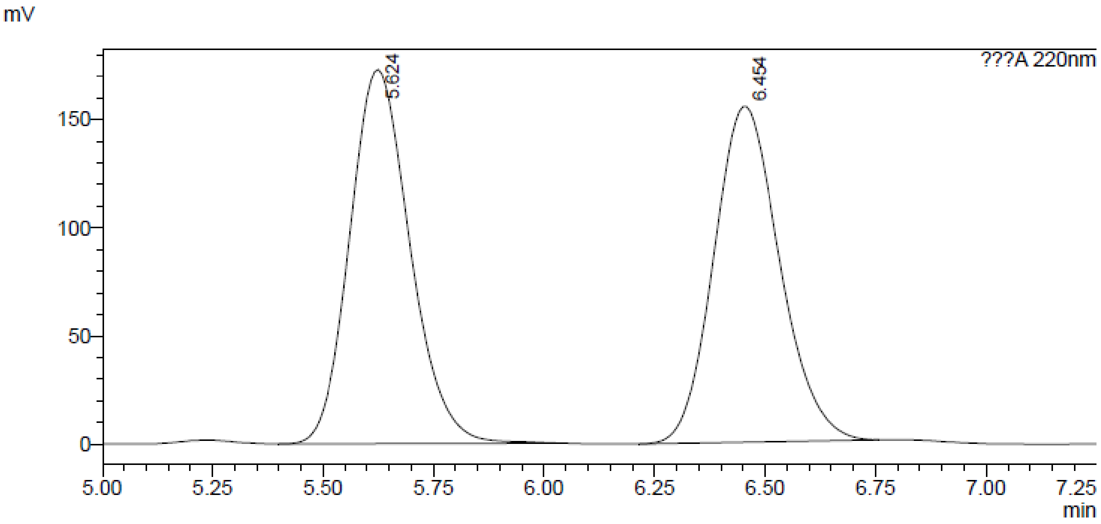

<Peak Table>

???A 220nm

Peak#	Ret. Time	Area	Height	Conc.	Unit	Mark	Name
1	5.624	1641949	173163	50.372		M	
2	6.454	1617727	155226	49.628		M	
Total		3259677	328389				

<Chromatogram>

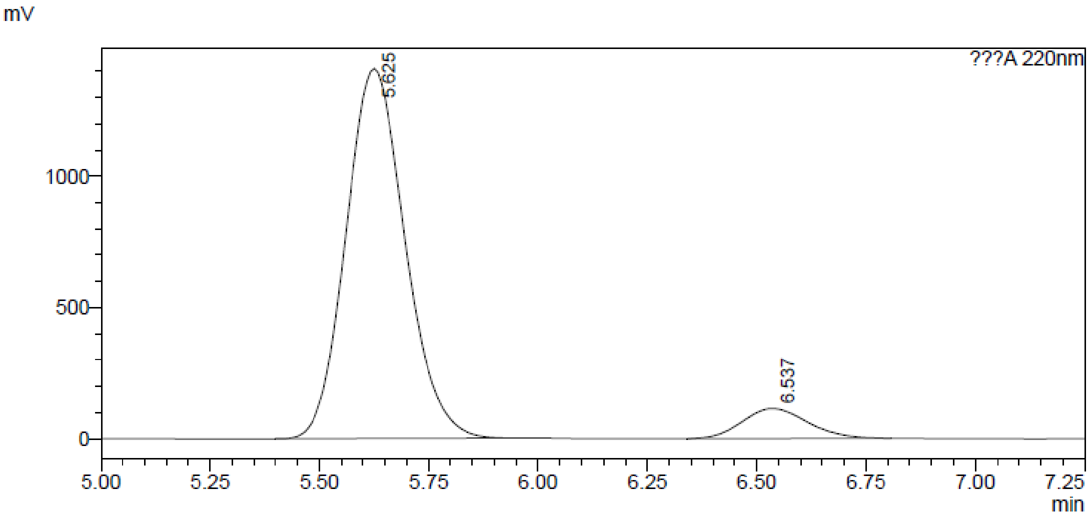

<Peak Table>

???A 220nm

Peak#	Ret. Time	Area	Height	Conc.	Unit	Mark	Name
1	5.625	13255579	1408613	91.735		M	
2	6.537	1194283	113832	8.265		M	
Total		14449861	1522444				

Ethyl (2*R*,3*S*,*E*)-2-allyl-3-hydroxy-2-methyl-5-phenylpent-4-enoate (**5o**)

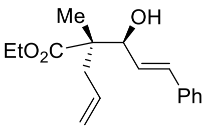

5o

<Chromatogram>

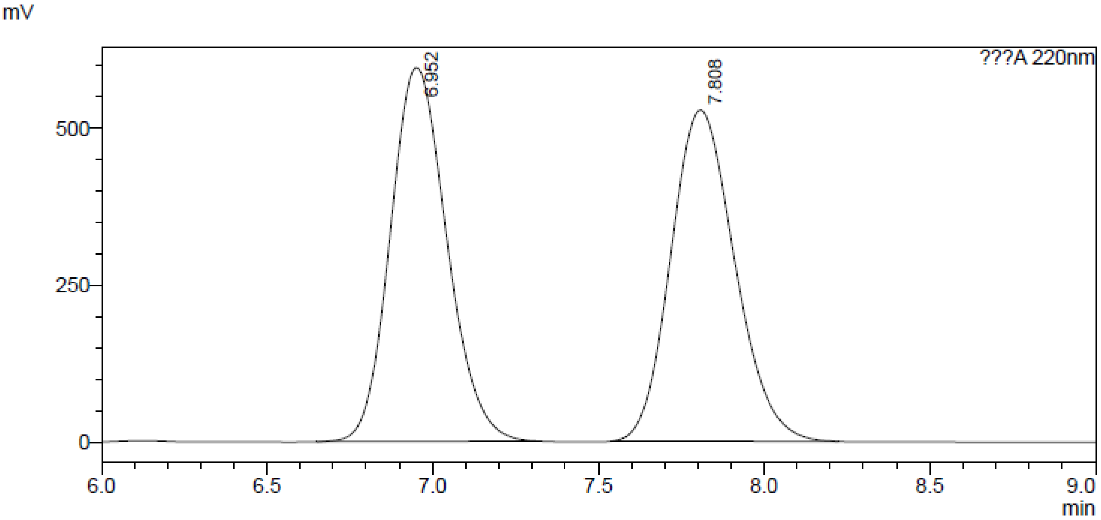

<Peak Table>

???A 220nm

Peak#	Ret. Time	Area	Height	Conc.	Unit	Mark	Name
1	6.952	7075648	596303	49.978		M	
2	7.808	7081865	527869	50.022		M	
Total		14157513	1124172				

<Chromatogram>

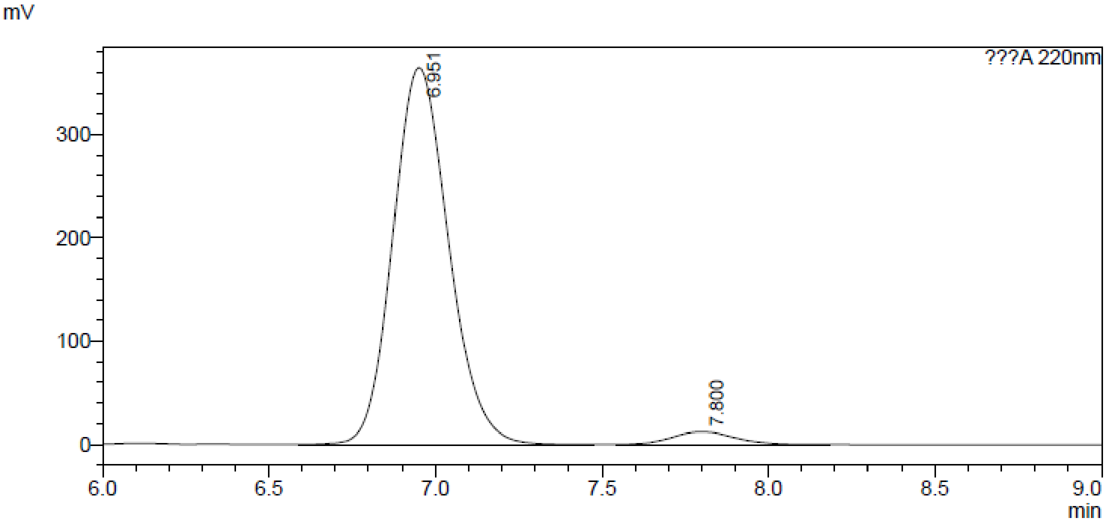

<Peak Table>

???A 220nm

Peak#	Ret. Time	Area	Height	Conc.	Unit	Mark	Name
1	6.951	4242280	364739	96.422		M	
2	7.800	157409	12303	3.578		M	
Total		4399689	377042				

Ethyl (2*S*,3*R*,*E*)-2-allyl-2-methyl-5-phenyl-3-(propionyloxy)pent-4-enoate (**6o**)

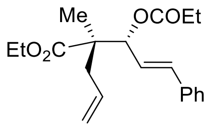

6o

<Chromatogram>

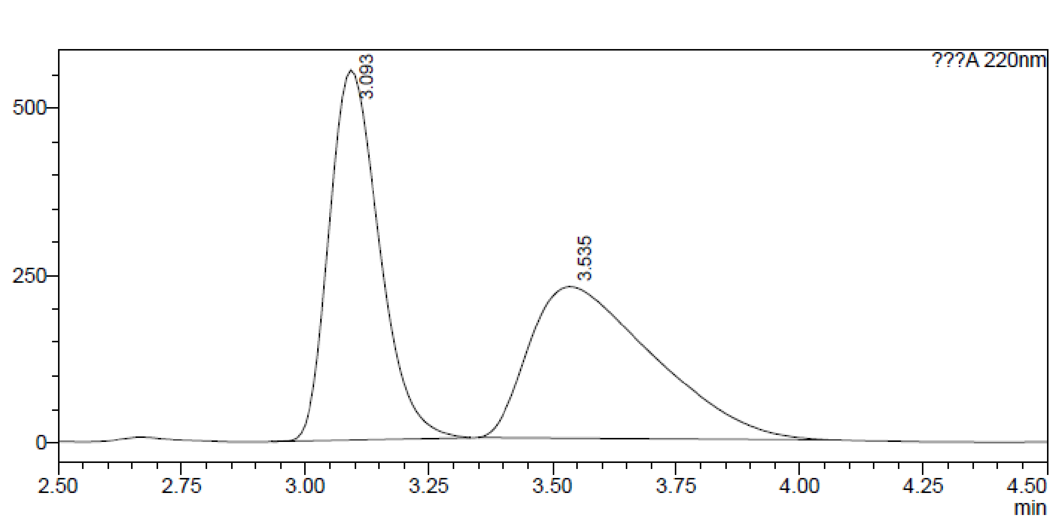

<Peak Table>

???A 220nm

Peak#	Ret. Time	Area	Height	Conc.	Unit	Mark	Name
1	3.093	3907863	552865	49.630		M	
2	3.535	3966177	226141	50.370		M	
Total		7874040	779006				

<Chromatogram>

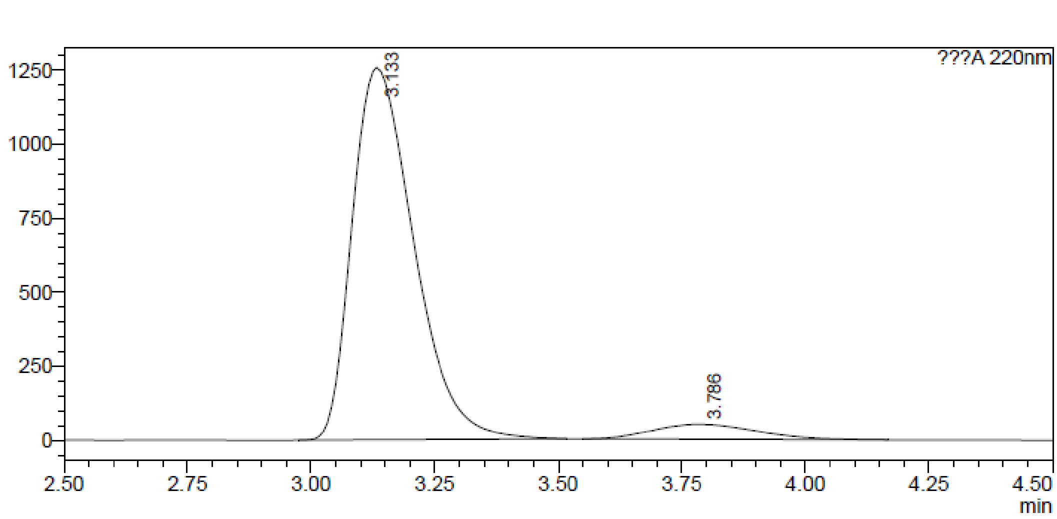

<Peak Table>

???A 220nm

Peak#	Ret. Time	Area	Height	Conc.	Unit	Mark	Name
1	3.133	10926464	1256395	93.616		M	
2	3.786	745092	51264	6.384		M	
Total		11671555	1307659				

(1*R*,2*S*)-2-methyl-1-phenyl-2-vinylpent-4-en-1-ol (**7a**)

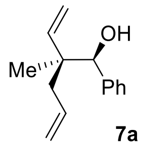

7a

<Chromatogram>

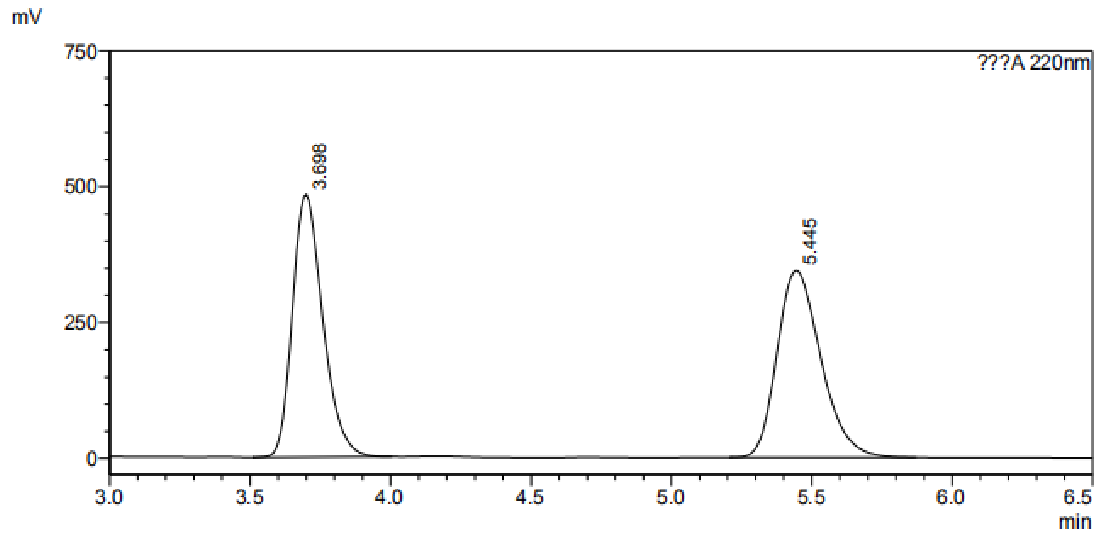

<Peak Table>

???A 220nm

Peak#	Ret. Time	Area	Height	Conc.	Unit	Mark	Name
1	3.698	3637493	482927	49.319			
2	5.445	3737989	344136	50.681			
Total		7375482	827062				

<Chromatogram>

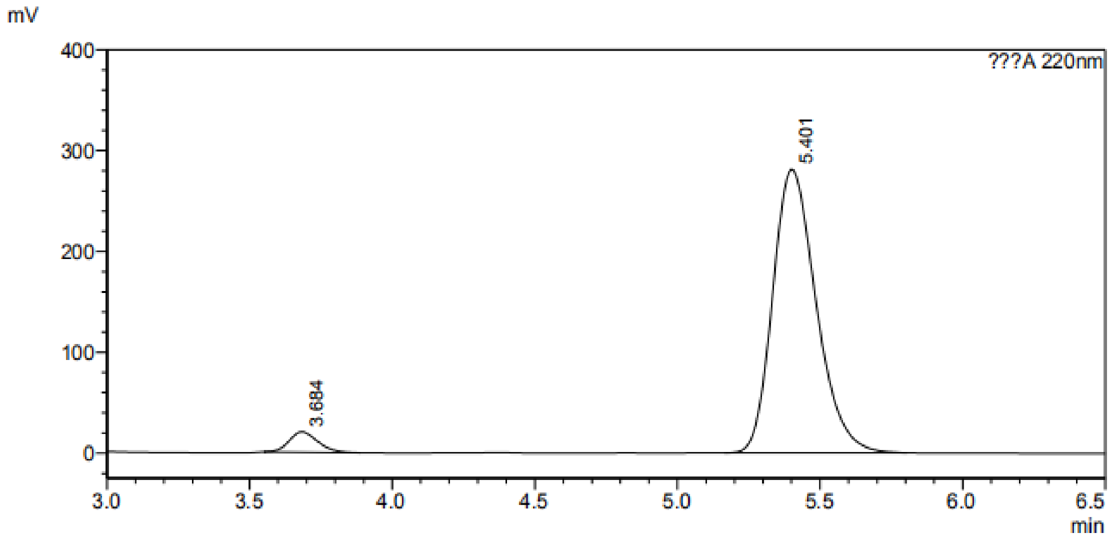

<Peak Table>

???A 220nm

Peak#	Ret. Time	Area	Height	Conc.	Unit	Mark	Name
1	3.684	140175	20100	4.497			
2	5.401	2976578	281180	95.503			
Total		3116753	301280				

(1*S*,2*R*)-2-methyl-1-phenyl-2-vinylpent-4-en-1-yl propionate (**8a**)

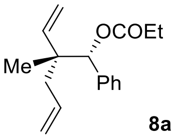

8a

<Chromatogram>

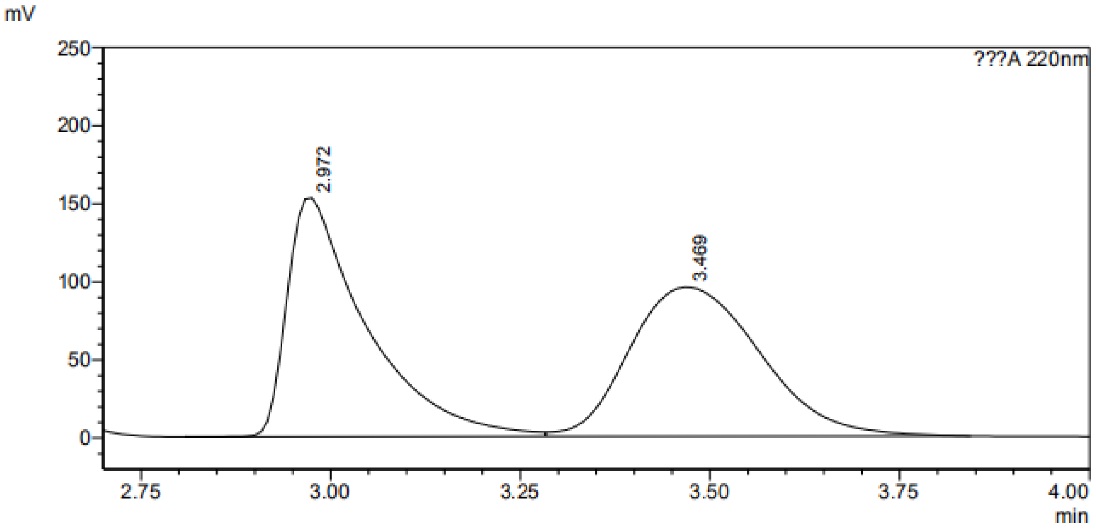

<Peak Table>

???A 220nm

Peak#	Ret. Time	Area	Height	Conc.	Unit	Mark	Name
1	2.972	1111579	153177	49.325			
2	3.469	1142000	95478	50.675		V	
Total		2253579	248654				

<Chromatogram>

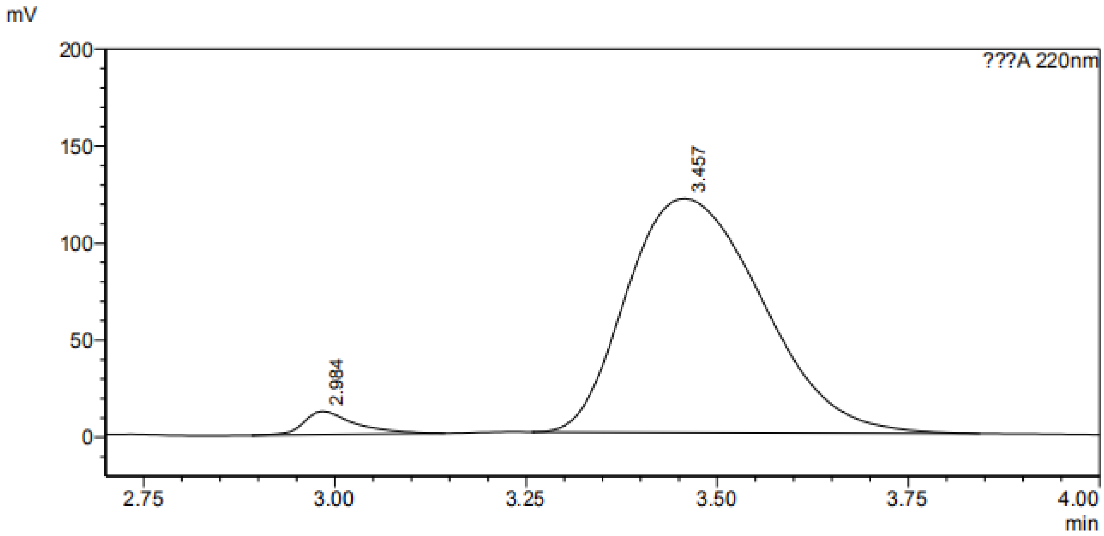

<Peak Table>

???A 220nm

Peak#	Ret. Time	Area	Height	Conc.	Unit	Mark	Name
1	2.984	55976	12124	3.634			
2	3.457	1484334	120608	96.366			
Total		1540311	132731				

(1*S*,2*S*)-2-ethynyl-2-methyl-1-phenylpent-4-en-1-ol (**7b**)

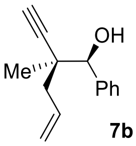

7b

<Chromatogram>

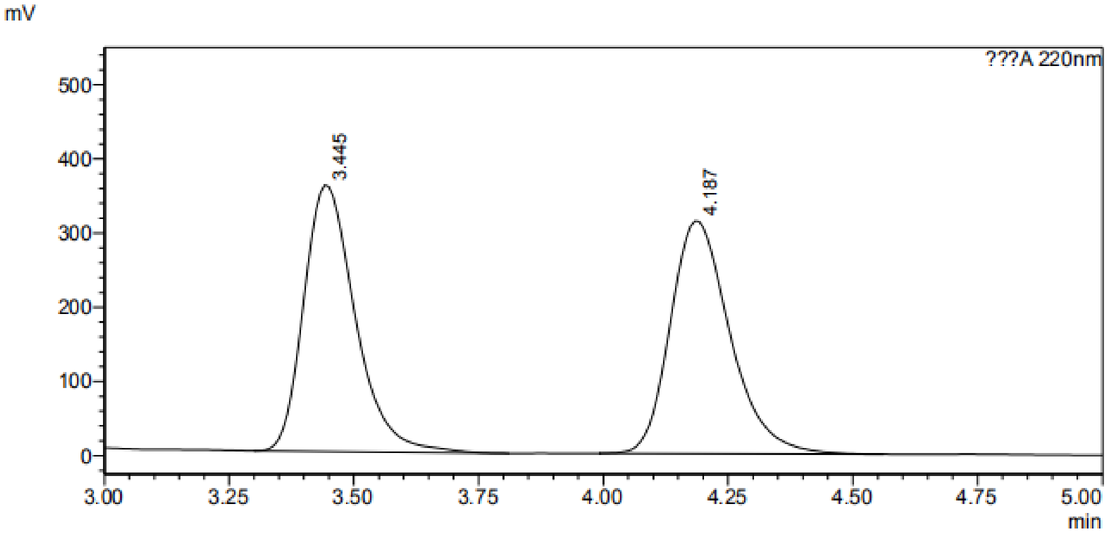

<Peak Table>

???A 220nm

Peak#	Ret. Time	Area	Height	Conc.	Unit	Mark	Name
1	3.445	2574542	359007	49.628			
2	4.187	2613134	313142	50.372			
Total		5187675	672149				

<Chromatogram>

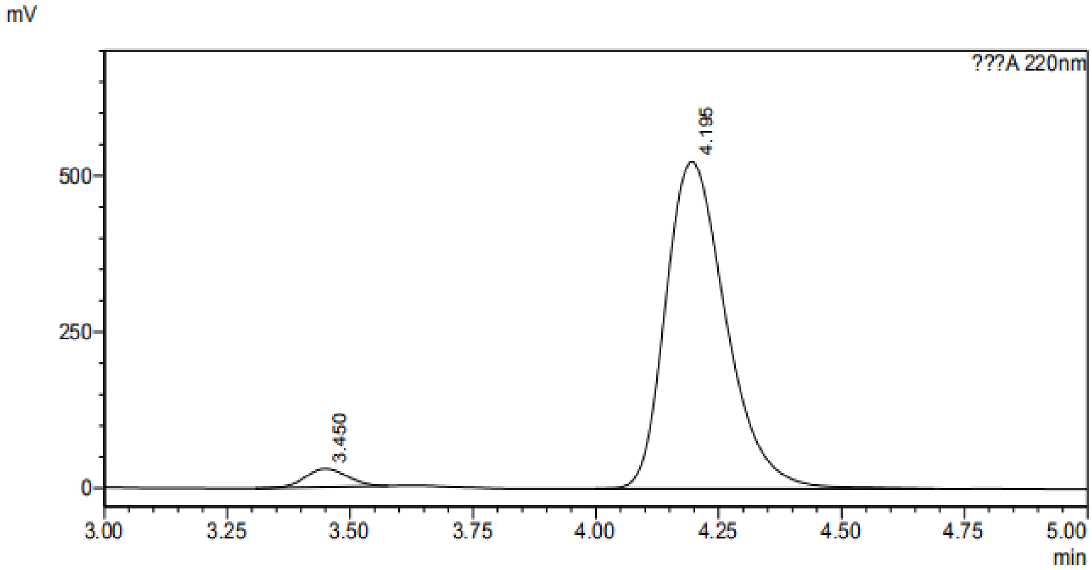

<Peak Table>

???A 220nm

Peak#	Ret. Time	Area	Height	Conc.	Unit	Mark	Name
1	3.450	175608	29448	3.777			
2	4.195	4473805	523423	96.223			
Total		4649413	552870				

(1*R*,2*R*)-2-ethynyl-2-methyl-1-phenylpent-4-en-1-yl propionate **(8b)**

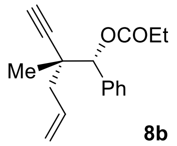

8b

<Chromatogram>

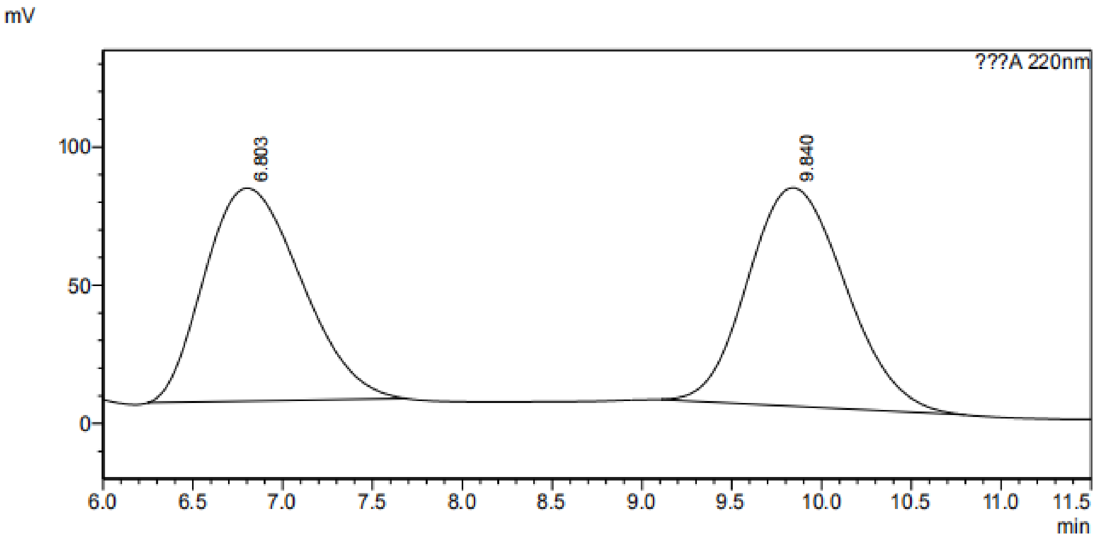

<Peak Table>

???A 220nm

Peak#	Ret. Time	Area	Height	Conc.	Unit	Mark	Name
1	6.803	2889439	77047	49.049			
2	9.840	3001522	78993	50.951		M	
Total		5890961	156040				

<Chromatogram>

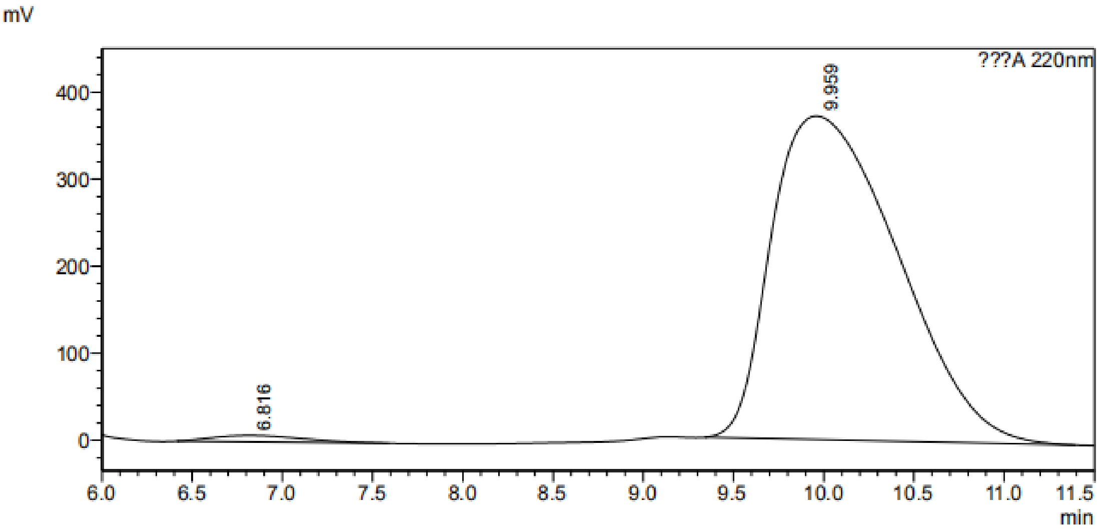

<Peak Table>

???A 220nm

Peak#	Ret. Time	Area	Height	Conc.	Unit	Mark	Name
1	6.816	252782	7213	1.378		M	
2	9.959	18093861	371727	98.622			
Total		18346643	378940				

(*S*)-2-benzyl-2-((*S*)-hydroxy(phenyl)methyl)butyl propionate (**7c**)

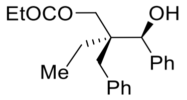

7c

<Chromatogram>

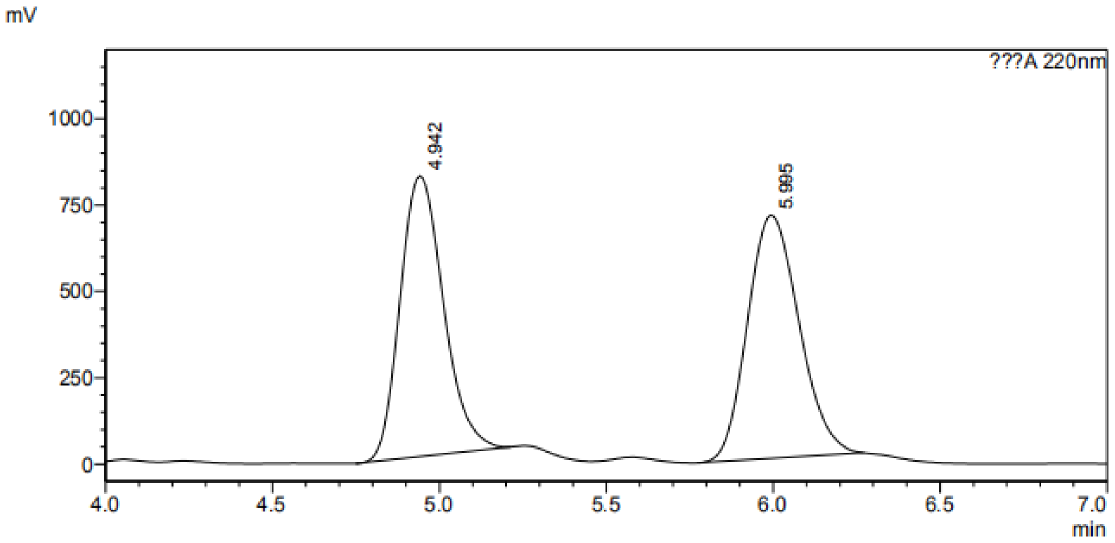

<Peak Table>

???A 220nm

Peak#	Ret. Time	Area	Height	Conc.	Unit	Mark	Name
1	4.942	7359056	813223	49.300		M	
2	5.995	7567958	703261	50.700		M	
Total		14927014	1516484				

<Chromatogram>

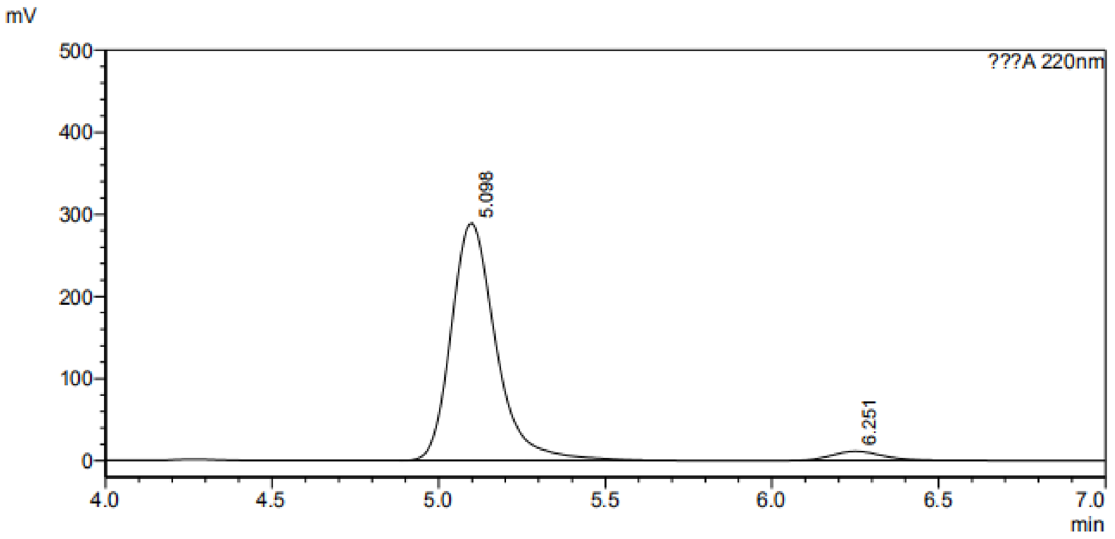

<Peak Table>

???A 220nm

Peak#	Ret. Time	Area	Height	Conc.	Unit	Mark	Name
1	5.098	2686683	288763	96.007			
2	6.251	111730	11031	3.993			
Total		2798412	299794				

(1*R*,2*R*)-2-benzyl-2-ethyl-1-phenylpropane-1,3-diyl dipropionate (**8c**)

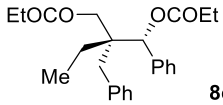

8c

<Chromatogram>

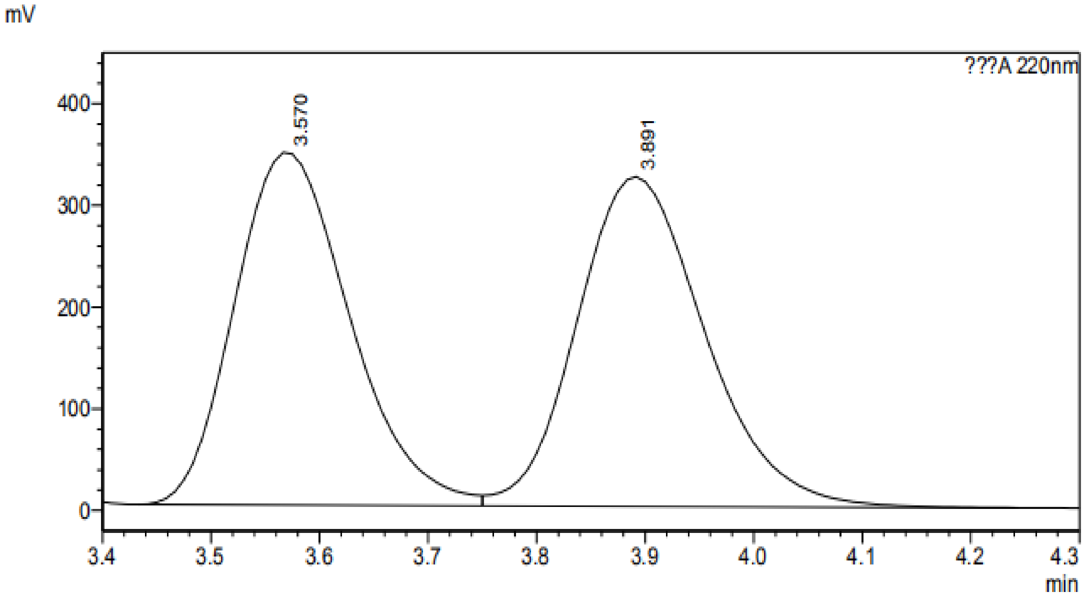

<Peak Table>

???A 220nm

Peak#	Ret. Time	Area	Height	Conc.	Unit	Mark	Name
1	3.570	2557211	346592	49.091			
2	3.891	2651947	324124	50.909		V	
Total		5209158	670717				

<Chromatogram>

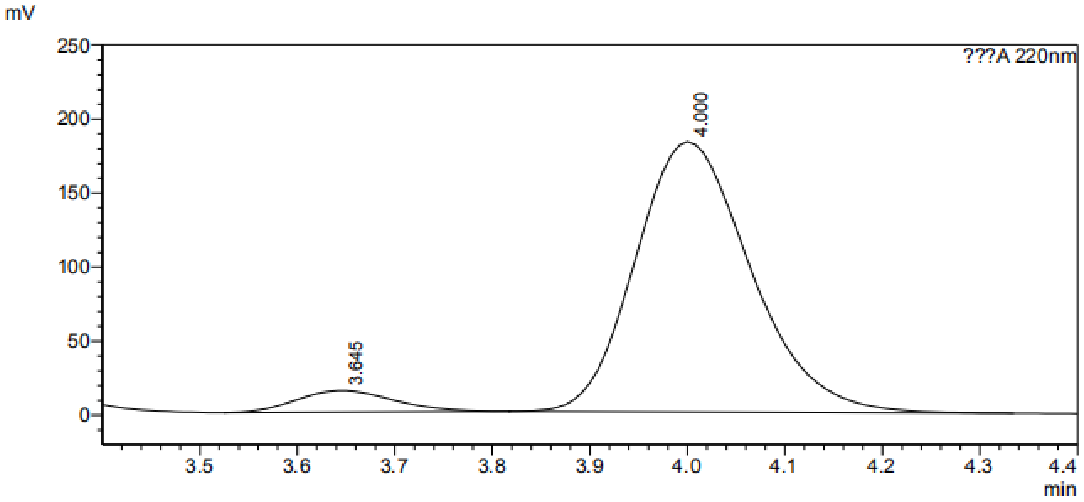

<Peak Table>

???A 220nm

Peak#	Ret. Time	Area	Height	Conc.	Unit	Mark	Name
1	3.645	101601	14547	6.333			
2	4.000	1502822	182723	93.667		M	
Total		1604423	197271				

(*S*)-2-(4-fluorobenzyl)-2-((*S*)-hydroxy(phenyl)methyl)butyl propionate (**7d**)

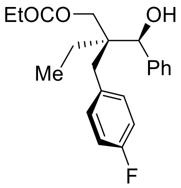

7d

<Chromatogram>

mV

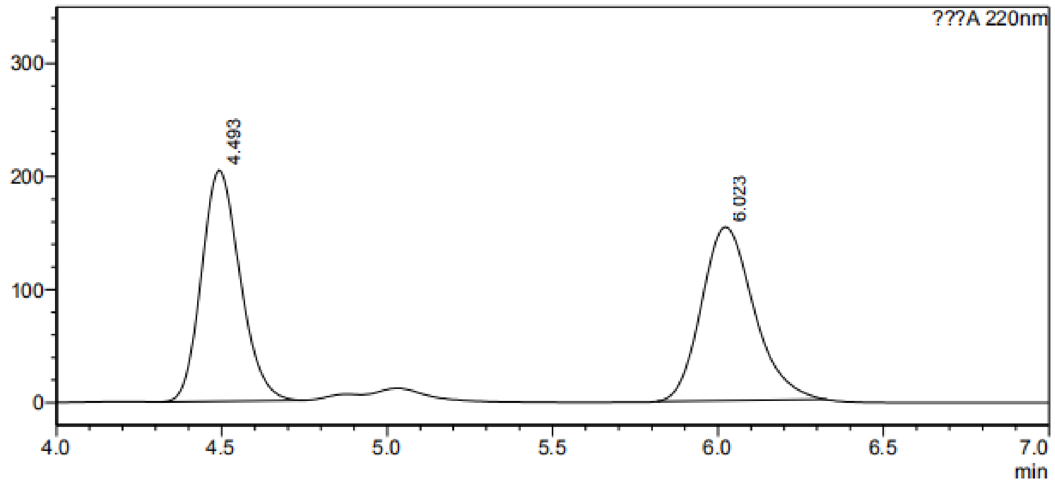

<Peak Table>

???A 220nm

Peak#	Ret. Time	Area	Height	Conc.	Unit	Mark	Name
1	4.493	1659769	204481	49.423		M	
2	6.023	1698551	153591	50.577		M	
Total		3358320	358072				

<Chromatogram>

mV

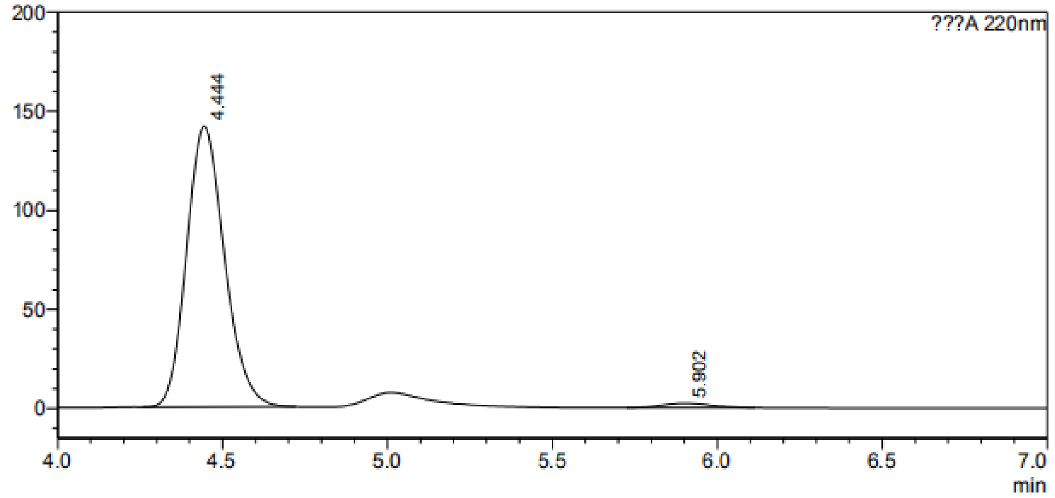

<Peak Table>

???A 220nm

Peak#	Ret. Time	Area	Height	Conc.	Unit	Mark	Name
1	4.444	1132741	141744	97.995			
2	5.902	23179	2317	2.005		M	
Total		1155920	144062				

(1*R*,2*R*)-2-ethyl-2-(4-fluorobenzyl)-1-phenylpropane-1,3-diyl dipropionate (**8d**)

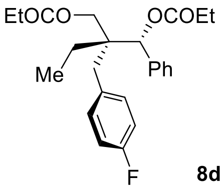

8d

<Chromatogram>

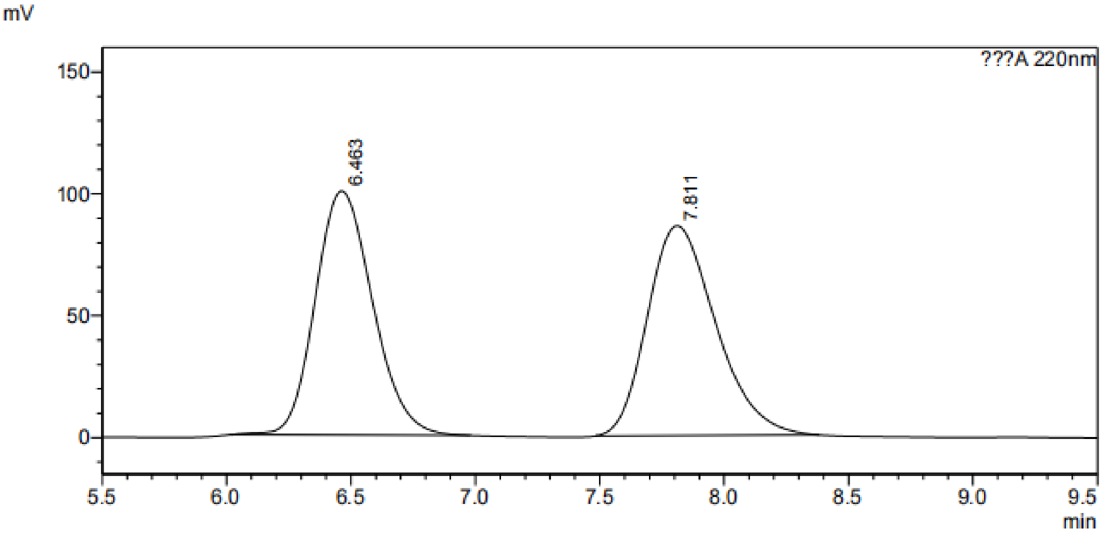

<Peak Table>

???A 220nm

Peak#	Ret. Time	Area	Height	Conc.	Unit	Mark	Name
1	6.463	1612894	100150	49.451		M	
2	7.811	1648689	86101	50.549			
Total		3261582	186250				

<Chromatogram>

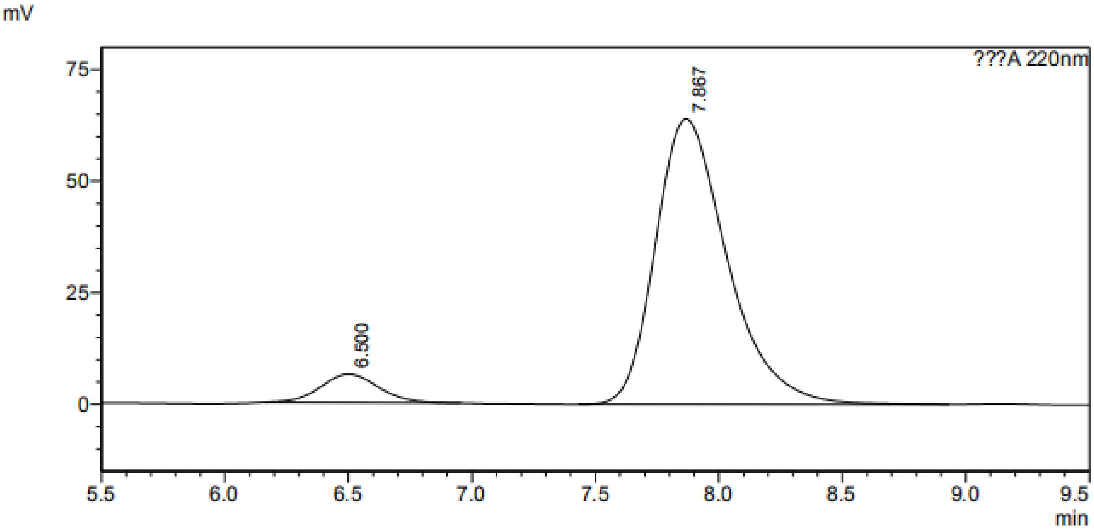

<Peak Table>

???A 220nm

Peak#	Ret. Time	Area	Height	Conc.	Unit	Mark	Name
1	6.500	104407	6375	7.440		M	
2	7.867	1298842	63984	92.560		M	
Total		1403249	70359				

(1*R*,2*R*)-2-methyl-1-phenyl-2-vinylpent-4-yn-1-ol (**7e**)

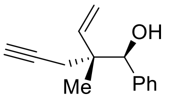

<Chromatogram>

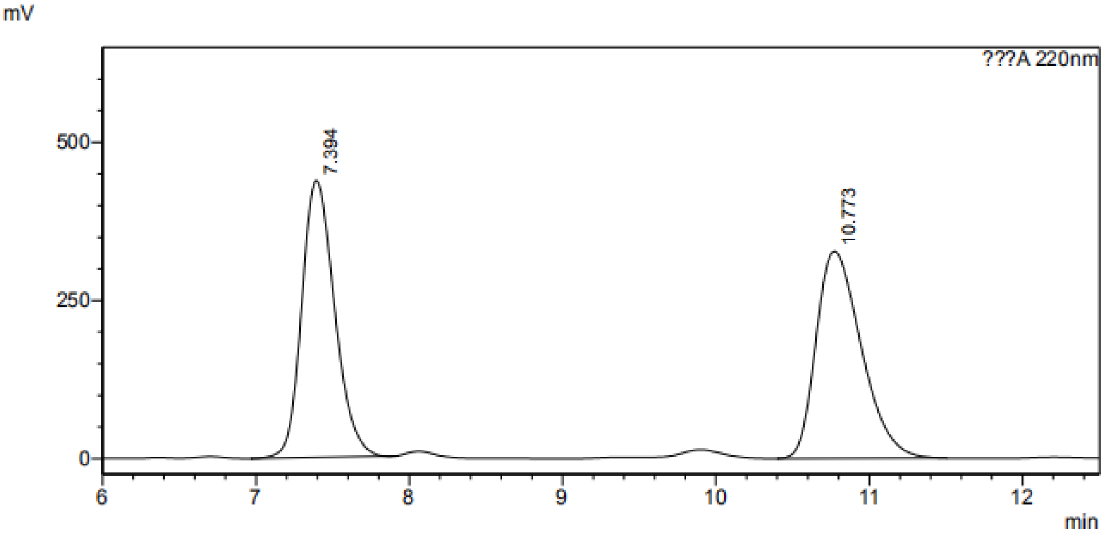

<Peak Table>

???A 220nm

Peak#	Ret. Time	Area	Height	Conc.	Unit	Mark	Name
1	7.394	6528211	437831	49.305		M	
2	10.773	6712359	327337	50.695		M	
Total		13240570	765167				

<Chromatogram>

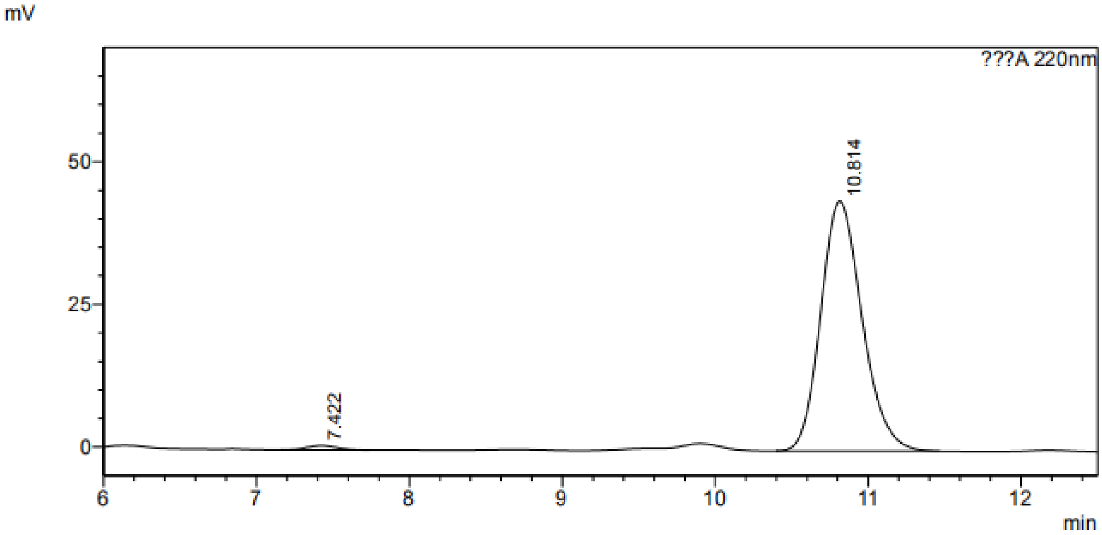

<Peak Table>

???A 220nm

Peak#	Ret. Time	Area	Height	Conc.	Unit	Mark	Name
1	7.422	10162	758	1.217		M	
2	10.814	824950	43750	98.783		M	
Total		835113	44509				

(1*S*,2*S*)-2-methyl-1-phenyl-2-vinylpent-4-yn-1-yl propionate (**8e**)

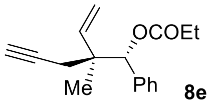

<Chromatogram>

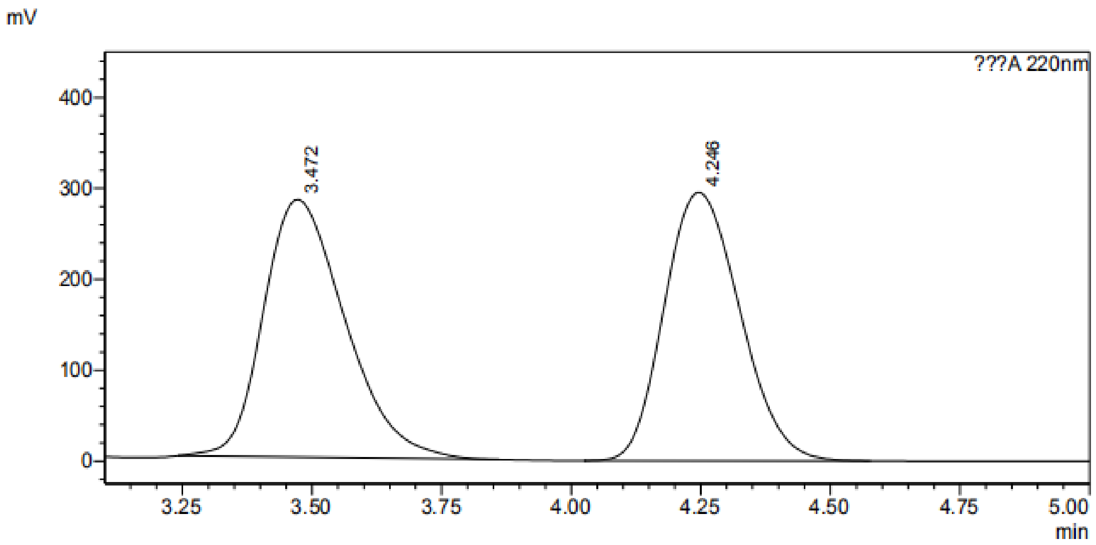

<Peak Table>

???A 220nm

Peak#	Ret. Time	Area	Height	Conc.	Unit	Mark	Name
1	3.472	3109263	283481	50.868		M	
2	4.246	3003122	294878	49.132			
Total		6112385	578359				

<Chromatogram>

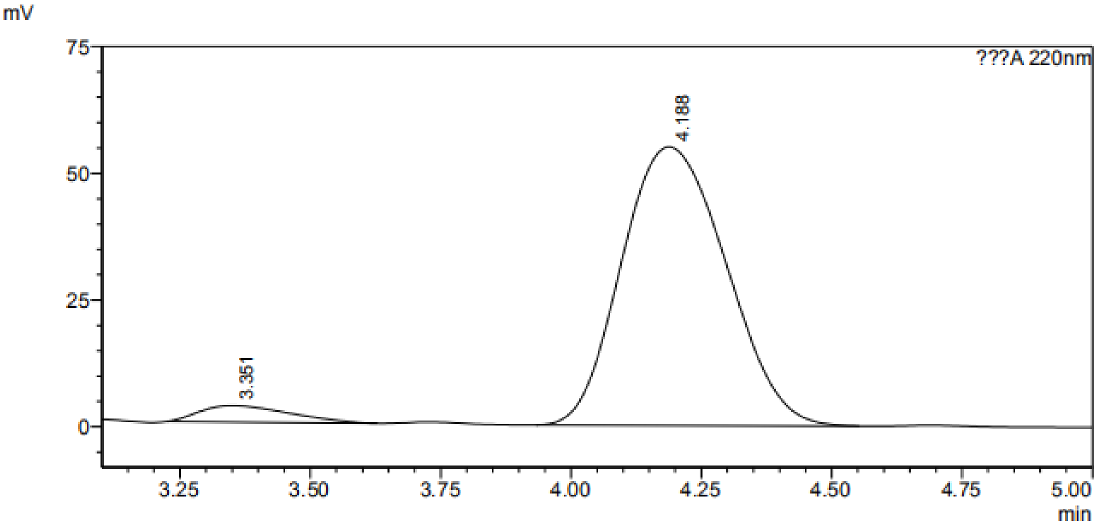

<Peak Table>

???A 220nm

Peak#	Ret. Time	Area	Height	Conc.	Unit	Mark	Name
1	3.351	39101	3270	4.856		M	
2	4.188	766171	54935	95.144		M	
Total		805272	58205				

(1*S*,2*S*)-2-(methoxymethyl)-2-methyl-1-phenylpent-4-en-1-ol (**7f**)

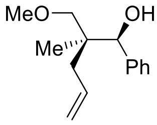

7f

<Chromatogram>

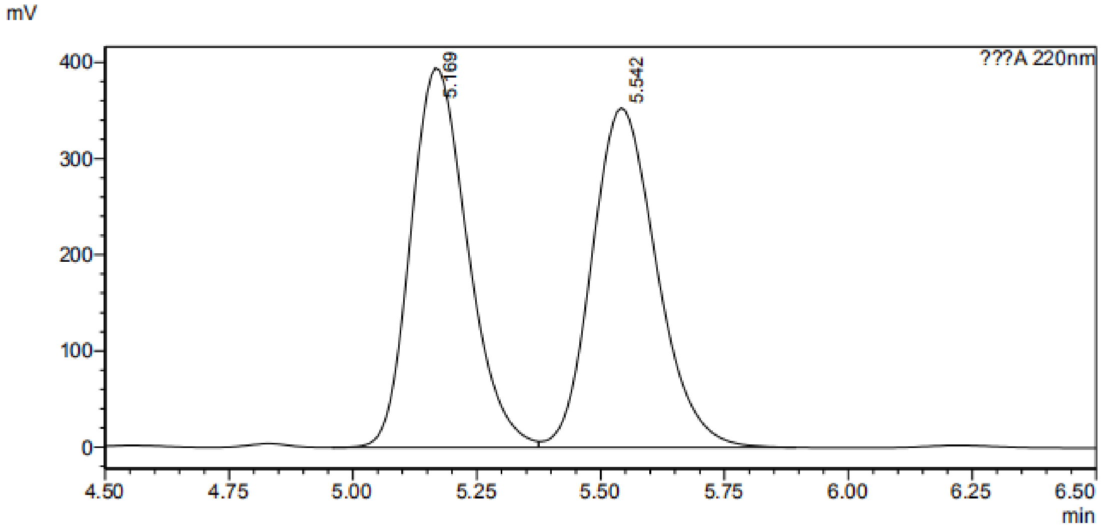

<Peak Table>

???A 220nm

Peak#	Ret. Time	Area	Height	Conc.	Unit	Mark	Name
1	5.169	3150924	394474	49.561			
2	5.542	3206770	352971	50.439		V	
Total		6357694	747445				

<Chromatogram>

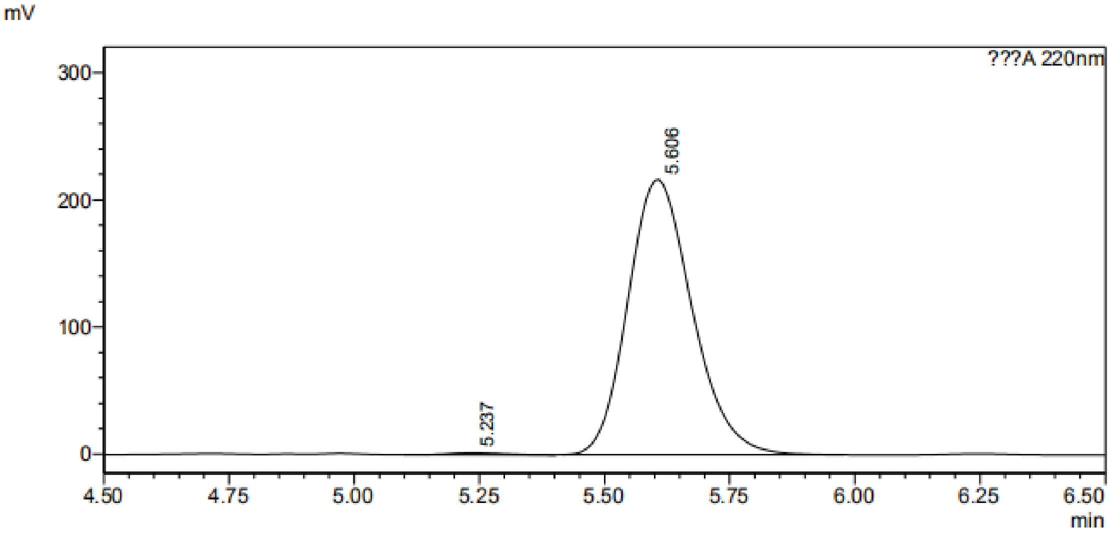

<Peak Table>

???A 220nm

Peak#	Ret. Time	Area	Height	Conc.	Unit	Mark	Name
1	5.237	12363	1681	0.634		M	
2	5.606	1938432	216542	99.366		M	
Total		1950796	218223				

(1*R*,2*R*)-2-(methoxymethyl)-2-methyl-1-phenylpent-4-en-1-yl propionate (**8f**)

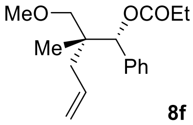

8f

<Chromatogram>

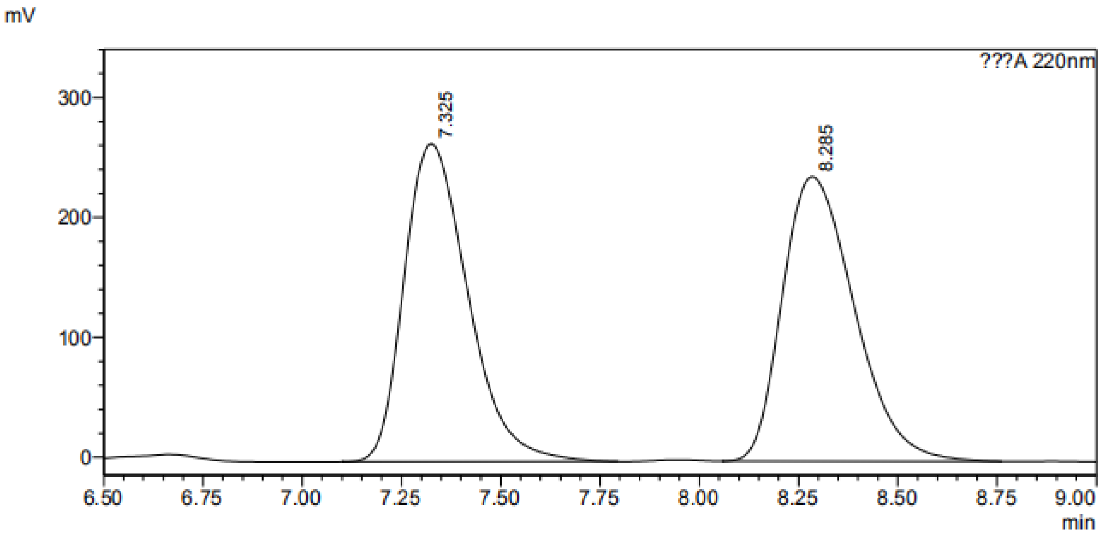

<Peak Table>

???A 220nm

Peak#	Ret. Time	Area	Height	Conc.	Unit	Mark	Name
1	7.325	2972222	265254	49.794			
2	8.285	2996844	237202	50.206			
Total		5969066	502456				

<Chromatogram>

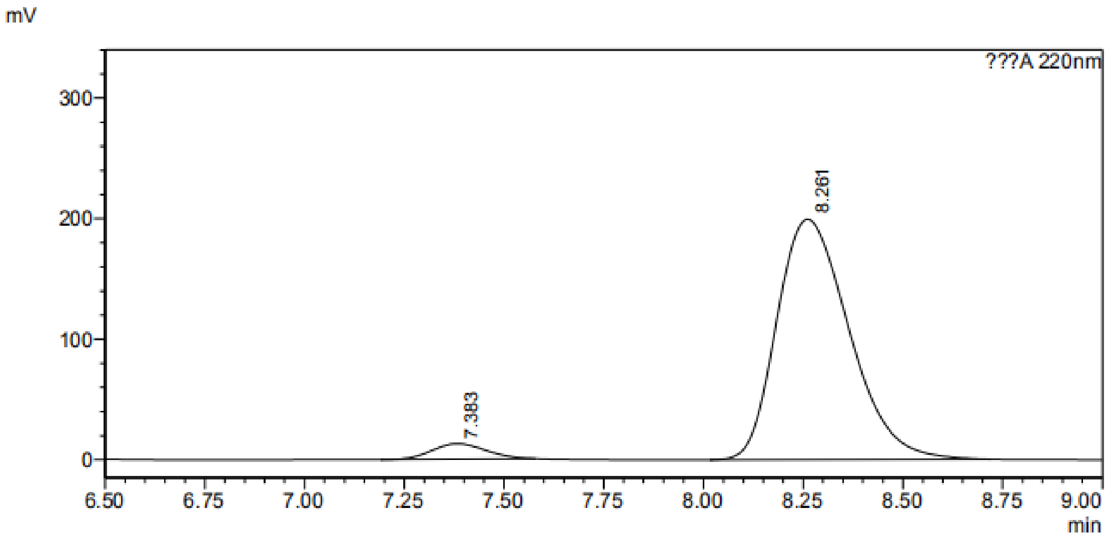

<Peak Table>

???A 220nm

Peak#	Ret. Time	Area	Height	Conc.	Unit	Mark	Name
1	7.383	121651	12913	4.611		M	
2	8.261	2516627	199573	95.389			
Total		2638278	212486				

(2*S*,3*S*)-3-hydroxy-2-methyl-2,3-diphenylpropyl propionate (**7g**)

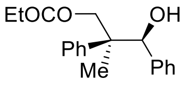

7g

<Chromatogram>

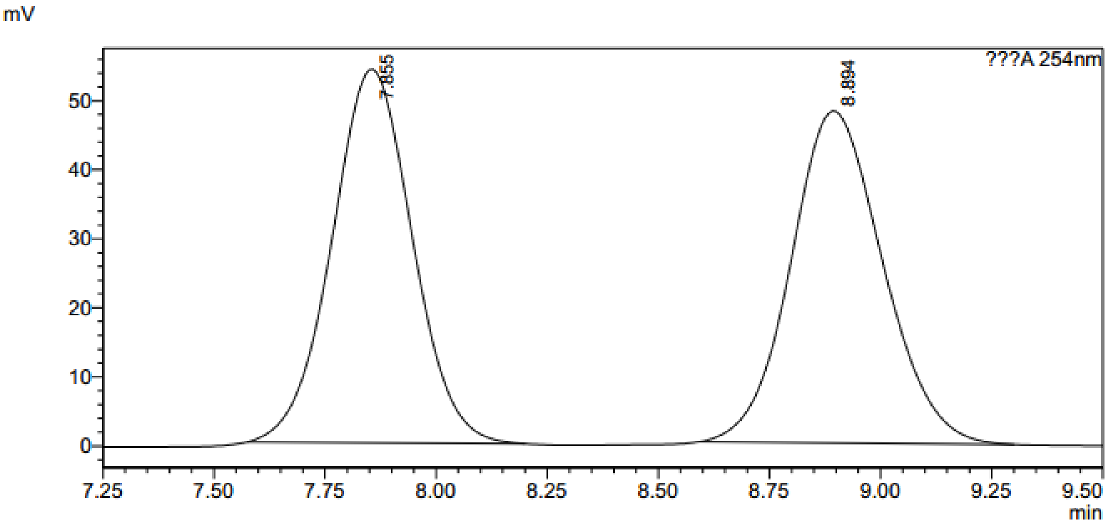

<Peak Table>

???A 254nm

Peak#	Ret. Time	Area	Height	Conc.	Unit	Mark	Name
1	7.855	682345	54093	49.619		M	
2	8.894	692820	48105	50.381		M	
Total		1375165	102198				

<Chromatogram>

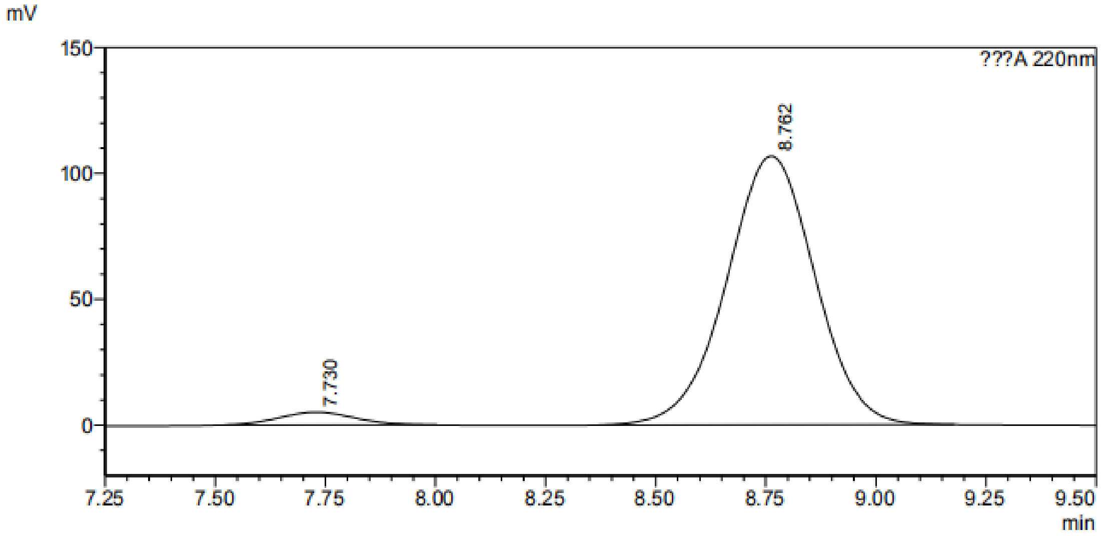

<Peak Table>

???A 220nm

Peak#	Ret. Time	Area	Height	Conc.	Unit	Mark	Name
1	7.730	62750	5174	4.024		M	
2	8.762	1496662	106810	95.976		M	
Total		1559412	111984				

(1*R*,2*R*)-2-methyl-1,2-diphenylpropane-1,3-diyl dipropionate (**8g**)

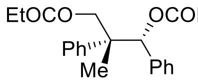

8g

<Chromatogram>

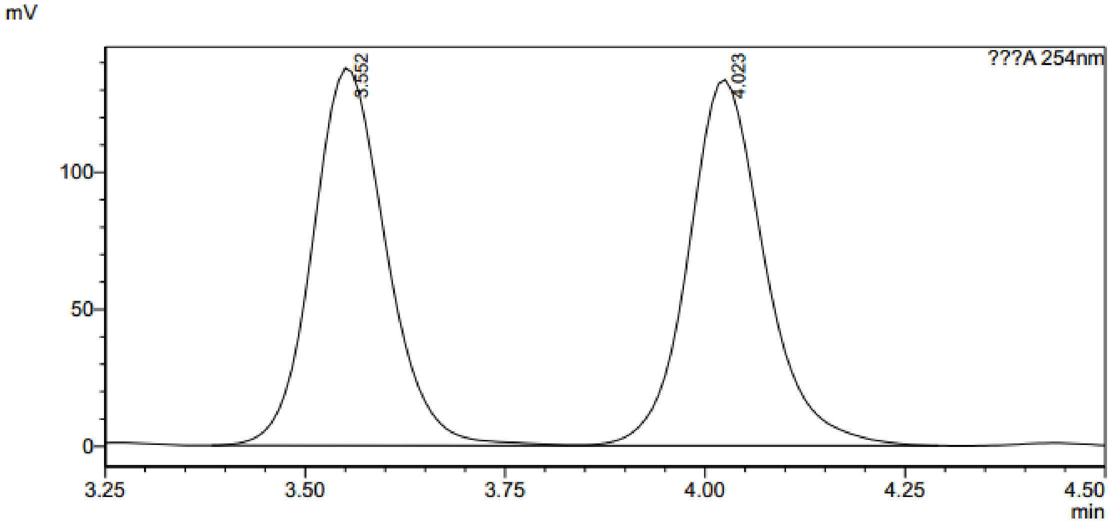

<Peak Table>

???A 254nm

Peak#	Ret. Time	Area	Height	Conc.	Unit	Mark	Name
1	3.552	878846	137702	49.740			
2	4.023	888034	133558	50.260		V	
Total		1766880	271261				

<Chromatogram>

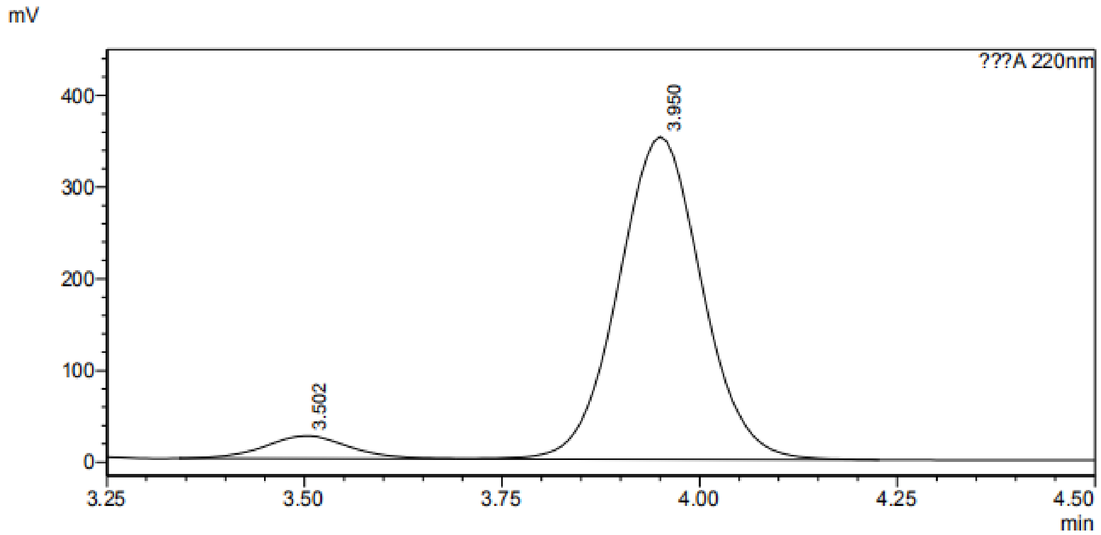

<Peak Table>

???A 220nm

Peak#	Ret. Time	Area	Height	Conc.	Unit	Mark	Name
1	3.502	185621	24959	6.694			
2	3.950	2587409	351800	93.306		V	
Total		2773030	376759				

(1*S*,2*S*)-2-methyl-1,2-diphenylpent-4-en-1-ol (**7h**)

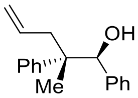

7h

<Chromatogram>

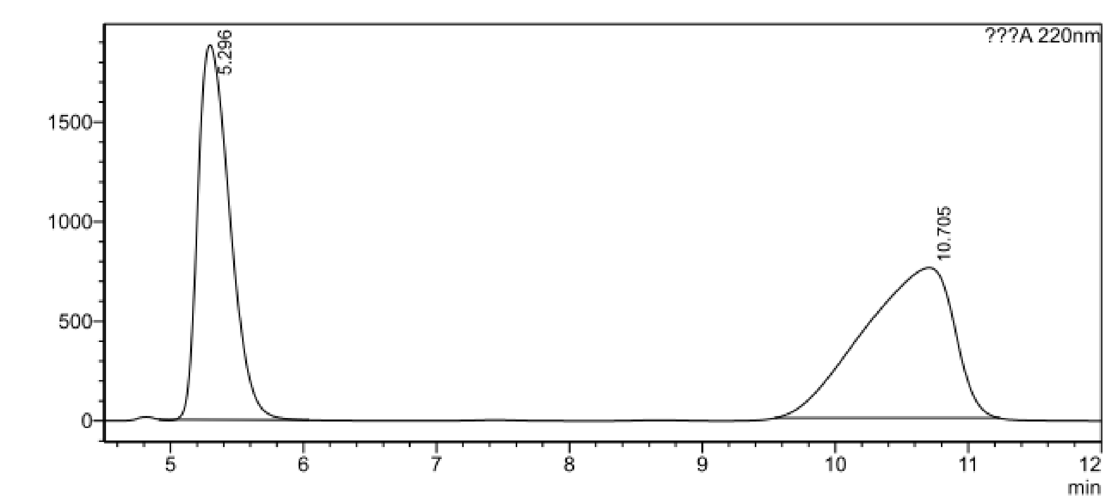

<Peak Table>

???A 220nm

Peak#	Ret. Time	Area	Height	Conc.	Unit	Mark	Name
1	5.296	31423638	1880169	46.808		M	
2	10.705	35709989	755260	53.192		M	
Total		67133627	2635430				

<Chromatogram>

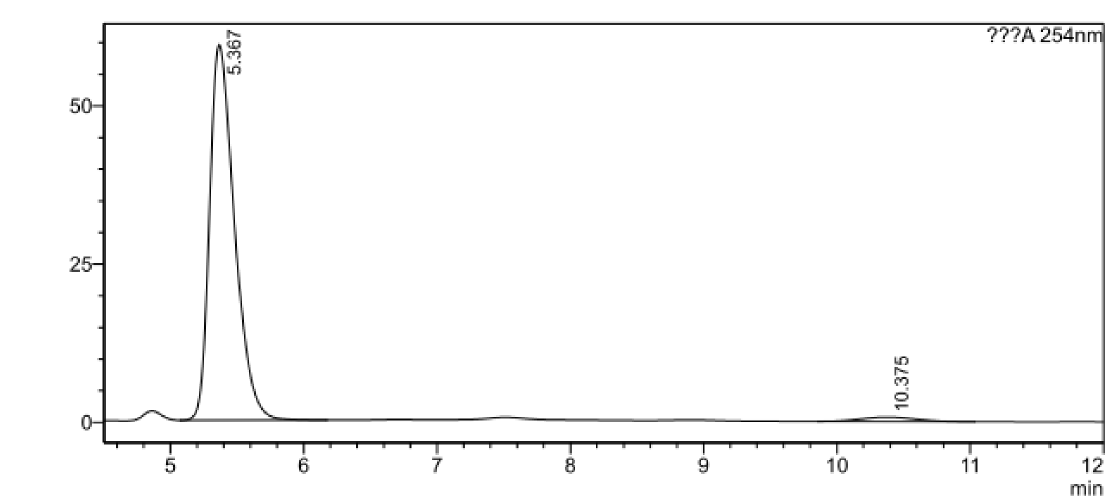

<Peak Table>

???A 254nm

Peak#	Ret. Time	Area	Height	Conc.	Unit	Mark	Name
1	5.367	774255	59290	97.521			
2	10.375	19683	690	2.479			
Total		793938	59979				

(1*R*,2*R*)-2-methyl-1,2-diphenylpent-4-en-1-yl propionate (**8h**)

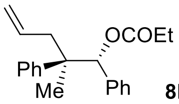

8h

<Chromatogram>

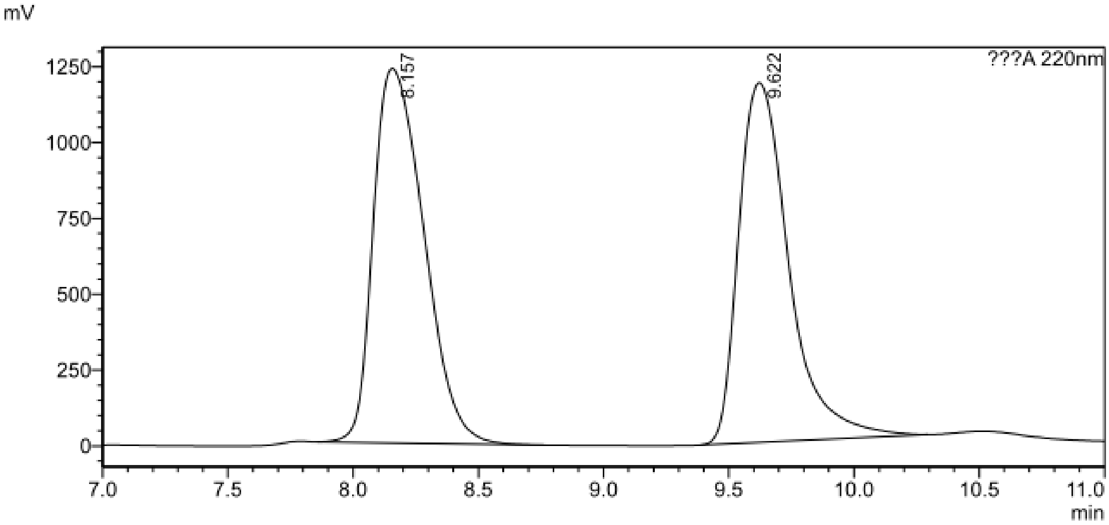

<Peak Table>

???A 220nm

Peak#	Ret. Time	Area	Height	Conc.	Unit	Mark	Name
1	8.157	17350044	1234991	51.097			
2	9.622	16605263	1185197	48.903			
Total		33955307	2420188				

<Chromatogram>

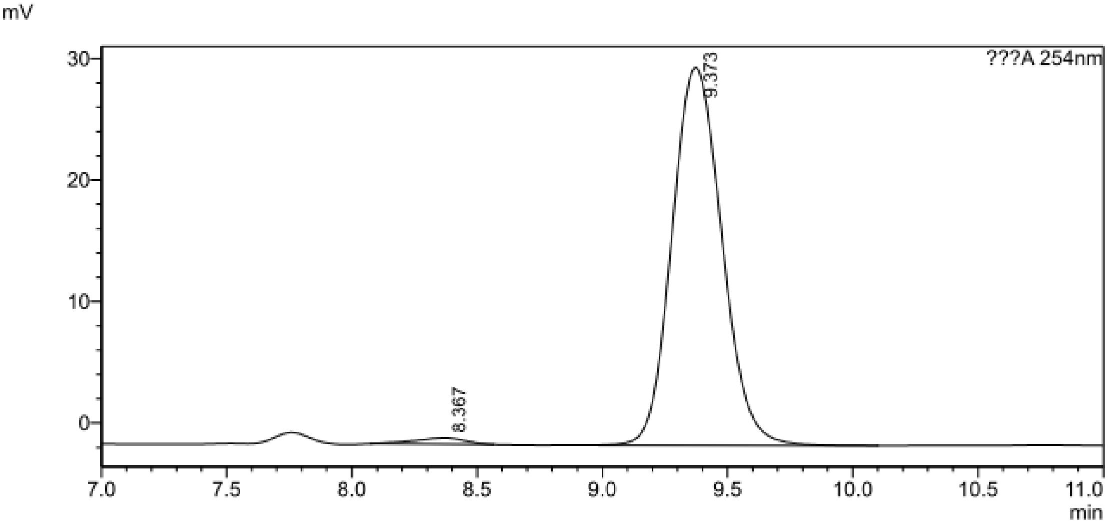

<Peak Table>

???A 254nm

Peak#	Ret. Time	Area	Height	Conc.	Unit	Mark	Name
1	8.367	6899	497	1.572		M	
2	9.373	432037	31107	98.428		V	
Total		438936	31603				

(*R*)-((1*R*,2*S*)-1-methyl-2-phenylcyclopropyl)(phenyl)methanol **(7i)**

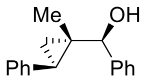

7i

<Chromatogram>

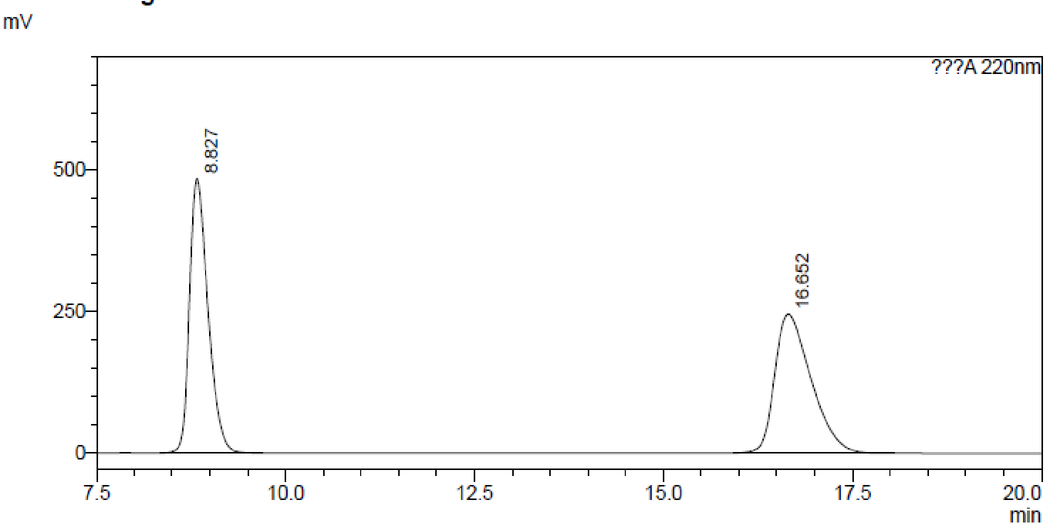

<Peak Table>

???A 220nm

Peak#	Ret. Time	Area	Height	Conc.	Unit	Mark	Name
1	8.827	8238630	484113	49.996		M	
2	16.652	8239883	245169	50.004		M	
Total		16478513	729281				

<Chromatogram>

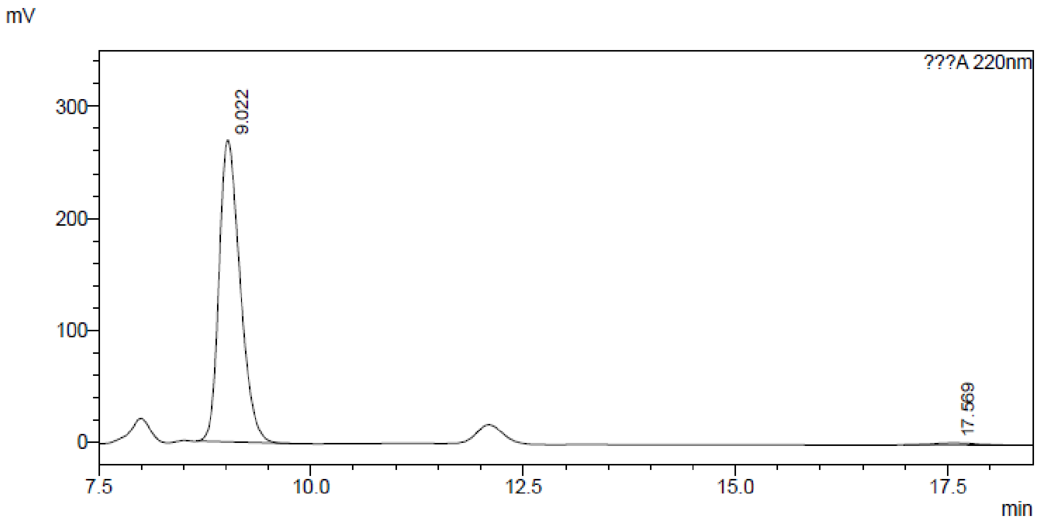

<Peak Table>

???A 220nm

Peak#	Ret. Time	Area	Height	Conc.	Unit	Mark	Name
1	9.022	4656543	269795	98.816		M	
2	17.569	55802	1794	1.184		M	
Total		4712346	271589				

(*S*)-((1*S*,2*R*)-1-methyl-2-phenylcyclopropyl)(phenyl)methyl propionate (**8i**)

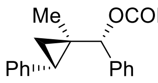

8i

<Chromatogram>

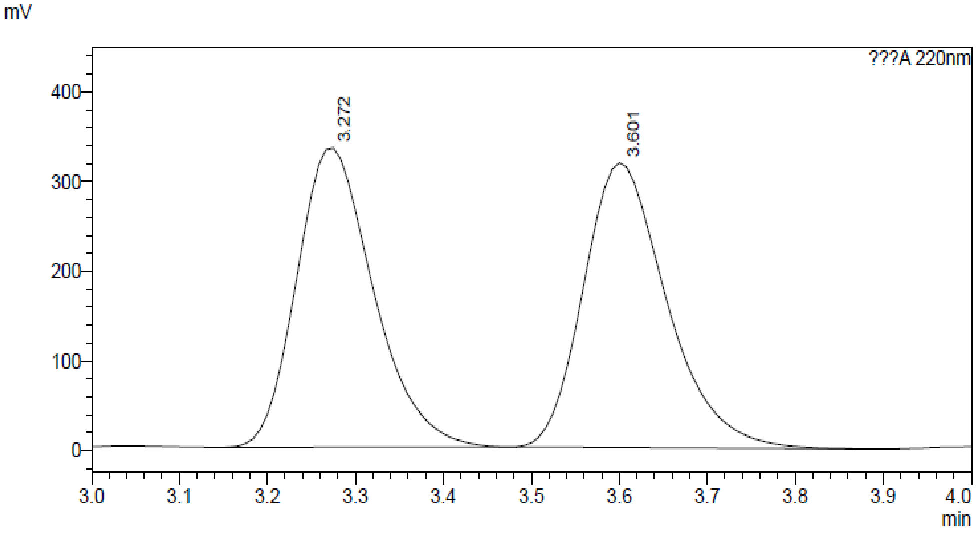

<Peak Table>

???A 220nm

Peak#	Ret. Time	Area	Height	Conc.	Unit	Mark	Name
1	3.272	2032751	334110	49.277		M	
2	3.601	2092390	317817	50.723		M	
Total		4125140	651927				

<Chromatogram>

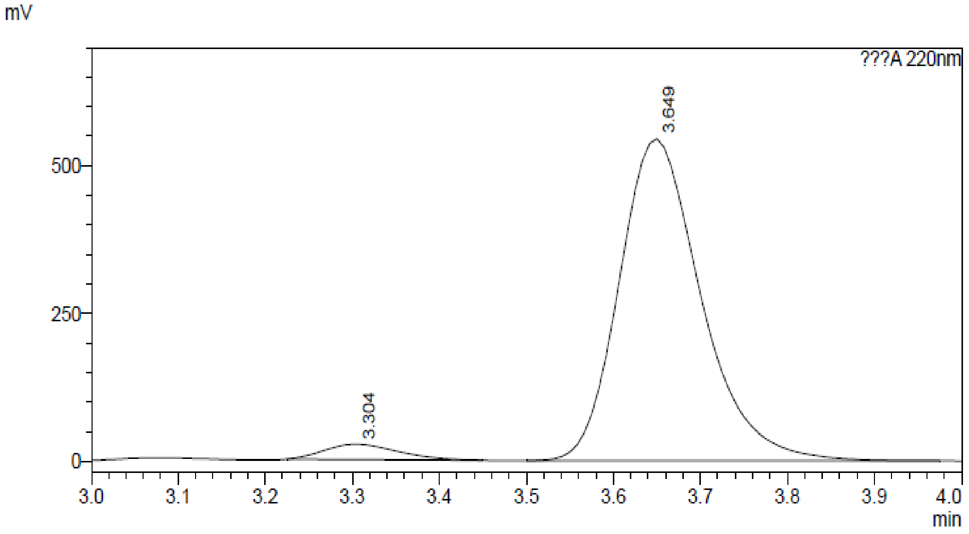

<Peak Table>

???A 220nm

Peak#	Ret. Time	Area	Height	Conc.	Unit	Mark	Name
1	3.304	151627	26062	4.082		M	
2	3.649	3562788	544881	95.918		M	
Total		3714415	570943				

(*R*)-((1*R*,2*S*)-1-methyl-2-phenylcyclopropyl)(phenyl)methanol **(7j)**

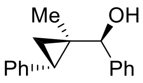

7j

<Chromatogram>

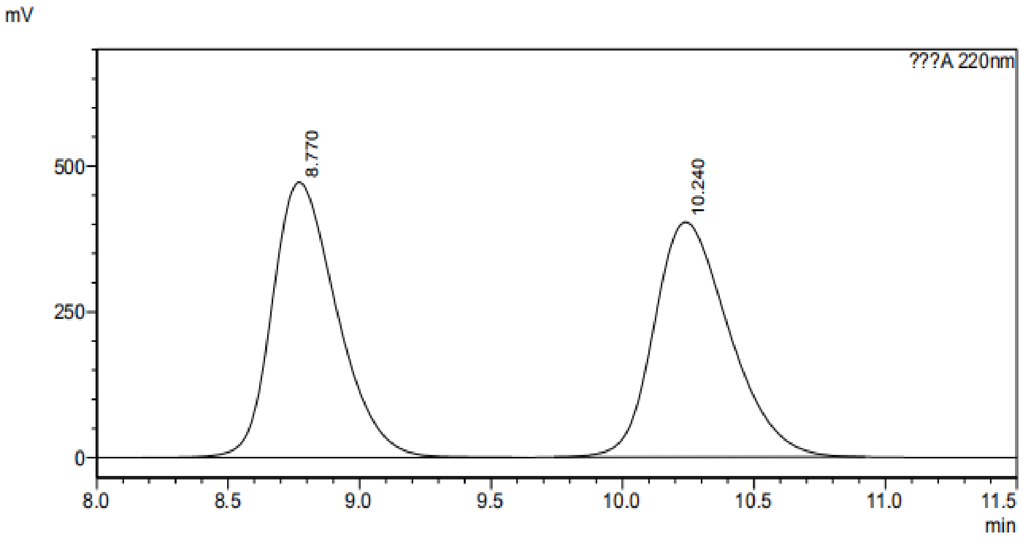

<Peak Table>

???A 220nm

Peak#	Ret. Time	Area	Height	Conc.	Unit	Mark	Name
1	8.770	8085039	472035	50.133			
2	10.240	8042106	403043	49.867		M	
Total		16127145	875078				

<Chromatogram>

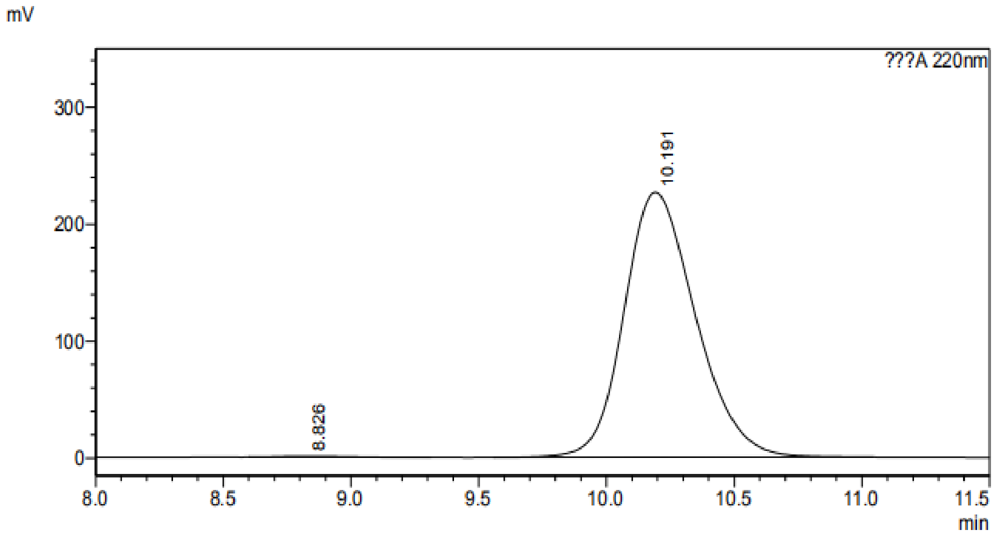

<Peak Table>

???A 220nm

Peak#	Ret. Time	Area	Height	Conc.	Unit	Mark	Name
1	8.826	14156	705	0.319		M	
2	10.191	4419962	226649	99.681		M	
Total		4434118	227353				

(*S*)-((1*S*,2*R*)-1-methyl-2-phenylcyclopropyl)(phenyl)methyl propionate (**8j**)

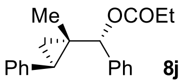

8j

<Chromatogram>

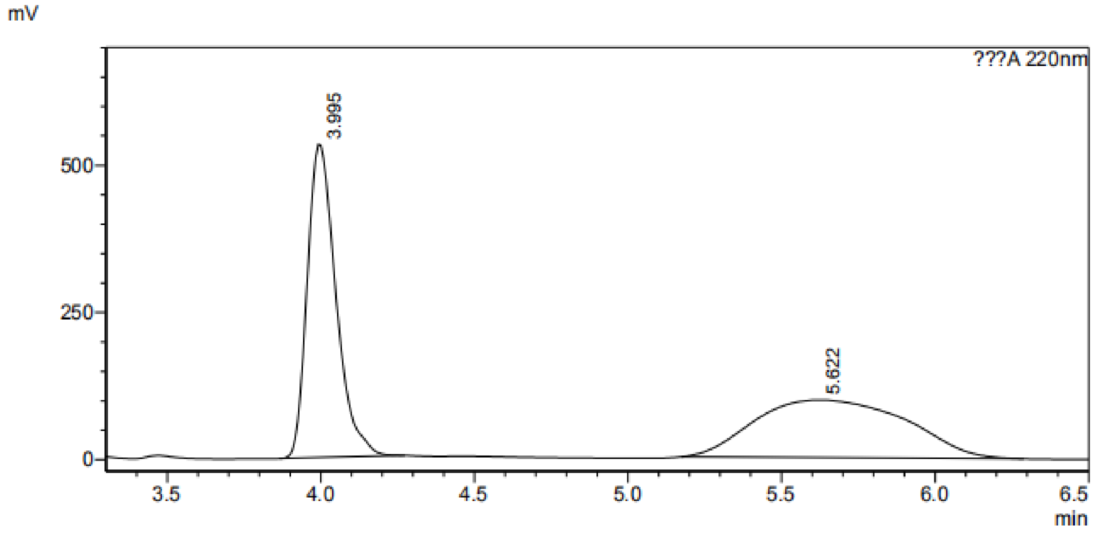

<Peak Table>

???A 220nm

Peak#	Ret. Time	Area	Height	Conc.	Unit	Mark	Name
1	3.995	3461637	531653	50.661		M	
2	5.622	3371305	98000	49.339		M	
Total		6832941	629653				

<Chromatogram>

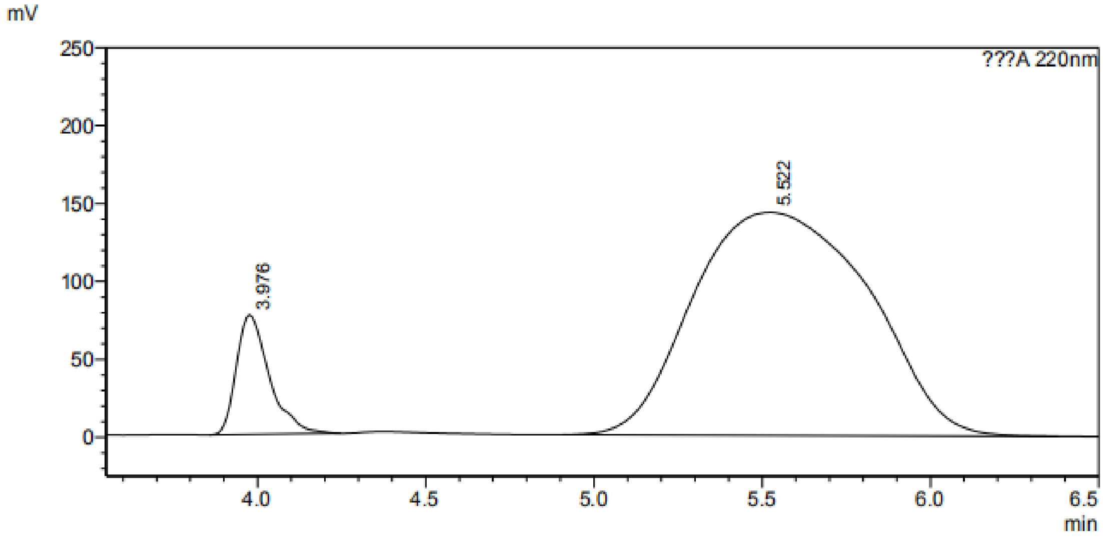

<Peak Table>

???A 220nm

Peak#	Ret. Time	Area	Height	Conc.	Unit	Mark	Name
1	3.976	522937	76633	9.119		M	
2	5.522	5211619	143139	90.881		M	
Total		5734556	219772				

(*S*)-2-((*S*)-hydroxy(naphthalen-2-yl)methyl)-2-methylpent-4-en-1-yl propionate (**7k**)

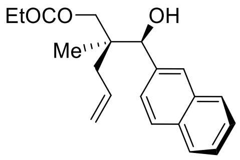

7k

<Chromatogram>

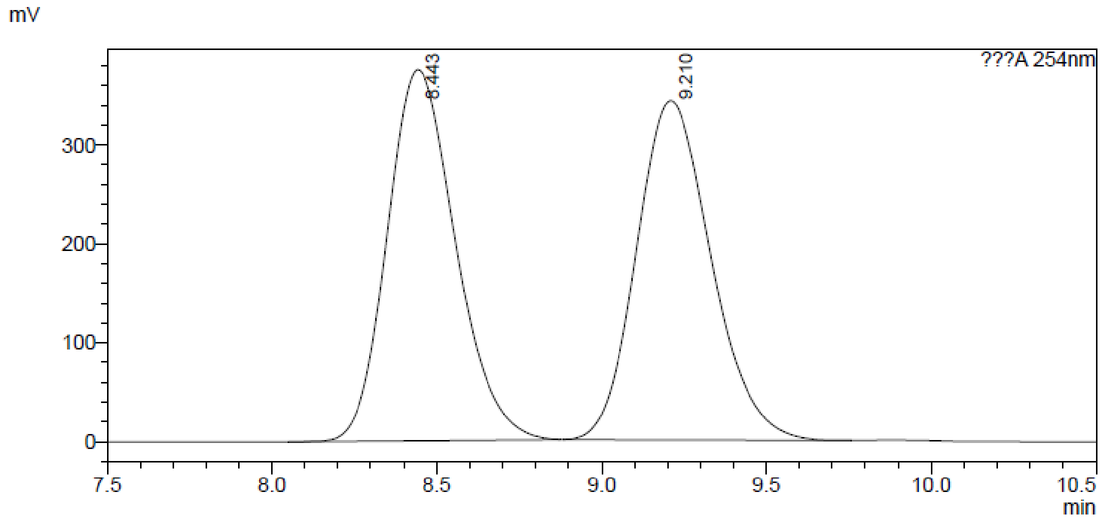

<Peak Table>

???A 254nm

Peak#	Ret. Time	Area	Height	Conc.	Unit	Mark	Name
1	8.443	5398639	375535	49.984		M	
2	9.210	5402159	343518	50.016		M	
Total		10800799	719053				

<Chromatogram>

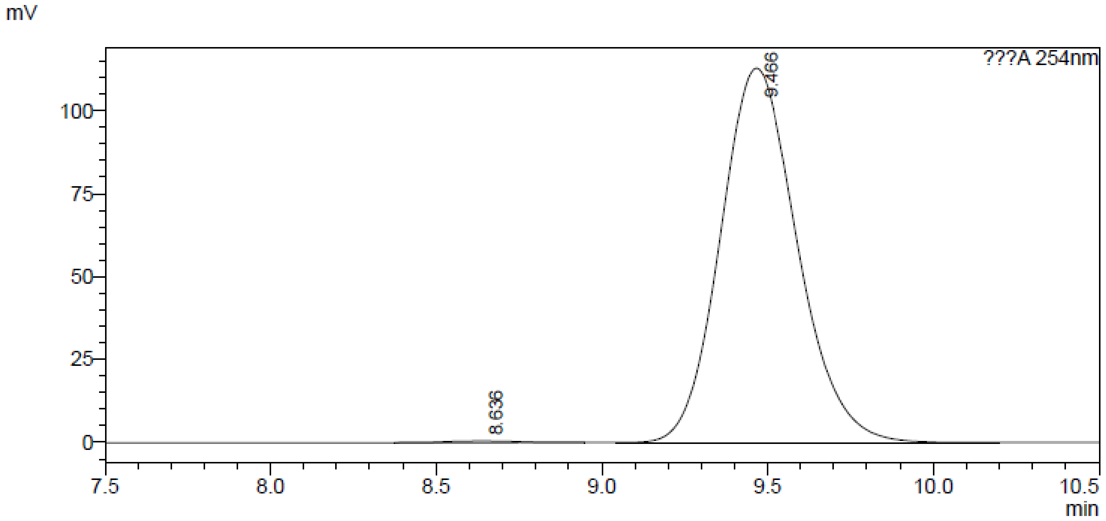

<Peak Table>

???A 254nm

Peak#	Ret. Time	Area	Height	Conc.	Unit	Mark	Name
1	8.636	7324	511	0.399		M	
2	9.466	1828713	113075	99.601		M	
Total		1836037	113587				

(1*R*,2*R*)-2-allyl-2-methyl-1-(naphthalen-2-yl)propane-1,3-diyl dipropionate (**8k**)

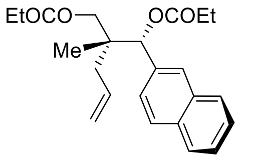

8k

<Chromatogram>

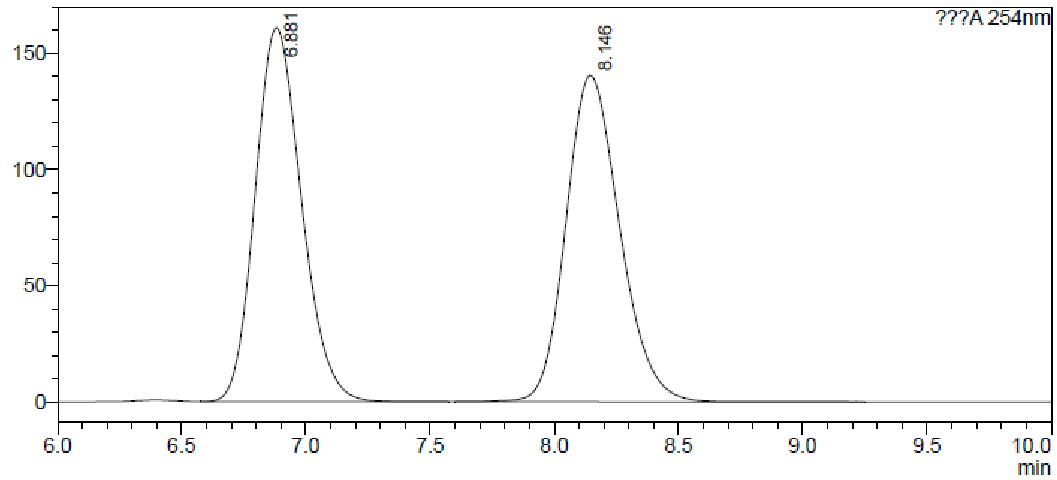

<Peak Table>

???A 254nm

Peak#	Ret. Time	Area	Height	Conc.	Unit	Mark	Name
1	6.881	2099584	161025	49.853			
2	8.146	2111954	140406	50.147		S	
Total		4211538	301431				

<Chromatogram>

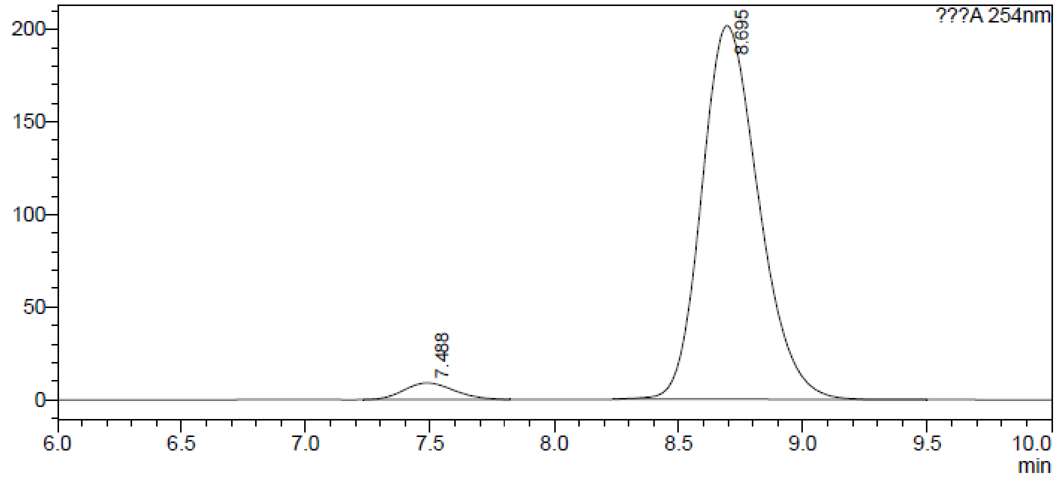

<Peak Table>

???A 254nm

Peak#	Ret. Time	Area	Height	Conc.	Unit	Mark	Name
1	7.488	125137	8929	3.720		M	
2	8.695	3238567	201835	96.280		M	
Total		3363703	210764				

(1*R*,2*S*)-2-methyl-1-(naphthalen-2-yl)-2-vinylpent-4-en-1-ol (**7l**)

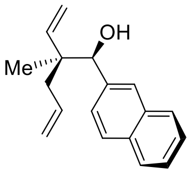

7l

<Chromatogram>

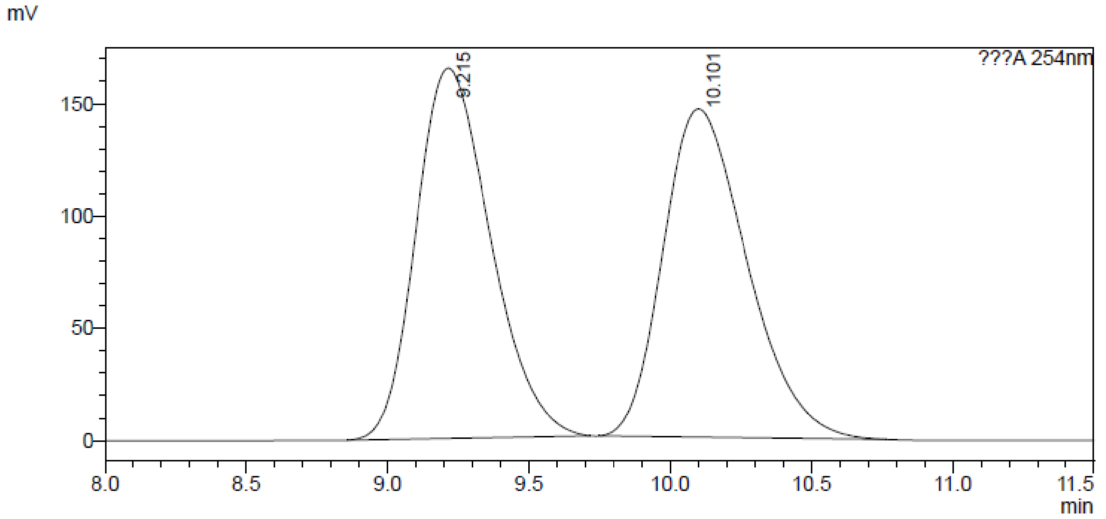

<Peak Table>

???A 254nm

Peak#	Ret. Time	Area	Height	Conc.	Unit	Mark	Name
1	9.215	3025714	164874	50.011		M	
2	10.101	3024327	146091	49.989		M	
Total		6050041	310965				

<Chromatogram>

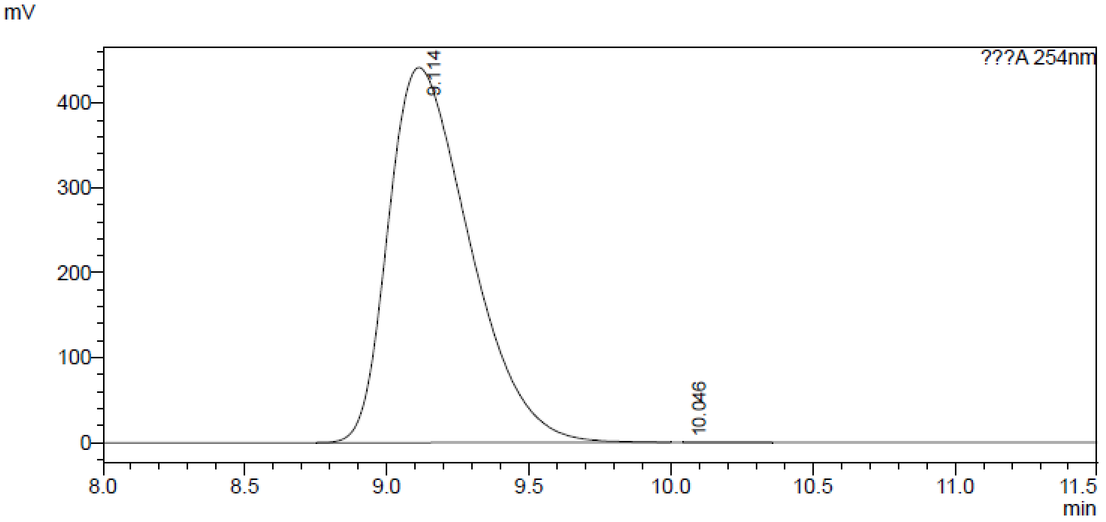

<Peak Table>

???A 254nm

Peak#	Ret. Time	Area	Height	Conc.	Unit	Mark	Name
1	9.114	8775525	441546	99.996		M	
2	10.046	337	4	0.004		M	
Total		8775861	441551				

(1*S*,2*R*)-2-methyl-1-(naphthalen-2-yl)-2-vinylpent-4-en-1-yl propionate (**8l**)

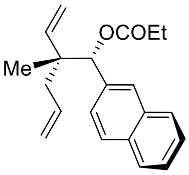

8l

<Chromatogram>

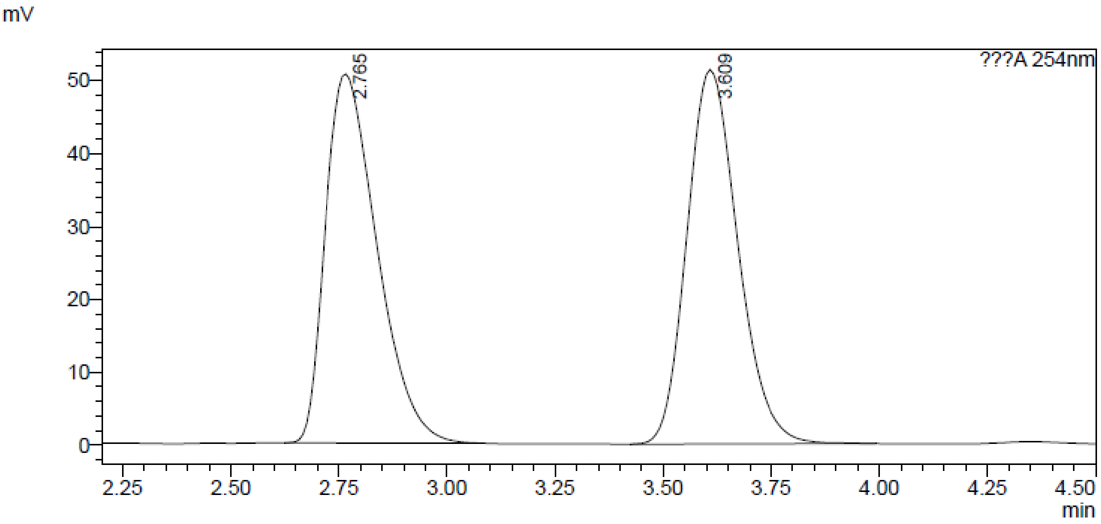

<Peak Table>

???A 254nm

Peak#	Ret. Time	Area	Height	Conc.	Unit	Mark	Name
1	2.765	421836	50684	50.023		M	
2	3.609	421441	51426	49.977		M	
Total		843277	102110				

<Chromatogram>

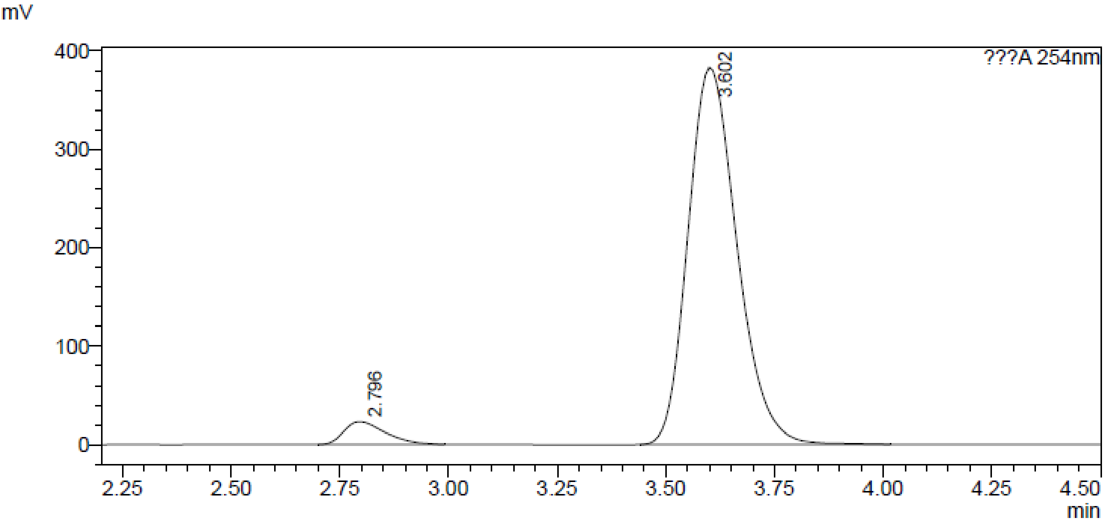

<Peak Table>

???A 254nm

Peak#	Ret. Time	Area	Height	Conc.	Unit	Mark	Name
1	2.796	151392	22806	4.830		M	
2	3.602	2983009	382861	95.170		M	
Total		3134402	405667				

(1*S*,2*S*)-2-ethynyl-2-methyl-1-(naphthalen-2-yl)pent-4-en-1-ol (**7m**)

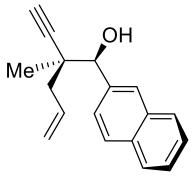

7m

<Chromatogram>

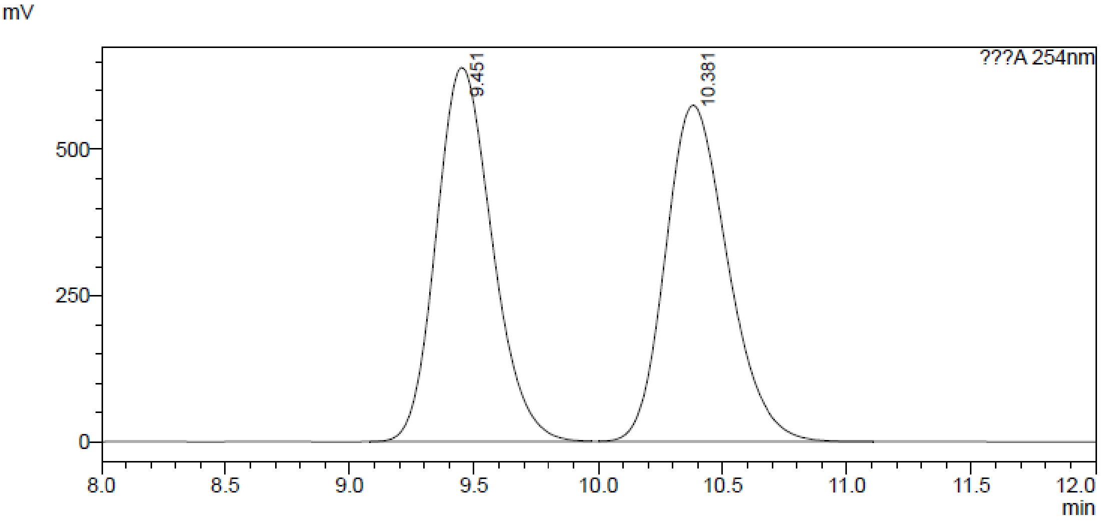

<Peak Table>

???A 254nm

Peak#	Ret. Time	Area	Height	Conc.	Unit	Mark	Name
1	9.451	10005008	640245	49.968		M	
2	10.381	10017999	574798	50.032		M	
Total		20023007	1215043				

<Chromatogram>

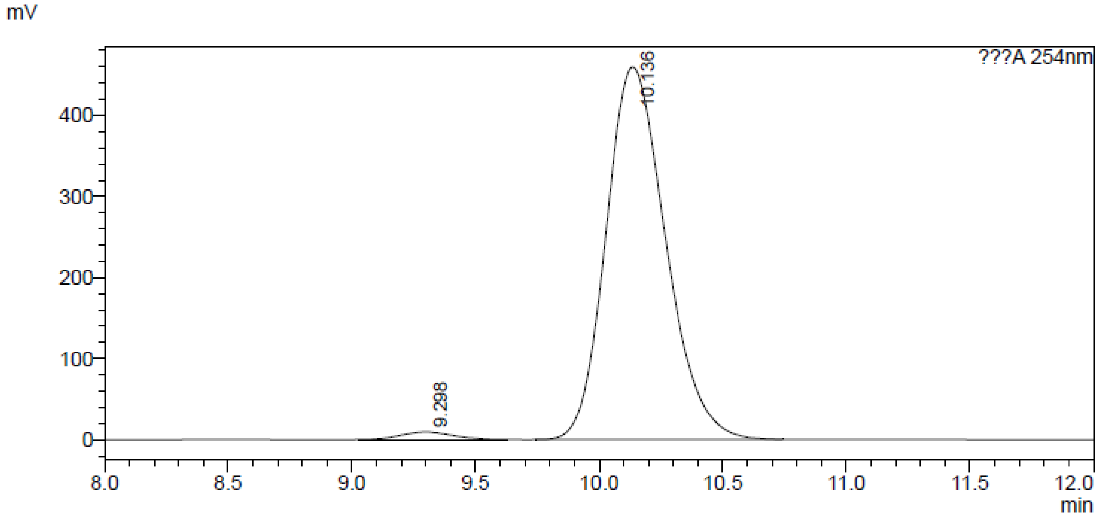

<Peak Table>

???A 254nm

Peak#	Ret. Time	Area	Height	Conc.	Unit	Mark	Name
1	9.298	135360	9229	1.723		M	
2	10.136	7719787	459364	98.277		M	
Total		7855146	468592				

(1*R*,2*R*)-2-ethynyl-2-methyl-1-(naphthalen-2-yl)pent-4-en-1-yl propionate (**8m**)

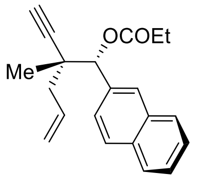

8m

<Chromatogram>

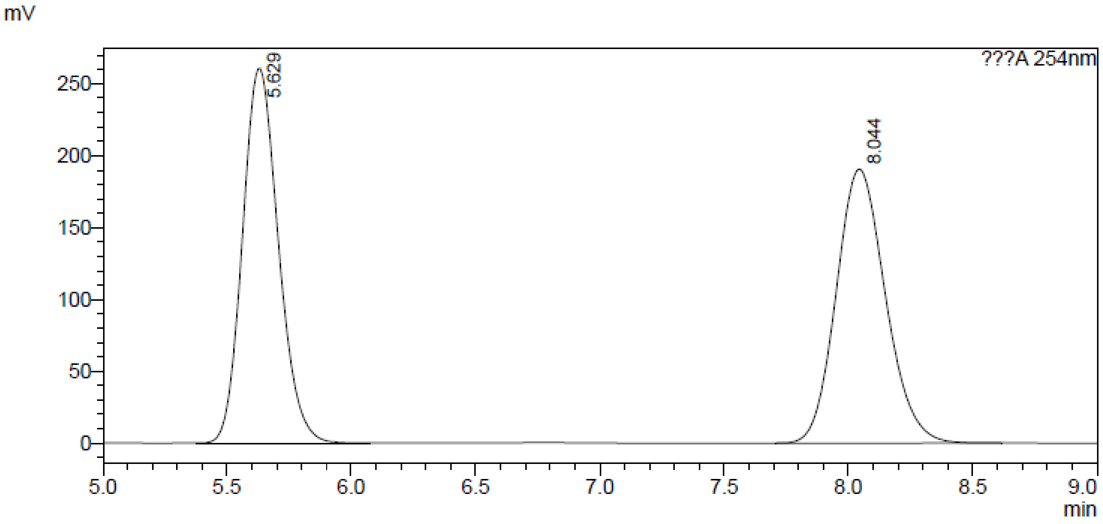

<Peak Table>

???A 254nm

Peak#	Ret. Time	Area	Height	Conc.	Unit	Mark	Name
1	5.629	2614253	260437	49.920		M	
2	8.044	2622638	190568	50.080		M	
Total		5236891	451005				

<Chromatogram>

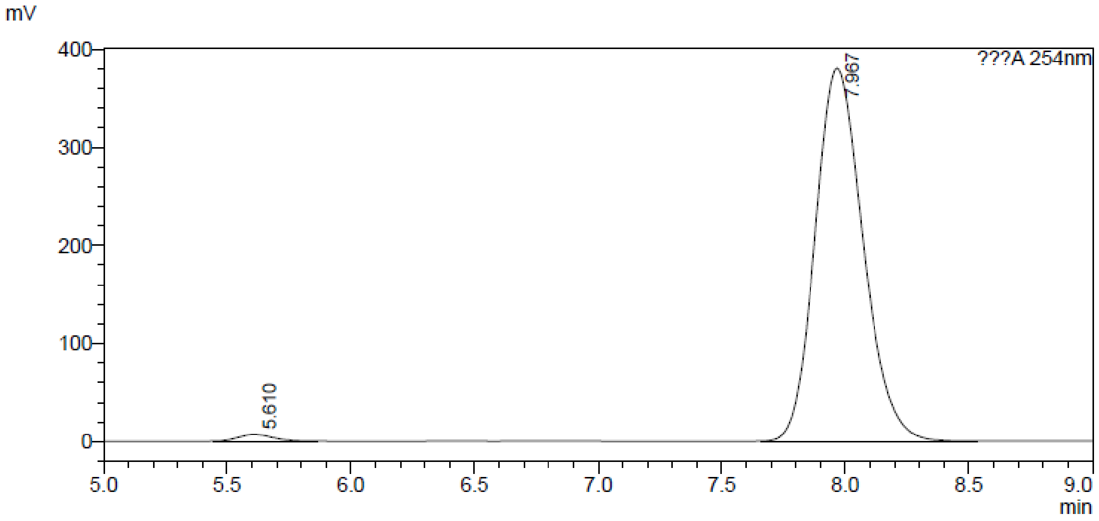

<Peak Table>

???A 254nm

Peak#	Ret. Time	Area	Height	Conc.	Unit	Mark	Name
1	5.610	68030	7106	1.310		M	
2	7.967	5124478	380416	98.690		M	
Total		5192507	387522				

(1*R*,2*R*)-2-benzyl-1-(naphthalen-2-yl)-2-vinylpent-4-en-1-ol (**7n**)

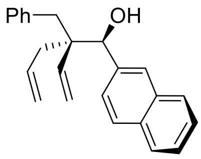

7n

<Chromatogram>

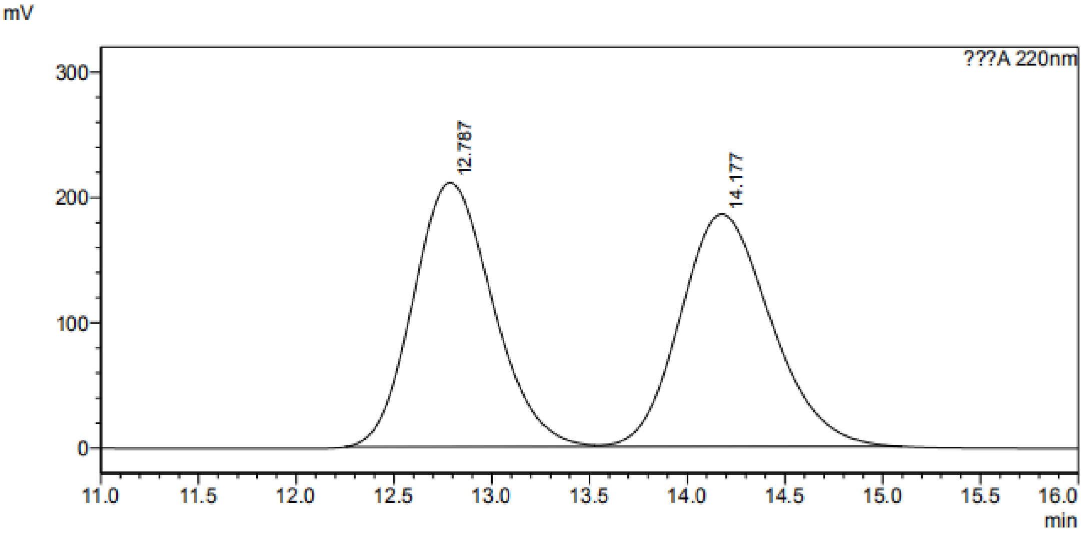

<Peak Table>

???A 220nm

Peak#	Ret. Time	Area	Height	Conc.	Unit	Mark	Name
1	12.787	5978006	210909	49.838			
2	14.177	6016763	185431	50.162		V	
Total		11994769	396340				

<Chromatogram>

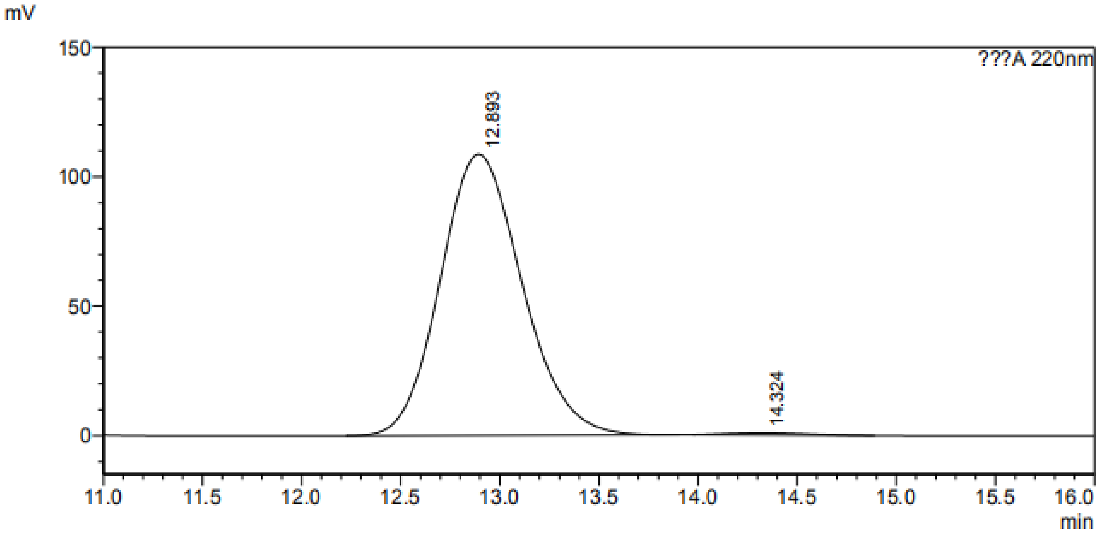

<Peak Table>

???A 220nm

Peak#	Ret. Time	Area	Height	Conc.	Unit	Mark	Name
1	12.893	3122084	108673	99.255		M	
2	14.324	23436	863	0.745		M	
Total		3145520	109536				

(1*S*,2*S*)-2-benzyl-1-(naphthalen-2-yl)-2-vinylpent-4-en-1-yl propionate (**8n**)

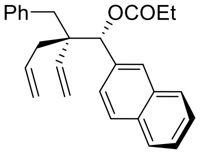

8n

<Chromatogram>

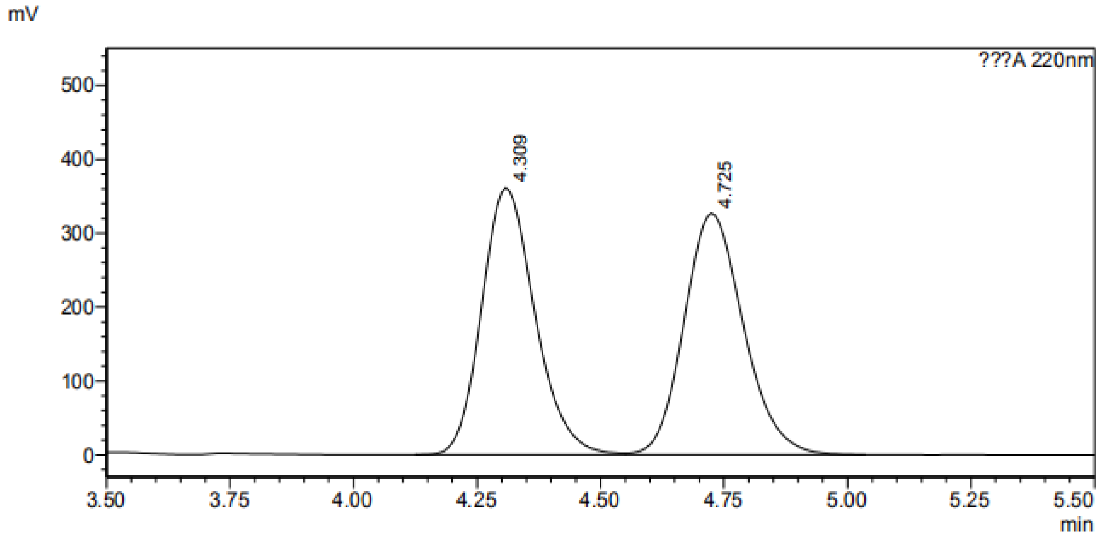

<Peak Table>

???A 220nm

Peak#	Ret. Time	Area	Height	Conc.	Unit	Mark	Name
1	4.309	2705940	360990	49.803			
2	4.725	2727321	326705	50.197		V	
Total		5433261	687696				

<Chromatogram>

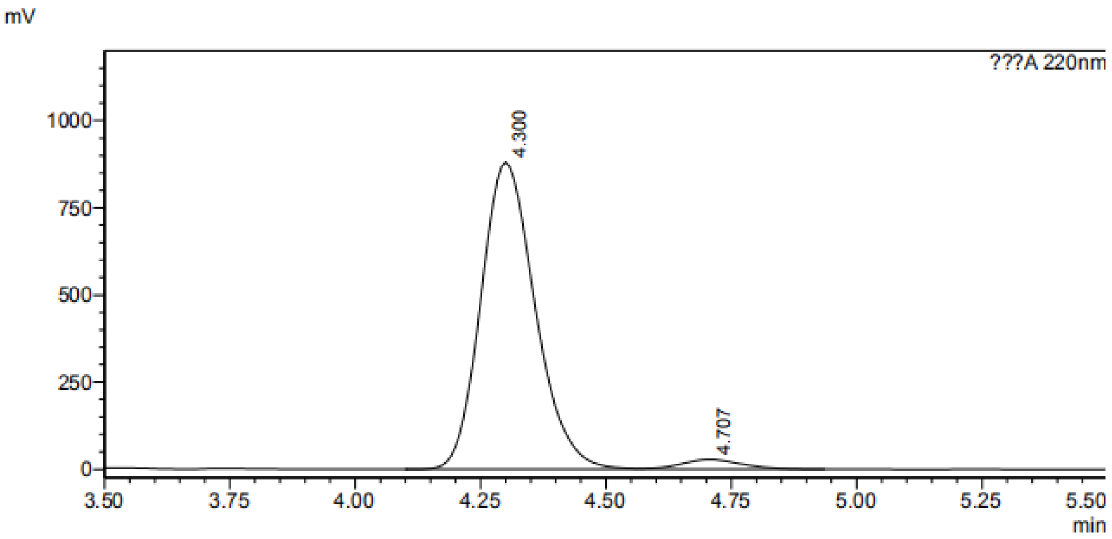

<Peak Table>

???A 220nm

Peak#	Ret. Time	Area	Height	Conc.	Unit	Mark	Name
1	4.300	6628743	880566	96.624			
2	4.707	231610	27760	3.376		V	
Total		6860353	908327				

(*R*)-1-(4-Bromophenyl)-1-(4-chlorophenyl)-1-hydroxypropan-2-one (**9a**)

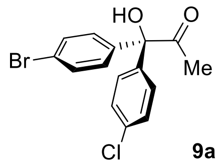

9a

<Chromatogram>

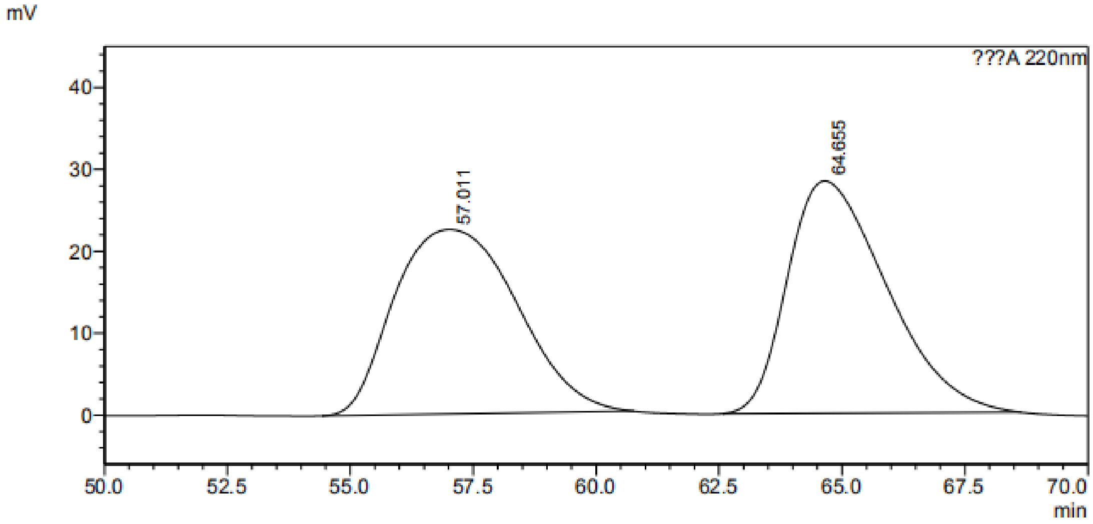

<Peak Table>

???A 220nm

Peak#	Ret. Time	Area	Height	Conc.	Unit	Mark	Name
1	57.011	3916732	22525	49.618		M	
2	64.655	3977002	28338	50.382		M	
Total		7893733	50863				

<Chromatogram>

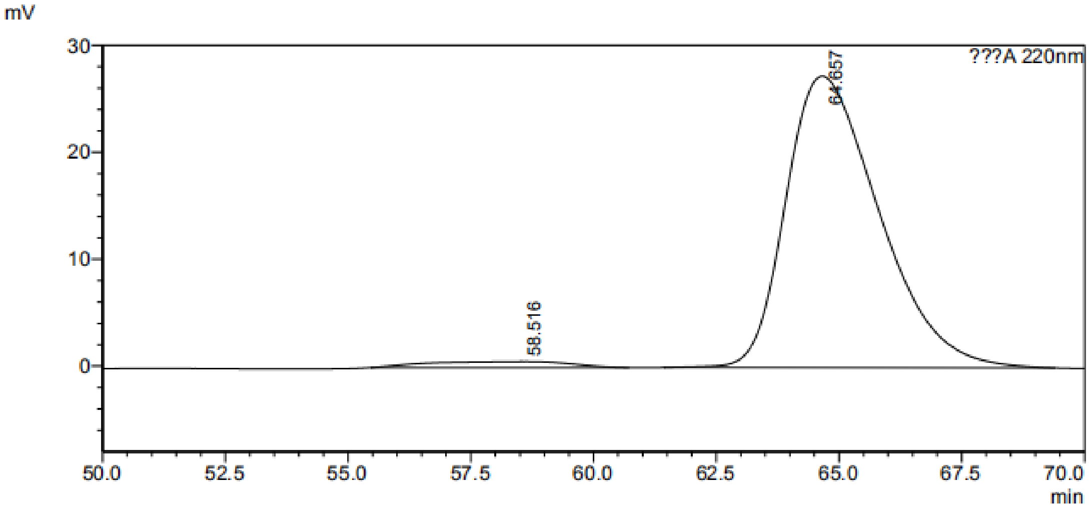

<Peak Table>

???A 220nm

Peak#	Ret. Time	Area	Height	Conc.	Unit	Mark	Name
1	58.516	113514	561	3.050		M	
2	64.657	3607653	27270	96.950		M	
Total		3721167	27831				

(*R*)-2-Hydroxy-2-phenethyl-1-phenylhexan-1-one (**9b**)

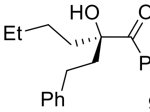

9b

<Chromatogram>

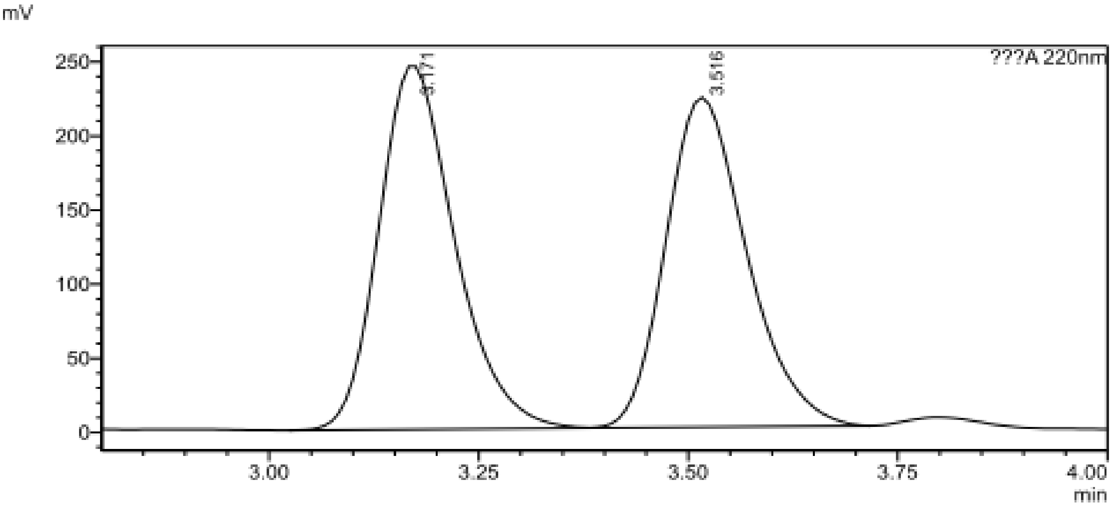

<Peak Table>

???A 220nm

Peak#	Ret. Time	Area	Height	Conc.	Unit	Mark	Name
1	3.171	1547489	244705	50.833			
2	3.516	1496784	222326	49.167		V	
Total		3044273	467032				

<Chromatogram>

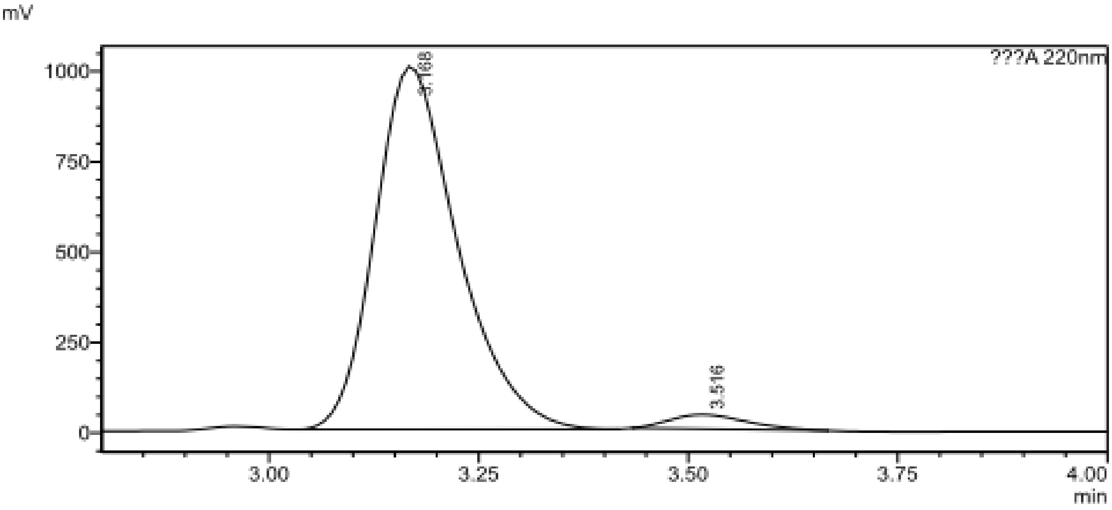

<Peak Table>

???A 220nm

Peak#	Ret. Time	Area	Height	Conc.	Unit	Mark	Name
1	3.168	6903031	1003393	96.399		M	
2	3.516	257861	40028	3.601		M	
Total		7160893	1043421				

(*R*)-2-Hydroxy-2,3-dimethyl-1-phenylbutan-1-one (**9c**)

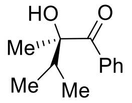

9c

<Chromatogram>

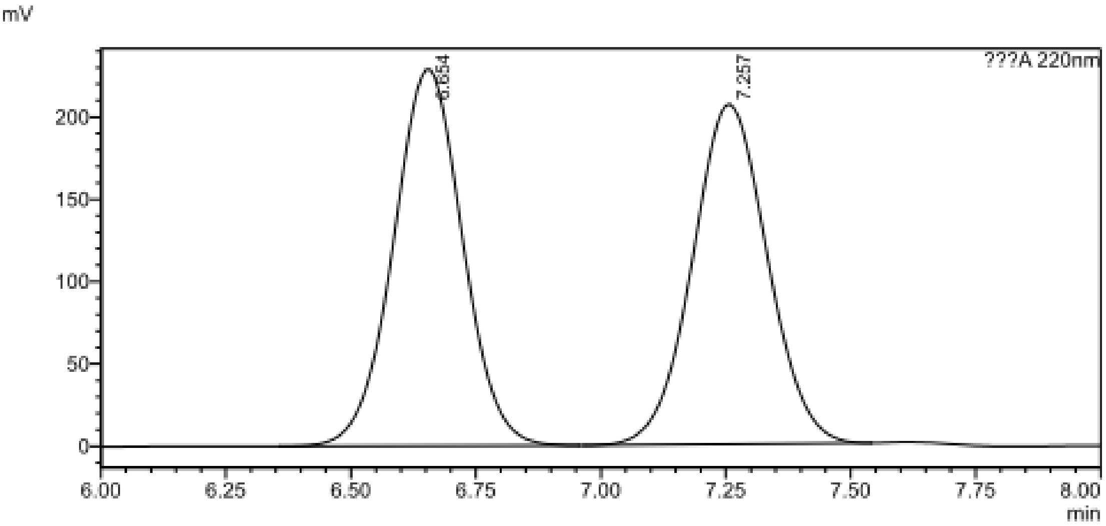

<Peak Table>

???A 220nm

Peak#	Ret. Time	Area	Height	Conc.	Unit	Mark	Name
1	6.654	2209798	228502	50.581			
2	7.257	2159048	206514	49.419			
Total		4368846	435017				

<Chromatogram>

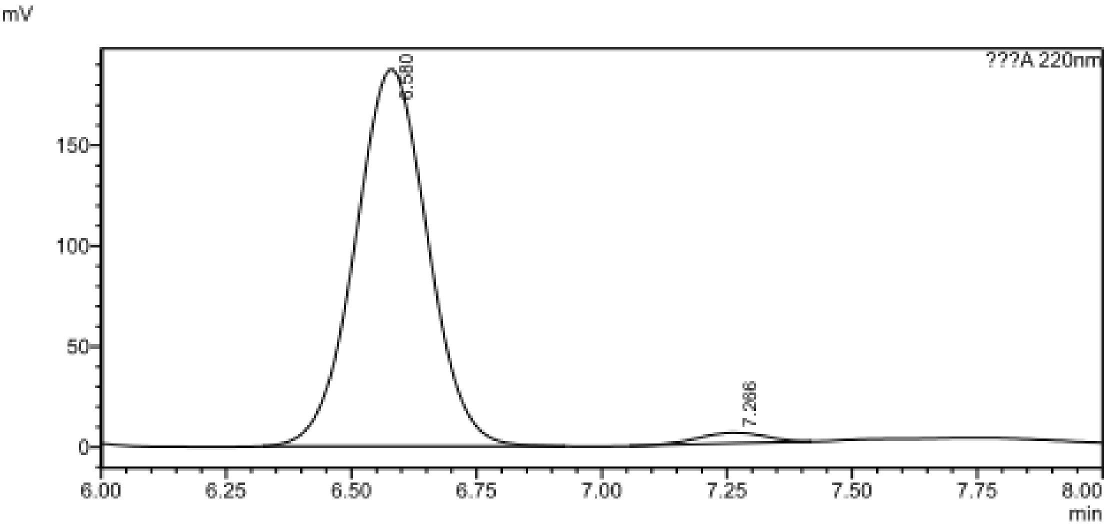

<Peak Table>

???A 220nm

Peak#	Ret. Time	Area	Height	Conc.	Unit	Mark	Name
1	6.580	1889227	187061	97.384		M	
2	7.266	50744	5346	2.616		M	
Total		1939970	192407				

(*S*)-2-(Methoxymethyl)-2-methyl-1-phenylpent-4-en-1-one (**9d**)

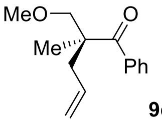

9d

<Chromatogram>

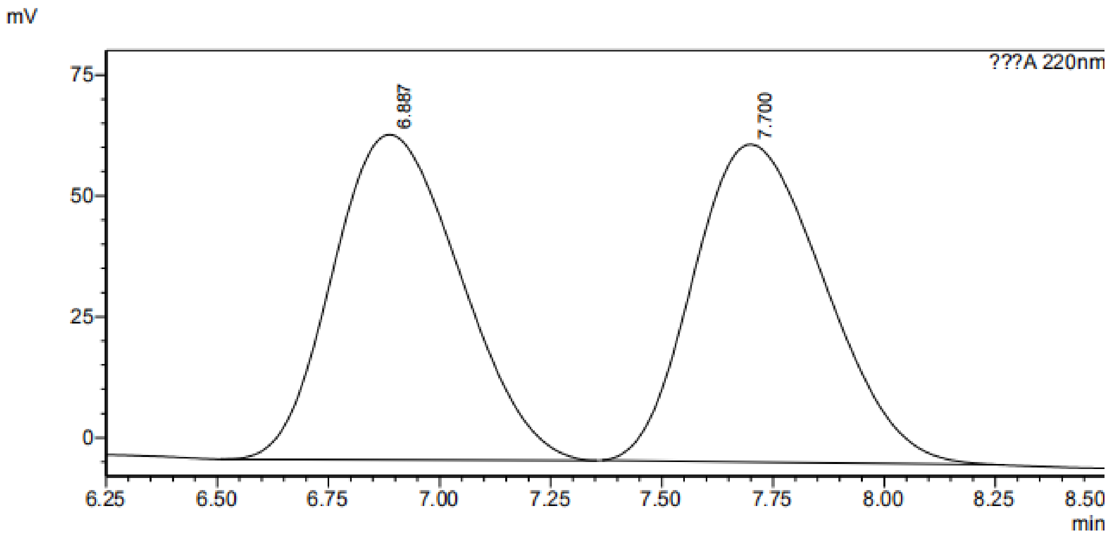

<Peak Table>

???A 220nm

Peak#	Ret. Time	Area	Height	Conc.	Unit	Mark	Name
1	6.887	1338624	67223	49.784			
2	7.700	1350242	65685	50.216		M	
Total		2688865	132907				

<Chromatogram>

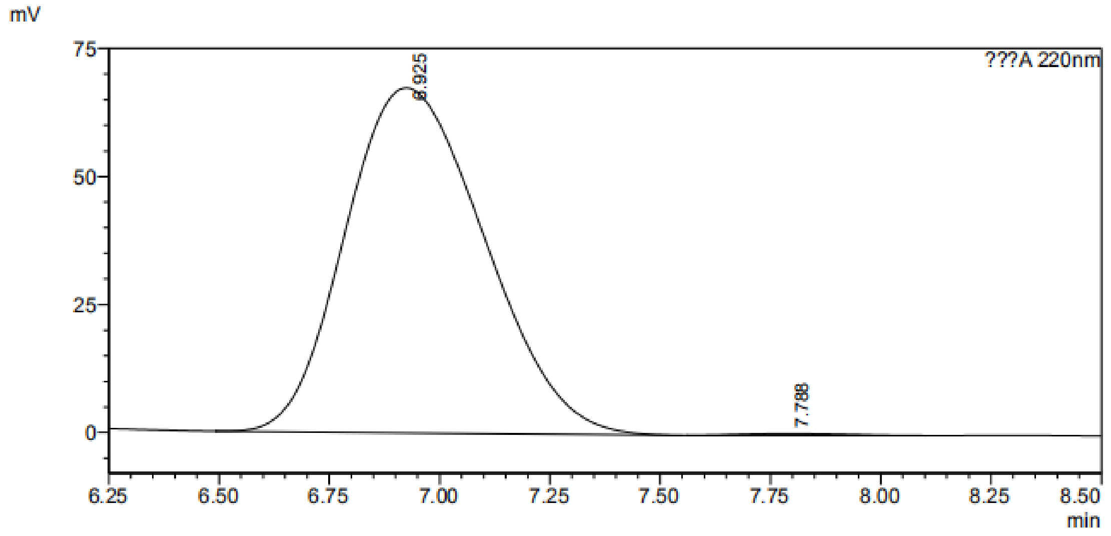

<Peak Table>

???A 220nm

Peak#	Ret. Time	Area	Height	Conc.	Unit	Mark	Name
1	6.925	1481247	67477	99.604			
2	7.788	5888	360	0.396		M	
Total		1487135	67837				

(*R*)-2-Benzyl-1-(naphthalen-2-yl)-2-vinylpent-4-en-1-one (**9e**)

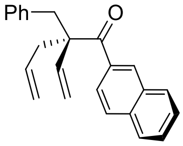

9e

<Chromatogram>

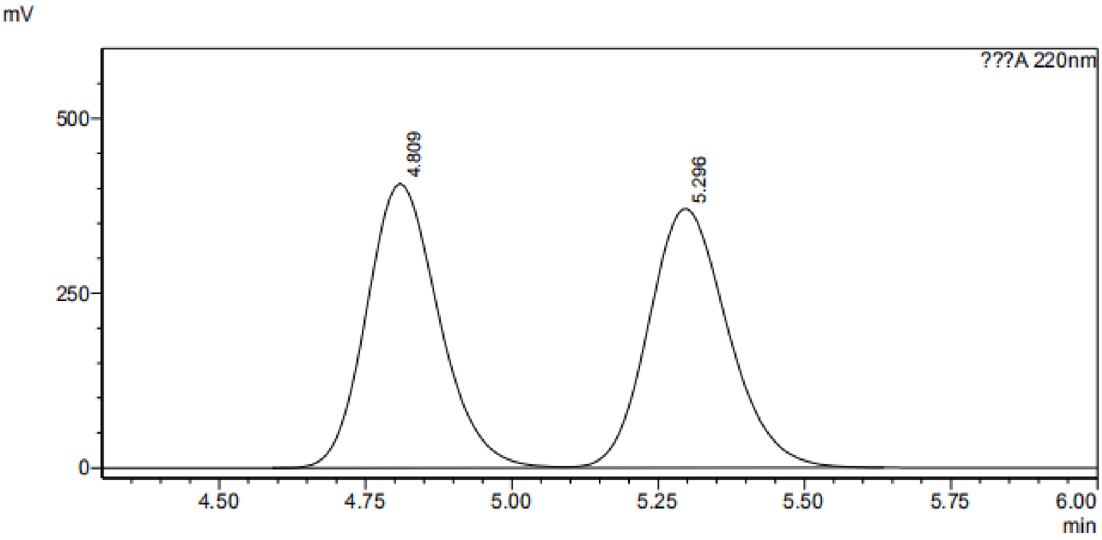

<Peak Table>

???A 220nm

Peak#	Ret. Time	Area	Height	Conc.	Unit	Mark	Name
1	4.809	3493663	406903	50.014			
2	5.296	3491711	370437	49.986		V	
Total		6985374	777340				

<Chromatogram>

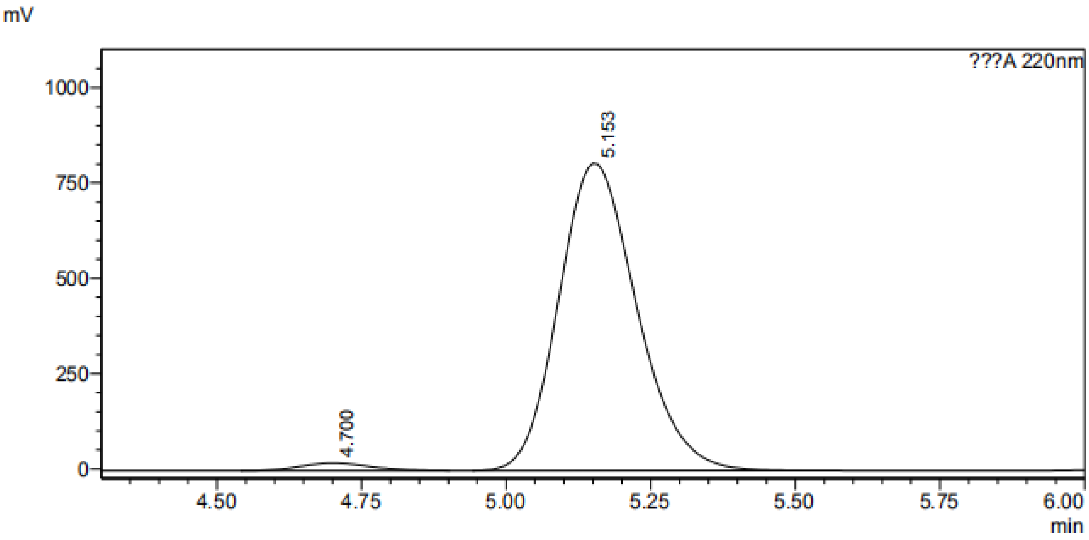

<Peak Table>

???A 220nm

Peak#	Ret. Time	Area	Height	Conc.	Unit	Mark	Name
1	4.700	169064	20148	2.188		M	
2	5.153	7557179	806392	97.812		M	
Total		7726244	826540				

(*S*)-2-Benzoyl-2-(4-fluorobenzyl)butyl propionate (**9f**)

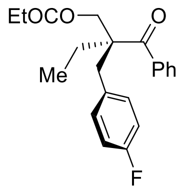

9f

<Chromatogram>

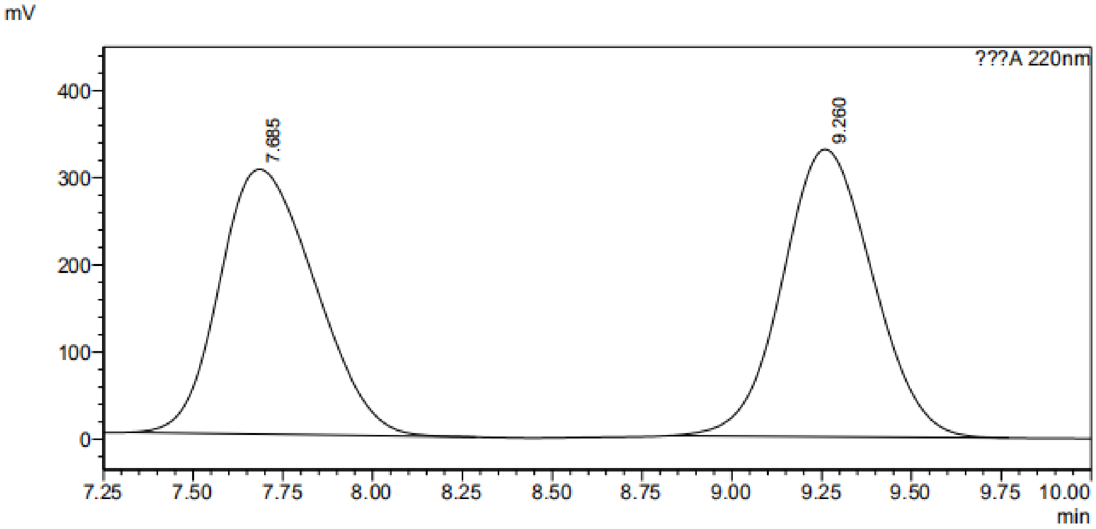

<Peak Table>

???A 220nm

Peak#	Ret. Time	Area	Height	Conc.	Unit	Mark	Name
1	7.685	5627089	303912	49.278			
2	9.260	5792004	329863	50.722			
Total		11419093	633775				

<Chromatogram>

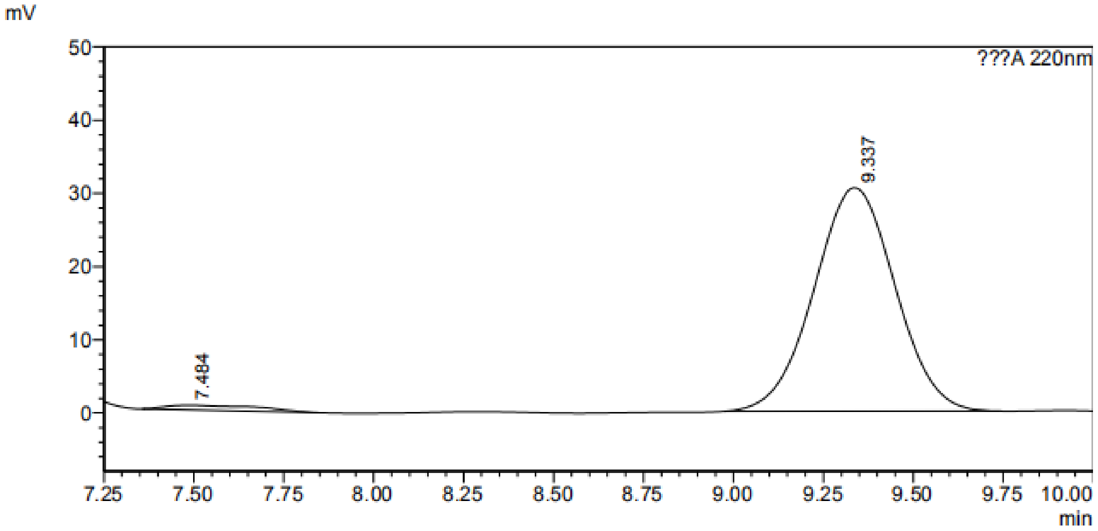

<Peak Table>

???A 220nm

Peak#	Ret. Time	Area	Height	Conc.	Unit	Mark	Name
1	7.484	12462	646	2.530		M	
2	9.337	480126	30513	97.470		M	
Total		492588	31159				

(*S*)-2-Methyl-3-oxo-2,3-diphenylpropyl propionate (**9g**)

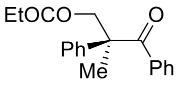

9g

<Chromatogram>

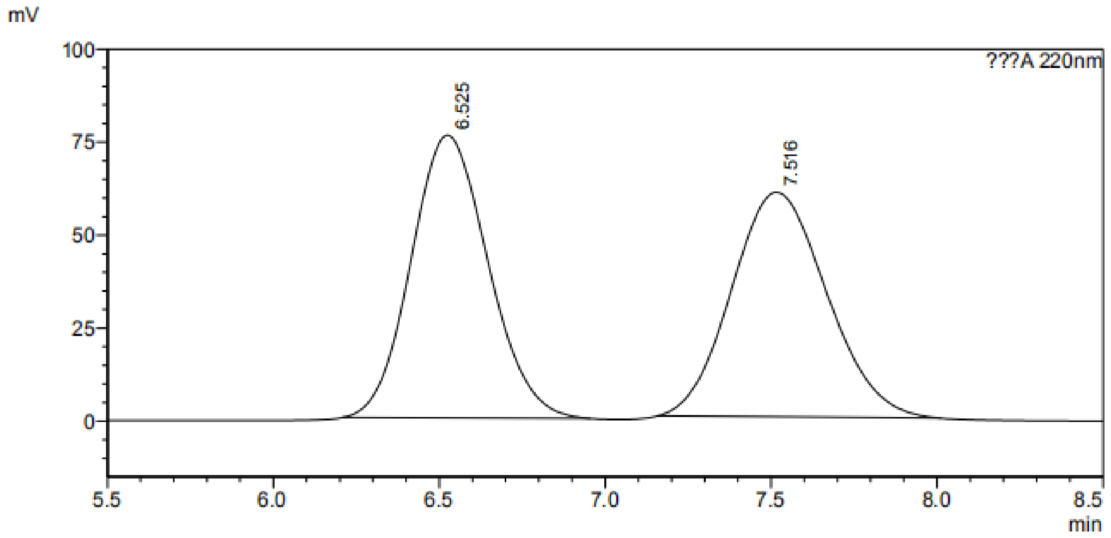

<Peak Table>

???A 220nm

Peak#	Ret. Time	Area	Height	Conc.	Unit	Mark	Name
1	6.525	1229003	76041	50.448			
2	7.516	1207178	60440	49.552			
Total		2436181	136481				

<Chromatogram>

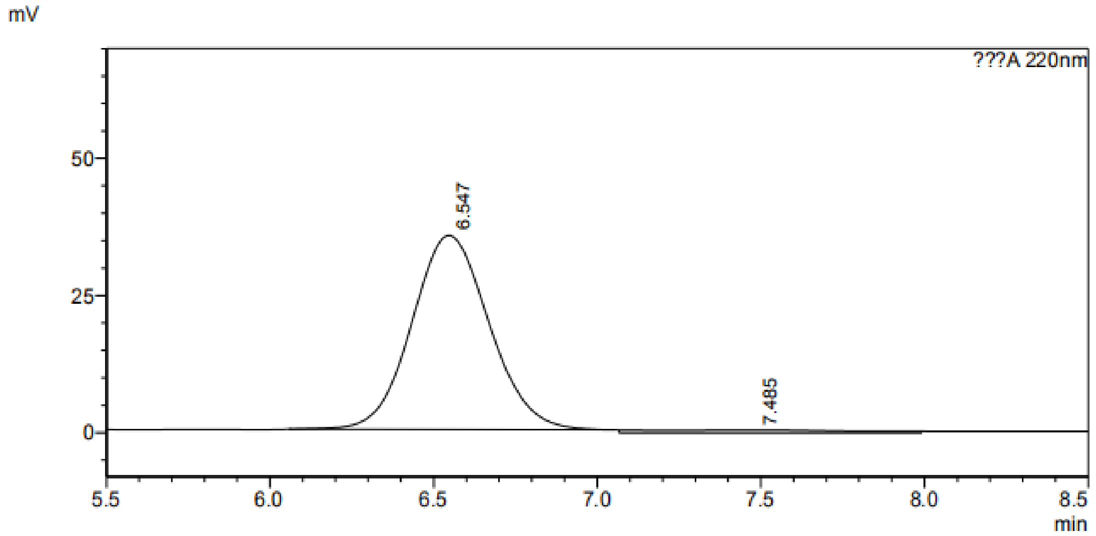

<Peak Table>

???A 220nm

Peak#	Ret. Time	Area	Height	Conc.	Unit	Mark	Name
1	6.547	584186	35392	96.629		M	
2	7.485	20380	471	3.371		M	
Total		604566	35863				

Methyl (*R*)-2-(4-chlorobenzoyl)-2-(naphthalen-2-ylmethyl)pent-4-enoate (**9h**)

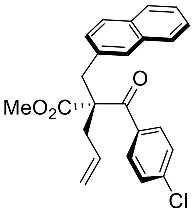

9h

<Chromatogram>

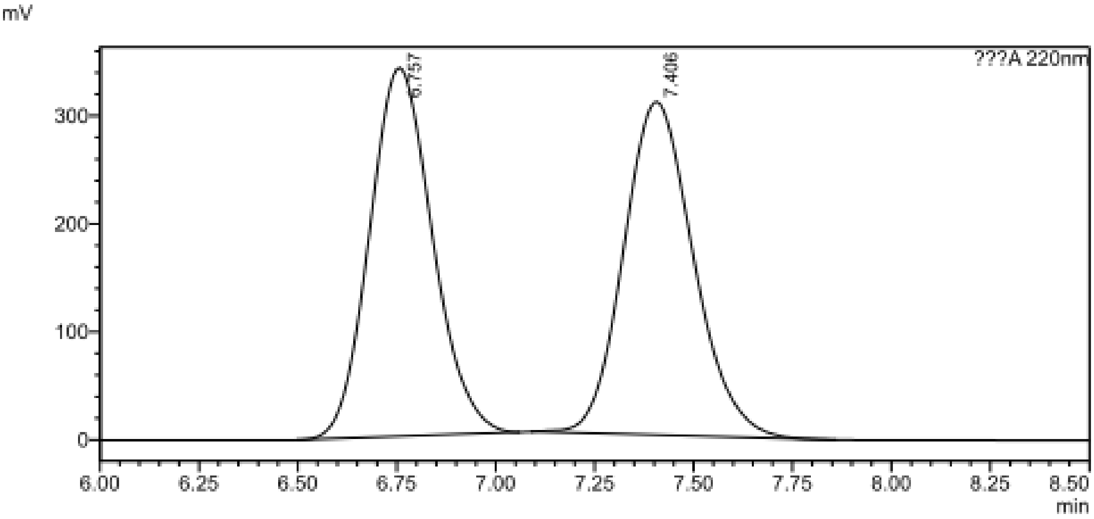

<Peak Table>

???A 220nm

Peak#	Ret. Time	Area	Height	Conc.	Unit	Mark	Name
1	6.757	3776935	341328	49.914			
2	7.406	3789907	308512	50.086		M	
Total		7566842	649840				

<Chromatogram>

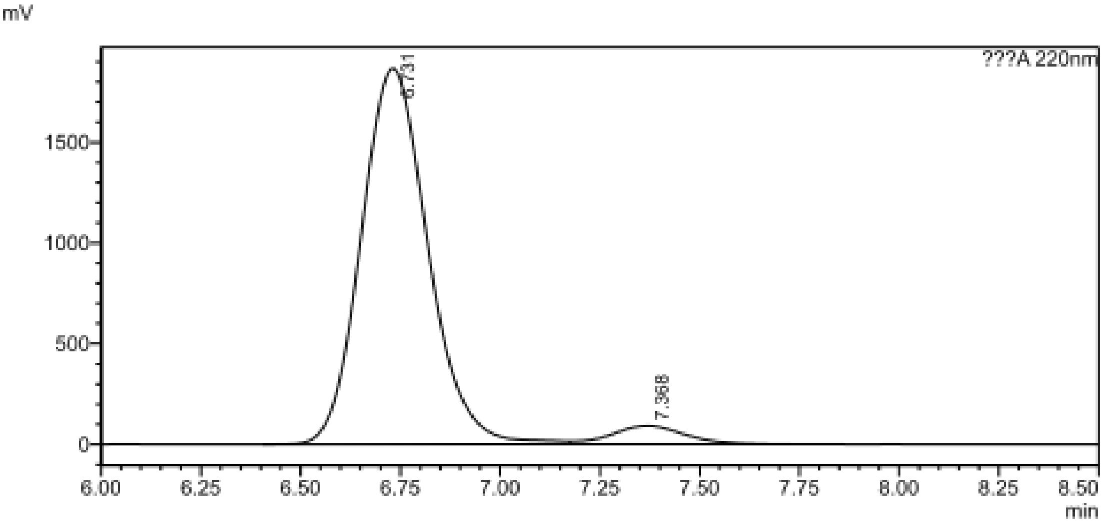

<Peak Table>

???A 220nm

Peak#	Ret. Time	Area	Height	Conc.	Unit	Mark	Name
1	6.731	21740369	1868313	94.907			
2	7.368	1166660	92011	5.093		V	
Total		22907029	1960324				

(*R*)-Methyl 2-(2-naphthoyl)-2-benzylpent-4-enoate (**9i**)

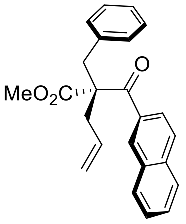

9i

<Chromatogram>

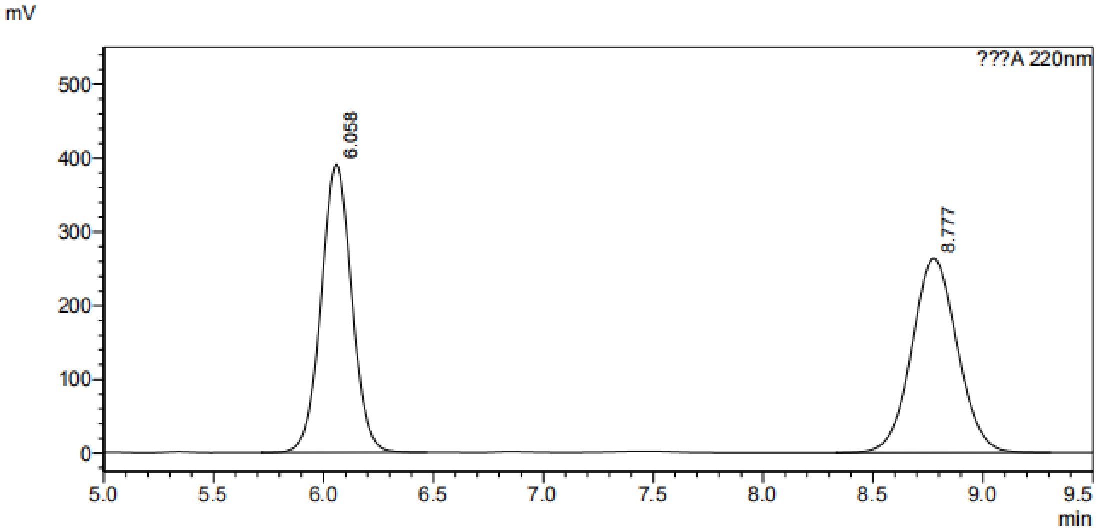

<Peak Table>

???A 220nm

Peak#	Ret. Time	Area	Height	Conc.	Unit	Mark	Name
1	6.058	3793379	390626	49.968			
2	8.777	3798237	263429	50.032			
Total		7591617	654054				

<Chromatogram>

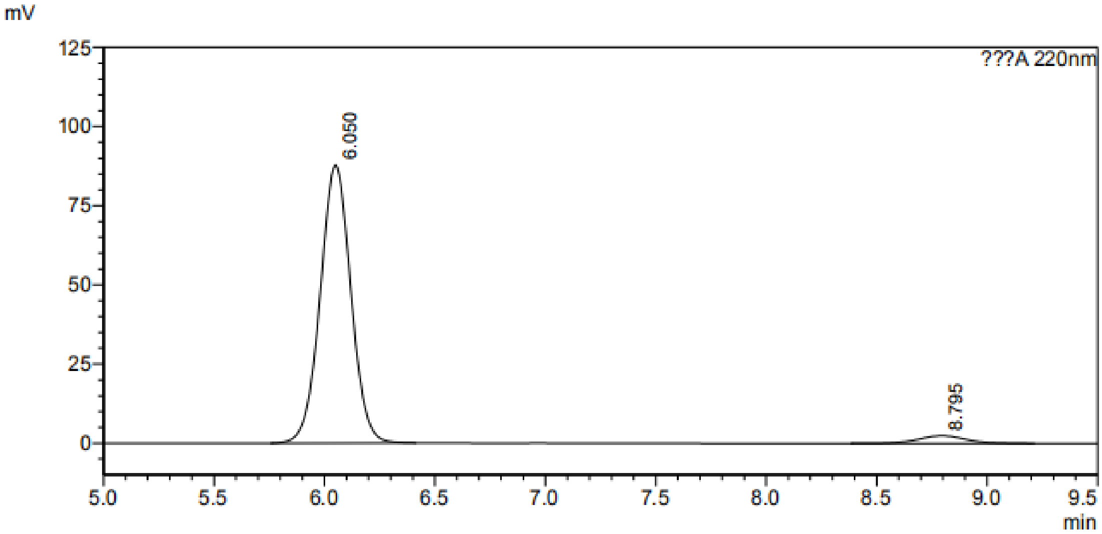

<Peak Table>

???A 220nm

Peak#	Ret. Time	Area	Height	Conc.	Unit	Mark	Name
1	6.050	847811	87698	95.906			
2	8.795	36187	2437	4.094		M	
Total		883998	90135				

((2*R*,3*R*)-3-Hexyl-3-pentyloxiran-2-yl)(phenyl)methanone (**9j**)

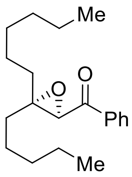

9j

<Chromatogram>

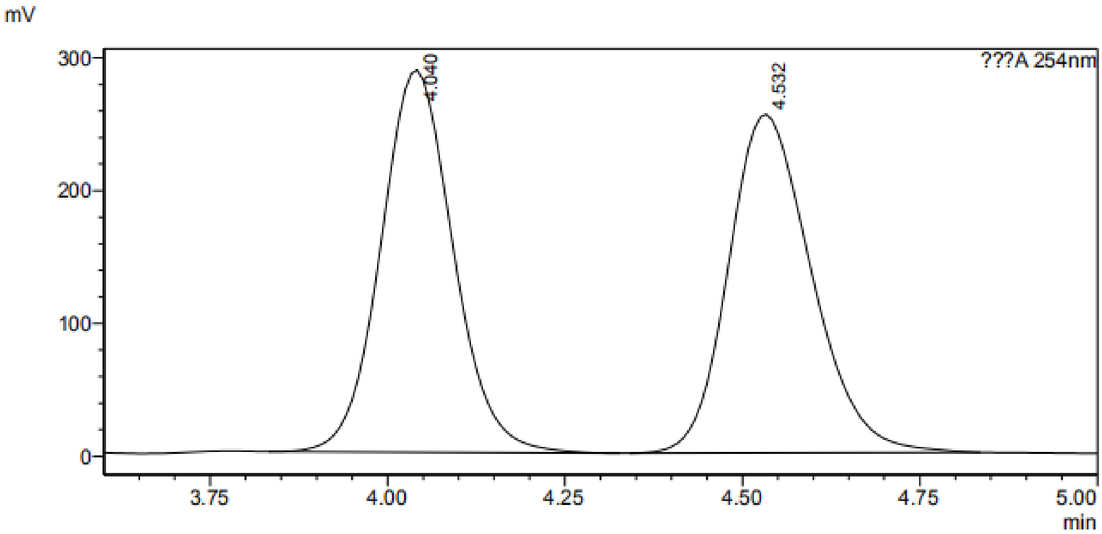

<Peak Table>

???A 254nm

Peak#	Ret. Time	Area	Height	Conc.	Unit	Mark	Name
1	4.040	2048382	287771	49.678			
2	4.532	2074908	254943	50.322		M	
Total		4123290	542715				

<Chromatogram>

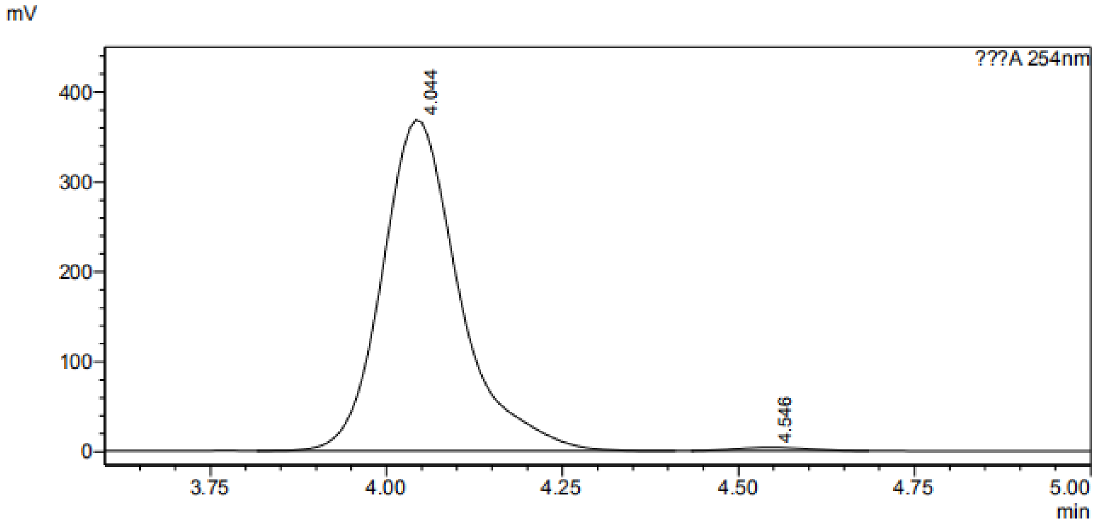

<Peak Table>

???A 254nm

Peak#	Ret. Time	Area	Height	Conc.	Unit	Mark	Name
1	4.044	2811072	368492	99.009			
2	4.546	28126	4043	0.991		M	
Total		2839198	372535				

Naphthalen-1-yl((2*R*,3*R*)-3-phenyl-3-(*p*-tolyl)oxiran-2-yl)methanone (**9k**)

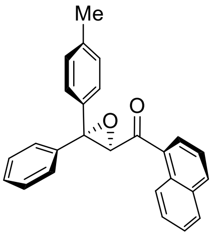

9k

<Chromatogram>

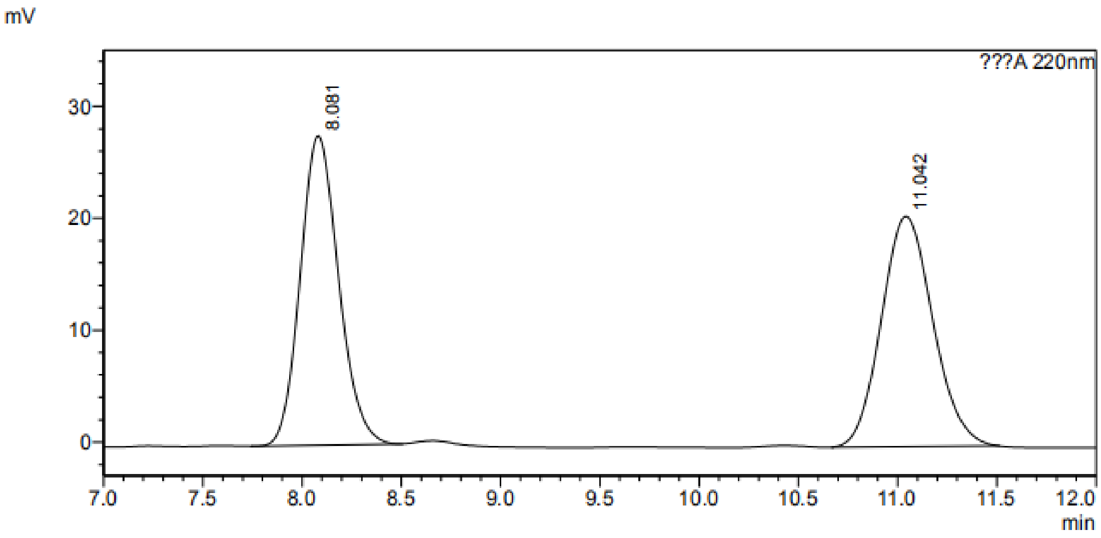

<Peak Table>

???A 220nm

Peak#	Ret. Time	Area	Height	Conc.	Unit	Mark	Name
1	8.081	372010	27641	49.978		M	
2	11.042	372332	20569	50.022		M	
Total		744342	48209				

<Chromatogram>

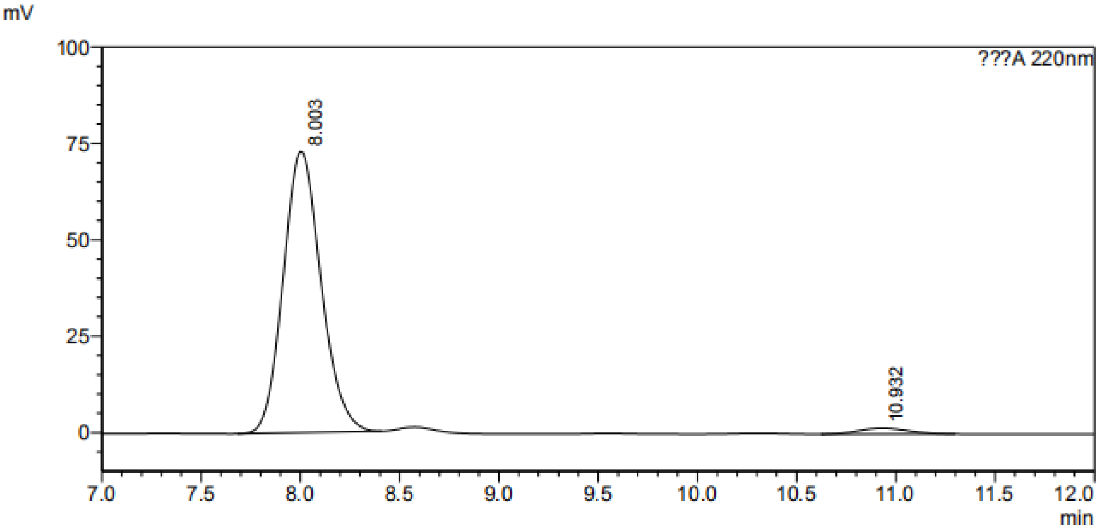

<Peak Table>

???A 220nm

Peak#	Ret. Time	Area	Height	Conc.	Unit	Mark	Name
1	8.003	971626	72974	97.295		M	
2	10.932	27009	1567	2.705		M	
Total		998635	74541				

(*R*)-3-Benzyl-1-phenylheptan-3-ol (**10a**)

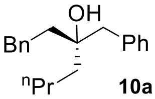

10a

<Chromatogram>

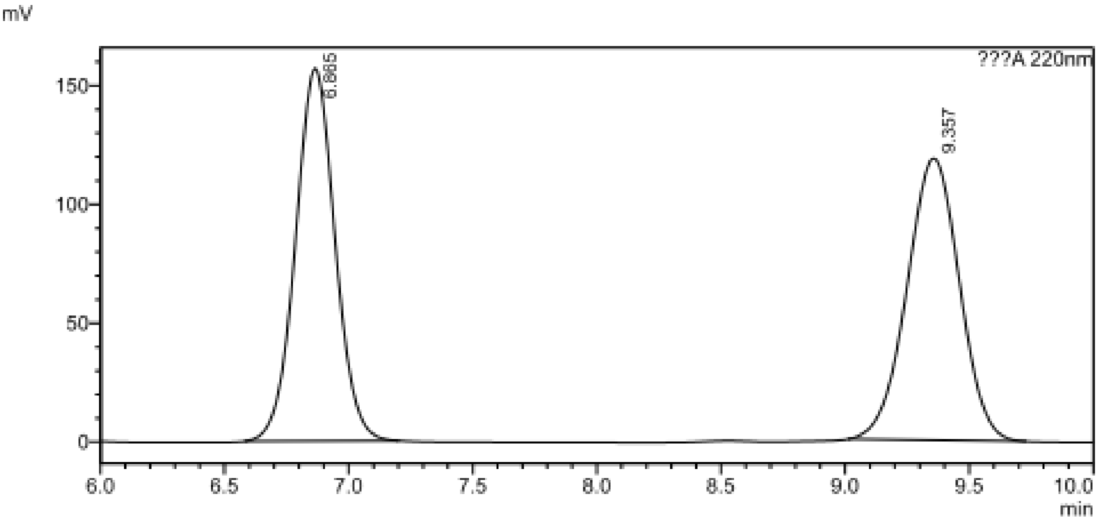

<Peak Table>

???A 220nm

Peak#	Ret. Time	Area	Height	Conc.	Unit	Mark	Name
1	6.865	1742847	156823	49.978			
2	9.357	1744387	118547	50.022			
Total		3487234	275370				

<Chromatogram>

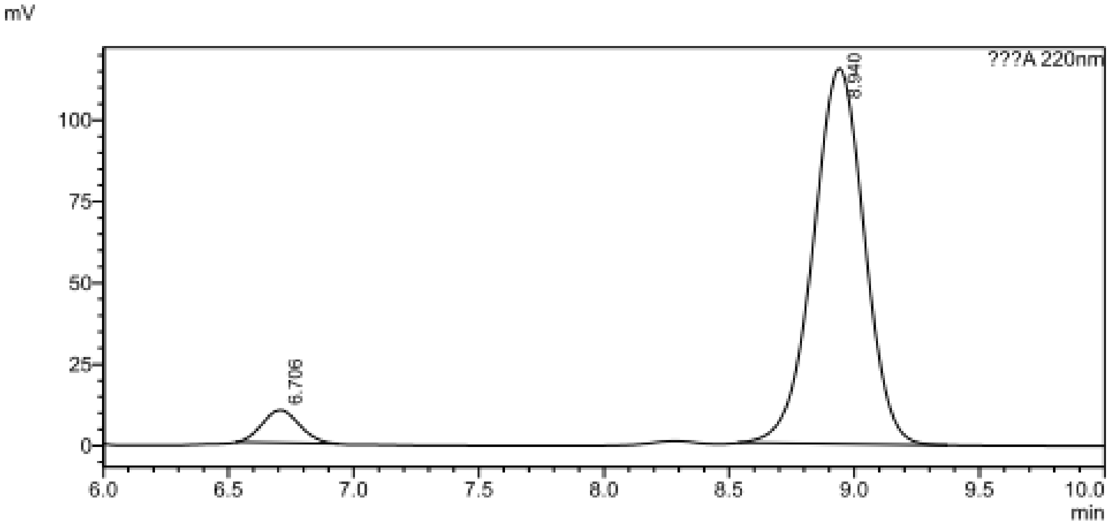

<Peak Table>

???A 220nm

Peak#	Ret. Time	Area	Height	Conc.	Unit	Mark	Name
1	6.706	104853	10074	6.002			
2	8.940	1642170	115309	93.998		M	
Total		1747023	125383				

(*S*)-2,3-Dimethyl-1-phenylbutan-2-ol (**10b**)

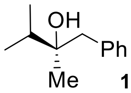

10b

<Chromatogram>

mV

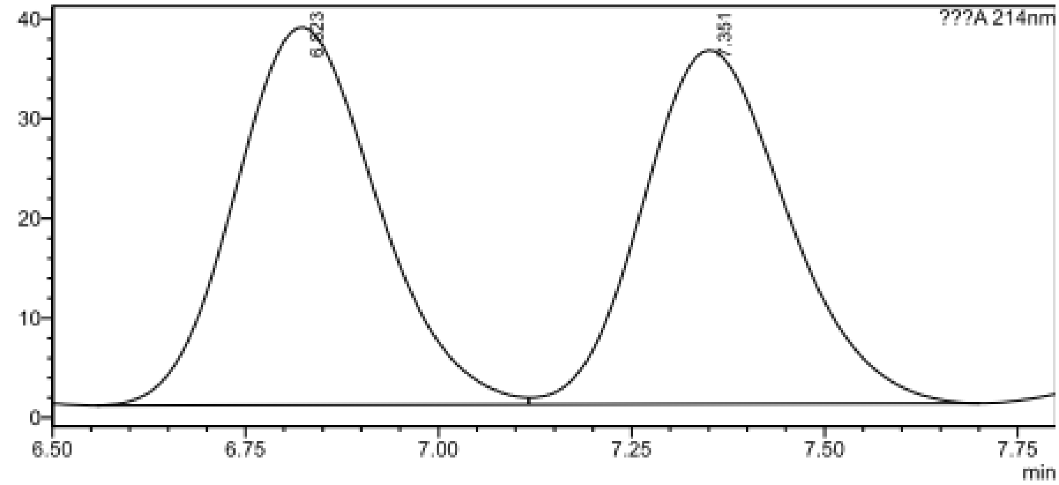

<Peak Table>

???A 214nm

Peak#	Ret. Time	Area	Height	Conc.	Unit	Mark	Name
1	6.823	484990	37883	51.156			
2	7.351	463072	35497	48.844		V	
Total		948062	73379				

<Chromatogram>

mV

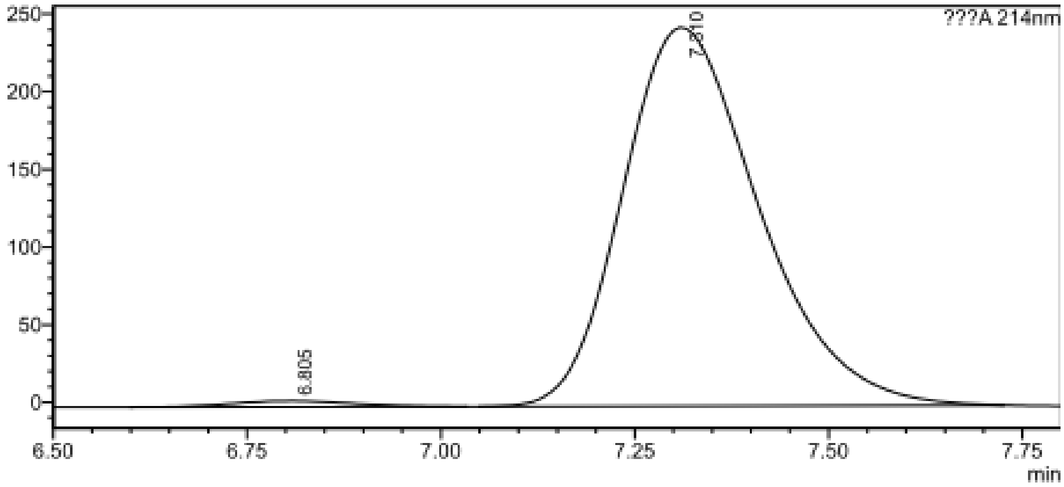

<Peak Table>

???A 214nm

Peak#	Ret. Time	Area	Height	Conc.	Unit	Mark	Name
1	6.805	40602	3809	1.344			
2	7.310	2980178	243761	98.656		M	
Total		3020780	247570				

(*R*)-(2-(Methoxymethyl)-2-methylpentyl)benzene (**10c**)

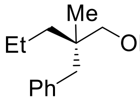

10c

<Chromatogram>

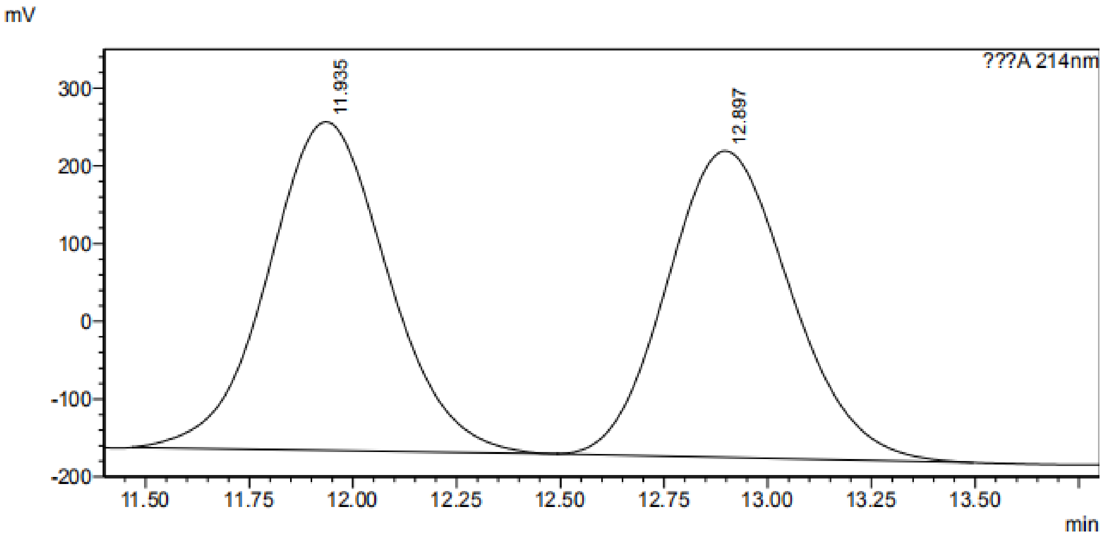

<Peak Table>

???A 214nm

Peak#	Ret. Time	Area	Height	Conc.	Unit	Mark	Name
1	11.935	8494067	422706	50.906		M	
2	12.897	8191580	394260	49.094		M	
Total		16685647	816966				

<Chromatogram>

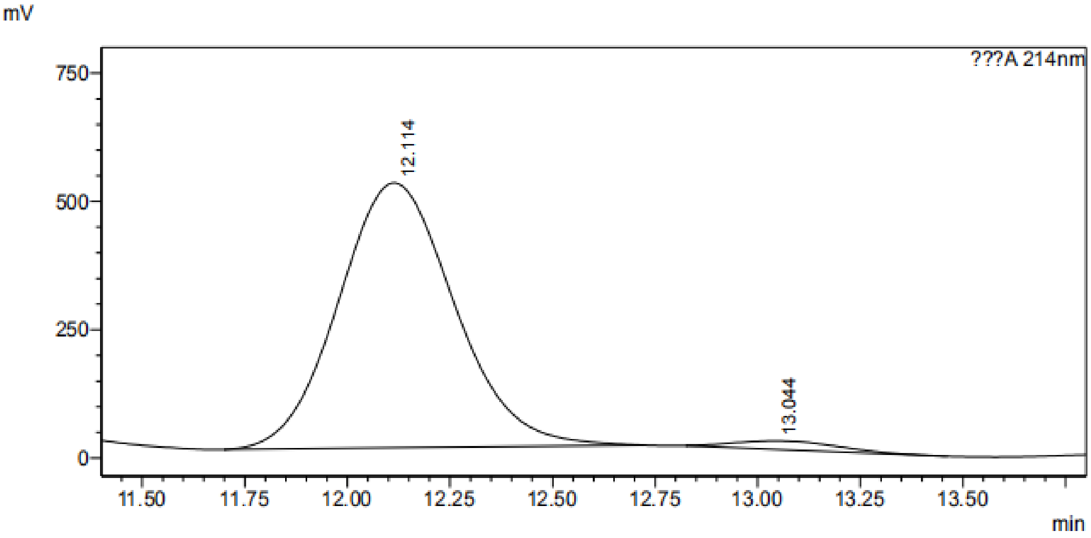

<Peak Table>

???A 214nm

Peak#	Ret. Time	Area	Height	Conc.	Unit	Mark	Name
1	12.114	10158033	515703	97.284		M	
2	13.044	283616	16264	2.716		M	
Total		10441649	531966				

(*S*)-2,3-Dimethyl-2-phenylbutan-1-ol (**10d**)

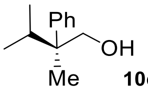

10d

<Chromatogram>

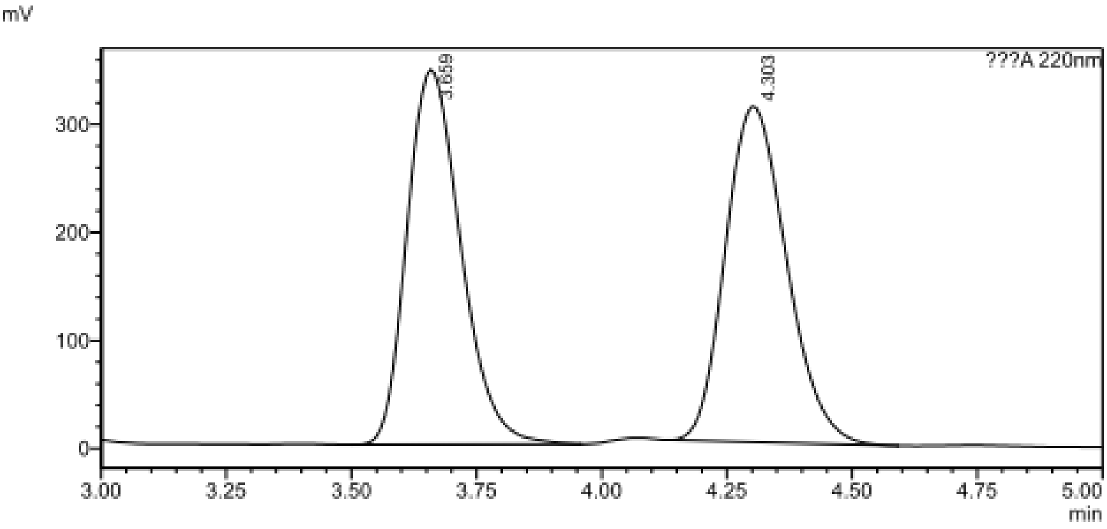

<Peak Table>

???A 220nm

Peak#	Ret. Time	Area	Height	Conc.	Unit	Mark	Name
1	3.659	2602035	347404	49.525			
2	4.303	2651970	311152	50.475			
Total		5254004	658556				

<Chromatogram>

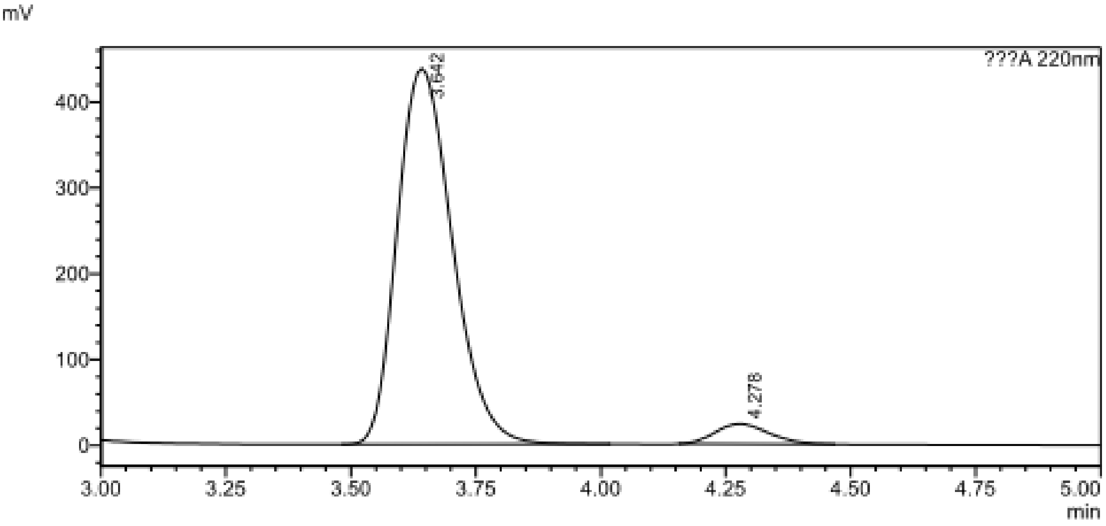

<Peak Table>

???A 220nm

Peak#	Ret. Time	Area	Height	Conc.	Unit	Mark	Name
1	3.642	3366033	437579	95.029		M	
2	4.278	176094	23430	4.971		M	
Total		3542127	461009				

(*S*)-2-Methyl-2,3-diphenylpropyl propionate (**10e**)

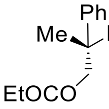

10e

<Chromatogram>

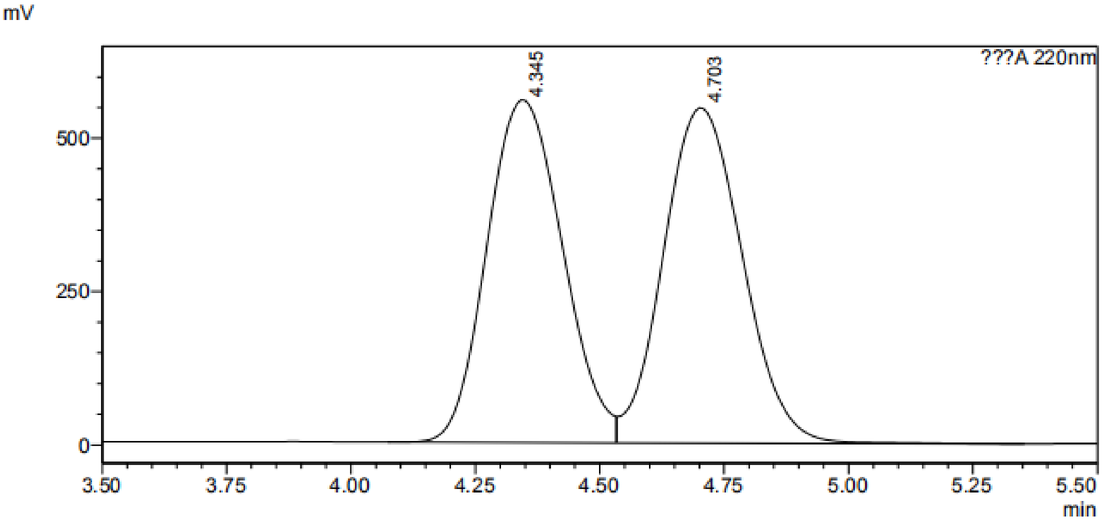

<Peak Table>

???A 220nm

Peak#	Ret. Time	Area	Height	Conc.	Unit	Mark	Name
1	4.345	5921875	558517	49.537			
2	4.703	6032631	546299	50.463		V	
Total		11954506	1104816				

<Chromatogram>

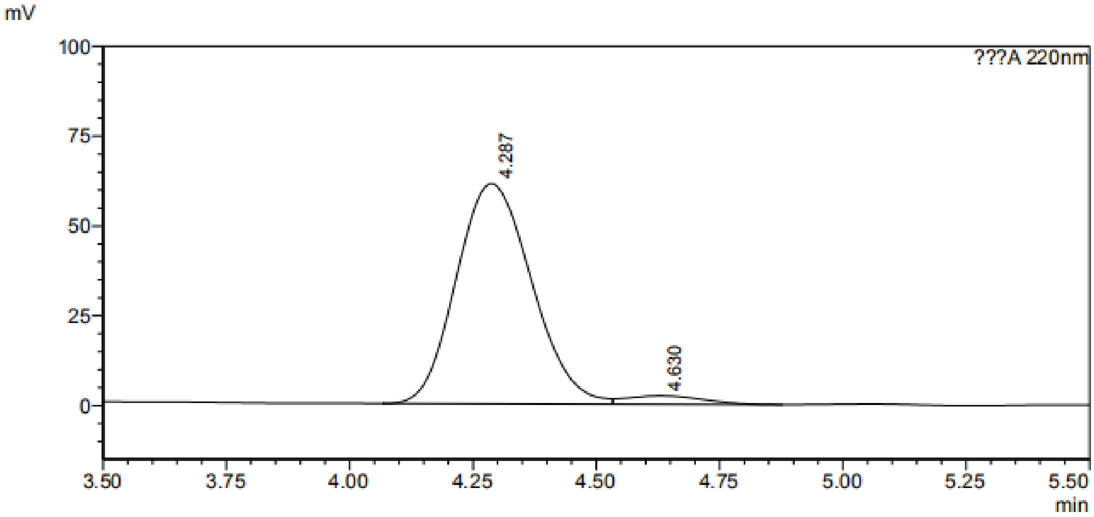

<Peak Table>

???A 220nm

Peak#	Ret. Time	Area	Height	Conc.	Unit	Mark	Name
1	4.287	653046	61274	96.319			
2	4.630	24959	2350	3.681		V	
Total		678005	63623				

(*S*)-2-(2-Benzyl-2-vinylpent-4-en-1-yl)naphthalene (**10f**)

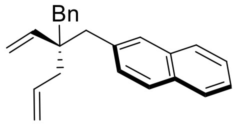

10f

<Chromatogram>

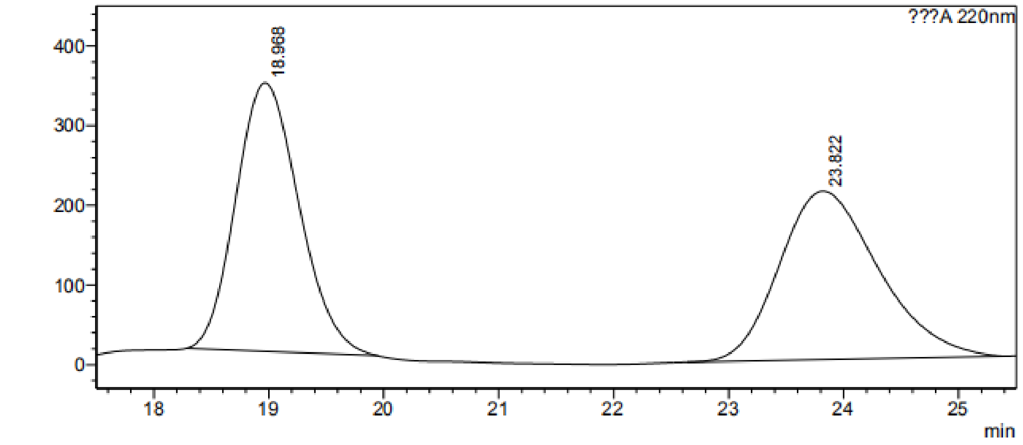

<Peak Table>

???A 220nm

Peak#	Ret. Time	Area	Height	Conc.	Unit	Mark	Name
1	18.968	12923375	337197	50.434		M	
2	23.822	12701058	211498	49.566			
Total		25624433	548695				

<Chromatogram>

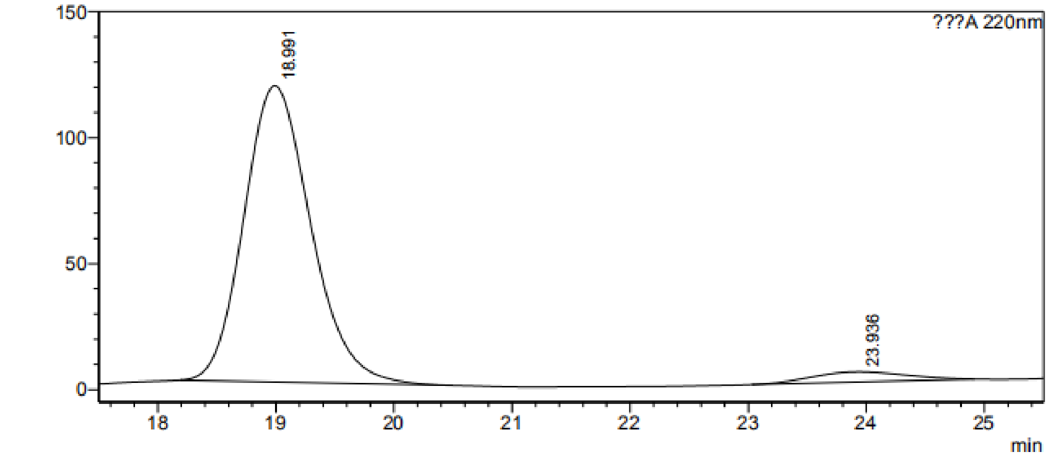

<Peak Table>

???A 220nm

Peak#	Ret. Time	Area	Height	Conc.	Unit	Mark	Name
1	18.991	4637832	117579	95.414			
2	23.936	222888	4017	4.586		M	
Total		4860720	121597				

(*S*)-2-Benzyl-2-(4-fluorobenzyl)butyl propionate (**10g**)

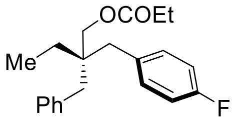

10g

<Chromatogram>

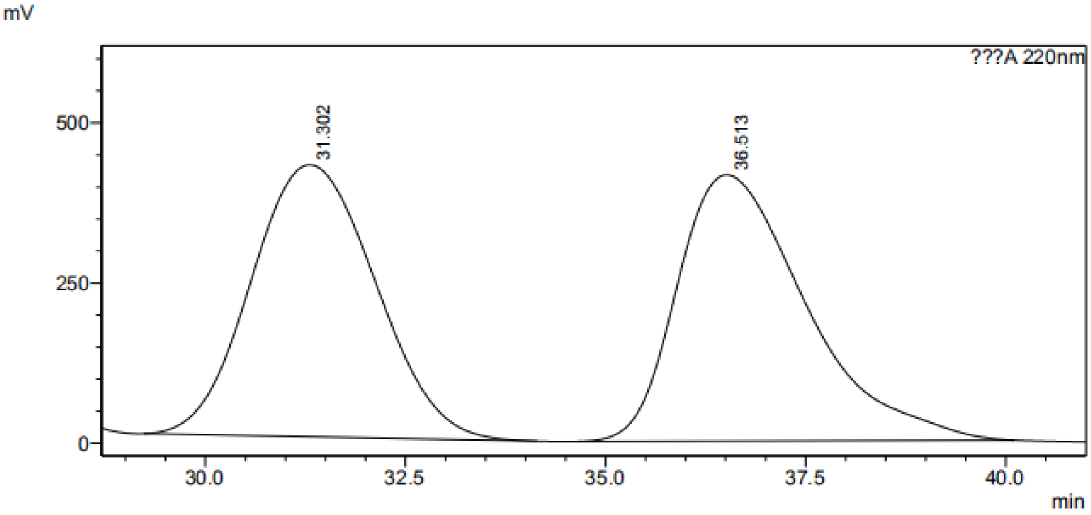

<Peak Table>

???A 220nm

Peak#	Ret. Time	Area	Height	Conc.	Unit	Mark	Name
1	31.302	45860431	424664	49.685		M	
2	36.513	46441800	415318	50.315		M	
Total		92302231	839982				

<Chromatogram>

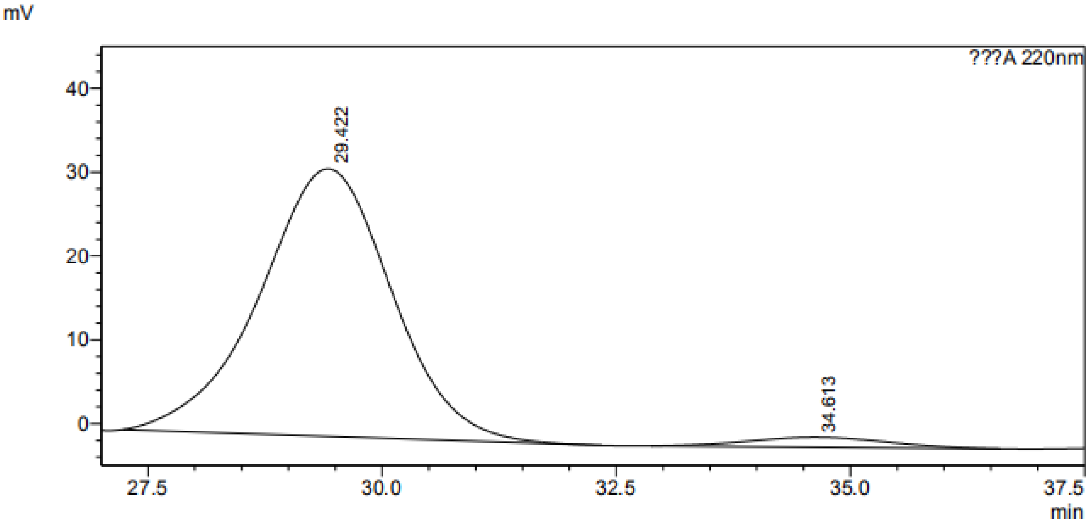

<Peak Table>

???A 220nm

Peak#	Ret. Time	Area	Height	Conc.	Unit	Mark	Name
1	29.422	3133526	31933	96.301		M	
2	34.613	120368	1175	3.699		M	
Total		3253894	33108				

(*S*)-Methyl 2-benzyl-2-(naphthalen-2-ylmethyl)pent-4-enoate (**10h**)

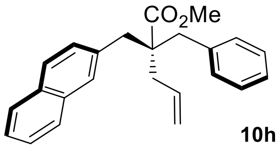

10h

<Chromatogram>

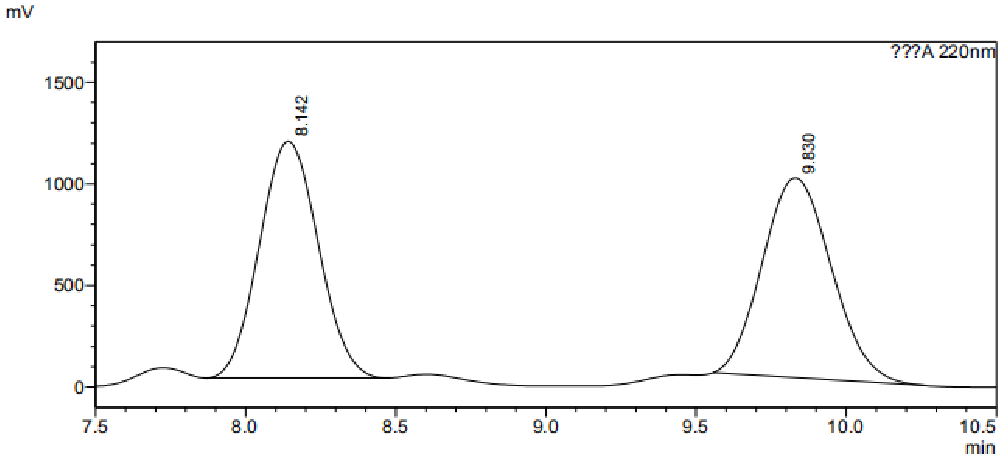

<Peak Table>

???A 220nm

Peak#	Ret. Time	Area	Height	Conc.	Unit	Mark	Name
1	8.142	15883638	1166178	50.214		M	
2	9.830	15748473	982425	49.786		M	
Total		31632112	2148603				

<Chromatogram>

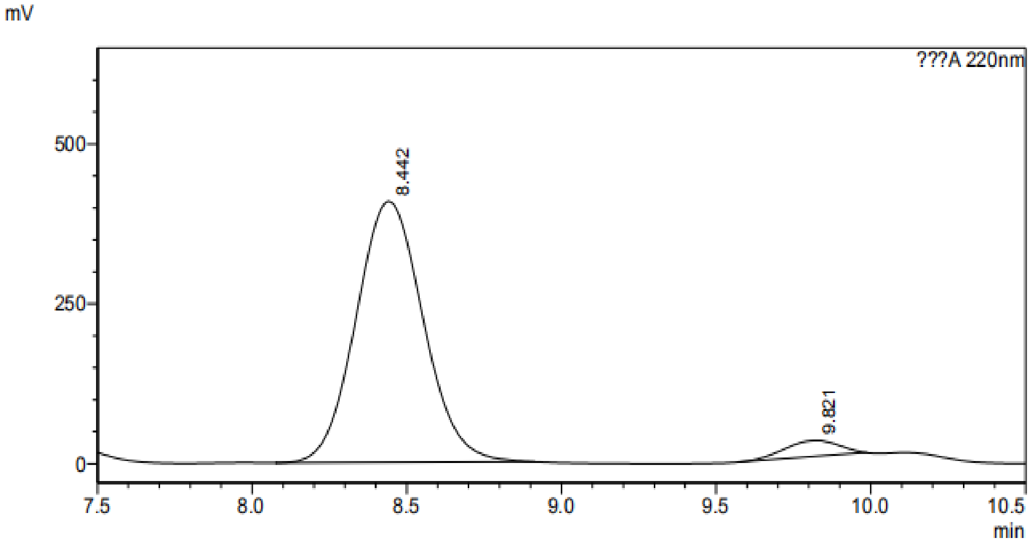

<Peak Table>

???A 220nm

Peak#	Ret. Time	Area	Height	Conc.	Unit	Mark	Name
1	8.442	6177741	408156	95.215		M	
2	9.821	310438	24874	4.785		M	
Total		6488179	433030				

(1*S*,2*R*,3*S*)-3-Azido-3-pentyl-1-phenylnonane-1,2-diol (**11a**)

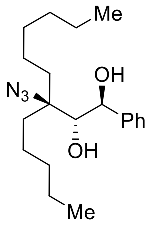

11a

<Chromatogram>

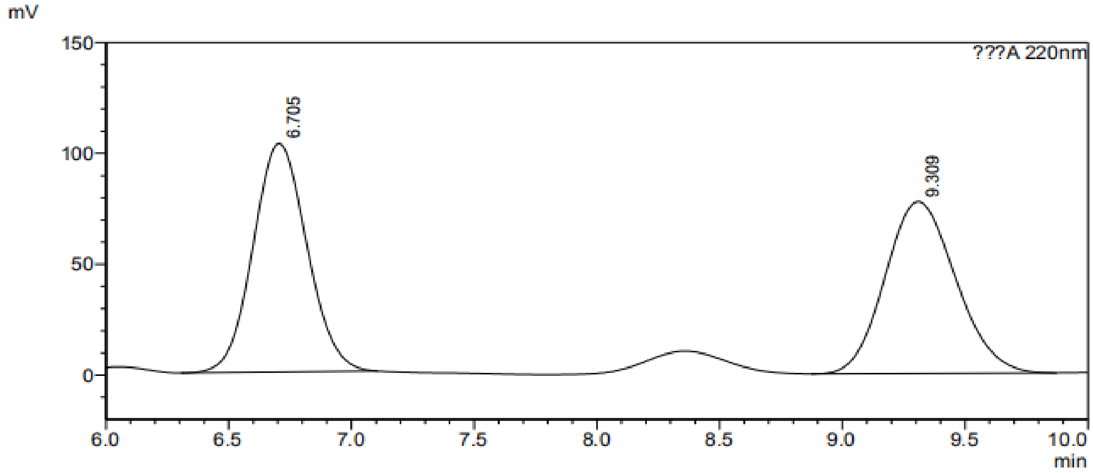

<Peak Table>

???A 220nm

Peak#	Ret. Time	Area	Height	Conc.	Unit	Mark	Name
1	6.705	1575349	103017	50.360		M	
2	9.309	1552821	77473	49.640			
Total		3128169	180490				

<Chromatogram>

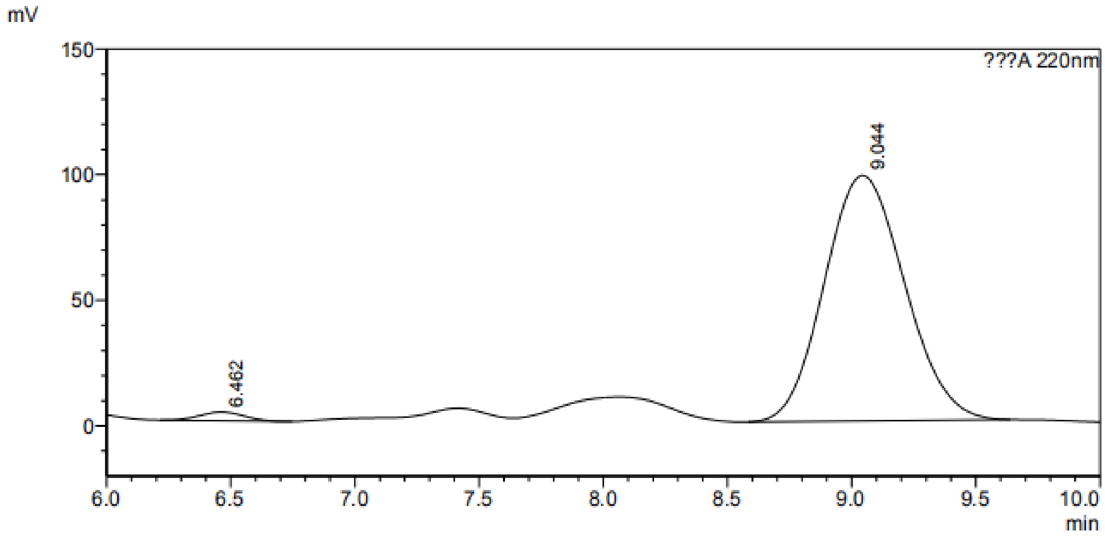

<Peak Table>

???A 220nm

Peak#	Ret. Time	Area	Height	Conc.	Unit	Mark	Name
1	6.462	42980	3482	1.915		M	
2	9.044	2201508	97704	98.085		M	
Total		2244488	101187				

(*R*)-Ethyl 2-((*R*)-amino(naphthalen-2-yl)methyl)-2-methylpentanoate (**11b**)

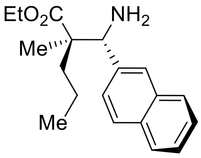

11b

<Chromatogram>

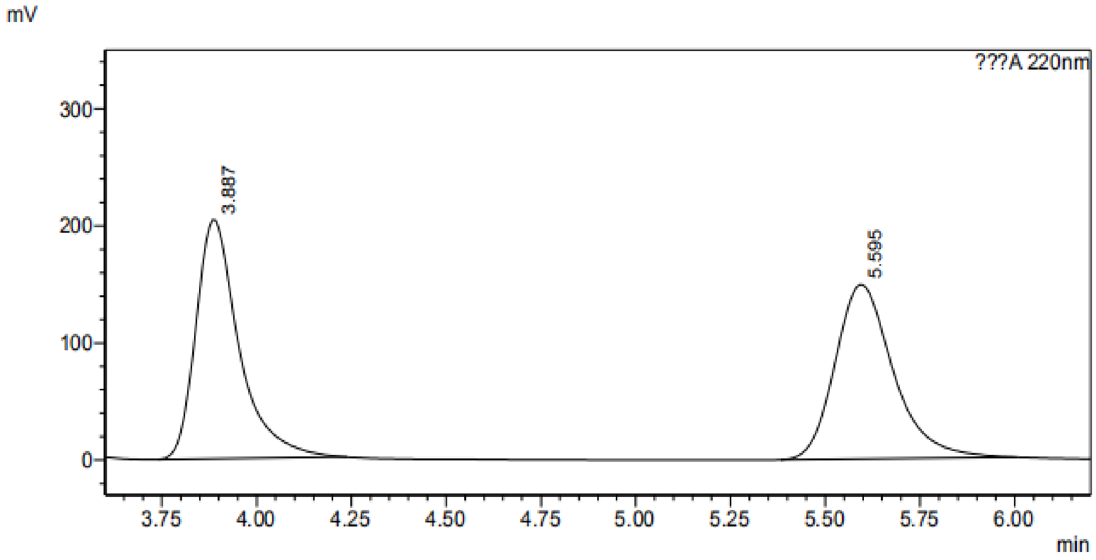

<Peak Table>

???A 220nm

Peak#	Ret. Time	Area	Height	Conc.	Unit	Mark	Name
1	3.887	1615565	204079	50.378		M	
2	5.595	1591348	148935	49.622		M	
Total		3206913	353013				

<Chromatogram>

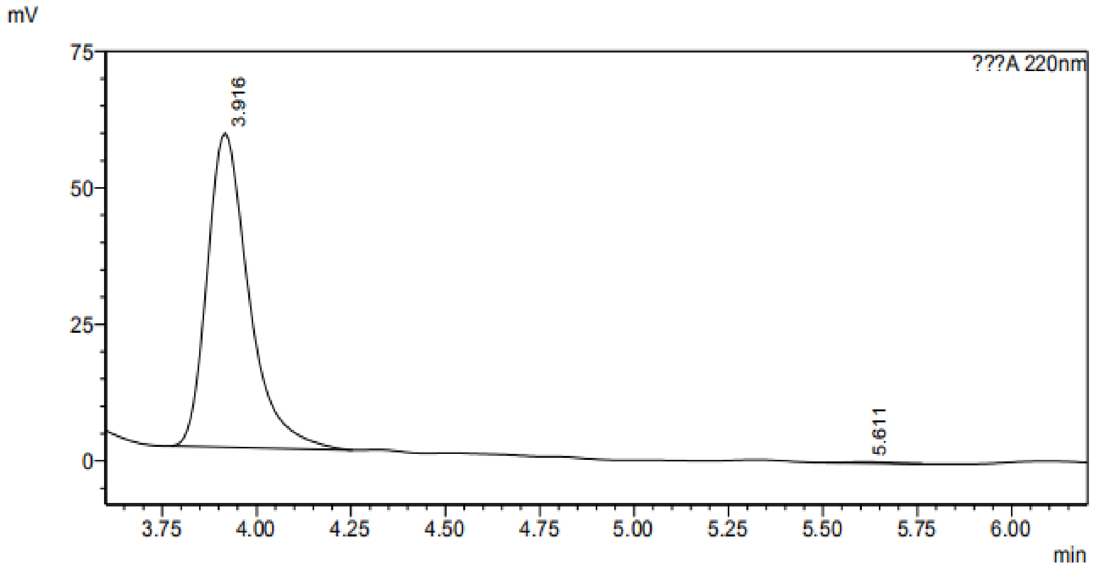

<Peak Table>

???A 220nm

Peak#	Ret. Time	Area	Height	Conc.	Unit	Mark	Name
1	3.916	441326	57497	99.618		M	
2	5.611	1693	226	0.382		M	
Total		443019	57722				

(1*R*,3*S*)-1,3-Diphenyl-1-(*o*-tolyl)-1,3-dihydroisobenzofuran (**11c**)

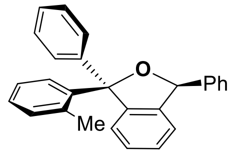

11c

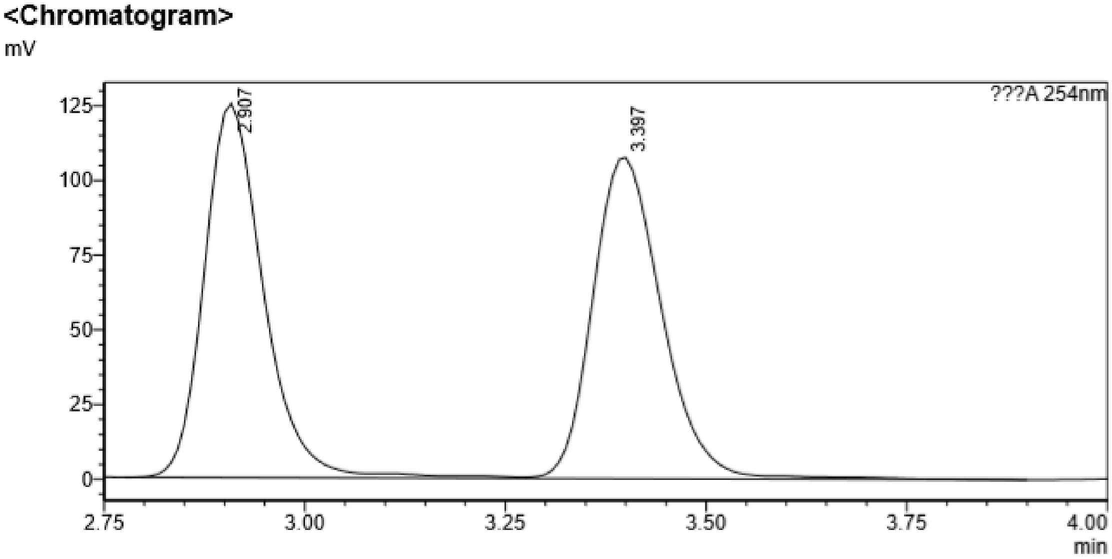

<Peak Table>

???A 254nm

Peak#	Ret. Time	Area	Height	Conc.	Unit	Mark	Name
1	2.907	645100	125113	50.084		S	
2	3.397	642949	107380	49.916		V	
Total		1288050	232493				

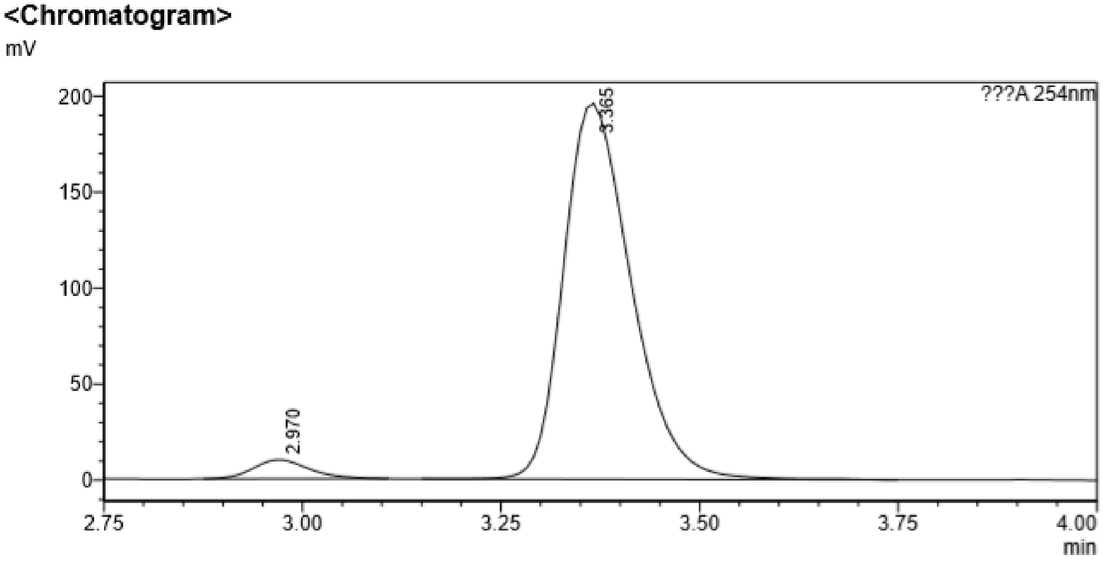

<Peak Table>

???A 254nm

Peak#	Ret. Time	Area	Height	Conc.	Unit	Mark	Name
1	2.970	49095	9906	4.053		M	
2	3.365	1162075	195757	95.947		S	
Total		1211169	205663				

Methyl(1*S*,2*R*)-2-allyl-1-(4-chlorophenyl)-2,3-dihydro-1*H*-cyclopenta[*a*]naphthalene-2-carboxylate (**11d**)

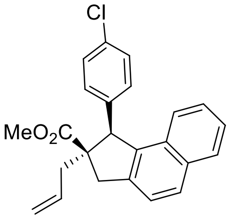

11d

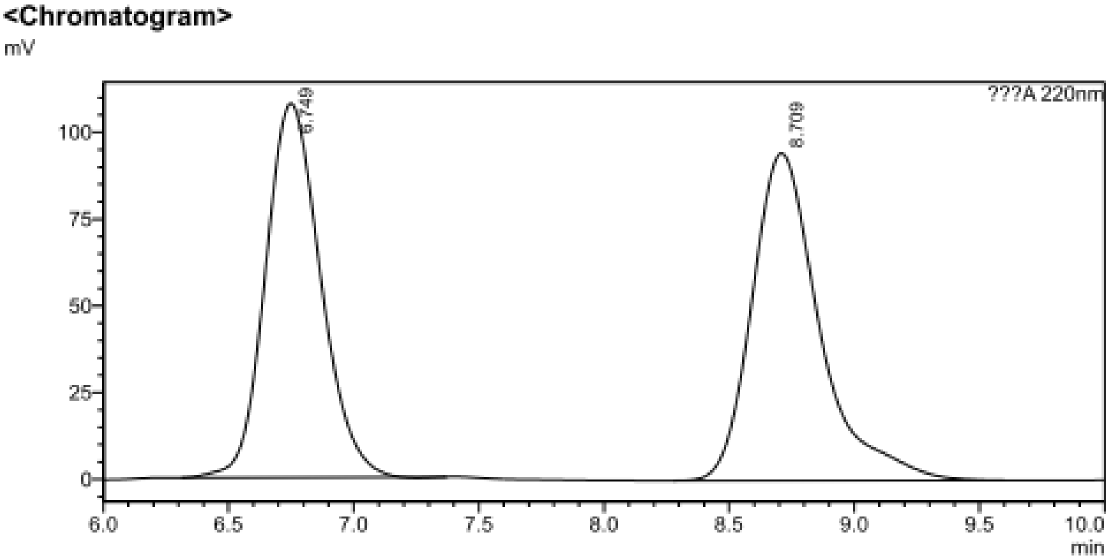

<Peak Table>

???A 220nm

Peak#	Ret. Time	Area	Height	Conc.	Unit	Mark	Name
1	6.749	1652952	107771	48.254		M	
2	8.709	1772545	94329	51.746		M	
Total		3425497	202100				

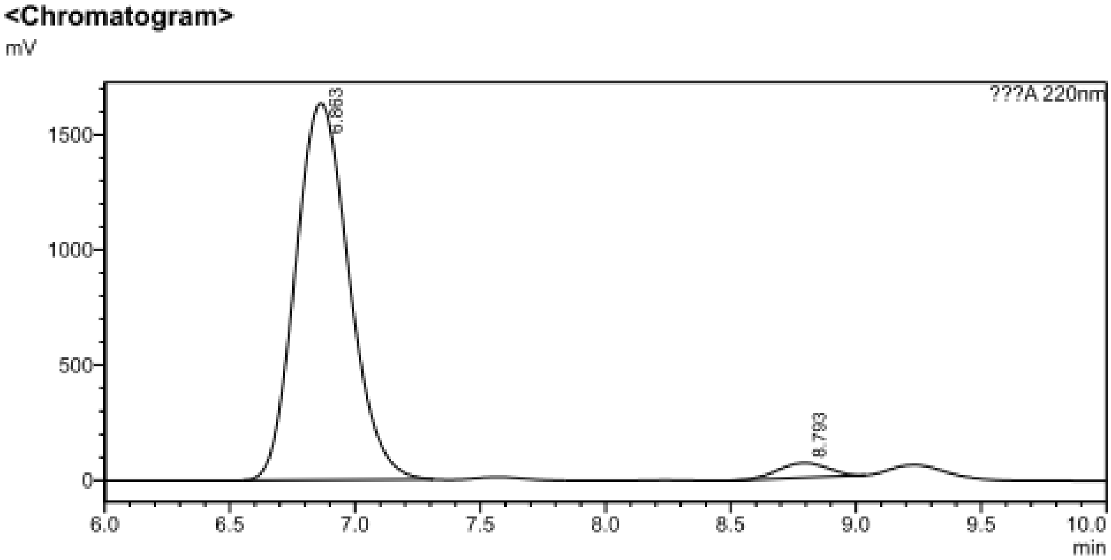

<Peak Table>

???A 220nm

Peak#	Ret. Time	Area	Height	Conc.	Unit	Mark	Name
1	6.863	24308046	1633707	96.490		M	
2	8.793	884186	63447	3.510		M	
Total		25192233	1697154				

(2*S*,3*R*)-2-(4-Methoxyphenyl)-3-methyl-2-(*p*-tolyl)oxirane (**11e**)

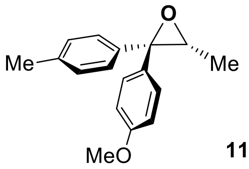

11e

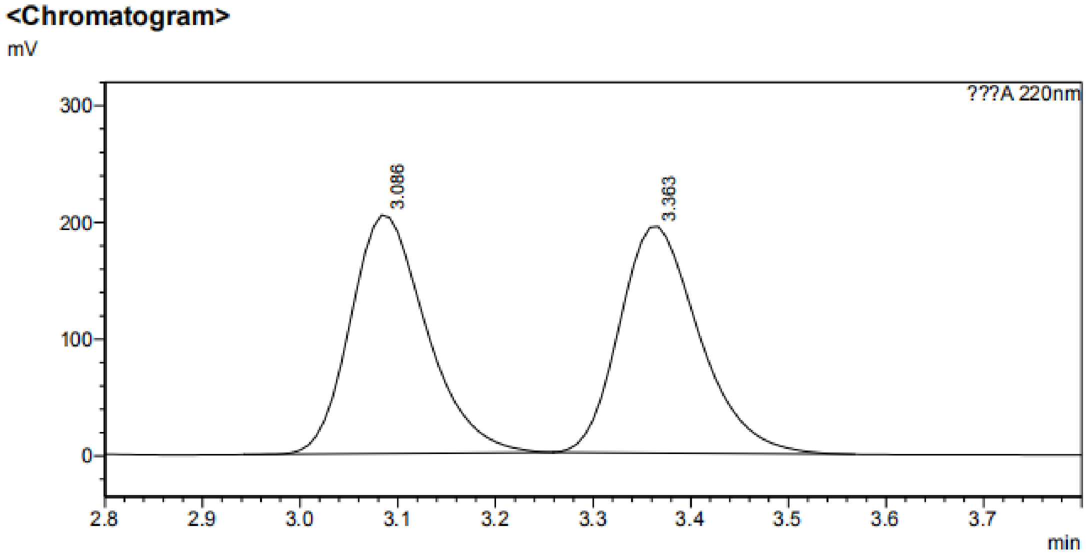

<Peak Table>

???A 220nm

Peak#	Ret. Time	Area	Height	Conc.	Unit	Mark	Name
1	3.086	1107971	204383	50.048			
2	3.363	1105844	193962	49.952		M	
Total		2213815	398344				

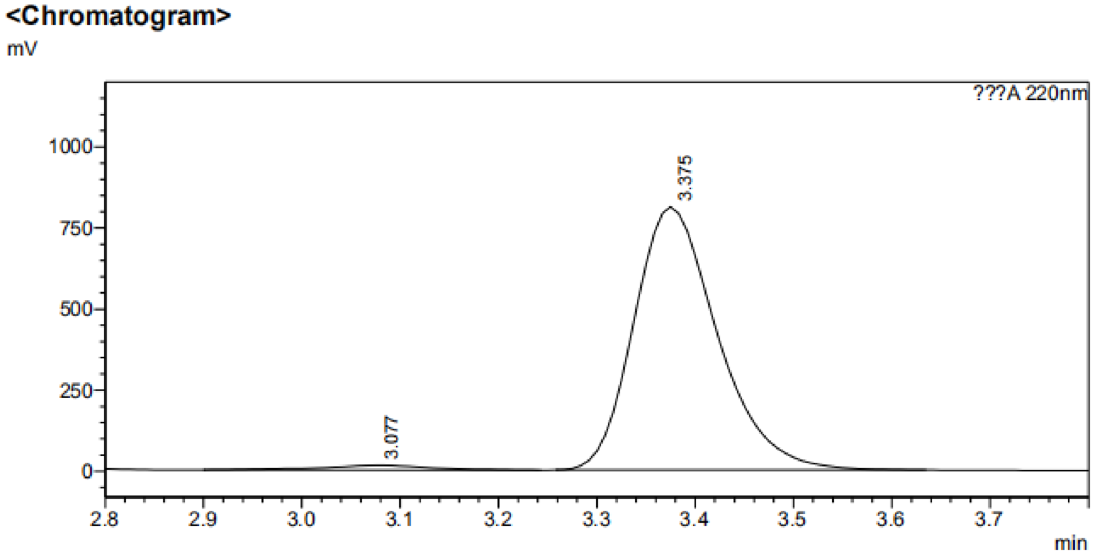

<Peak Table>

???A 220nm

Peak#	Ret. Time	Area	Height	Conc.	Unit	Mark	Name
1	3.077	108972	14370	2.223		M	
2	3.375	4792430	811322	97.777		M	
Total		4901402	825692				

(*S*)-2-Methyl-1,2-diphenylpent-4-en-1-one (**11f**)

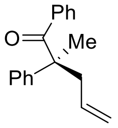

11f

<Chromatogram>

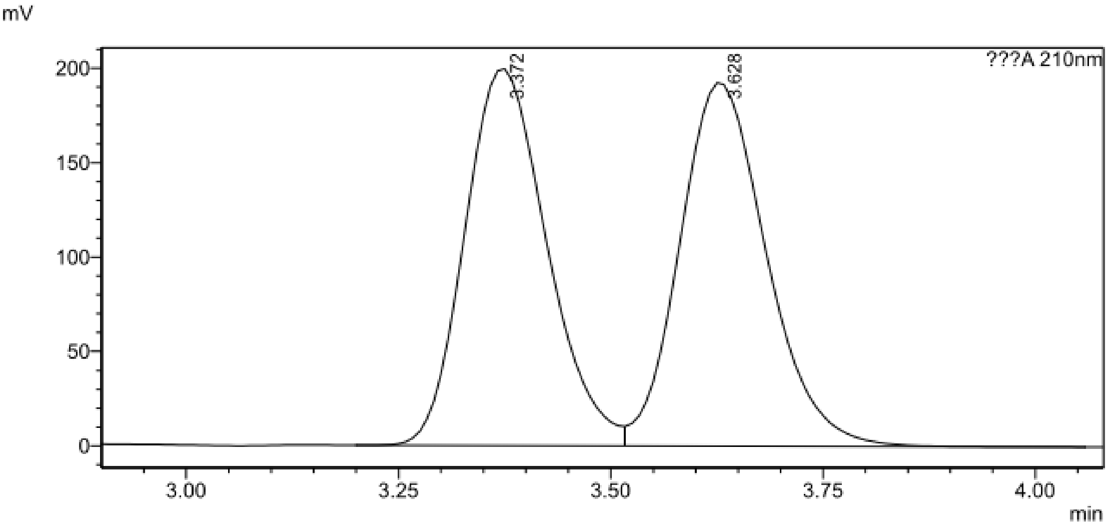

<Peak Table>

???A 210nm

Peak#	Ret. Time	Area	Height	Conc.	Unit	Mark	Name
1	3.372	1344293	199556	49.624			
2	3.628	1364684	192321	50.376		V	
Total		2708978	391877				

<Chromatogram>

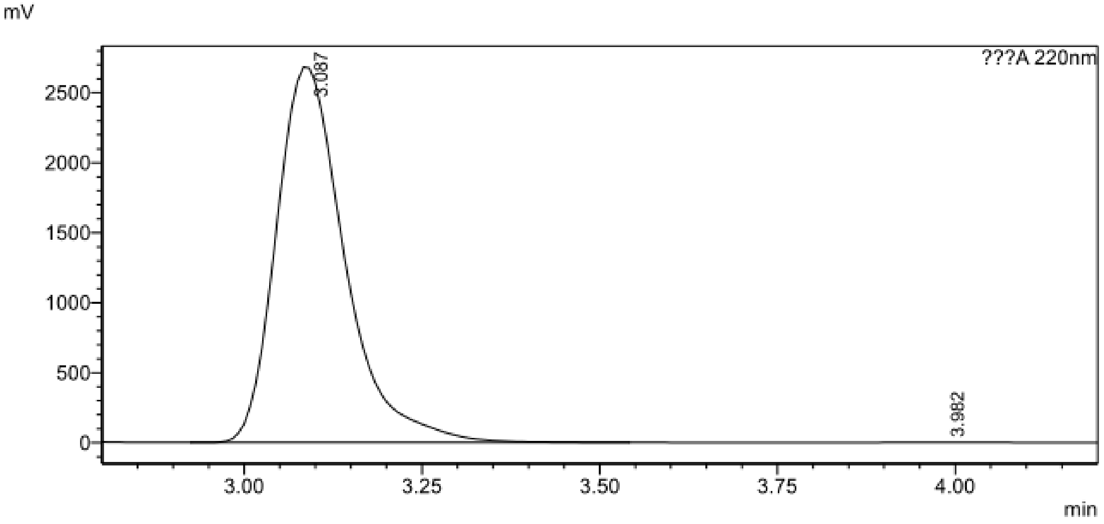

<Peak Table>

???A 220nm

Peak#	Ret. Time	Area	Height	Conc.	Unit	Mark	Name
1	3.087	18011955	2682134	99.942		M	
2	3.982	10463	1152	0.058		M	
Total		18022418	2683285				

IX. Limitation of State-of-Art Asymmetric Synthesis of the Enantioenriched Products Obtained in This Study

A General Summary:

In this work, all racemic diols were made using the following method (**Supplementary Figure 359**). Therefore, if α-hydroxy ketones **I** can be obtained in high enantiopurity and practical yield, **1a-1ad** all can be obtained as the enantiopure form.

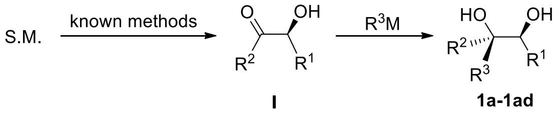

Supplementary Figure 359. A general method to make racemic diols

However, a thorough survey shows that only four α-hydroxy ketones used in this work can be obtained in >60% yield with >95% ee, and all others cannot.

Similarly, if we can get chiral disubstituted ketoesters **II** in high enantiopurity, then all **5a-5o** can be obtained using diastereoselective reduction (**Supplementary Figure 360**).

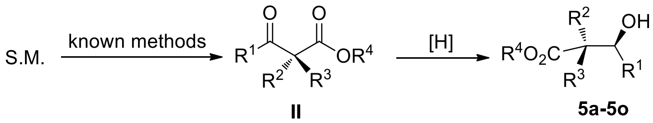

Supplementary Figure 360. A general method to make racemic esters

However, we found that all seven types of disubstituted ketoesters used in this work cannot be obtained with high ee, and for most of them, there are only racemic synthetic reports so far.

Moreover, principally all the epoxy alcohols can be gotten using the following two methods (**Supplementary Figure 361**).

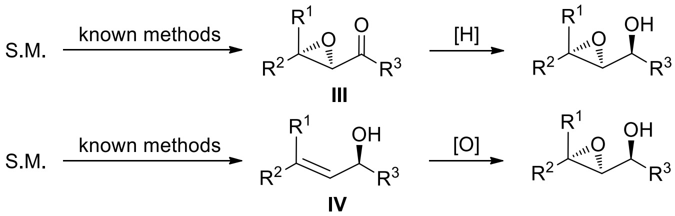

Supplementary Figure 361. A general method to make racemic epoxy alcohols

However, there is no method that can afford **III** in high ee when R^1 and R^2 are both aryl groups or both alkyl groups. Although **IV** can be obtained in high enantiopurity, the following epoxidation afforded *cis*-product in low yield in our tests.

Therefore, among the 96 enantioenriched products we obtained in this work, only very small amount of them can be obtained starting from chiral chemicals. Actually we can easily synthesized and afford a large amount of new products that cannot be produced using known methods. A detailed analysis is shown below:

Asymmetric Synthesis of Diols with Tertiary Alcohol Units

Generally, all diols **1a-1ad** shown in Figure 2 of our manuscript can be synthesized from the following retro-synthetic route (**Supplementary Figure 362**). Therefore, as long as we can get **I** in high enantiopurity and practical yield, the access to **1a-1ad** is guaranteed. This way is traditionally called chiral source synthesis.

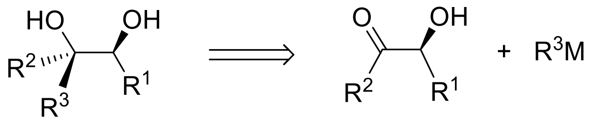

Supplementary Figure 362. A general method to make enantiopurity **1a-1ad** via chiral source

The following **Supplementary Figure 363** lists 12 types of α-hydroxy ketones that were used in our work. Of course, we used racemic ones in our study, and almost all of them can be made through the same route. However, to get them in high enantiopurity and yields, it turns to be a tedious and challenging task, because we found that most of them cannot be obtained in satisfactory results. Asymmetric synthesis from chiral chemicals usually needs that a chiral chemical is with 99% ee and can be commercially available or easily obtained. In this respect, we found that most of the following α-hydroxy ketones (**I**) cannot meet the requirements. Therefore, a lower standard of getting **I** in >60% yield with >95% ee is thought to be useful for the next step to get tertiary alcohols **1a-1ad**. However, it is still beyond the conventional cognition that the state-of-art asymmetric synthesis of all the following 12 types of α-hydroxy ketones are far from being satisfactory.

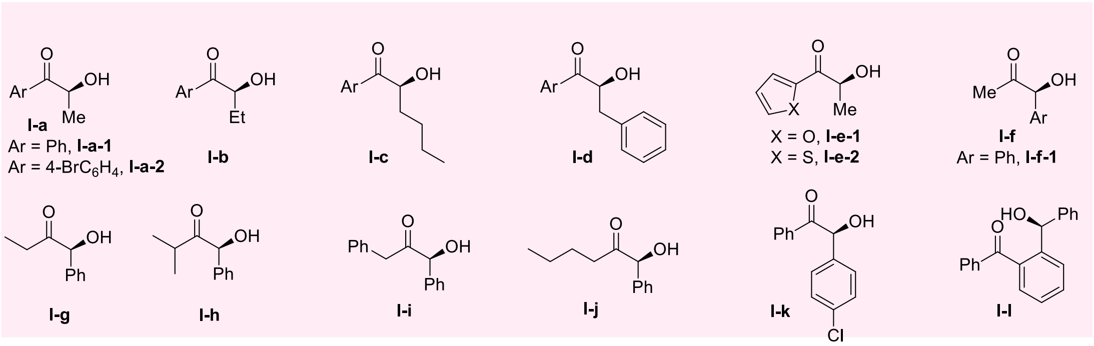

Supplementary Figure 363. Types of enantiopurity α-hydroxy ketones in our work.

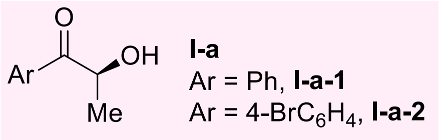

The asymmetric synthesis of type **I-a** especially **I-a-1** has been studied intensively using both enzymatic and chemical methods. Usually three methods are used for enzyme route: reduction of 1,2-diketones, benzoin reaction, and α-hydroxylation of ketones.

Enzyme-catalyzed asymmetric reduction of 1,2-diketones:

There are many reports using enzyme-catalyzed asymmetric reduction of 1,2-diketones. In most cases, only **I-a-1** was obtained in good to high yield with excellent ee[1-9]. Chadha et. al reported the highly enantioselective synthesis of **I-a-1**, but when the method was also used to get **I-b**, only 36% ee was observed (Scheme 3a)[5]. Hanefeld's protocol can afford the mixture of both **I-a-1** and **I-f-1** with ee values lower than 90% (**Supplementary Figure 364**)[6]. Ohno's work could produce both **I-a-1** and **I-b** with excellent 99% ee, but for **I-b**, the yield dropped sharply to 26% (**Supplementary Figure 364-c**)[9].

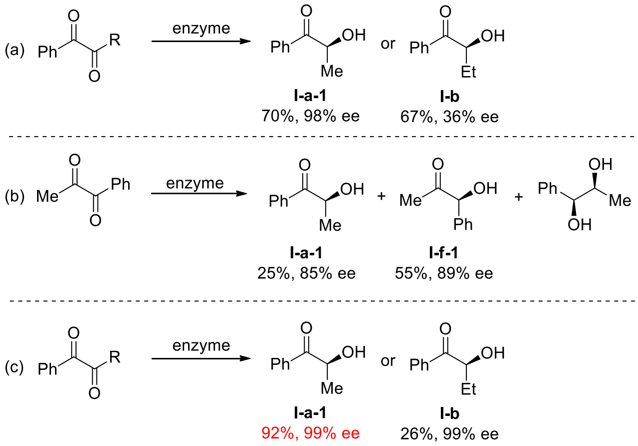

Supplementary Figure 364. Enzyme-catalyzed asymmetric reduction of 1,2-diketones Enzyme-catalyzed asymmetric benzoin reaction

There are also many reports using enzyme-catalyzed benzoin reaction to produce α-hydroxy ketones, but again in most cases they were compatible with only **I-a-1**[10-21]. Pohl and co-workers reported recently an enzyme-catalyzed benzoin reaction, affording **I-a-1** in 99% yield with 92% ee, but low yields and ee values were found in the synthesis of **I-a-2**, **I-e-1**, and **I-e-2** (**Supplementary Figure 365**-a)[16]. Müller et al. described the asymmetric synthesis of **I-a-1** in high selectivities, but when the method was applied to EtCHO, the mixture of both **I-b** and **I-g** was obtained (**Supplementary Figure 365**-b)[17].

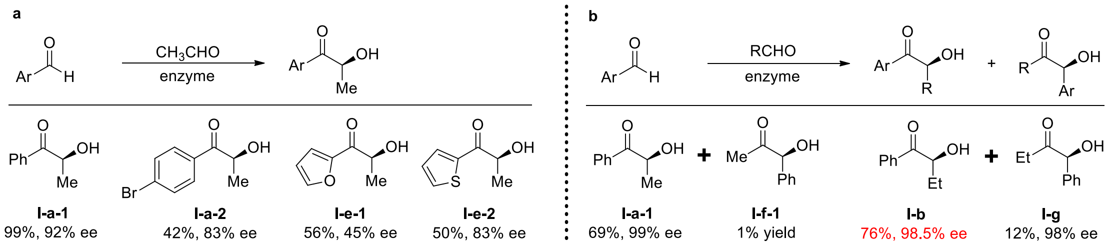

Supplementary Figure 365. Enzyme-catalyzed asymmetric benzoin reaction.

Enzyme-catalyzed asymmetric α-hydroxylation of ketones:

Ref 22 reported an enzyme method to afford **I-a-2** and **I-a-3** in good results through asymmetric α-hydroxylation of ketones. However, using similar method to make **I-f** gave less satisfactory results (**Supplementary Figure 366**).

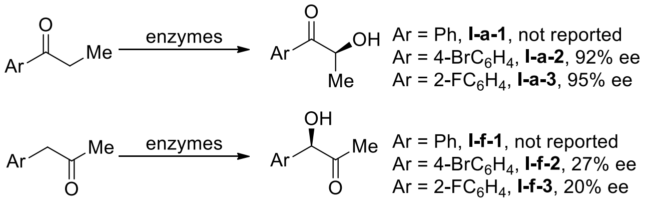

Supplementary Figure 366. Enzyme-catalyzed asymmetric α-hydroxylation of ketones

----

Chemical method I: oxidation of enol derivatives

The oxidation (epoxidation, dihydroxylation) of enol derivatives is the mostly used non-enzymatic method to get enantioenriched α-hydroxy ketones. **Supplementary Figure 367** lists one of the general way using catalytic asymmetric epoxidation of enol ethers[23]. After hydrolysis, the final products are α-hydroxy ketones. However, all results were not satisfactory.

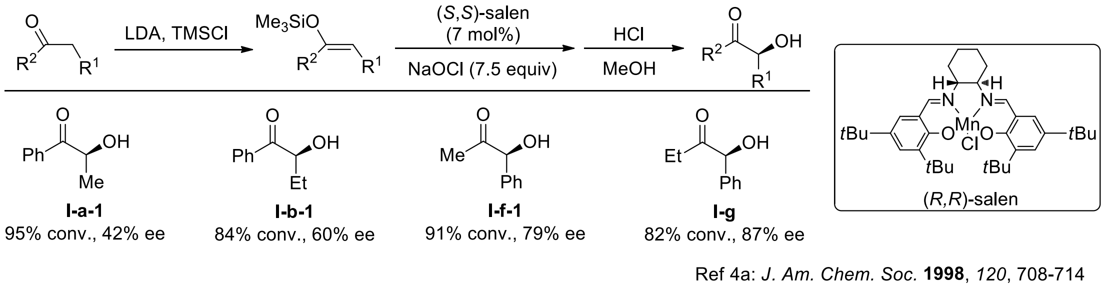

Supplementary Figure 367. Oxidation of enol derivatives

Later, an updated method using enol derived phosphate gave better results[24]. As shown in **Supplementary Figure 368**, **I-a-1** can be obtained with 96% ee but in 52% total yield, and when **I-b-1** was gotten, the ee has dropped to 93% and when R^1 was a Pr group, the ee was 83%, indicating that this method cannot be extended to R^1 groups “bigger” than Et. In 2012, the same group reported an alternative method using 1.5 equiv of AD-mix-α or AD-mix-β, and similar results were observed[25].

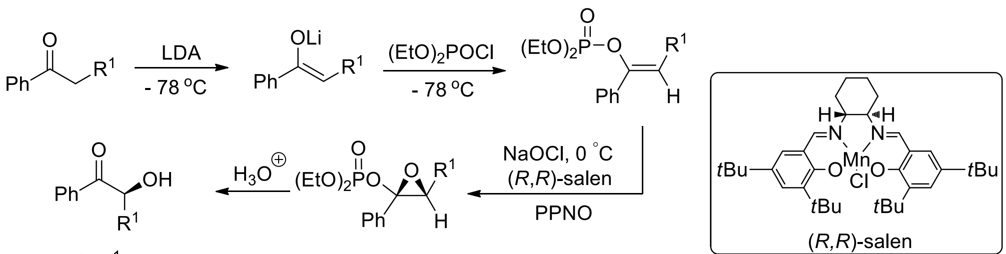

I-a-1, R[1] = Me, 52% total yield, 96% ee
I-b-1, R[1] = Et, 45% total yield, 93% ee
R[1] = Pr, 50% total yield, 83% ee

Supplementary Figure 368. Oxidation of enol derivatives

Stoichiometric Davis reagent (camphorylsulfonyl oxaziridine) has also been used to oxidize ketone enolate to afford **I-a-1** and **I-f-1**, but with only 62% and 40% ee, respectively[26-28].

Chemical method II: asymmetric reduction of 1,2-diketones

Ru-catalzyed transfer hydrogenation can afford **I-a-1** in high yield with excellent ee, but together with 11% of **I-f-1** with low ee (**Supplementary Figure 369**)[29]. When the method was extended to the synthesis of **I-b**, the yield has been very low (34%), together with **I-g** in 26% yield with 60% ee.

Scheme 8

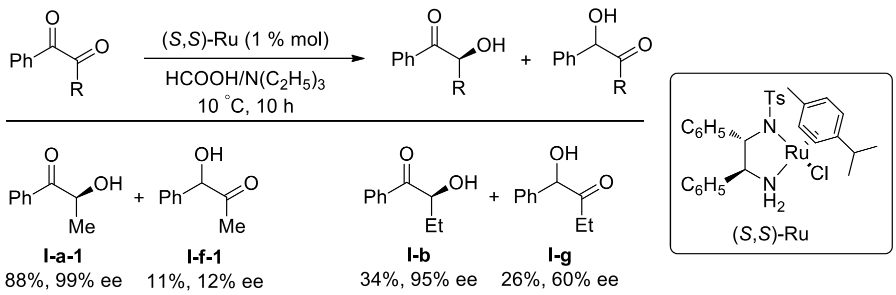

Supplementary Figure 369. Asymmetric reduction of 1,2-diketones

Chemical method III: asymmetric α-hydroxylation of ketones

Pd-catalyzed asymmetric hydroxylation of ketones has been reported[30], but as shown in the following **Supplementary Figure 370**, lower than 75% ee was observed.

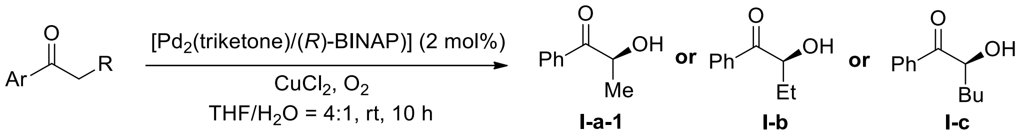

I-a-1 64%, 68% ee; **I-b** 61%, 71% ee; **I-c** 64%, 67% ee

Supplementary Figure 370. Asymmetric α-hydroxylation of ketonesChemical method IV: catalytic kinetic resolution

Oestreich et al has reported a kinetic resolution of hydroxyl imines. The recovered enantioenriched hydroxyl imine can be transformed to *ent*-**I-a-1** in 35% total yield with 98% ee (**Supplementary Figure 371**)[31].

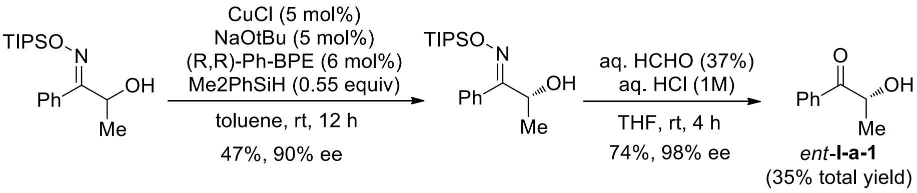

Supplementary Figure 371. Catalytic kinetic resolution (I)

Zhao reported a divergent resolution of racemic diol to afford **I-a-1** and **I-f-1** in low yields with 72% and 91% ee, respectively (**Supplementary Figure 372**)[32].

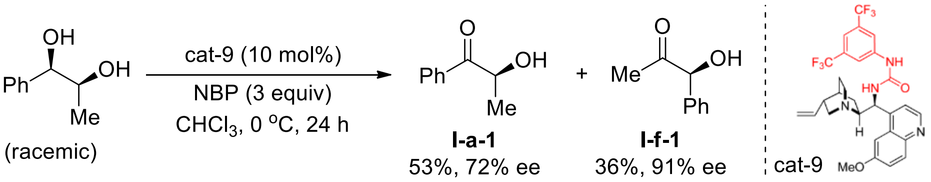

Supplementary Figure 372. Catalytic kinetic resolution (II)

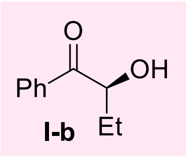

The above literature reports have shown that the asymmetric synthesis of **I-b** has been a trouble since almost no method can afford **I-b** in >60% yield with >95% ee. Ref 33 reported a method using stoichiometric chiral oxidant to get **I-b** but in 22% total yield with about 93% ee (**Supplementary Figure 373**).

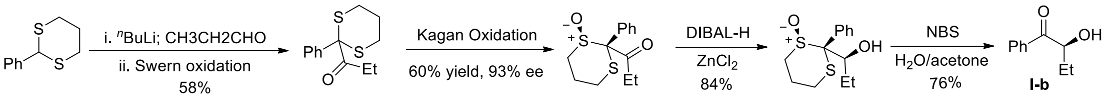

Supplementary Figure 373. Asymmetric synthesis of **I-b**

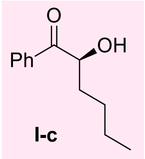

Besides the above method shown in Scheme 9, no more report was found to get **I-c**.

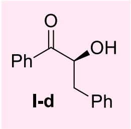

Maruoka and co-workers developed an elegant kinetic resolution method to afford the TES-protected **I-d** in 49% yield with 86% ee (**Supplementary Figure 374-a**)[34]. Starting from chiral amino acid, David et al also achieved the synthesis of **I-d** using three steps in 37% total yield, but the final ee was not shown

(**Supplementary Figure 374**-b)[35].

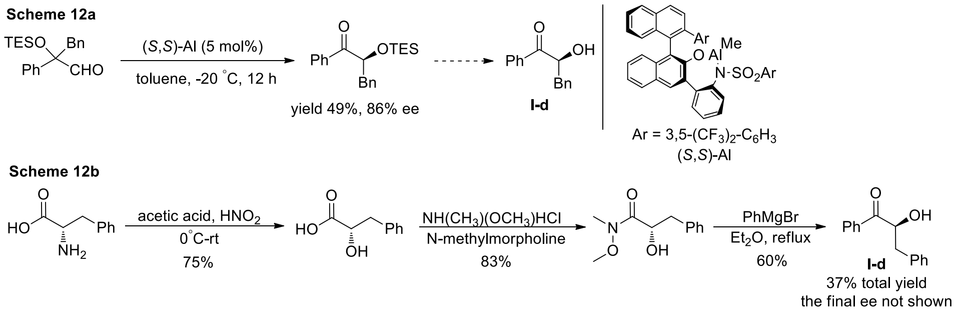

Supplementary Figure 374. Asymmetric synthesis of **I-d**

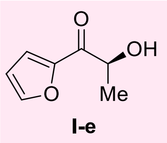

No other report was found to make **I-e** besides the method shown in Scheme 5a.

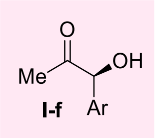

The asymmetric synthesis of **I-f** As has been shown in **Supplementary Figures 365-367** and **369,** but all results were not good. Until 2014, Müller and co-workers achieved the enzymatic synthesis of *ent*-**I-f-1** in good yield with 99% ee (**Supplementary Figure 375**)[36].

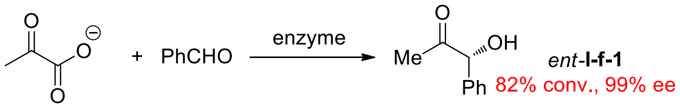

Supplementary Figure 375. Asymmetric synthesis of *ent*-**I-f-1**

Later Massi et al also achieved the asymmetric formation of **I-f-1** with good results, together with the formation of **I-g** with excellent ee (**Supplementary Figure 376**)[37].

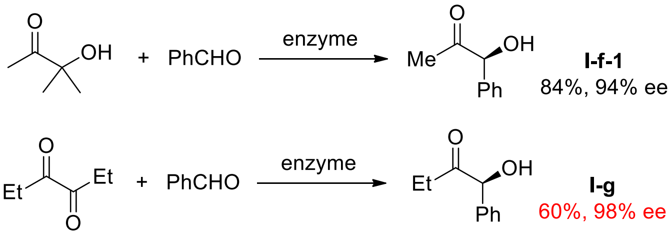

Supplementary Figure 376. Asymmetric synthesis of **I-f-1** and **I-g**

An enzyme-catalyzed 1,2-diketone reduction has also been developed to get *ent*-**I-f-1** but with 86% ee (**Supplementary Figure 377**)[38].

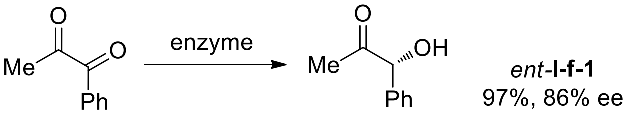

Supplementary Figure 377. Enzyme-catalyzed asymmetric synthesis of **I-f-1**

Extremely few chemical method has been reported to afford **I-f**. Shibasaki ever described a kinetic resolution method to get **I-f-2** but with only 33% ee (**Supplementary Figure 378**)[39].

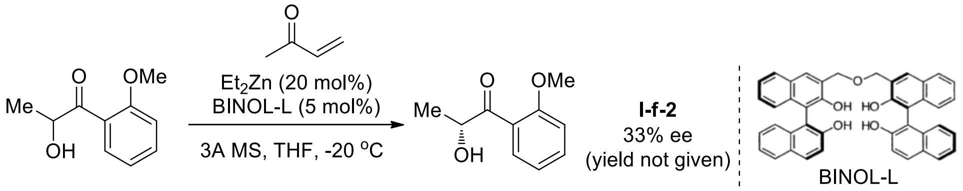

Supplementary Figure 378. Asymmetric synthesis of **I-f-2**

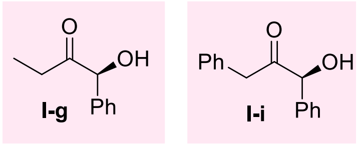

Besides the above methods in **Supplementary Figures 365**, **367**, and **369**, an enzyme-catalyzed reduction of ketones followed by ozonolysis afforded **I-g** with 99% ee, but in only 39% yield (**Supplementary Figure 379**-a)[40]. **NHC-1** catalyzed benzoin reaction has been reported to give both **I-g** and **I-i** with moderated ee values (**Supplementary Figure 379**-b)[41]. A relatively tedious four-step method has been developed to release both **I-g** and **I-i** with excellent ee values, but in low total yields (**Supplementary Figure 379**-c)[42].

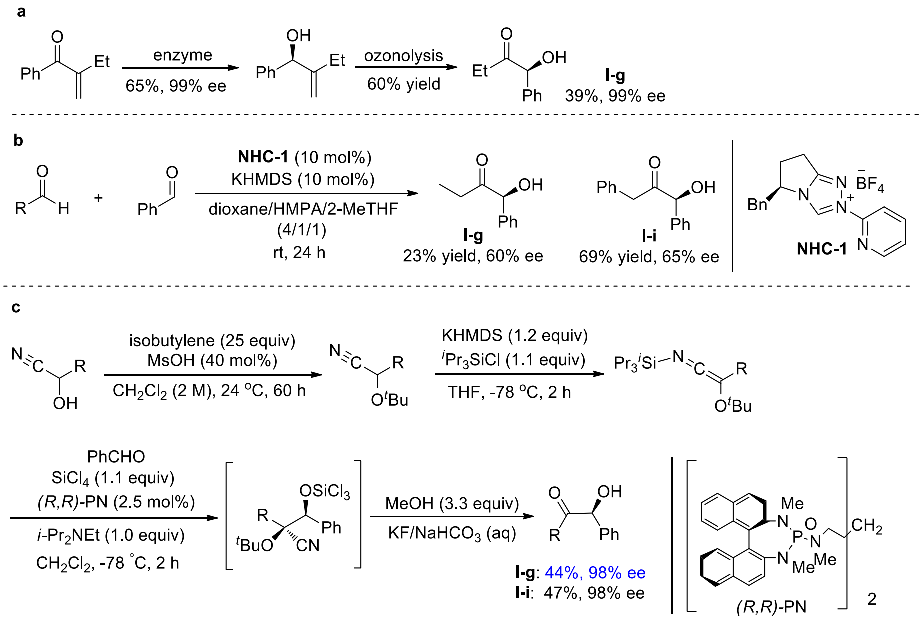

Supplementary Figure 379. Asymmetric synthesis of **I-g** and **I-i**

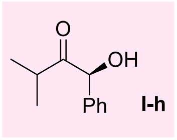

Enzyme method to make **I-h** has been reported by de Mar ía and co-workers[43]. The reaction afforded the 1:1 mixture of **I-h** and another reduction product (**Supplementary Figure 380**).

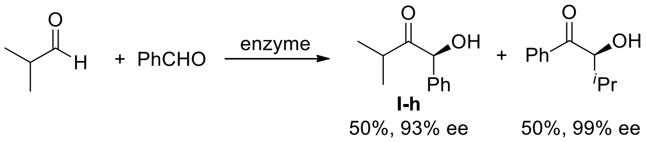

Supplementary Figure 380. Asymmetric synthesis of **I-h**

A catalytic kinetic resolution method has been developed to recover **I-h** in 48% yield with 92% ee (**Supplementary Figure 381**)[44]. The work can also produce **I-g** and **I-i**.

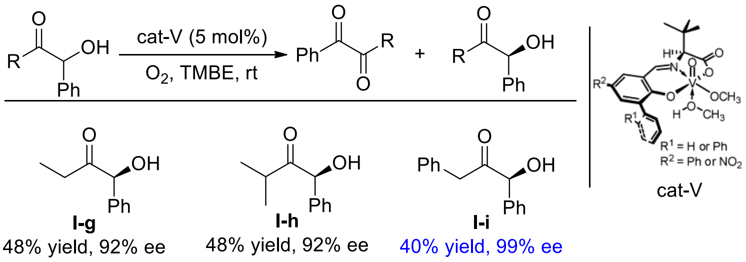

Supplementary Figure 381. Asymmetric synthesis of **I-g** and **I-i**

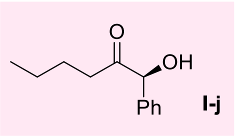

Only one report was found to make chiral **I-j** starting from (*R*,*R*)-1,3-dithiane-1,3-dioxide using a tedious six-step method (**Supplementary Figure 382**)[45]. The exact yield and the final ee were not given.

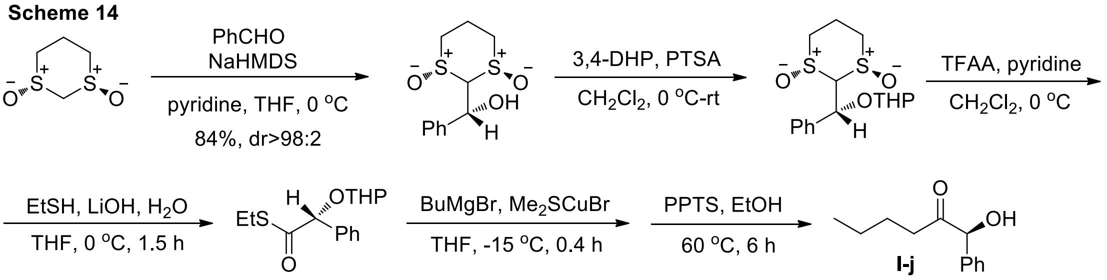

Supplementary Figure 382. Asymmetric synthesis of **I-j**

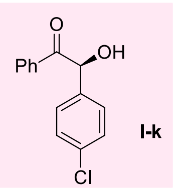

I-k is an representative benzoin type hydroxyl ketone. However, this type of compound is far from being obtained in satisfactory results. A seven-step synthesis

from chiral hydroxyl acid was reported, and the final yield was not given (**Supplementary Figure 383**)[46].

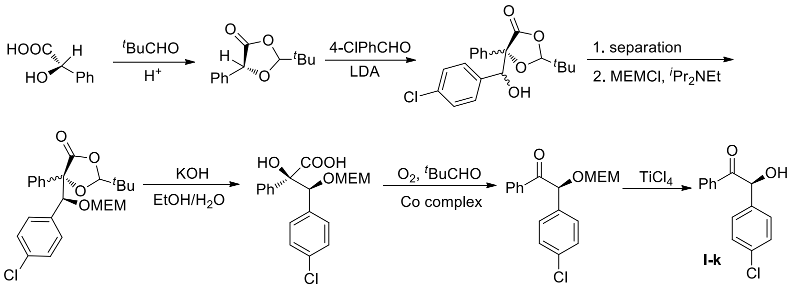

Supplementary Figure 383. Asymmetric synthesis of **I-k** via chrial source

Oxidative kinetic resolution methods have been developed to afford **I-k** but either with low ee or in low yield (**Supplementary Figure 384**)[47,48].

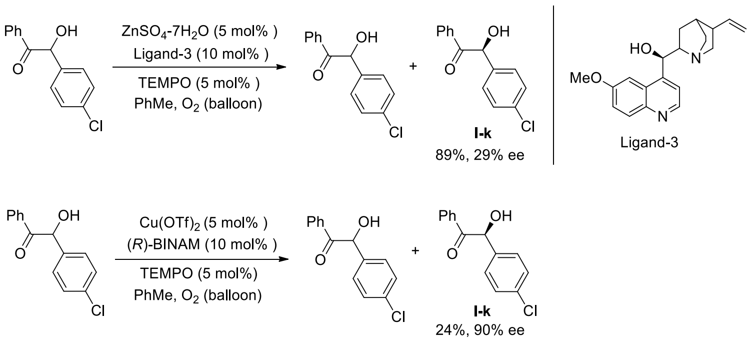

Supplementary Figure 384. Asymmetric synthesis of **I-k** via oxidative kinetic resolution

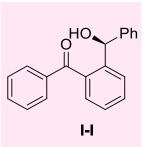

No method was found to make chiral **1-l** or its analogues.

Summary:

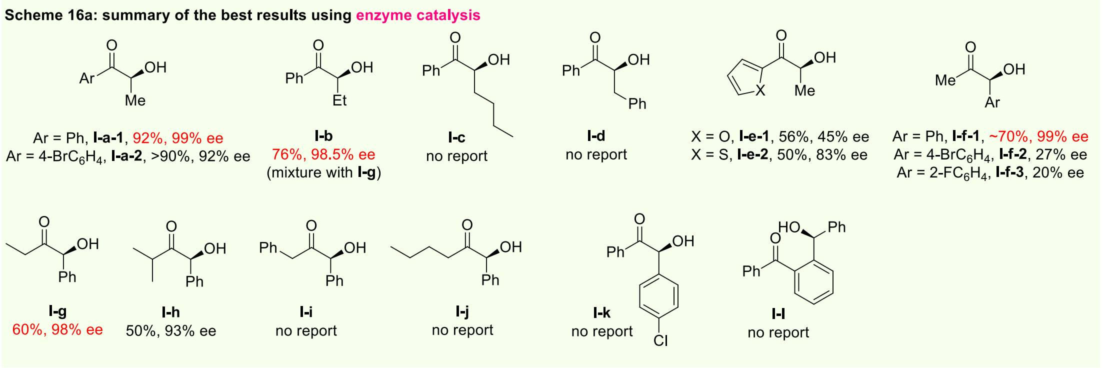

Supplementary Figure 385. Summary of the best results using enzymes methods

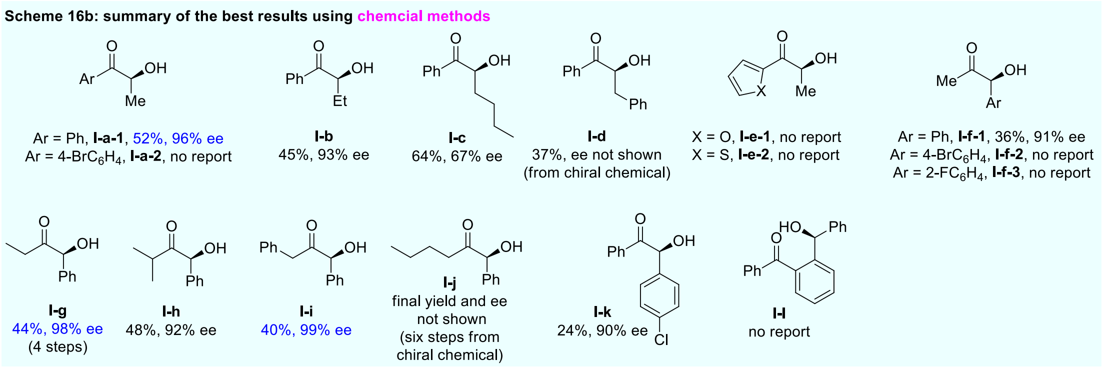

Supplementary Figure 386. Summary of the best results using chemical methods

A final summary of the best results using enzymes and chemical methods has been shown in **Supplementary Figure 385** and **386**, respectively. Overall, it is relatively surprising that, despite more than 30 different protocols having been developed so far to make enantioenriched α-hydroxy ketones, only four results (**I-a-1**, **I-b**, **I-f-1**, and **I-g**) meet the standard of >60% yield and >95% ee, and all of them use enzyme catalysis, although **I-b** was actually obtained as a mixture with **I-g** (**Supplementary Figure 385**). Also we should mention that only four specific hydroxyl ketones can be gotten with good results, not four types of hydroxyl ketones. Structures from **I-a** to **I-l** actually represent 12 types of hydroxyl ketones.

A more detailed analysis shows that enzyme catalysis is really very specific, since a small change of the substitution patterns greatly affects the final yield and ee.

In contrast, none of the chemical methods can meet the 60% yield/95% ee standard (**Supplementary Figure 386**). If only the ee is considered, then **I-g** and **I-i** are also OK; **I-d** and **I-j** were made from chiral chemicals through multiple steps, and the final ee values were not shown (racemization may happen). All other types of hydroxyl ketones still cannot be obtained in good results. Therefore, the state-of-art asymmetric synthesis of α-hydroxy ketones is far from being synthetically useful for the next step to get enantiopure diols we used in our work, and new methods are highly desired to improve the filed.

In sharp contrast, almost all the above racemic α-hydroxy ketones can be easily gotten using the following simple two-step method (**Supplementary Figure 387**). Moreover, we can draw a lot more types of α-hydroxy ketones that cannot be obtained in highly asymmetric fashion, but can be simply produced as the racemic form.

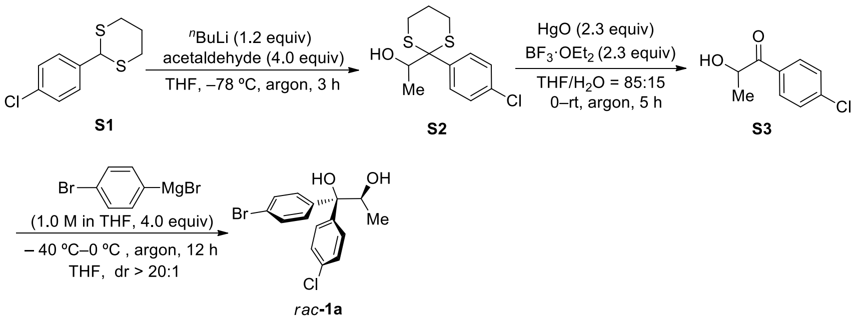

Supplementary Figure 387. A general method to make racemic **1a**

Asymmetric Synthesis of Enantiopure Esters with β-Hydroxyl Groups

Again, as long as we can get **II** in enantiopure form, the synthesis of **5a-5o** in Figure 4 of the manuscript should be easy (**Supplementary Figure 388**).

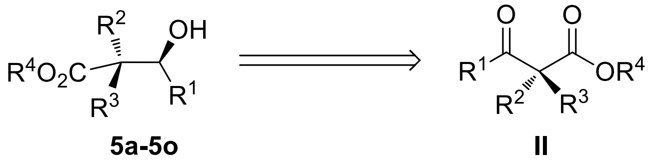

Supplementary Figure 388. A general method to synthesis of **5a-5o**

The following **Supplementary Figure 389** summarizes all seven types of ketoesters we used in our work, and a comprehensive literature search found that compounds from **II-a** to **II-e** are all not available even with low ee values, and there are only racemic synthetic reports before 2021.01[49-56]. During the submission of this manuscript, Feng and co-workers achieved the asymmetric synthesis of **II-a**, **II-c**, and **II-d**[56]. However, only methyl products were obtained with >90% ee.

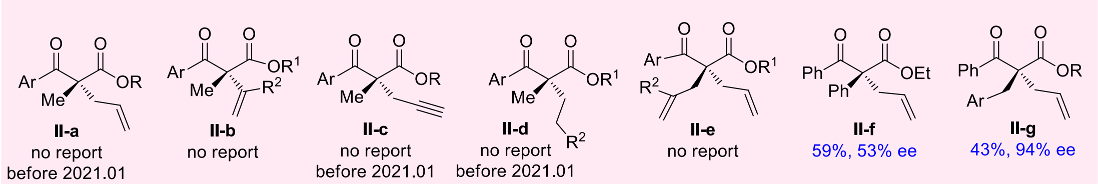

Supplementary Figure 389. Types of enantiopurity ketoesters in our work

Chen and co-workers reported a rearrangement reaction affording a series of racemic α-aryl ketoesters. They screened several chiral ligands and gave **II-f** with 53% ee as the best result (**Supplementary Figure 390**)[57].

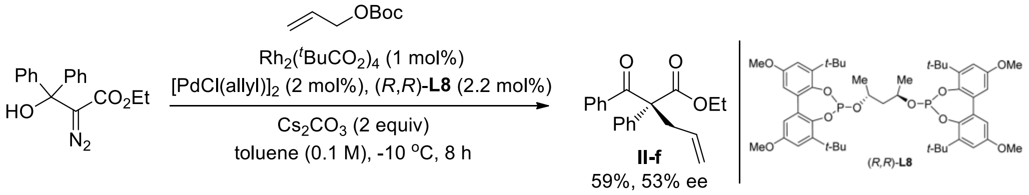

Supplementary Figure 390. Asymmetric synthesis of **II-f** via rearrangement reaction

There is only one report allowing access to **II-g-1**, which was reported by our group (**Supplementary Figure 391**)[58]. The method employs intramolecular benzoin reaction to achieve the kinetic resolution of ketoesters with a formyl benzyl group.

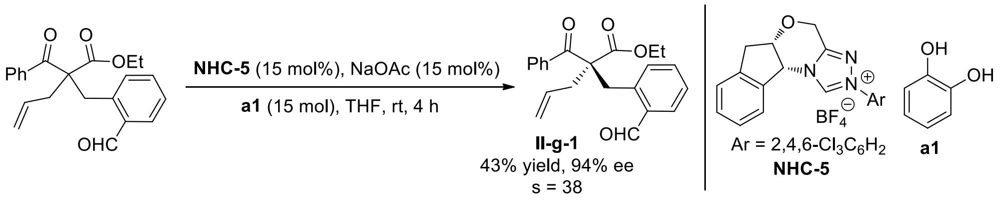

Supplementary Figure 391. Asymmetric synthesis of **II-g-1** via benzoin reaction

Hartwig at co-workers ever reported an elegant Ir-catalyzed asymmetric allylation of ketoesters (**Supplementary Figure 392**)[59]. The protocol afforded **II-h** type branched products with high ee, and **II-i** type products were obtained as a minor one. Noteworthy is that the method cannot produce products with a simple allyl group. Pd-catalyzed asymmetric allylation has been widely studied, but usually applicable for Me-ketone esters[60-66].

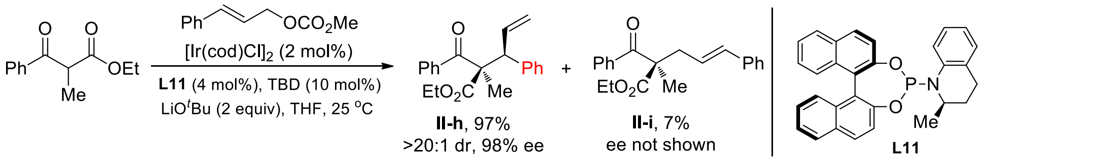

Supplementary Figure 392. Asymmetric synthesis of **II-h** via Ir-catalyzed asymmetric allylation

Notably, **Supplementary Figure 389** only lists a limited number of disubstituted ketoesters, and we can draw a lot more types of structures that can be easily obtained in racemic form, but hard to be gotten in enantiopure fashion. Such an inability of asymmetric synthesis in the access to enantiopure α,α-disubtituted β-ketoesters has been analyzed in our recent review paper[67].

Asymmetric Synthesis of Epoxy Alcohols

In our work we synthesized racemic epoxy alcohols using the following method (**Supplementary Figure 393**). Therefore, if we can get epoxy ketones in high ee values, it is easy to produce enantiopure epoxy alcohols in satisfactory results.

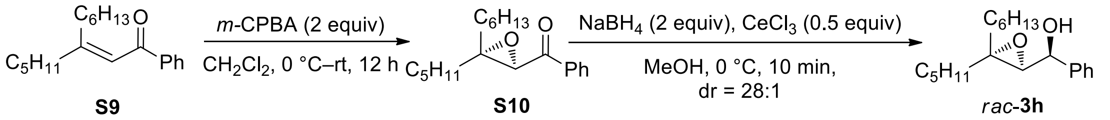

Supplementary Figure 393. A general method to make racemic **3h**

However, the asymmetric epoxidation of β,β-disubstituted enones turns out to be an extremely underdeveloped field because of the stereocongestion at the β-carbon. Not until 2011, Yamamoto and co-workers reported the first catalytic asymmetric epoxidation of β,β-disubstituted enones. **III-a** can be gotten with 91% ee, but for dialkyl substituted III-b, only 20% yield and 50% ee were observed. For **III-c** that derived from *cis*-enone, 33% yield and 6% ee were detected[68]. All these have shown the substrate limitation of the method (**Supplementary Figure 394**).

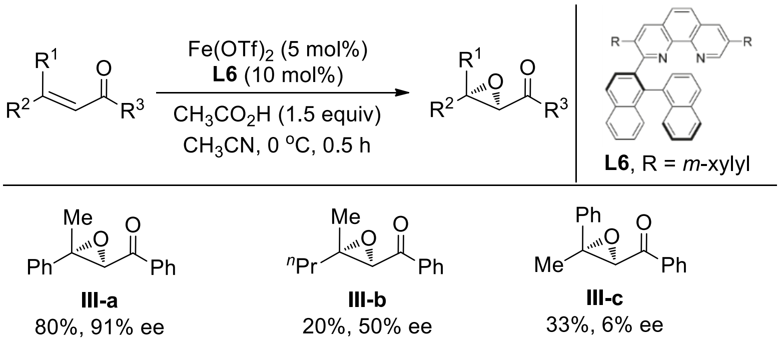

Supplementary Figure 394. Asymmetric synthesis of **III-a** via asymmetric epoxidation

The Chen group disclosed a highly selective asymmetric epoxidation of CF_3/aryl disubstituted enones, and **III-d** can be formed in 99% ee. They did not test substrates with two aryl groups and two alkyl groups such as CF_3/CH_3 (**Supplementary Figure 395**)[69]. A similar report was also reported by Shibata and co-workers[70].

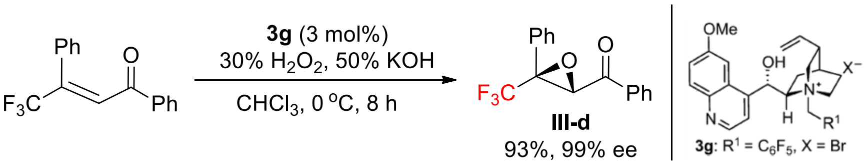

Supplementary Figure 395. Asymmetric synthesis of **III-d** via asymmetric epoxidation

A two-step transformation was described by Zhang *et al.*. They used Cu(II)-catalyzed enantioselective β-boration of β,β-disubstituted enones for the construction of a CF_3 and boron contained quaternary stereocenter[71]. Epoxy ketone **III-e** was formed via a further step but only 24% ee remained (**Supplementary Figure 396**).

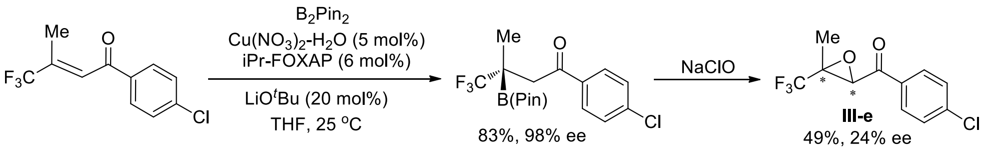

Supplementary Figure 396. Asymmetric synthesis of **III-d** via asymmetric Cu(II) catalysis

Using Sharpless asymmetric epoxidation and the following oxidation, Molander and co-workers obtained **III-f**, but the epoxidation step produce **IV-a** with only 18% ee (**Supplementary Figure 397**-a)[72]. A seven-step synthesis of **III-g** has been revealed by Lindel et al. (**Supplementary Figure 397**-b)[73]. The key sharpless epoxidation step afforded **IV-b** in 88% yield with 89% ee.

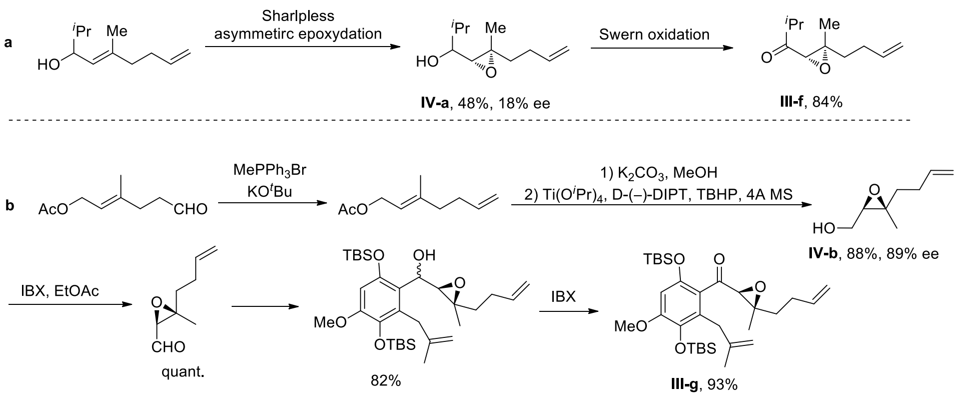

Supplementary Figure 397. Asymmetric synthesis of **III-d** via Sharpless asymmetric epoxidation

Conclusion:

Currently there is no efficient method allowing access to the following epoxy ketones in high yield and ee:

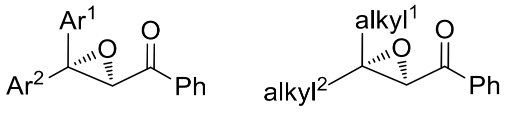

Another method that can be easily imagined is getting enantiopure allylic alcohols first, and then using a diastereoselective epoxidation to get the final epoxy alcohols (**Supplementary Figure 398**).

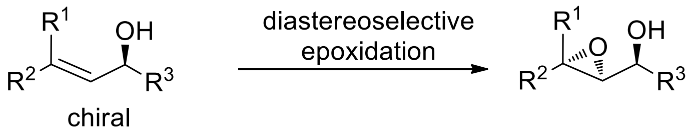

Supplementary Figure 398. Enantiopure epoxy alcohols via enantiopure allylic alcohols

The following **Supplementary Figure 399** lists many examples that can be effectively reduced in an enatioselective fashion[74-78]. Generally, such a field has been a developed one and the asymmetric synthesis of the enantiopure allylic alcohols used for the preparation of the epoxy alcohols used in our work should be possible.

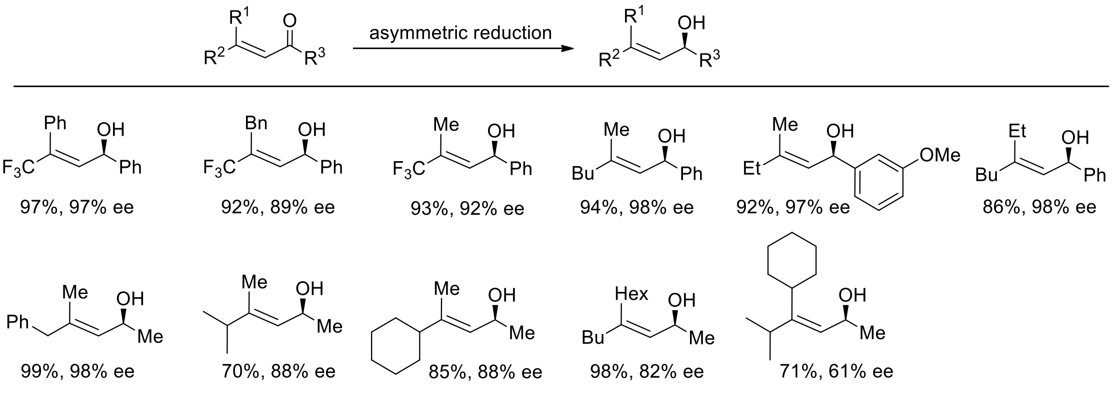

Supplementary Figure 399. Asymmetric synthesis of the enantiopure allylic alcohols

Using Sharpless epoxidation-mediated kinetic resolution, the following allylic alcohols can also be obtained (**Supplementary Figure 400**)[79].

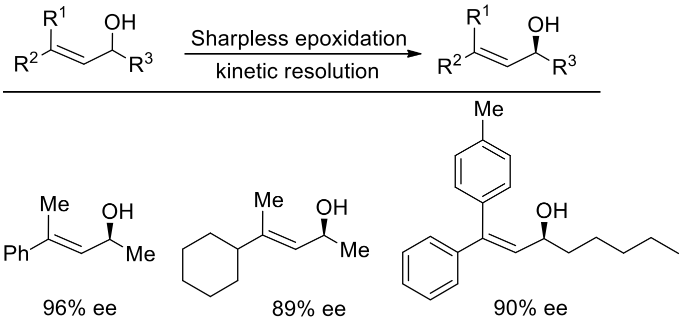

Supplementary Figure 400. Asymmetric synthesis of the enantiopure allylic alcohols via Sharpless epoxidation-mediated kinetic resolution

Hence, if the next diastereoselective epoxidation step is OK, then the final asymmetric synthesis of the epoxy alcohols used in our work can be gotten. However, as listed in the following **Supplementary Figure 401**-a, we have already tried the epoxidation of diaryl allylic alcohol using *m*CPBA, but failed to get **IV-c**, probably owing to the congestion of epoxy unit made by the two aryl groups, which leads to the instability of **IV-c** in acidic conditions. Using Sharpless conditions, the epoxy alcohol **IV-d** can be gotten in low yields, however, the major product is *cis*- (**Supplementary Figure 401**-b). In contrast, the reduction of epoxy ketones under mild $NaBH_4$ conditions leads to **IV-e** with *trans*- as the major diastereomer (**Supplementary Figure 401**-c).

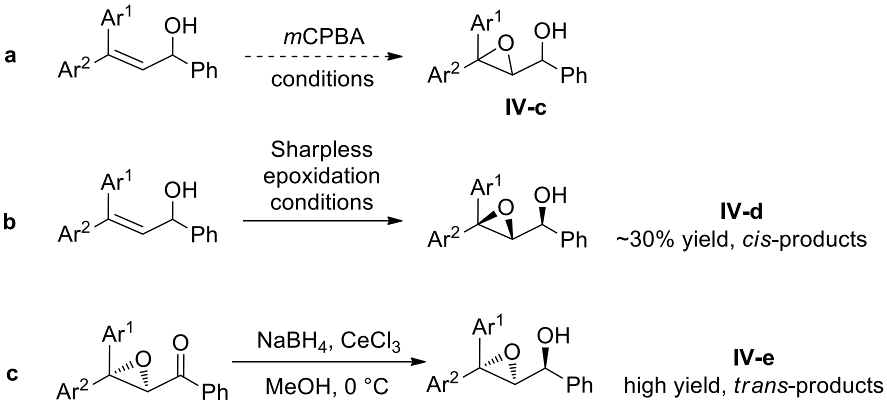

Supplementary Figure 401. Asymmetric synthesis of the epoxy alcohols via different ways

Summary:

The reduction of epoxy ketones and the epoxidation of allylic alcohols give different diastereomers of epoxy alcohols. Therefore, most of the epoxy alcohols used in our work are currently cannot be readily obtained in high enantiopurity using known methods.

Concluding Remarks:

Despite the great achievements having been obtained in the field of asymmetric catalysis, there are still many troubles that cannot be well solved to date even in well-studied fields. During the last several years, we conducted a series of work trying to address them[58,67,80-87], and this work is one of the series. We sincerely thank global experts for their supports.

Supplementary References:

1. Contente, L., Serra, I., Brambilla, M., Eberini, I., Gianazza, E., Vitis, V. D., Molinari, F., Zambelli, P. & Romano, D. Stereoselective reduction of aromatic ketones by a new ketoreductase from Pichia glucozyma. *Appl. Microbiol. Biotechnol*. **100**, 193−201 (2016).
2. Dall'Oglio, F., Contente, M. L., Conti, P., Molinari, F., Monfredi, D., Pinto, A., Romano, D., Ubiali, D., Tamborini, L. & Serra, I. Flow-based stereoselective reduction of ketones using an immobilized ketoreductase/glucose dehydrogenase mixed bed system. *Catal. Commun*. **93**, 29−32 (2017) .
3. Chênevert, R. & Thiboutot, S. Baker's yeast reduction of 1,2-diketones. Preparation of pure (*S*)-(-)-2-hydroxy-1-phenyl-1-propanone. *Chem. Lett.* 1191–1192 (1988).
4. Andreu, C. & Lí del Olmo, M. Comparison of the biocatalytic activity of some halotolerant yeasts in freshwater and seawater. *ChemBioChem.* **21**, 1621−1628 (2020).
5. Mahajabeen, P. & Chadha, A. Regio- and enantioselective reduction of diketones: preparation of enantiomerically pure hydroxy ketones catalysed by Candida parapsilosis ATCC 7330. *Tetrahedron: Asymmetry*. **26**, 1167−1173 (2015).
6. Médici, R., Stammes, H., Kwakernaak, S., Otten, L, G. & Hanefeld, U. Assessing the stereoselectivity of Serratia marcescens CECT 977 2,3-butanediol dehydrogenase. *Catal. Sci. Technol.* **7**, 1831−1837 (2017).
7. Nakamura, K., Kondo, S., Kawai, Y., Hida, K., Kitano, K. & Ohno, A. Enantio-and regioselective reduction of α-diketones by Baker's yeast. *Tetrahedron: Asymmetry.* **7**, 409−412 (1996).
8. Bortolini, O., Fantin, G., Fogagnolo, M., Giovannini, P. P., Guerrini, A. & Medici, A. An easy approach to the synthesis of optically active vic-diols: a new single-enzyme system. *J. Org. Chem.* **62**, 1854−1856 (1997).
9. Kawai, Y., Hida, K., Tsujimoto, M., Kondo, S., Kitano, K., Nakamura, K. & Ohno, A. Asymmetric reduction of α-keto esters and α-diketones with a Bakers' yeast keto ester reductase. *Bull. Chem. Soc. Jpn.* **72**, 99−102 (1999).
10. Dominguez de Maria, P., Stillger, T., Pohl, M., Kiesel, M., Liese, A., Groger, H. & Trauthwein, H. Enantioselective C–C bond ligation using recombinant escherichia coli-whole-cell biocatalysts. *Adv. Synth. Catal.* **350**, 165−173 (2008).
11. Berheide, M., Kara, S. & Liese, A. Reversibility of asymmetric catalyzed C–C bond formation by benzoylformate decarboxylase. *Catal. Sci. Technol*. **5**, 2418−2426 (2015).

12. Wachtmeister. J., Jakoblinner, A. & Rother. D. Stereoselective two-step biocatalysis in organic solvent: toward all stereoisomers of a 1,2-diol at high product concentrations. *Org. Process Res. Dev.* **20**, 1744−1753 (2016).
13. Tural, B., Tarhan, T. & Tural, S. Covalent immobilization of benzoylformate decarboxylase from pseudomonas putida on magnetic epoxy support and its carboligation reactivity. *J. Mol. Catal. B Enzym.* **102**, 188–194 (2014).
14. Tural, B., Tural, S. & Demir, A. S. Carboligation reactions mediated by benzoylformate decarboxylase immobilized on a magnetic solid support. *Chirality,* **25**, 415–421(2013).
15. Tural, B., Tural, S. & Demir, A. S. Heterofunctional magnetic metal-chelate-epoxy supports for the purification and covalent immobilization of benzoylformate decarboxylase from pseudomonas putida and its carboligation reactivity. *Chirality,* **27**, 635–642 (2015).
16. Iding, H., Dünnwald, T., Greiner, L., Liese, A., Müller, M., Siegert, P., Grötzinger, J., Demir, A. S. & Pohl, M. Benzoylformate decarboxylase from pseudomonas putida as stable catalyst for the synthesis of chiral 2-hydroxy ketones. *Chem. Eur. J.* **6**, 1483−1495 (2000).
17. Beigi, M., Gauchenova, E., Walter, L., Waltzer, S., Bonina, F., Stillger, T., Rother, D., Pohl, M. & Müller, M. Regio-and stereoselective aliphatic-aromatic cross-benzoin reaction: enzymatic divergent catalysis. *Chem. Eur. J.* **22**, 13999−14005 (2016).
18. Demir, A. S., Şeşenoglu, Ö., Eren, E., Hosrik, B., Pohl, M., Janzen, E., Kolter, D., Feldmann, R., Dünkelmann, P. & Müller, M. Enantioselective synthesis of α-hydroxy ketones via benzaldehyde lyase-catalyzed C–C bond formation reaction. *Adv. Synth. Catal.* **344**, 96−103 (2002).
19. Baykal, A., Chakraborty, S., Dodoo, A. & Jordan, F. Synthesis with good enantiomeric excess of both enantiomers of α-ketols and acetolactates by two thiamin diphosphate-dependent decarboxylases. *Bioorganic Chemistry* **34,** 380–393 (2006).
20. Pérez-Sánchez, M., Müller, C. R. & de María, D. Multistep oxidase-lyase reactions: synthesis of optically active 2-hydroxyketones by using biobased aliphatic alcohols. *ChemCatChem.* **5**, 2512–2516 (2013).
21. Tural, B., Şimşek, İ., Tural, S., Çelebi, B. & Demir, A. S. Carboligation reactivity of benzaldehyde lyase (BAL, EC 4.1. 2.38) covalently attached to magnetic nanoparticles. *Tetrahedron: Asymmetry.* **24**, 260–268 (2013).
22. Agudo, R., Roiban, G.-D., Lonsdale, R., Ilie, A. & Reetz, M. T. Biocatalytic route to chiral acyloins: P450-catalyzed regio-and enantioselective α-hydroxylation of ketones. *J. Org. Chem.* **80**, 950-956 (2015).
23. Adam, W., Fell, R. T., Stegmann, V. R. & Saha-Möller, C. R. Synthesis of optically active α-hydroxy carbonyl compounds by the catalytic, enantioselective oxidation of silyl enol ethers and ketene acetals with (salen)manganese(III) complexes. *J. Am. Chem. Soc.* **120**, 708−714 (1998).
24. Koprowski, M., Łuczak, J. & Krawczyk, E. Asymmetric oxidation of enol phosphates to α-hydroxy ketones by (salen)manganese(III) complex. Effects of the

substitution pattern of enol phosphates on the stereochemistry of oxygen transfer. *Tetrahedron*. **62**, 12363−12374 (2006).

25. Krawczyk, E., Mielniczak, G., Owsianik, K. & Łuczak, J. Asymmetric oxidation of enol phosphates to α-hydroxy ketones using Sharpless reagents and a fructose derived dioxirane. *Tetrahedron: Asymmetry*. **23**, 1480–1489 (2012).
26. Davis F. A., Sheppard, A. C., Chen, B.-C. & Haque, M. S. Chemistry of oxaziridines. 14. Asymmetric oxidation of ketone enolates using enantiomerically pure (camphorylsulfonyl) oxaziridine. *J. Am. Chem. Soc.* **112**, 6679–6690 (1990).
27. Davis, F. A. & Chen, B.-C. Asymmetric hydroxylation of enolates with N-sulfonyloxaziridines. *Chem. Rev.* **92**, 919–934 (1992).
28. Davis, F. A. & Haque, M. S. Stereochemistry of the asymmetric oxidation of ketone enolates using (camphorylsulfonyl) oxaziridines. *J. Org. Chem.* **51**, 4083–4085 (1986).
29. Koike, T., Murata, K. & Ikariya, T. Stereoselective synthesis of optically active α-hydroxy ketones and anti-1,2-diols via asymmetric transfer hydrogenation of unsymmetrically substituted 1,2-diketones. *Org. Lett*. **2**, 3833−3836 (2000).
30. Hamed, O. A., ElQisairi, A., Qaseer, H., Hamed, E. M., Henry, P. M. & Becker, D. P. Asymmetric α-hydroxy ketone synthesis by direct ketone oxidation using a bimetallic palladium(II) complex. *Tetrahedron Lett.* **53**, 2699−2701 (2012).
31. Dong, X., Kita, Y. & Oestreich, M. Kinetic resolution of α-hydroxy-substituted oxime ethers by enantioselective Cu-H-catalyzed Si-O coupling. *Angew. Chem. Int. Ed.* **57**, 10728−10731 (2018).
32. Rong, Z.-Q., Pan, H.-J., Yan, H.-L. & Zhao, Y. Enantioselective oxidation of 1, 2-diols with quinine-derived urea organocatalyst. *Org. Lett.* **16**, 208−211 (2014).
33. Philip, P., Mark, P. & David, L. Enantioselective synthesis of α-hydroxyketones using the DiTOX asymmetric building block. *Tetrahedron Lett.* **37**, 8929–8932 (1996).
34. Ooi, T., Ohmatsu, K. & Maruoka, K. Catalytic asymmetric rearrangement of α, α-disubstituted α-siloxy aldehydes to optically active acyloins using axially chiral organoaluminum Lewis acids. *J. Am. Chem. Soc.* **129**, 2410–2411 (2007).
35. Giordani, A., Carera, A., Pinciroli, V. & Cozzi, P. Nucleophilic substitution on α-mesyloxy-O-alkyloximes–I. Enantiospecific synthesis of 2-(imidazol-1-yl)-1, 3-diphenylpropan-1-one O-alkyloximes. *Tetrahedron: Asymmetry*. **8**, 253–263 (1997).
36. Loschonsky, S., Wacker, T., Waltzer, S., Giovannini, P. P., McLeish, M. J., Andrade, S. L. A. & Müller, M. Extended reaction scope of thiamine diphosphate dependent cyclohexane-1,2-dione hydrolase: from C–C bond cleavage to C–C bond ligation. *Angew. Chem. Int. Ed*. **53**, 14402–14406 (2014).
37. Giovannini, P. P., Lerin, L. A., Müller, M., Bernacchia, G., De Bastiani, M., Catani, M., Di Carmine, G. & Massi, A. (*S*)-Selectivity in phenylacetyl carbinol synthesis using the wild-type enzyme acetoin: dichlorophenolindophenol oxidoreductase from *Bacillus licheniformis*. *Adv. Synth. Catal.* **358**, 2767−2776 (2016).
38. Muschallik, L., Molinnus, D., Jablonski, M., Kipp, C. R., Bongaerts, J., Pohl, M., Wagner, T., Schöning, M. J., Selmer, T. & Siegert, P. Synthesis of α-hydroxy

ketones and vicinal (*R*,*R*)-diols by Bacillus clausii DSM 8716 T butanediol dehydrogenase. *RSC Adv.* **10**, 12206−12216 (2020).
39. Harada, S., Kumagai, N., Kinoshita, T., Matsunaga, S. & Shibasaki, M. Direct catalytic asymmetric Michael Reaction of hydroxyketones: asymmetric Zn catalysis with a Et_2Zn/Linked-BINOL complex. *J. Am. Chem. Soc.* **125**, 2582−2590 (2003).
40. Conceição, G. J. A., Moran, P. J. S. & Rodrigues, J. A. R. Highly efficient extractive biocatalysis in the asymmetric reduction of an acyclic enone by the yeast *Pichia stipites*. *Tetrahedron Asymmetry* **14**, 43−45 (2003).
41. Soeta, T., Mizuno, S., Hatanaka, Y. & Ukaji, Y. Asymmetric cross-benzoin condensation promoted by a chiral triazolium precatalyst bearing a pyridine moiety. *Tetrahedron*, **73**, 3430−3437 (2017).
42. Denmark, S. E. & Wilson, T. W. N-silyl oxyketene imines are underused yet highly versatile reagents for catalytic asymmetric synthesis. *Nat. Chem.* **2**, 937−943 (2010).
43. Müller, C. R., Pérez-Sánchez, M. & de María, P. D. Benzaldehyde lyase-catalyzed diastereoselective C–C bond formation by simultaneous carboligation and kinetic resolution. *Org. Biomol. Chem.* **11**, 2000–2004 (2013).
44. Chen, C. T., Kao, J. Q., Salunke, S. B. & Lin, Y. H. Enantioselective aerobic oxidation of α-hydroxy-ketones catalyzed by oxidovanadium(V) methoxides bearing chiral, N-salicylidene-tert-butylglycinates. *Org. Lett.* **13**, 26−29 (2011).
45. Aggarwal, V. K., Thomas, A. & Schade, S. *trans*-1,3-Dithiane-1,3-dioxide; a chiral acyl anion equivalent. Enantioselective synthesis of α-hydroxy-carboxylic acids, esters, amides and ketones. *Tetrahedron* **53**, 16213−16228 (1997).
46. Blay, G., Fernandez, I., Monje, B. & Pedro, J. R. Enantioselective synthesis of unsymmetrical benzoins from (*S*)-mandelic acid enolate and aromatic aldehydes. *Tetrahedron Lett*. **45**, 8039−8042 (2004).
47. Muthupandi, P. & Sekar, G. Chiral Zn-catalyzed aerobic oxidative kinetic resolution of α-hydroxy ketones. *Tetrahedron: Asymmetry.* **22**, 512−517 (2011).
48. Alamsetti, S. K., Mannam, S., Mutupandi, P. & Sekar, G. Galactose oxidase model: biomimetic enantiomer-differentiating oxidation of alcohols by a chiral copper complex. *Chem. Eur. J.* **15**, 1086−1090 (2009).
49. Nakamura, M., Endo, K. & Nakamura, E. A modular approach to α-arylated carbonyl compounds via indium tris(bistriflylamide)-catalyzed regioselective addition of β-ketoesters to 1,3-diynes. *Adv. Synth. Catal.* **347**, 1681–1686 (2005).
50. Miura, T., Shimada, M. & Murakami, M. Acyl 1,3-migration in rhodium-catalyzed reactions of acetylenic β-ketoesters with aryl boronic acids: application to two-carbon-atom ring expansions. *Angew. Chem. Int. Ed.* **44**, 7598–7600 (2005).
51. Nakamura, M., Endo, K. & Nakamura, E. Indium-catalyzed addition of active methylene compounds to 1-alkynes. *J. Am. Chem. Soc.* **125**, 13002–13003 (2003).
52. Endo, K., Hatakeyama, T., Nakamura, M. & Nakamura, E. Indium-catalyzed 2-alkenylation of 1,3-dicarbonyl compounds with unactivated alkynes. *J. Am.*

Chem. Soc. **129**, 5264–5271 (2007).
53. Bacchi, A., Costa, M., Della Cà, N., Gabriele, B., Salerno, G. & Cassoni, S. Heterocyclic derivative syntheses by palladium-catalyzed oxidative cyclization-alkoxycarbonylation of substituted γ-oxoalkynes. *J. Org. Chem.* **70**, 4971–4979 (2005).
54. Lu, Z., Chai, G., Zhang, X. & Ma, S. Controllable highly stereoselective reaction of *in situ* generated magnesium dienolate intermediates with different electrophiles. *Org. Lett.* **10**, 3517–3520 (2008).
55. Saito, A., Enomoto, Y. & Hanzawa, Y. Pd-catalyzed cycloisomerization-allylation of 4-alkynones: synthesis of 5-homoallylfuran derivatives. *Tetrahedron Lett.* **52**, 4299–4302 (2011).
56. Tan, F., Pu, M., He, J., Li, J., Yang, J., Dong, S., Liu, X., Wu, Y.-D. & Feng, X. Catalytic asymmetric homologation of ketones with α-alkyl α-diazo esters. *J. Am. Chem. Soc.* **143**, 2394−2402 (2021).
57. Wang, X.-X., Huang, X.-Y., Lei, S.-H., Yang, F., Gao, J.-M., Ji K. & Chen, Z.-S. Relay Rh (II)/Pd (0) dual catalysis: synthesis of α-quaternary β-keto-esters via a [1, 2]-sigmatropic rearrangement/allylic alkylation cascade of α-diazo tertiary alcohols. *Chem. Commun.* **56**, 782–785 (2020).
58. Zehra, S. T., Zhang, G., Yang, S. & Fang, X. Kinetic resolution of β-ketoesters with quaternary stereocenters via a carbene-catalyzed benzoin reaction. *Org. Biomol. Chem.* **17**, 2169–2173 (2019).
59. Liu, W.-B., Reeves, C. M. & Stoltz, B. M. Enantio-, diastereo-, and regioselective iridium-catalyzed asymmetric allylic alkylation of acyclic β-ketoesters. *J. Am. Chem. Soc.* **135**, 17298–17301(2013).
60. Cheng, H.-G., Feng, B., Chen, L.-Y., Guo, W., Yu, X.-Y., Lu, L.-Q., Chen, J.-R. & Xiao, W.-J. Rational design of sulfoxide–phosphine ligands for Pd-catalyzed enantioselective allylic alkylation reactions. *Chem. Commun.* **50**, 2873–2875 (2014).
61. Zhou, H., Zhang, L., Xu, C. & Luo, S. Chiral primary amine/palladium dual catalysis for asymmetric allylic alkylation of β-ketocarbonyl compounds with allylic alcohols. *Angew. Chem., Int. Ed.* **54**, 12645–12648 (2015).
62. Liu, Q.-L., Chen, W., Jiang, Q.-Y., Bai, X.-F., Li, Z., Xu, Z. & Xu, L.-W. A D-camphor-based schiff base as a highly efficient N,P ligand for enantioselective palladium-catalyzed allylic substitutions. *ChemCatChem.* **8**, 1495–1499 (2016).
63. Zhao, M., Tian, Y. & Zhao, X. Thieme chemistry journals awardees-where are they now? Chiral sulfinamide ligands and Pd-catalyzed asymmetric allylic alkylations of ethyl 2-fluoroacetoacetate. *Synlett*, **28**, 1801–1806 (2017).
64. Xu, Y.-N., Zhu, M.-Z. & Tian, S.-K. Chiral α-amino acid/palladium-catalyzed asymmetric allylation of α-branched β-ketoesters with allylic amines: highly enantioselective construction of all-carbon quaternary stereocenters. *J. Org. Chem.* **84**, 14936–14942 (2019).
65. Wang, Y., Zhou, H., Yang, K., You, C., Zhang, L. & Luo, S. Steric effect of protonated tertiary amine in primary-tertiary diamine catalysis: a double-layered sterimol model. *Org. Lett.* **21**, 407–411 (2019).

66. Wang, Y., Chai, J., You, C., Zhang, J., Mi, X., Zhang, L. & Luo, S. π-Coordinating chiral primary amine/palladium synergistic catalysis for asymmetric allylic alkylation. *J. Am. Chem. Soc.* **142**, 3184–195 (2020).
67. Sun, D., Yang, S. & Fang, X. Asymmetric catalytic construction of fully substituted carbon stereocenters using acyclic α-branched β-ketocarbonyls: the "Methyl Rule" widely exists. *Org. Chem. Front.* **7**, 3557−3577 (2020).
68. Nishikawa, Y. & Yamamoto, H. Iron-catalyzed asymmetric epoxidation of β,β-disubstituted enones. *J. Am. Chem. Soc.* **133**, 8432–8435 (2011).
69. Wu, S., Pan, D., Cao, C., Wang, Q. & Chen, F.-X. Diastereoselective and enantioselective epoxidation of acyclic β-trifluoromethyl-β,β-disubstituted enones by hydrogen peroxide with a pentafluorinated quinidine-derived phase-transfer catalyst. *Adv. Synth. Catal.* **355**, 1917–1923 (2013).
70. Kawai, H., Okusu, S., Yuan, Z., Tokunaga, E., Yamano, A., Shiro, M. & Shibata, N. Enantioselective synthesis of epoxides having a tetrasubstituted trifluoromethylated carbon center: methylhydrazine-induced aerobic epoxidation of β,β-disubstituted enones. *Angew. Chem. Int. Ed.* **52**, 2221–2225 (2013).
71. Liu, B., Wu, H. & Zhang, J. Cu(II)-catalyzed enantioselective β-boration of β-trifluoromethyl, β,β-disubstituted enones and esters: construction of a CF_3 and boroncontained quaternary stereocenter. *ACS Catal.* **8**, 8318–8323 (2018).
72. Molander, G. A. & Losada, C. P. Sequenced reactions with samarium(II) iodide. Domino epoxide ring-opening/ketyl olefin coupling reactions. *J. Org. Chem.* **62**, 2935–2943 (1997).
73. Frichert, A., Jones, P. G. & Lindel, T. Enantioselective total synthesis of terreumols A and C from the mushroom *Tricholoma terreum*. *Angew. Chem. Int. Ed.* **55**, 2916–2919 (2016).
74. Bizet, V., Pannecoucke, X., Renaud, J.-L. & Cahard, D. Ruthenium-catalyzed redox isomerization of trifluoromethylated allylic alcohols: mechanistic evidence for an enantiospecific pathway. *Angew. Chem. Int. Ed.* **51**, 6467–6470 (2012).
75. Wu, M., Kong, L., Wang, K., Jin, R., Cheng, T. & Liu, G. Enantioselective 1,2-reductions of β-trifluoromethylated-α,β-unsaturated ketones to chiral allylic alcohols over organoruthenium-functionalized mesoporous silica nanospheres. *Catal. Sci. Technol.* **5**, 1750–1757 (2015).
76. Xia, X., Wu, M., Jin, R., Cheng, T. & Liu, G. One-pot relay reduction-isomerization of β-trifluoromethylated-α,β-unsaturated ketones to chiral β-trifluoromethylated saturated ketones over combined catalysts in aqueous medium. *Green Chem.* **17**, 3916–3922 (2015).
77. Cobb, K. M., Rabb-Lynch, J. M., Hoerrner, M. E., Manders, A., Zhou, Q. & Watson, M. P. Stereospecific, nickel-catalyzed Suzuki–Miyaura cross-coupling of allylic pivalates to deliver quaternary stereocenters. *Org. Lett.* **19**, 4355–4358 (2017).
78. Narczyk, A. & Stecko, S. An entry to non-racemic β-tertiary-β-amino alcohols, building blocks for the synthesis of aziridine, piperazine, and morpholine scaffolds. *Org. Biomol. Chem.* **18**, 5972−5981 (2020).
79. Le, H., Batten, A. & Morken, J. P. Catalytic stereospecific allyl-allyl

cross-coupling of internal allyl electrophiles with AllylB(pin). *Org. Lett.* **16**, 2096−2099 (2014).

80. Liu, J., Vasamsetty, L., Anwar, M., Yang, S., Xu, W., Liu, J., Nagaraju, S. & Fang, X. Organocatalyzed kinetic resolution of α-functionalized ketones: the malonate unit leads the way. *ACS Catal*. **10**, 2882−2893 (2020).
81. Zhao, Z., Bagdi, P. R., Yang, S., Liu, J., Xu, W. & Fang, X. Stereodivergent access to enantioenriched epoxy alcohols with three stereogenic centers via ruthenium-catalyzed transfer hydrogenation. *Org. Lett.* **14**, 5491−5494 (2019).
82. Zhao, Z., Yang, S., Lan, S., Liu, J., Liu, S. & Fang, X. Asymmetric synthesis of dihydronaphthalene-1,4-diones via carbene-catalyzed stereodivergent reaction. *Adv. Synth. Catal.* **361**, 3943–3949 (2019).
83. Perveen, S., Yang, S., Meng, M., Xu, W., Zhang, G. & Fang, X. Asymmetric total synthesis of rotenoids via organocatalyzed dynamic kinetic resolution. *Communications Chemistry* **2**:8 (2019).
84. Xu, W., Li, Y., Liu, R., Yang, S., Liu, J. & Fang, X. Kinetic resolution of 2,2-disubstituted-1,3-diketones via carbene catalysis. *Org. Chem. Front.* **6**, 290–298 (2019).
85. Vasamsetty, L.; Kong, X., Meng, M., Yang, S., Xu, W., Reddy, P. S. & Fang, X. Divergent dynamic kinetic resolution of a racemic mixture of four stereoisomers via N-neterocyclic carbene organocatalysis. *Chem. Asian J.* **13**, 3838−3844 (2018).
86. Wen, G., Su, Y., Zhang, G., Lin, Q., Zhu, Y., Zhang, Q. & Fang, X. Stereodivergent synthesis of chromanones and flavanones via intramolecular benzoin reaction. *Org. Lett.* **18**, 3980–3983 (2016).
87. Zhang, G., Yang, S., Zhang, X., Lin, Q., Das, D. K., Liu, J. & Fang, X. Dynamic kinetic resolution enabled by intramolecular benzoin reaction: synthetic applications and mechanistic insights. *J. Am. Chem. Soc.* **138**, 7932–7938 (2016).
