# Supplementary material for: Access to enantioenriched compounds bearing challenging tetrasubstituted stereocenters via kinetic resolution of auxiliary adjacent alcohols
Source: Nat Commun. 2021 Jun 18;12:3735. doi: 10.1038/s41467-021-23990-4 (PMC8213810; doi:10.1038/s41467-021-23990-4)
Supplement: Supplementary file 2 — Description of Additional Supplementary Files [file 41467_2021_23990_MOESM2_ESM.docx]

**Description of Additional Supplementary Files**

Title: Supplementary Data 1

Description: single crystal data for 2j

Title: Supplementary Data 2

Description: single crystal data for 1s

Title: Supplementary Data 3

Description: single crystal data for 2ac

Title: Supplementary Data 4

Description: single crystal data for 4d

Title: Supplementary Data 5

Description: single crystal data for 7k

Title: Supplementary Data 6

Description: single crystal data for 11
